# Supplementary figures and images for: Comparative transcriptional profiling of the early host response to infection by typhoidal and non-typhoidal Salmonella serovars in human intestinal organoids
Source: PLoS Pathog. 2021 Oct 20;17(10):e1009987. doi: 10.1371/journal.ppat.1009987 (PMC8570492; doi:10.1371/journal.ppat.1009987)

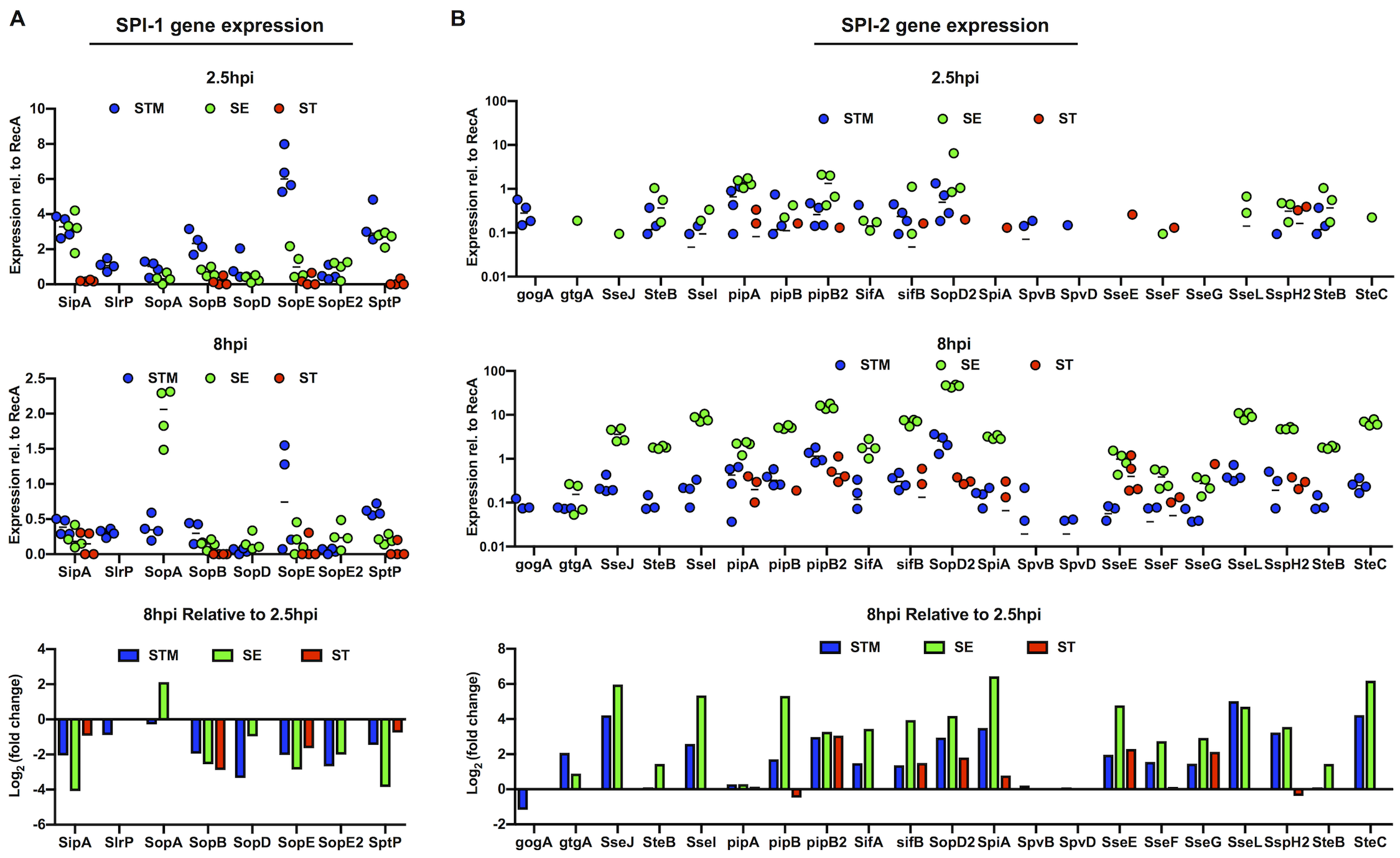

Supplement: S1 Fig — (A and B) SPI-1 and SPI-2 gene expression normalized to RecA expression at each time point in the HIOs. Log2(fold change) at 8h relative to 2.5hpi was calculated and shown in the bottom row. (TIFF) [file ppat.1009987.s001.tiff]

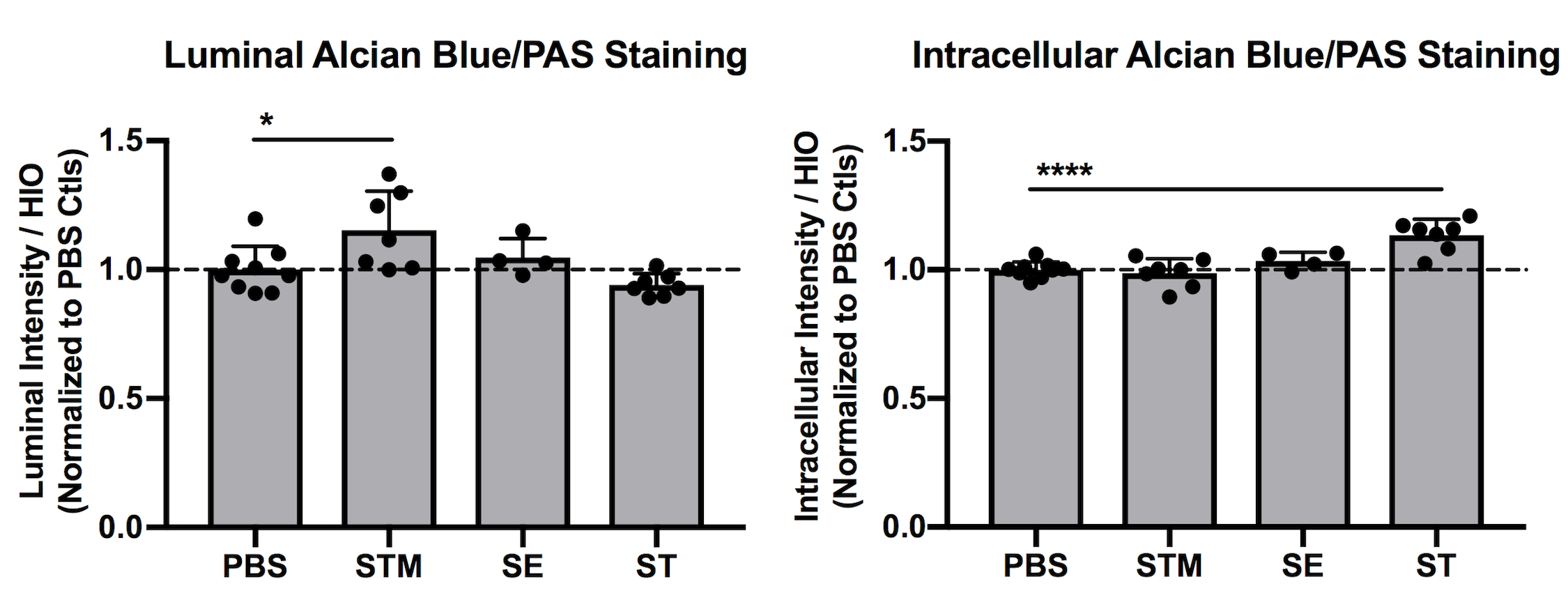

Supplement: S2 Fig — (A) Luminal and intracellular staining intensity from n>4 HIOs based on images shown in Fig 1G. Significance was determined by one-way ANOVA where P value: *<0.05 and ****<0.0001. (TIFF) [file ppat.1009987.s002.tiff]

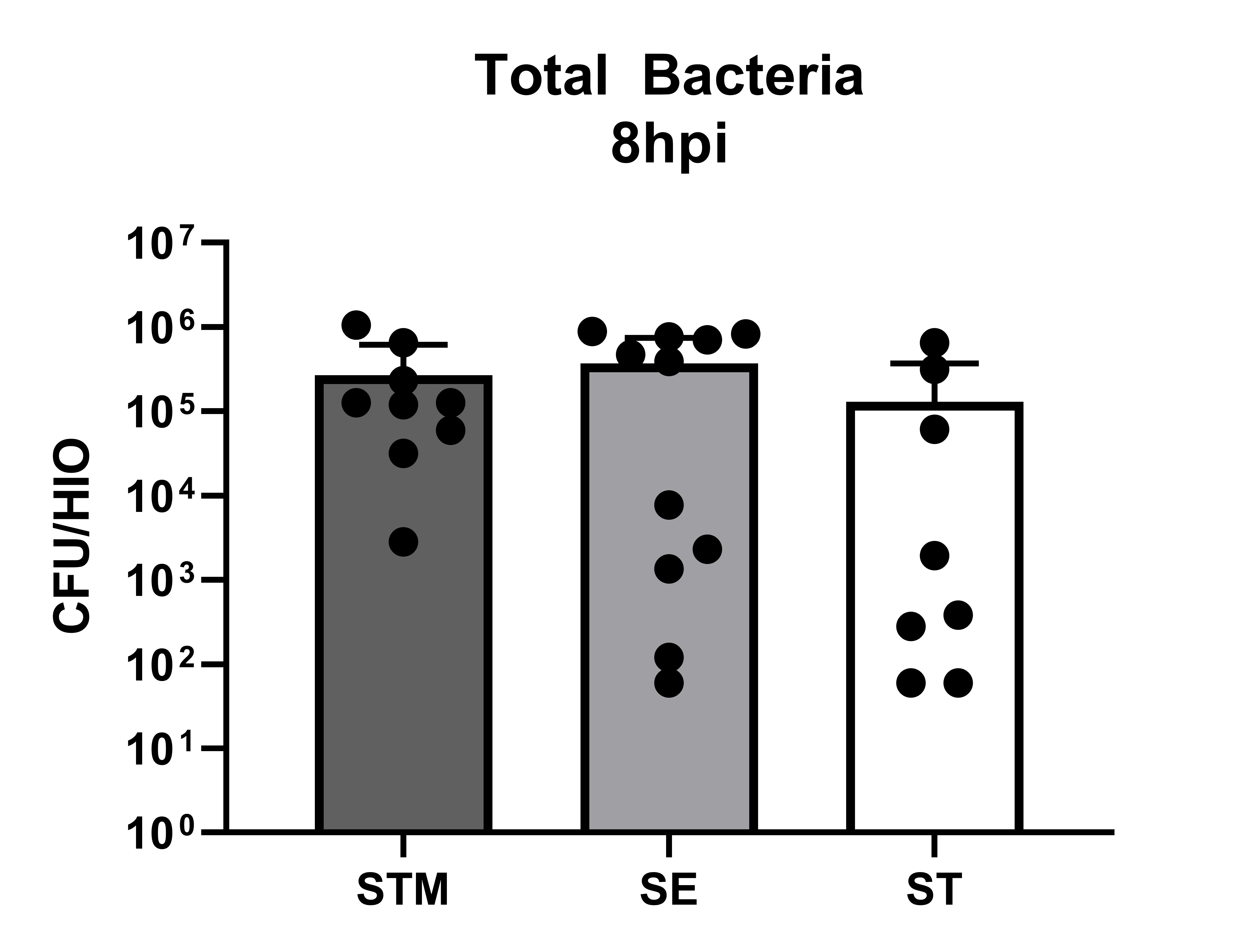

Supplement: S3 Fig — HIOs were infected with 105 CFU of STM, SE or SE and total bacterial burden per HIO was enumerated at 8hpi. Graph represents the mean of n>8 HIOs. (TIF) [file ppat.1009987.s003.tif]

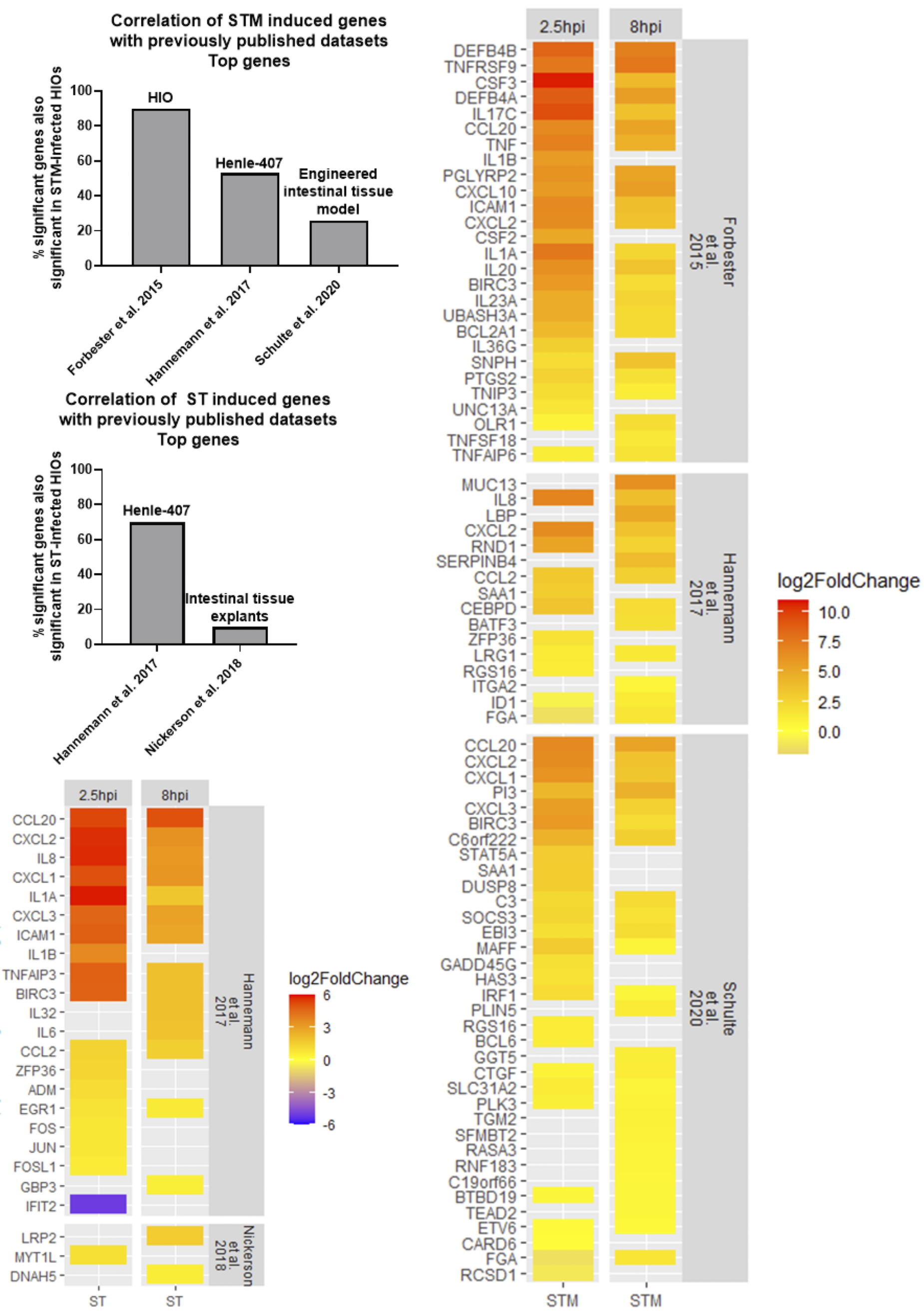

Supplement: S4 Fig — Top 30–50 genes reported in publications listed above were compared to our significant gene sets. The percentage of those genes that were also significant in either STM or ST-infected HIOs was plotted in the bar graphs with the model system used in each study listed at the top of each bar. Conserved gene changes were plotted in heatmaps to compare fold change across the different model systems. (TIFF) [file ppat.1009987.s004.tiff]

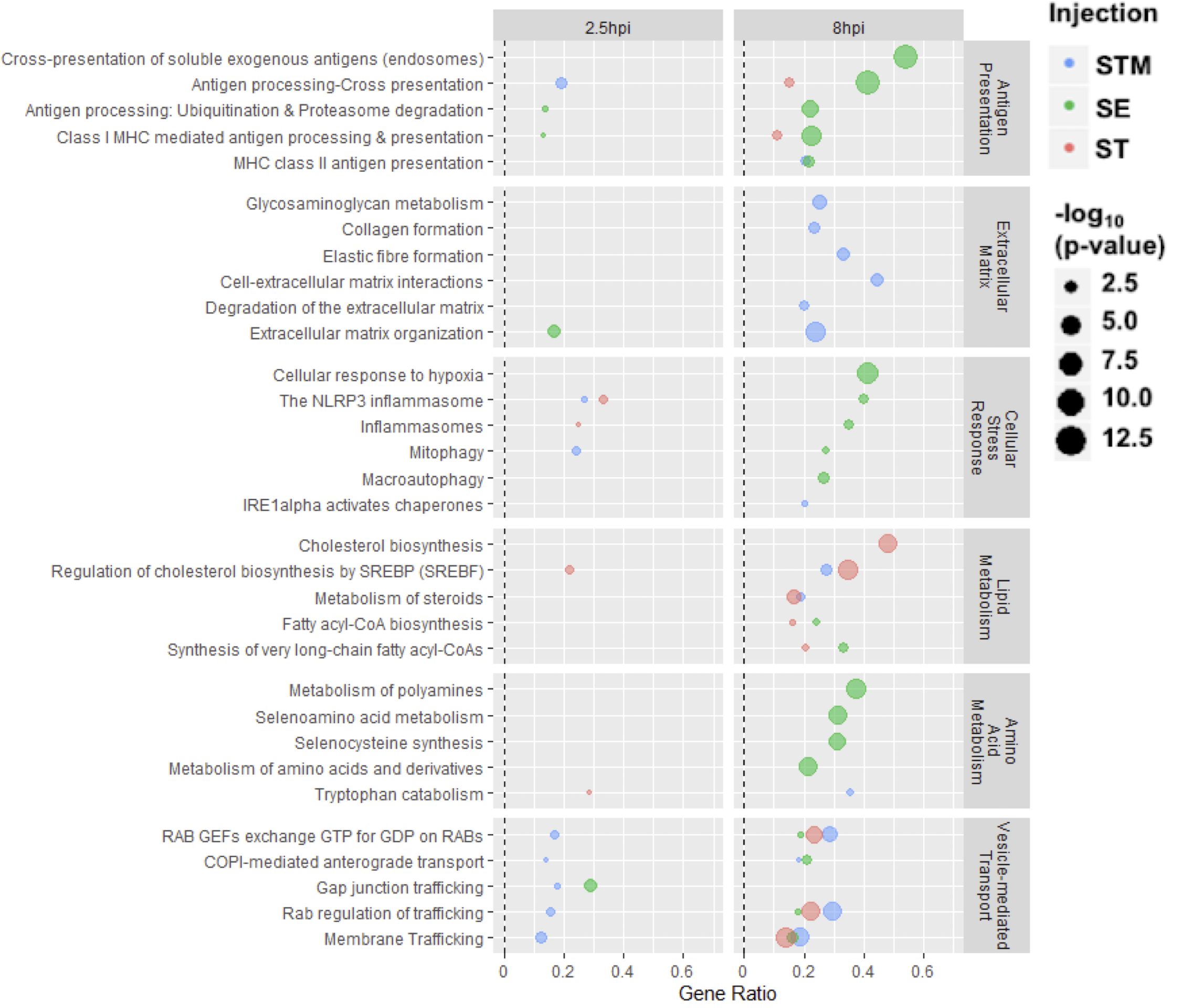

Supplement: S5 Fig — Dotplot shows select Reactome pathways that are significantly enriched (P value < 0.05) from upregulated gene sets of HIOs infected with different Salmonella serovars relative to PBS control. (TIFF) [file ppat.1009987.s005.tiff]

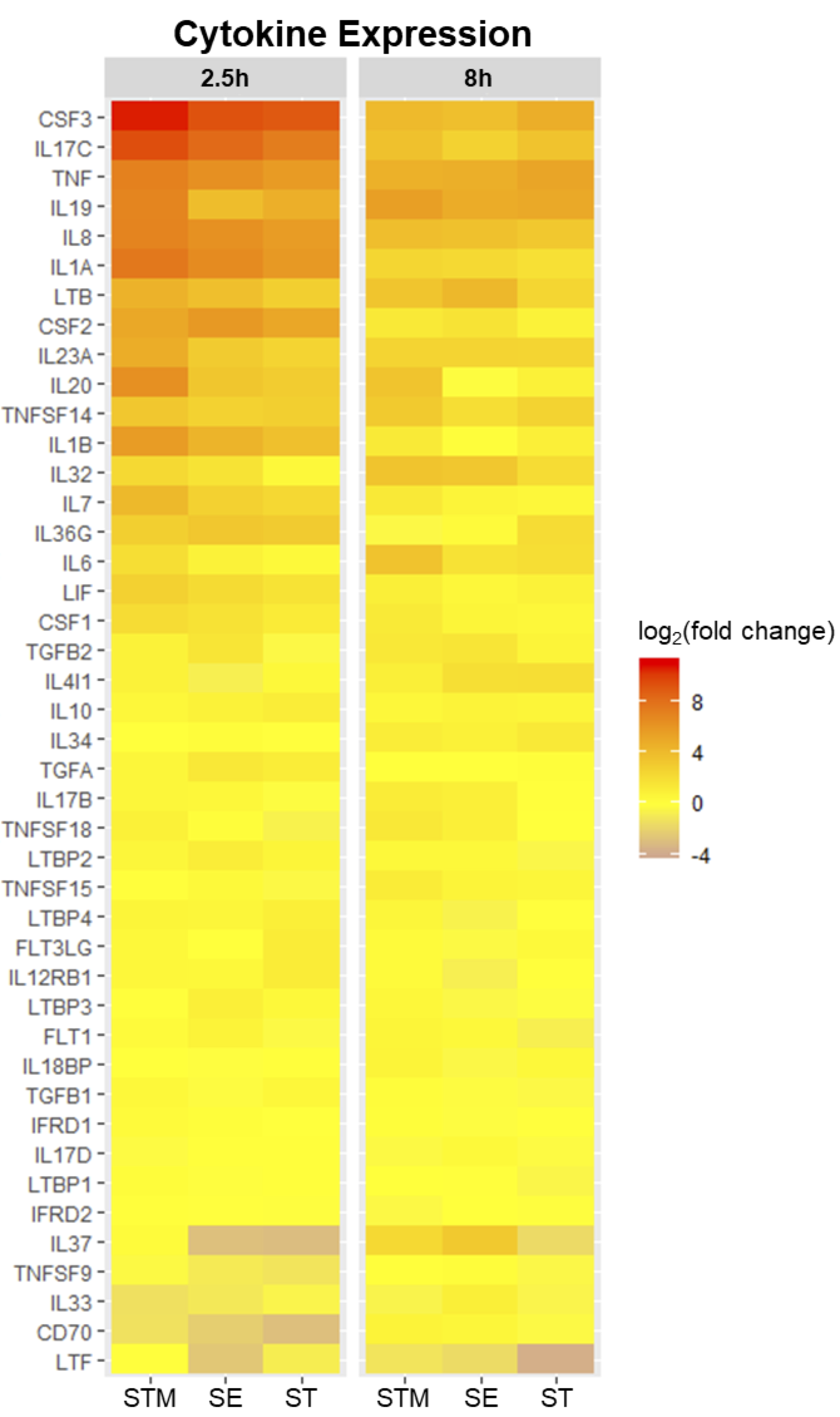

Supplement: S6 Fig — Gene expression presented as log2 fold change during STM, SE or ST infection relative to PBS at 2.5h and 8hpi. (TIFF) [file ppat.1009987.s006.tiff]

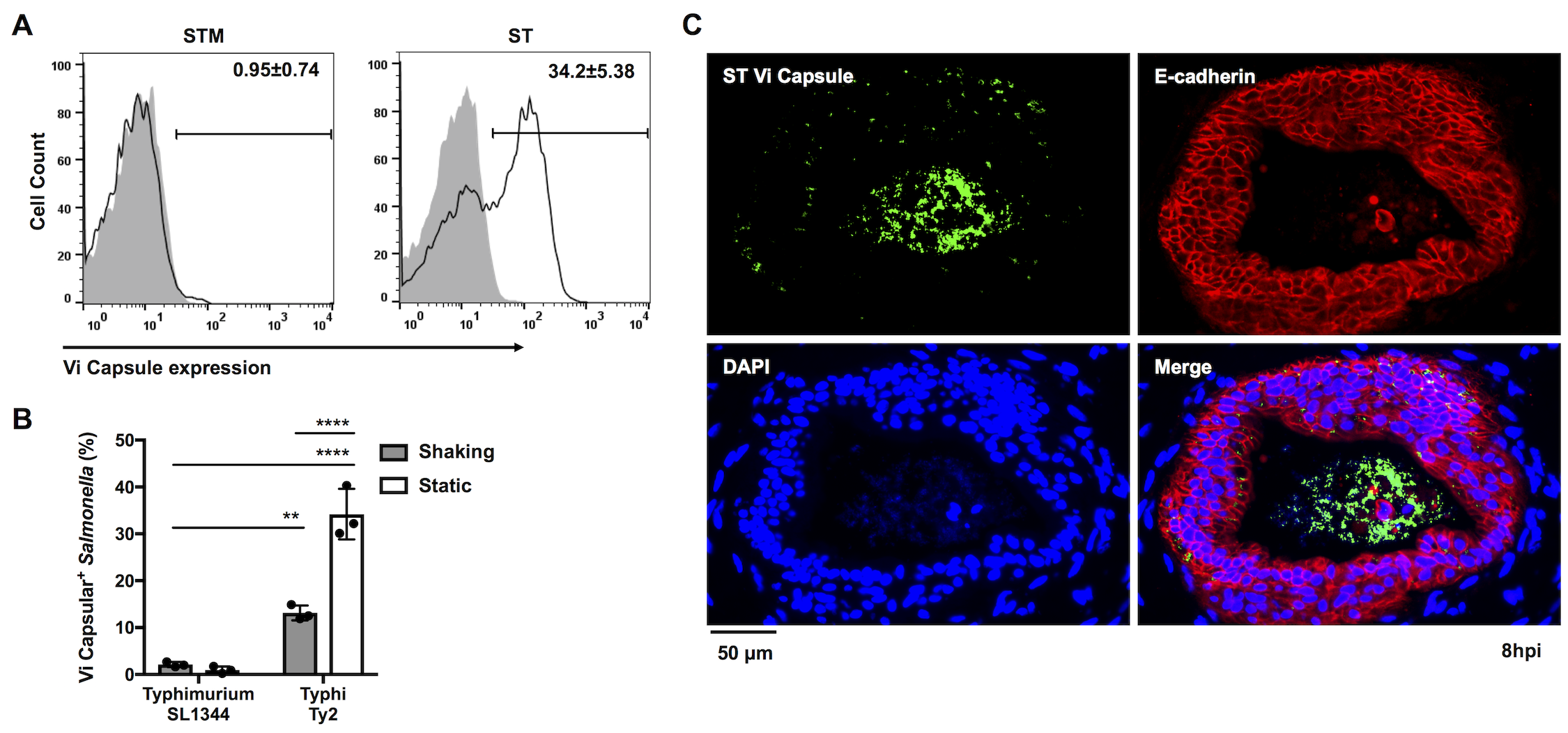

Supplement: S7 Fig — (A) Representative flow cytometry histograms of Vi polysaccharide capsule expression by STM and ST. (B) Vi polysaccharide capsule expression was quantified by flow cytometry using rabbit Vi antisera. STM strain SL1344 and ST strain Ty2 were cultured overnight at 37°C under static or shaking conditions. Bacteria were washed, stained with rabbit Vi antisera and analyzed by flow cytometry. Percent capsule+ cells was determined by gating against unstained cells. Graph indicates means +/- SD of n≥3 experiments. (C) Representative fluorescence microscopy images of ST-infected HIOs at 8hpi. Sections were stained with Rabbit antisera (green), anti-E Cadherin antibody (red) and DAPI (blue). P-value was calculated using two-way ANOVA with Sidek’s post-test for multiple comparisons. P value: **<0.01 and ****<0.0001. (TIFF) [file ppat.1009987.s007.tiff]

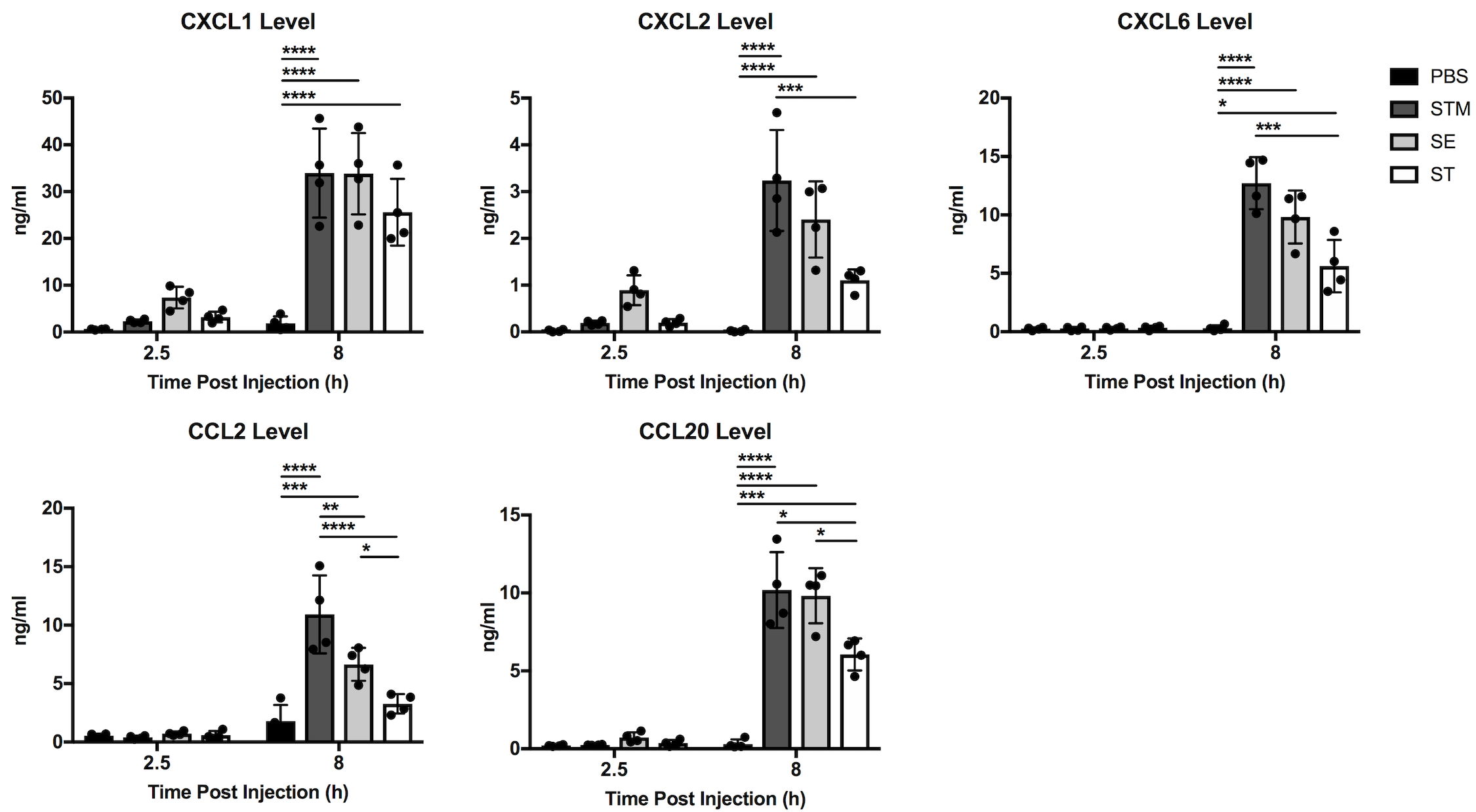

Supplement: S8 Fig — Graphs are presented as mean of n = 4 biological replicates with standard deviation (SD) error bars. P value was calculated using two-way ANOVA with Tukey’s post-test for multiple comparisons. P value: *<0.05; **<0.01, ***<0.001 and ****<0.0001. (TIFF) [file ppat.1009987.s008.tiff]
