# Supplementary material for: Comparative transcriptional profiling of the early host response to infection by typhoidal and non-typhoidal Salmonella serovars in human intestinal organoids
Source: PLoS Pathog. 2021 Oct 20;17(10):e1009987. doi: 10.1371/journal.ppat.1009987 (PMC8570492; doi:10.1371/journal.ppat.1009987)
Supplement: S1 Table — Significant DEGs (P < 0.05) in at least one infection condition are listed. (PDF) [file ppat.1009987.s009.pdf]

Table S1: DEGs 2.5h pi

	STM		SE		ST	
Symbol	log_2(fold change)	p-value	log_2(fold change)	p-value	log_2(fold change)	p-value
CXCL2	6.615801663	9.63E-103	5.534928478	1.98E-72	5.721038458	2.94E-77
NFKBIZ	2.283908472	3.39E-74	1.987182352	1.41E-56	1.939547012	5.88E-54
CXCL1	6.194937458	4.87E-73	5.374109333	2.05E-55	5.131674448	1.11E-50
NFKBIA	3.975903931	4.06E-73	3.543400103	1.91E-58	3.051124562	9.17E-44
CXCL3	5.652319061	1.85E-72	4.449282194	1.46E-45	4.660110999	8.18E-50
BIRC3	5.882106382	1.39E-69	5.141575068	1.39E-53	4.69835922	5.14E-45
ZC3H12C	2.37057614	1.33E-69	1.569329897	2.95E-31	1.335887702	5.54E-23
ZC3H12A	4.20255591	7.84E-65	3.364832388	3.69E-42	3.254690035	1.60E-39
TNFAIP3	5.508226484	4.62E-63	4.996544489	3.35E-52	4.764797609	1.32E-47
CLUHP3	2.471734213	2.45E-61	1.502132111	1.95E-23	1.995518099	2.49E-40
CCL20	6.71265219	1.91E-55	5.99233078	1.52E-44	5.304494296	2.85E-35
CXCL6	6.620945383	3.59E-55	5.098998956	2.46E-33	4.912374384	5.08E-31
REL	2.536079009	1.81E-50	1.971530992	3.71E-31	1.971922666	3.78E-31
RCAN1	3.85744704	4.71E-50	2.601510827	1.16E-23	2.123382496	2.88E-16
C6orf222	4.474396198	6.83E-46	3.788226139	2.45E-33	3.677477424	1.77E-31
SOD2	2.881461485	1.44E-45	2.082389281	1.33E-24	1.599221446	3.77E-15
TNFAIP2	4.836014333	2.49E-45	3.991992063	2.00E-31	3.271076811	1.26E-21
CX3CL1	4.760616528	2.21E-44	3.795715899	9.43E-29	3.523415798	6.23E-25
MAP3K8	3.362067202	4.40E-42	2.901193178	9.38E-32	2.736768761	2.26E-28
CASP10	3.165107087	6.64E-42	1.795045245	1.91E-14	2.429128124	2.97E-25
IKBKE	2.043267184	2.71E-41	1.478391035	3.39E-22	1.494802142	1.78E-22
SDC4	3.217873309	8.20E-41	2.490695579	4.03E-25	2.107753656	1.94E-18
ICAM1	6.683670443	2.04E-40	5.983587599	9.92E-33	4.787978985	1.55E-21
CFLAR	1.992214216	4.61E-40	1.372226241	7.34E-20	1.447614505	6.44E-22
CXCL5	5.623953884	7.45E-37	4.512719349	2.57E-24	4.456927736	9.30E-24
CDC42EP2	2.022097185	2.53E-36	1.127275162	2.76E-12	1.304637066	6.01E-16
KLF6	2.101878825	2.52E-36	1.360881342	3.87E-16	1.245031892	9.85E-14
TIFA	2.977773533	3.42E-35	2.4515861	2.59E-24	1.925131444	1.69E-15
IL8	7.015250208	6.81E-34	6.314745102	8.85E-28	5.772199312	1.77E-23
NCOA7	2.775844734	5.66E-33	1.912176081	1.83E-16	1.916287863	1.61E-16
NFKB2	3.049506011	7.28E-33	2.220425422	3.76E-18	2.154187656	3.73E-17
ANKRD33B	5.243636897	1.96E-32	4.792837489	2.54E-27	3.620582552	6.15E-16
ABTB2	3.910688729	8.62E-31	3.047157322	2.90E-19	2.739703286	8.21E-16
MAFF	3.069516386	4.79E-30	2.676176091	3.22E-23	2.023464073	7.29E-14
DEFB4B	8.529450849	6.35E-30	6.395654947	1.89E-17	6.138809668	3.63E-16
RND1	5.312025187	2.41E-29	4.177309043	9.54E-19	4.039895459	1.27E-17
SLC6A14	4.009310783	3.70E-29	3.26316319	7.44E-20	2.856943492	1.40E-15
SGPP2	2.779400717	3.61E-27	1.946220495	4.34E-14	1.465205743	1.40E-08
IL1A	7.59718865	2.84E-26	6.645901228	1.76E-20	5.914507821	1.54E-16
NFKB1	2.111039718	1.95E-25	1.79634815	7.43E-19	1.369344966	1.48E-11
BCL3	2.739360509	2.14E-25	2.233123498	2.51E-17	2.236113679	2.26E-17
CEBPD	3.449835774	2.20E-25	2.715650462	2.62E-16	2.636857627	1.89E-15
IRF1	2.043041475	2.23E-25	1.799412121	4.67E-20	1.307279399	3.20E-11
LDLR	1.177490947	2.08E-25	0.778287226	5.85E-12	0.777449349	6.28E-12

IRAK2	3.649313339	3.62E-25	2.837121723	8.45E-16	2.506551929	1.26E-12
HDAC9	3.10336985	1.24E-24	2.493341717	2.04E-16	2.117910296	3.42E-12
SAV1	2.291922907	3.24E-24	1.45636498	1.18E-10	1.405729788	5.32E-10
SGK1	2.600118244	6.30E-24	1.436839422	2.60E-08	1.746850935	1.30E-11
IFNGR1	1.740105298	1.88E-23	1.121819521	1.28E-10	0.85882856	8.80E-07
ZFAND5	0.804447457	1.85E-23	0.526431057	6.50E-11	0.308618257	0.00013
VNN3	3.63693001	4.65E-23	2.956939646	1.75E-15	2.990272496	8.11E-16
DUSP5	3.217158659	6.73E-23	2.431975592	9.66E-14	1.885837521	7.97E-09
CD69	4.806380611	1.21E-22	3.988968163	4.65E-16	2.916452369	3.36E-09
LIPG	1.76816937	3.58E-22	0.537348355	0.00335	1.049110403	9.60E-09
C2CD4A	3.7901461	4.33E-22	3.364799629	9.96E-18	3.101411431	3.00E-15
JUND	1.341976453	8.32E-22	0.544138543	0.00011	0.763842678	5.21E-08
RNF145	1.019456121	1.58E-21	0.692275335	1.02E-10	0.617585948	8.91E-09
TMPRSS2	2.401348235	1.65E-21	1.336019957	1.18E-07	1.560067535	6.22E-10
CACNG8	1.718047724	3.05E-21	1.375267023	4.05E-14	1.863578371	4.84E-25
ADAMTS9	2.168377076	5.79E-21	1.524253102	4.07E-11	1.251506831	6.11E-08
SQSTM1	1.797744679	6.94E-21	1.10878073	7.50E-09	0.989671565	2.52E-07
ITGB8	1.850364788	7.89E-21	1.379339345	3.05E-12	1.166858996	3.78E-09
TICAM1	3.166682874	1.30E-20	2.359222556	4.93E-12	2.406355823	1.94E-12
TNFSF14	3.302721875	1.38E-20	2.634311052	1.59E-13	2.824746171	2.47E-15
NINJ1	1.942390635	1.55E-20	1.322360264	2.76E-10	0.823208853	9.29E-05
PROX1	2.840503868	1.84E-20	2.438963672	1.73E-15	2.055480594	2.15E-11
CXCL10	5.822795639	3.28E-20	5.581188182	1.07E-18	3.668178502	7.37E-09
OAS3	1.446742372	3.66E-20	0.865266973	4.13E-08	1.263126829	9.73E-16
STAT5A	2.992247442	4.05E-20	2.438976431	7.64E-14	1.869954099	1.17E-08
IL17C	9.49583194	5.04E-20	8.336227769	8.85E-16	7.361009985	1.29E-12
BBC3	2.942048984	6.53E-20	1.882172166	6.37E-09	1.439837632	1.04E-05
SLC2A6	2.766167786	7.99E-20	1.879135104	7.97E-10	1.748826576	1.23E-08
CSF3	10.81361385	1.25E-19	9.361768416	4.32E-15	9.048417483	3.43E-14
CYP3A5	1.952226902	2.22E-19	1.110345077	3.11E-07	1.323537269	1.06E-09
TUBB2A	1.375637374	2.33E-19	0.9058759	3.32E-09	0.444074908	0.00396
HIVEP1	1.277857282	3.11E-19	1.061730016	9.67E-14	0.93511626	5.88E-11
IL23A	4.783281807	3.29E-19	3.090562445	1.08E-08	2.553345589	3.01E-06
IL7	4.130611548	3.28E-19	2.688781501	7.45E-09	2.274055403	1.41E-06
PLK2	2.029899193	3.39E-19	1.276182842	1.85E-08	0.800241074	0.00042
HIVEP2	1.720344629	3.60E-19	1.238467427	1.22E-10	0.784957708	4.69E-05
IL17RB	1.579097353	4.69E-19	0.658524598	0.00023	0.781818905	1.23E-05
ZNFX1	0.972698453	4.88E-19	0.385601726	0.00042	0.500484609	4.90E-06
BAZ1A	1.208574289	7.15E-19	1.065059718	5.37E-15	0.50834527	0.0002
IL1B	5.768983172	8.32E-19	4.415000342	1.28E-11	3.695990525	1.52E-08
ETV3	1.013376981	8.95E-19	0.626758772	4.67E-08	0.692939436	1.66E-09
LIF	2.688049691	1.17E-18	2.120534257	3.63E-12	1.659584188	5.56E-08
BCL10	1.1391055	1.31E-18	0.412971304	0.00148	0.478136217	0.00024
LTB	4.463330623	1.43E-18	3.770934866	1.26E-13	2.823493476	4.12E-08
TNIP1	1.533268357	2.07E-18	0.950856791	5.84E-08	0.865726787	8.00E-07
EFNB1	1.296846898	3.12E-17	0.9810922	1.72E-10	0.498192217	0.00126
B4GALT5	0.80779472	5.47E-17	0.411682815	2.00E-05	0.388696205	5.82E-05

BMP2	2.469997358	2.09E-16	1.513065909	4.91E-07	1.619190262	7.35E-08
PLAUR	1.864478113	2.11E-16	1.339182145	3.80E-09	1.104628258	1.23E-06
SPRR2A	7.137167727	3.74E-16	4.51532748	3.34E-07	4.13741647	3.31E-06
CSF1	2.033777985	4.37E-16	1.68884974	1.53E-11	1.179460618	2.76E-06
PIM2	1.885268375	4.34E-16	1.034195905	9.48E-06	1.234575254	1.24E-07
HS3ST1	2.065059483	5.48E-16	0.988141932	0.00011	1.208213663	2.37E-06
EPHA2	1.586905022	8.66E-16	1.276396653	9.73E-11	0.789726523	6.46E-05
OXTR	2.413350757	8.64E-16	1.972903683	5.01E-11	1.010759426	0.00095
ADAR	0.445101289	9.00E-16	0.267054942	1.35E-06	0.250151883	6.48E-06
NEDD4L	1.357556998	1.33E-15	1.037579723	1.03E-09	1.036068895	1.09E-09
TRAF3	1.158072703	1.32E-15	0.858942146	3.18E-09	0.895059312	7.12E-10
B3GNT5	1.119425574	1.34E-15	0.607869592	1.47E-05	0.424456697	0.00255
ZBTB38	1.102221995	1.40E-15	0.638952832	3.70E-06	0.354369695	0.01046
TRIM16	1.414374978	1.54E-15	0.959301867	6.65E-08	0.680270367	0.00013
IFNGR2	1.436482993	1.70E-15	0.913608666	4.17E-07	0.628781086	0.0005
EHD1	1.87520531	1.79E-15	1.22242158	2.45E-07	0.803839931	0.00072
ARRDC3	2.514627345	1.83E-15	2.666142978	3.37E-17	2.044396965	1.02E-10
C8orf4	3.119492729	2.10E-15	3.07590488	4.91E-15	1.724572521	1.28E-05
IL18R1	1.899346031	3.13E-15	1.314334011	5.43E-08	0.919082609	0.00015
IRGQ	0.698095991	3.17E-15	0.743223973	3.45E-17	0.652772234	1.71E-13
STX11	4.833583142	3.34E-15	4.152107098	1.55E-11	2.968845132	2.37E-06
FAM222A	1.945554799	3.95E-15	1.384369261	2.59E-08	0.936097785	0.00019
ICOSLG	2.407164067	5.07E-15	1.680064819	5.06E-08	2.064909871	1.91E-11
ANKRD36C	1.955679169	5.90E-15	1.159134371	3.79E-06	1.238852689	7.82E-07
DCUN1D3	1.071046101	8.17E-15	0.027164134	0.84527	0.137350883	0.32425
IER3	2.589488192	8.80E-15	2.377265889	1.08E-12	1.729582592	2.27E-07
NFKBIE	2.247033536	9.49E-15	1.411709639	1.52E-06	1.75461339	1.76E-09
PPP1R3B	-1.26599918	9.66E-15	-0.312410867	0.05255	-0.336073478	0.03735
RAB11FIP1	1.356397558	1.36E-14	1.001793646	1.29E-08	0.86870075	8.24E-07
KYNU	2.657295891	1.66E-14	1.918607808	2.98E-08	2.252049542	7.52E-11
KBTBD7	-0.821056125	2.86E-14	-0.644754361	6.82E-10	-0.310403529	0.00268
KLHL25	1.260125494	3.02E-14	0.696866244	2.97E-05	0.884895483	1.10E-07
ZNF217	0.787014183	3.80E-14	0.517179309	6.42E-07	0.468696554	6.80E-06
PPAP2B	1.423781217	4.27E-14	0.893736699	2.17E-06	0.366249425	0.05328
TRIM69	1.42042355	4.43E-14	0.720105319	0.00013	0.729897745	0.00011
NABP1	1.262692142	4.49E-14	0.461511589	0.00613	0.461876304	0.006
TNFAIP8	1.686401449	8.89E-14	1.196125595	1.25E-07	0.60200457	0.00797
FAM101A	2.634837695	1.35E-13	1.771970272	6.54E-07	1.917919018	7.29E-08
BID	1.61192077	1.47E-13	0.746532149	0.00063	0.714926834	0.00109
PPAPDC2	1.283805349	1.46E-13	0.783019252	7.11E-06	0.626435709	0.00036
FRAT2	-1.373859344	1.55E-13	-0.817971965	5.63E-06	-0.662935202	0.00023
OTUD1	1.501738959	1.54E-13	1.204961736	3.16E-09	1.143574426	2.04E-08
TNF	7.16562878	1.70E-13	6.406863885	4.63E-11	5.827747816	2.41E-09
TBC1D10A	1.201586133	2.03E-13	0.644973008	8.77E-05	0.561012074	0.00066
ARL14	1.983015286	2.13E-13	1.963844224	3.42E-13	0.79741266	0.00336
ZNF607	-0.894342003	2.39E-13	-0.530290011	4.01E-06	-0.4019839	0.00054
SPRR1A	5.167015134	2.41E-13	4.035123007	1.26E-08	2.403912377	0.00096

NFKBID	1.534520353	3.24E-13	1.677904275	7.91E-16	0.975353448	5.26E-06
PPP1R15A	1.805073916	3.30E-13	1.725183236	3.35E-12	1.02798692	3.49E-05
CT83	4.73743086	3.33E-13	3.628813742	3.67E-08	4.090429892	4.35E-10
BTG2	1.498646555	4.24E-13	0.973269333	2.55E-06	0.763922731	0.00022
SLC37A1	0.971662431	4.22E-13	0.85073921	2.04E-10	1.06914297	1.37E-15
ANKRD36	1.125867938	4.95E-13	0.610256188	9.15E-05	0.551295526	0.00042
IL32	2.245783163	6.44E-13	1.605709944	3.07E-07	0.428126585	0.18077
IER5	1.383515185	7.34E-13	1.087530585	1.73E-08	0.653528249	0.00081
NFKBIB	1.401633246	8.77E-13	0.90009353	4.76E-06	0.791398523	6.21E-05
CLEC2D	1.824600999	1.17E-12	1.766981276	5.10E-12	1.013780741	8.86E-05
RAB9A	1.513924455	1.58E-12	0.899484902	3.20E-05	0.692522548	0.00152
IFNAR2	1.428858611	1.59E-12	0.693447617	0.00062	0.822171864	5.10E-05
MOB3C	1.134721146	1.64E-12	0.751358676	3.09E-06	0.526173437	0.00119
PPP1R15B	0.904193555	1.99E-12	0.389375996	0.00247	0.420479204	0.00108
UBQLN4	0.878627957	2.00E-12	0.534409392	1.96E-05	0.636569545	3.61E-07
ZBTB10	1.17372463	2.23E-12	1.070891973	1.48E-10	0.538897138	0.0013
DEFB4A	8.888424608	2.69E-12	7.247247397	1.34E-08	6.670086233	1.91E-07
ZNF792	-1.089814875	2.96E-12	-0.976421564	1.80E-10	-0.42397127	0.00485
PHLDA1	1.463355019	3.12E-12	0.999689994	1.92E-06	0.808516354	0.00012
SAT1	1.795936683	3.13E-12	1.196682002	3.40E-06	1.10492759	1.80E-05
RIPK2	1.417438382	3.91E-12	0.886616767	1.47E-05	0.52670216	0.01055
KLRK1	2.183328226	3.98E-12	1.127627296	0.00046	1.869068013	3.43E-09
RAB4B	1.504651145	4.73E-12	0.723468896	0.00095	1.136444334	1.87E-07
ALAS1	1.126055274	5.08E-12	0.511130347	0.00175	0.568023539	0.00051
P11-986E7	3.28200507	5.65E-12	2.210439269	5.30E-06	2.964375496	5.70E-10
CCNL1	0.864911449	6.33E-12	0.485726982	0.00012	0.337554888	0.00741
IL19	6.968164513	6.56E-12	3.900564927	0.00017	4.625029221	7.16E-06
MFHAS1	1.016501617	6.69E-12	0.580799452	8.65E-05	0.383316767	0.00983
LCN2	5.63821581	7.06E-12	4.101582374	6.20E-07	4.416358025	7.96E-08
JAK2	1.542833051	7.67E-12	1.079345901	1.72E-06	1.150507031	3.40E-07
IL4R	1.057477395	8.06E-12	0.507317626	0.00108	0.734309352	2.15E-06
GJB2	2.244405452	8.62E-12	2.176426664	3.44E-11	1.357640091	3.92E-05
DUSP16	1.335715473	9.59E-12	1.20055415	9.60E-10	0.829089266	2.54E-05
SOWAHB	1.144275144	9.77E-12	0.331738984	0.04987	0.434555545	0.01026
SPRR2F	6.507675014	1.02E-11	4.345624673	6.83E-06	3.884005145	6.52E-05
GCNT3	1.736908116	1.07E-11	0.818791642	0.00136	0.575587389	0.02446
PTS	0.932116402	1.09E-11	0.521893718	0.00015	0.656184242	1.89E-06
TRIM31	2.50337826	1.29E-11	1.640526293	9.45E-06	1.592550405	1.75E-05
RAB3IP	1.072379338	1.35E-11	0.93139783	4.18E-09	0.611756877	0.00012
CCSAP	-0.576623256	1.47E-11	-0.223084785	0.00573	-0.278753216	0.00069
C3	2.25746585	1.50E-11	1.458129325	1.32E-05	1.864575486	2.51E-08
NFATC2	1.666323776	1.59E-11	1.628882732	3.96E-11	1.668283207	1.35E-11
RND3	1.06639598	1.73E-11	1.156228357	2.98E-13	0.4805097	0.00248
DUSP4	1.283231304	1.94E-11	0.760777309	7.03E-05	1.238138355	9.37E-11
MESDC1	0.837385314	2.27E-11	0.652549483	1.73E-07	0.469120149	0.0002
ANKLE2	0.824629444	2.56E-11	0.284064208	0.02155	0.271693966	0.02819
FAM43A	3.132223489	3.19E-11	2.151440123	5.63E-06	1.641474664	0.00058

LAMC2	1.255188588	4.04E-11	0.665785239	0.00046	0.112819965	0.55319
CCRN4L	1.529825708	4.10E-11	1.175059644	4.09E-07	0.794426042	0.00066
TRAF3IP2	0.974070887	4.28E-11	0.361151025	0.01482	0.58829482	7.20E-05
ZBTB21	0.955640064	4.57E-11	0.64561205	8.79E-06	0.650276888	8.32E-06
PELI1	0.905510036	5.15E-11	0.623812519	6.12E-06	0.501919476	0.00028
SH2B3	1.425091757	5.39E-11	1.043055389	1.60E-06	0.999372378	4.50E-06
HES1	-0.848260386	5.52E-11	-0.402003043	0.00159	-0.318093161	0.01258
PPP1R3D	-0.962579607	6.78E-11	-0.482427951	0.00037	-0.371964702	0.00626
SMAD3	1.28481985	6.99E-11	1.27145603	1.08E-10	0.68279098	0.00054
ZFP36	1.677238543	7.05E-11	1.618180199	3.07E-10	1.348498346	1.62E-07
RBBP8NL	-1.918982353	7.75E-11	-2.548285551	4.50E-17	-1.183915301	2.37E-05
NUAK2	2.165806343	7.99E-11	1.838449016	3.52E-08	0.632574602	0.06808
GPR37L1	1.660161214	8.24E-11	1.13185519	1.20E-05	1.189741788	4.72E-06
UBASH3A	4.813833876	8.83E-11	4.317122953	6.21E-09	2.92592353	0.00012
BTG1	1.088425096	1.06E-10	0.318483437	0.05887	0.641903505	0.00014
CAB39	0.65783769	1.26E-10	0.250714299	0.01441	0.577140031	1.70E-08
CFB	1.46576135	1.42E-10	0.485680839	0.03403	0.523813322	0.02233
DUSP6	1.2212528	1.47E-10	0.954050816	5.57E-07	0.360673327	0.05875
NEURL3	5.227028624	1.77E-10	3.898215695	2.58E-06	3.424488183	4.50E-05
SLC5A5	2.272959365	2.05E-10	2.160467624	1.11E-09	1.686434916	3.09E-06
FNBP1	0.757392907	2.73E-10	0.817784165	9.40E-12	0.761365199	2.10E-10
TAF4B	1.299543664	2.75E-10	0.776395803	0.00017	0.695815741	0.0008
F2RL1	1.008342722	2.81E-10	0.328938603	0.0398	0.237304334	0.13834
KLHL5	0.639434179	3.23E-10	0.581096875	9.23E-09	0.15725136	0.12472
CCL2	3.204770151	3.25E-10	2.674789548	1.56E-07	1.409165412	0.00578
P1-145M24	3.524064556	3.59E-10	3.577062918	1.46E-10	3.944223734	1.31E-12
TMEM217	2.3892582	3.71E-10	1.332003626	0.00061	1.437618259	0.00023
TNFRSF10E	0.988747421	3.77E-10	0.350431784	0.02657	0.290310405	0.06633
BACH1	0.856832844	3.91E-10	0.579132875	2.35E-05	0.408235807	0.00293
GPBP1	0.457963598	4.86E-10	0.201434329	0.00615	0.101699766	0.16855
AC159540.1	2.614350848	6.20E-10	1.791032158	2.91E-05	2.329172112	3.99E-08
TLE4	0.855814881	6.18E-10	0.559677877	5.38E-05	0.554032923	6.54E-05
SOCS3	2.398482444	6.26E-10	2.252956424	6.26E-09	0.889371851	0.02231
TNFRSF9	7.655762415	7.55E-10	7.81333138	3.27E-10	4.614747906	0.00026
RELB	3.865862186	1.05E-09	3.034258521	1.74E-06	3.08302079	1.22E-06
CTTNBP2NL	0.670808282	1.35E-09	0.454766008	3.98E-05	0.130655828	0.24082
TRIP10	0.737189045	1.40E-09	0.340476036	0.00521	0.327271928	0.0075
TRIB1	1.069964371	1.45E-09	0.486320281	0.00601	0.467547917	0.00842
UBD	2.641294127	1.47E-09	2.773991767	1.98E-10	2.209925553	4.41E-07
NFATC1	2.390811127	1.50E-09	2.371620523	1.95E-09	1.300798505	0.00124
PPP4R2	0.632660838	1.53E-09	0.445992936	2.03E-05	0.210513021	0.04468
CCDC130	0.595720303	1.78E-09	0.120938428	0.23038	0.31781005	0.0016
MED24	0.780437562	1.83E-09	0.100573906	0.44037	0.505003746	0.0001
PMAIP1	1.665596092	1.98E-09	1.124200695	5.30E-05	0.618643665	0.02673
CLIP2	1.035375455	2.14E-09	0.548381858	0.00153	0.563144521	0.00115
PRRG1	1.389641439	2.17E-09	0.807987989	0.00051	0.499875219	0.03169
TAPBP	0.7437541	2.25E-09	0.286142003	0.02151	0.73965681	2.64E-09

CPEB2	1.16870669	2.28E-09	0.887704602	5.69E-06	0.966365395	7.89E-07
RARRES1	1.892233002	2.62E-09	0.622644164	0.05206	0.178644953	0.58031
ZKSCAN1	-0.665105788	2.62E-09	-0.452819471	3.35E-05	-0.596977184	6.28E-08
ZNF697	1.014635296	3.99E-09	0.726624659	2.52E-05	0.298922453	0.08583
SGMS2	1.120511235	4.47E-09	0.648890798	0.00069	-0.035627802	0.85258
TXNRD1	0.839416297	4.91E-09	0.4469838	0.00184	-0.048876001	0.73425
JAG1	1.095774712	4.97E-09	0.851587236	5.49E-06	0.276642016	0.14034
PATL1	0.724817987	5.47E-09	0.306449237	0.0139	0.770676883	5.14E-10
TYK2	0.650297657	5.52E-09	0.314249538	0.00503	0.353391315	0.00159
HIPK1	0.560260926	5.58E-09	0.322750458	0.00079	0.425917514	9.51E-06
GRAMD4P7	2.115499727	5.87E-09	1.41558356	0.00013	1.623162265	1.05E-05
PHKG2	0.667345954	5.96E-09	0.479838097	2.67E-05	0.466100141	5.04E-05
TTC9	1.0829132	6.01E-09	0.738382578	7.42E-05	0.583891753	0.00181
BEND3	1.278670641	6.07E-09	0.611429697	0.00551	0.991204385	6.66E-06
NAMPT	1.846051828	6.18E-09	1.511389309	1.96E-06	1.096005932	0.00056
SLC52A3	1.352317058	6.40E-09	0.624218958	0.00852	0.772089312	0.00114
ING2	-0.830460204	6.55E-09	-0.372721579	0.00671	-0.480735117	0.00053
PRDM1	1.582649491	6.57E-09	1.230765481	6.82E-06	0.649385875	0.01832
NR4A1	1.835234274	6.64E-09	0.943858239	0.00294	0.952126803	0.00269
VMP1	0.680869159	7.16E-09	0.361294205	0.00213	0.484029322	3.91E-05
ZSWIM4	1.280901412	9.53E-09	1.087966332	9.99E-07	0.847930527	0.00017
MTND4P23	3.805654512	1.00E-08	3.53084381	1.05E-07	4.033268861	9.65E-10
TLR4	1.184060272	1.05E-08	-0.05705039	0.78352	0.23176095	0.26412
PLSCR1	0.723644117	1.10E-08	0.34009008	0.00733	0.397394259	0.00175
CXCL16	1.097651813	1.14E-08	0.325871047	0.09171	0.568527125	0.00321
PRKAB2	-0.972853978	1.15E-08	-0.692309261	4.14E-05	-0.673216034	6.95E-05
DUSP1	2.115196352	1.20E-08	1.393112779	0.00018	0.942473533	0.01141
FRK	0.841036073	1.20E-08	0.324797137	0.02782	0.793426482	7.56E-08
SLC8B1	1.318449818	1.21E-08	1.219312343	1.39E-07	0.821310836	0.00044
ATF3	2.705966196	1.26E-08	1.958258427	3.87E-05	1.425852175	0.00278
LAMA3	1.298037566	1.31E-08	0.727513793	0.00144	0.131116455	0.56597
EHF	1.299161433	1.33E-08	1.08069105	2.29E-06	1.128715944	8.00E-07
ITPKC	1.09716854	1.36E-08	0.821605832	2.13E-05	0.481324862	0.01298
B4GALT1	1.115604832	1.62E-08	0.542743383	0.00604	0.7288085	0.00023
MACC1	0.759313775	1.79E-08	0.441720886	0.00105	0.537834236	6.67E-05
LPIN1	0.82146875	1.82E-08	0.34703239	0.0176	0.671400163	4.31E-06
GCNT4	1.230691352	1.84E-08	1.207147362	3.21E-08	0.797284977	0.00028
NRARP	1.853718454	2.14E-08	1.467477505	9.71E-06	0.956657905	0.00458
ICAM4	2.267458828	2.24E-08	1.477799168	0.00035	1.807748034	1.03E-05
RAC1	0.650513785	2.32E-08	0.220137806	0.05872	0.248091501	0.03326
PSEN1	0.490822145	2.55E-08	0.187139782	0.03364	0.111797438	0.20584
NFE2L2	0.71177422	2.63E-08	0.268908916	0.03578	0.449534385	0.00045
C22orf29	-0.708600679	2.68E-08	-0.514926233	4.14E-05	-0.230197258	0.06561
USP54	0.829382997	2.77E-08	0.485693121	0.00115	0.865372967	6.83E-09
AASDHPPT	-0.547715944	3.21E-08	0.040413082	0.67833	-0.399419675	4.75E-05
HBEGF	1.916802913	3.41E-08	1.362226111	8.95E-05	1.061479988	0.00235
ZNF174	-0.881269003	3.55E-08	-0.654225773	2.01E-05	-0.521884496	0.00073

CREB1	0.749389686	3.96E-08	0.595454758	1.25E-05	0.307547377	0.02431
FUT2	0.879864821	3.99E-08	0.413108751	0.01001	0.742475726	3.60E-06
SOWAHC	-0.694632914	3.98E-08	-0.546718404	1.24E-05	-0.744747814	3.62E-09
BTG3	0.887944717	4.10E-08	0.543645537	0.00079	0.361846927	0.02616
KBTBD6	-0.702569004	4.32E-08	-0.440321425	0.00046	-0.593401976	2.98E-06
MRFAP1L1	-0.496605297	4.38E-08	-0.231735071	0.00936	-0.398004676	9.81E-06
FOSL1	1.717539229	4.45E-08	0.812519332	0.01018	0.633614707	0.04606
RASD1	2.596289802	4.45E-08	1.263605034	0.01038	1.173465941	0.0188
HAUS3	-0.561782037	4.89E-08	-0.271513853	0.00686	-0.41356709	4.42E-05
LRRC49	1.403713636	4.94E-08	1.307817943	3.17E-07	0.615526366	0.01768
PLAU	3.011383875	5.48E-08	3.359218973	1.32E-09	1.323551979	0.01715
STEAP2	0.890231049	5.49E-08	0.280376557	0.08839	1.213502654	1.04E-13
ETS1	0.828584335	5.82E-08	0.751705232	8.48E-07	0.463892593	0.00248
DUSP8	2.94146364	6.29E-08	2.189462055	6.47E-05	2.272561623	3.38E-05
KITLG	-0.996424683	6.86E-08	-1.372983685	1.05E-13	-0.843727194	4.64E-06
DYRK2	0.657999406	6.99E-08	0.193808262	0.11246	0.273032032	0.02542
CCDC66	-0.773949121	7.28E-08	-0.006466093	0.96312	-0.09191743	0.5157
SERPINA3	2.978587362	7.66E-08	2.199083619	7.25E-05	1.814142911	0.00106
LRRC8A	0.624346602	7.72E-08	0.450273638	0.0001	0.218943255	0.06044
MYADM	0.800240417	7.99E-08	0.279787552	0.06141	0.807548859	6.07E-08
AHCYL2	1.178923757	8.02E-08	0.278857929	0.20644	0.898516172	4.36E-05
CFL2	0.832830221	8.04E-08	0.749989673	1.29E-06	0.365117264	0.01921
C3orf58	-0.540513232	8.24E-08	-0.433506979	1.45E-05	-0.46012392	4.58E-06
RNF19A	1.249945501	9.21E-08	1.921513591	1.32E-16	0.95604234	4.92E-05
SLFN5	1.430774817	9.41E-08	1.283195523	1.68E-06	0.931336803	0.00052
RNF43	-1.490723998	9.51E-08	-1.586874422	1.26E-08	-0.929874865	0.00081
ZNF267	0.588055518	9.77E-08	0.123777906	0.26258	0.201985378	0.06776
SESTD1	0.743611853	1.02E-07	0.394481339	0.0047	0.427729159	0.0022
MCMDC2	-0.8322481	1.03E-07	-1.62E-05	0.99991	-0.74358638	1.03E-06
ZFP30	-0.825277026	1.04E-07	-0.455626809	0.00282	-0.43642393	0.00436
TPI1P3	2.895633959	1.07E-07	2.374141366	1.56E-05	3.140046984	5.55E-09
USP12	0.614215359	1.09E-07	0.089449482	0.44034	0.182842561	0.11495
ERRFI1	1.619558769	1.10E-07	1.28773143	2.42E-05	0.859915564	0.00483
SLC30A1	-0.63295571	1.16E-07	-0.773221441	9.18E-11	-0.444387895	0.00019
ZNF253	-0.640727605	1.31E-07	-0.262026647	0.02745	-0.372546146	0.00189
CYTH1	0.723495406	1.36E-07	0.727658103	1.12E-07	0.359975464	0.00892
VPS9D1	1.248689778	1.39E-07	0.44519008	0.06066	0.509708244	0.03151
SDCBP	1.03531459	1.41E-07	0.804145244	4.36E-05	0.44685284	0.02319
CEBPA	-1.300896884	1.52E-07	-1.817984239	2.67E-13	-1.077180147	1.27E-05
SNRPF	-0.768523008	1.53E-07	0.081263276	0.57165	-0.751118576	2.80E-07
IFIH1	0.952291189	1.63E-07	0.821899017	5.82E-06	0.624708723	0.00063
MAFK	0.868532604	1.73E-07	0.350958534	0.03534	0.45736749	0.00656
ZNF710	0.664737192	1.84E-07	0.295538575	0.02093	0.468036188	0.00027
CCDC51	-0.743184772	2.15E-07	-0.66889832	1.21E-06	-0.817187007	9.54E-09
GPX3	1.672737396	2.36E-07	0.890448169	0.0061	0.651589279	0.0465
ELK3	0.984042883	2.61E-07	0.513038173	0.00728	0.423591077	0.02733
PANX1	0.572156316	2.71E-07	0.321249902	0.00386	-0.021363168	0.84962

RDH10	0.903395368	2.92E-07	0.792033838	6.90E-06	0.392479358	0.02603
GTPBP2	0.575464255	2.94E-07	0.46082544	3.90E-05	0.495773697	1.03E-05
ZNF766	-0.526620197	3.23E-07	-0.01753305	0.86024	-0.017993951	0.85757
CXCL11	5.722261787	3.29E-07	5.585289044	6.41E-07	3.952959964	0.00064
ETAA1	-0.657238162	3.36E-07	0.092083258	0.45882	-0.138302186	0.27252
DBF4	-0.591464827	3.45E-07	-0.263080899	0.02146	-0.446786983	0.00011
TRIM47	1.680509902	3.45E-07	1.198537413	0.0003	0.832349186	0.01275
EGR1	1.330791409	3.56E-07	0.979670696	0.00018	0.878329534	0.00079
PPP2CB	0.473796803	3.58E-07	0.104631848	0.2624	0.146156617	0.11761
DUSP10	0.897423832	3.59E-07	0.753040635	1.87E-05	0.251248973	0.1599
MAPK6	0.426010333	3.60E-07	0.30687738	0.00024	-0.073108728	0.38432
NMNAT2	1.859408589	3.81E-07	1.731227331	2.16E-06	1.451527285	7.92E-05
DENND4A	0.752253966	3.83E-07	0.556254366	0.00017	0.379251658	0.01062
SAC3D1	-2.114226336	3.99E-07	-0.56247453	0.14616	-0.773995598	0.04907
SPIDR	-0.61576584	4.26E-07	-0.599945647	8.43E-07	-0.258231034	0.03394
C5orf45	0.708451767	4.27E-07	0.698313901	5.27E-07	0.824605791	3.27E-09
NEDD9	1.152209049	4.31E-07	0.803054187	0.00043	0.633377934	0.00552
C2CD4B	2.151849374	4.65E-07	1.139049361	0.00838	1.475271083	0.00061
NCEH1	1.098277895	4.76E-07	0.938270907	1.72E-05	0.231574306	0.29045
MYCN	-1.784631558	5.03E-07	-0.768016286	0.02434	-1.074484326	0.00183
SERPINB8	1.22442184	5.12E-07	0.622090016	0.01091	0.447331657	0.06771
PATZ1	-0.568162239	5.27E-07	-0.455687704	3.96E-05	-0.259970073	0.01893
CDK17	0.695045642	5.58E-07	0.80992548	4.58E-09	0.250118105	0.07169
FRMD8	1.063175104	5.60E-07	0.674660016	0.00152	0.439446337	0.03979
LRG1	1.157162351	5.69E-07	0.125706445	0.58911	0.524184801	0.02387
S100A3	2.604319402	5.71E-07	1.937472949	0.00022	0.47975069	0.39923
NAMPTL	1.793967566	5.74E-07	1.267777415	0.00041	0.919929109	0.01047
BCAR1	0.848444237	6.05E-07	0.80570746	2.19E-06	0.540346918	0.00149
ZSCAN20	-0.70081908	6.24E-07	-0.539568134	4.11E-05	-0.371611142	0.00473
C17orf80	-0.540339135	6.28E-07	-0.154714975	0.14522	-0.524268155	1.12E-06
PLEKHA4	1.049647449	6.65E-07	0.568860987	0.00721	0.172802888	0.41745
ELF3	1.057401973	6.70E-07	1.079258669	3.90E-07	1.236677628	6.10E-09
RAP1B	0.418711202	6.71E-07	0.483471163	8.73E-09	0.044373744	0.59915
CYLD	0.661873488	6.84E-07	0.567102376	1.93E-05	0.390072848	0.00356
LAMB3	0.963589844	6.91E-07	0.426119355	0.0282	0.479979458	0.01345
ZNF429	-0.632636302	7.56E-07	-0.101494316	0.40525	-0.278085489	0.02585
CSRNP1	1.229016206	7.72E-07	0.605667866	0.01506	0.393901234	0.11481
DCBLD1	-0.730166756	8.07E-07	-0.036905161	0.79771	-0.133664301	0.35489
FANCF	-0.615975654	8.38E-07	-0.274431197	0.02222	-0.177012628	0.14147
ZNF530	-0.783813335	8.44E-07	-0.556523181	0.00026	-0.101573188	0.50288
GPAM	-0.605166562	9.21E-07	-0.527001013	1.51E-05	-0.527091611	1.69E-05
BCL9L	1.261461903	9.47E-07	0.72920278	0.00469	1.020612706	7.32E-05
NONOP2	1.389766752	1.04E-06	0.768964016	0.008	0.70397431	0.01671
PTPRH	1.094784847	1.09E-06	0.56051461	0.01274	0.584607709	0.00932
RASGRP1	1.463668467	1.10E-06	0.93781489	0.00182	1.162965037	0.00011
UBE2Z	0.392512562	1.15E-06	0.002118228	0.97912	0.163729584	0.04288
OSBPL7	-1.13128222	1.16E-06	-0.960776993	3.09E-05	-0.198226101	0.3864

PIEZO1	0.975993628	1.18E-06	0.611360161	0.00234	0.514700795	0.01046
EFNA1	1.666268401	1.18E-06	1.786861112	1.83E-07	1.222664171	0.00037
MTERF	-0.604735974	1.24E-06	-0.409810151	0.00077	-0.409151384	0.00082
MAGOHB	-0.617957651	1.30E-06	-0.297846593	0.01664	-0.561663533	8.11E-06
ACKR3	1.027456081	1.35E-06	0.927365851	1.29E-05	0.75799254	0.00037
HELB	0.687852864	1.52E-06	0.757420508	1.00E-07	0.546449323	0.00013
SYS1	0.596501017	1.54E-06	0.271385971	0.02859	0.251717209	0.04312
PI3	4.225509644	1.56E-06	4.163676384	2.10E-06	1.766210099	0.05852
IMP3	-0.551758308	1.61E-06	-0.47071975	3.36E-05	-0.47701231	2.94E-05
SELE	6.222952591	1.62E-06	5.274754171	4.99E-05	4.800445752	0.00023
RWDD2A	-0.752703538	1.70E-06	-0.604007707	6.81E-05	-0.364039595	0.01573
PML	0.84108132	1.72E-06	0.332780024	0.05894	0.735269711	2.98E-05
GADD45G	1.809134361	1.78E-06	0.429915712	0.28132	-0.211439461	0.61704
CCDC38	-2.602234649	1.83E-06	-0.147090367	0.75794	0.169981489	0.71587
SLC31A2	1.136162338	1.84E-06	0.46128057	0.05896	0.471396774	0.05773
BCL2L15	1.916282768	1.87E-06	0.596922883	0.13836	1.518965162	0.00016
ZNF189	0.564542189	1.99E-06	0.27737816	0.01953	0.342956864	0.00394
ZBTB14	-2.188054804	2.01E-06	-1.069973255	0.01044	-0.714695923	0.08463
UGCG	0.90210073	2.23E-06	0.656678208	0.00057	0.188943241	0.32257
GRINA	0.620048177	2.43E-06	-0.028836681	0.82742	0.422450003	0.00135
NCR3LG1	0.822014913	2.47E-06	0.427404695	0.01445	0.000963921	0.99564
OSGEP	-0.469300411	2.51E-06	-0.011318742	0.90634	-0.177943207	0.06755
ZBTB24	-0.624317323	2.62E-06	-0.514997193	8.76E-05	-0.20152107	0.12321
SLC12A7	1.34598807	2.78E-06	0.198653556	0.49096	0.661401399	0.02155
ZNF550	-0.661046212	2.89E-06	-0.341025733	0.01218	-0.307324795	0.02514
NXT2	0.534354501	2.97E-06	0.446246813	8.16E-05	0.26414264	0.02156
NUDT21	-0.308728993	3.00E-06	-0.166736707	0.01111	-0.408773334	6.21E-10
LIMK2	-0.53530805	3.25E-06	-0.213201517	0.06165	0.140000668	0.2184
OPTN	0.748474565	3.28E-06	0.376453821	0.01928	0.164301088	0.30837
ARHGEF2	0.58200651	3.30E-06	0.502035842	5.78E-05	0.26617335	0.03392
SLC25A38	-0.506590098	3.46E-06	-0.616013081	1.24E-08	-0.372275926	0.00055
FTSJ2	-0.581007299	3.57E-06	-0.577016678	3.17E-06	-0.578413269	3.24E-06
PARP8	0.591247803	3.69E-06	0.504793917	7.42E-05	0.451155091	0.00042
KDM6B	1.087005472	3.72E-06	0.406477712	0.08467	0.979052554	3.04E-05
LRIG3	0.5319574	3.81E-06	0.20968292	0.06873	0.521047882	6.07E-06
RTKN	0.533550977	3.82E-06	0.002018566	0.98613	0.320846585	0.00563
VPS37B	0.621126937	3.89E-06	0.179879903	0.18215	0.328199145	0.01483
SH3BP2	0.770032226	3.96E-06	0.572581006	0.0006	0.571214778	0.00062
SOX2	-1.454922717	4.03E-06	-0.357730598	0.25236	-0.456201565	0.14483
RAD17P1	-1.770969971	4.08E-06	-0.349556399	0.27539	0.140368424	0.65484
DSE	0.98080748	4.18E-06	0.954706269	7.32E-06	0.454575015	0.03319
ZNF398	-0.536053712	4.20E-06	-0.389078643	0.00072	-0.233515851	0.04197
TTC13	-0.781162557	4.46E-06	-0.087411196	0.6015	-0.272683301	0.10485
USP25	-0.562098196	4.54E-06	0.062531898	0.60676	-0.329250513	0.00697
ZNF197	-0.398591398	4.65E-06	-0.023006037	0.77731	-0.334194602	7.82E-05
CSNK1E	0.399673535	4.69E-06	0.218492638	0.01208	0.310327834	0.00038
P11-745A24	1.53556437	4.70E-06	1.379664464	3.49E-05	1.360922899	5.15E-05

CIPC	-0.425080276	4.81E-06	-0.478636889	2.00E-07	-0.222533598	0.0154
TNIP3	1.976863968	5.03E-06	1.501607142	0.00055	1.182421046	0.00745
TFE3	0.656237073	5.06E-06	0.686437179	1.72E-06	0.589763043	4.32E-05
PLK3	0.949667402	5.10E-06	0.745473091	0.00034	0.474704954	0.02403
FRMD4B	0.830378789	5.29E-06	1.116022017	7.90E-10	0.416194616	0.0232
VANGL1	-0.637491104	5.28E-06	-0.368449042	0.00785	-0.122107785	0.37757
FARP2	0.55512736	5.35E-06	0.058151709	0.63389	0.478678407	8.59E-05
TNFRSF10D	0.852242812	5.37E-06	0.117013469	0.53281	0.061009274	0.74524
CRIM1	0.620168563	5.41E-06	0.042643156	0.75504	0.258968092	0.05816
HMGCS1	0.8511569	5.77E-06	0.502307065	0.00745	0.343107158	0.06759
N4BP3	1.123771154	5.76E-06	1.373573748	1.86E-08	0.85515434	0.00059
PTGS2	2.647852273	6.04E-06	1.633764433	0.00525	0.673939404	0.24994
TMCC3	1.065224814	6.03E-06	0.733895964	0.00177	0.350195377	0.13829
VNN1	1.666100907	6.03E-06	0.644842609	0.08722	-0.49141794	0.22745
BIRC2	0.614889607	6.26E-06	0.56694521	3.05E-05	0.282314648	0.03846
RIPK4	0.813900284	6.43E-06	1.017523927	1.29E-08	0.807407718	7.26E-06
ARID5A	1.028481903	6.52E-06	0.382507208	0.09711	0.391478179	0.09123
GCH1	2.04218145	6.57E-06	1.457158189	0.00131	0.087597568	0.84804
KIAA1586	-0.596341126	6.59E-06	-0.046122402	0.72026	-0.498655517	0.00015
ZNF92	-0.501146476	6.60E-06	-0.295157737	0.00707	-0.471573417	2.04E-05
PDP1	0.678742114	7.16E-06	0.603341121	6.52E-05	0.000260348	0.99863
N4BP1	0.435981067	8.61E-06	0.139758493	0.15369	-0.00211114	0.98289
CA5B	0.991008147	8.75E-06	0.550851809	0.0137	0.678059115	0.00246
TBCA	-0.445822351	8.75E-06	-0.454352107	4.46E-06	-0.707106323	2.18E-12
CCDC85A	1.820847961	8.80E-06	1.173792594	0.00443	0.936829568	0.02442
EHD4	0.494230224	8.89E-06	0.014431938	0.8973	0.174097756	0.11991
ZNF232	-0.617858243	9.01E-06	-0.429719673	0.00163	-0.479876064	0.00049
KATNBL1	0.758819226	9.41E-06	0.219301922	0.20074	0.054558373	0.75049
ZKSCAN4	-0.719796813	9.59E-06	-0.680574114	1.36E-05	-0.378878477	0.01396
CASP8AP2	-0.428794543	9.71E-06	-0.230092244	0.0161	-0.217408753	0.02392
RAB20	-1.02066793	1.02E-05	-0.833298249	0.00022	-0.106392472	0.62866
TP53INP2	1.161403369	1.04E-05	0.763771287	0.00386	0.91113069	0.00056
SESN1	-0.882667731	1.05E-05	-0.998430521	5.70E-07	-0.488493005	0.01416
ACOX1	0.523716598	1.05E-05	0.242865623	0.04093	0.491332219	3.52E-05
CLCF1	0.889938038	1.06E-05	0.76774129	0.00014	0.511857865	0.01253
C9orf69	-0.512830808	1.06E-05	-0.499804334	1.34E-05	-0.340553429	0.00298
CPEB4	0.716336992	1.08E-05	0.383622895	0.01856	0.672446935	3.64E-05
KDELC1P1	1.411831082	1.08E-05	1.372672286	1.63E-05	2.195695262	2.49E-12
IRF6	0.770042641	1.08E-05	0.211616524	0.22704	0.286618599	0.10206
CDC42EP1	0.859195127	1.10E-05	0.321728504	0.1001	0.69037975	0.00041
FOXO1	0.865587164	1.11E-05	0.986333012	5.11E-07	0.512262806	0.00945
MBLAC2	-0.709292198	1.12E-05	-0.2285212	0.13874	-0.159623013	0.30582
ZFP36L1	0.603325364	1.12E-05	0.473353062	0.00056	0.651102447	2.09E-06
DDI2	-0.551249168	1.14E-05	-0.305085551	0.01435	-0.040681653	0.74416
OAF	0.59184808	1.16E-05	0.33608308	0.01322	0.187275977	0.17137
EDN1	1.151218842	1.19E-05	0.380022461	0.15097	0.670117291	0.01116
MED15	0.706769956	1.33E-05	0.789665992	1.08E-06	0.615545732	0.00015

DNAAF2	-0.612653206	1.33E-05	-0.343326591	0.01143	-0.328431132	0.01629
RPL15P3	11.39284259	1.34E-05	0.043729422	0.98758	8.9792916	0.0006
FZD7	0.704339369	1.40E-05	0.440005695	0.00666	0.448537062	0.0057
HIF1A	0.59725814	1.40E-05	0.90958072	3.62E-11	0.261748276	0.0572
RNF168	-0.42808997	1.40E-05	-0.428042633	1.26E-05	-0.267863857	0.0062
TRAF1	1.535126745	1.41E-05	1.352291313	0.00012	0.520612791	0.14615
NEK2	-0.884384468	1.44E-05	-0.100943946	0.61285	-0.436491639	0.03016
ACAA1	-0.592583019	1.48E-05	-0.459488766	0.00073	-0.527879282	0.00011
PSMD4	-0.412601112	1.50E-05	-0.215911822	0.02274	-0.4135416	1.51E-05
PPP1R3C	-1.610453779	1.53E-05	-0.748931554	0.04078	-0.732209531	0.0458
ALPK3	0.7902101	1.54E-05	-0.039816301	0.82806	0.68576292	0.00018
RFFL	0.53103616	1.56E-05	0.247104715	0.04386	0.315444285	0.0105
GPATCH11	-0.501478929	1.58E-05	-0.276487474	0.01388	-0.363619149	0.00141
JAK1	0.506751353	1.64E-05	0.329175536	0.00511	0.195468123	0.09673
JUNB	1.46027644	1.66E-05	1.434398461	2.32E-05	0.769103987	0.0235
PNRC1	0.565340627	1.66E-05	0.430034796	0.00105	0.341745179	0.00927
ABCF1	0.462975225	1.67E-05	0.249069121	0.02057	0.189422934	0.07858
PPCS	-0.509123356	1.69E-05	-0.359048243	0.00221	-0.373041699	0.00154
VRK2	0.503091807	1.72E-05	0.21886679	0.06127	0.172765611	0.14217
UBE2Q2	-0.556608911	1.90E-05	-0.163566498	0.20366	-0.443352909	0.00064
TATDN2	0.674082072	1.98E-05	0.286682415	0.06951	0.665547149	2.43E-05
TGDS	-0.549169193	1.98E-05	-0.355032769	0.00471	-0.451257835	0.00038
TRIM68	-0.621528313	2.05E-05	-0.540533417	0.00017	-0.201452599	0.15867
ZSCAN12	-0.479618026	2.07E-05	-0.101007615	0.35456	-0.243136836	0.02787
RIMBP3C	-0.658281871	2.07E-05	-0.536470632	0.00052	-0.861661654	2.52E-08
SASS6	-0.736965651	2.13E-05	-0.357964151	0.03618	-0.44399441	0.00969
IRAK3	1.874468484	2.23E-05	2.118162775	1.55E-06	1.681590311	0.00015
PIK3R1	-0.616279207	2.25E-05	-0.366572525	0.01153	-0.396722265	0.00626
ZMYM5	-0.49427064	2.26E-05	-0.134764304	0.23233	-0.137503928	0.22819
ZNF480	-0.562421743	2.28E-05	-0.164434553	0.21026	-0.241861127	0.06656
KIF18A	-0.569361548	2.34E-05	0.056100483	0.6704	-0.294178909	0.02765
SRP9P1	9.110520817	2.38E-05	7.171528343	0.0009	7.652697887	0.00039
CTNNB1	0.402736685	2.39E-05	0.020423452	0.83039	-0.044562462	0.64037
GATSL3	1.227875218	2.40E-05	0.60176384	0.04145	0.312098897	0.29359
EBI3	1.876634636	2.43E-05	1.041051813	0.02097	-0.5680601	0.25452
ZNF107	-0.726851504	2.43E-05	-0.172126372	0.30165	0.001917438	0.99088
TBC1D24	-0.632373537	2.47E-05	-0.271105969	0.0664	-0.013743685	0.9255
ASF1A	-0.470342602	2.49E-05	-0.328172265	0.00274	-0.339136794	0.00217
CCDC8	-0.587423376	2.52E-05	-0.433747248	0.00173	-0.366197143	0.00824
ANKK1	2.924847325	2.60E-05	2.015271752	0.0039	1.62218744	0.02263
DKK1	-1.890515494	2.61E-05	-1.89733117	2.32E-05	-1.414538988	0.00147
STK40	0.49249537	2.61E-05	0.247852441	0.03432	0.089549435	0.44706
ZNF57	-1.087387596	2.61E-05	-0.621690116	0.01418	-0.705129008	0.00572
ZNF730	-0.631446801	2.61E-05	-0.031477767	0.82404	-0.384076219	0.00863
CYP27C1	1.130368129	2.65E-05	1.158054872	1.44E-05	1.07151301	6.59E-05
MSANTD4	-0.560747101	2.65E-05	-0.278672585	0.03458	-0.693632762	1.86E-07
SBNO2	1.215267519	2.73E-05	1.035425438	0.00035	1.108233556	0.00013

AP4B1	0.832482343	2.78E-05	0.538177488	0.00679	0.812833399	4.37E-05
ZFP36L2	-0.744388441	2.82E-05	-0.399739177	0.02373	0.143292278	0.41641
RAPGEF3	1.287677369	2.85E-05	0.935618267	0.00244	0.719558988	0.02075
FBXO45	-0.380592387	2.87E-05	-0.114005279	0.19894	-0.290486125	0.00121
CSF2	5.056552137	3.06E-05	5.928263671	8.96E-07	5.124851503	2.33E-05
KIAA1217	0.762639118	3.05E-05	0.436280807	0.01708	0.776339161	2.18E-05
B3GNT1	-0.55316625	3.16E-05	-0.565136011	1.44E-05	-0.322564029	0.01279
FAIM3	1.965773595	3.26E-05	0.796624591	0.10274	1.679263072	0.00038
VCAM1	2.587062563	3.29E-05	2.577892896	3.50E-05	0.885362174	0.15589
GLRX2	-0.70547441	3.31E-05	-0.453088644	0.00491	-0.457223508	0.00514
ARIH2	0.345953887	3.33E-05	0.263366721	0.00148	0.404182638	1.16E-06
ZNF224	-0.466171229	3.34E-05	-0.117868517	0.28256	0.008253825	0.94029
RRP1	0.593085006	3.38E-05	0.122062277	0.39454	0.429097842	0.00276
SNRPFP1	5.697105126	3.40E-05	5.879241809	1.82E-05	4.430599077	0.00139
UQCR11	0.480197555	3.40E-05	-0.063318186	0.5861	0.288172605	0.01296
PGBD2	-0.745057192	3.42E-05	-0.338205963	0.05158	-0.265918544	0.12722
LIAS	-0.700103365	3.46E-05	-0.595193049	0.00033	-0.114408195	0.48269
PRR15L	-1.508576013	3.57E-05	-1.066563192	0.00337	-0.687004219	0.05877
MAPKAPK2	0.30644473	3.74E-05	0.182326056	0.01374	0.286674791	0.00011
TP53	0.984178985	3.81E-05	0.926464682	0.0001	1.06727022	7.73E-06
ZNF816	-0.801447004	3.87E-05	-0.796189099	3.61E-05	-0.406180881	0.03396
C12orf29	0.486968615	3.89E-05	0.256474256	0.0301	0.007889862	0.94711
MRPS31P4	-1.081802688	3.91E-05	-0.171486921	0.47305	-0.579195139	0.01957
CTB-50E14.	0.962822953	3.92E-05	0.442065679	0.06168	0.527666489	0.0262
EGR3	2.168300097	3.94E-05	2.015317306	0.00014	1.177330295	0.02734
GATA6	-0.87932755	3.98E-05	-0.513365273	0.01601	0.136635863	0.5206
ZNF606	-0.618694356	4.01E-05	0.008835239	0.95167	-0.156425296	0.28972
SAP30L	-0.799226008	4.02E-05	-0.115629859	0.54908	-0.446531373	0.02118
AC024592.1	5.265116192	4.14E-05	5.952116298	3.56E-06	4.036523449	0.00172
DEDD2	0.711302868	4.15E-05	0.667054984	0.00011	0.194989642	0.2665
FGFR3	-0.697439913	4.14E-05	-1.045595482	8.56E-10	-0.571688811	0.00077
SERTAD2	0.707682688	4.14E-05	0.61114634	0.00039	0.714342022	3.41E-05
SKIV2L2	-0.327708072	4.14E-05	-0.276529239	0.0005	-0.260312069	0.0011
GPR132	1.804925313	4.18E-05	1.356824489	0.00214	1.313832112	0.00331
CREB3	0.410849628	4.25E-05	0.090744283	0.3663	0.05453934	0.58987
CD55	1.236832612	4.45E-05	1.046377114	0.00055	0.035837382	0.90606
ATP5H	-0.31497533	4.56E-05	-0.234464537	0.00216	-0.441796141	1.04E-08
FAM213B	0.50204248	4.56E-05	0.103566871	0.40282	0.398645861	0.00118
LINC00888	-0.542504184	4.55E-05	-0.193729397	0.12882	-0.686859871	2.56E-07
RHOG	0.456100227	4.63E-05	-0.331716096	0.00412	-0.155421873	0.17867
ZNF433	-0.735713016	4.84E-05	-0.286775929	0.09045	-0.250006974	0.14578
COX11	-0.373076639	4.93E-05	-0.340569889	0.00018	-0.206159494	0.02347
NADK2	-0.40197961	5.02E-05	-0.236064159	0.01569	-0.216763119	0.02719
DAPP1	2.297192573	5.04E-05	2.525846953	7.54E-06	1.46809552	0.00998
SNAPC3	-0.434082031	5.20E-05	-0.066592329	0.52924	-0.178661924	0.09317
ZSWIM3	-0.704897256	5.23E-05	-0.614107501	0.00022	-0.471121847	0.00466
FSTL3	0.97137198	5.36E-05	0.218232288	0.36737	0.143425977	0.55426

ZNF605	-0.527998835	5.45E-05	-0.106172482	0.41054	-0.090067554	0.48634
RBAK	-0.350048495	5.59E-05	-0.245277338	0.00401	-0.188239101	0.02861
TRIOBP	0.358703023	5.61E-05	0.309246938	0.0005	0.340177824	0.00013
FAM83A	-0.640364782	5.68E-05	0.252292629	0.11185	-0.293168639	0.06499
TMEM106A	2.108475374	5.68E-05	1.272396253	0.01657	1.863759935	0.00042
TC-479C5.1	1.062459859	5.77E-05	-0.172618436	0.52097	0.503748396	0.05654
ZNF440	-0.509605365	5.82E-05	-0.153301808	0.21826	-0.072119562	0.56225
DOLPP1	-0.43552029	5.84E-05	-0.42470298	7.00E-05	-0.419076421	9.53E-05
UBE2V2	-0.345308722	5.87E-05	-0.088893974	0.29262	-0.442450548	2.22E-07
AFF4	0.532636983	5.90E-05	0.361955999	0.00632	0.386191933	0.00358
CLIC4	0.531626251	5.89E-05	0.508491186	0.00012	0.140750627	0.28788
TNIP2	0.551417645	6.05E-05	0.048258272	0.7275	0.13813967	0.32132
MNX1	-1.416890555	6.08E-05	-1.893856891	1.11E-07	-0.978212234	0.00393
SORD	0.819886207	6.25E-05	0.023737896	0.90779	0.542743159	0.00806
GGCT	-0.470512137	6.44E-05	-0.111024761	0.33459	-0.444340441	0.00015
TNFRSF11B	1.442031464	6.52E-05	0.75100994	0.03803	0.40862553	0.26013
AC112218.1	0.413503314	6.58E-05	0.173422114	0.09385	0.286846864	0.00563
SREK1IP1	0.651382672	6.59E-05	0.106779014	0.51214	0.21093792	0.1965
BCL11B	-1.214201559	6.74E-05	-0.787396168	0.00829	-0.537659113	0.07076
LYSMD4	-0.716939894	6.73E-05	-0.460456114	0.00897	-0.092459551	0.59756
FAM171A1	0.452524944	6.80E-05	0.330402434	0.00347	0.319670915	0.00486
STAG3L4	0.640329354	6.83E-05	0.413321871	0.00821	0.514259605	0.0011
C2ORF15	-0.944521221	7.06E-05	-0.633934312	0.00595	-0.166352363	0.46375
RPA4	1.775722151	7.09E-05	1.677550574	0.00015	1.988480924	6.23E-06
FAM217B	-0.797189745	7.12E-05	-0.396894282	0.04477	-0.789878062	7.21E-05
SPRR1B	3.244276858	7.15E-05	2.779138496	0.00069	1.253719255	0.14569
PCNXL3	0.443237959	7.19E-05	0.053511734	0.63162	0.600417885	6.05E-08
CTBP2	0.337236058	7.24E-05	0.187042517	0.0271	0.30800647	0.00028
BCL6	1.061517768	7.32E-05	0.351140165	0.19047	0.228666901	0.3947
FAM57A	0.471343491	7.36E-05	0.05223736	0.66136	0.217002741	0.06825
EBLN2	0.711195156	7.40E-05	0.80563049	4.80E-06	0.856721086	1.29E-06
PTAFR	1.406392449	7.44E-05	0.412170124	0.24816	0.404647413	0.25719
ZMYND15	1.745401216	7.47E-05	0.953713178	0.03323	1.18460894	0.0087
ATP1B1	0.669426678	7.65E-05	0.065016038	0.701	0.153339735	0.36515
CYP2C18	1.114048509	7.71E-05	1.11724089	7.17E-05	1.1521968	4.28E-05
TSEN54	-0.444833809	7.84E-05	-0.230053357	0.03746	-0.030134148	0.78379
ZNF761	-0.531116384	7.84E-05	-0.558808391	2.92E-05	-0.143711725	0.27986
EEF1A1P5	0.563303042	7.87E-05	-0.291375499	0.04115	0.350858622	0.01392
NUDT9	-0.542037945	7.91E-05	-0.195706414	0.14649	-0.35023624	0.0105
SNPH	2.041931067	7.95E-05	-0.026856376	0.95947	0.753836282	0.14932
RAB1A	0.345065418	8.11E-05	-0.094019721	0.28315	0.165794704	0.05843
ZNF703	-0.70193854	8.37E-05	-1.107877826	7.06E-10	-0.294559891	0.09028
OAS1	0.708941885	8.40E-05	0.087773817	0.62684	0.312999517	0.08294
TSPAN15	0.670245306	8.42E-05	0.317389788	0.06294	0.608030989	0.00036
ALG10B	-0.409767467	8.71E-05	-0.203291856	0.04854	-0.079116586	0.44396
MACROD2	0.613885123	8.71E-05	-0.335608057	0.03518	0.498608405	0.00142
RTF1	0.352676292	8.75E-05	0.253082497	0.00455	0.36847953	4.04E-05

PAPD5	-0.451625305	8.79E-05	-0.186744651	0.09987	-0.31109796	0.00626
PANK1	-0.518850383	8.86E-05	-0.208081862	0.11063	-0.376784644	0.00404
DNAJA3	-0.53640172	9.07E-05	-0.256849576	0.05804	-0.103147638	0.44758
PFKFB3	0.852932802	9.23E-05	1.53727071	1.66E-12	1.269193941	5.77E-09
AMOTL2	0.846957573	9.25E-05	0.302418324	0.16346	-0.010643923	0.961
SLC5A2	1.888006477	9.30E-05	1.612835087	0.00095	1.524926809	0.00171
CEP76	-0.601195167	9.35E-05	-0.322724893	0.03281	-0.223438081	0.13876
LACC1	0.697153893	9.66E-05	0.409382182	0.02189	0.24364124	0.17543
SF3B2	-0.573621796	9.69E-05	0.027291934	0.85023	-0.282266538	0.05244
ZNF112	-0.496746611	9.72E-05	-0.17373767	0.15501	-0.370569303	0.00291
MDM1	-0.880375097	9.99E-05	0.192658823	0.38321	-0.474011797	0.03443
GEMIN6	-0.532027076	0.0001	-0.284522973	0.03278	-0.476388035	0.00038
BAIAP2L1	0.515354831	0.000103	0.322294748	0.01518	0.398805832	0.00265
CKAP4	0.31780308	0.000103	0.180230471	0.02736	0.167952883	0.04013
LUZP6	1.319060171	0.000103	0.495638761	0.15266	1.233097755	0.00028
CSTF1	-0.388058719	0.000105	-0.268593815	0.00673	-0.318717616	0.00135
ST5	0.614007116	0.000105	0.471039853	0.0028	0.649107873	3.96E-05
TMEM165	0.456402148	0.000106	0.204442936	0.08248	0.337225047	0.0042
ADAM8	1.111723537	0.000109	0.336713002	0.2437	0.769317879	0.00751
PHIP	-0.300449356	0.000114	-0.184530522	0.01725	-0.109351573	0.15852
CTSS	0.918853593	0.000115	0.536139179	0.02446	0.850504223	0.00036
PDE4B	1.595482424	0.000118	1.165138329	0.00485	0.394451275	0.35179
MEF2D	0.678690247	0.000118	0.237020696	0.17893	0.448558206	0.01106
PTP4A3	1.225997586	0.000121	1.131431743	0.00038	0.339614058	0.29114
MEGF9	-0.384799474	0.000122	0.054891299	0.56813	-0.0617799	0.52608
FEM1C	0.542146839	0.000125	0.491269222	0.00049	0.451458718	0.00139
FAM46C	-1.295878508	0.000125	-1.32909507	8.14E-05	-0.227481482	0.49829
MYO1C	0.412176743	0.000125	0.175377623	0.10246	0.379046747	0.00041
CENPE	-0.861174708	0.000128	-0.250665982	0.26404	-0.272213709	0.22529
GNPTAB	-0.388767628	0.00013	0.041651907	0.67921	-0.183002901	0.07032
VPS29	-0.340995468	0.00013	0.231694029	0.00781	-0.277489347	0.00172
RELT	0.949527936	0.000131	0.347513979	0.16138	0.350028922	0.16662
TRIT1	-0.550193066	0.000131	-0.301418702	0.03511	-0.175872713	0.21842
P11-39K24	1.908327678	0.000133	0.859247792	0.09777	2.127564878	1.60E-05
RNF207	0.6849542	0.000133	0.604031843	0.00072	0.982940603	3.34E-08
SRP9	-0.230184	0.000134	-0.046879403	0.43378	-0.279325777	3.48E-06
ZNF225	-0.577774243	0.000134	-0.130582655	0.36881	-0.170372229	0.24851
GPR108	0.519443693	0.000136	0.257168164	0.05923	0.153690618	0.26081
HS3ST3B1	0.894041924	0.000139	0.730565351	0.0018	-0.160880795	0.50607
WIZ	0.473586733	0.000141	0.144237437	0.24663	0.576641303	2.96E-06
MAPK14	-0.378873259	0.000141	-0.4189173	2.41E-05	-0.114589371	0.24645
FADD	-0.381180574	0.000146	-0.505275717	3.46E-07	-0.557462711	3.10E-08
UPF2	-0.356893074	0.000147	-0.211912765	0.02298	-0.252124169	0.00699
ATP10D	0.673223691	0.000148	0.533866375	0.00258	0.534221067	0.00265
CMPK2	-0.899042586	0.000148	-0.19360753	0.38156	-0.355096288	0.11344
ZNF37A	-0.471154271	0.000148	-0.115949171	0.34134	0.061139159	0.61567
B3GNT2	0.501352991	0.000153	0.160751709	0.2249	0.199040503	0.13356

CACYBP	-0.559739936	0.000153	-0.116527127	0.42488	-0.494248741	0.0008
TNKS1BP1	0.613162014	0.000155	0.552852706	0.00065	0.498163665	0.00212
CAPN7	-0.532103215	0.000156	-0.175523184	0.21017	-0.225256594	0.10806
H3F3B	0.384027485	0.000156	0.129134994	0.20322	-0.15571333	0.12553
CENPC	-0.368061998	0.000157	-0.027929232	0.76955	-0.190134491	0.04821
RNF44	0.560881345	0.000158	0.060326562	0.68624	0.398403867	0.00731
TAP1	0.815084849	0.00016	0.620627603	0.00402	0.232291845	0.2883
GOPC	-0.29785012	0.00016	-0.084703694	0.27529	-0.28238741	0.00032
RUNX1	0.641488836	0.000161	0.385718573	0.02324	1.001930036	3.40E-09
ZNF514	-0.500241959	0.000162	-0.151608642	0.24134	0.073213646	0.56927
GPR107	0.410526734	0.000163	-0.067003058	0.53951	0.38146567	0.00046
KLF3	0.38139209	0.000164	0.163616382	0.10586	0.303640951	0.0027
TD-2008A1	1.020233411	0.000166	0.254882466	0.34927	0.862330433	0.00146
WDR11	0.376743504	0.00017	0.519587245	2.04E-07	0.340185084	0.00067
FEZ1	1.05811239	0.000171	0.594196521	0.03541	0.483408809	0.08817
INO80C	0.633356616	0.000171	0.385974953	0.02179	0.297612381	0.07698
TTC30B	-0.64820183	0.000171	-0.410936287	0.01382	-0.429123207	0.0109
SPRY4	0.543194838	0.000183	0.317479302	0.02865	0.34840238	0.01654
RASA1	-0.321648721	0.000187	-0.107231073	0.21043	-0.24762907	0.00388
TRAPPC1	0.383290646	0.000187	0.026808923	0.79478	-0.020626841	0.84352
BLNK	0.88643212	0.000189	0.619061944	0.00916	0.89996723	0.00015
STAP2	0.59642359	0.000192	0.273005278	0.08816	0.301429048	0.0599
CTCF	-0.266606147	0.000194	-0.167369659	0.01809	-0.242524789	0.00066
LEO1	-0.333764623	0.000194	-0.183461579	0.03673	-0.468105812	1.95E-07
SLC30A7	0.379054257	0.000194	0.088264494	0.38477	0.198585048	0.05083
FOSL2	0.842266111	0.000195	0.50683151	0.02502	0.630465164	0.00529
DBT	-0.471571249	0.000197	-0.220164198	0.08096	-0.035864789	0.77545
MAST4	0.748321567	0.000207	0.915338112	5.50E-06	0.753547987	0.00018
COTL1	0.749442021	0.000208	0.214609863	0.28872	0.206838003	0.30682
PEF1	0.369382156	0.000209	0.020747695	0.83513	0.102326143	0.3071
SLC20A1	0.496696682	0.000211	0.199171487	0.13764	0.004607937	0.97265
ZNF138	-0.547994019	0.000211	-0.133572872	0.35532	-0.382877444	0.00881
KIAA0391	0.465380227	0.000217	0.177756701	0.15648	0.14278412	0.25656
MLX	-0.307837076	0.000217	-0.188379185	0.02142	-0.253164438	0.00218
ANKRD36B	0.675897587	0.000219	0.535157108	0.00346	0.438837867	0.01662
FAM136A	-0.46419771	0.000219	-0.296043035	0.01751	-0.316867569	0.01123
CCSER2	0.420199074	0.000222	0.318628102	0.00508	0.135247629	0.23477
SNX30	-0.684247957	0.000223	-0.468199552	0.01147	-0.717439914	0.00011
PDE8A	0.513147817	0.000224	0.367964826	0.00816	0.550184354	7.52E-05
EGR2	1.309462517	0.000226	0.726546579	0.04133	0.365437459	0.30791
PEX1	-0.477754347	0.000227	-0.04495096	0.72411	-0.054778929	0.66934
PISD	0.484095358	0.000229	-0.090091714	0.49478	0.123989847	0.3472
HARS2	-0.357988083	0.00023	-0.187782853	0.04898	-0.299759305	0.00197
CRNKL1	-0.440238808	0.000231	-0.43464814	0.00026	-0.480021228	5.88E-05
ZNF555	-0.576257352	0.000231	-0.018114242	0.90505	-0.320836229	0.03693
LYPD6B	0.845254952	0.000233	1.263698479	2.50E-08	0.94264677	3.70E-05
UBL4A	-0.501114413	0.000232	-0.465290167	0.00046	-0.358192201	0.00734

SLFN11	1.433658632	0.000234	1.598551662	4.05E-05	1.969878153	4.23E-07
PVRL2	0.433660409	0.000235	0.291565972	0.01335	0.229999834	0.05128
PRKD3	0.509802861	0.000237	0.462805	0.00083	0.327966439	0.01814
BTN2A1	0.425449022	0.000238	0.022311792	0.84749	-0.085673595	0.46237
MAP10	-0.69679068	0.00024	-0.481563648	0.0078	-0.37522005	0.03896
SSX2IP	-0.42648532	0.000242	-0.493003998	1.93E-05	-0.292416563	0.01131
POLG2	-0.650296541	0.000244	-0.139470195	0.39682	0.274598768	0.09079
ESRP2	-0.461547714	0.000247	-0.296692894	0.01776	-0.098759566	0.42967
XPO1	-0.324701522	0.000248	0.09867221	0.26447	-0.159639629	0.07125
ZNF813	-0.673895082	0.000251	-0.576811121	0.00156	-0.281832615	0.12144
RASGEF1B	0.54072922	0.000256	0.474213051	0.00136	0.427149169	0.00401
RTKN2	-0.626226332	0.000257	-0.152853808	0.36276	-0.196275075	0.24458
CAMK2G	-0.666405802	0.000257	-0.380217831	0.03584	-0.352349313	0.05196
ANKRD42	-0.556102159	0.000257	-0.330833835	0.02832	-0.146639476	0.33064
P11-680E19	-1.984623833	0.000262	-1.34771088	0.00763	0.101469816	0.82858
ZNF593	0.666101988	0.000265	0.063122814	0.73245	0.030374249	0.87025
DCTN1	0.533202747	0.000266	0.29595891	0.04291	0.222910361	0.12762
AP006216.1	-1.398019052	0.00027	-0.741592116	0.04928	-1.325322893	0.00052
FAM203A	-0.560117706	0.000275	-0.393773803	0.00901	-0.446018855	0.00333
HIC2	-0.475350098	0.000282	-0.584506111	7.37E-06	-0.195507673	0.13191
LRRC37A6P	-1.369028714	0.000289	0.047497437	0.88544	-0.948680915	0.00916
FAM185A	-0.668980736	0.00029	-0.1218924	0.49336	-0.354062492	0.04692
SOX21	-1.301640444	0.00029	-0.658160189	0.06473	0.202560969	0.56832
CCT8P1	-0.97741627	0.000293	-0.651300911	0.01028	-0.346809881	0.1666
AC005280.1	-0.4068017	0.000296	-0.5347725	1.45E-06	-0.453995699	4.77E-05
ATP8B5P	1.736453735	0.0003	0.712691689	0.14956	1.282115998	0.00838
MGAT4B	0.372503767	0.000305	-0.17620647	0.08899	0.40488074	8.45E-05
PRDX1	-0.473202198	0.000306	-0.383269276	0.00341	-0.523961193	6.41E-05
ZSWIM6	0.372017328	0.000306	-0.005159716	0.96018	0.179725388	0.08171
KIAA0586	-0.463282663	0.000308	-0.111396297	0.37427	-0.183058748	0.14951
LAPTM4B	1.304712454	0.000309	0.757200219	0.03674	0.686557375	0.05836
ZNF273	-0.498012516	0.000311	-0.219854756	0.10079	-0.181752866	0.17749
CROT	-0.828809982	0.000313	-0.45731298	0.04464	-0.217737514	0.33962
ZNF486	-0.543359486	0.000314	-0.38484385	0.00972	-0.196762837	0.18619
PARS2	-0.498630594	0.000316	-0.348889388	0.00826	-0.356418783	0.00793
PLEKHG3	0.484357959	0.000318	0.455720666	0.00069	0.48135469	0.00034
MAP1LC3B	0.627144777	0.00032	0.27152802	0.1196	0.233074261	0.18244
SEPHS2	-0.50639728	0.00032	-0.308756306	0.0263	-0.063099827	0.64912
ZNF493	-0.480483658	0.000319	0.020169928	0.87785	0.206443519	0.11558
PPP2R1A	0.308196269	0.000321	0.117286674	0.17048	0.216541716	0.01146
GATA5	-1.6908684	0.000323	-1.088761945	0.01588	0.344447755	0.43149
FAM53C	0.449061626	0.000327	-0.02221029	0.85942	-0.039600545	0.752
PIM3	1.125785633	0.000329	0.505084006	0.10749	0.51792461	0.09902
ZNF845	-0.654714663	0.000328	-0.43978787	0.01521	-0.419994673	0.02046
GDF15	1.343379041	0.00033	0.535429972	0.15263	1.157142453	0.00199
IKBKB	0.563332862	0.000331	0.514231742	0.00102	0.554411123	0.00041
ZBTB41	-0.410689033	0.000336	-0.474752201	3.06E-05	-0.291898453	0.01036

HNRNPA3	0.478743164	0.000337	-0.106534704	0.4249	0.420479436	0.00161
RPUSD2	-0.531148787	0.000344	-0.571867807	8.53E-05	-0.63983267	1.56E-05
DAGLB	0.436999736	0.000347	0.134929669	0.26549	0.463981692	0.00012
ZNF184	-0.404154915	0.000347	-0.022165734	0.83893	-0.448725538	6.45E-05
WDR5B	-0.506642612	0.000349	-0.145217105	0.28025	-0.009769134	0.94217
CDK19	0.349542922	0.000351	0.073483852	0.45115	0.256295121	0.00863
PHC1	0.855395298	0.000363	0.83315439	0.00051	0.605249436	0.01167
GPR180	-0.324824806	0.000364	-0.153492028	0.08691	-0.287478446	0.0015
CDV3	0.333954852	0.000365	0.14687888	0.11689	-0.085837099	0.36011
CHMP4B	0.281799001	0.000368	0.120781829	0.12543	-0.044292164	0.57818
PLEKHA7	-0.689332182	0.000379	-0.448469671	0.02024	0.041201288	0.83083
TANK	0.410173587	0.00038	0.286798829	0.01269	0.163896204	0.15721
AGL	-0.299479993	0.000383	-0.095259033	0.25266	-0.028122206	0.73664
IMMP1L	-0.451894102	0.000383	-0.454064194	0.00022	-0.252474123	0.04109
MARS	0.452688889	0.000383	0.37948681	0.00282	0.231305471	0.06945
OSMR	0.864458016	0.000383	0.553759552	0.02296	0.604835522	0.01301
PSMD1	1.039881904	0.000384	0.073890646	0.80074	0.809957461	0.00571
TNFAIP1	0.476449653	0.000385	0.128182763	0.33959	0.048352215	0.71968
TGIF1	0.431630958	0.000388	-0.397226886	0.00118	-0.242534654	0.04806
CLDN12	0.463452802	0.000393	-0.06170153	0.6383	0.358383017	0.00618
NDUFV2	0.422497589	0.000392	0.234661721	0.04858	0.209915227	0.07843
MAL2	2.90596238	0.000393	1.916308796	0.01977	1.71803067	0.03692
LSM3	-0.417539922	0.000397	0.114960005	0.32062	-0.4892227	3.23E-05
CCDC138	-0.516299018	0.000398	-0.301972368	0.03474	-0.40242298	0.00521
RPUSD3	-0.312747714	0.0004	-0.139453333	0.10063	-0.375782296	1.68E-05
N6AMT1	-0.503828924	0.000405	-0.247233545	0.07314	-0.284027261	0.04203
PTBP3	0.477295492	0.000414	0.01949533	0.88532	0.125580661	0.35286
ISCA1	-0.415623507	0.000415	-0.414499123	0.00039	-0.460490981	8.95E-05
PEA15	0.460831423	0.000415	0.226489188	0.0824	0.004987776	0.9696
ADM	1.277788895	0.000422	1.591963855	1.05E-05	1.123531236	0.00195
RSBN1L	-0.510296113	0.000426	-0.031845019	0.82401	-0.413456881	0.00398
CLDN4	0.640657268	0.00043	-0.105568474	0.56218	0.138283047	0.44761
WIBG	-0.329277747	0.00043	-0.379960473	3.91E-05	-0.262149565	0.00467
PPRC1	0.468022784	0.000436	-0.108146068	0.41758	0.454991208	0.00062
ZNF724P	-0.470712462	0.000435	0.003857406	0.97647	-0.166615874	0.20786
P11-761N21	20.27289824	0.000439	19.9014056	0.00056	19.26205544	0.00084
CWF19L2	-0.37090818	0.000442	-0.189825937	0.06638	-0.399207001	0.00014
CBX3	-0.241976388	0.000446	-0.088914863	0.19338	-0.302937226	1.08E-05
MAPK1IP1L	0.615472566	0.000446	0.282709014	0.10675	0.326350922	0.06258
SLC12A2	0.69515615	0.000445	0.197490033	0.31853	0.751337133	0.00015
ARL5B	0.359672893	0.000451	0.206459643	0.04393	-0.155230551	0.13074
ZNF706	0.356362741	0.000454	0.333397873	0.00099	0.058741007	0.56384
FASTKD1	-0.505023594	0.000457	-0.396656684	0.00547	-0.282030937	0.0476
EEF1A1P9	-0.630351324	0.000462	0.220835385	0.18431	-0.615941932	0.00052
ZNF780A	-0.62635093	0.000462	-0.372934991	0.03539	-0.235055178	0.18246
CAPZB	0.261506524	0.000469	0.103211881	0.16605	0.165167103	0.02701
MLYCD	-0.468052033	0.000477	-0.388977751	0.00288	-0.086762572	0.50536

ITGAV	0.611800241	0.000479	0.353422927	0.04364	0.045284355	0.79611
BTBD19	0.571481142	0.000479	0.331115875	0.04329	0.546217788	0.00082
MGAT2	-0.271310236	0.000482	-0.275271744	0.00034	-0.260468812	0.00075
ZNF721	-0.452133916	0.000488	-0.189311528	0.14122	-0.125808347	0.32713
C10orf10	0.930496655	0.000495	0.642689902	0.01613	0.677952258	0.01132
PDGFB	2.438046833	0.000495	1.765149697	0.01178	1.967229744	0.00499
RMDN2	-0.626846439	0.000501	-0.264966341	0.12637	0.097537227	0.57728
SAMD4A	0.842349122	0.000501	0.808610242	0.00083	0.333080113	0.16933
KNOP1	-0.490809737	0.000502	-0.486826113	0.00054	-0.474932071	0.00074
GCKR	1.147848316	0.000506	0.127530788	0.70535	1.096244142	0.0009
DENND3	0.769778927	0.000508	0.586118707	0.00789	0.890249458	5.53E-05
LARP6	0.826056723	0.000508	0.837426315	0.00039	0.094633293	0.69265
GNA15	1.572418424	0.000512	1.03249435	0.02291	1.443669498	0.00143
CNKSR1	0.823260189	0.000516	0.345142577	0.15012	0.884089311	0.00019
C9orf40	-0.6835022	0.00052	-0.260767085	0.1532	-0.478157682	0.01154
KCTD11	0.761920816	0.000525	1.147323012	1.36E-07	0.556392517	0.01144
PPFIBP2	-0.770584521	0.000529	-0.259980181	0.23975	-0.217982041	0.32445
ZNF10	-0.667038281	0.000531	0.052170342	0.78043	-0.110308275	0.56034
PLCXD1	0.704953214	0.000532	-0.267938852	0.18857	0.128482796	0.52801
PPIE	-0.320268293	0.000539	-0.363362336	5.63E-05	-0.376988905	3.39E-05
HAUS6	-0.400358976	0.000547	-0.20303952	0.07669	-0.370438505	0.00136
MRPL27	-0.417591153	0.000549	-0.280858355	0.01865	-0.572280548	2.15E-06
MRPS18C	-0.413627494	0.000549	-0.241498922	0.04088	-0.456933128	0.00012
ERI2	-0.375710937	0.00055	-0.065403282	0.53611	0.062358193	0.55675
ETF1	0.271763431	0.000553	0.258561216	0.00099	-0.04547632	0.56443
TBCE	-0.362247699	0.000554	-0.170131137	0.0963	-0.210302179	0.04259
EPS8L3	0.624809146	0.000558	-0.396488269	0.0291	0.202798826	0.26337
EEA1	-0.432677295	0.000559	0.183072768	0.14153	-0.091610722	0.46366
FOXJ1	-1.64901596	0.000566	-2.364351032	1.83E-06	-1.285348325	0.00553
DENND5A	0.573756233	0.000568	0.514620928	0.00197	0.228913568	0.16985
AURKA	-0.616561342	0.000573	-0.442316875	0.0129	-0.602822651	0.00074
NR3C2	0.965582672	0.000579	0.769759907	0.00601	0.877243541	0.00169
RFX5	0.40311504	0.000583	0.258781156	0.02692	0.298972703	0.01076
G6PC3	0.392632449	0.000584	0.1233542	0.28011	0.045069253	0.69476
ARHGEF19	-0.505579429	0.000586	-0.702734827	1.81E-06	-0.299790286	0.04006
SH2D4A	0.679254648	0.00059	0.498766903	0.01168	0.470366631	0.01738
DTX4	-0.540505682	0.000596	-0.224558564	0.15297	0.043915321	0.77928
GNPDA2	-0.386197625	0.0006	-0.220565823	0.04599	-0.422792283	0.00015
TLR3	1.086675541	0.000602	1.064595417	0.00066	1.075814019	0.00057
AGFG1	0.412899566	0.000605	0.089685674	0.45634	0.207965215	0.08399
CDC23	-0.28910263	0.000605	-0.120774128	0.14496	-0.273382376	0.00112
MAP3K11	0.383283414	0.00061	0.103266582	0.35803	0.332346863	0.00293
PTBP1	0.4409333	0.00061	0.351974157	0.00616	0.315402022	0.01419
NET1	0.448933052	0.000612	0.409650216	0.00175	0.241966768	0.06486
TNFRSF10A	0.786494428	0.000617	0.630764047	0.00608	0.297021151	0.19795
ZNF141	-0.356229212	0.000629	-0.166891396	0.10257	0.020686279	0.83967
DUSP2	1.252281782	0.000632	-0.000727889	0.99844	0.365185821	0.32321

RCL1	0.621169148	0.000635	0.134647815	0.46125	0.418248721	0.02148
ANAPC4	-0.523270365	0.000636	-0.280383615	0.06365	-0.04767368	0.75333
DENND4C	-0.41765179	0.000637	-0.144283339	0.237	-0.065676807	0.59019
KANK1	-0.495883851	0.000641	-0.231706055	0.1064	-0.48011658	0.00087
CCDC121	-0.664528773	0.000652	0.080389571	0.66022	-0.338741423	0.07219
RNF113A	-0.548719346	0.000663	-0.578643628	0.00025	-0.459128956	0.00372
TTC39B	0.422038218	0.000668	-0.007078854	0.95465	0.223885704	0.071
GCN1L1	0.367026098	0.000676	0.160711474	0.13672	0.416254851	0.00011
COX19	-0.657024436	0.000679	-0.567292551	0.00316	-0.398099576	0.03824
GPR137	0.736834637	0.000681	0.801725284	0.00021	0.379815337	0.08077
CEBPB	0.832764374	0.000684	0.686914859	0.00497	0.559455305	0.02298
GPALPP1	-0.346231655	0.000689	0.020606026	0.83707	-0.136376819	0.17729
STAMBPL1	-0.646195278	0.000699	-0.599946062	0.00146	-0.177559481	0.34419
KIAA0040	1.480508214	0.000701	1.108423158	0.01143	-0.561052502	0.22985
KLHDC7A	-0.85967138	0.000704	-0.58886972	0.01693	-0.188263748	0.44054
NELFE	0.28007279	0.000712	-0.062478811	0.45106	-0.132200767	0.11462
MAP2K3	0.79148941	0.000716	0.207418687	0.38059	0.445449089	0.05816
P11-1220K2	1.411071573	0.00072	-0.049287924	0.90615	1.497833443	0.00033
TCEA1	-0.294130024	0.000723	-0.245040656	0.0046	-0.273325242	0.00163
DAB2	0.877123997	0.000737	0.586225477	0.02407	0.34852948	0.18079
EMP3	0.524402443	0.000741	-0.369771161	0.01833	-0.350210289	0.02583
MLEC	-0.37027311	0.000741	-0.538801023	9.12E-07	-0.267939948	0.01456
CCDC43	-0.379859029	0.000753	-0.114634655	0.30047	-0.430036842	0.00013
ROCK2	-0.428215164	0.000753	-0.354077361	0.00526	-0.489140013	0.00012
DDR1	0.402532864	0.000755	0.270559496	0.02351	0.331767228	0.00551
PALB2	-0.583435155	0.000761	-0.250691321	0.13976	-0.259339663	0.12938
PER1	1.062475245	0.000767	0.938361635	0.00302	1.035848968	0.00105
RCE1	0.442026194	0.000769	-0.03643554	0.78425	-0.045231391	0.73636
AGPAT3	0.336831114	0.0008	0.126886847	0.20584	0.474707351	2.03E-06
GJC1	0.589984732	0.000807	0.498711027	0.00453	0.338566563	0.05502
ZNF33A	-0.374811269	0.000809	0.121523905	0.27058	-0.197972112	0.0752
SUZ12P	-0.378698014	0.000813	0.093015419	0.38891	0.346919823	0.00126
AGGF1	-0.314713121	0.000817	0.069188208	0.4537	-0.215695791	0.0209
MRPS14	-0.377572859	0.000818	-0.219731755	0.04514	-0.312543053	0.00503
TMEM127	0.308544555	0.000825	-0.019562525	0.83192	0.210519261	0.02202
ADIPOR2	0.397476862	0.000827	0.011727343	0.92151	0.190700136	0.1091
ABCD3	-0.300127866	0.000833	-0.247576506	0.00561	-0.145927538	0.10293
BRICD5	-0.677892381	0.000834	-0.710246515	0.00036	-0.179919761	0.36039
RAB5B	0.210315998	0.000833	0.01032401	0.86979	0.121692666	0.05318
ZNF69	-0.545526307	0.00084	-0.427127904	0.00792	0.018503186	0.90757
FAM177A1	0.371512996	0.000855	0.306011291	0.00578	0.190755156	0.08699
CSRP2BP	-0.449431221	0.000862	-0.027929481	0.83171	-0.395584462	0.0031
DDX21	0.456741196	0.000863	0.379470933	0.00562	0.024148119	0.86026
MYB	1.24047372	0.00087	0.942432994	0.01146	0.52335115	0.16304
RANBP6	-0.354858152	0.00088	-0.205744369	0.05103	-0.346868285	0.0011
SETD5	0.360308709	0.000888	0.366536798	0.00071	0.528450604	1.01E-06
NECAP2	0.368841596	0.000889	0.15414353	0.16429	0.000667974	0.99522

SLAIN1	-0.670918892	0.000891	-0.260372446	0.19381	-0.30210399	0.13079
PDCD6IP	0.312529123	0.000895	0.310715647	0.00094	0.004584795	0.96116
HAS3	1.708579977	0.000902	0.95129468	0.0652	1.864660102	0.00029
ZBTB7A	0.479236961	0.000903	0.275172092	0.05643	0.454188805	0.00164
TNNT2	1.204416236	0.000905	-0.174273787	0.63384	0.370030614	0.31001
SFRP2	3.164463058	0.000907	4.663515167	8.43E-07	2.386404531	0.01286
SENP2	-0.518016641	0.000912	-0.056045323	0.71897	-0.398605181	0.01069
IRX2	-1.333156318	0.00092	-0.822010556	0.03877	-0.526586189	0.18488
TBC1D22B	0.441176683	0.000926	0.225025255	0.09023	0.18517731	0.16694
VPS26B	-0.275115753	0.000929	-0.04726518	0.55887	0.056697123	0.48645
SLC9A8	0.731189351	0.000932	0.409021208	0.064	0.63426733	0.00414
ZNF200	-0.440617186	0.000948	-0.293543219	0.02488	-0.1441828	0.26896
GKN1	-4.524989034	0.000951	2.225947304	0.09913	-3.184989444	0.01891
NKRD36BF	0.78476271	0.000958	0.731658841	0.00199	0.835185606	0.00042
C5orf22	-0.355496453	0.000957	0.052233101	0.62004	-0.072629238	0.4965
RPL38	0.445454384	0.000971	0.149426908	0.26848	0.289232537	0.0322
FAM179B	-0.551253108	0.000973	0.104645037	0.5297	-0.401244082	0.01613
ARIH1	0.308785712	0.000975	0.188604763	0.04343	0.251227511	0.0072
TRMU	-0.434097163	0.000982	-0.212995036	0.10267	-0.190632921	0.14394
C1orf131	-0.589556958	0.000986	-0.368004338	0.03759	-0.486296946	0.00612
CSNK1G2	0.467903288	0.000989	0.325219127	0.02194	0.157914966	0.26753
USP7	0.318353093	0.001	0.265849297	0.00587	0.282653078	0.00345
ZNF484	-0.38829799	0.00101	-0.114651928	0.31403	-0.187213959	0.1038
PKDCC	-0.640868821	0.001011	-0.995953737	3.25E-07	-0.757554194	0.0001
CCDC112	-0.916749532	0.001028	0.012444654	0.96341	-0.932329306	0.00078
LPHN1	0.407889437	0.001035	0.232940128	0.05942	0.494618435	6.33E-05
MAP3K4	-0.47126016	0.001034	-0.073564267	0.60685	-0.278768815	0.05178
OARD1	-0.464560324	0.001034	-0.050951991	0.71535	-0.242012087	0.08455
ELOVL7	0.719508745	0.001051	0.161025084	0.46518	0.217825679	0.32431
MEX3C	0.667748378	0.001056	0.516542679	0.01133	0.251929721	0.21867
ESRRA	0.414986085	0.001058	-0.149954616	0.23903	0.284831886	0.02495
KCNK1	0.687262141	0.001062	0.397734267	0.05794	0.173274371	0.41056
RAB30	1.002249638	0.001069	0.018232526	0.95285	-0.013275807	0.96609
MPHOSPH9	-0.44817383	0.001072	0.108782511	0.4213	-0.24806585	0.06881
OLFM4	3.083963121	0.001074	1.040015365	0.27211	1.236075592	0.19172
THG1L	-0.335330693	0.001081	-0.080894203	0.41591	-0.455638127	7.47E-06
MFSD8	-0.442958416	0.00109	-0.226043737	0.09205	-0.255267267	0.05739
KLHL11	-0.388690875	0.001106	-0.381426692	0.00114	-0.266269838	0.02342
NPTN	0.297257595	0.001112	0.266403988	0.00338	0.202952774	0.02589
C1orf35	-0.519948534	0.001118	-0.278119095	0.06975	-0.170218783	0.26994
CRTC2	0.710166418	0.001118	0.586152694	0.00722	0.665468416	0.00227
SLC25A46	-0.277118094	0.001133	0.017152789	0.83867	-0.202896171	0.01651
R3HDM2	0.568733642	0.001141	0.42537379	0.01472	0.598925136	0.00057
SPAST	-0.374250928	0.001148	-0.370673852	0.00115	-0.262582594	0.02145
SDCBP2	0.565329592	0.00115	0.159380725	0.3597	-0.003517964	0.98392
LRRC58	-0.256193068	0.001154	-0.094426865	0.22508	-0.072396878	0.35393
VEGFA	0.716413096	0.001163	0.203143237	0.35729	0.64283221	0.00356

FLRT3	-0.526001336	0.001166	-0.347365031	0.03139	-0.604322869	0.00019
GATAD1	-0.311208889	0.001165	-0.108802717	0.24997	-0.051403837	0.58724
CLK3	0.487758578	0.00117	0.712334816	1.95E-06	0.512291215	0.00064
EID3	1.326542477	0.00117	0.531264447	0.20343	0.859265834	0.03807
PPP2R5C	-0.301817685	0.001173	-0.076283589	0.41012	-0.27602462	0.00298
EPN3	-1.068145067	0.001179	-0.818859651	0.00936	-0.530908234	0.09651
ZFP69	-0.616781126	0.001199	-0.234554375	0.1979	-0.515110893	0.00583
PLEKHB2	0.291364904	0.001205	0.007999803	0.92919	0.055685112	0.53673
ACOT11	-0.349387943	0.001206	-0.451676556	2.45E-05	0.114213088	0.28209
PHF3	-0.291782298	0.001235	-0.095940685	0.28595	-0.241064089	0.00752
PARG	-0.622116878	0.001248	-0.141807472	0.45725	-0.24449572	0.20248
SH3TC1	0.784723855	0.001252	0.278648041	0.25315	0.614435967	0.01169
RAB32	0.554873144	0.001269	0.221477539	0.19936	0.027932901	0.87371
SNRNP48	-0.32321586	0.001269	0.15658507	0.10685	-0.221692921	0.0255
ZBTB7C	0.884039544	0.001268	0.329769129	0.23033	0.879486935	0.00134
REM2	1.237347115	0.001289	0.70880359	0.0675	0.192912659	0.63058
LONRF1	0.432614676	0.001298	0.500415265	0.00019	0.320010378	0.01742
AZIN1	0.369693047	0.0013	-0.080473762	0.48357	0.107465717	0.35001
EYA3	0.363186795	0.001322	0.447254337	7.25E-05	0.27927216	0.01338
KIF21A	0.501630437	0.001326	0.213851062	0.17117	0.412872451	0.0083
CPOX	-0.494557089	0.001341	-0.408641056	0.00776	-0.054744876	0.72016
COIL	-0.272819036	0.001343	-0.315274205	0.00018	-0.381060382	7.36E-06
P11-85G20	1.134059237	0.001343	-0.35045274	0.38281	1.091265337	0.00188
TOLLIP	0.421828559	0.001373	-0.033913798	0.79749	0.340936609	0.00974
CSNK1D	0.362864627	0.001379	-0.047725228	0.67412	0.443763293	8.73E-05
PLEKHM2	0.433201372	0.001381	0.263938654	0.05099	0.470522593	0.0005
NR4A3	2.698989346	0.001386	2.438780298	0.00385	1.562192012	0.06694
TEP1	-0.562190106	0.001391	-0.187519505	0.28529	0.029338934	0.86702
TFAP4	-0.67917455	0.001405	-0.492482816	0.01901	-0.652153376	0.00185
X1A-SULT1	-0.602268019	0.001424	-0.456508196	0.01408	0.114413281	0.53367
BUD13	-0.329691877	0.00143	-0.373721079	0.00026	-0.37749268	0.00024
C5orf51	-0.261513181	0.001428	-0.260274256	0.00121	-0.223937532	0.00573
HYLS1	-0.520752699	0.001431	-0.428255053	0.00698	-0.296329675	0.06255
MAP2K7	0.850764983	0.001432	-0.077368388	0.77245	0.681470715	0.01064
THUMPD1	-0.286707733	0.00144	-0.302672492	0.00073	-0.378900157	2.53E-05
CITED2	-0.636517722	0.001444	-0.324975604	0.10128	-0.085769678	0.66507
NKRD20A5	-1.78778954	0.001455	-0.237044148	0.64466	-0.469905299	0.36442
ALG6	-0.317096409	0.001464	-0.102069155	0.28181	-0.350344833	0.00039
ATP1A1	0.406727267	0.001465	-0.182905457	0.15267	0.158488053	0.21514
DCLRE1A	-0.483681971	0.001466	-0.072401543	0.62817	-0.014393524	0.92336
PRSS22	0.735084683	0.001462	0.016152211	0.94477	0.393051305	0.09044
SRD5A3	0.596811536	0.001466	0.930213143	6.53E-07	0.500504523	0.00765
ATM	-0.390546105	0.001476	-0.009756445	0.93652	0.048427306	0.69295
SPG21	0.304136317	0.001486	0.086414115	0.36585	0.137097311	0.15311
AMER1	-0.395320235	0.001493	-0.378793812	0.00216	-0.127089091	0.30187
BCL2L14	0.616885942	0.001494	0.20943883	0.28223	0.582957408	0.00269
ZNF419	-0.506233349	0.001494	-0.339940544	0.02988	-0.223243396	0.15458

ARF3	0.336479297	0.001499	0.091739701	0.38662	0.018582105	0.86104
COBLL1	-0.609696446	0.001507	-0.508871148	0.00792	-0.00695815	0.97097
LGALS9C	1.863084575	0.001512	1.363041421	0.02018	1.971771413	0.00073
PLEKHM1	0.772668385	0.001513	0.00599375	0.98054	0.391666319	0.11004
ZZZ3	-0.287509786	0.001514	-0.158364128	0.07868	-0.153703326	0.08893
SERTAD1	0.609671973	0.001515	-0.144536831	0.45815	0.01854166	0.92415
ASB13	-0.630314329	0.001527	-0.567966403	0.00383	-0.296174077	0.13303
CDCA7	-0.641520322	0.001555	-0.217847989	0.27873	-0.34771173	0.08433
MICAL3	0.497781823	0.001555	0.137600177	0.38131	0.459673753	0.00336
RBM34	-0.429336463	0.001561	-0.130166884	0.33195	-0.350554099	0.00962
VPS28	0.291133129	0.001566	-0.071584449	0.43809	-0.05550479	0.55068
FRAT1	-1.143385845	0.00157	-0.621606267	0.06572	0.055356633	0.87118
AREG	1.710369946	0.001572	0.717649323	0.18541	-0.170096057	0.75453
ARHGAP11/	-0.558856829	0.00158	-0.005507136	0.97503	-0.402317966	0.02255
IDH1	-0.392659823	0.001597	-0.044064856	0.72275	-0.18439466	0.13791
WTAP	0.280583337	0.001627	-0.060717545	0.49575	0.04395042	0.62241
FOS	0.702604592	0.001629	0.610978056	0.0061	0.74929877	0.00077
MTRNR2L1	1.298362531	0.001638	-0.05695047	0.89472	0.663544225	0.11352
FBXO25	-0.637867377	0.001653	-0.400986341	0.04681	-0.430276269	0.0331
CCNI	0.342303844	0.00166	-0.074817711	0.49201	0.364290901	0.00081
HEG1	0.836964109	0.001658	0.63077047	0.01738	0.342146693	0.1999
ZNF513	0.543655789	0.00166	0.075327231	0.66314	0.113981886	0.51212
PIWIL2	-1.567069128	0.001662	-0.162737686	0.72478	0.646821511	0.15555
A16c-17H1.	-0.838996307	0.001689	-0.398413137	0.11307	-1.20790784	1.03E-05
FAM107B	0.537190478	0.001708	0.441895031	0.00984	0.292378688	0.08801
LRRC47	-0.463909931	0.001716	-0.154702287	0.29288	-0.152279161	0.301
TADA1	-0.324868426	0.001716	-0.194473684	0.05364	-0.128976573	0.20216
THBS1	0.910506592	0.001714	0.245277411	0.3983	0.237533789	0.41337
ZNF84	-0.347471904	0.001722	-0.111479902	0.31041	-0.194551475	0.07803
GAS2L3	-0.791527629	0.001738	-0.287650498	0.25336	-0.219187665	0.3842
BRK1	0.211150746	0.001747	0.0371787	0.58022	-0.037566253	0.57923
CDH8	1.036603181	0.001747	0.308896405	0.35377	0.464878273	0.16556
FRG1	-0.304578448	0.001753	0.09247335	0.30165	-0.409205652	2.69E-05
ZBTB8B	-0.620332055	0.00176	-0.196830043	0.30267	0.063105233	0.73994
RNASEL	-0.392867945	0.001774	-0.279604837	0.02166	-0.015317069	0.89915
POT1	-0.501465929	0.001778	-0.334668615	0.03558	-0.437015044	0.00632
DFFB	-0.608100069	0.001781	-0.398390316	0.03737	-0.203882144	0.28251
ELP5	0.285930252	0.001789	-0.034416534	0.70871	-0.008562397	0.92624
PHC2	0.33192635	0.001799	0.157317079	0.13861	0.187305906	0.07843
PSMC4	0.379489983	0.001806	0.049926663	0.68177	0.069395921	0.56992
MRPL44	-0.301325882	0.001811	-0.400490955	2.83E-05	-0.284361966	0.00301
MTRF1	-0.431979887	0.001821	-0.230722531	0.0893	0.15954386	0.23539
C6orf89	0.265425314	0.001832	0.090638161	0.28572	-0.023060917	0.78668
ZNF331	-0.431083762	0.001839	-0.081364319	0.54899	0.126522201	0.35073
NME4	0.566702334	0.001847	-0.272509263	0.13581	0.039378683	0.82913
CCDC90B	-0.270094917	0.001852	-0.207392905	0.01514	-0.121644029	0.15386
IL6ST	0.431463199	0.00186	0.326315064	0.01857	-0.031984381	0.81763

CIC	0.480397762	0.001862	0.155301068	0.31442	0.698836068	5.65E-06
ARRDC2	0.723689473	0.001867	0.309698371	0.18588	0.379035855	0.10576
ASTE1	-0.79750942	0.001865	-0.442482931	0.07924	-0.527374945	0.03722
KDELR1	0.35656007	0.0019	-0.267364532	0.02003	0.13650189	0.23484
LSR	0.526542401	0.001903	0.139894909	0.40956	0.514074472	0.00243
POC5	-0.503929324	0.0019	0.193652952	0.21349	-0.336129701	0.03433
RPL14	0.367414259	0.001903	-0.199902637	0.09116	0.075365862	0.52427
ZNF33B	-0.769250975	0.001904	-0.950948831	0.0001	-0.647667405	0.00895
FLOT2	-0.471714696	0.001908	-0.468873765	0.00193	-0.24507304	0.1053
POU2F3	1.671877112	0.00191	1.587897775	0.00315	0.615635903	0.25832
ZNF252P	-0.378426907	0.001917	-0.158039929	0.18977	-0.222146383	0.06547
TAB2	0.336070573	0.001921	0.208898148	0.05352	0.335007836	0.00197
HELLS	0.703612363	0.001923	0.393893881	0.08252	0.660556915	0.00359
LRRC4	0.916583001	0.001934	0.446500103	0.13096	0.341059339	0.25035
UTP14C	-0.236656665	0.001944	-0.061179862	0.41267	-0.051317648	0.49473
NDNL2	-0.447474947	0.001958	-0.405230147	0.00445	-0.6711798	3.78E-06
RHOV	1.435377253	0.001962	2.015373141	1.17E-05	0.919142588	0.04873
C1orf109	-0.475703598	0.001967	-0.252450606	0.09545	-0.368308881	0.01561
TTC4	-0.324884013	0.001978	-0.282622988	0.00637	-0.555862753	1.29E-07
INTS6	-0.26159187	0.001982	-0.145945189	0.08298	-0.183347489	0.0297
ZSCAN29	-0.293134422	0.001997	-0.264975677	0.00448	-0.165901174	0.07631
CA13	-0.486434461	0.002018	-0.750548993	2.08E-06	-0.262261426	0.09377
COL9A2	0.856218267	0.002026	0.428459515	0.1233	0.66416123	0.0168
ZNF625	-0.773852006	0.002036	-0.215521952	0.37466	-0.506988472	0.03917
JADE2	-0.563647268	0.002038	-0.638451892	0.00048	-0.20140369	0.26812
CHKA	-0.473313412	0.00204	-0.258480153	0.09013	-0.243015842	0.11093
C2orf44	-0.411340062	0.002047	-0.172651767	0.18162	-0.419569687	0.00148
RSC1A1	-0.547691163	0.00205	-0.129077996	0.45431	-0.079305362	0.64688
PQLC1	0.423807889	0.002061	0.284519224	0.03851	0.038969635	0.7806
LIN9	-0.486362036	0.002066	0.033223909	0.82891	-0.344463793	0.02751
MAP4K4	0.391677805	0.002067	0.527043734	3.27E-05	0.117646115	0.3552
EPB41L2	-0.472774171	0.00207	0.150660421	0.32448	-0.416444668	0.00662
TMEM184B	0.455731672	0.002103	0.61194247	3.59E-05	0.560957986	0.00015
TMEM57	-0.404603213	0.002106	-0.180382557	0.16754	-0.270676067	0.03947
ZHX1	-0.297785411	0.002118	-0.243326981	0.0115	-0.149147223	0.12197
EPHA1	-0.500640229	0.00212	-0.419723434	0.00959	-0.089136752	0.5817
KLHL21	-0.549262863	0.002122	-0.468879682	0.00843	-0.236725384	0.18302
MTATP6P1	0.9675036	0.002132	-0.078905276	0.80375	0.738825939	0.01911
SLC7A1	0.521692562	0.002134	0.394759522	0.01995	0.612044445	0.00031
GSK3A	0.4539761	0.002139	0.052640453	0.72262	0.041870048	0.77778
PARP16	-0.50160129	0.002138	-0.364870071	0.02186	-0.410798396	0.01026
TCF20	0.245818644	0.002144	0.148235741	0.06137	0.133226852	0.0955
DRAM1	0.58151546	0.002147	0.178566827	0.34677	0.534087947	0.00483
FAM118B	0.384595745	0.002153	0.460557845	0.00021	0.306374829	0.01455
FAM83G	0.5188605	0.002154	0.279766913	0.09788	0.4870658	0.00393
RPP30	-0.298334708	0.002182	-0.231627622	0.01464	-0.375546932	9.68E-05
SERPINE1	1.625294558	0.002182	0.800393499	0.13177	0.521002057	0.3272

MYLIP	-0.446225304	0.002192	-0.510606337	0.00041	-0.327133952	0.02335
DNAJB1	0.29572645	0.002199	-0.122672934	0.20543	-0.140969756	0.14612
P11-480I12	-0.898055639	0.002204	-1.03090114	0.00041	-0.703280731	0.01558
JD7-PLA2G	-1.03749623	0.002225	-0.416786415	0.21363	-0.214035173	0.52312
DHX9	0.265677477	0.002232	0.116600214	0.1793	0.025803069	0.76674
ISG20L2	0.524796046	0.002233	0.256096953	0.13627	0.212039758	0.21703
B2M	0.42598581	0.002238	0.13769121	0.32314	0.141229614	0.31093
M47E-STBI	-0.754804612	0.002238	-0.679363161	0.00575	-0.527311517	0.03201
ABHD10	-0.370327719	0.00224	-0.295617637	0.01318	-0.149542006	0.21213
CBX8	-0.758763109	0.002254	-1.153220179	2.04E-06	-0.59251924	0.01348
ısa-mir-1199	-0.82793343	0.002255	-0.323493854	0.21076	0.261538195	0.30204
RASGRP3	0.73921039	0.002265	0.67966368	0.00491	0.920132203	0.00014
MEIS3P1	0.575803981	0.00228	0.010493459	0.95642	0.215020206	0.26123
RAP2B	0.316791959	0.002277	0.316883961	0.00219	0.25422916	0.01425
TEFM	-0.57249225	0.002278	-0.465971765	0.01128	-0.316041663	0.08561
UNC119B	-0.291984987	0.002279	-0.341885627	0.00032	-0.174316353	0.0663
SAP30BP	0.342461345	0.002284	0.415306327	0.00021	-0.004913335	0.96532
SMC4	-0.450087247	0.002299	0.251946936	0.08707	-0.212258183	0.15006
GDI1	0.326299661	0.002304	0.064309541	0.54793	0.003369062	0.97502
ADCY10P1	-1.24168038	0.002309	-0.569128755	0.15107	0.030023824	0.93862
ATMIN	-0.269725831	0.002311	0.052470113	0.54931	-0.200840247	0.02278
RDH14	-0.383515135	0.002309	-0.404866344	0.00106	-0.065730901	0.59356
PRUNE	-0.459629331	0.002326	-0.322613328	0.03096	-0.387353014	0.01005
ANKRD32	-0.644396796	0.00233	-0.172726929	0.40866	-0.299201655	0.1545
MAT2B	-0.262218342	0.002332	-0.161371855	0.05641	-0.290479249	0.00069
FAM160A1	0.710036743	0.002345	0.414642325	0.07672	0.656696746	0.00497
MLK4	-0.515383606	0.002347	-0.436377291	0.00949	-0.071875652	0.66733
C1orf64	1.202766095	0.002362	0.544099778	0.17965	1.43145813	0.00022
SALL4	0.703850119	0.002368	0.631799778	0.0063	0.701486586	0.00244
WRNIP1	-0.290960386	0.002371	-0.180859433	0.05429	-0.267873831	0.00476
LYN	0.614764397	0.002384	0.60195328	0.00281	0.531046709	0.00867
ZFPM1	-0.882372155	0.0024	-0.942571226	0.00113	0.214757933	0.45425
DRD1	1.083521509	0.002419	0.457922659	0.20494	0.069225674	0.85147
FBRS	0.493215206	0.002426	0.384316282	0.01838	0.456693847	0.00489
BIK	1.233514101	0.002438	0.851298673	0.03691	1.06578248	0.00889
SSH2	0.558784377	0.002437	0.198988189	0.28155	1.012261753	3.17E-08
CENPA	-0.766951209	0.002459	-0.175202444	0.4732	-0.679187483	0.00659
TOR4A	0.491163581	0.002468	-0.201461092	0.21858	0.274810704	0.09089
UFSP2	-0.438204084	0.002478	-0.192160044	0.18097	-0.087029489	0.54665
PELI3	-0.66169185	0.00248	-0.251669987	0.22836	-0.080509713	0.7032
DERL2	-0.324985212	0.002485	-0.353467165	0.00091	-0.447847085	2.94E-05
PTGER4	1.182608659	0.002506	0.97931324	0.01222	-0.448014317	0.25768
CDC40	-0.554241378	0.002517	-0.244904918	0.17953	-0.490795342	0.00734
RGS16	1.063261054	0.002517	1.101133398	0.00169	0.615975437	0.08147
PDP2	-0.613087565	0.002531	-0.367759501	0.06793	0.046454501	0.8161
TMEM223	-0.358719844	0.00254	-0.228321058	0.04863	-0.296872904	0.01135
ZBTB5	0.329808458	0.002542	0.215132417	0.04776	0.190833561	0.08084

SLBP	-0.358439855	0.002558	-0.151246663	0.19896	-0.385297227	0.00114
ZNF700	-0.501075462	0.002576	-0.225021773	0.16713	-0.328781547	0.04525
TMEM80	-0.563157656	0.002585	-0.291707132	0.10952	0.143163013	0.42984
OVOL2	-1.103580251	0.002596	-0.42062942	0.21343	-0.12029247	0.71417
SHPRH	-0.449457117	0.002596	-0.176272362	0.23531	0.012750444	0.9316
ZNF30	-0.855184335	0.002614	-0.666260626	0.01733	-0.467718453	0.09408
EBPL	-0.334464634	0.002655	-0.265178443	0.01515	-0.431887425	0.0001
SDHAF1	-0.532208685	0.002654	-0.5676258	0.00106	-0.278543073	0.10453
SMG8	-0.35870545	0.002657	-0.545803335	4.80E-06	-0.444122492	0.0002
ZFP14	-0.539009754	0.002656	0.204473224	0.24392	-0.270492607	0.12726
OXNAD1	-0.35900124	0.002665	-0.14297853	0.21821	-0.0867334	0.45717
ZNF717	-0.808483252	0.002679	-0.142169509	0.59024	-0.498326173	0.06052
P11-206L10	-1.270518461	0.002702	-0.175636202	0.66465	-0.444475501	0.27695
NAB1	0.335952266	0.002709	0.474574053	2.21E-05	0.059292003	0.59763
TPCN1	0.48798022	0.002725	-0.014648806	0.92847	0.345452106	0.03383
DNAL1	-0.561940048	0.00274	-0.279353357	0.13476	-0.096644132	0.60471
MMAB	-0.363752141	0.002742	-0.246822629	0.0405	-0.311524815	0.00988
PRKCZ	-0.504148681	0.002742	-0.674796341	5.73E-05	-0.145202861	0.38602
RN7SL467F	-0.898113217	0.002747	0.270066937	0.30093	-0.015642005	0.95371
MRPS6	-0.264064018	0.002753	-0.490831792	2.41E-08	-0.627799008	1.62E-12
IFT81	-0.432726871	0.002765	0.184492333	0.19331	-0.166700295	0.24502
TES	0.258211821	0.002764	-0.044822662	0.60321	0.00588296	0.94578
SACM1L	-0.310607871	0.002767	-0.323767044	0.00165	-0.124143303	0.22849
AKIRIN1	0.340179985	0.002776	-0.060325785	0.59617	-0.039036789	0.73173
BHLHE40	0.754957697	0.002777	1.076827467	1.94E-05	1.130780451	7.29E-06
PHACTR2	0.315596338	0.002777	0.593126279	1.75E-08	0.066131162	0.53096
SYNGR2	0.383299269	0.002779	0.03667771	0.7749	0.151027492	0.23895
MFN2	0.340638989	0.002793	0.131042066	0.24888	0.221273985	0.0525
KCNE3	-0.989012204	0.002812	-0.6968853	0.0348	0.284799321	0.38742
MPV17L2	-0.424341907	0.002829	-0.455498816	0.00097	-0.342429845	0.01357
NDUFC2	-0.308049785	0.002827	-0.140769969	0.16852	-0.541820666	1.55E-07
SLC25A32	0.382598312	0.002828	0.034052476	0.79065	0.151034159	0.23883
CINP	-0.279570451	0.002836	-0.192414975	0.03315	-0.134879026	0.14048
WWC1	0.552331378	0.002853	0.546276817	0.00315	0.26991884	0.1454
ANKRD49	-0.390061867	0.002858	-0.219326755	0.08897	-0.285976477	0.02774
SRPK1	0.437375648	0.002856	0.515431518	0.00044	0.452705372	0.00203
GEM	0.779213225	0.002882	0.148799373	0.56982	-0.130903565	0.61783
CHCHD4	-0.56497878	0.002895	-0.215190069	0.24362	-0.58865169	0.00183
MRPS34	-0.448989991	0.002894	-0.474355078	0.00154	-0.463046981	0.00207
RAPGEF1	0.437808186	0.002906	0.019249164	0.89614	0.539194386	0.00024
SZRD1	0.306732706	0.002931	-0.005108433	0.96047	0.008610167	0.9335
EPS8	0.343817035	0.002934	0.293125005	0.01112	0.078467086	0.49802
PSTPIP2	0.897238856	0.002975	0.608151772	0.04479	0.358555163	0.23781
TMEM30A	0.368366806	0.002976	-0.261880203	0.03488	0.054707671	0.65938
AGO2	0.555968989	0.003003	0.501747124	0.00739	0.424079296	0.02353
SPPL2A	0.334027921	0.003006	0.314035582	0.00515	0.172343894	0.12552
THUMPD2	-0.424719936	0.003034	-0.414405335	0.00337	0.197624864	0.15682

SLC16A3	0.996644464	0.003049	0.349707357	0.29821	1.187456217	0.0004
ZNF439	-0.873603868	0.003059	-0.401311462	0.15726	-0.441611754	0.12305
CHEK2	-0.400036708	0.00307	-0.27884548	0.0364	0.0336967	0.79943
SAMD14	0.705187628	0.003077	0.243764603	0.30497	0.086406902	0.72347
TCEAL8	-0.247532175	0.003078	0.018190403	0.82551	-0.449150295	8.32E-08
TMTC4	-0.376827971	0.003076	-0.005846735	0.96252	-0.154090166	0.22299
SNRNP35	-0.367301397	0.003083	-0.349364439	0.00409	-0.450047571	0.00026
SAMD8	0.330140203	0.003086	0.194839683	0.07956	0.042056281	0.70629
NUP93	0.44566212	0.003089	0.460549476	0.00214	0.175328187	0.24475
NUDT16	-0.314086057	0.003095	-0.120650148	0.25007	-0.249719116	0.01806
TOR1AIP2	0.185019267	0.003095	0.060674743	0.33162	0.138444172	0.02675
CCNF	-0.541539807	0.003132	-0.20524214	0.25897	-0.285731603	0.1171
FOXO3	0.341337393	0.003136	0.508698014	9.77E-06	0.260894322	0.02374
GRAMD1B	-0.886440892	0.003138	-0.958434231	0.00137	-0.395484044	0.18417
MAP4K2	0.571276714	0.003151	0.167314779	0.38673	0.110700457	0.56737
CELF1	0.318032693	0.003172	0.122598016	0.25552	0.316693168	0.00329
P11-384K6	0.675500786	0.003174	0.019990884	0.93145	1.191481032	1.14E-07
AACS	-0.346231745	0.003192	-0.176951626	0.12924	0.119622506	0.30402
BBS10	-0.412983288	0.003188	0.18404818	0.16758	-0.215856533	0.1154
SHISA9	0.599653661	0.00319	0.714460218	0.00039	0.928294278	3.97E-06
DNAJC21	-0.249307247	0.003203	0.028318932	0.73524	-0.132314245	0.11578
PROX2	-1.170684224	0.003211	-0.362268907	0.27742	0.037651452	0.90753
PET117	-0.594517401	0.003223	-0.724355489	0.00029	-0.578558305	0.00383
TRAF4	0.33896332	0.003233	0.059035775	0.6082	0.019743803	0.86408
FAM150B	1.148927013	0.00325	0.992250352	0.01041	0.667138297	0.08922
ACTR6	-0.415002024	0.003271	-0.167773276	0.22047	-0.292697754	0.03629
RAB11FIP4	-0.566819488	0.003287	-0.729274155	0.00015	-0.179959646	0.34678
KDM5C	0.239396722	0.003293	0.08175279	0.31551	0.190361725	0.0195
GNL1	0.470931923	0.003303	0.156739705	0.32806	-0.09498465	0.55424
ARMC7	-0.464523468	0.003307	-0.715367534	6.45E-06	-0.319497997	0.03892
ARL4A	-0.676211461	0.003311	-0.753741896	0.00101	-0.916630707	6.95E-05
SMAD2	0.198546309	0.003318	0.03984298	0.55489	-0.005123128	0.93949
GATAD2A	0.277788733	0.003333	0.32120625	0.00065	0.205094023	0.02984
PDHB	-0.215076905	0.003353	-0.10860622	0.13382	-0.414785053	1.62E-08
ZBTB6	-0.285833572	0.00335	-0.187732685	0.04889	-0.32108463	0.00092
AC040977.1	0.492566318	0.003357	-0.183112945	0.27924	0.103875953	0.53818
SLC6A15	1.351152831	0.003361	0.607213312	0.18837	-0.756431723	0.10906
FAM76A	-0.350855882	0.003368	-0.042560688	0.7129	0.066726758	0.56471
WWC3	0.476464367	0.003376	0.597517591	0.00021	0.397475142	0.01427
ZNF180	-0.375409812	0.003378	-0.450617009	0.00038	-0.235486488	0.06337
GSPT1	0.320247509	0.003391	0.309334792	0.00461	0.293082749	0.00731
SNURF	-0.4517386	0.003392	-0.078759142	0.59926	-0.300975317	0.04824
TMEM64	-0.348486476	0.003388	-0.419160829	0.0004	-0.206954174	0.08045
C19orf44	-0.701015891	0.003395	-0.199609578	0.3808	-0.107903548	0.63657
ELF2	-0.261461471	0.003404	-0.133112365	0.1309	-0.040995299	0.64314
NFXL1	-0.457705534	0.003403	-0.328039735	0.034	0.028736307	0.85239
SS18	0.287776277	0.003399	0.373639955	0.00013	0.184811788	0.06015

ZC3H7A	-0.455627428	0.003408	-0.176704288	0.25466	-0.209566564	0.17624
CCDC85B	1.067740167	0.003413	0.17693367	0.63547	0.424120996	0.25384
MEGF11	0.888673412	0.003427	1.033095234	0.00055	0.932405247	0.00169
DCTN3	0.332753135	0.003437	-0.064777344	0.57093	0.071259968	0.53293
CD3EAP	-0.632902379	0.003445	-0.902955394	3.05E-05	-0.788941587	0.00027
P11-396K3	-0.435964267	0.003447	-0.158491743	0.28604	-0.445925782	0.00276
CEP44	-0.401366774	0.003459	-0.146207768	0.28011	-0.10793864	0.42592
KIAA1143	-0.464973661	0.003465	0.069857775	0.65846	-0.380144122	0.01659
SEC24A	0.379251432	0.003466	0.288773374	0.02597	0.291457714	0.02466
STAT6	0.266605099	0.003465	0.115992317	0.20314	0.328864319	0.00031
TMEM92	0.658764542	0.003492	-0.637143739	0.00535	0.11900374	0.59978
MKS1	-0.504100801	0.003502	-0.339455361	0.04661	-0.408346195	0.01716
NIPSNAP3A	-0.298471024	0.003511	-0.125867957	0.1995	-0.156942559	0.11533
APC	-0.302921774	0.003542	-0.393725493	0.00014	-0.103552888	0.31729
NCCRP1	1.200694687	0.00354	0.616225521	0.13941	1.076938044	0.00889
RCOR3	-0.406279223	0.003541	-0.073973201	0.59076	0.063178173	0.64616
ING5	-0.412072683	0.003548	0.055267453	0.69156	-0.093937361	0.50094
MB21D1	0.852657876	0.0036	0.331545157	0.26256	0.902960253	0.00196
FBXW2	-0.269882786	0.003628	-0.184899524	0.04452	-0.19604514	0.03351
MRPL33	-0.277603278	0.003626	-0.37570665	6.61E-05	-0.362237241	0.00014
GPSM2	-0.503889736	0.003651	-0.080241322	0.64155	-0.13272131	0.44143
ZNF702P	-0.521063017	0.003665	-0.440202171	0.01335	-0.163159773	0.35869
IL33	-1.381558254	0.00367	-1.044348104	0.02612	-0.532844147	0.25132
L3HYPDH	-0.548363624	0.003694	-0.219468386	0.22047	-0.001577241	0.99302
MTG2	-0.32912038	0.003695	-0.264002712	0.01857	-0.249643279	0.02537
IRS4	1.761820394	0.0037	1.546925143	0.01076	0.830505487	0.17686
FAM200A	-0.37474615	0.003713	-0.114779208	0.35307	-0.299099516	0.01796
IRF2BPL	0.593648008	0.003709	0.657527712	0.00127	0.415130151	0.04252
SCRN1	0.234211843	0.003713	0.026294716	0.7444	-0.09498686	0.24054
CH25H	1.351649797	0.003718	1.047108045	0.02469	1.034001133	0.02709
TBCC	-0.351186628	0.003719	-0.419063178	0.00046	-0.474934755	8.67E-05
SIN3A	0.274700358	0.003754	0.258497633	0.00613	0.281938985	0.00289
ASB8	-0.282151376	0.003772	-0.129290614	0.17258	0.061429584	0.51655
PIGA	0.675348872	0.003793	1.021120356	1.17E-05	0.280552197	0.23012
MPHOSPH6	-0.37196316	0.003803	-0.143831366	0.2532	-0.364203139	0.00443
ZNF182	-0.430845844	0.00381	-0.072614915	0.61002	-0.180395659	0.21276
BCDIN3D	-0.547839331	0.003824	-0.222231656	0.22203	-0.152140684	0.40782
YAE1D1	-0.370526525	0.003826	-0.159259889	0.2032	-0.366652537	0.00397
ERN1	0.617732925	0.003834	0.575456014	0.0071	0.902285701	2.37E-05
CHD2	0.41505816	0.003844	0.260991586	0.06903	-0.024924277	0.86238
QTRT1	0.492509403	0.003897	-0.076958294	0.6544	0.188978917	0.269
CASP7	1.290673038	0.003921	0.626610566	0.16187	1.224854351	0.00618
MRPL35	-0.325891819	0.003937	-0.223931556	0.04516	-0.348431009	0.00196
CTDSPL	-0.28067539	0.003953	-0.213467846	0.02757	-0.070268346	0.46838
DCP1A	0.511119256	0.003952	0.013169851	0.94122	0.048573331	0.78694
PSMC2	-0.245090704	0.003964	-0.090176925	0.2844	-0.440286561	2.45E-07
C9orf152	-0.982848337	0.003975	-0.980793663	0.00378	0.361437682	0.27631

DNAJC24	-0.413295994	0.004003	0.019486474	0.8888	-0.002133115	0.98788
HDHD3	-0.372006092	0.004021	-0.628075783	1.15E-06	-0.124980218	0.32951
JUP	0.470444118	0.004015	0.430457357	0.00847	0.379113746	0.02042
KRT15	-0.804783078	0.004021	-0.816595255	0.00333	-0.951913335	0.00062
ORMDL1	-0.244326141	0.00402	0.110602585	0.18228	-0.078377415	0.3494
MAD2L1	-0.398709758	0.004046	-0.172313762	0.21022	-0.465693876	0.00077
TPBG	0.58380522	0.004082	0.557275854	0.00598	0.315924319	0.1207
KLF9	0.976545664	0.004091	0.91115571	0.00726	-0.172397396	0.61806
FANCM	-0.502005589	0.004099	-0.138260414	0.42031	-0.145449386	0.40101
ZFP62	-0.437907286	0.004102	-0.083518373	0.57999	-0.236397975	0.1185
DENR	-0.201141717	0.004108	-0.062596355	0.36757	-0.327169576	3.11E-06
FAM156B	-1.09146696	0.004139	-1.374066436	0.00031	-0.268968163	0.47281
UFL1	-0.27965746	0.004147	-0.139965951	0.14804	-0.275202392	0.00466
IGF2BP1	0.390192455	0.004178	0.315265359	0.02041	0.402064164	0.00312
AKIRIN2	0.418152045	0.004203	0.384865498	0.00752	0.534441731	0.00021
YWHAH	0.242341517	0.004203	0.284134687	0.00075	0.170824601	0.04346
CCDC34	-0.447864951	0.004214	-0.272225799	0.07685	-0.504589356	0.00118
SF3B4	0.281202646	0.004214	-0.114767025	0.24617	0.135784228	0.17213
FAM187A	-0.992323245	0.00422	-1.119278156	0.00127	-0.843230759	0.01406
ALKBH5	-0.273017	0.004231	-0.18590857	0.0502	-0.160500126	0.09141
IL6	1.935042331	0.004246	0.818285416	0.23619	0.376875889	0.59523
LIMD2	0.654685283	0.004243	0.574206212	0.01187	-0.038923853	0.86691
TMEM39A	0.437816131	0.004245	-0.015126248	0.92133	0.014771203	0.9233
SNAPC4	0.434426801	0.004262	-0.04466391	0.77015	0.40222161	0.00799
DBI	-0.338432236	0.004323	-0.075423804	0.52363	-0.391368669	0.00096
RASSF9	-0.709727893	0.004324	-0.325734888	0.18468	-0.262022815	0.28662
DMXL1	0.329998915	0.004349	0.278799469	0.01584	0.268634557	0.02018
ELK4	0.397189727	0.004341	0.25247718	0.06949	0.427878091	0.00207
GJA1	0.711543304	0.004344	0.59567721	0.01694	-0.337520132	0.17681
SAP30	-0.440625165	0.004343	0.200749846	0.15362	-0.279942093	0.06153
TMEM70	-0.367229963	0.00435	-0.399566894	0.00177	-0.407390316	0.00142
FGF23	0.816637857	0.004368	0.025580367	0.93023	0.720351746	0.01184
DHRS13	-0.423242546	0.004376	-0.348028579	0.01686	-0.228151011	0.1187
LTBP4	0.600436524	0.004384	0.525766673	0.01245	0.908981208	1.51E-05
F816-ZNF32	-0.600452753	0.004386	-0.564774387	0.00671	-0.054220911	0.79254
TFF3	0.796413756	0.004414	-0.512846799	0.06689	0.752481996	0.00715
ZCCHC4	-0.440224542	0.004414	-0.061798578	0.67782	-0.060278828	0.68901
C1orf172	-1.064498739	0.004427	-1.489799164	7.08E-05	-0.41727094	0.26018
NR0B2	-0.869581826	0.004436	-0.822784411	0.00689	-0.113638984	0.70762
KCTD10	0.320545431	0.004447	0.135276401	0.22914	-0.050269309	0.65644
FBXO9	-0.326491618	0.00446	-0.004845013	0.96593	0.086915222	0.44313
CBR4	-0.339700628	0.004468	-0.185313942	0.1145	-0.094958684	0.42125
SMARCAD1	-0.219006232	0.004466	0.033375903	0.66152	-0.214257822	0.00535
GPR39	-0.660724267	0.004471	-0.832132595	0.00034	-0.291461757	0.20572
LRP11	-0.302251637	0.004478	-0.384159218	0.00028	-0.333066952	0.00169
KIF20B	-0.419857755	0.004509	0.102669181	0.48385	-0.36941914	0.01228
C3orf38	-0.245986871	0.004544	-0.27491044	0.00127	-0.357687763	3.49E-05

CYCS	0.284337836	0.004582	0.276542653	0.00573	-0.085602099	0.39392
CASZ1	0.861284957	0.004599	0.722860593	0.01749	1.225297567	4.97E-05
FBXO18	-0.250000935	0.004626	-0.076447341	0.38369	-0.028787235	0.74376
LRR1	-0.453342299	0.004641	-0.521258627	0.00108	-0.335754708	0.03428
RP4-669L17.	0.865988536	0.004647	-0.291387194	0.35355	0.907705926	0.00291
AB019441.2	-0.844548969	0.004652	-0.111148073	0.68403	-1.205247897	8.32E-05
YWHAZ	0.222275531	0.004658	0.056675489	0.47047	0.099364461	0.20586
ZNF420	-0.620153912	0.004655	-0.568582528	0.00873	-0.439595036	0.04345
BMPR1APS	6.388509824	0.004669	7.304123572	0.00119	6.123196193	0.00672
FBXO5	-0.393807383	0.004664	-0.159128184	0.2478	-0.297479927	0.03166
USP47	-0.237178108	0.004666	0.049194428	0.55578	-0.31713302	0.00015
FBXO34	0.32662844	0.004698	-0.172314486	0.1374	-0.066204598	0.56828
TLR7	1.378022641	0.004725	0.973143394	0.04517	0.451828609	0.3714
ZNF181	-0.410664477	0.004733	-0.204492274	0.14778	-0.406073779	0.00457
KIAA0556	0.542665528	0.004739	0.619383055	0.00126	0.593767953	0.002
NR2F2	-0.790224147	0.004744	-0.367383508	0.18725	-0.49136669	0.07829
PEX2	-0.246713883	0.004758	-0.096431321	0.26388	-0.347561989	6.56E-05
MYO10	0.459936388	0.004777	0.264507217	0.10451	0.343407872	0.03512
GLIPR2	0.629541774	0.004805	0.777179974	0.00047	0.393480813	0.07836
NEFM	1.948356525	0.004825	1.345268171	0.05108	0.428326168	0.5421
TMEM2	0.340956891	0.004838	-0.132531695	0.27368	0.335767696	0.00554
USP1	-0.261350733	0.00486	-0.064415843	0.48473	-0.215430776	0.01996
RBM26	-0.268301281	0.004872	-0.064419852	0.49563	-0.151427829	0.1105
PDS5B	-0.363547849	0.004888	0.180422553	0.15971	-0.240077599	0.06228
ZNF14	-0.48990459	0.004901	-0.529046151	0.00209	-0.300522355	0.07946
PFDN4	-0.397196103	0.004911	-0.095061681	0.48625	-0.91278927	2.91E-10
CCDC104	-0.375221797	0.004923	-0.198459688	0.13341	-0.478535333	0.00035
GADD45B	1.018060446	0.004947	0.745199879	0.03941	-0.103059454	0.77995
ATG3	-0.378261028	0.004961	0.025824913	0.84605	-0.224645123	0.09361
ZNF782	-0.528593035	0.004981	-0.087020897	0.63222	-0.056429567	0.75657
CHD9	-0.319060242	0.004996	0.136117536	0.22945	-0.010550575	0.92591
PLA2G6	0.588432436	0.005	0.257754436	0.21855	0.428702796	0.04091
KLHL29	0.434020856	0.005053	0.375427452	0.01473	0.305821124	0.04821
POLR3B	-0.379686329	0.005058	0.282817886	0.03381	-0.349469836	0.00949
COX6C	-0.262807083	0.005073	-0.311150142	0.00083	-0.466237144	6.94E-07
UBTF	-0.337954597	0.005071	0.017655324	0.88296	-0.186353975	0.12137
RELA	0.669475271	0.005077	0.698991876	0.00342	0.548566135	0.02169
PRSS3	1.695188593	0.005093	0.917400944	0.13629	1.403880173	0.02108
AHDC1	0.70533095	0.005105	0.53742356	0.03293	1.194081531	1.92E-06
DNAJA1	0.375460735	0.005107	0.182227634	0.17413	-0.189587837	0.15802
LPAR2	0.410213515	0.005101	0.198157716	0.17629	0.371716308	0.01092
LZTFL1	-0.37456437	0.005111	-0.079545644	0.54894	-0.324008264	0.01515
ITPR1	0.627536209	0.005124	0.298096151	0.18302	0.404644108	0.071
NKX3-1	1.186571732	0.005136	2.196430392	1.44E-07	0.619028224	0.14914
PI4K2A	0.311320904	0.005142	-0.053896331	0.62905	-0.130110594	0.24741
CD248	0.582200443	0.005148	0.585484192	0.0048	0.245851847	0.2385
NXF1	0.422999682	0.005174	0.239892321	0.11308	0.265038619	0.07994

PRR15	-0.474693393	0.005179	-0.260110043	0.12105	0.043689715	0.79388
RFT1	-0.377418797	0.005192	-0.296493332	0.0262	-0.338285692	0.01129
NSMAF	-0.318270388	0.005203	-0.04811218	0.66864	0.046010517	0.68456
CUL4B	-0.286300279	0.005216	-0.122649392	0.22956	-0.195991787	0.05523
CYP2R1	-0.554759059	0.005226	-0.046580166	0.81046	-0.177806403	0.36059
SLC38A9	-0.362836896	0.00523	-0.049358385	0.6982	-0.378201344	0.00342
HIBCH	-0.530442775	0.005243	-0.296868939	0.11572	-0.07425458	0.69434
RFC4	-0.527607201	0.005255	-0.348227037	0.06356	-0.463522512	0.01404
CNOT6L	-0.283998664	0.005282	0.023912573	0.81162	0.214919742	0.03224
CUTC	-0.481731461	0.005278	-0.404602019	0.01664	-0.154415161	0.3592
OXA1L	0.295580049	0.005281	-0.188893702	0.07573	0.164107434	0.12157
SENP5	0.345325545	0.00527	0.174172465	0.15891	0.11352417	0.35875
SRP14	-0.432004708	0.005281	0.384630124	0.01268	-0.675536636	1.30E-05
CA8	0.928937961	0.005335	0.36233203	0.27762	1.289383968	0.0001
IPCEF1	1.413762155	0.005332	0.188758462	0.71268	0.870242326	0.0839
SRSF10	-0.232651152	0.005339	-0.00917996	0.91222	-0.275859512	0.00095
CYTIP	-1.431651507	0.005354	-0.185167339	0.71039	-0.179438008	0.71682
ELAVL1	0.240024663	0.005373	0.092075782	0.28485	0.325929594	0.00015
ZNF441	-0.508448251	0.005383	0.075583576	0.66579	-0.295736803	0.09653
PNKD	0.293643763	0.005389	0.002601409	0.98032	0.343155397	0.00111
CASP6	-0.360348288	0.005398	-0.28100702	0.02769	-0.634089294	9.28E-07
RUNX2	0.714437029	0.005433	0.448899243	0.07919	0.985502491	0.00013
SLC9A6	-0.304578027	0.005444	-0.07669649	0.4734	-0.196956103	0.06886
LSM6	-0.351197241	0.005455	-0.00342273	0.97782	-0.507257525	6.32E-05
STAT3	0.356578938	0.00547	0.280180071	0.029	0.252931888	0.04881
TCAIM	-0.317611843	0.005471	-0.247302723	0.02965	-0.22063563	0.05275
POLR3K	-0.548633512	0.00548	-0.472090435	0.01427	-0.20914309	0.27571
ZNF675	-0.35134744	0.005479	-0.080052254	0.51707	0.012543015	0.91939
AGPS	-0.346132931	0.005491	-0.204799313	0.0994	-0.061415221	0.62144
ZMYND19	-0.366690224	0.005499	-0.461579118	0.00043	-0.328821223	0.01221
C1QL1	1.178424504	0.005513	0.552452122	0.20123	0.587230528	0.17741
DHX16	-0.363623772	0.005536	-0.267044799	0.04053	-0.30294015	0.02063
FKBP1A	0.379985798	0.005523	-0.036575106	0.79033	0.173861907	0.20522
GIT2	-0.33164416	0.00552	0.04953908	0.67322	0.048610931	0.67952
HCCS	0.339486626	0.005536	0.121167986	0.31785	-0.015333199	0.90094
PCDH19	1.480056172	0.005529	1.335624344	0.01154	0.721618832	0.17349
PLK1	-0.755379944	0.005524	0.045536578	0.86623	-0.085793508	0.751
RAB34	0.349569879	0.005508	0.045589199	0.71747	0.017037436	0.89271
UHMK1	-0.18010135	0.005531	-0.274667486	2.11E-05	-0.174995961	0.00683
PEX19	0.27052002	0.005555	-0.246719623	0.01157	-0.134440153	0.16943
ZCCHC9	-0.342379823	0.005565	-0.184540456	0.13011	-0.238597149	0.0512
HOXB7	-0.820276024	0.005584	-0.560338569	0.05506	-1.073009788	0.00033
ZNF260	-0.324476698	0.005612	-0.035745825	0.75805	-0.422619578	0.0003
BORA	-0.681167755	0.005619	-0.674792623	0.0058	-0.485052497	0.04686
ZNRF3	-0.46622953	0.005622	-0.514365253	0.00203	-0.011263584	0.94564
AMMECR1L	0.191814011	0.005635	-0.026712412	0.69885	-0.045752821	0.51158
CDC42	0.208807533	0.005655	0.030507465	0.68591	-0.016051463	0.83165

ZYX	0.529488336	0.005651	0.326264316	0.08819	0.20207226	0.29147
CCNG2	0.503833084	0.005697	0.664149835	0.00026	0.394533674	0.03037
DTNB	-0.556769621	0.005701	-0.042194184	0.83217	0.129923778	0.5127
ADTRP	1.223618377	0.005719	0.890389491	0.04035	1.129315268	0.00924
BLOC1S5	-0.384216716	0.005716	-0.28188482	0.04066	-0.130648776	0.34268
P11-261C1(	0.915660998	0.005712	0.14187371	0.67549	1.323295203	4.75E-05
G0S2	1.014751849	0.00577	-0.18893091	0.61457	0.378648486	0.30768
C12orf44	0.37414745	0.005802	-0.046869209	0.73142	-0.13124254	0.3379
TJP1	0.363112029	0.005834	0.515787271	8.70E-05	0.322470735	0.01433
ID1	-0.601733632	0.005854	-0.355992239	0.10209	-0.545028827	0.01246
ATXN2L	0.415652341	0.005864	0.021079937	0.8891	0.663124813	1.01E-05
ZNF329	-0.335317681	0.005862	-0.475684366	8.43E-05	-0.339682521	0.00501
MDH2	0.361542388	0.005902	-0.088193076	0.50182	0.105018562	0.42407
PRDX3P1	1.209971026	0.005907	0.725477985	0.10316	1.526820553	0.00037
AP1G1	0.196203377	0.005923	-0.093281687	0.18977	0.070887327	0.31922
AMN1	-0.355347777	0.005937	-0.252620387	0.04651	-0.15213466	0.23252
RNF135	-0.537533989	0.005937	0.206708847	0.26732	0.078548562	0.67745
TMEM163	0.614325522	0.005929	0.088209894	0.69373	0.865010171	0.0001
CREB5	1.047966471	0.005946	0.950359391	0.01243	0.405284492	0.28766
RPP25	-0.650532268	0.005967	-0.348127053	0.13199	-0.104808366	0.64923
WDFY1	-0.190548795	0.005985	-0.159615512	0.02063	-0.19844523	0.00414
WSB2	0.296138307	0.006002	-0.170098988	0.11496	0.126818855	0.23947
LRRC45	-0.497672903	0.006018	-0.545998794	0.00263	-0.475824613	0.00777
CCDC169	0.767276939	0.006024	0.647561042	0.01901	0.749060534	0.00631
GOLPH3	0.348772224	0.006042	0.011657179	0.92691	0.131785777	0.2998
ICA1L	0.766630723	0.006038	0.91047289	0.00095	0.70136873	0.01172
RCN1	0.354504241	0.006075	0.036247023	0.77891	-0.106463154	0.41068
SF3B5	0.304347184	0.00607	-0.181533017	0.10389	0.034717032	0.75529
UPF3A	-0.349902695	0.006075	0.131203506	0.29696	-0.182593902	0.14968
ABHD13	-0.234297136	0.006097	-0.37004844	1.29E-05	-0.216480075	0.01072
GRB14	-0.745182107	0.006135	-0.622685426	0.02094	-0.346921601	0.19638
KPNA4	0.238002196	0.006168	0.155352072	0.07354	0.12311573	0.15644
ZNF131	-0.319754336	0.006167	-0.084352728	0.46658	-0.233565007	0.04497
CERKL	-0.597084546	0.006215	-0.132955653	0.52949	-0.269668092	0.21181
DOT1L	0.465853932	0.006229	0.183524331	0.2818	0.509885895	0.00269
NRAS	0.187655581	0.006251	0.102566901	0.13374	-0.102819268	0.13536
ABHD15	-0.512345941	0.006272	-0.422166998	0.02228	0.12762004	0.48421
CLDN18	0.761330035	0.006274	0.615745953	0.02707	0.804964117	0.00385
FA2H	0.640297308	0.006291	0.178130243	0.44748	0.494014362	0.03495
TNFRSF19	-0.632598477	0.006306	-0.796857663	0.00057	-1.270037876	5.42E-08
TRMT13	-0.409031397	0.006312	-0.028554574	0.84654	-0.182090504	0.22049
P11-691N7	1.691180635	0.00635	1.251929247	0.04413	1.997568468	0.00115
LRRC37A3	-0.741930917	0.006357	0.188828002	0.47891	0.059393056	0.82441
PSPC1	-0.405577638	0.006383	-0.040527763	0.78169	-0.150604932	0.30685
STEAP4	0.909614228	0.006411	0.693483311	0.03695	0.931924441	0.0051
NFE2	-1.312270306	0.00644	-1.965420581	5.84E-05	-0.521366815	0.26933
VWA7	0.760003078	0.006439	0.501767256	0.0743	1.131624137	4.50E-05

C16orf46	0.702934828	0.006452	0.226395784	0.39014	0.173894311	0.51491
CTU2	0.605856252	0.006484	0.001176896	0.9958	0.19434638	0.38523
EIF4G3	0.225374226	0.00649	0.073227221	0.37405	0.196846777	0.01722
.36A-HNRN	-1.882975584	0.006495	-0.340091062	0.59417	-1.856803591	0.00667
DNASE1L1	0.653453887	0.006508	-0.115725388	0.62951	0.017965516	0.94027
CSTB	0.615567212	0.006513	0.569988118	0.01177	0.427107726	0.05911
CRLS1	-0.292013851	0.006526	-0.121583275	0.25012	-0.254117589	0.01703
FER1L6	0.666447604	0.006557	0.771153745	0.00165	0.630576273	0.0101
NSMF	0.475116175	0.006571	0.079620678	0.65103	0.081595114	0.64254
ARHGAP31	0.953798153	0.006591	0.964255934	0.00595	0.55021441	0.11736
TMEM167A	-0.364604798	0.006602	-0.268310923	0.04528	-0.403398489	0.00266
DCP1B	-0.40955645	0.006616	-0.078767467	0.59599	-0.429561002	0.0042
RPL28	0.39934328	0.006616	-0.16290678	0.26824	-0.032976988	0.82266
JOSD1	0.378215899	0.006635	0.058035036	0.67675	0.067889858	0.62644
RNF170	-0.305178174	0.006672	-0.086746472	0.43663	-0.048078982	0.66738
IRX5	-0.938893629	0.006709	-0.621992156	0.0649	-0.023703581	0.94323
GRAMD3	0.505794554	0.006745	0.482152617	0.00933	0.246133664	0.1901
SPRTN	-0.392109957	0.006767	-0.520825244	0.00032	-0.402982068	0.0052
MST1R	0.651905517	0.006795	0.266607961	0.26721	0.814657771	0.00068
TSEN2	-0.664453881	0.006812	-0.146224307	0.54803	-0.043797359	0.85711
ZNF436	-0.461957278	0.006865	0.076420974	0.65251	-0.358592925	0.03544
IRF3	0.325048055	0.006898	0.129182991	0.28343	0.100384548	0.40637
KCMF1	0.36963595	0.006904	0.139565143	0.30669	0.021336088	0.8762
LRCH4	0.528494099	0.0069	-0.039327684	0.84143	0.138545413	0.48057
STAG3	-0.647371766	0.006914	0.244749	0.28816	0.246096903	0.28907
SLC15A4	0.492900006	0.006923	0.514751895	0.00436	0.48834977	0.0074
DOPEY1	-0.325287624	0.00694	-0.348887634	0.00369	0.006050576	0.95984
ZNF41	-0.329557396	0.006973	-0.052155589	0.66219	-0.177045329	0.14155
CXorf40A	-0.490179297	0.006993	-0.24738156	0.16486	-0.407508902	0.02367
DIDO1	-0.205555332	0.006998	0.072871512	0.33496	0.046762968	0.53619
RANBP3	0.339881142	0.006994	0.184291465	0.14271	0.110474606	0.3806
PTCD3	-0.204291415	0.007007	-0.107280008	0.15432	-0.119881105	0.11217
AC005042.2	1.127131805	0.007061	0.241455814	0.57551	1.206876867	0.00363
MANEA	-0.47996455	0.007079	-0.510966498	0.00398	-0.537991623	0.00248
MAP1S	0.622632832	0.007098	0.040800045	0.86015	0.159139571	0.49114
ZNF250	-0.466373643	0.007103	0.030540764	0.85819	-0.33143063	0.05389
RAP2A	-0.200258774	0.007117	-0.09869958	0.17851	-0.282361175	0.00015
AP1B1	0.279291738	0.00716	0.199326127	0.05445	0.350920989	0.00071
SPAG7	-0.19003017	0.007168	0.023941816	0.72937	-0.320354712	6.18E-06
IKZF3	0.590524488	0.007206	0.787513111	0.00027	1.00698146	2.96E-06
MICALL1	0.345429177	0.007205	0.161829538	0.20522	0.35717476	0.00528
PDLIM5	0.377833916	0.007218	0.244998628	0.08147	0.343623561	0.01451
TMEM65	-0.217460264	0.007231	-0.079266954	0.31524	-0.126751699	0.11232
NR4A2	0.958830541	0.007238	0.791121629	0.02678	0.920570785	0.00986
NDUFB3	-0.289720043	0.007245	-0.204139735	0.05462	-0.375614953	0.00048
GAREM	0.421594321	0.007251	0.337402966	0.03151	0.307651211	0.05016
BRMS1	-0.290809414	0.007279	-0.279935259	0.00853	-0.519985935	1.86E-06

C2orf69	-0.253062719	0.007275	-0.290942243	0.00174	-0.268105973	0.00418
PRR22	0.676090737	0.007305	0.248088955	0.33104	0.415286777	0.10176
?11-466H18	0.371591801	0.007305	-0.02264666	0.87019	0.080207379	0.56283
PRKD2	0.428955171	0.007314	0.277727898	0.08106	0.572648336	0.00031
ACSL4	0.525602075	0.007327	0.163465764	0.40444	0.220593813	0.2605
F3	1.300030341	0.00733	1.835300451	0.00015	0.63857084	0.18807
RPS19BP1	-0.338837234	0.007331	-0.579468069	4.08E-06	-0.317026819	0.0114
?1-130H16.	0.602303535	0.007359	0.431331378	0.05387	0.769681074	0.00053
?11-274B21	0.96286762	0.007368	0.312645538	0.39351	0.647670771	0.07415
ZNF286A	0.32263333	0.00737	0.457063726	0.00014	-0.132767903	0.27318
TTC30A	-0.334547121	0.007405	-0.217790892	0.07504	-0.240995232	0.05081
USP22	0.234203897	0.007402	0.178154337	0.04142	0.179600418	0.04
DHX15	0.185131176	0.007421	0.139728271	0.04302	-0.070647421	0.30759
DUT	-0.42147162	0.007498	-0.17240916	0.26903	-0.347145405	0.02689
NSD1	-0.23613176	0.007512	0.031168191	0.72246	0.037101928	0.67298
DGAT2	0.612289658	0.007521	0.10144786	0.65905	0.095646567	0.67779
ZIK1	-0.454414356	0.007538	-0.494835459	0.00344	-0.490233171	0.00387
SMAD1	0.575568074	0.007549	1.266974671	3.40E-09	0.595526315	0.00572
ANKRD65	-1.549980581	0.007554	-0.392569801	0.46105	-1.344533059	0.01706
ARL15	-0.550457832	0.007574	-0.3600984	0.07889	0.010061902	0.96059
APEX2	0.306472538	0.007598	-0.061663016	0.59205	-0.146863719	0.20705
ACTR2	0.195869519	0.007623	0.075285196	0.30416	0.103524665	0.15816
LSM11	-0.223935703	0.00769	-0.464757774	2.62E-08	-0.175785437	0.03286
C19orf53	0.205541241	0.007706	-0.35752787	4.96E-06	-0.158689032	0.04238
MIA2	-0.885805783	0.00771	0.070127029	0.82785	-0.207103408	0.52399
NOC4L	0.565773323	0.007729	0.512443104	0.01534	0.152154409	0.47533
IL17RE	-0.63728658	0.007787	-0.07318214	0.75888	0.377393963	0.11251
CAPRIN1	0.186056845	0.007798	0.159936967	0.02207	0.071595813	0.30593
CCNB1	-0.542368931	0.007822	-0.236789413	0.24429	-0.433778677	0.03325
FAM213A	-0.276293816	0.007821	-0.314819417	0.00227	-0.501493123	1.45E-06
HSBP1L1	-0.422225792	0.007823	-0.390325091	0.01277	-0.300606451	0.05539
AC004057.1	1.048926111	0.007838	1.29333431	0.00104	0.562711137	0.15396
CCNA2	-0.461677657	0.007865	0.06573278	0.70196	-0.39821955	0.02146
FAM46B	-0.781081989	0.007863	-0.720806388	0.01129	-0.094723348	0.73027
SRF	0.262831741	0.007871	-0.025429575	0.79714	-0.048447333	0.62606
GMPR2	-0.301793169	0.007891	-0.265544804	0.01903	-0.423521393	0.0002
ARMC1	-0.236990956	0.007912	-0.253059404	0.00414	-0.380404751	1.98E-05
AUH	-0.479800056	0.007935	-0.140109701	0.43132	-0.360133506	0.0454
ZNF470	-0.350522021	0.007943	-0.100732669	0.4352	0.169875976	0.18998
SMEK1	-0.191632466	0.007968	-0.135886253	0.05882	-0.203703899	0.0047
STOML1	0.589946436	0.008006	-0.082456957	0.71284	0.092552022	0.67856
SNRPB2	-0.253612858	0.008018	-0.150649222	0.11158	-0.510293141	1.05E-07
XDH	0.7384313	0.008067	0.184379676	0.50887	0.772961458	0.00537
AC024560.3	-0.377146549	0.008074	-0.186610038	0.17948	0.175032563	0.20449
PPWD1	-0.294188159	0.008082	-0.115649569	0.29151	-0.213536885	0.05392
DNMT3B	-0.483019041	0.008178	0.07790556	0.65246	-0.147743886	0.40505
CBX4	-0.712455461	0.008224	-0.877041762	0.00105	-0.45324036	0.09025

CPHL1P	0.912580225	0.008229	0.715348564	0.03832	0.763353177	0.02686
FNDC1	1.479176984	0.008224	1.265620543	0.02357	2.784419414	5.44E-07
TTK	-0.497779795	0.008233	0.27436314	0.14234	-0.15077681	0.42205
SLC25A6	0.327079216	0.008253	-0.066956881	0.5887	-0.020086745	0.87123
DCLK3	1.667263214	0.008262	2.209518502	0.00039	0.668959255	0.30016
THAP3	-0.489541139	0.008311	-0.072617703	0.6884	-0.32979692	0.07429
MALSU1	-0.291252428	0.00833	-0.507490816	2.29E-06	-0.313808727	0.00368
ZNF146	-0.21017967	0.008332	-0.102584474	0.19607	-0.34669081	1.37E-05
CLN8	0.421439124	0.008339	-0.25081242	0.11697	-0.123090907	0.44096
TD-2623N2	-0.881252106	0.008361	-0.305302976	0.32701	0.02654481	0.93135
TMEM179B	0.331657933	0.008358	-0.005149186	0.96714	-0.182032344	0.15086
UTP6	0.279144525	0.008365	-0.004568048	0.96545	0.120788295	0.25344
HRSP12	-0.348186173	0.008386	-0.444542039	0.00064	-0.242548885	0.06203
THUMPD3	-0.333917657	0.008397	-0.045319802	0.71834	-0.222340354	0.07785
ZNF655	0.179125859	0.008397	0.14355288	0.03324	0.160236902	0.01789
IPO8	-0.436792971	0.008439	-0.293656846	0.07499	0.000526369	0.99745
DACT2	-0.922161453	0.008453	-0.205403003	0.5467	-0.612176099	0.0766
AOC2	0.867898889	0.008472	-0.244411023	0.48002	0.493371953	0.13925
CYP3A7	1.189132771	0.00848	0.644072877	0.16471	0.212378694	0.65684
ANKRD50	0.38442838	0.008533	0.377480665	0.0097	0.16035346	0.27282
ZNF549	-0.380375765	0.00853	-0.35649088	0.01273	-0.198317229	0.16537
TOMM34	0.283538031	0.008548	0.368283585	0.00056	-0.007568984	0.94435
MCL1	0.394799511	0.008572	0.190112246	0.20552	0.024298956	0.87158
ZNF140	0.327046065	0.008588	0.248161723	0.0454	0.172896714	0.16565
MED29	0.94860575	0.008596	-0.314351878	0.39492	0.856595387	0.01713
RIMKLBP2	-1.094427313	0.008607	0.389971839	0.24395	0.150430791	0.6641
ZNF510	-0.347929822	0.008622	-0.03805335	0.76983	-0.12264779	0.34836
USF2	-0.325554222	0.008749	0.02906724	0.81277	-0.289306489	0.01942
TTC3	-0.200335154	0.008762	-0.162826792	0.03294	-0.103144532	0.17687
HMMR	-0.544415944	0.008775	-0.154506749	0.45225	-0.594862925	0.00412
SH2D3A	0.403717614	0.008777	0.22115547	0.15092	-0.004300439	0.97776
POP7	-0.309322224	0.008791	-0.530457499	6.19E-06	-0.415708713	0.00041
USP45	-0.463481727	0.008787	0.030106557	0.86253	-0.035161907	0.84022
NEDD1	-0.275880486	0.008806	0.189850141	0.06636	-0.206075215	0.04967
FOSB	1.432174216	0.008838	-0.383943803	0.4943	0.559282687	0.31434
ARHGAP12	-0.263227546	0.008875	-0.192769164	0.05446	-0.161488466	0.10763
BYSL	0.456300929	0.00886	-0.185679792	0.29139	0.166903635	0.34099
RUNDC1	-0.280983829	0.008865	-0.363029599	0.00066	-0.02082923	0.84366
SERF2	0.26628851	0.008864	-0.144570498	0.15555	-0.083846277	0.41037
ZNF214	-0.752286636	0.008873	0.29406001	0.28036	-0.980521209	0.00072
ZNF704	0.628784961	0.008868	1.145801672	1.75E-06	0.731238398	0.00231
MTIF2	-0.380613083	0.008916	-0.091751433	0.52393	-0.276138157	0.05649
PLTP	0.451965329	0.008933	-0.105747113	0.54175	0.296918982	0.08594
RCN2	-0.245562467	0.008946	0.145105306	0.11821	-0.311481485	0.00089
ALDH7A1	-0.396428014	0.008982	0.005874975	0.96885	-0.279366135	0.06488
ATP5SL	0.35501379	0.008993	0.031012883	0.81976	0.0326862	0.8107
IFT172	0.417975292	0.008993	-0.10149149	0.52383	0.429208492	0.00697

VGLL4	0.233519552	0.008984	0.052329595	0.55458	0.199873689	0.02534
FOXL1	0.746240007	0.009045	0.877133037	0.00204	0.41464874	0.14806
GKN2	-3.563529996	0.009041	2.307309516	0.08715	-2.458958092	0.0697
NDUFA4	0.260354633	0.009057	-0.047167022	0.63656	-0.001186911	0.99052
TAF10	0.360795815	0.00906	0.331138849	0.01607	-0.107826047	0.43998
TP53BP2	0.384051329	0.009063	0.653760871	8.52E-06	0.119885652	0.41594
ZMIZ2	0.379441006	0.009061	0.216518547	0.13593	0.767047529	1.05E-07
CKAP2	-0.395869339	0.009072	-0.038224867	0.80042	-0.299622661	0.04798
TWF1	-0.248521939	0.009076	-0.147858954	0.11992	-0.337154733	0.0004
MTERFD3	-0.391200522	0.009084	-0.064563115	0.65775	0.036801499	0.80169
SYDE1	0.645407937	0.009099	0.037434885	0.88002	0.167384931	0.50132
PDLIM3	0.781355277	0.00912	0.249224457	0.40758	-0.076672525	0.80075
DIAPH1	0.48072616	0.009167	0.054901748	0.76615	0.749218254	4.75E-05
TOR1B	0.361440859	0.009168	0.109608897	0.43051	-0.049934265	0.72019
ZNF24	-0.191546842	0.009203	-0.348912448	2.01E-06	-0.404907776	3.80E-08
HLA-A	0.401013842	0.009259	-0.187230382	0.22482	-0.244693893	0.11295
WBP5	-0.248049812	0.009266	-0.041494091	0.65907	-0.386146276	5.22E-05
ZNF641	0.422023154	0.009273	0.17454012	0.28092	-0.089809101	0.58199
FHL3	0.492533363	0.00933	0.372634134	0.04815	0.125384462	0.51117
RRP36	-0.238742494	0.009338	-0.19711335	0.02961	-0.139837834	0.12426
RHBDD2	0.326726961	0.009366	0.405665498	0.00122	0.202878307	0.10884
ADAMTS1	0.353803829	0.009386	-0.002081613	0.98783	-0.087361246	0.52283
HSPA1A	0.469554103	0.009415	0.626330723	0.00053	0.163384207	0.3665
PRKX	-0.40932822	0.009434	-0.057192464	0.71534	-0.204783254	0.19262
PSMB10	0.335007612	0.009492	-0.141502985	0.27648	-0.219763732	0.09766
IFI35	0.431801162	0.009505	0.293585276	0.0768	0.533707264	0.00126
MRPS35	-0.343340504	0.009502	-0.245827173	0.06251	-0.260144758	0.04912
CDCA8	-0.47836373	0.00953	-0.09127935	0.6175	-0.117776788	0.52035
MTUS1	-0.419727371	0.00952	-0.551954087	0.00065	0.018867204	0.90708
ZNF688	-0.623721535	0.009527	-0.261463167	0.26431	0.288843644	0.21372
MED25	0.389691047	0.009626	-0.053473095	0.72438	0.61735854	3.58E-05
METTL17	-0.241215547	0.009634	-0.191376928	0.03734	-0.189310183	0.03989
SOWAHA	-0.723340289	0.009648	-0.330923073	0.22253	0.227980886	0.39464
FBRSL1	0.658559018	0.009665	0.41719596	0.10113	0.724260348	0.00428
SLC43A2	0.707225931	0.009711	-0.013132552	0.96187	0.623477188	0.0225
ARMC10	-0.248397876	0.009752	-0.277950974	0.00327	-0.069502294	0.46384
DFNA5	-0.676280083	0.009732	-0.863779656	0.00095	-0.701484495	0.00711
GK5	-0.400844225	0.009755	-0.13538036	0.37712	0.199741963	0.19253
GPATCH2L	0.269432619	0.009766	0.061100166	0.5573	0.044628142	0.6684
RBM25	0.232557623	0.009776	0.272562946	0.0024	0.042730963	0.63511
RHBDF1	0.429028021	0.009774	0.111347725	0.50254	-0.030409971	0.85523
UNKL	0.875445843	0.009751	0.652663822	0.05198	0.551333858	0.10291
UTP20	0.259057367	0.00977	0.253057014	0.01108	0.155051787	0.12244
ZADH2	-0.399689862	0.009751	-0.096663437	0.52669	-0.290015795	0.05875
ZBTB44	-0.210814155	0.009736	0.002210039	0.97811	0.12277163	0.12806
PRPSAP1	-0.330737933	0.009835	-0.136910377	0.28252	0.068719857	0.58524
ATXN1L	0.209915197	0.009864	0.054430257	0.50236	0.069946695	0.39045

STRIP1	0.334233307	0.009875	0.307111752	0.01751	0.311405447	0.01631
ASUN	-0.372989824	0.009919	-0.102992447	0.47265	-0.490765331	0.00069
MOB4	-0.297618267	0.009939	-0.341659375	0.00289	-0.297675845	0.00983
CMC1	-0.489839901	0.009954	-0.662768307	0.00042	-0.334757858	0.07283
P11-644F5.	-0.631044915	0.009989	0.113204735	0.63125	0.061532717	0.79479
ZNF12	-0.27247704	0.010007	-0.016452947	0.8753	-0.046698781	0.65711
MTX3	-0.373915523	0.010041	-0.120304513	0.39553	-0.260270546	0.07052
SELK	0.472544211	0.010044	-0.201443017	0.27602	-0.273258956	0.14056
CAMK1G	0.865241452	0.010074	0.668364124	0.04387	-0.4505554	0.21048
EXOC1	-0.311303264	0.010104	-0.350075371	0.0036	-0.085386312	0.4792
LGMNP1	-0.737066936	0.010091	-0.040729804	0.87804	-0.103070574	0.70162
SCYL3	-0.235114979	0.010092	-0.054037457	0.53913	-0.098517434	0.26522
ZNF415	-0.443139937	0.010103	-0.022241432	0.89543	-0.235334661	0.16635
AUNIP	-0.517380529	0.010127	-0.093431953	0.63295	-0.224617462	0.25776
C1orf159	0.445603539	0.010154	0.118196672	0.49378	0.397030629	0.02116
HINT3	-0.23622471	0.010169	-0.112271418	0.21419	-0.126732631	0.16356
PTX3	1.637801543	0.01016	1.07564563	0.09156	0.040218357	0.94988
PUS7L	-0.33624083	0.010169	-0.28862689	0.02685	-0.276347125	0.03417
ZNF786	-0.427774935	0.010145	-0.20258981	0.20889	-0.114722671	0.47791
DYRK4	-0.337176779	0.010194	-0.203593635	0.10934	-0.36659882	0.00465
FAM167A	0.771071237	0.0102	0.014886725	0.96113	0.478988642	0.12191
TPRN	-0.462698151	0.010225	-0.861365676	1.99E-06	-0.286090826	0.10891
MFSD2A	0.76225284	0.010232	0.899723273	0.00225	0.112159815	0.71261
ICMT	-0.279700397	0.010253	-0.167927049	0.12163	-0.355401506	0.00109
NFATC3	-0.300234385	0.010241	-0.222456703	0.05594	-0.193271041	0.097
SEMA7A	1.288465183	0.010259	1.02511601	0.04116	0.516984512	0.30421
VN1R2	-0.956255643	0.01025	0.268244517	0.39688	0.392885474	0.21568
TBC1D2B	0.40807464	0.01027	0.017473001	0.91261	0.324210898	0.04164
CLINT1	0.311843487	0.010318	0.081293734	0.50358	0.329690517	0.00667
ACPP	0.619434874	0.010375	0.902190687	0.00017	0.698919738	0.00381
TUBB6	0.536822369	0.010373	0.395015368	0.05906	-0.064319137	0.75939
ALG1	-0.34257616	0.010382	-0.486514697	0.00026	-0.158964637	0.2303
ENGASE	-0.387613103	0.010392	-0.479048474	0.00158	0.035908276	0.81134
ZSCAN16	-0.392044559	0.010407	-0.18063075	0.21647	-0.342019106	0.02281
AARS2	-0.250270671	0.010455	-0.29022599	0.00267	-0.115796779	0.2274
MLF2	0.211962693	0.010455	-0.250958869	0.00256	-0.168641192	0.04324
NACC1	0.503980598	0.010461	0.101164445	0.60763	0.249622993	0.20483
EPRS	-0.311014103	0.010478	-0.0101426	0.93333	-0.345300102	0.00447
TAF13	0.362793757	0.010492	0.168848281	0.23297	-0.072196255	0.61352
SCAI	-0.54737379	0.010543	-0.131440265	0.53769	0.277992805	0.19159
GDPD3	-0.621164667	0.010586	-0.05848223	0.79584	-0.099951838	0.66355
PTP4A2	0.299034995	0.010587	0.194202525	0.09679	0.123193014	0.29238
FMNL3	0.685035861	0.010614	0.889367046	0.00088	0.238339319	0.37487
ZNF211	-0.447167757	0.01061	-0.351524531	0.04227	-0.344664205	0.04707
XRCC2	-0.431290601	0.010629	0.140200219	0.39942	0.077274173	0.64365
YEATS2	-0.260055303	0.01069	0.11484	0.25354	0.027590396	0.78485
EXO5	-0.428877656	0.010698	-0.507845521	0.00231	-0.405596382	0.01521

CRY1	0.440338462	0.010747	0.197309038	0.25291	0.20212088	0.24258
IFRD1	0.296620094	0.010796	0.118436405	0.3084	-0.013750478	0.90629
ANKRD9	-0.427423205	0.010828	-0.562965619	0.00076	-0.342339177	0.04058
LSM1	-0.272614454	0.010845	-0.247133095	0.01908	-0.363229743	0.00067
MAN2B1	0.335518535	0.010879	0.158651764	0.22854	0.308526974	0.01912
PNMA1	-0.373168852	0.010881	0.16176463	0.26009	-0.063962251	0.65866
ATG16L1	-0.272681814	0.010917	-0.279996625	0.00804	-0.21558761	0.0428
CXCL17	1.665904883	0.010919	1.4201633	0.03004	1.832497905	0.00492
DIS3L	-0.299124578	0.010932	0.085648699	0.45971	0.108659725	0.35062
CHPT1	-0.533216187	0.010948	-0.335983062	0.10675	-0.326020637	0.11847
PLGLB1	-0.455148576	0.010953	0.159645281	0.3237	0.01593224	0.92388
ANXA2	0.277122338	0.010974	0.154843883	0.15519	-0.038173034	0.72613
MRPL19	-0.290456665	0.010968	-0.029784856	0.79218	-0.333752047	0.0033
TEX30	-0.392674888	0.011118	-0.243799915	0.10003	-0.388282861	0.01064
SLC30A4	0.575309974	0.011137	0.620648267	0.0059	0.804840984	0.00036
GAL	1.604347377	0.011164	1.197262496	0.05939	0.602478824	0.35943
SPR	-0.422069561	0.011187	-0.511357913	0.00195	-0.038486274	0.81366
KIAA0753	-0.367939482	0.011203	0.167214954	0.24218	0.067085691	0.64001
VKORC1L1	-0.321501768	0.011214	0.2229419	0.07691	-0.01311758	0.91714
ZNF649	-0.300689358	0.011213	-0.291280938	0.01307	-0.222982643	0.05853
TBX15	0.563634992	0.011234	0.476937069	0.03097	0.442380595	0.04659
NSUN6	-0.416723903	0.011254	-0.164899439	0.30341	0.009823733	0.9511
CEP170B	0.430903486	0.011285	-0.071944578	0.67249	0.394282288	0.0203
ABCA10	1.451254552	0.011318	0.830981733	0.14781	0.700397915	0.22802
DNAJB14	-0.226649833	0.011324	0.013532669	0.87896	-0.220151607	0.01348
PHPT1	0.241537597	0.011314	-0.273938064	0.00434	-0.032933069	0.73076
AMPD3	0.724862749	0.011332	0.2956223	0.30203	0.536840825	0.06153
AP3D1	0.292548523	0.01135	0.127435023	0.26859	0.493450242	1.86E-05
ATP5EP2	-3.785649668	0.011346	0.186437431	0.89162	-2.145607371	0.12585
CALM1	-0.269304195	0.011402	-0.195884572	0.06535	-0.333515732	0.00173
CAMKK1	-0.501184411	0.011429	-0.604725878	0.00197	-0.089317819	0.63971
METTL14	-0.34318499	0.011422	0.08717403	0.51698	-0.46254807	0.00062
ZNF677	-0.349545235	0.011429	-0.201931168	0.1393	0.131710457	0.33418
VASP	0.271222188	0.01144	-0.155416472	0.14847	0.145515069	0.17528
PCYT2	-0.312409312	0.011459	-0.247035711	0.04397	-0.118561061	0.33391
CC2D2A	-0.383903345	0.011521	0.210860967	0.15874	-0.205902364	0.17305
CDH13	1.036557753	0.011525	0.727772417	0.07638	0.801994888	0.05141
CHML	-0.263465792	0.011508	-0.290777423	0.00503	-0.310859261	0.00281
GOLGA2P5	-0.565026427	0.011527	-0.56542309	0.01039	-0.14933143	0.49502
P11-22B23	-0.496393272	0.011524	-0.398664887	0.03841	-0.297118805	0.12374
RSAD1	-0.379915334	0.011511	-0.675008807	7.21E-06	-0.514404713	0.00061
FAM58A	-1.700315286	0.011573	0.138169574	0.83206	-0.188375304	0.77358
INHBA	1.008792844	0.011594	1.213167727	0.00237	0.069406003	0.86265
TOP2B	-0.256929263	0.011589	0.399115833	8.31E-05	-0.086176093	0.39669
CHTF8	-0.299487477	0.011638	-0.118329372	0.31908	-0.344303338	0.00371
SCO1	-0.274657903	0.011636	-0.186055468	0.08514	-0.227880052	0.0354
TBC1D31	-0.337330198	0.011636	-0.195219086	0.13291	-0.074181464	0.57252

ZNF75D	-0.344313187	0.011668	-0.105611162	0.42926	-0.039055133	0.77057
ZNF449	-0.359791381	0.011691	0.449583359	0.0009	-0.092132065	0.50952
AC068279.3	-1.318736025	0.011734	-1.490587826	0.00405	-0.195919545	0.68735
SRRT	0.499615497	0.011732	0.171966696	0.38617	0.373967664	0.05944
ZNF239	-0.447448859	0.011725	-0.350395674	0.0444	-0.291744107	0.0942
METTL9	-0.407125551	0.011756	-0.294243877	0.06792	-0.476722802	0.0032
EXOSC3	-0.349112501	0.011764	-0.184397775	0.17667	-0.192916815	0.1584
A2M	0.627981273	0.011779	0.309466734	0.21455	0.153128546	0.53917
REPIN1	0.366998758	0.011804	-0.140439205	0.33645	0.201559041	0.16665
F2RL2	0.560835415	0.011877	0.228324084	0.30599	0.585428896	0.00856
DSP	-0.326361399	0.011912	0.124866951	0.33548	0.034196951	0.79202
LARS2	-0.306929096	0.011919	-0.424992029	0.00048	-0.110290997	0.36513
THAP5	-0.345369923	0.011913	0.177927105	0.18928	-0.254219945	0.06259
LRRC27	-0.585389961	0.011949	-0.256778537	0.25536	-0.310994858	0.16853
1-Mar	-0.484710101	0.011952	0.023341026	0.90101	0.154093456	0.41127
LIPT2	-0.584097473	0.011984	-0.516270993	0.01998	-0.297136625	0.17817
YLPM1	-0.343668525	0.01198	0.097158343	0.47443	0.268950439	0.04782
DNAH14	-0.280999141	0.012016	-0.014926431	0.89175	-0.248979791	0.02481
SEC11C	-0.329171616	0.012017	-0.275516296	0.03399	-0.230122052	0.07651
MICU2	-0.206850146	0.012031	-0.064848419	0.4216	-0.236143292	0.0039
SCNM1	-0.282568512	0.012025	-0.291518758	0.00831	-0.302538615	0.00678
RNF222	0.892095146	0.012052	0.011713769	0.97518	1.183675452	0.00057
DDX39B	0.215266133	0.012079	0.251361202	0.00331	0.079827159	0.35166
H6PD	0.262912417	0.01208	0.155734578	0.13641	0.239424813	0.02208
PLK4	-0.387763965	0.012087	0.124608819	0.41636	-0.175316326	0.2554
TPRKB	-0.290180635	0.012085	-0.172332491	0.12666	-0.455614214	8.49E-05
CSTF3	-0.192059223	0.012126	0.020214058	0.78598	-0.116443448	0.12732
DEPDC1	-0.532503325	0.012119	0.140522197	0.50466	-0.251293233	0.23468
HSPB11	-0.274533119	0.012132	-0.069017514	0.51595	-0.165715763	0.12432
ZNF234	-0.389214869	0.012134	0.079884726	0.60069	-0.219248125	0.15419
COX6B1	0.260305306	0.01215	-0.26153332	0.012	0.104217786	0.31566
NCBP2	-0.30758963	0.012154	-0.214888673	0.07919	-0.437784282	0.00036
GRTP1	-0.527021444	0.012165	-0.30500038	0.13788	-0.166865191	0.41733
ADPGK	0.304735793	0.012208	0.051032654	0.67472	0.17822071	0.1427
TAF5	-0.342093492	0.012226	0.172098133	0.18644	-0.376583501	0.00548
NIF3L1	-0.286408905	0.012246	-0.166502812	0.13515	-0.073421672	0.51287
NDC80	-0.382843931	0.01227	0.118307973	0.4292	-0.120948949	0.42587
ARHGEF11	0.32292036	0.012306	-0.00374934	0.9768	0.36306381	0.0048
METTL18	-0.348215004	0.012312	-0.018304861	0.88924	-0.369595526	0.007
TRIM32	-0.252005029	0.012325	-0.49148814	9.09E-07	-0.195624857	0.04847
EID1	-0.334916708	0.012356	0.142811563	0.28489	-0.670122054	5.63E-07
ITGB6	0.709189584	0.012345	0.195633407	0.49018	0.26860799	0.34346
SCNN1G	-1.588503943	0.012362	-0.657578489	0.28343	0.611411471	0.31121
ZNF44	-0.335320589	0.012358	-0.249384006	0.05903	0.045645508	0.72751
HDDC3	-0.577822431	0.012373	-0.688918568	0.00254	-0.389384656	0.08618
MTF2	-0.279795767	0.012381	-0.084281176	0.44919	-0.361585576	0.00123
SMIM22	0.456770459	0.012388	-0.145371671	0.42977	0.269730745	0.14021

TLDC1	0.251077316	0.012403	-0.061018392	0.54478	-0.05289463	0.60031
HSPA4	-0.243908857	0.012452	0.018211273	0.85109	-0.331903464	0.00067
TP53INP1	-0.288087901	0.012457	0.048628696	0.66858	-0.22643954	0.04834
EFHD2	0.516103759	0.012536	0.190243601	0.35803	0.506077148	0.01433
GK	0.753382104	0.012531	0.15294264	0.61314	-0.331472546	0.27606
PIWIL4	-0.630266417	0.012545	0.11778914	0.6314	0.146536023	0.54647
ZNF808	-0.431698038	0.012545	-0.465612111	0.00678	-0.101352402	0.55349
RUSC1	0.268772497	0.012556	-0.194972396	0.07354	0.237771672	0.02689
TYSND1	-0.478493603	0.012562	-0.34851852	0.06387	-0.182322302	0.33116
DTWD1	-0.287748707	0.012605	-0.115506908	0.30624	-0.146516762	0.19747
UBR2	-0.171658971	0.012627	-0.200172402	0.00331	-0.087053848	0.20302
SLC22A23	0.51665456	0.012675	0.343906897	0.09664	0.75571278	0.00026
FAM73A	-0.288418893	0.012702	-0.169448285	0.1397	-0.132038742	0.25005
STOX1	-0.476611339	0.012699	-0.173944455	0.34285	-0.572154348	0.00269
KLHL8	-0.250376282	0.012712	-0.10360625	0.29203	-0.223742046	0.02455
MLXIPL	-0.917817916	0.012744	-1.551339619	4.27E-05	-0.214720568	0.55268
SSPN	0.856722381	0.01274	0.390642086	0.2543	0.213881139	0.53491
ZNF701	-0.306106036	0.012756	-0.263541984	0.02911	0.177337538	0.13801
TMEM170B	-0.354776617	0.012793	-0.402659279	0.00441	-0.024701311	0.86043
CLDN10	-2.453889662	0.012801	1.185180699	0.19364	-1.740386711	0.06991
NAT1	-0.442375978	0.012822	-0.464642993	0.00837	-0.545355299	0.00213
RFC5	-0.351712366	0.012835	-0.200492719	0.15221	-0.267994576	0.05659
RCSD1	-1.045119063	0.012862	-0.014735201	0.96955	0.224711576	0.56307
LEKR1	1.349908251	0.012878	0.535801838	0.31736	1.607568291	0.00299
KIAA0368	0.380594784	0.012938	0.484235185	0.00156	0.168522142	0.27113
MIER3	-0.291855262	0.012933	-0.366847035	0.00172	-0.311060007	0.00808
TCHP	-0.403820986	0.012939	0.173785594	0.27238	0.166115844	0.29858
TB-50L17.1	1.20361125	0.012947	1.06738819	0.02633	0.979531064	0.04421
ANKRD30B	0.598506728	0.012985	-0.032952648	0.893	0.50485251	0.03556
DDX27	0.799752283	0.013028	0.031267153	0.92227	0.395139693	0.21661
DGKE	-0.500944656	0.013024	0.016894281	0.93138	-0.082231869	0.6757
FAM98A	0.285547246	0.013008	-0.002240002	0.98447	0.026808349	0.8166
LSM12	0.263694706	0.01299	0.023242521	0.8267	0.184056725	0.08334
MSH6	-0.315851038	0.01304	-0.102528665	0.419	-0.390379746	0.00213
NCK1	0.385554264	0.013036	0.462413101	0.00284	0.00295636	0.98488
PHF5A	-0.266568539	0.013031	-0.297598	0.00508	-0.330238436	0.00206
TRIB2	0.638508312	0.013015	0.424292477	0.09887	0.313139266	0.22345
RWDD4	-0.304612142	0.013058	0.08680985	0.47076	-0.227190448	0.06254
FAM46A	-0.452709305	0.013087	-0.855306099	2.77E-06	-0.351569688	0.0539
TOMM7	-0.280434635	0.013087	-0.507695004	6.76E-06	-0.443466798	8.77E-05
C9orf114	-0.378372297	0.013111	-0.257419635	0.08752	-0.066726772	0.65947
HIST4H4	-0.413355253	0.013153	-0.424553025	0.01056	-0.388971378	0.01936
IFNAR1	0.224988298	0.013173	0.04655948	0.60746	0.15086117	0.09625
CHD4	0.258874575	0.013209	0.437189332	2.79E-05	0.219761435	0.03541
?11-1023L1	-0.795447815	0.013225	0.07919709	0.79518	0.462643926	0.1269
TIPARP	0.54258038	0.013233	0.097053569	0.65787	0.365194922	0.09553
CMAS	-0.27692471	0.013315	-0.151270928	0.17155	-0.343986757	0.00212

CSNK2B	0.271379609	0.013287	0.129979905	0.23484	-0.045844379	0.67661
RP11-274B21	0.47661307	0.013304	0.428047514	0.02511	0.724070096	0.00014
TMEM206	-0.696483114	0.013298	-0.673531583	0.0147	-0.809331437	0.00413
VSIG1	0.658820967	0.01331	0.806553918	0.00244	0.683474081	0.01023
PPP1R7	-0.255264777	0.013369	-0.183828128	0.0713	-0.326352944	0.00152
ZNF488	-0.784671345	0.013394	-1.275783289	6.73E-05	-0.851488031	0.00716
CDK1	-0.389461267	0.013482	-0.119263187	0.44744	-0.391268079	0.01299
ABHD16A	0.26133628	0.013491	0.153191445	0.14651	-0.04209724	0.69323
TMEM248	0.236745455	0.013505	0.089974549	0.34679	0.063629089	0.50626
ADGB	1.230595255	0.013563	0.585619426	0.24232	1.206660917	0.01194
PYURF	-0.254983754	0.013654	-0.030440239	0.76319	-0.261719231	0.01086
OSBPL1A	-0.252262823	0.013668	-0.158859331	0.11562	-0.079366091	0.43335
LEPREL2	0.80672223	0.013679	0.037551468	0.90908	0.012812749	0.96919
NRDE2	-0.381816648	0.013705	-0.299345662	0.05185	-0.219381977	0.1543
ACTR3B	-0.53107191	0.013752	0.063464335	0.76463	0.063684548	0.76446
EMD	0.333236809	0.013733	0.290122362	0.02952	0.134677613	0.31985
ISY1	-0.424354648	0.013722	-0.033296426	0.84463	-0.422572101	0.01363
LYPD2	1.1271554	0.013743	0.048809003	0.91561	0.510588981	0.26616
MOBP	0.957918416	0.013755	0.975706175	0.00877	1.147962316	0.00178
PDLIM7	0.560569556	0.013758	0.241374798	0.28893	0.265644328	0.24365
TMEM134	0.520248236	0.013763	-0.222690395	0.29614	0.132728587	0.53014
SC5D	0.278753617	0.013797	0.205214614	0.06936	0.033093321	0.77004
FST	0.723177577	0.013807	0.103587537	0.72465	0.408045099	0.16483
MKNK1	0.320508735	0.013842	0.105470374	0.41398	0.351110662	0.00652
ADAM2	-1.006210504	0.013849	-0.889957204	0.02303	0.147459824	0.68103
AP3M2	-0.371497755	0.013858	-0.295127547	0.04834	-0.137320032	0.36088
GPR116	0.888294698	0.013873	0.734232517	0.04107	0.473587074	0.1887
GPR135	-1.028365174	0.013985	-0.259234993	0.50534	-0.380037841	0.33639
R3HDM4	0.324383589	0.013989	-0.0721773	0.58539	-0.002396809	0.98557
ALCAM	0.469404429	0.01408	0.680021598	0.00037	0.151184371	0.42933
U2AF2	0.217527475	0.014093	-0.035458328	0.68839	0.024698013	0.78069
NCK2	0.31548618	0.014115	0.538624815	2.44E-05	0.070387611	0.5838
MSL3P1	-0.523361608	0.014125	-0.213839439	0.29279	-0.422735781	0.04269
NCAPD3	-0.411189165	0.014144	-0.25847258	0.12096	-0.169784802	0.30874
ACSF3	-0.345387525	0.014181	-0.515364569	0.00023	-0.220408924	0.11144
STAG3L5P	-0.416741913	0.014183	-0.123726624	0.45366	0.107019337	0.51612
ZNF595	-0.374567229	0.014176	-0.059377114	0.69357	0.063486567	0.67353
OTUD5	0.308828656	0.014193	0.130301521	0.30018	0.304394062	0.01585
RAB11FIP2	-0.480901695	0.014209	-0.45724939	0.01928	-0.261662928	0.18044
ALKBH8	-0.332660183	0.014238	-0.060489326	0.64583	-0.280409705	0.03622
AC010970.2	-1.245404445	0.014308	-0.704756326	0.16494	-0.110445097	0.82762
IST1	0.186338215	0.014325	0.062545323	0.41172	-0.041469563	0.58695
PTHLH	1.731925406	0.014318	1.601748939	0.02323	0.956619914	0.17864
DEPDC7	-1.038381102	0.014357	-1.451146983	0.00064	-1.961987919	7.12E-06
C12orf45	-1.001049942	0.014367	-0.813730182	0.04358	-0.752156047	0.06126
GTPBP4	0.362241977	0.014389	-0.030229768	0.83822	0.009716414	0.94771
YRDC	0.409019616	0.014413	-0.525681337	0.00208	-0.159907185	0.34527

THYN1	-0.287777708	0.014453	-0.296996247	0.01036	-0.569051353	1.67E-06
COL5A3	1.322265027	0.014465	-0.158013974	0.77369	-0.835508377	0.13768
MRPS7	-0.227880835	0.014473	-0.276132651	0.0028	-0.502828543	8.89E-08
PPM1D	-0.227512347	0.014485	-0.141735421	0.12177	-0.411421856	9.69E-06
TXNIP	-0.500347792	0.01455	-0.386751165	0.05891	-0.326436363	0.11085
LRRC8D	-0.347644238	0.014575	-0.016167113	0.90816	-0.447680435	0.00161
PDE4A	0.811784229	0.014587	0.151310807	0.65116	1.141499207	0.00039
CLPX	-0.276567357	0.014612	-0.031570422	0.77896	-0.126607626	0.26138
ELMOD2	-0.284037902	0.014654	-0.090416924	0.43395	-0.505900576	1.36E-05
IER2	0.518636741	0.014712	0.059703585	0.7792	0.461468617	0.02985
ZNF106	-0.279918618	0.01476	0.109121135	0.34	-0.128335708	0.26258
FBXW11	0.236441436	0.014772	-0.030328482	0.75414	0.122886312	0.20536
SRPR	0.262492629	0.014778	-0.196228388	0.06945	0.12689539	0.23849
C1orf27	-0.233956757	0.014798	-0.159328104	0.09116	-0.197762572	0.03878
MFF	-0.224139171	0.014799	-0.014516458	0.87327	-0.151883894	0.09752
SLC16A13	0.487663995	0.014827	0.077878759	0.70211	0.678908793	0.00052
USF1	-0.249575988	0.014825	-0.234271036	0.02062	-0.366729546	0.00034
RNF11	0.259146546	0.014862	-0.009793561	0.9266	-0.180014027	0.0915
TUBGCP3	-0.395728917	0.01486	-0.448012356	0.00554	-0.062406625	0.69881
CPSF2	-0.250683459	0.014979	-0.093496905	0.36099	-0.253762003	0.01352
PLA2G10	-2.993158146	0.015003	-0.802837851	0.48501	-0.407180546	0.72201
RTN4	0.203851959	0.01504	0.204185315	0.01462	0.156220646	0.06238
GLE1	-0.268598498	0.015066	-0.130475146	0.23063	-0.131253327	0.22997
MT-ATP6	0.643446938	0.015051	-0.130996951	0.62076	0.556549667	0.03548
MZT1	-0.32374717	0.015067	-0.3660022	0.00539	-0.340078058	0.01019
CDKN2AIP	-0.485653371	0.015089	-0.326010187	0.10204	-0.452461509	0.02337
PEX6	-0.306398139	0.015128	-0.382357853	0.00221	-0.029505033	0.81164
TIMM21	-0.322089359	0.015277	-0.335848909	0.01007	-0.476483707	0.00029
CDC6	-0.483193811	0.015294	-0.221199084	0.26474	-0.480723559	0.01557
KIAA0355	0.348682214	0.015293	0.333964936	0.01991	0.086748036	0.54664
CYR61	0.742392119	0.015315	-0.166659336	0.58985	-0.386854605	0.21502
GPATCH2	-0.24416394	0.015348	-0.020511492	0.83692	-0.0781768	0.43681
TMEM41A	-0.246693768	0.01535	-0.501310452	7.93E-07	-0.065772546	0.51437
OSBPL11	-0.179760167	0.015367	-0.177245713	0.01476	-0.22709507	0.00206
URI1	-0.343547648	0.015407	0.001438877	0.99188	-0.384596235	0.00663
COQ7	0.346273028	0.015442	0.358917708	0.01115	0.171901761	0.22923
ATL3	-0.187672301	0.015487	-0.165101914	0.03179	-0.037004835	0.63051
ZBTB22	0.249809576	0.015501	-0.029040618	0.77833	-0.149670238	0.15409
TUBA3FP	0.662667166	0.015533	0.836940312	0.00152	0.598946654	0.02582
C19orf55	0.50694662	0.015562	0.310993684	0.13453	0.56667004	0.00586
GCNT1	-0.644608439	0.015593	-0.358101565	0.17899	0.135098578	0.61195
GMEB2	0.315684673	0.0156	0.128798642	0.32214	0.198621725	0.12807
GPRC5C	0.403932986	0.015599	-0.201204116	0.22946	0.222105039	0.18373
KLHDC2	-0.342274396	0.015584	-0.127880189	0.36457	-0.093398699	0.50748
PCNT	-0.362176012	0.015588	0.041340016	0.78167	0.22061922	0.13897
APH1A	0.243915695	0.015672	-0.080033528	0.42791	0.028835475	0.77546
C4orf27	-0.319379139	0.015639	0.056942185	0.65449	-0.391945681	0.00293

MFSD9	-0.411303502	0.015669	-0.419835049	0.01342	-0.431400141	0.01097
NFYB	-0.300554242	0.015675	0.042201418	0.73104	-0.5068421	4.47E-05
PREPL	-0.283530052	0.015654	-0.273492467	0.01932	-0.177907313	0.1284
TRPC1	0.679225349	0.015672	0.54429364	0.05083	0.150975598	0.58944
TRUB1	-0.293316677	0.015632	-0.161132874	0.18064	-0.299412455	0.01353
FBXO48	0.521030376	0.015716	0.661887422	0.00158	0.736680031	0.00047
SSBP1	-0.200877744	0.015711	0.004702806	0.95419	-0.281475848	0.00068
NSL1	-0.374556245	0.015751	0.166108572	0.27804	-0.495974971	0.00134
KCNJ13	-0.847873823	0.015958	0.219242203	0.50783	0.2927538	0.37631
MAP4K3	-0.233297331	0.015964	-0.061454684	0.52332	-0.034205269	0.72365
UBB	0.183695456	0.015972	0.057201116	0.45274	-0.133798077	0.07956
GUSBP1	-0.42704092	0.016018	-0.343903745	0.04773	-0.389730263	0.02652
C8orf44	-0.841571559	0.016032	0.488948878	0.13041	0.021487989	0.94811
HNRNPU	0.179885974	0.016036	0.104359048	0.16198	0.024771253	0.74022
VANGL2	0.390429848	0.016045	-0.018078835	0.91152	0.580242402	0.00032
PPM1N	-0.769700964	0.016056	-0.332431781	0.27167	-0.541130899	0.08163
DHX32	-0.26087644	0.016084	-0.216015892	0.04414	-0.118374618	0.27079
DLGAP5	-0.468956528	0.016102	0.086212507	0.65658	-0.38960798	0.04529
ZNF302	-0.345129765	0.01617	0.019249674	0.89224	-0.262004251	0.06686
ZNF354A	-0.371438746	0.01617	-0.017922035	0.90554	-0.308740002	0.04453
MPEG1	-0.862646223	0.016178	-0.811840965	0.01825	0.080060975	0.80302
BTN2A2	0.50549212	0.016204	0.54580523	0.00881	0.012879543	0.95179
PHLPP1	-0.481467469	0.01626	-0.277503142	0.16529	0.158501289	0.42684
SHC2	0.443371955	0.016306	0.071748454	0.69827	0.240117336	0.19323
DDX19B	0.280748481	0.016327	0.414871228	0.00034	0.013006017	0.91152
HIST1H4C	-0.426962972	0.016337	-0.12711519	0.47387	-0.655409854	0.00023
LLGL1	0.407230677	0.016426	0.061504856	0.71716	0.089426413	0.59896
CLUAP1	-0.389307031	0.016485	-0.3625363	0.02284	-0.428587984	0.00749
RHPN2	0.455429538	0.016497	0.102300449	0.59043	0.262080689	0.16791
C1orf86	0.58125243	0.016509	-0.035087584	0.88518	0.427586753	0.07712
ARPC4	0.265186616	0.016523	-0.100046802	0.36677	-0.005646919	0.9594
MBNL1	0.236660372	0.016553	-0.020471169	0.8356	0.139370997	0.15786
FIZ1	0.431901666	0.016572	0.195614812	0.27587	0.251191129	0.1612
CAMLG	-0.324507348	0.016614	-0.24329958	0.07003	-0.253600453	0.05992
AKR1C2	-0.801102798	0.016644	0.396208524	0.22982	-0.754008729	0.02391
NUDT7	-0.860052535	0.016662	-0.093856881	0.77521	0.333921505	0.30124
SEC61G	-0.278204134	0.016743	-0.087760162	0.4457	-0.509276794	1.26E-05
URGCP	-0.326730197	0.016766	-0.443256261	0.00117	-0.581293711	2.09E-05
ZNF438	0.397630321	0.016784	0.177810074	0.28167	0.275615225	0.09793
ZFP64	-0.342685059	0.016854	-0.111859317	0.42813	-0.101859052	0.47165
9-Mar	-0.379749611	0.016865	-0.532874796	0.00079	-0.103990188	0.50317
MSN	0.435111998	0.016906	0.417559986	0.02178	-0.117328627	0.52031
PTPRJ	0.521622316	0.017014	0.321041619	0.14052	0.448556137	0.04012
POU2F2	1.482538918	0.017024	1.135178343	0.07202	-0.233136741	0.71469
RPL13P12	0.426447471	0.017064	-0.183516173	0.30578	-0.046865165	0.7937
MOSPD1	-0.31746804	0.017155	-0.083415128	0.52382	-0.434050005	0.00118
PLEC	0.508261394	0.017153	0.811629187	0.00014	0.979971052	4.26E-06

PRPF18	-0.328195945	0.017137	-0.014010857	0.91716	-0.432719121	0.00161
RPL9	-0.377257118	0.017153	-0.385634415	0.01482	-0.463644977	0.0034
SLC16A10	-0.897617301	0.017127	-0.036533406	0.92159	-0.419520692	0.26042
PTEN	-0.299534801	0.017187	0.067558835	0.59048	-0.154223248	0.21947
ZNF395	-0.384249727	0.017179	-0.385654997	0.01677	-0.24595891	0.12686
CCDC18	-0.45294852	0.01722	-0.356191808	0.05692	-0.26570373	0.15934
SF3A2	0.2784167	0.017223	-0.073543675	0.53298	0.347258697	0.00287
SLC25A40	-0.280940267	0.017316	-0.056145265	0.62866	-0.014583788	0.90043
TIAL1	-0.339375783	0.017352	-0.339521422	0.01698	0.052574107	0.71076
SLC25A37	0.304896242	0.017419	0.429072434	0.00075	0.298319455	0.01971
CACFD1	0.402487813	0.017433	0.10577887	0.53347	0.224825482	0.1859
ASB2	1.025726658	0.017467	0.399033138	0.35739	0.371929906	0.39878
CRYGS	-1.044363807	0.017477	0.054901964	0.88767	0.195460962	0.61345
KRCC1	-0.325020476	0.017477	0.182742833	0.17154	-0.315541229	0.02042
PUS7	-0.426489378	0.017478	-0.125189912	0.4831	-0.406321475	0.0234
HIST1H2AI	-0.499139072	0.017594	-0.166872549	0.4264	-0.687628882	0.00108
SLC9A2	-0.810628675	0.01763	-0.071853879	0.83272	0.079164137	0.81601
RABGAP1L	-0.310286249	0.017775	-0.211473995	0.10511	-0.060065761	0.64477
STX4	0.300060968	0.017821	0.143678137	0.25488	0.286029331	0.02379
GPR3	0.837461718	0.017846	0.031424357	0.93207	0.311170277	0.39345
ZNRD1	0.453413283	0.017856	-0.173260987	0.36841	-0.099246574	0.60837
NCOA3	0.21437339	0.017866	0.166656138	0.06506	0.308994615	0.00062
XBP1	0.585705175	0.017879	0.128901272	0.60235	0.342623759	0.16603
SET	-0.215579259	0.017899	0.114991762	0.20593	-0.412896452	5.80E-06
IFITM3	0.329246563	0.017972	-0.06448701	0.64323	-0.155275938	0.26536
HIPK2	0.413808665	0.017986	0.660893105	0.00015	0.848614872	1.17E-06
ACOT9	0.279694515	0.018081	0.259317766	0.02699	0.162992428	0.16711
CYTH4	0.942673125	0.018077	0.869719631	0.02871	1.278173874	0.00138
ARHGEF17	0.490809644	0.018113	0.230856723	0.26594	0.040149453	0.84688
SPATA6	-0.428725866	0.018136	-0.337823023	0.05995	-0.097821866	0.58548
TRNT1	-0.252230804	0.018124	-0.028380265	0.78744	0.07127676	0.49744
VPS13D	-1.016789409	0.018142	-0.159081207	0.71051	-0.030522821	0.9434
GPX2	0.491452125	0.018202	0.433699718	0.03712	0.577420097	0.00551
AC004980.9	1.2055639	0.018234	1.020231344	0.04504	1.403727666	0.00548
FNBP1L	-0.269751312	0.018236	-0.042446721	0.70978	-0.249757244	0.02869
MFAP3L	-1.116068432	0.018229	-0.451682195	0.3342	-1.796546315	0.00018
TBL1X	0.288798598	0.018343	0.073722585	0.54648	0.509318926	2.80E-05
TNFRSF12A	0.50850634	0.018333	0.097092398	0.65266	-0.401019682	0.06452
UBXN11	0.331000903	0.018341	-0.28364451	0.04616	-0.109891917	0.43821
SLC28A3	0.980138619	0.018378	0.58598228	0.15954	0.730699104	0.07888
EEFSEC	0.408090644	0.018389	0.052166879	0.76429	0.227833087	0.18951
DAPK3	0.322007175	0.018415	0.092517342	0.49952	-0.009105375	0.94725
NLGN2	0.545788626	0.018415	-0.007923871	0.9728	0.646459203	0.00506
GABPA	-0.183680533	0.018425	-0.177430781	0.02151	-0.277922599	0.00036
EAF1	0.289392597	0.018442	-0.074513272	0.5453	0.050185047	0.68354
DEDD	0.250465161	0.01853	-0.037588867	0.72392	0.024141245	0.82049
UGDH	-0.239584542	0.018595	-0.077874506	0.44337	-0.203431507	0.04536

ZBTB7B	-0.38684107	0.018612	-0.561624386	0.00062	-0.18892994	0.2478
APH1B	0.503325155	0.018645	-0.143557699	0.50274	0.297872808	0.16404
PLCB2	0.984769201	0.018647	0.786373186	0.06142	0.512368664	0.22024
RNPS1	0.233470464	0.018636	0.317275034	0.00136	0.147455628	0.13711
MRPL43	-0.19560419	0.018689	-0.169923948	0.03656	-0.273252586	0.00093
PSMB4	0.19620046	0.018721	-0.058641439	0.48247	-0.119871519	0.15291
ATAD5	-0.365953291	0.018763	-0.034409553	0.82376	0.038444701	0.80349
DDX20	-0.289627966	0.018784	-0.376587857	0.00217	-0.364687458	0.00307
RGP1	0.221425067	0.018791	-0.032491316	0.72983	0.125741686	0.18083
MIF	0.419453291	0.01885	-0.289976387	0.10523	0.084742527	0.63549
TP5J2-PTCI	-0.681027033	0.018928	-0.830297131	0.00413	-0.667699936	0.02076
MID2	0.542151489	0.018921	0.129898461	0.57421	0.524382202	0.02271
PIGO	0.347504179	0.018949	0.063200695	0.6702	0.286615957	0.05257
P11-575G13	-1.268440782	0.018933	-0.646737025	0.19191	0.139237208	0.77006
TRAF6	0.348220767	0.018949	0.253896204	0.08685	-0.086781542	0.55977
ZKSCAN5	-0.242094218	0.018937	-0.344808912	0.00073	-0.349447522	0.00068
G2E3	-0.319697627	0.018975	-0.130314765	0.33499	-0.19611133	0.14855
ZNF160	-0.2684227	0.018976	0.252677992	0.02475	0.121118327	0.28351
MOCOS	-0.74158783	0.018997	-0.389240254	0.21602	-0.644459553	0.0398
RAP2C	0.643794994	0.018993	0.332636793	0.22571	-0.312475825	0.25795
SERPINA4	0.371991955	0.019082	0.014709062	0.92618	0.336364047	0.03397
PLEKHA5	-0.305914697	0.019099	-0.170317729	0.19082	-0.031288429	0.81012
RAB11B	0.445039228	0.019111	0.174651655	0.35913	0.366328373	0.05301
JUN	0.598045341	0.019126	0.77571272	0.00234	0.748753432	0.00333
P11-777B9	-2.20142774	0.019195	-0.077871143	0.93382	-1.945989891	0.03832
TSHZ1	-0.450470735	0.019194	0.043848473	0.81651	-0.283388517	0.13868
TLR2	0.527748482	0.019248	0.107043956	0.63696	0.762819179	0.00061
DUSP3	0.207641734	0.019262	-0.028302362	0.74966	0.076337604	0.38988
EIF5A	0.278646952	0.019296	-0.055988345	0.63838	-0.11042368	0.35453
WIPI1	0.293520698	0.019289	0.095572675	0.4447	0.118698857	0.34568
LAMTOR1	0.19660935	0.019323	-0.033623705	0.68795	-0.04730555	0.57616
POGK	-0.189251168	0.019327	-0.260572742	0.00122	-0.088857201	0.26983
DSCC1	-0.503920205	0.019364	-0.194939945	0.35583	-0.455403426	0.03355
DYNLL1	-0.206022322	0.019373	-0.161557754	0.06533	-0.44283154	5.28E-07
INCENP	-0.435852771	0.019374	0.043194931	0.81563	0.175696286	0.3443
FKBP14	-0.403705592	0.019393	-0.09208269	0.59262	-0.60915072	0.00042
ZBTB40	-0.220536573	0.019393	-0.224687073	0.0163	0.000317759	0.99728
SPATA21	0.886622687	0.019446	0.765460886	0.04408	1.065040699	0.00445
ZNF860	-0.596912636	0.019442	-0.255582427	0.29859	0.284267534	0.24072
AP005901.1	0.708327144	0.019512	0.058860192	0.84702	0.303613956	0.31864
HSDL1	-0.424641599	0.019525	-0.220889312	0.21985	-0.398698939	0.02788
TCN1	0.688031239	0.01953	0.73458449	0.01263	0.645326572	0.02848
CDKN2AIPN	-0.273095086	0.019652	-0.155058832	0.16973	-0.152857736	0.18121
HES6	-0.692924704	0.019677	-0.57260144	0.04841	-0.813869555	0.00641
ZFAND2B	-0.410595556	0.01968	-0.246181014	0.15652	-0.226049423	0.1948
SPOP	-0.242816846	0.019701	-0.128282779	0.21367	-0.227597206	0.0285
TBK1	0.240714668	0.019737	0.229954109	0.02482	0.082948621	0.42274

TPM3P9	-0.371912957	0.019778	-0.339190988	0.03078	0.097447135	0.52997
MANEAL	-0.774317313	0.019789	-0.44521069	0.15771	-0.392116562	0.21767
FOXA3	0.523525566	0.019853	0.237074586	0.29164	0.35128366	0.1184
KAT2A	-0.410498581	0.019835	-0.584555797	0.00094	-0.029984814	0.8646
OR7E14P	-0.649751521	0.019847	-0.176835169	0.50242	0.266619195	0.30423
FAM195A	-1.0196412	0.019874	-0.430011408	0.31169	-0.04780825	0.91016
DUOXA2	1.160630251	0.019887	-0.230210597	0.64515	0.797828164	0.10964
ETF1P2	0.802532541	0.0199	0.331573481	0.34067	1.173305943	0.00051
RGN	-0.933058824	0.019912	-0.450616004	0.24092	-0.593346662	0.12577
SFR1	0.381268306	0.02	0.504690311	0.00159	0.388434809	0.01673
BRF2	-0.376570452	0.020026	-0.030373252	0.84776	-0.35304578	0.02819
TOMM70A	-0.204944439	0.020053	-0.315467528	0.00033	-0.180314758	0.04046
SGOL2	-0.330583143	0.020125	-0.139811976	0.31929	-0.044411438	0.75231
SPRR3	1.771428253	0.020128	1.394337755	0.06759	0.678436821	0.37789
HPS3	-0.384108749	0.02015	0.043526767	0.79099	0.172210289	0.29473
RPP14	-0.223930487	0.020142	-0.304926554	0.00143	-0.181252604	0.05893
CNST	-0.263740204	0.020179	-0.307179041	0.0064	-0.232799855	0.03905
LPAR3	0.830278893	0.02016	1.113332022	0.00128	0.239863148	0.51103
TAP2	0.806802636	0.020171	0.162315635	0.64055	0.036670912	0.91638
FPGS	-0.341920411	0.020221	-0.08305122	0.56482	-0.316560658	0.03046
SH3BGRL3	0.407278123	0.020237	0.169097247	0.33529	-0.017099241	0.92249
WDTC1	0.293306197	0.020326	0.046877796	0.70994	0.421105274	0.00077
PNISR	-0.256118039	0.020416	-0.151898724	0.16817	-0.310362693	0.00495
SP110	0.42859577	0.020481	0.329353351	0.07067	0.413200589	0.02391
ATP10B	0.587298534	0.020513	-0.014810631	0.95347	0.532925374	0.03552
POLM	-0.377986175	0.020588	-0.18089853	0.25139	0.054898495	0.72823
UNC13A	1.484630923	0.020628	0.052697811	0.93572	0.084143949	0.89757
ELMOD3	-0.360518358	0.020647	0.050726971	0.7394	0.257843412	0.08897
IVNS1ABP	0.185864447	0.020648	-0.043344643	0.58846	0.136425604	0.08894
HDAC2	0.239108085	0.020691	0.13960696	0.17565	0.092702351	0.36985
ULBP3	-0.522598833	0.020709	-0.526107907	0.01846	-0.401067118	0.07275
ZNF629	-0.236038298	0.020712	-0.097257097	0.33581	-0.170839066	0.0925
P11-735A19	-0.211509358	0.020787	-0.318247985	0.00047	-0.215095258	0.01828
RWDD3	-0.304345879	0.020801	-0.264126347	0.0397	-0.3255424	0.0125
PRRC1	-0.225022479	0.020815	-0.353435134	0.00028	-0.126337316	0.19323
TRIM35	-0.531703742	0.02083	-0.625323168	0.00648	-0.207247574	0.36683
MXD1	0.648524877	0.020897	1.04385109	0.0002	0.678592785	0.01562
MED13	0.22010471	0.020987	0.225782445	0.01775	0.274686019	0.00395
DDX58	0.33082771	0.021022	0.479959221	0.00069	0.257887211	0.07154
TFEB	-0.46282999	0.021044	-0.552945212	0.00586	0.20264971	0.29309
YEATS4	-0.270013089	0.021063	0.026689488	0.81424	-0.335153736	0.00416
ZDHHC13	0.315656768	0.021073	0.340316985	0.01203	0.27611529	0.04259
CYP2B7P	0.912502292	0.021169	6.18E-05	0.99988	0.134708692	0.74241
RREB1	-0.370903619	0.021176	-0.117801413	0.46267	0.110151655	0.49121
AC241952.1	-0.488285581	0.021192	0.309864485	0.12483	0.24951719	0.21979
WARS	0.322017712	0.021199	0.493601748	0.0004	0.060005122	0.66807
ZDHHC8	0.399476704	0.021245	-0.106047923	0.54467	0.681050524	7.24E-05

CRBN	-0.233682422	0.021326	-0.237002781	0.01799	-0.359870616	0.00036
MPC2	-0.318801442	0.021317	-0.384359528	0.00529	-0.289940821	0.03561
PCDH12	1.239108985	0.021347	-0.407885601	0.45196	0.962582478	0.07138
HELZ2	0.587809226	0.021402	0.316632194	0.21553	0.701282913	0.00597
PHAX	-0.264713287	0.021466	-0.228528863	0.04612	-0.461024006	6.06E-05
MRPS22	-0.2062386	0.021503	-0.061508405	0.48589	-0.263406622	0.00327
ARAP2	0.55721003	0.021542	0.515813419	0.03316	0.189328292	0.43548
FBL	0.304763948	0.021559	-0.211263242	0.11329	-0.099158274	0.45731
LYPLA2	0.226193554	0.02163	-0.059799208	0.54391	-0.171243563	0.08471
PNO1	0.320825175	0.021603	0.019887535	0.88687	0.023333817	0.8679
SMURF1	0.231064289	0.021625	0.279720646	0.00529	0.407831323	4.79E-05
ZNF204P	-0.424059836	0.02162	-0.559440361	0.00234	-0.319306629	0.08172
CARD8	-0.334044674	0.021656	-0.212147058	0.14135	-0.06027752	0.67461
KIF11	-0.374119682	0.021684	0.055911943	0.73036	-0.192165674	0.23726
PPL	-0.714455647	0.021708	0.604726927	0.05121	-0.194133856	0.5321
MMS19	-0.21515245	0.021726	-0.103288412	0.26874	-0.01804803	0.8469
MTCH1	0.20560426	0.021761	0.168652147	0.05952	0.062083664	0.48869
NCAPD2	-0.431077997	0.021741	0.24242276	0.19611	-0.070503142	0.70718
TOB1	-0.486828317	0.021756	-0.652901619	0.00206	-0.280879532	0.18412
NAA16	-0.364044422	0.021773	0.030515335	0.84629	0.272166564	0.08331
UHRF2	-0.282239241	0.021786	-0.14177231	0.24548	-0.00720069	0.95307
THAP6	-0.297028001	0.02187	-0.108804176	0.38111	0.122461062	0.32609
CNKSR3	0.406816	0.02194	0.249584837	0.15875	0.16642431	0.34778
RPS26	-0.302596353	0.021939	-0.514560913	9.64E-05	-0.444360057	0.00077
EBF2	2.284847032	0.021964	3.175550216	0.00144	0.511053102	0.61017
KATNA1	-0.364967021	0.02201	-0.099307901	0.52704	-0.49006742	0.00216
TNRC6C	0.696154239	0.022033	0.619839932	0.04097	0.817397422	0.00708
PRELID1P1	-0.570675531	0.02206	-0.543099245	0.02599	-1.222702378	2.24E-06
TYRO3	-0.310647191	0.022122	-0.503835835	0.00021	-0.381631665	0.00492
SLC7A6OS	-0.346151067	0.022152	-0.082457775	0.58335	-0.236212446	0.11673
RPS6KB1	0.21920217	0.022166	0.282781512	0.00309	0.226384475	0.01838
MGAT5	0.256194249	0.022258	0.076000957	0.49777	0.357182248	0.00142
STRA6	0.928714035	0.022242	1.152087708	0.0045	0.451777451	0.26714
ZNF548	-0.289787361	0.022261	-0.075299394	0.54281	-0.176098078	0.15934
ANXA10	0.541232918	0.022321	0.009662886	0.96747	0.810122153	0.00062
TRMT44	0.313009079	0.022336	-0.239256729	0.08541	0.141821751	0.29985
GPSM1	0.510567172	0.022365	0.19036265	0.39472	0.157048341	0.48419
TC-359D24	-0.741261695	0.022406	0.054869031	0.86065	-0.349898729	0.27182
SHF	0.637121484	0.022412	0.375157479	0.17613	0.389095297	0.16202
WDR66	-0.379086774	0.02246	-0.586407399	0.0004	-0.210109364	0.20065
TRIM71	-0.746727218	0.022471	-0.955370436	0.00349	-0.717869087	0.02814
SRP19	-0.281310452	0.022499	-0.023743933	0.8463	-0.466118275	0.00016
ORC1	-0.41117399	0.022535	-0.400114785	0.0253	-0.285366184	0.11091
FILIP1L	0.388435282	0.022586	-0.164204787	0.33572	0.012021815	0.94381
LCN9	-0.674655436	0.022595	-1.152507904	0.00011	-0.075057566	0.79719
ESYT3	-0.868491788	0.022659	-0.29864052	0.41258	0.107501802	0.76613
FNDC4	0.424916126	0.022694	0.191936262	0.30282	0.064154131	0.73294

SMOX	0.740612585	0.022717	0.332118517	0.30729	-0.136602702	0.67615
PSD	0.712714092	0.022764	0.316081001	0.3131	0.109187624	0.73066
SVIL	0.347746662	0.022759	0.503322352	0.00096	0.459274877	0.00263
RFX1	0.623300733	0.022805	0.72901948	0.00717	1.222634718	5.78E-06
SAFB	-0.233584203	0.022797	0.170781877	0.09291	-0.010520911	0.91799
RLF	0.203634915	0.022862	0.433102142	1.08E-06	0.038482715	0.66736
HDHD2	-0.259058125	0.023065	-0.093207251	0.40723	-0.272511436	0.01668
VDR	0.530384972	0.023085	0.166103136	0.47691	0.676784024	0.0036
HEATR3	-0.35521147	0.023149	-0.118211874	0.44561	-0.012626434	0.93503
EPHX1	0.568147654	0.023186	0.440588305	0.07699	0.526394605	0.03514
BSCL2	0.425855259	0.02324	-0.126041574	0.50685	-0.500038946	0.00897
OTUB1	0.177835545	0.023234	-0.154468249	0.04891	-0.072223295	0.35876
SNX7	-0.22629172	0.023235	-0.353733242	0.00035	-0.195326115	0.04834
ZNF718	-0.559769708	0.023213	-0.422698525	0.08051	-0.072689701	0.76201
MRS2	-0.301515438	0.023305	-0.291219622	0.02734	-0.331878427	0.01229
TUBE1	-0.33702556	0.023341	0.32040319	0.02626	0.246361633	0.0915
RHOB	0.506857177	0.02338	0.005868535	0.97907	0.08708421	0.69712
RAE1	-0.244363528	0.023393	-0.466080212	1.56E-05	-0.351821534	0.00109
DLEC1	-0.78916697	0.02344	-0.737027133	0.03349	-0.016197543	0.96182
FNDC3B	0.401936932	0.023445	0.271744766	0.12542	0.102419948	0.56366
MAPK7	0.451104651	0.023418	0.579902214	0.00341	0.374494613	0.06008
MTHFD2	0.44768189	0.023436	0.342092009	0.08324	0.007174641	0.97105
AKAP13	0.268653235	0.023499	0.266128642	0.02469	0.540944301	4.95E-06
KIAA1958	0.327072138	0.023489	-0.138077109	0.33646	0.402965319	0.00457
PSD4	-0.360126565	0.023584	-0.556068554	0.00046	-0.25496654	0.10782
MITD1	-0.270299858	0.023716	-0.236990978	0.04221	-0.089749964	0.44394
CEP290	-0.286551281	0.023812	-0.079057646	0.53016	-0.001691026	0.98931
PAXBP1	-0.227762842	0.023814	-0.223424943	0.02573	-0.105618207	0.29254
TNFAIP6	1.032932899	0.023929	0.460155213	0.31791	-0.020095481	0.96591
CERS6	0.234612505	0.024001	0.244927236	0.01819	0.159005684	0.12593
CCT6P1	-0.430406315	0.024055	-0.30899276	0.09503	-0.025510838	0.88937
PSRC1	-0.505390423	0.024046	-0.13137965	0.55207	-0.498245135	0.0256
CCDC117	-0.276734418	0.024081	0.045471231	0.70827	-0.114990611	0.347
RP1-152L7.!	-0.54700809	0.024151	-0.40508904	0.09243	-0.076307804	0.75016
ZNF337	-0.228080109	0.024154	-0.191147072	0.0515	-0.070261671	0.47523
MMP25	1.136965408	0.024194	0.808981076	0.10611	1.176365692	0.02182
UBC	0.35094261	0.024195	-0.030984135	0.84226	-0.034497218	0.82467
ARF4	0.235515215	0.024257	-0.168800227	0.1064	-0.089020496	0.39472
EFCAB2	-0.385551184	0.024255	0.243342025	0.13672	-0.309847107	0.06295
FAM129A	0.643759919	0.024289	0.121300065	0.67288	0.809153055	0.00458
NRBF2	0.227342156	0.024257	-0.194827078	0.05467	-0.032659308	0.74728
ORC2	-0.278186824	0.024287	-0.012244732	0.92034	-0.084101337	0.4935
PPP1CC	-0.17130455	0.024281	-0.004712399	0.95041	-0.252870069	0.00088
SQLE	-0.245432306	0.024294	0.180941361	0.09647	-0.146044288	0.17995
MORN2	-0.374724843	0.024365	-0.151106128	0.34388	-0.462325371	0.00522
NUP37	-0.253245734	0.024511	-0.095112808	0.38658	-0.278630304	0.01232
SERPINI1	-0.800028928	0.024507	-0.661843493	0.05901	-0.957468175	0.00901

USP53	0.38745617	0.02459	0.318526173	0.06455	-0.184181897	0.28585
CAD	-0.319924105	0.024642	0.123595826	0.38227	0.397765173	0.00489
DNMBP	-0.249492004	0.02466	-0.022281811	0.83946	-0.014372776	0.89631
HSF1	0.329888542	0.024646	0.208045503	0.15557	0.344196116	0.01866
KNSTRN	-0.340245054	0.024654	0.174249689	0.24148	-0.144034386	0.33732
VGLL3	0.901159076	0.024635	0.56456261	0.15921	0.25953785	0.51781
SYF2	-0.245557991	0.024696	-0.073588097	0.4933	-0.498437244	5.86E-06
ZNF75A	-0.313004466	0.024685	-0.088370778	0.52118	-0.024713044	0.85841
FKBP11	0.450683277	0.02471	-0.341539892	0.08856	0.361244235	0.0694
C19orf52	-0.427764186	0.024762	-0.336634664	0.07049	-0.181591321	0.33128
DDB1	0.159448185	0.024775	0.08214605	0.24665	-0.069469004	0.32848
MT-ATP8	0.751311143	0.0248	0.170134441	0.61173	0.593790339	0.07611
PCDHGB4	0.593427016	0.024796	0.732753853	0.00542	0.536180839	0.04243
TMEM74B	0.782364659	0.024843	-0.869187453	0.01991	-0.079534097	0.82462
DECR2	-0.672970619	0.024944	-0.397903299	0.18111	0.019870738	0.94622
SPIN3	0.436413615	0.024991	-0.234132803	0.24258	-0.003086217	0.98755
NAA50	0.337323994	0.025058	0.318575314	0.03389	0.220015028	0.14355
LRCH1	0.250681658	0.025121	0.09595931	0.38885	0.29529024	0.00795
APOL1	0.907955894	0.025134	1.220099868	0.00243	1.617780986	5.62E-05
SCML4	-0.862290344	0.025154	-1.339039324	0.0005	-0.061308973	0.87048
EIF4G2	0.338785163	0.025198	0.502565764	0.0009	0.116433948	0.44174
HS2ST1	-0.276222997	0.025258	-0.15688151	0.20258	-0.277518712	0.02459
RAD54L2	0.211439348	0.025257	0.128206966	0.17209	0.350973219	0.00018
CTSE	0.44830057	0.025413	0.916888886	4.83E-06	0.615756466	0.00214
DHODH	-0.360782292	0.025431	-0.284050511	0.07027	-0.307570227	0.05004
ADAM11	1.204622458	0.025445	0.079552695	0.88296	0.286504617	0.60431
ANKS4B	-0.86677357	0.025522	-1.311823426	0.00073	-1.175672189	0.00248
NR1H2	0.336301987	0.025516	-0.091775574	0.54387	0.278182996	0.06517
NBR2	1.216630059	0.025536	0.13499088	0.80296	0.68273955	0.20352
SPAG9	0.307735784	0.025562	0.243798082	0.07695	-0.023299864	0.86595
RBMXL1	-0.233158205	0.025584	-0.077431262	0.45299	-0.113219308	0.27506
SMPD1	0.426382069	0.025626	0.192049713	0.31364	0.105173511	0.58663
SPATA2	0.341680035	0.025667	-0.058981171	0.7021	0.415407816	0.00597
ZBTB9	-0.322261057	0.025721	-0.646265506	8.07E-06	-0.402569339	0.00521
EEF1A1P1	-0.611453725	0.025761	-0.336619235	0.19763	-0.942041955	0.00076
B4GALT7	0.281615526	0.025779	0.034290982	0.78541	0.167423138	0.18332
SPEG	0.696847733	0.0258	0.044221258	0.88762	0.224906138	0.47195
ASAH2B	-0.416983556	0.025828	-0.324268342	0.07865	-0.123050753	0.50486
CEP70	-0.241916449	0.025853	-0.077891411	0.46367	-0.309048858	0.00421
OXSM	-0.490953775	0.025888	-0.253776346	0.22732	-0.141710154	0.50597
ZNF554	-0.325719341	0.025889	-0.067097674	0.63783	0.009222639	0.94831
PPP2R1B	0.26823186	0.025913	-0.089192298	0.45893	0.071490153	0.55259
CTGF	0.754524391	0.025978	-0.333138871	0.32676	-0.38355591	0.25926
B3GALT2	-0.762410289	0.026038	-0.470224343	0.1658	-0.856902292	0.01231
CDC37	0.237845458	0.026104	-0.115548715	0.27994	-0.094257492	0.37891
SFN	-0.483295496	0.026129	-0.022926051	0.91467	-0.209816571	0.32974
WDR60	-0.471393584	0.026143	-0.389663541	0.06569	-0.339618061	0.10865

AC243772.2	0.452012846	0.026159	0.018385554	0.92863	0.436573254	0.03047
DDX3X	0.438738783	0.026205	0.565715582	0.00412	-0.036526987	0.85336
HSPE1P3	-1.516515334	0.026228	-0.792347594	0.23424	-0.797200269	0.23268
RQCD1	0.201626608	0.026242	0.222811839	0.01364	0.08436721	0.35234
RP11-762I7.	0.784439182	0.026262	-0.279299346	0.43634	0.389191331	0.27108
TPMT	-0.259749301	0.026325	-0.137286552	0.23319	-0.242792446	0.03672
TRIM65	-0.378329617	0.026317	-0.292076125	0.08126	-0.136931343	0.41388
EMC10	0.224023248	0.026343	0.065587332	0.51465	-0.173659355	0.08583
CD101	-0.933764214	0.026404	-0.182791861	0.64968	-0.267491015	0.52089
MRPS26	-0.339063421	0.026479	-0.626901265	4.10E-05	-0.324884835	0.0325
XPA	-0.354898153	0.026474	-0.02054214	0.89581	-0.093926309	0.5534
TNFRSF14	0.802344582	0.026529	0.088536557	0.81462	0.635380946	0.09013
KCNE1	0.794728504	0.026599	0.709991567	0.04598	0.649344855	0.07016
PPAT	-0.327851134	0.026597	-0.305893759	0.03761	-0.163607962	0.26682
ARHGAP18	-0.285514561	0.026769	-0.606537284	2.57E-06	-0.178070528	0.1665
CHST1	0.924758861	0.026773	0.759936458	0.06767	-0.443200258	0.32779
EEF1GP1	0.297135011	0.026702	-0.255552738	0.05962	0.174923684	0.19196
ERBB3	-0.329992539	0.026689	-0.608641429	4.36E-05	0.09557167	0.52061
FAM174A	-0.378007292	0.026717	-0.043037402	0.79569	-0.328595539	0.05262
ISG20	0.585516081	0.02673	0.272383353	0.30292	-0.010819123	0.96748
MCTP2	-0.522179835	0.026729	-0.304718857	0.19284	-0.876419743	0.0002
SEZ6L2	0.978048746	0.026713	-0.151197084	0.73267	1.473087722	0.00083
STRBP	-0.276488959	0.026739	-0.00914567	0.94101	-0.010641	0.93172
TPX2	-0.38550881	0.026773	0.003183682	0.98537	-0.502549518	0.00389
AFM	-1.355400414	0.02695	-1.603368829	0.00878	-0.879877504	0.14676
TB-134H23	1.056634535	0.027007	1.061478223	0.02	0.928212538	0.04445
EFNA4	-0.397354249	0.026969	-0.269882248	0.11768	-0.666694932	0.00026
GATA4	-0.678833283	0.027019	-0.694793041	0.02332	-0.378579676	0.21449
IFT74	-0.30548953	0.026919	0.080626917	0.55181	-0.446833988	0.00121
KDM1A	-0.163762043	0.026924	-0.017882135	0.80794	-0.272065461	0.00024
KIF14	-0.901973019	0.026912	0.50320619	0.20992	0.139680568	0.72861
LMAN2L	-0.223697273	0.026991	-0.36295064	0.00031	-0.194260614	0.0541
LYSMD3	-0.359992061	0.026948	-0.144734782	0.37206	-0.339240656	0.03674
MCEE	-0.444437084	0.02692	-0.313610688	0.11275	-0.261501981	0.1879
MEOX1	1.698311037	0.026838	2.140756727	0.0052	0.474234499	0.5389
NDUFAF6	-0.350420693	0.026962	-0.106737995	0.48321	0.268507568	0.07597
NUF2	-0.405715749	0.026907	0.085933595	0.63259	-0.326149488	0.07334
RHOQP1	-0.875421946	0.027014	0.016394321	0.96324	-0.17159831	0.63785
RPS17L	-0.359276816	0.026962	-0.384261118	0.01794	-0.452057694	0.00538
SH3GL1	0.236706546	0.02699	-0.043041394	0.68767	0.043400347	0.68568
STARD3NL	0.172054262	0.027021	0.176416699	0.02136	-0.150563357	0.057
TMEM245	-0.195881062	0.026915	-0.372482098	2.54E-05	-0.217396861	0.01385
UBALD1	0.46165248	0.027028	-0.189226585	0.37014	0.146652728	0.48584
EIF1	0.381779668	0.027111	-0.211775414	0.22075	0.22754786	0.18783
ADNP	-0.34679237	0.027141	-0.150046898	0.3379	0.254000372	0.10413
LGR4	-0.26557916	0.027157	-0.200426973	0.09507	-0.081553177	0.49715
PRPF8	-0.364568321	0.027162	0.270818266	0.10022	0.151308132	0.35831

TXNL4B	-0.350205167	0.027136	-0.227494476	0.14081	-0.287195861	0.06672
GLP2R	0.742777577	0.02719	0.911113881	0.00668	0.209720776	0.53482
KBTBD3	-0.642959623	0.027267	-1.166151846	5.19E-05	-0.330562183	0.24386
ACN9	-0.390546821	0.027305	-0.226723474	0.1944	-0.546261122	0.00211
CCDC134	-0.37599814	0.027316	-0.285650486	0.08363	-0.184912304	0.26703
TNNC1	-0.676516236	0.027303	-0.842371312	0.00571	-1.336136918	2.40E-05
JMJD6	0.308204091	0.027347	0.215576827	0.12028	-0.047577355	0.73374
TD-2561J22	-0.487940415	0.027414	-0.313335268	0.14696	-0.181403738	0.40154
SPTSSB	-0.904066637	0.027424	0.159069796	0.69339	0.36912289	0.36069
C14orf142	-0.38269604	0.027446	-0.177042964	0.276	-0.501093473	0.00377
BFAR	-0.288438163	0.027473	-0.102927446	0.42957	-0.316261879	0.01555
SF3B14	-0.1985233	0.027488	-0.076109217	0.39177	-0.470033919	2.10E-07
BAIAP2L2	0.812751677	0.027533	-0.270654336	0.46783	0.01277884	0.97266
P11-438J1.	0.51164207	0.027551	0.493629048	0.03215	0.679074896	0.00318
RC3H1	0.311299749	0.027572	0.207297969	0.14197	0.347772482	0.01375
U2SURP	-0.216851975	0.027597	-0.055275843	0.57376	-0.291844907	0.00303
APLP1	0.664491239	0.027628	0.302208502	0.31827	0.471641225	0.11846
FAM53B	-0.318771408	0.027622	-0.577859674	6.34E-05	-0.332562524	0.02104
GPR50	1.41110208	0.027664	1.63114444	0.01071	-0.532331151	0.4163
STX8	-0.234911169	0.02767	-0.195483407	0.06197	-0.453723751	2.28E-05
MCAT	-0.310090058	0.027748	-0.34754537	0.0125	-0.263560236	0.05861
PSMC5	-0.222618636	0.027761	0.027926565	0.77919	-0.282764568	0.00513
FAS	0.585235471	0.027793	-0.301965099	0.25864	0.393387045	0.13933
CDC42BPB	0.189479632	0.027809	0.017397548	0.83954	0.300333197	0.00047
CDKN2B	0.762973838	0.027825	0.596287607	0.0853	0.12885329	0.71055
USP11	0.324761702	0.027833	0.182235413	0.21694	0.138341363	0.34929
CD59	0.364208282	0.027911	0.145412122	0.37985	0.116409153	0.48235
TNFRSF21	0.275085337	0.027908	0.025599479	0.83786	0.107314553	0.39119
TUBA1C	0.341675672	0.027905	0.331567357	0.03283	-0.042926833	0.78247
AP001885.1	-0.794186173	0.028007	0.395109066	0.19674	0.146314064	0.64416
FAM178A	-0.320624848	0.028	0.272579335	0.05917	0.082841796	0.56796
FNIP1	-0.172462336	0.027983	-0.054197406	0.48417	-0.035561979	0.64727
PRUNE2	-0.690526047	0.028005	-0.356133724	0.25705	0.484100256	0.11828
SLAIN2	0.390687693	0.028024	0.191867812	0.28019	0.148388736	0.40374
C6orf47	-0.229789587	0.028063	-0.197712129	0.05474	-0.227691753	0.02837
SERAC1	-0.275221059	0.02807	-0.123358412	0.31541	0.066157142	0.59078
NRIP1	0.180161635	0.02823	-0.342526819	3.14E-05	-0.032454083	0.69296
ZBTB46	0.72200242	0.028246	0.430625723	0.18942	0.248756715	0.45044
FHDC1	0.487292915	0.028264	-0.003119808	0.98882	0.655658036	0.00307
ASPM	-0.408436587	0.028311	0.293046271	0.11442	-0.214218514	0.24946
FAM129C	-0.374063885	0.028405	0.080399	0.62632	0.330669661	0.04349
USP30	-0.48865175	0.028428	-0.331501674	0.13604	-0.422034431	0.05731
CDHR3	-0.754953035	0.028447	-0.138403536	0.67872	0.323744947	0.32313
TD-2521M24	-0.845151991	0.028455	-0.596011557	0.10398	0.19467017	0.57897
RAB4A	-0.274276817	0.028497	-0.302987939	0.01512	-0.400125858	0.00135
PTPRC	-0.631483987	0.028682	-0.279452367	0.31374	0.091037434	0.74053
STX3	-0.433208826	0.028678	-0.491920456	0.01281	-0.467752806	0.01808

EMILIN3	1.042154132	0.028699	1.245170716	0.0083	0.678947009	0.15663
SAPCD2	-0.516593695	0.028723	-0.520995363	0.02662	-0.242683335	0.30075
PI4KB	0.250216929	0.028755	-0.174417215	0.12812	0.162910887	0.15468
RAB7A	0.403225434	0.028763	0.646355396	0.00045	0.207800268	0.25994
TTLL4	0.210293991	0.028765	-0.238762025	0.01331	0.513096256	7.92E-08
ADAM17	0.234584952	0.028832	0.117141044	0.27414	-0.006144403	0.95444
RPL36A	-0.396611833	0.028882	-0.650276889	0.00034	-0.438420043	0.01567
KDELC1	-0.414680834	0.028915	-0.085709832	0.64667	-0.44507221	0.01927
GOSR2	-0.204668145	0.028932	-0.024638424	0.78952	-0.051269685	0.57935
ATP2B4	0.284716885	0.028966	-0.030447146	0.81537	0.348632152	0.00745
MMP7	1.522311111	0.028975	0.996615641	0.15293	0.68978994	0.32287
USP20	-0.443953922	0.028977	-0.181966125	0.36949	-0.078380619	0.69828
FAM63B	-0.222994533	0.029025	-0.204321594	0.04452	-0.151873177	0.13555
C6orf211	-0.240353927	0.029069	-0.228763076	0.03633	-0.318422291	0.00386
DEK	-0.292969595	0.02907	0.247955715	0.06408	-0.458222102	0.00064
ZNF83	-0.210494516	0.029124	0.03535253	0.70942	0.240181334	0.01127
CCDC25	-0.268067937	0.029189	-0.055208186	0.65159	-0.442526402	0.00031
PIGM	-0.316863929	0.029214	-0.138366814	0.3323	-0.206199068	0.15177
SERPINB5	-1.824333267	0.029254	0.668127755	0.38864	-1.633812098	0.0447
ACAD11	-0.501430545	0.029276	0.117985073	0.6049	-0.468112773	0.04096
HJURP	-0.534250013	0.029288	-0.089868458	0.71184	-0.193839175	0.42655
ABHD6	-0.392771882	0.02938	-0.422276179	0.01827	-0.122972094	0.4903
SLC35D1	-0.386611873	0.029393	-0.22630102	0.20093	-0.257927553	0.14541
PEAR1	0.930647461	0.02942	0.897967459	0.03465	0.250031009	0.55896
SETD4	-0.324059821	0.029437	-0.104300885	0.47533	0.167562054	0.24863
SYTL1	-0.554101578	0.02943	-0.996082686	9.34E-05	-0.016082947	0.94912
SLFN12L	0.615951481	0.029533	0.723082887	0.00927	0.945538322	0.00064
CNPPD1	-0.267239459	0.02962	-0.321353719	0.00798	0.040638241	0.7346
FGD3	-1.50373223	0.029636	-1.461635447	0.03337	-2.609571029	0.00023
ABRACL	-0.277639212	0.029834	-0.068025289	0.58608	-0.359599402	0.00481
KRT5	-3.34410056	0.029831	1.027841809	0.49792	-3.711110543	0.01603
LIMD1	0.228633267	0.029853	0.005774438	0.95588	0.391142982	0.00016
PDIK1L	-0.683474734	0.029815	-0.531219399	0.08951	-0.323639765	0.30077
TPD52L2	0.215932145	0.029842	-0.104895732	0.29179	-0.02868589	0.7734
TRIM8	0.31425408	0.029838	-0.089127452	0.53781	0.354525566	0.01409
SP6	-0.916623391	0.02994	-0.175458271	0.66771	-1.957042274	1.08E-05
SLC13A3	0.62936715	0.029975	-0.273177467	0.35312	-0.004743266	0.98714
C4orf46	-0.323235571	0.030023	-0.084474986	0.56669	-0.324193735	0.02898
KHDRBS1	0.191470707	0.03003	0.161887466	0.06578	0.022582355	0.79816
SPSB2	-0.439368078	0.030023	-0.421924199	0.03358	0.007085075	0.97109
BCAM	0.477750704	0.030056	0.358701211	0.10284	0.338606904	0.12412
RFWD2	-0.2051336	0.030093	-0.268433324	0.00429	-0.170464699	0.07019
C11orf48	-0.249222095	0.030115	-0.132721933	0.24127	-0.329099217	0.004
ZNF638	-0.22591212	0.030135	0.048446475	0.64151	-0.119146305	0.25259
CDK11B	0.378270745	0.030216	0.487167152	0.00506	0.023314589	0.89415
SLC38A2	0.278144551	0.030217	0.311520302	0.01516	-0.12044966	0.34809
SNX2	-0.253703735	0.030242	0.021875442	0.85095	0.00132095	0.99096

NAIF1	-0.505830401	0.030256	-0.850196559	0.00021	-0.297288522	0.19142
RBM12B	-0.415577744	0.030267	0.016412393	0.93134	-0.199127859	0.29671
NDUFA10	0.654310272	0.030296	0.638019083	0.03376	0.550558635	0.06701
FUT10	-0.313534386	0.030329	-0.013413721	0.92511	0.006775534	0.96209
NSUN2	0.169394131	0.030336	0.030123788	0.69899	0.093957775	0.22963
ZNF532	0.320869808	0.030333	0.687411921	3.31E-06	0.521706178	0.00042
PIP4K2A	-0.359506819	0.030452	0.174252196	0.29081	-0.220892606	0.18342
RBM38	0.549308824	0.030478	0.381378922	0.1323	0.017650916	0.94485
NPC2	0.204004188	0.030622	-0.015293048	0.87136	-0.049528856	0.60023
TOX4	0.262155915	0.03066	0.334277029	0.00571	0.040729435	0.7373
VCL	0.235539591	0.030665	0.145356445	0.1821	0.047429584	0.66347
TLN1	0.294767701	0.030683	0.132459696	0.33151	0.057323268	0.67431
MTO1	0.295535336	0.030698	0.208799959	0.1236	0.355553607	0.00866
ACE	0.846179186	0.030808	0.349581669	0.36424	0.318031525	0.41703
AC010240.2	0.485147309	0.030843	0.344505862	0.12031	0.737621413	0.0007
RBL2	-0.168357938	0.030834	-0.263726517	0.00068	-0.012234591	0.87456
BNIP3P1	-1.936824543	0.030872	0.147367076	0.86805	-2.230420238	0.01425
HHIP	0.714280839	0.030908	0.949152438	0.00407	0.864250322	0.00895
ZNF608	-0.332747367	0.030936	-0.060598766	0.69239	-0.126778031	0.40925
ZNF594	-0.409911858	0.030957	0.198402846	0.27855	0.004319022	0.98139
FBXO22	-0.302540539	0.03101	-0.482083784	0.00054	-0.247552913	0.07528
PIGY	0.69647308	0.03101	-0.102276793	0.75625	0.371344398	0.25343
STYK1	0.642935999	0.030988	0.789225666	0.0079	0.863224021	0.0037
MRPL2	-0.308107206	0.031044	-0.384984594	0.00649	-0.20281266	0.15411
UHRF1BP1	0.160752746	0.03104	0.142107405	0.05388	0.290119799	8.31E-05
NUP188	0.300166159	0.031118	0.322869785	0.02018	0.233054936	0.0938
ZNF598	0.416512241	0.031131	-0.03548526	0.85464	0.226642585	0.24085
CCDC124	0.398864647	0.031161	0.250541445	0.17466	0.040201301	0.82929
EMC8	0.247737643	0.031161	0.236854264	0.03779	0.243817626	0.03341
ZNF570	-0.37674228	0.031266	-0.156895084	0.36238	-0.315040078	0.06924
RNASE10	0.675109463	0.031398	-0.164246504	0.61412	0.515066236	0.10119
C6orf163	-0.632751713	0.03148	0.034042363	0.90161	0.2189828	0.42511
SMTNL2	-1.225091735	0.031467	-0.298262902	0.58574	-1.456238051	0.01084
C6orf62	0.234758763	0.031595	0.094219529	0.38742	0.097521814	0.37177
GCSH	-0.324139559	0.03166	-0.297331243	0.04735	-0.413625284	0.006
NUDT6	0.583669003	0.031664	0.57231654	0.03065	-0.11396116	0.67084
PLAG1	-0.316423945	0.031614	-0.011189297	0.9389	-0.15819403	0.28042
PLRG1	-0.193307063	0.031665	-0.278636455	0.0018	-0.391515981	1.38E-05
YBX3	0.305272008	0.031635	0.28825621	0.04215	0.110074456	0.43823
KCNK5	-0.520712752	0.031682	-1.063304504	1.21E-05	0.095372271	0.69239
GFRA1	0.802577307	0.031704	1.535692801	3.60E-05	0.571274908	0.12633
BAG2	-0.454775402	0.031785	-0.070929981	0.73366	-0.392075178	0.06276
EID2B	1.078304763	0.031765	0.466570487	0.34017	0.80287851	0.10774
KIF12	-0.61122548	0.031779	-0.759397301	0.00764	-0.488332769	0.08538
NPC1	0.259942215	0.031782	0.230601939	0.05658	0.383172909	0.00154
PIF1	-0.690302729	0.031809	0.051786793	0.86836	0.324008339	0.29782
SIMC1	0.26550562	0.031805	0.075239788	0.54171	0.692944611	1.47E-08

SPOCD1	0.783674972	0.031808	-0.663657139	0.07283	0.526034564	0.15055
THAP11	-0.336421892	0.031822	-0.48855798	0.00173	-0.133676388	0.38766
SKA2	-0.314068411	0.031899	0.350500819	0.0159	-0.436156224	0.00288
ESD	-0.189354954	0.031954	-0.201093463	0.02189	-0.348624908	7.90E-05
KLLN	-0.418323556	0.032029	0.035553877	0.84194	-0.218320901	0.242
KTN1	-0.185940181	0.032012	-0.068732726	0.42699	-0.251845924	0.00368
P11-835E18	-0.498230739	0.032019	0.038879591	0.85602	0.250833373	0.23974
MEGF8	-0.53026695	0.03211	0.13188292	0.59338	-0.158924634	0.52007
MRPS5	-0.202745134	0.032109	-0.069960772	0.45249	-0.202953983	0.03079
PIK3R3	-0.360350408	0.032114	-0.192429916	0.25206	-0.399399335	0.01751
TMEM259	0.235176392	0.032176	0.058895354	0.59138	0.074918348	0.49526
HAPLN4	0.99367012	0.032214	0.695916289	0.13012	0.733251936	0.11189
ZNF93	-0.236498362	0.032219	0.016636236	0.87761	-0.16433712	0.13491
ASXL1	-0.311733805	0.032277	-0.36991859	0.01086	-0.176288246	0.2249
DHX8	0.170228954	0.032299	-0.011437714	0.88492	0.013996158	0.86016
MND1	-0.494181536	0.032392	0.118902797	0.58987	-0.307257035	0.17508
EXPH5	-0.482580558	0.032437	-0.178795465	0.42625	0.136078758	0.54424
ANTXR2	-0.313029314	0.032466	-0.450834298	0.00205	-0.653828301	8.40E-06
POLR2H	-0.250047117	0.03253	-0.065250162	0.57566	-0.150666778	0.19625
ATF7IP2	-0.345673563	0.032611	-0.506930643	0.00171	0.040878125	0.79985
STXBP4	-0.366002836	0.032705	-0.071992985	0.673	0.03370334	0.8434
BDKRB2	0.949437492	0.03275	0.379546575	0.39461	-0.400245601	0.37606
CDK9	0.305962173	0.032763	-0.3534945	0.0136	0.152276116	0.28769
TSG101	0.265888167	0.032765	0.148450603	0.23218	-0.015974079	0.89824
ZNF578	-1.021077135	0.032767	-0.653465849	0.15599	0.0017062	0.99701
BZW2	0.239666092	0.032855	0.029935444	0.78967	-0.051354853	0.64787
C1orf226	-0.567203146	0.032883	-1.042967043	0.00012	-0.44202627	0.09483
C3orf70	0.437482319	0.032841	0.303414366	0.13718	0.194432847	0.34469
TMEM161B	-0.23530858	0.032875	-0.253010388	0.01932	-0.136242801	0.2077
TSPYL4	0.261262928	0.032886	0.199726482	0.10009	0.229972997	0.05942
ZNF836	-0.346846391	0.032895	-0.279626878	0.07474	-0.213540597	0.17878
MRPL16	-0.229636223	0.032917	-0.253301732	0.0176	-0.251422859	0.01901
AC010620.1	-0.283211868	0.033016	0.084911743	0.51769	-0.077156968	0.55869
GNAQ	0.225424095	0.033027	0.279606653	0.00799	0.405829518	0.00012
NLGN4X	0.536101485	0.033031	0.56802205	0.0234	0.365035737	0.14669
BBS7	-0.467236603	0.033083	-0.473745169	0.02995	-0.663834264	0.00257
ARHGAP42	0.222611321	0.033172	0.140216207	0.17944	0.095466491	0.36213
BUB1	-0.390748463	0.033202	0.010258779	0.95516	-0.226027	0.21711
FAIM	-0.39307656	0.033191	0.072486165	0.68348	-0.588624556	0.00136
PXDC1	0.293428214	0.033267	0.040294236	0.77001	0.208603617	0.13007
ATP5D	0.768805667	0.033389	0.291344478	0.42152	0.900463814	0.01239
PITX2	-0.902551595	0.033477	-0.213318043	0.61334	-0.647853471	0.12622
SH3PXD2A	0.282685318	0.033487	-0.06335794	0.63376	0.340151675	0.01039
C11orf95	-0.303709172	0.033541	-0.536750499	0.00017	-0.519387328	0.00028
MRPS10	-0.190264419	0.033533	-0.083390993	0.34253	-0.104578227	0.23756
ABT1	-0.191554107	0.033759	-0.359806431	6.13E-05	-0.466965215	2.76E-07
GSTP1	0.209214838	0.033745	-0.188960807	0.05518	-0.007154816	0.94213

PARVA	0.274588697	0.033742	0.084184056	0.51374	0.242343791	0.06022
SIX5	0.645202166	0.033757	0.380715318	0.2113	0.679612443	0.02548
TD-2328D6	1.10791064	0.033805	-0.052857795	0.9195	2.004532601	0.00012
SLC39A8	-0.636591893	0.033792	0.254845148	0.38448	-0.236719482	0.42658
ARNTL	-0.528072255	0.033892	-0.327791039	0.18756	-0.338866346	0.17144
PPIL4	-0.172895119	0.033902	0.06824069	0.39305	-0.240561853	0.00313
ART5	0.995234575	0.033947	0.545584928	0.24399	0.339876479	0.4751
PTPN2	0.223992419	0.033935	0.322466948	0.00205	0.260968218	0.01308
TMED9	0.236435242	0.034025	-0.25312873	0.02365	0.025713149	0.81793
TP53TG5	0.759504449	0.034052	0.631268663	0.07727	0.798880082	0.02537
VMA21	-0.180714669	0.034085	-0.160272376	0.0585	-0.161174178	0.05808
C15orf48	0.796017166	0.034161	1.669436444	7.09E-06	0.55191771	0.14247
SHROOM2	0.502382403	0.034197	0.414362509	0.07978	0.659443722	0.00519
TRIQK	-0.342218675	0.034195	-0.221526227	0.16934	-0.281703626	0.0808
C21orf2	0.26398645	0.03427	-0.132339759	0.29344	0.336430025	0.00672
ZNF692	-0.358757946	0.034282	-0.525662744	0.00186	-0.135855035	0.41939
ENDOD1	-0.269702367	0.034307	-0.108956673	0.38844	-0.367205114	0.00393
SAE1	-0.218591717	0.03432	0.038891426	0.70529	-0.333515299	0.00124
ITGB1BP2	-0.898902411	0.034449	-0.313639431	0.41558	0.002528132	0.99469
C8orf48	-0.621441406	0.034505	-0.587079308	0.04049	-0.491122693	0.08793
ZNF708	-0.236837549	0.034542	0.08769983	0.42699	0.027550226	0.80359
C4A	0.938877604	0.03462	0.170034963	0.70236	0.263618245	0.5517
CABIN1	-0.321573151	0.034619	-0.618266809	4.94E-05	0.331828527	0.0289
KCNJ6	0.601356349	0.034593	0.742588724	0.00805	0.956446858	0.00063
PIM1	0.67118045	0.034625	0.950507629	0.00269	0.419424511	0.1875
ZNF304	-0.250224786	0.034578	-0.208447289	0.0737	-0.349329362	0.00311
ZSCAN9	-0.348349542	0.034649	0.146438853	0.35655	-0.201994099	0.21278
PPME1	0.271312377	0.034709	-0.275923319	0.03298	0.129302206	0.31626
DUS4L	-0.375401418	0.03477	0.235919495	0.16249	-0.149281595	0.38802
NOLC1	0.373079613	0.034761	0.514084923	0.00361	0.067172707	0.70402
GXYLT1	-0.258108936	0.034817	-0.165781638	0.17314	-0.37708218	0.00203
SEMA4B	0.470499095	0.034833	0.528312915	0.01772	0.529664811	0.0175
ZNF681	-0.402587511	0.034837	-0.093146622	0.62062	-0.402201614	0.03353
ZNF714	-0.682778309	0.034807	-0.591587879	0.05919	0.014908718	0.96097
PRKRIP1	-0.303892685	0.034908	-0.327195943	0.02192	-0.341265765	0.01722
ZNF468	-0.382875121	0.034902	-0.430384898	0.01757	-0.211529065	0.24273
KIF3A	-0.351436791	0.03493	-0.303636713	0.06664	-0.485897496	0.00337
RAB8A	-0.312792931	0.034966	0.127415178	0.38913	-0.453804224	0.00222
P11-460N11	0.854327055	0.034993	0.18590032	0.65031	1.466306799	0.00024
S1PR2	0.573325892	0.035225	0.517233412	0.05703	0.349210014	0.20003
ZNF90	-0.380150748	0.035221	-0.278960358	0.11523	0.057988543	0.74212
DNAL4	-0.309954478	0.035255	-0.55520051	0.00015	-0.367137048	0.013
RBM7	0.264317265	0.035253	0.023172471	0.85342	-0.260733281	0.03883
LGALS9	0.625734763	0.035281	0.491642552	0.09757	0.665405006	0.02491
WDR3	-0.326294349	0.035297	-0.362594422	0.01916	-0.207105475	0.18113
PARD6B	0.309101261	0.035322	0.319126779	0.0293	0.035947742	0.80695
FZD6	0.353928669	0.035379	0.361999404	0.03074	0.26565548	0.11402

YWHAG	0.162390286	0.035381	0.024972537	0.74601	-0.167466383	0.03033
EEF1A1P12	0.383692815	0.035523	0.13810467	0.44805	0.237754297	0.19306
RNF219	-0.294974359	0.035545	-0.221894369	0.1127	-0.454122015	0.00122
VPS33A	-0.294238129	0.035536	0.067392848	0.62568	-0.114755998	0.40922
ACTG1	0.300918597	0.035617	0.208470774	0.14543	-0.00847688	0.9528
HDGFRP3	-0.245999973	0.035614	0.325069574	0.00516	-0.416576425	0.00037
LAMA5	-0.401736393	0.035596	-0.23415864	0.22048	0.270011479	0.1568
MRPL51	-0.213972533	0.035593	0.039215791	0.69519	-0.372439843	0.00027
BCKDHA	-0.874013509	0.0357	-0.132289154	0.74224	0.124790276	0.75617
HOXB9	-1.597640659	0.035731	-1.156348424	0.12543	-2.419306145	0.00169
CBX2	-0.394194552	0.035796	-0.00320213	0.98618	-0.242347991	0.19415
PSMG2	-0.176581637	0.035795	0.035586284	0.66323	-0.222835426	0.00771
DZIP1L	0.439778483	0.035811	-0.236813796	0.26077	0.911765084	1.15E-05
OLR1	0.742044742	0.035886	0.355536581	0.31535	0.084781547	0.81284
ECT2	0.334315768	0.035933	0.377331325	0.0178	0.058775635	0.71248
ALDH3A2	-0.365754283	0.035982	-0.250902562	0.14934	-0.224773715	0.19663
CTSV	-0.369192883	0.035993	0.040884966	0.8123	-0.294779621	0.09096
P11-570P14	-1.199764802	0.036007	-0.203806322	0.70395	-1.96386502	0.00114
P11-38O23	-0.934268326	0.036074	-0.232321705	0.57349	-0.168221067	0.68518
DDX26B	0.268971809	0.03623	0.033706967	0.79285	0.034738439	0.78753
FABP5P7	-0.471610773	0.036212	-0.220452726	0.32509	-0.338021987	0.13224
KCTD2	-0.299939271	0.036252	-0.203710056	0.15219	-0.184134651	0.19774
MGRN1	0.358391283	0.036244	0.118966665	0.48747	0.828684885	1.16E-06
PURA	-0.265345423	0.036251	-0.163190137	0.18995	-0.019977847	0.87219
RAP1GDS1	-0.230429242	0.036194	-0.177638698	0.10382	-0.167553844	0.12655
ARF6	-0.194048934	0.036326	0.024806824	0.78761	-0.154201756	0.09539
CNNM1	-0.601962969	0.036327	-0.004545729	0.98713	0.234319153	0.40309
DNTTIP1	0.244221885	0.036325	0.00870749	0.9401	0.032539195	0.78149
PYGL	-0.319649334	0.036294	-0.240086571	0.11373	-0.511751494	0.00082
TARSL2	-0.314017543	0.036296	-0.206940104	0.1651	-0.087356576	0.55832
P11-254B13	0.879636463	0.036356	0.971865986	0.01836	1.38570794	0.00067
ST20	-0.477119444	0.036361	-0.120093353	0.58563	-0.363887466	0.10114
MRPS31	-0.226678021	0.036385	-0.225613497	0.03463	-0.381836116	0.00047
ST8SIA3	-1.638451651	0.036414	-0.541145428	0.48792	-1.940932544	0.01565
MRPL22	-0.260763213	0.036428	-0.328152001	0.00783	-0.446983613	0.00034
ZBTB4	-0.270155369	0.036449	-0.165756976	0.19492	0.127786783	0.31613
ACBD5	-0.245569064	0.03663	-0.279811418	0.01701	-0.162277791	0.16524
BBS12	-0.459596724	0.036605	-0.086325482	0.68417	-0.208256121	0.33294
GNB1	0.238495009	0.036633	0.286910631	0.01188	-0.090774569	0.42657
C1QTNF6	0.291383155	0.03665	0.163096666	0.23857	0.156640261	0.26045
B9D1	0.342939544	0.036733	-0.161285676	0.33266	0.037935789	0.81962
BSDC1	0.235138181	0.036718	-0.119878459	0.28735	-0.003900218	0.97244
IQSEC3	0.857705136	0.036726	-0.479416323	0.24935	0.328017105	0.41316
ITM2A	1.439791426	0.036705	2.088037977	0.00237	0.077188056	0.91148
TTC7A	-0.380072646	0.036724	0.059597308	0.7384	0.339401965	0.05681
CCDC157	0.414195366	0.036775	0.220581795	0.25856	0.639459923	0.00103
HTR1B	-0.945986836	0.036805	-1.100277638	0.015	0.411949702	0.35698

GNAS	0.163926593	0.03689	0.209110398	0.00771	0.173184774	0.02741
ENTPD4	0.24431233	0.036956	0.092606292	0.42769	0.243899343	0.03692
FAM86EP	-0.589471067	0.036952	-0.521836144	0.0586	-0.188776895	0.48861
INTS12	-0.480556642	0.037061	-0.160592684	0.47611	-0.206951255	0.36166
AC004381.6	0.566586431	0.037114	0.915149028	0.00047	0.709960443	0.00811
DECR1	-0.204011126	0.037119	-0.005723871	0.95269	-0.23416881	0.0166
RAB21	0.224880068	0.037122	0.095741786	0.3737	0.002794568	0.97932
VHL	0.2096988	0.037178	0.249257023	0.01281	0.155487625	0.12181
TACO1	-0.287788171	0.037199	-0.398412653	0.00342	-0.333655142	0.0146
ETFA	-0.215751135	0.037265	-0.286469319	0.0055	-0.403313293	9.60E-05
GLYCTK	-0.425721693	0.037247	-0.571949135	0.005	-0.337254536	0.09707
IRGM	0.57079102	0.037267	0.294813753	0.27874	0.4033205	0.1416
KIAA1731	-0.226884928	0.037339	-0.038018891	0.7245	-0.012645553	0.90705
NOL11	-0.220070491	0.037362	-0.150416812	0.15285	-0.232695403	0.02772
RAC2	0.600810002	0.037314	-0.163380679	0.57513	0.133854824	0.6444
STK36	0.356398561	0.037331	0.130391569	0.44503	0.462667011	0.00671
TOE1	-0.438931039	0.037354	-0.740584791	0.00046	-0.365267242	0.08273
C19orf26	0.880707501	0.037394	0.350161101	0.41477	0.317585058	0.45863
FAM208A	-0.198244358	0.037452	-0.207396656	0.02884	-0.197186675	0.03823
FXYD6	0.489578032	0.037438	0.372209547	0.11347	0.009902065	0.96655
FBXL3	-0.187840738	0.037469	0.012294469	0.89048	-0.206975432	0.02134
CAPG	-0.250544945	0.037492	-0.291833077	0.01517	-0.260120511	0.03069
CLDN1	0.555014558	0.037521	0.314258108	0.23888	0.106824828	0.68907
CD70	-1.369876204	0.037542	-2.224909631	0.001	-2.929782484	5.41E-05
PRRC2B	0.256338957	0.037591	0.288448423	0.01912	0.419467946	0.00065
CCDC102A	0.535716002	0.037636	0.520533659	0.04035	0.313305926	0.22697
CCDC12	-0.23759969	0.03771	-0.402245584	0.00041	-0.245404128	0.03123
PCF11	-0.180724427	0.037723	-0.059618441	0.49003	-0.228602916	0.00847
FXN	0.34616442	0.037754	0.041943271	0.79988	0.048871974	0.7692
RHEB	0.15992578	0.037758	0.304872644	5.85E-05	-0.033281904	0.6664
DDX18P1	-0.838893013	0.037793	-0.412084714	0.27768	0.139951469	0.70533
EBF3	2.093786179	0.037826	3.566697245	0.00039	0.345071021	0.73409
GLUL	-0.289978679	0.037865	-0.114811146	0.41062	-0.328344866	0.01868
COG6	-0.346273319	0.037932	-0.40120206	0.01627	-0.398674058	0.01674
SLC35B4	-0.289438774	0.037925	0.108117527	0.43391	0.054652146	0.69331
ZNF207	0.157646374	0.037987	0.20476795	0.00691	-0.022116137	0.77092
ZNF669	-0.250592457	0.038018	-0.391866454	0.00103	0.042022764	0.72001
ZNF592	0.180998982	0.03804	0.039542399	0.64888	0.387920305	6.88E-06
HNRNPA2B	0.224098066	0.038077	0.207827441	0.05437	0.00026393	0.99805
FAM105A	-0.815701105	0.038111	-0.385101778	0.3256	-1.537458575	0.0001
GOLIM4	-0.354812257	0.038167	0.063311903	0.71068	-0.434805546	0.01107
MRPL4	0.283695059	0.038188	-0.107829686	0.43216	0.082283304	0.54914
NMUR2	0.706721556	0.038181	0.341463179	0.31758	0.399495419	0.24341
SLMAP	0.262420571	0.038188	0.360173662	0.00439	0.283870365	0.0248
IFI6	0.626372529	0.038238	0.607164665	0.04392	0.473588573	0.11726
NEK4	-0.209292111	0.038232	0.05985247	0.54679	0.020021478	0.84125
ADAMTS4	0.597162489	0.038328	0.561505254	0.04917	0.393243341	0.1706

HNRNPK	0.142099427	0.038325	0.172709197	0.01171	-0.035201877	0.60809
IDH3A	0.242768452	0.038294	0.355548017	0.00214	0.254487223	0.02928
MNS1	-0.478703284	0.038326	0.196967661	0.37273	0.104518739	0.64357
NOG	0.696079337	0.038291	0.894694034	0.00685	0.667859732	0.04605
ETV6	0.318611926	0.038357	0.204884461	0.1824	0.460247435	0.00267
SMAP2	-0.366691872	0.038387	-0.234028598	0.18531	0.006701208	0.96969
RPL26L1	-0.240999867	0.038407	0.026592368	0.81193	-0.580844724	8.97E-07
FASTKD3	-0.384412524	0.038491	-0.374165559	0.04244	-0.356499137	0.05481
VARS2	0.369010937	0.038497	0.34097942	0.05438	0.076419221	0.66911
MAFB	1.594579027	0.038589	1.954775978	0.011	-0.230181369	0.76905
SMIM5	-0.569588499	0.038587	-0.425819602	0.11579	-0.600803929	0.0276
TM4SF1	0.387114734	0.038598	0.225366639	0.22846	0.087300101	0.64093
TMEFF2	1.740815759	0.038573	2.839877027	0.00056	0.995257781	0.23611
ZC3H11A	0.139059036	0.038584	-0.105013581	0.11785	0.004153887	0.9507
ZNF367	-0.479124762	0.038568	-0.479143624	0.03755	-0.211719076	0.35718
ZGLP1	0.894367085	0.038641	1.197074842	0.00329	0.713772756	0.09407
BAHD1	-0.260112085	0.038658	-0.349900731	0.00503	0.178247204	0.1485
BCL7A	-0.294197327	0.038687	0.047918698	0.72481	-0.304256518	0.03038
P11-139H15	-0.418450353	0.038708	-0.116189419	0.54631	-0.187366566	0.33913
ATG7	0.345372554	0.038754	0.419154866	0.01186	0.548867261	0.00096
L17-C18orf	-0.333860112	0.038762	-0.10256768	0.52357	-0.438817333	0.00658
ZNF684	-0.5229873	0.038743	-0.101884416	0.67859	-0.251567447	0.31158
PBDC1	0.289537865	0.038876	-0.074750235	0.59438	-0.003215782	0.98175
SMIM7	-0.173163686	0.039055	-0.025326399	0.75997	-0.209691518	0.01196
UBR4	0.275316355	0.039089	0.184552635	0.16638	0.57839179	1.42E-05
MSANTD2	-0.295055946	0.039185	0.198114312	0.15423	0.017903691	0.8988
PRPF4B	-0.187299924	0.039173	-0.055125833	0.54253	-0.150037325	0.09825
PRRC2A	0.252947326	0.039191	0.279233812	0.02266	0.606830784	7.18E-07
KBTBD2	0.234136678	0.039239	0.153505345	0.17494	0.151063545	0.18351
RBBP5	-0.176162682	0.039218	-0.211026077	0.01195	-0.070685901	0.4001
RGMA	0.609899487	0.039239	0.876096656	0.00293	0.29736441	0.31522
AC000367.1	-0.60607375	0.039258	0.49125442	0.06712	0.296026954	0.27795
DCTN4	-0.180246716	0.039293	-0.109613665	0.20794	-0.114547193	0.18916
PXMP2	-0.381841522	0.039327	-0.061964674	0.732	-0.17186339	0.34487
ABCA9	0.776471127	0.039394	1.119510115	0.00271	0.766859072	0.04082
HLA-E	0.287108576	0.039402	-0.089735062	0.51996	-0.066591223	0.63338
PPFIA1	0.234751393	0.0394	0.130868805	0.24869	0.237241071	0.03715
WBP2	0.376062023	0.039419	0.163439549	0.37107	0.354683193	0.05188
MEN1	0.218999143	0.039469	0.061747164	0.55578	0.230999674	0.02813
RFXAP	-0.585881647	0.039577	-0.678099856	0.01558	-0.197336225	0.47094
RNPEP	0.246244394	0.039567	-0.020710328	0.86249	0.459884792	0.00011
CDKN3	-0.42657923	0.039655	0.079276952	0.69739	-0.412086847	0.04626
LPIN2	0.247135297	0.039682	-0.106155741	0.37809	-0.039774134	0.74106
FBXL15	-0.474528526	0.039698	-0.698723737	0.00259	-0.313620563	0.16723
LIN7C	-0.189231031	0.039739	-0.189961975	0.03831	-0.207719668	0.02378
SLC10A5	-0.683900251	0.039732	-0.202316493	0.51142	0.45864275	0.12296
MAPRE1	0.215927766	0.039778	0.101908678	0.33087	-0.015741912	0.88102

RPLP0P6	-1.091825807	0.039873	-1.056108271	0.0426	-0.988897619	0.05887
CCNJL	-0.394606859	0.039951	-0.566520957	0.00312	-0.421316389	0.02764
PRTG	-0.651193689	0.03997	0.251660503	0.42537	-0.30242152	0.33875
BOLA2B	-0.340425354	0.039996	-0.332824638	0.04251	-0.653040553	9.19E-05
CARD6	0.265566492	0.040025	-0.050480719	0.69705	0.309865101	0.01622
AP3S1	-0.19428375	0.04008	-0.075930671	0.41685	-0.237779171	0.01166
ZNF557	-0.213979706	0.040085	-0.161586281	0.11512	-0.186185901	0.06967
PCDH17	-0.515903857	0.040145	-0.082053981	0.74226	-0.629790876	0.01224
TWISTNB	0.316507114	0.040142	-0.046209001	0.76412	0.002090171	0.9892
RANBP1	-0.24477004	0.040214	0.05587923	0.63709	-0.446547889	0.00018
RAD52	-0.501687967	0.040312	-0.175825862	0.46536	-0.443188739	0.0682
SIK2	0.352424013	0.040338	0.308984591	0.07217	0.560249834	0.0011
SLC41A2	0.35357145	0.040338	0.449729406	0.0089	0.293442236	0.08829
TBC1D2	-0.438301928	0.040296	-0.398120996	0.05854	-0.253497296	0.22829
ZNF292	-0.284355438	0.040284	0.200672458	0.14662	0.103984813	0.45238
ILK	0.239879662	0.040391	-0.176969983	0.13079	-0.221284855	0.05961
SSBP4	0.373793437	0.040387	0.144038669	0.42925	0.196079614	0.28242
YME1L1	0.159649386	0.040391	0.065568703	0.39851	-0.093052252	0.23276
FGFR1	0.505988683	0.040407	0.977569768	7.24E-05	0.146588251	0.55304
HIST1H4A	-0.43781599	0.040439	-0.175481421	0.41087	-0.588150346	0.00591
AKT1S1	0.240882919	0.040515	-0.049887199	0.67061	0.059602767	0.61255
EBAG9	-0.298872608	0.040525	-0.143810222	0.31595	-0.060789203	0.67383
NIPAL2	-0.369479999	0.040641	0.199334643	0.26132	0.005549073	0.97521
C17orf107	0.747806337	0.04069	0.465070725	0.20253	0.224417808	0.54769
DPY30	-0.227672103	0.040689	-0.298753997	0.00674	-0.207514268	0.05924
ACADM	-0.188262004	0.040794	-0.170777187	0.06112	-0.105369111	0.25063
MLH3	-0.205502207	0.040802	-0.153662856	0.12036	0.228785815	0.02085
TMEM200A	-2.240807355	0.040784	0.442002341	0.63599	-0.749062547	0.44101
TMEM11	0.264270881	0.04084	-0.169975208	0.19041	-0.100289426	0.44078
CDCA2	-0.487430823	0.040917	-0.341392258	0.14989	-0.295182644	0.21392
GTPBP1	0.310958945	0.040928	0.135151894	0.37356	0.307904163	0.0425
MTERFD2	-0.323451772	0.040976	-0.009903173	0.94952	-0.296469127	0.0602
SERINC2	0.280205913	0.041002	0.028305955	0.83638	0.402663966	0.00323
DCTPP1	0.350321256	0.041124	-0.003192263	0.98518	0.229428125	0.18107
STC1	0.863758175	0.041109	0.062846511	0.88227	-0.113037165	0.79065
AKTIP	-0.300119336	0.041153	-0.107973036	0.45571	-0.303698684	0.03688
UBAP1	0.245016262	0.041166	-0.039474023	0.74222	-0.034102198	0.77688
UBR3	-0.273934614	0.041274	0.09671183	0.46898	-0.001126281	0.99329
ANO8	0.393513556	0.041333	0.415381501	0.02911	0.331669492	0.08447
SMAD4	0.192765732	0.04141	0.244896466	0.00917	0.313021939	0.00088
CDS1	-0.303215322	0.041435	-0.377966876	0.01093	-0.070087021	0.63669
INSIG1	0.331187218	0.041531	0.002327006	0.98857	-0.015264065	0.92519
GREM1	0.808238017	0.041738	0.703095064	0.07637	0.214579424	0.58961
MED14	0.363652466	0.041896	0.459244258	0.01006	0.650797479	0.00026
RARS2	-0.192979125	0.041889	-0.094484972	0.31408	-0.260181672	0.00607
MCMBP	-0.166621996	0.041979	-0.075728277	0.35217	-0.128566457	0.11587
MFAP4	0.431857507	0.041955	0.29288566	0.16789	0.071600684	0.73617

ONECUT1	-0.885037	0.041985	-1.025967486	0.01833	-0.040224456	0.92588
ZNF665	-0.39226118	0.041939	-0.088144868	0.63766	0.211612894	0.25325
ARHGAP33	0.882509289	0.042042	0.658853578	0.12946	0.836427584	0.05374
SLC25A16	0.447359065	0.042055	0.220089044	0.31639	0.613784032	0.0052
DPP9	0.384623763	0.042175	0.273086405	0.14889	0.522372269	0.00559
MEGF10	1.521056874	0.042184	2.493115335	0.0008	0.498075767	0.51037
TMEM261	-0.21083775	0.042175	-0.086714767	0.39651	-0.319912321	0.002
P11-641D5	-0.261893124	0.042219	-0.498920761	0.00011	-0.454871226	0.00042
OSGEPL1	-0.337869784	0.042238	-0.340101387	0.03775	-0.407384919	0.0137
PIK3CD	1.175130176	0.042405	0.830926244	0.15477	0.266019704	0.64689
SCAF8	-0.120891776	0.04239	-0.095195095	0.10718	-0.087479382	0.14051
MPC1	-0.237269688	0.042478	0.260781448	0.01923	-0.209782595	0.06956
SIRT2	0.244254211	0.042487	-0.01336927	0.91174	0.040966975	0.73397
LEPREL1	-0.555707296	0.042518	-0.151562662	0.57889	-0.341665821	0.21166
ELP4	-0.215563137	0.042652	-0.273389211	0.00839	-0.33233548	0.00164
MBD4	-0.246975631	0.042649	-0.162575897	0.17986	-0.238520846	0.04995
PAX5	1.198355141	0.042687	1.642216888	0.00369	1.967034765	0.00058
SGSM1	-1.387809783	0.042705	0.67377285	0.30961	0.202664913	0.76072
COQ3	-0.471139248	0.042731	-0.217126085	0.33055	-0.393257392	0.08453
PDE7B	1.419826242	0.042758	1.411141883	0.04437	-0.065167372	0.92901
MRE11A	-0.223628349	0.042811	-0.184983668	0.09019	-0.326406168	0.00293
CCRL2	0.890933973	0.042858	-0.14990592	0.73974	-0.234743419	0.60342
CNOT7	-0.241184854	0.042842	0.036875385	0.75613	-0.389653376	0.00106
P11-459D22	-0.96901497	0.042937	-0.70608786	0.11395	0.285576588	0.49115
TB-147C22	0.893440097	0.043058	-0.290539561	0.53142	0.810038555	0.06609
DDX11	-0.282996588	0.043018	-0.091068149	0.51245	0.001649513	0.99055
ELAC2	-0.23711401	0.043043	-0.162356745	0.16465	-0.164458048	0.15967
ENPP4	-0.394272625	0.043036	-0.596319226	0.00219	-0.39204468	0.04382
POM121	0.767703027	0.043029	0.959754671	0.01131	1.645929515	1.35E-05
PMPCB	-0.196047466	0.043132	0.018882161	0.84381	-0.278033186	0.00408
SUPT7L	0.193745401	0.043124	0.027605104	0.77192	-0.012041369	0.89991
CYB5R3	0.306890546	0.043157	-0.32959001	0.03041	0.091472219	0.54683
FAM86DP	-0.375926512	0.043209	-0.268958091	0.14336	-0.145668825	0.42779
SLC39A7	0.44367071	0.04321	0.114365175	0.60222	0.172422463	0.43242
SNRPA1	0.234551656	0.043203	0.057323441	0.61971	0.126161677	0.27693
ZDHHC3	0.210517867	0.043239	0.100944114	0.32946	0.158770945	0.12645
NSUN5P2	-0.820864399	0.043268	-1.380279445	0.0007	-0.803278963	0.04703
TMBIM6	0.185514679	0.043318	0.060956273	0.5067	0.101763812	0.26767
NPM1P25	0.621513241	0.043408	0.542550189	0.07459	0.70755962	0.01999
HHEX	-0.878911005	0.043523	-1.421173834	0.00113	-0.947073911	0.02949
CBR3	0.934734825	0.04354	0.876595355	0.05537	0.852794004	0.06479
RALGAPA1I	-0.754277294	0.043596	0.019464559	0.95684	-0.023604012	0.94795
AC098826.5	0.901385671	0.043618	0.078711448	0.86244	0.806122387	0.07092
CCDC84	-0.417409755	0.043665	0.204265192	0.29548	0.05453373	0.78309
NIT1	0.223564649	0.043657	-0.213046827	0.05554	0.131327919	0.23363
ADAT2	-0.384176059	0.043736	-0.189135292	0.30995	0.17031956	0.35806
SPRED1	0.28126576	0.043786	0.154242041	0.26813	-0.121466838	0.3843

UBALD2	0.576189606	0.043822	0.765223572	0.0072	0.292995885	0.30646
CCDC142	-0.492221648	0.043839	-0.131771314	0.58378	0.052609403	0.82705
GPRC5A	0.514660863	0.043877	-0.508511779	0.04659	0.070283441	0.7832
ADRA2A	-0.73391469	0.044119	-0.667132602	0.06552	0.206919071	0.56408
C16orf62	-0.211240258	0.044112	-0.103510219	0.32188	-0.066164401	0.52717
IGLON5	0.512489263	0.044104	0.646016575	0.01042	0.262614513	0.30389
CHD6	-0.278995975	0.044179	-0.031371922	0.82091	-0.302009321	0.02927
PNPO	-0.406722146	0.044163	-0.425004525	0.03333	-0.014723852	0.94087
APBA3	0.363950534	0.044254	0.043798558	0.80733	0.309582813	0.0838
FGA	-1.394838157	0.044253	-1.640539043	0.0179	-1.288232396	0.06277
BTF3L4P2	0.317938631	0.044317	-0.088884705	0.57602	-0.196527929	0.22212
OGFOD2	-0.278468543	0.04431	-0.381070691	0.00527	-0.106769806	0.4329
LIG1	-0.279246088	0.044335	-0.124733952	0.36704	-0.185614499	0.18184
CSTF2T	-0.192978439	0.044389	-0.288949821	0.00248	-0.328209511	0.00063
C3orf52	0.239086463	0.044454	0.199802117	0.09056	0.087935049	0.45989
TD-2287O16	-0.357570047	0.044453	-0.514799651	0.00378	-0.701495604	8.26E-05
FAHD2A	0.338350076	0.044443	0.021412916	0.89915	0.18933191	0.26197
ARHGEF34P	0.329966829	0.044584	0.051150742	0.75554	0.331160305	0.04361
EIF5B	0.468659646	0.044634	0.266241944	0.25362	0.567604084	0.015
PCDP1	-0.595293705	0.04465	0.249542114	0.37917	0.032244647	0.90977
RANGRF	-0.305738732	0.044733	-0.195743366	0.18606	-0.321451738	0.03323
S100A6	-0.31324682	0.044725	-0.333689129	0.03245	-0.629289087	5.55E-05
SIKE1	-0.193936395	0.044726	-0.158381113	0.09872	-0.208065449	0.03112
UBE2L6	0.445875009	0.044724	0.373890143	0.09163	0.014204515	0.94935
TMEM5	-0.249486433	0.044767	-0.268872358	0.02771	-0.162502307	0.18597
ZNF544	-0.2160008	0.044753	-0.241308194	0.02385	-0.023954386	0.82189
DAZAP2	0.243425305	0.044823	-0.192317949	0.11292	0.030548987	0.8012
HDAC11	-0.402536106	0.044809	-0.319827308	0.10779	-0.713822405	0.0004
HECTD3	-0.288510657	0.044901	-0.153222297	0.28185	-0.008675995	0.95159
NAT6	0.477545696	0.044894	0.328342714	0.16507	0.358508641	0.13128
PIN4	-0.336642065	0.044932	0.062547138	0.70399	-0.692889006	4.21E-05
DACT3	0.419826387	0.044984	0.605963887	0.0036	0.155531409	0.45832
RTN2	0.51539915	0.04497	0.50061077	0.04975	0.201492421	0.4372
ZNF77	-0.3130084	0.04496	-0.371877227	0.0144	0.020696788	0.8892
TIMP2	0.36546508	0.045016	0.245009352	0.17853	-0.005599279	0.97555
DDX47	0.176150562	0.04506	-0.032893237	0.70794	-0.089396488	0.31092
LACTB2	-0.365515445	0.045121	-0.158896522	0.3773	-0.446512975	0.01444
BRINP3	0.969911751	0.045149	0.539824967	0.26521	0.763547804	0.11475
AC242988.1	-0.372278342	0.045205	-0.338988053	0.06672	-0.259239043	0.16133
EIF2AK1	0.186190657	0.04522	-0.001126339	0.9903	0.361695713	9.61E-05
KLC4	-0.330238133	0.045176	-0.224107808	0.17319	-0.07513724	0.6474
ZHX3	-0.30066694	0.04522	0.086428421	0.55849	-0.011555066	0.93774
FAM153C	-0.959394391	0.0453	-0.351086572	0.44423	0.565723129	0.2076
FAM161A	-0.426963782	0.045291	0.122029889	0.55553	0.05240668	0.80296
GINS1	-0.315662096	0.045344	0.066821037	0.66872	-0.105509502	0.50226
UBE2K	0.232597412	0.045342	0.039070173	0.73603	0.159706293	0.16859
C14orf28	-0.659326917	0.045415	-0.397203154	0.21665	-0.485667771	0.13732

FAM115A	-0.146850838	0.045432	-0.020857858	0.77481	-0.207777084	0.0046
NCLP1	-0.648477891	0.045455	0.16732205	0.5743	0.917921868	0.00153
CTNNA1	0.158649951	0.045537	0.409440431	2.37E-07	0.195896212	0.01354
GSTM2	0.398376949	0.045527	0.228687879	0.24945	0.465958671	0.01872
PDK1	0.315451638	0.045515	-0.032603072	0.83612	0.311824758	0.04774
TRIM2	-0.204637092	0.045516	-0.241799649	0.01791	-0.327048707	0.00139
MANBAL	-0.198601352	0.045564	-0.215322749	0.02607	-0.203252459	0.03793
ASB14	-0.670569003	0.045612	-0.266241441	0.39592	0.016013483	0.95922
APOD	1.901490945	0.045656	2.834920161	0.00271	0.923951652	0.33603
IER3IP1	-0.235963697	0.045648	-0.420181012	0.00035	-0.513243212	1.38E-05
IFT80	-0.31492479	0.045662	0.267000947	0.08756	-0.190017553	0.22705
RPAP1	-0.39264606	0.045865	-0.242839349	0.21471	0.372718428	0.05588
ABCC6P1	-0.866180714	0.045933	-1.114816006	0.01022	-0.466506338	0.28009
AC243756.2	0.547123671	0.046122	-0.055725148	0.84219	0.016418481	0.9535
BCL2L12	0.381064111	0.046122	-0.187662439	0.3286	-0.245126712	0.20904
MORC2	-0.264193387	0.046105	0.163412728	0.21263	-0.091000046	0.49004
NUDT4P1	0.547123671	0.046122	-0.055725148	0.84219	0.016418481	0.9535
TFAP2A	-1.333743143	0.04611	0.961215753	0.12211	-1.192364762	0.08488
PFN2	-0.162694688	0.046149	-0.034621033	0.6695	-0.383343823	2.70E-06
APIP	-0.217836882	0.04638	-0.190223138	0.07459	-0.264564553	0.01417
C5orf56	0.65532606	0.046348	0.433635262	0.18119	0.644582213	0.04597
RNF24	0.350301	0.046395	0.128496278	0.46482	0.188077683	0.28473
SIRT7	-0.321169388	0.046357	-0.435402243	0.00646	-0.179665772	0.25896
SNX10	-1.542405401	0.046386	-2.223451086	0.00415	-1.5896245	0.03211
SRSF12	0.467619341	0.046355	1.121271315	1.21E-06	0.24111377	0.30703
STAT5B	0.247428401	0.046316	0.172053086	0.16552	0.39580737	0.00142
TGFBR2	-0.238671385	0.046275	-0.586829878	9.72E-07	-0.164536044	0.16892
ZCRB1	-0.225288777	0.04636	0.152863636	0.16782	-0.311617198	0.00583
ZNF354C	-0.386748077	0.046271	-0.240163133	0.21328	-0.463221257	0.01691
UBA52	0.201897115	0.046458	-0.206932161	0.04142	-0.114595789	0.25885
CKMT2	-0.963824042	0.046592	0.029621198	0.94701	0.217749162	0.62224
ENSA	0.211965569	0.046615	0.152977108	0.1503	-0.072533882	0.49618
UBE2V1	0.19398115	0.046813	0.079026667	0.41699	0.13735128	0.15975
CDAN1	-0.402234166	0.046877	-0.209759422	0.29608	0.038239417	0.84921
LTB4R	0.406998117	0.046867	0.198457811	0.32292	0.72513859	0.0003
EPM2A	0.930405587	0.046965	0.149655556	0.75143	0.346905497	0.46656
ERF	0.318452746	0.046994	-0.14601636	0.36325	0.003549694	0.9824
RNF138	-0.252298947	0.046974	-0.165001719	0.19066	-0.08542077	0.49896
SPACA4	-0.837313559	0.046982	-0.554776438	0.15859	0.299903269	0.41795
HDAC1	-0.149657278	0.047061	-0.187731891	0.01246	-0.172710856	0.02177
TMEM14A	-0.262942131	0.047025	-0.177792606	0.16802	-0.38554589	0.00356
TMX1	-0.189231731	0.047068	-0.348182937	0.00024	-0.413863806	1.45E-05
TNK1	-0.648963203	0.047068	-0.214754306	0.50822	-0.284729688	0.37744
POGZ	0.244569335	0.047184	0.156248713	0.20416	0.582355239	2.11E-06
KIF7	0.517847713	0.047263	0.778141378	0.00266	0.318948121	0.22418
PEX11A	-0.39344212	0.047264	-0.578885215	0.00311	-0.403720653	0.03727
THNSL1	-0.322583024	0.047264	-0.17873405	0.25479	-0.036449254	0.81639

ERCC4	-0.344564922	0.047368	-0.096341453	0.57524	0.009242287	0.95727
ZNF844	-0.385782652	0.04737	0.059901975	0.75232	0.200401754	0.29008
C11orf52	-0.444702754	0.047465	-0.399689723	0.07201	-0.472808465	0.03439
METTL23	-0.270783538	0.047497	-0.166116922	0.21256	-0.01155487	0.93109
NKX6-3	-2.717998006	0.04751	-1.370760485	0.30871	-2.926769194	0.03296
RNF125	-0.682984422	0.04748	-0.25883648	0.44767	-0.22932491	0.50092
EEF1A1P3	0.765076857	0.047562	0.800998176	0.03389	1.107918678	0.00307
APOPT1	-0.231328635	0.047664	-0.2904917	0.01131	-0.229275617	0.04625
CEP57	-0.289193945	0.047709	0.269225395	0.06392	-0.259028583	0.0756
DUOX1	-0.700627856	0.047703	-0.532409713	0.12633	0.552210498	0.10615
DYNLRB1	0.239937637	0.047676	0.185240707	0.12514	-0.090688137	0.45563
RNF8	-0.224991322	0.047652	-0.161635818	0.14594	-0.373633633	0.00094
SKIL	0.348444666	0.047722	0.159715398	0.3641	0.268491714	0.12711
EM56-RWD	0.681491503	0.047726	0.276714829	0.42443	0.576433786	0.09358
ARL8B	0.557576339	0.047768	1.138585308	4.49E-05	0.53133153	0.05899
NOL6	0.256690103	0.047792	0.013839509	0.915	0.256314355	0.04804
TMEM106B	-0.195585138	0.047795	-0.052291661	0.59503	-0.229856795	0.01982
ZNF767	-0.422302659	0.047824	-0.336663339	0.1103	0.048526993	0.81818
PSMD5	-0.223635743	0.047862	-0.018485665	0.86718	0.021114131	0.84985
TRMT11	0.265967985	0.047949	0.205338862	0.12447	0.049652958	0.71292
C11orf83	-0.311908947	0.048017	-0.506127464	0.00125	-0.423681585	0.00714
VDAC3	-0.156242707	0.048001	-0.148639272	0.05798	-0.21939115	0.00543
ZNF671	-0.412204464	0.048004	-0.191320298	0.34629	0.047969167	0.81363
AKR1B10	-1.414445938	0.048103	1.339439027	0.05788	0.752490842	0.28735
HOOK3	-0.308540182	0.048113	-0.240293017	0.12303	-0.256752222	0.09933
ZBTB33	-0.181437207	0.048128	0.012677261	0.88892	-0.307300915	0.00081
PCBP1	0.238665187	0.048194	0.082899451	0.49181	0.419341915	0.00049
COG3	-0.219756997	0.048273	-0.162279758	0.14306	-0.177667512	0.10993
KBTBD4	-0.292045573	0.048229	-0.41797841	0.00455	-0.389011892	0.00819
RPUSD4	0.187124378	0.048259	0.029586963	0.75453	0.349308394	0.00021
TMEM185B	0.236805625	0.048271	0.168916771	0.15452	0.121141847	0.31196
ARFGAP1	0.291678547	0.048386	-0.143421364	0.3325	-0.014187023	0.9236
DOCK8	-0.519700511	0.04838	-0.470254596	0.06773	0.399132314	0.11154
SDK1	-0.704961516	0.048321	-0.254862964	0.46966	-0.464250593	0.19182
SURF2	-0.348477416	0.048373	-0.281824929	0.09459	-0.456748791	0.00951
TOP2A	-0.358185816	0.048366	0.047038251	0.79524	-0.325900247	0.07239
AC005517.3	-0.685010656	0.048675	-0.685103796	0.04244	-0.339431246	0.30678
NARG2	-0.214918665	0.04868	0.136378886	0.20492	0.123150277	0.25405
ABCD1	0.415207834	0.048721	0.047336278	0.82155	0.219036016	0.2953
ARHGAP32	0.251922004	0.048757	0.05768662	0.65124	0.383458193	0.00263
COG2	-0.257078499	0.048732	-0.315278987	0.0153	-0.195452534	0.13262
FBXO33	-0.193536153	0.048743	-0.27430549	0.00454	-0.351149856	0.00035
FAM199X	-0.211732901	0.048802	-0.308235773	0.00401	-0.177392887	0.09775
SAMD4B	0.325692707	0.048828	0.0346123	0.83276	0.614115216	0.00013
LATS2	0.36200741	0.048977	-0.262355959	0.15499	-0.104313891	0.57175
OPA3	0.223702973	0.048978	0.07954375	0.48272	0.20839968	0.06556
SCOC	-0.264307183	0.04903	-0.452738119	0.00075	-0.203664409	0.12852

ATP9A	0.193275836	0.049179	0.14676658	0.13454	0.504541925	2.57E-07
PLA2G7	1.433137177	0.049224	3.326265916	2.53E-06	2.896368489	4.53E-05
PBK	-0.509513453	0.049286	-0.057238044	0.82396	-0.603126807	0.01986
ACSM2B	-0.549311409	0.049371	0.081652198	0.76292	0.202604253	0.45369
AFAP1L1	-0.78230063	0.049371	-0.370800875	0.33971	-1.113288653	0.00552
MYO5C	-0.328263958	0.049354	0.176880665	0.28643	0.218579617	0.18813
COA7	-0.273640267	0.049492	-0.382924343	0.00579	-0.239347323	0.08462
KRT13	-2.284348613	0.049565	0.744850625	0.51829	-2.135494552	0.06648
DOK1	-0.338727861	0.049599	-0.380210751	0.02332	-0.160658057	0.33854
ACVR1B	-0.29989494	0.049634	-0.259064439	0.08849	0.002830738	0.98514
ZNF799	-0.34774588	0.049685	-0.582745941	0.00099	-0.303549586	0.0856
ZNF136	-0.355037697	0.049764	-0.482652757	0.00756	-0.26667007	0.14055
ZNF28	-0.281049155	0.049749	-0.245713238	0.08591	0.003986674	0.97773
BICC1	0.544903499	0.049889	0.701684978	0.01142	0.398347454	0.15167
RAB5C	0.185194307	0.04992	0.145904288	0.12126	0.047098677	0.61871
ZNF501	-0.679417092	0.049919	0.115745818	0.72561	-0.153126868	0.6459
AC003989.4	2.013637948	0.020591	1.254172357	0.16226	2.487856982	0.00318
AC005037.6	3.605849814	0.007114	2.092025796	0.13305	3.194725609	0.01782
AC005488.1	-2.539495518	0.042295	-0.564325848	0.54672	-0.40967099	0.66195
AC006548.1	-3.832014766	0.010888	-2.161306634	0.09397	-0.490461053	0.67625
AC007272.3	3.939941069	0.001338	4.139099705	0.00057	3.641698171	0.00329
AC010733.5	2.656517205	8.87E-06	1.888686815	0.00194	1.95029591	0.00144
AC011737.2	-4.614755928	0.020302	1.688744813	0.32495	0.680341266	0.6935
AC011933.2	2.146057047	0.048015	2.369505771	0.02593	2.162128709	0.04496
AC092338.5	-2.15830943	0.008262	-0.093728067	0.87058	0.500187914	0.36867
AC092798.2	1.633288943	0.035093	1.169909198	0.1351	0.80227169	0.32338
AC106827.1	-2.443551536	0.032097	-2.34669258	0.02761	0.483620307	0.56175
AC112229.1	-1.789686905	0.002352	-1.939313774	0.00086	-1.273800143	0.02369
AC133644.3	2.479327836	0.046963	0.569795502	0.67132	0.913235873	0.49109
AC139452.2	-2.524945525	0.042164	-0.521825783	0.57139	-0.351110511	0.70289
ACTBP2	1.519094627	0.045234	0.329729902	0.68839	0.364635573	0.66196
ADAMTS7P3	3.181054061	0.010011	0.866535694	0.48675	2.60326027	0.03213
ADAT3	1.283811605	0.015241	0.571904836	0.28583	0.601439079	0.26413
AGXT	1.289003065	0.028681	1.115423605	0.05897	1.109970084	0.05867
AIRE	1.453183246	0.018961	0.617880009	0.32052	1.386115927	0.02428
ALG1L	1.495697295	0.046029	0.626675836	0.41752	0.628270975	0.42094
ALG1L2	-1.047889477	0.044616	-0.813648079	0.0988	-0.662546833	0.17119
AOAH	-1.552664784	0.029406	-2.038956891	0.0047	-2.30460187	0.00169
AP000619.5	3.821942669	0.029023	0.733650483	0.69985	0.179079934	0.92726
AP001258.5	3.00974155	0.004826	2.155727416	0.04837	2.384591674	0.02846
AP004289.2	-1.90827877	0.046301	-0.30374721	0.67989	0.858696142	0.21171
APOBEC2	0.945208878	0.034667	0.851636168	0.05273	0.772507045	0.08527
ATP6V1G1P	-3.576184027	0.014709	-1.856934986	0.16717	-0.832102464	0.49591
B3GAT2	2.555854065	0.04381	1.466443842	0.25422	2.303348261	0.06975
BCL2A1	4.061528171	0.00031	3.728048541	0.00094	2.4934211	0.03192
BLID	3.649364338	0.010839	3.968834172	0.00452	2.56960368	0.09109
C11orf87	4.339792573	0.012821	4.529482684	0.00926	1.55368503	0.38984

C12orf74	1.748719637	0.003867	0.600135879	0.34496	1.213154043	0.05097
C14orf178	-1.260873201	0.034264	-0.019418368	0.96893	0.409045242	0.40165
C15orf43	1.534830644	0.032596	1.030066698	0.15899	1.518156046	0.03444
C17orf99	1.202992646	0.032055	0.094190557	0.86755	1.086111249	0.05222
C19orf73	-1.528605151	0.021514	0.36921209	0.46822	-0.292069695	0.59271
C1DP1	-2.843480913	0.038389	-6.618553254	5.15E-05	-0.400639873	0.75688
C2orf74	-4.500961707	0.011979	-2.47147661	0.1191	-0.796772663	0.59845
C5orf49	-1.575724329	0.020066	-0.769947424	0.23492	-0.660466235	0.31299
CACNG7	1.38717585	0.025194	0.363552562	0.5638	0.862670555	0.1673
CADM3	-3.234743221	0.038225	0.273824391	0.82659	-1.088179327	0.40915
CAMK2N2	-2.944062281	0.023134	-0.581728781	0.54235	-1.337573668	0.21221
CCDC135	-1.809618455	0.010414	-0.825406254	0.20916	-0.886652456	0.16702
CCDC155	1.919234498	0.01935	2.455750673	0.00181	2.333682923	0.00348
CCDC96	-1.134543111	0.019964	-1.223128022	0.01	-0.664149487	0.14371
CCL11	1.336618227	0.042417	1.44208038	0.02637	0.925527504	0.16522
CD274	3.928974123	4.48E-07	3.839942167	7.40E-07	2.957110545	0.00024
CD33	1.913151829	0.004499	1.653031974	0.01484	2.29324917	0.0004
CPN2	1.222879554	0.015559	0.835826574	0.10038	1.535496603	0.00184
CPNE5	1.406260531	0.008895	1.010109399	0.06093	1.021165549	0.05862
CRH	3.250780598	0.01154	1.739772929	0.21324	2.498379208	0.06085
CRYBA1	-2.410088037	0.035843	-0.580609516	0.43845	-0.198796482	0.78515
CSNK1A1P	-1.812751369	0.047172	-0.034130269	0.95932	0.823073342	0.17817
CTAGE3P	-1.49066348	0.011275	-0.980572761	0.07042	-0.144082035	0.78047
CTBP2P2	4.431846306	0.013593	2.861912998	0.1241	3.475535472	0.05771
TC-260E6.1	-1.991362913	0.018802	0.120586072	0.84484	0.412587768	0.49905
CTC-459F4.	3.044718167	0.01664	1.5781165	0.24158	2.770280706	0.03017
TD-2349B8	-4.346836483	0.000251	-0.145994144	0.83139	0.608359032	0.3717
TD-2372A4	-2.919283867	0.049903	-3.211485503	0.031	-0.9632571	0.47734
CYP4A22	-2.958407842	0.007259	-0.953087187	0.17735	-0.384871906	0.56493
CYP7A1	3.453665236	6.38E-05	2.938900303	0.00071	1.52041614	0.09857
DDX10P1	-2.718228573	0.02361	0.464670419	0.53287	1.164559954	0.10207
DOC2B	3.939288474	0.001179	3.138568066	0.01086	0.77704713	0.5916
DUTP1	-2.852290336	0.006566	-0.498514006	0.4903	-0.39338091	0.58759
EIF4BP6	-2.757411499	0.041372	0.642070336	0.50312	-0.03781284	0.96957
ETV7	2.26515219	0.025842	1.200688663	0.24687	1.631880521	0.11275
FBLL1	2.604735537	0.021657	2.670430382	0.01712	1.152926862	0.34741
FCF1P8	1.624095211	0.010715	1.738963677	0.00553	1.568465044	0.01342
FDPSP3	2.190800175	0.017571	1.271633852	0.18348	1.411565601	0.14028
FGF19	2.825055022	0.049008	3.55553432	0.01122	1.773570142	0.23436
FTH1P16	-2.223938957	0.015607	-0.332241041	0.61617	-0.695301819	0.31999
GABRB3	1.934221952	0.038385	1.540625085	0.10571	1.680197222	0.07067
GAPDHP21	-1.593455087	0.032958	-0.477072712	0.44912	-0.729894254	0.26517
GAPDHP40	-2.779925868	0.032288	-1.096208329	0.29125	-2.915850258	0.02474
GAPDHP49	4.270600648	5.48E-07	4.339943083	3.12E-07	3.983268201	3.17E-06
GAPDHP61	-1.706609445	0.032834	-0.260727135	0.67958	0.813264671	0.1692
GBX2	4.577063989	0.020283	3.706652683	0.06043	2.238500141	0.27993
GJB7	-1.158376816	0.048445	-0.713886545	0.20053	-0.387094958	0.47879

GLDN	-1.640788874	0.030499	0.536450372	0.38643	0.650499178	0.29814
GUCA1A	1.006449066	0.034116	0.061705628	0.90098	-0.67281636	0.21398
H3F3C	-3.399232913	0.023148	0.347620512	0.76676	-0.27138068	0.82438
HGFAC	2.554148837	0.016183	1.085641277	0.32514	1.391582856	0.20531
HIST1H1T	-4.092160719	0.001632	-1.121806696	0.24753	-1.749702821	0.10306
HLA-J	1.870107801	0.007665	0.733420672	0.31361	-0.305597901	0.70857
NRNPA1P1	3.217559066	0.007437	2.807014461	0.02006	3.112455737	0.00955
NRNPA1P2	-1.763602507	0.043678	-0.088509331	0.90116	-0.49309545	0.50601
NRNPA1P3	2.156529961	0.022244	1.864529485	0.04816	-0.38781412	0.72076
HSD17B14	1.239655511	0.005368	0.4001506	0.38238	0.207210824	0.6637
HSD17B6	-1.970604386	0.025594	0.136630185	0.86215	-2.85402398	0.00373
HSFX1	1.414727562	0.015629	1.33035758	0.01973	1.645807775	0.00368
HSPD1P2	5.910700673	0.000227	3.278773133	0.04674	2.313206883	0.17675
HSPD1P3	5.323482882	0.00047	3.291301974	0.03842	3.710878524	0.01839
HSPE1P10	-4.148997062	0.000586	-0.637852226	0.38499	-1.376585633	0.08625
HSPE1P13	-2.016964539	0.007243	-1.184064306	0.06877	0.46889042	0.42555
HTR3B	-0.936923431	0.044786	-0.081705632	0.83839	0.131270736	0.74178
ICAM5	2.589929449	0.00069	1.563915108	0.04295	1.344583345	0.08573
IGHEP2	3.35871414	2.77E-05	1.564252207	0.06163	2.779773481	0.00059
IL10RA	2.524276358	0.000334	1.227615681	0.08747	1.152512569	0.11043
IL20	6.374034775	4.36E-05	3.329304641	0.04225	2.98803351	0.07229
IL36G	2.813134258	0.031608	3.270112733	0.01056	3.060625191	0.0205
IMPDH1P10	3.586381567	4.19E-05	1.306623385	0.14888	2.038945984	0.02229
INSRR	3.064862508	0.034316	3.182703743	0.02699	1.966408279	0.18519
IRX3	-3.225373867	0.002879	-0.290465838	0.74558	-1.931552261	0.04786
JSRP1	2.897335722	0.026898	2.080357144	0.11798	0.994371049	0.48825
KARSP1	-2.976904285	0.037934	-0.640672848	0.59756	1.243601556	0.2473
KCNE1L	1.648500219	0.031167	1.548073085	0.04027	1.410191169	0.06728
KDM5D	-2.032856547	0.037614	-0.163719918	0.82118	-0.977707581	0.19454
KIF25	2.530749714	0.035995	1.089382283	0.40423	2.784427699	0.02181
KRT18P19	-3.494803936	0.015226	-1.673793515	0.19709	-1.184983023	0.34142
KRT18P34	1.507084367	0.017813	0.515429492	0.43865	0.686870406	0.30148
KRT8P9	3.548075476	0.000423	2.954792007	0.00362	2.24958452	0.03259
LGALS9B	1.668138528	0.02029	0.581958818	0.42397	0.821067201	0.2612
LRRIQ3	-1.939422996	0.014733	-2.326273654	0.00204	-1.275606591	0.07524
MADCAM1	2.068298794	0.037526	2.094311441	0.03515	1.332219889	0.1913
F2BNB-MEI	-1.977040528	0.04424	-0.484901928	0.583	-0.016601322	0.9849
MIA-RAB4B	5.696158624	0.000804	4.552864802	0.00798	4.491898772	0.00909
MKRN3	-2.055392856	0.034322	0.309350148	0.67101	0.348231318	0.62664
MOG	1.887253261	0.014449	1.24096501	0.09769	2.354704229	0.00158
MRPL35P3	-4.512279973	0.005697	-1.525600111	0.27053	-0.941127474	0.48797
MSC	2.666362269	0.04077	0.857421709	0.52738	-0.713345569	0.6266
MSMP	3.671961121	0.039306	-0.974243003	0.63036	0.131185732	0.94533
MTND4P14	1.701990866	0.035122	2.431352927	0.00173	2.786980113	0.0003
MTND5P14	2.239490869	0.003451	1.703682257	0.02778	2.117107868	0.00568
MTND5P25	3.759142871	0.000441	3.506836869	0.00104	4.548846433	1.62E-05
NANOGP2	5.348988834	9.47E-06	4.146701661	0.00073	3.549820928	0.00466

NOTO	-1.098911464	0.023446	-0.225677553	0.58876	0.326052282	0.41872
NPHS1	2.680468365	5.19E-05	1.881039084	0.00417	1.799079791	0.00683
NPIPA5	4.893695199	0.037035	0.04387728	0.98619	4.798285257	0.04084
NPM1P21	-2.722714143	0.042264	-0.971450532	0.36936	-1.071905108	0.33805
NPR1	1.36038779	0.033085	0.747828985	0.2451	-0.32482033	0.63243
NT5C3AP1	-1.755526133	0.039851	0.665350752	0.27896	-1.743356878	0.0411
OR13H1	1.654709967	0.005085	1.489660444	0.01101	2.082480972	0.00028
OR2I1P	2.088552085	0.014479	1.849522355	0.03004	1.482865984	0.08978
OR52N1	3.775244192	0.004626	3.389050663	0.0114	4.006720119	0.00224
OR52N3P	4.628029175	0.000278	2.461234476	0.08191	4.71250612	0.0002
OR52N5	2.908000492	0.032436	0.043973921	0.97825	1.517791889	0.31092
OR52W1	3.284389478	0.024634	2.236232153	0.13944	0.20025713	0.90725
OR7E11P	3.751584246	0.003898	1.45074611	0.34698	2.984417771	0.0275
OR7E62P	2.15604065	0.009859	0.539906154	0.54948	0.505122492	0.58175
OR7E7P	-1.00460887	0.042651	-0.040397348	0.92527	0.536099619	0.19941
OR9Q1	3.600673871	0.019854	2.013729391	0.23005	3.460738064	0.02523
P2RY2	-1.069190635	0.032112	-0.77177591	0.10566	-1.726275952	0.0011
P2RY8	-1.704452593	0.024081	-0.492102377	0.42284	0.028758899	0.96143
PABPN1L	-3.095620961	0.035198	-0.61154451	0.62402	0.261486198	0.8226
PDE6A	-4.078043736	0.011219	-0.155133285	0.90496	0.119034585	0.92731
PENK	3.767219525	0.018611	5.598724136	0.00038	1.327534198	0.43772
PES1P2	3.650783588	0.000445	2.947112103	0.00494	2.536144555	0.01723
PGLYRP1	-3.020063201	0.032378	-1.323272516	0.30426	0.124660178	0.91097
PGLYRP2	6.228305428	2.75E-06	4.63994554	0.00052	5.097719366	0.00019
PIH1D3	1.731032967	0.024302	1.424171717	0.06413	1.77761337	0.01953
PLAC8L1	-1.580874339	0.034496	-0.414784575	0.47486	0.545390019	0.30509
PLP1	2.921371282	0.024673	3.77658478	0.00345	0.788431845	0.55764
PPP1R14D	2.183145347	0.007824	-0.119705013	0.89958	1.163365332	0.17575
PPP4R4	1.266174412	0.014789	1.45334715	0.00384	0.622421465	0.24413
PRSS30P	-2.455815815	0.013258	-0.729827659	0.40705	0.02361593	0.9777
PSG1	-3.221753607	0.037285	-1.464667791	0.32322	0.338600231	0.79049
PTCHD2	1.953800882	0.038095	1.892573651	0.04032	1.247201396	0.20861
PTGDR	-3.51183332	0.045577	-0.819312133	0.60109	-2.691124726	0.12551
PTGER1	3.403705664	0.022159	1.434438896	0.39448	4.039358115	0.00504
RAET1K	1.650340336	0.048823	0.819594452	0.34579	1.356423523	0.10725
RANP1	-1.607333449	0.025529	-1.423987788	0.03507	-1.706197236	0.0171
RBMS1P1	6.248107474	2.09E-06	5.870464291	8.45E-06	6.098599824	3.66E-06
RCVRN	1.379001486	0.011416	1.006224598	0.07019	1.243558143	0.02431
RGSL1	-1.904486837	0.028186	-1.227916803	0.13173	0.127555052	0.86977
P1-131F15.	2.761374595	0.000168	1.304035616	0.09211	2.197390424	0.0032
P1-228H13	3.334920085	0.041201	3.490403874	0.02964	2.433796073	0.15408
P1-232L24.	-3.523033182	0.045631	-2.425722596	0.1634	0.228697568	0.87906
P1-273G13	-0.882343438	0.026653	-0.297278475	0.40553	-1.215176485	0.00303
P11-100G15	3.076333442	0.028939	2.710728894	0.0551	1.954082468	0.19107
P11-137L10	2.434219743	0.021357	1.343978737	0.22635	2.551259315	0.01468
P11-17P16	2.500744665	0.044831	2.130864739	0.08861	2.339982009	0.06079
P11-182I10	3.019359213	0.01857	3.462430249	0.00588	1.774273891	0.19018

RP11-1J11.1	-1.306382818	0.019802	-1.426189054	0.00943	-0.087417764	0.86046
P11-210K20	1.755711947	0.036169	0.354057021	0.69766	2.790519854	0.00049
RP11-236F9.	2.597175487	0.005885	1.852463059	0.05554	3.565172613	0.0001
P11-247I13	-3.345598802	0.012078	-0.001826192	0.99852	-0.715123706	0.50497
P11-267C16	3.015924725	0.013639	0.128753953	0.92858	1.779267453	0.16284
P11-288E14	-2.684442994	0.032846	-3.701888861	0.0038	0.227771659	0.78856
P11-311B14	2.125102933	0.02143	3.126331601	0.00035	1.796237003	0.05478
RP11-34E5.4	1.911245497	0.022977	0.560464078	0.53071	1.39798088	0.10236
P11-366M4.	-1.54235084	0.046793	-0.531816144	0.46988	-0.554374521	0.45347
P11-380G5	-0.994219021	0.025666	-0.522058503	0.19129	0.023737186	0.95057
P11-382A18	2.885706545	0.035523	-1.205401612	0.47138	1.506779701	0.29302
P11-397P13	-1.911743924	0.033456	-0.362094098	0.58108	-0.05990823	0.92607
P11-39K24.	2.959279527	0.012241	2.526198728	0.03323	3.546351673	0.00229
P11-39K24	2.925094119	0.002056	2.559360818	0.00716	3.24229093	0.00054
P11-39K24	2.298704754	0.032077	2.224686194	0.03687	3.7395936	0.00032
P11-39K24	3.644864255	0.004784	2.855163336	0.02918	3.709718325	0.00394
P11-432A8	-3.461755682	0.004684	-0.185030832	0.8116	0.149306853	0.84534
P11-432N13	1.050691889	0.025609	0.743833064	0.11439	1.116754722	0.01583
P11-462D18	1.759106532	0.037484	0.98720728	0.25701	1.507474396	0.07652
P11-467H10	-2.169503026	0.014019	-0.951796972	0.2133	0.089664601	0.90253
P11-470B22	5.870842698	4.06E-06	5.23895107	4.29E-05	6.434282356	3.63E-07
P11-480I12	-2.762187786	0.047776	0.740522527	0.46593	-0.389523962	0.72311
P11-529H22	1.547028444	0.023844	0.337429535	0.64399	1.93661664	0.00376
P11-537I16	-3.397684698	0.007395	0.36368518	0.67221	0.838423767	0.31812
P11-561C5	4.129128297	0.046474	2.60632297	0.21308	2.518462878	0.23022
P11-568G11	-1.05747689	0.036616	0.285890163	0.47716	0.291313464	0.47552
11-655M14	3.14761817	0.039374	0.76531086	0.65708	3.887510724	0.00804
P11-655M14	3.52462685	0.001517	3.406297555	0.00212	2.102629246	0.06712
P11-734J24	-3.556337011	0.025913	-0.552986191	0.6801	-0.566654008	0.67564
P11-74E24	3.417569028	0.041753	1.26706903	0.48874	-0.521091803	0.78519
P11-777B9	2.213991944	0.033361	2.672169486	0.00772	1.886218384	0.07326
RP11-79D8.	2.692341713	0.018644	-0.953434588	0.47617	1.794297199	0.12206
P11-813N20	-2.638434532	0.041969	-2.930636235	0.02388	-1.084017564	0.31638
P11-829H16	1.737561593	0.009203	1.968617782	0.00254	2.558297743	6.91E-05
RP11-83J16.	-2.108882906	0.006664	0.116611195	0.85237	0.094191284	0.88138
RP11-8H2.1	2.620441231	0.007098	2.027844509	0.03978	2.803813984	0.00357
P13-401N8	-1.860408211	0.02045	-0.199996246	0.77125	-0.599806315	0.39685
P3-342P20	3.397124247	0.021146	0.043978745	0.97976	1.57807961	0.32996
P3-468K18	-3.08408933	0.046866	-1.213827494	0.39051	-0.689491782	0.61291
P5-1041C10	1.36968293	0.046441	1.202243448	0.07821	1.587444321	0.01851
P5-1049G16	1.106706278	0.039954	0.751232618	0.16534	1.991827622	7.75E-05
P5-1059H15	-3.050966293	0.038979	-2.08108806	0.12664	-3.908237065	0.00818
P5-1073F15	3.718901718	0.046403	1.474726592	0.46987	2.941372105	0.12315
RP6-24A23.	1.982185917	0.032789	1.858164761	0.04427	1.006086977	0.29706
RPL12P2	-2.728550427	0.048431	-0.646411349	0.58653	0.166609751	0.87992
RPL13AP20	-2.665824839	0.004186	-1.780138431	0.0294	-1.303557242	0.10228
RPL13P4	-3.490097335	0.048717	-0.068171734	0.96425	-0.926287672	0.56052

RPL21P23	3.940403245	0.001478	4.461003326	0.00024	3.219882344	0.01108
RPL23AP5(	-2.464071956	0.033289	0.125231947	0.85803	1.022290068	0.12056
RPL31P20	-4.003287582	0.000675	-1.629808742	0.03764	0.595430436	0.35299
RPL37P1	2.723601569	0.034666	2.377432909	0.06585	2.300152377	0.07832
RPL4P3	-1.645235614	0.018526	-0.436012761	0.45189	-1.080867425	0.08641
RPL5P9	-1.57015913	0.047158	0.331501517	0.57511	-1.972082254	0.01798
RPL7L1P1(	2.196834461	0.042464	1.207935731	0.28736	1.341008646	0.23856
RPL7P4	-1.973769408	0.01156	0.689296695	0.16493	-1.25506137	0.05353
RPL7P6	-2.418664953	0.007468	-0.27089213	0.66562	0.815682082	0.16632
RPL7P7	-1.684399857	0.048349	-0.133156507	0.85296	-0.151175223	0.83497
RPS15AP3{	3.766820486	0.021999	2.008311666	0.25905	1.605847938	0.38528
RPS4XP10	-2.648661892	0.047454	-0.452580075	0.65874	-0.68339758	0.52278
RPS6P16	2.685390653	0.000353	1.943608776	0.01134	3.37108628	4.15E-06
RTEL1P1	1.942911527	0.043709	1.525671559	0.11603	2.071232147	0.02909
SAA1	2.943114899	0.045099	-0.013357626	0.99376	0.142919654	0.93327
SEPT7P9	-4.258364125	0.006032	-2.822156259	0.0412	-1.632257892	0.19906
SETP6	3.399652569	0.02414	1.964227555	0.23003	3.860528546	0.00861
SH2D1B	2.048087289	0.001355	-0.102138325	0.88315	0.723493842	0.29311
SIRPB2	1.388766827	0.032909	-0.217151803	0.75966	0.836136298	0.20146
SLC22A16	-1.057509389	0.047824	-0.483140908	0.31326	-0.237184196	0.63084
SLC6A6	-1.741225713	0.03706	-1.00345946	0.21712	-1.324188585	0.11015
SNRPGP5	2.605353731	0.032063	3.306312819	0.00475	2.458711011	0.04317
SNX25P1	-2.630315481	0.011925	-0.365045657	0.611	-0.447798854	0.54091
SNX5P1	-1.112990078	0.019852	-0.612709478	0.15893	-0.926789897	0.04299
SPHKAP	-3.867143699	0.047673	-0.658873357	0.7042	-2.122559913	0.25796
SPINK13	1.735426109	0.008247	0.630376302	0.36163	0.700715954	0.31147
SPRR2D	5.019423825	7.19E-05	2.320303486	0.09114	0.200249385	0.90066
SPTA1	-2.85206089	0.027738	-2.444511218	0.04158	-1.248068555	0.29935
SRGAP2B	4.669795821	0.027622	5.781562902	0.00592	3.905965961	0.06785
SULT1A2	-1.399255698	0.040246	-0.46399979	0.46409	-0.844841911	0.1903
TBX10	-1.426929485	0.020528	-0.188258034	0.71106	-0.396843788	0.45058
TCAP	-1.38623861	0.005268	-0.670539723	0.12891	-1.239531801	0.00916
TCP10	-3.440682954	0.040746	-1.354599433	0.37988	0.207009581	0.8844
TDRD6	-2.474056791	0.040407	0.904739364	0.41773	-0.636449645	0.57838
TFAP2B	-18.63118294	1.35E-22	0.03544385	0.98273	-2.621329438	0.16131
TMIE	2.098730744	0.020802	0.66898188	0.48292	-0.928080803	0.41062
TMPRSS11I	-1.560575756	0.044315	1.278646331	0.04559	0.498621872	0.4483
TNFRSF18	2.931341059	0.024996	2.179291348	0.10191	1.61720421	0.24466
TNNI2	-1.519814645	0.014625	-0.61134295	0.26813	-1.267782654	0.03349
TPM3P6	-1.955872055	0.035117	-1.269631134	0.12536	-0.711673472	0.37452
TSPAN32	1.959131288	0.014559	0.88375842	0.26516	1.449658331	0.07008
TUBB4A	-2.616478682	0.038248	-1.988115333	0.06104	0.353486459	0.72575
TYRO3P	-1.306110969	0.022414	-0.142684303	0.78028	0.406921761	0.41806
UBA52P6	3.070037365	0.039099	3.461827948	0.01573	2.711241592	0.07285
UBE2QL1	2.358930141	0.031739	3.51457098	0.00118	-0.898145834	0.47756
ULBP2	3.107399165	0.035942	-0.677376695	0.69494	1.909061781	0.22422
USP2	-1.574307282	0.029841	-0.558108297	0.38484	-1.104110876	0.09259

USP26	-2.691686902	0.035613	-1.899443915	0.09701	0.97060411	0.37309
WNT10B	2.456355844	0.038196	4.08977328	0.00029	2.131069966	0.07679
YBX1P10	-2.844573245	0.048133	-0.366719356	0.76034	0.052301819	0.96445
ZNF300P1	1.756872901	0.038032	0.698710531	0.42611	0.260494633	0.77858
ZNF587P1	-1.440403506	0.041382	-0.998523816	0.12503	-0.374487725	0.54997
ZNF831	3.52997655	0.016249	4.0067739	0.00493	2.625180361	0.08841
ZNF962P	-4.081942535	0.003238	0.00688232	0.99456	-0.245416982	0.81183
ZSWIM5P2	-3.020861539	0.027239	0.037248684	0.97029	-0.777951087	0.47029
P11-270C12	0.954395578	0.151806	-8.600527341	1.54E-12	1.359362071	0.0409
ZMYM3	-0.137376033	0.146743	-0.569086145	2.13E-09	-9.96E-05	0.99916
GSTK1	0.121225052	0.140662	-0.488793074	4.16E-09	-0.067737288	0.41125
CLK1	0.191135649	0.247675	0.941486151	1.01E-08	0.272321653	0.09883
TTC31	-0.013332229	0.878723	-0.492663245	1.97E-08	-0.054521421	0.52799
PRKAB1	-0.18321689	0.105986	-0.635808496	2.60E-08	0.007629752	0.9459
PSPH	-0.233172483	0.099125	-0.7892185	4.30E-08	0.062591348	0.65439
SFPQ	-0.162726419	0.153465	0.613702948	6.84E-08	0.020024724	0.86043
SPCS2P4	-0.205660549	0.877691	-9.003954102	8.18E-08	-2.140106702	0.11122
PFKFB4	-0.397966177	0.161723	-1.495070527	1.69E-07	-0.595907001	0.03618
DOCK9	0.398413385	0.072442	1.155663256	1.74E-07	0.555826304	0.01211
TMEM136	0.320885931	0.083026	0.934518291	1.73E-07	0.387247411	0.03491
FTL	-0.102396631	0.365497	-0.588188837	2.04E-07	-0.324586109	0.00413
HPS6	-0.144093336	0.224125	-0.622532913	2.03E-07	-0.234080481	0.04783
RSRC2	0.091817754	0.501829	0.705431098	2.17E-07	-0.095396274	0.48527
SEPW1	-0.131764285	0.174434	-0.496431586	3.20E-07	-0.552606523	1.41E-08
P11-583F2.	0.282471834	0.399641	1.564745222	3.95E-07	1.357685956	1.54E-05
ELMO3	-0.110245356	0.373642	-0.632370839	5.03E-07	-0.112069902	0.36157
XRCC5	0.05625623	0.648909	0.615616197	6.16E-07	-0.147567152	0.23249
SH3YL1	-0.175518276	0.17221	-0.641404894	6.33E-07	-0.148685346	0.24693
GANAB	0.14904148	0.093702	-0.443372936	6.62E-07	0.012577523	0.88758
EWSR1	0.172053012	0.097391	0.51037358	8.40E-07	0.004340788	0.96664
USP38	-0.120262019	0.14847	-0.409269161	8.54E-07	-0.12414522	0.13429
MARS2	-0.232407443	0.242704	-0.985389377	8.97E-07	-0.278659414	0.16082
YTHDF2	-0.119002531	0.291824	-0.553169617	9.96E-07	-0.275843069	0.01458
HMOX1	-0.24539086	0.212691	-0.984546748	1.05E-06	-0.374739	0.05751
ARAP1	-0.014067623	0.923765	-0.716086781	1.22E-06	0.125127538	0.39402
SSXP10	-0.215147026	0.404877	-1.269095138	1.26E-06	-0.172958745	0.50218
TCTN3	0.027543664	0.786558	-0.493637637	1.37E-06	-0.222355979	0.02918
FAM76B	-0.185306007	0.193843	0.675517629	1.49E-06	-0.011485023	0.93573
ZNF581	-0.23497764	0.187891	-0.861669658	1.77E-06	-0.319711267	0.07179
NAA30	-0.038304116	0.711336	-0.490859009	1.92E-06	-0.379335895	0.00024
GUSBP9	-1.075761253	0.384631	-7.577547732	1.93E-06	-0.107685095	0.93018
TMEM101	-0.16382598	0.172669	-0.575826148	2.13E-06	-0.314108424	0.00921
TMEM141	-0.155731581	0.330279	-0.753069661	2.69E-06	-0.406038379	0.01123
SCO2	0.131178009	0.286241	-0.595421518	2.86E-06	-0.158589313	0.20241
CD46	-0.131442113	0.198064	-0.47775162	2.90E-06	-0.279014991	0.00629
EPS8L2	-0.230989053	0.196385	-0.837064459	2.93E-06	-0.070896612	0.69145
ALDH18A1	-0.14136242	0.317174	-0.657205171	3.38E-06	-0.331253509	0.0191

DDB2	-0.047645709	0.848542	-1.164618029	3.56E-06	-0.597824495	0.01685
DDX28	-0.113516069	0.444674	-0.702143055	3.61E-06	-0.163803572	0.26771
AADAT	0.148287412	0.352424	0.71978465	4.28E-06	0.092264126	0.56152
PGPEP1	-0.404424635	0.053139	-0.958365309	4.69E-06	-0.29585618	0.15665
FBXO2	-0.133778345	0.581874	-1.121640535	4.86E-06	-0.579508059	0.01748
PAIP2B	-0.210484403	0.083156	-0.556620191	4.95E-06	-0.084965866	0.47892
NDUFA8	-0.064990356	0.550716	-0.500328666	5.51E-06	-0.283708661	0.00963
PPT2-EGFL	-0.613373252	0.633454	-7.366082018	5.78E-06	-0.155784207	0.90287
RCC2	0.202553699	0.055611	0.47566814	5.88E-06	0.091635723	0.38636
XRN1	0.247836197	0.188052	0.849287068	6.10E-06	0.402220946	0.0324
TMSB4XP6	0.23463737	0.197622	0.820379162	6.34E-06	0.141908378	0.43581
TMEM139	0.385646308	0.074921	-1.00084832	6.83E-06	-0.528920958	0.0163
ZNF148	-0.07816002	0.300726	-0.338367944	7.11E-06	-0.077074408	0.30577
AGPAT1	0.224359495	0.069156	-0.55728307	7.29E-06	0.028032782	0.82052
RP5-850E9.	0.803257177	0.865823	-21.78690403	7.38E-06	-21.5214404	9.53E-06
MAP4K5	0.18233396	0.063088	0.436854988	7.55E-06	0.228649216	0.01972
MRPS18B	-0.121035294	0.195463	-0.419074467	7.80E-06	-0.124290284	0.18076
RPS27L	-0.11966694	0.376614	-0.604180237	8.19E-06	-0.347297408	0.01029
RPS9	0.119910379	0.624787	-1.096717947	8.18E-06	-0.334941658	0.17226
CDX2	-0.586958213	0.120764	-1.694793305	8.45E-06	-1.033970155	0.0063
TAC4	0.611046896	0.209915	2.002267127	8.59E-06	1.31128302	0.00461
WEE1	0.181790547	0.090196	0.472352607	8.83E-06	0.172243367	0.10646
MGAT1	0.038621854	0.677592	-0.412783225	9.84E-06	-0.184318566	0.04807
ZNF124	-0.154818525	0.127528	-0.448778842	9.85E-06	-0.277602285	0.00626
PTPRR	-0.478527528	0.109988	-1.322634841	1.07E-05	-0.74237469	0.01325
ZNF622	-0.135390886	0.205014	-0.469751551	1.15E-05	-0.464411234	1.66E-05
MAMDC4	-0.257592867	0.122953	-0.734523859	1.21E-05	0.066750975	0.68671
FBXO44	-0.197873215	0.285074	-0.810814245	1.29E-05	-0.016742398	0.92708
TMSB10	-0.016998144	0.820322	-0.325824968	1.34E-05	-0.303848274	4.95E-05
RRM2B	-0.350554388	0.114808	-0.96758337	1.35E-05	-0.4334989	0.05142
OPRK1	-0.123799851	0.684631	-1.327805767	1.42E-05	-0.094112685	0.75767
PXDNL	0.979069813	0.08796	2.366097448	1.48E-05	0.675354413	0.23426
GLRX	-0.014097974	0.934304	-0.751683316	1.50E-05	-0.697424213	6.04E-05
SAT2	-0.267464497	0.065529	-0.630257138	1.51E-05	-0.530857907	0.00027
SGK2	-0.286940696	0.098482	-0.751312049	1.57E-05	-0.213310175	0.21789
GTF2H2B	-0.306557085	0.055406	-0.692911395	1.60E-05	-0.219265123	0.16905
TGFA	0.555236175	0.069432	1.303871004	1.65E-05	1.027375434	0.00072
TESK2	-0.007265819	0.964795	-0.727743549	1.66E-05	-0.476060734	0.00432
CLIP1	0.117384006	0.21598	0.40252512	1.73E-05	0.14669261	0.1208
LAMTOR4	-0.240484795	0.050172	-0.526112059	1.82E-05	-0.287866928	0.01858
RP4-765C7.	-0.051625844	0.804687	-0.900560682	1.88E-05	-0.375697809	0.07263
SLC2A8	-0.161805867	0.437713	-0.900255732	1.87E-05	-0.210844056	0.31403
INPP5D	-0.505425996	0.116268	-1.404030725	1.90E-05	-1.153353071	0.00042
RAB17	-0.249807688	0.275922	-0.990075281	2.08E-05	-0.353926976	0.12258
FNBP4	-0.044649692	0.720432	0.527427638	2.10E-05	0.324328493	0.0091
C17orf58	0.101532111	0.556567	0.694614184	2.16E-05	0.103156477	0.54674
PEX13	-0.167377027	0.094632	-0.425185767	2.16E-05	-0.194059506	0.05232

SLC9A3	-0.089359458	0.772102	-1.353324075	2.16E-05	-0.372049294	0.23399
PRMT2	-0.042601396	0.525627	-0.283779788	2.21E-05	-0.101027341	0.13092
ATP6V0C	0.550357793	0.065684	1.25307171	2.28E-05	0.589363238	0.04832
RPS21	-0.068726994	0.639341	-0.618649854	2.56E-05	-0.320038708	0.02924
TM9SF1	-0.047424008	0.602469	-0.381444621	2.81E-05	-0.110098269	0.22553
RNF185	0.024228901	0.796969	-0.395899305	2.93E-05	-0.200728029	0.03371
ACSL3	0.106792331	0.391524	0.519707475	2.95E-05	0.006130676	0.96077
TFIP11	-0.061865193	0.668291	-0.607083774	2.97E-05	-0.244406296	0.09033
BZW1	0.062749288	0.476025	0.366956178	3.02E-05	-0.265149619	0.00261
RPS19	0.041772307	0.764944	-0.583074766	3.03E-05	-0.24771681	0.07628
MPP5	-0.074270586	0.34961	-0.329391784	3.10E-05	-0.040835433	0.60508
MISP	-0.375880778	0.052821	-0.8076884	3.23E-05	-0.376809507	0.05208
HEXIM1	-0.191122424	0.142052	-0.54107389	3.33E-05	-0.122304123	0.34542
CLP1	-0.219102657	0.077504	-0.511586795	3.53E-05	-0.395315673	0.00141
APMAP	-0.041888274	0.709689	-0.465930822	3.62E-05	-0.358333102	0.0015
ZNF582	-0.175821514	0.444744	-0.969484972	3.64E-05	-0.064444975	0.77426
RPL41	-0.018626156	0.844175	-0.390477747	3.78E-05	-0.290538209	0.00217
FMR1	-0.217117985	0.066068	0.47750423	3.82E-05	-0.029311239	0.80285
CD99	0.056516018	0.787431	0.858795137	3.96E-05	-0.053434138	0.79884
POLL	-0.072863392	0.58396	-0.546918499	4.07E-05	-0.014341821	0.91349
ZMAT3	-0.065960235	0.668224	-0.632665763	4.07E-05	-0.262247153	0.08829
HDX	0.237865477	0.295068	0.915973967	4.14E-05	0.09485948	0.67672
GPX4	0.117917285	0.815431	-2.08196116	4.35E-05	-0.161044518	0.75002
MIEN1	-0.12921519	0.327311	-0.540234885	4.50E-05	-0.301953268	0.02199
RPUSD1	-0.119553376	0.489419	-0.710050337	4.54E-05	-0.215535815	0.21156
PSMD13	-0.122139323	0.130703	-0.328966604	4.65E-05	-0.216015824	0.00757
PKHD1	0.008848775	0.954961	-0.644547396	4.78E-05	0.172162088	0.26838
ARID2	0.098532628	0.307016	0.389122473	4.79E-05	0.199591433	0.03793
TPRG1L	-0.089725304	0.490296	-0.530625008	4.98E-05	-0.126709732	0.32893
TRIP12	0.022339102	0.805312	0.366470694	5.11E-05	0.048128929	0.59511
BCOR	-0.003550177	0.983411	0.681926073	5.37E-05	0.387242293	0.02241
RPS5	0.194534539	0.113456	-0.496837176	5.39E-05	-0.066910589	0.58627
RPLP2	0.073781836	0.605558	-0.576923715	5.51E-05	-0.216663688	0.1296
RPL8	-0.04031011	0.759134	-0.529447767	5.67E-05	-0.268408143	0.04121
BNIP3	-0.129113609	0.43587	-0.66752016	5.78E-05	-0.429680205	0.00959
ANTXR1	0.149288581	0.457635	0.799151937	6.36E-05	-0.002421338	0.99039
TMEM256	-0.101682083	0.408924	-0.494054485	6.47E-05	-0.534381262	1.76E-05
ZNF79	-0.322933095	0.197431	-1.013551514	6.57E-05	-0.339550927	0.1741
GJC2	-0.02416705	0.931948	-1.195923403	6.67E-05	0.007306524	0.97924
RPL13	0.026194734	0.826845	-0.477757883	6.67E-05	-0.216388262	0.07083
RIMKLA	-0.357175918	0.193159	-1.098452643	6.83E-05	-0.029422751	0.91411
HPGD	-0.279332137	0.444118	1.449722976	6.98E-05	0.406753753	0.26477
PPP1R16A	-0.043942358	0.765982	-0.588396972	7.10E-05	0.000110654	0.9994
SMARCC1	0.100013307	0.238469	0.335074854	7.31E-05	0.166296815	0.0499
HERPUD2	0.191132228	0.212079	0.601003247	7.39E-05	0.18154065	0.23506
PDE4D	0.297277956	0.101123	0.715672495	7.43E-05	0.333736517	0.06557
HDAC10	-0.185421175	0.399882	-0.88508779	7.77E-05	0.28881077	0.18439

SEC13	-0.068532709	0.358852	-0.294340251	8.03E-05	-0.325654469	1.38E-05
TMEM88	0.450988848	0.299857	1.658354773	8.28E-05	0.049695228	0.91068
KIAA2013	-0.125370756	0.354062	-0.532952856	8.53E-05	-0.174657797	0.19601
SLC39A14	-0.2250657	0.091694	-0.524508258	8.51E-05	-0.20122829	0.13127
CYP2W1	-0.230658054	0.463356	-1.244454584	8.66E-05	-0.178405037	0.57008
FGF20	-0.301117995	0.572696	1.918499225	8.76E-05	1.192925087	0.01656
GOLT1A	-0.195181927	0.428013	-0.979766394	8.85E-05	-0.459634156	0.06312
PROC	-0.212772021	0.400405	-1.003621377	8.85E-05	-0.049419477	0.84436
SPRY2	-0.269904517	0.136437	-0.710603078	8.98E-05	-0.590172238	0.00115
TACSTD2	0.129374577	0.541068	0.823989975	9.06E-05	0.396997966	0.06003
FOXI3	0.552100002	0.383181	2.325746442	9.40E-05	0.718205306	0.24984
ATHL1	-0.078004826	0.675075	-0.728383829	9.94E-05	-0.311215075	0.09501
CCDC160	1.053540467	0.062375	2.048459026	0.0001	1.574270218	0.00369
UST	-0.06047661	0.847127	1.19737475	0.0001	0.258943138	0.40628
YS1-DBNDI	0.501490761	0.658397	-5.87235677	0.0001	0.25926923	0.81956
MPG	-0.263582135	0.110621	-0.641907645	0.00011	-0.407220525	0.01385
PTPRG	-0.026016995	0.893876	0.750859503	0.00011	-0.014072594	0.94244
TMEM115	0.024172549	0.831212	-0.442615331	0.00011	-0.049675867	0.66068
NDUFA13	-0.066757553	0.394683	-0.302702796	0.00011	-0.354573699	6.95E-06
NTN1	0.364613667	0.300621	1.350944387	0.00011	0.648355766	0.0649
COX20	-0.110439932	0.257722	0.370288473	0.00011	-0.019948571	0.8363
PCDHB2	0.218168239	0.152643	0.583760664	0.00011	0.035667025	0.81532
TSC22D2	0.19316114	0.129439	0.488850259	0.00011	0.393235358	0.00191
CHD3	-0.149138648	0.455204	0.770214362	0.00011	0.012050066	0.95187
LAMTOR5	-0.015579571	0.878484	-0.39263812	0.00012	-0.466960821	7.27E-06
ANKMY2	-0.059185049	0.574377	-0.406661122	0.00012	-0.072714482	0.48892
NTPCR	-0.104553889	0.331215	-0.412041552	0.00012	-0.330657762	0.00193
EDA2R	-0.106488696	0.641398	-0.888982934	0.00012	-0.322538566	0.15917
KIAA1549	0.210945592	0.150239	0.551625866	0.00012	0.503753611	0.00048
RPS27	-0.080679961	0.48623	-0.444087086	0.00013	-0.262357209	0.02357
TFG	-0.140571351	0.15966	-0.382529963	0.00013	-0.29564383	0.00311
USP42	0.061829452	0.613047	0.460740485	0.00013	0.3138101	0.00978
LINC00493	-0.103597814	0.323453	-0.402702819	0.00013	-0.132212456	0.20545
ME3	-0.088673261	0.569168	-0.601763847	0.00013	-0.350157478	0.02603
RPS14	-0.108684882	0.390757	-0.484337888	0.00013	-0.315060696	0.01287
C14orf105	-0.336635249	0.22279	-1.054197538	0.00014	-0.625026145	0.02352
SMIM20	0.039374802	0.772026	-0.522367931	0.00015	-0.148206711	0.27651
ATP5G2	0.113300392	0.200358	-0.336691113	0.00015	-0.146554356	0.09814
RRC37A16	0.083401323	0.595002	0.588220471	0.00015	0.263420068	0.09112
KLHL3	-0.201817912	0.332433	-0.795089177	0.00015	0.31192763	0.13164
UBAP2L	0.261335077	0.076488	0.557937444	0.00015	0.413617565	0.005
LPIN3	-0.254059515	0.232356	-0.807046851	0.00015	-0.054723542	0.79602
EEF1A1P6	-0.265300623	0.209375	-0.802132483	0.00015	-0.641456716	0.00247
RUFY3	0.167518663	0.105943	0.38556836	0.00015	0.315830173	0.00203
COX16	-0.137365824	0.255556	-0.45653117	0.00016	-0.152700848	0.20522
GALK2	-0.26835155	0.064884	-0.551305496	0.00016	-0.276804804	0.05656
MRPL54	-0.097694979	0.39742	-0.437679805	0.00016	-0.447534215	0.00015

NCLN	-0.158518089	0.195828	-0.463337574	0.00016	-0.009528005	0.93799
TOP3B	-0.175182199	0.135729	-0.443112494	0.00016	-0.226772012	0.05236
:TB-63M22.	5.523524305	0.263633	-19.02531591	0.00016	-18.83077271	0.00019
AREL1	0.156430791	0.174139	0.431430526	0.00017	0.07798163	0.49797
LTBP3	0.04914222	0.835753	0.884261345	0.00017	0.403947098	0.08669
TMEM41B	-0.143903447	0.121005	-0.348462433	0.00017	-0.176168167	0.05743
RPS28	0.153060684	0.251406	-0.502618692	0.00017	-0.049746422	0.70949
FAM69B	0.012727306	0.934441	-0.586159208	0.00018	-0.466254736	0.00286
VEZF1	0.130978702	0.127626	0.319969574	0.00018	0.027389745	0.74984
TRANK1	-0.398352335	0.051409	-0.768536907	0.00018	-0.332810013	0.1037
AEN	0.165720505	0.326209	-0.635206929	0.00018	-0.259144898	0.1254
ETFB	-0.092288922	0.491592	-0.502152423	0.00018	-0.36083197	0.00709
NFIB	0.299989616	0.062942	0.601550551	0.00018	0.426700097	0.00803
PLCD1	-0.291395272	0.115244	-0.694284582	0.00018	-0.139178359	0.44955
BAALC	-0.056907405	0.927495	2.234701972	0.0002	0.196542447	0.75245
CNKSR2	0.023424917	0.971752	2.414238465	0.0002	-0.12167427	0.85641
KDM4C	-0.205597601	0.170769	-0.558227149	0.0002	-0.164205364	0.27346
HDAC4	0.468078402	0.050614	0.883208134	0.00021	0.749135266	0.00168
RAB26	-0.095054231	0.700081	-0.928636458	0.00021	0.056668876	0.81564
SLC38A11	-0.908728807	0.051607	-1.774914991	0.00021	-0.305995422	0.50776
MFGE8	0.061546138	0.636078	-0.483649985	0.00022	-0.159007205	0.2229
MRPL17	-0.225670453	0.145719	-0.572880065	0.00022	-0.44242372	0.00437
OAZ1	0.08593331	0.293183	-0.301853407	0.00022	-0.192771568	0.01851
TSPAN18	0.344171355	0.177293	0.931573335	0.00022	0.174497122	0.49677
MAP7D3	0.00811826	0.977037	1.028713869	0.00023	-0.151166783	0.59323
BCL2L1	-0.032561374	0.841143	-0.599619599	0.00023	-0.839460761	2.60E-07
CACNA2D1	-0.423642217	0.180863	1.154093376	0.00023	-0.003713358	0.99064
NAALADL2	0.209765235	0.371798	0.862418057	0.00023	0.195437832	0.40474
ECHS1	-0.176076857	0.080659	-0.369425848	0.00024	-0.392172451	0.0001
ALDOC	0.008362167	0.969954	-0.817291585	0.00024	-0.134286069	0.54528
BCL2	0.492449992	0.211241	1.436301173	0.00024	0.326238	0.40733
TCEB2	-0.048479832	0.577783	-0.320145266	0.00024	-0.286257784	0.00107
C16orf91	-0.035814206	0.827377	-0.615278487	0.00024	-0.291012713	0.07891
METTL13	-0.224026343	0.135641	-0.550461266	0.00024	-0.409667966	0.00633
ROBO1	0.048149806	0.707955	0.469958568	0.00024	-0.050723127	0.69291
PBRM1	-0.052834807	0.455297	0.257244619	0.00025	0.04421444	0.53128
STK24	-0.095377743	0.396455	0.410456939	0.00025	0.205582074	0.06681
GXYLT2	0.279432832	0.290392	0.955290104	0.00025	0.057809005	0.82721
UBL5	0.00778359	0.945566	-0.418793613	0.00025	-0.292694187	0.0105
POLG	0.093366957	0.465415	-0.469149117	0.00025	0.029969972	0.81415
PRDX2	0.082917523	0.316888	-0.303326592	0.00026	-0.179269836	0.03074
SRSF5	-0.195680726	0.078438	-0.406521748	0.00026	-0.436354081	8.76E-05
ANAPC16	-0.126952073	0.243016	-0.395115701	0.00026	-0.297220271	0.00618
C19orf33	-0.04287016	0.790745	-0.592723751	0.00026	-0.399014873	0.01386
SIM1	-0.361082416	0.412185	-1.631655839	0.00026	1.057702235	0.01478
SLC26A6	-0.280053943	0.106424	-0.63385065	0.00026	-0.203152795	0.24006
GCHFR	-0.118923506	0.436013	-0.563485694	0.00026	-0.398512218	0.00964

KTI12	-0.266205809	0.066209	-0.527841232	0.00026	-0.4095501	0.00474
HCAR1	-0.249664613	0.231108	-0.764429136	0.00027	0.081860289	0.69191
KIAA1107	-0.105294671	0.555132	-0.664894929	0.00027	-0.347806905	0.05336
ETV1	0.331967706	0.121973	0.778229967	0.00027	-0.217108937	0.31339
PPP4R1	0.003057883	0.977103	0.385666657	0.00028	0.058979581	0.5799
SFSWAP	0.129767913	0.467438	0.647144782	0.00028	0.107746118	0.54557
IFIT3	0.42290265	0.132796	1.002147098	0.00028	0.259680001	0.35652
EXOC8	-0.014173373	0.857911	-0.287401132	0.00028	-0.034508644	0.66106
SLIT3	0.587473076	0.083114	1.226188089	0.00029	0.758213019	0.02519
PODXL	-0.552394396	0.284353	1.846890794	0.00029	-0.457911218	0.37375
FECH	0.247619709	0.152138	0.620162519	0.00029	0.11291488	0.51374
BCKDHB	-0.236696803	0.072454	-0.47625512	0.00029	-0.29384905	0.02497
RPS18	-0.090042482	0.4911	-0.473491368	0.00029	-0.292618008	0.02526
P11-381E24	0.090857343	0.771911	1.024413677	0.0003	0.034975678	0.91035
GART	-0.026827953	0.861396	0.554451782	0.0003	0.010475943	0.94565
NIPSNAP1	-0.169135581	0.135381	-0.409311299	0.0003	-0.459780333	5.18E-05
MAPKAP1	0.059775593	0.480742	0.302457919	0.0003	-0.020061291	0.81295
TRPT1	0.010179857	0.952918	-0.628573698	0.00031	-0.278201769	0.10753
CNPY3	0.011929772	0.902465	-0.352704909	0.00031	-0.410539096	3.36E-05
SNAI2	0.264212305	0.338298	0.991804655	0.00031	0.413929731	0.13308
ENDOV	-0.068748365	0.555454	-0.421741856	0.00031	0.203135563	0.07213
HPN	0.033449795	0.886391	-0.845958059	0.00031	-0.247210639	0.29121
IFITM1	0.145422965	0.464683	-0.71858426	0.00032	-0.527806694	0.00822
PAGR1	-0.176090573	0.088072	-0.369106251	0.00032	-0.230515696	0.02497
VOPP1	0.016354694	0.876588	-0.378922069	0.00032	-0.048726058	0.64263
WLS	-0.061811724	0.629033	-0.460034701	0.00033	-0.135310066	0.2911
FBXO11	0.046011092	0.659844	0.371798768	0.00034	0.307835063	0.00306
FAM134A	-0.118924467	0.265161	-0.382117126	0.00034	-0.234239507	0.02808
CCDC167	0.017258732	0.916867	-0.607673288	0.00035	-0.279810195	0.09463
RSU1	0.145373462	0.222525	0.422336354	0.00035	-0.049196151	0.67964
TINAGL1	0.249149474	0.166428	-0.644454634	0.00035	0.055312817	0.75867
MED8	-0.21553007	0.091772	-0.456292261	0.00035	-0.390291708	0.00229
ZFAND3	-0.003927018	0.974804	0.439783591	0.00035	0.120210523	0.33089
COX15	-0.097780478	0.348062	-0.371413688	0.00036	0.007471025	0.94242
PLAGL2	0.05917904	0.711005	-0.573628806	0.00036	0.330284875	0.03765
TRIM38	-0.122639885	0.296646	-0.419698712	0.00036	-0.155894544	0.18348
DDX25	0.072352869	0.845417	1.261041197	0.00037	0.366523665	0.3118
DDIT4	0.040715474	0.887592	1.020486247	0.00038	0.697138865	0.01529
CARKD	-0.144626297	0.156869	-0.36190005	0.00038	-0.119970484	0.23711
TAMM41	-0.214050676	0.188514	-0.574191294	0.00039	0.100110946	0.52347
SMIM3	-0.15780844	0.471675	-0.7807159	0.00039	-1.072610852	1.33E-06
RWDD4P2	0.74869994	0.23593	2.206728001	0.0004	0.567735193	0.36914
CTDSPL2	0.110863224	0.143935	0.265573971	0.00041	-0.029800517	0.69433
EXOSC10	0.116703586	0.091994	0.241901417	0.00041	0.218010373	0.0016
HNRNPH1	0.046455167	0.655037	0.36683253	0.00041	-0.140180098	0.17761
PHLDA3	-0.129807893	0.427759	-0.581907836	0.00041	-0.348465702	0.03339
TULP3	-0.081627155	0.554561	0.482652743	0.00041	-0.025555959	0.85318

MT-ND1	0.416174942	0.154914	-1.033345436	0.00042	-0.117197592	0.68883
VWA2	-0.137243488	0.751015	-1.577944754	0.00042	-0.750154162	0.08627
THOC3	-0.145558595	0.289768	-0.486561699	0.00043	-0.338762056	0.0142
C19orf48	-0.123145989	0.361579	-0.475930777	0.00043	-0.06773166	0.61557
MBD2	0.007201454	0.95502	0.446231058	0.00043	0.160159247	0.20796
ISX	-0.511120628	0.31315	-1.793151393	0.00043	-1.398447653	0.00595
ERBB2IP	0.161087469	0.201053	0.44277711	0.00043	-0.001477795	0.99064
HEMK1	-0.225669981	0.152113	-0.5525233	0.00043	-0.298186957	0.05619
RPL10A	0.034098649	0.760317	-0.393383346	0.00043	-0.232462552	0.03761
FOXK1	0.147912515	0.355289	0.560608811	0.00044	0.565861342	0.00039
CBR1	-0.006602269	0.96998	-0.629424131	0.00044	-0.632076247	0.00049
COX7C	0.062670931	0.562782	-0.380189116	0.00045	-0.13863675	0.20069
GBE1	-0.146297333	0.449293	-0.6799553	0.00045	-0.26972421	0.1631
LGI2	0.10973466	0.750832	1.178862419	0.00046	-0.221815361	0.52141
MT-ND2	0.195829466	0.512112	-1.048112767	0.00046	-0.235312291	0.43096
NUTF2	0.065635772	0.531246	0.365406941	0.00045	-0.186287735	0.07595
TMEM219	-0.192330952	0.083113	-0.389751869	0.00045	-0.190303874	0.0856
POLR1D	-0.117865506	0.353541	-0.445191219	0.00046	-0.286509026	0.02406
WRAP73	-0.147882396	0.335571	-0.535711615	0.00046	-0.402538281	0.00813
RAB27A	0.422132861	0.054323	0.765591733	0.00047	0.872476682	6.66E-05
RAB40C	-0.236775666	0.107318	-0.514448535	0.00047	-0.076543749	0.59892
CCAR1	-0.140276451	0.27716	0.449625391	0.00047	0.042640543	0.74115
C11orf35	-0.169505779	0.54807	-1.019143963	0.00048	0.08388674	0.76291
RHOQ	0.211103011	0.214308	0.589845032	0.00048	0.225227691	0.18447
FASTKD5	0.117550712	0.371466	-0.462503953	0.00048	-0.064257111	0.62557
MED6	0.021514522	0.799226	-0.296260486	0.00048	-0.264063338	0.00201
DDHD1	0.216627021	0.302264	0.728832942	0.00049	0.181846665	0.38853
PDRG1	-0.261739096	0.140666	-0.620800474	0.00049	-0.599891204	0.00079
LYPD6	0.38255062	0.051908	0.675469209	0.0005	0.223234231	0.25703
CDK20	0.032356345	0.855195	-0.623651395	0.0005	-0.412225341	0.02181
NREP	0.382400504	0.061515	0.710914481	0.0005	-0.084800309	0.67854
PAQR7	-0.294276722	0.050751	-0.522598516	0.0005	-0.190906606	0.20001
SART1	-0.042983402	0.788744	0.552958408	0.00051	0.077534606	0.62812
FGFR2	0.28174921	0.102123	0.598266576	0.00051	0.359115802	0.03712
IL1R2	0.602723862	0.169146	1.492189968	0.00051	0.034079477	0.93909
JTB	-0.160853017	0.117876	-0.355983633	0.00052	-0.126539716	0.21658
RPA3	-0.296159699	0.081365	-0.581554147	0.00052	-0.196718167	0.23999
TNFRSF10C	-0.158516752	0.608163	-1.08137296	0.00052	-0.506661212	0.10223
PMM1	-0.233725639	0.226143	-0.675886968	0.00053	-0.59846309	0.00217
ARID4B	0.181014411	0.056245	0.326357621	0.00054	0.008224018	0.93099
CTC-503J8.[illegible]	0.046984176	0.955541	-3.290264259	0.00054	-0.00082796	0.99922
RPL7A	-0.077015373	0.5048	-0.399152334	0.00055	-0.316209346	0.00619
DNAJB6	0.153864656	0.127323	0.346375504	0.00056	-0.124837994	0.21647
PCNX	0.120417502	0.398678	0.491441839	0.00056	0.245560253	0.08509
RITA1	-0.232165485	0.085603	-0.464012846	0.00056	-0.316246819	0.01877
HEMGN	0.002840823	0.99553	1.542126431	0.00057	1.288924163	0.00465
SMARCA4	-0.047991704	0.717043	0.454343929	0.00057	0.298681599	0.02367

SIX4	0.011798652	0.965989	0.944154637	0.00057	0.233509653	0.39762
SPATA18	-0.111737049	0.642738	-0.837599254	0.00057	-0.232688308	0.33474
MORC3	-0.042438198	0.650217	0.318633585	0.00058	-0.140319636	0.13372
PI4KAP2	-0.11478801	0.357429	-0.429186529	0.00058	0.130548922	0.29168
SFMBT2	-0.040067267	0.861962	0.774932359	0.00058	0.473591956	0.03757
PSMD8	-0.024234603	0.824372	0.373461262	0.00059	-0.176998029	0.10525
LAMP1	-0.017691602	0.880193	0.400540309	0.0006	0.090297045	0.44064
DBN1	0.124857471	0.576159	0.765126047	0.0006	-0.176668076	0.42922
ZNF330	-0.185625441	0.068709	-0.348099057	0.0006	-0.211171992	0.03794
SLC35A1	-0.188139272	0.06611	-0.348681737	0.0006	-0.17510512	0.08489
SLC2A1	0.035254399	0.90002	-0.962695593	0.00061	-0.463509644	0.0987
CYHR1	-0.156223951	0.185242	-0.401259693	0.00061	0.097476648	0.39969
PTCH1	0.303925069	0.25257	0.905408715	0.00063	0.436339888	0.09986
BAK1	-0.204456608	0.12751	-0.457562702	0.00063	-0.557123964	4.42E-05
ADPRHL2	0.076963698	0.47296	-0.369237017	0.00063	-0.226495176	0.03617
ARHGAP5	-0.143530755	0.17908	-0.364925994	0.00063	-0.134028776	0.2094
AURKAIP1	-0.095552832	0.433357	-0.417101235	0.00064	-0.38351936	0.00179
ADCK3	-0.182516994	0.337802	-0.651766155	0.00064	-0.006902402	0.97098
KDM7A	-0.03464568	0.861903	0.676332564	0.00064	0.381702053	0.05441
NID2	0.286351051	0.414747	1.196109519	0.00064	0.180337985	0.60769
TMEM43	0.045170518	0.653585	-0.344191801	0.00064	-0.232588473	0.02117
TMEM87B	-0.173963058	0.142139	-0.403773009	0.00064	-0.130591546	0.26937
C6orf48	-0.122342477	0.305427	-0.407473525	0.00065	-0.240167741	0.0442
ZNF121	0.029250964	0.768217	-0.338567754	0.00065	-0.061987235	0.53216
RPS3A	-0.147774572	0.233983	-0.422573198	0.00067	-0.293425817	0.01812
NONO	0.112287237	0.524712	0.599469069	0.00068	-0.193897421	0.27208
SEPT7P6	0.692345648	0.313203	2.08484678	0.00068	1.964349448	0.00156
FAM162A	-0.13323649	0.431155	-0.575197312	0.00069	-0.277529665	0.10108
HLA-DQB1	0.099174869	0.803078	-1.361308176	0.00069	-1.819554507	6.34E-06
JMY	-0.056681261	0.805301	0.76999658	0.0007	0.300686637	0.18841
NUB1	0.197796359	0.052806	0.344534247	0.0007	-0.126845176	0.2161
CD93	0.484571465	0.337368	1.670109255	0.0007	0.008275079	0.98706
DCTN6	-0.02792398	0.73299	0.270719294	0.0007	-0.123176323	0.13357
SNRPGP15	1.061933442	0.065035	1.931213909	0.0007	1.021287361	0.0757
CAP2	0.419664884	0.146195	0.942967375	0.00072	-0.031340199	0.91531
TRIAP1	-0.266636916	0.071042	-0.499043437	0.00072	-0.41766526	0.00472
ZNF324	-0.19611772	0.17724	-0.490364154	0.00072	-0.133178297	0.35575
AAR2	0.089799751	0.38438	-0.351875222	0.00072	-0.029031886	0.77858
TMEM208	0.234892391	0.169226	-0.589631965	0.00072	-0.186624622	0.27868
PITHD1	-0.181486947	0.056573	-0.319513477	0.00074	-0.180910349	0.05633
VASH2	0.323726454	0.317044	1.081769489	0.00074	0.37895179	0.24195
ZDHHC18	-0.169009614	0.21824	-0.463562318	0.00074	-0.320357925	0.01914
TMEM176B	0.148591492	0.648899	-1.10537057	0.00075	-0.793483665	0.01541
MYCBP2	0.245292182	0.090598	0.487229703	0.00075	0.502053763	0.00052
RPS15A	-0.159679704	0.137826	-0.362416457	0.00076	-0.295976963	0.00595
RNF7	-0.0436299	0.700027	-0.381286032	0.00077	-0.274552834	0.01545
DVL1	-0.134052882	0.407115	-0.544426856	0.00078	-0.234961776	0.14592

ASB7	-0.045663494	0.710134	-0.409128613	0.00078	-0.188209981	0.12468
TKT	-0.081755674	0.588403	-0.507255608	0.00079	-0.248145881	0.10052
CCND2	0.007992431	0.947894	-0.41024778	0.0008	-0.061055006	0.6176
SCIN	-0.135329228	0.645242	-0.987359274	0.0008	-0.103033643	0.72585
ACADS	-0.001353878	0.99363	-0.575614195	0.00081	-0.247614536	0.14612
PCM1	-0.120844136	0.101707	0.245595938	0.00082	0.004118978	0.95545
UQCC1	-0.068688815	0.547054	-0.380236536	0.00082	0.021938752	0.84642
CHMP3	-0.018060026	0.864671	0.352044356	0.00083	-0.15829575	0.13524
IPO9	0.147725955	0.277753	0.45360608	0.00084	0.049255879	0.71726
SYTL2	0.334790609	0.132502	0.742510408	0.00084	0.753669604	0.0007
SMC6	-0.16014551	0.111721	0.328555054	0.00084	-0.12225497	0.22246
AP1S1	-0.035474257	0.789077	-0.443253086	0.00085	-0.459171618	0.00057
PDSS2	-0.117933792	0.321501	-0.398003601	0.00085	-0.330520633	0.00567
SERINC5	0.052728236	0.729353	-0.509056495	0.00085	0.046741532	0.75908
TTI1	-0.044498268	0.662012	-0.338266838	0.00086	-0.074979655	0.45823
SYMPK	0.046477194	0.747399	0.477215645	0.00087	0.537582155	0.00018
ZNF263	-0.158751375	0.092992	-0.313452258	0.00087	-0.202076644	0.03266
C4orf3	-0.107313126	0.274102	-0.325800442	0.00089	-0.387195175	8.03E-05
MYC	0.476845822	0.057456	-0.835952709	0.00089	0.140667313	0.57529
DNAH2	0.256585073	0.476318	1.151973766	0.00089	0.684392778	0.05236
UBAC2	0.077281143	0.419158	-0.318504622	0.00091	-0.097168566	0.31016
RCCD1	-0.265077948	0.13472	-0.578388381	0.00091	-0.326859042	0.06077
EEF1B2	-0.125621537	0.300069	-0.40141552	0.00093	-0.304896058	0.01191
CCDC149	-0.079847568	0.717757	-0.7294811	0.00093	-0.200765568	0.36039
MEIS2	-0.02865775	0.850901	0.503285391	0.00093	-0.173423045	0.25528
RPP25L	0.047266932	0.76907	-0.550178083	0.00093	-0.106790867	0.50839
FBXO38	-0.047657384	0.729996	0.453637802	0.00095	0.010681114	0.93822
EIF4A2	-0.145108617	0.107498	-0.297464482	0.00097	-0.401812784	8.41E-06
ITGB4	-0.169116603	0.522	0.869454647	0.00098	0.652284677	0.01339
ZNF485	-0.33858094	0.082153	-0.640147129	0.00099	-0.186709922	0.3305
TMC6	-0.073310532	0.650839	-0.5342153	0.00099	0.056158422	0.72827
CCDC137	-0.161239127	0.336454	-0.551841144	0.00102	-0.420104678	0.01243
GJB1	-0.103165365	0.654377	-0.758918016	0.00102	-0.300056843	0.19311
ZFYVE21	-0.225821054	0.052275	-0.379186851	0.00104	-0.134836062	0.24171
SLC39A5	-0.107253316	0.541384	-0.576285601	0.00105	-0.228253327	0.19362
EPHB3	0.279877551	0.327039	0.926122843	0.00106	0.555279101	0.05131
COL8A1	0.730468533	0.089326	1.395934412	0.00107	0.721598824	0.09297
GLYR1	0.126048811	0.143496	0.2802125	0.00107	0.221701808	0.00994
MUL1	-0.006600043	0.959471	-0.426747128	0.00107	-0.245712131	0.05939
SMUG1	-0.059005555	0.58222	-0.35160007	0.00107	-0.101906769	0.33609
TMEM170A	-0.169028158	0.08385	-0.318931663	0.00107	-0.103741767	0.28742
PPIAP22	-1.749359002	0.194451	-4.427612715	0.00109	-1.777813757	0.18726
FKBP9L	0.828003315	0.065634	-1.485805647	0.00109	0.363274133	0.41974
PARP1P1	0.479911417	0.146324	1.012257529	0.0011	0.733894495	0.0216
TMED4	0.044993031	0.570039	-0.258850915	0.0011	-0.116867919	0.14048
BOC	0.889693949	0.058102	1.528497054	0.00111	0.033201108	0.94377
GLTPD1	-0.050330461	0.775568	-0.581726366	0.00112	-0.307957555	0.08259

RNMTL1	-0.121133022	0.363738	-0.436357252	0.00112	-0.252329911	0.05908
FILIP1	0.006353799	0.984552	1.039761569	0.00112	0.003431825	0.99165
C2orf72	-0.27824545	0.293981	-0.862488199	0.00114	-0.120953045	0.64754
HIGD2A	0.185246321	0.073334	-0.340031984	0.00114	-0.070313313	0.4989
PEG10	0.093997762	0.668198	0.71205055	0.00115	-0.005074559	0.98157
MAPK8IP2	0.245486123	0.550161	1.20602381	0.00116	0.890884451	0.01987
GBP1	0.529649678	0.088005	1.005826658	0.00117	-0.197666583	0.52527
NVL	-0.027222537	0.828861	0.400436831	0.00117	0.144925304	0.24338
RPL6	-0.202273247	0.076956	-0.370811768	0.00118	-0.498806418	1.30E-05
KIAA0232	-0.099231402	0.332759	-0.331931294	0.00119	-0.287315598	0.00506
TBC1D1	0.257503699	0.09678	0.500364961	0.00119	-0.02911275	0.85113
PURB	0.12269042	0.192605	0.302831772	0.0012	0.34609223	0.00022
TRMT2A	-0.04726407	0.705959	-0.405469206	0.0012	-0.118091667	0.34376
NENF	0.044972454	0.674003	-0.348116804	0.00121	-0.115402916	0.28104
COX17	-0.069410396	0.583777	-0.410181477	0.00122	-0.141850414	0.26139
RPL36	-0.118390522	0.260144	-0.339865216	0.00122	-0.392257188	0.00019
CACNA1B	0.456945003	0.584938	2.325078531	0.00123	1.144539757	0.13709
HVCN1	0.198558976	0.676789	1.440501831	0.00123	0.330001541	0.48343
ADM5	0.021484151	0.948996	-1.145626526	0.00123	-0.295283364	0.38373
PLEKHH3	-0.235626186	0.314987	-0.753770789	0.00123	-0.278100517	0.23168
LDLRAD3	0.375365666	0.147039	0.822220759	0.00124	0.231801329	0.37068
SBF2	-0.182343036	0.405882	-0.707589857	0.00124	-0.087309806	0.68967
FCGRT	0.099829397	0.487991	-0.465159082	0.00125	-0.039322392	0.78474
SHC4	-0.302056379	0.264854	0.841379985	0.00125	0.537373704	0.04188
RIMS4	-0.648272766	0.378882	-2.627328828	0.00125	-0.983182223	0.19005
DHX29	0.188505113	0.222221	0.495967038	0.00126	0.270360375	0.07943
CECR1	0.063386978	0.862101	-1.202377501	0.00126	0.143830106	0.69318
FKBPL	-0.274775492	0.128288	-0.583184005	0.00127	-0.404318267	0.02513
P11-152F13	-1.234107071	0.411014	-5.383028579	0.00128	-1.530898925	0.30836
CCNT2	0.095906526	0.261817	0.272023096	0.00131	0.112180317	0.18779
ORM1	0.135053072	0.729921	1.250870158	0.00132	0.368171905	0.34582
RPLP0	0.091933144	0.413325	-0.361026139	0.00132	-0.08818342	0.43264
ELF1	-0.234301931	0.062859	-0.403534645	0.00132	-0.115907903	0.35551
PPP1R10	0.203991642	0.274983	0.599116395	0.00133	-0.026073727	0.88904
UQCRH	-0.107077046	0.25217	-0.29952079	0.00134	-0.239398659	0.01047
LNX2	-0.274255655	0.077291	-0.497584875	0.00134	-0.008309156	0.95717
RPL15	0.075564115	0.511829	-0.369133925	0.00135	-0.170887175	0.13797
PGP	-0.248699376	0.065782	-0.432360515	0.00136	-0.1753161	0.19273
PDE12	0.027844989	0.82088	-0.393506894	0.00137	0.127358361	0.29893
ATP6V1F	-0.00700028	0.944631	-0.323029036	0.00137	-0.3394671	0.00085
NR1H3	-0.2402437	0.172616	-0.56532633	0.00139	-0.075396917	0.66787
PREP	-0.016806078	0.887903	-0.381974203	0.00138	-0.029541759	0.80393
FUT4	-0.03307108	0.849864	-0.559830242	0.00139	0.277040271	0.11156
ILF3	-0.00657726	0.954506	0.367818322	0.00139	0.169285784	0.14173
SPDYA	-0.174852706	0.644502	1.116838574	0.0014	0.463615896	0.19831
LEF1	0.183338149	0.753688	1.846885659	0.00141	-0.226810442	0.69952
GPR83	0.35567358	0.442179	1.378717192	0.00141	0.990036714	0.02498

ZNF696	-0.319042254	0.192827	-0.782445919	0.00141	-0.406470545	0.09504
SCRN2	-0.234538185	0.160063	-0.533543812	0.00142	-0.259949973	0.11929
CHSY1	0.080247961	0.634189	0.536074216	0.00142	-0.151811249	0.36863
ZNF711	0.235146908	0.138519	0.503169881	0.00143	-0.069904308	0.66051
ENO1	-0.024800545	0.838072	-0.386797651	0.00144	-0.330606828	0.00645
IFI44	0.766798406	0.077308	1.367294925	0.00145	0.149989549	0.73361
HTN3	0.16256266	0.551097	0.841039192	0.00146	0.70072048	0.00841
ADAM9	0.268732038	0.055211	0.445898854	0.00146	0.220445259	0.11571
DDT	0.132155612	0.394419	-0.502423511	0.00146	-0.107259614	0.49005
MOGS	0.006231717	0.964998	-0.452350235	0.00147	-0.127119943	0.37057
ECE1	0.456960732	0.057939	0.765076197	0.00149	0.417550534	0.08321
RPL21	-0.202882097	0.089579	-0.379619813	0.00149	-0.337167494	0.00478
PLOD1	0.111893838	0.401698	-0.424148235	0.00149	-0.294611351	0.02738
MGAT4A	-0.306468813	0.286257	-0.914153814	0.0015	-0.723112787	0.01203
C5orf42	0.247669593	0.278132	0.721980026	0.00151	0.380517124	0.09515
CTSD	0.23133499	0.119647	0.47089	0.00151	0.370168217	0.01267
UBE2J1	0.122684781	0.169866	0.28190378	0.00151	-0.059432275	0.50646
TMEM177	-0.271801844	0.133839	-0.575227564	0.00151	-0.140969747	0.43015
HLA-B	0.198416657	0.480756	-0.895117673	0.00152	-0.496560593	0.07823
ASB1	0.057281818	0.652458	-0.402063529	0.00152	-0.201748161	0.11226
MPV17	-0.019228072	0.897095	-0.473877771	0.00153	-0.15456999	0.29881
KIAA0226	0.170393696	0.272566	0.48347386	0.00155	0.584151428	0.00013
ZNF444	-0.139420034	0.427259	-0.557792236	0.00156	0.156754823	0.36154
BCAP31	-0.045406757	0.624061	-0.292768825	0.00157	-0.275514897	0.00304
RPL22	0.027856303	0.796058	-0.340821032	0.00157	-0.151192857	0.16076
ANKRD37	-0.202954759	0.705734	1.647682461	0.00158	1.312145188	0.01219
CORO2A	-0.077591353	0.691122	-0.617768371	0.00159	-0.129527335	0.50695
C10orf55	0.761168388	0.086617	1.329931524	0.0016	0.259862782	0.57071
DOK4	-0.290162296	0.087636	-0.536117484	0.0016	-0.480746185	0.0047
PSAP	0.011986287	0.926784	-0.41173909	0.0016	-0.144480007	0.26809
NME3	-0.090744279	0.69005	-0.731797341	0.00161	-0.247350135	0.27869
CCDC24	0.030318897	0.874723	-0.609837054	0.00162	-0.066559417	0.72853
MEIS1	0.293660289	0.23129	0.772423624	0.00162	0.067517372	0.78319
HMGCL	-0.211265398	0.073467	-0.371067768	0.00163	-0.134227416	0.25323
C6orf106	0.070557815	0.458821	-0.300246035	0.00164	-0.047808621	0.61538
RRC37A15	-0.613687657	0.168289	-1.526775484	0.00165	-0.503281221	0.24496
EIF3K	-0.030030437	0.723107	-0.266659968	0.00166	-0.314754915	0.00022
FAM13A	0.07202439	0.771373	0.778061828	0.00166	0.455490088	0.06581
MCFD2	-0.135591572	0.122215	-0.27572124	0.00166	-0.176539599	0.0442
STYXL1	-0.013102253	0.911639	-0.371909303	0.00165	-0.227543476	0.05597
KIF13B	-0.113951216	0.381769	-0.409966288	0.00166	0.263830383	0.04239
FGFR4	-0.153188992	0.444613	-0.631678246	0.00167	-0.016228045	0.93541
TRMT61A	0.054967264	0.693317	-0.44291642	0.00167	-0.072445183	0.60313
PTPN4	-0.179702451	0.160306	-0.400191596	0.00167	-0.158085463	0.21471
NEO1	-0.035065046	0.817261	0.475249985	0.00168	0.039545202	0.79439
HAUS4	-0.089231178	0.48592	-0.402539547	0.00168	-0.114216406	0.37154
AGPAT2	-0.198560762	0.365694	-0.689932684	0.00169	-0.275999112	0.20856

DDX10	-0.098667207	0.593258	0.575387742	0.0017	-0.036188865	0.84439
PWWP2B	-0.21764675	0.324445	-0.698109705	0.0017	0.247767287	0.25385
CSNK1G1	-0.135825028	0.169327	-0.309215116	0.00171	0.009324568	0.92444
NOP56	-0.139470793	0.267036	-0.394166293	0.00171	-0.330988389	0.00847
ESPN	-0.161525085	0.607427	-0.993566688	0.00172	-0.041096483	0.89598
FBXO42	-0.105904041	0.508164	-0.501575495	0.00172	-0.086285344	0.58822
RPL37A	-0.02145732	0.854198	-0.366062242	0.00172	-0.292270588	0.01233
NDRG1	0.344057055	0.242063	-0.921488167	0.00173	0.137519368	0.6401
PAPSS2	-0.252614442	0.315978	-0.791261005	0.00174	-0.434075107	0.08517
SCN5A	0.651748392	0.25496	1.752289291	0.00174	0.6198391	0.27795
ATP2B1	-0.00349188	0.979428	0.42296141	0.00175	-0.196226841	0.14747
DHDH	-0.059462091	0.905491	-1.90239024	0.00175	-0.393803285	0.44198
PPP1CA	0.020350952	0.803423	0.253282817	0.00176	-0.054156868	0.50709
C11orf30	0.212257737	0.14184	0.446055901	0.00179	0.46936305	0.00105
PTPRZ1	0.022537199	0.968347	1.694826164	0.00179	-0.193558341	0.72952
ZNF551	-0.180732996	0.192261	-0.432462947	0.00179	-0.055846351	0.68496
MANF	-0.212150522	0.206715	-0.524158705	0.0018	-0.315790378	0.06019
RPL12P38	-0.278646708	0.098157	0.515453611	0.0018	-0.196933373	0.24005
REEP4	-0.281341287	0.050104	-0.4402091	0.00181	-0.01676355	0.90379
STK35	0.041073544	0.552234	-0.215149213	0.00183	0.160311159	0.01906
C11orf54	-0.032744082	0.790183	-0.383863841	0.00183	0.048501074	0.69266
WNT9A	0.243367613	0.559424	1.182312005	0.00183	0.857701913	0.02816
PTPLAD1	-0.105721235	0.295621	0.313708591	0.00184	-0.114240831	0.25808
NOS1	0.135083964	0.670923	0.960739545	0.00184	0.593501776	0.05513
CHMP1A	0.173649879	0.080131	-0.309490807	0.00186	-0.101092772	0.30976
OTUD4	0.189444592	0.100619	0.358032784	0.00186	0.204700052	0.07609
GEMIN7	-0.098823199	0.472216	-0.429079282	0.00187	-0.04839098	0.72266
ADORA2B	-0.089195546	0.715228	-0.772293641	0.00187	-0.085784417	0.72432
P4HA2	-0.186575454	0.245979	-0.500797276	0.00187	-0.349043872	0.03007
YTHDF1	0.110039187	0.2339	-0.288135722	0.00188	0.043232243	0.63964
TBRG4	0.019817491	0.856104	-0.340909088	0.00188	0.066241484	0.54365
QKI	0.18665646	0.161552	0.413546188	0.00189	-0.022050184	0.86873
MMP19	-0.019514844	0.949364	-0.973383882	0.0019	-0.52144295	0.09372
ABI1	0.084808186	0.304208	0.253174885	0.00191	-0.046204265	0.57514
NARF	0.002635537	0.979503	-0.317661888	0.00191	-0.033117362	0.74577
ZFHX2	-0.305627105	0.590937	-1.770960516	0.00191	-0.629330787	0.25581
PRRG3	0.286463591	0.342738	0.905089146	0.00192	0.890898785	0.00237
UPK3B	0.115112286	0.890453	-2.833455423	0.00195	0.62561228	0.45254
NARS	-0.080768556	0.51247	0.380418897	0.00198	-0.318652503	0.00978
ZNF558	-0.087311593	0.466029	-0.369442685	0.00199	-0.231770752	0.05239
PLEKHG6	0.016073027	0.941977	-0.68329146	0.00199	-0.163219151	0.45896
FERP1	0.79468657	0.251391	-2.540312686	0.002	1.419844366	0.03816
P11-137H2	-0.230517521	0.058538	-0.374497379	0.002	-0.294963276	0.01522
ADCK2	-0.201613582	0.299175	-0.605674568	0.00201	0.05508149	0.77618
ESPNP	0.417826055	0.464341	-1.951096081	0.00201	0.201796863	0.72032
FHOD3	0.482004066	0.412265	1.66409148	0.00201	-0.456198378	0.4556
FAM19A2	0.328239668	0.364453	1.054282819	0.00202	0.862358119	0.01497

AFMID	-0.021636354	0.867823	-0.401750275	0.00203	-0.157574642	0.22558
CHST15	0.372949637	0.220696	0.935586229	0.00203	-0.231658506	0.44821
NTRK3	0.00338621	0.994091	1.369138568	0.00203	0.243917666	0.5865
INRNPU-AS	-0.437624996	0.06144	0.664884404	0.00203	0.338434398	0.12436
LRP6	0.079560201	0.534124	0.392388182	0.00204	0.219126766	0.08602
TSFM	-0.145783629	0.197968	-0.343485016	0.00205	-0.38183465	0.00074
GPR125	0.094283619	0.42957	0.366428274	0.00207	0.143469504	0.22926
HERC5	0.275820043	0.452253	1.087038057	0.00207	0.535654549	0.13858
TECRL	0.901967229	0.060843	1.46609514	0.00207	0.949177508	0.04803
FAF2	-0.020672621	0.784682	-0.232770795	0.00208	-0.056149403	0.45805
PNCK	0.344783406	0.333878	-1.104844782	0.00208	0.011074841	0.97525
PS10-NUDT	0.127356583	0.474716	0.537687679	0.00208	-0.420588363	0.02029
ABCA12	0.280236269	0.658296	1.904181288	0.0021	-0.049176899	0.9387
TMPRSS5	0.184872745	0.539586	-0.93657018	0.0021	0.546626133	0.06891
EMILIN2	-0.301903294	0.25628	-0.822295381	0.00211	-0.341485619	0.20038
B4GALNT3	0.180000482	0.430175	0.695797107	0.00211	0.435190309	0.05522
NCOA2	-0.016344795	0.866866	0.298085749	0.00212	0.164707247	0.09014
CDC42SE2	-0.025550878	0.787635	-0.291266407	0.00214	-0.187744987	0.04803
MAGEH1	-0.208457993	0.148354	-0.442253361	0.00214	-0.496478101	0.00061
SCYL2	-0.019708483	0.860238	0.342926047	0.00214	-0.062387161	0.5773
CDKN1C	-0.283720908	0.134814	-0.582615631	0.00216	-0.39084913	0.03942
RNF144B	-0.057414108	0.890804	1.265998762	0.00216	0.241040351	0.56234
KCNMA1	1.000804113	0.076167	1.711721043	0.00216	1.154673439	0.03971
NOSIP	0.080047857	0.467105	-0.34125921	0.00216	-0.075095927	0.49684
SHE	0.578228654	0.177271	1.249458395	0.00217	0.97042619	0.01814
FAM219B	-0.17575847	0.187866	-0.40736137	0.00217	-0.133250006	0.3157
RNF123	-0.372520887	0.061188	-0.609603675	0.0022	-0.330002643	0.09672
NGF	1.554852795	0.100607	2.790194939	0.00222	-0.023011774	0.98223
FPGT	-0.28323521	0.067167	-0.466373292	0.00226	-0.354583899	0.02011
LANCL2	0.013054868	0.917862	0.381994566	0.00227	-0.034966971	0.78237
LRP4	0.037832535	0.876329	0.739818162	0.00227	0.480387697	0.04767
MUC15	0.173222008	0.681613	1.253395552	0.00228	0.12299431	0.76992
LIMA1	-0.018486312	0.836191	-0.272653996	0.00229	-0.000261243	0.99767
TLE3	0.263750859	0.290168	0.758433632	0.00229	0.300107482	0.22793
TD-3074O7.	0.021369327	0.993634	-8.72812875	0.00231	-1.360221039	0.6118
DENND2D	0.152358604	0.537861	-0.7548371	0.00232	-0.010524258	0.96607
GORASP1	-0.044024764	0.671073	-0.315192631	0.00232	-0.166380833	0.10675
PDXDC1	-0.102133943	0.283405	-0.289935414	0.00231	-0.047843298	0.61497
M6PR	-0.083327311	0.499778	-0.376608228	0.00233	-0.379428282	0.00215
FBXW8	-0.137493705	0.335634	0.427235349	0.00235	0.347207924	0.01395
UBA2	0.074513752	0.426846	0.284711391	0.00235	-0.041914212	0.65502
USP39	-0.09269931	0.561667	0.482426254	0.00238	0.273834037	0.0857
GBP5	0.338785375	0.266985	0.913159054	0.00239	0.432127915	0.15478
PTCHD4	-0.346357351	0.319318	-1.064464167	0.00239	-1.177565116	0.00085
ENTPD6	0.055573255	0.603565	-0.326162243	0.0024	-0.103832717	0.33222
STUB1	0.255744378	0.071344	-0.436407069	0.0024	0.019147999	0.89304
BCAS1	0.002489943	0.991561	0.7131815	0.00241	0.35781052	0.12816

EPB41L4A	-0.026403281	0.909915	0.693123963	0.00244	0.526872254	0.0226
CCDC183	0.010211103	0.965324	-0.715334552	0.00244	0.096606554	0.68036
MED27	0.20659761	0.058501	0.319014076	0.00246	0.130609281	0.22952
PBX3	0.237272919	0.289101	0.675487267	0.00246	0.314742711	0.15933
NME6	-0.102540356	0.353292	-0.330999085	0.00248	0.094895594	0.37657
MPI	-0.03692977	0.764553	-0.372383616	0.00251	-0.210614394	0.08741
ESYT2	-0.090197279	0.397488	-0.321793323	0.00254	-0.176617323	0.09749
LRRC32	0.269109737	0.314747	0.797101354	0.00254	0.567268144	0.03286
APRT	-0.073440078	0.649243	-0.489021716	0.00254	-0.220798148	0.17173
C15orf39	0.209885002	0.211013	-0.509722275	0.00254	0.477166232	0.0043
ENO2	0.022011332	0.948867	-1.037723681	0.00254	-0.108668482	0.75155
TRIM62	0.070586291	0.763635	0.69673121	0.00255	0.260606748	0.26383
HSDL2	-0.111490196	0.384896	-0.386745535	0.00256	-0.196083291	0.12661
HSPA1L	8.98E-05	0.999897	-2.120505648	0.00256	-0.126285672	0.85547
TIGD5	-0.280637311	0.102357	-0.518361314	0.00256	-0.300999664	0.07605
CAPN15	-0.16986763	0.223775	-0.422600365	0.00258	-0.167221849	0.22961
FAM180B	0.422433237	0.449787	1.563688803	0.0026	1.16541594	0.02804
GPM6B	0.280859906	0.231119	0.687600146	0.0026	-0.054259825	0.81635
HNRNPC	0.00270114	0.977535	0.288616023	0.0026	-0.345157753	0.00032
MED10	-0.049352818	0.725095	-0.424709505	0.0026	-0.222196582	0.11386
PALM3	-0.48684147	0.119034	-0.959356892	0.00261	-0.235606688	0.44122
FAM179A	0.082930837	0.698636	0.628255734	0.00261	0.654302523	0.00172
ENKD1	0.099444952	0.718841	-0.837930234	0.00262	-0.181155261	0.51145
GSPT2	-0.022097727	0.846614	-0.343968076	0.00264	-0.225781952	0.04859
CLDN2	-0.117462644	0.773573	-1.23402712	0.00265	-0.601730544	0.14129
FIG4	-0.339310642	0.075215	-0.575086586	0.00265	-0.424607964	0.02625
RNF165	0.318346	0.490845	1.368868994	0.00264	0.232224621	0.61434
LIN28A	-0.748507006	0.188843	1.657217226	0.00265	0.190413334	0.73308
NR2F6	-0.269339359	0.065806	-0.439380283	0.00266	0.033268943	0.81961
RPL35	-0.027775912	0.80705	-0.341658349	0.00267	-0.309710552	0.00649
RPS8	-0.104410713	0.36186	-0.344007002	0.00267	-0.342009662	0.00282
RINL	-0.00513025	0.980223	-0.633412108	0.00268	0.001776918	0.99313
ADNP2	0.144243586	0.249837	0.376043132	0.00269	0.12756579	0.30907
COMMD5	0.021987236	0.890463	-0.483290975	0.0027	-0.222223278	0.16595
DDOST	0.113188913	0.314553	-0.337879555	0.0027	-0.065011669	0.56361
DCBLD2	-0.083323094	0.570775	0.439564483	0.00271	0.222491003	0.13024
ARSE	-0.17978834	0.365044	-0.597019795	0.00272	-0.367685617	0.06407
FAM103A1	-0.131267506	0.250633	-0.341188876	0.00273	-0.191926342	0.09196
PGRMC2	-0.304817187	0.079996	-0.520695841	0.00275	0.157168328	0.36413
MRPL38	-0.007877186	0.948487	-0.365074504	0.00276	-0.272217875	0.02633
RPS7	0.145354657	0.2393	-0.369793249	0.00277	-0.103009996	0.4044
ATP5G3	-0.001803187	0.986452	-0.31753731	0.00277	-0.198813958	0.06129
IARS2	-0.058465383	0.537958	-0.283601301	0.00278	-0.129327014	0.17259
NGFR	0.678755385	0.324695	2.039024378	0.00278	-0.433343655	0.53457
FOXC1	1.17228888	0.10797	2.171528146	0.00279	0.087217943	0.90546
GLTPD2	0.228704082	0.595882	-1.359713149	0.0028	-1.162566166	0.01042
MINK1	0.026856687	0.846188	-0.41428188	0.0028	0.060199135	0.6634

RPL10	0.018397314	0.859916	-0.311589105	0.0028	-0.254063078	0.01483
VPS4A	-0.12889947	0.107386	-0.237585879	0.0028	-0.067247363	0.3969
ACER3	0.086271612	0.601509	0.491450779	0.00282	0.255724387	0.12052
RBMS1	0.094149572	0.289281	0.264562653	0.00282	-0.066094377	0.4571
NDST1	0.311316869	0.051314	0.475613612	0.00284	0.222203013	0.16367
KLHL12	-0.13319322	0.187239	-0.298996927	0.00285	-0.326015186	0.0012
NRP2	0.342242222	0.15227	0.710977558	0.00287	-0.157718599	0.51022
GLI2	0.427194088	0.087963	0.742200445	0.00288	0.768147209	0.00206
TMEM126B	-0.172243387	0.150056	0.348437909	0.0029	-0.114236025	0.33854
ZNF609	0.161363839	0.118264	0.305669051	0.0029	0.30415043	0.0031
1-Sep	-0.11146853	0.776192	1.076032143	0.0029	0.701885549	0.05197
C12orf65	-0.060444904	0.514324	0.266949134	0.00291	0.22296449	0.01335
NLGN1	0.317881545	0.257771	0.832254824	0.00291	0.475657869	0.08992
ACTB	0.126646063	0.133314	0.251064695	0.00292	0.039488451	0.63974
CTB-89H12.4	-0.112935835	0.507191	-0.507250604	0.00294	0.047218559	0.7809
HHAT	-0.235328598	0.334191	-0.724783075	0.00295	-0.774662949	0.00199
LRRC3	0.072465006	0.6277	-0.452262746	0.00295	-0.014307763	0.92347
ZNF846	-0.302712469	0.063544	-0.479842273	0.00296	0.023810698	0.88148
BNC2	0.445511229	0.17731	0.979471224	0.00296	0.027511278	0.93364
EFCAB14	0.131156802	0.348507	0.415002181	0.00298	0.495421322	0.00039
TDRD7	0.022008602	0.886057	-0.458069404	0.00298	0.060003256	0.69489
FAM211A	0.025812857	0.836494	-0.371329981	0.003	-0.063075753	0.61401
ADRBK1	0.185793441	0.225681	-0.456362798	0.00305	-0.057678937	0.707
DLG1	0.046510928	0.631266	0.285523814	0.00305	0.180785743	0.06137
SALL2	0.925963038	0.067752	1.482430961	0.00305	0.744212211	0.14547
JAGN1	-0.002671698	0.979549	-0.309042471	0.00307	0.000319644	0.99754
RASSF3	0.054049281	0.684486	-0.394074067	0.00307	0.068780192	0.6044
RBCK1	0.068810165	0.605091	-0.396278757	0.00308	-0.096199323	0.47012
GFPT1	-0.074526666	0.665542	-0.510001804	0.0031	-0.003580135	0.98343
PPM1K	0.182392898	0.360749	0.581868758	0.00312	0.117007384	0.55549
GLUD2	-0.232599129	0.392464	-0.81022982	0.00312	-0.032860395	0.90316
CCSER1	0.007561102	0.966542	-0.533763837	0.00316	0.10831483	0.54731
IL17RD	0.423398105	0.29556	1.189695505	0.00316	0.01400042	0.97241
NSUN5P1	-0.26896259	0.211258	-0.633344733	0.00317	0.030040319	0.88837
UNC5D	0.328062963	0.384963	1.082176846	0.00318	0.102859283	0.78541
HCFC1	0.245619423	0.194404	0.556733378	0.0032	0.705872613	0.00018
MRPL10	-0.011371893	0.931196	-0.389232742	0.0032	-0.299493245	0.0233
EPOR	-0.038080291	0.817577	-0.488499219	0.00326	0.13557558	0.4026
CD34	0.953669275	0.210002	2.181238931	0.00327	-0.21786618	0.77379
PBXIP1	-0.121848552	0.469413	-0.495738403	0.00327	-0.519512596	0.0021
SH3D21	-0.308369632	0.168381	-0.658345726	0.00329	-0.369816082	0.09778
GPR35	-0.061199355	0.785937	-0.664351605	0.00331	0.193824198	0.38786
NANP	-0.159354657	0.266408	-0.420772713	0.00332	-0.149949514	0.29347
CYB561D2	0.048385248	0.787664	-0.537944977	0.00333	-0.038373342	0.83085
RPL34	-0.095318927	0.404334	-0.335478218	0.00333	-0.292992242	0.01039
NSMCE2	-0.136302635	0.243313	0.327275486	0.00335	0.02733084	0.81297
C19orf24	0.109456813	0.543349	-0.535712654	0.00337	-0.328098186	0.07091

RPS6KB2	-0.027084321	0.777107	-0.278011686	0.00337	-0.082069084	0.38899
C1orf174	-0.12151017	0.182623	-0.264685284	0.00338	-0.157234566	0.08261
POLD4	0.021156887	0.879017	-0.40887242	0.00338	0.033329985	0.80995
CUEDC2	0.040511936	0.76204	-0.392793991	0.00339	-0.426602023	0.00164
FN3KRP	-0.135567243	0.188608	-0.301011439	0.0034	-0.267700734	0.0098
PHB2	0.024074294	0.785589	-0.258929208	0.00343	-0.224486234	0.01129
KEAP1	-0.088234241	0.590116	-0.479025273	0.00343	-0.131369515	0.42202
EEF1A1P24	-0.401068326	0.417449	-1.646104359	0.00345	-0.69449344	0.1678
LETM2	0.376355065	0.141677	0.73683857	0.00345	0.065843424	0.79652
ACAP3	-0.149515488	0.432632	-0.556677599	0.00347	-0.228091937	0.23116
CHCHD3	-0.10718417	0.295352	0.29529341	0.00347	-0.146305031	0.15271
KRBOX4	-0.195465966	0.21423	-0.460623693	0.00347	-0.290538118	0.06534
TP63	-0.754132348	0.243014	1.803347403	0.00347	0.504121163	0.42706
CRELD1	-0.104573851	0.563642	-0.531462202	0.00349	-0.259706266	0.15225
SLC12A8	-0.438222279	0.072473	-0.711487241	0.0035	0.11928219	0.6204
PCDHB3	0.421527324	0.062581	0.644112859	0.00352	0.484126863	0.03066
HMGCS2	-0.032033813	0.930532	-1.073258413	0.00352	-0.106042377	0.7729
TMEM86A	0.514799483	0.123639	0.949161013	0.00353	0.311137952	0.3555
EEF1A1P22	-0.19091398	0.491066	-0.843163583	0.00354	-0.395528927	0.15672
SEC23A	-0.115700831	0.295319	-0.322308685	0.00354	-0.367426985	0.0009
TRABD2A	-0.173207474	0.516715	-0.779268783	0.00355	-0.114596939	0.6653
RPS27A	-0.177030874	0.159493	-0.366470828	0.00358	-0.358844543	0.00435
SMCHD1	0.009200083	0.92654	0.290092992	0.0036	0.067141125	0.50082
PIP4K2C	0.034450055	0.83298	0.473003436	0.00361	0.314635246	0.05275
TMEM130	0.57558026	0.07037	0.915555766	0.00361	0.524646768	0.09648
RPL35A	0.05676327	0.62024	-0.333422735	0.00362	-0.163624706	0.15331
EPB41	-0.003597282	0.982843	0.485325111	0.00363	0.333124862	0.04606
DCUN1D5	-0.144567851	0.18678	-0.315130917	0.00365	-0.377294658	0.00059
MBTPS1	0.137625486	0.058401	0.209919076	0.00368	0.026217193	0.71837
SNRPD2	-0.147092189	0.231554	-0.356868044	0.00368	-0.491623661	6.75E-05
SOX11	0.071446691	0.750762	0.650787211	0.00369	-0.072604407	0.74689
P11-682B13	0.950971864	0.277061	2.266996793	0.00369	1.347607871	0.10563
BLMH	0.036619337	0.813056	0.444037427	0.00371	-0.14489189	0.34908
CA9	0.219287163	0.556037	-1.082339717	0.00371	-0.035218504	0.92467
P11-266K4	0.212629189	0.742795	1.736557163	0.00371	0.992740569	0.1063
TIGD4	-0.127235287	0.69382	-0.992185438	0.00375	-0.223462947	0.48786
KLF16	0.011868377	0.959016	-0.673204532	0.00378	-0.002664324	0.99073
MAN1A2	0.084036254	0.439546	0.313930811	0.00379	0.044808975	0.67996
FLT4	0.709746595	0.198671	1.501457686	0.00382	0.51035635	0.3379
FAM210A	-0.023618662	0.877223	-0.44085976	0.00385	-0.226882715	0.13687
ZNF789	-0.200601231	0.196143	0.432715571	0.00385	0.587154641	8.52E-05
SLC25A39	-0.056584034	0.6345	-0.343344216	0.00391	-0.182909093	0.12434
MKNK2	0.203017657	0.317368	-0.586695252	0.00392	-0.007103961	0.9721
C2orf47	-0.041932038	0.807517	-0.498841626	0.00392	-0.404015821	0.01956
NAALAD2	0.181925303	0.590238	0.967443972	0.00393	0.022819041	0.94614
P11-83M16	0.070810956	0.830448	0.891456771	0.00394	-0.0048094	0.98834
PARVB	-0.171238981	0.598984	-0.942043384	0.00395	-0.611404484	0.061

PBX1	-0.011447533	0.940587	0.439437189	0.00398	0.300009999	0.0498
BCAT2	-0.153993675	0.336388	-0.461401366	0.004	-0.209051249	0.19094
IGFBP2	0.19369703	0.27437	-0.510235756	0.00399	-0.004974871	0.9776
11-Sep	0.161078511	0.16816	0.336159577	0.004	-0.158241188	0.1759
SH2D3C	1.251190465	0.078314	1.948830984	0.00399	0.683615425	0.32549
SHISA3	0.331071366	0.472108	1.31886408	0.00399	-0.023006422	0.96021
MCAM	0.413779097	0.078291	0.667030292	0.00403	0.247437566	0.29063
C7orf55	-0.449590196	0.190504	-0.973140709	0.00405	-0.007768799	0.98144
CARD10	-0.310219564	0.064125	-0.482697429	0.00408	-0.113307968	0.49748
KRIT1	-0.114996058	0.505918	0.492904148	0.00409	0.063314198	0.71358
ANAPC2	-0.231606438	0.282259	-0.618839915	0.0041	-0.161895142	0.45265
TOMM20	-0.10251753	0.314643	-0.292408515	0.0041	-0.177944313	0.08088
C19orf10	-0.087334193	0.502199	-0.373643455	0.00411	-0.284583845	0.02898
DNAJC7	-0.049564096	0.620313	0.284592424	0.00412	-0.120606131	0.2277
DUS1L	0.130966326	0.227384	-0.312268935	0.00412	-0.025215578	0.81696
DNAJC16	0.445214685	0.073881	-0.717717205	0.00414	-0.12635885	0.61172
SREBF1	0.113709171	0.380904	0.370381419	0.00413	0.302063018	0.01942
SFT2D1	0.001610367	0.986795	-0.278622314	0.00416	-0.035315116	0.71511
RUNDC3B	0.880393927	0.173943	1.795340925	0.00417	1.011812236	0.11574
EIF4BP7	-0.453452491	0.058645	-0.685562943	0.00419	-0.668763922	0.00544
PARP4	0.102957144	0.167185	-0.213700101	0.00419	0.059023396	0.4283
ZNF317	-0.112620324	0.31917	-0.322917978	0.00419	-0.023318665	0.83601
P11-736N17	1.561377834	0.051173	2.281718432	0.0042	2.047791091	0.01088
EEF1A1	-0.182331598	0.122966	-0.338263658	0.00422	-0.38977554	0.00098
TMEM216	-0.042834262	0.782632	-0.446735496	0.00422	-0.441610067	0.00496
PLEKHA3	0.164803988	0.236214	0.396104027	0.00422	0.243267597	0.07817
LDHB	-0.188237069	0.102869	-0.330061062	0.00422	-0.37002768	0.00135
ZNF524	-0.18727936	0.392146	-0.631456972	0.00423	-0.112134324	0.60413
DPYSL4	0.460491233	0.134374	0.87284897	0.00424	0.22000693	0.47695
VWC2	1.108997092	0.180873	2.340773327	0.00425	0.093374732	0.91169
CDH11	0.17971303	0.406915	0.619129225	0.00425	-0.16320973	0.45144
AC002310.1	1.600779598	0.147648	2.895594675	0.00428	3.269943695	0.00116
DNASE1L2	-0.256177899	0.635848	-1.649624678	0.00429	-0.503109509	0.34212
NEDD4	0.03403925	0.852859	0.523578824	0.00429	0.672379933	0.00025
CD320	-0.036916008	0.862157	-0.61018774	0.0043	-0.195975769	0.35773
FGF12	-0.625646676	0.388709	1.960814327	0.00431	-0.599125696	0.4086
GRSF1	-0.153623598	0.06012	-0.232402168	0.00431	-0.025843869	0.75074
LDOC1L	-0.020260009	0.826575	-0.263773434	0.00431	-0.103739306	0.2615
TAX1BP1	0.034543864	0.725771	0.279936559	0.00432	-0.047796338	0.6275
PIEZO2	0.225422675	0.157231	0.45456311	0.00432	0.157856585	0.32253
PHACTR3	-0.817735452	0.076115	-1.321749191	0.00434	-1.998186403	2.37E-05
LONP2	-0.145334837	0.081162	-0.236732851	0.00434	-0.027823849	0.73756
PCDHB13	0.31191547	0.207067	0.675927554	0.00435	0.355361464	0.14533
SYT14	1.359015575	0.070438	2.083957262	0.00436	1.321949912	0.0749
TMBIM1	0.224920743	0.121892	0.413741993	0.0044	0.138871301	0.33945
LCOR	-0.124541322	0.314367	-0.352388466	0.00441	-6.58E-05	0.99958
SH3BGRL	0.053825594	0.552433	0.256282208	0.00441	-0.162819616	0.07218

ZNHIT2	-0.283118363	0.328946	-0.836699787	0.00441	-0.187142766	0.51363
TAF5L	0.074516592	0.399221	-0.252141839	0.00443	-0.097566839	0.27045
TEAD2	0.21770482	0.201898	0.483674866	0.00443	-0.12754162	0.45535
ZBTB42	-0.095898127	0.540323	-0.447689708	0.00443	0.164516339	0.28189
IKBIP	-0.269726183	0.070044	-0.42224454	0.00444	-0.625041783	2.95E-05
TM7SF2	-0.013272774	0.926057	-0.406564784	0.00446	-0.131183068	0.35875
MEF2A	0.103046154	0.356666	0.316096004	0.0045	0.308959384	0.00556
PNP	0.075123135	0.535038	-0.344684203	0.0045	-0.149614313	0.21716
RPL18	-0.063437622	0.580939	-0.326423885	0.0045	-0.295897081	0.01005
SPDYE3	0.084775554	0.641892	0.495467251	0.0045	0.565073029	0.00125
TMEM184A	-0.325647257	0.073548	-0.51573588	0.00451	-0.067871894	0.70789
SCHIP1	0.326497334	0.3846	1.050903255	0.00451	-0.239086006	0.52194
SPIRE2	0.082357729	0.603682	-0.453121398	0.00453	0.351634334	0.02563
PPARA	-0.054789053	0.753687	-0.496533019	0.00454	0.076534139	0.66074
CDIP1	0.049753547	0.829157	-0.660074804	0.00456	-0.336229334	0.14753
OR52K3P	-0.129076812	0.819147	-1.625548623	0.00457	-1.137599673	0.04597
PLA2R1	-0.082039173	0.799858	-0.921746236	0.00458	-0.235986788	0.46671
ETHE1	0.09227696	0.638947	-0.559725707	0.0046	-0.361453783	0.06722
COMMD2	0.075289082	0.459817	0.285873731	0.00462	-0.046057506	0.65126
ZNHIT1	-0.001628537	0.985071	-0.245422875	0.00462	-0.205900499	0.01774
MIEF1	-0.184851843	0.097815	-0.315841413	0.00462	-0.114925832	0.30142
ALG2	-0.057390843	0.636542	-0.343969882	0.00463	-0.164423037	0.17567
GAN	-0.14896125	0.159121	-0.298786846	0.00463	0.017037629	0.87162
DDX31	-0.18521413	0.186911	-0.396098673	0.00465	-0.183528385	0.18952
CLMP	0.20643652	0.422649	0.725906406	0.00469	0.097404803	0.7051
TFF1	0.099338449	0.658738	0.635640468	0.00471	0.204614122	0.36296
HNRNPR	-0.081075437	0.349862	0.244066088	0.00471	-0.303312801	0.00047
OLFM1	0.41580003	0.37158	1.230079391	0.00472	0.968744177	0.03132
PLIN5	0.490118186	0.166708	0.97694479	0.00473	1.25419163	0.00025
EIF5	0.056989887	0.725621	0.45832132	0.00475	-0.066227147	0.68341
DCAF16	-0.000523301	0.995636	0.267414024	0.00476	0.049976806	0.59982
BCKDK	0.189076429	0.225643	-0.440979222	0.00478	-0.102468065	0.51211
METTL15	-0.096604568	0.400151	-0.327500957	0.00479	-0.050052956	0.66168
TMEM150B	-0.575184731	0.337425	-1.745447834	0.00479	-1.836025374	0.00284
DPP7	-0.094945149	0.72361	-0.758807403	0.00481	-0.460251527	0.08708
PTGER2	-0.185069462	0.467624	-0.720587297	0.00481	0.204718546	0.41951
TXNDC12	0.012291778	0.919223	-0.340989346	0.00481	-0.229912025	0.05814
CECR5	-0.088091509	0.524289	-0.389784838	0.00482	-0.316108156	0.02272
AGPAT5	-0.154077525	0.300155	-0.419072107	0.00483	-0.043007139	0.77225
CSNK2A1	-0.017648716	0.871	-0.306207915	0.00484	0.058295886	0.5915
MAX	0.153584518	0.189482	0.327214535	0.00484	-0.200481168	0.08846
TMEM59L	1.365849992	0.088657	2.222756314	0.00484	0.445192353	0.60057
FMN1	-0.28768975	0.507554	1.210984634	0.00486	0.607977498	0.15924
AVPI1	0.052353262	0.85385	-0.813955111	0.00487	-0.426420631	0.13723
AFF2	0.381138785	0.408892	1.290049108	0.00488	0.129003432	0.78029
GBP6	-0.377368902	0.395846	1.137559192	0.00493	0.664830825	0.10798
GARS	0.260456773	0.073029	0.407438603	0.00498	0.144415058	0.32033

FAM124B	0.11277857	0.887917	2.028583308	0.00498	0.691172277	0.36534
ACBD4	-0.145889877	0.488491	-0.598682278	0.005	-0.20104391	0.33868
RPL18A	0.168520306	0.261615	-0.421691629	0.00499	-0.176353671	0.24028
P11-512H23	-0.728715726	0.139594	-1.463629084	0.00501	-0.748031511	0.12438
USP48	-0.166390588	0.170075	-0.33931362	0.00504	0.014383564	0.90524
SNRPN	-0.010111051	0.916332	-0.269709236	0.00505	-0.378451801	8.99E-05
CNIH1	-0.063263596	0.537214	-0.287545048	0.00505	-0.203762476	0.04699
SYNE2	0.124262132	0.518171	0.538425158	0.00507	0.582654361	0.00243
UBA1	0.147432756	0.069908	0.227472544	0.00506	0.16976434	0.03673
PGM5	0.339671857	0.367583	1.049479421	0.00512	0.484381337	0.1994
PADI2	-0.679770749	0.175402	-1.41023289	0.00513	-1.30204937	0.0098
3-Mar	0.123638409	0.587003	0.623346782	0.00514	0.169290621	0.45485
PFDN2	-0.026515726	0.824557	-0.336580766	0.00514	-0.444033991	0.00029
SEMA3E	0.36761122	0.378597	1.156478032	0.00516	-0.01834221	0.96497
ZNF416	-0.057048104	0.726698	-0.459089644	0.00517	-0.222329225	0.17389
ELF5	-0.30043811	0.256052	-0.736392601	0.00518	-0.868857926	0.00109
ADO	-0.127606086	0.094913	-0.211285394	0.0052	-0.017889637	0.81259
GDF7	-0.400148741	0.471438	1.479162637	0.00519	0.108181977	0.84228
SPRED2	0.184354589	0.224978	0.420897355	0.00521	0.196320759	0.1949
WNK1	0.206801388	0.20261	0.452206928	0.00521	0.758367275	2.80E-06
HILPDA	0.46749539	0.333888	1.348263181	0.00524	0.612023211	0.20562
MTSS1	0.306629132	0.204542	0.670043493	0.00525	0.57820515	0.01623
CCT6B	0.173946244	0.548735	0.766643924	0.00527	0.459290895	0.10112
MFSD6L	0.028915333	0.907525	-0.712450466	0.00526	0.245519165	0.31664
VPS39	0.089768951	0.403742	0.296937616	0.00529	0.410434205	0.00012
ST3GAL3	0.309246038	0.164208	0.607293951	0.0053	0.062850067	0.78
USP13	0.138987985	0.610002	0.752844422	0.0053	0.265690962	0.32626
XYLT2	0.05301012	0.689204	-0.370572758	0.00533	0.069126481	0.60104
GLB1L2	-0.05307595	0.782616	-0.536818556	0.00535	-0.101530923	0.59743
ZNF256	-0.243829229	0.063415	-0.360455796	0.00535	-0.368721579	0.00487
NRNPA1P5	0.514399129	0.367617	1.462752268	0.00535	1.139809244	0.0344
YTHDC2	-0.048655043	0.742621	0.40913824	0.00536	0.189754631	0.19811
SLC1A2	0.64589416	0.213317	1.392465943	0.00541	0.995487321	0.04907
AGTR2	1.017868668	0.283671	2.60482285	0.00542	-0.422570455	0.66474
MINPP1	-0.241661996	0.070147	-0.367841704	0.00543	-0.349790263	0.00863
HEPACAM2	-0.224148213	0.784101	-3.379915557	0.00546	0.932938713	0.21336
IL37	0.261273086	0.772602	-2.923848082	0.00548	-3.058764262	0.00514
COX7A2L	-0.028220501	0.77686	-0.276305801	0.00549	-0.19777105	0.04723
TMUB2	0.077830619	0.432892	-0.276768165	0.00549	0.073862009	0.45356
CHST2	0.59106343	0.052438	0.838777268	0.00551	0.196295467	0.52184
FAM83C	1.059184051	0.08371	1.613200956	0.0055	1.217734949	0.04226
LGR5	-0.337199907	0.444428	-1.228019984	0.0055	-0.19488225	0.65817
FOXRED1	0.135785026	0.297474	-0.364415662	0.00551	-0.140181422	0.28475
RPL13A	-0.015838157	0.897538	-0.341302339	0.00552	-0.256567173	0.03699
PRDX6	-0.102622078	0.301251	-0.274921207	0.00555	-0.280735187	0.00471
BLOC1S3	0.037206574	0.844817	-0.532018215	0.00558	0.272479784	0.144
EXOC6	0.083269047	0.642957	0.4948644	0.00558	0.091587804	0.60909

THOC6	0.085311683	0.53836	-0.387558241	0.00558	-0.243690207	0.08133
YWHAQ	0.013465098	0.868316	0.224354567	0.00558	-0.22071571	0.0066
ADD3	-0.114497385	0.263861	0.283121529	0.00562	0.009681169	0.92466
RBM3	-0.007784071	0.905567	0.180546008	0.00563	-0.188151684	0.00417
TAF11	-0.09371522	0.334148	-0.266949479	0.00563	-0.22630508	0.01972
ANKLE1	0.070331654	0.773356	0.647349338	0.00565	0.412733223	0.08211
UNC5CL	-0.008312771	0.967704	-0.569467704	0.00565	0.430341999	0.03508
ZNF765	-0.216711567	0.18518	-0.451669029	0.00565	0.074133503	0.64927
RPS6	-0.026829647	0.837927	-0.362868774	0.00567	-0.152858033	0.24387
STIM2	0.168752476	0.160505	0.329111457	0.00567	0.141463023	0.23864
STRN3	-0.049020085	0.694558	0.344203524	0.00567	-0.028623847	0.81858
UNK	0.114484769	0.360942	0.341974595	0.00567	0.46995257	0.00014
ABI3BP	0.338497989	0.162301	0.667338113	0.00572	0.808069799	0.00075
AGTRAP	0.037524051	0.816618	-0.452230433	0.00575	-0.214104341	0.18823
DSC3	-0.694282013	0.30335	1.837216092	0.00578	-1.009652548	0.13421
NUMA1	0.157042893	0.147671	0.298135405	0.00579	0.401457777	0.00021
TRAPPC6A	-0.148982507	0.386504	-0.476265583	0.00581	-0.328876352	0.05623
E4F1	-0.122308969	0.410452	-0.408972525	0.00582	-0.196199428	0.1845
VPS25	-0.04713437	0.602211	-0.248522918	0.00582	-0.212787081	0.01882
CTNND2	0.227194996	0.62025	1.217037586	0.00586	-0.180521678	0.69503
GLIS3	-0.193838722	0.419299	-0.662555044	0.00585	0.092467682	0.69958
TIMM17B	-0.061264021	0.557565	-0.286902857	0.00586	-0.358779234	0.00067
ARRDC4	0.079919701	0.726929	-0.630659692	0.00588	-0.410945741	0.07266
FANCL	0.087962521	0.475417	-0.340808072	0.00588	0.161076533	0.19009
SWI5	-0.137798314	0.516004	-0.591368496	0.00588	-0.600817321	0.0055
'I2-2610K16	-0.094923048	0.854335	-1.525919827	0.00589	0.367156193	0.46885
MYH11	0.930177912	0.085839	1.489515206	0.0059	1.249676581	0.0209
TMED2	-0.081874051	0.373479	-0.253045093	0.00591	-0.117079481	0.20297
ZIM3	1.03775709	0.078747	1.555203831	0.00591	2.167531281	9.51E-05
GLI3	0.46660176	0.124898	0.833242023	0.00593	0.358856179	0.2368
EZH1	0.047798582	0.802409	0.517043079	0.00595	0.191067333	0.31416
MARK2	0.049146243	0.688295	-0.338777484	0.00595	0.251717343	0.03916
GOLGA8A	-0.171862206	0.183233	-0.354887009	0.00596	0.140724157	0.27476
UNC50	0.011071551	0.921822	-0.309277793	0.00596	0.067960521	0.54388
PCYT1A	-0.154348101	0.12637	-0.276276219	0.00597	0.061961663	0.53714
SPIB	0.600234556	0.158446	1.156889222	0.00597	0.813933985	0.0559
P11-651P23	0.289936228	0.246696	0.680335704	0.00599	-0.021610637	0.93147
PSCA	0.409147465	0.627724	2.316359769	0.00601	0.555547574	0.51018
VSIG2	0.277495432	0.366153	0.842745904	0.00601	0.555723085	0.07019
XXYLT1	0.166693685	0.217695	0.364362942	0.00603	-0.139178434	0.30816
LACTB	0.228182705	0.272234	0.568053049	0.00604	0.082294198	0.69177
RPL23AP7	-0.094904222	0.622524	-0.526479933	0.00606	-0.345291786	0.0727
HNRNPDL	-0.004961951	0.948918	0.21185138	0.00611	-0.28792088	0.0002
FHOD1	-0.198207919	0.246702	-0.466561561	0.00613	-0.044831061	0.79193
MAK	0.34725092	0.231919	0.747665754	0.00616	0.767076142	0.00523
NACA	-0.123205258	0.23874	-0.286330117	0.00616	-0.317304585	0.00242
NAA38	0.022122792	0.88038	0.399101738	0.00617	-0.214590398	0.1433

ANKRD11	0.172091425	0.11969	0.301218334	0.00619	0.362648205	0.001
ARHGEF10I	-0.088360818	0.617549	-0.485294979	0.0062	0.031884046	0.85665
UGT1A7	0.732653184	0.220526	1.532403085	0.0062	1.191895059	0.03784
CANT1	-0.165733067	0.331122	-0.46642329	0.00621	-0.068811913	0.68585
CASC10	0.203074047	0.633186	1.121732676	0.00622	0.062271641	0.88371
ALG8	-0.062764527	0.722431	-0.490413135	0.00626	-0.301518344	0.08951
C19orf70	-0.061522171	0.630981	-0.348477063	0.00628	-0.1092402	0.38994
GSTM3	-0.003767773	0.978503	-0.382541899	0.00628	-0.278531292	0.04659
CEL	0.15030169	0.771215	-1.552613786	0.00629	-0.393412256	0.45583
SSNA1	0.04127317	0.742489	-0.343820794	0.0063	-0.192472805	0.12703
MST1	0.036641791	0.892571	-0.74521925	0.00633	-0.473989183	0.08152
NR2C2AP	-0.136023476	0.474864	-0.520367575	0.00635	-0.282556186	0.13676
CAPN9	0.084632372	0.828696	1.062300456	0.00636	0.550928537	0.15786
CYP7B1	0.35086382	0.516465	1.434888105	0.00644	0.310336509	0.56907
MRPS12	0.138758601	0.338937	-0.399802002	0.00646	-0.083098189	0.56879
RP11-159J3.	-0.027848164	0.856406	-0.419740332	0.00646	-0.334606072	0.03006
NRK	0.350332192	0.403093	1.135820774	0.00657	0.068655456	0.86994
GDE1	0.03351574	0.776583	-0.320962087	0.00658	-0.032319464	0.78428
IRAK1BP1	-0.210376284	0.348454	0.591906857	0.00659	0.461180209	0.03546
PRKRIR	-0.191928504	0.215815	0.420084635	0.00659	-0.342342632	0.02726
TMEM56	-0.361007391	0.05865	-0.517941458	0.00659	-0.285013549	0.13418
CDC27	-0.02569213	0.861399	0.398630515	0.00662	-0.002003442	0.98913
TBC1D8	0.072476067	0.519634	0.301371052	0.00662	0.323435813	0.00376
SKIDA1	-0.123184668	0.726946	0.934300127	0.00663	-0.052609135	0.88045
AGO4	-0.009335131	0.966331	0.597815142	0.00669	0.333275181	0.13086
LRRC4C	0.381473329	0.403522	1.228636055	0.00671	-0.293231668	0.52366
RFWD3	-0.183421509	0.176757	-0.366650516	0.00672	-0.132826845	0.32711
NMD3	-0.085589203	0.341201	-0.243250527	0.00673	-0.084652939	0.34559
SOCS2	0.511848532	0.133014	0.91265186	0.00675	-0.235555166	0.49442
AASS	0.261833313	0.266099	0.633248738	0.00677	0.069504324	0.76806
MEX3A	0.134827258	0.391762	0.423979345	0.00676	-0.191154944	0.22569
NIN	0.093692904	0.614746	0.501563794	0.00677	-0.086837681	0.64087
PCNP	-0.161192612	0.050653	-0.222101993	0.00677	-0.262554278	0.00146
TAF9B	0.07932691	0.58537	0.390156403	0.00677	0.250670788	0.08308
TBC1D30	-0.358625952	0.088742	-0.564350427	0.00678	0.22233655	0.28018
PTAR1	0.081378259	0.396755	0.258798258	0.00679	0.009860866	0.91812
TMEM99	0.135131351	0.513404	-0.580013416	0.00679	-0.057519484	0.78184
HECTD1	-0.133361882	0.108301	0.223640557	0.00682	0.096682997	0.24289
ADCY6	-0.017611549	0.903959	-0.395268873	0.00684	-0.037167716	0.79867
AGAP2	1.006595667	0.06232	1.454498465	0.00686	0.568128134	0.29327
RGR	0.722846464	0.146252	1.293770541	0.00686	1.665863513	0.00041
RPL3	0.118365724	0.339878	-0.335248517	0.00688	-0.079188489	0.52316
FGD5	0.625307226	0.175836	1.22644583	0.00689	0.534554244	0.24879
LAMTOR3	0.038093682	0.694016	0.259743457	0.00689	-0.127885076	0.18681
YIPF6	0.13326308	0.254277	-0.315695369	0.00689	-0.102249019	0.3821
COLEC12	0.203361873	0.427642	0.689639833	0.0069	0.061642439	0.81
CDC7	0.018791139	0.90586	0.423217209	0.00693	-0.411670383	0.00951

SEMA3A	0.200989428	0.413999	0.663638118	0.00694	0.24653274	0.31636
TCIRG1	0.040884664	0.794551	-0.4270544	0.00694	0.192036771	0.21929
EPB41L3	-0.085830543	0.769061	0.782817523	0.00696	0.032914579	0.91057
KDM3A	-0.05427463	0.750627	0.458491424	0.00696	0.463723403	0.00643
SF3B3	0.012862432	0.868827	0.209432114	0.00698	-0.086393185	0.26695
FLAD1	-0.065890711	0.647323	-0.388650698	0.00699	-0.12378763	0.38951
TMEM147	0.153444752	0.298722	-0.400296567	0.00701	-0.185634844	0.21039
AC009120.3	0.096265944	0.824883	1.051744304	0.00703	0.682194327	0.09214
ARID5B	-0.152032681	0.251732	-0.357102705	0.00706	-0.411382153	0.00194
CCT4P2	0.612346051	0.43019	1.865032435	0.00707	1.267905976	0.07967
HTRA2	-0.213469636	0.132856	-0.379998674	0.00707	-0.265871211	0.06005
RBM4B	-0.174545934	0.235096	-0.395950619	0.00707	-0.080497667	0.58313
SLC1A5	0.045389111	0.765519	-0.410498055	0.00707	-0.016383897	0.91424
ULK1	0.301502176	0.158446	0.572708693	0.00707	0.302637925	0.15633
TRIM9	0.512832046	0.20645	1.092466557	0.00708	-0.014410346	0.97206
ANKRD44	0.443676592	0.164306	0.85534778	0.00712	0.462391286	0.14726
UBE2D3	0.087736538	0.360951	0.258055211	0.00713	-0.181643577	0.05878
LRRC6	-0.524160481	0.125812	-0.908303532	0.00716	-0.339003816	0.31478
PDK3	0.164972258	0.285629	-0.419677657	0.00718	0.069644801	0.65182
DIMT1	0.158616913	0.166596	0.306325984	0.00719	0.105458445	0.357
LPHN3	0.405188491	0.161765	0.777014487	0.0072	-0.084992193	0.7695
NID1	0.323020815	0.054128	0.450349457	0.0072	0.031664744	0.85033
PLCH1	-0.155631229	0.491033	-0.607635868	0.00721	0.409484666	0.06836
RPS10	0.139548196	0.223856	-0.308438977	0.0072	-0.074698581	0.51507
CRTC3	0.078230018	0.687356	0.519075253	0.00723	0.299805876	0.12086
IFI16	0.25933241	0.346256	0.737687651	0.00724	-0.291502757	0.29068
SEMA5B	-0.479367116	0.591559	2.285258245	0.00728	-0.95162071	0.29181
ARHGEF12	-0.08231529	0.331595	0.226718954	0.00728	0.034728244	0.68165
UBR5	0.207722425	0.180517	0.415721152	0.0073	0.088917605	0.56626
PSENEN	-0.102140574	0.432283	-0.348274366	0.00735	-0.464174071	0.00041
MFSD5	0.03920723	0.790896	-0.396808891	0.00735	-0.085027017	0.56611
LGI4	-0.419053494	0.455425	-1.512027051	0.00738	-1.38893248	0.01443
SUMO2	-0.039574449	0.628679	0.218463045	0.00739	-0.276023603	0.00075
GON4L	-0.066209094	0.581806	0.320357044	0.00742	0.103597933	0.38951
STAG1	-0.102808973	0.39741	0.322548999	0.00741	-0.132199283	0.27647
IMPACT	0.038209466	0.759603	0.330972489	0.00743	-0.026459882	0.83187
LGALS2	0.142536635	0.699831	-1.014050353	0.00742	0.1573135	0.66964
RPL29	0.091154978	0.456515	-0.327775786	0.00743	-0.216344604	0.0773
TRIM29	-0.632859113	0.424257	2.077809758	0.00743	-0.517557762	0.51302
ERLEC1P1	0.068259537	0.850143	0.880306456	0.00747	-0.105867743	0.77038
PGK1	-0.067105256	0.701017	-0.467520056	0.00748	-0.220923816	0.20622
SNAPC1	0.185366179	0.281438	0.450698818	0.00751	0.043908286	0.79872
ALX4	0.645271156	0.336836	1.739261024	0.00754	0.34698939	0.60776
ETV5	0.165070163	0.239709	0.370691509	0.00757	-0.098726166	0.48127
C3orf33	-0.220957525	0.249239	-0.511142047	0.00759	0.11342501	0.54259
NFIA	0.450590082	0.073245	0.670216319	0.00759	0.313294466	0.21262
NR3C1	0.265882289	0.194454	0.545169714	0.00758	0.109877583	0.5916

TIAM1	0.013883048	0.966773	0.884667902	0.00759	-0.268056039	0.42248
GPD1L	-0.127549583	0.399113	0.397068093	0.0076	0.165578545	0.26858
ANXA9	-0.150769613	0.59703	-0.769696448	0.00763	-0.405416764	0.15663
LPAR5	-0.235627437	0.462063	-0.881809019	0.00765	-0.000180966	0.99954
AMACR	-0.353266197	0.061248	-0.501055809	0.00769	-0.434046539	0.02152
PDCD2L	-0.39512618	0.050584	-0.533131229	0.00768	-0.123866342	0.52856
ZNF587B	-0.205946751	0.090951	-0.323143017	0.0077	0.097053882	0.42018
GABRR1	1.455589125	0.090686	2.283162351	0.00772	-0.096562933	0.91177
LEMD1	-0.169320647	0.677182	-1.091548665	0.00772	-0.866913551	0.03414
SLC16A9	0.751110112	0.096395	1.194914559	0.00772	0.161066907	0.72306
TNPO2	0.164014588	0.177813	0.32342589	0.00774	0.154209778	0.20453
IFNLR1	0.312392492	0.087631	0.482832911	0.00776	0.228695076	0.2093
ATP6V1B2	0.037497197	0.652915	-0.220966985	0.00776	-0.152781761	0.0673
IGF2R	-0.177598279	0.278825	0.435492154	0.00782	0.399144051	0.01481
MRPL12	-0.009867463	0.967731	-0.655784162	0.00781	-0.368367483	0.13348
MOCS3	0.018330791	0.858205	-0.272809474	0.00784	0.0486503	0.63108
RRAS	-0.058277814	0.772439	-0.537926853	0.00784	-0.294863561	0.1443
AHCYL1	0.108873178	0.347213	0.307478121	0.00786	0.034690617	0.76446
POLH	-0.199559449	0.194054	-0.406912565	0.00788	0.004992116	0.97394
NSUN7	-0.150715963	0.416652	-0.494161994	0.00789	0.033972156	0.85374
CNTNAP5	1.032255361	0.260835	2.38718558	0.0079	0.874622712	0.3505
ZNF277	-0.211296111	0.117237	-0.355430623	0.00792	-0.210916989	0.11567
ATP6V0E1P	1.047324951	0.087368	1.548487233	0.00796	1.377385334	0.02036
CTC-471F3.4	1.8467305	0.103668	2.941232371	0.00798	2.540770287	0.02286
FRY	0.241458191	0.377392	-0.727576045	0.00799	0.58044689	0.03364
MAN2A1	-0.070266925	0.607433	-0.362724302	0.008	-0.210956062	0.12297
PFN1	0.200620909	0.181157	0.397610196	0.00801	-0.143660931	0.33847
PIK3R4	-0.105742036	0.464458	0.38100367	0.00801	-0.081079507	0.57434
ZNF408	0.020671931	0.917618	-0.535373223	0.00805	-0.225584107	0.2612
ZFPM2	0.487580171	0.231931	1.04758992	0.00808	0.318953801	0.43382
LIG3	0.200341691	0.253906	0.463344287	0.00809	0.01637736	0.92559
NOTCH4	0.214698248	0.420018	0.675230799	0.0081	0.199625606	0.44372
HNF4A	-0.148434606	0.630702	-0.819364726	0.00812	-0.556993016	0.07211
ISPE1-MOB	0.079018302	0.818166	-0.924553888	0.00814	-0.030400182	0.9295
KIAA1191	0.246040572	0.060565	-0.347964482	0.00814	-0.221074699	0.09248
NSRP1	0.065787767	0.673982	0.406392781	0.00814	0.395054565	0.01113
BAZ2B	-0.004686092	0.971188	0.342307545	0.00818	-0.075369629	0.56115
C11orf31	-0.104677014	0.438667	-0.357406802	0.00817	-0.25310779	0.06177
ZNRF2P2	0.945203111	0.086035	1.388020668	0.00817	1.306831396	0.01367
VAV3	-0.815438537	0.056976	-1.132141745	0.00819	-1.0507892	0.01844
GABRB2	-0.965396295	0.255084	1.932070033	0.00825	-0.13384721	0.8698
LAMP3	0.312696842	0.413911	0.967747922	0.00825	0.569174488	0.12665
SLC4A8	-0.026770314	0.933912	0.838483002	0.00825	0.136076175	0.67037
ICK	-0.052266883	0.716849	0.378215486	0.00829	0.382152578	0.0077
UAP1L1	-0.335739303	0.215634	-0.724271095	0.00829	-0.11391969	0.66556
ENPP5	-0.423272528	0.108826	-0.690958666	0.00833	-0.616565762	0.02039
NUDT16L1	-0.115620619	0.499191	-0.453223965	0.00835	-0.144352763	0.3968

ABAT	0.196064575	0.207565	0.406918647	0.0084	0.467398978	0.00251
ALKBH7	0.089400696	0.652798	-0.531163843	0.00838	-0.089118415	0.65361
C1orf51	0.276594675	0.193762	0.539905965	0.0084	0.147523859	0.48081
CIZ1	0.175039525	0.167505	0.332997875	0.00838	0.265298052	0.03608
RPS23	-0.088193768	0.516295	-0.358322735	0.00836	-0.283232822	0.03713
ZNF852	-0.433241715	0.118992	-0.73443916	0.00837	-0.002632271	0.99213
2-Sep	0.081997221	0.132292	0.143025914	0.00847	-0.067316793	0.2168
FOXG1	0.617590237	0.573404	2.7965256	0.00849	-1.086793734	0.35518
SRCRB4D	0.111155918	0.776626	0.97779462	0.0085	-0.466792985	0.26811
TBC1D20	-0.100027086	0.159449	-0.185122359	0.0085	-0.089143609	0.20565
ZC3H4	-0.077296327	0.663136	0.464258773	0.00851	0.417159259	0.01805
ZNF165	-0.229391408	0.220906	-0.492533473	0.00851	-0.245197673	0.18821
DTNA	0.312830099	0.281413	0.744421555	0.00853	0.149340208	0.60589
EXD2	-0.166981826	0.102884	-0.264702503	0.00853	-0.19043387	0.06155
MDFIC	0.222675076	0.348199	0.621756212	0.00858	0.03893852	0.86963
TMEM176A	0.198878158	0.571596	-0.927130496	0.00858	-0.641157448	0.06886
RAB33B	-0.043552274	0.72137	-0.320538854	0.00861	-0.090123447	0.46191
SMYD3	-0.183042064	0.345994	-0.522502912	0.00862	-0.234686057	0.22773
MYH10	0.073602949	0.643576	0.417291908	0.00865	0.077738093	0.62503
AC005013.1	0.321161688	0.532689	1.348775258	0.00869	0.111705212	0.82823
AL589743.1	0.87319505	0.245699	-2.851338227	0.0087	-0.388816033	0.62727
SHANK1	0.195187194	0.599057	0.929389124	0.00873	0.637797062	0.07622
FBXL14	-0.233083642	0.308261	-0.60075204	0.00875	-0.024912202	0.91244
TA-407F11	0.928756617	0.141558	1.55972979	0.0088	1.41005401	0.0198
MAPRE3	0.057750271	0.799107	-0.609694625	0.00881	0.200822927	0.37329
CAPN12	-0.081401933	0.777312	-0.7590836	0.00882	-0.176475345	0.53871
ADC	-0.411962497	0.28994	-1.020621955	0.00885	-0.054287566	0.88611
PYGO1	0.113964878	0.630087	0.616081551	0.00885	-0.178597954	0.45031
ARG2	0.037536637	0.861308	-0.564834479	0.00888	-0.483687504	0.02579
MAMDC2	-0.31168797	0.605459	1.460602279	0.0089	0.953056038	0.09766
AIMP1	-0.212096017	0.058693	-0.291939229	0.00892	-0.22514638	0.04464
ST8SIA2	1.175272528	0.090859	1.767219977	0.00893	0.866540784	0.20379
UBA3	-0.086618829	0.345635	0.23744655	0.00893	-0.225805277	0.01396
TARBP2	-0.182941271	0.175048	-0.351208165	0.00896	-0.292266643	0.03088
TRIM26	-0.049473634	0.646842	-0.28204557	0.00897	-0.169310958	0.1176
CUL7	0.007311524	0.956807	-0.352432723	0.00907	-0.034341535	0.79898
HAPLN1	-1.698542144	0.057771	2.248142636	0.00907	-2.526572046	0.00987
KLHL31	-0.560460627	0.099382	-0.889010101	0.00907	-0.464851757	0.1655
MAST3	-0.058784573	0.8282	-0.708146057	0.00906	0.328273472	0.22304
PTPN6	0.040992524	0.822404	-0.478683281	0.00907	0.176707638	0.32886
SNED1	0.771441803	0.076505	1.131937048	0.00914	0.933594363	0.03179
RNF115	-0.129641489	0.148178	0.227822101	0.00917	0.026630577	0.76281
C12orf23	-0.171409034	0.111642	-0.280216981	0.00921	-0.227669559	0.03454
RUNX1T1	0.621415192	0.068192	0.885839852	0.00921	0.611582351	0.07304
RASA4	-0.078669464	0.754137	-0.654387124	0.00926	0.197374867	0.42955
RHBG	0.364181427	0.491034	1.264881574	0.00925	1.206374038	0.01444
UBE2A	0.211670052	0.452878	0.721439248	0.00926	0.490853827	0.07741

ARHGAP21	-0.009531108	0.941644	0.33797526	0.0093	0.118717584	0.36129
WDR52	-0.368361476	0.061857	-0.511766695	0.00932	-0.018282507	0.92579
FAM204A	-0.176013749	0.059675	-0.241086711	0.00938	-0.239973527	0.01007
CCDC115	-0.158847509	0.257341	-0.362470356	0.00941	-0.480101931	0.00066
SS18L2	-0.085489287	0.578914	-0.401522488	0.00942	-0.168847667	0.27365
MORF4L1	-0.007680991	0.920283	0.198785352	0.00945	-0.231358203	0.00258
MTRNR2L1	-0.171439812	0.52278	0.693752942	0.00948	-0.460508873	0.08627
RPL14P1	-0.06132016	0.890217	-1.22887535	0.00948	-0.7246737	0.11528
FKBP8	0.05408258	0.605321	-0.271937143	0.0095	-0.277568336	0.00826
ITGA9	0.408104106	0.264934	0.946235321	0.00951	0.22195037	0.54407
LIN52	-0.140817847	0.42365	0.447869755	0.00951	0.081828802	0.64049
PRKCH	-0.068042711	0.83611	0.83477993	0.00956	0.199411956	0.54317
COPG2	-0.111453185	0.402746	0.337701126	0.00957	-0.033696473	0.79888
P11-315D13	1.229685122	0.127693	1.957678995	0.00959	1.908737948	0.01243
UBLCP1	0.080828148	0.436287	0.264784139	0.00959	0.009600141	0.92642
WDR53	-0.353875845	0.171739	-0.670910858	0.00959	-0.669043439	0.01008
PREB	0.013651516	0.939462	-0.465568216	0.00962	-0.253829483	0.15781
CCDC176	-0.070402069	0.725927	0.505667664	0.00968	-0.144811063	0.47112
CELF3	-0.458989279	0.609437	1.981254981	0.00969	0.19372955	0.81457
SOCS6	-0.109893381	0.291619	-0.268089059	0.0097	-0.248697694	0.01696
LRFN5	0.536674587	0.166525	0.997381279	0.00971	0.084567378	0.82806
GLS	0.035423551	0.818329	-0.399154537	0.00971	-0.261392177	0.09065
TD-2206N4	0.260120059	0.502569	0.946662639	0.00975	0.288079357	0.4436
CDK6	-0.003644032	0.981239	0.39969524	0.00976	-0.01361011	0.92997
CAPZA1	0.073308754	0.216091	0.152513154	0.00977	-0.094872745	0.10957
ANXA8	1.48373289	0.068376	2.093323764	0.00979	0.591580839	0.46989
ADRA2C	-0.709789551	0.090813	-1.085610599	0.00981	-0.12660605	0.75869
EPHB4	-0.112256223	0.26708	-0.260829081	0.00981	0.053334997	0.59661
P11-475E11	1.139868577	0.120186	1.78142587	0.00981	1.913114076	0.00561
CSTA	1.111640148	0.390148	3.134411519	0.00982	2.052868504	0.09841
CYB5A	0.050827075	0.701982	-0.343149394	0.00983	-0.024431279	0.85383
SLC5A7	0.854653156	0.272867	1.957447643	0.00985	0.606096749	0.44368
UGT2B11	-0.787118273	0.102963	-1.256507885	0.00986	-1.028551929	0.03506
MYZAP	-0.268111537	0.150108	-0.479917451	0.00987	-0.28791936	0.12146
ZNF397	-0.081249592	0.550034	0.344881631	0.00987	0.297855795	0.02634
FERMT2	0.297850028	0.105801	0.474263651	0.00995	-0.330993135	0.07275
LIMS1	-0.03220741	0.722197	0.232262662	0.00996	-0.162620077	0.07246
MRPL50P2	1.199747619	0.080183	1.752222028	0.00996	0.98448212	0.15159
XAF1	-0.117138353	0.587059	0.542556785	0.00997	0.491951033	0.01969
FBN3	0.337745675	0.530827	1.38310632	0.00999	0.341805667	0.52511
FGF13	-0.069993395	0.849625	0.945784129	0.00999	0.518846368	0.15773
SMYD4	-0.298168413	0.094032	-0.456419036	0.01003	-0.047393376	0.78713
TMEM203	-0.142585136	0.311804	-0.361847517	0.01004	-0.177554434	0.20585
ZNF287	0.200906979	0.460089	0.685746197	0.01005	-0.157222946	0.56389
PRMT3	0.020321587	0.88123	0.341575814	0.01009	0.053595913	0.69123
TMEM229B	-0.293734994	0.245196	-0.65301911	0.01015	0.116585657	0.64121
BRD2	0.236785796	0.056206	0.318606368	0.01017	0.129696588	0.29575

CDK10	-0.140182566	0.222961	-0.294207891	0.01018	-0.028344644	0.80404
MAP6	0.71275637	0.237924	1.446718772	0.01017	0.587928974	0.33853
VEZT	0.093412237	0.33191	0.246365701	0.01017	0.035910716	0.70885
HNRNPH2	-0.023056097	0.768663	0.199257248	0.01019	-0.301034859	0.00013
TRIM36	0.651584705	0.141583	1.137030697	0.01023	0.90398437	0.04626
FOXA1	-0.083388474	0.712088	-0.580070681	0.0103	-0.227076796	0.31471
STS	0.218957387	0.200556	0.435407506	0.01032	0.457937739	0.00708
GGCX	-0.100569071	0.35268	-0.276989178	0.01036	-0.222184125	0.04002
KLHL15	0.019831622	0.879119	-0.334587339	0.01035	-0.17829704	0.17181
LMTK3	-0.110049927	0.594368	-0.533551145	0.01036	-0.017681271	0.93117
EEF1G	-0.059575518	0.619454	-0.307235108	0.01043	-0.295328526	0.01383
PSD3	0.279360694	0.071961	0.396493316	0.01044	0.264680875	0.08774
RPS12	-0.023930465	0.854323	-0.333783985	0.01045	-0.245361949	0.05982
ARPP19	0.048455071	0.646786	0.270361752	0.01046	-0.045341642	0.66811
TRAK2	-0.141209655	0.15077	-0.25014474	0.01047	-0.08610383	0.3785
LOX	-0.205474048	0.53259	-0.842986347	0.01049	-0.645860387	0.05028
PDZD3	-0.303491844	0.642048	-1.706771294	0.01051	-1.356109277	0.04095
PSMB8	0.153679909	0.378276	-0.452799564	0.01052	0.018919997	0.91369
MYL4	-0.285268948	0.756685	2.130030008	0.01061	-0.004528786	0.99598
BAAT	-0.50152264	0.571583	-2.311648624	0.01062	-1.072584569	0.22865
TCF12	0.212526845	0.127365	0.355210418	0.01065	0.119127633	0.39277
UBE2G2	0.131940994	0.149422	0.231293127	0.01068	0.216705311	0.01711
HFE	-0.327699297	0.142942	-0.56989803	0.01069	-0.077731599	0.72659
CCDC9	0.187171392	0.206844	0.365005649	0.01073	0.187508792	0.20049
KIAA1432	-0.045710492	0.63412	-0.244487629	0.01075	-0.042052408	0.66072
AGBL2	0.042472042	0.918075	-1.054274818	0.01076	-0.300269435	0.45654
PLXDC2	0.042091606	0.852149	0.574512238	0.01077	-0.016819483	0.94062
MST1P2	-0.581276824	0.180091	-1.116634301	0.01078	-0.643466219	0.13693
GLYATL1	-0.780099189	0.14611	-1.376677759	0.01079	-0.646846396	0.22606
CHGA	1.029785386	0.173714	1.892628519	0.0108	2.08326236	0.00505
CHPF	0.120786862	0.511989	-0.470851894	0.01085	-0.107764361	0.55879
FBXL5	-0.092208361	0.356583	-0.254516608	0.01084	-0.223139102	0.0257
TMCO4	-0.20477742	0.272356	-0.475162647	0.01085	0.105577946	0.56625
CLDN9	-0.44650832	0.133141	-0.758538766	0.01088	0.034495107	0.90352
AC027612.6	-0.356813938	0.220254	-0.74140006	0.01092	-1.127145616	0.00014
PDCD5	0.040424961	0.71945	0.283023431	0.01093	-0.105275831	0.34935
SLC25A28	0.096796653	0.442141	-0.32195812	0.01092	-0.09625768	0.44555
FAM216B	1.210033276	0.157185	2.02050688	0.01095	1.773915866	0.02887
SSH3	0.083084377	0.6558	-0.475734969	0.01095	0.102483972	0.58157
ACSF2	-0.054595279	0.650963	-0.308219955	0.01096	-0.158094156	0.1913
C16orf52	-0.118180454	0.301455	-0.28954987	0.011	0.088058187	0.4361
IFI44L	0.547115951	0.172356	1.011708518	0.01101	-0.106499182	0.79216
NR1H4	0.160362157	0.578545	-0.746150352	0.011	-0.350396159	0.22847
RGPD5	0.288644684	0.290193	0.6884065	0.01101	1.147752287	2.14E-05
RPH3AL	0.269870318	0.405147	-0.845477222	0.01101	-0.226034494	0.49049
KCND3	0.58269293	0.118343	0.942877457	0.01103	0.359790747	0.33523
DDX60	0.151335987	0.475973	0.534282577	0.01106	0.265327845	0.21075

SLC25A10	-0.160423885	0.475578	-0.571812795	0.01105	0.115666395	0.60553
CDH17	-0.595750022	0.285105	-1.415946298	0.01107	-2.052394813	0.00023
SPTBN2	-0.016699916	0.960701	0.852147506	0.01107	-0.539159699	0.11097
HAND1	0.686673883	0.078943	0.987975916	0.01109	-0.408734414	0.30232
RP11-454L1.	-0.458412976	0.334183	-1.268700859	0.01111	-0.589303386	0.21429
AATK	-0.248276275	0.482663	-0.912555665	0.01115	-0.570387582	0.10859
SLC35E2B	0.540803064	0.123552	0.877763929	0.01115	0.388081502	0.26997
ZFAND6	0.177283452	0.204461	0.351532078	0.01118	0.061900378	0.65735
GABARAPL.	-0.064441944	0.543148	-0.268110951	0.0112	-0.219910094	0.03811
SUCLG1	-0.208400969	0.121274	-0.339985762	0.01119	-0.369598322	0.00604
MFSD6	0.006633771	0.968398	0.421488113	0.01127	0.290670044	0.08129
SORBS1	0.233805551	0.142391	-0.405030594	0.0113	0.247203586	0.1206
ASPHD1	0.297366652	0.245547	-0.679853778	0.01132	0.285215379	0.2596
MRPS11	-0.124214389	0.337751	0.320722198	0.01134	-0.161839252	0.20743
RPL4	-0.034286934	0.768514	-0.29490927	0.01136	-0.339102493	0.00361
NTRK2	0.601876782	0.289167	1.426115843	0.01138	0.136803088	0.8106
SDF2	-0.013886874	0.903942	-0.291245192	0.01138	-0.275033338	0.01763
CDH19	0.563141902	0.464571	1.820463931	0.01141	1.460055722	0.04749
NRNPA1P4	0.314106805	0.208611	0.604107732	0.01142	0.723127889	0.00252
EEF1A1P2	-0.094748757	0.841924	-1.396674192	0.01144	-0.332616301	0.48918
INSIG2	-0.144779165	0.562172	-0.633127157	0.01144	-0.140913844	0.57243
MDGA2	0.950525152	0.130084	1.571061806	0.01146	0.310870293	0.63245
PCBD1	-0.04512568	0.773263	-0.396155428	0.01148	-0.104349952	0.50492
IMPDH2	-0.175390637	0.125988	-0.289722934	0.01149	-0.365522598	0.00144
TMEM181	-0.004755739	0.964565	-0.270525889	0.0115	0.216036327	0.04309
NIP7	-0.115570668	0.463569	-0.39816269	0.01155	-0.296376291	0.06035
WDR59	0.186865875	0.195465	0.363341967	0.01156	0.251396043	0.0805
FER	-0.187312508	0.085524	0.272923516	0.01159	0.21684413	0.0455
FAM219A	0.044577165	0.81686	-0.488395095	0.0116	-0.282362536	0.14398
FAM96B	0.141541619	0.352237	-0.386144023	0.01167	-0.20462476	0.18184
STIP1	-0.190164715	0.096601	0.287541894	0.01169	-0.240028218	0.03605
DCHS1	0.478806263	0.075851	0.6792742	0.0117	0.435298118	0.10647
S100PBP	-0.059975909	0.726836	0.427198434	0.01171	0.203152014	0.2328
ISLR	0.624032871	0.102072	0.960898505	0.01172	0.264896678	0.48816
SURF6	-0.17860321	0.230275	-0.373319061	0.01173	-0.293011006	0.04748
MCF2L2	0.141076789	0.618107	0.703226296	0.01176	0.630100852	0.02391
RP11-137J7.	-0.120356135	0.847203	1.328313917	0.01176	-0.959264304	0.17014
P11-872D17	1.173208004	0.404019	3.472451348	0.01177	1.515723192	0.27758
SYTL5	-0.255552682	0.14408	-0.440291908	0.01177	0.055092681	0.75195
AQP5	1.213969964	0.277514	2.651928422	0.01182	1.264577482	0.25056
KANK4	0.763813179	0.057439	1.007219555	0.01183	0.270281983	0.50346
CRMP1	0.433148525	0.218457	0.881857885	0.01184	0.260943922	0.45863
PHF20L1	-0.103142101	0.410882	0.31400849	0.01185	-0.188915283	0.13173
BOLA1	-0.167597696	0.566215	-0.752528544	0.01189	-0.268293887	0.35807
PCMT1	-0.006219456	0.951095	-0.253596601	0.01189	-0.202046164	0.04655
MTPN	0.000762577	0.991296	-0.175504128	0.01191	-0.123328647	0.07788
ABCB1	-0.325545778	0.57429	-1.50516059	0.01197	-1.339856931	0.03289

EEF1DP3	-0.054462186	0.863498	-0.802622454	0.01195	0.635160285	0.03777
FBXL6	0.070564324	0.706452	-0.476640301	0.01196	-0.259597694	0.16836
SCD5	-0.001227854	0.994683	0.457658055	0.01196	0.251526467	0.16983
DCLK1	0.693764392	0.107949	1.064408319	0.01199	0.276295745	0.53214
METTL16	0.017591223	0.897314	-0.341504467	0.01202	-0.012748213	0.92537
PNPLA8	0.261744861	0.100557	0.399184423	0.01203	-0.147031736	0.3582
RPL12	0.024994475	0.857262	-0.348939274	0.01205	-0.177870088	0.2006
ATP12A	-0.599343781	0.604461	2.728442314	0.01206	0.18699188	0.86761
MRPL13	-0.225658293	0.085263	-0.32717856	0.01209	-0.396933272	0.00252
PAFAH2	-0.221943962	0.20425	-0.438146966	0.01208	-0.199629345	0.2534
PRDX3	-0.028831933	0.81351	-0.306728341	0.01208	-0.180814742	0.13907
SNX14	-0.145242247	0.26123	0.322843946	0.0121	-0.219263253	0.08906
TUFM	-0.166535015	0.277746	-0.384635188	0.0121	-0.361265356	0.01855
STAT2	-0.08273177	0.484776	0.295440735	0.01211	0.129182022	0.274
LPL	-0.264438315	0.712826	1.761999838	0.01216	-0.794270495	0.27703
MRPL14	0.165719705	0.081263	-0.239563725	0.01217	-0.12680085	0.18528
MRPL24	0.031751971	0.807287	-0.329477952	0.01219	0.026369682	0.83919
GRIP1	0.097589209	0.792379	0.906899257	0.01222	0.381141019	0.30041
P11-15H20	-0.37051133	0.061964	-0.49291598	0.01221	-0.053225997	0.78634
ARHGEF18	0.149540273	0.403892	-0.449387878	0.01227	0.11584881	0.51677
GIPC2	-0.28448304	0.333398	-0.739217293	0.01227	-0.541485953	0.067
GTF3C4	0.070874855	0.475696	-0.249027507	0.01227	0.126486942	0.20187
KCNQ5	0.763364032	0.491067	2.594608333	0.01227	0.937119871	0.37559
IKBKAP	-0.162645457	0.261267	-0.361857462	0.01229	-0.189461184	0.19016
MKRN2	-0.087521364	0.368037	-0.241846576	0.01231	-0.151825705	0.11694
NDUFB8	-0.048698798	0.654714	-0.272437875	0.01232	-0.333748239	0.00228
CRTAC1	0.70114907	0.137399	1.165257946	0.01233	1.231025837	0.0082
DYRK1B	0.082138582	0.665878	0.456674973	0.01236	0.352141808	0.05775
EIF3J	0.181341129	0.121648	0.292365287	0.01243	0.04632637	0.69258
SLC6A8	0.368065163	0.206776	-0.729626719	0.01244	0.370273685	0.20401
TSTD1	-0.080509168	0.609046	-0.395889312	0.01246	-0.186748777	0.23611
LAMA4	0.528540609	0.065406	0.716292655	0.01248	0.03831981	0.8938
PPP1R18	0.473512789	0.075429	0.660821763	0.01252	0.080975822	0.76192
NOTCH1	0.215869393	0.368321	0.596888698	0.01253	0.424844455	0.07594
UBN2	-0.060170215	0.739553	-0.451795839	0.01253	0.225400415	0.21161
DLG5	0.117524051	0.316549	0.291020773	0.01255	0.395658848	0.0007
FGF11	-0.011297547	0.964421	-0.634459773	0.01258	-0.045226277	0.85822
FLI1	0.542158336	0.358697	1.46138366	0.01259	-0.566259059	0.34716
TCF25	0.027320699	0.790046	0.254808286	0.0126	-0.006414114	0.95013
SLC25A26	-0.18411227	0.173407	-0.331517222	0.01263	-0.441749307	0.001
DHRSX	0.066795967	0.6159	-0.33521312	0.01264	-0.070633235	0.59654
HMGB2	-0.107676662	0.517962	0.413665755	0.01265	-0.34072603	0.04094
OGN	0.477272769	0.462929	1.620737542	0.01264	0.334157172	0.60732
EMCN	0.696692502	0.236278	1.464399859	0.01268	-0.142539905	0.80917
POLR2C	0.03293945	0.697926	-0.211372405	0.0127	-0.171691887	0.04396
CORO2B	0.918791994	0.142212	1.53181192	0.01273	-0.123802768	0.84821
TENC1	0.3344435	0.148032	0.575454052	0.01272	0.229604972	0.32062

APBA2	0.204393871	0.588644	0.9188043	0.01277	0.828650962	0.02663
CHCHD1	-0.118831604	0.408923	-0.357386469	0.01276	-0.328575016	0.02303
QPRT	-0.153020079	0.128253	-0.249885146	0.01277	-0.361621342	0.00033
TMEM9	0.054434976	0.584996	-0.248390556	0.01276	-0.129265443	0.19526
ANO1	0.494224943	0.100575	0.748205029	0.01279	0.831247377	0.00567
EYA4	0.395459154	0.583641	1.778457188	0.01281	0.230559074	0.74951
HSCB	-0.123328267	0.362727	-0.335147674	0.01282	-0.092738378	0.48715
PTBP2	-0.117519624	0.391023	0.336198871	0.01281	0.105349518	0.43808
RER1	0.055214334	0.62394	-0.279696273	0.0128	-0.078045347	0.48762
SRSF9	-0.045894056	0.697268	-0.293461053	0.01282	-0.412198043	0.00048
LHFP	0.523583141	0.215696	1.047407053	0.01283	0.396498987	0.34848
P4HA1	-0.080192973	0.7006	-0.519010017	0.01284	-0.223654643	0.28356
RBM12	0.053813493	0.744002	0.409385902	0.01287	-0.188138654	0.25354
LARP1	0.012066409	0.942089	0.412359498	0.01292	0.513869813	0.00195
LSM7	0.076047631	0.533721	-0.30498929	0.01293	-0.202457502	0.09938
MCOLN3	0.058455795	0.889487	0.976869978	0.01294	0.872759724	0.02808
TMPRSS15	0.232429579	0.843	2.726996672	0.01294	0.685411178	0.55113
C1QTNF7	-0.837484001	0.052232	-1.066671073	0.01305	-0.497393127	0.24789
COL23A1	0.401458879	0.221983	0.797144874	0.01307	0.05658875	0.86472
DUSP7	0.013096134	0.935974	-0.407486555	0.01306	-0.093037478	0.56766
KIF5C	0.540096269	0.20859	1.052135023	0.01309	-0.275676802	0.53297
ARL8A	0.36481642	0.100832	0.548699335	0.01315	0.0306358	0.89108
PACSIN3	-0.209932953	0.253762	-0.455177284	0.01318	-0.183518123	0.31688
SH3GL3	0.188564864	0.572029	0.809212464	0.01319	0.559174231	0.08921
SLC1A1	-0.23491267	0.550787	-0.977616482	0.01318	-0.907922359	0.02157
TMED3	-0.090986452	0.534171	-0.362698183	0.0132	-0.229737237	0.11643
PCDHA2	0.173996799	0.574623	0.713730876	0.01322	0.381542827	0.20427
FOXP1	-0.095207498	0.463829	0.320076891	0.01324	0.207608711	0.10917
PLVAP	-0.822253656	0.504147	2.705022971	0.01331	-2.629034767	0.07257
TCERG1L	0.541873478	0.344399	1.323658348	0.01332	0.568747214	0.31933
SLC29A2	-0.103680454	0.53186	-0.410610585	0.01334	0.285870618	0.0827
ADIPOR1	-0.009156738	0.929901	-0.25695289	0.0134	-0.19986428	0.05499
NRNPA1P2	0.102983199	0.770115	0.804743908	0.01342	-0.174187089	0.62623
ZC3H7B	0.132447763	0.136543	0.218609607	0.01346	0.05265405	0.55366
FAM126B	0.196346299	0.144752	0.331967859	0.01353	0.382445018	0.00438
TMEM178B	-0.606617282	0.655012	2.864855198	0.01362	-0.786434197	0.56069
CPNE3	-0.085965134	0.28159	-0.19612508	0.01365	-0.215886941	0.0068
CDC45	0.136418908	0.596872	0.632381821	0.01367	-0.168244089	0.51536
ACSL5	0.237187369	0.227953	-0.485737936	0.0137	-0.316486961	0.10828
BUB3	-0.119227436	0.224897	-0.241400062	0.0137	-0.240546932	0.0143
C14orf2	-0.149125367	0.144947	-0.250345118	0.01375	-0.389320455	0.00015
FBXL7	0.447103631	0.071678	0.606945376	0.01374	0.515154648	0.03732
GPR20	-12.13727267	0.092392	17.60140131	0.01373	17.19276923	0.01611
COPB1	-0.044047778	0.611469	-0.213564617	0.01377	-0.188089221	0.0302
P11-950K24	1.046688662	0.058867	1.315000227	0.01377	1.985876746	0.00014
TPP2	-0.176504768	0.29208	0.41168763	0.01378	-0.203311934	0.22466
CEP72	0.107920911	0.621463	-0.543104406	0.01378	0.28447767	0.18944

PPP1R1B	-0.467670775	0.41488	-1.42314204	0.01381	-2.478688358	3.77E-05
FAM134C	-0.130173782	0.186401	-0.242066544	0.01384	-0.123570533	0.20942
PRRX1	2.095426482	0.050115	2.629705797	0.01383	0.66186779	0.53665
CBLN4	4.073352458	0.05446	5.19363485	0.01387	-1.167242415	0.61794
DNER	0.891082082	0.360281	2.258550964	0.01387	-0.726315097	0.51664
SPATA2L	-0.083412315	0.640061	-0.449450748	0.01387	-0.211412452	0.23698
TRIM3	-0.215531822	0.331755	-0.547787696	0.01385	-0.494569718	0.02623
TTC5	-0.159958734	0.327226	-0.399879014	0.01385	-0.205964191	0.20442
RASA4CP	-0.238281112	0.456868	-0.792286189	0.0139	-0.125244066	0.69343
CLK4	-0.052202916	0.767671	0.429070921	0.01392	-0.10640647	0.54591
LPHN2	0.006048012	0.963827	0.327264498	0.01397	-0.179753897	0.1778
ZNF285	-0.483090648	0.056032	-0.610846361	0.01397	-0.463338904	0.06461
RAD23B	0.090965375	0.491726	0.324898358	0.01398	-0.152624211	0.24884
CCDC88C	0.120006907	0.554955	0.496783292	0.01401	0.621262612	0.0021
SLC20A2	0.017959422	0.87163	0.270019408	0.01407	0.017707692	0.87353
ZNF362	0.202073342	0.365125	0.543609234	0.01411	0.096668562	0.66427
PCDHA5	0.747038664	0.243122	1.531677461	0.01413	0.922295877	0.14678
SUPT3H	0.079731574	0.569719	-0.344672029	0.01426	-0.068210789	0.62727
RAB11FIP5	0.039849586	0.811333	-0.411522192	0.0143	-0.010651262	0.94881
OXR1	-0.161020881	0.18414	-0.295777593	0.01434	-0.030994727	0.79757
ADAMTS10	0.52331624	0.116405	0.810778157	0.01438	0.363598322	0.27584
SRP54	0.092130047	0.303928	0.217700322	0.01441	-0.062282797	0.48734
HLA-DRB1	-0.249308038	0.640661	-1.308506743	0.01442	-2.182912551	4.75E-05
RSAD2	0.102195087	0.84743	1.232591939	0.01445	0.463815054	0.36568
NDUFB9	-0.006726532	0.946805	-0.246380177	0.01448	-0.203284089	0.04432
BEND7	-0.021230015	0.918805	0.49693288	0.01451	0.109847412	0.59044
TSPAN14	-0.133984254	0.398486	-0.387182602	0.0145	0.043350539	0.78372
BTN3A2	-0.174621848	0.253716	-0.374409834	0.01455	-0.135757989	0.37262
CD276	0.212147708	0.08275	0.297893328	0.01454	0.040157117	0.74275
CLTA	0.102341939	0.139919	0.167848627	0.01455	-0.017427848	0.80146
NCMAP	-0.061648172	0.741931	-0.458889931	0.01454	0.026641173	0.88628
P11-255H23	-0.265318122	0.202288	-0.508120537	0.01454	-0.135041751	0.51464
ADCY1	0.389809883	0.35927	1.02572687	0.01457	0.12532241	0.76942
MAGED4B	-0.081774473	0.6598	-0.454061309	0.01459	-0.295812735	0.11169
SMARCB1	0.020497203	0.822841	0.216979848	0.01459	0.034988514	0.69958
TD-2132N18	-3.502433251	0.089565	-5.324188032	0.01463	-0.370845856	0.84869
FAM84A	-0.275257867	0.351484	-0.722087327	0.01462	-0.754542394	0.01101
RHBDL1	-0.744324062	0.058733	-0.966489351	0.01463	-0.885114431	0.02571
SERPINB6	-0.107999759	0.438455	-0.340180195	0.01463	-0.296274464	0.03358
REEP2	0.261470299	0.504925	0.900537481	0.01468	0.064618638	0.87295
AGT	0.152738887	0.792349	-1.420227899	0.01471	-1.652110563	0.00462
MAT2A	-0.149605768	0.216197	-0.294813613	0.01472	-0.069092695	0.56756
PSMA1	-0.181724365	0.053997	-0.228986692	0.01472	-0.345310137	0.00026
TMOD2	-0.251953976	0.421566	0.747885511	0.01469	0.420412011	0.17569
CCNDBP1	-0.145009816	0.265991	-0.316747814	0.01474	0.00522397	0.96782
HOXA6	0.358608783	0.405964	1.023070044	0.01476	0.556622657	0.19178
PIGV	-0.092366398	0.462176	-0.306560314	0.01477	-0.097335899	0.43467

DDRGK1	0.14575428	0.11753	-0.226156495	0.01479	-0.068138218	0.4652
NUMBL	0.483064422	0.081336	0.670364149	0.01482	0.327402223	0.23736
RPS12P26	0.560023083	0.272268	1.16278049	0.01484	1.426416567	0.00264
SYT15	0.417805214	0.073801	0.5646166	0.01485	0.768647828	0.00094
CBLB	-0.134864774	0.417832	0.401829501	0.01492	0.21155044	0.20133
PIGX	-0.137756075	0.212558	0.26331748	0.01491	-0.226455762	0.04042
FAT2	-1.047300554	0.22585	1.992826249	0.01495	-0.569513496	0.50824
VSTM4	-0.01078449	0.96535	0.591434908	0.01499	0.660521037	0.00668
SCARB2	-0.102496863	0.348887	-0.266038646	0.015	-0.128653405	0.23946
HRH1	0.279387503	0.482435	0.949155032	0.01502	0.067208267	0.86614
C1orf115	-0.414001931	0.320182	-1.014860312	0.01506	-1.344459841	0.00135
PLXDC1	-0.470780587	0.075421	-0.645107556	0.01506	-0.467673565	0.07693
TNPO3	-0.039332865	0.805664	-0.388890779	0.01505	-0.066633105	0.67659
ACCS	-0.261552298	0.340222	-0.660527809	0.01513	0.167679656	0.53524
CDC42EP5	-0.115669434	0.841644	-1.459608897	0.01512	-1.645028138	0.00729
MXRA7	0.069720487	0.560618	0.289286349	0.01514	-0.324500852	0.00694
TM9SF4	0.147207311	0.090249	-0.211361782	0.01515	-0.101564226	0.24308
ASAP1	0.361104671	0.063277	0.470125587	0.01518	0.494395341	0.0108
MRPS36	-0.121783312	0.494131	-0.429991031	0.01519	-0.375226867	0.0356
ZBED5	0.028615608	0.748168	0.212513885	0.01527	-0.020614208	0.81626
ADARB2	-0.22627238	0.700822	1.321443505	0.01528	0.491835625	0.37993
ZNF502	-0.304040533	0.119439	-0.464176962	0.01531	-0.055123651	0.7624
C18orf8	0.083807799	0.55652	0.340620567	0.01538	0.289800316	0.04077
RNF183	0.461950765	0.059847	-0.606430986	0.01542	0.464842798	0.05757
TMEM198B	0.224092757	0.148795	-0.383713687	0.01542	0.15034585	0.33185
DNPH1	-0.076102078	0.67922	-0.447650025	0.01543	-0.175219031	0.34071
CCNH	-0.018381718	0.871631	0.271008102	0.01549	-0.074103403	0.51263
NEIL3	-0.240226644	0.104698	0.341593793	0.01551	-0.153410207	0.29455
TGOLN2	0.048093396	0.741003	-0.352288596	0.01556	0.171442134	0.23837
HIP1	0.114172282	0.685152	0.678527685	0.01558	-0.10276023	0.71536
PTRH2	0.100145788	0.511306	-0.36919563	0.01557	-0.182190611	0.2323
CACNA1G	0.233048613	0.510871	0.850367917	0.01562	0.517395286	0.14213
BET1	-0.107071262	0.447619	-0.339349464	0.01565	-0.293241191	0.03737
PTPRA	0.137054385	0.377913	0.374817439	0.01567	0.068858116	0.65766
ZNF491	-0.064699729	0.844257	0.770169546	0.01568	-0.080581099	0.80904
P11-335G2(	0.717428971	0.541663	2.50227299	0.0157	1.890746403	0.07663
NUP98	0.206404409	0.053738	0.258251305	0.01571	0.078155002	0.46531
AEBP1	0.279250449	0.221013	0.549883293	0.01574	0.197306894	0.38706
COL22A1	0.749983517	0.185198	1.330156177	0.01573	-0.069508335	0.90488
HUS1	-0.055226885	0.682203	-0.324901284	0.01574	0.178069128	0.18169
NAGPA	-0.370859892	0.120525	-0.574752604	0.01575	-0.351231501	0.13818
AC002398.9	0.23509669	0.59502	-1.106980818	0.01576	-0.767793648	0.09136
TPM1	0.223934924	0.099097	0.327473762	0.01581	-0.067363821	0.61999
AKAP7	-0.480761227	0.317578	-1.161107776	0.01584	-1.3752581	0.00436
SUMF1	-0.036520162	0.72589	-0.25028561	0.01583	-0.017610936	0.86519
HECTD2	0.053983991	0.731306	0.375080142	0.01587	-0.02861201	0.85547
LNX1	-0.071994843	0.700418	-0.452435026	0.01589	-0.362388328	0.05305

TAF7	0.032618694	0.70861	-0.2102903	0.01592	-0.14674743	0.09302
GSKIP	-0.241047354	0.162504	-0.414682927	0.01595	-0.413376555	0.01659
SUMO3	0.10166402	0.272475	0.221684438	0.01596	-0.079632135	0.39096
AQR	0.070393521	0.561736	0.29139463	0.01599	-0.016442865	0.89209
GSTM4	0.012797948	0.953525	-0.532269035	0.016	-0.380014647	0.08591
STOML2	-0.068802213	0.49445	-0.242263831	0.01605	-0.289750293	0.00415
FJX1	0.511089222	0.172155	0.891037694	0.01606	-0.58053336	0.13396
DACT1	0.490799011	0.174976	0.868403611	0.01612	-0.505148141	0.16421
HNRNPUL1	0.051518824	0.458443	0.166100912	0.01617	0.085565175	0.21739
PRELID2	-0.118959546	0.542068	-0.469324153	0.01623	-0.082328071	0.67261
GPR146	0.049043671	0.906704	-1.09140361	0.01627	-0.28006678	0.51277
ZC3H3	0.193275566	0.26754	-0.425602552	0.01635	0.375076095	0.02898
POFUT2	0.022229186	0.906957	-0.456722757	0.01637	0.000990541	0.99584
TMEM132C	0.457617947	0.646245	2.302681798	0.01639	-0.736344104	0.48222
LEPROT	0.223140406	0.488959	0.772464824	0.0164	0.434425103	0.17766
TMEM120A	-0.087371179	0.663755	-0.484382664	0.01641	-0.212057041	0.2952
AIP	0.212405686	0.067125	-0.281137464	0.01643	-0.139761198	0.23564
PSMB5	-0.044963295	0.690766	-0.270964784	0.01643	-0.511545208	6.77E-06
SERPINA5	0.191895354	0.457044	-0.619574236	0.01646	-0.190640841	0.46018
SLC6A17	0.514724746	0.598389	2.105620394	0.01645	0.590411846	0.53793
TNNI3	-0.348322978	0.174718	-0.617320493	0.01646	-1.117744315	5.51E-05
PEX5	-0.140720113	0.327116	-0.344074324	0.01648	-0.140165358	0.32709
SORL1	-0.427540589	0.084797	-0.594569119	0.01652	-0.502511371	0.04279
SUPT6H	0.069561408	0.380975	0.189229263	0.01652	0.217339881	0.00605
KIAA1919	-0.206358027	0.061951	-0.262325806	0.01653	0.108965775	0.3153
GAREML	0.548148627	0.153109	0.913996434	0.0166	0.687987388	0.07241
KCNN3	0.810636329	0.051006	0.991549035	0.01662	1.101200727	0.00848
LHFPL2	0.124613449	0.587544	0.548136532	0.01664	-0.012888534	0.9553
ZCCHC10	0.222691472	0.095633	0.317481838	0.01668	0.007598526	0.95478
TXNDC9	-0.201340747	0.059735	-0.253118703	0.01671	-0.239994334	0.02408
FOXE1	-0.916869114	0.22388	1.747817384	0.01679	-0.761175522	0.30965
WHSC1L1	0.007351186	0.94138	0.237778507	0.01685	0.082662311	0.40647
ISOC2	0.167178306	0.194183	-0.310113125	0.01689	0.015886791	0.90193
NPRL2	-0.001804891	0.99312	-0.499501847	0.0169	-0.120403385	0.5644
GRID1	0.368947141	0.501105	1.232471945	0.01694	1.202435463	0.02103
SHROOM1	0.32163226	0.400109	-0.92191994	0.01695	0.292670879	0.44396
C16orf93	-0.225834673	0.501312	-0.822901755	0.01696	-0.305359067	0.36268
TUBA1B	0.121580247	0.407729	0.350497153	0.01697	-0.210730567	0.15135
INTS5	-0.053226734	0.694085	-0.322772325	0.01703	0.036569182	0.78535
SKP1	0.074862758	0.317623	0.17782486	0.01705	-0.208626351	0.00537
AKAP2	0.351347868	0.117198	0.534447896	0.01705	-0.00681685	0.97578
NXN	0.236255191	0.301421	0.541844275	0.0171	-0.210152715	0.35904
RPL31	0.054767347	0.662462	-0.299254487	0.01711	-0.168800807	0.17854
PLXNA4	0.450282862	0.212563	0.84728458	0.01714	1.281528161	0.0003
ZNF521	0.424294864	0.290646	0.954512055	0.01714	0.148809091	0.71109
CASP9	-0.296712091	0.163092	-0.507481789	0.01716	-0.206857058	0.32832
GALNT2	0.029027187	0.788762	-0.258259058	0.01716	-0.08570907	0.42903

ZNRF1	0.260107009	0.086518	0.357667157	0.01716	0.293955786	0.05173
YIPF2	0.055872363	0.715398	-0.365353137	0.01719	-0.328589162	0.03214
FUT9	-0.724823659	0.170484	-1.260279492	0.01724	-0.049339084	0.92559
PELO	0.001814623	0.991646	-0.412623123	0.01725	-0.414779572	0.01697
PPAPDC1B	0.10987001	0.378549	0.292671625	0.01724	0.196388623	0.11353
POLR1B	-0.201413142	0.271419	-0.435816975	0.01728	0.066200278	0.71745
RNF121	-0.080905514	0.554686	-0.324805417	0.0173	-0.146189203	0.28511
C1S5-TXNI	-0.433304594	0.100188	-0.627005861	0.01732	-0.080273739	0.76017
TSPAN31	0.065255263	0.605583	-0.300036604	0.01737	-0.162429418	0.19707
ST7L	-0.004992656	0.976443	0.39552885	0.01743	-0.079862338	0.63583
STK11	-0.189947862	0.085661	-0.261809515	0.01743	-0.160899853	0.14329
TRIO	0.425209644	0.094149	0.603578674	0.01743	0.039095979	0.87769
SMARCC2	0.098326359	0.424015	0.291087191	0.01746	0.435329821	0.00038
UBTD2	0.145772933	0.097829	0.20753081	0.01746	0.058796051	0.50385
PDIA6	-0.083883977	0.397668	-0.235570701	0.0175	-0.247517239	0.01258
CLRN3	-0.480791978	0.377563	-1.298216002	0.0176	-1.907068521	0.00054
COL6A4P2	-0.004905799	0.993499	1.323955949	0.01761	0.189375464	0.75172
MTND4P12	0.490430864	0.054194	-0.608674193	0.0176	0.569891848	0.02511
MTMR1	-0.086246282	0.544863	0.336341395	0.01763	0.117531904	0.4082
CCDC71	0.046905975	0.666889	-0.258882913	0.01766	-0.057309732	0.59811
NTNG1	0.981919108	0.071194	1.284390261	0.01766	0.001920669	0.99722
RP11-47I22.	-0.411716519	0.071162	0.524857339	0.01767	0.456288836	0.03994
KLHL36	0.094277711	0.27815	-0.205787459	0.01769	0.250440635	0.00356
P2RY11	-0.117735101	0.570515	-0.494823131	0.0177	-0.099227713	0.63115
NDUFA7	0.012663077	0.918577	-0.294235262	0.01772	-0.174994262	0.15878
GLTP	0.056286428	0.590859	0.244110546	0.01773	-0.137292723	0.19018
RHPN1	0.09010263	0.730707	-0.626419718	0.01776	-0.052952636	0.83964
KLF4	0.201302445	0.506681	0.716217582	0.01777	1.11507589	0.00022
CCDC78	-0.247782197	0.462134	-0.81269798	0.01782	-0.339091204	0.31409
TLE1	0.25017593	0.105517	0.365100629	0.01783	-0.027396863	0.85947
GFOD1	0.260374795	0.313107	0.60169104	0.01784	0.097488137	0.70595
SERTAD4	0.362529237	0.371173	0.950808351	0.01786	-0.341808097	0.40518
SPAG4	0.29570606	0.279442	-0.658511931	0.01786	0.121508131	0.6574
IFIT5	0.307606756	0.276063	0.664454281	0.01787	-0.12708028	0.65415
LZTS3	0.102058253	0.516272	-0.374510282	0.01788	-0.133560595	0.39674
NRBP1	-0.085923539	0.38465	-0.233825286	0.01794	-0.254205828	0.01029
SMIM14	-0.228097268	0.144329	-0.369741705	0.01796	-0.09488566	0.54336
DNMT3A	0.158575529	0.297743	0.359206553	0.01797	0.093278632	0.53993
RHOA	-0.084364877	0.13337	0.132294003	0.01799	-0.205671407	0.00025
SH3TC2	-0.447592195	0.250058	-0.93916966	0.01799	-0.850247455	0.03092
BAX	0.008718342	0.965398	-0.476842513	0.01802	-0.361318956	0.07275
PLA2G12A	-0.361426468	0.053156	-0.440906886	0.01803	-0.502822875	0.00697
ARID3A	-0.121463324	0.562711	-0.496240088	0.01804	-0.15227264	0.46774
CYP2S1	0.135825386	0.558305	0.545861674	0.01806	0.807409604	0.00047
COL27A1	-0.137330988	0.347622	-0.345713294	0.01809	0.238118814	0.10287
DPYSL2	0.136686122	0.156194	-0.227928211	0.01809	0.043215626	0.65387
SENP6	-0.078620157	0.241077	0.157278526	0.01809	0.121355273	0.06926

NPLOC4	0.110495624	0.222817	0.213560606	0.01811	0.143192077	0.11359
SIPA1L2	-0.063355936	0.731125	0.434598972	0.01812	-0.039041883	0.83234
PLEKHD1	0.311107784	0.264494	0.635342011	0.01814	0.883167074	0.00099
SORCS3	2.633398546	0.087672	3.59695269	0.01815	-1.177825324	0.46951
KHK	-0.580291209	0.164684	-0.991440899	0.01817	-0.766363136	0.06838
SPOCK1	0.904649098	0.212815	1.711286603	0.01821	0.381554007	0.60045
R3HDM1	-0.19895347	0.219247	0.378093965	0.01822	0.291860814	0.06983
CAMKK2	-0.048304591	0.667189	-0.264259401	0.01824	-0.02086671	0.85206
DEF6	-0.008247476	0.965996	-0.461613945	0.01827	0.005715989	0.97616
HLA-C	0.279214418	0.068573	-0.36235542	0.01826	-0.065517025	0.66939
CCDC47	0.131437087	0.28758	0.290726165	0.01828	0.04686959	0.70447
ATF2	0.032372263	0.782144	0.275268002	0.01832	-0.068787479	0.55674
MPST	-0.001294632	0.993691	-0.386089813	0.01835	0.040808368	0.80246
NRGN	0.618636754	0.096544	0.857918812	0.01838	0.421889892	0.25991
BEST1	0.269511395	0.254263	0.550828256	0.01842	0.511994277	0.0282
MAML3	0.005907911	0.982081	0.618388753	0.01842	0.303827528	0.24721
ETNK2	0.315788326	0.176755	0.546161882	0.01844	0.135990619	0.56276
MGEA5	0.098557977	0.286854	0.216896292	0.01844	0.172841322	0.06107
AKAP6	0.066001866	0.837528	0.744644588	0.01846	0.786015591	0.01319
PDIA4	-0.007045232	0.954745	-0.292508284	0.01847	-0.160654253	0.19566
MLST8	-0.113517437	0.356757	0.286890593	0.01851	-0.194666475	0.11376
FKBP10	-0.016808203	0.919248	-0.390571481	0.01852	-0.404573185	0.01476
ITSN1	0.192466161	0.087078	0.264172561	0.01852	0.106187769	0.34494
CDC42SE1	0.083027739	0.416387	-0.240599566	0.01858	-0.25531575	0.01258
MT-ND5	0.230090312	0.440794	-0.702817017	0.01858	0.127070447	0.67031
NPTX1	0.682141026	0.360475	1.741645113	0.0186	0.212353422	0.77785
SS18L1	-0.208550551	0.19777	-0.379458183	0.01866	-0.005497603	0.97271
SUSD2	0.17998885	0.712364	-1.178457993	0.01871	-0.424150521	0.38728
GPRC5B	0.202920793	0.361285	0.520627281	0.01873	0.673917436	0.00234
FBXO28	-0.060047929	0.571547	-0.249203887	0.01875	-0.207486231	0.05069
HEY2	0.30903187	0.671783	1.60090791	0.01878	-0.013392943	0.98554
MPHOSPH8	-0.256139606	0.071945	-0.333628527	0.01878	-0.005839458	0.96718
RP1-130L23.[illegible]	0.286405879	0.640573	1.305260368	0.01877	0.378809634	0.52755
RRS1	-0.127303815	0.513545	-0.458315701	0.01878	-0.169456886	0.38382
FAP	-0.181859354	0.755899	1.346759036	0.01885	-0.015133156	0.9793
ARHGAP20	0.946899058	0.134187	1.472939341	0.01894	-0.337442686	0.59808
LAPTM4A	-0.091328604	0.466052	0.293336083	0.01893	-0.432379882	0.00057
TSNARE1	0.134494931	0.505023	-0.47586841	0.019	0.226176699	0.25777
PDZD2	0.055623776	0.811309	0.542153367	0.01903	0.518789915	0.02492
KCNK3	0.412610658	0.282459	0.871280978	0.01904	0.638099949	0.09075
NUPL1	0.146226987	0.07089	0.189232844	0.01904	0.079486188	0.32617
APLNR	-0.037292459	0.972994	2.460019571	0.01908	-0.574261285	0.61242
IGDCC4	0.364264983	0.131441	0.562989374	0.01911	0.232780416	0.33507
RPS16P5	1.507559478	0.090968	2.000266362	0.01912	2.126649588	0.01284
SIRT1	0.242665952	0.062736	0.303426248	0.01913	0.064606955	0.62081
MAB21L1	0.771268637	0.37531	1.975236773	0.01914	0.643693096	0.45934
CCND1	-0.104145706	0.571141	-0.430252121	0.01925	-0.147282294	0.42299

ANTD3-TME	-0.1506669	0.598316	0.659658913	0.01926	-0.493630681	0.08571
RFNG	-0.314506307	0.119487	-0.470165062	0.01927	-0.340360829	0.08933
GOLGB1	-0.033147272	0.612564	-0.152956585	0.0193	-0.162975303	0.01275
HSPBAP1	-0.099513087	0.704843	0.585766924	0.01936	0.252140755	0.32707
TMC7	0.066055297	0.754064	-0.494172162	0.01937	0.035842393	0.86484
PHB	0.001434984	0.991343	-0.309007084	0.01939	-0.200428698	0.13021
DDX46	0.193958035	0.108883	0.281877722	0.01941	0.174576257	0.14876
PTMA	0.061426533	0.557309	0.244543972	0.01942	-0.275111438	0.00859
TLK2	-0.035735905	0.725081	0.235564736	0.01942	-0.09076603	0.37081
CDC14B	-0.149796027	0.272733	-0.318366095	0.01951	-0.050047294	0.71306
LARP7	-0.150113237	0.386395	0.403218573	0.01952	-0.316729858	0.06768
MAD2L1BP	0.147490343	0.182286	-0.259700034	0.01951	-0.151309046	0.17543
MTURN	0.343289806	0.193466	0.613181279	0.01952	-0.021572247	0.93503
SLC25A15	-0.26500174	0.058932	-0.324407261	0.01951	-0.214524869	0.12132
WDR83OS	0.089524145	0.501358	-0.311620338	0.01952	-0.257688833	0.05387
PPARGC1E	-0.183379567	0.416757	-0.527611226	0.01958	0.6561525	0.00348
FAM105B	-0.00596973	0.972504	-0.403318402	0.01962	-0.114531029	0.50716
DLL1	0.482423066	0.115858	0.69631035	0.01965	0.383303697	0.21059
SDPR	0.032342813	0.932187	-0.889640282	0.01966	-0.54026472	0.15609
SLCO2B1	-0.460658573	0.252637	-0.939830486	0.01972	-0.60054498	0.13586
AC079807.3	0.404656078	0.48455	1.230069871	0.0198	1.346979522	0.01091
BMP3	0.450778418	0.141709	0.713740686	0.01982	0.903288363	0.00319
NCOA5	-0.04562245	0.741901	-0.322637933	0.01986	-0.250920101	0.07039
SFRP4	1.784727685	0.23082	3.367563229	0.01989	1.584395666	0.28892
ANLN	-0.281298245	0.168024	0.473964159	0.01997	-0.315131295	0.12236
AP1S3	0.196585159	0.237075	0.382903187	0.02001	0.275325432	0.09519
APBB2	2.10E-05	0.99991	0.429738971	0.02001	0.372826034	0.0441
ESCO1	0.100613206	0.289052	-0.219532221	0.02012	0.184596277	0.05083
EYA2	-0.798017713	0.362006	1.969265579	0.02019	-0.515399321	0.55348
ACTR5	0.018334207	0.917905	-0.416315318	0.0202	-0.006640928	0.97007
ECI2	-0.027287198	0.836021	-0.306089204	0.0202	-0.370774051	0.00503
PXMP4	-0.369939277	0.062586	-0.457950702	0.02025	-0.344247421	0.08061
ZNF43	0.093137966	0.577311	0.384231428	0.02029	0.110690187	0.50745
SCG3	0.428224311	0.259508	0.823325764	0.02032	0.866105774	0.01563
CEBPZ-AS1	0.410597642	0.14757	0.651674497	0.02037	1.698437664	9.51E-10
TRMT10A	-0.19384958	0.349494	-0.479742639	0.02039	-0.586609475	0.00476
RNF180	0.257643907	0.242937	0.500380816	0.02041	0.180605527	0.41321
NSDHL	-0.112583824	0.366815	-0.289083775	0.02046	-0.301733895	0.01572
ERAL1	0.185935077	0.207164	-0.344597082	0.0205	-0.01314655	0.92906
SOX4	-0.167876744	0.250275	0.337362394	0.0205	-0.114540778	0.43243
PRSS16	0.33705119	0.25157	-0.716778343	0.02055	0.342856158	0.23456
MDC1	0.265517385	0.226483	0.503943505	0.02063	0.515251828	0.01839
ZNF333	-0.057108904	0.764877	0.43660297	0.02062	0.192965409	0.30912
ARHGEF5	0.099524132	0.535721	-0.37268695	0.02066	0.114521592	0.47549
C12orf5	-0.358041154	0.08437	-0.478698651	0.02066	-0.263345989	0.20353
ANP32A	-0.039986562	0.706419	0.244520555	0.02073	-0.282460244	0.00792
HOXA3	0.486944726	0.199918	0.867984117	0.02074	0.120240558	0.75331

KRT18	-0.190126617	0.158275	-0.311588466	0.02074	-0.533670949	7.52E-05
SCUBE3	0.338562489	0.417586	0.963214175	0.02075	0.121550445	0.77112
NDUFA1	-0.135991099	0.124314	-0.202212639	0.02076	-0.442899443	7.32E-07
RALGPS2	-0.165819671	0.239181	-0.325312956	0.02077	0.105526261	0.45281
SEMA3F	0.151258259	0.562979	0.599459196	0.02078	0.07799355	0.76477
CCDC61	0.170016842	0.538304	-0.661208257	0.02081	0.177622193	0.51548
CHKB-CPT1	0.270338097	0.15104	0.433918755	0.02082	0.378553367	0.04338
KLF12	0.373834374	0.107066	0.535183494	0.02084	0.35212649	0.12884
RPS13	0.056509881	0.609009	-0.255294603	0.02086	-0.074756717	0.49864
TSSC4	-0.01238658	0.957143	-0.534592807	0.02089	-0.458372008	0.04767
IFI27L2	-0.073097536	0.643293	-0.362626904	0.02091	-0.42366564	0.00847
UBXN10	-0.62553122	0.064352	-0.772756877	0.02093	-0.8300688	0.01465
GPT2	-0.285240582	0.094997	-0.393762295	0.02097	-0.107289611	0.52907
ANP32E	0.263668256	0.279874	0.560798294	0.02099	-0.258677397	0.28886
PTMS	0.051740746	0.759495	0.388518656	0.02099	-0.055797651	0.7413
SLC25A1P5	-0.302630148	0.474543	-1.041390845	0.02101	-0.126671512	0.75549
DICER1	0.058277434	0.659801	0.305119478	0.02107	0.089031327	0.5011
EMP2	0.090333327	0.687136	0.514416214	0.02107	-0.15429771	0.49126
EM189-UBE	-1.060437042	0.715714	-7.099289779	0.02117	-0.284127197	0.9222
DDX6	0.075846875	0.43362	0.222335077	0.0212	0.230590678	0.01711
EGFL6	2.453253905	0.066834	3.068966909	0.02121	4.082520753	0.00212
SEMA3D	-0.330625998	0.093799	0.451186256	0.02124	-0.631670899	0.00141
DDX5	0.108322526	0.245676	0.214805245	0.02126	-0.306043006	0.00105
FAR2P1	0.447056547	0.072365	0.567183971	0.0213	1.00690329	4.17E-05
TWF2	0.012447862	0.931847	-0.336179996	0.0213	-0.269154779	0.06658
SNAI1	0.146445439	0.505343	0.494254477	0.02131	0.477513433	0.02695
CDKL2	-0.609648019	0.229929	-1.159687359	0.02134	-1.29204474	0.01613
NTRK1	1.243124575	0.210656	2.219066148	0.02136	-0.198141945	0.84563
GALK1	0.205956948	0.232502	-0.401806778	0.02142	-0.083930116	0.62628
RPL7P41	0.284100515	0.712647	1.568996748	0.02143	0.560832731	0.44796
LEMD2	-0.196238372	0.10934	-0.280626932	0.02146	-0.212599136	0.08157
SSTR2	0.008954235	0.965736	0.464670997	0.02147	0.557661615	0.00588
C11orf84	-0.067121935	0.570044	-0.271159388	0.02149	-0.491791961	3.85E-05
DMRT3	-0.954373919	0.47874	2.683147827	0.0215	-0.690313532	0.61615
PPP2R4	-0.040094483	0.703186	-0.242443264	0.0215	-0.118336593	0.26195
B3GALT1	0.453615828	0.26015	0.908789957	0.02156	0.155764223	0.70062
HSPH1	0.034145376	0.88342	0.534893884	0.02157	-0.768736015	0.00099
PDZRN3	0.258152779	0.396841	0.698537283	0.02156	0.206143441	0.49948
POLK	-0.08273677	0.428667	0.233528343	0.02154	0.224000389	0.02922
P11-571F15	0.364794163	0.483408	1.095089341	0.02158	0.006471015	0.99033
SNN	0.136240437	0.57199	0.549801725	0.02154	-0.237944011	0.32549
SPIRE1	0.218095512	0.389117	0.574189967	0.02157	0.048161788	0.84983
PPP4R1L	-0.02139331	0.927997	0.535391255	0.02159	0.07465969	0.75219
RAB3IL1	0.752205728	0.174725	1.262560087	0.02161	0.249557209	0.65687
SY1-RAB43	-0.129563699	0.469298	-0.411214724	0.02165	-0.516417841	0.00401
DPH7	-0.196050483	0.211027	-0.354381552	0.02171	-0.162006665	0.29575
NUP107	-0.033772484	0.769178	0.261966215	0.02173	-0.111646155	0.33186

EFNA2	0.172256174	0.463467	-0.55496229	0.02175	-0.076716179	0.74577
ECE2	0.039770938	0.850974	-0.494184465	0.02176	-0.198229519	0.35248
KIAA2022	0.726881079	0.371091	-1.91173016	0.02182	-0.164453531	0.83983
ARHGEF40	0.361932416	0.07168	0.46022821	0.02186	0.007537108	0.97013
CXorf36	-0.049428511	0.900817	0.847638502	0.02189	0.685924779	0.06654
CYP4F3	0.005596038	0.992885	-1.46030585	0.02187	-0.330461562	0.59397
GLT8D1	-0.206722186	0.091427	-0.280240984	0.02188	-0.079816342	0.51396
SLC25A5P1	0.720371877	0.42724	-2.410056324	0.02185	0.363396249	0.6902
NME5	-0.375507409	0.259764	-0.77362891	0.02191	-0.589929364	0.08857
CEACAM7	-0.352301156	0.521846	-1.262038793	0.02195	-0.807110394	0.14255
GABRA3	0.623394406	0.266044	1.251235886	0.02196	0.864138223	0.12175
WDR20	-0.105222762	0.388948	-0.279246042	0.02194	-0.263277625	0.0312
FAM104A	-0.169228127	0.259846	-0.340972117	0.02198	-0.406091663	0.00658
POLR2J	-0.086913603	0.484543	-0.283400069	0.02198	-0.347146803	0.00565
NAPEPLD	-0.495301275	0.060376	-0.604548904	0.02202	-0.231256409	0.37858
AP2B1	0.657559418	0.135472	0.996557587	0.02207	0.664658627	0.12994
RNF10	-0.01850023	0.903596	0.348352534	0.02207	0.509622459	0.00081
RAP1GAP	0.126715336	0.595261	-0.550149398	0.02208	-0.054556884	0.81904
AL353671.3	0.146642856	0.689809	-0.855291055	0.02214	-0.416694247	0.26126
NUDT11	0.073135568	0.789664	0.617489054	0.02218	-0.191909852	0.4855
P11-423E7	1.26488408	0.097055	1.680465105	0.02218	1.284588775	0.08894
GTF3C5	-0.180893098	0.092221	-0.24546808	0.0222	-0.171628056	0.11006
E2F7	0.158919224	0.551385	0.607516929	0.02233	0.216384246	0.41774
RND2	0.127548202	0.609242	0.544011117	0.02234	-0.207685289	0.42257
ATRIP	-0.006628219	0.980308	0.606941925	0.02244	-0.083278945	0.75872
FAM110C	0.479124113	0.056044	0.56609772	0.02249	0.497037249	0.04642
RPS20P22	0.46417474	0.306639	0.990019883	0.02248	0.779637493	0.0768
TMEM238	0.517606233	0.19241	0.869008865	0.02247	0.61102419	0.11767
LZTS2	-0.169050739	0.207544	-0.305214847	0.0225	-0.027145535	0.83877
P11-430L17	0.685080465	0.222521	1.208187497	0.02251	1.097882297	0.04138
NLE1	-0.12174029	0.520192	-0.431677396	0.02252	-0.180711854	0.33904
SRSF11	-0.040367396	0.671682	0.216392001	0.02259	-0.168876338	0.07579
PGLS	0.116274072	0.315776	-0.265190624	0.02261	0.008742394	0.93978
NUDT4	-0.203195168	0.075768	-0.260496812	0.02262	-0.232966668	0.04156
OR7D2	-0.104658273	0.67458	0.530720342	0.02271	0.775969493	0.00081
CLMN	0.140668189	0.493998	-0.468876403	0.02275	0.207570832	0.31195
RPS6KA1	-0.022919152	0.887064	-0.36851084	0.02274	-0.001048634	0.99481
VAMP2	0.292886979	0.121176	-0.439516347	0.02274	0.410242825	0.02775
EPHB6	0.997932412	0.244109	1.893444124	0.02279	-0.263582379	0.76142
BMP5	0.08875382	0.789831	0.748556495	0.02281	-0.065788905	0.84347
MPP6	-0.363542378	0.092577	-0.490333471	0.02283	-0.369703942	0.08611
TSHZ3	0.055672329	0.832684	0.595459074	0.02282	0.140743511	0.59254
HEPH	-0.165334258	0.545547	-0.622475507	0.02288	-0.806495155	0.0032
EXOSC7	-0.078130228	0.581155	-0.320888948	0.02292	-0.035522814	0.80044
RHOT1P3	2.094741716	0.288498	4.336613361	0.02291	4.085334318	0.03253
HSPBP1	0.146432252	0.402642	0.390753273	0.02297	-0.07404604	0.67465
TIMM44	-0.086844334	0.390304	-0.229572137	0.02299	-0.2262128	0.02538

ZNF525	-0.279168626	0.147294	-0.436575548	0.02307	-0.077476427	0.68675
PSMA5	0.04742369	0.646097	-0.234061753	0.02309	-0.225819192	0.02887
TGIF2	0.138606934	0.610696	0.610630527	0.02312	0.171167705	0.526
ANO10	0.065965182	0.670585	0.348279174	0.02323	0.070721446	0.64704
PAQR5	0.021702624	0.952741	-0.834751105	0.02323	-1.110623481	0.00263
SH3BP5	0.050918509	0.838501	0.56224758	0.02327	-0.118426082	0.63573
FOXC2	1.115481831	0.063085	1.354204601	0.02336	0.599558127	0.32027
PALLD	0.121013384	0.580338	0.494715315	0.02337	0.203070462	0.35311
TMEM205	0.225785708	0.166349	-0.371447318	0.02341	-0.054708308	0.73785
ST8SIA4	0.657625534	0.096789	0.885367091	0.02345	-0.207676893	0.60176
CNTN4	0.051444298	0.86873	0.701488874	0.02349	-0.938204803	0.00269
AC004453.8	0.065825577	0.864195	-0.931424069	0.02353	-0.565181483	0.16005
PPP6R3	0.12385292	0.110865	0.175550956	0.02353	-0.058177518	0.4543
AMOT	-0.087070406	0.635409	0.414452129	0.02354	-0.118296733	0.51922
BAG6	0.086920018	0.499245	0.290512021	0.02359	0.267494543	0.03725
ELOVL2	0.386510556	0.419719	1.044656128	0.02359	-0.013252246	0.97826
SEL1L	-0.163351941	0.234859	-0.311203492	0.0236	-0.259422828	0.05916
FAM171B	0.484622414	0.243632	0.937904292	0.02363	-0.332545039	0.42575
SLC35F5	-0.155618266	0.240926	-0.300238643	0.02363	-0.360634483	0.00659
TP53I13	-0.140651646	0.410554	-0.385427097	0.02369	-0.038641236	0.8186
DDX24	0.223120771	0.196424	0.390098505	0.02372	-0.010530279	0.95141
STX7	-0.180183416	0.21734	-0.329852771	0.02378	-0.276872019	0.0577
TACC1	-0.318726844	0.083326	-0.415939158	0.0238	-0.158599339	0.3885
ZNF320	-0.21571133	0.109001	-0.30230297	0.02385	-0.000574436	0.99657
AKR1E2	-0.090878319	0.802063	-0.815827176	0.02391	-0.093669484	0.7964
ATP8B3	-0.146586006	0.637898	-0.707149852	0.0239	-0.189194312	0.54322
ZNF576	-0.171817817	0.298935	-0.371146743	0.02391	-0.337086774	0.04166
KLHL24	-0.168254999	0.367874	0.419506388	0.02396	0.027009532	0.88493
NDUFS4	-0.072332749	0.520566	-0.253638661	0.02397	-0.192024928	0.08808
TAPT1	0.170389034	0.230165	0.318676139	0.02397	0.325033416	0.02174
TP53I11	0.179122951	0.20519	0.318048718	0.02402	0.484454911	0.00058
CCDC85C	-0.046638963	0.758596	-0.342297079	0.02408	0.053443854	0.72418
DAAM1	0.20965975	0.218772	0.382879747	0.02408	0.364061592	0.03241
GPS1	-0.129522027	0.321937	-0.294507563	0.02406	-0.19979692	0.1263
LRRC55	0.792683533	0.22477	1.451691459	0.02406	-0.845145597	0.2197
GPR56	-0.18599529	0.437807	0.53802872	0.0241	0.555032682	0.01986
EIF3L	-0.002810991	0.977034	-0.220210971	0.02411	-0.171412692	0.07926
GPR173	0.520873194	0.217416	0.947354064	0.0242	1.300649375	0.00201
C1QTNF3	-0.00234451	0.995013	0.81834264	0.02426	0.070846392	0.84825
CMSS1	0.139180836	0.370059	0.342158035	0.02434	-0.167257522	0.28367
C1orf122	0.321812801	0.079668	-0.420672533	0.02436	0.033137115	0.85748
COX7A2	0.067271396	0.423347	-0.188891029	0.02438	-0.102775929	0.22181
MRPL28	0.028033456	0.840954	-0.3154449	0.02443	-0.406891515	0.00385
SLC25A19	-0.307275625	0.209368	-0.549757551	0.02449	-0.116147714	0.63103
ZNF202	-0.254042906	0.123889	-0.370340931	0.02456	-0.027800225	0.86571
AK9	-0.233092562	0.204395	-0.408964255	0.02459	-0.035793895	0.84414
TMED6	0.050952405	0.881052	-0.796030914	0.02461	0.057606749	0.86396

VIL1	-0.117225999	0.642252	-0.566963151	0.02464	-0.072086074	0.77508
SIX1	1.011294752	0.344149	2.3941122	0.02466	0.066864615	0.95032
C4orf47	-0.569797083	0.122215	-0.829148408	0.02468	-0.536004416	0.14228
GMPPB	-0.012593809	0.932547	-0.334265706	0.0247	0.058906845	0.69153
SNTB2	-0.021002227	0.83638	0.225724218	0.02472	0.23782719	0.01826
DCAF5	-0.153502072	0.075166	-0.192055394	0.02476	0.051141206	0.54969
EML4	0.011995667	0.867878	0.161317525	0.02474	-0.093901576	0.19281
SUGT1P3	-0.648026952	0.174765	-1.043666911	0.02477	-0.341571925	0.44653
ZNF274	0.24398837	0.097545	-0.332103165	0.02476	0.047592522	0.74685
ZNF16	-0.14730004	0.244552	-0.281268393	0.02479	-0.089931598	0.46932
HOXA9	-0.440945965	0.220589	0.787965694	0.02483	0.080706529	0.82032
SLC29A4	0.27810952	0.073579	-0.3501794	0.02486	0.25273309	0.1021
STX19	0.635218449	0.164616	0.99321657	0.02488	0.330069326	0.475
P11-512M8	-0.394719307	0.265399	0.71549379	0.0249	0.306781753	0.35215
DALRD3	0.150120875	0.308294	-0.333713488	0.02493	0.016174024	0.91259
CHD1L	-0.10203077	0.349555	0.242145468	0.02494	-0.009975621	0.92689
ARHGAP29	-0.12294108	0.448325	0.360608645	0.02496	0.021298098	0.89515
RPL7	0.08034476	0.480354	-0.255225193	0.02498	-0.167136993	0.14212
ALX3	-0.478136801	0.063097	-0.562242425	0.02501	-0.362217454	0.14687
FAM216A	-0.099407504	0.524814	0.330584474	0.02506	0.003943924	0.97931
FKBP5	0.200809805	0.35013	0.478819423	0.025	0.126786114	0.55479
MRPL18	-0.106841747	0.411218	-0.290629084	0.02506	-0.435135907	0.00088
SYNE4	0.017208967	0.951308	-0.635479389	0.02505	-0.206985314	0.46479
TACC2	-0.009072525	0.963492	0.440026252	0.02506	0.534902995	0.00655
TAGAP	0.935667424	0.072409	1.142617931	0.02502	0.991292429	0.05634
TCEAL7	0.277198234	0.336492	0.640832919	0.02507	-0.313193539	0.28107
TNFSF9	-0.263723191	0.521741	-0.929108072	0.02506	-1.207965685	0.00395
ZBTB3	-0.156115844	0.399162	-0.41331388	0.02505	-0.051273282	0.7793
CDCA5	-0.07781677	0.697364	-0.448215945	0.0251	-0.405017328	0.04315
RALGAPB	0.072923529	0.342343	0.170293452	0.02518	0.092513846	0.22606
LRPAP1	-0.0476861	0.717168	-0.294457137	0.02522	-0.174562958	0.18476
SCMH1	0.002109521	0.986477	0.274902687	0.02521	0.07185981	0.56056
SLC3A2	0.040109196	0.798009	-0.352003769	0.0252	-0.21756697	0.16597
UCHL1	0.689388639	0.078644	0.874073147	0.02524	-0.786171926	0.04934
COA3	-0.008471916	0.926776	-0.204843552	0.02531	-0.144874529	0.11596
ANKRD45	-6.31E-06	0.999983	0.641354722	0.02537	0.681768964	0.01773
LDHAL6A	0.044141358	0.932103	1.068362965	0.0254	0.605286393	0.21839
SNX33	-0.252951946	0.082188	-0.323528786	0.02541	0.0311312	0.82894
DPCR1	0.773441151	0.148838	1.192704424	0.02544	1.504936402	0.00479
UBE2L3	-0.052009523	0.576225	0.20651415	0.02546	-0.115382128	0.21461
BRCA2	-0.206434585	0.437246	0.591978984	0.02548	0.242308128	0.36129
SIGIRR	0.09232048	0.548996	-0.347665515	0.02549	0.116754546	0.44785
UBIAD1	-0.026030599	0.851464	-0.310520032	0.02548	0.069032894	0.61779
SUPT4H1	-0.110512004	0.306476	-0.240367782	0.02552	-0.479519901	1.01E-05
FBXO46	0.088811793	0.64635	0.42669925	0.02554	0.487645301	0.01127
KRT10	-0.053299995	0.715663	-0.326570949	0.02555	-0.236382628	0.10715
GDPGP1	0.237370018	0.177889	0.388781544	0.02558	0.693790429	6.42E-05

P11-333E13	1.003698494	0.100986	1.322954726	0.0256	1.773569073	0.00246
C9orf142	-0.245355803	0.206227	-0.433159301	0.02565	-0.403072772	0.03864
RPS2	0.245420317	0.115486	-0.34795251	0.02566	0.076939589	0.6217
ENAH	-0.130913586	0.327086	0.297199206	0.02573	-0.291246949	0.02922
KIF21B	0.257596902	0.21	0.45145043	0.02572	0.822606407	4.15E-05
MAPK11	0.572455594	0.154158	0.877611034	0.02572	-0.208471787	0.61481
PAK1IP1	-0.046790291	0.697444	-0.267943734	0.02573	-0.329824042	0.0064
QRSL1	-0.006807204	0.952173	0.24971338	0.02576	0.105682475	0.34913
ZNF776	0.00752771	0.938023	0.213493318	0.02579	0.246220935	0.01047
MRRF	-0.048169856	0.708472	-0.28673597	0.02583	-0.077600028	0.54531
SIAE	0.022904969	0.870332	-0.312809364	0.02584	-0.2598363	0.0643
SYT10	-0.661480061	0.411283	1.686856129	0.02587	-0.919264455	0.25441
FAM114A1	-0.153733594	0.274138	-0.313016177	0.02588	-0.281191578	0.04545
MIIP	-0.195289511	0.332173	-0.446226611	0.0259	-0.530733118	0.00898
PTPRD	0.349280105	0.14426	0.5318641	0.0259	0.851625275	0.00036
CYB5R1	0.015656085	0.915805	-0.330641701	0.02591	0.067146786	0.64769
EGLN3	0.306124832	0.209677	0.543041372	0.02592	0.651827738	0.00751
NOL3	-0.353036272	0.212147	-0.63289306	0.02598	-0.102924516	0.71234
BCL7C	-0.051150854	0.691179	-0.286379879	0.02602	-0.269593514	0.03696
CYP24A1	-1.451898976	0.387183	3.02597796	0.02601	0.254730835	0.86221
HAPLN3	0.45576339	0.23082	0.832866767	0.02601	0.703830055	0.06438
HSPA12A	0.285573534	0.393604	0.73450474	0.02602	-0.032433176	0.9231
ANKRD26	-0.178062127	0.280082	0.36371561	0.02609	-0.127558438	0.4382
HOXB4	0.112221456	0.686249	0.604499563	0.0261	-0.12707028	0.64875
MT-CYB	0.415329089	0.083802	-0.534661721	0.02611	0.592388044	0.01364
ROMO1	0.111808063	0.346701	-0.264306087	0.02618	-0.078355878	0.51169
TMEM125	-0.268903321	0.338546	-0.627136334	0.02618	-0.483921509	0.08397
EHBP1L1	0.291049934	0.076777	0.36378855	0.02621	0.386985066	0.01816
MACF1	0.105891348	0.375623	0.265532284	0.02622	0.113300675	0.34303
MIPEP	-0.287041692	0.091049	-0.375957719	0.02632	-0.295581407	0.0812
ARHGAP4	-0.31773437	0.304436	-0.699767962	0.02635	-0.273833734	0.37549
WDR17	-0.476633758	0.180971	0.755554736	0.02634	0.400171493	0.24471
NIPAL3	0.055468069	0.626275	-0.255142864	0.02643	-0.152336258	0.18278
RBMX2	-0.067717836	0.665571	-0.348215606	0.02643	-0.258242189	0.10013
SAMD5	-0.319504813	0.313673	-0.704163939	0.02643	-0.214502786	0.49863
ISYNA1	0.095210271	0.513074	0.321453219	0.02648	-0.057379387	0.69348
C11orf24	-0.077113136	0.534261	-0.274385399	0.02652	-0.119600486	0.33496
THAP7	-0.132936745	0.426636	-0.369746093	0.0266	-0.390556156	0.01999
URB2	0.076652753	0.654352	-0.380425561	0.0267	0.055073407	0.74757
ABHD17B	-0.022650102	0.847458	-0.260155484	0.02674	-0.039064575	0.73873
SOX7	0.28542923	0.568811	1.049149542	0.02673	0.441201312	0.37265
TAOK2	0.0349283	0.718256	-0.214338785	0.02674	0.048270735	0.61713
TMEM242	-0.25285298	0.064288	-0.299408864	0.02672	-0.031552642	0.81502
C16orf54	0.81232993	0.059628	0.919345851	0.02677	1.443613127	0.00035
NUDT10	0.207102084	0.640943	0.957103854	0.02677	0.067864697	0.87855
MAPK15	-0.494664373	0.071411	-0.610462342	0.02684	-0.222210449	0.41143
P11-977G19	-0.103846703	0.830939	-1.082300562	0.02685	-0.95578229	0.05065

BRINP1	0.311138196	0.627775	1.400308175	0.02691	0.162864969	0.7996
C8G	-0.4689138	0.593542	-2.110817822	0.02693	-1.458514534	0.11796
PRRT3	0.231292082	0.488033	0.705315704	0.02695	0.646163451	0.04534
C16orf72	-0.024786839	0.754046	0.171539089	0.02698	-0.088600347	0.26127
MMP14	0.330302608	0.053739	0.378401726	0.02697	0.111419066	0.51535
LRP5	0.060108174	0.691147	-0.33474913	0.02703	0.251042153	0.09653
MYH14	0.265494237	0.373592	0.658859142	0.02708	0.561543042	0.05965
PLEKHB1	0.149614958	0.680928	-0.806961863	0.02708	0.006345465	0.98609
TD-2302E22	0.229534047	0.678471	1.102793654	0.02711	0.788248388	0.12701
DENND1A	-0.205781869	0.178928	-0.337572827	0.02714	0.030263594	0.84221
PTPN23	-0.29348282	0.235597	-0.545855881	0.02716	0.291077566	0.23423
LZIC	-0.139594552	0.131461	-0.200837597	0.02718	-0.114651281	0.21215
P4-583P15.	-0.07841503	0.876308	-1.134237377	0.02726	-0.531077324	0.29521
MGAT3	-0.130830828	0.361058	0.312383813	0.02728	0.249300414	0.07912
TM2D3	0.101696353	0.296184	-0.215207449	0.02728	0.061487406	0.52572
ALKBH4	-0.213872098	0.282607	-0.434254357	0.02731	-0.361950098	0.06724
NDRG2	-0.03304871	0.867945	0.434245371	0.02736	0.199608021	0.31336
AVL9	-0.065591008	0.581954	-0.262172833	0.02742	0.047349004	0.69037
ACSS3	0.023893959	0.954115	0.909524474	0.02744	-0.294390118	0.47903
SAR1B	0.008531179	0.93365	-0.225940146	0.02746	0.158692803	0.12087
FNDC5	0.354105711	0.473619	1.07819742	0.02749	0.773319991	0.1163
MCF2	1.391509588	0.097736	1.777274913	0.02748	1.128360254	0.17188
ZEB2	0.211339186	0.433901	0.593975124	0.02749	0.023623932	0.93035
RABL6	-0.143772082	0.116609	-0.200940399	0.02757	-0.100744825	0.2698
RPL17	0.00919329	0.940641	-0.272025798	0.02758	-0.220444074	0.0742
ATP2C1	-0.119954064	0.227179	-0.217911039	0.02761	-0.135175761	0.17211
MET	-0.106931372	0.59294	-0.440472022	0.02764	-0.24459246	0.22147
SKA1	-0.154577912	0.716226	0.923630197	0.02765	0.408222492	0.33083
SORCS2	0.661165331	0.210548	1.158552535	0.02766	0.250220335	0.6356
TDP2	-0.538777189	0.107112	-0.736240877	0.02767	-0.677312471	0.04284
KLHDC8B	0.010438536	0.960782	-0.468987959	0.02769	-0.815229151	0.00015
C10orf107	0.762525686	0.197209	1.252190312	0.02772	0.189916146	0.74741
FANCG	-0.349214092	0.073926	-0.427923795	0.02774	-0.335553994	0.08589
TAS1R3	-0.234963319	0.389808	-0.606317125	0.02777	0.197441768	0.44557
CD300A	-0.060393508	0.842112	-0.669636788	0.02782	-0.033771065	0.91123
ERI1	-0.185645812	0.109193	-0.251996983	0.02785	-0.035583244	0.7584
IGBP1	-0.057292004	0.62347	-0.256344935	0.02785	-0.279191609	0.01692
ZFYVE28	-0.048454889	0.857637	-0.595757046	0.02788	0.115929675	0.66566
NAV3	-0.110607663	0.545138	0.398835866	0.02793	0.532571192	0.00339
RGL3	0.013688157	0.934936	-0.370720352	0.02795	-0.074848525	0.65451
COPZ1	0.004909366	0.954893	-0.190679217	0.02799	-0.130452355	0.13305
OMD	0.979170869	0.281799	1.966894003	0.028	1.3277172	0.14137
C7orf73	-0.089281755	0.32021	0.191996046	0.02802	0.029551832	0.73914
SLC16A12	0.547564004	0.297811	1.147800586	0.02806	0.66048964	0.20429
MTMR3	0.118825503	0.263464	0.233136858	0.02808	0.164854343	0.12005
PPP1R1C	-0.135340608	0.618136	0.569549575	0.02809	-0.225529459	0.40287
ZC2HC1A	0.129646749	0.49258	0.410504563	0.02811	-0.07809068	0.67961

SLFN13	0.210177757	0.485376	0.65906115	0.02823	0.873460245	0.00361
ART4	-0.287625538	0.696824	1.536979829	0.02826	0.958227261	0.17553
BDH1	-0.391137023	0.105393	-0.524221443	0.02826	-0.155604211	0.51308
SPAG1	0.309884934	0.313893	0.672196782	0.02826	0.219738454	0.47667
RAB38	-0.377696618	0.179295	-0.616020446	0.02829	-0.905507104	0.00147
IQSEC2	-0.230614392	0.450844	-0.66768206	0.02834	-0.230673272	0.43318
P11-262H14	0.419963698	0.543786	1.382948038	0.02834	1.656384214	0.00826
ATAD3C	-0.134755556	0.644773	-0.641565787	0.02835	0.218262818	0.44591
ZNF511	-0.083660847	0.511964	-0.27947955	0.02842	-0.412555215	0.00138
ANO9	0.255906979	0.132169	-0.374827904	0.02847	-0.0675236	0.69315
EME2	-0.159135288	0.24307	-0.29836616	0.02845	0.16437763	0.22442
FAM135A	-0.147291133	0.34012	-0.338189602	0.02847	0.154674954	0.31573
STK33	-0.106621603	0.678319	0.553763379	0.0285	0.305733261	0.22709
DAG1	0.154804023	0.283898	0.315880187	0.02853	0.535160833	0.00021
EPHB2	-0.039530226	0.821172	0.380940985	0.02852	-0.19940472	0.25365
SPRYD3	0.006254814	0.96134	-0.283631586	0.02852	0.054187288	0.67438
TMEM40	-0.086602585	0.883863	1.260985709	0.02854	0.312530024	0.59504
MPZL2	0.120742181	0.417525	0.325250348	0.02862	0.037745432	0.79993
CPA3	0.827265545	0.204901	1.403452134	0.02864	1.735604126	0.00644
YAF2	0.04837954	0.777572	0.369841845	0.02866	-0.13784797	0.41866
PDK2	0.179382602	0.397398	0.458149964	0.02868	-0.039504788	0.85366
ARHGAP35	-0.103675399	0.560732	0.388765066	0.02875	0.144373499	0.41691
POTEI	-0.034046996	0.824748	0.332705037	0.02875	0.359935855	0.01818
MRPL46	0.089267105	0.45143	-0.259445793	0.02881	-0.249650262	0.03598
AKAP11	0.030619976	0.681987	-0.162898445	0.02887	0.111556185	0.13324
BICD2	-0.158788996	0.139232	-0.233894321	0.02887	-0.196905731	0.06607
CHFR	0.028067444	0.829548	0.282523467	0.02891	0.29699814	0.02175
ANGPT4	0.221811734	0.65499	-1.091377041	0.02893	0.406145237	0.4127
NUP54	-0.00874702	0.933913	0.229747358	0.02892	-0.034768065	0.74178
BCL6B	0.067548074	0.954545	2.492857008	0.02895	0.019331143	0.98702
UPK1B	0.133859896	0.596994	0.552739042	0.02897	0.083744873	0.7408
TFCP2	-0.164760377	0.194138	-0.275859514	0.02899	0.091743544	0.46469
CLASP2	-0.036662713	0.770395	0.272070111	0.02904	0.138634965	0.26763
MMADHC	-0.091813371	0.308709	-0.196145039	0.02905	-0.277776727	0.0021
DRD2	-0.615376355	0.346215	-1.48941573	0.02906	0.423028642	0.48467
RNF157	0.369002914	0.141969	0.546684743	0.02913	-0.040106836	0.87312
TXNDC16	0.081336914	0.603379	0.336633741	0.02914	-0.073272157	0.63994
KDELR2	0.109623149	0.235015	-0.201414425	0.02916	-0.003410334	0.97051
RNASEH2A	-0.192577521	0.143051	-0.284291578	0.02916	-0.284282404	0.03063
PTPRB	-0.43362075	0.083898	-0.545847452	0.02918	-0.476819145	0.0571
AAMP	0.044214375	0.72785	-0.276775146	0.02923	-0.277149331	0.0294
FAM188B	-0.255644902	0.222157	-0.453852853	0.02925	-0.080610072	0.69451
OLFML2A	0.152664857	0.716212	0.904599921	0.02925	0.271086269	0.51703
PGM1	-0.090930655	0.379271	-0.225109426	0.02929	-0.04048846	0.69493
P11-767N6	0.119216061	0.730214	0.696276845	0.02936	0.827743258	0.00973
RGS6	0.388155168	0.553201	1.388700778	0.02938	0.157122358	0.8136
TOMM40L	-0.173731802	0.296221	-0.361586021	0.02943	-0.410558867	0.01408

CSPG4P12	0.22249488	0.314179	0.477285178	0.02946	0.579270665	0.00825
MST1L	-0.513946386	0.288288	-1.063715335	0.02945	-0.105667992	0.82565
AGAP1	0.093586225	0.554314	0.338937414	0.02948	0.054171636	0.72893
DENND4B	-0.163823508	0.321192	-0.359526104	0.0295	0.051621304	0.75306
EAF2	0.003390681	0.990667	-0.65178613	0.02952	-0.088403274	0.75709
VAT1	-0.027119228	0.851073	-0.314665044	0.02954	-0.392930449	0.00667
KRT8	-0.1422814	0.214565	-0.249330295	0.02962	-0.397268328	0.00053
RP11-98J23.	-0.351791509	0.473947	-1.082181397	0.02962	-0.447168164	0.36235
HLA-DRB5	-0.182340499	0.791187	-1.50474663	0.02967	-2.56684973	0.00025
NTF3	0.867962443	0.248048	1.571942954	0.02969	0.275265125	0.72307
ZNF536	0.406985049	0.465056	1.173799438	0.0297	0.648839116	0.23466
RASL11A	-0.246303953	0.314547	-0.535938224	0.02971	0.303223533	0.20298
TPI1	-0.093198955	0.381503	-0.231467499	0.02975	-0.316245922	0.00299
EPC1	0.031566376	0.837224	0.332532636	0.0298	-0.002694635	0.98599
MYO1B	0.160110657	0.162898	0.248550443	0.02979	-0.290828995	0.01166
RGPD8	-0.520121641	0.109372	-0.703376113	0.02978	-0.378042378	0.24232
P11-347C12	-0.218744669	0.52653	-0.758544334	0.0298	0.248208938	0.45082
OBSCN	-0.044804332	0.788005	-0.36191927	0.02981	-0.013068518	0.93737
LTV1	-0.166533706	0.219086	-0.293549848	0.02983	-0.334522829	0.01364
COLGALT2	0.234182881	0.566522	0.870750927	0.02988	0.220379676	0.5858
ZNF571	-0.205031355	0.165895	-0.317285501	0.0299	-0.134724151	0.35637
U2AF1	0.305197092	0.130241	0.435832861	0.02993	0.252910096	0.20813
ENC1	-0.115321647	0.462238	-0.340392891	0.03005	-0.104570769	0.50486
CDRT1	1.01259092	0.098309	1.294081233	0.03008	1.082778133	0.07617
RXFP2	2.788082671	0.073766	3.367855255	0.03015	-1.921658772	0.29215
CDC25A	-0.256976987	0.127318	-0.364530588	0.03022	-0.407738238	0.01588
NDST4	0.615144299	0.724436	3.516822721	0.03023	2.804639867	0.0868
SLC16A2	0.423019579	0.139512	0.61682923	0.03025	0.089586246	0.75467
CHCHD5	0.324978315	0.079009	-0.408080781	0.03032	0.029234754	0.87479
DOK5	1.003144218	0.38394	2.397561944	0.03033	1.094096903	0.33942
FOXP2	0.266983681	0.381037	0.659299524	0.03033	0.160882059	0.59759
SMIM12	-0.05826747	0.593265	-0.234429901	0.03032	-0.268536263	0.01357
CKB	-0.281475227	0.565728	-1.061460838	0.03034	-0.998542561	0.04163
SNRPGP18	-0.28996816	0.696357	1.361483599	0.03035	1.997666413	0.00118
KAT6A	-0.014126935	0.877479	0.197525016	0.03037	0.097460424	0.28695
PCDHA4	0.117316602	0.492307	-0.371747568	0.03041	-0.27926718	0.10476
GLI1	0.152456494	0.682458	0.803343361	0.03042	0.091236074	0.80662
MID1	0.24663783	0.223666	0.434370095	0.03045	0.18672674	0.35549
NBEAL1	0.052925901	0.671401	0.269402781	0.03044	0.36846772	0.00308
DNAJB5	0.362497299	0.083655	0.448993839	0.03053	-0.175940959	0.40729
NBPF3	-0.029279469	0.842525	-0.31656314	0.03052	-0.138573701	0.34432
NCSTN	0.131225875	0.301346	-0.274974089	0.03054	-0.04433207	0.72735
PA2G4P4	-1.048465172	0.177002	-1.751570556	0.03062	0.516375898	0.46104
CLDN11	0.650215757	0.319112	1.406687017	0.03068	0.306919611	0.63865
COL21A1	0.601730773	0.481962	1.844481727	0.03075	-1.02601626	0.23324
NAGA	0.054214811	0.543504	-0.191940788	0.03072	-0.118576794	0.18554
PDE3A	0.103438987	0.56859	0.390219518	0.03075	-0.241421067	0.18417

SLC23A1	-0.523399664	0.339048	1.043164058	0.03074	1.022858837	0.04018
ARNTL2	0.317491608	0.100536	0.415814998	0.03083	0.303135337	0.11595
CCDC103	-0.405567123	0.487792	-1.319342165	0.03081	-0.498798632	0.39346
EMC6	0.002096678	0.987553	-0.290548972	0.03082	-0.39312844	0.00414
GRIA1	0.214852903	0.420376	0.574629713	0.03079	0.622379012	0.01986
KIF9	0.053996278	0.793354	-0.444584535	0.03082	-0.281711733	0.17332
RP11-3J10.7	-1.146960014	0.09938	1.405952103	0.03083	-0.598846428	0.3763
ZBTB26	-0.065300543	0.678718	0.328231694	0.03082	0.358370359	0.01905
CTSB	0.054508652	0.711183	-0.317693804	0.03101	-0.316926202	0.03142
GALM	-0.114835736	0.482256	-0.35284315	0.03101	-0.168623429	0.30195
LMOD3	-0.046425553	0.922459	0.944385944	0.031	0.863585484	0.05117
STX12	0.219610084	0.050599	0.24149489	0.03099	0.175538821	0.11794
ADAMTS3	0.355546035	0.335931	0.789683815	0.03111	-0.114385855	0.75833
EID2	-0.216834686	0.181437	-0.346271401	0.0311	-0.392960787	0.01575
ENTPD7	-0.099548909	0.340259	-0.223518834	0.03109	-0.020590847	0.8426
RPE	-0.13318436	0.189174	-0.217020537	0.0311	-0.08475274	0.4007
SLC25A1	0.013607669	0.924516	-0.309610946	0.0311	0.070850172	0.62126
UGT2B17	0.025381678	0.976337	-1.8938193	0.03112	-2.085948125	0.01874
NDUFS6	-0.187231204	0.191304	-0.306789937	0.03114	-0.315517273	0.02782
GNPAT	-0.143841065	0.148962	-0.213558979	0.03116	-0.144443172	0.14661
ERCC2	-0.063488601	0.677681	0.324774221	0.03119	-0.027958132	0.85331
VAMP7	-0.141764627	0.240682	-0.258098724	0.03119	-0.328414585	0.00649
THY1	0.442837876	0.053524	0.493525323	0.03125	-0.016764969	0.94188
NAGS	-0.761993091	0.065254	-0.879075065	0.03129	-0.879618782	0.03309
GPR111	0.346999022	0.422147	0.910113007	0.0313	0.94467621	0.02602
ASCC2	-0.121440084	0.328264	-0.265999733	0.03133	0.174682087	0.15729
HADHA	-0.015148447	0.848297	-0.170299308	0.03131	-0.073169118	0.35542
ZBTB25	0.213224489	0.188057	0.344935907	0.03133	0.27577631	0.08559
WWOX	0.115122137	0.566288	0.425037089	0.03134	0.286941047	0.15011
GYG2P1	-0.14593941	0.573153	0.533693047	0.03138	0.759150056	0.00214
SBDS	-0.060414398	0.536706	0.208191319	0.03145	-0.192979126	0.04869
TUSC1	-0.28644749	0.193076	-0.470866254	0.03146	-0.36300627	0.09806
DBR1	-0.178480641	0.302257	-0.372105005	0.03147	0.2346021	0.17256
ATF7IP	-0.144382773	0.198226	0.240412519	0.03152	0.255421792	0.02237
RPL27A	-0.0454934	0.690234	-0.245440013	0.03153	-0.238570786	0.03664
FOXN3	0.220463622	0.255928	0.415946746	0.03158	0.227338536	0.24148
S100G	0.600292009	0.500011	-2.042856473	0.03157	-2.579684193	0.00941
SLC26A4	0.154222999	0.802891	1.298688454	0.03156	0.810472169	0.18903
COX8A	0.164316228	0.273521	-0.323210371	0.03165	-0.100419514	0.50401
ACADVL	-0.002530475	0.982699	-0.250582202	0.03172	-0.113777398	0.32955
P4HA3	0.953088863	0.110005	1.225141414	0.03169	0.593703104	0.30514
RHBDD1	0.009079054	0.949377	-0.306511848	0.03172	-0.137719145	0.33535
TFEC	0.059015773	0.945257	-1.919237389	0.03172	-1.635099916	0.06842
RHOBTB2	0.313425353	0.073239	0.37334634	0.03173	0.391938297	0.02504
GLB1	-0.002763994	0.985896	-0.335927144	0.03176	0.087287408	0.57642
BET1L	0.019127994	0.880476	-0.27436396	0.0318	0.220365912	0.08206
CRISPLD1	0.044880938	0.89528	0.729322741	0.03182	-0.388759148	0.25504

MRTO4	0.083956656	0.515249	-0.276852778	0.03182	-0.075223224	0.55959
RARS	-0.09938437	0.184684	-0.160377615	0.03182	-0.21126775	0.00484
ARF1	0.116931343	0.254558	-0.220396868	0.03184	-0.072652284	0.47925
KDM5B	0.079603435	0.467298	0.234553874	0.03192	-0.056432453	0.60627
STK32B	-0.275747898	0.636144	1.17450218	0.03195	0.038370222	0.94614
ZNF768	-0.163809284	0.198895	-0.272405134	0.03198	-0.054224297	0.66913
SUB1	-0.036461613	0.675084	0.18580912	0.03199	-0.321831089	0.00022
CEP57L1	-0.013959395	0.943162	0.412868531	0.032	0.030294688	0.87705
POPDC3	0.410560534	0.555985	1.447460359	0.03202	-0.414435862	0.56325
C6orf136	-0.073035651	0.715859	-0.428338213	0.03208	0.093951874	0.63109
GEMIN4	0.022855061	0.898114	-0.382924853	0.03207	0.038462965	0.82912
LRRC37B	-0.287841692	0.110218	0.374217803	0.0321	0.288631403	0.10154
NOP58	0.202315985	0.056733	0.226983265	0.03214	0.072502715	0.49532
SPDEF	-0.209276332	0.659695	-1.024320137	0.03218	0.425948193	0.36751
C7orf63	0.490623449	0.061064	0.557902204	0.0322	0.733386382	0.0048
ECHDC1	-0.042175583	0.673591	-0.213030291	0.03221	-0.092027796	0.35683
GJA9	-0.256262898	0.739714	1.445323399	0.03221	0.681459841	0.33437
GPATCH8	0.144143178	0.320822	0.309775837	0.03223	0.597332606	3.43E-05
STAT4	-0.759839194	0.123412	-1.05455112	0.03226	-0.373729888	0.44193
C12orf49	0.043096189	0.734018	-0.271456824	0.03228	0.130634808	0.30199
TRAPPC3L	-0.252475903	0.264834	0.452213639	0.0323	0.771245521	0.00023
HYPK	-0.412801016	0.407476	-1.070656422	0.03231	0.035345206	0.94343
ZFP92	0.129443601	0.791941	0.937994396	0.03232	0.056003101	0.90834
CACNB2	0.392153018	0.231673	0.696573268	0.03236	0.37894691	0.24909
ZNF20	-0.402485666	0.134814	-0.567591845	0.03237	-0.198612757	0.45071
RSF1	-0.093386085	0.366566	0.220700046	0.03239	-0.136869033	0.18478
AC124914.3	0.907482836	0.161339	1.32819107	0.03247	1.259775578	0.04494
GSDMB	0.159892587	0.441704	0.441297783	0.03249	0.609878349	0.00312
XKR8	-0.201853021	0.348684	-0.459474001	0.03251	-0.250127109	0.2454
DCDC2	-0.392391591	0.353616	-0.908076929	0.03255	-1.076992901	0.01153
TUBBP5	-0.486863942	0.246074	-0.906792392	0.03254	-0.404110136	0.32718
HMGB3	-0.096507076	0.575186	0.36563391	0.03259	-0.24872428	0.14849
LTBP2	0.557419589	0.237754	1.005862342	0.03263	0.628405106	0.18317
MAP7D1	0.090752727	0.621345	0.391099145	0.03263	-0.096443074	0.59966
SLC5A3	-0.224977091	0.097324	-0.289599123	0.03265	-0.529200513	9.78E-05
UBE2H	0.148374128	0.292116	0.300786135	0.03264	0.021763743	0.87721
CD2BP2	-0.114752535	0.241807	-0.208708413	0.03266	-0.419835748	1.97E-05
P11-726G1	0.288812803	0.20268	0.47475491	0.03268	0.243875389	0.28002
KIF5B	-0.004697475	0.96065	-0.203125891	0.03276	-0.232663404	0.01455
P11-453N18	0.089792057	0.699505	0.473160335	0.03275	0.63185144	0.00434
ZRANB1	0.143637961	0.159914	0.216986414	0.0328	0.080291039	0.43249
IGFN1	1.050453931	0.364044	2.434811638	0.03286	0.090613408	0.93835
ARHGAP17	0.255752051	0.074139	0.304723842	0.03291	0.579676682	4.86E-05
P11-613M1(	-0.510420925	0.486582	-1.608346963	0.03293	0.130894859	0.8562
TGFBR1	0.194033172	0.142324	0.281099127	0.03291	-0.05663743	0.66846
GGA1	0.172264549	0.109186	0.228009969	0.03295	0.357271688	0.00084
PFDN1	0.051991278	0.637393	-0.234182623	0.03298	-0.246156807	0.02593

FAM89A	-0.114501594	0.669648	-0.575452712	0.03299	-0.438324805	0.10446
SECISBP2	-0.116958377	0.216768	0.199225551	0.033	-0.001652697	0.98601
GRHL1	0.310275426	0.076192	0.369933882	0.03301	0.297180909	0.08744
CCNB1IP1	-0.029868549	0.805262	-0.258262793	0.03303	-0.125181748	0.3019
ITGA11	0.371525874	0.122504	0.511354378	0.03307	0.763975326	0.00144
PFDN6	-0.041668987	0.728991	-0.254024704	0.03307	-0.172111814	0.15185
AC096921.2	-0.588429016	0.300287	-1.24176075	0.03309	-0.12716805	0.81732
CDHR5	-0.117330684	0.846863	-1.296178171	0.03311	-1.117891166	0.06621
FAM227A	-0.185336623	0.583325	0.715495113	0.03315	-0.005457546	0.98701
WDR54	0.035738185	0.829091	-0.355520962	0.03313	-0.297461148	0.07448
RBM23	0.074170538	0.476181	0.221071235	0.03318	-0.004608827	0.96466
RPS6KA5	0.450371471	0.092093	0.564593318	0.0332	0.424289557	0.11472
SYNPO2	0.406312092	0.079723	0.493420697	0.0332	-0.062712269	0.78704
TTPA	-0.61873856	0.06369	-0.700011963	0.0332	0.11430264	0.7197
JAZF1	0.070577641	0.720823	0.414972848	0.03325	0.348154732	0.07659
DDX56	0.123929355	0.342483	-0.278070337	0.03326	-0.050117463	0.70097
FTO	0.053356101	0.568705	0.196278669	0.03328	0.042296386	0.64997
IER5L	-0.083699413	0.758169	0.569883097	0.03333	-0.029555572	0.91311
ADAMTS7	0.412740422	0.110833	0.549262389	0.03334	0.36599381	0.15705
ZNF37BP	-0.013104443	0.947759	0.421602609	0.03337	0.115709003	0.56109
ULK2	0.376009946	0.075041	0.447164934	0.03341	0.44442816	0.03479
KPNA7	-0.263044208	0.487053	0.734038219	0.03342	0.08533805	0.81402
NKAIN2	0.197818742	0.852787	2.176699521	0.03347	-0.576008078	0.61917
BSG	0.128939914	0.281971	-0.254808488	0.03354	-0.12522943	0.29621
MR1	0.165186275	0.425718	0.436721775	0.03354	0.355416759	0.08385
ZBTB34	-0.020657539	0.795404	-0.168570774	0.03353	-0.168780134	0.03426
BRWD3	-0.118152652	0.375904	-0.28357277	0.03359	0.074063964	0.57835
CNTROB	0.180286891	0.304013	0.370374894	0.03357	0.253903473	0.14688
THRA	0.101277307	0.385491	-0.248023431	0.03365	0.209255409	0.0713
VPS18	-0.068403083	0.465814	-0.198570268	0.03367	-0.060097114	0.52017
TCP11L1	-0.029399008	0.84836	-0.326273766	0.03373	-0.245809345	0.11209
TSEN15	-0.123740232	0.283056	0.238483578	0.03373	-0.026486703	0.81699
CNN3	0.109182398	0.471106	0.321311315	0.03379	-0.193749508	0.20118
SSBP3	0.195179044	0.234468	0.346999868	0.03379	0.216147929	0.18709
ATP6V1A	-0.056548597	0.68921	-0.299771151	0.03383	-0.256018766	0.07024
CLPP	0.166358675	0.300952	0.338053145	0.03382	0.040287008	0.80242
EFR3A	-0.045227649	0.53938	0.154827827	0.03385	0.063402493	0.38719
HERC2P8	0.113035428	0.739358	0.714735581	0.03385	1.040265386	0.00199
HK1	-0.139401446	0.301267	0.285062285	0.03391	-0.071735099	0.59436
PAN2	-0.266571338	0.14337	-0.386527712	0.03391	-0.113536088	0.53296
SHPK	-0.016980656	0.870644	-0.220380415	0.03394	0.099376025	0.33512
NUDT3	0.092112274	0.260575	0.172464101	0.03398	0.189107695	0.02036
OGDH	0.03993131	0.696246	0.215532179	0.03404	0.200921308	0.04871
CRYL1	0.079259644	0.657655	-0.379765336	0.03409	-0.054578621	0.76027
PELI2	0.315736664	0.112054	0.420320528	0.03409	0.382540242	0.0542
SLC51B	-0.589869574	0.349526	-1.355267066	0.03408	-3.185213217	1.12E-05
NTM	-0.421045627	0.364477	0.958466791	0.03412	-0.653520524	0.16246

SYT8	-0.36257039	0.348212	-0.803576051	0.03412	0.079755506	0.83282
NAT14	0.122834749	0.433903	-0.337623916	0.03414	-0.098440022	0.53557
COG1	0.294449359	0.051631	0.318390636	0.03417	0.404454247	0.00725
GJA3	1.579533422	0.069246	1.810518891	0.03425	-0.431338341	0.66216
IRS2	0.245794214	0.327354	0.521395841	0.03425	0.356922141	0.15128
MLH1	-0.098106946	0.508979	0.312194626	0.03427	-0.166193673	0.26369
VPS35	-0.157235698	0.084119	-0.1920165	0.03427	-0.102303959	0.26024
ADAL	0.0026833	0.987533	0.348872948	0.03428	0.391940989	0.01818
AP5B1	-0.064074751	0.681597	-0.330386381	0.03431	0.154956674	0.31606
TMEM212	-0.026850255	0.899533	0.447466738	0.0343	0.764011188	0.0003
ARSK	-0.347600746	0.053532	-0.377849494	0.03434	-0.323277965	0.07293
RAMP1	0.218364225	0.374777	-0.524739821	0.03435	-0.132554132	0.5919
ID4	0.326862566	0.210289	0.549820776	0.03437	0.000151858	0.99954
WDR35	-0.245315364	0.056591	0.26525164	0.03443	-0.111096089	0.38661
TIAM2	-0.165376605	0.388029	0.39604207	0.03445	0.460703871	0.01465
PHF19	-0.052683336	0.813731	-0.474157476	0.03446	-0.268036058	0.23195
AC010980.2	0.664454158	0.09129	0.821698594	0.03449	0.613789387	0.11913
POLE4	-0.095857319	0.595784	-0.381988441	0.03449	-0.224886027	0.21198
ACP6	-0.195061541	0.317912	-0.41208914	0.03452	-0.233209381	0.22963
ECSIT	-0.057132125	0.738077	-0.36203829	0.03452	-0.182126528	0.28645
P11-402K9	0.957977024	0.078068	1.128900273	0.03453	0.956396799	0.0769
FGFBP1	-0.345303798	0.445461	0.947983376	0.03455	-0.495658183	0.27353
NOS3	-0.200674828	0.409144	-0.516186899	0.03456	-0.27204891	0.26528
RHOJ	0.555472681	0.051536	0.600885615	0.03461	0.34004338	0.234
WDR36	-0.023270285	0.86255	0.280787951	0.0346	-0.039318771	0.76953
STX1A	0.009378126	0.962903	-0.427209087	0.03467	-0.111054673	0.58274
CAPS	0.036076989	0.87578	-0.488008721	0.03469	-0.017469394	0.93959
GHDC	0.153878814	0.467215	-0.45419609	0.03471	-0.060260239	0.77678
PPIH	-0.204761807	0.052727	-0.218427209	0.03475	-0.357769819	0.00071
ZDHHC5	0.221889917	0.053022	-0.242275142	0.03475	0.186871265	0.10314
HCFC1R1	0.076411874	0.612291	-0.319334247	0.03476	-0.316098022	0.03781
TUBB2B	0.476498569	0.248751	0.868892246	0.03489	-0.672186109	0.1068
BTBD8	-0.264311116	0.459964	-0.766873841	0.03492	-0.382646814	0.28276
RPL9P30	1.085663246	0.10445	1.360682719	0.03499	1.889873163	0.00287
BMPER	0.827402086	0.190931	1.332336575	0.03501	0.105186074	0.86874
PUSL1	-0.135094829	0.473232	-0.396418284	0.03501	0.027643265	0.88131
MGMT	0.083498771	0.591648	-0.329921249	0.03504	-0.133655591	0.39192
APLF	0.015988432	0.918449	0.321735036	0.03513	0.014640078	0.92505
CELF2	0.475159898	0.054401	0.518870736	0.03513	0.44712049	0.06984
TRAPPC12	0.171046276	0.332125	0.368787023	0.03511	0.629960967	0.00031
ERCC6L2	-0.177542536	0.079649	0.211385398	0.03516	-0.164316073	0.104
KIAA1147	-0.136128685	0.104234	-0.175234953	0.03519	0.106175708	0.20037
TRIM41	0.020476171	0.845512	0.217876229	0.03519	0.051355206	0.62167
SPATS2	0.17838996	0.303308	0.363433156	0.03521	0.155821047	0.3681
OLFML1	0.186570111	0.598235	0.73846787	0.03527	0.119290428	0.73639
P11-34P13.	0.439627346	0.322144	-0.954052094	0.03527	0.77763337	0.07843
SEPHS1	-0.018902318	0.855246	0.216206475	0.03528	-0.140624526	0.17466

CALB2	-0.017549523	0.973921	-1.13252294	0.03533	-0.209061176	0.69708
CCDC87	0.060757692	0.881838	-0.947244085	0.03538	0.275732179	0.48347
FHL2	0.117251657	0.568587	-0.43330353	0.03539	-0.358666196	0.08167
RORB	1.194343595	0.13746	1.682712053	0.03539	0.088930712	0.91304
SLC51A	-0.362487318	0.430833	-0.969967171	0.03536	-1.380675061	0.00285
NOX1	-0.345213076	0.509527	-1.157135701	0.03542	-0.768180916	0.16775
EOGT	0.016371593	0.909927	0.291591292	0.03546	0.138904529	0.32628
CKAP2L	0.032669869	0.875199	0.433926568	0.03548	-0.037838772	0.85547
BACH2	0.410610258	0.499659	1.273116407	0.03552	-0.302339628	0.6202
CDK5RAP3	-0.15750912	0.242305	-0.282376348	0.03555	-0.184168245	0.17093
EVC	0.047641891	0.850388	0.526736632	0.03554	0.017498066	0.94474
PPM1A	-0.036520389	0.628375	0.156161304	0.03562	0.175150811	0.01905
CD74	-0.095703088	0.745708	-0.621040888	0.03567	-0.506674415	0.08699
NMI	0.137612494	0.547471	0.466359989	0.03568	-0.031458243	0.89083
PRSS8	-0.071749711	0.708463	-0.403285425	0.03568	-0.090014815	0.63888
TMEM243	0.122468664	0.337487	-0.273472173	0.03567	0.207563117	0.10077
MYO1E	-0.000517522	0.997185	-0.308257555	0.03572	-0.273186253	0.06301
FBXO30	-0.06078576	0.56828	-0.222717732	0.03579	-0.239629185	0.02476
ZBED2	-0.16450986	0.891812	2.487146574	0.03578	-0.673277216	0.58045
CCDC94	0.009611288	0.938086	-0.259929184	0.0358	-0.210645584	0.09062
SMYD2	0.126155329	0.352722	0.282679277	0.03586	0.042667745	0.75264
NT5C	-0.094997638	0.438569	-0.256630373	0.03588	-0.150960611	0.21762
TRAPPC6B	-0.142433319	0.286633	0.277771093	0.03591	-0.215844697	0.10586
CNIH3	0.1880035	0.530881	0.600509013	0.03592	0.676254068	0.01994
NANOGP1	0.904041129	0.052562	0.959288418	0.03593	0.959195195	0.03752
FEM1A	0.043460652	0.660982	-0.207704315	0.03597	0.012656037	0.89815
LRRC41	0.056216416	0.750775	0.365552003	0.03596	0.735994811	2.30E-05
TMCC2	0.452358854	0.288756	0.877161392	0.03597	-0.106456625	0.80721
AKR1A1	-0.041645833	0.646504	-0.189984986	0.03606	-0.184823031	0.04189
STAG3L1	0.378884224	0.296577	0.726138443	0.03616	0.910633635	0.00839
SIPA1L1	-0.172068087	0.226502	0.29656897	0.0362	0.152532314	0.28149
DZIP3	0.124234312	0.216748	-0.210901749	0.03621	-0.146186426	0.14793
ZNF668	-0.066334507	0.821886	-0.615994663	0.03623	-0.651910772	0.02689
ETNK1	0.021926224	0.883415	-0.313063999	0.03625	0.141164107	0.34478
HERC2P5	0.105034177	0.756989	0.704534923	0.0363	1.035637108	0.00206
HSPB8	0.646985308	0.108563	0.841921852	0.0363	-0.634719489	0.11947
ORAI2	0.208499059	0.423744	-0.547251857	0.0363	-0.429328055	0.10086
NAPA	0.009830172	0.927858	-0.226499786	0.03633	-0.154218884	0.15623
GRIPAP1	-0.159552716	0.200811	-0.259205702	0.03637	-0.151652811	0.22159
P11-687F6.	0.884527022	0.180022	1.31632121	0.0364	1.760772486	0.00453
ZDHHC11	0.215010684	0.527071	-0.720918638	0.0364	0.044557744	0.89542
COL1A2	0.181578088	0.602407	0.729065528	0.03645	-0.063686912	0.85503
SEPT7P2	-0.288625356	0.111591	0.369168069	0.03647	-0.238518515	0.18362
ATP2A3	0.02778332	0.914732	0.539828263	0.03649	0.588394894	0.02284
KRTCAP3	-0.15162704	0.419159	-0.393784994	0.03651	-0.151556652	0.41815
EPGN	1.476950198	0.349072	3.189197278	0.03656	-1.690970432	0.34915
CDH3	-0.322022727	0.056675	-0.353277172	0.03657	-0.548168723	0.00119

CLEC2A	-0.930368125	0.164282	-1.439474923	0.03659	-0.200378279	0.7413
ZNF154	0.099072766	0.521889	0.319649507	0.03665	0.360525344	0.01867
AC004797.1	0.080117575	0.682903	0.405427616	0.03675	-0.284541758	0.14899
PCDH20	0.02963267	0.898326	-0.486372513	0.03675	-0.260462241	0.26273
PIK3CB	-0.219149111	0.440479	-0.592709408	0.03675	0.747645243	0.008
P11-104O19	-0.43644038	0.14553	0.557903689	0.03669	0.374598861	0.1706
P11-713H12	-0.000595075	0.99954	1.946842686	0.03676	1.30666215	0.17039
THRAP3	0.003609214	0.965787	0.175273717	0.03673	0.030662418	0.71557
LIN37	0.152838787	0.608842	0.6078384	0.03688	0.245433688	0.40807
TRMT12	0.100571913	0.517952	-0.322196791	0.03688	0.048994249	0.75197
CCDC171	0.436625311	0.178382	0.672203826	0.03694	0.218403653	0.50175
FAM188A	-0.144488409	0.288531	-0.283002778	0.03694	-0.359367235	0.00848
LMNB2	-0.101338707	0.552144	0.35491976	0.03698	-0.105595013	0.5354
SCN11A	0.385799967	0.259579	0.679039634	0.03697	0.660092926	0.04568
TBC1D15	-0.065688963	0.636847	0.2869779	0.03699	0.051498146	0.7091
AIFM2	-0.022675449	0.930495	-0.546347538	0.03728	-0.012190234	0.96243
COA4	-0.130047417	0.293797	-0.257037001	0.03727	-0.201578979	0.10346
ISPD	-0.222663389	0.349959	0.477555407	0.03727	-0.175525056	0.45554
NOX4	0.084778623	0.838065	0.852370681	0.03726	-0.369762831	0.3741
SRSF3	0.229884158	0.084191	0.277019715	0.03725	-0.179358132	0.1784
PURG	0.67696066	0.109134	0.865990985	0.03733	0.537885017	0.20086
ABHD17C	-0.037131162	0.829314	-0.358683793	0.03737	0.104268703	0.54399
DDX55	-0.217731559	0.080913	-0.255373289	0.03744	-0.091627493	0.45753
LMO4	-0.12162629	0.255911	-0.222205202	0.03745	-0.084638093	0.42841
AIM1	-0.351569377	0.214732	0.584665395	0.03748	0.272325348	0.33369
ZRANB2	-0.190972792	0.064518	-0.213707989	0.03759	-0.010526861	0.91849
TMPPE	0.019046189	0.914022	-0.368595531	0.0376	0.3733508	0.02946
5P-PVRIG2	-0.065210531	0.754655	-0.435129811	0.03771	-0.194401254	0.35026
LCN12	0.003696523	0.991227	-0.7377265	0.03774	0.491081458	0.12982
MYO6	0.141954087	0.390168	-0.343358528	0.03773	0.221211647	0.18005
DNALI1	-0.246816661	0.112086	-0.32115946	0.03783	-0.718988076	3.68E-06
NUDT22	-0.161503925	0.231529	-0.279830489	0.03788	-0.063959734	0.63354
DNAJC30	-0.298350244	0.100505	-0.370264599	0.03793	0.013745391	0.93754
EPHA4	-0.096870567	0.758484	0.652936776	0.03794	-0.473766085	0.13288
OPRD1	0.557427923	0.165123	0.824175372	0.03795	0.629457556	0.11536
TRPM3	-0.572429272	0.312251	1.062022168	0.03795	0.7877429	0.1279
RPL32P29	-0.186024929	0.316124	-0.3838257	0.03799	-0.405372603	0.02952
OTUB2	0.101280736	0.593823	-0.398905858	0.038	0.135317421	0.47294
A1CF	-0.776835599	0.134189	-1.076832744	0.03804	-0.842363508	0.10443
AIFM3	-0.029640341	0.951296	-1.035824972	0.03804	-0.893892316	0.07562
FDXR	0.083731546	0.74516	-0.541067637	0.03807	-0.568569191	0.02866
TUSC2	-0.027059376	0.814185	-0.23825031	0.03807	-0.150669975	0.19066
ZNF443	-0.346088292	0.06549	-0.386324467	0.03815	-0.381428755	0.04194
DLX1	2.002370466	0.134115	2.755751043	0.03817	-0.946434587	0.50661
RP2	0.097478874	0.339413	-0.211714319	0.03821	-0.214010943	0.03736
NDUFA3	0.055243457	0.692021	-0.286840777	0.03824	-0.363500631	0.00978
TACR2	1.267970848	0.179175	1.889519116	0.03826	0.740548408	0.44301

YDJC	0.315641632	0.081812	-0.384462383	0.03831	0.136022162	0.45374
ACLY	-0.061314665	0.602446	-0.243535435	0.03844	-0.208216713	0.07691
ENTPD2	-0.704923478	0.212117	-1.198874305	0.03842	-0.356589882	0.51669
FAM153A	-0.588352715	0.233243	-1.024126031	0.03845	0.047424926	0.92236
SGTB	0.081174183	0.615082	0.327308935	0.03843	0.193761867	0.22617
TMEM140	-0.028306346	0.843269	-0.294768821	0.03843	0.276834543	0.0493
DDX53	1.027068696	0.230465	1.697620418	0.0385	2.392602059	0.00306
MYO3B	0.51990923	0.099749	0.642451483	0.03853	0.492728163	0.11695
NUDCD1	-0.064500458	0.537413	-0.214668872	0.03853	-0.256491075	0.01429
C6orf226	0.456232338	0.395359	1.052838771	0.03859	0.185102977	0.733
LGALS3	-0.349887566	0.290532	-0.68478542	0.03859	-1.171873915	0.00041
CMTM7	-0.149981725	0.366433	-0.341219452	0.03862	-0.028467325	0.86221
SLC17A1	-0.670380446	0.341634	-1.521415244	0.03862	-1.894978126	0.01349
SMARCE1	-0.066209243	0.525055	0.21445296	0.03874	-0.387113434	0.0002
P4HTM	-0.043165483	0.791626	-0.338930787	0.03883	-0.202215496	0.21517
CAMKMT	-0.124071418	0.547838	-0.430908589	0.03885	0.217502795	0.28746
EDAR	-0.576539803	0.541229	1.802912329	0.03886	-0.645538426	0.49237
OIP5	-0.383215083	0.089056	-0.457031752	0.0389	-0.14197426	0.5192
ZMYND11	-0.001052927	0.994363	0.306422821	0.03891	-0.203632322	0.17145
MYCL	0.1934811	0.53875	0.622738595	0.03892	-0.03720927	0.90579
RAB3GAP2	0.178937345	0.119198	0.236309103	0.03898	0.112833231	0.32501
METTL3	-0.157703964	0.070255	-0.177250186	0.03903	0.001785565	0.98343
UBXN1	0.004530895	0.975155	0.297947005	0.0391	-0.275211761	0.0594
GALNT3	-0.159854625	0.251972	-0.287710423	0.03912	-0.127081293	0.36215
SYNPO	0.5202115	0.190398	0.817489253	0.03923	0.3127233	0.43174
ZNF614	-0.054598099	0.68144	-0.273544272	0.03925	-0.267977287	0.04421
ADAMTS15	1.470582718	0.061728	1.620557277	0.03936	-0.203154633	0.79755
CDH26	-0.004496189	0.989149	0.678319399	0.03935	0.244112324	0.45953
TPRA1	0.185177375	0.134615	-0.257694908	0.0394	-0.09459783	0.4511
POLR2K	0.005266447	0.965071	0.244480994	0.03943	-0.162286753	0.17768
AAMDC	-0.078884616	0.601775	-0.310279793	0.03946	0.091188634	0.53812
ATP6V0B	-0.021682814	0.876827	-0.287953406	0.03949	-0.329957079	0.01877
GCLC	0.031699692	0.887881	0.461011229	0.0397	0.00778026	0.97239
ZBTB45P1	-0.691357043	0.322018	-1.453128034	0.03989	-0.254122713	0.71293
DTD2	0.031428255	0.796042	0.244839299	0.04002	0.047241307	0.69482
GPA33	-0.301896686	0.646562	-1.353527817	0.04	-1.926615001	0.00353
GPR141	0.895196767	0.226131	1.457120083	0.04002	1.776879713	0.01133
HAS2	0.087884897	0.867122	1.076069166	0.04001	-0.924377198	0.07951
RGAG1	0.13628163	0.782841	0.96446983	0.04002	0.324519915	0.49971
UTP3	0.157069013	0.230216	-0.269645905	0.04003	-0.106181344	0.41862
ATP5C1	0.040799923	0.736605	0.248438627	0.04007	-0.024596308	0.83928
POLDIP3	-0.113777784	0.159316	-0.165669592	0.0401	-0.234522738	0.00371
PGAP2	0.079157549	0.628889	-0.337953255	0.04017	-0.099639288	0.54222
MRPS33	-0.018325902	0.88147	-0.250921904	0.04019	-0.150532568	0.22009
TSHZ2	0.219383996	0.382926	0.51514068	0.04021	-0.231779142	0.35699
NCAM1	0.331670267	0.41449	0.832881581	0.04023	0.319513734	0.43164
ZBTB2	-0.11791657	0.204636	-0.18871552	0.04027	-0.234611401	0.01155

TLE6	0.112552306	0.809024	-0.984385019	0.0403	-0.430204165	0.36102
IRS1	-0.137657753	0.347757	-0.300162011	0.04036	-0.062682529	0.66858
BFSP1	-0.222474769	0.355383	-0.492999736	0.04039	-0.611750912	0.01203
MMP16	0.199443201	0.63987	0.872393061	0.04039	-0.224559208	0.59883
GPN2	0.535779202	0.06145	0.586417117	0.04043	0.308941862	0.28032
GAS6	-0.088532805	0.702949	-0.478229849	0.04046	0.098317692	0.66931
ITPR2	-0.323273955	0.149047	0.456831661	0.04045	-0.187839958	0.40085
ANGPTL1	0.529322114	0.337496	1.1265918	0.04052	-0.002741563	0.99606
CTSO	0.064926594	0.710819	0.354459577	0.04048	-0.180088381	0.30529
TMEM106C	0.086455456	0.623748	-0.361022951	0.04051	-0.073430491	0.67678
ZBTB48	-0.126699195	0.532982	-0.417125801	0.04054	-0.343017467	0.0912
LNPEP	-0.081683392	0.340646	-0.174855215	0.04059	0.096180359	0.25942
DMTF1	0.066754544	0.58909	0.252087468	0.04068	0.063748405	0.60607
MMRN1	0.221442853	0.786823	1.614909437	0.04072	-0.396105568	0.62695
ITGAX	-0.441017587	0.314487	0.819170002	0.04076	1.159042573	0.00299
PCCA	0.003987498	0.979079	-0.310358891	0.04073	-0.020005174	0.89499
SAMD13	0.016848941	0.939807	-0.457735232	0.04077	0.105373345	0.63327
SEC22B	-0.086381536	0.327102	-0.179872643	0.04075	-0.113890831	0.19568
AUTS2	0.09944889	0.72352	0.570930466	0.04082	0.669222985	0.01648
TMEM14C	-0.150023141	0.111058	-0.190758631	0.04094	-0.245781503	0.00903
CERS5	0.124014867	0.390955	0.294142663	0.04097	0.428869939	0.0029
P11-49K24	-0.057462317	0.929987	1.161105296	0.04098	0.532147234	0.37755
APOBEC3B	0.24933471	0.323064	0.510315337	0.04103	-0.083079366	0.74309
MRPS25	-0.148914127	0.294926	-0.288682641	0.04107	-0.006536048	0.96301
BRIX1	-0.086586183	0.525091	-0.277720505	0.04112	-0.23523348	0.08411
PRKAR1A	0.008630263	0.944272	0.252027148	0.04112	-0.321974622	0.00914
RNF187	-0.135134033	0.244462	-0.236053713	0.04114	-0.262815416	0.02358
FAT3	0.759576571	0.28436	1.445464122	0.04125	-0.078918901	0.91152
NFIL3	0.328592929	0.150925	0.465229026	0.04126	0.365284666	0.10979
PPP2R5A	-0.152670392	0.157202	-0.218863283	0.04133	0.088791097	0.40636
NLGN4Y	1.252804887	0.054267	1.30279744	0.04135	1.235741678	0.05574
METTL21EP	0.458108297	0.414915	1.064188456	0.04138	1.55638415	0.00235
FLT1	0.286906572	0.440662	0.748765677	0.04141	-0.298395342	0.42648
F10	0.027052076	0.941827	-0.765133418	0.04154	-1.571034515	6.23E-05
TEF	-0.161356752	0.518641	-0.510267735	0.04155	-0.003930142	0.98742
PSIP1	-0.145136527	0.320483	0.296422959	0.04162	-0.239869013	0.10065
AMICA1	-0.281127711	0.368371	-0.63619939	0.04167	-0.319199629	0.30671
CD40	1.081679862	0.050642	1.120350697	0.04166	0.212548466	0.70875
SLC7A5	0.453610601	0.106316	0.571424074	0.04167	0.51362712	0.06733
ZNF689	-0.373185062	0.05376	-0.392429379	0.04174	-0.364881588	0.0584
ATF1	0.04084224	0.754408	-0.265131333	0.04176	-0.068119653	0.60215
CDC34	0.14623679	0.22607	-0.245800714	0.04177	-0.015376755	0.89886
FAM83E	-0.160237885	0.430777	-0.413684474	0.04177	0.19615731	0.3316
ATXN7L3	-0.093883453	0.371664	-0.213872292	0.04183	-0.0899416	0.39154
EIF2S1	-0.15214875	0.147208	-0.213114234	0.04182	-0.482574301	4.39E-06
OLA1P2	-0.384043019	0.433063	0.860615175	0.04185	0.641177026	0.1396
IP6K2	-0.073964017	0.366026	-0.165936204	0.04189	-0.215872105	0.00841

PLCB3	-0.171507197	0.10353	-0.212808953	0.04189	-0.0606185	0.56282
TNFRSF11A	-0.088106195	0.705183	-0.476359968	0.04189	0.240349001	0.30173
TSPY26P	0.166424644	0.419308	0.408878678	0.04189	0.020052648	0.92234
OLFML2B	0.48499652	0.435213	1.261660702	0.04194	-0.305178444	0.62389
SH3GL1P2	0.819660014	0.229514	1.307539421	0.04204	0.879968596	0.18932
ADAMTSL2	-0.229344459	0.588535	-0.894691435	0.04205	-0.784917884	0.07602
P13-128O4	-0.01660799	0.96879	0.784088344	0.04209	0.284846769	0.48274
WDR65	-1.220031441	0.050575	-1.630488486	0.04215	-0.173084816	0.77993
EFCC1	0.363662777	0.277742	0.652536712	0.04233	-0.269340191	0.43246
BMS1P10	-0.002489516	0.991509	-0.478833953	0.04248	0.155923908	0.50019
KBTBD8	0.203387027	0.45814	-0.559322345	0.04249	0.071085677	0.79891
P11-589M4	0.744252084	0.223696	1.184483373	0.04247	0.67950512	0.26404
SNX19	-0.03943131	0.779455	0.284368886	0.04248	0.113567867	0.4185
UNC13D	0.223195944	0.488288	0.649770621	0.04249	0.080538169	0.80203
SLC2A4RG	0.033462452	0.859456	-0.383901274	0.04265	0.002467795	0.98954
COMMD8	-0.167839506	0.305384	-0.330105158	0.0427	-0.268953541	0.09998
PALM	0.19922908	0.439326	0.52010994	0.04273	0.303682223	0.23809
TUBGCP4	-0.082492013	0.563926	-0.287893224	0.04274	0.317893766	0.02405
AJAP1	0.16184614	0.782257	1.084750186	0.04278	1.327692732	0.0138
EHHADH	-0.209181347	0.387136	-0.488341146	0.04279	0.04641877	0.84731
PDCD4	0.07760837	0.683888	-0.386147492	0.04283	0.043897054	0.81778
ADAP2	0.226363663	0.760177	1.443035528	0.04285	0.927528189	0.19457
ALKBH2	-0.242635843	0.141907	-0.329664548	0.04287	-0.415232233	0.01178
ZNF564	-0.324287782	0.06231	-0.351009929	0.04286	0.018020999	0.91701
ZNF175	-0.395873594	0.226205	-0.649029959	0.04299	-0.031416423	0.92233
SLC26A2	-0.148718823	0.55886	-0.515209985	0.04304	-0.538983655	0.03454
C9orf116	-0.245712331	0.249368	-0.428447897	0.04306	-0.285685942	0.17976
LMAN2	0.03818255	0.762741	-0.255746645	0.04308	-0.227343473	0.07256
SCNN1B	0.523844032	0.410143	1.27801479	0.04309	1.254749591	0.04737
TMUB1	0.171851251	0.333291	-0.361242783	0.04311	0.112365377	0.52623
DTWD2	0.004890134	0.975519	0.313786597	0.04313	0.174480558	0.26636
C10orf129	0.317052498	0.602469	1.136983153	0.04317	1.611049947	0.00353
PSTK	-0.21947155	0.294286	-0.416599302	0.04316	-0.513988802	0.01313
EHMT1	0.101938195	0.477216	0.288396988	0.0432	0.292526858	0.04069
ENOX2	0.066821951	0.760678	0.43419174	0.04328	0.161556447	0.45879
HTATIP2	-0.123925458	0.501336	-0.372298946	0.04328	-0.427504409	0.02092
7SK	-0.047376038	0.723961	-0.271079174	0.0433	-0.221713323	0.09838
LSM10	0.070251831	0.720189	-0.400596744	0.04337	0.302805159	0.11457
STMN3	0.403952706	0.124081	0.527624531	0.0434	-0.178092901	0.50136
RNF212	-0.104990015	0.742548	-0.650326187	0.04347	-0.463693593	0.15104
METTL21B	0.021945903	0.884452	-0.305283827	0.04356	0.222533307	0.13164
ASNSP1	0.524379307	0.192805	0.78470791	0.04361	0.259310232	0.52372
RARB	-0.195654705	0.20915	0.306906767	0.04362	-0.035881506	0.8156
C21orf91	0.020712775	0.911008	0.371793272	0.04389	-0.137434322	0.45835
PLCG1	-0.046543734	0.799835	0.368815846	0.04389	-0.009178123	0.9601
IFT27	-0.360918889	0.080098	-0.41178203	0.04392	-0.435134758	0.03433
EPCAM	-0.267604405	0.175102	-0.397259684	0.04409	-0.513543964	0.00927

MAL	-0.329346972	0.699577	1.630038093	0.04419	1.230276077	0.1313
MYO16	0.157200733	0.771678	1.057898989	0.04414	0.251053413	0.65347
MZT2B	0.262287014	0.296589	-0.508009993	0.04418	0.07697178	0.7595
PIK3CA	0.05067274	0.755546	0.326879451	0.04414	0.069649649	0.6688
RAMP2	0.274949729	0.327663	0.553418874	0.04407	0.121007275	0.66733
P11-407G23	0.37216894	0.127874	0.478505773	0.04409	0.431977326	0.07296
SEMA4F	-0.288022315	0.285676	-0.543156626	0.04415	-0.667617355	0.01427
STAC	-0.84730285	0.154182	-1.229436921	0.04402	-1.891747526	0.00303
TCERG1	-0.022071867	0.877799	0.28823711	0.04419	8.36E-05	0.99953
TTYH2	0.238358431	0.574124	0.847156026	0.04415	0.024893892	0.95343
YPEL4	0.090759381	0.725504	0.511117593	0.04417	0.338784067	0.18635
ZNF876P	-0.080486643	0.767558	0.523656547	0.04409	0.237375761	0.37134
TXLNG2P	0.742920203	0.437559	1.848257939	0.04427	0.825566325	0.37915
P11-889L3.	-0.011084926	0.957234	-0.418934008	0.04429	-0.314298954	0.13257
FRRS1L	0.345545625	0.272624	0.623692695	0.04434	0.529021783	0.09003
CFL1	0.129070436	0.140773	0.175994677	0.0444	-0.211069975	0.01611
TUBB	0.128151688	0.297512	0.247221234	0.04439	-0.067722813	0.58198
CCDC91	0.198375747	0.315862	0.393999474	0.04451	0.07695662	0.69676
WDFY3	0.122704811	0.348679	0.262574745	0.04451	0.364137874	0.00531
CELSR1	-0.214977167	0.31522	0.427456751	0.04455	0.609061218	0.00418
BRI3BP	-0.098980274	0.477949	-0.27994908	0.04461	0.180011789	0.19569
YOD1	0.094008915	0.366816	-0.209253324	0.04469	-0.030565514	0.76919
AMPD2	0.131270051	0.290629	-0.249781713	0.04476	0.042192065	0.73424
AGR3	-0.147702808	0.44673	-0.38934301	0.04486	0.06691811	0.72989
COG7	-0.054358777	0.613001	-0.215870414	0.04487	0.225751738	0.03399
TUBA1A	0.269794371	0.317081	0.540637036	0.0449	-0.408274441	0.13025
SDR16C5	0.098340624	0.867569	1.166049049	0.04492	-0.790992522	0.187
METTL4	-0.094530919	0.667704	-0.439241468	0.04495	-0.312126541	0.15532
FAM124A	0.792334994	0.056451	0.817293	0.04497	0.593652071	0.1493
PYGO2	-0.175465191	0.239518	-0.297534662	0.04501	-0.013863832	0.92533
SOS2	0.077131315	0.434521	0.196130656	0.04504	0.022382111	0.82023
P4-595K12	-1.096599183	0.2017	-1.780654939	0.04505	-0.116757208	0.88478
EIF4B	-0.022740876	0.840725	-0.226687894	0.04512	-0.183265493	0.10534
NPDC1	0.124161921	0.744961	-0.773690988	0.04517	-0.266215268	0.48744
NDUFB7	0.08298677	0.449079	-0.219737948	0.04521	-0.242177058	0.02833
UGT1A6	1.149297613	0.109773	1.393377539	0.04522	0.624859849	0.39836
SLC34A2	0.043908704	0.939741	1.128937458	0.04524	0.149123127	0.79548
TMEM173	0.581419618	0.060694	0.616586802	0.04523	-0.057543723	0.85386
ARL4C	-0.55078812	0.079489	0.617346804	0.04527	-0.515810104	0.09957
KCTD9	0.036755569	0.708273	0.194127283	0.04532	-0.003676126	0.9701
RIMKLB	0.269626455	0.234943	0.453042875	0.04534	-0.039135247	0.86335
CDH24	0.179650114	0.504752	0.533142081	0.04537	0.16981952	0.52755
PDZD8	0.224230811	0.268732	0.40522504	0.04539	0.322998431	0.11087
OSBPL8	-0.119083099	0.277252	0.218650834	0.04543	-0.077762541	0.47816
ZNF223	-0.323177	0.078578	-0.362726859	0.04549	-0.34222185	0.06188
MOGAT3	-0.474358908	0.306151	-0.929372029	0.04553	-0.94571174	0.04235
MTND4P20	0.142487443	0.648249	0.611485462	0.04553	0.800191686	0.00882

AP1M2	0.102577417	0.472119	-0.284864168	0.04556	-0.131488774	0.35647
SIDT2	0.40049219	0.165737	0.576637592	0.04555	0.89204848	0.00193
TDRD3	0.025599613	0.903168	0.414595359	0.0456	0.116133751	0.57813
ZNF280D	0.048865863	0.788271	0.356719749	0.0456	0.579089932	0.00114
PCCB	-0.102446592	0.528448	-0.324543068	0.04571	-0.25560208	0.11598
SULT1A1	-0.256012781	0.5262	-0.808911648	0.0457	-0.99079809	0.01451
TSPO	0.222353719	0.499605	-0.666391632	0.04572	-0.234154912	0.47975
MICU3	-0.064226342	0.792182	0.480534776	0.04575	-0.013223191	0.95683
POLR1A	0.172966737	0.201572	0.269100264	0.0458	0.498763798	0.00022
EXTL3	0.227810845	0.13688	0.305098214	0.04582	0.303130044	0.04759
MYOM2	0.080333914	0.892594	-1.215913535	0.04585	-0.215417835	0.72703
RPL3P2	0.025170337	0.884186	-0.34580327	0.04587	-0.289244849	0.09574
B3GAT3	0.195324559	0.151278	-0.273387845	0.04592	-0.08825746	0.51958
MN1	0.81828626	0.097351	0.984910107	0.04589	0.32928769	0.50491
PLIN4	0.152398835	0.572544	-0.549225778	0.04593	0.145353774	0.58872
P11-254B13	1.005991632	0.138754	1.298234533	0.04594	1.654069637	0.01007
RPF2	0.150588446	0.321398	0.302217143	0.0459	0.014595975	0.92348
ALOX5	0.568279141	0.245587	-0.98679058	0.04608	0.119361035	0.80782
GADD45A	0.288598998	0.343643	-0.615469616	0.04615	-0.410683318	0.18365
SLC25A39P	0.263192429	0.640093	1.042190598	0.04615	1.023942551	0.05224
TAF6	0.147342439	0.192579	0.222479751	0.04615	0.344805442	0.00217
ALDH1A3	0.137404472	0.816272	1.173736599	0.04619	-0.466637144	0.43108
PTPRN	0.098751421	0.88434	-1.38267195	0.04618	-1.08986194	0.13008
ERICH2	-0.051176083	0.926438	-1.219033117	0.04626	-0.287889633	0.60689
P11-288H12	0.350441774	0.138067	0.465985353	0.04628	0.433397931	0.06128
ADCYAP1	0.885253729	0.376246	1.878808064	0.0464	1.521608766	0.11617
ISL2	0.490860915	0.180224	0.719606804	0.04641	-0.469641368	0.21119
CTC-429P9.	-1.694745395	0.204077	-2.700693336	0.0465	-0.806888045	0.53938
EIF1B	-0.180993972	0.165446	-0.25786659	0.04649	-0.343512929	0.00852
EIF4G1	-0.065762912	0.616564	0.261279262	0.04647	0.048043717	0.71437
RPL32	0.098479801	0.385032	-0.225751884	0.04646	-0.136457335	0.2288
TSPAN8	-0.334599358	0.207657	-0.528625822	0.0465	-0.507040966	0.05622
IGSF9B	0.647950563	0.41328	1.466626915	0.04661	0.095671495	0.89842
JAG2	-0.476446364	0.160412	0.659330398	0.04659	0.433481815	0.19364
CHRNA7	0.481618078	0.402047	1.128423449	0.04665	0.063327806	0.91358
RPS7P11	0.160384446	0.680192	0.749579344	0.04666	-0.637160337	0.11517
GTF2A2	-0.078422378	0.481774	0.218992211	0.04669	-0.224498726	0.04424
PINLYP	-0.178032466	0.540642	-0.571651828	0.04671	-0.267029871	0.35127
RPL17P19	0.212748555	0.751984	1.249451137	0.04672	0.379029441	0.56548
EEF1A1P4	-0.122320523	0.52511	-0.381622799	0.04675	-0.010254607	0.9566
GTF2B	-0.009350489	0.922389	-0.190035163	0.04677	-0.346308029	0.00037
HNRNPA1P8	-0.478342471	0.452699	1.14237889	0.04676	-0.556637438	0.37989
SAMM50	-0.173285328	0.095956	-0.204722695	0.04679	-0.228046531	0.02813
C3orf83	-0.451787392	0.156451	-0.627840653	0.04682	0.286931301	0.33622
C6orf1	-0.274568578	0.332716	-0.561201821	0.04684	-0.587441319	0.03885
DIRAS2	-0.449044599	0.534406	1.283889273	0.04693	-1.083192047	0.15477
PITPNA	0.09589661	0.379784	0.215911005	0.04696	0.009802064	0.92839

PRELID1	0.071635467	0.692855	0.35958314	0.04698	-0.377650814	0.03762
CNTN6	0.798348471	0.333192	1.620315185	0.04701	-0.547504464	0.51728
DNAH10	0.297431737	0.490875	0.850259185	0.04701	0.391153606	0.36448
HP	0.462651247	0.297514	0.865286674	0.04701	0.361183103	0.40843
MDM4	0.058214584	0.762101	0.380520436	0.04704	0.462138981	0.01597
FGF10	0.240494738	0.741555	1.401836386	0.04712	-0.057878742	0.93727
PNPLA4	-0.065290646	0.689207	-0.320597007	0.04715	-0.119118001	0.46285
FABP6	-0.093083897	0.939075	-2.425638554	0.04717	-7.353178819	9.29E-08
MTTP	-0.340666426	0.53387	-1.086914385	0.04719	-1.661512885	0.00243
AAGAB	0.004517956	0.969023	0.229081047	0.04724	-0.025190798	0.82869
C1orf145	-0.003397763	0.992594	-0.728384633	0.04726	0.02311716	0.94899
HMGN4	-0.03796056	0.719477	-0.208261659	0.04735	-0.164133305	0.12024
LRP1B	-0.088411894	0.899652	1.380878627	0.04737	-0.384414399	0.58709
SLC27A5	-0.23067289	0.169084	-0.325863156	0.0474	-0.176994323	0.28053
DIS3L2	0.101439993	0.514012	0.305813008	0.04742	0.293053884	0.05738
TAF6L	-0.009997412	0.96108	-0.405792499	0.04743	0.038021241	0.85235
ATP11B	-0.082333373	0.401391	0.194122886	0.0475	0.063214961	0.51874
MECR	0.15291317	0.374709	-0.342336038	0.04754	-0.153468037	0.37575
APOBEC3F	-0.017487445	0.944742	0.489719763	0.04756	-0.073145896	0.76589
FAM122A	0.146065926	0.136791	-0.1950069	0.0476	0.10546355	0.28079
TARS2	-0.066833683	0.580485	-0.238211378	0.04772	0.026854789	0.82104
SSR4	0.12321643	0.355991	-0.264450713	0.04776	-0.054871973	0.68112
SKIV2L	0.084812551	0.480066	-0.237377102	0.04783	-0.063829128	0.59465
P3-449O17	-0.015587869	0.951115	0.482323456	0.04786	0.411245788	0.09358
IGSF9	-0.259817202	0.308857	-0.504880886	0.04788	0.545843451	0.03082
ANKRD6	-0.041837038	0.819517	0.355888901	0.04789	0.148465572	0.41517
WDR48	0.077304173	0.480818	0.215617935	0.04792	0.002074538	0.98493
GRM3	-0.530887511	0.233446	-0.892439719	0.04798	-0.250445688	0.5348
MITF	0.058756807	0.742989	0.349407162	0.04804	0.362023495	0.04061
HTN1	0.227558428	0.405178	0.533634637	0.0482	0.876327367	0.00114
NEGR1	0.560074729	0.067884	0.600523648	0.0483	0.596863173	0.05005
DDX23	-0.170759947	0.094003	-0.200476859	0.04859	-0.395336859	0.00011
DNAJB7	0.437272733	0.165795	0.604903144	0.04859	0.700304551	0.02286
KLF3P1	0.47092475	0.270146	0.798183159	0.04855	1.089761538	0.00655
MDP1	-0.620608325	0.174016	-0.899369181	0.04856	-0.351028949	0.43886
SLC23A2	0.236102481	0.094522	0.2755423	0.04858	0.235131621	0.09398
CWC22	-0.147194234	0.0762	-0.161596692	0.04863	-0.405684439	1.14E-06
CDR2L	-0.27860481	0.097053	-0.329025107	0.04868	0.032311345	0.84576
CHPF2	-0.113082916	0.313266	-0.22021246	0.04867	0.016195683	0.88472
ARMC5	0.261402838	0.072956	-0.290164426	0.04876	0.153972593	0.29084
S100A16	-0.147952787	0.446585	-0.382882014	0.04882	-0.608951725	0.00177
ADCK5	-0.323973784	0.107345	-0.391586362	0.04887	-0.194384418	0.32586
C5orf30	-0.203979345	0.159366	-0.284261503	0.04892	-0.176895578	0.2203
SH3BP4	-0.147258112	0.269046	-0.262365358	0.04892	-0.176433488	0.18508
GSTO1	-0.141808363	0.347608	-0.29668444	0.04897	-0.425378949	0.00491
LETMD1	-0.222819346	0.085946	-0.255424371	0.04899	0.135102909	0.29395
FAM163A	0.659783597	0.183597	0.945835301	0.04911	0.419248767	0.40151

KANSL1L	-0.016250507	0.882024	0.211070058	0.04911	0.209200429	0.05262
REEP5	0.021208076	0.83199	-0.19583244	0.04916	-0.112944413	0.2579
HEBP1	-0.370162563	0.10922	-0.452629368	0.0492	-0.052252579	0.81899
MAVS	-0.08764105	0.270562	-0.155881915	0.04922	0.228478356	0.00382
ATP1B1P1	-0.470853936	0.375958	0.87702679	0.04928	0.27705779	0.56017
DEFB1	-0.825845642	0.333321	1.592437603	0.04932	-1.114324164	0.1948
CHMP6	-0.089546713	0.573995	-0.315083909	0.04934	0.041468313	0.79441
HAUS1	0.060365778	0.720626	0.327481865	0.04936	-0.072858249	0.66576
KIFC3	-0.102951417	0.517301	-0.313855168	0.0494	0.250390177	0.11496
AZI2	-0.082470432	0.473913	0.224749589	0.04944	-0.070688571	0.5384
RMND1	-0.267215662	0.055131	-0.269652457	0.04946	-0.204266668	0.13862
ERICH1	-0.043587466	0.780428	0.303238442	0.04961	0.253955635	0.10114
ANXA1	0.097588212	0.737882	0.571843942	0.04974	-0.30625201	0.29375
GPR85	0.172667943	0.583807	0.595361728	0.04971	0.211121988	0.4957
PTPRK	0.168391521	0.061935	0.176052734	0.04972	0.157627277	0.08005
P11-84C13	-0.289011744	0.067071	-0.307552293	0.04976	-0.176756924	0.25971
ZNF587	0.013119673	0.902405	-0.209700207	0.04974	0.263773455	0.01337
MTX2	-0.028750461	0.799723	-0.221372983	0.04977	-0.158672229	0.16122
SLC25A30	0.054076842	0.818282	0.454229992	0.04979	0.187540618	0.42058
GOLGA6L9	0.775510381	0.513564	2.224870381	0.04981	0.796923916	0.49934
TALDO1	-0.078640961	0.539533	-0.250426558	0.04987	-0.101312406	0.42929
RBM18	-0.055700291	0.649376	0.237618911	0.04991	-0.144423489	0.23757
SERP1	-0.130674098	0.256826	-0.225761198	0.04992	-0.149752409	0.19356
CYP2J2	-0.301606757	0.289681	-0.559797913	0.04995	-0.240942656	0.39542
LRRC9	-0.268781779	0.692159	-1.370721087	0.04995	-0.043713033	0.94787
AC009961.2	2.359569443	0.103661	2.879331477	0.03779	2.49399692	0.08007
AC011242.5	-1.120240469	0.432553	-2.844168127	0.04889	0.530731431	0.64769
AC018804.6	0.851166187	0.563509	-3.495949038	0.045	-0.736628837	0.63916
AC068657.2	-0.551603555	0.719926	-3.686561318	0.03366	-0.384099568	0.79977
ACTG1P12	0.380904539	0.726583	1.917346444	0.04181	0.199050927	0.85572
AKAP4	-1.044170501	0.648702	4.303830009	0.03715	-1.771988944	0.43946
AP000350.1	-2.34707488	0.167498	-5.633916021	0.00289	-0.999250537	0.54038
AP000568.2	0.068123543	0.920442	-2.225154289	0.0121	-1.014935683	0.177
APOL4	-0.011901299	0.993921	3.055870522	0.02198	1.727678359	0.21697
ASCL1	1.345276338	0.417538	3.835273281	0.01169	1.886900498	0.23842
BATF	-0.886881497	0.650983	3.495648778	0.04024	1.546034237	0.3912
BCORP1	1.353510533	0.158263	1.900337988	0.03521	1.36463107	0.14597
BIN2	0.765411346	0.57534	2.40060378	0.03975	2.517984104	0.03005
BTK	1.070572851	0.580727	3.857255571	0.03347	0.233408681	0.90755
C10orf71	1.824727036	0.13882	2.653633147	0.02587	1.906666021	0.11876
C11orf42	-0.46648207	0.46228	-1.828478422	0.0141	-0.482514372	0.4405
C1orf173	1.334307229	0.309149	2.506094661	0.03875	1.087003236	0.39006
C1QTNF8	-1.620781165	0.247823	-3.344421901	0.01847	-1.727937201	0.21415
CABP7	1.629192918	0.26341	3.280720982	0.00964	2.476252509	0.06323
CACYBPP1	1.160642609	0.263777	1.94364666	0.04312	2.779842485	0.0029
CALCA	0.423252986	0.818771	3.865650648	0.01493	1.454224288	0.40925
CALML3	-0.55560748	0.802254	4.137985751	0.03821	-2.200780524	0.32476

CCDC177	1.479552081	0.222563	2.532056937	0.02818	1.17178725	0.33889
CCER2	-0.460103753	0.43228	-1.682075981	0.01338	-0.476437567	0.40616
CD86	1.966083371	0.277321	3.368085386	0.04549	0.898947252	0.63387
CDH5	-0.336326622	0.801832	3.275549429	0.00477	-0.714878535	0.59058
CLVS2	-0.124989296	0.922348	2.264915706	0.04878	1.34003931	0.25833
CRISP3	1.326204647	0.200142	1.916693748	0.04828	-0.723541155	0.5752
:TB-133G6.	0.730162582	0.563968	2.611064224	0.01682	1.931539425	0.08828
TD-2194F4	1.057520682	0.527363	3.61314611	0.01059	0.92159631	0.58177
TD-2535L24	0.336070421	0.897957	5.533744303	0.02261	4.058276957	0.09824
CYP4F12	-0.088667652	0.937838	2.144267058	0.02704	1.690465614	0.0907
DBH	-1.031200344	0.360042	2.062217631	0.04217	-1.598690315	0.17144
DLX6	1.018522037	0.583295	3.396079357	0.03755	3.820305035	0.01914
EPHA8	0.457475095	0.633019	-2.377302058	0.03524	-0.249089592	0.7998
FDPSP5	-1.901540384	0.076986	-3.181383898	0.01125	-0.166985089	0.84964
FDPSP8	-0.406881093	0.744857	-2.728509276	0.04852	1.108394858	0.28906
FKBP6	-0.382455114	0.661762	-4.082513966	0.0013	-0.415426825	0.63124
FNDC9	-1.12177644	0.336019	-2.535076413	0.03931	-1.051362019	0.33353
FPR2	1.769440924	0.240802	3.785969765	0.00668	2.96713114	0.03758
FTH1P10	-3.206441465	0.065525	-3.498642979	0.04449	0.479829032	0.74512
FTLP8	-1.350293555	0.358552	-3.093903634	0.03775	0.038098341	0.97549
GAPDHP65	-0.432425807	0.701264	-2.789048325	0.04311	-1.315125474	0.2901
GOLGA6A	0.690799871	0.58968	2.678248031	0.03442	0.107503569	0.93652
GPR88	2.763948138	0.118525	3.598067067	0.03313	2.209258202	0.22479
HADHAP1	0.616748331	0.54958	1.985457359	0.03318	0.771050296	0.4431
HBBP1	-1.474667137	0.162221	-3.105533994	0.01568	-2.37897976	0.04488
HBE1	2.717286172	0.231595	5.071890811	0.02083	1.880334849	0.4165
NRNPA1P4	2.772501586	0.094856	3.44114573	0.02962	3.335338197	0.03695
NRNPA1P4	-0.19242704	0.900155	2.756129302	0.02331	-0.313423231	0.83714
HNRNPCP6	0.934862881	0.322838	1.785884519	0.04155	2.016609729	0.02092
HSPA8P18	1.953713516	0.120846	2.467682861	0.03978	2.883413199	0.01514
HSPE1P8	2.375872224	0.088051	2.727407823	0.0415	1.870899354	0.19233
INHBB	-1.116608041	0.442897	2.48560594	0.04054	-0.534020243	0.69439
KCNA2	1.930991138	0.110557	2.826335486	0.0169	1.56663649	0.20956
KCNB2	2.712189003	0.082282	3.097672158	0.04171	3.007842716	0.04984
KL	0.404098661	0.662454	2.445245996	0.00224	-2.341413126	0.0669
KRT17P1	0.95809699	0.596145	3.12259789	0.04848	2.392799185	0.14829
KRT18P39	-0.732985056	0.354277	-1.749469923	0.04503	-1.861269422	0.04143
LMOD2	-0.35627063	0.769427	-3.350512557	0.02284	0.137045685	0.90661
LY6K	1.39028432	0.129048	2.156507173	0.01016	2.365714519	0.0047
MED28P3	-1.179977498	0.208606	-2.47684654	0.02533	-1.550165893	0.11004
MMP17	1.053241657	0.149602	1.651481697	0.02058	0.272455826	0.72503
MTND2P26	1.768542931	0.27364	2.876042021	0.04268	3.637238628	0.00797
MTND6P5	2.291772614	0.168789	3.779357753	0.01192	3.487117789	0.02252
MYCT1	-1.875003669	0.149324	2.86683284	0.00121	2.095796359	0.02226
NME8	-0.912785707	0.459272	-3.685537627	0.01211	-0.537811285	0.65187
NR0B1	1.752415899	0.291405	3.312824916	0.03571	-0.948454657	0.6059
NUDT19P4	-0.318363105	0.782909	-3.659357402	0.01029	-1.560071139	0.23498

NUS1P2	-0.153832034	0.865562	1.705669435	0.02681	-2.100634445	0.08608
OMP	-0.215518573	0.820501	-2.805755005	0.0263	0.391275353	0.65402
OR52N2	-2.126333409	0.143727	-3.139881557	0.03085	0.09336476	0.93733
P2RY4	-0.084127692	0.944872	-3.157095655	0.02972	0.00771071	0.99484
PAX3	2.19645891	0.168623	3.252535283	0.03104	1.999943485	0.19948
PAX4	1.031956177	0.393316	2.251682937	0.03929	1.549499097	0.17557
PCDH8	0.724737234	0.608684	3.079947128	0.01367	1.460976776	0.26907
PDCL3P3	-1.643985889	0.281713	3.031781289	0.00994	2.17298545	0.07358
PLCG2	-2.841911385	0.064728	-4.702306137	0.00404	-0.926211095	0.5009
PLK5	1.756391174	0.201265	2.630952459	0.03974	3.086085737	0.01422
PPP1R16B	-1.090922338	0.385341	2.206727863	0.02072	-0.80618243	0.49123
PTMAP4	0.76853797	0.388518	1.757864803	0.03584	-1.51111756	0.19025
RAD23BP1	-1.131207941	0.281447	-3.137435127	0.01376	0.117327025	0.89443
RAP1AP	1.868402404	0.165345	2.744318727	0.03079	1.114141676	0.43129
RNGTTP1	2.771955947	0.075202	2.985472553	0.0477	1.665170326	0.32517
RP1-154J13.	2.151768273	0.086692	2.741318673	0.02253	3.214833098	0.00683
RP11-10B2.	0.261404292	0.778935	-2.333454085	0.04944	-1.773145965	0.11214
P11-114H24	-0.533429992	0.472892	-2.560049614	0.00681	-1.1924968	0.13242
P11-129B9	1.576746044	0.072685	1.719111732	0.04361	1.274868203	0.15213
P11-168A11	-0.231991164	0.830879	-2.848298495	0.03767	0.012202627	0.99072
P11-214N16	0.423733742	0.711947	-3.766609859	0.01051	-1.515854865	0.25688
P11-344N10	0.379227858	0.774175	2.256211105	0.04046	2.469194776	0.02458
P11-371E8	-0.37520451	0.727251	-2.962982717	0.02931	0.520525549	0.59894
P11-446E9	2.768133223	0.111381	3.500182726	0.03509	3.963048397	0.01604
P11-466F5.	0.933085459	0.376883	2.343779477	0.01218	0.22095982	0.84532
P11-477G18	0.645142713	0.481317	-2.69699793	0.03705	0.923108215	0.29838
P11-504P24	-1.841541514	0.1076	-3.820773537	0.00461	-0.347494725	0.72154
P11-506B6	1.819655357	0.10503	2.141542025	0.04898	3.010946039	0.00453
P11-543P15	3.405835928	0.086056	5.250812208	0.00617	1.984691682	0.34529
P11-662B19	1.03612125	0.500105	2.802785343	0.02753	2.666974793	0.03927
P11-720N19	0.3669385	0.779794	2.283177114	0.03585	2.14242281	0.05218
P11-740N7	-1.015074996	0.273496	-2.352134195	0.03334	-0.337852734	0.68842
P11-86K22	0.766152958	0.563644	2.977830784	0.00951	1.294241593	0.30043
RP11-8L18.2	-0.132834712	0.893885	1.618947104	0.04896	1.901381801	0.01996
P11-90O23	0.34183476	0.816903	3.497478459	0.00205	3.036671471	0.00865
P11-95J11.	0.319579602	0.785527	1.968816094	0.0477	1.523807938	0.13877
RP3-406A7.3	-0.743715089	0.326668	-1.694795672	0.03891	-0.895099142	0.2382
P5-1120P11	0.336174059	0.847418	3.48757	0.02072	2.998570217	0.05387
RPL13AP25	-1.214823565	0.317929	-3.529500639	0.01662	-1.91788041	0.13019
RPL34P21	0.940423768	0.514055	2.935286408	0.01781	2.885794759	0.02069
RPL7P47	-0.026004162	0.974596	-2.150083559	0.03294	-0.644526678	0.44804
RRAD	1.625814203	0.085295	2.577599881	0.00513	0.377230832	0.7073
RSU1P2	1.747141626	0.1029	2.227597701	0.03308	1.199901361	0.27188
SERPINA12	0.338066451	0.736225	-2.839346526	0.03488	0.840869369	0.37941
SLAMF9	-1.393483079	0.280505	-4.530386603	0.00277	-4.374109346	0.00387
SLN	1.190539241	0.610791	5.551866782	0.00881	1.536139871	0.50831
SMTNL1	0.351305178	0.781252	2.412701527	0.02616	-0.756137712	0.5971

SNRPEP10	1.673445458	0.204297	3.52469168	0.00307	3.399384883	0.00457
STMN4	-2.546328762	0.111076	2.768168844	0.02761	0.088430767	0.94926
TMEM82	0.337309514	0.675299	-2.69507098	0.01636	-0.875973696	0.31764
TSPYL5	-1.785793129	0.190278	-3.413798426	0.02197	0.728134015	0.52133
TTBK1	-1.438832262	0.147421	-2.160724631	0.04167	-0.07698691	0.93009
UBE2V1P1	1.303506809	0.160499	1.74357332	0.04993	1.708673318	0.05704
VAX1	0.608410388	0.68817	3.259201326	0.02425	0.635421465	0.67504
ZMYND12	0.754052303	0.532282	-3.119091746	0.0399	-0.978920298	0.44098
LCN15	-1.41354401	0.092811	-1.516366448	0.07119	-4.580268665	7.84E-08
IDH2	-0.12101997	0.328874	-0.145404201	0.23937	-0.66679095	8.89E-08
SHFM1	-0.18700169	0.098622	0.002930201	0.97912	-0.6083464	8.91E-08
SOD1	-0.125175954	0.138771	-0.145392663	0.08364	-0.445956999	1.56E-07
POMP	-0.12918221	0.088888	-0.050104125	0.50257	-0.400756636	1.64E-07
SNX27	0.195653509	0.098263	-0.157783244	0.18201	0.6042248	2.73E-07
C12orf76	0.286855191	0.065887	0.191218288	0.21489	0.769354411	4.31E-07
MYL6	-0.127673224	0.148119	-0.077483037	0.37956	-0.442393869	5.54E-07
STOM	-0.01840512	0.921354	-0.18380243	0.32419	-0.931049106	6.54E-07
HECTD4	-0.068153339	0.650296	0.127769564	0.39393	0.725639547	1.20E-06
SHISA2	-0.445112504	0.096098	-0.241471187	0.36576	-1.29618954	1.40E-06
ASH1L	0.143160376	0.128132	-0.021497833	0.81908	0.450298076	1.58E-06
SEC16A	0.151414304	0.358663	0.228095998	0.16601	0.776518371	2.34E-06
CDC5L	-0.092770071	0.163748	-0.085809164	0.19458	-0.313912402	2.60E-06
NUGGC	0.424262283	0.125709	0.434002522	0.11011	1.232867705	2.73E-06
SRRM1	0.062758269	0.458886	0.065837618	0.43627	0.394736167	2.83E-06
PSMD7	-0.139625474	0.10641	-0.041395042	0.62957	-0.400395307	3.97E-06
TRIM72	0.185187599	0.340072	0.335744906	0.07808	0.867637483	4.01E-06
SFT2D2	0.10298366	0.397781	0.134463017	0.26881	0.559434213	4.08E-06
SERPINH1	-0.131277596	0.338656	-0.174242446	0.20402	-0.629980766	4.48E-06
TRMT112	-0.091085187	0.333236	-0.126493315	0.17651	-0.434022559	4.73E-06
SLC22A20	0.357685435	0.070082	-0.325069473	0.11	0.871368943	5.27E-06
TRIM56	0.170776114	0.130195	0.16311761	0.146	0.510463353	5.25E-06
ANKFY1	0.069513218	0.570278	0.232200791	0.05697	0.549734827	6.60E-06
HM13	-0.136973501	0.141112	-0.065368622	0.48133	-0.420331296	6.72E-06
SLX1B	-0.671348741	0.4943	-0.761424388	0.43631	-6.185064858	8.49E-06
CYP39A1	-0.414827102	0.153108	-0.411680379	0.15421	-1.395024531	8.76E-06
CAPN5	0.273036313	0.088227	0.22042421	0.16836	0.709011289	8.81E-06
NEDD8	-0.038705027	0.615151	-0.094152377	0.216	-0.343210577	8.86E-06
P11-1277A3	-0.06457503	0.753079	0.14532075	0.4604	0.837242216	1.05E-05
ZMAT2	-0.131828348	0.232172	-0.003779309	0.97244	-0.486089888	1.17E-05
PSMB1	-0.095851015	0.305294	-0.169561973	0.06835	-0.410526149	1.21E-05
SNRPD3	-0.150387738	0.171716	-0.021728684	0.84261	-0.481500638	1.23E-05
SLC9A1	0.330742662	0.224125	0.369792933	0.1735	1.175673299	1.26E-05
TMA7	-0.138662836	0.069095	8.86E-05	0.99906	-0.333792575	1.26E-05
IDH3B	-0.046437682	0.587354	-0.061455631	0.46976	-0.375919926	1.31E-05
PFDN5	-0.100194429	0.241926	-0.159266152	0.0621	-0.372425753	1.42E-05
SREBF2	0.219708752	0.074519	-0.097500114	0.42909	0.532752286	1.49E-05
SACS	-0.182195276	0.279153	-0.221590382	0.18762	-0.729985199	1.51E-05

PRKACA	0.244800466	0.224078	-0.010868648	0.95683	0.852625918	1.85E-05
ZCCHC14	0.128879956	0.354752	0.024986622	0.85709	0.59086589	1.85E-05
MYL12A	-0.137989027	0.102978	0.012717919	0.88015	-0.361275592	2.00E-05
WWP2	0.098793538	0.373309	0.205162674	0.06105	0.468951583	2.03E-05
TCEAL4	-0.261252086	0.092985	0.227277048	0.14084	-0.662495823	2.19E-05
MUC5B	0.936402939	0.104602	0.205447665	0.7218	2.441774942	2.28E-05
CA12	-0.360944682	0.532945	-0.92912947	0.10865	-2.471031862	2.30E-05
PLEKHH2	0.260411522	0.191689	0.348211838	0.07973	0.83779844	2.48E-05
RPF1	-0.078367346	0.368971	-0.164316563	0.05764	-0.369789915	2.58E-05
FAM103A2P	-0.216002257	0.098398	0.076611335	0.5452	-0.557146381	2.64E-05
SLC27A1	0.194584655	0.171421	-0.26565382	0.0616	0.585767656	2.71E-05
FAM175B	-0.135606463	0.141205	-0.122167075	0.17823	-0.387210272	3.03E-05
PHF23	-0.170759584	0.18805	-0.227296812	0.07883	-0.542085662	3.08E-05
TSPAN6	-0.118576013	0.21006	-0.005317758	0.95484	-0.395193622	3.13E-05
R3HCC1	-0.220052453	0.09498	0.108071351	0.406	-0.5516699	3.24E-05
POLR2F	-0.003717378	0.975295	-0.049286656	0.67567	-0.507597077	3.43E-05
RP11-313J2.	0.59566469	0.063307	-0.076872213	0.8113	1.31344968	3.66E-05
PSMB7	0.01301824	0.879271	-0.056090383	0.5103	-0.356636415	3.69E-05
SCAF4	0.086303664	0.450974	0.170688324	0.13254	0.466355517	3.82E-05
MEA1	-0.149503867	0.12156	-0.093566022	0.32664	-0.399536255	3.84E-05
MLXIP	0.028644635	0.858328	0.278817816	0.07935	0.644761633	4.40E-05
GLO1	-0.093521543	0.224633	-0.080861053	0.29187	-0.314730317	4.49E-05
CC2D1A	0.014403224	0.900946	0.140227046	0.22084	0.46594976	4.57E-05
FAM122C	0.46905825	0.094328	0.148648867	0.59305	1.110319475	4.82E-05
OVCA2	0.208634279	0.432669	-0.469573068	0.08068	-1.127910546	4.88E-05
SARNP	-0.161674698	0.084801	0.109548662	0.23503	-0.382159642	4.89E-05
FAM32A	-0.008899605	0.899912	0.031411991	0.65401	-0.288760782	5.01E-05
EP300	0.109638954	0.450054	0.076214124	0.59903	0.585824735	5.15E-05
CHERP	0.17999628	0.290258	0.019320618	0.90949	0.682003319	5.30E-05
USP36	0.340667866	0.050266	-0.058672205	0.73622	0.699097561	5.62E-05
ITPR3	0.122791837	0.458076	0.090337259	0.58496	0.665091752	5.73E-05
MYL12B	-0.167734562	0.076689	-0.055609324	0.55647	-0.381483555	5.72E-05
SMG6	0.259065273	0.152703	0.102908235	0.5681	0.724017002	5.83E-05
XRN2	0.002675243	0.968357	0.112440762	0.09278	-0.272029305	5.85E-05
ZMIZ1	0.318868061	0.150807	0.322977811	0.14388	0.886008211	5.95E-05
C16orf80	-0.215488545	0.173563	-0.243105344	0.12239	-0.63635026	6.08E-05
PAPSS1	-0.098442875	0.259397	-0.049323975	0.5703	-0.349685326	6.32E-05
SLC26A9	0.038422402	0.886698	-0.316234803	0.24095	1.076247891	6.43E-05
EIF4H	-0.063466609	0.431677	-0.077821221	0.33416	-0.3227276	6.53E-05
TRPM7	-0.03627066	0.651678	0.128078631	0.10795	0.317869534	6.76E-05
ANXA13	-0.053833206	0.925363	-1.029707802	0.07379	-2.305380445	7.19E-05
ATP11A	0.272908164	0.1609	0.031663324	0.8706	0.770934989	7.23E-05
CLIC6	0.378636085	0.274805	0.197941766	0.56795	1.367714567	7.66E-05
SPTLC1	-0.087204886	0.275694	-0.069564401	0.37828	-0.317070736	7.75E-05
P11-466P24	0.308243956	0.946012	3.19761001	0.48012	-18.26166354	8.27E-05
C16orf87	-0.100325233	0.494205	-0.001290968	0.9929	-0.576238246	8.32E-05
NPM1	-0.19564973	0.081696	-0.072845789	0.51671	-0.441873089	8.45E-05

CTSL	-0.241440343	0.218267	-0.186362814	0.33922	-0.776959979	8.55E-05
SPCS1	-0.134574456	0.091476	-0.08343323	0.28745	-0.313933798	8.63E-05
HIST1H1E	-0.281269716	0.086262	-0.22454615	0.17045	-0.6433748	8.83E-05
P11-169L17	0.108891579	0.776042	0.413909552	0.25909	1.379679536	9.14E-05
SEC14L6	0.446074443	0.098168	0.171547978	0.5241	1.001882486	9.31E-05
LRP1	-0.175688651	0.348808	0.145279224	0.43831	0.731808677	9.38E-05
PKIA	-0.339482724	0.234024	0.244817737	0.3848	-1.125143468	0.0001
ARL6IP4	-0.123496126	0.214574	-0.10651432	0.28147	-0.387828991	0.0001
NOP10	-0.079905843	0.356386	-0.102443639	0.23285	-0.335954923	0.00011
SCIMP	0.1502852	0.51395	0.017217264	0.93839	0.82593402	0.00011
P11-460N11	0.469647155	0.06918	0.043877469	0.86549	0.988690717	0.00012
CHMP2A	-0.020111837	0.800904	-0.096698992	0.221	-0.309422565	0.00012
BRPF3	0.286303662	0.139239	0.318672869	0.09971	0.738533482	0.00013
P11-598P20	-0.3745153	0.258582	-0.578593764	0.08055	-1.291560909	0.00013
HTT	0.113380616	0.48804	0.237092518	0.14578	0.622141607	0.00013
ZNF814	-0.025824446	0.822733	-0.029815314	0.79479	0.43395396	0.00014
CYP27A1	0.116645009	0.785441	-0.794190052	0.07152	-1.755273591	0.00014
FOXP4	0.155867659	0.40855	-0.125021051	0.50753	0.713465011	0.00014
YIPF4	-0.225834088	0.102679	-0.234536058	0.08867	-0.524920606	0.00014
NPIPB3	0.387521897	0.098408	0.192785415	0.41094	0.887353399	0.00014
TMEM230	-0.097543324	0.144406	-0.029076699	0.65913	-0.253998464	0.00015
ARID1B	0.090476646	0.434702	0.174811903	0.12836	0.434844649	0.00016
GIN1	-0.451977614	0.055873	-0.257848379	0.25544	-0.884338829	0.00016
HMGN1	-0.051845053	0.497666	-0.044295451	0.56106	-0.288413859	0.00016
HAX1	-0.212136103	0.082239	-0.166916149	0.1679	-0.460812633	0.00017
TBC1D10B	0.152632006	0.091449	-0.175548626	0.05211	0.334774304	0.00018
TMEM246	0.119201515	0.348827	0.15106737	0.23205	0.472030743	0.00018
SECISBP2L	0.118776733	0.431799	0.28653093	0.05755	0.562879703	0.00019
NDNF	-0.137745118	0.735633	-0.586616021	0.15433	-1.57842822	0.00019
AK3P5	0.678278037	0.350523	0.637995794	0.37129	2.473839007	0.0002
FRS3	-0.042184921	0.878323	0.254771069	0.33303	0.963831889	0.0002
DNAJA2	-0.085163603	0.316523	-0.060233746	0.47597	-0.31691617	0.0002
P11-10O17	0.131689611	0.435407	0.090120135	0.59048	0.616398301	0.0002
MRPL11	-0.144298657	0.250953	-0.201323425	0.10573	-0.471192795	0.0002
P11-288K12	0.245176172	0.44815	0.06628416	0.83635	1.134766785	0.00021
CBWD7	1.380841837	0.349978	1.772808636	0.22977	-6.617631395	0.00022
AL445665.1	0.632483222	0.077354	0.253746045	0.48042	1.287663971	0.00022
DDX50	-0.096699198	0.29465	0.141803309	0.11887	-0.341477673	0.00022
EDNRB	-0.437728165	0.264479	-0.039674163	0.9192	-1.458362316	0.00022
ANKRD1	-0.478505559	0.324876	0.251448026	0.59854	-1.877269508	0.00023
C14orf166	-0.136349658	0.096418	-0.069726595	0.39172	-0.302623722	0.00023
SCAMP4	0.203284931	0.15076	0.032831853	0.81615	0.515163663	0.00024
RP5-886K2.	0.194062015	0.530345	0.234855139	0.44013	1.089150579	0.00024
APOA2	-0.370827747	0.667691	-0.302659636	0.72591	-3.207428833	0.00024
KIAA1522	0.012471063	0.947526	-0.268768082	0.15606	0.692341697	0.00025
CREBBP	-0.11371569	0.563941	0.195015152	0.32104	0.718564782	0.00025
DOPEY2	-0.110576788	0.594046	0.345908306	0.09404	0.755058859	0.00025

RBM8A	-0.067804384	0.504867	0.05192239	0.60795	-0.371879089	0.00026
LRRC40	-0.141127861	0.080741	-0.027640223	0.72574	-0.29559959	0.00026
IGFBP3	-0.317472312	0.391293	-0.042532524	0.90854	-1.351780422	0.00026
SLC10A2	-0.215646347	0.829893	-1.081531273	0.28239	-3.819772327	0.00027
RBM17	-0.106823232	0.265277	0.083101457	0.38156	-0.348852858	0.00028
BEX2	-0.032948238	0.903586	0.075603751	0.77828	-1.047799303	0.00028
UCP2	0.30571271	0.289716	-0.527794478	0.06999	1.042521508	0.00028
LEPREL4	-0.116742598	0.494789	-0.293860198	0.08434	-0.620952833	0.00028
GDI2	-0.14818294	0.050888	-0.015526722	0.83755	-0.275259667	0.00029
PEX12	-0.346691472	0.103261	-0.180637586	0.38721	-0.787821787	0.00029
CTNND1	0.111216634	0.242163	0.045899367	0.62932	0.342888451	0.00031
TET3	0.264668527	0.089168	0.302680584	0.05141	0.559954793	0.00031
BDNF	0.156787515	0.617357	0.388356143	0.21322	-1.158646702	0.00031
PEBP1	-0.105527045	0.282021	-0.152294903	0.11923	-0.353666305	0.00031
DHX33	0.32446279	0.060574	0.28748596	0.09429	0.616307702	0.00032
SNX6	-0.090510263	0.18086	-0.087301161	0.19495	-0.242884574	0.00033
WIPF3	0.328923409	0.288797	0.202349521	0.50773	1.053572714	0.00033
RTFDC1	-0.149044881	0.169206	-0.134212529	0.21286	-0.390363195	0.00033
ABHD2	0.2170677	0.278123	0.163725878	0.41309	0.716747814	0.00034
C1orf186	0.034845616	0.89942	-0.033076764	0.90212	0.916559982	0.00034
SMARCA5	-0.075537473	0.431539	0.179801335	0.06044	-0.343685371	0.00034
TMEM63A	0.046770182	0.752178	-0.032483832	0.82641	0.527937643	0.00035
GSG1	0.063506064	0.789433	0.30470271	0.19314	0.83054061	0.00035
NBEAL2	-0.165597197	0.293697	-0.06764784	0.66766	0.562320728	0.00035
TRIML2	1.066470034	0.161154	1.153056984	0.12042	2.532183461	0.00036
NPIPB4	-0.031308725	0.902788	0.021224538	0.93392	0.904390021	0.00036
SMS	-0.091631009	0.406594	-0.120857316	0.27242	-0.393839717	0.00037
WDHD1	-0.274511231	0.081502	-0.205920128	0.18866	-0.561263787	0.00037
CRIPAK	-0.025038508	0.926439	-0.098859565	0.71168	0.9098819	0.00039
ANKRD52	0.153691408	0.448943	-0.035953561	0.85915	0.715074364	0.0004
RNPUL2-BS	0.148709351	0.389478	-0.007769318	0.96408	0.607475708	0.0004
CYB5B	-0.137255074	0.107782	0.090074834	0.2888	-0.302211499	0.0004
CDA	-0.509971475	0.363443	-0.393130338	0.48092	-2.073522603	0.00041
C8orf86	0.037426573	0.926463	0.294813466	0.4431	1.282368096	0.00041
VPS53	0.314338063	0.052504	0.208559309	0.19686	0.569547695	0.00042
HTATSF1	-0.115749092	0.196582	-0.010424398	0.9065	-0.316953222	0.00042
RRN3P3	0.131687973	0.464379	0.067810658	0.70213	0.611514457	0.00042
FAR2P4	0.06634625	0.892924	0.632033065	0.16766	1.565651064	0.00043
SYNE3	0.279027045	0.211709	-0.055148277	0.80474	0.767545315	0.00045
PCSK7	0.133893046	0.257709	0.153215017	0.19337	0.412036686	0.00046
IFIT2	1.745782848	0.093174	1.558963095	0.13352	-5.065893771	0.00046
KANSL3	0.206525287	0.070815	0.024612377	0.82916	0.399065646	0.00046
RECQL	-0.086032759	0.443859	-0.058762803	0.59896	-0.395815647	0.00047
PHYHIPL	-0.576845367	0.315344	-0.97455312	0.09098	-2.17545488	0.00047
P11-192H23	0.053405801	0.791895	0.293466983	0.1358	0.680949673	0.00048
PHACTR4	0.018075574	0.879069	0.21691695	0.06572	0.41045088	0.00049
IWS1	-0.115573319	0.160808	-0.136588086	0.09486	-0.287588128	0.0005

EDF1	0.009477046	0.914067	-0.065031964	0.4576	-0.306670345	0.0005
SLC26A8	0.290007884	0.343409	0.371171334	0.21152	1.005846954	0.0005
CEACAM19	0.301857652	0.130073	0.202467354	0.29955	0.667851464	0.00051
ENTPD1	0.487770669	0.087268	0.304162647	0.28534	0.987724975	0.00052
KLHDC4	0.12506285	0.464632	0.189016776	0.26463	0.582103664	0.00054
EMB	-0.314832339	0.243712	-0.165182249	0.53907	-0.944384761	0.00054
GATAD2B	0.084859401	0.665536	0.082341911	0.67328	0.671259933	0.00054
SRRM2	0.059262581	0.768183	-0.155637008	0.43885	0.694806043	0.00054
TMEM131	0.067849877	0.63758	0.053514613	0.70967	0.496879852	0.00054
GYS1	0.091988577	0.410267	0.113185778	0.31059	0.384629571	0.00055
ADH5	-0.133564401	0.241111	-0.144493146	0.20268	-0.393492462	0.00055
DPP4	-0.058670166	0.903947	-0.928347403	0.05632	-1.680874395	0.00056
ARHGAP22	0.316883919	0.266349	0.471755268	0.09343	0.964140783	0.00056
PPIL2	-0.066823325	0.610313	0.06220486	0.63339	0.447093952	0.00056
STARD8	0.313824038	0.190261	0.461824178	0.05093	0.817378047	0.00056
CETN2	-0.151194758	0.077934	0.071031324	0.39732	-0.295801164	0.00057
NUFIP2	-0.283385311	0.057226	0.071123867	0.63302	-0.513643342	0.00057
PLCE1	0.153123569	0.234571	-0.12294706	0.33992	0.442795064	0.00057
RP11-393I2.	0.087077171	0.873314	0.3552787	0.49708	1.702542974	0.00057
XRCC6	-0.061488642	0.456906	0.062178897	0.45071	-0.284943172	0.00057
C2orf50	0.262507502	0.542122	0.285013147	0.49521	1.355546673	0.00058
SLCO2A1	0.462366511	0.123756	0.23849954	0.42742	1.028439116	0.0006
FLT3LG	0.424958015	0.216272	-0.007297267	0.98302	1.114925705	0.00061
NAP1L1P1	-0.109909074	0.494561	0.264221159	0.08907	-0.564311991	0.0006
WDR26	-0.017746126	0.879503	0.218731933	0.06048	0.399621155	0.00061
CCNK	0.238901454	0.420843	0.070836075	0.81054	1.006931913	0.00061
LAMTOR2	-0.010537674	0.921672	-0.174273477	0.10027	-0.372774103	0.00061
CIRBP	-0.191428491	0.124038	0.028866912	0.81615	-0.426153943	0.00062
MOB3B	0.023126864	0.90059	0.120191954	0.51364	0.627095819	0.00062
NDUFS5	-0.007599434	0.930439	-0.040011402	0.64376	-0.299140391	0.00063
CYP2C9	0.076003553	0.757346	0.331912697	0.17429	0.830743773	0.00063
KLHL7	0.059604135	0.666161	-0.165904622	0.2265	-0.473557638	0.00063
WDR55	0.043859075	0.492348	0.010882653	0.86211	0.213557838	0.00064
ADAMTSL3	-0.034027942	0.86607	0.098200717	0.62593	0.686368056	0.00064
RFX7	-0.009764377	0.9581	0.025011155	0.89261	0.630384922	0.00064
ENY2	-0.047454578	0.68213	-0.011378605	0.92088	-0.396566661	0.00065
HNMT	-0.11275965	0.236973	-0.156918504	0.09623	-0.324383975	0.00066
WDR1	0.122918656	0.216568	0.050874066	0.60872	-0.338787729	0.00067
FBXL18	0.056285371	0.755	0.110482561	0.53199	0.591271289	0.00068
GUCA2A	-0.839332239	0.372242	-0.115038104	0.90103	-3.767214409	0.00068
FGF5	-0.081537173	0.744223	0.349967463	0.15193	0.823454159	0.00068
MRPS17P1	0.373595242	0.354661	0.51936277	0.18741	1.302088848	0.00069
CRTC1	0.219376021	0.269658	0.212760426	0.28285	0.664383813	0.0007
A4GNT	0.848832053	0.086972	-0.661315465	0.18751	1.674294144	0.0007
NCOA6	0.090782104	0.566367	0.066180034	0.6748	0.531660813	0.0007
TPST2	0.057869034	0.742488	-0.285753227	0.10474	-0.60324833	0.0007
ANKHD1	-0.044639283	0.750219	-0.17330277	0.21594	0.472594567	0.00071

?11-1072C1	-0.253369965	0.652606	-0.230689719	0.67625	-2.38690703	0.00071
MOCS2	-0.198497	0.071333	-0.200919168	0.06438	-0.3717548	0.00074
GAK	0.272045725	0.082722	0.038946248	0.8037	0.527664648	0.00075
APOM	0.012282884	0.97906	-0.805629479	0.08646	-1.598222131	0.00076
EEF1A1P25	0.641102593	0.16617	0.667581764	0.1387	1.45658287	0.00077
TMEM182	-0.042224211	0.833268	0.04314909	0.82918	0.670518505	0.00077
EHD2	0.361894451	0.168319	0.318004504	0.22542	0.877777819	0.00077
BMS1P5	-0.234114961	0.191961	0.213256771	0.22719	0.590202731	0.0008
GPANK1	-0.045501922	0.764823	-0.147205862	0.32685	-0.51571562	0.0008
EIF4E2	-0.108159759	0.27369	0.012951233	0.89528	-0.33175343	0.0008
TATDN3	-0.005727773	0.963729	0.166836354	0.17361	0.412962266	0.00083
ARHGAP1	0.141490321	0.157123	0.139040193	0.16208	0.331825522	0.00083
NPIPB5	-0.283970701	0.324212	-0.041365935	0.88546	0.956710255	0.00083
SFRP1	0.045141442	0.937766	-0.099566941	0.86326	-1.933868038	0.00084
?11-252A24	-0.10949481	0.648886	0.228530489	0.33135	0.774971002	0.00085
GNG4	-0.170753722	0.584071	0.382890235	0.21492	-1.051266166	0.00086
METAP2	0.031525724	0.67236	-0.007830531	0.916	-0.249176948	0.00086
WDR37	-0.125230656	0.324199	-0.162720513	0.19927	-0.425178813	0.00087
RBMX	0.032797124	0.673287	0.058858412	0.44827	-0.259195358	0.00087
DHX36	0.179955127	0.183422	-0.027353514	0.83902	0.448795365	0.00088
PSMB6	-0.153096022	0.095802	-0.140448116	0.12247	-0.3059988	0.00089
C4orf32	0.000314468	0.99871	0.353520233	0.06874	0.645104307	0.00089
NAALADL1	-0.511961991	0.431928	-1.13682981	0.09932	-2.945262429	0.0009
PIGZ	0.129850517	0.502535	-0.017178697	0.92889	0.632199424	0.0009
BCORL1	-0.23099153	0.484814	0.049465007	0.87922	1.063620085	0.00092
CHRM5	0.318124759	0.469103	0.349422301	0.4063	1.377453382	0.00092
GABARAP	-0.029920421	0.815156	-0.026974591	0.83264	-0.425492345	0.00092
SNCG	-0.068171688	0.835544	-0.172640734	0.59758	-1.123605879	0.00093
S100A13	-0.184337589	0.320839	-0.2522983	0.17196	-0.619250777	0.00093
ZNF208	0.569531635	0.089826	0.440629166	0.18364	1.07029704	0.00093
10-Sep	-0.184938148	0.079513	-0.042930354	0.68271	-0.349138347	0.00093
MROH1	0.257055858	0.117116	-0.048649385	0.76693	0.537373321	0.00093
REXO2	-0.090447743	0.46439	0.022641905	0.85289	-0.412462398	0.00094
CLIC5	-0.640365886	0.279181	-1.08471718	0.0668	-1.966563056	0.00095
LEAP2	0.382621836	0.186313	0.53349249	0.05534	0.916374685	0.00095
TYRP1	-0.739792425	0.190541	-0.437919576	0.41743	-1.90478959	0.00095
BRD4	0.277302828	0.111811	0.038061952	0.82682	0.571393363	0.00097
SLC2A3	-0.171393777	0.559439	0.081991503	0.7799	-0.970551632	0.00097
STEAP1	0.273091364	0.173195	-0.010226544	0.95931	0.655235478	0.00098
DNM2	0.044483461	0.755684	0.013009709	0.92747	0.470591694	0.00098
DTX2	0.296823888	0.092482	-0.080714965	0.64865	0.578420975	0.00099
ERI3	0.108271599	0.351004	-0.218854991	0.05959	-0.386978934	0.00099
OPN3	-0.116423127	0.415924	-0.139896927	0.32266	-0.476922314	0.001
FASN	0.08987933	0.735486	0.222017654	0.40386	0.874036359	0.00101
HAUS8	-0.289464047	0.128813	-0.130925432	0.48816	-0.628001452	0.00102
ACTR3	0.064984523	0.458566	-0.031141635	0.72201	-0.288026569	0.00103
BMS1	-0.062931558	0.457921	-0.108223021	0.19979	-0.278608923	0.00104

TMCO1	-0.100398355	0.206928	-0.038949565	0.6203	-0.261013106	0.00104
TTR	0.204393932	0.800313	-0.702280725	0.38513	-2.670119181	0.00104
VTI1B	-0.118393037	0.155005	-0.130893383	0.11213	-0.272241441	0.00104
SLC7A14	0.552330936	0.167772	-0.069647081	0.86092	1.278202002	0.00105
APOE	-0.113119658	0.634592	-0.16005691	0.5015	-0.78651906	0.00105
ZFHX3	-0.032467383	0.908171	0.001645426	0.99533	0.917259996	0.00105
7-Sep	-0.110991648	0.362513	-0.014230442	0.90689	-0.398722121	0.00107
GADL1	-0.101771611	0.699765	0.405010845	0.11111	0.823646373	0.00107
HOOK2	-0.222781121	0.084963	-0.071403472	0.57855	0.417861956	0.00108
SLC12A6	-0.035182501	0.824271	0.209013914	0.18468	0.514771261	0.00109
CYSLTR1	0.022670992	0.926774	0.267053509	0.27777	0.80219474	0.0011
KCNJ16	0.589465886	0.069713	0.469610356	0.14843	1.058523414	0.00111
CTCFL	0.155378323	0.601191	0.150445128	0.60455	0.905374665	0.00112
NANOG	-0.089972646	0.743466	0.14051138	0.60069	0.856608668	0.00112
COLEC11	-0.772511622	0.173185	-0.821289791	0.14715	-1.867395936	0.00112
HTR7	-0.678100418	0.382157	-0.944677684	0.22331	-2.75809303	0.00113
EP400	0.207668073	0.299415	0.116880477	0.55762	0.646622218	0.00113
ORC3	-0.135376925	0.115654	0.070771361	0.39809	-0.280650183	0.00113
ADAMTS20	-0.039089988	0.909002	0.184857118	0.5824	-1.172602067	0.00114
SIGLEC10	0.119625322	0.674027	0.10845945	0.6985	0.870253468	0.00115
NPC1L1	0.396058641	0.082955	-0.231934977	0.31175	0.739563052	0.00115
ST3GAL6	-0.649566273	0.053009	0.133195835	0.68741	-1.097941791	0.00117
NUP214	0.132456707	0.50246	0.260244293	0.18702	0.63949421	0.00117
PBOV1	0.494610048	0.218368	0.399465645	0.31394	1.242438106	0.00117
PSMD14	-0.110951255	0.329489	0.002492001	0.98241	-0.370058969	0.00117
SYT4	-0.537817475	0.503059	0.292101172	0.71231	-3.055355646	0.00118
BTBD2	0.167545032	0.153153	-0.222262335	0.05901	0.379267711	0.0012
RRP9	-0.049321323	0.732335	-0.264898399	0.06564	-0.472095999	0.0012
RLIMP1	0.895599104	0.110468	1.051131657	0.05379	1.719847875	0.00121
KRT23	0.394676888	0.55887	0.830021795	0.21767	-2.262672351	0.00122
MORC4	-0.114138264	0.568047	0.270828118	0.17514	0.645699948	0.00122
SZT2	-0.109530333	0.477837	0.165665744	0.28101	0.493971066	0.00127
P11-82O19	1.018057146	0.078755	0.819397252	0.15387	1.760344631	0.00127
INF2	0.126042267	0.392861	-0.186932108	0.20652	0.473562828	0.00128
PHYKPL	0.006706869	0.955982	-0.068935443	0.56871	0.387318152	0.00128
CEP112	0.110007852	0.456918	0.194394662	0.18074	0.47006082	0.00129
HNRNPF	-0.147643946	0.088357	-0.0985687	0.25417	-0.27856585	0.0013
FUT1	-0.183232886	0.562313	0.540660635	0.07464	0.972075517	0.00131
HS6ST1	0.0381321	0.763178	-0.124199202	0.32566	-0.409583488	0.00131
P11-56B16	0.713510615	0.245065	0.528694656	0.3843	1.813531754	0.00131
ERV3-1	0.062770289	0.596087	0.002410245	0.98363	0.375600883	0.00132
MATR3	-0.083866253	0.323195	0.067177381	0.42798	-0.272629957	0.00132
CASP14	0.687993308	0.226417	0.664699286	0.23004	1.731799314	0.00135
DSG4	-1.125125228	0.074744	-0.553002275	0.34495	-2.403186397	0.00136
FAM3C2	-0.324825341	0.39599	-0.293865066	0.44134	-1.237686354	0.00136
SMG1	-0.023410094	0.871358	0.122706196	0.39565	0.462063148	0.00137
PACS1	-0.046655731	0.878339	-0.00239432	0.99373	0.970396418	0.00139

TMEM63C	0.110130162	0.907734	-0.505414002	0.59417	-3.43434122	0.00139
P11-182I10	1.399532747	0.052308	1.232599057	0.08465	2.187725057	0.0014
POLR3GL	-0.201175497	0.139198	-0.200679265	0.13457	-0.437737882	0.0014
BAMBI	-0.210944244	0.423976	0.010237778	0.9689	-0.848341656	0.0014
PHF20	0.274559418	0.068751	-0.015881254	0.9159	0.480471648	0.00141
AGO3	0.224412304	0.102836	0.069556554	0.61225	0.437560431	0.00142
TNRC18	-0.070573976	0.635218	0.262170759	0.07733	0.472126595	0.00145
ACOXL	0.189284337	0.675377	0.06077629	0.8908	1.331597636	0.00146
KLRD1	-0.000684647	0.997684	0.416759618	0.07455	0.741896994	0.00146
SLC6A11	-0.151295939	0.802452	-1.11816435	0.07287	-2.09503153	0.00146
CXCL14	-1.00352575	0.193585	-0.985522949	0.20099	-2.488299671	0.00147
TBX3	-0.76042578	0.137036	0.318695709	0.53185	-1.633463165	0.00148
SERBP1	-0.165864737	0.140076	-0.021370247	0.84914	-0.3567445	0.00151
SF1	0.048194652	0.675537	0.107657519	0.34902	0.364180489	0.00152
DRAP1	-0.08922396	0.599054	-0.063760325	0.70585	-0.540737909	0.00153
PNRC2	0.110656729	0.109867	0.107647204	0.11874	-0.21974436	0.00153
FOLR1	-0.001969855	0.995325	-0.416206706	0.21798	-1.109692182	0.00153
CS	0.138227837	0.154089	0.071860062	0.45804	0.306970625	0.00154
B3GALT6	-0.061481334	0.589732	0.145881637	0.19143	-0.364228398	0.00154
AHSA2	-0.003394334	0.97536	0.153868904	0.15687	0.343575098	0.00155
EIF3CL	0.532140101	0.091713	-0.007183577	0.98185	0.996934697	0.00156
FAM35CP	-0.096104416	0.939055	1.661254936	0.10499	3.08563223	0.00156
PSMC1P1	0.0011644	0.99158	0.140803705	0.19837	-0.350915792	0.00156
GALNT6	0.491217998	0.112166	0.281520947	0.36207	0.976239183	0.00156
ROS1	0.989516027	0.060777	0.728485565	0.16149	1.620493658	0.00157
WDPCP	0.060529255	0.726461	0.190321617	0.26877	0.543058651	0.00158
RALB	-0.181338163	0.095356	-0.089227368	0.40851	-0.343985814	0.00158
LRCH3	0.025906539	0.843448	-0.011628229	0.92917	0.413095694	0.00159
ABLIM3	-0.11344981	0.826545	-0.293042591	0.56697	-1.711765931	0.0016
PPT1	-0.064488903	0.640871	-0.113748365	0.40984	-0.437636031	0.0016
VEPH1	-1.090790297	0.051364	0.16559988	0.73543	-1.915756843	0.00161
FKBP7	-0.041986592	0.824179	-0.141259159	0.452	-0.597866745	0.00162
AC002543.2	0.633733285	0.136265	0.200463765	0.64166	1.268235126	0.00163
GPD1	-0.075865014	0.908938	-0.960690832	0.14993	-2.155238792	0.00163
MKL2	-0.035089182	0.83587	0.132353078	0.43303	0.531055619	0.00163
RPL7P36	-0.054798732	0.934599	0.212027476	0.73683	1.818885405	0.00165
PRAP1	0.015839617	0.986782	-1.408305639	0.14199	-3.040785005	0.00167
AFAP1	0.252262924	0.09352	0.147057653	0.3269	0.471556847	0.00167
HIST1H1C	-0.317866507	0.0763	-0.317627592	0.07636	-0.563607369	0.00168
DIP2A	-0.054549582	0.636172	-0.061848739	0.59085	0.359284099	0.00169
HIST1H2AC	-0.11680988	0.418694	-0.256430718	0.07562	-0.454190837	0.00169
NXPH1	0.050889518	0.968922	1.801430698	0.12922	3.655418034	0.0017
C19orf69	0.119200054	0.894848	-1.747775989	0.07085	-4.124345931	0.0017
PPP1R35	-0.209063097	0.256357	-0.294037581	0.1066	-0.582489995	0.00171
MYRF	0.083622387	0.576621	-0.162469287	0.27848	0.467554882	0.00173
PTPN22	0.269403525	0.610645	-0.079670669	0.88192	1.612667071	0.00174
CHD1	-0.129787296	0.238054	-0.077699607	0.47958	-0.344383443	0.00177

P11-39K24.	1.250084799	0.1028	1.204433963	0.1107	2.269073985	0.00177
TIMM10B	-0.097401668	0.30924	-0.108523537	0.25449	-0.299713924	0.00177
NAV1	0.217307279	0.348359	0.184048625	0.42575	0.719177547	0.00179
ZNF529	0.064763156	0.572496	0.204597044	0.07089	0.354385918	0.00179
ERP29	-0.245658417	0.063112	-0.126360241	0.33821	-0.412871136	0.0018
SNW1	-0.185286464	0.073413	-0.109718677	0.28606	-0.323533394	0.0018
CLN6	0.264274186	0.050327	0.108990066	0.41762	0.419126623	0.0018
CREB3L1	0.480698102	0.325548	0.683531334	0.15367	1.470569413	0.00182
GID4	0.026502013	0.910972	-0.000264596	0.9991	0.729190014	0.00183
KIAA0195	0.210521095	0.212808	-0.078540753	0.64225	0.524738697	0.00184
NPFFR1	0.260947114	0.390066	0.450930775	0.12743	0.908447033	0.00186
SPG20	-0.289760422	0.069297	0.023051627	0.88468	-0.49608017	0.00187
ABCC3	-0.278758032	0.196728	0.213273573	0.32259	0.669798216	0.00188
ACSS1	0.158687195	0.454743	-0.053477249	0.801	0.655938711	0.00188
FNIP2	0.097240292	0.508121	-0.127455733	0.38556	0.455917948	0.00188
HAMP	0.152894634	0.647324	0.070132503	0.82982	0.980101071	0.00189
HSPG2	-0.214481342	0.46539	0.367918949	0.21039	0.912285316	0.00189
UBQLN2	0.186712178	0.091052	-0.123414572	0.26458	0.33938185	0.0019
MAGI1	-0.058132372	0.685003	-0.177408224	0.2137	0.441918157	0.00191
CCDC58	-0.08936852	0.528187	-0.198161277	0.15972	-0.441489	0.00195
MRPS9	-0.209385199	0.085145	-0.103945518	0.38775	-0.37765773	0.00195
PGBD1	-0.12055335	0.519442	-0.105775877	0.56678	-0.589487491	0.00198
IGF2BP2	0.072511272	0.475965	0.03451766	0.73365	0.31387309	0.00199
HAND2	-0.211334495	0.452002	0.160047585	0.56608	-0.876537835	0.00199
KCNE4	-0.080021443	0.71844	0.276152412	0.21005	0.680192041	0.002
SEC62	-0.011051208	0.920658	-0.12226713	0.27011	-0.342874227	0.002
MIPOL1	-0.058391266	0.768054	0.314288729	0.11199	0.610657842	0.00201
TRIM21	-0.139196163	0.47257	-0.332859246	0.0859	-0.60069617	0.00201
CNDP2	0.031984585	0.760417	0.177532271	0.08875	0.32214852	0.00202
LRRC19	0.119617487	0.803504	-0.915543707	0.05755	-1.496081663	0.00203
FAU	-0.012284771	0.903577	-0.046151727	0.64868	-0.313461102	0.00203
DLG3	-0.148948477	0.267176	-0.212237514	0.11337	-0.414041027	0.00204
PRICKLE3	0.409305929	0.063361	0.230771371	0.29257	0.667039926	0.00204
DMWD	0.22398058	0.111837	9.23E-06	0.99995	0.430789508	0.00204
SOX9	0.306376092	0.12494	0.012254826	0.95106	0.615030838	0.00205
ARMCX2	-0.083284251	0.623456	-0.262642426	0.12068	-0.52419736	0.00206
BCLAF1	-0.089239588	0.300951	0.050946286	0.5543	-0.265875771	0.00206
NAA60	0.145194111	0.294786	0.265052835	0.0545	0.423643819	0.00206
P11-308D16	0.223895983	0.238379	0.1800758	0.33691	0.570953834	0.00206
ZNF471	0.013596611	0.957352	0.08674717	0.73112	0.770756646	0.00205
VTI1A	0.166558868	0.246469	0.198039637	0.16515	0.439979133	0.0021
CDYL	-0.19164377	0.149471	-0.183870329	0.1648	-0.408357885	0.00212
QCJ-SCHIP	-0.193564673	0.546426	-0.541951031	0.09178	-1.005334112	0.00212
DAB2IP	0.197010611	0.146145	-0.060749002	0.65416	0.415364553	0.00214
ERCC5	-0.300961984	0.19404	-0.195872017	0.39374	-0.710746606	0.00216
HLA-L	0.195255757	0.285131	0.167130851	0.34867	0.539943101	0.00218
SFXN1	-0.209153293	0.065701	-0.212749568	0.06057	-0.348138151	0.00218

C15orf40	0.101427523	0.542233	0.20000458	0.22657	0.505286397	0.00219
VIMP	-0.117970223	0.379522	-0.20561204	0.12453	-0.41293265	0.00219
DPM1	-0.107992974	0.18983	-0.053264673	0.51028	-0.252299203	0.00222
ATP5F1	-0.075287714	0.427749	-0.006474696	0.94545	-0.290341241	0.00223
GUCY2C	0.004066296	0.996032	-1.417224313	0.08369	-2.518694593	0.00223
RPL17P17	-0.850393523	0.358578	1.023599331	0.16118	2.136161791	0.00224
UROD	-0.075043724	0.571994	-0.091547174	0.48804	-0.408179097	0.00224
TTC1	-0.02391944	0.841842	-0.163113015	0.17092	-0.367136652	0.00225
RFX3	0.437332248	0.228093	0.258794878	0.47765	1.0898234	0.00227
GABRA2	-0.217081402	0.545541	-0.5289093	0.1407	-1.105942079	0.00227
TOX3	0.148271394	0.454635	-0.195988828	0.32322	0.602655822	0.0023
EML5	-0.580188271	0.076208	-0.370389376	0.25123	-1.005612434	0.00233
MNT	0.09845398	0.504032	-0.121129217	0.40873	0.44040664	0.00233
NPM1P26	0.288590256	0.275411	0.441981963	0.08466	0.771885087	0.00234
OGT	0.138422336	0.126922	0.005223036	0.954	0.275094717	0.00237
HN1L	-0.022343671	0.852094	-0.060066651	0.61565	-0.364456201	0.00237
DERA	-0.16309277	0.195575	0.058731986	0.63536	-0.384220379	0.00239
P11-229P13	0.447509969	0.21952	0.4538322	0.20359	1.056026575	0.0024
FAM153B	-0.043303141	0.891414	0.265483391	0.37556	0.894719203	0.00242
COL6A6	-0.566253792	0.28156	0.434433615	0.40526	-1.607982717	0.00242
CALU	-0.09332305	0.385604	-0.040820447	0.70405	-0.326151786	0.00244
CCZ1	-0.13998103	0.113014	-0.059665457	0.49502	-0.267597298	0.00244
HIF3A	0.392512371	0.216956	-0.203863552	0.52207	0.959810199	0.00245
HNRNPA1	0.09297238	0.254612	0.004455429	0.95645	-0.247377118	0.00244
NAP1L1	-0.082773057	0.372965	0.056772107	0.54064	-0.281591482	0.00245
PRNP	0.051686314	0.674057	0.027255531	0.82395	-0.373952707	0.00245
RGS14	-0.288159786	0.251608	-0.438209624	0.0792	-0.765176901	0.00245
SAP18	0.028871163	0.731164	-0.157213515	0.0603	-0.255073916	0.00245
STX1B	0.380399412	0.445762	0.884193724	0.07313	1.491005157	0.00244
P11-809N15	0.497123686	0.277649	0.453596113	0.31255	1.304553043	0.00245
CLDND1	-0.023214231	0.79561	-0.098503073	0.266	-0.272370671	0.00246
DNM1P47	0.218527948	0.508595	0.360264013	0.27199	0.989338081	0.00246
BCAR3	0.272312794	0.200215	0.266357076	0.20815	0.641280361	0.00248
INPP1	0.082706075	0.67389	0.060950431	0.75588	-0.598378543	0.0025
NCOR1	-0.082051753	0.362075	0.049499424	0.58148	0.271427145	0.00251
AIG1	-0.056428629	0.802066	-0.389448645	0.08379	-0.684211781	0.00253
PRKCA	0.291676499	0.051588	-0.009462162	0.9496	0.451204883	0.00253
SEPN1	0.272283477	0.080788	-0.047782295	0.75937	0.46824019	0.00256
SLC22A3	-0.220598123	0.618718	-0.693634682	0.11899	-1.370586599	0.00258
ERVW-1	0.095119097	0.705073	-0.057162178	0.81896	0.745371578	0.00258
AC004980.7	0.30589711	0.199121	0.073191409	0.75804	0.702945325	0.00259
MAP4	0.18145552	0.262352	0.147961324	0.36048	0.487098777	0.00259
HBS1L	-0.102979472	0.244298	0.032412263	0.71188	-0.266511071	0.0026
P11-767L7.	0.10691799	0.818706	0.818526112	0.05387	1.252355866	0.00266
ZNF117	0.126748224	0.459401	0.025770238	0.87979	0.508374793	0.00268
SNRNP200	0.167118604	0.065027	-0.03833141	0.67185	0.271365717	0.0027
RNF213	-0.023223935	0.892412	0.166091212	0.33232	0.51346298	0.0027

ARL3	-0.166607101	0.140315	0.091976698	0.40142	-0.339697027	0.00271
SUSD1	0.20018479	0.075298	0.008473861	0.93987	0.336318995	0.00271
C6orf120	0.004047263	0.959215	-0.111170628	0.15719	-0.238493898	0.00272
MGAM	0.971860794	0.051586	0.198824521	0.69098	1.493706612	0.00273
ERP27	-0.380970346	0.28811	-0.347984061	0.33126	-1.088293821	0.00274
CAPN8	0.163857437	0.311524	0.283087428	0.08003	0.484244564	0.00274
RNASE1	0.365290829	0.275644	-0.075696613	0.82135	1.001405173	0.00276
ALDH1A2	-0.789492868	0.124412	0.037543235	0.94164	-1.539566156	0.00278
NGDN	-0.173789503	0.094184	-0.161044491	0.11709	-0.309611329	0.00278
AC004166.6	0.273532548	0.481977	0.389063626	0.30116	1.084771038	0.0028
GPR55	-0.645270121	0.532148	-0.267492556	0.79008	-4.192289106	0.00281
ALOX15B	0.029137872	0.949173	-0.282920668	0.53684	-1.451243615	0.00285
FREM1	-0.177832555	0.593263	0.11114169	0.73836	-0.994763768	0.00285
TEN1	-0.112749542	0.471131	-0.267224403	0.08447	-0.475493354	0.00285
HOXA13	-1.501137461	0.125662	-0.642211014	0.50864	-3.066290904	0.00287
EFNB2	-0.099722416	0.378676	0.040464548	0.71997	-0.337961645	0.00288
NDUFB10	-0.179273131	0.075006	-0.149074613	0.13451	-0.299999786	0.00289
AP000275.6	-1.068923411	0.106066	-0.117443151	0.85817	-1.984456821	0.00291
TRERF1	0.108964175	0.395549	0.104329489	0.41348	0.37803106	0.00294
PTPLA	-0.182556463	0.507876	-0.412708844	0.13132	-0.826224497	0.00295
HLA-DMB	-0.332632713	0.539641	-0.206439586	0.70038	-1.675900595	0.00295
ST6GAL1	-0.123814307	0.704591	-0.262621036	0.42094	-0.982630824	0.00295
ADAM28	0.241957426	0.232724	0.094088022	0.64254	0.601937235	0.00298
SP2	0.203146834	0.385278	-0.012508124	0.95722	0.676775609	0.00298
CADM4	-0.225419368	0.257475	-0.295023941	0.1369	-0.596185733	0.00302
POLR2B	-0.081183602	0.324736	0.098774884	0.22946	-0.244610941	0.00302
PMF1	-0.042649334	0.670935	-0.101136185	0.30985	-0.299099335	0.00303
VLDLR	0.035796345	0.892818	0.347443915	0.18861	0.782239905	0.00303
PDAP1	-0.04467859	0.706785	0.108564738	0.35771	-0.352530584	0.00304
SLC39A1	0.000904019	0.993398	-0.206655451	0.05838	-0.324458098	0.00305
MORF4L2	0.050550196	0.521998	-0.086564824	0.27256	-0.233869997	0.00307
MIR3916	0.003875041	0.966954	-0.065137379	0.48297	0.27269873	0.00308
ABCC10	0.141616825	0.425455	-0.001625919	0.99263	0.519465723	0.00309
SIK3	0.11427406	0.467412	-0.141743021	0.36624	0.462078265	0.0031
RP11-347C12	0.51257334	0.340097	0.566317655	0.28485	1.54758238	0.00312
HMGB1	-0.05686041	0.592972	0.194115113	0.06767	-0.314334058	0.00313
URAD	-0.216643394	0.838421	-1.888184949	0.08552	-3.686697791	0.00315
RP11-298I3.	-0.107486502	0.804367	0.006704068	0.98763	-1.303128676	0.00317
CYP4F11	0.888639981	0.069735	0.91220601	0.05819	1.399045927	0.00317
ISCU	-0.08301737	0.406008	-0.049463006	0.61564	-0.295836671	0.00323
PSMA4	-0.015222487	0.859749	0.154058962	0.07126	-0.25425162	0.00324
HIBADH	-0.112213933	0.368967	-0.071089727	0.56607	-0.369551021	0.00326
MYH7B	0.406502962	0.261958	0.643095425	0.07165	1.046193593	0.00328
ZFC3H1	-0.070497648	0.625663	0.254258359	0.07734	0.422725442	0.0033
ATP6V0D2	0.035928294	0.932372	0.383425874	0.34918	1.190511924	0.00333
GADD45GIP	0.059682665	0.673177	-0.225308785	0.11138	-0.419383251	0.00333
SSRP1	-0.094403509	0.321786	-0.053686358	0.57184	-0.279647711	0.00335

LRP5L	0.179363333	0.402182	0.13994475	0.50706	0.611631382	0.00338
PGF	-0.223364931	0.487582	-0.578809916	0.0714	-0.960092244	0.00339
TCF7L1	0.320914967	0.283323	0.447260465	0.13391	0.864196591	0.00341
TIGD1	-0.035207475	0.838212	0.142651918	0.40252	0.496559459	0.00343
FAM228B	-0.037477313	0.859471	0.316309265	0.1329	0.61491173	0.00345
PPP3CC	-0.374413528	0.062396	0.291761207	0.14149	-0.589700967	0.00346
ADAMDEC1	0.663539986	0.308701	-0.296314813	0.65115	1.892512195	0.00349
RNPEPL1	0.175298468	0.279052	-0.268239372	0.09783	0.469711896	0.00349
PSMC1P2	0.505037655	0.411974	1.001711733	0.08879	1.682581509	0.00353
ATXN7	0.06133432	0.768125	0.272848892	0.18861	0.60442112	0.00356
H3F3A	-0.177313414	0.0728	-0.037441451	0.70429	-0.287978194	0.00357
UXS1	-0.021878103	0.819808	0.001133585	0.99051	-0.281736831	0.00357
TTLL3	0.247472798	0.165355	0.09006944	0.61322	0.515716365	0.00358
MRPL32	-0.049396125	0.682613	-0.105106661	0.38221	-0.352801892	0.00361
PLEKHM3	0.022480416	0.874644	-0.120668172	0.38902	0.398471836	0.0036
PTGES3L	0.259361591	0.283189	0.191529936	0.42202	0.684065734	0.00361
TPPP	-0.116425504	0.592006	-0.306240613	0.15493	0.594453275	0.00362
CTNNAL1	-0.180087178	0.082198	0.055554972	0.58725	-0.301786351	0.00362
KIAA1244	-0.087059333	0.638631	-0.331598105	0.07363	0.537574548	0.00364
SNRPD1	-0.163598984	0.250731	0.169743099	0.23129	-0.414114646	0.00365
SDHB	-0.041374201	0.725344	-0.224046954	0.05658	-0.343515438	0.00366
LGALS4	-0.298391949	0.594468	-1.011850057	0.07107	-1.629611236	0.00367
LINGO1	0.38508406	0.133144	0.112092236	0.66159	0.734903189	0.00367
IL12RB1	0.493701461	0.200098	0.41536953	0.27551	1.0801513	0.00367
RGS10	-0.004342382	0.981883	-0.011455064	0.95142	-0.56768356	0.00368
C12orf55	-0.145008256	0.548159	0.332725901	0.16689	0.697953669	0.00371
LPAL2	0.68983472	0.148151	0.499747671	0.29205	1.350020535	0.00372
PARP15	0.027190421	0.916223	0.464400323	0.06989	0.742236489	0.00372
CCDC41	0.080614945	0.631554	0.194574269	0.23934	0.481448943	0.00377
MRPS16	-0.05171311	0.678177	-0.172507768	0.16465	-0.360975935	0.00377
MYLK3	-0.135274331	0.598037	0.251735023	0.31858	0.726835602	0.00377
C12orf50	0.057548019	0.854028	0.436569482	0.15896	0.895538813	0.00378
EP400NL	-0.29208703	0.172309	-0.028237729	0.89387	0.606353861	0.00381
ZNF222	-0.381028163	0.064748	-0.219075476	0.26862	-0.601357054	0.00384
CLNS1A	-0.136887891	0.149728	-0.163825968	0.08317	-0.274894685	0.00386
IQCF3	0.458296125	0.55843	0.360576497	0.63893	2.017394573	0.00386
TMEM44	0.174231618	0.261841	-0.033331469	0.82906	0.441925986	0.00385
PRCP	-0.142430417	0.090675	-0.063850978	0.44312	-0.243086036	0.00388
FAM193A	0.24691579	0.064539	0.146006244	0.27183	0.383216429	0.0039
HMGB1P14	0.365262968	0.324747	0.507571768	0.15362	0.999507868	0.00392
ASGR1	-0.213405562	0.571104	-0.6796822	0.07212	-1.107330442	0.00394
ZNF546	0.066785413	0.78258	0.163306175	0.47778	0.665244044	0.00395
TMED1	-0.129306926	0.328948	-0.020545665	0.87515	-0.3842073	0.00398
P11-201O14	0.53008334	0.286518	0.825941907	0.08477	1.354691042	0.00399
IAH1	-0.136021725	0.280044	-0.164978189	0.17623	-0.356948496	0.004
NAT8	-0.497664277	0.619473	-0.823512202	0.41183	-3.521441366	0.00401
NFATC2IP	-0.045060374	0.726427	0.216270233	0.08896	0.365036851	0.00403

RPL11	-0.143433769	0.260511	-0.160947385	0.20663	-0.366610661	0.00403
\16c-431H6	0.027743191	0.88804	0.012549517	0.94843	0.547768764	0.00405
NOL9	0.036402109	0.803625	0.284244767	0.0511	0.418718505	0.00406
MALL	-0.510814723	0.238262	-0.085375699	0.84357	-1.247337009	0.00407
ITK	0.120302516	0.68524	0.386564439	0.18357	0.831422189	0.00408
MALRD1	-0.499322941	0.253435	-0.630480637	0.15283	-1.304070278	0.00411
FSTL5	0.163313935	0.636544	0.160842247	0.64131	-1.002428503	0.00414
BCCIP	-0.055382287	0.617201	0.04994491	0.64994	-0.318411711	0.00416
CBFA2T2	0.249176862	0.176062	0.20268909	0.26905	0.523994818	0.00415
TFB2M	-0.262483549	0.051784	-0.183537626	0.16712	-0.386721798	0.00415
\C006042.8	-0.000873445	0.99874	0.318643623	0.53976	1.397168279	0.00419
RP1-95L4.4	-0.491339914	0.272525	-0.733576663	0.09984	-1.396947029	0.00422
SLX4IP	-0.250077802	0.050182	-0.180040617	0.13869	-0.354191365	0.00421
NAP1L6	-0.203515909	0.667006	0.064740009	0.88324	1.162032169	0.00424
ASNSD1	-0.228010208	0.153145	0.082287323	0.6052	-0.45627364	0.00427
GNAI1	-0.217762555	0.104052	-0.25965711	0.05153	-0.383004783	0.00428
RRN3P1	0.078934856	0.645608	0.143675717	0.39649	0.480196425	0.00429
TRAM1	-0.173156605	0.190518	-0.10313982	0.43524	-0.377780393	0.00429
FAM149A	0.042001099	0.82448	-0.115550493	0.54035	0.532812683	0.0043
CALM3	-0.006462355	0.948849	-0.117600844	0.24296	-0.287810329	0.00431
PRDM16	-0.05953257	0.777071	0.236458528	0.2596	0.59626194	0.00432
TATDN2P2	0.050624584	0.84069	-0.014203709	0.95463	0.699943127	0.00431
ATG10	0.224491657	0.146462	0.028387609	0.85232	0.434403266	0.00436
COPS4	-0.185201359	0.140865	0.150418313	0.22717	-0.358687677	0.00436
UPRT	-0.181164435	0.27299	-0.273186907	0.09513	-0.471677298	0.00435
DCP2	-0.210132157	0.089227	-0.047838662	0.69814	-0.352239272	0.00438
PPTC7	0.203696747	0.157942	-0.084981262	0.55591	0.409714105	0.00439
PDE4C	0.162235376	0.42492	0.186494575	0.35927	0.573899255	0.00439
CSF1R	0.000954307	0.998273	0.552751772	0.1946	1.214263074	0.00443
ARMCX3	-0.114901496	0.327379	-0.228998182	0.05047	-0.333940111	0.00444
HBD	0.093642922	0.883736	-1.206295302	0.06827	-2.172517243	0.00445
ABCB5	0.311488974	0.295988	0.339308662	0.2354	0.801523824	0.00446
PRPF40A	-0.054207818	0.549857	0.088538848	0.32724	-0.257804021	0.00447
GAB3	1.081155283	0.076986	0.347259965	0.56909	1.709409257	0.00449
KCND2	-0.049242419	0.91871	0.384817777	0.41457	-1.496377601	0.0045
UBE2D1	-0.163734621	0.380325	-0.213612824	0.2537	-0.533603352	0.00452
GGT1	-0.065360989	0.884001	-0.521271277	0.24742	-1.307095349	0.00454
MFAP1	-0.044871662	0.668897	-0.203992592	0.05111	-0.299378747	0.00454
TMSB10P1	-0.730424244	0.231278	-0.112259022	0.8431	-2.012335016	0.00455
ZNF219	-0.261600956	0.2536	-0.012839505	0.95483	-0.657039476	0.00455
PDCD10	-0.122138186	0.240306	-0.090869588	0.37798	-0.295106717	0.00457
TBX6	0.137324821	0.622208	0.066708679	0.80649	0.74101787	0.00459
ATP6V0A1	0.08432081	0.532203	0.068175511	0.61345	0.380635432	0.00459
TRIM46	0.583922401	0.110664	0.414703164	0.25733	1.018430408	0.00461
HID1	0.139156435	0.506662	0.213687854	0.30686	0.591291084	0.00465
\C063976.7	0.273263001	0.517201	0.379907701	0.35245	1.115426586	0.00466
F7	0.336715954	0.447315	0.21114016	0.63171	-1.367727326	0.00469

THSD7B	0.285068822	0.66088	0.097986425	0.88025	-1.894145396	0.00474
PCNA	-0.239358126	0.146632	-0.222011485	0.17751	-0.465600703	0.00478
NAT2	-0.314707086	0.638442	-1.167622012	0.08746	-2.044579567	0.00482
CICP27	-0.022721105	0.908672	0.027908165	0.88619	0.541623421	0.00486
P11-193H22	-0.105957999	0.659699	0.302653614	0.20326	0.667798199	0.00488
ZFR	-0.242829378	0.062172	0.250115242	0.05429	-0.36652451	0.00489
RRAGD	0.267264465	0.538163	-0.199614624	0.64191	-1.261768382	0.00489
TD-2547E1(	0.061484	0.721184	0.078938581	0.64591	0.48280559	0.0049
NUP133	-0.146892812	0.111517	0.037088825	0.68612	-0.259906026	0.0049
EXD3	0.155731416	0.347417	0.026596481	0.87097	0.452030365	0.00491
CDKAL1	-0.046953019	0.596118	-0.037963707	0.66298	-0.249643387	0.00492
RALGAPA2	0.010941346	0.955458	-0.269534491	0.169	0.550251779	0.00493
CTNNBL1	-0.004349528	0.972494	0.069681889	0.57489	-0.355652896	0.00494
UTP14A	-0.067195193	0.545729	-0.090794262	0.41039	-0.313641749	0.00496
ADH1B	1.581385769	0.148379	0.65409792	0.55107	2.888849935	0.00499
S1PR1	0.085502026	0.82251	0.407008744	0.28305	-1.085286056	0.00502
ALDOB	-0.121923463	0.892075	-1.635926174	0.069	-2.531925952	0.00503
TCF23	0.483659654	0.085205	0.362848441	0.19159	0.767307755	0.00507
HIST1H2BC	-0.265736956	0.107953	0.174712746	0.28979	-0.463289796	0.00508
AC016712.1	0.054740666	0.903516	0.142136714	0.74486	1.15876022	0.00509
POLR2A	-0.063343215	0.823184	0.229496041	0.41416	0.781951426	0.00509
KMT2C	0.034715316	0.839234	-0.006331113	0.97047	0.47853419	0.0051
DNAH10OS	0.195139761	0.454781	0.461967827	0.06575	0.699113114	0.00514
HIST1H2BD	-0.237667582	0.163324	0.086527863	0.61114	-0.477264404	0.00514
EPN1	0.229051529	0.15605	-0.227173872	0.1596	0.449876576	0.00518
SF3B1	0.033990835	0.650982	0.040001646	0.59401	-0.210027571	0.00519
CNR2	-0.274545417	0.401616	0.240136266	0.4274	0.820135822	0.00523
SEC61B	-0.057192862	0.591109	-0.061805036	0.55881	-0.298178296	0.00524
TERF1	-0.090817106	0.475261	-0.003501659	0.97791	-0.355229449	0.00526
SCN3A	-0.452659238	0.365509	-0.02695425	0.9567	-1.436953446	0.00529
TBPL1	-0.257642378	0.145409	-0.063487539	0.71521	-0.495717479	0.00531
PROS1	-0.036353757	0.906139	-0.389778077	0.20624	-0.861021254	0.00533
GID8	-0.125542765	0.059056	0.102947894	0.11293	-0.183962741	0.00534
PGD	-0.162835899	0.062249	0.054605254	0.52942	-0.243280252	0.00534
P11-742N3	-0.059760787	0.862529	0.132483318	0.69913	-0.978360224	0.00535
IRF2	0.039444119	0.850473	0.148982598	0.47055	0.578874445	0.00536
ATP2A2	0.044863397	0.699281	-0.106976944	0.35672	0.322552088	0.0054
YIF1B	-0.034852443	0.81122	-0.175338873	0.22709	-0.408362896	0.0054
NFASC	0.493371549	0.334842	0.664573098	0.18841	1.392871309	0.00542
CALCR	-0.501215324	0.473429	-1.212131679	0.0851	-2.031089238	0.00549
CHIC1	-0.115438752	0.462099	-0.247753117	0.11302	-0.437770051	0.00548
DDX50P1	0.443764106	0.194666	0.517410186	0.12149	0.91518413	0.0055
MYL6B	-0.055553048	0.740697	-0.166312854	0.3206	-0.468038697	0.0055
PPP1R26P1	1.407606815	0.089551	1.381004245	0.09151	2.215139367	0.00548
SERPINE2	-0.104391739	0.748073	0.149619717	0.645	-0.904683181	0.00549
TRRAP	-0.060941926	0.734789	0.080002483	0.65582	0.497700958	0.0055
TRUB2	-0.011102022	0.930648	-0.20992911	0.09728	-0.353821268	0.00548

ATN1	0.296961116	0.268428	-0.250621486	0.35214	0.740078596	0.00551
PTPRN2	0.314490921	0.233767	0.437234992	0.09705	0.730813421	0.00551
AGRN	0.146365499	0.360153	0.099228196	0.53465	0.443014688	0.00553
C4orf36	-0.003736905	0.983809	0.207595031	0.25328	0.502899127	0.00553
P11-386I23	0.122955976	0.74563	0.116240312	0.75366	0.983208189	0.00554
FUT8	0.239039222	0.209312	0.096888926	0.61066	0.527043348	0.00557
C2orf49	-0.173810207	0.211507	-0.140980831	0.30849	-0.384426127	0.00559
MTHFR	0.173800227	0.1736	0.229164278	0.07105	0.351596254	0.00559
FARP1	0.130418303	0.24865	-0.000835646	0.99409	0.312069743	0.00563
RPL5	-0.138352288	0.298725	-0.090890819	0.49472	-0.368571683	0.00564
GPR75-ASB	-0.051714961	0.708552	0.160695694	0.24246	0.379917121	0.00569
MYO1F	-0.148123333	0.641386	0.230979868	0.44322	0.80984024	0.00568
OLA1P3	-0.00314911	0.991618	0.417510752	0.15464	0.806721531	0.00569
SIGLEC15	-0.120951969	0.720587	0.282508537	0.37703	0.868203846	0.00568
SLC17A4	-0.412724912	0.567023	-1.36631722	0.05854	-2.004898984	0.00568
ATP9B	0.178286006	0.324436	0.309985986	0.08591	0.498632354	0.00572
IDO2	0.363499162	0.311066	0.138602281	0.69741	0.936136803	0.00574
LUZP2	-0.699213732	0.135182	-0.233370583	0.60654	-1.300104043	0.00578
MED12	-0.088465021	0.565852	0.191464994	0.21219	0.422526025	0.00583
TXN	-0.216647054	0.086064	-0.047268182	0.70722	-0.348100078	0.00583
ZNF785	-0.198912878	0.097627	0.092038469	0.42428	0.316784966	0.00584
ELOVL5	0.254395047	0.104172	0.236144738	0.1309	-0.433051983	0.00585
VTA1	-0.098078029	0.334144	-0.101508169	0.31352	-0.28032658	0.00586
PKD1L1	0.27919103	0.541822	-0.299861279	0.51521	1.255264178	0.00588
MAGEA10	0.575898658	0.227964	0.554850819	0.24063	1.241192494	0.00589
LRRCC1	-0.340813571	0.065643	0.17982351	0.32105	-0.509006463	0.00592
KLHL6	-0.105440613	0.678398	0.277669228	0.25763	0.665819677	0.00593
TRIM15	0.856811568	0.284603	-0.219878647	0.78512	-2.303345752	0.00595
MKL1	0.254495847	0.297775	-0.041560298	0.86497	0.668747542	0.00596
SPEN	-0.163239026	0.422177	0.105951749	0.6017	0.5573015	0.00598
AC093642.5	0.087268262	0.804556	0.264284013	0.44717	0.934297331	0.006
SMC3	-0.192456428	0.069363	0.125413769	0.23382	-0.291127651	0.00599
KAT2B	0.107601732	0.623835	0.216900167	0.32126	0.600375067	0.006
SGK3	0.11165929	0.5497	-0.038075352	0.83685	0.505618346	0.00601
FAM195B	0.013519975	0.938919	-0.295759447	0.09389	-0.487655419	0.00602
RPS20	-0.080254052	0.426379	-0.102017417	0.3117	-0.277225553	0.00602
SLA2	-0.366394491	0.35405	0.297617996	0.41276	0.965376342	0.00602
P11-196I18	-1.022880791	0.126862	-0.938305026	0.14892	-2.025372767	0.00603
KLK10	0.141039599	0.710729	0.55495954	0.12638	0.981666285	0.00603
ZZEF1	0.021858256	0.89426	0.047381111	0.77256	0.449312125	0.00605
GYPC	0.007358171	0.967768	0.286332486	0.11172	-0.505438416	0.00606
P11-124D2	0.178600519	0.629882	0.276421234	0.438	0.934008027	0.00606
RGS17P1	0.33437182	0.364024	0.057757673	0.87513	0.944515367	0.00607
MRPS15	-0.194029143	0.140588	-0.09819148	0.45358	-0.361189953	0.00608
AC012360.2	-0.031948434	0.767805	-0.068300437	0.52399	0.291951917	0.00609
CADPS2	0.238145223	0.290757	0.3331848	0.13761	0.614965477	0.00611
ZDHHC15	-0.146534949	0.575867	0.260563824	0.31055	0.705644734	0.00611

TINAG	-0.305905323	0.549887	-0.562397952	0.2762	-1.500481869	0.00616
ARPC3	-0.138974488	0.112255	-0.163383811	0.06116	-0.239680287	0.00616
FRMPD2	0.507584921	0.550128	-0.952973635	0.27878	-2.59692775	0.00622
PPP1R13B	-0.10388059	0.52053	0.192166483	0.22986	0.438236912	0.00621
CCT2	-0.052116279	0.59911	0.045199992	0.64768	-0.27153559	0.00623
NGRN	0.093740817	0.270908	-0.0129912	0.87772	-0.233579781	0.00622
PPP1R14B	-0.087896734	0.464295	-0.113768456	0.34049	-0.32914339	0.00623
POLR3D	0.044907644	0.718655	-0.194542063	0.11896	-0.342357968	0.00626
ABCA5	0.230135482	0.221854	0.104393154	0.57777	0.511309754	0.00628
RPAP2	-0.045045207	0.804972	0.272671451	0.13444	0.497650555	0.00629
RSPH9	0.263040987	0.250805	0.410410986	0.06352	0.602113154	0.00629
PNPLA5	0.582175565	0.52501	1.557218991	0.05686	2.178051831	0.00629
MESDC2	-0.081736393	0.344623	0.003038222	0.97172	-0.235910322	0.00631
MYRFL	-0.003113696	0.995646	-0.891553072	0.11957	-1.648100019	0.0063
XKR9	0.102963823	0.665609	0.424136851	0.07055	0.639625264	0.00631
MPPE1	-0.017597035	0.893007	0.176609592	0.16812	0.350566989	0.00632
ZNF827	0.259309156	0.19687	-0.054147386	0.78719	0.546489045	0.00632
PHTF1	-0.158113902	0.30482	-0.070433106	0.64468	-0.419669026	0.00634
PSAT1	-0.294435205	0.072005	-0.164842416	0.31062	-0.446883121	0.00635
USP32P1	0.022828331	0.915091	0.137572181	0.51305	0.572114741	0.00637
ASTN2	0.031925194	0.890124	0.284082471	0.21828	0.628995189	0.00639
ARMCX4	-0.110039392	0.579889	0.275282215	0.15913	0.533218582	0.00642
GAB2	-0.12280957	0.457475	0.144407402	0.37776	0.44483657	0.00643
P11-814E24	0.45634391	0.485796	-0.846000488	0.2323	1.688802336	0.00646
LAMA1	-0.578426272	0.063881	-0.275140262	0.3772	-0.850874277	0.00651
SLC36A3	0.412967644	0.44393	0.565202215	0.2816	1.385632494	0.00653
SFTPB	0.67963259	0.075816	0.432885099	0.25779	1.031720293	0.00653
PJA1	-0.04086409	0.751617	0.143345206	0.26368	-0.351571454	0.00661
EFCAB12	-0.051793368	0.848579	0.233025974	0.36781	0.694948984	0.00667
SUFU	0.248208309	0.194845	-0.095832382	0.61765	0.512387712	0.00668
SPON1	0.440637798	0.281502	0.242831954	0.55281	-1.113426933	0.0067
AC007279.2	-0.171593022	0.817274	0.578198453	0.38557	1.702467513	0.00672
CAMK4	-0.069607091	0.8413	0.510507706	0.13947	-0.943478746	0.00671
SF3A3	-0.223871985	0.075597	0.094908987	0.44997	-0.341909659	0.00672
TARDBP	0.194779911	0.052961	0.050195177	0.61735	-0.273409818	0.00679
SLC28A2	-0.128768832	0.782496	-0.665155485	0.15447	1.2561814	0.00681
GRK4	-0.147470725	0.460097	0.256354958	0.1825	0.524255312	0.00683
MRPL1	-0.295994875	0.056619	-0.061985858	0.68683	-0.418943244	0.00687
TMPRSS3	0.319412071	0.200449	0.299851976	0.22728	0.668882058	0.00693
H2AFZ	-0.26211165	0.088891	-0.24993941	0.10425	-0.415587897	0.00699
FAM106A	0.209997949	0.575317	-0.01334495	0.97143	0.950322193	0.00702
PCDHA10	1.325129855	0.208158	0.727073506	0.49146	2.809010984	0.00702
UXT	-0.069053844	0.552614	-0.16795599	0.14695	-0.314567009	0.00702
DNAJB11	0.02734117	0.823268	0.070431885	0.56412	-0.330647864	0.00705
ZNF556	-0.021386677	0.935572	0.394009491	0.1238	0.686138929	0.00707
DPY19L2P2	0.005617955	0.978169	0.183746339	0.36367	0.541294099	0.00712
RP11-203L2.	0.668721737	0.322909	0.670070157	0.31006	1.685391442	0.00713

CNOT2	-0.122165468	0.161028	-0.15391618	0.07605	-0.234566479	0.00713
LRRC42	-0.114124058	0.274902	-0.175289711	0.09023	-0.281546406	0.00714
MBOAT1	-0.044921769	0.780206	-0.072821771	0.6495	0.428614425	0.00716
EMC3-AS1	0.026646458	0.927831	0.477906932	0.0976	0.771668188	0.00717
PRRG4	0.112327054	0.456707	0.253148664	0.08747	0.398420411	0.00717
FAM131A	0.305149242	0.083948	0.300956311	0.08478	0.470749378	0.00719
NIFKP4	0.73699435	0.159445	0.205108894	0.7012	1.340924405	0.00721
CUX1	-0.01463753	0.915736	0.06430455	0.64107	0.370253216	0.00724
SPACA3	0.426156675	0.504285	0.267703188	0.66975	1.533505437	0.00724
MLC1	-0.509421078	0.54403	-0.085072567	0.91872	-2.311029988	0.00724
AFF1	0.131845393	0.410033	-0.03706122	0.81655	0.428177575	0.00726
CNBP	-0.025085951	0.749866	0.000768994	0.99218	-0.211134051	0.00732
NEFH	-0.172581513	0.565797	-0.098124108	0.74192	-0.819323706	0.00734
WNT11	-0.954383827	0.182941	1.010860905	0.1392	-2.021076681	0.00733
TAF4	0.219741384	0.449335	-0.042371905	0.88326	0.766894697	0.00738
TBC1D8B	-0.04603401	0.831848	0.126988888	0.55773	0.580116557	0.00738
KDR	-0.225997433	0.832388	-0.036256958	0.9729	-2.874846366	0.00741
NOTCH3	0.241634812	0.410889	0.144887451	0.62193	0.784330352	0.00753
ASPA	-0.040124399	0.86957	0.252191771	0.28428	0.622438285	0.00755
CREG2	0.084166424	0.739573	-0.014820237	0.95301	0.65985742	0.00756
CYB5D1	-0.22460205	0.143273	-0.024059364	0.87322	-0.409989697	0.00756
MARK2P8	0.286440202	0.714079	0.325085947	0.66911	1.88709507	0.00758
ACAA2	-0.195338582	0.103276	-0.144700011	0.22559	-0.319930129	0.00759
CLIC2	-0.233943508	0.429996	-0.433370808	0.13902	-0.816175613	0.0076
ERAP2	0.077919273	0.811867	0.446172655	0.1673	0.86759636	0.00761
MYEOV2	-0.232787203	0.112691	-0.030831934	0.82868	-0.39291449	0.00761
CBX1	-0.216980715	0.10414	0.096980764	0.46565	-0.356450475	0.00761
PRRC2C	0.248548831	0.1222	0.098958398	0.53821	0.428817386	0.00763
SAMD12	0.017619411	0.940364	-0.135763814	0.5641	0.626216333	0.00766
SLC16A7	0.056981742	0.830937	0.372384724	0.15885	0.703980431	0.00772
S100A14	-0.47236591	0.104723	-0.181222578	0.53263	-0.775890271	0.00773
NYNRIN	0.243989889	0.197135	-0.009503547	0.95993	0.503091935	0.00776
EAPP	-0.12332665	0.27924	0.152570133	0.17195	-0.303928582	0.00776
FKBP3	-0.190018718	0.065575	-0.007545901	0.941	-0.274708677	0.00777
STX2	-0.35734435	0.109472	0.045608224	0.83735	-0.595395754	0.00777
TPP1	0.3217857	0.300418	-0.20109207	0.51917	0.824844483	0.00779
CISD1	-0.310151344	0.094177	-0.102115706	0.57874	-0.49331004	0.00783
SIDT1	-0.032838507	0.925106	0.472412783	0.15637	0.873886554	0.00784
FAM210B	-0.201812342	0.102815	-0.14107341	0.25058	-0.328615438	0.00789
KRT8P45	-0.42208365	0.488599	-1.167326788	0.07085	-2.065402624	0.00787
MYO1D	0.158044883	0.25118	0.214626261	0.11844	0.365393583	0.00788
NDUFB5	-0.051653226	0.722631	-0.166868977	0.24905	-0.38678115	0.00788
RPL30	-0.086880971	0.41952	-0.054419094	0.6129	-0.286019374	0.00788
YIPF5	-0.014071186	0.908966	-0.037095085	0.76276	-0.32738295	0.00789
ARHGDIG	0.233669613	0.514006	0.227695495	0.52257	0.908750505	0.00795
NRNPA1P3	0.233774259	0.705496	1.02621376	0.07333	1.498554983	0.00795
KIAA1551	-0.462664112	0.102416	0.002486441	0.99299	-0.752119996	0.00794

MUT	-0.101911372	0.202067	-0.077862435	0.32228	-0.211802581	0.00795
CCDC125	-0.137103294	0.688144	0.644446259	0.055	0.889685891	0.008
DGKH	-0.018152355	0.918256	0.310577315	0.07715	0.466259043	0.00798
SPP2	-0.007852964	0.977004	0.405627021	0.12743	0.703122121	0.00799
USP24	0.065744777	0.42399	-0.08627822	0.29347	0.217564166	0.00799
C2CD3	-0.206211371	0.214883	0.198970544	0.22871	0.437546221	0.00801
LURAP1	0.656521086	0.251921	0.930089895	0.09175	1.436087654	0.00801
ARID1A	-0.136654471	0.473461	-0.013052104	0.94531	0.503680156	0.00803
ZNF431	-0.100098478	0.3081	0.003720456	0.96944	0.257600991	0.00803
CMC2	-0.001764237	0.988785	-0.21438259	0.08462	-0.330759661	0.00805
RPS25	-0.053070524	0.617893	0.137017965	0.19634	-0.282187227	0.00805
SLC7A9	0.966261928	0.164597	-1.174115219	0.10113	-1.939816592	0.00805
VTN	0.02655693	0.971027	-0.748378788	0.30635	-1.946466551	0.00804
PEAK1	0.124489278	0.366593	0.194260206	0.1581	0.364122097	0.00807
P11-22C11	0.354547614	0.68084	0.581419954	0.47546	1.980714611	0.00811
TRMT10B	-0.12416288	0.302651	0.046620019	0.68612	0.304078747	0.00813
TBCB	-0.040014825	0.711702	0.03700739	0.73019	-0.289301505	0.00814
SCAND3	0.008425266	0.97046	-0.337753995	0.13568	-0.605037447	0.00816
TMC2	-0.052718569	0.853536	0.283228714	0.30282	0.717296648	0.00817
XK	-0.060000285	0.785101	-0.044086417	0.84095	0.579868229	0.00817
PEX5L	-0.356914389	0.58225	0.841938291	0.17403	1.610467043	0.00818
PRSS3P1	0.765559395	0.10197	0.334583102	0.47481	1.23767806	0.00818
P11-484L7.	0.574136066	0.078901	0.372119756	0.25288	0.848770105	0.00818
SKP2	-0.25874218	0.05948	-0.206442444	0.1273	-0.360178731	0.00819
MAPK1	-0.150799204	0.068448	-0.077897509	0.34589	-0.218455265	0.00822
CHST12	-0.178470364	0.295032	0.124042756	0.45735	0.439012848	0.00823
C15orf27	0.227304595	0.502957	0.480364483	0.14383	0.858255423	0.00823
COMMD9	-0.152639795	0.154581	0.005120514	0.96125	-0.28268918	0.00824
SHOX	-0.014268247	0.964459	-0.096848912	0.75534	0.785173911	0.00824
REN	-0.239422282	0.820941	-1.157887877	0.27695	-2.957862218	0.00828
TMEM14E	0.751610438	0.052345	0.562002691	0.144	0.997507049	0.00831
VPS8	-0.090001442	0.616421	0.282408397	0.11542	0.472393772	0.00831
GJA1P1	0.393788963	0.569338	1.07665169	0.10157	1.707370382	0.00832
POLD3	0.138719311	0.315759	-0.147212442	0.28695	0.363780492	0.00833
PRTFDC1	-0.110758744	0.540484	-0.026690018	0.88143	-0.479378697	0.00836
AB11FIP1F	0.198391121	0.33419	0.10131212	0.61841	0.529582248	0.00836
SDF4	-0.027218148	0.800892	-0.199113816	0.0648	-0.285113234	0.00835
ITGB1	0.043664397	0.720424	0.177054075	0.14656	-0.321757867	0.00837
ACTG1P9	0.874281227	0.052246	0.541566865	0.2306	1.157374387	0.00838
GNL2	0.082395912	0.556256	0.184235965	0.18779	-0.369733355	0.00839
HLA-DPA1	-0.012510821	0.97874	0.620301945	0.18143	-1.306137115	0.00839
SULT1B1	0.03971519	0.845549	0.270300999	0.18444	0.536694096	0.0084
DOCK6	0.014912269	0.948049	-0.266511636	0.24403	0.60036857	0.00841
SOS1	0.129823878	0.583498	-0.078895397	0.73904	0.62326707	0.00843
MTA1	0.11203796	0.377521	0.003090787	0.98052	0.332897132	0.00846
TTC23L	-0.070699153	0.926584	-0.081184244	0.91444	-2.268506686	0.00849
MZF1	0.009554877	0.946601	0.061540635	0.66355	0.369858393	0.00854

NRBF2P5	-0.02014774	0.974294	0.343467611	0.56209	1.478783958	0.00859
DPM3	-0.210833874	0.26802	-0.207679453	0.26868	-0.504356248	0.0086
RHOU	-0.393891241	0.16988	0.25857044	0.36668	-0.75443644	0.00861
C14orf119	-0.054178229	0.563836	-0.152683266	0.10176	-0.246408943	0.00864
RAPH1	0.069539431	0.831013	0.270139565	0.40632	0.852376886	0.00864
RNF181	-0.022181609	0.835135	-0.157971885	0.13664	-0.281149686	0.00865
AFF3	0.033204356	0.898754	0.485210635	0.06188	0.68145784	0.00868
ADAM10	-0.08597982	0.361026	-0.017351347	0.85351	-0.246747014	0.0087
PKNOX2	0.38882751	0.244437	0.602411891	0.06951	0.870615319	0.0087
HELZ	-0.026695521	0.843202	0.076572822	0.56979	0.352979072	0.00874
CCT3	0.046109384	0.678157	-0.032648769	0.76873	-0.291720365	0.00876
SLC25A24	-0.157062933	0.081788	-0.105043648	0.2427	-0.236341287	0.00878
LY9	0.343007915	0.257534	0.317530265	0.28361	0.765687713	0.0088
TRAF5	0.052749603	0.720485	0.152170654	0.29763	0.382002734	0.0088
SLC24A4	-0.025538297	0.948854	-0.176737684	0.65511	0.995723415	0.00883
FBXW10	0.625550193	0.198382	0.478171299	0.32117	1.261825394	0.00884
HIST1H2AE	-0.187292222	0.372558	-0.032224347	0.87793	-0.550297742	0.00885
PPP6R1	0.153281787	0.21026	-0.024454306	0.84163	0.319881802	0.00885
THSD4	0.311712466	0.238668	0.406557663	0.12229	0.688539432	0.00886
ZNF839	0.055976696	0.645644	0.104630417	0.37914	0.309569411	0.00887
MRPL15	-0.094232907	0.428296	-0.214584806	0.06896	-0.312974859	0.00888
TC-527H23	0.657371483	0.152375	-0.048735333	0.91922	1.138870063	0.00891
OSTC	-0.127171137	0.264305	-0.177605819	0.11803	-0.297948908	0.00891
C1orf123	-0.090392284	0.430748	0.041065809	0.71538	-0.299971086	0.00893
CHST7	-0.741963757	0.167856	-0.381339423	0.44245	-1.570866836	0.00893
KMT2B	0.299247776	0.146197	0.134310681	0.51367	0.536311053	0.00897
ARSA	0.314362242	0.108453	-0.010981362	0.95564	0.503799379	0.00901
POU5F1P5	0.348877619	0.645593	0.536740574	0.45951	1.773696855	0.00904
HGSNAT	0.124874857	0.376768	0.145366563	0.30184	0.367861075	0.00904
EIF2B2	0.049157735	0.638999	0.024577439	0.81274	-0.274697497	0.00905
ZNF99	0.103977487	0.814898	0.419258402	0.32362	1.078779693	0.00906
GPRIN1	-0.363545546	0.288963	-0.594123231	0.08151	-0.933626864	0.00907
P11-113D6.	-0.312794569	0.116411	-0.122582596	0.52047	-0.522824474	0.00908
DPY19L1P1	-0.14726323	0.516318	-0.044649203	0.83941	0.559485175	0.00911
TRPM4	-0.171590233	0.376205	-0.039542285	0.8377	0.501534708	0.00913
NDUFS3	-0.122187736	0.228928	-0.191533033	0.05749	-0.265954004	0.00915
LCT	-0.090711012	0.910964	-1.187374388	0.14723	-2.203953426	0.00916
TMEM132A	0.34699437	0.11904	-0.120599904	0.58822	-0.583371277	0.00921
TBC1D17	0.230798673	0.122582	0.114032126	0.44169	0.384494036	0.00927
UNC5C	-0.143192956	0.826444	-0.891359237	0.17407	-1.729006662	0.00929
7-Mar	-0.075477688	0.382808	-0.014994639	0.86181	-0.224660359	0.00935
LRFN3	0.133922199	0.63959	0.156353128	0.58492	0.733068101	0.00937
SSR2	-0.041087455	0.666566	-0.124747625	0.19011	-0.247900438	0.00937
DDAH2	0.254427292	0.050962	-0.22263685	0.08858	-0.341811449	0.00939
ATXN3	-0.18441299	0.259323	-0.011008401	0.9459	-0.425635761	0.00942
ATP5J2	-0.024197184	0.840415	0.150053403	0.20897	-0.312817828	0.00943
AXL	0.052510783	0.797899	-0.32054468	0.11822	-0.534885175	0.00943

SURF4	-0.015427911	0.879462	-0.078468417	0.44003	-0.264401596	0.00946
CERS2	-0.200366914	0.059462	-0.178864977	0.0914	-0.275620102	0.00947
DLK1	-0.275778808	0.631539	-0.249404046	0.66443	-1.493786794	0.00949
MRGBP	-0.188913058	0.074962	-0.146325319	0.15907	-0.274308756	0.00949
CTNS	0.104097431	0.50444	0.147765041	0.33796	0.398632738	0.00951
GPLD1	-0.177928757	0.548088	0.193948153	0.50308	0.750654916	0.00954
RABGAP1	0.130390449	0.355606	0.242876075	0.08413	0.364955485	0.00956
DAPK2	0.616110447	0.088373	0.299807912	0.4063	0.930604197	0.00958
EXOC6B	0.025467335	0.872067	-0.007265741	0.96329	0.4087221	0.0096
MS4A7	0.86718006	0.136257	0.890050806	0.12216	1.477098681	0.0096
GOLT1B	-0.164664456	0.326009	-0.268712934	0.10826	-0.434264931	0.00964
PTGES3	-0.174753027	0.077227	-0.102283396	0.29992	-0.255916626	0.00964
P11-820K3	0.75307516	0.485577	0.71952797	0.49584	2.515134297	0.00965
HSP90AB1	0.11648698	0.27264	-0.191307591	0.07162	-0.274782188	0.00969
TMTC3	-0.024411801	0.802552	0.040610932	0.67531	-0.252657214	0.0097
DPY19L1	0.25788627	0.10442	0.232544123	0.14211	0.40983222	0.0098
FAM21A	-0.079300151	0.480929	0.140888561	0.20846	-0.291697677	0.00979
P11-112N13	-0.116036433	0.646923	0.274136866	0.2739	0.6455329	0.00978
TMEM66	-0.041946051	0.587182	-0.10047555	0.19165	-0.199737271	0.00977
PHKA2	0.001957981	0.990432	-0.284160663	0.08198	0.42070964	0.0099
MAPKBP1	0.169319171	0.349521	0.135530134	0.45293	0.46563884	0.00993
TMEM214	0.046419555	0.728953	-0.040560424	0.7617	0.344491917	0.00994
ZNF830	-0.113215536	0.511861	-0.008406793	0.96077	-0.446628973	0.00994
AF121898.1	0.846346164	0.096155	0.183816352	0.7275	1.255810783	0.00995
HLA-DQA1	0.016274177	0.94337	0.297824992	0.19148	0.587008484	0.00997
AC004893.1	0.292958005	0.667613	0.528591642	0.41938	1.606799969	0.00997
CDC123	-0.111917341	0.276676	-0.015085748	0.88249	-0.265710689	0.00998
IL23R	0.108725597	0.754868	0.438789721	0.18515	0.837836605	0.01006
LYST	0.27301077	0.322145	0.400247438	0.14603	0.707887705	0.01006
NLRP12	0.386527959	0.226401	0.405035144	0.19654	0.796535909	0.01007
SNCA	-0.256143511	0.316064	-0.391956448	0.12354	-0.65907705	0.01005
OSER1	0.031162615	0.786132	-0.110511468	0.33209	-0.29901848	0.01008
C22orf26	-0.062640313	0.924461	0.606640129	0.32282	1.522549193	0.0101
ARSG	-0.001178048	0.996007	-0.040700039	0.86149	0.594051402	0.01012
HSD17B11	-0.350716572	0.098717	-0.395575603	0.062	-0.546463232	0.01013
SERPINB10	0.509809724	0.510002	1.135342163	0.1188	1.827215419	0.01017
CYSTM1	0.344260238	0.121978	0.433567945	0.05137	0.571758057	0.01018
TSPAN12	-0.177866251	0.389978	-0.017477514	0.93234	-0.532400539	0.01023
PWP1	0.062610699	0.503599	0.036112652	0.69775	-0.241324632	0.01025
CIITA	0.807928545	0.062136	0.3575105	0.40679	1.102598958	0.01026
TIMM22	-0.081103271	0.470221	-0.152374322	0.16295	-0.290801433	0.01029
ILF2	0.020124752	0.813156	0.158682105	0.06121	-0.218722027	0.01031
P11-749H20	0.024790185	0.930806	0.258469482	0.35166	0.703009252	0.01031
CARNS1	0.624324456	0.120805	0.578668042	0.15012	1.02605505	0.01032
CDC42P1	0.460101662	0.62979	1.127583211	0.20238	2.174442048	0.01036
HSPD1	-0.142536812	0.338824	-0.199415867	0.18083	-0.381765818	0.01043
UNC80	-0.065174125	0.78928	0.14364505	0.55056	0.611664481	0.01044

FAM189B	-0.182480031	0.345491	-0.21764725	0.26123	0.483602342	0.01045
RBP2	-0.396105172	0.68562	-0.75643636	0.43959	-2.53193806	0.01045
VWCE	0.807150336	0.054554	0.705168647	0.09944	1.074006498	0.01047
PRKAR1B	-0.141216359	0.241403	-0.145217213	0.2214	-0.310354309	0.01048
P11-504G3	0.267483211	0.553305	0.812776248	0.05488	1.079343647	0.01048
STARD9	0.261016362	0.334192	0.412722112	0.12527	0.689797029	0.0105
USP14	-0.008382964	0.969802	-0.257461681	0.24402	0.564219901	0.01052
CNTNAP1	0.094080368	0.678597	-0.039519089	0.86162	0.580164308	0.01061
MFSD7	0.224430607	0.291947	-0.105587843	0.62361	0.535004214	0.01061
SPOCK2	-0.4347813	0.509285	-1.251670391	0.05754	-1.685439201	0.01061
MORN3	0.003792616	0.987991	0.414884533	0.08368	0.611349691	0.01062
GOT1	0.115160124	0.296513	0.07548366	0.49097	-0.284023136	0.01065
FAM189A2	0.052731315	0.928413	0.893844293	0.11115	1.421175248	0.0107
UBE2N	0.04655448	0.589725	0.079595966	0.35435	-0.220608421	0.01071
PARM1	-0.344008346	0.226298	-0.506066642	0.07505	-0.725632738	0.01073
PSMG4	0.24957754	0.138127	0.145802942	0.38228	0.420958165	0.01073
CMTM6	-0.171279131	0.065357	-0.001132679	0.99024	-0.237019228	0.01073
RAI1	0.084438716	0.655828	0.32407859	0.08578	0.48049726	0.01076
UQCRB	-0.175860098	0.186807	0.141377135	0.28727	-0.339707363	0.01078
ANKRD13C	-0.190953121	0.091714	-0.147043769	0.19278	-0.288814033	0.01081
LUM	-0.387738732	0.19158	-0.145503099	0.62392	-0.756951554	0.01081
CCDC122	-0.185952897	0.300287	0.052273247	0.76705	0.447549897	0.01084
DSCR3	0.106324281	0.225471	-0.13099805	0.13189	0.221160517	0.0109
FAM126A	-0.002537433	0.9907	0.18811307	0.3858	-0.553755797	0.0109
GABRE	-0.393553595	0.316057	-0.714284258	0.06941	-1.006157493	0.01089
C11orf86	-0.869968548	0.306786	-1.373894847	0.10789	-2.231674271	0.01094
MAGT1	0.124424873	0.781738	0.478847849	0.27343	1.100183925	0.01101
SLC30A9	-0.049917148	0.670805	0.13247777	0.25757	-0.298666714	0.01102
TLK1	0.135824578	0.379357	0.281010721	0.06803	0.390958319	0.01104
CUL1	-0.131759985	0.064104	-0.018604151	0.7915	-0.180366306	0.01106
KIAA0100	0.096856968	0.361126	0.076888297	0.46792	0.269222304	0.01107
CD3G	0.586534034	0.136661	-0.040142696	0.91899	0.99910352	0.0111
RBBP8	-0.16815252	0.304269	0.025173456	0.8772	-0.416165099	0.01109
XRRA1	0.236287775	0.222129	-0.065645211	0.73051	0.482390732	0.0111
FAM57B	-0.149604337	0.704608	0.23114904	0.53422	0.910121745	0.0111
RNF130	0.0026499	0.977571	0.174212888	0.05914	-0.240596821	0.01114
PLEKHA6	-0.15049952	0.350996	-0.126384891	0.43266	0.408140043	0.01116
ZNF716	0.58967517	0.149066	0.155620324	0.70562	1.012401561	0.01116
CSDE1	0.027280285	0.680218	0.007523626	0.90941	-0.167905091	0.01123
ISL1	-0.453411218	0.496073	-0.220368036	0.73795	-1.752700353	0.01123
ITGB3BP	-0.370458478	0.066721	-0.33808812	0.09323	0.508573411	0.01122
ZNF281	-0.111576071	0.262072	-0.028815241	0.77093	-0.25234176	0.01123
GPC4	0.167338155	0.491192	0.406732382	0.09248	0.612410881	0.01126
PSME1	-0.00865107	0.923795	-0.063458382	0.48021	-0.229699466	0.01128
HSPD1P6	0.762737296	0.112641	0.570323278	0.22392	1.164175377	0.01132
VPS13B	0.23972353	0.244992	0.091887027	0.6556	0.521491355	0.01135
SPATA6L	0.1977861	0.323064	0.13142841	0.50699	0.497623914	0.01136

PIK3C2B	-0.279625245	0.095476	-0.269227009	0.10814	0.4224088	0.01137
DNAJC8	0.000187788	0.998362	0.103327308	0.25583	-0.232244494	0.01141
PAIP2	0.061489731	0.583644	0.189326079	0.08841	-0.28476119	0.01143
PLA2G12B	-0.67752241	0.493826	-1.686400943	0.09205	-2.60436842	0.01146
AP000662.9	0.215489901	0.743675	0.578397158	0.35234	1.50050519	0.01151
C1orf116	-0.228356767	0.22919	0.117529672	0.53526	0.478636348	0.01153
CETN3	-0.236269288	0.173552	-0.202468424	0.23739	-0.438427701	0.01154
SNX4	-0.178716761	0.072806	-0.162118531	0.10156	-0.25101825	0.01157
SYVN1	0.099068477	0.609357	-0.179560869	0.35385	0.486981459	0.01157
P11-210K20	0.588098933	0.259485	-0.001783834	0.99732	1.274956694	0.01159
IDUA	0.276364454	0.230816	0.020458877	0.92974	0.57270726	0.0116
USP34	0.174498776	0.207828	0.190604818	0.16848	0.349249123	0.01162
RNF25	0.073719368	0.526968	-0.17879196	0.12471	-0.299040396	0.01163
MRPL36	-0.165773715	0.160867	-0.194936428	0.09354	-0.29820033	0.01166
KLF11	0.205982833	0.109726	-0.179616062	0.16431	0.322863072	0.01171
SRCAP	0.09262854	0.733468	-0.171303476	0.52873	0.684190721	0.01171
CXorf40B	-0.177985547	0.206112	-0.260725712	0.06189	-0.356228246	0.01172
LYPD1	-0.799043342	0.139331	-0.467415894	0.38387	-1.37518225	0.01174
EIF4EBP1	-0.000607676	0.997165	-0.232522973	0.17363	-0.434660083	0.01175
CAPN13	0.377061714	0.136902	0.061925725	0.80691	0.634427204	0.01176
MIER2	0.89892355	0.129237	-0.014778803	0.9807	1.481843886	0.01177
IHH	0.192505116	0.517865	-0.269739396	0.36525	0.748340361	0.01178
PITPNB	0.313691791	0.063423	0.026173885	0.87682	0.424921593	0.01178
BTNL3	-0.486199232	0.502616	-0.476817375	0.50965	-1.867501688	0.01179
N6AMT2	-0.199549765	0.303334	-0.111149188	0.55135	-0.497929386	0.0118
NBN	-0.189110095	0.057143	0.048758707	0.62008	-0.249679146	0.01181
GTF2IRD2	-0.023670973	0.912611	0.331610477	0.11935	0.535167127	0.01185
SLC11A1	-0.027482258	0.917284	-0.004443077	0.98657	0.646256901	0.01186
AKR1B15	-0.300840131	0.190266	0.321732301	0.15045	0.561393634	0.0119
PDX1	0.411255606	0.247468	-0.449449703	0.21329	0.8846559	0.0119
TXNL4A	-0.214443755	0.052585	-0.157062176	0.15221	-0.276902469	0.01194
CADPS	-0.528818638	0.242664	-0.771626164	0.08811	-1.144940842	0.01195
HGF	0.068428592	0.919674	0.43726063	0.51899	-1.71325297	0.01199
UFM1	-0.11097581	0.351291	-0.208905625	0.07895	-0.299139624	0.01204
PHF6	-0.150632678	0.416256	-0.170584308	0.35627	-0.465543745	0.01205
RIN2	0.061580769	0.762366	-0.241728139	0.23473	-0.514135652	0.01207
CAND1	-0.138073481	0.133784	-0.076839521	0.40331	-0.231052116	0.01208
FAM131C	-0.440041621	0.53309	-0.59744225	0.39601	-2.053318508	0.01208
PDXP	-0.900464241	0.202362	-1.046194562	0.13825	-1.78277364	0.0121
SFXN4	-0.257151075	0.199983	-0.186147828	0.35155	-0.506871836	0.0121
CD3D	-0.126658704	0.857039	-0.80861453	0.25621	-1.869988211	0.01212
MKKS	-0.169115004	0.21707	-0.134746041	0.32102	-0.342593611	0.01211
MROH6	-0.114751018	0.738965	0.45857548	0.18058	0.844102962	0.01214
GYS2	-0.85019365	0.383655	-0.285881383	0.76492	-2.68642332	0.01217
ARFGAP2	-0.158756262	0.072043	-0.168759779	0.0542	-0.220866167	0.01219
NRNPA1P5	-0.313637727	0.162179	0.15359108	0.48547	0.549510562	0.01218
ARL5AP2	1.148071255	0.249359	1.06575781	0.27951	2.366473792	0.0122

MTHFD1P1	0.260461707	0.567034	0.262884918	0.5545	1.071978528	0.0122
PPIL1	-0.196228797	0.070221	0.029232458	0.78498	-0.271110913	0.01225
SLC1A6	-0.170933051	0.438337	0.128839194	0.54182	0.517145223	0.01226
FAM200B	-0.266914387	0.122587	0.230519037	0.17772	-0.434355741	0.01226
BLOC1S6	-0.116364679	0.592338	0.128046632	0.55551	0.543661314	0.0123
CPSF1	0.338405043	0.075599	0.296635613	0.11669	0.473511377	0.0123
C10orf99	-0.413837279	0.714017	-1.404610284	0.22733	-3.596294046	0.01231
TSPAN10	0.387646632	0.209913	0.023145334	0.94054	0.760132848	0.01231
SETD1B	-0.09061105	0.686287	0.023096325	0.9168	0.547255486	0.01235
ARL1	-0.00720907	0.937856	-0.084732612	0.35909	-0.23125808	0.01244
CA4	-0.251984273	0.786839	-0.458961776	0.62181	-2.403875456	0.01245
GIF2-C20orf	0.214710972	0.483075	0.29626557	0.33052	-0.780512997	0.01245
CYP3A43	-0.349139705	0.402246	0.346519755	0.3612	0.925420699	0.01246
SDHD	-0.041583012	0.664102	-0.124672199	0.19043	-0.239707559	0.0125
CLCN6	-0.073267656	0.651029	0.051253576	0.75048	0.40184648	0.01253
UBP1	0.222595581	0.064054	0.105008204	0.38115	0.299393552	0.01254
BMS1P17	0.715866438	0.114286	0.116550226	0.79968	1.112717899	0.01255
WBP4	-0.118691632	0.346417	-0.006119893	0.96047	-0.315773764	0.01259
C11orf58	-0.110925118	0.238822	-0.034220115	0.71527	-0.23476178	0.0126
RAX2	1.111452413	0.153891	0.668292596	0.397	1.837635447	0.01263
P11-495P10.	0.054696851	0.903577	0.637899253	0.12808	1.029974275	0.01265
8-Sep	0.098931451	0.320439	0.118660604	0.2302	0.246889029	0.0127
FIP1L1	-0.197967756	0.14105	0.037317426	0.78075	-0.335231181	0.01273
CCDC144A	-0.218817223	0.293077	0.059841409	0.77137	0.509394008	0.01275
FAM45A	-0.218698412	0.118671	0.081841257	0.55467	-0.348262661	0.01276
EEF1D	0.0361912	0.733025	-0.035764708	0.7358	-0.264401231	0.01279
C9orf64	0.682149628	0.097079	0.695396117	0.08306	0.99445006	0.01284
INGX	1.193539876	0.100852	1.165554776	0.10426	1.751710051	0.01285
PLCL1	0.305971605	0.498177	0.576879154	0.20027	1.118251596	0.01285
SGMS1	0.30071908	0.069512	0.153686256	0.35275	0.411659882	0.01284
SYCP1	0.25020785	0.49958	0.518474318	0.14782	0.88108671	0.01284
ANKUB1	-0.15301433	0.817423	0.507939698	0.41928	1.543773272	0.01291
MPV17L	-0.007446197	0.968236	0.079571706	0.66857	0.461122274	0.01291
P11-390M11	0.464476008	0.264849	0.415705241	0.3063	0.976274479	0.01292
P11-61N20	-0.079585239	0.804548	0.019123419	0.95195	-0.822932256	0.01295
TMEM234	0.140304138	0.464455	0.212304338	0.26289	0.471487222	0.01297
GHITM	-0.021154428	0.813965	-0.097609756	0.27635	-0.223523936	0.013
KIAA0226L	0.333349791	0.678246	-1.103446119	0.18367	-2.242440761	0.013
RASA3	0.167845611	0.434752	0.202723052	0.33802	-0.551874724	0.01307
AC110926.4	0.594868682	0.12061	0.279553745	0.46657	0.919657294	0.0131
SNAP23	-0.146892398	0.239119	-0.121025004	0.33022	-0.309440598	0.01312
COLEC10	-0.491425819	0.238884	-0.660902362	0.11305	-1.037019405	0.01316
LYPLAL1	-0.143298769	0.358605	-0.111180518	0.46837	-0.3886033	0.01315
STAG2	-0.053307187	0.535263	0.104637449	0.22191	-0.21335179	0.01315
FSCN1	-0.046761997	0.870292	-0.043013383	0.8804	-0.712186664	0.01318
NDUFV3	-0.165880822	0.051747	-0.064768819	0.4403	-0.209486757	0.01322
ANGPTL2	0.001165711	0.995813	0.136262554	0.53745	-0.557800863	0.01329

HEY1	-0.655801704	0.122237	-0.072363025	0.86266	-1.053911979	0.0133
VPS13C	-0.067537457	0.621205	-0.138071172	0.31227	0.337747431	0.0133
FAHD2CP	0.017471209	0.949434	-0.11120629	0.68429	0.665460669	0.01339
CAP1	-0.025177949	0.792407	0.150138576	0.11609	-0.236604251	0.01344
CENPB	-0.079797348	0.527311	-0.07716943	0.53902	-0.312660011	0.01346
CEP85	-0.047299691	0.696476	-0.025383066	0.83341	-0.300779468	0.01346
P11-162P23	0.811636926	0.135559	0.689651633	0.20417	1.33717115	0.01351
RPL7AP11	-1.251258041	0.067889	-0.88534921	0.17325	-1.759952073	0.01351
RPL17P22	-0.490443661	0.45868	-1.388611211	0.0502	-1.953170467	0.01354
CSMD1	-0.042070753	0.959442	0.723479406	0.36077	1.958095946	0.01356
INTS3	0.1109904	0.230042	-0.06272899	0.49678	0.227394206	0.01358
MRPL48	-0.12032838	0.356978	-0.165702744	0.20128	-0.321447539	0.01358
PPP2R2D	-0.095867007	0.282524	0.005977623	0.94603	-0.220385763	0.01359
ASAP2	0.069172884	0.573246	0.136483966	0.26478	0.301906049	0.01367
FDPSP7	0.814494219	0.080696	-0.289979737	0.55651	1.12593585	0.01367
POLR2G	-0.123442984	0.308215	-0.127910208	0.28648	-0.299124199	0.01374
ZNF826P	0.445690103	0.34234	0.4853118	0.28444	1.0978703	0.01376
NRP1	-0.2429857	0.122644	-0.15706711	0.31726	-0.387393219	0.01377
MAGI2	-0.219947707	0.374129	-0.008302048	0.97274	0.596603849	0.01379
P11-139K1	0.42326592	0.419167	0.699944251	0.16552	1.220342772	0.01379
CLCN5	-0.103022389	0.618019	0.106084657	0.60604	-0.509479976	0.01384
GYG1	-0.045873101	0.710519	-0.020705303	0.86521	0.298930432	0.01389
L1CAM	-0.57569041	0.454517	0.276255395	0.72197	-1.999424863	0.01389
MAN1B1	-0.243298413	0.145624	-0.090641556	0.58691	-0.41125789	0.0139
NDUFAB1	-0.178897034	0.154853	-0.239035004	0.05587	-0.309899287	0.01392
TNS4	0.663994744	0.234655	0.215952973	0.70185	1.333848793	0.01395
PTPN3	0.018551817	0.89807	0.002778922	0.98463	0.353975322	0.01397
CHRNB1	0.025835187	0.881483	0.12128678	0.47267	0.413220423	0.01398
KCNG4	-0.22422397	0.559513	0.034289942	0.92569	0.868891184	0.01399
CPSF6	-0.150290507	0.102531	-0.0431379	0.63808	-0.225810961	0.01403
GGPS1	-0.087757669	0.317484	0.050456645	0.55977	-0.214826094	0.01404
KRT8P39	0.473593114	0.203504	0.257193575	0.48718	0.880081967	0.01403
ELAC1	-0.028064431	0.890816	-0.156581986	0.43609	-0.506408206	0.01413
PAPLN	0.139477329	0.566163	-0.002073588	0.99312	0.584564253	0.01412
P11-618I10	-0.359770353	0.672138	0.999322629	0.15163	1.652903298	0.01414
UVSSA	0.118093802	0.492817	0.053215002	0.75592	0.417843319	0.01414
ITGB7	-1.284757989	0.224245	-1.277310738	0.23005	-2.890359026	0.01419
ZUFSP	-0.091749993	0.488704	-0.152933713	0.24306	-0.325904893	0.01419
SNRPC	-0.038014921	0.738259	0.142079667	0.2073	-0.279678336	0.01422
MAOB	-0.139004416	0.784996	-0.220374049	0.66433	-1.271351325	0.01423
BGN	0.425665258	0.189669	-0.333210809	0.30676	0.794222818	0.01424
HIST3H2BA	-0.528807292	0.359139	0.368500465	0.5046	-1.492420538	0.01427
KLB	-0.219561907	0.405345	0.056709826	0.82271	0.606567432	0.01427
ARL13B	0.157739124	0.329438	0.221149317	0.16468	-0.398706887	0.01432
HOXC11	-1.442285095	0.080267	-1.587419621	0.054	-2.035900158	0.01432
PIGP	-0.16367944	0.265468	-0.122384928	0.39308	-0.353608448	0.01433
TUBAL3	-0.666685621	0.444632	-1.434476501	0.10435	-2.228180277	0.01436

DARS	-0.18644165	0.053213	-0.089200962	0.35392	-0.236163125	0.01437
ANGPTL7	0.139432183	0.847478	0.249441579	0.72588	1.636522824	0.01437
GRAMD1A	0.275429816	0.095567	0.132844528	0.42112	0.403313213	0.01439
RDH13	-0.18665393	0.257003	-0.084945286	0.59813	0.388927125	0.01444
P11-551G24	-0.034217535	0.962081	0.210805351	0.7554	1.519159141	0.01442
ZNF285B	0.560057136	0.419471	0.86043233	0.19223	1.56090753	0.01443
HNRNPMP1	0.049861105	0.9412	1.040807804	0.08948	1.478608181	0.01446
ARL6	-0.415770611	0.052913	-0.277044571	0.18636	-0.522797867	0.0145
RX5-TAX1E	0.084134468	0.68971	0.374468665	0.06642	0.499137456	0.01455
PCDHB5	-0.017861013	0.953709	0.377651475	0.2166	-0.758940864	0.01454
APEH	-0.246345955	0.052941	-0.242554093	0.05569	-0.310586935	0.01458
SLC35B1	0.095389237	0.289791	-0.144625244	0.10814	-0.221386749	0.01458
AP002387.1	0.002843049	0.995531	0.660084883	0.1634	1.13939535	0.01463
ZNF227	-0.315667812	0.061716	-0.299430112	0.07423	-0.411482099	0.01463
NARS2	-0.018607326	0.918348	-0.338853893	0.06054	-0.442025364	0.01464
TGS1	-0.141526281	0.243881	-0.237132553	0.05004	-0.296094979	0.01469
MRPL20	-0.125090525	0.196487	-0.05370771	0.57327	-0.235682522	0.01472
STAM	-0.214854015	0.146167	0.05917596	0.6881	-0.361143002	0.01479
P11-427H3	-0.166794656	0.326906	0.139483643	0.40657	0.408676495	0.01483
HIAT1	0.018335066	0.818479	-0.028942068	0.71587	-0.195080891	0.01485
P11-274B21	-0.316047066	0.076532	0.143912495	0.39948	0.413921002	0.01484
PLA2G2D	0.573454805	0.234205	0.199602659	0.68065	1.127299457	0.01496
NDOR1	0.19828241	0.177517	0.087312252	0.54757	0.350857076	0.01498
GPR4	-0.254111274	0.786647	0.780934679	0.3239	1.814532308	0.01499
PPIG	-0.137373918	0.180733	-0.024463393	0.81088	-0.249792309	0.01501
TRAPPC2	0.004199028	0.971994	0.144160822	0.22197	0.285503164	0.01508
GHRL	0.68190313	0.202074	0.783585667	0.14183	1.272170072	0.01509
RYR2	0.408859184	0.360859	-0.034324599	0.93808	-1.089891838	0.0151
CSE1L	-0.166256565	0.176523	0.103679542	0.39812	-0.298910072	0.0151
INO80D	-0.074862928	0.671383	0.08061359	0.64666	0.426804906	0.01513
LIN28B	-0.363027279	0.283065	-0.300508495	0.37376	-0.822486628	0.01514
CCDC178	-0.638858848	0.110497	-0.684073075	0.07694	-0.967785389	0.01516
PTCHD1	-0.110541846	0.743141	0.10267978	0.76061	0.81775944	0.01517
FUBP1	-0.129278975	0.091943	0.080140034	0.29387	-0.186156238	0.01522
PPDPF	0.360273893	0.118932	-0.204962542	0.37627	0.558392134	0.01523
C2orf91	-0.253520398	0.285546	0.401018109	0.07384	0.543631933	0.01524
NEU1	0.011934457	0.941555	-0.255074374	0.11718	-0.395924192	0.01526
P11-307A17	-0.064973345	0.814681	0.258349342	0.33948	0.650873184	0.01533
ADH1C	-0.266554265	0.61006	-0.8493647	0.10487	1.253524663	0.01538
PCTP	-0.189087831	0.16557	-0.1628716	0.22887	-0.330280848	0.01539
P11-496H1	0.070238915	0.838281	0.182500254	0.58301	0.781378097	0.0154
LIPA	0.002183971	0.986236	-0.166813781	0.18618	-0.306969133	0.01547
C11orf74	-0.186024377	0.342138	0.355605922	0.06382	-0.478092233	0.0155
SEC14L2	0.35845032	0.27882	0.364446487	0.26856	0.790479518	0.01551
C4orf19	0.349081005	0.059088	0.101756014	0.58205	0.446440262	0.01553
CAMTA2	0.205929537	0.356368	-0.163045827	0.46596	0.535299896	0.01555
LRP2BP	-0.268258526	0.234597	0.173224475	0.42101	0.517985985	0.01556

PDGFA	-0.398560524	0.080579	0.090118135	0.68611	-0.550676303	0.01559
?11-578F21.	-0.486639787	0.326026	-0.884742247	0.07395	-1.203729967	0.01559
FAM120C	-0.014965203	0.951939	0.243399045	0.31517	0.588264382	0.01567
FAM92A1P2	-0.212950525	0.676197	0.053325364	0.91199	1.096848036	0.01576
AP2A1	0.020785615	0.845803	0.092695854	0.38261	0.256100786	0.01579
CPLX4	1.022956818	0.136465	0.79971521	0.24192	1.583655038	0.01579
ERC1	-0.038488643	0.824158	0.200853032	0.24484	0.416803614	0.01579
RPS7P1	-0.409662388	0.855938	-1.009679276	0.65509	-5.944591239	0.01579
GATA3	-0.318740012	0.560644	0.608214745	0.2582	-1.384740044	0.01581
SNRPG	-0.105015164	0.384126	0.054686983	0.64753	-0.291641841	0.01582
LATS1	0.149799816	0.134415	-0.022432825	0.82217	0.240094509	0.01584
PAFAH1B2	-0.044432578	0.577236	0.022683626	0.77503	-0.192177081	0.01585
RNF5	0.211220501	0.071794	-0.171736825	0.14414	-0.285547586	0.01586
EXOC5	-0.129976805	0.279248	0.093651233	0.43393	-0.29028011	0.01589
POTEF	0.085950255	0.632692	0.046439886	0.79562	0.43128398	0.01589
CYB5RL	-0.036284998	0.824312	-0.050012718	0.75618	0.383451363	0.0159
GPR82	-0.329685756	0.39331	0.314835809	0.39643	0.886166019	0.0159
MRFAP1	0.055879779	0.574265	-0.097163769	0.32799	-0.240287336	0.01592
AC008746.5	-0.479214033	0.459715	0.362527302	0.51954	1.279365481	0.01593
OTUD6B	-0.302084925	0.195098	-0.081089533	0.72542	-0.562058685	0.01595
RILPL2	-0.040278209	0.80372	0.05000564	0.75399	0.382072308	0.01597
KIAA0513	-0.097297635	0.578618	-0.025542569	0.88331	0.417178823	0.01613
MPHOSPH1	-0.169639372	0.277939	-0.130628839	0.40236	-0.376780104	0.01616
NUDT15	-0.130323658	0.1729	-0.094084855	0.31834	-0.229588161	0.01619
NME1-NME2	-0.137115684	0.253504	-0.191354025	0.11054	-0.288751057	0.0162
AC012066.1	0.805698564	0.141646	0.599281643	0.27228	1.281272877	0.01623
ZNF628	0.227389587	0.491743	-0.061847834	0.85211	0.77484003	0.01623
ANO6	-0.013201684	0.906415	-0.17549661	0.11791	-0.270238562	0.01624
HMGB3P4	0.455696623	0.533572	1.190446076	0.07708	1.594139009	0.01636
AC024937.6	0.0161238	0.985792	0.65511363	0.44537	1.986914103	0.01638
ABCA6	0.5410823	0.127009	0.553907607	0.11449	0.841451995	0.0164
CHI3L2	0.182092656	0.735767	-0.25249437	0.65215	1.226654525	0.01642
FEZ2	0.079906939	0.463516	-0.175058309	0.10719	-0.261771863	0.01642
PDLIM2	-0.115017549	0.723505	-0.232327947	0.4723	-0.784539409	0.01644
PRSS1	0.726616367	0.170019	-0.017580764	0.97352	1.269961163	0.01646
MRPL52	-0.196270142	0.1484	-0.184237852	0.16773	-0.323660264	0.0165
SLC4A1	0.540209021	0.541619	0.202081045	0.82013	1.910210904	0.01652
LTB4R2	-0.003196938	0.990614	-0.016226595	0.95181	0.629371379	0.01661
MYT1L	0.073699248	0.864837	0.288907444	0.49273	0.982287115	0.01662
C3orf72	0.083547167	0.784693	0.470128082	0.10693	0.694749409	0.01667
TRAV8-5	-0.045389367	0.843018	0.349030445	0.11514	0.529689251	0.01668
SLC36A4	0.167319732	0.201653	0.222806159	0.08501	0.31130803	0.01671
GTF2F1	-0.143882452	0.197245	-0.117936624	0.28931	-0.26729466	0.01675
KIAA1671	0.000866783	0.995692	-0.050604088	0.75211	0.382414848	0.01675
RP1L1	0.059174553	0.906405	0.390710972	0.40863	1.118600238	0.01675
AL162431.1	1.363762808	0.062115	-0.066725173	0.93218	1.717038469	0.01677
OMG	-0.353520332	0.352083	0.136270368	0.70761	0.866595846	0.01679

TERF2	0.2600794	0.104854	-0.204104717	0.20413	0.380203111	0.01684
STRIP2	0.173262058	0.507856	0.251109098	0.3327	0.618868002	0.01684
KRT16	-2.100021881	0.217189	-1.70633621	0.30378	-4.55448786	0.01688
SLC6A10P	1.074416068	0.099914	0.465907759	0.48316	1.490528847	0.01693
DYNC1I2	-0.041708689	0.590524	0.099496276	0.19692	-0.185294113	0.01696
PGBD4	0.012861832	0.937016	0.054290985	0.73422	0.378743044	0.01696
RBFOX2	-0.220114039	0.156159	0.204337397	0.18655	-0.370560572	0.01698
INIP	0.015843005	0.879238	-0.108603242	0.29597	-0.249784113	0.017
RP3-522J7.5	-0.085987141	0.876932	0.264695279	0.60977	1.170894407	0.01701
MAGED2	0.01200941	0.935751	-0.098122588	0.51	-0.355323239	0.01714
P11-305M3	-0.32763173	0.116086	0.108790154	0.58799	-0.498131085	0.01716
RPS20P15	-0.158378493	0.824011	0.620722273	0.32485	1.432931236	0.01714
LSP1	-0.035703916	0.936381	0.027253293	0.94957	-1.160639481	0.01717
AC006509.7	-0.055953554	0.905929	0.003210374	0.99442	1.03216896	0.01719
ERGIC2	-0.232705337	0.052496	0.134510551	0.25472	-0.284089503	0.01721
MICA	-0.028018185	0.847777	-0.192688455	0.18455	-0.348238827	0.0172
MOB2	0.812307399	0.093545	0.620547528	0.19895	1.148110253	0.0172
BMS1P2	-0.097339729	0.689015	0.249867954	0.29202	0.563310704	0.01726
CCL22	-0.078639119	0.839176	0.375121197	0.30635	0.857748132	0.01725
HSPE1	0.036735771	0.785878	-0.069540337	0.60662	-0.322748066	0.01728
G3BP2	-0.055877038	0.62431	-0.051172025	0.6536	-0.271542449	0.01737
P11-192M23	-0.638561878	0.25602	0.108857237	0.82593	1.104121702	0.01739
USP4	0.078111891	0.551218	0.031560528	0.80896	0.30949854	0.01746
FUOM	-0.063130626	0.814721	-0.010044786	0.96994	-0.656568394	0.01752
ZNF19	0.122001773	0.578956	0.164643016	0.44449	0.506738793	0.01753
KLHL1	-0.023795485	0.975291	0.107428147	0.88595	-2.375748253	0.01755
RP11-3J10.4	-0.181172966	0.301823	0.014896399	0.93089	-0.419075898	0.01756
P11-472I20	0.223247642	0.650155	0.567530309	0.22488	1.085503038	0.01763
ARHGAP30	0.114996983	0.738077	0.273192573	0.40845	0.773020686	0.01771
SERPIND1	0.513173567	0.356171	0.129756885	0.81618	1.231600274	0.01771
HDAC1P1	0.015471328	0.973675	-0.105560526	0.81876	1.015145377	0.01774
TD-2194F12	0.290918561	0.561692	0.735502131	0.11284	1.082751385	0.01775
LSMEM1	0.492867965	0.301725	0.360354012	0.4251	1.055122272	0.01779
OR51E2	0.000923653	0.998837	0.081072458	0.89396	1.332309899	0.01779
SKAP2	-0.142201033	0.516929	0.001698786	0.99379	-0.52021718	0.0178
PVRL1	-0.063560512	0.743188	0.032050237	0.86807	0.456437523	0.01781
COL10A1	0.153733375	0.753719	0.88248438	0.06402	1.110403993	0.01784
MPLKIP	-0.038208863	0.723006	0.073365435	0.48571	0.248999448	0.01784
ADAMTS6	-0.238929148	0.382338	0.335597107	0.21218	-0.649476626	0.01788
PLAA	-0.127979953	0.280372	-0.153666411	0.19309	-0.280818441	0.01788
FBLN7	0.854590721	0.131908	0.972700473	0.08229	1.302067857	0.01791
PLXNA2	0.100048795	0.612413	0.279677957	0.15555	0.466393436	0.01793
SLC16A4	-0.251244825	0.464818	0.007495859	0.98248	-0.819279924	0.01793
LURAP1L	-0.328702689	0.087881	-0.259561184	0.17229	-0.456378876	0.01794
AC012513.4	-0.163292812	0.680802	0.503102776	0.17348	0.864927162	0.01798
COPB2	-0.028574065	0.71551	-0.037815282	0.62881	-0.185615574	0.01798
CYP2E1	0.487994073	0.273531	0.454812724	0.3008	1.031672348	0.01798

F2	-0.005452183	0.991992	-0.81203402	0.13532	-1.287623026	0.01803
LDHAL6CP	0.438452479	0.203101	0.254978059	0.45674	0.795717152	0.01803
CSDC2	-0.430272556	0.191802	-0.578294114	0.07692	-0.796454563	0.01804
RPTOR	0.225037223	0.144295	0.287688749	0.06017	0.362090665	0.01804
CTSC	-0.098631411	0.542708	-0.19288481	0.23359	-0.383061216	0.01808
PARK7	-0.093469768	0.393885	0.02095626	0.84743	-0.259434558	0.01808
UBE2C	-0.309018396	0.07244	0.001497506	0.99301	-0.406593361	0.01808
PPIL3	-0.199536241	0.089066	-0.049236251	0.67053	-0.277094451	0.0181
P13-130D24	0.325034682	0.396097	-0.463587635	0.25442	0.850286875	0.01815
FOXJ2	-0.017665226	0.919197	0.044033314	0.79796	0.40405465	0.01817
ACBD6	-0.157207935	0.183492	0.178308632	0.12494	-0.279525162	0.01821
SERPINF1	0.087457174	0.642244	-0.191180909	0.3098	-0.446241635	0.01822
KIFAP3	-0.034700103	0.832675	0.071102188	0.66313	-0.389542529	0.01824
GTF2F2	-0.094762615	0.37962	-0.057648978	0.58768	-0.255857135	0.01832
PHGDH	-0.169126434	0.133914	-0.190609299	0.08989	-0.266201379	0.01838
AC093668.2	0.824521309	0.650969	-0.430422399	0.81488	-4.88895712	0.01843
SEPSECS	0.286699908	0.107706	0.322287576	0.06606	0.42189784	0.01845
TROVE2	-0.05918395	0.406406	0.018522882	0.79387	-0.167725268	0.01844
ZNF879	-0.290553084	0.179233	-0.363879522	0.0861	-0.505026216	0.01844
MAK16	-0.119289804	0.406236	-0.223457641	0.1193	-0.338585135	0.01847
TD-3113P16	-0.140090075	0.761201	0.433994016	0.31195	0.988023328	0.01848
GLA	-0.186665685	0.342732	-0.19998475	0.30802	-0.466724359	0.0185
COPS2	-0.156867137	0.097593	-0.08771604	0.35278	-0.222749546	0.01853
NFIC	0.219347637	0.484763	0.538017491	0.08399	0.728428939	0.01855
C8orf33	-0.14945999	0.30821	-0.218643117	0.13526	-0.345469725	0.01856
P11-44F14.	0.079825167	0.830361	0.41229792	0.24537	0.822342881	0.0186
SEMA4A	-0.044992015	0.882362	0.566373211	0.05298	0.696553913	0.01875
TOPORS	0.02834609	0.746235	0.068549898	0.42982	-0.206588928	0.01875
PTRH1	0.310027172	0.178789	0.128410085	0.57387	0.527140165	0.01877
AEBP2	0.129300761	0.271445	0.155110556	0.18539	0.274410427	0.0188
SCGN	-0.353423861	0.61523	-0.428311403	0.54173	-1.679933991	0.0188
GPR75	0.363199435	0.184672	0.156110356	0.56684	0.62989561	0.01884
ST8SIA6	-0.206770913	0.537268	-0.158748805	0.63405	-0.796922665	0.01886
TOP1MT	0.004477816	0.96951	-0.166292707	0.15267	-0.27664924	0.01886
FOXD2	0.173244276	0.775853	-0.260671792	0.6687	-1.453092028	0.01891
SRP68	0.141804702	0.316267	-0.037987425	0.78833	0.330936646	0.0189
C6orf132	-0.144482227	0.425444	0.012628408	0.94413	0.420978231	0.01894
ARHGDIB	0.150602763	0.561507	-0.24589547	0.34398	-0.616828524	0.01898
RALGPS1	0.020714906	0.911136	-0.193442067	0.29628	0.432407157	0.01905
RP11-266I3.	0.92032293	0.150628	0.922785467	0.14265	1.449459589	0.01904
TD-2522E6	-0.345009761	0.679381	0.077449326	0.92088	1.689658742	0.01909
ZCWPW2	0.336123382	0.286231	0.49127044	0.10628	0.705864048	0.01914
EPB41L4B	-0.496136263	0.056087	-0.36937384	0.1531	-0.608205259	0.01916
BCAT1	-0.171061911	0.356945	0.276538782	0.13576	-0.435087714	0.01921
KCTD6	-0.232515602	0.152486	-0.193661868	0.22363	-0.376996406	0.01921
PPP1R8	-0.052898422	0.569679	-0.111250509	0.22841	-0.218135476	0.01918
PTPN11	0.138937827	0.141808	0.169243844	0.07308	0.221410844	0.0192

P11-343C2.	0.073903468	0.613721	0.051330543	0.72325	-0.347457907	0.0192
RTCA	-0.003714827	0.972422	0.017711443	0.86774	-0.252378316	0.01921
H2AFY2	-0.05642766	0.784225	0.203078672	0.32215	-0.484098267	0.01926
CELSR2	0.004826384	0.986564	0.506736393	0.07219	0.659381045	0.01928
ACHE	0.731143653	0.082769	0.271115778	0.52494	0.989651569	0.01945
SOCS4	0.021257653	0.814671	-0.141250349	0.11801	-0.211979071	0.01948
XPO6	0.03688801	0.763984	0.11574479	0.34516	0.286550416	0.0195
TXNDC17	-0.232787192	0.177731	-0.173428161	0.31033	-0.403501949	0.01957
CERK	-0.014565593	0.887515	-0.092576789	0.36681	-0.241152285	0.01958
DPT	0.275004151	0.719353	-0.172752925	0.82274	1.710017989	0.01959
MYLPF	-0.756310984	0.360759	0.359663204	0.64758	-2.326377722	0.0196
DHRS9	-0.554566573	0.431979	-0.700728326	0.31969	-1.660180734	0.01969
MEP1A	-0.298799859	0.663253	-1.223578761	0.07467	-1.601334845	0.01971
JHRF1BP1I	-0.178026619	0.238598	-0.290885049	0.05337	-0.352159203	0.01972
GOLGA7	-0.085378444	0.453209	0.005137025	0.96385	-0.265755646	0.0198
CDS2	0.056966706	0.588043	-0.189024612	0.07239	0.244400774	0.01995
MEFV	0.423752679	0.261076	0.205830736	0.57948	0.850774987	0.01996
TIMMDC1	-0.205863894	0.061067	0.03851986	0.72024	-0.255202869	0.01993
YIPF1	-0.178114154	0.113472	-0.114750703	0.29995	-0.262558429	0.01992
NOL7	-0.07259881	0.45356	-0.068920082	0.47234	-0.22560882	0.02009
S100A4	-0.392678958	0.156535	-0.535192155	0.05334	-0.644544267	0.0201
SLC25A35	0.451499379	0.43726	0.685739681	0.23002	1.339678606	0.02013
JDP2	0.032799786	0.857358	0.0406794	0.82226	-0.425627532	0.0202
TRIM25	0.133558399	0.336752	0.120178026	0.3869	0.32206092	0.0202
HERC2	-0.204101323	0.240355	0.216314447	0.2119	0.402396332	0.02026
CAMSAP3	-0.115156407	0.524428	-0.331653976	0.06662	0.416793805	0.02027
FAM198B	-0.209643234	0.374209	-0.393031574	0.09538	-0.547914959	0.02028
TERF2IP	-0.030955373	0.793724	0.228329968	0.05008	-0.275728259	0.0203
ACTN4	0.183765066	0.136502	0.067705068	0.58319	0.28627953	0.02033
TUSC3	-0.138840849	0.243899	0.028530913	0.81008	-0.276584213	0.02037
TRIM22	-0.062623384	0.809361	0.152428644	0.55656	0.600510675	0.0204
MLANA	0.109669347	0.743332	-0.220289761	0.50226	0.740084172	0.02043
KPNA6	0.224155043	0.058306	0.028852592	0.80741	0.27424254	0.02047
P11-666G4	0.542587546	0.260699	0.578284241	0.21882	1.066564622	0.02048
PIGQ	0.160363528	0.174951	-0.089840549	0.44951	0.273043775	0.02049
HSPA13	0.037681635	0.828164	-0.112022246	0.51839	-0.403099649	0.02055
P11-530N7	0.644298653	0.37233	0.594168304	0.40185	1.564508435	0.02062
LUC7L2	-0.025799717	0.92728	-0.039939471	0.88732	-0.658986764	0.02065
B4GALNT2	-0.363417427	0.061484	0.001341447	0.9944	0.439693328	0.02071
NTHL1	-0.078414437	0.674541	-0.211095607	0.2519	-0.442541774	0.02071
PTGR2	-0.260513606	0.176473	-0.259271131	0.17355	-0.442030052	0.0207
PTPN7	0.019315577	0.962477	-0.098805536	0.80834	0.889709003	0.0207
RBM28	-0.068690113	0.58658	-0.013769635	0.91277	-0.290943056	0.02076
SETD9	-0.124299384	0.536328	-0.195314987	0.32629	-0.472288726	0.02074
DNHD1	0.051101061	0.813919	-0.049047933	0.82127	0.497700231	0.02084
FAM168A	0.12117662	0.167379	0.129703335	0.13746	0.20170969	0.02084
PER2	0.049499576	0.810691	0.004080352	0.98423	0.476041566	0.02084

PLCB4	0.290044542	0.211536	-0.175999402	0.44851	0.533904579	0.02088
NFAT5	0.333577011	0.108681	0.139845427	0.50097	0.479659053	0.0209
TMEM180	0.105513837	0.650404	0.190972364	0.40207	0.524914553	0.02092
UBE3C	0.003806944	0.976057	0.022078787	0.86175	0.292653512	0.02095
DRAXIN	-0.051532212	0.867071	0.501011777	0.08724	0.675290787	0.02104
GCSHP5	0.053593718	0.902785	-0.641730117	0.14932	-1.053945145	0.02103
TCEA1P2	-0.117192131	0.515965	-0.151674752	0.39725	-0.418618248	0.02104
GABBR2	-2.285861772	0.088298	0.34999943	0.71907	2.193135885	0.02109
PRSS36	-1.069685775	0.057381	-1.062784314	0.06165	-1.31212492	0.0211
SERINC1	0.055525406	0.60073	-0.005474167	0.9588	-0.244960847	0.02111
CBWD3	0.355308483	0.058447	0.168513748	0.36762	0.429465953	0.02116
STARD10	0.175120037	0.233011	0.00297052	0.98386	0.338077942	0.02116
C1QBP	-0.169580638	0.247629	-0.146415018	0.31707	-0.338185339	0.02118
SMOC2	0.076750492	0.757669	0.059556626	0.81063	-0.575137028	0.02121
PRSS23	-0.053318105	0.788599	-0.368574049	0.06342	-0.458131785	0.02122
STK17B	-0.168779171	0.355954	-0.157654756	0.38755	-0.421911483	0.02124
FZD5	-0.041245441	0.851903	0.27314045	0.21577	0.508184277	0.02126
CCDC107	-0.295315882	0.30031	-0.036649855	0.89575	-0.663338892	0.02129
DDX41	-0.031257668	0.761216	-0.030394334	0.76628	-0.237587607	0.02133
G6PC	0.290429194	0.637337	0.925705942	0.10047	1.281959385	0.02136
TMEM45A	-0.012904046	0.941189	0.054678528	0.75431	0.401988454	0.02135
FOPNL	-0.251243818	0.293185	-0.106484303	0.6545	-0.552165172	0.02139
MYH8	-1.062391976	0.333899	0.816201677	0.44808	-2.645650507	0.02147
MNAT1	-0.061686376	0.586935	-0.001949119	0.98614	-0.261362861	0.02151
GGH	-0.116695948	0.491933	-0.108346564	0.52205	-0.390368734	0.02153
CXXC1	-0.231522046	0.092429	-0.185564073	0.17627	-0.316260692	0.02157
ATP6V1E2	-0.346256672	0.251039	-0.166462842	0.55756	-0.68975058	0.02163
RP11-15J10.	-0.164233242	0.617883	-0.076692539	0.81203	0.721256087	0.02165
MYH9	0.099593621	0.449406	0.24203005	0.06593	0.302260796	0.02166
RPL3L	-0.321769309	0.547409	0.22435298	0.65821	1.123573463	0.0217
RAB8B	0.085662483	0.60037	0.197150722	0.22798	-0.375693261	0.0218
RAN	0.04408926	0.679674	0.015718007	0.88286	-0.245053907	0.02181
ARGFXP2	-0.055643063	0.905796	0.342550238	0.43083	0.959120278	0.02187
ASCC3	-0.140611157	0.1499	-0.076542634	0.43207	-0.223870332	0.02187
ARHGAP15	-0.1864763	0.623824	0.555017119	0.13243	0.843446757	0.0219
GALNT1	-0.126468949	0.323719	0.017002252	0.89433	-0.293779026	0.02189
ATP5I	0.05365756	0.599273	-0.187141563	0.06634	-0.234960098	0.02194
GCSAML	0.517655312	0.20071	0.612016454	0.11821	0.887960164	0.02193
INPP5F	-0.0617707	0.631828	0.106943527	0.40486	-0.295760836	0.02198
SRM	0.188657591	0.250852	-0.112360638	0.49393	0.37548007	0.02198
AC009967.3	0.487752543	0.393852	0.571697727	0.30254	1.228875403	0.02204
TFAM	-0.093971503	0.470891	-0.172734973	0.18404	-0.298312304	0.02204
TMEM107	-0.086644347	0.514058	-0.145214522	0.26441	-0.302909416	0.02208
C6orf205	-0.716391107	0.475146	0.896409857	0.28182	1.838997095	0.02218
HKR1	-0.21076104	0.05799	-0.111752509	0.30631	0.250931315	0.0222
C20orf194	0.111846147	0.308053	-0.025605005	0.81419	0.247749749	0.02223
YKT6	-0.069679245	0.294355	0.058985257	0.36947	-0.152045995	0.02225

ANPEP	0.430288966	0.635388	-1.023325264	0.25966	-2.077082397	0.02231
IL1RL1	0.798072711	0.106859	0.366233699	0.46193	1.117360013	0.02234
PPP1R13L	-0.03372372	0.887954	-0.226124467	0.34288	0.539351235	0.02243
BEX1	-0.08357662	0.864805	-0.155005967	0.75122	-1.150730461	0.02244
KRR1	-0.15542767	0.268525	0.070337568	0.61605	-0.320818078	0.02245
SI	-0.666918728	0.492748	-1.864762381	0.05553	-2.227489168	0.02246
IFT122	-0.152042882	0.353478	0.191605456	0.24138	0.372496187	0.0225
ARNT	0.082365063	0.473038	0.058276925	0.60957	0.260802775	0.02252
PGAM1	-0.053972887	0.591362	-0.153764257	0.12584	-0.229403138	0.02254
UFD1L	-0.147330968	0.142154	0.071104655	0.47221	-0.227467479	0.02256
CCDC144CP	0.263078329	0.368521	0.40106668	0.16555	0.65910988	0.02259
ITGBL1	1.225504733	0.285516	0.862631084	0.44849	2.567073199	0.0226
MRPS23	-0.242206233	0.156289	-0.241462245	0.15579	-0.388389417	0.02262
TUBB4B	-0.139083545	0.297458	-0.020506949	0.8778	-0.304250368	0.02267
FOXRED2	0.323964557	0.1379	0.414274314	0.0555	0.494046385	0.0227
GRIK2	0.253018922	0.472327	0.572757715	0.10124	0.796363681	0.02278
IQUB	-0.197443687	0.620597	0.028717584	0.94092	-0.976908846	0.02281
MTND1P10	1.040288672	0.197539	0.974661238	0.22234	1.774270673	0.02281
NBAS	-0.174041529	0.123528	-0.180369537	0.10997	-0.257281782	0.0228
PHKB	-0.179826865	0.069572	0.042742623	0.66517	0.22450441	0.02282
SCAP	0.107253348	0.453326	0.037714898	0.79135	0.32466388	0.02285
NETO2	-0.036964969	0.900775	0.273570659	0.35443	-0.6769814	0.02296
SMIM18	0.847107026	0.186751	0.752048999	0.23381	1.391281824	0.02299
ZSCAN30	-0.162033863	0.384728	0.134258725	0.46643	0.417872776	0.02299
TRIB3	-0.366077929	0.211956	-0.533218785	0.06871	-0.668081173	0.023
CROCCP3	0.187210327	0.597614	0.630945586	0.07182	0.797773204	0.02301
EIF3E	-0.258547191	0.105352	0.139775811	0.38111	-0.362885612	0.02303
ADCY7	-0.044072898	0.768861	-0.034009248	0.82012	0.338203642	0.02307
PKP2	-0.279824681	0.065298	-0.251550674	0.09669	-0.344449053	0.02307
TFCP2L1	0.063082677	0.848826	0.143010482	0.6644	0.748032512	0.02307
RPL10P3	-0.055038651	0.923074	-0.558110274	0.33701	-1.524966248	0.02311
.P3-499B10	0.083666077	0.896615	0.357840036	0.56447	1.359904346	0.02314
VDAC2	0.007212914	0.941677	-0.132514417	0.17831	-0.224093943	0.02322
TUBB3	0.123397756	0.646867	0.302396666	0.25979	-0.616266608	0.02325
MRO	-0.167291252	0.718083	-0.301722004	0.53291	1.022295129	0.02329
MRPS28	-0.06741957	0.653728	-0.213591215	0.15351	-0.342171628	0.02333
ZNF418	-0.125392242	0.609795	0.266571944	0.26685	0.545610637	0.02333
PPM1H	0.126954731	0.540725	-0.150829342	0.46743	0.469250685	0.02345
FAM162B	-1.496635768	0.055795	0.380054629	0.58726	-1.801250706	0.02346
PCDHB1	0.031261066	0.91362	-0.017510108	0.95071	0.623498368	0.0235
CUL2	-0.105501801	0.185137	-0.137670916	0.08028	-0.180084245	0.02353
WT1	-0.592863027	0.390594	-0.027695599	0.96746	-1.598200157	0.02358
DPEP1	0.076079836	0.920224	-0.623156659	0.41798	-2.015803243	0.02363
DTD1	0.003901946	0.97928	-0.133112231	0.3726	-0.342551297	0.02367
CHRNA5	-0.430886506	0.131537	-0.444358308	0.115	-0.641010927	0.02372
FTLP10	1.070476538	0.10682	0.510685576	0.45219	1.447171432	0.02373
MTMR10	0.319113906	0.054955	0.031729511	0.84877	0.374659301	0.02374

PTPMT1	-0.152717492	0.174676	0.013036004	0.90661	-0.253542919	0.02375
SLC25A5	-0.02273278	0.830827	-0.205557725	0.05317	-0.240719323	0.0238
SLC35G1	-0.166654682	0.541308	-0.180994805	0.50627	-0.618846393	0.02382
NDUFA11	-0.008875748	0.943439	-0.154770069	0.21585	-0.283322693	0.02387
GRIK4	-0.765162647	0.508486	-1.045671885	0.3508	-3.082853241	0.02388
TAF9	0.052434603	0.613505	0.037659539	0.71469	-0.236149679	0.02389
ACTL6A	0.027897009	0.860143	0.306920649	0.05154	0.357370554	0.02393
CBL	0.010189801	0.933849	-0.033432182	0.7845	0.275343609	0.02396
UQCR10	0.023636687	0.856196	-0.054780596	0.67274	-0.29628647	0.02397
DCAF12	0.149474703	0.103301	-0.167217821	0.06857	0.206103536	0.02399
TD-2311M2	0.128481413	0.763045	0.350670197	0.40314	0.933039373	0.024
PQBP1	0.177111647	0.099545	0.096786893	0.36331	-0.245552829	0.024
OR5BA1P	0.833152268	0.309908	0.179752249	0.83349	1.699979021	0.02404
PKIB	-0.155748327	0.183026	-0.207422746	0.07522	-0.263797006	0.02408
P11-274B21	0.457727667	0.371718	0.453852435	0.36954	1.119637281	0.02408
ZNF670	-0.086567117	0.566436	-0.057930551	0.69757	0.334416049	0.02408
TMEM178A	-0.436958049	0.15988	0.055560456	0.85155	-0.722181256	0.0241
EIF3M	-0.032842607	0.743383	0.081463828	0.41572	-0.226334084	0.02411
CLCN7	0.070407914	0.623901	0.276526688	0.05177	0.320793048	0.02412
XYLT1	0.266796083	0.381742	0.298676168	0.32595	0.685975158	0.02414
DYNLT1	0.058190532	0.524447	0.104237868	0.24908	-0.206465442	0.02415
GOLGA8B	0.154718526	0.372153	0.114011422	0.51019	0.390173822	0.0242
ADAM32	0.110606118	0.622812	0.224348832	0.31454	0.5017806	0.02421
PPIB	-0.078383067	0.487079	-0.078040357	0.48865	-0.254187858	0.02425
CEP85L	-0.23575482	0.106178	-0.192348608	0.18551	-0.328358912	0.02431
ARPC5	0.108624491	0.280625	0.088678684	0.37685	-0.226888902	0.02433
KDM2A	-0.067285666	0.504989	0.097736051	0.33082	0.226275061	0.02438
KMT2E	-0.027244337	0.845731	-0.013642742	0.92227	0.314171592	0.02441
CACNA1F	-0.453633938	0.341765	-0.079261274	0.8706	1.038947111	0.02445
NAA25	0.078707744	0.539839	0.171072291	0.18052	0.287930368	0.02447
MAPK6PS3	-0.000627347	0.998847	0.273688502	0.51003	0.908695835	0.02451
PPARG	-0.002287489	0.990968	0.176081285	0.38308	0.45367561	0.02453
TRAPPC10	0.156537171	0.203464	0.137552132	0.26081	0.276009574	0.02452
SNX1	0.066119509	0.410737	0.122787807	0.12277	0.179733008	0.02456
DNAJC15	-0.065651895	0.778836	0.257607696	0.26477	-0.526893054	0.0246
GPR183	-0.259293599	0.390493	-0.281773113	0.34674	-0.686468674	0.02461
RGS1	0.151311608	0.810792	0.073358562	0.90765	-1.438682187	0.0246
ASTN1	-0.842370954	0.241389	0.276509414	0.6847	-1.659715011	0.02462
C5orf63	-0.390605355	0.368273	0.05118319	0.90212	0.922177678	0.02465
PCNXL2	-0.118839258	0.561173	-0.080093423	0.6937	0.457645383	0.02466
SLC39A6	-0.129126445	0.468457	0.188570348	0.28831	-0.400277707	0.02465
RARA	-0.269287189	0.090201	-0.092105445	0.5595	0.354011498	0.02467
AC018638.1	1.089400433	0.176277	0.771552739	0.3391	1.725914748	0.02475
B3GALNT1	-0.037606034	0.85968	0.097548511	0.64187	-0.479118001	0.02476
KLHL14	-0.885883349	0.088083	0.803188011	0.11652	-1.167720221	0.02476
ANKRD61	-0.396749468	0.302022	0.074864594	0.83018	0.747125051	0.02482
NAAA	0.012676845	0.970499	-0.251680639	0.45975	-0.783076546	0.0249

P11-567M21	1.351461556	0.13306	1.602563655	0.0676	1.951150094	0.02497
COPE	-0.117343012	0.548357	-0.252892624	0.19436	-0.438151797	0.02503
TPM3	-0.010745681	0.93064	0.173818465	0.15879	-0.276650681	0.02507
CPA6	0.168904739	0.779276	0.519788528	0.36726	1.25180631	0.02517
HSP90AB2F	-0.257196842	0.287039	0.046467991	0.84606	-0.542555525	0.0251
INTS1	0.291313867	0.083864	0.207297822	0.21746	0.376226304	0.02515
LASP1	0.030132211	0.766051	-0.076772393	0.44801	0.22637333	0.02514
LRIG2	-0.146967563	0.291275	0.211457712	0.12575	0.309578113	0.02516
PAK4	0.340967281	0.122096	-0.032512001	0.88297	0.492960679	0.02514
RPS6KL1	0.077681298	0.812298	0.188839457	0.55657	0.711832085	0.02516
SNX22	0.233658393	0.137236	0.161789038	0.29777	0.347358659	0.02518
TMEM14B	-0.143746686	0.152447	-0.061024773	0.53724	-0.223999371	0.02519
WDR90	0.053774736	0.740479	0.007887544	0.96118	0.360576867	0.02517
ZNF169	0.026287777	0.900877	-0.308521215	0.13848	0.454549081	0.02511
CDKN1B	-0.275967489	0.163165	0.246867704	0.20668	-0.441588956	0.02522
CSF3R	0.467353871	0.140151	0.477419229	0.13153	0.7066956	0.02522
GTF3C6	0.006280024	0.953301	0.080374801	0.44841	-0.240951565	0.02522
HLA-DPB1	-0.133352351	0.545935	-0.128309976	0.55478	-0.500688288	0.02528
P11-227P3	-0.204929617	0.767012	0.563615183	0.35436	1.300067298	0.02529
DYX1C1	-0.304981732	0.09526	0.142400881	0.41635	-0.408958501	0.02531
C5AR2	-0.202822589	0.391737	-0.00454584	0.9842	0.504110879	0.02537
OGFOD1	-0.073050635	0.465559	0.110403208	0.2623	-0.223721207	0.02535
LIPC	-0.190864176	0.79886	-0.604144235	0.4213	-1.697873339	0.0255
RP11-54I5.1	0.679036324	0.346646	0.186839061	0.7997	1.494635569	0.02551
PDCL	-0.187110469	0.093683	-0.129468288	0.24099	-0.249097843	0.02553
PLEKHS1	-0.24900121	0.713392	0.418443457	0.52298	1.388081221	0.02553
ITIH2	0.443238663	0.088475	0.500148917	0.05454	0.580735855	0.0256
THOC7	-0.132857064	0.1813	-0.05743374	0.55856	-0.221743674	0.02559
CHRD	0.490904512	0.249545	0.371481053	0.38047	0.930007566	0.02563
GMIP	0.124878835	0.564195	0.354657029	0.09905	0.479438972	0.02563
SLC4A4	0.065405723	0.728987	0.057359694	0.76107	0.420629413	0.02566
QRICH1	-0.00477398	0.946567	0.094286789	0.18265	-0.15916043	0.02567
NPPB	-0.52316411	0.561346	-0.852749991	0.34466	-2.207702434	0.02568
FAM135B	-0.028851785	0.966341	-0.228451941	0.7326	-1.566565662	0.02572
P11-397P13	-0.308292418	0.539983	-0.228425722	0.64064	1.032594637	0.02572
ZBTB20	-0.019098835	0.922516	0.012831346	0.94779	0.433322025	0.02573
FTH1P7	-0.514644731	0.324538	-0.595900515	0.24994	-1.199636787	0.02579
ZNF585B	0.379481008	0.204908	0.345410133	0.24888	0.665935036	0.02579
MAMLD1	0.203987722	0.652616	0.344494071	0.43878	0.983306595	0.02584
C9orf78	-0.208667794	0.076438	0.172998189	0.13537	-0.262972495	0.02588
SLX4	-0.153328388	0.289451	0.134730691	0.34326	0.316641963	0.02588
ZBP1	0.512607586	0.425908	0.641989494	0.31135	1.389528307	0.02587
NRNPA1P6	0.83081988	0.090033	0.872836189	0.0694	1.068407959	0.02591
SNRPE	-0.015700589	0.895911	-0.153891619	0.19807	-0.2680653	0.02593
GDA	-0.116610439	0.792461	-0.524680762	0.23668	-0.99171147	0.02597
GNB2L1	0.048996618	0.665884	-0.212345109	0.06132	-0.252612517	0.02601
MAN2B2	0.183676158	0.136307	0.158354531	0.1963	0.27348086	0.02599

NUDT12	-0.234369473	0.054219	-0.214160872	0.07613	-0.270127356	0.02603
RPS6P20	-0.075479779	0.891236	-0.030940325	0.95384	1.112969846	0.02601
ZNF764	-0.114659529	0.511576	0.024142795	0.88766	0.378023391	0.02601
HKDC1	-0.241756458	0.644571	-0.958733084	0.06767	-1.168827477	0.02608
MUM1	0.048285652	0.733442	0.197629077	0.16075	0.312750845	0.02612
MYOM3	-0.374295146	0.40428	-0.436611717	0.32707	-0.998136634	0.02612
SPDYE8P	-0.001762489	0.994579	0.119368096	0.63053	0.539054698	0.02612
SMIM13	0.004838384	0.97361	-0.089372414	0.53962	-0.326162767	0.02614
PAPL	0.285213032	0.414267	0.526445778	0.10864	0.726849137	0.02615
GBA3	-0.443114881	0.668997	-0.943973833	0.36307	-2.346375388	0.02618
HIST1H2AH	-0.192612466	0.334613	-0.110822799	0.57824	-0.44413549	0.02619
COL7A1	-0.13969139	0.642805	-0.374130788	0.21539	-0.67126351	0.02622
SIN3B	0.186220227	0.165501	0.130080378	0.33181	0.297495443	0.02626
TAS2R4	-0.16561121	0.674985	0.226686096	0.54306	0.806488849	0.02628
P11-345I18	-0.107043185	0.906215	1.434936158	0.05896	1.679710558	0.0263
BTAF1	-0.043981235	0.644089	-0.081025983	0.39382	-0.211540756	0.02631
ATP6V1G1	0.050435347	0.560413	0.012028543	0.88916	-0.192769058	0.02635
CIDEB	-0.210150127	0.780177	-0.505374445	0.50212	-1.686131497	0.02637
MRPL40	-0.122141459	0.334139	0.049873407	0.68612	-0.281236791	0.02639
PRKAG1	-0.109507132	0.181056	0.080311499	0.31826	-0.181697289	0.02639
NCOR2	0.036661268	0.872667	-0.014617903	0.949	0.505702737	0.02641
KIF6	0.244674933	0.56993	-0.094167255	0.82525	0.938481713	0.02643
HSPA8	0.069290398	0.637319	0.184987147	0.2081	-0.326178105	0.02648
RPL21P8	0.497039871	0.446977	0.856284248	0.17017	1.359820142	0.02649
SRSF1	-0.033204236	0.776276	0.096815537	0.40694	-0.259341739	0.02649
RPL36AP15	-0.274287293	0.267369	-0.359941764	0.14218	-0.552571238	0.0265
FABP4	0.434803406	0.471174	-0.168780065	0.78679	1.287710978	0.02655
ARMC4	0.888765485	0.134274	0.986712408	0.09679	1.317356018	0.02665
BATF2	-0.020460154	0.964813	-0.584938842	0.21967	-1.097005439	0.02664
C19orf54	0.029910783	0.925367	0.144501073	0.64958	0.696904632	0.02664
ELMSAN1	-0.136160335	0.36484	0.219979155	0.14055	0.330963647	0.02665
PABPC1	0.024581171	0.89952	-0.026626884	0.8912	0.431428837	0.02664
LSMEM2	0.53241927	0.345847	0.402823856	0.47029	1.189483569	0.0267
WDR62	-0.085779598	0.709734	0.359570015	0.11538	0.505104399	0.02673
NPY2R	0.339511814	0.636186	-1.01967599	0.16907	-1.717986237	0.02674
INSR	0.02747457	0.856003	0.153653303	0.30895	0.334348349	0.02679
ZNF618	-0.077337601	0.800022	-0.105124937	0.73094	0.672537635	0.02679
MPDZ	-0.048597748	0.761516	0.004998488	0.97507	-0.354653475	0.02681
PLA2G4A	-0.017757077	0.922531	-0.318440747	0.08151	-0.405937266	0.02685
MLPH	-0.061156266	0.817918	0.201372175	0.44743	0.586189252	0.0269
MTND1P32	0.321173333	0.705574	0.804120557	0.31326	1.695363816	0.02692
CYP2C19	0.175916804	0.467928	0.198119394	0.4124	0.534074374	0.02694
TIAF1	-0.184445835	0.599182	0.169407829	0.61807	0.740257406	0.02694
NUBP1	-0.090755641	0.474199	-0.054404208	0.66545	-0.282041886	0.02695
ATP6V0E2	-0.007354966	0.971936	0.062017243	0.76441	-0.469860856	0.02702
NF1	-0.021275915	0.909791	-0.023924806	0.89854	0.414731253	0.02702
RBM22	0.11345525	0.167557	-0.145511705	0.07593	-0.182778793	0.027

HAVCR2	0.105337603	0.775872	0.479144112	0.17219	0.774152779	0.02706
RHOBTB3	0.038867698	0.79341	0.226605195	0.12609	-0.328308452	0.02707
SHMT2	-0.016472626	0.914972	-0.015904075	0.91782	-0.341392615	0.0271
PPM1L	0.095440272	0.48092	0.119828996	0.37389	0.297610641	0.02713
ATXN1	0.050397524	0.727677	0.171647313	0.23524	0.318538657	0.02717
C2orf80	0.8413226	0.27846	1.158655127	0.1198	1.605056265	0.02725
TSLP	0.341643054	0.741269	-0.764868846	0.46354	-2.444601359	0.02726
L1TD1	-0.651617366	0.130971	-0.066091735	0.87769	-0.953688689	0.02728
PIGB	0.141643259	0.240734	0.047791864	0.68546	0.263220289	0.02734
SLC22A25	0.090064647	0.824751	-0.474895078	0.25512	0.845699938	0.02741
NFRKB	-0.007721209	0.935928	0.011377948	0.90476	0.210014057	0.02747
HIST1H3J	-0.16494826	0.480721	-0.089984459	0.69998	-0.516251573	0.02754
PARP1	-0.154681348	0.154488	0.198124336	0.06729	-0.239496545	0.02754
DCUN1D1	-0.15837079	0.221067	0.170358421	0.18698	-0.285000414	0.02755
AC073551.1	0.756262049	0.173342	0.275225877	0.62604	1.174646879	0.02758
ATIC	-0.196266772	0.45221	-0.165826592	0.52437	0.571081469	0.02759
KIAA2018	-0.083515769	0.581033	-0.151652936	0.31502	0.331572921	0.0276
PROSER2	0.306016259	0.202582	0.301546085	0.20624	0.525077903	0.02761
HEATR2	0.108566508	0.425087	0.082001054	0.54419	0.29723001	0.02764
SDS	0.353864245	0.428507	0.566157455	0.18997	0.942270949	0.02765
DNM3	0.599022491	0.13286	0.560904862	0.15819	0.872478359	0.02767
POLR2M	-0.139424141	0.401952	-0.114849127	0.48905	-0.366005897	0.02777
S1PR5	1.217400281	0.147721	1.346921047	0.10512	1.816792857	0.02779
ARHGAP28	-0.304803629	0.330971	0.248342507	0.42758	-0.690306114	0.02782
NDUFA4L2	-0.088908398	0.839649	-0.609917928	0.16591	-0.971919373	0.02782
POMZP3	-0.355616366	0.242564	-0.046555715	0.87354	0.625824947	0.02783
SKAP1	0.359986118	0.673259	-1.173679936	0.2055	-2.370325486	0.02785
ZNF268	-0.182143457	0.127221	-0.196882567	0.09801	-0.2619126	0.02785
IL10	0.496927361	0.317048	0.805409887	0.0901	1.036984198	0.0279
SEMA5A	-0.062655279	0.845948	0.019314199	0.95215	-0.711223162	0.02791
VPS37A	-0.102896869	0.246183	0.036864202	0.67425	-0.193970538	0.02799
MTFP1	0.178224066	0.391835	-0.042710948	0.83672	-0.464835867	0.02804
FAM166A	-0.521213338	0.267599	-0.37665551	0.41431	-1.090023951	0.02817
HIST1H1B	-0.291956057	0.230055	-0.090361184	0.71008	-0.533946072	0.0282
SEPP1	-0.310484175	0.49941	-0.387284614	0.39949	-1.009169225	0.02822
HSBP1	0.006225025	0.936683	-0.046778662	0.54809	-0.171920024	0.02835
IVD	-0.033585694	0.860371	-0.262912624	0.16757	0.415940258	0.02835
LRSAM1	0.130407323	0.411467	0.001212263	0.99386	0.342827176	0.02834
RP11-332O19	0.380205589	0.625654	0.680748352	0.36065	1.569804096	0.0284
SMLR1	-0.129941982	0.807469	-0.807779032	0.13839	-1.247866887	0.02848
C1orf74	-0.312419209	0.081223	-0.111140817	0.52152	-0.390735566	0.02854
DRG1	-0.186731213	0.051059	-0.095984907	0.31338	-0.209672661	0.0286
C9orf131	0.146922827	0.709425	0.215164987	0.58221	0.850552869	0.02862
MAP3K7	-0.111040159	0.175082	0.133587397	0.09898	-0.179038398	0.02869
SYNGAP1	0.308057772	0.379097	-0.025809926	0.94146	0.760301318	0.02876
TRAFD1	-0.037027902	0.743356	-0.099874722	0.3742	-0.24809159	0.0288
BRAP	-0.176982727	0.054045	0.111706787	0.21162	-0.199279472	0.02886

CCDC144B	0.326870458	0.716352	1.499227528	0.07069	1.800191993	0.02887
P11-369G6	0.523221802	0.358489	0.317029829	0.57556	1.185334728	0.02889
AIF1	-0.577166001	0.144429	-0.182640232	0.62271	-0.870912269	0.02895
GBF1	0.020731174	0.845155	-0.154240877	0.14556	0.230912731	0.02902
P11-474L11	0.323383119	0.379753	0.345201686	0.33366	0.766322483	0.02902
TC-325H20	-0.191815588	0.786624	0.371497706	0.56846	1.350011263	0.02906
SATB1	-0.049496331	0.791576	0.118746676	0.52533	0.40778426	0.02906
TEK	0.001299715	0.996197	0.064079768	0.8139	-0.597671376	0.02909
MRPL49	-0.114822871	0.122628	-0.124151801	0.09062	-0.161587913	0.02917
HSP90B1	-0.047080341	0.704652	-0.179745662	0.14784	-0.270868415	0.02921
TBC1D27	-0.248293255	0.779984	0.951797679	0.20794	1.600678644	0.02927
BPIFB1	0.311552153	0.632933	0.735379326	0.25917	1.41971088	0.0293
PC	-0.142232896	0.589041	-0.092362066	0.7244	-0.575534318	0.0293
UTS2B	0.515836293	0.087925	0.193614486	0.51644	0.642225403	0.02932
CYP4F62P	0.774090081	0.352055	0.644185026	0.43226	1.746087719	0.02944
MED31	-0.114168947	0.470281	-0.259220113	0.10414	-0.345002357	0.02944
P11-444E17	0.301451699	0.490125	0.471729704	0.26224	0.899847844	0.02944
PPP1R12B	-0.009548337	0.94443	-0.066308565	0.628	0.296706152	0.02949
NEK10	0.143360063	0.705997	0.103980895	0.77973	0.79771676	0.02951
ZYG11B	-0.14291551	0.096016	-0.097415682	0.25341	-0.186322159	0.02953
C6orf141	0.365833613	0.07106	0.132815133	0.51219	0.439524821	0.02961
CYP4V2	0.168129788	0.495551	0.342572088	0.15992	0.530205099	0.02959
FAM186B	0.145199531	0.800118	0.504024787	0.3678	1.206589653	0.02959
FYTTD1	-0.247466333	0.053666	0.106968186	0.40218	-0.278750753	0.02959
PDIA3	-0.183664732	0.118288	0.003102781	0.97894	-0.255771481	0.0296
ETFDH	-0.186089729	0.153242	0.039877297	0.75771	-0.283075828	0.02968
AP2M1	0.025182011	0.752136	-0.093323518	0.24106	-0.173587319	0.02969
ZNF736	0.165290833	0.806988	0.663431396	0.30273	1.365939957	0.02974
WNT9B	0.494322945	0.314757	0.276879103	0.56893	1.005853996	0.02976
MCCC2	0.166093947	0.185784	0.163853655	0.18707	0.270606728	0.02978
SDC3	0.278301579	0.142886	0.046503758	0.80618	-0.414806926	0.02981
NLRP3	-0.585548465	0.407084	0.496629269	0.41145	1.293881441	0.02985
P11-385D13	0.642626644	0.240019	0.578122115	0.28545	1.156097091	0.02984
CENPJ	-0.262786606	0.056428	-0.209050335	0.12681	-0.299286786	0.02987
DLAT	-0.188139028	0.072921	-0.118273488	0.25613	-0.227358331	0.02988
ZC3H12B	0.226493863	0.129004	-0.051062305	0.73192	0.318577018	0.02991
HMG20B	-0.163871112	0.171022	-0.138281469	0.24652	-0.260010817	0.02996
PLEKHG5	0.015220241	0.921291	-0.2268532	0.14005	-0.336113761	0.02998
SMIM4	-0.531968119	0.050317	-0.500748934	0.05992	-0.572137139	0.03003
TGFB1	0.402218654	0.075021	-0.166638285	0.46247	0.489910557	0.03002
BEX5	-0.248132951	0.473506	-0.52657918	0.12793	-0.776183743	0.03004
HOXD9	0.601632583	0.415706	1.114103568	0.12699	1.578248895	0.03006
NHLRC2	-0.018796709	0.857682	0.035961302	0.73031	0.225877215	0.03019
CCDC120	-0.068936973	0.754314	-0.079268006	0.71875	0.470716046	0.03026
ELOF1	-0.003735438	0.973902	0.013970722	0.90161	-0.249313166	0.03026
SETD1A	0.252232326	0.308279	0.07506182	0.76069	0.533187668	0.03027
TANC2	0.239386955	0.284773	0.23603733	0.29088	0.483561955	0.03028

FYB	0.864072554	0.185614	-0.524436824	0.40573	1.293416847	0.03033
LAPTM5	-0.485583411	0.5026	-0.355381951	0.61942	-1.666672922	0.03034
WDR4	0.29476223	0.138394	-0.008810648	0.96451	0.4248033	0.03047
ZSWIM7	-0.220052858	0.142406	0.136975477	0.34256	-0.318707668	0.0305
CLSTN1	0.035864065	0.656107	-0.046978076	0.55776	0.173006939	0.03051
SBNO1	-0.109452012	0.123468	-0.089452122	0.20594	-0.153318658	0.03054
C11orf57	-0.222282078	0.117958	0.143346618	0.31147	-0.307485009	0.03056
CEP164P1	0.723729748	0.057847	0.282701904	0.46125	0.815236669	0.03062
CCDC82	-0.158973232	0.194659	0.187763376	0.12038	-0.264361082	0.03066
TNNT1	-0.259479202	0.372485	-0.343184475	0.23777	-0.630395443	0.0307
ZNF891	-0.258881106	0.096676	0.03819022	0.80319	0.330057109	0.0307
ADIPOQ	0.027982434	0.944567	0.151418774	0.69676	0.812414759	0.03076
P11-85F14.	0.455498647	0.625888	0.293868218	0.75032	1.836574328	0.03077
SRRM1P3	-0.140626271	0.863068	-0.277854056	0.7276	1.491210158	0.03077
PIP5K1B	0.277277546	0.418728	0.554925102	0.10354	0.736285623	0.03082
FAM110A	0.000989219	0.996554	-0.260707507	0.25254	0.471015068	0.03083
HOXC5	-0.155721731	0.723656	-0.542771009	0.221	0.896719627	0.03093
NRD1	-0.063797432	0.662658	0.06671068	0.64685	0.313799359	0.03092
DNAJC25	-0.246860794	0.104893	-0.179726424	0.23238	-0.327666418	0.03103
SLC4A5	-0.221232384	0.611871	0.401325874	0.34908	0.920167426	0.03106
TNK2	-0.081447268	0.657025	-0.082865167	0.6506	0.392262886	0.03109
MRPL47	0.024593788	0.848201	5.79E-05	0.99964	-0.27787489	0.03119
PIK3C3	0.009205962	0.940008	-0.08438467	0.4876	-0.26295557	0.03124
TCEAL3	-0.22183396	0.234318	-0.007881631	0.96562	-0.402705253	0.03127
PKP1	-2.396843416	0.077647	1.522996215	0.21717	-3.013736275	0.0313
P11-58H15	-0.186520297	0.648272	0.386471261	0.30223	0.791678008	0.0313
CRAT	-0.203769534	0.213071	-0.179983814	0.26907	-0.351556955	0.03136
PLXNB2	0.239890344	0.145427	0.195945072	0.23429	0.354539229	0.03135
P11-711M9	0.831767433	0.224411	-0.539485018	0.48019	1.408362778	0.03136
SAG	0.320217673	0.362212	0.591401253	0.08554	0.731804184	0.03133
STRC	-0.349582217	0.436379	-0.196356296	0.65978	0.935795092	0.03137
TRIM4	-0.104246537	0.746756	0.46679655	0.12825	0.659403902	0.03136
ALG11	-0.015181811	0.897472	-0.163271694	0.1622	-0.252609077	0.03139
CDADC1	-0.392555781	0.154572	-0.437932555	0.11198	-0.589837489	0.0314
LARP4B	0.165471113	0.168395	-0.015779814	0.89537	0.257824489	0.03143
LYAR	-0.02283717	0.903517	-0.065194405	0.72869	-0.406553114	0.03146
IMPA2	0.032941579	0.841441	-0.140834861	0.39137	0.352737856	0.03151
TPT1	-0.054538099	0.610746	-0.092730915	0.38673	-0.230429348	0.03151
HTRA4	0.122012381	0.649862	0.16537883	0.52824	0.555497314	0.03153
SDC1	-0.335984195	0.121038	0.03471901	0.87231	-0.466046251	0.03157
ZNF460	0.214499497	0.122149	-0.085147037	0.53931	0.297448003	0.03158
ERMAP	-0.216355092	0.096853	-0.045141087	0.72341	-0.279204482	0.0316
CIAO1	-0.145801088	0.057132	-0.141650545	0.06089	-0.16336852	0.03166
KRT8P36	0.489485818	0.093749	-0.126311226	0.67388	0.615834994	0.03167
NIFK	-0.047001586	0.762565	-0.05569206	0.72001	-0.334523745	0.03167
P11-78H18	-0.656145532	0.102035	-0.632317626	0.10289	-0.87005376	0.03166
SMIM6	-0.242039081	0.314117	-0.2704127	0.258	-0.518831171	0.03165

POLR2D	-0.117268388	0.336422	-0.179459566	0.13964	-0.261500945	0.0317
AHCY	-0.076794205	0.549804	-0.224800168	0.07979	-0.27591505	0.03172
GNE	0.15986585	0.411265	0.030682303	0.87449	0.416658461	0.03172
SLC35G2	0.571648167	0.128903	0.337082658	0.36703	0.781125198	0.03176
PDPK2	-0.260809889	0.3157	-0.169786406	0.50632	0.538006107	0.03178
GALR1	0.094966923	0.748325	0.508057622	0.074	0.611956794	0.0318
MTAP	-0.180525255	0.09395	-0.189853712	0.0763	-0.230582352	0.03181
SLC25A14P	0.296740121	0.653002	0.688118641	0.26571	1.286494286	0.03182
CCDC127	-0.262165125	0.116825	-0.125748282	0.44719	0.354737076	0.03185
CA5A	0.002020607	0.993762	0.077157893	0.76194	0.540422687	0.03203
ODF2	-0.055897548	0.572546	0.034983654	0.72129	-0.212688098	0.03204
CD209	-0.317208208	0.483146	0.052870486	0.9011	0.852935405	0.03209
AC141586.5	-0.001187204	0.995044	-0.190766968	0.31559	0.400135666	0.03214
DNTTIP2	0.056632917	0.667977	-0.054017804	0.68209	-0.282979499	0.03214
CAMK2N1	-0.329053213	0.256095	-0.39054334	0.17715	-0.621429928	0.03225
HTR1D	0.457528417	0.268699	0.446443683	0.26992	0.851928309	0.03225
PSMD6	-0.037328596	0.677494	0.054966969	0.5364	-0.191615267	0.03223
HUWE1	-0.096223274	0.492266	0.272146565	0.05132	0.299069076	0.03227
RNGTT	-0.136132599	0.263217	0.16788537	0.15889	-0.260986248	0.03236
P11-252I14	0.019421455	0.980936	0.610387498	0.4068	1.492228319	0.03235
DEGS2	0.456973118	0.227431	-0.443900273	0.24168	0.804243278	0.03238
P11-108K14	0.389196154	0.136626	0.083681708	0.74975	0.551579859	0.03246
APLN	0.000383686	0.999265	0.165332174	0.67697	-1.004202807	0.03253
DNAJB9	-0.202996847	0.473948	-0.1557852	0.58295	-0.606688242	0.03252
FAM171A2	0.061921478	0.826913	0.01077439	0.96954	-0.610458498	0.03251
TRAPPC9	-0.024075658	0.918614	0.210529671	0.36918	0.499575215	0.03253
C19orf18	-0.377789719	0.335782	-0.560991294	0.14739	-0.880286801	0.03256
PSMC6	-0.121628969	0.23721	0.039795856	0.69744	-0.219780278	0.03259
YBX1	0.02225096	0.849383	0.018978533	0.87127	-0.250483311	0.03258
MAB21L3	-0.13200987	0.589137	0.310885729	0.20085	0.519151723	0.03261
C1orf194	1.391911335	0.061835	0.883187067	0.26346	1.561626825	0.03263
T6GALNAC	-0.17418344	0.235338	-0.081694224	0.57623	-0.313922973	0.03272
RPS6KA3	0.124151145	0.203029	0.122186645	0.20948	0.208059098	0.03274
SPATA2P1	0.682206381	0.207331	0.693170678	0.18882	1.105856491	0.03275
APOA4	0.752778783	0.510412	-1.008674064	0.37791	-2.442559542	0.03286
FKBP2	-0.056562543	0.660558	-0.015569734	0.9032	-0.275678421	0.03284
LRRC2	-0.527119148	0.170535	0.315475415	0.38457	0.770064726	0.03283
RALGAPA1	0.098728011	0.359463	-0.040676303	0.70469	0.229404246	0.03284
RBM22P2	0.575020264	0.221744	0.392652522	0.40144	0.978868807	0.03283
TNPO1	-0.153206187	0.12875	0.075718931	0.45211	-0.21520004	0.03282
FMO3	0.548250303	0.300259	0.504551029	0.32861	1.081079328	0.03291
TTC38	-0.263573788	0.103425	-0.26257699	0.10433	-0.346122785	0.03291
SH3GL2	-0.161476817	0.761788	0.242850231	0.63824	-1.213083099	0.03296
SLC25A3	0.046895267	0.676466	-0.189295283	0.09201	-0.23979024	0.03296
RALA	-0.128200287	0.267578	0.003704983	0.97427	-0.246394221	0.03301
KRTAP9-6	0.011212571	0.987514	0.644432374	0.3394	1.401770111	0.03307
BTBD18	-0.068356105	0.840296	0.106472347	0.7463	0.684366499	0.03308

CPLX1	-0.052822346	0.930171	0.773106488	0.16067	1.170496862	0.03316
EGF	-0.07473815	0.901934	-0.692829345	0.25009	-1.412708106	0.03319
FAM3C	-0.10852917	0.363219	-0.047663495	0.68835	-0.254265879	0.03315
FKBP1B	0.338373567	0.06203	0.021996244	0.90364	0.381001728	0.03319
LRP10	0.147015316	0.233023	0.083267339	0.49896	0.262271349	0.03318
TVP23C	0.355872887	0.074417	-0.016681344	0.9333	0.422238437	0.03321
VPS72	-0.14091789	0.242387	-0.076044559	0.52432	-0.256511573	0.03321
P11-597D13	-0.219293381	0.673935	-0.181116954	0.72165	1.04886147	0.03335
TDO2	-0.577158061	0.333534	-0.055203925	0.9261	-1.275158205	0.03334
AHNAK	-0.04077036	0.778733	0.080389636	0.57952	0.308639424	0.03339
NCS1	0.044941756	0.812576	0.087011141	0.64405	-0.406402661	0.03345
SCN8A	0.445324843	0.298239	0.455960281	0.28309	0.902729853	0.03344
TMEM213	-0.212740173	0.603092	-0.058854739	0.87922	0.813106219	0.03352
GUSBP5	0.622901215	0.086388	0.40044847	0.26238	0.760365175	0.03357
MROH7	0.238347561	0.508664	-0.465490465	0.19992	0.755040442	0.03361
UQCRC1	0.137826456	0.310855	-0.246608129	0.0699	-0.289327464	0.03361
DHPS	-0.043505483	0.715166	-0.199962329	0.09299	-0.254181732	0.03365
LRRC37BP1	-0.245999918	0.073131	-0.059212653	0.65897	-0.287600062	0.03379
TMEM71	-0.956056241	0.378696	0.944798679	0.36624	-2.521206992	0.03387
TEX10	-0.098969675	0.41911	0.176423654	0.14683	-0.259658753	0.0339
ATP5J	2.65E-05	0.999775	0.030102202	0.74613	-0.199760208	0.03397
P11-64D22	0.486807242	0.565152	0.837589848	0.3043	1.682333268	0.03397
PELP1	0.310236552	0.080587	0.105091469	0.55281	0.375978008	0.03406
CALR	0.003375152	0.975527	-0.052390516	0.63387	-0.233129266	0.03413
NEIL1	0.018264686	0.925037	-0.213159758	0.27034	0.404963478	0.03419
PRICKLE4	0.241767622	0.182473	0.245549037	0.17284	0.380368739	0.03421
AC005154.7	0.077333087	0.865682	0.212647447	0.62695	0.888662998	0.03423
CPED1	0.020312654	0.934891	0.237455778	0.33864	-0.527484235	0.03422
TTC25	-0.927012706	0.275746	-0.554344228	0.52295	-1.921093207	0.03427
VIPR1	-0.545755869	0.313601	-0.8833593	0.10329	-1.154947768	0.0343
C19orf47	0.32052881	0.069171	0.266326272	0.12885	0.371294923	0.03438
ACE2	-0.236098163	0.682645	-0.339419523	0.55663	-1.222350999	0.03442
CNTN2	0.940791102	0.305942	0.62804333	0.48408	1.916825585	0.03443
SPDYE1	-0.430539987	0.202602	0.202868341	0.53016	0.676093872	0.03448
SLC45A3	-0.127746994	0.586318	0.016009131	0.94522	0.489979535	0.03452
CLDN3	-0.768841083	0.12068	-0.27301846	0.56439	-1.059315459	0.03457
HYAL4	0.104398483	0.842895	0.240266071	0.64374	1.073147458	0.03458
SH3BP1	0.086052046	0.589024	-0.029520119	0.85318	0.335358072	0.03462
INVS	0.078663449	0.620397	-0.026070037	0.86884	0.331901979	0.03464
LRRD1	0.525929171	0.231504	0.419722796	0.33311	0.896026245	0.03467
PLCD3	0.185844432	0.40425	0.07238838	0.74466	0.468016519	0.03466
SPIN1	-0.121298749	0.268337	0.156579157	0.15161	-0.231427803	0.03466
COL6A1	0.328801296	0.087257	0.24985421	0.19372	0.405893197	0.03475
TOMM5	-0.002383915	0.983374	-0.102983696	0.36686	-0.241848732	0.03479
B3GALT4	0.111766741	0.670646	0.127015836	0.626	0.544439385	0.03485
CDHR2	0.00768565	0.992326	-0.703445619	0.37936	-1.696876049	0.03491
CXorf38	-0.154099249	0.1483	-0.196231502	0.06394	0.219113428	0.03495

NDUFAF1	-0.088497671	0.555558	0.202678704	0.15769	-0.311876405	0.03505
ABCA1	0.008570864	0.969969	0.217410828	0.33829	-0.481310928	0.03507
RPL10AP6	-1.40968847	0.094314	-1.230079673	0.13055	-1.820286965	0.03515
PNPLA1	-0.194177875	0.732031	0.690132883	0.17403	1.055167151	0.03516
LPCAT2	-0.408709192	0.250766	0.282211398	0.42398	-0.749699195	0.03517
GLIPR1L2	0.078657681	0.775213	0.383451981	0.15324	0.564947062	0.0352
TTLL5	-0.226290963	0.105742	0.210748293	0.12888	0.292959385	0.03521
EIF4BP5	0.936781471	0.320534	1.114202086	0.2249	1.88367922	0.03525
HOMER3	0.130736167	0.590863	0.381614047	0.11227	-0.517717055	0.03524
TIMM8B	0.046618696	0.673411	0.006680102	0.95136	-0.234646635	0.03526
NXNL2	-0.189231729	0.508714	0.319920441	0.23067	0.5592452	0.03533
P11-631M6	0.435996751	0.279953	0.699731227	0.07225	0.820189254	0.03547
C4BPB	0.350239673	0.213495	0.348598766	0.2082	0.579666584	0.03557
FGF9	-0.501947708	0.357064	0.151945503	0.77953	-1.161206565	0.03562
AC008565.1	0.112335434	0.767096	0.468228132	0.19976	0.762044293	0.03568
TRAK1	0.279110812	0.053179	-0.100402711	0.4864	0.302754328	0.03566
TTC34	0.360444313	0.497501	0.029782373	0.95541	1.076741189	0.03567
TM4SF20	-0.008903039	0.990285	-0.924620955	0.20815	-1.557117333	0.0357
RNF217	0.436683376	0.055389	0.337790564	0.13622	0.477685829	0.03585
TRA2B	0.093247689	0.380321	0.085754221	0.41915	-0.223102716	0.03586
NRG4	-0.023707719	0.931641	0.087245698	0.74871	0.564911491	0.03588
P11-804M7	-0.472571349	0.430571	0.388459222	0.46954	1.090524897	0.03591
C4BPA	0.440138794	0.494345	0.257788196	0.69289	-1.487827146	0.03606
SPTY2D1	-0.166872135	0.36404	-0.243957075	0.18468	-0.385578007	0.03607
FGB	-0.39985719	0.599114	-0.704143264	0.3547	-1.597436859	0.0361
LSMD1	-0.144794255	0.196105	-0.098117058	0.37406	-0.234257517	0.03614
CD96	0.658544085	0.208807	-0.219184712	0.67745	1.092326079	0.03618
RSL1D1	-0.099032654	0.445195	-0.205364061	0.11326	-0.27174079	0.03622
LIPE	-0.011448689	0.966352	-0.034255014	0.89797	0.551249358	0.03637
SNRPA	-0.129617031	0.386109	0.108404815	0.46608	-0.313037785	0.03638
EIF2D	-0.036597504	0.71669	-0.107399016	0.28643	-0.211292552	0.03643
MYL1	-0.802235803	0.747639	0.947003771	0.70184	-5.583739858	0.03653
CUL3	-0.027601241	0.757352	-0.007563349	0.93214	0.186034792	0.03654
ZNF280C	-0.283512081	0.108804	0.293219679	0.09095	-0.368824748	0.03655
P11-1012A1	-0.263346643	0.435935	-0.383005767	0.25608	-0.709777325	0.0366
COPS3	-0.107024034	0.364411	0.024697242	0.83362	-0.246752018	0.03663
KIF15	-0.299108064	0.106601	0.262838103	0.1484	-0.3858302	0.03668
CENPCP1	0.252695618	0.753187	0.200619777	0.80013	1.576632425	0.03669
PCK1	0.335104771	0.623755	-0.464177983	0.49646	-1.460149475	0.0368
DTX2P1	0.260833897	0.334471	-0.258228436	0.34541	0.553767257	0.03681
ATP6V1E1	0.084141945	0.384522	0.06160997	0.52222	-0.202658126	0.03683
MUC3A	-0.185755395	0.766477	0.087834648	0.88803	1.290883917	0.03688
S100A8	-2.664378111	0.084558	0.694873986	0.6159	-3.323387334	0.03687
LRGUK	0.772551507	0.178725	0.788606059	0.15064	1.12981096	0.03701
CARD16	0.093132399	0.731724	-0.077969602	0.77295	-0.582448682	0.03708
SGK494	-0.162277594	0.498396	-0.267531599	0.25805	0.485614884	0.03708
CEMP1	-0.390330324	0.740076	-1.006283055	0.39664	-2.562429154	0.03711

DNASE1	-0.122357251	0.721558	-0.384210793	0.26249	-0.71523726	0.0371
KHSRP	0.102706743	0.529828	-0.210560371	0.19708	0.340120364	0.03719
BACE2	0.257095711	0.216486	0.129938001	0.53186	0.432836874	0.03724
PNPLA7	0.187324406	0.674211	0.541604891	0.22455	0.891751194	0.03726
RGS17	-0.127162102	0.648085	-0.024941883	0.928	-0.58372346	0.03727
C16orf13	0.082489094	0.59173	-0.252593155	0.10146	-0.321664972	0.0374
ZNF562	-0.039542883	0.809951	-0.048830375	0.76363	0.338356502	0.0374
P11-153M7	0.055063014	0.863058	-0.075868642	0.81162	0.656541748	0.03742
MDN1	-0.054186642	0.765915	0.168297153	0.35416	0.378279782	0.03745
TAS2R30	-0.52251434	0.051487	0.249962666	0.32142	0.521998991	0.03745
CHRM2	-0.008680217	0.973655	-0.142806564	0.58659	-0.547742065	0.03749
TFF2	0.643670987	0.07049	0.499258029	0.16063	0.740188786	0.03753
NUTM2B	0.035895765	0.927375	0.026818269	0.94532	0.803007979	0.03764
P11-368J21	1.116281964	0.10279	0.353645032	0.61437	1.398368722	0.03765
PAIP1	0.055846808	0.563678	0.082411511	0.39219	-0.201104092	0.0377
BTF3L4P1	0.200407026	0.798401	1.08709951	0.13447	1.492892735	0.03774
CREB3L2	0.141138448	0.478497	-0.118351925	0.55237	0.41280189	0.03772
EIF1AY	-0.558921893	0.306106	0.655116278	0.16675	0.97485545	0.03774
HIST1H2BJ	-0.136044586	0.48613	0.166857805	0.39249	-0.405630427	0.03791
ARL10	-0.055120414	0.866185	0.303811166	0.3503	0.673286068	0.03797
EBF4	0.340632475	0.100029	0.174008881	0.40196	0.429468581	0.03801
NCOA1	0.063956252	0.558647	0.13831135	0.2042	0.226210882	0.03805
CDK5	-0.17783275	0.382725	-0.346560455	0.08622	-0.424277093	0.03811
IGSF1	0.428243545	0.160544	-0.030554507	0.92029	0.632169225	0.0381
SLC26A3	-0.114107364	0.89932	-1.080780789	0.23203	-1.890070884	0.03812
SOD3	0.199173772	0.662023	-0.890054886	0.0537	-0.961120204	0.03813
ZNF22	-0.173906778	0.167845	-0.175608687	0.1614	-0.261196338	0.03816
ATP6V1C2	-0.250472919	0.522491	0.306339034	0.42289	0.786824728	0.03817
OR13K1P	1.280098018	0.1139	1.490010857	0.06048	1.645141503	0.03818
P11-121L10	-0.338987265	0.560709	-0.707750742	0.22819	-1.352391942	0.03822
HIST1H4J	-0.339293768	0.215676	-0.079460434	0.77166	-0.56781709	0.03831
CD4	-0.293802718	0.337539	-0.509310737	0.09605	-0.659230367	0.03836
P11-456P18	0.130154675	0.856584	0.367118942	0.58443	1.322081897	0.0384
PRR23C	0.048004785	0.942121	0.91036274	0.13487	1.249284929	0.03843
STMN1	-0.164843825	0.395749	0.370984188	0.05564	-0.401867836	0.03846
PI4KA	0.122121687	0.426726	-0.00817916	0.95746	0.317248834	0.03851
RASSF10	-0.128049922	0.612295	-0.398435274	0.11475	0.515433286	0.03851
P11-225N10	-0.107937441	0.841496	0.559378577	0.25671	1.001250534	0.03851
FAM50A	0.09450571	0.394097	-0.156761758	0.15827	-0.231887911	0.03855
KRBA2	-0.258258273	0.218345	-0.063119749	0.7551	-0.436162484	0.03857
ELMO1	-0.078744949	0.840644	-0.655318772	0.0954	-0.815361518	0.0386
ACP1	0.017931517	0.807028	-0.065845725	0.36706	-0.151803928	0.03867
AOX2P	0.494145718	0.458903	0.346112812	0.60012	1.296513756	0.03867
AP3B2	0.119210833	0.732897	-0.201280847	0.55779	0.703464893	0.03887
MZB1	0.450082673	0.393151	0.794847243	0.11362	1.018084389	0.03886
P11-820K3	-0.068885947	0.885925	-0.506785961	0.29867	0.907732751	0.03884
RP4-800G7.	-0.489793646	0.407328	-0.770214288	0.19179	-1.344352622	0.03898

DDX49	0.107689897	0.514585	-0.028525396	0.86278	-0.341561675	0.03911
HMGN3	-0.183598828	0.155663	0.125409163	0.32857	-0.26654696	0.03916
NFX1	-0.007874163	0.9061	0.042446876	0.51753	0.135917443	0.03915
RAB10	-0.137201509	0.277451	0.215225538	0.08794	-0.260578047	0.03914
SPTLC2	-0.113264945	0.360098	-0.064990503	0.59868	0.254441281	0.03917
C20orf202	-0.212216921	0.7335	-0.800619447	0.21287	-1.483259353	0.0393
CKS2	-0.052797098	0.711298	-0.191115497	0.17935	-0.294575732	0.03933
RPL18AP3	0.029892583	0.834671	-0.20864106	0.14513	-0.295320853	0.03936
RAB3A	-0.712840675	0.093004	-0.372763145	0.34296	-0.878688324	0.03938
RPRD2	-0.038478585	0.741929	-0.179911761	0.12248	0.237970983	0.03939
CASP2	-0.012478089	0.943725	-0.200857256	0.25405	0.361774085	0.03942
CES3	0.044769266	0.868527	-0.201120575	0.45423	0.538458586	0.03943
P11-744O11	-0.380361339	0.500773	0.686528737	0.16469	1.00676281	0.03945
AL512503.1	2.300793983	0.471377	3.980660893	0.2004	6.352051486	0.0395
PPHLN1	-0.000425607	0.995667	-0.076571236	0.32347	-0.162179457	0.03957
YIPF3	0.035715779	0.737407	-0.039476083	0.70989	-0.219791701	0.03957
CCNE1	-0.396586735	0.058357	-0.394637294	0.05875	-0.430506974	0.03967
C1orf222	-0.326255048	0.421346	-0.503811725	0.21829	0.808347782	0.03969
PNMA2	0.542537092	0.053634	-0.327564117	0.24521	0.577709766	0.0398
TMF1	-0.020116187	0.885529	-0.264009727	0.05875	-0.287276844	0.03979
DMD	-0.079812358	0.71812	0.206903747	0.34918	0.45428302	0.03982
SMG7	-0.060528921	0.791656	-0.019520337	0.93205	0.469332298	0.03986
ZNF423	0.352432516	0.131695	0.408717243	0.07871	0.479284561	0.03987
LLGL2	-2.49E-05	0.999888	-0.133666684	0.45123	0.364062341	0.03997
SIGLEC8	0.219721737	0.524732	-0.094975039	0.78143	0.672689897	0.03997
PPIC	0.196433445	0.191893	0.239309734	0.10982	0.307785798	0.04
IFT140	-0.107632499	0.430749	0.099799045	0.46264	0.279002905	0.04005
PCBP2	-0.108452069	0.611926	-0.387337036	0.06999	0.438307524	0.04004
MAN2C1	0.157748923	0.323507	-0.045621948	0.7748	0.327039618	0.04008
SLC25A14	-0.229700102	0.18481	-0.211495392	0.21558	-0.358897128	0.04007
FAM26F	-0.430445692	0.329502	0.007724266	0.98497	-0.941693133	0.04013
CDH15	-0.320700654	0.50387	0.054475371	0.90189	0.875098807	0.04018
RAB2B	0.209708309	0.193111	0.168949004	0.292	0.328652161	0.04021
CTC1	0.227752033	0.240492	0.069378933	0.71955	0.395866446	0.04029
RDH16	-0.142777789	0.60066	-0.237054814	0.37867	0.534268887	0.04033
RPS2P7	1.276416459	0.057653	1.120515485	0.09189	1.354645032	0.04041
ART3	0.496162988	0.244606	0.759215772	0.06194	0.829070798	0.04046
SBDSP1	-0.255799296	0.0672	0.08543024	0.52496	-0.284431257	0.04047
LSM4	-0.103632041	0.469993	-0.108096734	0.44948	-0.293944294	0.04054
BIVM	-0.077564513	0.585295	0.25482216	0.06795	0.286718549	0.04066
CUL4A	0.160289841	0.063933	0.093006779	0.27784	0.17638762	0.04065
PPP1R1A	-2.148437574	0.080258	-0.137111352	0.89844	-2.595220126	0.0407
C19orf43	0.189315226	0.065787	0.091344485	0.37322	-0.211497357	0.04078
EYS	0.153688067	0.504198	0.062787021	0.78288	0.462453625	0.04078
FABP2	0.318986783	0.650521	-0.095106617	0.8926	-1.454053984	0.04077
CDYL2	-0.495549239	0.299612	-0.692516659	0.14636	-0.979224269	0.04094
GTF2A1	-0.109963058	0.293861	0.069828339	0.50202	-0.213836574	0.04095

C20orf144	0.287540561	0.434002	0.428957291	0.22642	0.717950469	0.04098
UBE2I	-0.128157616	0.301284	0.175845326	0.15453	-0.253451655	0.04101
TSN	-0.084732046	0.324734	-0.046601259	0.58517	-0.17564208	0.04103
RORC	0.303114102	0.61572	0.525589341	0.36514	1.153215951	0.04105
P11-231C14	0.697354183	0.45996	-0.736123151	0.4451	1.917388723	0.04106
GPR89A	-0.125372757	0.405714	0.091718406	0.53895	-0.308778698	0.04108
FAHD2P1	-0.385665747	0.416863	-0.791329091	0.09828	-1.003812968	0.04114
ABCC1	0.198379277	0.368363	0.383246448	0.08053	0.448730682	0.04116
OLFM2	0.209760736	0.335625	-0.324517755	0.14161	-0.456929765	0.04119
DMGDH	-0.051494309	0.916645	-0.024310299	0.9596	0.93602125	0.0413
RP1-224A6.:	0.203551516	0.702635	0.404192735	0.42358	0.992884398	0.04136
DCAF12L2	1.303426728	0.080254	1.186715232	0.10765	1.492520464	0.04137
RPL22L1	-0.064617697	0.746476	-0.280040887	0.16067	-0.408646347	0.0414
TCF7	-0.002016276	0.992193	-0.110156339	0.5872	0.411296971	0.04146
TJP3	0.172755823	0.467149	0.15362151	0.51759	0.483561734	0.04145
NFE2L1	-0.160024606	0.380826	0.062781234	0.73089	-0.372259887	0.04147
RWDD1	-0.163449698	0.117723	0.137941884	0.18056	-0.212550965	0.0415
COX14	-0.182340394	0.248698	-0.275211964	0.07713	-0.323279971	0.04154
CYCSP2	0.712472577	0.301872	-0.160778562	0.82578	1.334188513	0.04155
H2BFS	-0.139946948	0.507018	-0.049377494	0.81432	-0.430496268	0.04161
LHCGR	-0.151644274	0.789189	0.40669696	0.43709	1.030369849	0.0416
LRRC37A2	-0.053737552	0.614606	0.145549526	0.15874	0.211493265	0.04161
MBL1P	-0.425667906	0.545869	0.592177896	0.35249	1.283373271	0.04159
IGSF6	0.290681653	0.368697	0.235903756	0.4585	0.639460963	0.04168
ABCG5	0.865453149	0.239964	0.900785129	0.21112	1.437581248	0.0417
PRR14	0.221745378	0.306289	0.104146439	0.63188	0.439557534	0.04172
OPHN1	-0.112092717	0.547693	0.252964127	0.1719	0.376524941	0.04175
CCNE2	-0.383063001	0.118804	-0.090378925	0.71053	-0.500637244	0.04187
SRRM4	-0.14239086	0.831951	0.835241484	0.18224	1.262376461	0.04186
SLC36A2	0.338614648	0.354041	0.10200187	0.78019	0.724053531	0.04193
EIF3G	0.014931805	0.866068	-0.095564171	0.27901	-0.180448767	0.04196
RDH5	-0.446236487	0.124852	-0.327169405	0.25689	-0.603349807	0.042
P11-645C24	0.094153511	0.789573	-0.11794632	0.73802	0.71231219	0.042
SUGT1	-0.119202985	0.175038	-0.100102446	0.25247	-0.177913438	0.04206
CDC37L1	-0.109210527	0.515079	0.008380302	0.95969	-0.340453954	0.0421
TD-2013N17	0.845943568	0.257099	0.644289465	0.38486	1.448169303	0.0421
ZKSCAN8	0.036377857	0.814513	0.002358512	0.98781	0.313594299	0.04214
KLHL20	-0.153933904	0.199585	-0.210562677	0.07757	-0.244088585	0.04216
PDZK1	-0.546829726	0.299362	-0.676427916	0.19855	-1.072472477	0.04217
ABCB7	-0.057641651	0.725182	-0.190723103	0.24273	-0.333447695	0.04219
C3orf14	-0.363227595	0.113173	0.165967995	0.44229	-0.463379387	0.0422
MYO7B	-0.590557655	0.354944	-0.786162678	0.21808	-1.297663581	0.04225
MYO1G	-0.047251165	0.899959	-0.117031515	0.75749	0.738051979	0.0423
FAM177B	-0.068708673	0.847383	0.458823979	0.19478	0.718630863	0.04234
MESTP1	-1.223604839	0.074657	-0.27935873	0.64196	-1.401226209	0.04232
UBE2CP1	-0.05358806	0.940141	-0.124887412	0.85759	1.282230601	0.04234
TD-2083E4	0.231521726	0.76664	-0.027896245	0.97145	1.519082545	0.04239

GALNT10	0.253931273	0.175451	0.352302946	0.05926	0.379544436	0.04239
HINT1	0.087971687	0.289288	-0.094201264	0.25594	-0.168658951	0.04264
FUNDC2	-0.159892748	0.340619	-0.241343259	0.14963	-0.340246804	0.04269
AKT3	-0.185180892	0.41718	0.273562502	0.22592	-0.462974995	0.04272
CACUL1	-0.226839262	0.070349	-0.044205814	0.7236	-0.253651654	0.04271
GPT	-0.089809311	0.802828	-0.234013836	0.51817	0.712574261	0.04272
TD-2124B8	-0.219192741	0.611525	-0.232375265	0.58164	-0.927591579	0.04274
GPR114	-0.044138731	0.900562	-0.114207565	0.74654	0.705902546	0.04276
MUC17	-0.546546093	0.471576	-1.312031101	0.08431	-1.544257741	0.04276
PCBP3	0.129645356	0.72276	0.507192356	0.15967	0.721185725	0.04277
ERLEC1	-0.165433315	0.11944	-0.004670501	0.96471	-0.214958036	0.04283
MFN1	0.135237958	0.174588	0.135445514	0.17219	0.201184444	0.04283
KCNN4	-0.093742872	0.826984	-0.16111339	0.7085	0.839343264	0.04286
UBE2L4	-0.512660538	0.53831	-0.539534095	0.50158	1.396618244	0.04292
AC005884.1	-0.526057982	0.408789	-0.734982612	0.24579	-1.373584989	0.04296
CUTA	0.031837926	0.722098	-0.155346315	0.08152	-0.181774716	0.04295
MBIP	-0.209517235	0.210325	0.041900201	0.79979	-0.339319365	0.04297
MSH5	0.503831959	0.131084	0.219586375	0.51052	0.674249634	0.04299
SLC25A34	0.455717922	0.189127	0.36869575	0.28041	0.685391028	0.0431
SPDYE6	1.371919757	0.19531	1.734077061	0.0999	2.127794043	0.04319
RPL36AL	-0.083345908	0.47994	0.080327904	0.49483	-0.238538037	0.04325
RPL26	-0.192406522	0.292049	0.160509231	0.37933	-0.369059452	0.04329
KMO	-0.07162951	0.861534	0.649569339	0.09909	0.789588698	0.04331
QSER1	-0.06636629	0.770791	0.078956355	0.72849	0.459570087	0.04331
ARHGAP26	0.349229751	0.079595	0.282027486	0.1558	0.401387448	0.04336
GPN1	0.038199967	0.658156	0.043679979	0.60806	-0.175749189	0.0434
HIST1H4F	-0.325675323	0.171821	-0.363127309	0.12729	-0.481500589	0.0434
MAFG	0.194746284	0.083824	0.057041733	0.60984	0.226462859	0.04337
MYOM1	0.433770314	0.339881	0.604674389	0.17537	0.899041005	0.04338
AHRR	-0.595788322	0.114927	-0.176866235	0.63732	-0.769926253	0.04349
BRINP2	0.290821255	0.670715	1.148907324	0.0905	-1.45939691	0.04345
CPN1	0.792725396	0.403444	-1.124704509	0.26899	-2.446176602	0.04347
TNFAIP8L3	0.139854979	0.714065	0.212713492	0.56992	0.74350073	0.0435
UPB1	0.531592116	0.131563	0.191518431	0.58372	0.689450386	0.04343
ZNF621	0.085605967	0.461645	0.129280856	0.26054	0.232722811	0.04348
ISM1	-1.157315009	0.05021	0.767954737	0.14966	-1.182845106	0.04352
FBXL8	0.031150295	0.934424	-0.007878952	0.9833	0.730976054	0.04356
IFLTD1	0.752352187	0.202037	0.669184762	0.24372	1.155314588	0.0436
ZNF383	-0.015352472	0.946812	0.19075675	0.40386	0.461137417	0.0436
ZSCAN22	0.12352571	0.274739	0.051180434	0.64317	0.222545457	0.04368
RB1CC1	0.036740416	0.732743	0.129645227	0.22582	-0.217414501	0.04371
TCTE1	0.078227721	0.873357	0.34768486	0.45627	0.940204323	0.0437
TAS2R3	-0.926380389	0.211768	-0.722923686	0.29791	1.21964697	0.04374
PROK1	-0.885752343	0.478998	-0.01522886	0.99013	-2.677048736	0.04377
NR1I2	-0.527257579	0.386626	-0.981510902	0.10764	-1.235759259	0.04379
PPCDC	0.233940954	0.123297	0.068187336	0.65367	0.303770115	0.04387
AC253572.2	0.003437044	0.988195	0.014355255	0.95039	0.462294348	0.0439

FAHD1	-0.1320439	0.191824	-0.157557255	0.11386	-0.203108728	0.04391
SH3RF3	0.132337185	0.663342	0.493924964	0.09614	-0.63408641	0.04392
RNF215	0.168396392	0.4091	0.188051817	0.354	0.407243199	0.04396
EPB41L1	0.221138188	0.326487	0.113360724	0.61522	0.453011898	0.04402
SLC2A4	0.265462189	0.471663	-0.415294194	0.26572	0.733927366	0.04402
P11-381O7	0.116712715	0.909898	-0.721161556	0.50368	1.914246772	0.0441
TRNAU1AP	0.069040425	0.514071	0.0987397	0.33246	-0.212130388	0.04413
ZNF26	-0.052759599	0.688972	-0.103293151	0.43021	0.262362817	0.04416
PSME4	-0.048825188	0.730898	0.113722471	0.42163	0.284791924	0.04426
AMIGO1	-0.3922489	0.058285	-0.226469747	0.25363	-0.413489272	0.04431
RP11-391L3.	-0.234591202	0.793496	0.401242785	0.62158	1.535727285	0.04435
NMT1	-0.065569799	0.569615	-0.125188884	0.27743	-0.231863431	0.04447
SMCR8	0.00217515	0.983308	-0.03968886	0.70123	0.207444164	0.04454
PIGS	0.126437928	0.195458	0.040671238	0.67598	0.195673166	0.04456
CD9	0.035496104	0.895699	0.250825256	0.35328	-0.545022508	0.04468
CHP1	0.22381285	0.12574	0.064840999	0.65733	0.293324644	0.04466
SGALNACT	0.348962602	0.20323	0.083380361	0.76079	0.549145621	0.04464
SH3BP5L	-0.118548282	0.211587	-0.049071588	0.60092	-0.190784404	0.04469
XKR7	-0.974253176	0.334939	1.358058834	0.14419	-2.23695828	0.04466
AC005077.1	0.793153098	0.148957	0.449651324	0.41502	1.074827814	0.04473
CSPG4	0.40382029	0.141921	0.372783833	0.17412	0.550312849	0.04477
ATRAID	-0.062901858	0.517551	-0.17615323	0.06883	-0.195055855	0.04481
FAM206A	-0.257752166	0.050727	-0.086098137	0.50754	-0.263247326	0.0448
SARS	-0.020565922	0.846964	-0.075843069	0.47565	-0.213945956	0.04482
TCTN1	-0.015309981	0.9014	0.216778357	0.07485	0.245345131	0.04479
DOCK5	-0.099754836	0.657413	0.271497321	0.22618	0.450135876	0.04485
RP11-473I1.	0.108168658	0.209236	0.064185599	0.45551	0.172547308	0.0449
CNPY4	-0.098751754	0.598001	-0.135263798	0.4667	-0.379024362	0.04495
P3-324O17	0.087635365	0.687676	0.144103764	0.50166	-0.447739138	0.04495
COX7B	-0.060903954	0.555204	0.027082015	0.79215	-0.206883905	0.045
COPRS	-0.027473792	0.841986	0.039067911	0.77288	-0.278105037	0.04512
TNKS	-0.00216075	0.986591	0.011565139	0.92806	0.25637912	0.04513
SP140L	0.291707576	0.076707	0.176363934	0.28366	0.329365485	0.04522
GAPVD1	-0.052493076	0.508408	-0.030771901	0.69612	0.157936346	0.04535
GRB7	-0.108461542	0.695136	0.006094841	0.98219	-0.564241731	0.04532
MYCBPAP	-0.039273761	0.914829	0.286977014	0.41474	0.704840247	0.04535
RPS7P14	-1.049932609	0.085526	-1.036771829	0.07621	-1.22867389	0.04542
HYKK	0.070520988	0.693157	0.033514874	0.84804	0.345086703	0.04546
SLMO2	-0.004773363	0.963946	-0.041418798	0.69412	-0.211118513	0.04558
CANX	-0.12838279	0.22353	-0.122056141	0.24707	-0.210794515	0.04565
METTL12	-0.421986542	0.075135	-0.111744608	0.6347	-0.472030335	0.04571
HAP1	-0.072991325	0.827156	0.327940805	0.31368	0.637100328	0.04573
SERTAD3	0.113129658	0.414668	-0.216384366	0.11936	-0.278721507	0.04573
RLIM	0.030219922	0.682928	0.056297471	0.44487	-0.147881375	0.04575
CHMP4BP1	0.729458132	0.128488	0.695024852	0.14092	0.937936548	0.04579
DDTL	0.163572605	0.629281	0.397594973	0.22121	0.644977724	0.04586
NDUFB11	-0.010026025	0.932608	-0.208801329	0.07742	-0.237026074	0.04598

REXO4	-0.230871636	0.138033	-0.190535611	0.21404	-0.309557121	0.04599
COMMD6	-0.156283635	0.296027	-0.092020647	0.53676	-0.298473744	0.04606
PRPF38A	-0.079582519	0.308596	-0.011078678	0.88536	-0.154848934	0.04607
CSPG4P5	2.508495339	0.210359	3.389825921	0.08811	3.957716431	0.04612
PAK6	-0.567903441	0.135661	-0.362830069	0.33316	0.729121434	0.0461
P11-600F24	0.731952747	0.385858	0.779244769	0.3408	1.561008987	0.04611
SMPDL3A	-0.296795601	0.455052	-0.09745831	0.8059	-0.79374345	0.04612
KCTD12	0.23031345	0.272276	0.006607598	0.97487	-0.418752406	0.04616
ABCA2	0.119925613	0.664951	0.135576389	0.62452	0.550836012	0.0462
GRIK3	-2.9247334	0.05774	1.255467204	0.33228	-3.214346793	0.04618
HMGB1P5	0.012573547	0.954301	-0.031288814	0.88641	-0.438741781	0.04621
KIAA1328	-0.076412304	0.757829	0.240292628	0.33176	0.493405657	0.04623
OR11A1	-0.050799153	0.935528	0.398740262	0.50212	1.146938212	0.04627
S100P	0.460900369	0.158513	0.354169095	0.27842	0.651274814	0.04625
CORO6	0.745877033	0.13259	0.863570015	0.07604	1.014468341	0.04637
SPINK9	-0.137779176	0.613584	-0.199856212	0.462	0.536299138	0.04642
ANAPC10	-0.207751722	0.096926	-0.174622529	0.15344	-0.248871329	0.04644
ENPP7	0.000388201	0.999676	-1.036073326	0.29577	-2.219345354	0.04647
CIT	-0.401033221	0.1399	0.054094722	0.84166	0.538448719	0.04661
GOLGA5	-0.196364077	0.174121	-0.213921033	0.13764	-0.287581835	0.04657
RXRA	-0.04491285	0.709163	-0.069828997	0.56039	0.237907818	0.0466
ANKRD46	-0.042380939	0.772569	-0.230753087	0.11369	-0.292821608	0.04665
P11-731D1	0.630774816	0.392024	0.502463402	0.49017	1.388777106	0.04665
KLKB1	1.067171685	0.187072	0.29497097	0.73083	1.605666202	0.04668
NUP62	0.142376659	0.234068	-0.005183586	0.96533	0.237346587	0.04667
THSD1	-0.192101965	0.629141	-0.113190858	0.77043	-0.824215469	0.0467
SETD3	-0.036553235	0.788532	-0.01748924	0.89762	-0.271236677	0.04674
CAPZA2	-0.069959109	0.48733	-0.133823972	0.18309	-0.200165751	0.04679
RYK	-0.148107755	0.109385	-0.124913362	0.17506	-0.183579839	0.04687
AP000350.6	-0.278986401	0.625455	0.416468462	0.41965	0.995039069	0.04692
JADE1	-0.368369709	0.335732	-0.386728892	0.31126	0.753692904	0.04695
TAF7L	1.299850298	0.100962	0.98705875	0.20805	1.504142364	0.04717
EPG5	0.355163878	0.070117	0.330062332	0.09137	0.387873335	0.04725
GNAI3	0.06714697	0.270001	-0.032833361	0.5883	-0.120934487	0.04726
AC010642.1	-0.038458751	0.6698	-0.073777587	0.40821	-0.179009404	0.04728
PARP9	-0.188293508	0.136957	-0.034467099	0.78366	-0.250336368	0.04731
MFNG	-0.46847548	0.385561	-0.851634056	0.12057	-1.063684495	0.0474
MRPL21	-0.145092449	0.239001	0.011747767	0.92223	-0.243804144	0.04741
NCL	0.114913455	0.388333	0.0229665	0.86309	-0.264206863	0.0474
SSR1	-0.128091347	0.123771	-0.154131858	0.0632	-0.164684826	0.04738
IZUMO4	0.12002831	0.736446	-0.036450028	0.91786	0.695291138	0.04749
PPID	-0.212195399	0.151243	0.019732104	0.89306	-0.293015409	0.04746
PIWIL3	0.725535925	0.369934	-0.055914713	0.94766	1.49102188	0.04751
HSD17B12	0.233763095	0.053733	0.003610623	0.9762	0.23990976	0.04753
SMC2	-0.205347566	0.07369	0.146503659	0.19878	-0.227231362	0.04754
GJB3	-0.039907017	0.934698	0.288147088	0.55127	-0.979861493	0.04758
CCDC146	0.281975154	0.24361	-0.020546328	0.93193	0.472652163	0.04763

GPR18	0.181113844	0.655887	0.206468679	0.60261	0.770656412	0.04767
ZFAND2A	-0.16065236	0.467593	0.009046497	0.96706	-0.440503497	0.04765
FAM115C	-0.050343138	0.82242	0.080945336	0.71541	0.43774052	0.04774
CDKN1A	0.655293418	0.061816	-0.244066588	0.48723	-0.696890503	0.04781
CCDC113	-0.214461661	0.368046	-0.045906603	0.84321	-0.473452751	0.04785
SLC6A4	-0.522797762	0.23122	-0.412299536	0.33135	-0.856254262	0.04788
CLN3	0.283742902	0.074268	-0.086489803	0.5854	0.311919423	0.04791
KLF14	0.38953987	0.496213	1.047595424	0.0566	1.090403774	0.04803
BX088651.1	0.63008708	0.263063	1.01629113	0.05721	1.063757668	0.0481
ATSPER2P	0.075581106	0.811297	0.222923143	0.47054	0.611615379	0.0481
C17orf51	0.010410612	0.990965	0.442756873	0.62103	1.716475318	0.04812
P11-16E23	0.038858867	0.947145	0.309688211	0.58814	1.107460351	0.04819
SWAP70	0.117080846	0.345834	0.050025258	0.68381	0.244320229	0.04818
SHROOM3	0.211056107	0.184266	0.103559572	0.5143	0.313524583	0.04827
PATE4	-0.061845918	0.858929	-0.259523938	0.44915	0.636790238	0.0483
P11-927P21	-0.217583898	0.537655	-0.154055832	0.65704	0.668305331	0.04836
ACACB	0.082071315	0.764514	0.266857843	0.32792	0.537263579	0.0484
CD28	0.925278726	0.111991	0.967024359	0.09069	1.127822088	0.04841
CEACAM6	-0.256534738	0.631666	-0.29695672	0.57894	-1.056296802	0.04841
PAPOLA	0.043593574	0.590125	0.028296631	0.72624	0.159575311	0.04838
SCAF11	-0.100408452	0.287356	0.020630076	0.82636	0.185611048	0.04842
LAIR1	0.1525323	0.732463	0.217439597	0.61914	0.845172786	0.04848
UBR7	-0.11273876	0.45493	0.028483292	0.84767	-0.296633026	0.04847
PSME3	0.26071601	0.06285	0.142357185	0.30876	0.27634498	0.04849
FIS1	-0.001058835	0.993128	-0.208251079	0.08869	-0.243071716	0.04859
SCAF1	0.049244597	0.820713	0.232409233	0.28167	0.425261961	0.04864
BCAS2	-0.052708374	0.564143	0.0274787	0.76053	-0.180056816	0.04869
LANCL3	0.188146536	0.618357	0.080137877	0.83291	0.736120146	0.04867
KRT8P3	0.653409107	0.234354	0.237099432	0.66944	1.041713324	0.04871
PPP4C	0.067359265	0.511998	-0.027350279	0.78918	-0.203495103	0.04873
DOC2A	0.147187457	0.696359	0.091653783	0.80637	0.726639739	0.04878
MBD6	0.239085754	0.494382	-0.435930944	0.21562	0.683640748	0.04881
HLA-V	0.335444178	0.350345	0.426263791	0.23127	0.701345683	0.04884
STK32C	-0.25299245	0.206802	-0.176200988	0.36478	-0.388182879	0.04886
SRC	-0.147239936	0.267891	-0.245530432	0.0643	0.260281511	0.04903
P11-74M13	0.517575334	0.222088	0.472300204	0.25946	0.817323818	0.04906
OMMD3-BM	0.129448581	0.654395	0.507073096	0.07673	-0.574066158	0.04914
CCNJP2	-0.191984987	0.788835	-0.414911973	0.56006	1.268927079	0.04917
ADAM1B	0.270431574	0.68276	0.223213856	0.72928	1.190404542	0.04918
VIPR2	0.314197105	0.546903	-0.148490212	0.77697	1.01636347	0.04926
AC005077.1	0.075857536	0.835248	0.018446185	0.95872	0.701554547	0.0493
COL5A2	0.029851234	0.901743	0.013691695	0.95484	-0.475341471	0.04937
SLC34A3	0.35689574	0.528939	0.791828283	0.13432	1.032710343	0.04939
NAA20	-0.089732756	0.37181	-0.018833888	0.84818	-0.196985541	0.04943
SPOCK3	-0.57458254	0.4242	0.870695064	0.21081	-1.486095111	0.04945
WNT2B	-0.106358813	0.589854	-0.216673436	0.26215	0.375286411	0.0495
FITM2	-0.126660043	0.31236	-0.123181549	0.31077	0.234269401	0.04958

SIAH3	-0.571084269	0.433883	0.932036624	0.12462	1.184647615	0.04959
FAM92A1	-0.191221318	0.143788	0.133835165	0.29865	-0.2550161	0.04961
PARK2	0.236328433	0.278294	0.197401222	0.36614	0.421877754	0.0497
CA6	0.367231893	0.194118	-0.284586639	0.33325	0.538901984	0.04978
DFFA	-0.070149081	0.504706	-0.033307614	0.74982	-0.205548621	0.04984
PAQR8	0.038049352	0.82836	-0.030820305	0.86048	0.343599164	0.04986
EPS15L1	-0.189163103	0.262959	-0.122420702	0.46577	0.328120577	0.04993
FUS	0.426859391	0.051644	-0.150320642	0.49346	0.429616104	0.04994
MTIF3	-0.192632702	0.085172	-0.082321764	0.45575	-0.218690716	0.04997
ERH	0.0342136	0.774936	-0.05035028	0.67334	-0.234955684	0.05
AC002985.3	-0.605912562	0.698937	-0.975946157	0.53458	-4.329111097	0.01648
AC010900.2	2.104987388	0.15257	2.003747437	0.16695	3.23459319	0.01809
AC011242.6	-0.260134098	0.748473	-0.309937292	0.69589	-2.268911852	0.03519
AC013474.4	1.036115718	0.290871	1.107245207	0.24447	1.89088557	0.03858
AC068137.1	1.057528553	0.554013	0.043980582	0.98037	3.634792628	0.01937
AC073869.1	-1.213085813	0.212686	-1.328031997	0.16619	-2.116023367	0.04527
AC079250.1	-1.377223793	0.451415	-1.817852046	0.3229	-4.682154983	0.02029
AC090286.2	0.686026438	0.407301	0.397989437	0.63031	1.993014547	0.00736
AC098614.1	-0.754922692	0.406972	-2.111221611	0.05325	-2.520971707	0.03701
AC110299.5	0.228985602	0.79689	0.443675989	0.60307	1.703475236	0.03419
ADRA1D	0.414930179	0.710351	0.464188693	0.67208	-3.396049242	0.01817
ATP5G2P4	-1.200539987	0.468235	-1.102085899	0.49497	-3.662921194	0.04107
ATP5J2LP	1.791011687	0.320145	3.042580047	0.05996	3.87309767	0.01399
C13orf45	-1.083842138	0.427094	-1.194999522	0.37483	-3.215241309	0.04087
C9orf135	-2.2645782	0.098691	-0.968382933	0.37384	-3.084955768	0.02568
CATSPER3	-0.703833994	0.493917	-1.109087676	0.28891	-2.652786295	0.03873
CD244	-0.467800567	0.70461	-1.211093695	0.32331	-3.864841921	0.01146
CLEC2L	2.090412666	0.163928	1.039667583	0.51343	3.004906785	0.04881
CTB-23I7.1	-2.21802301	0.239659	-3.250781742	0.08734	-3.815850491	0.04477
CTB-46B19.	-1.375148926	0.26732	-1.134501382	0.32322	-2.796610679	0.04113
CTBP2P7	1.764243522	0.33375	0.765316911	0.6771	3.42460997	0.03511
TC-250P20	0.73453946	0.495287	0.448650716	0.6769	1.984781461	0.04729
TD-2165H16	0.501517225	0.677337	-1.212444958	0.37914	-3.029165112	0.04224
D-2192J16.	-1.457507522	0.329103	-0.809832285	0.56452	-3.992514301	0.01576
CTXN2	-0.542286834	0.599905	-0.662780296	0.51384	-3.01493981	0.02344
ECEL1	-0.335444816	0.748995	0.022529861	0.98243	-2.820355369	0.04036
EGLN1P1	2.311292806	0.318758	0.043940365	0.98531	4.547168428	0.03781
FTH1P4	1.041566533	0.269302	1.496352116	0.0955	2.134756794	0.01502
FTLP15	-1.571176688	0.298687	-0.078724436	0.95035	-3.136622608	0.04
GRK1	1.610346335	0.371245	2.562102047	0.11691	3.889948937	0.0128
H2AFZP4	-0.962355897	0.539181	0.784416941	0.54956	2.636792783	0.03358
HMGN1P7	-0.921728437	0.41369	0.365949731	0.68364	1.629280087	0.04854
HNRNPA1P3	0.820509021	0.391675	0.771774757	0.41159	1.860533119	0.03696
NRNPA1P5	-0.882419803	0.500937	-0.293939983	0.80361	-3.018939049	0.03517
NRNPA1P6	-1.218452456	0.368369	-0.738168921	0.56064	-3.722753017	0.01468
NRNPA1P7	0.926331435	0.558741	2.537270021	0.07311	2.928023109	0.03727
HRH2	-0.140684668	0.926521	0.238633718	0.86944	2.549600666	0.04358

HSD17B1P1	0.18480234	0.850993	0.100279488	0.91749	-2.831985692	0.03356
IL13RA2	-0.682537741	0.528388	-0.912526455	0.40063	-3.464747535	0.01371
ISM2	-1.753469097	0.129283	-1.447489173	0.15745	-2.146195262	0.04447
KATNBL1P3	0.413577377	0.692876	1.736442888	0.06262	2.150508126	0.01979
KB-1090H4.	0.336184462	0.847048	1.703788054	0.28499	3.153645292	0.03429
KCNMB3P1	0.292464311	0.868678	1.753382748	0.2891	3.2869808	0.04156
LENEP	-0.013840473	0.989996	-0.322083326	0.77024	1.861297675	0.04105
LY86	0.471656242	0.672271	0.748264843	0.51173	-2.909564019	0.04236
MCCD1	1.220753024	0.195109	0.091012739	0.92883	1.932930277	0.03088
MUSTN1	-1.357546624	0.454959	-1.125681745	0.52862	-4.414930187	0.02673
NKX2-5	-1.557228767	0.347116	-0.904054831	0.57414	-4.240602536	0.02268
OR1AB1P	0.158586352	0.890437	0.397093028	0.71727	2.28528839	0.02293
PLA1A	1.283005587	0.302708	0.297542457	0.81888	3.025500063	0.01138
POTEKP	1.391258475	0.211488	2.044063183	0.0555	2.184182475	0.04082
PPP1R42	0.527026767	0.627844	-0.295614678	0.79967	2.157939453	0.03091
RBFOX1	1.674799943	0.234653	1.929039128	0.13173	2.725188746	0.03418
RBMS2P1	0.753302071	0.701374	2.270332102	0.21268	3.657334879	0.04067
P1-102E24	0.338320297	0.776267	-0.165882272	0.89105	-2.980330475	0.04191
P1-149A16.	1.625854153	0.255355	-0.672966193	0.67972	2.674210582	0.04655
RP1-154J13.	1.057521027	0.523841	2.421947307	0.10665	3.779983377	0.00678
11-1149O2	0.905725477	0.322822	1.124670868	0.1999	1.825998153	0.03128
P11-129H15	-0.749321337	0.361976	-0.927914664	0.25255	-3.041898742	0.01032
RP11-15J10.	0.392793963	0.707254	0.589263587	0.55813	2.095337404	0.02627
P11-177A2	-0.439133744	0.727349	-0.69224943	0.58131	-3.352576667	0.02426
P11-317N8	0.391099437	0.763019	0.799858223	0.51124	2.389229702	0.03482
P11-384C4	1.317356573	0.26017	1.544024881	0.16938	2.730506684	0.0105
P11-403F21	2.027740024	0.110124	-1.298687841	0.40592	2.649715262	0.03195
P11-407P18	0.496856709	0.583569	1.156911545	0.16525	1.67143391	0.04048
RP11-417J1.	2.372005488	0.145619	2.785907292	0.0716	3.174746482	0.03748
P11-460E7	1.916409374	0.226284	0.951860905	0.56348	3.06970648	0.04312
RP11-466F5.	1.071205466	0.563652	0.833151264	0.6504	3.275779312	0.04467
P11-521A24	0.336173865	0.852026	1.645992821	0.3229	3.432825737	0.02642
P11-522L3.	-0.434757595	0.712713	0.613518819	0.55295	1.979111913	0.04214
P11-578F21	0.415420644	0.773505	1.814173552	0.167	3.050483812	0.01712
RP11-60E8.	0.336192418	0.84877	2.618131644	0.08816	3.144604355	0.03779
P11-624D20	1.701212044	0.269106	1.74990476	0.24689	2.920062347	0.04486
P11-690I21	0.302306279	0.8096	0.287410846	0.81326	2.185702683	0.0439
P11-697H9	0.84299207	0.374538	0.682926106	0.46726	1.783491081	0.04474
P11-841C19	1.336589931	0.257743	1.249787708	0.2817	2.272836212	0.0391
RP11-98L4.	0.723303018	0.43824	1.029350352	0.25018	2.021960152	0.01887
P4-620E11	1.649665306	0.303463	2.135323859	0.15762	2.874669344	0.04995
P4-791C19	-1.919099648	0.059134	-0.567789313	0.49091	-2.04441057	0.04379
RPL21P4	2.741074464	0.06315	1.961508365	0.20407	3.971396268	0.00351
RPL23AP2	2.236005494	0.25572	1.938488348	0.32473	5.195254246	0.00369
RPL23AP51	1.057506223	0.584316	2.096311065	0.25411	4.277813042	0.01149
RPL24P8	2.106102107	0.115249	2.435237493	0.06038	3.00012091	0.01887
RPL7L1P3	0.376294739	0.662774	0.649733422	0.42392	1.628513299	0.03323

RPL7P49	0.299293445	0.795387	-1.709755124	0.20903	2.23362097	0.02097
RXRG	-1.236214986	0.395044	-1.423877073	0.3069	-4.010827968	0.01344
SAMD7	0.603089843	0.501799	1.175750656	0.15595	1.666310319	0.03981
SCAND3P1	-2.268629277	0.133362	-0.620523458	0.62022	-3.124498869	0.0409
SLC35D3	1.48119454	0.110568	1.242310622	0.17934	1.798979946	0.04675
ST13P20	1.715320556	0.1122	1.3926329	0.19803	2.100627105	0.04593
TDGF1P6	0.311581558	0.822216	0.004214428	0.99758	2.518782078	0.03094
TMEM196	-0.897104693	0.571735	-1.92736056	0.23343	-3.57153617	0.0448
TMPRSS11I	-3.040254433	0.098623	-0.390430858	0.80774	-3.897524689	0.03424
UNC5A	3.151405232	0.074602	2.235036667	0.2142	3.747397093	0.03135
WFIKKN2	-0.883169685	0.477843	-1.182875784	0.35971	-3.592219707	0.01492
I2-1896O14	-2.683862005	0.128987	0.372201538	0.80427	-3.541132393	0.04518
