# Supplementary material for: Comparative transcriptional profiling of the early host response to infection by typhoidal and non-typhoidal Salmonella serovars in human intestinal organoids
Source: PLoS Pathog. 2021 Oct 20;17(10):e1009987. doi: 10.1371/journal.ppat.1009987 (PMC8570492; doi:10.1371/journal.ppat.1009987)
Supplement: S2 Table — Significant DEGs (P < 0.05) in at least one infection condition are listed. (PDF) [file ppat.1009987.s010.pdf]

Table S2: DEGs 8h pi

	STM		SE		ST	
**Symbol**	**$\log_2$(fold change)**	**p-value**	**$\log_2$(fold change)**	**p-value**	**$\log_2$(fold change)**	**p-value**
CXCL6	5.597614945	1.91E-43	5.719778783	2.67E-45	4.888350653	1.65E-33
SOD2	2.81705125	1.23E-43	2.83169936	4.47E-44	2.38588314	8.77E-32
CCL20	5.300510006	4.49E-35	5.414192127	1.59E-36	5.090657662	1.76E-32
DEFB4B	7.243911346	1.55E-32	6.909222524	9.59E-30	6.420773856	6.96E-26
IKBKE	1.772301168	5.87E-32	1.609305473	1.20E-26	1.817166418	1.38E-33
CXCL2	3.549344875	1.91E-29	3.611847679	1.73E-30	3.427795606	1.45E-27
IL32	3.488754107	7.37E-27	3.325298192	1.59E-24	2.034517807	6.43E-10
CXCL5	4.548879155	7.53E-25	4.860781376	3.83E-28	4.208901901	1.67E-21
TNFAIP2	3.528253199	7.49E-25	2.853404944	8.61E-17	2.915110806	1.86E-17
ZC3H12A	2.524102503	1.70E-24	2.193919281	6.79E-19	2.452064109	3.20E-23
CXCL1	3.381493656	7.73E-23	3.426233721	2.07E-23	3.349726474	1.93E-22
NFKBIA	2.140364484	3.10E-22	1.899982903	7.32E-18	1.914383742	4.25E-18
LAMC2	1.760129217	2.23E-20	1.648316494	4.58E-18	1.078276316	1.47E-08
SLC6A14	3.254708399	8.97E-20	2.954361369	1.45E-16	2.716163587	3.11E-14
IL6ST	1.240230314	3.66E-19	0.892216691	1.22E-10	0.596142341	1.72E-05
C6orf222	2.81489137	4.21E-19	1.960670409	5.61E-10	2.573662145	3.36E-16
TNFSF14	3.113410599	7.88E-19	1.957557773	3.85E-08	2.616815726	1.34E-13
CXCL10	5.644047208	8.80E-19	4.994595788	4.97E-15	4.204744449	4.70E-11
NFKBIZ	1.098090646	3.10E-18	0.828541855	5.13E-11	1.21216762	6.72E-22
VNN3	3.025897821	3.58E-18	2.153474423	1.12E-09	2.346383559	2.85E-11
CXCL3	2.72446395	6.00E-18	2.909313296	2.89E-20	2.981728147	3.33E-21
OPTN	1.384529312	1.54E-17	1.151661555	1.25E-12	1.040081297	1.57E-10
GGCT	-0.96413316	5.42E-16	-0.098831551	0.38634	-0.451479418	0.0001
UBD	3.513513093	2.93E-15	4.771918153	5.47E-27	3.032430079	1.04E-11
TMEM14A	-1.109393403	3.28E-15	-0.464527201	0.00039	-0.742466523	4.09E-08
RARRES1	2.498000834	8.90E-15	2.822009608	1.63E-18	1.725034418	9.88E-08
ADAR	0.427531921	1.38E-14	0.288082969	2.02E-07	0.272088054	9.98E-07
KLHL5	0.77103178	3.95E-14	0.919414435	1.25E-19	0.554144056	6.10E-08
ICAM1	3.796347735	4.52E-14	3.254866603	1.00E-10	2.839174958	1.72E-08
ZC3H12C	1.041126776	5.48E-14	0.787221344	1.30E-08	1.033121756	8.04E-14
TNFAIP3	2.43530681	7.90E-14	2.052970383	3.05E-10	2.005836154	7.76E-10
ATP5H	-0.575098915	1.54E-13	0.052735268	0.48928	-0.271529303	0.00043
GCNT3	1.881369483	2.26E-13	1.35549111	1.29E-07	1.799239576	2.35E-12
SPRR2A	7.554988969	3.06E-13	6.231360974	1.97E-09	5.831614912	2.08E-08
P11-761N2	41.59064444	5.54E-13	39.13427327	1.17E-11	42.05169719	3.07E-13
CBX3	-0.500354773	5.82E-13	-0.01073835	0.87544	-0.340417192	8.42E-07
DUT	-1.13899228	6.40E-13	-0.542391121	0.00045	-0.683566927	1.14E-05
LRP11	-0.775958413	6.51E-13	-0.552606445	2.11E-07	-0.373235764	0.00046
FANCL	-0.901078799	1.03E-12	-0.492073443	7.11E-05	-0.446505849	0.00033
GRINA	0.928222913	1.87E-12	0.329985446	0.01277	0.390657248	0.00319
LIPG	1.302639796	2.45E-12	1.404362173	3.91E-14	1.217277899	5.76E-11
ARF3	0.739554728	3.20E-12	0.466176953	1.12E-05	0.543480448	3.07E-07
SGK1	1.793845931	4.34E-12	1.340174041	2.32E-07	1.357914486	1.63E-07
SDCBP2	1.210653999	4.79E-12	0.480299022	0.00629	1.018839133	6.21E-09

RHOG	0.8240319	6.26E-12	0.282044285	0.02138	0.508077	3.01E-05
DUOXA2	3.408401335	7.75E-12	2.491337444	5.72E-07	2.939429793	3.62E-09
MYO1B	0.780714645	8.83E-12	0.987148518	5.09E-18	0.310214831	0.00684
UNG	-1.124012539	9.25E-12	-0.506296037	0.00184	-0.584328716	0.00034
SNPH	3.512013436	1.02E-11	2.892940143	2.16E-08	3.101654282	1.93E-09
TUBB2A	1.046674747	1.43E-11	0.900377661	6.12E-09	0.845810282	5.05E-08
BCL3	1.747587301	1.70E-11	0.663174109	0.01159	1.396126912	8.49E-08
LITAF	1.246092133	1.90E-11	0.72614108	9.21E-05	0.45196029	0.01509
OAS3	1.043870529	2.42E-11	0.628798076	5.95E-05	0.786965862	5.11E-07
HRSP12	-0.920525472	2.53E-11	-0.203165824	0.11594	-0.524094141	8.22E-05
IL8	3.833444329	3.34E-11	3.644030892	2.91E-10	3.223127984	2.49E-08
MAD2L1	-0.916182482	4.68E-11	-0.280543852	0.04112	-0.738882265	9.89E-08
DEFB4A	5.643649266	5.57E-11	5.208241057	1.48E-09	4.671139723	6.18E-08
JAK1	0.770275454	5.84E-11	0.730199694	5.28E-10	0.590139577	5.33E-07
SRD5A3	1.220683742	6.87E-11	0.543102651	0.00381	1.098479647	4.40E-09
TOMM7	-0.74908048	6.90E-11	-0.078910178	0.48054	-0.325449047	0.00396
VRK1	-1.022921749	7.25E-11	-0.279136057	0.07026	-0.589118668	0.00015
CX3CL1	2.250984176	8.76E-11	2.129433112	8.25E-10	2.057218889	3.06E-09
IL19	5.591551302	9.81E-11	4.881354784	1.78E-08	5.008147328	7.63E-09
LYN	1.292225838	1.06E-10	1.366445235	7.88E-12	1.294902752	9.64E-11
TMEM261	-0.673174017	1.11E-10	-0.073809635	0.46736	-0.461845089	7.61E-06
SBNO2	1.883607379	1.13E-10	0.824335766	0.00524	0.481856509	0.10288
IFNGR1	1.129848143	1.42E-10	1.499901672	1.45E-17	1.138367258	1.02E-10
SRP9	-0.387462927	1.48E-10	0.106902423	0.07442	-0.207457114	0.00058
TNIP1	1.123604486	1.48E-10	0.64698799	0.00023	1.001929243	1.12E-08
TAPBP	0.793432194	1.66E-10	0.172888869	0.1648	0.655283798	1.33E-07
GDI1	0.686535517	1.73E-10	0.195463111	0.07039	0.278756691	0.01009
C3	2.128478	1.90E-10	1.991804248	2.52E-09	1.970481528	3.71E-09
MAGI1	-0.912158151	2.32E-10	-0.978421228	9.38E-12	-0.675196789	2.36E-06
EGR1	1.657152958	2.61E-10	0.408812403	0.12077	0.652340656	0.01324
ST5	0.989822237	2.93E-10	0.131412143	0.40568	0.612919174	0.0001
SRF	0.635346464	3.40E-10	0.238234044	0.01924	0.252376039	0.01351
WARS	0.873175691	3.50E-10	0.618856174	8.65E-06	0.48384187	0.00052
LTB	3.420563091	3.61E-10	4.164845757	1.17E-14	2.40345957	1.57E-05
NDUFA1	-0.571958584	3.66E-10	-0.05680589	0.51718	-0.142053152	0.10915
.P006216.1	-2.397634892	4.31E-10	-0.789084368	0.03434	-2.504415102	7.52E-11
KYNU	2.153901238	4.64E-10	2.471586366	8.50E-13	2.042011636	3.47E-09
CTPS2	-0.532610633	4.79E-10	-0.163242384	0.05333	-0.323429885	0.00015
NFKB2	1.601585115	4.87E-10	0.948149696	0.00024	1.137456842	1.03E-05
AHCYL2	1.36495043	4.98E-10	0.799870748	0.00028	1.353034367	7.04E-10
THBS1	1.803482362	5.33E-10	0.871861294	0.00269	0.382409854	0.18816
CYB5A	-0.832269799	5.47E-10	-0.331820821	0.01264	-0.220611795	0.09726
MCMDC2	-1.011402836	5.85E-10	0.311798412	0.03051	-0.908093247	1.68E-08
RPL36A	-1.122643901	6.83E-10	-0.216764206	0.23123	-0.668541463	0.00023
DAPK3	0.862217556	7.35E-10	0.35568423	0.01146	0.128189908	0.36666
SLC2A6	1.898462139	7.42E-10	1.266306501	4.68E-05	1.585661404	3.16E-07
CEBPD	2.034039059	7.73E-10	1.272567766	0.00012	1.254729815	0.00015

TNFRSF9	7.659960442	7.90E-10	7.94087172	1.76E-10	5.60535509	1.06E-05
PCNXL3	0.681966835	9.01E-10	-0.293847542	0.00951	0.377160068	0.00074
OSBPL1A	-0.65202699	9.71E-10	-0.325749235	0.00169	-0.240968592	0.0204
BIRC3	2.031788226	9.94E-10	1.92512654	6.94E-09	2.037402471	8.83E-10
PI3	4.514295293	1.04E-09	3.215553656	1.59E-05	2.881852173	0.00012
SPRR1A	5.042591801	1.05E-09	4.003183428	1.49E-06	3.596918401	1.75E-05
S100A3	4.154951994	1.15E-09	4.029482733	3.50E-09	2.47175039	0.00048
MAP3K11	0.693739125	1.27E-09	0.029937964	0.79585	0.346195695	0.00257
DHRS7	-0.803439786	1.30E-09	-0.371184814	0.00469	-0.201752397	0.12434
PCNA	-1.005738298	1.30E-09	-0.270477419	0.10056	-0.516608617	0.00175
TCEA1	-0.527456712	1.56E-09	-0.022422456	0.79512	-0.317766366	0.00026
PKHD1	-0.982112426	1.64E-09	-0.515141788	0.00113	-0.390281521	0.01361
ARHGEF1(	-0.906836507	1.76E-09	-0.805129579	5.78E-08	-0.820432355	3.89E-08
TRIM31	2.283307349	2.01E-09	0.895641065	0.01929	1.936334578	3.59E-07
OXTR	1.872062874	2.31E-09	2.124516386	8.63E-12	1.193008288	0.00017
ADAMTS1	0.818513386	2.43E-09	0.156536749	0.25621	0.20596668	0.13543
AKT1S1	0.712082873	2.41E-09	0.263558899	0.02855	0.476933637	7.08E-05
TD-2287O1(	-1.060655442	2.95E-09	-0.133922016	0.45028	-0.522256221	0.00333
CFLAR	0.893497947	3.09E-09	0.556564642	0.00022	0.77764878	2.50E-07
P11-274B2	1.14397746	3.28E-09	-0.296231678	0.14306	0.814082575	2.91E-05
SPRY4	0.857090204	3.44E-09	0.062572372	0.66753	0.219032013	0.13297
CRIM1	0.808057485	3.49E-09	0.519868782	0.00014	0.168089677	0.22141
XK	-1.307413552	3.52E-09	-0.619755803	0.00481	-0.20710306	0.34539
ZFP36L1	0.80995857	3.58E-09	0.189846962	0.16748	0.648845961	2.29E-06
RBMS2	0.831072823	4.02E-09	0.468602922	0.00095	0.179917011	0.20604
SMIM14	-0.920356608	4.72E-09	-0.17901044	0.25283	-0.419931497	0.00736
TFPI	0.857527696	5.05E-09	0.819913282	2.28E-08	0.411931463	0.00501
MANEA	-1.043933113	5.16E-09	-0.135092186	0.44065	-0.864931408	1.08E-06
HMGCS2	-2.145084551	5.81E-09	-1.171739045	0.00143	-1.549183602	2.54E-05
PTAFR	2.08269672	5.89E-09	1.528362359	2.00E-05	1.292949825	0.00032
SLC37A1	0.778740834	6.32E-09	0.519449843	0.00011	0.918361482	6.82E-12
EBPL	-0.666519522	6.68E-09	0.006087077	0.95561	-0.552730796	1.13E-06
PIEZO1	1.161804005	7.16E-09	0.381246773	0.05817	0.331782344	0.09948
ZSWIM8	0.848846927	7.16E-09	0.075420673	0.60918	0.394385008	0.00739
TXNDC16	-0.924801854	7.67E-09	-0.156708075	0.30974	-0.361783432	0.02013
PRPS2	-1.128194735	7.89E-09	-0.117249042	0.54624	-0.488376009	0.01213
AKR7A2	-0.537260994	8.57E-09	-0.184562036	0.04071	-0.209027326	0.02181
RAC1	0.668410322	9.92E-09	0.676153397	6.53E-09	0.600627854	2.59E-07
PAPSS1	-0.505658916	1.03E-08	-0.076159242	0.38083	-0.290046651	0.00092
SMAD3	1.12838624	1.01E-08	0.372724053	0.05884	0.466482483	0.01811
ZDHHC20	-0.511427155	1.02E-08	-0.212528522	0.01685	-0.190230134	0.03256
CTDSPL	-0.558971646	1.08E-08	-0.138319911	0.15189	-0.302840322	0.00182
ARHGAP18	-0.73930156	1.12E-08	-0.231517652	0.07116	-0.235378538	0.06713
ABCD3	-0.513848332	1.22E-08	0.003631165	0.96759	-0.097668652	0.27566
NUAK2	1.931246631	1.23E-08	0.91165196	0.00847	0.726624957	0.03807
ACKR3	1.209052734	1.29E-08	0.631360243	0.00309	1.025715531	1.42E-06
GPD1L	-0.86539446	1.46E-08	-0.716056066	2.28E-06	-0.128520287	0.39189

OLR1	2.021675274	1.51E-08	1.30486254	0.00027	0.419320203	0.2489
PEA15	0.740141492	1.67E-08	0.432025279	0.001	0.380694834	0.00382
ACADM	-0.523580814	1.72E-08	-0.031232506	0.72979	-0.096087283	0.29208
CXCL16	1.08631005	1.92E-08	1.181697831	9.18E-10	0.875816403	6.06E-06
MAP7D2	2.044438195	1.96E-08	1.825494679	5.32E-07	1.720293396	2.40E-06
CSNK1G2	0.800056244	2.02E-08	0.437192919	0.00219	0.474112654	0.00092
DSE	1.190835587	2.01E-08	0.733012273	0.00056	0.877795387	3.57E-05
TNFAIP1	0.758281013	2.01E-08	0.38615197	0.0043	0.32684981	0.01594
CDC23	-0.482935901	2.18E-08	0.06288155	0.45349	-0.301708989	0.00041
SGPP2	1.44730145	2.17E-08	1.064843253	3.87E-05	1.280389401	7.43E-07
DNA2	-0.987236533	2.20E-08	-0.25223125	0.14653	-0.809292514	4.04E-06
LRBA	-0.495041609	2.28E-08	-0.172837849	0.05011	-0.136738001	0.12139
TBCA	-0.564954188	2.32E-08	-0.032117963	0.74633	-0.429329882	2.00E-05
RND1	2.639282658	2.57E-08	1.792070134	0.00016	1.949267783	4.09E-05
MCM3	-0.676708143	2.75E-08	-0.228342001	0.05965	-0.368148909	0.00244
VNN1	2.340496975	3.18E-08	2.22110991	1.45E-07	1.033519514	0.02007
SCCPDH	-0.628845452	3.22E-08	-0.050784177	0.64718	-0.134647498	0.22775
PTPN1	0.434070669	3.35E-08	0.103233778	0.19203	0.024826626	0.75598
SDC4	1.32910536	3.35E-08	1.061377174	1.04E-05	1.311865868	5.02E-08
ASAH1	-0.585421283	3.49E-08	-0.030827662	0.76927	0.058359423	0.57912
TOMM70A	-0.492287517	3.87E-08	-0.003662349	0.96699	-0.201304291	0.02366
LRG1	1.271465963	3.98E-08	1.029662895	8.63E-06	1.083370906	2.92E-06
SH3BGRL2	-0.798155847	4.28E-08	-0.270417042	0.06265	-0.113399388	0.43496
TMEM181	-0.586624764	4.47E-08	-0.340333392	0.00145	-0.076149639	0.47573
RHBDF1	0.925268543	4.75E-08	0.320405579	0.06061	0.209765502	0.21942
CCL2	2.782813523	4.84E-08	1.895068209	0.0002	1.553269129	0.00234
MAP3K8	1.356221998	5.00E-08	1.37738799	2.92E-08	1.57017301	2.61E-10
AASDHPPT	-0.539255337	5.08E-08	0.285743122	0.00335	-0.391806394	7.16E-05
TP53INP2	1.496945909	5.10E-08	0.034192922	0.90463	0.925360222	0.00087
CXCL11	5.853814455	5.15E-08	6.163437607	9.04E-09	4.127453574	0.00016
KLF6	0.914796385	5.50E-08	0.476874978	0.00461	0.625622042	0.0002
TSPAN18	1.42294795	6.09E-08	0.547564875	0.04029	0.757529748	0.00444
EIF4BP7	-1.347281078	6.85E-08	-0.069254096	0.76926	-0.461293569	0.05422
GPR153	0.957383812	7.21E-08	0.194876314	0.28267	0.403035949	0.02586
TNFRSF1A	0.633545417	7.50E-08	0.215642934	0.06751	0.280609758	0.01757
HDAC9	1.619039692	7.60E-08	1.265311035	2.66E-05	1.495068001	6.93E-07
ID1	1.168442558	8.01E-08	0.779302209	0.00035	0.97288087	7.93E-06
MYO1C	0.578392991	8.36E-08	-0.090223369	0.40441	0.442253145	4.29E-05
CAPS	-1.251932187	9.13E-08	-0.959080516	4.00E-05	-0.248924736	0.28297
BRCA1	-0.872049436	1.01E-07	-0.254833526	0.11468	-0.281091428	0.08285
PTPRE	1.17733911	1.02E-07	0.7171128	0.0012	0.932139516	2.56E-05
SCOC	-0.714552048	1.09E-07	-0.172820712	0.19592	-0.160095105	0.2312
GYS1	0.58303835	1.12E-07	-0.045234484	0.68254	0.33606923	0.00233
B4GALT1	1.048442096	1.16E-07	0.762820427	0.00012	0.840472068	2.17E-05
RUFY1	-0.535723893	1.21E-07	-0.308129355	0.002	-0.231481278	0.02077
RPL21	-0.633187951	1.22E-07	0.085995882	0.47123	-0.30108637	0.01177
AKAP2	1.18526146	1.25E-07	0.49604652	0.02707	0.017765785	0.93701

GEMIN6	-0.709914204	1.26E-07	0.150239158	0.2481	-0.295997666	0.02588
USP1	-0.49615332	1.35E-07	0.036940546	0.69114	-0.173139944	0.06393
SPRR2F	4.053799865	1.43E-07	3.389883564	1.16E-05	3.103809161	6.34E-05
HMGN3	-0.687799968	1.45E-07	-0.053921746	0.67614	-0.330096418	0.01101
IVNS1ABP	-0.423326084	1.48E-07	-0.157508025	0.04893	-0.186699783	0.01997
IL6	3.511151041	1.51E-07	1.712122322	0.01179	1.922490079	0.00452
NID1	0.881937449	1.50E-07	-0.120061474	0.4756	0.247300768	0.14141
POLA1	-0.67444416	1.50E-07	-0.269321178	0.03464	-0.346876403	0.00662
NFKBIB	1.038855762	1.52E-07	0.364207113	0.06871	0.705815746	0.00039
MCM4	-0.69462919	1.60E-07	-0.218980754	0.09728	-0.251342628	0.05727
GALNT12	-0.934885043	1.63E-07	-0.537227285	0.00237	-0.094812727	0.59063
STOM	0.976999974	1.69E-07	0.884283265	2.19E-06	0.198113351	0.29033
SPCS1	-0.41677615	1.79E-07	0.132507966	0.08615	-0.212563664	0.00698
PLAGL1	0.854843779	2.01E-07	0.298711806	0.07092	0.337367514	0.04133
BLOC1S5	-0.714605911	2.24E-07	-0.320628706	0.01834	-0.325840691	0.01706
ALG6	-0.533303869	2.29E-07	-0.085193528	0.37667	-0.311902249	0.00169
CREG1	-0.655728071	2.36E-07	-0.044368192	0.72239	-0.257125138	0.04068
CSNK1E	0.448218471	2.36E-07	-0.032284315	0.71086	0.046023826	0.59775
ADAMTS9	1.190503262	2.54E-07	0.658612204	0.00436	0.38497281	0.09596
TMEM64	-0.618296935	2.53E-07	-0.195154509	0.0996	-0.142345914	0.23126
FAM46C	-1.750895847	2.60E-07	-0.639417175	0.0577	-0.656876086	0.05137
VPS35	-0.468363447	2.76E-07	-0.133618054	0.14045	-0.060122213	0.50801
UBQLN4	0.640123181	2.97E-07	0.511513661	4.15E-05	0.714945224	9.89E-09
PRMT3	-0.695099075	3.04E-07	0.053278927	0.68434	-0.239551126	0.0719
SCO1	-0.562437887	3.04E-07	0.042402167	0.69608	-0.270102871	0.01328
WDHD1	-0.8036096	3.09E-07	-0.186004291	0.2309	-0.401634208	0.01009
MUC13	6.398486039	3.23E-07	6.072160044	1.25E-06	6.223505083	6.73E-07
COX6C	-0.482592484	3.36E-07	-0.034092371	0.71313	-0.272983805	0.00358
BID	1.121048	3.59E-07	1.120538336	3.42E-07	0.658957302	0.00281
UBL3	-0.582110744	3.72E-07	-0.448317652	8.10E-05	-0.120944574	0.28607
CCDC25	-0.628812016	3.91E-07	-0.041781025	0.7327	-0.381374766	0.00197
CUL4B	-0.522396839	4.01E-07	-0.174506179	0.08815	-0.258939394	0.01162
P11-384K6	1.179112045	4.02E-07	-0.227336956	0.35299	0.972602556	3.17E-05
CLUHP3	0.785496306	4.05E-07	0.084693083	0.58969	0.787386812	3.50E-07
ITGA5	0.889990115	4.08E-07	0.542829293	0.00202	0.127756618	0.46893
LIG1	-0.714199386	4.07E-07	-0.777421236	2.97E-08	-0.672036528	1.61E-06
MRPL33	-0.503374954	4.25E-07	-0.169791211	0.07642	-0.14825466	0.12475
SC5D	0.574205717	4.50E-07	0.495350532	1.31E-05	0.614493722	6.43E-08
NIPSNAP3/	-0.536128249	4.59E-07	-0.039449661	0.69277	-0.075198611	0.45851
CABLES1	-1.234910499	4.71E-07	-0.512544732	0.03268	-0.610420462	0.01148
FBXL4	-0.545370296	4.70E-07	-0.332039353	0.00192	-0.128500394	0.23
PPIL1	-0.548659241	4.96E-07	0.195992208	0.06682	-0.205412868	0.05735
SNX7	-0.507076038	5.01E-07	0.013667657	0.88897	-0.070851685	0.47308
EEF1A1P9	-0.916693706	5.08E-07	0.506479686	0.00208	-0.84227705	3.05E-06
ENPP1	1.107080551	5.45E-07	1.782963302	4.19E-16	0.970234568	1.15E-05
ANKRD33E	3.341831341	5.51E-07	3.261522697	9.62E-07	3.008898035	8.79E-06
SUMF1	-0.529263909	5.59E-07	-0.135799713	0.19162	-0.117507613	0.26028

C1GALT1C	-0.669930627	5.63E-07	-0.170618341	0.19461	-0.044782611	0.73361
EEF1A1	-0.591335907	5.67E-07	0.096081994	0.41632	-0.334080025	0.00471
APIP	-0.551225877	5.71E-07	-0.136126602	0.2036	-0.253780341	0.01876
NSMF	0.878847173	5.84E-07	0.659946286	0.00019	0.745606652	2.48E-05
PCGF3	0.407124359	5.85E-07	0.196379427	0.01589	0.348626037	1.94E-05
NKIRAS2	0.378858071	6.01E-07	-0.017637755	0.81795	0.156905277	0.04056
MAT2B	-0.421227378	6.29E-07	0.025869339	0.75447	-0.194958238	0.01974
IARS2	-0.473342785	6.46E-07	-0.032925937	0.72713	-0.047182567	0.61772
SLCO3A1	1.504914907	6.50E-07	0.774635729	0.01145	1.214760288	6.53E-05
C1S	0.97412808	6.53E-07	0.585072017	0.00281	0.376458612	0.05531
DSCC1	-1.091980736	6.93E-07	-0.375220831	0.07649	-0.594868808	0.00551
NADK2	-0.499677664	7.30E-07	0.033378915	0.73341	-0.158631129	0.10932
RNF207	0.8932138	7.28E-07	0.138861802	0.44735	0.503170145	0.00552
CCDC130	0.513670429	7.37E-07	0.027064954	0.79808	0.367295336	0.00043
ZYX	0.946462435	7.50E-07	0.011960552	0.95038	0.052672557	0.78401
CSF1	1.24039724	7.59E-07	0.599220613	0.01735	0.482371087	0.05594
TSFM	-0.576696808	8.04E-07	0.138280665	0.21154	-0.172999401	0.12801
JUND	0.701981327	8.13E-07	0.040033366	0.77987	0.56329084	7.70E-05
SLC25A44	0.625078986	8.11E-07	0.47420204	0.00019	0.604177195	1.91E-06
ALDH9A1	-0.546783513	8.53E-07	-0.170666473	0.11946	-0.2562628	0.01994
TSPAN13	-0.561188496	8.54E-07	-0.240393381	0.03391	0.040094657	0.72318
MRPS18B	-0.466576021	8.81E-07	-0.074563091	0.41956	-0.017023242	0.8545
DUOX2	2.318599347	8.90E-07	1.200643304	0.01095	2.05346041	1.35E-05
SUCLG2	-0.577784514	9.75E-07	-0.143209739	0.22225	-0.103968582	0.37593
IGFBP3	1.811827051	1.01E-06	0.918917547	0.01316	0.166938198	0.65255
GABPB2	-0.676590344	1.06E-06	-0.38998443	0.00492	-0.310722829	0.02371
ZNF330	-0.516474086	1.07E-06	-0.03576986	0.72724	-0.058061247	0.57548
SCIN	-1.438482694	1.08E-06	-0.517004372	0.07865	-0.738049189	0.01213
RPIA	-0.653891471	1.10E-06	-0.156798528	0.22015	-0.231814066	0.07359
OXR1	-0.594548976	1.13E-06	-0.287828668	0.0176	-0.202551013	0.09559
RFC3	-0.767587385	1.13E-06	-0.182711168	0.23795	-0.588958493	0.00017
RPL39	-0.601378097	1.13E-06	0.250251163	0.04238	-0.311293685	0.01168
AP2A1	0.516328055	1.16E-06	-0.095547343	0.37136	0.155673488	0.14531
FAM83A	-0.773166667	1.17E-06	0.299578026	0.059	-0.291504858	0.06648
MFSD6L	-1.228724965	1.21E-06	-0.535555642	0.02658	-0.363978065	0.13126
FAM213A	-0.511037054	1.25E-06	-0.102559124	0.32142	-0.316286336	0.00246
ATP10B	1.224474043	1.26E-06	0.703554968	0.00539	1.002171561	7.32E-05
NUDT21	-0.321790268	1.30E-06	0.185513618	0.00482	-0.197837472	0.00284
MAGOHB	-0.621730678	1.31E-06	-0.065256076	0.60408	-0.614308401	1.76E-06
ETV3	0.567853672	1.33E-06	0.445888942	0.00014	0.612296795	1.79E-07
PIK3C2G	-1.333581847	1.35E-06	0.190126455	0.48859	-0.383310093	0.16322
GLP1R	-2.575182931	1.37E-06	-1.09988236	0.02244	-1.163861199	0.01654
RAP1B	0.408723317	1.40E-06	0.603644721	8.61E-13	0.291895223	0.00057
CFB	1.101375117	1.41E-06	1.245897236	4.73E-08	0.740467721	0.00119
EIF4A2	-0.435320321	1.44E-06	0.103000587	0.25309	-0.138475182	0.12483
SCP2	-0.510171822	1.47E-06	-0.261716026	0.01294	-0.12870895	0.22158
TMEM168	-0.639837172	1.48E-06	-0.325440565	0.01351	-0.068630415	0.60249

TMEM167A	-0.647614567	1.48E-06	-0.086083247	0.52125	-0.493094533	0.00024
ESD	-0.428521913	1.51E-06	0.089404602	0.30857	-0.384380562	1.55E-05
H3F3A	-0.475975491	1.52E-06	-0.030483001	0.75744	-0.308506185	0.00181
IFITM3	0.66870278	1.52E-06	0.424430103	0.00228	0.24080553	0.08384
DPY30	-0.545066693	1.55E-06	0.072455798	0.5084	-0.42020004	0.0002
ATP6V0A1	0.642880587	1.60E-06	0.002721256	0.98391	0.594841819	9.32E-06
TAP1	1.024852498	1.69E-06	1.029097311	1.43E-06	0.682273998	0.00153
ITGA1	0.942835069	1.76E-06	0.66008459	0.00082	-0.226231519	0.2529
NACC1	0.941533591	1.80E-06	0.191820244	0.33141	0.593933421	0.00261
OSMR	1.160395242	1.80E-06	0.887811453	0.00026	0.614543708	0.01156
SLC39A11	-0.520249949	1.79E-06	-0.313442605	0.00335	-0.098823273	0.35369
CDR1	2.071113333	1.81E-06	0.276974156	0.5383	0.916760641	0.03855
PHIP	-0.371507937	1.85E-06	-0.085592968	0.26903	-0.197828276	0.01086
PYURF	-0.501004238	1.86E-06	0.130735624	0.19667	-0.314504997	0.00244
TMEM14C	-0.453898719	1.89E-06	0.187768684	0.04193	-0.266303319	0.00469
POLR2G	-0.578711815	2.05E-06	0.078468328	0.50838	-0.306488162	0.01097
SMIM7	-0.408783085	2.05E-06	-0.142558599	0.09351	-0.144319467	0.09054
AFM	-3.08488267	2.13E-06	-0.163134435	0.78553	-1.382384642	0.02366
KBTBD7	-0.50166227	2.17E-06	-0.337706735	0.00114	-0.366140958	0.00047
STAT6	0.433542481	2.17E-06	-0.094219799	0.30462	0.306650077	0.00082
C8orf4	1.847978081	2.22E-06	1.579948276	5.24E-05	0.650640256	0.0979
CAPG	-0.57596398	2.30E-06	-0.338346781	0.00531	-0.067176044	0.5789
MED15	0.766315889	2.31E-06	-0.05456016	0.73851	0.35459477	0.02936
C1R	0.936266324	2.32E-06	0.578896246	0.00351	0.354412527	0.07462
PRCP	-0.406951245	2.35E-06	0.175764295	0.03581	-0.171912025	0.04351
CSF3	4.026403194	2.38E-06	3.785282167	9.15E-06	4.739803389	2.57E-08
MTAP	-0.510253785	2.42E-06	-0.142249876	0.18477	-0.36239276	0.00078
TRIOBP	0.422513589	2.45E-06	0.150326521	0.09454	0.350903902	9.18E-05
CFL2	0.745229846	2.47E-06	0.834782046	1.22E-07	0.267453415	0.09393
PARP14	0.646243065	2.47E-06	0.807366471	3.61E-09	0.3894101	0.00459
PPARD	0.75801454	2.54E-06	0.181252985	0.26573	0.287732378	0.07743
TMEM107	-0.638754916	2.54E-06	0.142427099	0.26733	-0.269016743	0.0414
ZMIZ2	0.674559555	2.66E-06	-0.590951911	5.36E-05	0.285768951	0.04776
SERPINB8	1.177067863	2.70E-06	0.804321567	0.00139	0.92355605	0.00024
ZNF367	-1.104576565	2.79E-06	-0.172470583	0.45363	-0.537491977	0.02061
POGZ	0.580964174	2.81E-06	-0.049211207	0.69288	0.52356756	2.39E-05
CREB1	0.642529691	2.84E-06	0.364639018	0.00781	0.424212638	0.00201
SLC30A1	-0.577339325	2.90E-06	-0.157200439	0.19818	-0.180665969	0.14012
RIMBP3C	-0.723429782	2.91E-06	-0.279151614	0.07101	-0.743708622	1.52E-06
NACAD	1.588655262	2.92E-06	0.080489182	0.82052	0.232886154	0.51119
FEZ1	1.343141834	2.98E-06	1.525516631	9.99E-08	0.973303414	0.00073
AB019441.2	-1.518951105	3.01E-06	-0.16054906	0.56415	-1.495860774	3.44E-06
RNF19A	1.124616064	3.04E-06	1.123979958	2.55E-06	0.95066514	8.51E-05
STAP2	0.752695027	3.13E-06	0.888870108	3.35E-08	0.856144506	1.10E-07
RFC5	-0.66527524	3.17E-06	-0.287908526	0.03985	-0.579386567	4.26E-05
LIMD2	1.058881159	3.21E-06	0.401030921	0.07925	0.261284061	0.25528
IL3RA	1.538833057	3.22E-06	0.819845676	0.01571	0.434399081	0.22028

EEF1E1	-0.692621497	3.30E-06	-0.016575355	0.90966	-0.227324265	0.12236
SS18	0.45833907	3.36E-06	0.220254761	0.02521	0.244428494	0.01336
FAM219A	0.893062176	3.38E-06	0.342039993	0.07715	0.406976359	0.03579
COMT	-0.741010438	3.48E-06	-0.525648238	0.00093	-0.274220463	0.08388
GSTA4	-0.555520919	3.52E-06	-0.230228268	0.05057	-0.331361281	0.00527
HAUS4	-0.607047145	3.55E-06	-0.197570505	0.12055	-0.212949549	0.09843
WBP2	0.847702033	3.58E-06	0.257527885	0.16089	0.659366436	0.00032
SAT1	1.193562608	3.61E-06	1.180184176	4.62E-06	1.080675661	2.73E-05
MESDC1	0.600541126	3.80E-06	0.086683199	0.51064	0.358363188	0.00622
RBPMS	0.593155037	3.81E-06	0.128150922	0.32005	0.289006075	0.02488
C14orf159	-0.642535192	3.87E-06	-0.616725575	7.76E-06	0.055783778	0.6821
CHEK2	-0.627964942	3.92E-06	-0.24500084	0.06369	-0.393050402	0.00338
TYMP	1.14852637	3.92E-06	0.377055833	0.13719	0.859671523	0.0006
HSDL2	-0.59458585	3.96E-06	0.13596197	0.28708	-0.132116222	0.30246
INSR	0.697254202	4.05E-06	-0.072765203	0.63155	0.424872888	0.00501
MACC1	-0.624377025	4.04E-06	-0.136439134	0.31252	-0.023458513	0.86214
MGRN1	0.788461206	4.12E-06	0.100500435	0.56015	0.274766938	0.11007
RPS21	-0.677511455	4.18E-06	0.040169322	0.78365	-0.251038404	0.08697
SH3BGRL3	0.807600037	4.26E-06	0.37883592	0.03124	0.448763389	0.01073
FAM136A	-0.579788571	4.46E-06	-0.005737544	0.96328	-0.17016274	0.17403
PDCD4	-0.875971652	4.49E-06	-0.112209806	0.55594	-0.053883332	0.77738
TFAM	-0.601365478	4.52E-06	0.048063152	0.71185	-0.22285756	0.08784
ALDH3A2	-0.801645623	4.56E-06	-0.488514594	0.00506	-0.418156551	0.01649
OSGEP	-0.458990413	4.83E-06	-0.195440066	0.04726	-0.252343072	0.01088
TMEM127	0.427735882	5.01E-06	-0.116779936	0.21697	0.300949011	0.00134
DLGAP4	0.778878219	5.11E-06	0.026089524	0.88008	0.21257252	0.21619
ORC1	-0.81943997	5.14E-06	-0.310005367	0.07945	-0.484605554	0.00645
RNF24	0.801926715	5.25E-06	0.325146317	0.06575	0.240305579	0.17424
ENDOD1	-0.582611444	5.34E-06	-0.026451908	0.83392	-0.403278041	0.00154
PSMG2	-0.393065606	5.35E-06	0.230480459	0.00513	-0.194210684	0.02222
KCTD10	0.516045788	5.57E-06	0.431049209	0.00014	-0.052177924	0.64791
PUS7L	-0.596682157	5.58E-06	0.009105627	0.94439	-0.48880362	0.00019
GLTP	0.472884376	5.66E-06	0.41153983	7.08E-05	0.503446459	1.40E-06
SLC35A1	-0.466040654	5.67E-06	-0.062634126	0.53191	-0.021355246	0.83185
IL4R	0.708990067	5.73E-06	0.04225398	0.78715	0.360988096	0.02094
CSNK2B	0.497334108	5.79E-06	0.459438169	2.72E-05	0.439002671	6.29E-05
HIF1A	0.623237901	5.84E-06	0.720857717	1.56E-07	0.308562656	0.02494
DRAM1	0.855125061	5.94E-06	0.908269733	1.43E-06	0.498777377	0.00839
MCM6	-0.781239796	5.99E-06	-0.243057483	0.15646	-0.413710784	0.01623
CREB3L4	-0.956096274	6.12E-06	-0.405755521	0.04672	-0.735679993	0.00038
PTPMT1	-0.511955301	6.38E-06	0.043154919	0.6988	-0.285892591	0.01115
ZZZ3	-0.417207916	6.36E-06	-0.075626375	0.40735	-0.204971155	0.02566
DECR1	-0.44974227	6.43E-06	-0.048183078	0.62049	-0.145781144	0.13771
DCLRE1A	-0.681369191	6.56E-06	0.092179589	0.53164	-0.183881354	0.21647
SNX2	-0.532206863	6.56E-06	-0.002528542	0.98276	-0.215099266	0.06697
EEF1A1P1	-1.335905287	6.61E-06	-0.022494537	0.93067	-0.623639358	0.0224
HDGF	0.535215328	6.65E-06	0.735660395	5.70E-10	0.497431034	2.83E-05

GLRX	0.792560974	6.70E-06	0.902253125	2.43E-07	0.298954689	0.09401
WWC3	0.725905219	6.80E-06	0.392088176	0.01523	0.164175444	0.31183
DPY19L4	-0.639521215	6.95E-06	-0.095432658	0.5001	-0.342268091	0.01573
AGR2	-0.802562411	7.10E-06	-0.165763896	0.35354	-0.164885429	0.35611
DEPTOR	-1.056353226	7.10E-06	-0.873258787	0.0002	-0.431301415	0.06603
FABP6	5.712007943	7.24E-06	6.226773807	9.94E-07	0.401191353	0.76014
TMEM230	-0.303291935	7.24E-06	0.182293181	0.00547	-0.132588754	0.04698
WIZ	0.57003278	7.20E-06	-0.227688676	0.08005	0.167114888	0.19373
PFKM	-0.642922254	7.52E-06	-0.251622132	0.07753	-0.511786438	0.00035
SYNPR	-2.187842129	7.55E-06	-1.062734707	0.02831	-1.010075288	0.03723
TFE3	0.644136971	7.72E-06	-0.016380324	0.91005	0.30282005	0.03625
TBC1D10B	0.40914729	7.74E-06	-0.369474567	8.77E-05	0.24805285	0.00695
CIC	0.690142859	7.77E-06	-0.431750444	0.00559	0.133956513	0.38721
SAMD4A	1.089802622	7.98E-06	0.52799502	0.03129	0.455002781	0.06329
RAD51AP1	-0.815631332	8.08E-06	-0.298887419	0.096	-0.58529136	0.00122
CTTNBP2N	0.495991709	8.16E-06	0.188452943	0.08987	0.324942248	0.00356
RCL1	-0.867430004	8.20E-06	0.012057867	0.94889	-0.193709499	0.30962
CDCA7L	-0.826756645	8.23E-06	-0.348089768	0.05869	-0.639470648	0.00054
SESN1	-0.890617201	8.34E-06	-0.490259858	0.01346	-0.434053474	0.02912
TBC1D5	-0.46012424	8.33E-06	-0.246523442	0.01606	-0.341421998	0.00093
EBI3	1.986774319	8.44E-06	1.415220014	0.00164	0.871121582	0.05879
CCDC85A	1.947054685	8.54E-06	1.447209892	0.00102	0.300050916	0.51695
FNDC3B	0.789140483	8.57E-06	0.448320822	0.01147	0.560024375	0.00159
IRAK2	1.609667509	8.68E-06	1.101931221	0.00241	1.327087046	0.00025
ORC3	-0.384414766	8.83E-06	0.029188018	0.72694	-0.30375202	0.00041
SF3A2	0.528615819	8.91E-06	-0.336988208	0.00627	0.255851566	0.03264
DHX40	-0.472555001	9.11E-06	0.09494488	0.36703	-0.209213414	0.04867
P11-407G23	1.12221621	9.12E-06	0.935953406	0.00022	0.537553504	0.04013
FAM213B	0.556245936	9.37E-06	0.152946118	0.22896	0.45795043	0.00028
SCML4	-1.787517969	9.43E-06	-0.932145358	0.01701	-0.568195036	0.14558
NUP37	-0.502960889	9.53E-06	0.237022589	0.02852	-0.421172782	0.00018
PEX2	-0.393047867	9.64E-06	0.193560035	0.02563	-0.113501794	0.19731
HMGN2P46	1.074211746	9.95E-06	0.136112737	0.58404	1.029335051	2.45E-05
ATXN2L	0.67124505	1.01E-05	-0.503463895	0.0011	0.457021932	0.00272
DUOXA1	2.015210504	1.03E-05	1.330429047	0.00392	1.934763184	2.32E-05
ERI1	-0.518837503	1.02E-05	-0.153893999	0.18364	-0.121664298	0.29954
SF3B4	0.452292628	1.02E-05	0.120638222	0.24228	0.315673098	0.00209
TMEM87A	-0.477370911	1.02E-05	-0.12276945	0.25145	-0.084054702	0.43385
ASUN	-0.634810094	1.03E-05	0.222454652	0.1173	-0.171090842	0.233
GRAMD1B	-1.349987836	1.03E-05	-1.699631502	2.48E-08	-0.53495408	0.07538
EFNA2	1.101850961	1.04E-05	0.447525278	0.08056	0.853054153	0.00072
ATP5I	-0.457604045	1.06E-05	0.084581125	0.40608	0.040724974	0.69067
ADAT2	-0.826156412	1.14E-05	-0.447105829	0.0155	-0.394395256	0.03377
CRISPLD2	1.276438209	1.13E-05	0.418213276	0.15135	0.160159506	0.58381
RDX	0.564529062	1.17E-05	0.49209978	0.00013	0.3186659	0.01351
TEX9	-0.758003691	1.17E-05	-0.098857289	0.5614	-0.293893909	0.08642
BCAT2	-0.720079014	1.18E-05	-0.411715649	0.01082	-0.572066994	0.00046

MYADM	0.659512254	1.19E-05	-0.172710047	0.25543	0.421412723	0.0053
BCL2L1	0.717145679	1.21E-05	0.56084495	0.00062	0.196407722	0.23185
ANO5	-0.874728283	1.22E-05	-0.579803948	0.00307	-0.490613531	0.01236
GK	1.320617427	1.22E-05	1.216706369	5.55E-05	0.834172229	0.00585
HLTF	-0.648660966	1.22E-05	0.071072147	0.62909	-0.325198936	0.02778
WDR74	-0.815607153	1.22E-05	-0.031463218	0.86596	-0.566897172	0.00236
IL18R1	1.058961584	1.25E-05	0.784772047	0.0012	0.823370385	0.00069
KRTCAP3	-0.842478399	1.26E-05	-0.121345323	0.51422	-0.322711031	0.08555
VCAM1	2.716676685	1.28E-05	1.86371229	0.00276	1.331633716	0.03256
NEURL3	3.08237699	1.29E-05	3.104032957	1.03E-05	2.961683629	2.93E-05
SH3GL1	0.470639724	1.30E-05	-0.002813101	0.97932	0.165995984	0.12581
BDKRB2	1.966028203	1.31E-05	1.360487415	0.00262	0.44812044	0.32668
ELK1	0.559827022	1.31E-05	0.213283927	0.09988	0.247728151	0.05629
MANBA	-0.748573885	1.31E-05	-0.248865936	0.14489	-0.331314269	0.05286
PCDHGB6	0.841864418	1.34E-05	0.085071542	0.6644	-0.159114811	0.42306
SLC25A46	-0.370897711	1.35E-05	-0.029332647	0.72817	-0.107982633	0.20277
ADRBK2	0.940355525	1.37E-05	0.773503278	0.00035	0.593915719	0.00612
SP3	-0.263245013	1.40E-05	0.062311061	0.29918	-0.01599279	0.79061
LRRC40	-0.35918451	1.41E-05	0.081263929	0.30672	-0.332081926	5.42E-05
IARS	-0.411740295	1.43E-05	0.021652018	0.81893	-0.16224433	0.08674
SPATA2	0.711469178	1.45E-05	0.192470761	0.25145	0.618786135	0.00017
CENPU	-0.81379286	1.48E-05	-0.280191581	0.1298	-0.532910349	0.00426
PITHD1	-0.433491482	1.49E-05	-0.113323959	0.24655	-0.095568286	0.33089
THUMPD2	-0.635125596	1.50E-05	0.032458748	0.8201	-0.393681613	0.00675
CCSAP	-0.366574525	1.51E-05	-0.065016585	0.42549	-0.307939732	0.00025
WSB2	0.469730425	1.51E-05	0.145393904	0.18034	0.434389321	6.28E-05
NUP133	-0.402242655	1.53E-05	0.003217911	0.97199	-0.306394942	0.00093
AEBP1	0.982687828	1.54E-05	-0.071473384	0.75406	0.022805292	0.92055
AGL	-0.362374538	1.54E-05	-0.028112155	0.73395	-0.130104898	0.11749
CENPH	-0.78888077	1.58E-05	-0.036489854	0.83619	-0.370736155	0.03924
GALNT7	-0.576408669	1.58E-05	-0.162486917	0.22136	-0.142169322	0.28509
SOCS3	1.668618432	1.58E-05	0.749999002	0.05258	0.426644108	0.27074
RGP1	0.408426289	1.59E-05	0.133242379	0.16017	0.317509619	0.00081
P4-669L17	1.363190427	1.59E-05	-0.150410066	0.64845	1.25847925	6.91E-05
GXYLT2	1.128849132	1.62E-05	1.114869706	1.97E-05	0.729116163	0.00569
TGDS	-0.559829838	1.63E-05	0.029726877	0.81028	-0.055187191	0.66128
DMXL1	0.498706906	1.65E-05	0.393143246	0.00067	0.561358246	1.20E-06
NR0B2	-1.315513801	1.65E-05	-0.915892664	0.00254	-0.622177537	0.04008
RNASEH2A	-0.570721234	1.66E-05	-0.326130987	0.01184	-0.30833424	0.01792
WIPI1	0.553326948	1.66E-05	0.384528393	0.0027	0.42357802	0.00099
PXK	0.529359317	1.67E-05	0.310669286	0.01148	0.218909176	0.07861
PCCA	-0.662618344	1.68E-05	0.131714366	0.38492	-0.206267649	0.17719
UBE2E2	0.607610216	1.68E-05	0.548793919	8.87E-05	0.579596609	3.78E-05
RPL9	-0.681423846	1.69E-05	0.20090642	0.20421	-0.36060779	0.02275
WISP1	1.237188332	1.72E-05	0.494283235	0.0864	0.103147373	0.72154
CADPS2	-0.962185794	1.75E-05	-0.191647035	0.38749	-0.447534806	0.04432
FAHD2CP	-1.244879343	1.76E-05	-0.785839481	0.00498	-0.784439943	0.00523

PDE8A	0.599771442	1.77E-05	0.670108417	1.52E-06	0.648626409	3.31E-06
ITGAV	0.751600463	1.79E-05	0.791027248	6.30E-06	0.397066245	0.02346
RRM1	-0.52779715	1.79E-05	0.009954933	0.93501	-0.316413746	0.00983
KDELC2	-0.530550771	1.82E-05	-0.377032481	0.00219	-0.406159503	0.001
GABARAPL	0.84629437	1.83E-05	0.425467802	0.03142	-0.036611322	0.85425
VCL	0.467314153	1.83E-05	0.085014099	0.43588	0.122378179	0.26232
DBT	-0.53654859	1.90E-05	0.139619701	0.26255	-0.085120831	0.4956
DSN1	-0.684842837	1.90E-05	-0.351514114	0.02543	-0.39014835	0.014
NMD3	-0.392355201	1.93E-05	0.102395963	0.25712	-0.125896303	0.16735
GNL3	-0.518122025	1.99E-05	-0.015487628	0.89785	-0.232446611	0.05481
MBNL1	0.422572507	2.01E-05	0.55394949	2.00E-08	0.390986544	7.88E-05
NR2C2AP	-0.854341097	2.01E-05	-0.265359699	0.16958	-0.125549523	0.51855
UTRN	-0.624283427	2.02E-05	-0.323202187	0.02709	-0.474297916	0.00119
LCN15	3.622785171	2.06E-05	3.965146529	3.15E-06	0.143068864	0.86821
TMEM128	-0.662838919	2.07E-05	-0.074662484	0.61637	-0.295331105	0.05208
ATP13A3	-0.54681781	2.11E-05	-0.136316739	0.28828	-0.446113963	0.00052
SEC11C	-0.554226464	2.15E-05	-0.025889472	0.83996	-0.057703141	0.65345
DOC2B	4.54943835	2.15E-05	3.153418085	0.00378	3.218787229	0.00329
AC131180.1	-8.006798869	2.19E-05	-0.855204709	0.59117	-0.981284004	0.53797
AGR3	-0.826007669	2.23E-05	-0.35643241	0.06596	-0.156413151	0.41958
RAD1	-0.469774803	2.26E-05	-0.041531268	0.70248	-0.0949212	0.38586
RPS18	-0.553249246	2.36E-05	0.058396154	0.65507	-0.258146212	0.0484
ETFB	-0.569142264	2.39E-05	-0.227853313	0.08974	-0.262427098	0.05072
KRT6B	4.78955966	2.37E-05	4.071249952	0.00033	3.886552871	0.00061
MCEE	-0.895944307	2.38E-05	-0.604238128	0.00361	-0.41371514	0.04205
MGAT4B	0.436818737	2.37E-05	0.093861149	0.3643	0.503321296	1.08E-06
SKIV2L2	-0.339485574	2.38E-05	0.130459692	0.09956	-0.228629126	0.00424
ASS1	1.453945168	2.40E-05	1.496766275	1.33E-05	1.44978772	2.56E-05
MUT	-0.344798671	2.41E-05	0.009867004	0.9011	-0.017302982	0.82878
CCNI	0.460334823	2.45E-05	0.162099902	0.13733	0.435127721	6.61E-05
UNC119	0.535349568	2.45E-05	0.625093428	7.93E-07	0.411962866	0.00119
CDC42BPE	0.362820417	2.48E-05	-0.176839502	0.04056	0.173015547	0.04489
ALG5	-0.451130231	2.49E-05	0.03440977	0.74127	-0.003794342	0.97117
STK40	0.50781574	2.50E-05	0.108367295	0.3705	0.142138776	0.24132
SVIP	-0.664161853	2.50E-05	-0.150805307	0.33235	-0.156266245	0.3161
POU2F2	2.610235987	2.51E-05	1.827882588	0.00357	2.011980229	0.00135
PTGES	1.691356467	2.51E-05	0.889558612	0.02712	0.677980629	0.0929
ARHGAP26	0.836290869	2.53E-05	0.167173002	0.40051	0.87874716	9.40E-06
CD69	2.014266205	2.56E-05	1.894732732	7.46E-05	0.581786171	0.22713
BCL9L	1.092898723	2.58E-05	-0.24565375	0.34876	0.81262578	0.00178
LRRC45	-0.778013455	2.58E-05	-0.649673438	0.00034	-0.373207488	0.03814
EIF2S1	-0.448383751	2.62E-05	0.213944228	0.04296	-0.26210702	0.01381
RPS3A	-0.522090509	2.63E-05	0.098235326	0.42874	-0.184304905	0.13776
ATAD2	-0.57112469	2.66E-05	-0.136984456	0.31089	-0.360727713	0.00781
ECHS1	-0.427529334	2.67E-05	0.067032571	0.50257	-0.137639964	0.17208
AKR1A1	-0.389090322	2.72E-05	0.006531737	0.94327	-0.019073237	0.83555
C10orf32	-0.626010226	2.71E-05	-0.319084357	0.02999	-0.36350595	0.01383

GRB14	-1.157058967	2.72E-05	-0.023708198	0.92965	-0.43621434	0.10691
NDST2	0.774442339	2.72E-05	0.185970355	0.31552	0.155895895	0.40059
ASF1B	-0.730879989	2.78E-05	-0.243975501	0.15372	-0.322649435	0.06088
TMEM101	-0.512043202	2.80E-05	-0.274695143	0.02127	-0.178257529	0.13584
ARHGAP29	-0.67879364	2.81E-05	-0.44382107	0.0059	-0.52385279	0.00119
FNBP1	0.505808102	2.81E-05	0.366279177	0.00248	0.480495757	7.29E-05
MAPK1IP1L	0.736581728	2.85E-05	-0.006871386	0.96886	0.420401388	0.01695
TMX1	-0.403018767	2.86E-05	0.118324491	0.21257	-0.125494717	0.18991
ACAA1	-0.578132395	2.94E-05	-0.356824778	0.00936	-0.26960587	0.04991
NRP2	0.999232888	2.92E-05	0.122467215	0.60967	0.148845584	0.5354
OAT	-0.600057743	2.94E-05	-0.22232254	0.11893	-0.555504899	0.00011
RPL36	-0.440771015	2.94E-05	0.175583407	0.09458	-0.172791737	0.10076
RPS12	-0.545064536	2.93E-05	0.167521649	0.19826	-0.197502096	0.12966
ZNF146	-0.336818018	3.02E-05	0.133338594	0.09538	-0.25767969	0.00138
ACTN1	0.450678794	3.04E-05	0.254801517	0.01837	-0.058771381	0.58741
HIVEP1	0.602153405	3.10E-05	0.08959633	0.53689	0.136987827	0.34519
PLA2G12A	-0.781499774	3.11E-05	-0.168942913	0.36424	-0.300549005	0.10743
MOCOS	-1.308460154	3.14E-05	-0.499827615	0.10899	-0.445036728	0.15487
RNF141	-0.423256354	3.19E-05	-0.064232669	0.52115	0.044424194	0.65756
ZDHHC4	-0.538528481	3.21E-05	-0.217914662	0.08858	-0.362029637	0.00487
HKB-CPT1	0.770572929	3.29E-05	-0.003825672	0.98379	0.587172873	0.00158
SYDE1	1.030614041	3.30E-05	0.073713231	0.76846	0.047066362	0.8511
GPN2	1.193870965	3.30E-05	0.461819135	0.10833	1.053242033	0.00025
SULT1C4	1.150557605	3.34E-05	0.155825028	0.58432	0.018934271	0.94734
RFC4	-0.786938398	3.40E-05	-0.229482226	0.22055	-0.519583369	0.00587
TXNRD3	-0.590097036	3.40E-05	-0.048064345	0.72051	-0.288253296	0.03469
ZBTB7A	0.614514163	3.41E-05	0.148063801	0.32084	0.556551303	0.00017
SLC39A13	0.588043149	3.45E-05	-0.033415892	0.81588	0.171952577	0.23041
AGPAT3	0.414075501	3.47E-05	0.108534857	0.27863	0.368380385	0.00023
CYB561	-0.779468378	3.50E-05	-0.773850534	3.49E-05	-0.137921149	0.45703
ATP6V0C	1.232779578	3.51E-05	0.917342567	0.0021	0.780841486	0.00906
ELOVL5	0.648687175	3.53E-05	0.577485032	0.00023	0.138012124	0.38017
TPRKB	-0.493112319	3.61E-05	0.03344885	0.76933	-0.106424095	0.35826
EHBP1L1	0.680990553	3.64E-05	-0.20481699	0.2171	0.359143396	0.02976
RHOT2	0.525627705	3.68E-05	-0.071271461	0.57997	0.330736845	0.00951
SLC44A1	-0.575556779	3.71E-05	-0.342152054	0.01398	-0.012941328	0.92591
CEP44	-0.563751928	3.87E-05	-0.006211037	0.96315	-0.222523187	0.101
CPSF1	0.784504928	3.86E-05	-0.214262519	0.27284	0.404884178	0.03535
LRRC8A	0.484827945	3.87E-05	0.196731718	0.09537	0.169378507	0.15224
IL17C	3.632521577	3.96E-05	2.651911469	0.00281	3.503008819	7.43E-05
IL23A	2.52230968	3.96E-05	2.542581451	3.12E-05	2.445589467	6.83E-05
MRPL13	-0.544709057	3.99E-05	0.143487576	0.26715	-0.273207539	0.03732
PQLC3	-0.784084467	4.05E-05	-0.31166959	0.09163	-0.535523163	0.00447
F2R	0.700179671	4.10E-05	0.243492822	0.1542	0.2062452	0.22797
KIAA1919	-0.454657018	4.10E-05	-0.531714973	1.43E-06	-0.332071919	0.00253
UBALD2	1.165636567	4.11E-05	0.652927336	0.02191	0.946143009	0.00089
NDUFB3	-0.444297723	4.13E-05	0.124335354	0.23757	-0.18325517	0.08638

BCAR1	0.703551829	4.18E-05	0.06858332	0.69064	0.313591142	0.06855
ENKUR	2.437664284	4.20E-05	1.347271214	0.02619	1.698220321	0.00479
ERMP1	-0.457085833	4.20E-05	-0.254770504	0.02157	-0.029650728	0.78901
SMC2	-0.470228564	4.21E-05	0.082700754	0.46759	-0.281036169	0.01401
TOM1	0.871051221	4.24E-05	0.440035579	0.03935	0.590182312	0.00569
ATP13A2	0.652293038	4.35E-05	0.270184387	0.09132	0.527132417	0.00097
IRAK3	1.777228976	4.36E-05	1.481778014	0.00066	1.604822861	0.00023
SETD5	0.443699123	4.37E-05	-0.017074689	0.87527	0.362605557	0.00084
CAPZB	0.306691102	4.42E-05	0.251977667	0.00076	0.320015439	1.98E-05
LRRC58	-0.322358929	4.47E-05	0.057381953	0.46075	-0.179169627	0.02251
VPS18	0.399559855	4.48E-05	0.098338027	0.31651	0.038649831	0.69683
RPS27L	-0.55401488	4.50E-05	0.038631602	0.77493	-0.314621959	0.02024
MRPS14	-0.462603815	4.53E-05	-0.01075309	0.9222	-0.312582758	0.00513
HIST1H4C	-0.72553868	4.56E-05	-0.142471956	0.42218	-0.582421294	0.00106
BCKDHB	-0.541817935	4.59E-05	-0.168663641	0.19775	-0.10984593	0.40336
NLGN3	1.909281911	4.60E-05	0.891074398	0.06637	1.02414601	0.03388
NCOA7	0.949727855	4.61E-05	1.089359848	2.86E-06	0.849792755	0.00027
MBOAT2	-0.680599458	4.69E-05	-0.701104963	2.71E-05	-0.067849466	0.68357
TCF19	-0.723896497	4.71E-05	-0.329194981	0.0602	-0.37601656	0.03249
CHAF1B	-0.6279341	4.74E-05	-0.39457833	0.00934	-0.296586969	0.05183
MICU2	-0.342845404	4.73E-05	0.063033973	0.44208	-0.136556126	0.10108
KIAA1324	-2.19358513	4.76E-05	-0.640942361	0.22901	-1.30367222	0.01484
SLC38A4	-1.135369636	4.78E-05	-0.319075238	0.24808	-0.3758241	0.17424
OSGEPL1	-0.664500968	4.85E-05	-0.18152225	0.24713	-0.459057838	0.00399
PRICKLE2	0.86444935	4.86E-05	0.159371412	0.45577	0.160599753	0.4555
CRLS1	-0.438922656	4.90E-05	0.141728723	0.17913	-0.162515097	0.12786
SESTD1	0.569826406	4.90E-05	0.556288514	7.10E-05	0.498418591	0.00038
SH2D1B	4.620154233	4.92E-05	3.654643813	0.00141	2.457236196	0.04238
EEF1A1P6	-0.861823018	4.94E-05	-0.384843611	0.06809	-0.592381149	0.00512
STYK1	-1.197504962	4.95E-05	-1.370355893	3.47E-06	-0.111751374	0.70281
NUDT15	-0.398918657	4.96E-05	-0.017551842	0.85444	-0.106839393	0.26882
ACSL4	0.794687758	4.97E-05	1.018460808	1.98E-07	1.018842765	1.96E-07
CCDC138	-0.602393569	4.98E-05	-0.366475255	0.01204	-0.701902566	2.16E-06
RPL22	-0.438257418	5.00E-05	0.089498127	0.40603	-0.107057551	0.32088
UBE2M	0.439649284	5.00E-05	0.101850306	0.34788	0.323502195	0.00281
CA8	1.32422422	5.03E-05	1.3607971	2.98E-05	1.866457375	1.00E-08
PPARG	-0.816691478	5.02E-05	-0.417580775	0.03787	-0.235035434	0.2425
STT3B	-0.325743805	5.04E-05	0.20698584	0.00963	-0.07954782	0.32088
MIPEP	-0.692647188	5.06E-05	-0.154602658	0.35706	-0.272772894	0.10613
NKX3-1	1.765761903	5.06E-05	0.193148123	0.66429	0.120590894	0.78787
C4orf21	-0.666542158	5.08E-05	-0.54128373	0.0009	-0.371809047	0.02335
TOR1AIP2	0.254564009	5.17E-05	0.192403149	0.00218	0.237300567	0.00016
CIAO1	-0.312392545	5.21E-05	-0.048470228	0.52317	-0.139584702	0.06831
MLXIP	0.639118566	5.23E-05	-0.30494553	0.057	0.207821927	0.19168
BMPR1A	0.471831403	5.26E-05	0.488394044	2.74E-05	0.356260648	0.00228
GCNT1	-1.077023259	5.30E-05	-0.199759265	0.45305	-0.259086799	0.33045
GEM	1.061202334	5.31E-05	0.928570706	0.0004	-0.112779025	0.66965

NOX1	2.172403175	5.30E-05	1.664199155	0.0019	1.925725692	0.00035
GPX3	1.342196531	5.34E-05	0.210867207	0.53222	1.233138455	0.00021
SLC4A4	-0.761749098	5.35E-05	-0.373551065	0.04718	-0.312836465	0.09656
CA5B	0.902148671	5.38E-05	0.34905319	0.1184	0.597233044	0.00768
TMCO1	-0.32386302	5.42E-05	0.167884759	0.03276	-0.163790924	0.03976
USP32P2	1.013359366	5.44E-05	0.296987927	0.24354	0.137905151	0.58917
PGRMC1	-0.384046454	5.51E-05	-0.027199959	0.77421	-0.202151282	0.03341
PRCC	0.59669524	5.52E-05	0.048478638	0.74435	0.281624403	0.05763
SSX2IP	-0.477945221	5.51E-05	-0.098157076	0.40051	-0.331420196	0.00508
NXF1	0.612478846	5.53E-05	0.104411854	0.49297	0.45907309	0.00254
DIS3L	-0.471547937	5.55E-05	-0.215873826	0.06136	-0.256375204	0.02717
HIVEP2	0.781103034	5.60E-05	0.222933973	0.25088	0.457441475	0.01851
BMPR2	0.607422994	5.63E-05	0.234861638	0.11958	0.300034627	0.04684
CDC14B	-0.556003544	5.65E-05	-0.115378247	0.39963	0.025518295	0.85226
CNOT3	1.033375642	5.73E-05	0.240041154	0.35582	0.836551373	0.00117
SRFBP1	-0.515064715	5.76E-05	0.087518701	0.48544	-0.275780912	0.02988
GIT2	0.474765368	5.82E-05	0.039486699	0.73894	0.315053047	0.00786
PMS1	-0.585224717	5.82E-05	-0.173898472	0.22586	-0.381697347	0.00832
GOT1	0.446071366	5.87E-05	0.333335586	0.00249	0.316835368	0.00426
HSPB11	-0.453507644	5.98E-05	-0.057566136	0.59627	-0.312802917	0.00496
THYN1	-0.467661121	6.12E-05	0.144171383	0.20164	-0.295723001	0.01044
MRPL40	-0.506823253	6.14E-05	0.076805165	0.52785	-0.334561639	0.00737
TMEM65	-0.325891891	6.14E-05	-0.181370779	0.02294	-0.310163193	0.00013
MYEF2	-0.632773496	6.21E-05	-0.002157285	0.98899	-0.396290291	0.01178
H2AFZ	-0.617545975	6.26E-05	-0.073233518	0.63381	-0.314399796	0.04119
STXBP6	-0.79574914	6.26E-05	-0.383464918	0.05109	-0.519325188	0.00855
RASGRP1	1.198289083	6.31E-05	0.811922732	0.0067	1.253532272	2.77E-05
CMC1	-0.751435629	6.32E-05	-0.094873933	0.6079	-0.099059989	0.59022
CHI3L1	2.943779914	6.41E-05	2.677626471	0.00028	3.081887667	2.82E-05
SF3B14	-0.362965301	6.50E-05	0.37944556	1.77E-05	-0.228171544	0.01148
FBP1	-1.193819798	6.54E-05	-0.291927909	0.31769	-0.310898989	0.28851
MAMLD1	1.74975069	6.54E-05	0.056275057	0.90304	0.578180772	0.2123
NDUFA10	1.204436497	6.53E-05	0.618583321	0.04147	0.671509545	0.02648
UBE2V1	0.391041256	6.73E-05	0.225562814	0.02142	0.250907099	0.01059
CDCA7	-0.807345294	6.75E-05	-0.177702362	0.37584	-0.562405121	0.00532
ANKRD13D	0.577928552	6.82E-05	-0.066456093	0.65119	0.279519368	0.05565
PQLC1	0.563874124	6.82E-05	0.136772844	0.33675	0.480870376	0.00077
LDB2	-0.844361216	6.93E-05	-0.37058537	0.07777	-0.857708468	5.47E-05
PIK3AP1	1.394076318	6.98E-05	1.188092668	0.0007	1.205173969	0.00059
TRNP1	-0.837547218	7.03E-05	-0.827264105	8.60E-05	-0.37102815	0.07787
PEX7	-0.666253952	7.04E-05	-0.352081937	0.02588	-0.41051641	0.01169
HMGCL	-0.485718954	7.09E-05	-0.233120092	0.05296	-0.093464792	0.43753
TNFSF15	1.062355114	7.18E-05	0.656591889	0.01429	0.565200196	0.03555
STAT3	0.509262629	7.21E-05	0.262248743	0.04096	0.248879597	0.05258
PHF5A	-0.446125511	7.26E-05	0.006811095	0.95021	-0.419845073	0.00017
KSR1	0.844941558	7.30E-05	-0.090061121	0.67446	0.330242469	0.12322
MOB3A	0.59946299	7.32E-05	0.073563114	0.62993	0.528618937	0.00047

TLN1	0.540761799	7.36E-05	0.103164034	0.44967	0.179716537	0.18814
FANCI	-0.54591367	7.50E-05	-0.299638113	0.02865	-0.303000413	0.02733
IFI16	1.090389154	7.56E-05	0.899839486	0.00109	0.595729515	0.03082
PATL1	0.49841503	7.61E-05	0.081437332	0.51986	0.577416488	4.40E-06
EFR3A	-0.294340242	7.68E-05	0.110454643	0.1323	0.085978595	0.24358
RC3H1	0.561592978	7.71E-05	0.124647648	0.38021	0.54906754	0.00011
MAPK7	0.791884036	7.78E-05	0.182872009	0.36661	0.51228215	0.01114
KIAA1147	-0.33337689	7.80E-05	-0.142181171	0.08842	0.082886405	0.3195
HNRNPA3	0.527197217	7.86E-05	0.018846723	0.88781	0.542273823	4.84E-05
MZT1	-0.539416565	7.86E-05	0.044565855	0.73447	-0.298908489	0.0261
ANAPC16	-0.434726165	7.89E-05	-0.154842477	0.15441	-0.046210202	0.67128
CHD1L	-0.432653739	7.96E-05	-0.155377058	0.15184	-0.155505283	0.15324
RHBDD2	0.491524325	7.98E-05	0.157735179	0.2086	0.241991132	0.05395
FMO5	-0.714956778	8.11E-05	-0.499079515	0.00542	-0.11412512	0.52278
LRRCC1	-0.71200343	8.11E-05	0.130909034	0.45952	-0.531858082	0.003
COX11	-0.368194271	8.13E-05	-0.033009701	0.71759	-0.131657492	0.15442
PKD1	0.69559477	8.19E-05	-0.222156161	0.21126	0.379764665	0.03188
C1S5-TXN	-1.035946307	8.22E-05	-0.6451645	0.01395	-0.853971639	0.00116
MTATP6P1	1.261052453	8.23E-05	0.689114025	0.0321	1.309453405	4.27E-05
GATM	-0.74590676	8.27E-05	-0.214086379	0.25683	0.007771586	0.96715
ZKSCAN1	-0.432432693	8.28E-05	-0.375457769	0.00059	-0.386577258	0.00047
B3GNT2	0.532258636	8.36E-05	0.647859659	1.48E-06	0.659622636	1.01E-06
P11-958N24	1.722030103	8.44E-05	0.522141206	0.24062	0.731566771	0.09692
ELL	0.674624596	8.54E-05	-0.027212706	0.87602	0.062784894	0.71923
TMEM139	0.873750433	8.61E-05	0.836032228	0.00017	0.632455926	0.00467
CHMP4B	0.319438031	8.71E-05	0.466903049	6.68E-09	0.280466154	0.00057
IGLON5	1.025271115	8.70E-05	0.028060347	0.91659	0.018090793	0.9465
CDC5L	-0.267457725	8.74E-05	0.063373234	0.34539	-0.180478321	0.00784
YEATS4	-0.462182617	8.73E-05	0.117797619	0.2973	-0.27336969	0.01806
DHRS7B	-0.470900842	8.84E-05	-0.012043557	0.91798	-0.277479767	0.01933
RPS27A	-0.493600357	8.89E-05	0.245073533	0.05131	-0.18726904	0.13676
LETM2	1.007998298	9.05E-05	0.59202506	0.02202	0.416548624	0.10936
PSEN1	0.349509391	9.06E-05	0.234180114	0.00855	0.35030375	8.46E-05
DCP1A	0.724784169	9.08E-05	0.322312823	0.08429	0.431832398	0.02092
ACN9	-0.697188799	9.14E-05	0.009966862	0.95365	-0.372788956	0.033
C1orf112	-0.558098438	9.13E-05	-0.196169758	0.16364	-0.266240612	0.0601
NPTN	0.356576522	9.16E-05	0.229798905	0.01155	0.295422327	0.00119
AAK1	0.69996484	9.23E-05	-0.188020688	0.30433	0.311093902	0.08557
ABCE1	-0.477057804	9.37E-05	0.284041758	0.01919	-0.26763491	0.02798
CENPM	-0.861795492	9.36E-05	-0.572558839	0.00741	-0.19626645	0.3542
NHLRC3	-0.44204925	9.47E-05	-0.337119385	0.00274	-0.107778938	0.33719
EMP3	0.617440695	9.48E-05	0.448271543	0.00455	0.057448554	0.71893
FASTKD2	-0.435605262	9.54E-05	0.050559856	0.64547	-0.336503116	0.00245
RPS15A	-0.420170694	9.59E-05	0.095573463	0.3743	-0.134653758	0.21095
PPP1CC	-0.29731672	9.62E-05	0.19160994	0.0114	-0.048840931	0.52042
MCM2	-0.623737957	9.74E-05	-0.26102705	0.10145	-0.577755194	0.0003
MEN1	0.395510047	9.87E-05	-0.054080294	0.5987	0.2684934	0.0083

NFKB1	0.792042761	9.99E-05	0.428080897	0.03562	0.362133256	0.07576
SERPING1	0.858146314	0.0001	0.27807704	0.20898	0.324227481	0.14372
DHFR	-0.667348598	0.0001	-0.120561647	0.48055	-0.251250936	0.14201
FBRS	0.640860622	0.0001	-0.359396081	0.03181	0.466179433	0.00477
LGR5	-1.723171916	0.0001	-1.075482558	0.01478	-1.166415288	0.00829
KIF20B	-0.573966426	0.0001	0.024261582	0.86862	-0.330945503	0.02472
CDC42EP2	0.632788347	0.0001	0.302896119	0.06346	0.662262141	4.77E-05
PDCD6IP	0.365819157	0.0001	0.29077078	0.00201	0.291048949	0.002
MTMR8	-0.987652552	0.00011	-0.502109004	0.04266	-0.18612977	0.44852
RPS23	-0.526872373	0.00011	0.138668704	0.30728	-0.366260268	0.00704
TNF	4.563542268	0.00011	4.698828868	6.26E-05	5.23426185	6.42E-06
SUPT6H	0.308037662	0.00011	-0.03108199	0.69639	0.202496368	0.01111
TBC1D31	-0.520027289	0.00011	-0.151785587	0.24292	-0.384898723	0.00368
CMBL	-0.883717486	0.00011	-0.636205943	0.00502	-0.174189798	0.44119
ANGPTL2	0.863120195	0.00011	0.411555454	0.06693	-0.252391186	0.2667
GINS2	-0.795575443	0.00011	-0.341305903	0.09359	-0.420802259	0.03967
FERMT2	0.712126409	0.00011	0.757242919	3.92E-05	0.14139247	0.4438
CNBP	-0.30621875	0.00011	0.205963961	0.00887	-0.028920814	0.71423
COL9A2	1.055109792	0.00011	0.645040862	0.01843	1.433803238	1.31E-07
TSPAN6	-0.368055976	0.00011	0.10369289	0.27026	-0.32644761	0.0006
EFCAB3	1.515319674	0.00011	0.576426736	0.15926	1.177166424	0.0031
RPS14	-0.489480106	0.00011	0.007146457	0.95499	-0.266761693	0.03526
FAF1	-0.476600849	0.00011	-0.088146366	0.46962	-0.192224835	0.11649
C14orf2	-0.396778195	0.00011	-0.082099172	0.41933	-0.16250072	0.11229
RPS27	-0.447488322	0.00011	-0.042013822	0.71687	-0.273690711	0.01823
ROBO1	0.493956503	0.00012	0.37882495	0.00309	0.022133688	0.863
MRPL16	-0.427604178	0.00012	0.086171149	0.42293	-0.144689514	0.18486
PHKA1	-0.635734849	0.00012	-0.097354789	0.54356	-0.294589423	0.0691
PHLPP2	0.624504489	0.00012	0.365179862	0.02466	0.36538473	0.02487
RNY4P13	-1.212954904	0.00012	-0.290982883	0.30554	-0.349278228	0.22529
ASF1A	-0.434428377	0.00012	-0.002972859	0.97841	-0.420787805	0.00019
CH25H	1.770810819	0.00012	0.553625766	0.23738	0.844050951	0.07065
ANTXR1	0.770173786	0.00012	0.138657215	0.48915	0.113253104	0.57347
IFITM2	0.676774275	0.00012	0.553362353	0.00166	0.353165607	0.04487
AC040977.1	0.648697377	0.00012	0.238717246	0.15845	0.509539135	0.00255
GNA15	1.742006319	0.00012	1.380946873	0.00233	1.878800446	3.28E-05
PTPRK	0.344339919	0.00012	0.299281964	0.00079	0.157759587	0.07844
TRAPPC2L	-0.470927216	0.00012	-0.061993754	0.60236	-0.171790714	0.15334
BEND4	3.071026386	0.00012	1.106444989	0.1851	1.989021708	0.01461
REXO1	0.940475062	0.00012	-0.091768584	0.70968	0.606310231	0.01374
STK35	0.276886791	0.00012	-0.130838149	0.0727	0.238015973	0.00096
C9orf114	-0.583732301	0.00012	-0.510784417	0.00075	-0.422897834	0.00551
CRNKL1	-0.464749473	0.00012	0.019571736	0.8702	-0.19542621	0.10577
LRCH4	0.762175826	0.00012	-0.474979089	0.01928	0.258452535	0.19611
DDX39B	0.328936526	0.00013	0.286558854	0.00083	0.219322485	0.01056
IER3IP1	-0.4604589	0.00013	-0.024782193	0.83489	-0.101345313	0.39626
CEP85L	-0.555216012	0.00013	-0.019814912	0.89055	-0.406414134	0.00499

FAM189B	0.72053746	0.00013	0.046357411	0.80811	0.372997466	0.04888
MSH6	-0.489221291	0.00013	0.001576629	0.99011	-0.379777033	0.00292
TFEB	0.756198207	0.00013	0.50497751	0.01062	0.577817446	0.00337
ADTRP	1.575744781	0.00013	2.140412775	1.06E-07	2.094811304	2.40E-07
ARHGAP5	-0.4097401	0.00013	0.036291345	0.73398	-0.169802536	0.11221
TK1	-0.664777248	0.00013	-0.583606361	0.00072	-0.297276335	0.08469
FBXL19	0.712817747	0.00013	-0.004129298	0.98241	0.409121878	0.02865
PFN2	-0.312717746	0.00013	0.137583715	0.08924	-0.126820399	0.11985
TUBB2B	1.583278812	0.00013	1.269887037	0.00217	0.37305197	0.37048
PML	0.676986572	0.00013	-0.156707115	0.38374	0.311077184	0.08174
NUP155	-0.409467657	0.00013	-0.033403611	0.75361	-0.357466254	0.00082
GRTP1	-0.817082805	0.00013	-0.469521177	0.02477	-0.277481527	0.18376
ADRM1	0.422925598	0.00013	0.106375149	0.33824	0.293476704	0.00816
ALG10B	-0.395884954	0.00013	-0.104314471	0.30697	-0.262658194	0.01074
RAB1A	0.338055484	0.00013	0.238923596	0.00684	0.372011543	2.59E-05
NRBP2	0.549329218	0.00014	-0.280764331	0.05488	0.315953874	0.02773
TMEM184E	0.565550162	0.00014	0.174986517	0.23883	0.356471399	0.01631
FLII	0.472390002	0.00014	0.21668056	0.0803	0.284083382	0.02188
OSTC	-0.436612503	0.00014	0.021978385	0.84668	-0.292448623	0.01042
EIF4G2	0.577140685	0.00014	0.54766881	0.0003	0.466938165	0.00204
PRRC2B	0.469012967	0.00014	-0.36918097	0.00277	-0.00839713	0.94567
ARHGEF28	-0.954990541	0.00014	-0.427154073	0.08681	-0.487386944	0.05103
ALDH1A1	-0.685190796	0.00014	0.002010078	0.99108	-0.091134921	0.61217
AP3D1	0.439092291	0.00014	-0.055317602	0.63188	0.315134613	0.00633
DUSP7	0.653117336	0.00014	0.076399418	0.66246	0.227303556	0.19371
MYEOV2	-0.563234416	0.00014	0.062280382	0.65916	-0.360005306	0.01327
PSMC4	0.461329431	0.00014	0.681525379	1.51E-08	0.41925098	0.00054
CENPQ	-0.654805254	0.00014	0.046839185	0.77431	-0.427207842	0.01146
MTRF1	-0.533896695	0.00015	-0.534380494	0.00011	-0.125269113	0.36188
STYXL1	-0.436967437	0.00015	-0.12514541	0.25857	-0.27359825	0.01529
PPAT	-0.564977338	0.00015	-0.043309396	0.76867	-0.381008427	0.01028
GPX2	0.789934092	0.00015	0.782703063	0.00017	1.042786419	5.42E-07
TJP1	0.501058057	0.00015	0.212929417	0.1069	0.333124648	0.01168
KNTC1	-0.511098115	0.00015	-0.380808526	0.00453	-0.262766464	0.05073
SSBP1	-0.318462741	0.00015	0.340815957	3.10E-05	-0.108671777	0.19089
RNF38	0.603239899	0.00015	0.330207858	0.03787	0.391309012	0.01405
RPE	-0.380272396	0.00015	-0.0919686	0.35288	-0.084938302	0.39303
CIZ1	0.480729878	0.00015	-0.009080629	0.94313	0.411447182	0.0012
MTHFS	-0.717252971	0.00015	-0.108324628	0.55834	-0.058152607	0.75393
PLK4	-0.593638191	0.00015	-0.035545302	0.81822	-0.317128335	0.04165
PRTFDC1	-0.714140414	0.00015	-0.017837144	0.92272	-0.376438304	0.04307
ETS1	0.582635316	0.00015	0.170749058	0.26834	0.189413018	0.22035
HLA-DQB1	1.521710333	0.00016	1.814798097	6.23E-06	0.478556901	0.23803
SPRR1B	3.559524678	0.00016	2.622136247	0.00606	3.510138515	0.00019
NINJ1	0.81099563	0.00016	0.6026724	0.00499	0.175774534	0.41726
NUDCD2	-0.546957874	0.00016	-0.009855345	0.94534	-0.077882405	0.58901
PHGDH	-0.432385335	0.00016	-0.150914754	0.18345	-0.143890918	0.20607

MT-CYB	0.907656998	0.00016	0.442119223	0.06603	1.121673514	3.08E-06
MTND4P12	0.970833217	0.00016	0.720213666	0.00512	1.232086941	1.59E-06
PTBP1	0.485991881	0.00016	-0.101331356	0.43166	0.153903159	0.23233
STX8	-0.416111357	0.00016	-0.00032489	0.99756	-0.383381481	0.00046
SARNP	-0.35829512	0.00016	0.101514445	0.27455	-0.166931679	0.07617
JUP	0.617846066	0.00016	0.229179116	0.16187	0.51098843	0.00181
METTL12	-0.888831962	0.00016	0.23439241	0.31391	-0.506196886	0.03073
WASF1	0.714689977	0.00016	0.49208913	0.00946	0.231300104	0.22602
IRAK1BP1	-0.831158769	0.00017	-0.220675531	0.30652	-0.254521872	0.24359
AREL1	0.437589831	0.00017	0.452699179	9.32E-05	0.274542621	0.01807
RPS4XP22	-0.866294083	0.00017	-0.006992065	0.97357	-0.385494949	0.07833
COX15	-0.392163429	0.00017	-0.006583732	0.94883	-0.091041358	0.37821
ALPK3	0.688783507	0.00017	0.135791415	0.45911	0.594789383	0.00116
EEF1A1P4	-0.753520843	0.00017	-0.232058488	0.21626	-0.415756744	0.03101
HDAC7	0.657740043	0.00017	0.154269429	0.3792	0.3450628	0.04939
SENP6	-0.254066373	0.00017	0.006556965	0.92192	-0.161490646	0.0164
ZEB2	1.014788304	0.00017	-0.038267441	0.88762	0.471206968	0.08138
PAIP2B	-0.46593349	0.00017	-0.10194197	0.39865	0.0519868	0.66669
CENPE	-0.843263619	0.00017	0.058469854	0.79418	-0.609544625	0.00661
PI4KB	0.437977633	0.00017	0.173624049	0.13641	0.38456778	0.00097
C7orf43	0.601983242	0.00018	-0.094458066	0.56765	0.210699135	0.19752
DEK	-0.504464174	0.00018	0.196894023	0.14166	-0.280753596	0.03649
AC004057.1	1.480206383	0.00018	1.685239251	1.94E-05	1.326454565	0.00078
ZNF703	0.687048039	0.00018	-0.231866836	0.22161	0.151438468	0.41844
RP11-762I7.	1.357839369	0.00018	0.735621932	0.04317	1.068331767	0.00324
NDRG4	0.879471857	0.00018	0.138786613	0.55499	-0.415057323	0.08659
FUT2	0.603213527	0.00018	0.145445464	0.36706	0.807139204	4.97E-07
TUFM	-0.575193848	0.00018	-0.132117048	0.38807	-0.284344874	0.06355
IGDCC4	0.903894495	0.00018	-0.057737521	0.8132	0.138367722	0.57033
C6orf211	-0.412660027	0.00018	0.163298993	0.13158	-0.158273278	0.14816
TD-2005D2	-2.475820074	0.00018	-0.393642684	0.4613	-1.293440577	0.02383
HIPK1	0.362057333	0.00018	0.18014447	0.06209	0.259850364	0.00721
LSM3	-0.445050414	0.00018	0.359437693	0.002	-0.334382832	0.00472
MAPKAPK2	0.285553194	0.00018	0.178078586	0.01901	0.168649741	0.02729
PSPC1	-0.555659533	0.00018	-0.384141789	0.00907	-0.348211842	0.01847
RPL31	-0.470007451	0.00018	0.094981537	0.44885	-0.163180343	0.19349
SH2B3	0.828173414	0.00018	0.289177726	0.1931	0.380480194	0.08778
SUSD4	-1.227370509	0.00018	-0.056265368	0.85944	-0.415469903	0.19684
ZFAND1	-0.479845236	0.00018	0.062328861	0.62093	-0.095054168	0.45319
CLCN6	0.606380192	0.00018	-0.130078757	0.42777	0.377851268	0.02015
ELMO2	0.497037262	0.00018	0.107636266	0.41864	0.350196458	0.00848
FABP5	-0.668798467	0.00018	-0.045550369	0.79789	-0.032575832	0.85479
RPA3	-0.639352377	0.00019	-0.129997942	0.43388	-0.426967896	0.01129
STEAP3	-0.895211679	0.00018	-0.361083899	0.12886	-0.316554918	0.1833
TMEM259	0.412313557	0.00018	-0.060504417	0.58517	0.290247973	0.00857
EPRS	-0.454523014	0.00019	0.256002368	0.03473	-0.166172908	0.17137
MFHAS1	0.555820091	0.00019	0.356855231	0.0165	0.256596075	0.08539

SP110	0.68960074	0.00019	0.379408686	0.0419	0.514640922	0.00576
RNF223	-1.031189752	0.00019	-1.272989165	4.45E-06	-0.320646463	0.22832
JARID2	0.418692301	0.00019	0.125254293	0.26535	0.320316057	0.00438
RARS2	-0.363969716	0.00019	0.20327826	0.03311	-0.148628997	0.125
PRRC2A	0.456357563	0.00019	-0.424023189	0.00056	0.193067215	0.11542
?11-459D2?	-1.725836162	0.00019	-0.519863323	0.19329	-0.577153783	0.15482
GJB6	-2.51581248	0.0002	-1.183641564	0.05707	-0.462377052	0.44837
NDUFAB1	-0.471428337	0.0002	0.147632182	0.23367	-0.196387507	0.11694
DKK3	-0.791796717	0.0002	-0.3631042	0.081	-0.467490844	0.02682
ATRAID	-0.364412858	0.0002	0.063316779	0.51194	-0.21530927	0.02711
CASP5	2.111397195	0.0002	1.835833814	0.00124	2.063284909	0.00028
ATAD1	-0.356647763	0.0002	0.061954613	0.51371	-0.199005158	0.03717
NHSL2	1.277923856	0.0002	0.032480424	0.92719	0.256059881	0.47066
EXOSC9	-0.46802296	0.0002	0.130797737	0.29158	-0.229303229	0.06765
MATR3	-0.315552027	0.0002	0.168475785	0.04675	-0.199514408	0.01873
NAT10	-0.506160821	0.0002	-0.03383404	0.80209	-0.308545014	0.02314
XPO1	-0.329070409	0.0002	0.05066698	0.56631	-0.173817971	0.04955
SVIL	0.56580148	0.0002	0.262564816	0.08489	0.323279551	0.03405
FANCF	-0.451947992	0.00021	-0.035664623	0.76243	-0.339091175	0.00492
TMED3	-0.54476604	0.00021	-0.117225654	0.42314	-0.198838159	0.1746
SULT1C2	-1.189537067	0.00021	-0.970480356	0.00244	-0.751585183	0.01881
FAT1	0.500451098	0.00021	0.393444006	0.00357	0.173199306	0.19971
JTB	-0.386160338	0.00021	-0.029462258	0.773	-0.072329715	0.48189
PLK3	0.794335596	0.00021	0.18788751	0.38485	0.212233981	0.3285
SLC28A2	-1.728652509	0.00021	-0.393873548	0.39503	0.076334864	0.86898
SMIM6	-0.927706099	0.00021	-0.825412983	0.00084	-0.196069459	0.42106
IMP3	-0.438240221	0.00021	0.23036411	0.04456	-0.152023799	0.19235
MAP4K4	0.470161806	0.00021	0.329339236	0.00945	0.019911568	0.87554
PIEZO2	0.591935904	0.00021	0.161192944	0.31327	0.361550312	0.02395
TTC9	0.69266673	0.00021	0.600670908	0.0013	0.554028617	0.00309
C1orf27	-0.357114906	0.00021	0.044331766	0.63783	-0.285040818	0.00291
AL353671.?	-1.415809973	0.00021	0.090402525	0.80463	-0.3556024	0.33567
PRDX3	-0.454169014	0.00021	0.035903869	0.7684	-0.279609891	0.02233
C17orf62	0.434391514	0.00021	0.096747438	0.41403	0.202135318	0.08716
?11-1220K?	1.547510961	0.00021	1.729109558	3.52E-05	1.940593322	3.43E-06
IER5L	0.98221434	0.00022	0.069074435	0.79616	0.330933261	0.21518
RASA1	-0.321098145	0.00022	0.012594288	0.88377	-0.181848309	0.03555
PDLIM7	0.843803118	0.00022	-0.089628646	0.69584	0.036098021	0.87481
AGPS	-0.461726228	0.00022	-0.061847902	0.61864	-0.074478717	0.54916
ARHGAP1?	0.539215198	0.00022	0.232866866	0.11244	0.246018864	0.09442
GSK3A	0.549881557	0.00022	-0.102542525	0.49536	0.406117554	0.00637
UBR4	0.493011221	0.00022	-0.016902533	0.89928	0.482364254	0.0003
PLAT	0.962077717	0.00022	0.340049529	0.19186	0.244135683	0.34889
COPG2	-0.490615207	0.00022	-0.12161092	0.35144	-0.303233392	0.02136
NOP58	-0.402218833	0.00022	0.188267152	0.08004	-0.235863951	0.02959
RSBN1L	-0.531532366	0.00022	-0.164070572	0.25134	-0.362419351	0.01147
TESK1	0.571265595	0.00022	-0.15658884	0.32207	0.296244272	0.05732

WDR76	-0.857290961	0.00023	-0.127759591	0.57907	-0.262738948	0.25592
SNX29P2	1.02031957	0.00023	0.541974461	0.0579	0.674438627	0.01707
P11-155G1	1.325099223	0.00023	0.550546327	0.13449	1.325736536	0.00023
TPM1	0.499834421	0.00023	0.441273243	0.00115	0.129669949	0.3399
NBN	-0.368020631	0.00023	-0.049518867	0.61677	-0.306128315	0.00215
CDC34	0.4481459	0.00023	0.097142164	0.42725	0.211664579	0.08444
ANXA10	-0.872010815	0.00023	-1.07993231	5.21E-06	-0.027264544	0.90834
NR1D1	-1.92998094	0.00023	-2.153501874	4.11E-05	-1.842298458	0.00042
SREBF2	0.454669504	0.00024	-0.114945587	0.3541	0.400537782	0.00119
ERI2	-0.397718357	0.00024	-0.017950462	0.86388	-0.290014358	0.00657
NIT2	-0.547305181	0.00024	0.01347769	0.92725	-0.217740429	0.14174
DNHD1	0.812827864	0.00024	0.334174427	0.13468	1.006013392	5.69E-06
KIRREL	0.794497176	0.00024	0.112183973	0.60424	0.087801327	0.68526
P11-257I8	-0.782570591	0.00024	0.403784586	0.02812	-0.424418726	0.03552
TSG101	0.460340593	0.00024	0.531809929	2.05E-05	0.49707418	7.13E-05
NACC2	0.441019253	0.00024	0.011883846	0.92174	0.073965873	0.542
PIGF	-0.555393083	0.00024	0.134672953	0.36185	-0.25229454	0.092
SPR	-0.610537249	0.00024	-0.074436924	0.64662	-0.016619054	0.91866
GTPBP8	-0.515651276	0.00024	-0.244849453	0.07007	-0.256195587	0.06381
MFSD1	-0.460841648	0.00024	-0.200581394	0.10512	-0.17357018	0.16214
MSH2	-0.743585319	0.00024	-0.115734712	0.56499	-0.593333118	0.00334
WBP1L	0.284913351	0.00024	0.217254354	0.00497	0.113348565	0.14646
WWC2	0.495283099	0.00024	0.300035882	0.02628	0.24626185	0.06896
NRG4	-1.043052721	0.00025	-0.480868913	0.08452	-0.390851726	0.15463
ICMT	-0.39993773	0.00025	-0.000160005	0.99882	-0.233855273	0.03154
EML5	-1.229742308	0.00025	-0.052238372	0.87116	-0.117274427	0.71722
PI4K2A	0.423787215	0.00025	0.173225919	0.13527	0.071904177	0.53925
VPS36	-0.358226566	0.00025	-0.137134849	0.15665	0.079693005	0.41181
PROX1	1.127016123	0.00025	0.660304341	0.0322	0.835154602	0.00678
CHCHD1	-0.530871311	0.00025	0.061970558	0.6588	-0.204254424	0.1519
KAZN	0.728430585	0.00025	-0.157978708	0.43685	0.357379202	0.07459
SH3PXD2A	0.485461332	0.00026	-0.22629478	0.08931	0.291684212	0.0281
CYP26B1	1.148643958	0.00026	0.232161037	0.46635	0.353410437	0.26687
GALNT13	-1.325425516	0.00026	-0.386810762	0.28211	-0.439924127	0.22157
RPL37A	-0.427265787	0.00026	0.153850046	0.18746	-0.263935548	0.02387
ZNF277	-0.496824298	0.00026	0.150693616	0.25799	-0.219573396	0.10272
NDUFB10	-0.372647033	0.00026	0.150482452	0.12957	-0.191246332	0.05885
SLC44A5	-0.834934053	0.00026	-0.746606308	0.0009	-0.382023863	0.08535
CDC25A	-0.625953268	0.00026	-0.310025209	0.06791	-0.436962778	0.01065
GPR37L1	1.02498485	0.00026	0.069641128	0.81586	0.650772052	0.02364
PDHB	-0.273843408	0.00027	0.12913546	0.07951	-0.107606738	0.14877
DCTN1	0.533688781	0.00027	0.100846956	0.49079	0.490392823	0.0008
PTCD3	-0.278532304	0.00027	0.046462725	0.53812	-0.086564168	0.25467
UGT8	-0.674575735	0.00027	-0.23887275	0.19554	-0.198667227	0.28189
MT-ATP8	1.235663135	0.00027	1.019009025	0.00267	1.639263166	1.28E-06
ANO9	0.641972679	0.00027	0.308583353	0.08012	0.261800123	0.13903
PPP1R9A	-0.392218851	0.00027	-0.107579158	0.31466	-0.063942991	0.55053

TCEB2	-0.321513137	0.00027	-0.051244486	0.55723	-0.076448881	0.38366
FRAT2	-0.673866128	0.00027	-0.275227053	0.12973	-0.154724156	0.39459
TROVE2	-0.259580274	0.00027	0.076385822	0.28093	-0.169878982	0.01715
LINC00888	-0.497133736	0.00028	-0.025360442	0.84603	-0.235179019	0.07679
PTPLAD1	-0.366937372	0.00028	0.111649081	0.26746	-0.181233291	0.07248
IHH	-1.082669074	0.00028	-0.783250165	0.00849	-0.136203507	0.64661
FHL3	0.68610946	0.00029	0.031184718	0.87035	-0.038951262	0.83934
SRSF10	-0.304568397	0.00029	0.027216376	0.7448	-0.230149446	0.00606
TCEA3	-1.045170736	0.00029	-0.699604828	0.01266	-0.639895544	0.02298
ZCCHC11	-0.37533842	0.00029	-0.176321497	0.08671	-0.201558144	0.05145
CBL	0.446528819	0.00029	-0.053729092	0.66447	0.175132142	0.15673
FAM193A	0.488841601	0.00029	-0.292118829	0.03211	0.137022489	0.31196
SPINK1	-0.828634329	0.00029	-0.642883697	0.00492	-0.215534822	0.34572
RAD18	-0.45499244	0.00029	-0.078283778	0.52409	-0.585177317	3.13E-06
ODF3B	1.729695444	0.00029	0.486112299	0.32435	1.612877794	0.00072
MOB1B	-0.39230136	0.0003	-0.113952607	0.28762	-0.341674838	0.00157
MTRNR2L4	-1.015741274	0.0003	-0.195865605	0.47898	-0.747786003	0.00738
RRM2	-0.712334189	0.0003	-0.30365269	0.12194	-0.417358662	0.0337
ITGB8	0.721133524	0.0003	0.711599453	0.00035	0.36576276	0.06714
MANEAL	-1.11805415	0.0003	-1.114880761	0.00025	-0.796652147	0.00859
MCAM	0.832154718	0.0003	-0.129717195	0.57782	0.451907302	0.05123
SF1	0.420102539	0.0003	-0.134783091	0.24881	0.213229255	0.0674
SNRPF	-0.529743625	0.0003	0.357497859	0.01295	-0.402663942	0.00576
OAS1	0.654197309	0.0003	0.334096827	0.06522	0.64717754	0.00035
SLC9A3	1.119281385	0.00031	0.264980302	0.40081	0.706047695	0.02353
ARPC2	0.334625972	0.00031	0.412937362	8.11E-06	0.21982809	0.01771
TRMT2B	-0.429085534	0.00031	-0.514235313	1.31E-05	-0.189373088	0.10461
SPCS3	-0.27786027	0.00031	-0.005285898	0.94506	-0.147407738	0.05526
GOPC	-0.285278049	0.00031	0.14099612	0.06964	-0.079369852	0.31187
CASP3	-0.376010928	0.00032	0.054301704	0.59761	-0.074694042	0.47027
HEG1	0.96105764	0.00032	0.537112731	0.0455	0.124710799	0.64463
SPATA5	-0.462283659	0.00032	-0.086205703	0.49567	-0.430851156	0.00074
CENPT	0.692862801	0.00032	0.387167558	0.04507	0.631624893	0.00102
TSEN15	-0.415028288	0.00032	0.172844523	0.12086	-0.155476847	0.16997
ILK	0.423326594	0.00032	0.204331464	0.08204	0.041119278	0.72722
SDHD	-0.34597067	0.00032	0.055198526	0.56026	0.010004928	0.9163
EIF2AK1	-0.337007424	0.00032	-0.297720502	0.00139	-0.064899524	0.48653
PBK	-0.93783237	0.00032	0.115829557	0.6516	-0.516197614	0.04634
BEND3	0.794993772	0.00032	0.74662112	0.00073	1.224196969	2.85E-08
ATG4C	-0.918193401	0.00033	-0.142471636	0.56951	-0.274019986	0.27761
SDC3	0.683299972	0.00033	0.15946563	0.40247	-0.07466698	0.69656
TADA1	-0.385208868	0.00033	0.055673798	0.58933	-0.154783127	0.14207
PITPNA	0.395696802	0.00033	0.415455804	0.00016	0.22439607	0.04219
TGM2	0.832848931	0.00033	0.392555493	0.09099	0.168304692	0.46963
TEAD3	0.642428138	0.00033	0.090231546	0.61555	0.135760161	0.45002
FAM20B	-0.233888844	0.00033	0.032783429	0.60943	-0.048404561	0.45383
NQO1	-0.632971649	0.00033	-0.549312535	0.00183	0.011939765	0.94594

CNTNAP1	0.808008745	0.00033	0.181743081	0.42261	0.325281019	0.15299
SLC38A11	-1.716045262	0.00033	-0.757687313	0.09957	-0.574256767	0.21591
RMI2	-0.763019866	0.00034	-0.53055826	0.01141	-0.540595436	0.01055
NAV1	0.824317885	0.00034	-0.129974579	0.57375	0.198048112	0.39111
PPCS	-0.427434137	0.00034	-0.018005941	0.87846	-0.01324178	0.9107
RCAN1	0.937421385	0.00034	0.797873679	0.00228	0.871031903	0.00087
GATAD2A	0.341845802	0.00034	0.066298404	0.48852	0.127413405	0.18303
HSPB1	0.591446701	0.00034	-0.199299803	0.22925	0.576275135	0.00048
GTF2H3	-0.466899956	0.00034	0.138928794	0.27818	-0.323709692	0.01223
FOCAD	-0.481014452	0.00034	0.051001262	0.70251	-0.15728606	0.24013
MRPL1	-0.57034944	0.00034	0.376871884	0.0151	-0.364542663	0.02104
POFUT1	-0.461155911	0.00034	-0.017750289	0.88988	-0.364236874	0.0046
PFDN5	-0.308798381	0.00035	0.165407573	0.05299	-0.17028867	0.04777
SMIM19	-0.611271517	0.00035	0.192537433	0.24778	-0.49706183	0.00337
DPYSL2	0.345257649	0.00035	-0.009907716	0.91831	0.122595303	0.2045
PPA2	-0.495407843	0.00035	-0.111181164	0.41758	-0.166235859	0.22683
FAM46A	-0.653527786	0.00035	-0.301391617	0.09882	-0.303861723	0.0962
SERP1	-0.412872209	0.00035	-0.085386841	0.45869	-0.179150417	0.12043
SGIP1	0.866736096	0.00035	0.287163572	0.24001	-0.176769996	0.48385
NHP2	-0.467643556	0.00035	0.008290723	0.94813	-0.174953581	0.17443
KIF24	-0.559361315	0.00036	-0.404774584	0.00871	-0.211678882	0.1692
POLR3B	-0.492354406	0.00036	0.213531704	0.11461	-0.188758327	0.16839
NARG2	-0.386459156	0.00036	0.091120764	0.39546	-0.220908789	0.04045
MCTS1	-0.37733155	0.00036	0.004508286	0.96514	-0.177555777	0.08929
FN3KRP	-0.393762477	0.00037	-0.261908426	0.01509	-0.162505396	0.13399
SLC2A10	-0.542949565	0.00037	-0.563323706	0.00021	-0.15886625	0.2949
C14orf105	-0.988287498	0.00037	-0.489068468	0.07674	-0.798784033	0.0039
MGME1	-0.535257713	0.00037	-0.01128059	0.9387	-0.240187109	0.10555
MIDN	0.596773426	0.00037	-0.214800648	0.20455	0.167921624	0.31867
CYP2C18	0.995533749	0.00037	0.384463128	0.16993	0.952907861	0.00066
BGLAP	2.136971622	0.00037	-0.093463053	0.89035	1.329337152	0.03154
PIAS3	0.433327898	0.00037	-0.012612061	0.91809	0.234544669	0.05549
LSM6	-0.459878949	0.00038	0.169322622	0.17258	-0.095778111	0.44746
PLEKHA4	0.756729724	0.00038	0.104228098	0.625	0.559941647	0.00871
PLIN3	0.584900403	0.00038	0.07703218	0.63998	0.184898986	0.2629
SIN3A	0.341315739	0.00037	0.13508269	0.15799	0.200561658	0.03675
ATG9A	0.496390863	0.00038	0.450181664	0.00128	0.071530817	0.60944
DDR1	0.425089217	0.00038	0.155693567	0.19319	0.336970136	0.00484
DNAJC19	-0.444910767	0.00038	0.090384894	0.45883	-0.103561146	0.40131
SLC44A3	-0.672633766	0.00038	-0.297978807	0.11339	-0.200467951	0.28745
P11-404P2	2.417771539	0.00038	1.906559278	0.00533	1.619407858	0.01895
PLCE1	-0.460978942	0.00038	-0.411746108	0.00145	-0.024290448	0.85061
CAND1	-0.327536738	0.00038	-0.020716961	0.82185	-0.219450289	0.01726
SPATA6	-0.653384779	0.00039	-0.059664144	0.74121	-0.328934331	0.07046
LAMTOR4	-0.446118786	0.00039	-0.108829738	0.3709	-0.103321136	0.39983
PEX11A	-0.705624193	0.00039	-0.266656257	0.17037	-0.194215417	0.32165
TRIM16L	-0.677100524	0.00039	-0.524102142	0.00479	-0.358762835	0.05187

VPS53	0.572838825	0.00039	0.215129733	0.18343	0.43388645	0.00725
HEMK1	-0.55745917	0.0004	-0.387382469	0.01414	-0.487826299	0.00191
SLC25A40	-0.417569524	0.0004	0.120021008	0.29802	0.062369218	0.59099
LDHB	-0.40875861	0.0004	-0.038386991	0.73916	-0.145476504	0.2073
PIP5K1C	0.546378185	0.0004	0.07657699	0.62494	0.042527808	0.78459
METTL15	-0.405606523	0.0004	-0.001309788	0.99069	-0.195006569	0.08582
SLC8B1	0.83407038	0.00041	0.305510172	0.19675	0.403656543	0.08793
RAB35	0.449298471	0.00041	0.111705469	0.37995	0.308368855	0.01573
E2F1	-0.626938804	0.00041	-0.434367028	0.01325	-0.401720169	0.02238
TUBB6	0.74079572	0.00041	0.358304145	0.08754	-0.054296988	0.79649
SAMM50	-0.367456621	0.00041	0.122406791	0.22795	-0.02901405	0.77723
CNN2	0.47326361	0.00041	0.038649244	0.77314	-0.032413641	0.80915
SP4	-0.507365083	0.00041	-0.33408356	0.01862	-0.366861518	0.01022
ACTR1A	0.323611204	0.00041	0.195008645	0.03293	0.146166689	0.11179
COMMD8	-0.590214975	0.00041	0.092636307	0.56303	-0.298260701	0.06845
P11-139J15	-2.971671753	0.00042	0.140002677	0.83253	-4.190723946	5.69E-05
RPS8	-0.404505023	0.00042	0.1672023	0.14418	-0.254795839	0.02612
HS3ST3B1	0.865566387	0.00042	0.117927853	0.63721	-0.043599592	0.86348
TNFAIP6	1.603998711	0.00042	1.15140473	0.01157	0.781887898	0.08981
WDR33	-0.336819871	0.00042	-0.103740884	0.27595	-0.176441294	0.06466
GGH	-0.604032756	0.00042	0.337461722	0.04543	-0.47381029	0.00545
BMP10	3.08945586	0.00042	0.534328335	0.56453	0.414216344	0.65925
ZNF532	0.521662903	0.00042	0.324370767	0.02849	0.168723362	0.25539
UQCRH	-0.331673889	0.00043	0.112552012	0.22688	-0.169305229	0.07094
LTB4R	0.748003298	0.00043	-0.207706478	0.34252	0.764563637	0.00031
ALDH5A1	-0.653291407	0.00043	-0.400035451	0.02879	-0.308048485	0.09378
RIF1	-0.252739818	0.00043	-0.057927016	0.41669	-0.113798857	0.11161
ZRANB3	-0.651451591	0.00043	-0.526151332	0.00398	-0.81968703	8.95E-06
RPS6KA2	0.988373599	0.00043	0.446347317	0.11368	0.695373216	0.01379
TTC13	-0.599301212	0.00043	0.09374917	0.57674	-0.025619023	0.87935
METTL9	-0.570598198	0.00044	-0.04164616	0.79631	-0.579907129	0.00035
UHRF2	-0.434182884	0.00044	-0.166425595	0.17394	-0.259279152	0.0347
NIPSNAP1	-0.405400662	0.00044	-0.151441165	0.18512	-0.520191061	6.45E-06
PPP1R12C	0.513321197	0.00044	-0.094056727	0.52344	0.343009066	0.01901
SCNN1G	-2.256484839	0.00044	-2.088874684	0.00095	-0.862399344	0.15919
PIGK	-0.406436417	0.00044	0.169993701	0.13263	-0.066624226	0.55881
ATM	-0.431794367	0.00044	-0.296153225	0.01566	-0.325122047	0.0081
ARL6	-0.761906259	0.00044	-0.255628433	0.22058	-0.343883426	0.10598
ZNF124	-0.361575916	0.00045	-0.114259374	0.2623	-0.159028735	0.12047
RPL7A	-0.405927537	0.00045	0.067201204	0.56051	-0.152975232	0.18545
GPR107	0.385657798	0.00045	0.080300672	0.4649	0.465408673	2.18E-05
P11-570P14	-2.366793641	0.00045	0.245339699	0.65299	-3.55684464	1.53E-05
FANCM	-0.607547131	0.00045	-0.056806655	0.73917	-0.211716116	0.21931
NACA	-0.367146049	0.00045	0.110605537	0.28981	-0.181807028	0.08224
TTC3	-0.2688192	0.00045	0.026787596	0.72628	-0.260433556	0.00067
PMAIP1	0.98670473	0.00046	0.87783775	0.00179	0.172101944	0.54423
HNRNPF	-0.306114511	0.00046	0.108631275	0.21114	-0.126196927	0.14752

THOC7	-0.35284874	0.00046	0.282805645	0.00378	-0.002471408	0.98008
ZNF24	-0.263359283	0.00046	0.090083754	0.22627	-0.056800417	0.44781
NDUFB5	-0.510982191	0.00046	-0.032140032	0.82372	-0.148264679	0.30564
MYD88	0.700062696	0.00046	0.622837579	0.0018	0.449639827	0.02518
NMNAT2	1.377697223	0.00046	0.995635509	0.01234	1.435204617	0.00034
RALGPS1	-0.660664881	0.00046	-0.687143585	0.00025	-0.160216359	0.39025
RAB3D	-0.574670297	0.00047	-0.380123525	0.02012	-0.216804968	0.18513
TRIAP1	-0.528579463	0.00047	-0.115906904	0.43415	-0.412871041	0.00597
FAM173B	-0.547408687	0.00047	0.032391408	0.82905	-0.378079899	0.01441
RNF145	0.374416824	0.00047	0.41609763	9.75E-05	0.305387357	0.00439
PLCD1	-0.66561032	0.00048	-0.705147442	0.0002	-0.349904655	0.06396
BMF	0.655199561	0.00048	0.197737233	0.29203	0.4447425	0.01793
HOXB4	0.94060957	0.00048	-0.012892602	0.96247	-0.027078525	0.92163
PDP1	0.539543287	0.00048	0.808349772	1.38E-07	0.358616056	0.02033
GEMIN5	-0.395362776	0.00049	0.069520197	0.5322	-0.265545668	0.01852
GNPDA1	-0.379685809	0.00049	0.12497173	0.23707	-0.289840609	0.00729
GPR108	0.478660258	0.00049	0.210511072	0.12527	0.345710253	0.01192
ATP6V0D1	0.346148653	0.00049	0.1581156	0.11124	0.176265962	0.07692
SPIRE1	0.893027358	0.00049	0.592627977	0.02187	-0.018418839	0.94375
CCL28	1.160352431	0.00049	0.396357021	0.24099	0.915453721	0.00625
FUT9	-1.845292927	0.0005	0.133663767	0.80007	-1.364384307	0.00989
EEF1A1P22	-1.08411427	0.0005	-0.106712576	0.69475	-0.535882996	0.06267
MRRF	-0.448300007	0.0005	0.146777821	0.24951	0.049158039	0.70046
SLC25A24	-0.316824486	0.0005	-0.050807888	0.57423	-0.003043476	0.97318
FARSB	-0.392346876	0.0005	0.114653959	0.30179	-0.152581893	0.17273
RAB5B	0.220471441	0.0005	0.070356137	0.26597	0.258320527	4.33E-05
NDFIP1	-0.28919216	0.0005	-0.037638669	0.64643	-0.067601363	0.4124
ERP29	-0.461521477	0.0005	-0.062053662	0.63851	-0.131724529	0.31919
LOXL2	0.778392259	0.0005	0.400631487	0.07337	0.129644522	0.56312
RNF217	0.784811501	0.0005	0.115784814	0.60768	0.003331481	0.98828
RP11-583M2	-2.219264838	0.0005	-0.020082915	0.97136	-1.318498899	0.02606
BTBD19	0.566708855	0.0005	-0.635661981	0.00018	0.139837762	0.39638
GGT5	1.050728527	0.0005	0.059314583	0.84675	0.381324609	0.21579
PET100	-0.468559603	0.00051	-0.084652466	0.51989	-0.092532586	0.48443
COA1	-0.299767923	0.00051	0.017932499	0.833	-0.253893675	0.00318
MLLT11	0.559830983	0.00051	0.26843359	0.09588	-0.088482416	0.58604
WNT9A	1.499092683	0.00051	1.321913432	0.00216	1.126473502	0.01046
LYZ	-1.155264706	0.00051	-0.437350619	0.18827	-0.456076507	0.17005
SLAIN1	-0.705406347	0.00051	0.137291183	0.49259	-0.204995117	0.30825
PSMD1	1.019698325	0.00051	0.47199842	0.10721	0.926939077	0.00157
SCYL1	0.434089392	0.00051	0.098714286	0.43	0.081428008	0.5166
TMPRSS5	-1.065525648	0.00051	-1.072375516	0.00044	-0.323022593	0.2865
ANKRD18C	-1.178742638	0.00052	-0.170357328	0.57136	-0.560083764	0.07513
C6orf48	-0.41831838	0.00052	-0.209365235	0.08026	-0.173649312	0.14757
STOX1	-0.719928756	0.00052	-0.164676367	0.39635	-0.473324909	0.0188
DCLK3	2.25948189	0.00052	0.852446932	0.19626	0.487169689	0.46514
MGAT5	-0.390371454	0.00052	-0.16260806	0.14736	-0.039548307	0.72459

TRIP10	0.430388836	0.00052	0.207444098	0.09428	0.461178398	0.0002
CASP8AP2	-0.340467393	0.00053	-0.015574813	0.87178	-0.160419345	0.09929
EEF2K	-0.517037575	0.00053	-0.677394145	5.38E-06	-0.191125147	0.19836
THRA	-0.406730131	0.00053	-0.610932328	1.99E-07	-0.112327904	0.33477
NAMPT	1.099984798	0.00053	1.038986769	0.00106	0.870480655	0.0061
FUNDC1	-0.461671822	0.00053	0.261452433	0.04187	-0.007726432	0.9527
MED25	0.52045506	0.00053	-0.476641181	0.00211	0.43229664	0.00417
SAPCD2	-0.815314753	0.00054	-0.120774912	0.60346	-0.19192282	0.41027
GLI2	0.863790474	0.00054	-0.143233771	0.57031	-0.034973399	0.88949
RABGGTB	-0.304975583	0.00054	-0.060993284	0.48472	-0.098661061	0.26026
CHEK1	-0.651797848	0.00054	-0.242060085	0.19589	-0.414663251	0.0273
CD248	0.717228365	0.00054	-0.352384836	0.09228	-0.001831525	0.993
GCSH	-0.526898376	0.00055	0.046823052	0.75463	-0.30536477	0.04361
TGFBR1	0.457969825	0.00055	0.419675506	0.0015	0.126517613	0.34068
FKBP3	-0.357671551	0.00055	0.042207849	0.67859	-0.248347889	0.01594
RBBP7	-0.251379008	0.00055	0.013330907	0.85362	-0.06395371	0.37776
DBF4	-0.413619173	0.00055	0.209005789	0.07408	-0.179518751	0.13043
ELP4	-0.377618552	0.00056	-0.054198048	0.60811	-0.261659206	0.0164
HADHB	-0.252526312	0.00055	-0.072865379	0.31228	-0.265797077	0.00026
HM13	0.322751539	0.00055	0.429068527	4.20E-06	-0.026092739	0.78089
NXN	0.786835169	0.00056	0.267845175	0.24065	-0.246058046	0.28421
OGFR	0.49959119	0.00056	0.027732812	0.8491	0.191762098	0.18697
OMA1	-0.666067953	0.00056	0.285152638	0.13196	-0.138414041	0.46776
TP53	0.82363525	0.00056	-0.04380636	0.85449	0.63394969	0.00798
RUSC2	0.567734244	0.00056	-0.215936185	0.19593	0.253705036	0.12528
MCPH1	-0.36569832	0.00056	0.092646797	0.37496	-0.139433888	0.18728
C8orf33	-0.512820086	0.00056	-0.132849382	0.36552	-0.424797297	0.00408
DAB2	0.908383361	0.00057	0.495384864	0.06039	0.300263675	0.25639
EHHADH	-0.831080796	0.00057	-0.152046782	0.52436	-0.259713989	0.27676
CCT6A	-0.339015978	0.00057	0.132959377	0.1744	-0.185484107	0.05895
PAQR6	0.865569155	0.00057	-0.114564652	0.66219	0.02519408	0.92257
SMIM20	-0.479070406	0.00057	-0.121639935	0.36709	-0.056999554	0.6744
TANK	0.407180855	0.00057	0.41558111	0.00042	0.460056209	9.97E-05
SNCAIP	0.848846734	0.00058	0.404972546	0.1007	-0.076308066	0.75995
GPR114	-1.260470349	0.00058	-1.079451148	0.00271	-0.804188355	0.02266
TMEM109	-0.474628381	0.00058	-0.255360493	0.06233	-0.357449877	0.00928
TMEM59	-0.405739524	0.00059	-0.034595375	0.76874	-0.208342994	0.07706
WDFY3	0.449629016	0.00059	0.079736327	0.54199	0.106895728	0.41401
SLC30A5	-0.309033307	0.00059	-0.085665528	0.3338	-0.153958517	0.08451
OPA3	0.397272786	0.00059	-0.052290604	0.65281	0.315965043	0.00631
ARHGEF17	0.716736611	0.00059	0.022928675	0.91284	-0.000373755	0.99858
MYO5C	-0.570424148	0.0006	-0.276834576	0.09417	-0.144630869	0.38195
PKNOX2	1.1578937	0.0006	0.618919362	0.06825	0.545123815	0.10861
MRE11A	-0.381019446	0.0006	-0.198053484	0.07082	-0.369753312	0.0008
TBC1D4	-0.660172005	0.0006	-0.627613449	0.00104	-0.337159665	0.07846
ACSS1	-0.738097915	0.0006	-0.478367894	0.02509	0.000732928	0.99725
P11-85G20	1.332465699	0.00061	0.264670729	0.52913	1.904030219	3.77E-07

GFRA1	1.268315159	0.00061	1.053808589	0.00438	0.369785374	0.31917
SFMBT2	0.763937064	0.00061	0.270773482	0.22742	0.120531899	0.59287
MT-ATP6	0.908107386	0.00061	0.592158274	0.02545	1.228418809	3.53E-06
FAM222A	0.917181727	0.00061	0.019647545	0.943	0.217537161	0.42666
GBP1	1.062246766	0.00061	0.846131992	0.00637	0.191864926	0.53717
TRIM69	0.638905858	0.00062	0.740475818	7.00E-05	0.496992634	0.00777
CDK1	-0.540568339	0.00062	-0.026080849	0.86806	-0.327200208	0.03778
IL1A	2.437287445	0.00062	2.229835403	0.00174	1.829876263	0.01025
SP2	0.872953213	0.00062	-0.199727932	0.45995	0.348733325	0.18342
EXT1	0.400311447	0.00062	0.293518117	0.01205	0.117934759	0.3144
ATP5A1	-0.31975035	0.00062	0.05416782	0.56137	-0.078221682	0.40207
BTN3A2	-0.541581648	0.00062	-0.086788355	0.57487	-0.224806826	0.15094
RHOQ	0.577987824	0.00063	0.350757644	0.03772	5.34E-05	0.99975
WDR1	0.340284655	0.00063	0.316983215	0.00144	0.233246983	0.01913
SLIT3	1.155594315	0.00063	-0.121312593	0.72084	0.421973695	0.21423
NCKAP5L	0.499533289	0.00064	-0.373761833	0.01226	-0.067454173	0.64979
LRRC32	0.902430657	0.00064	0.507071973	0.05666	0.531882825	0.0457
EMD	0.455842541	0.00064	0.326550858	0.01396	0.297104761	0.02698
ZNF485	-0.724193598	0.00064	0.003045193	0.9878	-0.369626078	0.07201
ANKRD36E	0.62695245	0.00064	0.441063259	0.01623	0.512827558	0.0053
CLDN12	0.445941737	0.00064	0.326980812	0.01207	0.475584772	0.00027
PTGIS	0.643958147	0.00064	0.059120587	0.7545	-0.0244474	0.8972
TMEM74B	1.261945491	0.00064	0.758325028	0.04142	1.247826725	0.00073
RNF168	-0.34171048	0.00064	-0.200857737	0.04307	-0.122646463	0.21682
CTNNAL1	-0.358911519	0.00065	0.187824902	0.06892	-0.249565623	0.0172
POGK	-0.281813409	0.00065	-0.112844356	0.16805	-0.114262959	0.16424
C2CD5	-0.531617834	0.00065	-0.232602825	0.13328	-0.164720266	0.2887
RFWD2	-0.325280152	0.00065	-0.080626207	0.3933	-0.146752025	0.12245
MLLT4	0.305746734	0.00065	-0.151508584	0.09122	0.29065407	0.00119
ARHGAP21	0.443716151	0.00066	0.321298387	0.01367	0.338260166	0.00945
NUDT9	-0.467692446	0.00066	-0.021713157	0.87202	-0.158148822	0.24596
MCM8	-0.585135077	0.00067	0.026740853	0.87324	-0.283276146	0.09513
FAM133B	-0.357674306	0.00067	0.060415625	0.55885	-0.211359518	0.04251
GSTA1	-1.420174599	0.00067	-0.419627247	0.31353	-0.387380188	0.35223
COX16	-0.417300653	0.00067	-0.060601727	0.61589	-0.154539778	0.20337
PLK2	0.778699722	0.00067	1.093546478	1.78E-06	0.10608498	0.64437
ZNF92	-0.386111756	0.00067	-0.093961362	0.40038	-0.259745673	0.02134
NOC3L	-0.3545088	0.00068	0.025749993	0.80017	-0.341541767	0.001
PTBP3	0.459888631	0.00068	0.55880288	3.59E-05	0.41660739	0.00207
RND3	0.543544982	0.00068	0.734626716	4.23E-06	0.265995053	0.09668
TMEM163	0.752017746	0.00068	0.677805056	0.00219	0.854657318	0.00011
PLEKHB2	0.307287558	0.00068	0.379821936	2.51E-05	0.357558626	7.58E-05
C9orf116	-0.803636704	0.00068	-0.380173913	0.08976	-0.232094347	0.30386
ZMIZ1	0.747985441	0.00069	-0.442795795	0.04591	-0.024190264	0.91281
FAM35A	-0.3544152	0.0007	0.258164597	0.01124	-0.106671973	0.30225
FOXRED1	-0.447806673	0.0007	-0.358458476	0.00581	-0.182924542	0.15896
SH3RF3	1.009466424	0.0007	0.422100969	0.16111	0.072807489	0.8124

E2F2	-0.975871893	0.00071	-0.743587989	0.00951	-0.610944387	0.03282
ANAPC4	-0.524298982	0.00071	-0.301762335	0.04816	-0.410600285	0.00771
CDK9	0.493584193	0.00071	-0.123770577	0.39727	0.346358339	0.0177
RTKN2	-0.581585272	0.00071	-0.236129865	0.16217	-0.446947445	0.00877
WIPF2	0.525531821	0.00071	-0.206552047	0.18515	0.368832586	0.01757
ZNF121	-0.341013713	0.00072	-0.031832484	0.75088	-0.249118031	0.01337
LPO	2.186889479	0.00072	-0.175869528	0.79707	2.405736293	0.00019
VPS29	-0.299973061	0.00072	0.211913399	0.01493	-0.151418591	0.08589
RAD54L2	0.320240513	0.00073	-0.208790431	0.02876	0.040540545	0.67023
CCT8	-1.382698698	0.00073	-0.184696556	0.65041	-0.867968712	0.03349
SMC6	-0.340359797	0.00073	0.074867746	0.44864	-0.206489432	0.03926
BRIP1	-0.568132682	0.00073	-0.345240211	0.03866	-0.318246644	0.05803
THOC3	-0.470617358	0.00073	-0.083962412	0.53872	-0.15983788	0.24526
RBMX2	-0.549827404	0.00074	-0.168020756	0.2923	0.008668908	0.95661
MRPL24	-0.4452011	0.00074	-0.129963201	0.31429	-0.012794535	0.92113
ZNF813	-0.622437391	0.00074	-0.261705079	0.15193	-0.237110603	0.19479
SEMA5A	1.087923275	0.00074	-0.302531551	0.34955	-0.267309174	0.40901
RAB43	1.643394423	0.00075	0.380717232	0.43621	0.886942024	0.0693
GCA	-0.62400814	0.00075	-0.186097245	0.30726	-0.122699907	0.50209
CCSER1	-0.615063779	0.00075	-0.298689681	0.09977	-0.02327526	0.89781
MAOA	-0.753600746	0.00075	-0.240139418	0.28163	-0.073206987	0.74278
PSMD3	0.250004453	0.00075	0.123092297	0.09609	-0.023971588	0.74805
CREBZF	-0.330265859	0.00076	-0.336588955	0.00058	-0.106988413	0.27271
CLIC4	0.446613926	0.00076	0.499110107	0.00017	0.171368369	0.19645
CDC42SE1	0.347940706	0.00076	0.056848553	0.5824	0.227415106	0.0279
RDH10	0.593267604	0.00076	0.506539425	0.00403	0.311225181	0.07748
ANKMY2	-0.365162726	0.00076	-0.069988125	0.51254	-0.084787084	0.42935
RAB13	0.424515778	0.00076	0.320142191	0.01104	0.299739945	0.01768
RTN4	0.282333644	0.00077	0.156994187	0.06122	0.185429768	0.02732
FAM160B2	0.442314328	0.00077	-0.311686539	0.01985	0.096330145	0.46722
BRD4	0.589918712	0.00077	-0.141155619	0.42359	0.324522292	0.06491
PSMB9	1.2778389	0.00077	1.430768898	0.00015	0.464125243	0.22628
GNPDA2	-0.385001031	0.00077	0.138790951	0.21657	-0.327278251	0.00431
FDX1	-0.444759129	0.00078	-0.11887811	0.36069	-0.114446822	0.38079
SKP2	-0.459473683	0.00078	-0.054477593	0.68682	-0.461248561	0.00071
IMPDH2	-0.386075482	0.00078	0.215283045	0.05978	-0.121644581	0.28856
ZSWIM4	0.762616406	0.00078	0.277952699	0.22747	0.27328095	0.23696
KLF7	0.919475988	0.00078	0.421274823	0.12454	0.515049564	0.06061
UGCG	0.64121699	0.00078	0.474464529	0.01292	0.143858155	0.45165
IMPDH1	-0.655217699	0.00078	-0.641713525	0.00097	-0.107049414	0.58119
SYPL1	-0.32878057	0.00078	0.016259009	0.86657	0.074015489	0.44573
PDIA3P1	-0.751737311	0.00079	-0.49409251	0.02318	-0.000169439	0.99937
OXCT1	-0.569174677	0.00079	0.030080436	0.85596	-0.43481511	0.00998
RALGDS	0.455271814	0.00079	0.167908112	0.21746	0.149097378	0.27572
POLR3GL	-0.482570829	0.00079	-0.280640084	0.04448	-0.169951254	0.22483
CLIP1	0.317808798	0.00079	0.086842869	0.35843	0.259018963	0.00622
ADAMTSL5	1.064670885	0.0008	0.722236881	0.02396	1.363891584	1.78E-05

TMA7	-0.257518817	0.0008	0.205906722	0.00639	-0.15802708	0.03874
PLIN5	1.178432364	0.0008	0.690256795	0.05328	0.945334637	0.00775
CHST15	1.018607822	0.0008	0.845754106	0.00539	-0.169840507	0.57922
SNRNP48	-0.336926426	0.0008	-0.217480641	0.02809	-0.258908038	0.00956
CWF19L2	-0.361792013	0.00081	0.052517376	0.61562	-0.04181805	0.69363
DOK3	1.019008382	0.00081	-0.178509676	0.58635	0.651996774	0.03627
SETD1B	0.758558805	0.00081	-0.169594594	0.46713	0.562525459	0.01342
GINS1	-0.529907393	0.00081	-0.168022677	0.28276	-0.325925082	0.03814
IDH1	-0.416919154	0.00081	-0.094585946	0.44675	-0.001771989	0.98863
HAT1	-0.372519825	0.00081	0.151832664	0.1665	-0.195423938	0.07749
CD1D	1.080062951	0.00082	0.789789944	0.01456	0.414836491	0.20471
IMPACT	-0.42364684	0.00082	0.245186837	0.04893	-0.0740206	0.55462
ASB9	-1.194929443	0.00082	-0.685955072	0.04928	-0.822729787	0.01931
HIBCH	-0.63982317	0.00082	-0.39338099	0.03773	-0.454765718	0.01674
CINP	-0.319638183	0.00083	-0.101196129	0.27913	-0.036609466	0.69656
PRRT2	1.063709089	0.00083	0.140411081	0.66501	0.619928248	0.05385
AFF4	0.444033456	0.00083	0.374218179	0.00484	0.324289127	0.01467
CALM2	-0.32528925	0.00083	0.133226759	0.17036	-0.085998874	0.37653
DENND5A	0.556576623	0.00083	0.230364814	0.16684	0.00950842	0.9546
RPL6	-0.382359168	0.00083	0.21784015	0.05674	-0.293981843	0.01018
SPAG7	-0.244785751	0.00083	-0.009040076	0.89934	-0.11456506	0.11489
3-Mar	0.786311385	0.00084	0.940805957	5.66E-05	0.643414714	0.00642
HSPE1P3	-2.279927015	0.00084	0.766338846	0.23937	-0.499990471	0.44714
ULK1	0.714615174	0.00084	-0.141447176	0.51777	0.138679119	0.52139
TEAD2	0.567671958	0.00085	0.270698331	0.11156	-0.108217247	0.52635
PPM1D	-0.32461485	0.00085	-0.066063703	0.4883	-0.105302334	0.27359
C9orf152	-1.112048204	0.00085	-0.240562448	0.4643	-0.316875649	0.33613
RUVBL1	-0.415137411	0.00086	0.076650407	0.53155	-0.269822982	0.02907
HEBP2	-0.350208756	0.00086	-0.03433853	0.74204	-0.059080129	0.57177
AVPI1	-0.970079055	0.00087	-0.304755695	0.2831	-0.796094859	0.00588
MEX3C	0.691528612	0.00087	0.548584852	0.00822	0.655077712	0.00161
PTDSS1	-0.34890697	0.00087	0.099972778	0.3351	-0.165905895	0.11194
BMP5	1.100744806	0.00088	0.621256955	0.06115	0.702412052	0.03439
HSF1	0.48922354	0.00088	0.002754924	0.98515	0.344302383	0.01945
MRPL19	-0.380445974	0.00088	0.242526955	0.03127	-0.01790434	0.87459
CCDC71L	0.690156861	0.00088	0.288168428	0.16613	-0.059783046	0.77583
SIPA1L2	0.612088249	0.00088	0.406067053	0.02732	0.230176305	0.21157
ADH5	-0.378106717	0.00088	-0.306227054	0.0068	-0.274476293	0.01556
FAM208A	-0.31627976	0.00088	0.007219606	0.93915	-0.184560486	0.05193
HEXB	-0.495129737	0.00088	-0.04816625	0.74569	-0.033147111	0.82349
ANAPC5	-0.343172633	0.00089	-0.133229935	0.1946	-0.138350314	0.17907
GJC2	-0.982266476	0.00089	-1.195198506	5.45E-05	-0.812670071	0.00524
EXOSC8	-0.405904771	0.00089	-0.129544958	0.28402	-0.213332345	0.07881
NUP54	-0.356629923	0.00089	0.1172672	0.26803	-0.194124959	0.06817
ARHGAP24	-0.658506774	0.00089	0.098131714	0.61368	0.119213883	0.54024
BRCC3	-0.351459334	0.0009	0.135921219	0.18928	0.010328699	0.92121
ARSD	-0.405284702	0.0009	-0.393740698	0.00121	-0.278282217	0.02203

SHC1	0.386944337	0.0009	0.315784226	0.00675	0.105700708	0.36607
IQGAP2	-0.67069868	0.0009	-0.278524607	0.16757	0.102040508	0.61296
PRKACA	0.664622622	0.0009	0.033721644	0.86789	0.420824329	0.03636
AZI2	0.384701197	0.00091	0.504405403	1.24E-05	0.43643621	0.00017
DBP	-1.204316317	0.00091	-0.902308383	0.01338	-0.883004978	0.01474
DTWD1	-0.376099397	0.00091	0.075827058	0.49688	-0.256062763	0.02335
U2SURP	-0.329233536	0.00091	-0.019206981	0.84578	-0.168269192	0.08917
ARHGEF34	0.550287183	0.00091	-0.148218871	0.37437	0.745740999	6.64E-06
VGLL4	0.294746792	0.00091	-0.133117621	0.13372	0.196376079	0.02709
CPNE3	-0.266090829	0.00092	0.202444962	0.01082	0.039913707	0.61683
EIF4G3	0.273696785	0.00092	0.016384514	0.84249	0.128602566	0.11949
UBL5	-0.380387424	0.00092	-0.010697936	0.92491	-0.197756559	0.08342
EHD4	0.380773799	0.00093	0.446670214	8.82E-05	0.391788955	0.00064
PRIMPOL	-0.706106975	0.00093	-0.113147568	0.5857	-0.720444338	0.00061
TMEM173	1.023012147	0.00093	0.361316693	0.24361	0.535502231	0.08456
CLIP3	1.075291126	0.00094	-0.204411676	0.53905	0.429093298	0.19297
COQ3	-0.765617763	0.00094	-0.102447687	0.63886	-0.624671267	0.00605
H2AFV	-0.427522863	0.00094	-0.140594053	0.27539	-0.241131693	0.06195
ZNF22	-0.422884153	0.00095	0.105732704	0.40129	-0.214731371	0.09121
TAF4B	-0.739352576	0.00095	0.070856007	0.74136	-0.23910193	0.27331
PSMA1	-0.31207614	0.00096	0.270237952	0.00383	-0.261056949	0.00563
CDK6	0.510806551	0.00096	0.387043267	0.0123	-0.04263159	0.78306
PSD	1.041266014	0.00096	0.375545495	0.24203	-0.136396325	0.6785
PPAP2C	-0.869562961	0.00097	-0.403358489	0.12091	-0.651480661	0.01267
VMA21	-0.283010754	0.00097	0.080058554	0.34507	-0.247224712	0.00384
NR1D2	-0.830186134	0.00098	-0.705752788	0.00485	-0.759917645	0.00249
EXOC7	0.312093081	0.00098	0.112029198	0.23688	0.038672774	0.68327
CENPK	-0.536095529	0.00099	-0.256062189	0.10906	-0.253043623	0.11546
ARL8B	0.934897311	0.00099	0.678923987	0.01659	0.799494195	0.00478
CCDC34	-0.512825432	0.00099	0.029244787	0.84783	-0.258477856	0.09306
ATP11B	-0.323105293	0.00099	0.015821716	0.87158	0.132110009	0.17712
MCM7	-0.509644106	0.00099	-0.258211532	0.0943	-0.310923465	0.04432
RAC2	0.957452439	0.00099	0.204689957	0.48473	0.328377048	0.26245
SORBS2	-0.774229814	0.00099	-0.125567176	0.58827	-0.256885713	0.2694
SAE1	-0.34101557	0.001	-0.076278324	0.45958	-0.307030532	0.00302
ANAPC1	-0.362531027	0.00101	-0.030717967	0.77922	-0.179861772	0.1016
SLC35F6	0.303484501	0.00101	-0.05153684	0.5795	0.010893292	0.90707
CTGF	1.116922777	0.00102	1.041471675	0.00219	0.441596615	0.19498
IMMP2L	-0.677226126	0.00103	-0.185271555	0.35152	-0.236131294	0.24153
C19orf66	0.582020317	0.00103	0.187648539	0.29362	0.398097399	0.02561
DVL3	0.337137206	0.00103	-0.183095046	0.07762	0.100972802	0.32805
LIN7C	-0.304695554	0.00104	-0.024384089	0.79149	-0.087442046	0.34435
FHOD3	1.721556498	0.00104	0.934200537	0.07945	0.623916299	0.27004
PLXNB2	0.540589263	0.00104	0.047531337	0.77322	0.341075824	0.03857
TOLLIP	0.442514268	0.00104	-0.146990681	0.27815	0.13805328	0.30994
CDC6	-0.656287352	0.00104	-0.130390894	0.51177	-0.463413099	0.02015
JAG1	0.616841254	0.00105	0.596960402	0.0015	0.335427767	0.0749

RAB7A	0.604801305	0.00106	0.96794251	1.54E-07	0.651342219	0.00042
ZNF443	-0.643726887	0.00106	-0.311646997	0.10403	-0.481816735	0.01319
SYNPO	1.300573348	0.00106	0.511632862	0.19897	0.453972691	0.25473
PGGT1B	-0.379036043	0.00106	0.063061578	0.58197	-0.001986837	0.98622
SLC19A2	-0.378132765	0.00106	0.045821375	0.68674	-0.102552242	0.37011
POT1	-0.528185328	0.00106	-0.014090024	0.92916	-0.330480934	0.03907
KDM4B	0.609027449	0.00107	0.09536257	0.60722	0.236203662	0.20338
CYLD	0.450686865	0.00107	0.171062101	0.21383	0.207340124	0.13485
TMEM19	-0.675683368	0.00107	-0.174522354	0.39485	-0.422533391	0.04001
ALDH18A1	-0.463513263	0.00107	-0.017448115	0.90182	-0.26996076	0.05649
SIRT6	0.605962302	0.00107	0.050165197	0.79151	0.260223502	0.16491
ANKRD11	0.363096082	0.00108	-0.241135072	0.03069	0.089533372	0.42165
SLC27A5	-0.545628406	0.00108	-0.27329984	0.09989	-0.393900681	0.01785
FAU	-0.332634861	0.00108	0.325819715	0.00128	-0.116306481	0.252
ALOX5	1.607460186	0.00109	0.952375719	0.05349	2.070276269	2.48E-05
DSP	-0.423768735	0.00109	-0.465830986	0.00033	-0.38486436	0.00301
SASS6	-0.56884907	0.00109	-0.014942914	0.9306	-0.196648819	0.25445
AIMP1	-0.373729917	0.0011	0.129938921	0.24673	0.030438238	0.78788
FAM3B	-0.948820464	0.0011	0.12286474	0.6665	-0.025236627	0.9297
GPD2	-0.453798436	0.0011	-0.039322445	0.77662	-0.01518551	0.91284
ALG13	-0.482622528	0.00111	-0.241192798	0.09851	-0.073692463	0.61441
AUH	-0.593457187	0.00111	-0.281228061	0.11544	-0.203778985	0.25652
RTFDC1	-0.356603482	0.00111	0.052307127	0.62812	-0.252062231	0.02049
ADAMTSL2	1.379997114	0.00112	-0.219722971	0.63696	0.475321209	0.28477
CSPP1	-0.446080704	0.00112	-0.0589599	0.6621	-0.319037807	0.01926
GPR124	0.810922243	0.00112	-0.213557719	0.39169	-0.046117131	0.85324
MGAT1	0.309214744	0.00112	0.062517171	0.51066	0.122181845	0.20024
HNRNPDL	-0.253104246	0.00112	0.059606813	0.44167	-0.177496707	0.02222
TARSL2	-0.498092599	0.00112	-0.13701265	0.36333	-0.132370179	0.38209
RASA4B	1.151732549	0.00113	-0.271375759	0.46199	0.549480755	0.12712
CHML	-0.34501751	0.00114	0.171086	0.10083	0.068201383	0.51515
TTC39B	0.408463175	0.00114	0.583582993	3.11E-06	0.442300324	0.00042
DNAJB5	0.68298442	0.00114	0.230029343	0.27666	-0.18391826	0.39172
CAMLG	-0.447623323	0.00115	0.156509654	0.24556	-0.370010947	0.00678
PLCH1	-0.737885446	0.00115	-0.029310656	0.89617	-0.144880534	0.51982
PRSS12	0.965928017	0.00115	0.484178993	0.10335	0.843077512	0.00457
CARM1	0.373220534	0.00115	-0.167318327	0.14683	-0.059134855	0.60859
CTSV	-0.58986167	0.00115	0.095328295	0.58776	-0.152858977	0.38981
MAP3K9	0.506554556	0.00115	-0.182549299	0.24425	0.458024395	0.00328
SEC24B	0.235330853	0.00115	0.215874385	0.00272	0.267976335	0.00021
COX7C	-0.352858292	0.00116	0.046888281	0.66474	-0.104651442	0.33428
UBXN10	-1.176946768	0.00117	-0.50323204	0.136	-1.20792149	0.00084
CASP4	0.571802913	0.00117	0.601843505	0.00062	0.856079493	1.11E-06
CYB5B	0.278985431	0.00117	0.634499027	1.12E-13	0.243713666	0.00456
DARS	-0.313187908	0.00117	0.070051388	0.46461	-0.163188575	0.0898
FAM168A	0.285798927	0.00117	0.027310939	0.7562	0.156627883	0.07565
HINT3	-0.3014493	0.00117	0.080048691	0.37794	0.01439315	0.87494

PTX3	2.06697005	0.00117	1.554015057	0.01471	1.137570224	0.0744
TASP1	-0.419882166	0.00117	0.041587752	0.73852	-0.119950397	0.34224
P1-130H16.	4.469109534	0.00118	4.539622251	0.00097	3.746266686	0.00672
ATN1	0.876623339	0.00118	-0.032757562	0.90452	0.691058906	0.01067
CRTC2	0.711911348	0.00118	-0.026288293	0.90605	0.621185251	0.00473
DHODH	-0.51369588	0.00119	-0.148526521	0.34512	-0.416874595	0.00859
CHST11	1.080212335	0.00119	0.895160296	0.00716	-0.021937745	0.94784
MLLT1	0.369632007	0.00121	-0.167445707	0.14659	0.120458066	0.29427
CMTM6	-0.301069767	0.00122	-0.014959313	0.87173	-0.140500962	0.13025
IL1R1	1.035486314	0.00122	0.425300712	0.18413	0.308468414	0.33582
PDLIM4	0.720628208	0.00121	0.511747614	0.02156	0.038551992	0.86439
PIK3CG	1.472870608	0.00121	0.398719703	0.40117	0.556302738	0.24695
VAMP2	0.653027645	0.00122	0.000382167	0.99853	0.765976689	0.00013
COL4A3BF	-0.358745574	0.00122	-0.184465749	0.09304	0.020098075	0.85505
PXMP2	-0.60289063	0.00122	-0.388930199	0.03323	-0.370146068	0.04246
TMEM245	-0.28982047	0.00122	-0.190725221	0.03232	-0.114928661	0.19781
RPS17L	-0.525350737	0.00122	0.070136425	0.66567	-0.354784265	0.02895
MIS18BP1	-0.498982872	0.00123	0.006484704	0.96626	-0.42533312	0.00579
ANKLE2	0.403323858	0.00123	0.092086988	0.46045	0.149469256	0.23152
PRDX6	-0.321378094	0.00123	0.081801556	0.40869	-0.343802432	0.00054
C4orf27	-0.419148802	0.00124	-0.081365279	0.51836	-0.574989796	1.03E-05
FPGT	-0.498454058	0.00124	-0.402867906	0.00715	-0.158213998	0.29261
PDPN	1.594290987	0.00124	0.981029342	0.04872	0.673929617	0.17742
RCN2	-0.302254067	0.00124	0.179953058	0.0523	-0.226545831	0.01538
PPP1R3B	-0.518312429	0.00125	-0.415334305	0.00958	-0.113777501	0.4776
SPRR3	2.570134907	0.00125	1.692416435	0.0347	2.530695432	0.00148
FZP761J14	0.715822911	0.00125	-0.149645516	0.50654	0.390893873	0.08424
FANCB	-0.767359488	0.00125	-0.317588371	0.17236	-0.598167217	0.01116
PGRMC2	-0.565177253	0.00125	-0.111951073	0.51993	-0.045042198	0.79583
FBXL7	0.799823969	0.00126	0.150074489	0.54855	0.233402935	0.3508
NAA15	-0.33853918	0.00126	0.106154448	0.30803	-0.22275899	0.03336
CENPC	-0.321526794	0.00127	0.056710981	0.56342	-0.159341237	0.10678
AKT2	0.379264699	0.00128	0.015146859	0.89785	0.22505239	0.0563
F2RL2	0.715699412	0.00127	-0.091163263	0.68313	0.773769192	0.00049
FAM222B	0.673764617	0.00128	0.132377096	0.5295	0.622498115	0.00299
HAPLN3	1.182867927	0.00128	1.180539015	0.00124	0.607613863	0.10218
TLDC1	0.335599897	0.00128	0.213592169	0.03971	0.429900873	3.63E-05
NUMB	0.367673372	0.00129	0.108720431	0.34043	0.29564884	0.00954
ERCC6L	-0.684014506	0.00129	-0.053405086	0.7983	-0.352045519	0.09463
PCCB	-0.526242529	0.00129	-0.067953371	0.67503	-0.308945556	0.05776
ZSWIM6	0.343404662	0.00129	0.037301392	0.72769	0.262540949	0.01397
GEN1	-0.583572823	0.00129	-0.322770999	0.07394	-0.492005184	0.00668
FBRSL1	0.819096465	0.00129	-0.289594473	0.25919	0.400686385	0.11619
SH3KBP1	0.616917339	0.00131	0.679757848	0.00039	0.402787547	0.03593
SNRPE	-0.388139825	0.00131	0.043881322	0.71257	-0.268373812	0.02567
TNFSF18	1.352924108	0.00131	0.919144175	0.02923	-0.001063735	0.99801
CEBPZ-AS1	-0.893292015	0.00131	-1.284465712	3.64E-06	0.037143018	0.89141

NDUFS3	-0.327840872	0.00132	-0.094396429	0.34636	-0.006914443	0.9452
PLBD1	-0.726952706	0.00132	0.15441605	0.49036	-0.117605522	0.60059
CASP10	0.758925808	0.00132	0.989373954	2.68E-05	1.049834083	8.32E-06
PIK3CD	1.860380234	0.00132	1.180542813	0.04537	1.033266435	0.07874
PTGR1	-0.484294756	0.00132	-0.179647638	0.23136	-0.201880975	0.17926
NPLOC4	0.295138162	0.00133	0.252406004	0.00612	0.095990783	0.29821
PFDN4	-0.471490522	0.00133	0.187752564	0.17757	-0.703392811	2.05E-06
SLC16A1	-0.368866211	0.00133	0.203860666	0.07361	-0.116100477	0.31059
SOD1	-0.273699777	0.00134	0.347151375	3.48E-05	-0.129414734	0.12749
NSMCE4A	-0.386801092	0.00134	0.052785195	0.65515	-0.110245188	0.35597
AGO3	0.441470779	0.00134	0.165794477	0.22898	0.205234082	0.13624
TMEM141	-0.515869034	0.00135	-0.408616094	0.01087	-0.110881426	0.48907
OTUD5	0.409415293	0.00136	0.266510779	0.03698	0.183248292	0.15454
SLBP	-0.388684974	0.00136	0.187265002	0.11665	-0.239298015	0.04757
EEF1G	-0.384389747	0.00136	0.157906708	0.18801	-0.122823308	0.30596
EEF1B2	-0.388702034	0.00137	0.206249562	0.08867	-0.099594316	0.41154
HIP1	0.899433518	0.00136	0.275077051	0.32818	0.179241083	0.52518
FBLN1	0.464913568	0.00137	-0.011096361	0.93924	-0.125799509	0.38889
HAUS3	-0.338146996	0.00137	-0.106010802	0.30837	-0.193713101	0.06431
PPP1R16A	0.4771855	0.00138	-0.210211858	0.16297	0.425895518	0.00421
HELZ2	0.823867473	0.00138	0.313445079	0.22769	0.512702297	0.04775
PSMD4	-0.305429167	0.00138	0.006761475	0.94297	-0.229985586	0.01582
QRSL1	-0.364634314	0.00138	-0.10648562	0.34364	-0.119913086	0.28938
DIO3	2.092139499	0.00138	1.484547812	0.02347	0.869010371	0.18667
PAN-P2RY1	3.204805515	0.00139	-0.114183362	0.91462	2.131120107	0.0351
TXN	-0.404436254	0.00139	0.548090129	1.29E-05	-0.222955688	0.07749
PKD1P6	0.58110745	0.0014	-0.332520484	0.07349	0.122604918	0.504
RPS26	-0.422661141	0.0014	-0.037142913	0.77794	-0.021593597	0.86988
RPL34	-0.365761045	0.0014	0.158935254	0.1642	-0.202299558	0.077
ZNF204P	-0.59906128	0.00141	-0.060615689	0.74159	-0.241046139	0.19325
MMS22L	-0.710108547	0.00142	-0.487802542	0.02804	-0.312851695	0.15902
TAGLN2	-0.470648271	0.00143	-0.376485896	0.0107	0.079485633	0.58969
TUSC3	-0.380794265	0.00143	0.199799167	0.09212	-0.365700715	0.00218
ZNF573	-0.562568323	0.00143	-0.320721752	0.0632	-0.199011865	0.24975
OTUB1	0.252408461	0.00143	0.107568111	0.17524	0.151143817	0.05673
TTYH3	0.600201585	0.00143	-0.218610739	0.24721	0.08725125	0.64378
SZRD1	0.330503149	0.00144	0.18818361	0.06913	0.247275668	0.01711
C15orf48	1.177909596	0.00144	0.710536059	0.05509	0.995065147	0.00716
PDIA3	-0.374678848	0.00145	0.194318639	0.09834	-0.143312694	0.223
WDR45	0.540831774	0.00145	0.175171599	0.30413	0.173537943	0.30918
MNT	0.470278205	0.00145	-0.43341266	0.00469	0.105033252	0.48124
RECQL	-0.358563932	0.00146	0.187457022	0.09133	-0.370910467	0.00097
UBE2R2	0.191650038	0.00146	0.039016988	0.51626	0.073917515	0.22114
CCRL2	1.565105988	0.00146	1.433167485	0.00321	1.055429063	0.03386
MIS18A	-0.516652219	0.00148	0.031559947	0.8415	-0.270477822	0.09152
BAIAP2L2	1.200776946	0.00148	0.863630078	0.02265	0.930972675	0.01407
EGR2	1.137172169	0.00148	0.095443175	0.79189	0.25805219	0.47553

LDLRAD4	0.962863222	0.00148	0.207071889	0.49739	-0.006837236	0.98218
ZNF75A	-0.450702475	0.00149	-0.173090656	0.2135	-0.304892564	0.03084
ABCF2	0.367204558	0.00149	0.419578424	0.00027	0.151056633	0.19193
SUMF2	-0.368837863	0.00149	-0.562200026	1.26E-06	-0.336701058	0.00369
RELA	0.760450715	0.0015	0.018747746	0.93786	0.296283296	0.21714
PPM1L	-0.430482486	0.0015	-0.304590788	0.02399	-0.120939786	0.3702
C19orf47	0.575481383	0.00151	0.257098792	0.16329	0.315327241	0.0849
ARGLU1	-0.360772126	0.00151	-0.182038416	0.10817	-0.060788845	0.59172
AGFG2	0.538347366	0.00151	0.114539921	0.50223	0.449332572	0.00814
GNB1	0.36242728	0.00152	0.390597259	0.00062	0.113204078	0.32215
CNTNAP3	1.01816229	0.00152	0.119837639	0.70897	0.178321822	0.58041
TMED1	-0.433296816	0.00152	-0.264925576	0.04744	-0.250229014	0.06273
MT-ND4	0.877301606	0.00153	0.789965561	0.00431	1.272210013	4.27E-06
PLAUR	0.719670661	0.00153	0.222322535	0.32817	0.362754893	0.11093
CHAC2	-0.914707434	0.00153	-0.016417214	0.95125	-0.450783587	0.10423
POU2F1	0.623877888	0.00153	0.389400415	0.04782	0.497933623	0.01142
MUC1	-1.880673082	0.00153	-1.027550118	0.08246	-0.606108616	0.30534
SBF1	0.526835348	0.00153	-0.078123151	0.63951	0.325288033	0.05062
ALDH7A1	-0.483131419	0.00153	-0.052030595	0.73058	-0.411589463	0.00671
KIAA0368	0.485297809	0.00154	0.781800941	3.26E-07	0.510142913	0.00087
DDX11	-0.445217112	0.00154	-0.795612965	1.70E-08	-0.354765857	0.01148
FBXO45	-0.304449619	0.00154	0.064074509	0.49309	-0.345518259	0.00033
MMP25	1.646197505	0.00154	0.670495646	0.20541	0.63931172	0.24258
NABP1	0.544477879	0.00155	0.508347323	0.00306	0.196568463	0.25493
C6orf57	-0.506616654	0.00155	-0.145819046	0.34488	-0.481601836	0.00265
TARBP1	-0.597855384	0.00155	-0.252219862	0.17907	-0.143609073	0.4439
AC010970.2	-1.614948473	0.00155	0.693140604	0.17186	-0.007620662	0.98803
LMO2	1.526541554	0.00155	0.959759654	0.0478	0.48186017	0.33218
HIPK2	0.552172318	0.00156	-0.033949765	0.84591	0.500729591	0.00412
P11-641D5	-0.408638278	0.00156	0.090945616	0.48027	-0.131150092	0.30929
NRNPA1P4	0.803974662	0.00156	-0.193876549	0.47723	0.316182678	0.2294
CSE1L	-0.389242758	0.00157	0.279480397	0.0227	-0.20775962	0.09114
ESCO2	-0.784876188	0.00157	-0.174829931	0.47867	-0.216435275	0.38178
HDDC3	-0.734758419	0.00157	-0.129548155	0.56606	-0.332632268	0.14369
VDAC3	-0.251939816	0.00157	0.195729166	0.0124	-0.045934259	0.56124
EPM2A	-2.013010162	0.00158	-1.345377912	0.01366	-0.288077245	0.57387
RNF170	-0.359542167	0.00158	0.012696491	0.90967	-0.177437046	0.11558
SNRNP40	-0.345197407	0.00158	0.070556044	0.51253	-0.233387453	0.03167
SPPL2A	0.357114207	0.00158	0.330113856	0.00342	0.345231116	0.00222
NIPAL3	-0.366941678	0.00158	-0.201316367	0.07749	-0.216991305	0.05917
AP3S1	-0.302497127	0.00158	0.211922272	0.02432	-0.088444977	0.35236
GOLPH3	0.4020979	0.00159	0.476050245	0.00018	0.468863072	0.00023
ZFYVE1	0.543222558	0.00159	-0.061653563	0.72148	0.175101832	0.30985
CHORDC1	-0.364475044	0.00159	0.05462879	0.63307	0.01026445	0.92864
LIF	0.962804322	0.00159	0.482189246	0.11438	0.818574915	0.00729
RBMS1	0.281784709	0.0016	0.304482685	0.00063	0.163802375	0.06669
TXNDC17	-0.541996102	0.0016	0.007353368	0.96548	-0.385672298	0.02432

ZSWIM1	0.356082352	0.0016	0.210055003	0.06311	0.366058408	0.00113
BRIX1	-0.440912611	0.00161	0.340586241	0.01324	-0.331690713	0.01704
ADCK3	-0.603301421	0.00162	-0.007883873	0.96687	-0.085017622	0.65477
CSMD1	2.619392155	0.00162	2.015226688	0.01516	1.389623457	0.09868
ITFG3	-0.382254762	0.00161	-0.47804277	8.27E-05	-0.180624867	0.13471
SCAF4	0.36794973	0.00162	-0.223141198	0.0594	0.078147015	0.50648
ZNF582	0.833630895	0.00162	0.049926301	0.85095	0.113332757	0.68231
P11-745A24	1.208391722	0.00162	0.605785677	0.12535	0.579334835	0.14809
GKAP1	-0.684945101	0.00163	-0.137046277	0.49646	-0.282773346	0.17246
GLRX5	-0.264027643	0.00164	-0.003861596	0.96262	-0.071796242	0.38693
RFTN1	0.688187082	0.00164	0.369559994	0.09171	0.199358555	0.36569
SLITRK3	1.534877618	0.00164	0.502448173	0.31106	1.585603425	0.00123
NUP35	-0.450945318	0.00164	0.147121811	0.29203	-0.292108942	0.0398
ELF1	-0.398932991	0.00165	-0.337806225	0.00737	-0.10384867	0.40987
MESDC2	-0.273078805	0.00165	0.073648626	0.39003	-0.137509245	0.11056
PI4K2B	-0.419361336	0.00165	0.247607166	0.05817	0.027402977	0.83511
PHLDA1	0.66168047	0.00165	0.511225501	0.01505	-0.071696417	0.73356
P11-355I22	-1.009723953	0.00165	-1.228906879	0.00013	-0.531115621	0.09488
ZNF846	-0.513155194	0.00165	-0.785337705	1.70E-06	-0.181624253	0.26
RGS7	-1.104317896	0.00165	0.080828371	0.80921	-0.468596134	0.16822
FOXA2	-0.910046886	0.00166	-0.668372878	0.02043	-0.3134127	0.27632
FAM204A	-0.298235337	0.00166	-0.049005153	0.60062	-0.209710516	0.02642
MRFAP1L1	-0.287482747	0.00166	0.109843134	0.22187	-0.132739662	0.14378
INPP5E	0.434291412	0.00167	-0.069242027	0.6251	0.17687165	0.20776
TPMT	-0.373356352	0.00167	-0.004669714	0.96796	-0.018540496	0.87396
AC243547.1	0.515801344	0.00168	-0.118391886	0.47394	-0.025187022	0.87903
ESM1	3.273093689	0.00168	1.618459642	0.12734	0.963693545	0.38519
IL18BP	0.652708757	0.00168	-0.376715869	0.08123	0.425357213	0.04278
MID1	0.630075917	0.00168	0.280223285	0.16158	-0.125805346	0.53352
NLGN2	0.735343731	0.00168	-0.490213987	0.04041	0.301423876	0.20203
ZNF702P	-0.570157674	0.00168	-0.341397679	0.05557	0.053047092	0.76583
DENND2D	-0.779146159	0.0017	-0.771448217	0.00187	-0.136692498	0.58091
ZNF718	-0.791508765	0.0017	-0.390473132	0.1108	-0.26582278	0.27797
C1QTNF6	0.440252431	0.0017	-0.135339815	0.34248	0.121858915	0.38984
MID1IP1	0.714529404	0.0017	0.614863527	0.00685	0.249296021	0.27792
NPIPA7	0.433472033	0.00171	-0.082916437	0.55119	0.289257017	0.03687
RPP30	-0.307089616	0.00171	0.082031743	0.38874	-0.375233491	0.00013
NOTCH1	0.748981146	0.00172	-0.161216849	0.50195	0.111584187	0.6416
STEAP4	1.024905578	0.00173	1.443184506	9.74E-06	1.340725844	4.16E-05
EXOC4	-0.435942977	0.00173	-0.638325271	4.25E-06	-0.11215548	0.41939
WDR11	0.314787195	0.00174	0.377476783	0.00017	0.587291629	4.71E-09
PHF14	-0.300230799	0.00175	-0.102552114	0.27996	-0.296427153	0.00191
ZWINT	-0.542171243	0.00176	-0.057828135	0.73669	-0.199899401	0.24648
ADAMTS7	0.807881333	0.00177	-0.538732835	0.03937	0.046841039	0.85715
SYTL5	-0.548945098	0.00177	-0.54577798	0.00184	-0.079287477	0.64992
KLHL12	-0.316190298	0.00177	0.013358313	0.89304	0.001535961	0.98772
NFATC1	1.319316756	0.00178	0.226672058	0.5995	1.173422017	0.00581

SMCO4	-0.665262627	0.00178	0.211899807	0.28084	-0.032835672	0.87055
EHF	0.711667689	0.00179	0.88192271	0.00011	1.006638016	9.87E-06
PPT1	-0.433970954	0.00179	0.286111154	0.03782	-0.260302866	0.06056
PCDHA14	-0.561907668	0.00179	-0.76755325	1.93E-05	-0.221160865	0.21423
SNRNP25	-0.48177827	0.00181	-0.045108062	0.76552	-0.109583933	0.47258
CTTN	0.196618847	0.00182	-0.052800549	0.40171	0.322611858	2.89E-07
ACTB	0.26306437	0.00182	0.091573728	0.27778	0.038730729	0.64623
GABBR1	0.743522972	0.00183	-0.319149487	0.18751	0.265865658	0.26998
MSN	0.567607049	0.00184	0.324483225	0.07495	-0.311247191	0.08868
PABPC4	-0.429559099	0.00184	0.044664536	0.74537	-0.468792831	0.00067
AC002398.9	1.42056493	0.00184	1.25622574	0.00586	1.027182512	0.02531
PLA2G7	2.168555183	0.00184	2.343383056	0.00073	1.90122248	0.00646
DTYMK	-0.472918843	0.00185	-0.196788979	0.18436	-0.31508297	0.03546
PRPF6	-0.235129296	0.00185	-0.203863267	0.00641	-0.181930099	0.0155
TCTN3	-0.320659744	0.00185	0.03240992	0.7502	-0.134753189	0.18822
TRAF6	0.474637341	0.00185	0.21970436	0.1485	0.331245369	0.02981
GDF10	1.736242542	0.00186	-0.36223748	0.5396	1.333006346	0.01754
ZDHHC8	0.534706263	0.00186	-0.269248548	0.12611	0.378022954	0.02893
NCOA6	0.491254965	0.00187	0.177561572	0.26218	0.425630973	0.00704
VPS54	-0.22848466	0.00188	-0.014390796	0.84236	-0.04509597	0.53596
CT83	2.963229282	0.0019	2.213707154	0.02359	1.774509768	0.07945
AEN	-0.538540804	0.00191	-0.200717143	0.24326	-0.398213291	0.02124
CLNS1A	-0.295732175	0.00191	0.218262342	0.02052	-0.080735081	0.39449
CYTH1	0.430478524	0.00191	0.121634445	0.38161	0.473707014	0.00064
GNRH2	2.615144079	0.00191	1.983140412	0.02009	-0.214829667	0.81458
TNFRSF10D	0.583410021	0.00192	0.362230963	0.05408	0.271160731	0.14973
SIN3B	0.419980369	0.00192	-0.103968127	0.44528	0.052781558	0.6985
ETV1	0.662567292	0.00193	0.702736502	0.001	0.178045668	0.40621
NDC1	-0.446313803	0.00194	0.118267125	0.40932	-0.121731669	0.39671
GLIPR1	0.96110918	0.00194	1.108888528	0.00035	-0.204883701	0.50995
CLEC2B	0.983176744	0.00195	1.16582713	0.00022	0.294239487	0.35744
MLF2	0.259388555	0.00196	0.10278961	0.21922	0.277847997	0.00089
ARMC1	-0.279283917	0.00196	0.006697392	0.93987	-0.21606989	0.01629
MECOM	-0.661695906	0.00196	-0.059092185	0.78169	-0.116133383	0.5862
MRPL27	-0.383646351	0.00197	-0.013773829	0.90969	-0.149193519	0.22393
LATS2	0.580053944	0.00198	0.337148094	0.07188	0.133418501	0.47907
POP4	-0.384250346	0.00198	0.248543196	0.03962	-0.027073845	0.8253
RTN3	-0.307222873	0.002	0.007563274	0.93881	-0.02724849	0.78259
ARHGEF40	0.620969718	0.00202	0.146623341	0.46683	0.050233969	0.80332
FAM84B	-0.576924795	0.00201	-0.140431592	0.45102	-0.330063413	0.07688
RPL9P7	-1.789124768	0.00202	0.148155249	0.78684	-0.860416417	0.12422
SAP30	-0.411292135	0.00202	0.103944655	0.41106	-0.361251708	0.0062
TRAF3	0.453203951	0.00201	0.109699141	0.45553	0.032852209	0.82366
TRIM47	0.991233966	0.00202	0.586191996	0.06954	0.713726519	0.02792
RMDN1	-0.363220432	0.00202	-0.107820303	0.35091	-0.022471915	0.84644
DICER1	0.408734502	0.00203	0.281571048	0.03341	0.253367094	0.05572
TGFBR3	0.759606609	0.00203	0.257190407	0.29682	-0.058805702	0.81199

PTP4A3	0.990619314	0.00204	0.582191902	0.07039	0.487373093	0.13149
RPS25	-0.32960493	0.00204	0.435846538	3.97E-05	-0.168205617	0.11475
ELF5	-0.90926535	0.00205	-0.361627084	0.20312	-0.645593176	0.02706
PGC	-2.200119145	0.00205	-1.625904991	0.02232	-2.24667814	0.00164
RPL22L1	-0.61755165	0.00205	0.258384603	0.19235	-0.051183712	0.79699
CKS2	-0.449054109	0.00205	0.030304342	0.83297	-0.31971803	0.02753
TOR1B	0.431045935	0.00206	0.279569569	0.04493	0.214090991	0.12698
LSM11	-0.268334513	0.00207	-0.100764055	0.23122	-0.177428999	0.03886
ZSCAN2	0.562703507	0.00207	0.16485987	0.36555	0.306418049	0.09556
IMMP1L	-0.408775489	0.00208	-0.113149221	0.3746	-0.302848446	0.02071
FAM118B	0.392456582	0.00209	0.535698748	2.17E-05	0.591686132	3.05E-06
NDUFB2	-0.415768201	0.0021	-0.117325954	0.37958	-0.353410068	0.0086
CSNK2A1	-0.336062875	0.0021	-0.227564414	0.03678	-0.130416759	0.23212
ORC5	-0.422024197	0.00211	-0.016605722	0.89932	-0.44857929	0.00098
ARNT	0.353258506	0.00212	-0.058355738	0.61224	0.223755129	0.05176
GPN3	-0.553651697	0.00212	0.053296952	0.76082	-0.392253125	0.02741
TSC1	0.282029204	0.00212	0.153049625	0.09597	0.271110843	0.00314
SCRN1	0.248663583	0.00212	0.355107143	1.07E-05	0.193177133	0.017
ATP5SL	0.421391992	0.00213	0.135068426	0.32514	0.246346451	0.07248
SPICE1	-0.470704221	0.00213	-0.361645366	0.01758	-0.214939834	0.15874
PTGS2	1.798036753	0.00213	0.768682115	0.18948	0.999470571	0.08797
SNRNP200	0.278228914	0.00215	0.106695436	0.23859	0.195572216	0.03092
ABCA12	1.890430674	0.00215	1.995418657	0.00117	1.260183971	0.04159
BACH1	0.421544028	0.00215	0.173044634	0.20752	0.11324338	0.41025
NUCB2	-0.693622905	0.00216	-0.105039545	0.63849	-0.340760991	0.13052
RP11-3J10.	0.55925306	0.00217	0.664813004	0.00023	0.465416173	0.01087
DMBT1	1.90687317	0.00217	2.243235604	0.00031	2.103005225	0.00072
THBS3	0.547502348	0.00217	0.0529352	0.76824	0.285048374	0.11228
CLK3	0.456455409	0.00217	0.167128369	0.26248	0.483530018	0.00116
RP11-20I23.	6.807466425	0.00218	5.01016728	0.02486	5.456500165	0.0144
ATP5S	-0.50565596	0.00219	-0.54278085	0.00095	-0.326244418	0.04651
CLUAP1	-0.487996562	0.00219	-0.253868951	0.10756	-0.693006636	1.34E-05
RPS19	-0.428424524	0.00218	0.071012396	0.61121	-0.245667671	0.07881
STIM1	-0.489565893	0.00219	-0.380898585	0.0169	-0.302497943	0.05809
HIST4H4	-0.513945685	0.0022	-0.073635598	0.65815	-0.228697767	0.17091
CTSS	0.726397895	0.00221	0.72379091	0.00227	0.862618254	0.00027
MAP3K2	0.325477269	0.00221	0.152957119	0.14928	0.189266877	0.07513
SHC2	0.569635378	0.00221	-0.164747698	0.37922	0.264394739	0.15598
SLC25A37	0.3895637	0.00221	-0.002536918	0.98414	0.131193424	0.30451
CHMP4BP1	1.554283312	0.00221	0.489078805	0.36192	1.301283642	0.01123
TAF1C	0.544772313	0.00222	-0.265451276	0.14238	0.326647211	0.06713
TMEM106B	-0.302571656	0.00222	0.113298947	0.2492	-0.196374088	0.04661
RORA	0.770314748	0.00223	0.306603772	0.22447	0.479026109	0.0577
PPIE	-0.290646069	0.00224	-0.000247758	0.99786	-0.181874684	0.05228
11-Sep	0.357098861	0.00224	0.557963285	1.77E-06	0.212896659	0.06852
ACSM3	-0.657246522	0.00224	0.152904538	0.47247	-0.126760821	0.55266
EPB41L3	0.891951871	0.00224	0.590401557	0.04345	-0.038524073	0.89587

HDGFRP2	1.125641169	0.00225	0.152062413	0.6831	0.483878898	0.19286
AKAP5	0.60806241	0.00225	0.538233979	0.00671	0.070842187	0.72555
GUCY1A2	-0.740946975	0.00225	-0.518934504	0.03212	-0.494925104	0.04111
PPIH	-0.325849361	0.00225	-0.029734495	0.77336	-0.038320778	0.71304
APOPT1	-0.37591953	0.00226	0.008554602	0.94291	-0.030392654	0.79992
RCN1	0.395176282	0.00226	0.38975121	0.00257	0.344079274	0.00784
C12orf23	-0.332435654	0.00227	-0.08640435	0.42513	-0.280939257	0.00978
PEBP1	-0.300597224	0.00227	0.014937896	0.8787	-0.247973687	0.01163
ACAN	2.397501369	0.00227	-0.194189098	0.8185	-0.924887156	0.29108
PLA2G6	0.661528743	0.00227	-0.089796544	0.68588	0.776868363	0.00033
PPP1R18	0.808428475	0.00227	0.204269236	0.44183	-0.079036916	0.76709
ZNF566	-0.639272342	0.00227	-0.429696745	0.03773	-0.309146578	0.13642
CABIN1	-0.464503081	0.00228	-0.675340763	9.09E-06	-0.303254331	0.0461
RAB31	0.846029644	0.00228	0.272727412	0.32598	0.048124125	0.86305
SLFN5	0.816196746	0.00228	1.208501751	6.14E-06	0.806957732	0.00256
TATDN1	-0.389139784	0.00228	0.221903495	0.07342	-0.035912593	0.7748
EXOSC2	-0.452241423	0.00229	0.031913593	0.82729	-0.161532398	0.27281
DNAJC24	-0.453579696	0.00229	-0.282147018	0.05194	-0.287303427	0.04955
HLA-E	0.426679013	0.00229	0.423949071	0.00241	0.288893427	0.03908
RASA3	0.67880598	0.00229	0.376240885	0.09295	0.052177124	0.82036
MRPL50P2	2.205051183	0.00229	2.736231865	0.00014	2.8128549	9.00E-05
CALM1	-0.325170895	0.0023	0.025698283	0.8092	-0.041060685	0.69994
IRS1	0.445563658	0.0023	-0.242555854	0.09791	-0.049720959	0.7346
TNFRSF1E	0.950600003	0.0023	0.143301232	0.65385	0.366364728	0.24874
POLE2	-0.71900256	0.0023	0.003105507	0.98924	-0.566485939	0.01533
EM256-PLS	0.655116892	0.00231	-0.072851522	0.74058	-0.019791491	0.92819
DDA1	0.467706643	0.00232	0.110003182	0.47718	0.050885092	0.74137
MRPS31	-0.3458995	0.00232	0.250974114	0.01954	-0.04148083	0.7087
AC006978.(	-0.621438737	0.00233	-0.393147937	0.04474	-0.109623585	0.57067
ING2	-0.437448842	0.00233	-0.095562334	0.49468	-0.232792898	0.10021
LTA4H	-0.374460925	0.00233	0.209869854	0.08606	-0.164870917	0.17897
SH3D21	0.699121174	0.00233	0.188804135	0.41478	0.467464025	0.04256
AC004980.1	1.573424163	0.00234	-1.772276561	0.02015	0.779556385	0.14937
MCM5	-0.501169672	0.00234	-0.329178054	0.04478	-0.39007452	0.01756
PRKAR1B	-0.372604886	0.00234	-0.193925466	0.10825	-0.051041659	0.67247
ZNF706	-0.320820672	0.00235	0.103363761	0.31915	-0.166609833	0.11156
TNIP2	0.444905803	0.00235	0.248622514	0.08887	0.386004557	0.00834
TMPO	-0.394586063	0.00235	-0.015762648	0.90307	-0.203439183	0.1165
UHMK1	-0.200305356	0.00236	0.10716429	0.09879	-0.045986632	0.48199
CACNA1C	0.845501891	0.00236	-0.065962766	0.8135	0.106876429	0.70382
FYB	-2.15866535	0.00236	0.004162633	0.99437	-0.204701972	0.73294
FIS1	-0.378797933	0.00237	-0.128543111	0.29501	-0.16632683	0.17824
P1-182O16	1.8604643	0.00237	1.775758913	0.00357	2.127040593	0.00043
MAML2	0.449504315	0.00238	0.111121314	0.45331	0.102715683	0.48902
SLC30A9	-0.357581062	0.00238	0.272651031	0.01975	-0.155649605	0.18528
FBLIM1	0.422286815	0.00238	0.024809024	0.85861	-0.004579697	0.9738
P11-261C1(	1.004919491	0.00239	-0.980101991	0.00783	0.845852598	0.01087

LCAT	0.653350989	0.00239	0.121981817	0.58425	0.302680578	0.16403
MAEA	0.282471971	0.00239	0.175329439	0.05794	0.249881862	0.00716
UBE2V2	-0.26314661	0.0024	0.235929803	0.00555	-0.275333277	0.00145
CYTIP	-1.523212986	0.00242	-0.038611448	0.93636	0.274219725	0.57012
ITGAE	-0.650843794	0.00245	-0.016402704	0.93757	-0.521281473	0.01469
SCARF2	1.166954871	0.00245	-0.13216341	0.74527	0.211366016	0.59861
CDC37	0.324033051	0.00245	0.325247631	0.00232	0.289096241	0.00687
HNRNPR	-0.262895813	0.00246	0.034416605	0.69078	-0.162183834	0.06142
KIF9	-0.661437002	0.00247	-0.158943425	0.45621	-0.374874841	0.08249
CLDN2	-1.237012606	0.00247	-0.33099938	0.4157	-1.217886043	0.00287
CHMP1B2F	-1.030863898	0.00248	-0.164530851	0.61235	-0.46344422	0.16031
RPSAP53	-0.734660186	0.00249	-0.046000632	0.84636	-0.308524938	0.19774
HERPUD2	0.46453689	0.00249	0.46135394	0.00259	0.197751151	0.19898
A16c-17H1	-0.816747395	0.00249	0.010651467	0.96609	-0.613138673	0.02025
LYSMD4	-0.542218902	0.00251	-0.327504059	0.06442	-0.301841397	0.08889
TMEM242	-0.427654624	0.00253	-0.251125548	0.06964	-0.181055274	0.19509
TBC1D10A	0.512143274	0.00253	-0.026959499	0.87485	0.424200855	0.01244
MRPS26	-0.466688164	0.00253	-0.082827036	0.58433	-0.316726311	0.03886
ANPEP	2.745586502	0.00254	1.873744692	0.03944	1.088472624	0.23176
OR2I1P	2.94805712	0.00254	3.489604448	0.00029	2.133483446	0.03333
DTL	-0.664000817	0.00254	-0.338068693	0.12251	-0.466675954	0.03337
DEF6	-0.614462762	0.00256	-0.50577093	0.01183	-0.046509881	0.81341
NCOA2	0.294902134	0.00256	0.06541486	0.5025	0.34299988	0.00044
P11-204M4	-1.619758119	0.00256	-0.268383632	0.56901	-0.367214984	0.44218
TNFRSF11I	1.088791658	0.00256	1.470216878	4.51E-05	0.223934151	0.53668
MAVS	0.239160281	0.00257	-0.198377459	0.01272	0.154498734	0.05153
MRPL11	-0.383334038	0.00257	0.061406368	0.62132	-0.182118231	0.148
AMOTL2	0.659229975	0.00257	0.221653029	0.31168	0.173390668	0.42939
PATZ1	-0.335922032	0.00258	-0.43073339	0.0001	-0.232898861	0.03601
FBXO21	-0.338505145	0.00258	-0.159824019	0.15089	-0.29555881	0.00834
KLC3	1.042095225	0.00258	0.437376521	0.22063	0.766430414	0.02898
JPH3	1.091416036	0.00259	0.476600908	0.20103	0.697553622	0.06099
CD40	1.733838051	0.00259	1.889336487	0.00093	0.650774314	0.27455
KIF21A	-0.471930193	0.00259	-0.080549635	0.60565	0.117943211	0.44966
MCL1	0.45462361	0.00259	0.149968254	0.32071	0.203342901	0.17829
MAML1	0.600062582	0.0026	-0.074688402	0.70853	0.236349073	0.23643
NAMPTL	1.078093051	0.0026	1.057546461	0.00313	0.836138231	0.01956
STRN4	0.298264228	0.0026	-0.183773991	0.06546	0.194178215	0.05045
PUS7	-0.543388644	0.00262	-0.074400083	0.67773	-0.311171859	0.0838
TRIT1	-0.439198779	0.00264	-0.262989711	0.06743	-0.198425305	0.17186
RFT1	-0.403138409	0.00264	0.127613173	0.33397	-0.273269322	0.04119
TMEM189	0.425416492	0.00264	0.143086593	0.31232	0.323592633	0.02219
ARHGEF37	0.984156488	0.00266	0.749117127	0.02175	0.683309621	0.03613
CEACAM1	1.288680075	0.00266	0.7007811	0.10225	1.512474138	0.00042
RNF138	-0.388477868	0.00266	0.078533632	0.53785	-0.091056116	0.47733
LRP2	1.718232841	0.00266	1.047308465	0.06724	1.65014205	0.00394
ARHGAP1(	-0.362666169	0.00267	-0.128316767	0.2801	-0.170856777	0.15282

CPD	-0.397842716	0.00268	-0.170174324	0.19886	-0.168693699	0.20278
STARD7	-0.301373138	0.00268	-0.032913783	0.74187	-0.031791453	0.75086
ABHD17B	-0.364893582	0.00268	-0.269311273	0.02445	-0.221610387	0.06562
CCDC120	0.667146634	0.00268	-0.600072032	0.00903	0.365579611	0.10254
LARS2	-0.359204402	0.00268	-0.080980451	0.49286	-0.197753446	0.09665
HIST1H4G	-1.101657861	0.0027	-0.386799637	0.28182	-0.192812715	0.59129
FYCO1	-0.345935817	0.0027	-0.116243072	0.30894	-0.192754525	0.09247
NSRP1	0.47405798	0.00271	0.013645648	0.93091	0.112070109	0.48047
OVCA2	0.800940316	0.00271	0.738331869	0.0056	0.754023143	0.00476
TBC1D2B	0.477505018	0.00271	0.219789326	0.16742	0.246097753	0.1223
WDFY1	-0.210058332	0.00272	-0.082424169	0.23509	-0.085973071	0.21755
MOB1A	-0.205833213	0.00272	0.05886649	0.38842	0.019577607	0.77473
SLC6A20	1.653620773	0.00272	1.868320738	0.00069	0.578352527	0.30066
JAK3	0.889954379	0.00273	0.151061185	0.61292	0.071496933	0.81119
DNAL4	-0.454373664	0.00275	-0.358597545	0.01529	-0.21303835	0.15063
PRPF40A	-0.272741018	0.00275	0.161314604	0.0752	-0.180309085	0.0475
MYCBP	-0.469176019	0.00277	0.188928921	0.22246	-0.017190228	0.912
TBC1D17	0.446499281	0.00278	-0.260724714	0.08838	0.133930203	0.37385
AP3M2	-0.454922664	0.00279	-0.325057349	0.03089	-0.291143711	0.05536
IL2RG	0.869702535	0.0028	0.757093475	0.00921	0.96959777	0.00085
MMADHC	-0.273734268	0.0028	0.164914772	0.0678	-0.101638938	0.26456
ABHD10	-0.361869356	0.00281	0.070078934	0.55138	-0.167304119	0.16268
C19orf43	0.310424335	0.00281	0.266256646	0.01018	0.304079797	0.00341
GPR160	-0.609913524	0.00281	0.07133802	0.72503	-0.066785625	0.74234
PMPCB	-0.293539494	0.00281	0.216748727	0.02478	-0.096788278	0.32156
MAP1LC3E	0.525051657	0.00281	0.514322212	0.00333	0.191774299	0.27665
TMEM138	0.448270692	0.00282	0.075904306	0.61545	0.326854272	0.02985
ARNTL	0.757254726	0.00283	0.586359747	0.01993	0.674714683	0.00743
NEDD8	-0.231648141	0.00283	0.062656312	0.41125	-0.126324044	0.10184
RPL13A	-0.367430263	0.00283	0.14947131	0.2243	-0.107587606	0.38185
SYNJ2	0.412987261	0.00284	0.00422771	0.97564	0.043654054	0.75323
RPSAP58	-0.843795067	0.00284	0.605206738	0.03036	-0.144585789	0.60674
DDAH1	-0.327691693	0.00286	-0.115295832	0.29274	0.141714481	0.1956
GLYCTK	-0.614009423	0.00286	-0.550309034	0.00732	-0.388458376	0.05742
RPLP0P6	-1.739896546	0.00286	-0.242785378	0.63976	-0.986190008	0.07005
TRIM62	0.705890266	0.00286	0.00180611	0.994	0.173876355	0.46819
CASP6	-0.396619842	0.00287	0.202408262	0.11605	-0.08525655	0.51337
RAD54L	-0.753719419	0.00287	-1.162482427	4.69E-06	-0.199304352	0.42735
PHF23	0.393421412	0.00288	0.031746976	0.80997	0.08519557	0.51981
IRS4	1.823326007	0.00288	1.580883778	0.00981	1.927562071	0.0016
ZNF165	-0.60190523	0.00288	-0.239602714	0.21919	-0.320520432	0.10463
CDC37L1	-0.511660171	0.00289	0.098831403	0.55715	-0.000621915	0.99708
MPP6	-0.645112739	0.00289	0.236780767	0.26992	-0.001214836	0.9955
COL15A1	1.088331598	0.0029	-0.038754608	0.92415	0.577901015	0.1343
E2F8	-0.601569317	0.0029	-0.086676697	0.66395	-0.190981304	0.34042
FEN1	-0.535600135	0.0029	-0.110080594	0.53741	-0.230101183	0.19871
GPR50	1.928282452	0.00291	1.666249645	0.01011	1.120227378	0.08537

EIF4ENIF1	0.429790669	0.00292	0.447549682	0.00193	0.346522155	0.01647
MPC2	-0.413443493	0.00292	-0.090952976	0.50882	-0.069200328	0.61591
ZNF107	-0.509198343	0.00292	-0.170798475	0.30872	-0.409385911	0.01626
ISG20	0.793167901	0.00293	-0.138217701	0.60799	0.67430352	0.01149
TRAPPC6A	-0.536473775	0.00293	-0.382608982	0.03019	-0.377609404	0.03381
RBBP8NL	-0.848547857	0.00293	-0.903169853	0.00145	-0.315006506	0.25825
DBN1	0.663950589	0.00294	0.192857105	0.38789	-0.081833817	0.71451
PLSCR1	0.377278884	0.00294	0.531653998	2.63E-05	0.585342325	3.79E-06
MTIF2	-0.431637472	0.00295	0.287339992	0.04474	-0.005301	0.97063
TRIM16	0.534392637	0.00296	0.581014488	0.00121	0.612437646	0.00065
SEC61A1	0.235420549	0.00298	0.109011425	0.16863	0.187918171	0.01772
FAM167A	0.897924229	0.003	0.915160447	0.00237	0.666281091	0.03006
PARP8	0.377751584	0.003	0.236873337	0.06182	0.376150466	0.00313
TRAPPC1	0.31228011	0.003	0.343254781	0.00101	0.152523547	0.14963
VWA8	-0.545890625	0.003	-0.186950323	0.30549	-0.376315737	0.03965
NDUFC2	-0.308029378	0.00301	0.104674688	0.30772	-0.360611277	0.00051
TPT1	-0.317974148	0.00301	0.205879648	0.05465	0.001860145	0.98615
HTATSF1	-0.267856806	0.00302	0.013760267	0.87727	-0.129434099	0.14922
POLH	-0.45094711	0.00303	-0.135017297	0.37083	-0.074548865	0.62231
P11-84C13	-0.479362837	0.00303	0.02092348	0.89492	-0.079632366	0.61736
FGFR1	0.731134885	0.00303	0.25891013	0.29438	-0.040810246	0.86892
GNPAT	-0.298126908	0.00304	0.163476516	0.09906	-0.089663956	0.37024
IMPA2	-0.489223539	0.00304	-0.502536887	0.00222	-0.001424471	0.99308
RNASET2	-0.447940345	0.00304	-0.345641241	0.02162	-0.098380183	0.51227
GNRHR2P1	-1.213328575	0.00305	-0.23527448	0.55383	-0.421357519	0.29207
MRPL21	-0.370372578	0.00307	0.321708144	0.00711	-0.099495433	0.41749
SYNGAP1	1.034960496	0.00307	-0.08054565	0.81888	0.458919837	0.19154
HHLA2	1.275899526	0.00308	1.085544598	0.0118	1.214209045	0.00485
TMPRSS2	0.747072469	0.00309	0.668597203	0.00807	0.8793875	0.00049
UBASH3A	2.192494521	0.0031	2.512560092	0.00057	2.102766231	0.00465
SUZ12	-0.350659558	0.0031	0.144878552	0.21705	-0.132139777	0.26293
EPB41L1	-0.668783369	0.0031	-0.658519231	0.00368	-0.217337859	0.33542
ADCK2	-0.574853786	0.00311	-0.484592049	0.01234	-0.320832036	0.09647
LRRC3	0.493862546	0.00311	-0.072109627	0.67572	0.437372799	0.00889
RAPGEF2	0.331347652	0.00312	0.117917982	0.29274	0.192795283	0.08561
ATF7IP2	-0.481080503	0.00312	-0.398991231	0.01396	0.068649187	0.67156
HARS2	-0.281007938	0.00312	-0.038454584	0.67891	-0.17654446	0.06085
MAP2K2	0.439309201	0.00312	0.287794263	0.05283	0.191943124	0.19686
TLE3	0.73907846	0.00312	0.319510089	0.20331	0.027444424	0.91294
KCNA1	1.923253431	0.00312	2.011443144	0.0023	0.620328871	0.35397
DCTN2	0.261342936	0.00313	0.319760988	0.00027	0.163645823	0.06416
PIGV	-0.373736051	0.00314	0.023175871	0.8494	0.06040494	0.6224
RPS7P11	-1.184859777	0.00314	0.263547449	0.47756	-0.862462794	0.02754
PHKA2	-0.484034018	0.00316	-0.582561002	0.00038	0.16055136	0.32614
TOMM5	-0.338918371	0.00317	0.273250154	0.01626	-0.101184294	0.37635
C2CD4C	1.24931483	0.00319	0.612654688	0.1537	0.113674464	0.7967
NLN	-0.349231692	0.00319	-0.035409857	0.76202	-0.170202987	0.14743

FBXO5	-0.41749373	0.0032	0.036541278	0.79337	-0.364580893	0.00983
SLC10A2	3.060995838	0.00321	3.786949472	0.00026	-0.595440401	0.58448
KLHL29	0.453639818	0.00321	-0.257300373	0.09935	0.089548902	0.56427
FAM214B	0.657068747	0.00321	-0.040808752	0.85562	0.265013099	0.23651
PLEKHM1	0.73472875	0.00322	-0.632108998	0.01198	0.346936556	0.16532
CAMK2A	1.535456376	0.00322	0.101683328	0.84828	0.758523043	0.15465
SEMA3C	0.62842943	0.00322	0.907535425	2.07E-05	0.69150063	0.00119
RPL30	-0.317235041	0.00323	0.314617673	0.00344	-0.181879205	0.09119
GRSF1	-0.241170701	0.00323	0.006403162	0.93726	-0.071413265	0.38173
PRIM1	-0.771366804	0.00324	0.010526815	0.96734	-0.145587694	0.57296
SNRPD2	-0.36363517	0.00325	0.102617295	0.403	-0.271539746	0.02773
GTF3A	-0.386023395	0.00326	0.21590943	0.09718	-0.078706436	0.54653
MMD	0.432606519	0.00326	0.666214251	5.21E-06	-0.097413645	0.51067
TES	-0.25672909	0.00326	-0.164648771	0.05809	-0.223834832	0.01019
UNC93A	-1.152797949	0.00326	-1.128098405	0.00365	-0.342064236	0.37355
PIFO	-1.242055594	0.00328	0.431891006	0.27546	-0.408654891	0.3221
MYO1H	1.868766252	0.00328	1.889795643	0.0028	1.915917993	0.00267
MICALL1	0.379947332	0.00329	-0.083283966	0.52297	0.156634433	0.22735
APMAP	-0.33237394	0.0033	0.031004148	0.78236	-0.216945362	0.05449
VPS37B	0.401366051	0.0033	0.073593437	0.5908	0.331157155	0.01531
PODN	0.982921561	0.00332	-0.145565034	0.67476	0.016776303	0.96178
DENND1A	0.456515872	0.00333	0.123172736	0.43287	0.392967129	0.01168
FAIM3	1.248281715	0.00335	0.881102275	0.03857	1.74633865	3.55E-05
FUCA2	-0.466278484	0.00335	-0.155021213	0.32696	-0.186478598	0.23904
NOL11	-0.316873516	0.00335	0.090818962	0.394	-0.282627871	0.0086
PSPH	-0.414862585	0.00335	-0.162315002	0.24382	-0.147134747	0.29306
TUBA1A	0.790858321	0.00336	0.609848207	0.02371	-0.176974312	0.51194
PDE4DIP	-0.45142046	0.00337	-0.065592938	0.66759	-0.149655324	0.32872
PDLIM1	0.361128029	0.00337	0.57143767	3.15E-06	0.343538676	0.00526
CLN5	-0.660826523	0.00337	-0.427546188	0.05422	-0.16265083	0.46516
KXD1	0.404209474	0.00338	0.504277777	0.00025	0.200332392	0.14673
RUNX1T1	0.99494258	0.00338	0.405982891	0.23176	0.480602914	0.15769
ACBD6	-0.347502751	0.0034	0.072544876	0.53359	-0.309649892	0.00881
ANKRD49	-0.393422947	0.00339	0.06838886	0.60339	-0.146502251	0.27109
DDX50	-0.270801868	0.00339	0.216377734	0.017	-0.269657755	0.00349
ODAM	-1.794419621	0.0034	-0.455285288	0.45092	-1.949603318	0.00146
WDR3	-0.46027847	0.0034	0.20185852	0.19447	-0.182727179	0.24242
TCF7L2	0.720470591	0.0034	0.153112965	0.53385	0.774299758	0.00164
RBBP9	-0.471761796	0.00341	0.23187361	0.14639	-0.206994438	0.19707
GLB1	-0.458343381	0.00341	-0.270454793	0.08349	-0.133818714	0.39198
NQO2	-0.461463006	0.00341	-0.189809179	0.22638	-0.494472724	0.00165
CALD1	0.556500174	0.00342	0.420071434	0.0271	-0.016296588	0.93173
ZNF33A	-0.326739916	0.00342	-0.191370561	0.08438	-0.171763157	0.1228
MRPL23	-0.633550246	0.00343	0.056308495	0.78909	-0.209410641	0.32492
DLK1	1.684208857	0.00344	0.116372437	0.83998	-0.143798536	0.80308
PTGR2	-0.566037716	0.00345	-0.125234063	0.51314	-0.184687324	0.33548
SQRDL	0.463777942	0.00345	0.847591726	7.59E-08	0.463100016	0.00347

NCR3LG1	0.515959518	0.00346	0.550513823	0.00178	0.388990104	0.02773
RPS24	-0.392595768	0.00349	0.387972644	0.00388	-0.128914131	0.33744
NDN	0.376928611	0.0035	0.514073451	6.14E-05	0.124801746	0.33579
RCAN3	-0.678232394	0.0035	-0.374882972	0.10589	-0.316764641	0.17183
P11-38O23	-1.397262065	0.00352	0.132655398	0.74956	-0.273930969	0.5232
SLC9A1	0.791288802	0.00352	-0.094498686	0.73201	0.870934778	0.00134
TENM3	0.948236074	0.00353	0.250287631	0.4418	-0.245736826	0.45103
COPS4	-0.369310193	0.00354	0.210815519	0.0914	-0.334822676	0.00813
GABPA	-0.233054783	0.00354	0.131676834	0.09281	-0.064918607	0.41253
STAG3L1	1.006259449	0.00354	-0.724845971	0.06451	-0.195029094	0.60371
P11-742N3	-1.021126788	0.00354	0.536441	0.11532	-1.173078181	0.00084
ENTPD3	-0.813048709	0.00356	-1.199433195	1.75E-05	0.056127712	0.835
NAA60	0.413928569	0.00356	0.140527229	0.32263	0.216617475	0.12863
VILL	-0.520073343	0.00358	-0.846580174	2.15E-06	-0.065946767	0.71089
ZNFX1	0.326019961	0.00359	0.094133845	0.4009	0.34609958	0.00198
ZNF77	-0.495462256	0.00361	-0.409030026	0.01345	-0.112218966	0.48923
OXLD1	-0.575426569	0.00362	-0.227419103	0.23081	-0.057853954	0.76285
CCHCR1	-0.579251522	0.00363	-0.527152348	0.00789	-0.228233196	0.248
IGF2	0.530868756	0.00363	-0.082250571	0.65229	-0.105200763	0.56443
RAB11FIP5	0.482783588	0.00363	-0.431244539	0.01028	0.143222171	0.39025
TNNI3	-0.982533578	0.00363	0.038290864	0.8978	-0.90276038	0.00621
ADD3	-0.298397723	0.00364	-0.15874659	0.12105	-0.130525132	0.20302
GBAS	-0.318923348	0.00364	0.080994877	0.4554	-0.218731269	0.04544
GBP2	0.560623491	0.00365	0.833853407	1.50E-05	0.536206828	0.00543
LSM4	-0.419958578	0.00366	-0.103039645	0.47192	-0.13896397	0.33315
CYP3A5	0.631815597	0.00367	0.32105584	0.14004	0.703833259	0.00121
KIF7	0.758435661	0.00367	-0.085377217	0.74658	-0.028718505	0.91378
TSEN2	-0.709728838	0.00367	-0.610608966	0.01204	0.06658368	0.7836
GNA12	0.384802705	0.00368	0.06636079	0.61672	-0.049150504	0.71213
TMEM130	0.952259162	0.00368	0.712583492	0.0318	0.956345909	0.0036
P11-432B6	-5.143241001	0.00369	1.853651463	0.20264	-3.320972314	0.05008
SPTLC1	-0.235703649	0.0037	0.038516706	0.629	-0.090005062	0.26426
MADD	0.375509039	0.0037	-0.190152352	0.14341	0.394831019	0.00227
HSD17B1	0.442595054	0.00371	0.04012559	0.79358	0.202577098	0.1881
RRNAD1	-0.505341222	0.00372	-0.678687732	8.59E-05	-0.161075241	0.34562
SMYD3	-0.587705325	0.00372	-0.137703219	0.48761	-0.596506919	0.00325
TTC5	-0.478362782	0.00372	-0.085964081	0.59688	-0.239267084	0.14412
USP16	-0.27668191	0.00372	0.084142731	0.37142	-0.086574489	0.36104
ZZEF1	0.475759096	0.00373	0.026140627	0.87372	0.31853584	0.05226
CYB5R1	-0.431364313	0.00374	-0.176066823	0.23072	-0.064929365	0.65834
LYPD6B	0.649613188	0.00375	0.86052643	0.00011	1.009417074	5.89E-06
DST	0.445629829	0.00376	0.398254254	0.00959	0.113433857	0.46089
ECHDC2	-0.453371451	0.00377	-0.620389016	8.24E-05	0.018987153	0.90237
LRRC8C	0.498730272	0.00377	0.338641792	0.04863	-0.470220261	0.0084
LLGL1	0.493983988	0.00378	0.015906958	0.92596	0.128206603	0.45388
MT-CO2	0.705986186	0.00379	0.389872892	0.10984	0.898713317	0.00023
COL5A1	0.739468012	0.00379	-0.313530246	0.2199	-0.249198423	0.32966

TOR4A	0.473830115	0.00379	0.088832576	0.58885	0.411709108	0.01191
RYK	-0.270164385	0.00381	0.016157228	0.86163	-0.145162651	0.11882
TMEM158	1.303649089	0.00381	0.488276614	0.28315	0.177297841	0.69963
HNRNPU	0.216130997	0.00381	0.184772103	0.01329	0.210491959	0.00483
NOS1	0.920082069	0.00382	0.49807364	0.12457	0.44395504	0.17024
PCDH11X	1.665826444	0.00382	0.234015771	0.68799	0.061111565	0.91696
WARS2	-0.359344238	0.00381	-0.017781363	0.88338	-0.0860567	0.48241
ABHD4	0.555925856	0.00382	0.066976399	0.72902	0.552734614	0.00392
ARSE	-0.578037994	0.00382	0.086132114	0.66319	-0.500944085	0.0119
STK36	0.49402262	0.00382	-0.103087156	0.54829	0.273031992	0.1103
BAG1	-0.34484645	0.00384	-0.108483443	0.35697	-0.220539209	0.06196
GAL	1.700582273	0.00384	0.912550262	0.12612	0.328029763	0.5922
INRNPA2B	0.312390284	0.00384	0.308362246	0.00431	0.351807348	0.00113
SMTN	0.634367452	0.00384	-0.27123901	0.21788	-0.081445954	0.71153
COMMD9	-0.318344	0.00385	0.16933909	0.11309	-0.307289061	0.00509
KIAA1324L	-0.519906492	0.00385	-0.148566458	0.40711	-0.20068881	0.26334
MRPL4	0.402143066	0.00385	0.302053663	0.02941	0.329396544	0.01792
FAM193B	0.443993051	0.00386	-0.300924969	0.05302	0.016728986	0.91361
SMC3	-0.307122506	0.00386	0.045860402	0.66426	-0.187983058	0.07635
IL7	1.276856335	0.00387	0.645007105	0.14955	0.490973804	0.28469
MT-ND1	0.847708772	0.00387	0.92015743	0.00171	1.252879915	1.95E-05
PRR14L	0.336077834	0.00387	0.258361719	0.02615	0.378496035	0.00113
PCYOX1	-0.290321407	0.00389	-0.030912004	0.75755	-0.108634005	0.27897
CD55	0.877382927	0.00389	1.102747777	0.00028	0.303924398	0.31815
WRNIP1	-0.271667887	0.0039	0.044376168	0.63163	-0.043759062	0.63891
PKD1L1	-1.34883529	0.00391	-0.972919039	0.03662	0.04173698	0.9276
LSM7	-0.361443973	0.00393	-0.127182377	0.30242	-0.175374786	0.15686
OTUD1	0.599888559	0.00393	0.180472187	0.38778	0.554141139	0.00772
LTBR	0.437019395	0.00394	-0.256257621	0.09288	0.163889591	0.28231
MAP2K7	0.777405159	0.00395	-0.287632417	0.28846	1.23218664	5.04E-06
MAPK11	1.128934752	0.00395	0.308372495	0.43611	0.35697599	0.37644
ZNRF1	0.440649681	0.00396	-0.023294071	0.87986	0.226015845	0.14108
HMGB3P9	-1.787029927	0.00397	-1.128177002	0.04248	-0.571956861	0.28374
ODC1	-0.463731819	0.00398	0.302064796	0.05929	-0.436896435	0.00665
RPS6	-0.377762181	0.00399	0.092881492	0.47885	-0.12086544	0.35685
BTF3L4	-0.259284374	0.004	-0.055929968	0.53093	-0.189912113	0.03456
HOMEZ	0.453344588	0.004	0.009616193	0.95134	0.422427191	0.00722
HSD17B11	-0.614571447	0.004	-0.0390642	0.85397	-0.462776843	0.03003
KRT23	1.965706885	0.00401	1.509770062	0.02736	1.543495451	0.02412
SHMT1	-0.54874468	0.00402	-0.262610867	0.16308	-0.409938777	0.03058
ABCC5	-0.447152534	0.00404	-0.704029463	5.86E-06	-0.46514807	0.00271
RAB34	0.364735942	0.00403	0.18821466	0.13758	0.1802765	0.15537
TSN	-0.250098043	0.00404	0.082553069	0.33578	-0.060143936	0.48621
ATL3	-0.222768909	0.00405	-0.000355096	0.99631	-0.016215881	0.83319
HMGN1	-0.220543519	0.00405	0.049412777	0.51776	-0.015047927	0.84431
BLVRB	-0.450048512	0.00407	-0.108255385	0.486	0.100194298	0.51854
ANO1	0.861762021	0.00408	0.463450561	0.12257	0.321220448	0.28471

GLI3	0.874450593	0.00408	0.395359946	0.19486	0.310633739	0.30988
PPIL3	-0.341359794	0.00409	0.109645464	0.34614	-0.155863714	0.18726
NDUFAF1	-0.436446183	0.00409	0.204425092	0.15288	-0.364185549	0.01471
TIMELESS	-0.482573267	0.0041	-0.308810895	0.06487	-0.32340616	0.05364
TANC2	0.640866106	0.0041	0.033785043	0.87989	0.372963444	0.09522
PDCD2L	-0.655829613	0.00411	-0.022419996	0.91669	-0.244472546	0.26639
GSS	-0.289995932	0.00413	-0.013590158	0.89122	-0.029418598	0.76839
HOXA3	1.082898345	0.00414	-0.342852314	0.37746	0.25999527	0.49941
SF3A1	0.398295536	0.00416	0.218821846	0.11519	0.293521307	0.03462
ORC2	-0.354618966	0.00416	-0.14940642	0.22258	0.055510938	0.65156
PTS	0.407401856	0.00416	0.292335934	0.03923	0.482112023	0.00067
AC141586.5	0.559425467	0.00418	-0.226253628	0.26037	0.162479583	0.41209
ZC3H11A	0.193572957	0.00418	0.10007482	0.13831	0.137679246	0.04163
MOB4	-0.334879809	0.00419	0.070640887	0.54155	-0.069379708	0.55112
HDHD2	-0.329623261	0.0042	-0.167128681	0.13883	-0.209001983	0.06549
GMPS	-0.283970188	0.0042	0.167307957	0.08994	-0.146844501	0.13807
TMEM5	-0.360851353	0.00422	-0.057147859	0.64134	-0.134360634	0.27928
MBTD1	-0.297524129	0.00422	-0.117505286	0.25059	-0.214219315	0.03863
C5	-0.876860774	0.00423	0.434797398	0.15046	-0.54035052	0.07744
FAM131A	0.506437175	0.00424	-0.035991282	0.84103	0.103271357	0.5635
NFYB	-0.354656337	0.00424	0.231688526	0.05861	-0.142296391	0.24931
SEC24D	0.457317343	0.00424	-0.054254522	0.73465	0.011381037	0.94334
C11orf31	-0.390878122	0.00425	0.054575649	0.68486	-0.206556869	0.12798
NPEPL1	-0.544652996	0.00426	-0.442689941	0.01981	-0.062410907	0.73973
UGGT1	-0.28462411	0.00426	0.112793326	0.25597	0.149929637	0.13138
ZNF644	-0.21048608	0.00426	0.053745197	0.46133	-0.223438751	0.0024
CHD8	0.293272036	0.00427	0.049481374	0.62909	0.03860274	0.70688
MAP3K12	0.698488102	0.00428	0.108261297	0.65918	-0.168911504	0.49203
NFU1	-0.338205093	0.00428	0.255404272	0.02674	-0.01206681	0.91756
TMEM144	-0.50752785	0.00429	-0.088535415	0.61565	-0.125745634	0.4765
LSM12	0.308085302	0.00429	0.247516724	0.02128	0.251063391	0.01983
FXYD6	0.677986434	0.0043	-0.003810261	0.98726	0.136508489	0.5674
GTF2F2	-0.313128619	0.0043	0.324720623	0.00208	-0.109311334	0.31216
LRRC55	1.887926132	0.0043	1.67676797	0.01124	0.736852496	0.27494
RPL4	-0.332708374	0.0043	0.21641401	0.06318	-0.154113695	0.18594
NEIL3	-0.421758376	0.00433	-0.007946104	0.95555	-0.310616323	0.03394
TMEM99	-0.60267076	0.00433	-0.13719006	0.49868	-0.211413765	0.30142
CABP4	1.511382095	0.00434	0.531026284	0.32669	1.311710068	0.01286
MED29	1.004865632	0.00434	0.361296485	0.31269	0.194138211	0.59044
OR7E38P	0.61234886	0.00434	0.279740866	0.19687	0.353792426	0.1041
TENC1	0.65952312	0.00434	-0.1906526	0.41147	0.18432135	0.42629
AC012501.3	-0.823417976	0.00435	-0.379219339	0.17093	-0.142293679	0.60549
GNG10	-0.428836239	0.00436	0.255850394	0.08537	-0.373382291	0.0129
RAB11B	0.548798554	0.00437	-0.059750638	0.76065	0.568239255	0.00321
COIL	-0.253503946	0.00438	0.083149445	0.3363	-0.241456665	0.00635
RPL14P1	-1.405369623	0.00438	-0.32655197	0.46467	-0.948068474	0.04381
CLK1	-0.46655642	0.00439	-0.51315598	0.00172	-0.44876116	0.00612

EEF1A1P5	0.406523299	0.00438	0.230856909	0.10567	0.787954606	3.33E-08
TMEM251	-0.606671828	0.00439	-0.178116204	0.36604	-0.346197909	0.08932
JAZF1	0.561226028	0.0044	0.282053206	0.1514	0.321538394	0.1041
RNF13	-0.339658672	0.00441	0.16000188	0.1739	-0.039716565	0.73729
SLC43A1	-0.644564612	0.00441	-0.66760343	0.00311	-0.362218466	0.10766
TLE4	0.400756391	0.00441	-0.088659929	0.52973	0.424142138	0.00256
TM9SF3	-0.250936318	0.00441	-0.006985636	0.93673	0.188602907	0.03213
IRF2	0.581059531	0.00442	0.073350152	0.71943	0.416072245	0.04118
TAPT1	0.409761383	0.00442	0.185550769	0.19609	0.09686057	0.50147
ZNF697	0.500352229	0.00443	0.171836496	0.32975	-0.086225473	0.62809
NEK4	-0.287228053	0.00444	-0.118005546	0.2382	-0.335654094	0.00086
SAP130	0.32718968	0.00446	-0.060389981	0.60228	0.201473263	0.0808
EMG1	-0.383500508	0.0045	0.175952555	0.18737	-0.086024273	0.52211
UBR7	-0.427147031	0.00451	-0.07475954	0.61239	-0.262420537	0.07944
NME1-NME	-0.341635702	0.00451	0.193241263	0.10658	-0.108915887	0.36428
ZER1	0.318921256	0.00451	-0.043962012	0.69773	0.01935875	0.864
FAM83H	17.59449634	0.00453	-0.200280619	0.97443	0.012856543	0.99836
POP7	-0.348540436	0.00454	0.081471588	0.4913	-0.268299157	0.02754
TCF21	0.752919781	0.00455	0.014418547	0.95685	0.079637416	0.76523
ZNF252P	-0.343867663	0.00456	-0.143158162	0.23271	0.01070866	0.92901
KANK1	-0.403053818	0.00456	-0.304408379	0.03157	-0.089581085	0.52675
LOX	0.934089914	0.00457	0.649307591	0.04867	0.049451227	0.88106
UBQLN2	0.31200718	0.00458	-0.007389669	0.94673	0.12051355	0.27609
KIAA0556	0.546132533	0.00459	0.182698597	0.34418	0.375596765	0.05094
SNAI1	0.626202232	0.00459	-0.246376415	0.27926	-0.156870067	0.49201
BBS2	-0.434893548	0.00459	-0.334784845	0.02823	-0.297832448	0.05085
GPAA1	0.488155394	0.0046	0.018296577	0.91549	0.258968519	0.133
PXN	0.572147169	0.00461	0.054011433	0.78985	0.2025763	0.31734
TAF1D	-0.313519933	0.00461	0.270541	0.014	-0.206225248	0.06186
ZNF287	-0.759271112	0.00461	-0.122060478	0.64557	-0.277312566	0.30208
ARID5A	0.678433981	0.00462	-0.091092371	0.71062	0.052357039	0.83111
POLR1E	-0.377169776	0.00462	-0.066058644	0.6125	-0.400208509	0.00264
CCDC115	-0.405315981	0.00463	-0.28005757	0.04804	-0.185028431	0.19149
SLC5A5	1.010820847	0.00465	0.241261449	0.51529	0.783051499	0.02998
UBE2K	0.329825505	0.00465	0.569102659	9.17E-07	0.313152873	0.00708
PFN1	0.424571606	0.00467	0.534885974	0.00036	0.361606408	0.01597
PHC1	0.679880761	0.00466	0.39000552	0.10469	0.19797178	0.41114
SNF8	-0.276977079	0.00466	0.007960955	0.93403	-0.239919402	0.01383
SH3YL1	-0.365863256	0.00469	0.012766188	0.92109	-0.074720923	0.56241
DYRK4	-0.37557897	0.0047	0.201947442	0.11361	-0.318845858	0.01659
ALOX15B	1.505008991	0.00472	1.711958511	0.00117	1.312910444	0.01442
FXR2	0.330137703	0.00474	0.034819735	0.76567	0.101585305	0.38608
MCMBP	-0.234495112	0.00476	0.054162989	0.50985	-0.13077541	0.11379
MRPS16	-0.352296689	0.00475	-0.009047316	0.94182	-0.205248131	0.09907
NR1H2	0.433328572	0.00475	0.079161642	0.60582	0.31826429	0.03776
HSPA4	-0.275329722	0.00476	0.438026517	6.07E-06	-0.101244832	0.29856
UBE2Z	0.231247818	0.00476	0.024084796	0.7682	0.229883989	0.00492

TD-3148I1(	1.735252728	0.00477	1.683566891	0.00594	1.720156425	0.0051
AIFM1	-0.383520754	0.00478	0.113007615	0.39908	0.017000995	0.8996
SNX5	-0.296429592	0.00479	0.114631452	0.2724	-0.215846762	0.03947
CA5A	0.717149799	0.00479	-0.265609963	0.30759	0.169470043	0.50986
REL	0.480962651	0.0048	0.226695763	0.18385	0.563694412	0.00094
LGALS9C	1.614037936	0.0048	0.334618143	0.57017	1.317783384	0.02077
GAS7	0.610314923	0.00483	-0.096920801	0.65576	0.232694457	0.28427
PARK7	-0.308971345	0.00482	0.214845751	0.04743	-0.121324028	0.26629
PVRL2	0.333718527	0.00483	-0.131474077	0.26822	0.054520108	0.6458
ZNF263	-0.283293796	0.00483	-0.15096589	0.12747	-0.254422558	0.01125
SLC38A2	0.362337271	0.00484	0.211349503	0.10017	0.036344464	0.77757
THUMPD1	-0.259795298	0.00484	-0.066365236	0.46732	-0.126142966	0.1696
SEMA6B	1.109949058	0.00484	0.026612985	0.94639	0.562472185	0.15894
LINC00493	-0.304050889	0.00486	-0.066170968	0.53047	-0.291564125	0.00668
ZNF71	0.585698875	0.00486	0.152139835	0.46612	0.111377948	0.5947
RANBP6	-0.302467414	0.00488	0.051157252	0.62919	-0.276576474	0.00988
CCDC97	0.498378358	0.00488	-0.424815617	0.01936	0.361561091	0.04171
KIAA1009	-0.469833653	0.00488	-0.061286183	0.70901	-0.08855709	0.59337
NDOR1	0.41211753	0.00488	-0.21059473	0.16328	0.17100397	0.2492
UBE2T	-0.432158836	0.00489	0.011512864	0.93778	-0.387476026	0.01094
ARMC10	-0.267777774	0.0049	-0.097241648	0.30288	-0.249099446	0.00877
LUZP1	0.38774277	0.0049	-0.008516658	0.95076	0.16807933	0.22316
TTC30B	-0.463633928	0.00493	-0.128358367	0.42576	-0.342422545	0.03629
HS3ST5	-1.252779672	0.00494	0.530730954	0.19521	-1.408248199	0.00185
FOXJ2	0.486456143	0.00495	-0.208288889	0.23247	0.498192523	0.00402
DTNB	-0.585267774	0.00495	-0.480403566	0.01943	-0.29799181	0.14872
SMPD1	0.536491144	0.00496	0.190134573	0.32288	0.226225148	0.23913
DEDD	0.305712888	0.00497	0.061716224	0.57007	0.257222304	0.01833
TPST2	0.510428854	0.00497	0.304768427	0.09387	0.301194307	0.10044
STRBP	-0.349369831	0.00498	0.143406152	0.24281	-0.071191149	0.56484
GTF2H5	-0.30228758	0.00498	0.076856691	0.46094	-0.20373932	0.05609
RBM12B	-0.535097918	0.00499	-0.200321517	0.29141	-0.308944018	0.10384
SDHA	-0.24736735	0.00499	-0.115118315	0.18917	-0.000505005	0.9954
SELE	3.49860886	0.00498	1.734170012	0.16863	2.162434958	0.08616
TYSND1	-0.529207217	0.00499	-0.25392252	0.17029	-0.044608199	0.80947
ZBTB43	0.372962919	0.005	0.130313119	0.32741	0.197405138	0.13644
SIM1	-1.219611019	0.00503	-0.162279014	0.70518	0.719848886	0.0927
DIAPH2	0.388742449	0.00503	0.550371879	7.01E-05	0.452444061	0.00109
FKBP9L	-1.260823611	0.00504	-1.11040753	0.0134	-0.46848688	0.29607
MEA1	-0.270753508	0.00505	0.186371189	0.04883	-0.099385843	0.29953
RP1-152L7.	-0.671857657	0.00506	-0.255087395	0.28149	-0.3728681	0.11619
BATF3	1.884203058	0.00506	2.567294158	8.41E-05	1.656850062	0.01536
YKT6	0.189818361	0.00511	0.336504697	5.10E-07	0.163169563	0.01581
ST3GAL1	0.605917684	0.00512	-0.087733499	0.68625	-0.285474834	0.18933
GTPBP1	0.426501004	0.00512	-0.235005347	0.12554	0.215267082	0.15891
HDAC1	-0.213262106	0.00513	-0.084130335	0.26608	-0.009316887	0.9022
AC004797.:	-0.552250106	0.00515	0.585294484	0.00242	-0.211465987	0.27984

PPP2R1A	0.239498952	0.00515	-0.072438435	0.39813	0.005537783	0.94848
TXNDC12	-0.340891035	0.00515	0.106830529	0.37721	-0.051395436	0.67221
YES1	-0.253965516	0.00515	0.106883316	0.23671	0.010369596	0.90878
PIWIL1	-2.024337666	0.00519	-1.136713068	0.1271	-0.115447081	0.86471
C2orf69	-0.275044847	0.00519	-0.005436169	0.95467	-0.082683208	0.39321
ARL8A	0.621773911	0.00521	0.293144014	0.19011	0.108743215	0.62705
FRMD8	0.601931374	0.00521	-0.029950898	0.89021	0.127014305	0.5576
ATAD5	-0.439310399	0.00525	-0.117033632	0.45332	-0.202992425	0.19447
ZDHHC23	-0.525906153	0.00525	-0.312619418	0.09156	-0.079023056	0.66832
SMUG1	-0.294951715	0.00525	-0.232942892	0.02596	-0.131708206	0.20788
LPIN1	0.40912426	0.00527	-0.078885737	0.59076	0.526480959	0.00032
AHCY	-0.358918651	0.00527	0.012177272	0.92437	-0.2206665	0.08597
F13A1	2.137788135	0.00529	0.478751183	0.54553	-0.425496162	0.60355
DGKA	0.676466604	0.00532	0.281479864	0.24638	0.324238799	0.18169
LIAS	-0.460639769	0.00533	-0.032747509	0.83821	-0.306582135	0.05847
ATP7A	-0.318684482	0.00534	-0.237659042	0.03704	-0.194857013	0.08786
VWA5A	-0.436570897	0.00535	-0.168673879	0.2776	-0.095748661	0.53779
CCDC15	-0.436367714	0.00537	-0.240690941	0.10732	-0.264895925	0.08068
MORN2	-0.483792902	0.00537	0.348469289	0.03183	-0.127437002	0.44778
TBC1D9	-0.411555847	0.00537	-0.235941649	0.10892	-0.222796646	0.13048
CSPG4	0.764668255	0.00538	-0.260550318	0.34732	0.126957552	0.64594
FAM169A	-0.471661612	0.0054	0.034140838	0.83914	-0.143160615	0.39632
HECTD3	0.401324157	0.0054	0.089134646	0.53782	0.413049251	0.00421
NCAPG2	-0.48055375	0.0054	-0.126277524	0.46089	-0.134663315	0.43277
SLC16A2	0.7930792	0.00539	0.214168639	0.45444	-0.053968413	0.85077
SPTSSA	0.327287685	0.0054	0.766908137	3.96E-11	0.587648776	4.87E-07
PPFIA4	0.608356089	0.00541	-0.105590974	0.63136	0.177459744	0.41828
PTGER4	1.104729277	0.00541	-0.241437918	0.54786	0.309033678	0.4387
CAPN2	0.411346551	0.00542	0.541942035	0.00024	0.175337819	0.23614
RINL	-0.593154164	0.00542	-0.007536188	0.97112	-0.175032643	0.40359
CCNE2	-0.702366312	0.00544	0.114414117	0.64304	-0.237655953	0.34185
CCNT1	0.212334199	0.00544	0.300460349	6.91E-05	0.120572553	0.11483
COL6A6	1.461785699	0.00544	0.459158171	0.38522	-0.870113456	0.10531
BOLA3	-0.459086939	0.00545	0.163475562	0.30467	-0.062663221	0.6982
MYOCD	1.05025852	0.00545	0.472364716	0.21188	0.136465375	0.71886
C10orf10	0.745524095	0.00547	0.296500319	0.27107	0.336087165	0.21371
EPHA1	-0.460628065	0.00547	-0.33834487	0.03935	-0.274511571	0.09563
HNMT	-0.267743812	0.00547	0.071463493	0.45001	-0.024339615	0.79921
RPA2	-0.411401907	0.00549	-0.011331548	0.93814	-0.219371955	0.13632
ATP6V0E2	-0.588110888	0.0055	-0.099306936	0.62837	-0.437165105	0.0359
FAM160A2	0.455326565	0.00552	-0.140142382	0.39506	0.397565777	0.01542
LGALS8	0.432743865	0.00552	0.508045917	0.0011	0.244671666	0.11717
ART3	-1.137498376	0.00553	-1.01304292	0.01131	-0.402440368	0.3099
FAM168B	0.335014985	0.00553	0.091214968	0.44946	0.158309487	0.1901
PM20D2	-0.356045988	0.00553	0.1435687	0.25799	-0.138228558	0.27908
TMEM256	-0.35574886	0.00553	0.235819031	0.05876	-0.114645104	0.36593
AFMID	-0.370937329	0.00554	-0.234350402	0.07731	-0.291650108	0.02878

DUSP19	-0.551052816	0.00555	-0.132042333	0.5004	-0.323353916	0.09946
PRDX1	-0.363861063	0.00554	0.079661632	0.5426	-0.018977781	0.88478
SMC4	-0.409322427	0.00555	0.100476373	0.49519	-0.277244635	0.0602
TMEM159	-0.446767713	0.00555	-0.189935884	0.23176	0.041551495	0.79334
NCOA1	0.304266401	0.00556	0.235427997	0.03186	0.300211872	0.00619
P11-598P2(	0.52193575	0.00557	0.067931449	0.72331	0.202974621	0.29011
ELOVL2	1.25332587	0.00557	1.29525983	0.00399	0.463853432	0.31558
AP1G1	0.200383118	0.00558	-0.04818867	0.5035	0.225179646	0.00179
ZC3H8	-1.000964317	0.00558	-0.642004899	0.07444	-0.742188626	0.03936
SLC16A13	0.525905336	0.00561	-0.008654942	0.96443	0.416667008	0.02865
RNF183	0.662840455	0.00561	0.449937295	0.06023	0.822726662	0.00057
ARID3A	0.581052113	0.00563	-0.407153974	0.05264	0.490223723	0.01951
SAMD13	-0.623864705	0.00564	0.118436117	0.58815	-0.069559862	0.75166
MYO15A	0.728645568	0.00565	-0.343158997	0.21721	0.455473805	0.08845
DES	0.905954021	0.00566	0.75179792	0.02158	0.456035514	0.16497
NAA20	-0.283194499	0.00566	0.175203212	0.07666	-0.037501253	0.70955
ADSS	-0.339618869	0.00567	0.093562914	0.44189	0.02817502	0.81733
DCLK2	0.816250087	0.00567	0.350929874	0.23954	-0.125862299	0.67846
RPL8	-0.363941388	0.00567	-0.061506763	0.63991	-0.170573675	0.19464
XPNPEP2	1.874601085	0.00567	1.814294313	0.00742	0.624159613	0.36551
FTL	-0.313142111	0.00568	-0.032962165	0.77079	-0.283624434	0.01224
FGF23	0.781360673	0.0057	0.248538813	0.38411	0.588556803	0.03808
ITIH2	-0.719663379	0.0057	-0.481256463	0.06441	0.195713334	0.45188
PTPRJ	0.612820001	0.00573	0.478011902	0.03086	0.45541481	0.04011
TBC1D20	0.204314843	0.00574	-0.075056343	0.31286	0.009286512	0.90086
CSRP1	0.491728415	0.00575	0.385572838	0.03031	-0.103366082	0.56222
FGF2	0.981086077	0.00575	0.369583835	0.30031	-0.076540548	0.8327
SRSF1	-0.322665512	0.00575	0.069161069	0.55337	-0.046095056	0.69301
HS3ST1	0.707874297	0.00575	0.298059688	0.24468	0.473243757	0.06465
IRAK1	-0.522203085	0.00577	-0.021804419	0.90776	-0.025977108	0.89024
LAMA1	-0.860416145	0.00577	-0.564779888	0.06937	-1.220383271	9.26E-05
NEK3	-2.507106349	0.00577	-0.130048888	0.87786	-1.244180194	0.15131
YBX3	0.392345713	0.00578	0.73818554	1.91E-07	0.353958978	0.01277
LAPTM4B	1.003852317	0.00578	1.147485592	0.00158	1.238081836	0.00065
TYK2	0.310655396	0.0058	-0.058724001	0.60304	0.013454668	0.90519
IDS	0.410583066	0.00581	0.198947644	0.18167	0.199025818	0.1818
DUSP5	0.902952457	0.00582	0.578442327	0.0773	0.310884482	0.34296
ZNF90	-0.50774206	0.00583	-0.599571775	0.00095	-0.257966336	0.15389
ZMYM4	0.32729417	0.00584	0.443894525	0.00018	0.197048009	0.09679
HS3ST3A1	1.822374954	0.00585	0.650428558	0.33076	-0.148442293	0.82931
CCDC104	-0.369918616	0.00586	0.028207009	0.8311	-0.245416624	0.06605
PTCH2	-0.847720314	0.00586	0.427250903	0.16232	-0.291492929	0.34194
YIPF4	-0.381949773	0.00587	-0.01928477	0.88901	-0.323140981	0.0196
CCT4	-0.284039408	0.00587	0.245472414	0.01675	-0.086626111	0.39999
SLC25A15	-0.391751775	0.00587	0.02078177	0.87835	-0.203009552	0.15077
NOP56	-0.349995742	0.00588	0.065933524	0.60201	-0.22097136	0.08145
SMARCAD1	-0.214828033	0.00588	0.081900671	0.28728	-0.188821254	0.01526

MT-ND2	0.825895919	0.00588	0.989464549	0.00096	1.28492296	1.81E-05
CABLES2	0.386171201	0.0059	-0.149756062	0.29358	0.182337867	0.19716
TOMM20	-0.281328432	0.00592	0.182649259	0.07301	-0.139230447	0.17267
MASTL	-0.367664215	0.00593	-0.0717315	0.58769	-0.2683305	0.04392
B4GALNT4	0.571909107	0.00594	0.090834449	0.66321	0.232958161	0.26407
POLE4	-0.506403462	0.00594	-0.03102014	0.86057	-0.209704876	0.24279
PPP1R13L	0.651637792	0.00594	-0.349558583	0.15418	0.579529048	0.01457
TFCP2L1	-0.911210207	0.00594	-0.748977109	0.02365	0.018067796	0.95624
TACR3	-1.040475281	0.00595	0.447459839	0.23418	-0.845873286	0.02517
LZTS3	0.444103253	0.00596	0.034576607	0.83167	0.438194344	0.00665
HEYL	1.062744656	0.00599	-0.642813031	0.11141	0.447393646	0.25272
HOXB9	2.118206863	0.00599	2.041082319	0.00805	-1.767398025	0.03593
MARK4	0.540373125	0.00599	-0.006840839	0.97252	0.272416835	0.16831
TMEM258	-0.35648995	0.00599	0.032714497	0.79864	-0.209864103	0.1045
CBWD1	-0.507349497	0.00601	0.233007324	0.20437	-0.06687026	0.71649
GUCY1B3	0.497034165	0.00603	0.187946313	0.29981	0.125136387	0.49159
KIAA0355	0.39735458	0.00605	-0.179188413	0.21736	-0.146328885	0.31413
TMEM219	-0.312068974	0.00605	-0.096440453	0.39031	0.013726107	0.90264
TMEM52	-1.201040978	0.00606	-0.563539831	0.16894	-0.772357992	0.06327
CCL11	1.739128006	0.00607	1.373512968	0.03101	0.964908676	0.13684
GPR176	0.895580004	0.00609	0.451804624	0.16967	-0.181986832	0.5888
RANBP1	-0.327248901	0.00611	0.269846108	0.02256	-0.120681082	0.31047
RPAIN	-0.25293634	0.00611	-0.23178925	0.01206	-0.395505731	1.92E-05
TMEM11	0.373323874	0.00611	0.31058838	0.02163	0.286379345	0.03543
CTSZ	-0.471549182	0.00613	-0.048102912	0.77849	-0.29015163	0.09085
BCL2L14	-0.554340061	0.00615	-0.154671877	0.43511	0.296508092	0.13263
COTL1	0.553633145	0.00614	0.614597409	0.00233	0.266566673	0.18744
PRKX	-0.432755464	0.00615	-0.03807092	0.8085	-0.311685985	0.0481
SIRPA	0.62767761	0.00615	0.259525647	0.25924	-0.183416072	0.43307
SPAG16	-0.357774614	0.00614	0.148585735	0.24324	-0.058739454	0.64703
PHF3	-0.247364969	0.00616	0.051406729	0.56771	-0.123090502	0.17238
EEF1A1P13	-0.681541882	0.00617	0.212598161	0.37381	-0.619782802	0.01229
LENG8	1.100199543	0.00617	-0.237062617	0.56735	1.458033605	0.00025
VNN2	1.175507904	0.00619	1.605068395	0.00014	1.372507359	0.00133
NUP43	-0.292247363	0.0062	0.118891086	0.26018	-0.128690962	0.22537
AGFG1	0.330999117	0.0062	0.347484481	0.00401	0.345537636	0.00425
ZNRF3	-0.463185702	0.00621	-0.206625101	0.216	-0.386236386	0.02224
CCDC141	-1.34095871	0.00624	-0.428119706	0.37897	-1.185104858	0.01556
GPM6B	0.634466754	0.00624	0.168095267	0.47015	0.242586281	0.29881
SH2D3C	1.947522956	0.00624	0.438636146	0.53944	1.343940596	0.05541
RSL1D1	-0.356486521	0.00625	0.289695363	0.02574	-0.079967582	0.53897
SLC29A1	-0.538556051	0.00625	-0.313668177	0.10985	0.041676368	0.8315
DACT3	0.569948112	0.00625	-0.18720027	0.37312	0.026971326	0.89774
SLC9C2	-1.631359034	0.00626	-0.422374291	0.47124	-1.445992128	0.0143
C1orf74	-0.479602833	0.00627	-0.049446365	0.77025	-0.189253068	0.27064
ZNF730	-0.411475762	0.00627	-0.042444176	0.7727	-0.463776166	0.00208
CYR61	0.851089069	0.00638	0.6988701	0.02499	0.353538097	0.26107

RRC37A15	-1.144276502	0.00638	-0.538007486	0.16698	-0.479126672	0.2212
PRL	3.353057574	0.00642	3.691639108	0.00267	1.114140831	0.36961
GLMN	-0.506726102	0.00643	-0.083125114	0.64773	-0.233073927	0.20625
COL23A1	0.936822097	0.00644	-0.477572705	0.19306	0.288126404	0.41952
LMNB1	-0.448356702	0.00644	-0.147107635	0.37008	-0.361360004	0.02799
TMEM132A	0.605941741	0.00644	0.039949986	0.85783	-0.109879743	0.62403
CHRNA7	1.571359047	0.00645	0.52367383	0.37019	0.294497329	0.61631
PNPLA2	0.710571549	0.00645	-0.366573272	0.16888	0.499077572	0.05753
SOWAHA	-0.755096553	0.00647	-0.254165826	0.3464	0.081184784	0.76227
DCUN1D3	0.38888402	0.00649	0.249545275	0.07973	0.365279855	0.01062
ECE1	0.655377787	0.00649	0.24161382	0.31598	-0.185487933	0.44197
STAG3L4	0.457135621	0.00649	-0.102069159	0.54405	0.023666594	0.88827
ZNF700	-0.446368998	0.0065	-0.520281267	0.00146	-0.30663988	0.05997
ENTPD5	-0.412733577	0.00652	-0.487096932	0.00131	-0.060828112	0.68765
FANCC	-0.549977399	0.00652	-0.454955642	0.02383	-0.61492032	0.00232
RNF186	1.817753425	0.00653	2.359877394	0.0004	1.27129068	0.05756
HEATR1	-0.347207486	0.00654	0.158189165	0.21336	-0.165241282	0.19489
ITGB6	0.772361151	0.00654	0.493668146	0.08219	0.52802006	0.06309
FKBP15	0.303297362	0.00655	-0.033507759	0.76376	0.213610378	0.055
KIAA0100	0.287858262	0.00656	0.093496028	0.37675	0.214879845	0.04227
MRPL52	-0.369266004	0.00658	-0.03358719	0.80205	-0.105530222	0.43344
NT5DC1	-0.538154662	0.00658	0.079946478	0.68423	-0.14081476	0.47498
ARID2	0.261557058	0.00658	0.259114438	0.00696	0.272019718	0.00469
PET117	-0.563558762	0.00659	0.025775064	0.89703	-0.232884676	0.25105
PRPS1	-0.380754312	0.0066	0.073556175	0.59683	-0.130676156	0.34915
PLEKHM2	0.36916583	0.00661	-0.007808332	0.95444	-0.011737354	0.93143
GUSBP1	-0.484274469	0.00663	-0.214486337	0.2235	-0.414250985	0.02015
LIMK2	-0.312828959	0.00664	-0.344918869	0.00264	-0.056689465	0.62054
NDUFB9	-0.276275787	0.00664	0.071480718	0.47571	0.021168224	0.83361
ZNF609	0.278218312	0.00664	-0.164751829	0.10874	0.15233908	0.13767
FGF5	0.668646689	0.00665	-0.165737499	0.50954	0.554551751	0.02463
SERPINI1	-1.016892745	0.00665	-0.46387161	0.20283	-0.683036432	0.06584
SUN2	0.403897784	0.00665	-0.12827991	0.39006	0.177484195	0.23287
FAM127B	0.421246288	0.00669	0.130423386	0.4047	0.291236195	0.06208
HIST1H2AI	-0.570983089	0.0067	-0.136588099	0.51558	-0.536266644	0.01085
ELK3	0.521280304	0.00671	0.263203716	0.17047	0.091669968	0.63525
ZDHHC2	-0.633041881	0.00672	-0.0349029	0.88082	-0.303495398	0.19279
LSM5	-0.359307817	0.00673	0.047979576	0.71479	-0.207075998	0.11691
IRF1	0.5400727	0.00674	0.186893527	0.35068	0.221130948	0.26866
MAP9	-0.344601179	0.00675	0.018757523	0.88117	-0.272666414	0.03162
MNAT1	-0.312124143	0.00675	0.16890254	0.13215	-0.220508097	0.05332
SYVN1	0.52718516	0.00675	-0.293224828	0.13725	0.305483814	0.11714
UACA	-0.40015586	0.00675	-0.225711709	0.12516	-0.101808792	0.48962
LRRC31	-0.91937486	0.00677	-0.495915284	0.1342	-0.633782735	0.0567
F816-ZNF3	-0.582405337	0.00677	-0.336461183	0.11339	-0.099737457	0.63742
TD-3088G3	1.114316036	0.00678	-0.110694045	0.79134	0.113665104	0.78784
GIPC2	-0.809885594	0.00678	0.086509282	0.76815	-0.215873145	0.46475

C19orf57	-0.91631917	0.0068	-1.058138803	0.00171	-0.317078369	0.33273
THSD7B	1.881429721	0.00681	1.16107306	0.09761	0.616758667	0.38871
.C024592.1	3.442894069	0.00682	3.583475303	0.00488	2.886914811	0.02344
ANXA2	0.294905352	0.00682	0.47998239	1.06E-05	0.336170765	0.00204
SRSF5	-0.304285335	0.00682	-0.151054332	0.17891	-0.148593248	0.18585
CA13	-0.431675616	0.00684	-0.230608271	0.14353	-0.348738385	0.02797
ELFN1	1.025118958	0.00684	-0.097127298	0.80588	0.08444141	0.83049
NDFIP2	0.45603034	0.00684	0.375172194	0.02559	0.365322551	0.03002
EME1	-0.508163583	0.00686	-0.338317649	0.06369	-0.277506004	0.134
TJP3	0.644330589	0.00687	0.160813348	0.5006	0.549058257	0.02125
DGKB	1.423064015	0.0069	0.447123182	0.4011	0.955948727	0.07276
MGST2	-0.556000782	0.00692	-0.103843972	0.61227	0.054170833	0.79138
APEH	-0.344095308	0.00693	-0.182176208	0.15125	-0.209005499	0.10017
DAAM2	0.80113555	0.00694	0.167662305	0.57339	0.283878263	0.34074
AMZ2P1	-0.58894733	0.00694	-0.159418015	0.45593	-0.278834185	0.20019
UBALD1	0.604742894	0.00695	0.062924481	0.78414	0.446137884	0.04738
SRRM1	0.232466711	0.00699	-0.073417782	0.3947	0.192851971	0.0249
FAM177B	-0.958092416	0.00701	-1.665031467	3.29E-06	0.104538953	0.76613
RPL41	-0.255681413	0.00701	0.083298766	0.37935	-0.116557153	0.21888
GPR183	0.842887908	0.00702	0.673556342	0.03111	-0.100051919	0.75706
MAST2	0.467156903	0.00702	0.381329849	0.0281	0.493843107	0.00445
C2CD4A	-1.039606296	0.00703	-1.339628182	0.00053	-0.65561964	0.08714
MTF2	-0.302226	0.00705	-0.175205147	0.11613	-0.316685113	0.0047
LRRC41	0.480660305	0.00706	-0.238348824	0.18853	0.136264824	0.44803
C19orf48	-0.370069999	0.00711	-0.275734487	0.04366	-0.226600946	0.09804
RPL27A	-0.30752201	0.00711	0.162284818	0.15508	0.050267935	0.65976
ECHDC1	-0.269997319	0.00712	-0.008982666	0.92754	-0.105678319	0.28915
BAG3	0.33645947	0.00713	-0.25248364	0.04641	-0.000731759	0.99538
CCND1	-0.494570179	0.00713	-0.808102887	1.09E-05	-0.408656001	0.02611
CORO7	0.44205756	0.00714	-0.227896328	0.17011	0.069060213	0.67659
SLC25A26	-0.37702818	0.00716	0.222956187	0.10471	-0.041468206	0.76405
ITPKA	-0.716989713	0.00717	-0.892865981	0.00072	-0.205693858	0.42783
ENY2	-0.31244876	0.00718	0.142737497	0.2154	-0.302079317	0.00938
CCDC93	0.44724803	0.00719	0.168024759	0.31187	0.501976052	0.00251
SEC61G	-0.312018957	0.00719	0.343985066	0.00263	-0.092476742	0.42315
EPS8L3	0.489262997	0.0072	0.059439778	0.74436	0.735615613	5.22E-05
RPS29	-0.389406475	0.00721	0.262801771	0.06899	0.025802658	0.85847
PILRB	0.432530168	0.00722	-0.26917412	0.10071	0.414364496	0.00973
KIAA1191	0.352618154	0.00723	-0.009566809	0.94198	-0.058049623	0.65951
LIPA	-0.343441703	0.00723	0.076804326	0.54289	-0.020422283	0.87241
MT-CO1	0.526281858	0.00726	0.370588612	0.0587	0.677086858	0.00055
DHDDS	0.289577648	0.00727	-0.108713024	0.31394	0.156530391	0.1465
HD1-EIF4E	0.721748092	0.00727	0.033308583	0.90173	0.806749688	0.00268
CMTM1	0.954432924	0.00729	0.147272677	0.68818	0.547731251	0.13023
ESRRA	0.355662541	0.00729	0.175772769	0.183	0.669856578	3.00E-07
RPL39L	-0.515312373	0.00729	-0.002978853	0.98706	-0.357348928	0.05823
THY1	0.614514223	0.00729	0.156637675	0.49461	-0.009732252	0.96619

TMEM106A	1.375811867	0.00729	0.823136033	0.10994	0.977757803	0.05718
SSR4	-0.359403833	0.0073	0.012238743	0.92691	-0.125806286	0.3466
QDPR	-0.545180846	0.00731	0.066514944	0.73222	-0.426186238	0.0341
SLC30A7	0.273456329	0.00731	0.371544671	0.00026	0.210890035	0.03847
CCDC90B	-0.229841425	0.00732	-0.027273275	0.74815	-0.123220755	0.14854
RPL10A	-0.300169241	0.00732	0.10050498	0.36854	-0.105473053	0.34568
DPH6	-0.605231754	0.00732	-0.119911771	0.5896	-0.229426304	0.30294
SEPHS2	-0.376868947	0.00732	0.354740374	0.01013	-0.045915399	0.74164
CSRNP2	0.367370848	0.00735	-0.020515567	0.88154	0.026798892	0.84591
ARID5B	0.355888455	0.00736	0.0094475	0.94328	0.025785386	0.84628
MMP3	1.735383793	0.00736	0.990765537	0.12624	0.800541947	0.21735
RBM44	-1.141168417	0.00736	-0.661519728	0.11333	-0.766485661	0.06401
SLC41A1	0.521303455	0.00737	0.098738672	0.61344	-0.226490595	0.24912
CRTAP	-0.333106631	0.00738	-0.018324234	0.88215	-0.26387719	0.03347
MAPK8IP3	0.563880621	0.00738	-0.279758484	0.1872	0.224067981	0.28779
PPP1R11	0.310455343	0.00738	0.23221859	0.04458	0.365934383	0.00156
SLC27A2	-0.864019868	0.00738	0.108640772	0.73299	-0.275254943	0.38898
DGKD	-0.611193918	0.00741	-0.382149204	0.09177	-0.203140157	0.37034
MPHOSPH	-0.341368045	0.0074	0.216963854	0.0796	-0.233588182	0.06487
SYNM	0.663266769	0.00741	-0.061614586	0.80499	-0.250328463	0.31912
NUF2	-0.493224943	0.00742	0.294365071	0.10144	-0.334153713	0.06703
P11-889L3	-0.574779025	0.00742	0.118276267	0.56147	-0.341459415	0.10479
GBP3	0.485629746	0.00743	0.512382441	0.00468	0.536360026	0.00309
TGIF2	0.75144646	0.00743	0.661230959	0.01839	0.302901463	0.28496
ALDH6A1	-0.4123273	0.00743	-0.138962988	0.36433	-0.154671431	0.31327
MICB	1.117090356	0.00745	0.446002455	0.31104	0.584445978	0.17502
P11-777B9	-2.516168117	0.00747	0.22938055	0.80675	-3.063941413	0.00114
LRRC6	-0.932885665	0.00748	-0.313949368	0.35659	-0.31933158	0.35482
PPP1R14B	-0.323003425	0.00748	0.020433339	0.86404	-0.250394091	0.03753
CCDC92	0.679680634	0.00748	0.066305146	0.79736	0.174366255	0.49865
ATPAF1	-0.406143049	0.0075	0.133524119	0.37381	-0.120124738	0.4263
TRIM9	1.070880221	0.00751	0.310367761	0.44023	-0.075253667	0.85308
TICAM1	0.924773169	0.00752	0.35231212	0.31295	0.623239485	0.07329
CRTC3	0.519645595	0.00757	-0.057287879	0.77021	0.061300226	0.7542
DOPEY2	-0.55332118	0.00757	-0.971278703	2.75E-06	-0.330234939	0.1099
EVI5	-0.361798652	0.0076	0.056498854	0.6757	-0.016486207	0.90312
ABHD17A	0.546062758	0.00764	-0.058456677	0.77629	0.307922864	0.1334
MTHFD1	-0.362912617	0.00764	0.12031352	0.3725	-0.072784547	0.59111
PTGER2	-0.701203416	0.00764	0.131581349	0.61016	-0.156319395	0.54713
DENND4C	-0.325576131	0.00764	-0.205596322	0.09157	-0.02475462	0.83893
PEX6	-0.328451228	0.00765	-0.432850806	0.00042	-0.276978315	0.02393
PRDM6	1.407225725	0.00766	0.784775406	0.1402	0.619587974	0.24599
TTPA	-0.897197264	0.00767	0.029637984	0.92455	-0.838155184	0.01114
BBS10	-0.353421142	0.00768	0.200863075	0.11875	-0.219389434	0.09514
LMBRD2	-0.609628953	0.00768	-0.153045368	0.50172	-0.11182783	0.62391
CYTH2	0.367964922	0.00769	-0.434259661	0.0018	0.048647359	0.72514
TGFBR2	-0.320607314	0.0077	-0.110006433	0.3591	-0.191785141	0.11042

TPCN1	0.43583339	0.00771	-0.154429071	0.34813	0.099022593	0.54516
LTBP4	0.561499536	0.00772	-0.597653522	0.0052	-0.044687621	0.83282
HOXC8	1.086120889	0.00776	0.488929004	0.23465	0.573232684	0.16407
DCK	-0.521228084	0.0078	-0.23632768	0.22297	-0.47461125	0.01461
HS6ST1	0.338658382	0.0078	-0.223747958	0.08064	-0.029501553	0.8177
ZNF449	-0.375720749	0.00783	-0.197550883	0.15503	-0.195812484	0.16155
GNG4	0.833323937	0.00787	0.982674831	0.0017	0.08325961	0.7927
PSMA6	0.278637505	0.00786	0.548856432	1.44E-07	0.222631062	0.03372
FAM13A	-0.654023082	0.00788	-0.755508154	0.00214	-0.199069851	0.41838
LDLR	0.300949288	0.00787	-0.284571903	0.01205	0.31369257	0.0056
SMAD1	0.574993677	0.00787	0.686704995	0.00151	0.022508966	0.91757
EHD1	0.649012734	0.00796	0.30761382	0.21182	0.354323625	0.14965
IFNAR2	0.543689253	0.00795	0.640747843	0.00171	0.303445884	0.13862
INCA1	0.896620391	0.00796	0.106295552	0.76587	0.905507217	0.0067
TAB3	0.33765963	0.00796	0.044473125	0.72679	0.330953235	0.00914
DNAH5	0.684751434	0.00797	0.501310459	0.05194	0.552362883	0.03215
FAM208B	-0.266059558	0.00797	-0.1096096	0.27225	-0.285163491	0.00443
TBC1D25	0.547795625	0.00798	-0.298970026	0.15227	0.281815973	0.17522
CECR2	1.388909252	0.00799	0.627292231	0.23288	-0.048367429	0.93106
LYAR	-0.515562613	0.008	0.175302437	0.35964	-0.201932391	0.2952
UQCC2	-0.414107977	0.00805	0.02389575	0.87719	-0.065623809	0.67212
NDUFS4	-0.304862394	0.00805	0.013912031	0.90201	-0.099616362	0.3822
IFT122	-0.438117936	0.00806	-0.512619364	0.00186	-0.278323121	0.09092
LRRN2	1.222671189	0.00807	0.303106021	0.51975	-0.228763724	0.62848
TMEM92	0.602806089	0.00807	0.039156077	0.86386	0.731115878	0.00127
CARD9	1.116529168	0.00807	0.401610627	0.34787	0.529202417	0.2157
C9orf142	-0.524340493	0.00808	-0.146629561	0.45141	-0.009883095	0.95942
TTC40	1.147338207	0.00814	0.669688275	0.12385	0.460448383	0.29651
GLB1L2	-0.512801803	0.00815	-0.397221473	0.03977	0.002689943	0.98887
TXNIP	-0.541735694	0.00815	-0.411577265	0.04438	-0.191795365	0.34878
POLE3	-0.369329158	0.00817	0.061968622	0.65373	-0.161932386	0.24398
PTP4A2	0.309897807	0.00817	0.469074395	6.16E-05	0.317814312	0.00667
CDC42EP1	0.518731495	0.00818	-0.033767023	0.86356	0.572840158	0.00348
CAB39L	-0.462669872	0.00819	0.010904557	0.94972	-0.014153402	0.93491
RQCD1	0.243418349	0.00819	0.283946946	0.00193	0.197714985	0.0318
TCAIM	-0.303313673	0.00822	0.034641316	0.75944	0.034195738	0.7632
EMB	-0.714013893	0.00823	0.046573783	0.86184	-0.687825917	0.01093
LIN9	-0.418347056	0.00823	0.090767074	0.55622	-0.33974472	0.03107
ZNF33B	-0.654647658	0.00823	-0.40732981	0.09627	-0.253765744	0.3055
KIAA0020	-0.274829658	0.00825	0.261642971	0.01066	-0.110998169	0.28343
GARS	0.385610906	0.00826	0.401992504	0.00581	0.34508122	0.01805
C2orf61	-0.699726412	0.00827	-0.114993553	0.64093	0.198193406	0.4177
WWTR1	0.557540365	0.00833	0.281600077	0.18252	0.201039321	0.34236
THNSL1	-0.403188673	0.00834	0.070493431	0.63122	-0.189023997	0.20789
FAM115A	-0.19479382	0.00836	-0.024936848	0.73401	-0.340838491	4.09E-06
C8orf37	-0.41889394	0.00838	-0.220924644	0.15016	-0.355733005	0.02365
NUTF2	0.2765645	0.00837	0.574424967	3.80E-08	0.128031842	0.22283

WBP5	-0.249801414	0.00837	0.118107775	0.20646	-0.215726278	0.02244
TRIM40	2.56866474	0.00838	1.411539418	0.15301	1.826977548	0.06444
PDHA1	-0.231871975	0.0084	0.068383545	0.4313	0.158908696	0.06801
UBE3D	-0.700147816	0.00841	-0.022620703	0.93088	-0.279311186	0.28752
HTR1B	-1.190183831	0.00847	-0.097850389	0.82677	-0.104044504	0.81615
CPSF3L	0.333222655	0.00852	0.133042619	0.29429	0.148837496	0.24072
RPL5	-0.350382682	0.00852	0.453277436	0.00066	-0.183234531	0.16881
TEX10	-0.324523513	0.00852	0.128072772	0.29485	-0.480762354	9.88E-05
BCCIP	-0.294124139	0.00854	0.212850032	0.05394	-0.125680692	0.25912
SCN8A	-1.133602475	0.00859	-0.953242501	0.02616	-0.349917856	0.41324
CDRT1	1.641315171	0.0086	0.55013998	0.37692	1.57765008	0.01023
MLPH	-0.701290672	0.00862	-1.113569279	3.12E-05	-0.079202379	0.76555
PTCD2	-0.459138115	0.00864	-0.306725678	0.07615	-0.570003341	0.00111
CSTF2T	-0.258266029	0.00869	-0.172758811	0.07655	-0.212133535	0.03059
YAE1D1	-0.347124566	0.00872	0.155739169	0.22306	-0.344970106	0.00892
NFKBIE	0.776686628	0.00874	0.360350639	0.22829	0.356839813	0.23651
FAM169B	2.118830301	0.0088	1.626614256	0.04831	2.083287833	0.00997
PDZD8	0.531694685	0.0088	0.534950806	0.0083	0.520134284	0.01037
RAB5C	0.247594422	0.00883	0.181203695	0.05471	0.15598402	0.09917
CD274	1.87534467	0.00883	1.169068619	0.10113	1.036593971	0.15362
MRPL15	-0.319664027	0.00885	0.294296998	0.01274	-0.171224504	0.15598
ENOSF1	-0.373290555	0.00887	-0.38195696	0.00727	-0.122934714	0.38536
STK10	0.553232174	0.00887	0.11604103	0.5831	0.241751168	0.25406
TNIP3	1.094525975	0.00891	0.712271213	0.09275	1.04768555	0.01239
RBMX	-0.204108025	0.00892	0.17011734	0.02876	-0.093392461	0.23102
STX10	-0.34180429	0.00893	0.008132064	0.94955	-0.275183895	0.03449
RALY	-0.228989945	0.00894	-0.019734118	0.82099	-0.069369631	0.42765
SCPEP1	-0.353047106	0.00894	-0.067886234	0.60816	0.06920441	0.60331
PPID	-0.386552272	0.00896	0.362189004	0.01322	-0.145406433	0.32293
NDNF	1.094215541	0.00897	1.063051433	0.01085	-0.018723144	0.96557
NTN1	0.908017372	0.00897	0.569774206	0.10125	0.450939055	0.19554
BMS1	-0.223188025	0.00901	0.183958112	0.02949	-0.077820046	0.3605
MARS	0.331454297	0.00901	0.249058065	0.05005	0.224408362	0.07724
AC010620.1	-0.347777354	0.00905	-0.451430083	0.00069	-0.446259042	0.00082
CNPY2	-0.344330044	0.00903	0.065759224	0.61554	-0.16099993	0.22086
DPH5	-0.338829769	0.00904	0.303765482	0.01544	-0.012248962	0.92348
PRKACB	-0.548822328	0.00905	-0.563213228	0.00727	0.266146132	0.20298
SH2D3A	0.414773029	0.00905	-0.154575765	0.33751	0.283674797	0.0746
SPIN2B	-0.475743852	0.00904	-0.385577142	0.02646	-0.038201866	0.82584
B3GALT5	1.003257688	0.00906	0.346950937	0.36818	1.012102452	0.00847
CD3G	-1.040925092	0.00908	-1.50857388	0.00016	0.887419217	0.02435
ERLEC1	-0.277811429	0.00908	0.037259936	0.72422	-0.169265823	0.11098
IGF1R	0.542543044	0.00908	0.003164353	0.98788	-0.076372668	0.71391
FKBP9	-0.254481806	0.0091	-0.218760518	0.02464	-0.186752021	0.05566
PDK4	-1.182297192	0.0091	0.011749077	0.97879	-0.472915001	0.29012
ELFN2	1.710335941	0.00911	0.320806672	0.62792	-0.266809624	0.6975
NCOR2	0.595992839	0.00911	-0.252348222	0.27299	0.384469319	0.09289

DKKL1	1.093179008	0.00912	0.205992859	0.63583	0.255180328	0.55689
PARP12	0.533328172	0.00913	0.537797444	0.00818	0.728496557	0.00034
IGSF3	0.408028791	0.00914	0.135533904	0.38609	0.360908679	0.02104
TCF20	0.211989787	0.00914	0.173309761	0.03169	0.207753866	0.01038
CMPK2	-0.66022944	0.00915	-0.260505965	0.27787	-0.600768046	0.01561
ZDHHC21	-0.406979558	0.00921	-0.276839253	0.0754	-0.145002614	0.3521
EIF1AXP1	-0.83362232	0.00924	0.076437079	0.80644	-0.141208056	0.65296
CWC22	-0.22544728	0.00925	0.132239871	0.11776	-0.055611105	0.51665
FABP2	1.86357825	0.00925	2.081389741	0.0036	1.196864288	0.0956
MYO7B	1.667478047	0.00925	1.311151116	0.04067	1.342760907	0.03633
TDO2	1.553114921	0.00925	0.923988598	0.12189	-0.226824121	0.70634
EFCC1	0.772856352	0.00927	-0.421847796	0.17879	-0.022271837	0.9425
PARPBP	-0.428623187	0.00928	0.065500532	0.68432	-0.229792144	0.15877
KLHL17	0.712634123	0.00929	0.047132329	0.86537	0.726181292	0.00768
ZNF677	0.370422447	0.00929	0.2113313	0.13778	0.111540502	0.43654
CCDC64B	0.505229069	0.0093	-0.712584979	0.00042	0.588319258	0.00232
METTL5	-0.300190361	0.00931	0.262577987	0.01922	0.036489894	0.74783
GSDMC	1.669829581	0.00933	-0.085783143	0.90716	0.276562562	0.68363
ARIH2	0.218526782	0.00934	0.037658926	0.65246	0.174479579	0.03787
EDEM3	-0.38014938	0.00934	-0.190033189	0.19311	0.069645595	0.63341
IKBKB	0.408979332	0.00933	-0.098533851	0.53223	0.098097286	0.53297
MTR	-0.246972197	0.00934	-0.187489423	0.04795	-0.157439474	0.09714
MARK2	0.322143571	0.00936	-0.165726383	0.18444	0.375973276	0.00241
GRHL2	-0.409326001	0.0094	-0.444353746	0.00472	-0.068171824	0.66406
POLR3K	-0.521906432	0.0094	-0.134598169	0.48855	-0.428682502	0.03076
P13-128O4	1.051134532	0.00941	0.301412966	0.47522	0.714083874	0.0837
ZNF180	-0.348513622	0.0094	-0.153152897	0.24503	-0.033919824	0.79758
SEPW1	-0.253900862	0.00942	-0.015064032	0.87678	-0.22707719	0.0201
PTHLH	1.84070771	0.00944	1.508651037	0.03371	2.261511535	0.00138
TAF7	-0.233123024	0.00943	0.181796157	0.0401	-0.115987431	0.19455
S1PR2	0.70609737	0.00946	-0.145032925	0.59628	-0.05701509	0.83513
CCNYL2	-1.636072794	0.00947	-0.734493609	0.20052	-1.02996348	0.08195
ABCA1	0.591904932	0.00948	0.283715901	0.21382	-0.207088278	0.3668
CKAP4	0.211788432	0.00949	0.191513535	0.01879	0.163890095	0.04468
XPO4	-0.277249461	0.0095	0.123446443	0.24273	-0.05146502	0.6281
FKTN	-0.475305162	0.00951	0.113616843	0.53236	-0.256651343	0.16004
HMGB1P14	0.995629852	0.00951	0.367143547	0.35774	0.762672962	0.05039
RAD21	-0.246573898	0.00953	0.122703993	0.1947	-0.099826069	0.29306
GBGT1	0.714021371	0.00956	-0.032657423	0.90764	-0.114666294	0.69476
SWAP70	0.322572592	0.00957	0.335103732	0.00657	0.419559288	0.0007
TAS2R4	-0.891788442	0.00957	-1.117864328	0.00121	-0.607889693	0.07069
ARPC3	-0.227617898	0.00959	0.134381701	0.12378	0.032967434	0.70649
C14orf166	-0.213003957	0.00958	0.219140562	0.00711	-0.091868956	0.26288
TYRO3	-0.354295197	0.00961	-0.462242219	0.00072	-0.463661694	0.0007
CDC45	-0.672077972	0.00962	0.068681425	0.78896	-0.280719419	0.27606
RAB3GAP2	0.297391549	0.00964	0.325181617	0.00448	0.154976503	0.17701
FCGRT	-0.373610119	0.00966	-0.149025759	0.30132	-0.231129279	0.10918

RELT	0.64658968	0.00968	0.253248321	0.31145	0.558624788	0.02555
WDR93	1.265823404	0.00968	0.235753412	0.64524	0.861489168	0.08315
SIX5	0.795142844	0.0097	-0.108003212	0.7289	0.483943017	0.11753
ATP1B2	0.994084725	0.00976	-0.332735771	0.40435	0.244948716	0.53023
BAI3	0.761925016	0.00974	0.025942548	0.93076	0.504825604	0.08952
PAX5	1.529772406	0.00975	1.482531986	0.0115	2.36123543	5.91E-05
PIP5K1A	0.303500003	0.00976	0.143089463	0.22267	-0.003305557	0.97762
RUSC1	0.284265925	0.00975	-0.239258264	0.03296	0.335377525	0.00219
P11-697N18	1.201163288	0.00977	0.507048035	0.28983	0.372734709	0.44544
BHMT	-1.184871878	0.00978	-1.591263004	0.00026	-0.29823052	0.46767
CLDN9	-0.884231562	0.00978	-0.798597368	0.0162	-0.028200658	0.92827
MCCC1	-0.460152151	0.00978	-0.21841333	0.2153	-0.136257717	0.44096
SQSTM1	0.497721564	0.00979	0.202176293	0.29419	0.376011279	0.05107
STX3	-0.512795642	0.00981	-0.248675712	0.20913	-0.321650273	0.10479
ADAMTS2	1.251412722	0.00982	0.66767287	0.1689	0.553660177	0.2558
UQCR11	0.300676891	0.00983	0.095155001	0.41291	0.334151545	0.00399
RPL35A	-0.296207258	0.00984	0.152807581	0.18211	-0.063909955	0.57718
ZNF592	0.227026092	0.00986	-0.405439342	5.65E-06	0.212959865	0.01548
BRE	-0.341564642	0.00987	-0.200486404	0.12369	-0.276747989	0.03497
MCM10	-0.605509805	0.00986	-0.397586911	0.08856	-0.712932466	0.00234
REEP4	0.375405441	0.00987	-0.307174979	0.04031	0.281803095	0.05317
CCDC17	0.86084217	0.0099	0.115548986	0.74196	0.966031947	0.00363
CPA6	-1.52838109	0.0099	-0.617991485	0.24643	-0.80699696	0.14295
OSBPL3	-0.33944308	0.00989	-0.279850839	0.03181	-0.162552162	0.21314
T6GALNAC	0.552157998	0.0099	0.132999657	0.53431	-0.145201501	0.49933
PDE7A	-0.215591366	0.00992	-0.260879919	0.00166	-0.073733398	0.37467
C19orf10	-0.338938194	0.00994	-0.096336223	0.46012	-0.106399525	0.41548
STK38L	-0.353791268	0.00994	0.053033586	0.69818	-0.050170084	0.7141
TARS2	-0.307730364	0.00993	-0.31149245	0.00771	-0.258761824	0.02776
ZNF281	0.260677774	0.00995	0.159165665	0.11489	-0.125264382	0.21905
RANBP2	-0.182752699	0.00997	0.105786326	0.13413	-0.14141579	0.04583
BDH2	-0.369056447	0.00999	-0.013131781	0.92605	-0.405036803	0.00469
COBLL1	-0.493604846	0.00998	-0.463106565	0.01544	-0.223289294	0.2427
FRS3	0.63502935	0.00999	-0.150852957	0.55535	0.3506104	0.15725
IFT74	-0.357226563	0.00999	0.050778332	0.70859	-0.285490851	0.03818
LPL	1.891399519	0.00998	1.623075827	0.02778	1.007019624	0.1768
CD96	-1.377791089	0.01001	-1.147923679	0.03014	-0.548429398	0.29686
11-1396O1	-1.808482418	0.01003	-1.913477033	0.0061	-0.596580535	0.35783
MRPL42	-0.333407257	0.01006	0.303800825	0.01855	-0.137297076	0.28841
CACNG8	0.479860744	0.01008	-0.365422925	0.05966	0.752116456	4.29E-05
C16orf62	-0.27166152	0.01012	-0.454478345	1.55E-05	-0.314360692	0.00286
DMC1	-1.002835087	0.01012	0.13039368	0.72335	-0.486462549	0.19704
LY6H	6.336879712	0.01013	5.204225052	0.03511	5.011465928	0.04273
1-Mar	-0.876436309	0.01013	-0.13360025	0.69401	-0.427455821	0.20851
MGAT3	0.361772591	0.01012	0.028668913	0.83882	0.391747713	0.00532
NTAN1	-0.42400354	0.01013	0.047338266	0.77252	0.01963984	0.90468
TMEM18	-0.347536039	0.01015	-0.07599768	0.56752	-0.044577677	0.73858

ALS2CL	0.643580923	0.01015	-0.15866641	0.5279	0.602533497	0.01611
NPM1	-0.288930147	0.01017	0.341009368	0.0024	-0.153575061	0.17186
LGR4	-0.309356172	0.01018	0.053618587	0.65554	0.163162821	0.17477
GPR137	0.55208374	0.01018	-0.070437433	0.7456	0.328694601	0.12622
ST3GAL3	0.574864183	0.01021	0.236089842	0.29208	0.075729019	0.73913
AC243772.2	0.490199879	0.01024	0.097755025	0.61226	0.324133861	0.09137
PDIA6	-0.25497213	0.01024	0.129074078	0.19303	-0.071242783	0.47283
SCAMP2	0.408806548	0.01025	0.005486798	0.97256	0.404984409	0.01092
PPP2R5A	-0.275393653	0.01026	0.030508953	0.77409	-0.036350852	0.73333
FBXO10	0.664635961	0.01028	0.152861179	0.56018	-0.156203528	0.55333
PCBD1	-0.403241756	0.01029	0.085758431	0.58215	0.148944222	0.33961
CHRNA5	-0.724927597	0.01029	-0.229820936	0.41188	-1.180153481	3.66E-05
CYP7B1	1.318879042	0.01031	1.052213406	0.0414	0.493926721	0.34521
DENR	-0.180829663	0.01032	0.08302922	0.23286	-0.146864764	0.0365
CPA3	-1.715883092	0.01036	0.280491721	0.65654	0.284297122	0.65472
DMTF1	0.317878865	0.01036	0.147026984	0.23505	-0.026043518	0.83378
NAA16	-0.409353607	0.01038	-0.451802132	0.00443	-0.319780178	0.0441
RSRC1	-0.285432016	0.01039	0.067181431	0.5408	-0.044860129	0.68487
ACTR10	0.293691977	0.0104	0.418627772	0.00023	0.194546335	0.08911
GSPT2	-0.304183007	0.01043	-0.060737819	0.60361	-0.17007182	0.14933
INTS7	-0.4735086	0.01043	-0.101417428	0.5818	-0.205731423	0.26405
KLF9	0.882510836	0.01043	0.244973936	0.48013	-0.052102087	0.88169
UNGP1	1.252991582	0.01042	0.293166639	0.57396	0.511989796	0.32204
ARHGAP11A	-0.454018025	0.01044	-0.004757029	0.97849	-0.073122906	0.67923
MRPL20	-0.249037335	0.01045	0.129443426	0.17439	-0.007928076	0.93433
KDM5A	0.198705028	0.01045	0.08841437	0.25253	0.084193515	0.27707
MAP4	0.414183807	0.01046	0.258611859	0.11001	0.24606394	0.12839
EBAG9	-0.374080712	0.0105	0.233018746	0.10104	-0.218291188	0.13135
TMEM136	0.46986172	0.01055	-0.071238148	0.70349	0.122985178	0.5092
DPH3	0.357017022	0.01058	0.38136201	0.00599	0.17743586	0.20468
TBC1D30	-0.538816553	0.01059	-0.491059567	0.01816	-0.390822695	0.06127
RNF128	-0.511182456	0.01061	-0.363066983	0.06935	-0.097607363	0.62527
ELMSAN1	0.381597017	0.01064	-0.385709168	0.01025	0.220454906	0.14021
GBP5	0.7826053	0.01064	0.829753739	0.00648	0.664743551	0.02991
PABPN1	0.359833247	0.01064	0.191833561	0.1736	0.302230936	0.03187
XRCC2	-0.428923599	0.01065	-0.323922391	0.05233	-0.428746219	0.01052
LACE1	-0.576290308	0.01066	0.018465308	0.93346	-0.083678175	0.70669
MMP19	0.818977229	0.01067	0.613525635	0.05564	0.296370076	0.36388
HIST1H1D	-0.586784848	0.01068	-0.063761269	0.78097	-0.30394534	0.1855
GLO1	-0.197528484	0.0107	0.243233744	0.00153	-0.092293295	0.23192
PDX1	0.924776165	0.0107	0.32189828	0.37881	1.094452064	0.00242
LDLRAD3	0.64506753	0.01072	0.684829366	0.00682	0.223829953	0.38304
NCCRP1	1.072289194	0.01072	0.09486504	0.82818	0.23261839	0.59456
CUEDC2	-0.350076693	0.01073	-0.207129118	0.1245	-0.395450422	0.00384
CCDC8	-0.352997357	0.01075	-0.365326747	0.00814	-0.294116387	0.03329
TERF1	-0.326582159	0.01074	0.013277671	0.91685	-0.249239965	0.05117
NTPCR	-0.272773504	0.01076	0.08524319	0.4194	-0.280875956	0.00838

USP35	0.596041642	0.01077	-0.084810627	0.72158	0.393615667	0.09484
ECI2	-0.341029409	0.01078	0.059639407	0.65185	-0.021524674	0.87118
C12orf75	-0.477581663	0.0108	-0.252151735	0.17695	0.043372229	0.8162
POLI	-0.393025576	0.0108	-0.337476441	0.02807	-0.138271389	0.36856
SLC22A3	1.15149182	0.0108	0.874950069	0.05304	0.087179138	0.84983
SPRED3	0.669136095	0.01084	-0.229634563	0.39292	-0.757057641	0.00585
NR2C2	0.410252374	0.01085	-0.452585856	0.00655	0.198885863	0.2167
KIAA0430	0.568440466	0.01086	0.17693465	0.42778	0.291299857	0.19205
MED24	0.331030529	0.01086	-0.16418832	0.2076	0.504629755	9.73E-05
NSDHL	0.32563919	0.01089	0.425167459	0.00083	0.431192029	0.00072
ALKBH8	-0.353468301	0.01092	0.102605916	0.4418	-0.171413124	0.20913
CCRN4L	0.610596043	0.01093	0.164983104	0.49515	0.086897087	0.72068
C5orf34	-0.654793987	0.01093	-0.492524779	0.05035	-0.410683141	0.10463
COPRS	-0.359522421	0.01094	0.178241227	0.18983	-0.154824742	0.26784
RAD51	-0.576978717	0.01094	-0.485559573	0.02976	-0.43931818	0.05059
FGF7	0.674942966	0.01098	0.764694262	0.00393	0.069814013	0.79311
LRCH3	0.334610947	0.01099	-0.03868197	0.76886	0.058617065	0.65625
TTC22	0.892014323	0.01101	-0.006833731	0.98459	0.405136983	0.25052
SP6	1.08549532	0.01104	0.815099911	0.05676	0.515163684	0.23414
AAR2	0.277539477	0.01106	0.124963794	0.25185	0.093070376	0.39774
PRMT1	0.280954018	0.01106	0.412433591	0.00019	0.186687884	0.09159
JADE1	-0.964024891	0.01108	-1.355230786	0.00036	-0.634820281	0.09363
HDDC2	-0.285644805	0.01109	0.124951357	0.25714	-0.110599493	0.32187
A2M	0.633039284	0.01112	0.425767541	0.08768	0.123935075	0.61921
DAPK1	-0.48851919	0.01113	-0.771843251	6.06E-05	-0.239062326	0.21384
CEP290	-0.321322731	0.01113	-0.035608232	0.77697	-0.076948554	0.54171
C4BPA	1.629929854	0.01118	1.15379167	0.07249	0.061331575	0.92634
?P4-765C7.	-0.533296239	0.01118	-0.513484147	0.01429	-0.417438941	0.04654
NPM1P40	-0.636762675	0.0112	-0.418365744	0.0815	-0.750517183	0.00303
YPEL4	0.643927541	0.01121	-0.521779831	0.04448	0.262107186	0.30502
UBE2L6	0.567463112	0.01121	0.752861007	0.00071	0.406901589	0.06971
GGA1	0.273924749	0.01123	-0.330722686	0.00247	0.128257467	0.23651
TSC22D4	0.412179044	0.01128	-0.052937369	0.74717	0.254267893	0.11991
MMP9	2.316200837	0.0113	1.292879336	0.1596	-0.056249905	0.95226
ARL6IP6	-0.358893831	0.01132	-0.232423822	0.09637	-0.245736658	0.08045
RPLP0	-0.284730463	0.01132	0.087615328	0.43561	0.014495209	0.89738
EP300	0.36836984	0.01134	-0.248507419	0.08855	0.239168403	0.10036
C11orf54	-0.316994631	0.01138	-0.140812567	0.25726	-0.129132287	0.29995
CLSPN	-0.527148951	0.01137	-0.346694736	0.09449	-0.210570225	0.30988
HOXB2	0.717532094	0.01138	0.189906402	0.50636	0.083649582	0.77131
MON1B	0.243469192	0.0114	-0.10147557	0.29171	0.21672743	0.02426
BRINP2	1.782366318	0.01141	-0.487778487	0.51695	-0.95837561	0.21844
EARS2	-0.284282656	0.01142	0.084701708	0.44469	-0.193664184	0.08349
AC004166.(	0.903463963	0.01145	-0.388980758	0.31949	0.25373903	0.49035
CMTM3	0.584956897	0.01144	0.142570914	0.53923	-0.217280776	0.35332
FAM210B	-0.312144234	0.01144	0.134839154	0.26854	-0.296189548	0.01617
GFPT2	1.05803239	0.01144	0.592359466	0.15786	0.154541338	0.7164

AP1S1	-0.340063427	0.01145	0.084263193	0.52701	-0.103039887	0.44143
ECI1	-0.429680999	0.01146	-0.348508518	0.03705	-0.232710042	0.16437
FZD3	-0.430659289	0.01149	-0.010605818	0.95023	-0.377323855	0.0266
UGGT2	-0.265070081	0.01152	-0.073227013	0.48279	-0.329772552	0.00165
C4orf46	-0.380758719	0.01154	0.146525555	0.32514	-0.253323842	0.09203
C19orf70	-0.323229766	0.01155	-0.280025115	0.02596	-0.034511954	0.78255
MRPL43	-0.216543405	0.01156	0.088570576	0.28647	-0.129013937	0.12867
ZNF561	-0.290461755	0.01156	-0.177606675	0.11868	-0.221574844	0.05345
SGTA	0.366100556	0.01157	0.221508743	0.1267	0.272783823	0.06048
GCHFR	-0.405314558	0.01158	-0.046702961	0.76609	-0.113858614	0.47191
NUBPL	-0.434209923	0.01158	-0.247695622	0.14242	-0.245399309	0.14863
PTPRH	0.570376658	0.01158	0.019333635	0.932	0.683703446	0.00246
YPEL2	0.461180465	0.01159	0.094534581	0.60581	0.340291022	0.06273
CENPV	-0.280910228	0.01161	-0.086486627	0.43047	0.072700556	0.50792
CACYBP	-0.368359144	0.01162	0.419353653	0.00371	-0.076683837	0.59762
DLD	-0.204858443	0.01162	0.224212386	0.00512	0.057453321	0.47604
PPM1H	-0.522926182	0.01165	-0.270056861	0.19081	0.019520034	0.92465
DUSP10	0.457185259	0.01171	0.199672372	0.27295	0.00584939	0.97472
CC1L-ADOI	0.797460362	0.01171	-0.058877385	0.85424	0.061639995	0.84756
RARS	-0.191725086	0.01175	0.186214833	0.0132	-0.10731328	0.1568
FAM199X	-0.271933824	0.01176	0.027592114	0.79685	0.058771097	0.5845
GLUD2	-0.706711113	0.01177	-0.36474836	0.18618	-0.219329706	0.4266
LIMS2	1.414624603	0.01177	0.25883984	0.65148	0.394023315	0.49086
NCKAP1L	1.426999441	0.01179	0.434144209	0.45175	0.250758939	0.66245
BLMH	-0.389278179	0.0118	-0.198835069	0.19419	-0.393582575	0.01083
P11-1143G	-1.27550852	0.0118	-0.85250832	0.06961	-0.838163826	0.07833
SPATA20	-0.249800611	0.01181	-0.146907752	0.13711	-0.127018406	0.19886
ARL5B-AS	-1.03260651	0.01184	-0.931258445	0.01756	-0.19381364	0.5952
PLEKHG2	0.455170365	0.01184	-0.399279204	0.02816	0.076673856	0.67279
SUPT16H	-0.209066683	0.01183	0.169490648	0.04019	-0.131992159	0.11156
BAZ2A	0.415619276	0.01187	-0.043179504	0.79409	0.499497791	0.0025
B3GAT1	0.894516379	0.01187	-0.264327475	0.4699	0.303767159	0.40189
PHF1	0.451693019	0.01187	-0.241989325	0.18131	0.02396177	0.89432
TRNAU1AF	0.269281102	0.01188	0.182220949	0.08671	0.16039796	0.13532
SLC45A4	-0.395530378	0.01188	-0.408290217	0.0096	-0.175676694	0.26314
MIB2	0.437342351	0.0119	-0.348301983	0.04785	0.211617153	0.2251
RPAP3	-0.305393511	0.01191	0.308787587	0.00992	-0.220626574	0.06811
NEDD1	-0.265618353	0.01192	0.146556391	0.15873	-0.154506458	0.14225
RPL11	-0.320679979	0.01193	0.309317308	0.01522	-0.047728892	0.70815
RPL28	0.370082101	0.01193	0.311842684	0.03409	0.39394412	0.00744
GPR133	0.74482939	0.01194	-0.092323184	0.75645	0.074675498	0.80199
CAMTA2	0.557385275	0.01194	-0.090088477	0.68848	0.260799459	0.23981
DCAF7	0.493973244	0.01195	0.412103088	0.03482	0.180572401	0.36319
IDH3A	0.292944917	0.01196	0.350415626	0.00238	0.292833095	0.01175
HYPK	-1.263695128	0.01198	-0.034773008	0.94439	-0.390452041	0.43554
FABP5P7	-0.566106676	0.01201	0.103980716	0.64207	-0.035861314	0.87289
FAM127A	0.387738519	0.01201	0.088390569	0.5677	0.122029088	0.43091

PPP1R10	0.471305711	0.01201	0.351511276	0.0611	0.426733245	0.02298
CYCS	-0.252646926	0.01201	0.253213185	0.0114	0.121154788	0.22689
FAM129B	0.427134885	0.01203	0.007046338	0.96697	0.167443986	0.32508
GABRE	1.001293796	0.01202	0.343490806	0.39116	0.514408326	0.19916
FBXW9	-0.447892503	0.01204	-0.46077998	0.00856	-0.365705468	0.03794
SEMA6D	0.858979543	0.01204	0.119655759	0.72716	-0.132748417	0.69906
SLC35D2	-0.33934287	0.01204	-0.174118519	0.19539	0.097179926	0.46948
MYL9	0.563233839	0.01206	0.321207292	0.15236	-0.132381976	0.55611
BDNF	0.799131401	0.01209	0.631997674	0.04721	-0.088702986	0.78369
CAPN9	-0.98906881	0.01212	-2.099110353	1.83E-07	0.156121865	0.68958
GNB2	0.285953698	0.01213	-0.056639079	0.61998	0.066376561	0.5613
IER3	0.837904351	0.01213	0.840502693	0.01183	0.427997585	0.20044
LSS	0.481758725	0.01212	-0.151134514	0.43187	0.639057164	0.00087
SH3BP5L	0.244828526	0.01211	-0.125651524	0.20087	0.157653713	0.10754
C9orf40	-0.454050092	0.01214	-0.145311974	0.4036	-0.878708157	2.98E-06
EPHA2	0.49796406	0.01213	0.239325279	0.22829	0.050118015	0.80116
PEG10	0.548349729	0.01215	0.53467059	0.01477	0.087600494	0.68957
MLEC	-0.275778783	0.01217	0.076392156	0.48654	-0.259255279	0.01835
TPM4	0.276414374	0.01218	0.327068507	0.003	0.038228336	0.72892
CARS2	-0.233163535	0.01219	-0.027125094	0.7668	-0.123150858	0.18352
GFM2	-0.364011784	0.0122	0.041898035	0.77078	-0.113547508	0.43145
TRAK2	-0.244525212	0.0122	-0.006736712	0.94419	-0.141902539	0.14387
MEX3A	0.39538977	0.01224	0.131166668	0.40615	-0.148760756	0.34901
MST4	-0.342875496	0.01224	0.18094516	0.18307	-0.012265737	0.92829
MTCH1	0.225068029	0.01224	0.20490965	0.02229	0.206661848	0.02136
GET4	-0.436624301	0.01225	-0.113553867	0.51213	-0.328953454	0.05944
FBXO17	-0.35021752	0.01232	-0.384384634	0.00561	-0.435879848	0.00194
ORMDL1	-0.214373836	0.01238	0.073352349	0.38227	-0.171590627	0.04408
BRSK1	0.691274374	0.01242	0.169891915	0.54287	0.171314186	0.54358
MKL1	0.609062003	0.01242	0.046442195	0.84973	0.425842118	0.0816
HIC1	1.125965445	0.01244	-0.051374842	0.91051	0.335851496	0.46124
KRIT1	-0.432777428	0.01244	0.042238125	0.80613	-0.039668474	0.81823
MAP3K3	0.439572473	0.01244	-0.056950811	0.74703	0.026032441	0.88298
RYR2	1.105975618	0.01245	1.780122159	6.59E-05	0.12889199	0.77297
FXYD3	-0.431562305	0.01246	-0.600155573	0.00051	-0.002042781	0.99055
CDC42BPA	-0.403702787	0.01248	-0.221515334	0.16996	0.20157925	0.21145
FAM50A	0.279275936	0.01249	0.285783285	0.00998	0.168280835	0.13325
P11-195F19	-0.859053512	0.01249	-0.337883689	0.32141	-0.042838403	0.89745
SERPINB1	-0.425458905	0.01249	-0.085836204	0.61366	-0.355975798	0.03647
WDR37	0.328490739	0.01248	-0.056355821	0.66942	-0.054334328	0.6805
BMS1P10	-0.622497712	0.01257	-0.505344301	0.039	-0.218733283	0.36772
LGALS9	0.735851725	0.01258	0.110593035	0.70983	0.953869557	0.00121
PRDX4	-0.349109939	0.01256	0.181428232	0.19344	-0.145409616	0.29802
RARB	0.378248004	0.01257	0.152393875	0.31414	0.049374138	0.74568
P11-583F2	0.736399086	0.01258	0.101659826	0.73762	0.421259255	0.1612
RPL3	-0.30965944	0.01257	0.099509163	0.42233	-0.055727633	0.65324
TTC37	-0.231164975	0.01259	0.050321046	0.58453	-0.16425476	0.07541

HAND1	0.991999488	0.01263	-0.15101457	0.70852	-0.319480492	0.43245
HCN3	0.735495998	0.01264	-0.023148483	0.93855	1.260795485	1.66E-05
GJB1	-0.577983093	0.01265	-0.352992172	0.12646	-0.353194123	0.12667
ID3	0.536671661	0.01266	-0.076831183	0.72223	0.367133223	0.08846
SVEP1	0.48133066	0.01269	-0.02632134	0.89168	0.001554492	0.99358
SLC31A2	0.683469724	0.0127	0.650276862	0.01706	0.525575946	0.05885
LY6G5B	0.559186116	0.01272	-0.344210007	0.13573	0.19105889	0.39781
ADH1C	-1.287247895	0.01273	0.2486104	0.62837	0.195722887	0.70327
FRG1	-0.26042965	0.01273	0.230268552	0.01777	-0.102778559	0.31715
SRPRB	-0.358006541	0.01273	0.228070418	0.10927	-0.021752902	0.87914
LRPAP1	-0.328831151	0.01274	-0.103379302	0.43233	-0.162898532	0.21663
MIS12	-0.356689138	0.01274	-0.150529073	0.28418	0.010918119	0.93847
HDX	0.562767915	0.01275	0.552064279	0.01415	0.030135744	0.8948
SAR1A	0.239823011	0.01277	0.244847477	0.01069	0.229808149	0.01695
MYLIP	-0.36746418	0.01279	-0.36851051	0.01173	0.061235402	0.67272
DAPP1	1.410878823	0.0128	1.24859626	0.0277	1.095220042	0.05428
KNOP1	-0.356042787	0.01282	0.023803657	0.86699	-0.268892189	0.05925
ALAS1	0.405086808	0.01284	0.36863062	0.02338	0.293279218	0.07159
MRPL48	-0.329832179	0.01285	0.225649745	0.08022	-0.001416412	0.99131
NLGN1	-0.707832006	0.01285	-0.130971244	0.64116	0.178253199	0.52557
ROCK1	-0.28554987	0.01285	-0.074791355	0.51344	-0.210967132	0.06576
XPA	-0.422490408	0.01286	0.113635254	0.49098	-0.008042311	0.96145
SRBD1	-0.282508536	0.01288	0.231807127	0.03734	0.001694325	0.98799
CD4	0.834705922	0.01289	0.192553053	0.5749	-0.119306566	0.74064
PSMC1P1	0.276918613	0.01289	0.688522564	4.20E-10	0.269212605	0.01554
ELOF1	0.289615898	0.0129	0.176084719	0.12893	0.091366913	0.43455
TMEM8A	0.319288269	0.0129	0.051978125	0.68627	0.293400829	0.02237
C5orf30	-0.3650505	0.01292	-0.042115014	0.77223	0.049855455	0.73174
DENND4B	0.406028642	0.01295	-0.287675045	0.0813	0.390738936	0.01729
FAM155A	0.693669145	0.01295	0.547023625	0.0497	0.108267606	0.70414
FUCA1	-0.273029726	0.01294	-0.082585113	0.44773	-0.101102424	0.35452
MAFF	0.671420227	0.01295	0.38742545	0.15199	0.529545589	0.05016
RP6-24A23.	2.220092787	0.01293	1.944074723	0.02984	1.780174295	0.04853
SORBS3	0.477834111	0.01294	-0.226063381	0.24505	-0.030305582	0.87573
ZNF131	-0.29741119	0.01292	-0.085978721	0.46746	-0.266477763	0.02551
PCMTD2	-0.331967144	0.01297	-0.323138114	0.0146	-0.205453736	0.12166
SLC22A23	0.515097904	0.01298	0.272834199	0.18789	0.641207699	0.00195
RPL7	-0.282909244	0.01299	0.264504915	0.02014	-0.054189473	0.63415
ZCCHC4	-0.382198923	0.01301	-0.145183462	0.33337	-0.101115298	0.50327
DAB1	1.282020373	0.01302	0.851865105	0.10062	0.944242139	0.0677
GPRC5A	0.634283232	0.01307	-0.104105319	0.68389	0.043326154	0.86545
SCAMP4	0.358062943	0.01308	-0.342177536	0.02065	0.15706968	0.27863
CAP1	0.237557208	0.0131	0.602665654	2.90E-10	0.206621746	0.03094
CRCP	-0.249749459	0.0131	0.013066399	0.89549	0.015641076	0.87558
EID1	-0.332363698	0.01309	0.198485262	0.13751	-0.344902186	0.01004
FIG4	-0.481019303	0.0131	-0.119326576	0.5338	0.003544107	0.98531
CHST9	-0.743612849	0.01313	-0.006479063	0.98227	-0.396416655	0.18076

FAM84A	0.740574259	0.01313	0.599712641	0.04449	0.581076149	0.05188
NOSTRIN	-0.655597814	0.01313	-0.040957228	0.875	-0.115372216	0.65953
POLQ	-0.520841947	0.01313	-0.183334092	0.37919	-0.381711908	0.06745
DUSP8P5	0.841761117	0.01314	-0.21897149	0.55463	0.721207236	0.03468
GPR37	0.956101182	0.01315	0.872077634	0.02333	-0.189100583	0.63822
NMNAT3	-0.956083602	0.01319	-0.723525063	0.04715	-0.435922677	0.23761
MRPL54	-0.303197354	0.0132	-0.215449936	0.07103	-0.282788918	0.01996
C6orf203	0.402764789	0.01322	0.690891236	1.56E-05	0.610056639	0.00015
FAM177A1	0.279851951	0.01324	0.234768491	0.03687	0.38893069	0.00055
LRP6	0.316793478	0.01324	0.134989439	0.28919	0.126085341	0.324
TBX19	0.920212852	0.01323	0.196989233	0.6048	0.613193708	0.10243
ZNF30	-0.717323697	0.01323	-0.438706028	0.12013	-0.750624463	0.00874
KCTD9	0.247575794	0.01325	0.312998627	0.00155	0.231996838	0.01989
SF3B1	-0.186388329	0.01325	0.083513779	0.26613	-0.181271453	0.01595
ADAM2	-0.84485309	0.01327	-0.038174355	0.90649	0.005402258	0.9868
BATF2	1.179556967	0.0133	0.341737809	0.48499	0.23549172	0.63559
CC2D1A	0.288885716	0.01331	-0.197031264	0.09593	0.204498151	0.0792
SRRM2	0.498007617	0.01332	-0.230731942	0.25203	0.546289898	0.00663
SNX4	-0.249997586	0.01335	0.149758912	0.13328	0.021643586	0.82901
DERL2	-0.269712288	0.0134	0.14276716	0.18632	-0.2140871	0.05021
HSPA1L	1.739161771	0.0134	0.418387281	0.55418	3.162922417	6.28E-06
MN1	1.215777873	0.01342	0.455476051	0.35839	0.125616482	0.79959
CLPTM1L	-0.294619275	0.01343	-0.039064192	0.74076	-0.084835763	0.4737
RPLP2	-0.353726651	0.01346	-0.066252321	0.64295	-0.082827166	0.56238
P11-255H23	-0.51574608	0.01349	-0.08724647	0.67346	-0.188249478	0.36451
VPS28	0.232606974	0.01351	-0.073538656	0.43828	0.210172154	0.02558
CYB5R3	0.37673188	0.01352	0.021119621	0.89011	0.158412503	0.29994
ZMYM5	-0.291947369	0.01354	-0.375418017	0.00139	-0.298353415	0.01177
ARHGAP12	-0.250559046	0.01355	-0.070140424	0.48688	0.054960931	0.58609
PAM16	-0.601213034	0.01357	-0.030475957	0.89662	-0.062687544	0.7917
EYA3	0.27807695	0.01359	0.170392692	0.12988	0.270994627	0.01614
RPL13	-0.295831188	0.01359	-0.003097399	0.97936	-0.097582731	0.41532
TMEM255E	1.268793216	0.0136	0.648993257	0.2006	0.35542353	0.49455
ERGIC2	-0.294016197	0.01362	0.345293541	0.00341	-0.178582547	0.13443
LPAL2	1.166428176	0.01363	0.356350257	0.45931	0.856019887	0.07241
SNTB1	-0.446300695	0.01365	-0.18496521	0.3048	-0.24197378	0.17999
UST	0.762048762	0.01367	0.770725748	0.0128	0.563043922	0.06882
TRAF4	0.288957609	0.01372	0.019956911	0.86496	0.030138425	0.79783
ZNF136	-0.459644067	0.01373	-0.522889166	0.00474	-0.378833618	0.04182
CYP4F11	1.204244911	0.01375	0.604821083	0.22639	1.014180272	0.03946
WEE1	-0.263114704	0.01382	-0.159557935	0.13428	-0.243387386	0.0229
NDUFB11	-0.29734745	0.01382	-0.047287344	0.69093	-0.138994169	0.24597
RPS2P5	-0.662459808	0.01394	0.282847459	0.27901	0.055389444	0.83338
WLS	-0.317883694	0.01394	-0.022292172	0.86198	-0.213323001	0.09798
COL7A1	0.740428652	0.01396	0.285385579	0.34464	0.190413097	0.52947
BTNL8	1.033475156	0.01401	0.494042544	0.24429	0.919744307	0.02888
GPATCH2	-0.252271794	0.01401	-0.083668373	0.40783	-0.290726832	0.00445

FAR1	-0.326212437	0.01402	0.248867602	0.05995	0.001101661	0.99337
NARS2	-0.444787129	0.01402	0.244524708	0.16821	-0.442985411	0.01365
SNX14	-0.317943084	0.01403	0.324666643	0.01165	-0.079595394	0.53733
LURAP1L	-0.467732638	0.01408	-0.257561921	0.17058	-0.441266156	0.02016
CPSF3	-0.28165788	0.01408	0.236926305	0.03629	-0.31222508	0.00635
PXDN	0.387715692	0.0141	-0.190640371	0.22789	-0.305442013	0.05373
ZBTB6	-0.247725645	0.01411	0.057801803	0.55558	-0.246183375	0.0144
TM9SF4	0.215506859	0.01411	0.143597141	0.10178	0.0662647	0.45078
PPP4R1L	0.579612229	0.01412	0.099224923	0.67688	0.232523882	0.32786
INHBA	0.980986879	0.0142	0.964401959	0.01585	0.372285821	0.35319
ZFAND5	0.200307309	0.0142	0.162791177	0.0458	0.027915568	0.73298
AVEN	-0.641096067	0.01421	-0.256061534	0.32184	-0.480208742	0.06536
GRIA2	1.883998514	0.01421	1.051740067	0.17022	-0.613414387	0.44954
DYRK3	0.73252346	0.01423	0.706209257	0.01769	0.483917918	0.11235
MUC17	1.87709491	0.01425	1.218795317	0.11163	0.644964973	0.40092
ALG1	-0.328745533	0.01427	-0.030394225	0.81763	-0.085969743	0.51739
TESC	-0.963693629	0.01429	-0.167105446	0.66962	-0.658028268	0.09386
PPME1	0.310698637	0.01431	0.07524997	0.55306	0.414131675	0.00108
TMEM171	-1.312786933	0.01431	-0.470483458	0.35678	-0.529462379	0.30313
PSTPIP2	0.754965173	0.01434	1.244816712	4.65E-05	0.674796275	0.02888
CHMP5	-0.293605245	0.01436	0.126265641	0.28632	-0.163583074	0.17079
UTP18	-0.434123329	0.01437	0.160855453	0.35935	-0.4550125	0.01029
UBE2D3	0.235893071	0.01438	0.507190977	1.34E-07	0.205431071	0.03296
WBSCR22	-0.36265136	0.01438	0.084758241	0.56336	-0.105736564	0.47319
GALNT5	-0.474253615	0.0144	-0.292531448	0.12995	0.059729739	0.75682
USP18	-0.678600286	0.01442	-0.238603391	0.36683	-0.488382748	0.0724
AMOTL1	0.371737064	0.01443	0.002114033	0.9889	-0.142609243	0.34874
GAREM	0.385490479	0.01444	0.173694981	0.27056	0.276225673	0.0798
NDST1	0.391003345	0.01444	-0.332846168	0.03768	-0.10730218	0.50297
TBCE	-0.262042138	0.01445	0.054241498	0.60194	0.023640698	0.82208
RPS13	-0.270449979	0.01449	0.192824787	0.08071	-0.052368118	0.63561
MXRA7	0.293075876	0.0145	0.366708356	0.00216	-0.079435328	0.50983
ERGIC1	-0.276896182	0.01451	-0.384004432	0.00069	-0.200226252	0.07673
TMED10	-0.268505954	0.01451	-0.051914851	0.63614	-0.131420366	0.23125
C11orf49	-0.332104258	0.01455	-0.426404254	0.00161	0.0307792	0.81927
LTB4R2	0.687887924	0.01455	-0.242427639	0.41064	0.875257462	0.00167
SV2A	0.555889369	0.01456	0.225141417	0.32375	-0.087038444	0.70699
KLHL14	1.259682668	0.01457	0.745187074	0.14895	-0.476845997	0.36172
FBXO48	0.519966303	0.01459	0.136949402	0.52596	0.565023967	0.00758
FTSJ2	-0.317915606	0.01462	0.162678932	0.20081	-0.160161262	0.21576
RILPL1	0.438209228	0.01464	0.471429557	0.0085	0.1912769	0.28984
CD99P1	0.495877583	0.01465	0.491238302	0.01435	0.434146201	0.0326
PARD3B	-0.850812126	0.01465	-0.817202955	0.01957	-0.52762001	0.12579
CMC2	-0.310927428	0.01467	0.069366593	0.58177	-0.211301793	0.09605
RPL3P2	-0.427636973	0.01467	0.034030503	0.84354	-0.30658649	0.07872
NREP	0.499487257	0.01468	0.161713686	0.4296	0.023838006	0.90736
P11-15H20	-0.476942864	0.01469	-0.08268756	0.67167	-0.452875576	0.0207

PLEKHF2	-0.334237809	0.0147	-0.124922637	0.35559	-0.062110077	0.6465
TRMT112	-0.234298242	0.0147	0.248882998	0.00823	-0.110103842	0.24777
RAP1GDS1	-0.266286947	0.01472	0.162173185	0.131	0.09742503	0.36763
TTK	-0.459881268	0.01474	0.306797208	0.10092	-0.334376208	0.07537
PLCB3	0.256965589	0.01478	-0.090462607	0.39129	0.064908108	0.538
ZNF484	-0.281250246	0.01478	-0.037996111	0.73655	-0.257356768	0.02519
PTPN9	0.291997762	0.01479	0.151460147	0.2044	0.413959726	0.00056
SUCLA2	-0.329325669	0.01485	0.396451411	0.00299	0.028013697	0.83491
OCLN	-0.517063483	0.01486	-0.059609219	0.77841	0.16664028	0.4315
CD93	1.242300226	0.01487	0.441088534	0.39376	0.621071664	0.22933
BCAS1	-0.575706883	0.01487	-1.057477304	7.96E-06	0.118732853	0.61452
CRBN	-0.251633842	0.01489	0.218944342	0.03072	-0.129982098	0.20612
DOCK9	0.538876346	0.0149	0.325734428	0.14107	-0.074524448	0.73682
TIPIN	-0.539559914	0.01489	0.017344561	0.93622	-0.366568456	0.09516
CETN3	-0.415773326	0.01493	0.35228134	0.03473	-0.132524287	0.43271
CHRM2	0.642787063	0.01496	-0.111787085	0.67251	-0.432834331	0.10328
MOSPD1	-0.334535008	0.01501	0.011168584	0.9335	-0.286357516	0.03812
STAG1	-0.293720793	0.01503	-0.095988418	0.42436	-0.281904614	0.01958
ZNF469	1.276429407	0.01511	0.098547259	0.85287	0.380940589	0.4725
POLR1D	-0.311533473	0.01514	0.170941291	0.17932	-0.095098334	0.45735
GIT1	0.444865905	0.01515	0.166198799	0.36484	0.644636111	0.00042
GAK	0.381744839	0.01516	-0.27461584	0.08136	0.290024084	0.0649
DUSP23	-0.414044074	0.01517	0.043989676	0.78296	0.004291005	0.9789
SLC7A9	1.750883899	0.01518	1.166940351	0.10688	0.73638678	0.31334
GGT1	1.105391342	0.01519	0.675121153	0.1387	-0.020395722	0.96492
KIF11	-0.396262138	0.0152	0.042832567	0.79218	-0.275313021	0.09126
VAT1L	1.436584294	0.01521	0.463227435	0.43753	0.612338086	0.30394
MAP3K1	0.263046133	0.01521	0.57374008	1.03E-07	0.172079473	0.11249
LETMD1	-0.315734499	0.01522	-0.314639656	0.01511	0.00837499	0.94818
ANKRD27	0.404276483	0.01523	0.288112642	0.08135	0.223249349	0.18049
KIAA0101	-0.496125293	0.01523	-0.345818166	0.08882	-0.325951466	0.10964
AJUBA	0.373030775	0.01525	0.162229294	0.29149	0.226933382	0.14015
ASRGL1	0.565204119	0.01527	0.487417852	0.03621	0.702401769	0.00242
FBXO16	-0.751974116	0.01529	-0.308736072	0.31136	-0.736673599	0.01645
FGD5	1.102212213	0.0153	-0.496198556	0.29208	0.372690101	0.42284
ORC6	-0.381404206	0.0153	-0.424223276	0.00689	-0.45304125	0.00401
COL4A5	-0.459558406	0.01532	-0.410623033	0.03014	-0.507035683	0.00745
PPP1R12B	0.330448414	0.01533	0.033572217	0.80573	0.062580822	0.64722
METTL17	-0.228705311	0.01537	-0.076663597	0.41118	0.012524082	0.89363
BAMBI	0.6458795	0.0154	0.266276095	0.31883	-0.067243277	0.80248
GLRX2	-0.427799412	0.01539	0.136091227	0.41117	-0.388934854	0.02577
SMIM12	-0.272400263	0.0154	-0.091795381	0.40922	-0.089660524	0.42072
ZNF83	-0.236376773	0.01539	-0.315173976	0.00123	0.003874845	0.96808
WDR34	-0.398996392	0.01542	-0.397008048	0.0154	-0.203669079	0.21359
NIF3L1	-0.273267283	0.01546	-0.042996588	0.69644	-0.13582656	0.22354
PLRG1	-0.220954282	0.01547	0.130000312	0.14905	-0.157098854	0.08419
MAF	0.659728181	0.0155	0.145694069	0.59363	0.362283628	0.18445

SLC9A2	-0.827902476	0.0155	0.183265683	0.59036	-0.0913214	0.78875
SGK2	-0.427132644	0.01552	-0.411019038	0.01942	-0.126011617	0.47219
RBM3	-0.160142348	0.01553	0.337411496	2.57E-07	0.048046598	0.46559
DNPH1	-0.458442475	0.01555	0.047808942	0.79525	0.036862642	0.84227
ANKRD50	0.355496863	0.01558	0.291413603	0.04723	0.11827241	0.42169
ZDHHC5	0.27981902	0.01564	-0.012075983	0.91685	0.208017443	0.07231
SLC26A2	0.616751943	0.01566	0.915740674	0.00032	0.483667815	0.0584
PPIP5K1	0.366349708	0.01568	-0.182297856	0.22805	0.600530479	6.67E-05
SPTBN1	-0.190698717	0.01567	-0.286543103	0.00028	-0.158541491	0.0445
TMSB4XP6	0.440932645	0.01568	0.881091977	1.32E-06	0.883746529	1.23E-06
KDELR1	0.278324526	0.0157	-0.138633239	0.22917	0.222204661	0.05374
MAP7	-0.433560387	0.0157	-0.2106341	0.23908	-0.041433783	0.81687
SCN5A	1.387416961	0.01571	0.224416676	0.69697	0.129143337	0.82493
ST3GAL2	0.491327659	0.01569	-0.150734883	0.45961	-0.076886621	0.70597
PHC2	0.257593668	0.01574	-0.070408311	0.50964	-0.088197864	0.41019
NDUFS1	-0.181307945	0.01577	0.130157486	0.08146	0.101405755	0.17522
PINLYP	-0.708657092	0.01578	-0.433768544	0.13852	-0.023148778	0.93655
CRTC1	0.489027458	0.01579	-0.764876055	0.0003	-0.015643919	0.93939
SFXN2	-0.533466409	0.01581	0.052677539	0.81073	-0.154585473	0.48307
USP32	0.249639354	0.0158	0.363574172	0.00042	0.070091457	0.49816
SFMBT1	-0.353853737	0.01582	-0.199742992	0.16526	-0.14382045	0.31846
DCUN1D4	-0.297527599	0.01583	0.127444827	0.29798	-0.121554134	0.32306
ZNF584	0.378411218	0.01585	0.196543234	0.21007	0.189592995	0.23127
TMEM237	-0.552900431	0.01591	-0.215716988	0.34382	-0.427892808	0.06188
CLEC2D	0.618034393	0.01594	0.287089366	0.26375	0.10168847	0.6937
INTS3	0.222847853	0.01594	-0.200452407	0.03068	0.180721493	0.0505
RELB	1.516312541	0.01595	-0.389269177	0.54686	1.076059005	0.08833
NPIPB3	0.567689904	0.01596	-0.374759288	0.11635	0.187576421	0.42883
RIPK2	0.500306999	0.01597	0.384576995	0.06379	0.202300853	0.33226
SLC25A38	-0.269037418	0.016	-0.019606491	0.85777	-0.095781751	0.38576
SPINK5	-0.447140282	0.01601	-0.764049285	4.00E-05	0.153262051	0.40357
SUSD1	-0.273959043	0.01601	-0.202172728	0.07341	0.107412554	0.34052
GSAP	-0.407550396	0.01603	-0.278805649	0.09486	0.105610299	0.5255
TSR2	-0.249821602	0.01606	-0.169972145	0.0956	-0.120508534	0.23992
SMPDL3B	-0.750318557	0.01607	0.065381634	0.83018	-0.386125995	0.21078
TMEM229E	-0.616150767	0.01607	-0.596016288	0.01908	-0.190063527	0.45123
FASTKD1	-0.345021417	0.01608	-0.005502622	0.96889	-0.079483882	0.57635
HCFC1	0.455057083	0.01608	-0.348505725	0.06643	-0.02646954	0.88878
ATP5EP2	-3.361576141	0.01611	-0.344089896	0.80039	-3.415923862	0.01448
SDSL	-0.703178656	0.01611	-0.31287217	0.25485	-0.651363783	0.0227
NAT14	-0.381743945	0.01617	-0.422557102	0.00718	-0.454459287	0.00452
PCNX	0.342522741	0.01619	0.199224545	0.16178	-0.014238046	0.92047
TPK1	0.526506417	0.0162	0.262431551	0.22956	0.229240322	0.29592
CREB3L3	0.991086157	0.01625	0.206927546	0.6166	1.31351884	0.00143
EFNB2	0.275779421	0.01625	0.378128574	0.00095	0.113694098	0.32245
IDH3G	0.255399154	0.01625	0.178829049	0.09016	0.237019261	0.02534
ITGB3	0.792748272	0.01624	0.051372389	0.87784	0.021725423	0.94841

SELM	2.462263926	0.0163	1.79382615	0.08028	2.043843174	0.0463
KANSL3	0.275566735	0.01632	-0.338127448	0.0033	0.065170036	0.5706
FBXL12	0.211013951	0.01633	-0.291315213	0.00116	-0.106875305	0.23452
SEC24C	0.225255079	0.01637	-0.016989819	0.85651	0.179235938	0.05609
KLC1	0.317692564	0.0164	-0.311387917	0.01907	-0.040808347	0.75849
RP1-20N2.6	-0.64032738	0.0164	-0.81835597	0.00212	-0.586347293	0.02676
TIMM8A	-0.4248065	0.0164	0.046983502	0.78014	-0.253149	0.14422
CDK16	0.215710772	0.0165	-0.045350195	0.61436	0.15380293	0.08726
EIF1AD	0.256775663	0.0165	0.123780352	0.24791	0.136263365	0.20612
TMEM9B	-0.247509217	0.01649	0.067106258	0.5075	0.146958737	0.14713
MPP5	-0.197815812	0.01651	0.00691757	0.93166	-0.192383483	0.01878
TIMM21	-0.315054968	0.01653	0.181482558	0.159	0.064720136	0.61778
FGD4	-0.404738084	0.01656	-0.158699733	0.34618	0.024438256	0.88469
MFSD2B	-1.06314509	0.01656	-0.356149516	0.40665	-0.486502808	0.26773
CAB39	0.247232884	0.01657	0.113146772	0.272	0.385248584	0.00018
SERBP1	-0.269376807	0.01662	0.249800302	0.02614	-0.051529864	0.64662
EFCAB2	-0.408630465	0.01663	-0.216887153	0.2015	-0.087405751	0.60636
MELK	-0.510242946	0.01667	-0.094102727	0.65685	-0.458084807	0.03146
MANBAL	-0.249088662	0.01668	0.006037288	0.95197	-0.150637463	0.14215
ABRACL	-0.303406782	0.01668	0.166574061	0.17736	-0.161885056	0.19773
ASAP1	0.463448085	0.01672	-0.038630272	0.84217	0.153865168	0.42878
FAM107B	-0.412019879	0.01671	-0.014398615	0.93314	-0.227986407	0.18485
KIF1C	0.239633498	0.01672	0.181558929	0.06965	0.399963029	6.45E-05
SNX6	-0.164389148	0.01673	0.19406836	0.00422	0.0248569	0.71596
ULBP3	-0.593873067	0.01674	-0.328894459	0.16918	-0.625214922	0.01151
CDH11	0.518017625	0.01676	0.316175699	0.14428	-0.231352249	0.28563
MBD1	0.232208465	0.01676	-0.250207637	0.01024	0.154088499	0.11266
SERPINA3	1.324695367	0.01676	2.223050044	5.96E-05	0.857980816	0.12134
EMP2	0.536783457	0.01679	0.391636408	0.08099	0.228119158	0.3104
RBM43	-0.488493014	0.0168	-0.043121705	0.82832	-0.423434822	0.03723
TMEM198B	0.401258913	0.01683	-0.175364893	0.30371	0.286662599	0.08769
RAP1A	-0.360350993	0.01686	0.113519466	0.44754	-0.088385383	0.55632
BAG2	-0.496752977	0.01688	0.169688692	0.40792	-0.448213746	0.03076
TAB2	0.261131307	0.01688	0.141534557	0.19468	0.259847846	0.01732
ABI3	1.217758773	0.01688	-0.246622521	0.64535	0.143892639	0.79039
FOXC1	1.739331515	0.01691	1.381316887	0.05796	1.248921421	0.08681
AFAP1L1	0.956799845	0.01696	0.845705909	0.03345	0.967863354	0.01612
IP6K1	0.412366305	0.01697	-0.016294587	0.9246	0.253598198	0.14172
TMEM106C	-0.422600196	0.01698	-0.070049458	0.69023	-0.052032239	0.76784
MAP1B	0.803471488	0.017	0.272431459	0.41852	-0.310316128	0.35735
GOLM1	-0.443057083	0.01702	-0.123208763	0.50681	-0.000827564	0.99644
RIC3	-1.987858705	0.01701	-0.943275776	0.25193	-1.442231403	0.081
BRWD1	-0.203430668	0.01705	0.07391682	0.38384	-0.05561815	0.51357
TMEM66	-0.185563195	0.01705	0.044261924	0.56649	-0.016585186	0.83047
RP11-213G2	-0.599921848	0.01705	-0.234306488	0.3313	-0.396517822	0.10751
PIGX	-0.264872739	0.01706	0.128586178	0.23582	-0.100002351	0.36351
PIGB	-0.298939256	0.01707	-0.446727198	0.00027	-0.242147876	0.04954

ABCB8	0.34107741	0.01712	-0.495493823	0.0007	0.00712582	0.96051
BCL2A1	2.155187858	0.01711	1.717966886	0.0592	1.671614772	0.06792
DDR2	0.604565283	0.01711	0.354128965	0.16246	-0.03059773	0.90406
RASGEF1A	1.404692228	0.01712	1.422122785	0.01236	0.734378949	0.2198
ZSWIM7	-0.354507843	0.01713	0.123173099	0.39659	-0.261485212	0.07738
CEP170B	0.406279666	0.01715	-0.338987446	0.04766	0.275239021	0.10643
TMEM117	-0.390671618	0.01715	0.000816682	0.99596	-0.212079068	0.19265
RPL10	-0.248657433	0.01716	-0.000197533	0.99849	-0.109139552	0.29537
P11-231C14	0.859527475	0.01717	0.893162847	0.013	1.098291585	0.00224
TGM1	1.548584274	0.01717	0.473754311	0.46828	0.293875136	0.65405
PITX2	1.012607386	0.01721	0.708335291	0.09588	-0.013516871	0.97479
ATP1A1	0.304492688	0.01726	0.407933482	0.00142	0.291715868	0.02253
HSBP1	-0.187112959	0.01731	0.191459113	0.0139	-0.008316422	0.91542
IFT43	-0.403338623	0.01731	-0.061964092	0.70335	-0.031340553	0.84843
SEMA4C	0.36340476	0.01733	-0.116889819	0.44586	-0.094837001	0.53749
UBE2Q2	-0.311637969	0.01733	0.136868058	0.28927	-0.345685619	0.00823
ABHD6	-0.43873384	0.01735	-0.130143991	0.47147	0.162702289	0.36939
C4orf3	-0.233858123	0.01736	-0.092511207	0.34535	-0.110072369	0.26197
ATP6V1A	-0.336238131	0.01737	0.009322536	0.94725	0.01520545	0.9142
DESI1	0.442458941	0.0174	0.673956579	0.00028	0.392458578	0.03484
DZIP3	-0.240523213	0.01743	0.18116123	0.06865	-0.13711197	0.17292
FH	-0.331625188	0.01744	0.239829955	0.08342	-0.05090276	0.71406
HSD17B8	-0.433723893	0.01745	-0.199072934	0.25893	0.06524115	0.70893
AC091654.1	-1.344087168	0.01747	-0.03760772	0.93876	-0.626371667	0.22691
WDR12	-0.447737674	0.01747	0.433207187	0.02072	-0.057492966	0.7599
ANKFN1	-2.015961632	0.01748	-1.760151661	0.04173	0.065583197	0.93127
BEX5	-0.902220844	0.01748	-0.224856172	0.52326	-0.276484977	0.43875
ABCC1	0.518054929	0.01752	-0.129827526	0.55191	0.269441017	0.2185
ANKRD32	-0.501875689	0.01753	-0.270701349	0.19529	-0.09776383	0.64078
ETV6	0.364392214	0.01759	0.101548999	0.50835	0.293955754	0.0554
TAF10	0.328805275	0.0176	0.461811523	0.00079	0.242308078	0.08054
PITPNM1	0.421235702	0.01761	-0.257717403	0.14913	0.164296822	0.35673
MFAP4	0.504420597	0.01763	-0.029097341	0.89131	0.059364849	0.78033
CYP20A1	-0.526036061	0.01764	0.107709029	0.61771	-0.113479596	0.60248
SAMD4B	0.388180825	0.01765	-0.30142759	0.07095	0.202825793	0.21497
ZMAT2	-0.265173828	0.01765	0.212461655	0.05404	-0.139749388	0.20955
CTDNEP1	0.494614457	0.01766	0.381733983	0.06659	0.383467251	0.06541
CA2	-0.613666001	0.01768	-0.365345181	0.1575	0.099725935	0.69951
SIPA1	0.566288293	0.0177	0.059048679	0.80934	0.460876089	0.0556
TNFRSF13C	0.807607767	0.0177	-0.357761035	0.32985	0.321722359	0.35855
CLIP2	0.412199723	0.01771	-0.321435196	0.06519	0.10036582	0.56411
ANKRD36	-0.370798223	0.01773	-0.406704655	0.00912	-0.576409242	0.00023
CCNA2	-0.412011989	0.01777	0.187745788	0.27511	-0.224699491	0.19445
VIM	0.472089367	0.0178	0.209684115	0.29255	-0.121602267	0.54173
RNF44	0.355278985	0.01781	-0.558285916	0.00027	0.123682219	0.41224
AC004951.5	1.308629242	0.01782	0.350139004	0.53706	0.139406918	0.80904
FOXD2	1.467922667	0.01784	1.312141053	0.03418	0.508197796	0.4161

6-Mar	-0.232856495	0.01786	-0.00165729	0.98651	-0.295567899	0.00262
COLEC11	1.354212583	0.01787	0.186476199	0.74577	-0.304439779	0.59982
ASB2	1.023784772	0.01791	0.544449639	0.21053	-0.065936262	0.88307
TNFRSF14	1.057596906	0.01795	1.09941621	0.01528	1.471448508	0.0008
BRD3	0.361124503	0.01798	-0.099301176	0.51601	0.191269607	0.21089
PIGM	-0.339016188	0.01797	0.037256906	0.79123	-0.232684229	0.10246
SAMD1	0.300953846	0.01797	-0.143319798	0.26271	0.266048599	0.03641
SYT2	0.871230133	0.01797	0.139451563	0.71025	0.258806963	0.49087
PLXNA1	0.355578269	0.01801	-0.258585357	0.08631	0.071787916	0.63384
IFI27L2	-0.397169125	0.01807	-0.057236283	0.72081	0.022436286	0.88895
LAMP1	-0.27652101	0.01807	-0.256795596	0.02785	-0.366822411	0.00172
SEC11A	-0.268290668	0.01807	0.318828569	0.00472	-0.048122026	0.67077
YJEFN3	0.559345712	0.01808	-0.465104883	0.06553	0.248982773	0.30043
EPHA4	0.745335481	0.01809	0.139538563	0.65816	-0.253868438	0.4214
LONP2	-0.196393087	0.01809	-0.100993852	0.22259	0.037677206	0.6495
AP000347.2	0.426418692	0.0181	-0.142939398	0.43098	0.379143528	0.03472
SIRT7	0.387402191	0.01816	-0.160125891	0.33475	0.403416748	0.01363
CROT	-0.541393414	0.01817	-0.543883898	0.01674	-0.102140549	0.65329
CHSY1	0.398491005	0.0182	0.244440226	0.14737	-0.129784623	0.44348
DCLK1	0.999933061	0.01819	0.674399638	0.10997	0.674367467	0.11331
PGS1	0.290148859	0.01821	-0.16202132	0.19244	-0.029138262	0.81445
SIPA1L3	0.56619239	0.01821	-0.23720302	0.32454	0.843689989	0.00043
SLC26A10	1.333582802	0.01821	0.217045545	0.70942	0.377312998	0.5149
STAC3	1.267947066	0.0182	0.433636639	0.42476	0.927647055	0.08077
FOXI3	1.471723371	0.0183	0.170670291	0.79397	0.892728084	0.15993
NAE1	-0.236236647	0.01832	0.088059139	0.37179	-0.083626404	0.40073
FDPS	0.407584719	0.01835	0.190110034	0.27134	0.371541923	0.03156
IRS2	0.596179256	0.01835	-0.037325636	0.88457	0.154511105	0.54696
GCFC2	-0.418362977	0.01838	0.142495306	0.41753	-0.121972855	0.49049
MRPL35	-0.267298815	0.01841	0.104991209	0.3472	-0.07113901	0.5271
FAM72B	-1.056247391	0.01847	0.278266234	0.50861	-0.335698139	0.4376
PRRC1	0.232714145	0.01847	0.031021661	0.75258	0.169685398	0.08526
CXCR4	-0.854778072	0.01848	-0.02445559	0.94612	-0.576255354	0.11186
CA5BP1	0.462560842	0.01853	0.154626748	0.432	0.547702444	0.0052
GCLC	-0.532500186	0.01852	-0.085303766	0.70448	-0.302822879	0.17878
TOPBP1	-0.361857495	0.01852	0.096708225	0.52774	-0.124933825	0.41554
DUSP9	-0.895839017	0.01857	-0.724148014	0.05478	-1.165705986	0.0023
ZUFSP	-0.318380801	0.01858	0.112234072	0.39604	-0.216582193	0.10769
COG5	-0.268596889	0.01859	0.13631889	0.22789	-0.074288247	0.51316
FUT10	-0.341253686	0.0186	-0.057028936	0.69057	-0.125769063	0.38249
LARP6	0.560324709	0.0186	0.509993837	0.03164	-0.060221971	0.80158
ROR2	0.469185204	0.01863	-0.036857923	0.85372	-0.192481792	0.33762
TIMMDC1	-0.256915559	0.01864	0.251487481	0.01809	0.048028059	0.65528
TMEM30A	0.292091221	0.01864	0.577181302	3.25E-06	0.442116827	0.00037
WDR75	-0.20974702	0.01864	0.24609262	0.00485	-0.102381687	0.24746
WDR46	-0.347872801	0.01869	-0.096652334	0.5098	-0.115345248	0.43348
MMP7	1.635223234	0.01873	2.664445084	0.00013	-0.00825483	0.99055

TM7SF3	-0.481463866	0.01876	0.126611232	0.53472	-0.202986259	0.32058
RNF126	0.368906741	0.01877	-0.13324646	0.40028	0.172631269	0.27264
OR7E12P	-1.369217164	0.0188	-0.687141037	0.2047	0.012789994	0.98055
ZC3H3	0.423958353	0.01882	-0.32428832	0.08305	0.233518867	0.20079
ZNF134	0.317215796	0.01881	0.132330164	0.32624	0.301542763	0.02572
ASNSD1	-0.380040701	0.01885	0.418854399	0.00906	-0.143257542	0.37501
NAGA	-0.215246238	0.01886	-0.086175333	0.33925	-0.147205869	0.10543
OXGR1	2.907784207	0.01885	2.152312028	0.0828	1.385167038	0.27
SYMPK	0.337938262	0.01886	0.192910299	0.18114	0.281267337	0.05093
CD101	0.993952841	0.01887	0.227931227	0.59424	0.172848093	0.68617
UQCRB	-0.313440113	0.0189	0.450633598	0.0007	-0.075156981	0.57289
SLC9A4	-0.811699148	0.01898	-0.003208024	0.99258	-0.015460856	0.96427
ASIC3	1.229182134	0.01903	0.074544007	0.8949	0.73771009	0.17512
RPS20P22	1.034880045	0.01904	0.051748464	0.91099	0.048659767	0.91597
TCEA1P2	-0.427398036	0.01909	0.292941563	0.0999	0.064327958	0.72005
BGN	0.761112906	0.01911	-0.066921656	0.83736	0.356065161	0.27391
CCT2	-0.233265541	0.01911	0.205930543	0.03762	-0.161821574	0.10366
PDE6B	-0.653711296	0.0191	-0.010946959	0.96812	-0.264344724	0.33785
PSMC1	-0.425612123	0.0191	-0.066584266	0.71227	-0.310818694	0.08691
ZNF652	0.294518654	0.01914	-0.038566913	0.75916	0.235146588	0.06133
PHGR1	1.596933281	0.01916	1.238744425	0.06953	1.725435877	0.01128
SNRPGP15	1.334530899	0.01915	0.757104392	0.18535	0.497305912	0.38654
SPAG9	0.324337317	0.01916	0.460939588	0.00084	0.159310457	0.24967
AARS2	-0.227519764	0.0192	-0.38527663	6.49E-05	-0.124176567	0.19705
CLCN4	-0.630928615	0.0192	-0.082133199	0.75634	-0.50922343	0.0616
EFCAB11	-0.406940339	0.01921	-0.070527952	0.68039	-0.094588947	0.58025
ZNF596	0.638458946	0.01921	0.419900684	0.12373	0.766006114	0.00505
FHOD1	0.39987823	0.01922	-0.101264911	0.55706	-0.083189104	0.62929
FHL2	0.482567756	0.01924	0.280337497	0.17391	-0.109659579	0.59584
IGF2BP1	0.318594907	0.01924	-0.200580645	0.14087	0.019435853	0.88658
RNF19B	0.367928391	0.01928	0.303180783	0.05288	0.406056745	0.00963
ASGR1	0.910312374	0.01932	0.541153472	0.16738	-0.106957042	0.79024
JPH2	0.881329053	0.01933	0.3576339	0.35014	0.199129022	0.60842
PCTP	-0.325679218	0.01935	-0.004876135	0.97171	-0.01815352	0.89532
SPG7	-0.252922715	0.01937	-0.434525067	5.85E-05	-0.17524054	0.10519
CRHR1-IT1	0.280935578	0.0194	-0.282986282	0.01942	-0.061916909	0.60785
RMND1	-0.331220281	0.01944	0.090806094	0.50995	-0.104437762	0.45453
BLCAP	0.342888759	0.01945	-0.056087391	0.7029	0.324325673	0.02698
GDA	1.03697183	0.01948	0.911887243	0.03988	0.470274094	0.28973
H3F3AP4	1.225456357	0.0195	0.593938045	0.25784	0.82891008	0.11426
PPP1R3C	-0.846338063	0.01952	-1.130967604	0.00181	-0.374674401	0.2999
ARSK	-0.413081105	0.01956	0.097432418	0.57473	-0.276590158	0.11632
KCTD11	0.502311929	0.01956	0.049249109	0.81953	0.299853563	0.16411
FAM127C	0.380924392	0.0196	0.077835864	0.63546	0.017130552	0.91782
MSH3	-0.266555092	0.01959	0.017030543	0.87936	-0.128537594	0.25674
PRDM5	-0.438710058	0.0196	-0.048863834	0.79152	-0.187311192	0.31499
PPIL4	-0.192801359	0.01962	0.110285745	0.17218	-0.044616559	0.58552

SLC35E1	0.292637579	0.01962	0.029035338	0.81697	0.092971669	0.45886
TNC	1.069763326	0.01963	0.801011509	0.0806	0.441584448	0.33552
SLC26A9	-0.630448405	0.01967	-0.883878567	0.00107	0.088931532	0.74174
TTI2	-0.504409209	0.01967	0.158331858	0.46003	-0.474072203	0.02814
SH3GLB1	0.213145773	0.01969	0.233624679	0.01031	0.238166014	0.00909
TP53BP2	0.345083207	0.01972	0.457186942	0.00197	0.278730463	0.05971
C15orf62	0.672487482	0.01975	-0.228936722	0.44826	0.248623984	0.39895
C16orf89	-0.896894728	0.01975	0.133301549	0.72439	0.033475797	0.92837
MTSS1	0.570439621	0.01976	-0.052959867	0.82879	0.531326623	0.0302
RAB11FIP3	0.548105806	0.01976	-0.013614169	0.95406	0.313835906	0.18326
NARFL	0.376273345	0.01977	-0.059095608	0.71505	0.364446409	0.02379
HADHA	-0.185459444	0.01981	0.04787987	0.54543	0.018503574	0.81558
FAM129A	-0.667112763	0.01982	0.009750495	0.97257	-0.318963415	0.26242
SMG1	0.337346614	0.01983	-0.098935299	0.49448	0.24196352	0.09467
GALE	-0.315052814	0.01984	-0.544356655	5.69E-05	0.038164196	0.77649
EGR3	1.24318274	0.01986	-0.008704771	0.98722	0.280300299	0.60533
GPR125	-0.277516955	0.01987	0.111567995	0.34728	-0.202624227	0.08884
MAP3K13	-0.223795718	0.01985	0.148593964	0.11845	-0.090794704	0.34227
NGDN	-0.255106473	0.01986	0.150758727	0.1564	-0.250904648	0.02137
CDKAL1	-0.208361375	0.01991	-0.054539991	0.53642	-0.282968295	0.00159
CLIC5	1.384897897	0.01993	1.079553705	0.06952	1.082614446	0.06893
CDH12	-0.901652794	0.01995	0.15682049	0.68268	0.190109763	0.62033
CROCC	0.4585893	0.01995	-0.639236067	0.0015	0.084535331	0.67044
KIAA0391	0.291397307	0.01995	0.407540355	0.00106	0.159167172	0.20348
SDC1	-0.504392729	0.01996	-0.281903273	0.19224	-0.477925086	0.0274
VWA9	-0.186052279	0.01997	0.17531669	0.0245	-0.076464291	0.33573
STAG3L3	0.293595682	0.01999	-0.266235856	0.03917	-0.008900299	0.94441
SLC5A3	0.315598131	0.02001	0.523567869	0.00011	0.156586653	0.24867
ATXN1L	0.194546342	0.02002	-0.125893706	0.13443	0.103217423	0.21832
FLAD1	-0.341876137	0.02004	-0.024886223	0.86399	-0.196616485	0.17967
TMEM60	-0.375210778	0.02004	0.106929511	0.48737	-0.147755694	0.34951
SAYSD1	-0.300150402	0.02008	-0.216442364	0.08668	0.012926788	0.91803
CD9	0.632162122	0.02008	1.047392635	0.00011	0.382997173	0.15941
CLDN23	-0.620600538	0.02011	-0.447508635	0.0878	-0.34685128	0.18656
SOX21	-0.839555348	0.02015	-0.8866882	0.01396	-0.148821222	0.67819
SELENBP1	-0.340423791	0.02019	-0.063411552	0.66095	-0.164566359	0.2592
ATG4D	0.423403185	0.02023	-0.12075367	0.51394	0.265765951	0.14717
EXD2	-0.235442288	0.02023	-0.127552501	0.19888	-0.19198779	0.05599
SMOC2	0.577680227	0.0203	0.461798247	0.06352	-0.413945541	0.09847
TTC17	-0.225351392	0.0203	-0.215799408	0.02551	0.005100783	0.95797
MAST3	0.6262001	0.02032	-0.352401853	0.19703	0.232201389	0.39093
MANF	-0.392339774	0.02034	-0.247560413	0.14138	-0.018893818	0.91056
UBE2G2	0.212980083	0.02037	0.058625281	0.52089	0.154768176	0.09095
NSUN3	-0.369776835	0.0204	-0.286925007	0.06842	-0.124884766	0.42962
NOMO3	0.825273764	0.02048	0.588808459	0.09849	0.981190669	0.00578
RTF1	0.209353049	0.02049	0.265790667	0.0031	0.09639659	0.28594
NUDT12	-0.281709095	0.02053	0.01936156	0.87126	0.005931454	0.96061

EPAS1	0.456804054	0.02055	-0.188430148	0.3399	0.759822562	0.00012
HIST1H1C	-0.415465375	0.02054	-0.173362754	0.33339	-0.26365323	0.14143
QRICH1	0.168646195	0.02055	0.054524685	0.453	0.028974401	0.69119
C14orf178	1.22187642	0.02057	-0.251892452	0.66709	0.37192618	0.50269
FNIP2	-0.340441003	0.0206	-0.241457347	0.09999	0.063505809	0.66519
RBL2	-0.182365527	0.02062	-0.137570393	0.07789	0.042826592	0.5839
RRAS	0.478821911	0.02064	0.35249534	0.088	0.277465964	0.18122
AKT1	0.243326457	0.02067	0.060544758	0.56459	0.088967661	0.39767
TNKS1BP1	0.376328156	0.02069	-0.262190562	0.10766	0.263966385	0.10491
CCDC147	-1.026198842	0.02072	-0.891052964	0.04263	-0.814812053	0.05819
RIOK1	-0.391720676	0.02074	-0.014289304	0.93179	-0.012338725	0.94156
ACER1	1.296383872	0.0208	0.392937212	0.50368	0.995207131	0.08023
EMC3	-0.225184764	0.02079	-0.025907937	0.78722	-0.128880068	0.18336
ESF1	-0.350303598	0.0208	0.184367166	0.21655	-0.200896953	0.18274
MRPS36	-0.417568903	0.0208	-0.109385663	0.53752	-0.103335296	0.56402
ERF	0.379001108	0.02081	-0.072936578	0.65851	-0.060286889	0.7146
TONSL	-0.410018516	0.02082	-0.916989047	3.07E-07	-0.284277747	0.10827
CCND3	0.410232618	0.02088	0.103791283	0.5588	0.11080965	0.53476
AMICA1	-0.726860606	0.02095	-1.000825408	0.00154	0.259186823	0.40731
LMBR1L	0.417782512	0.021	-0.029090224	0.87292	0.179693741	0.32396
CHFR	0.305313103	0.02105	-0.115670261	0.38463	0.290832969	0.02802
HIST1H2BN	-0.438482471	0.02108	-0.210204634	0.26528	-0.131436341	0.48714
NFIB	0.370357972	0.02109	0.07363234	0.64643	0.392678318	0.01445
DDIT3	-1.226801189	0.02113	-1.406680288	0.0082	-0.976976302	0.06572
PHYKPL	0.288215787	0.02113	-0.320336912	0.01116	0.264877452	0.03358
ACE2	1.332098492	0.02117	0.499548628	0.38756	1.333977751	0.02098
PISD	0.314785852	0.02121	0.164019612	0.22838	0.300247628	0.0278
AKAP12	0.586157979	0.02121	0.322417043	0.20503	-0.31199484	0.22038
C8orf47	-0.755259007	0.02122	-0.570135118	0.07742	-0.028894339	0.92811
PICALM	0.243316337	0.02123	0.342351466	0.00117	0.206150452	0.05095
SPATA33	-0.476254596	0.02127	-0.274086045	0.18785	-0.167443307	0.41044
GIMAP1	1.238100249	0.0213	0.526822227	0.34303	1.47166706	0.00588
RAB20	-0.498879049	0.02132	-0.268092934	0.21065	-0.296139666	0.16859
RAB6A	0.246299208	0.02134	0.307267813	0.00399	0.280699514	0.00866
TMX3	-0.277791011	0.02137	-0.005343909	0.9644	-0.263604082	0.02902
FREM2	-0.432654772	0.0214	-0.354331847	0.05912	-0.374154967	0.04637
C6orf223	1.205690809	0.02141	0.409945869	0.44142	1.213412577	0.02016
MSL2	-0.263842145	0.02142	-0.228981393	0.04477	-0.264891484	0.02097
METTL21A	-0.261581692	0.02143	0.072055331	0.52713	-0.165920819	0.14509
SIRT5	-0.37156802	0.02147	-0.083021018	0.60313	-0.169304012	0.2908
NEXN	0.52019818	0.02149	0.309369904	0.17066	0.120154488	0.5969
SH3BP1	0.363588662	0.02149	-0.18265105	0.24989	0.198620394	0.20942
ZBTB47	0.424548095	0.0215	-0.189945151	0.31386	0.182264302	0.32768
CNRIP1	0.778598303	0.02154	0.216384535	0.5254	0.119906227	0.72618
DSEL	0.59269456	0.02155	-0.136265651	0.59879	-0.001292173	0.99602
PRSS3P1	-1.075754622	0.02154	-0.015501538	0.97356	0.136189627	0.77095
AFF1	0.366639191	0.02158	-0.135833717	0.39485	0.288424283	0.07062

EXOC5	-0.275641208	0.02161	0.156366873	0.18956	-0.111715321	0.35014
SOX12	0.366517147	0.0216	-0.367811596	0.02323	0.018970349	0.90609
SLC12A4	0.469414209	0.02163	0.061367794	0.76456	-0.204423422	0.31928
GPN1	-0.203887944	0.02166	0.264565699	0.00205	-0.056611309	0.51959
KCTD21	0.408083487	0.02167	-0.17383254	0.33219	0.251988074	0.15722
RETSAT	-0.374602279	0.02167	-0.235786399	0.14705	0.096476998	0.55244
VTI1B	-0.194105893	0.02168	0.117905165	0.15791	-0.025298666	0.76347
ASTN1	1.643384777	0.02169	-0.625668435	0.40954	-0.396499135	0.61877
PPWD1	-0.25839005	0.02169	0.056931062	0.60711	-0.101044262	0.36583
ATP5O	-0.270074263	0.02171	0.143183918	0.22064	-0.162091856	0.16705
ZBTB8B	-0.434396259	0.02171	-0.78682892	3.68E-05	-0.619287381	0.00114
MFSD5	0.346717969	0.02173	0.194599795	0.19457	0.241121294	0.10984
MINPP1	-0.299926466	0.02173	-0.008942752	0.94475	-0.229310797	0.07837
RAMP2	0.651467135	0.02178	0.093326304	0.74564	0.461907404	0.10698
RHOBTB3	0.340581224	0.02177	0.300669696	0.04264	-0.167721113	0.25939
TARBP2	-0.314775483	0.02177	-0.234686284	0.08328	-0.19729434	0.14834
PCDHGB7	0.467140546	0.02179	-0.091094246	0.65624	-0.21567622	0.29413
BRI3BP	-0.3189054	0.02181	-0.0411128	0.7669	0.102528066	0.45976
AXL	0.473941729	0.02182	0.189116712	0.36051	-0.272000342	0.19166
NTRK2	1.290762012	0.02184	0.878235029	0.11909	-0.081413351	0.88622
AS3MT	-0.344342371	0.02192	-0.384983339	0.00965	-0.554743198	0.00027
STK39	-0.246287316	0.02194	0.068196642	0.52247	-0.067543708	0.52776
GRAMD4P	0.908791413	0.02197	0.574249778	0.15182	0.622646964	0.12245
GNS	-0.257841904	0.02199	-0.032939314	0.76917	-0.154371642	0.1697
C19orf26	0.976060234	0.022	0.122336038	0.78231	0.014555685	0.9737
DERA	-0.291883834	0.02201	-0.000593995	0.99623	-0.081213908	0.52047
PAPD7	0.29667521	0.02202	-0.105294313	0.41795	0.132525865	0.30794
CRYL1	-0.416309384	0.02204	0.008919298	0.96053	-0.053390723	0.76764
GTF2H2	-0.38384148	0.02203	-0.21291407	0.1993	-0.118338369	0.47643
P11-206L1	-0.947173032	0.02203	-0.290481893	0.47488	-0.959075139	0.02128
ELF2	-0.20594071	0.02206	-0.033742926	0.7042	-0.105636784	0.23744
RCCD1	-0.396656339	0.02207	-0.136718507	0.42432	-0.035871782	0.83412
VOPP1	-0.241652089	0.02214	-0.252802009	0.01599	-0.118001085	0.26094
C3orf33	-0.467641435	0.02215	-0.028610744	0.88301	-0.28170389	0.15869
ETF1P2	0.813662025	0.02218	0.202762977	0.57759	-0.200698776	0.59584
GLUL	-0.319890206	0.02218	0.131371951	0.34675	-0.008987139	0.94871
SPATA18	-0.556704278	0.02223	-0.189263249	0.43184	-0.171399306	0.4779
ARF1	0.235123562	0.02228	0.060814903	0.55426	0.153179144	0.13648
CD276	0.279739478	0.02228	-0.094890063	0.43876	-0.030367581	0.80447
HIST1H4J	-0.626639673	0.02228	-0.068610364	0.80216	-0.474422286	0.08344
MATN1	1.319411627	0.02228	0.391781888	0.51116	0.459567478	0.43542
MED16	0.348800536	0.02227	-0.172274911	0.26261	0.156369506	0.30707
POM121	0.86832741	0.02228	-0.275264564	0.47094	0.694778327	0.06764
ZFYVE27	0.32391569	0.02226	-0.349357153	0.01485	0.05663438	0.69095
OSBPL5	0.463073586	0.0223	0.029214879	0.88626	-0.047393786	0.81706
TOX4	0.279216897	0.02231	0.349184569	0.00417	0.112750738	0.35654
NUP210	-0.640798236	0.02239	-0.850765978	0.00238	-0.502939006	0.07221

ACADS	-0.408923843	0.0224	-0.397781163	0.0245	-0.003416349	0.98442
CD37	-1.776533193	0.02243	-1.306941293	0.09339	-1.413650786	0.06996
TVP23B	-0.325558822	0.02242	-0.095315373	0.50187	-0.015165914	0.91498
RFWD3	-0.313276619	0.02243	-0.022149219	0.87097	-0.254887117	0.06279
ZNF740	-0.326510572	0.02247	-0.249659568	0.07973	-0.066212607	0.64269
ZNF480	-0.306375153	0.02248	0.039487728	0.76604	-0.062270617	0.64085
DNALI1	-0.36004865	0.02251	-0.002671441	0.98635	-0.23769664	0.13125
MRPS17	-0.286007899	0.02252	0.168549645	0.16826	-0.119108701	0.3377
KIAA0141	-0.275712683	0.02255	-0.38071096	0.00166	-0.074146387	0.53803
CTAGE5	-0.261682271	0.02256	-0.168205472	0.13922	0.029132346	0.7977
ADPRM	-0.423217023	0.02257	-0.078263257	0.65905	-0.103409751	0.5655
TMEM38A	-0.568368491	0.02259	-0.413331359	0.0948	-0.26193354	0.28911
LSM2	-0.300876066	0.0226	0.152739569	0.23655	-0.207525822	0.11403
CAMK2D	-0.252477657	0.02261	0.153084442	0.16457	0.089780551	0.41587
MORC3	-0.215174552	0.02263	0.167178073	0.07335	-0.079863423	0.39642
ZNF747	0.385700122	0.02269	0.155750901	0.35831	0.187615226	0.27124
ABHD12	-0.260413927	0.02278	-0.187118915	0.09932	0.016667759	0.8833
COG4	-0.2521015	0.02279	-0.253526911	0.02099	-0.212576544	0.05331
HOOK1	-0.508530352	0.02279	0.136120687	0.54041	-0.061676522	0.78173
LPHN2	0.303590712	0.02279	0.24065236	0.07091	-0.039519358	0.76708
HIST1H2BD	-0.388822008	0.02282	0.029998016	0.86029	-0.128296147	0.45202
PAFAH1B2	-0.182610414	0.02282	0.210398283	0.00829	0.033346379	0.67698
TMEM14B	-0.230031365	0.02285	0.274352852	0.00539	0.071384901	0.47439
KIAA0930	0.32800823	0.02287	0.138182218	0.33734	0.120456203	0.40514
TRAF3IP2	0.340639356	0.02286	0.391404504	0.00875	0.448903647	0.00265
XRCC6BP1	-0.471283067	0.02287	0.130415677	0.50148	-0.342875312	0.08857
WTIP	0.551332068	0.02291	0.185248289	0.44603	0.292354937	0.2283
BDKRB1	1.212653054	0.02297	0.290629712	0.59229	0.037754385	0.94623
FSTL3	0.557765278	0.02296	0.414359692	0.09118	0.126380226	0.60875
TMEM37	0.617180775	0.02297	0.351091678	0.19593	0.733568966	0.00681
TMSB4XP4	-1.059082203	0.02297	0.225107656	0.58662	-0.309063594	0.47446
RAD50	-0.250018987	0.02299	0.059330628	0.58668	-0.021215869	0.84634
CYB5R2	-0.571010124	0.023	0.176771643	0.47769	-0.389935189	0.11936
EFR3B	0.668274011	0.02307	0.062515404	0.8326	-0.386382113	0.19659
PEAR1	0.968357797	0.02307	0.221136489	0.60502	0.464556706	0.27684
TTF2	-0.450954596	0.02309	-0.147721683	0.45485	-0.236063728	0.23336
NFE2L1	0.414587822	0.02313	0.252160077	0.1672	0.209978949	0.25011
IGIP	-0.496789683	0.02314	-0.53149324	0.01386	0.118330529	0.56934
ATAT1	0.348455968	0.02316	-0.11612657	0.46005	0.137293773	0.37652
SPG20	0.36255758	0.02316	0.283105934	0.07576	0.053476477	0.73821
ITGA7	0.947274242	0.02317	0.414461359	0.32078	0.483532929	0.25083
TRMT13	-0.340109918	0.02318	0.041153712	0.78058	-0.172320386	0.24663
ITSN1	0.255389344	0.0232	-0.101371043	0.36768	0.333831845	0.003
NRIP3	0.629393028	0.02325	0.652339512	0.01865	-0.223207415	0.43588
PNPLA6	0.499458319	0.02325	-0.100864233	0.64886	-0.13197922	0.55186
TIMP3	0.555158862	0.02324	0.550035775	0.02447	-0.053382136	0.8276
TMEM126A	-0.364080832	0.02324	0.089135688	0.56536	-0.02154451	0.89078

ACYP1	-0.529845045	0.02327	-0.237695615	0.29568	-0.370382372	0.1083
SPDYE3	0.399690703	0.02335	-0.087902893	0.62394	0.355758124	0.04333
ZNF433	-0.409442631	0.02337	-0.46256395	0.00862	-0.45168423	0.01137
P11-986E7	1.12977241	0.02339	1.714116656	0.00037	0.792588615	0.11815
UBAP2L	0.334334869	0.02338	0.16737026	0.25675	0.199528703	0.17635
C4orf19	0.418315978	0.0234	0.65005091	0.00041	0.857771856	3.03E-06
CD46	-0.232122546	0.02342	-0.069568965	0.49637	0.0751534	0.4625
GATA3	1.238676395	0.02347	0.504226395	0.36346	-0.047632798	0.93313
FAM92A1	-0.293573286	0.02349	0.308011234	0.01597	0.044447437	0.73122
GPA33	1.500162294	0.02349	1.845011702	0.00531	1.453789446	0.02814
DNAJC10	-0.24324816	0.02352	0.147453106	0.16879	0.131200982	0.22115
POGLUT1	-0.280635328	0.02353	-0.086883226	0.47744	-0.204021464	0.09823
ZNF362	0.503219253	0.02357	-0.226915422	0.30896	0.00777183	0.97217
KLHDC2	-0.323005173	0.02361	-0.14737609	0.29891	-0.084421728	0.55236
MPP7	-0.354678374	0.0236	0.257399239	0.09363	0.183098012	0.23512
CGN	-0.352236922	0.02368	-0.472459408	0.00243	0.060591594	0.69675
POU4F1	1.482869805	0.0237	1.1421339	0.08237	1.313646387	0.04559
AGBL4	-0.879381325	0.02373	-0.89022648	0.02043	-0.929509553	0.019
GDF15	-0.845970174	0.02373	-1.160954942	0.00192	-0.870909527	0.01991
RAB4A	-0.285753762	0.02373	0.150192382	0.22954	-0.130559016	0.29958
RGMA	0.667044273	0.02372	-0.328126087	0.26932	-0.035624819	0.90429
HOXB3	0.699074965	0.02376	0.018788592	0.95178	0.00267309	0.99315
RHOV	1.033624449	0.02376	0.89813765	0.04932	0.94324298	0.03918
FNTB	0.364331052	0.02377	0.019298406	0.90445	0.073539741	0.64807
LAMP3	0.880505055	0.02379	1.228795329	0.00118	0.850498997	0.02746
CYB561A3	-0.270899762	0.02383	-0.277756021	0.0204	-0.201773695	0.09202
SLC39A5	-0.399855211	0.02383	-0.546824084	0.00198	-0.147444444	0.40334
PHB	-0.299033857	0.02384	0.216877666	0.10001	-0.095787484	0.46839
AUNIP	-0.465588669	0.02387	0.015601746	0.93783	-0.208244716	0.30429
PTGFR	0.858015596	0.02387	0.657551794	0.08315	-0.565868008	0.15636
PRELID1	0.409986195	0.02389	0.791534444	1.24E-05	0.394319813	0.02977
HMGB1P10	-1.689129547	0.0239	-0.217097633	0.7593	-1.602845596	0.03095
CPNE5	1.316518943	0.02392	1.255549881	0.03187	1.717061434	0.00291
AC093642.5	-0.765980904	0.02395	-1.097934761	0.00138	-0.291853471	0.38055
C1QBP	-0.330905677	0.02396	0.318603299	0.02881	-0.020231417	0.88992
CDA	1.33519001	0.02396	1.247879876	0.03476	0.240966986	0.69192
NAPA	0.24522552	0.02397	0.029316093	0.78832	0.219655967	0.04304
SMAD6	0.464491761	0.02397	-0.404767475	0.04976	0.703569719	0.0006
TMCO3	-0.369846812	0.02394	-0.155096737	0.34217	-0.039114992	0.81075
ZNF684	-0.592031458	0.02394	-0.109639122	0.66559	-0.163136286	0.52283
HMMR	-0.465521206	0.024	0.125668583	0.5389	-0.273461423	0.18356
HEATR3	-0.352809945	0.02402	0.154396457	0.31717	-0.083774346	0.58881
PTPRF	-0.310418977	0.02403	-0.645353068	2.72E-06	-0.241698568	0.07878
AMPD2	0.281697619	0.02406	-0.183363077	0.14453	-0.15723842	0.21195
PRDM1	0.601814326	0.02408	-0.179650692	0.5032	0.042641507	0.87337
ASAH2B	-0.429183877	0.02412	0.05149352	0.78205	0.06628344	0.72382
HLA-A	0.347948524	0.02412	0.266372302	0.08414	-0.023474293	0.87919

HLCS	-0.359657467	0.02412	-0.408835907	0.00998	-0.291707523	0.06657
PHB2	-0.200075106	0.02411	0.17251716	0.05068	-0.019956294	0.82164
CSNK1D	0.259587898	0.02415	-0.31311254	0.00696	0.27574513	0.01679
TFB2M	-0.336539809	0.02418	0.329386143	0.02045	-0.091093223	0.53373
MGLL	0.79263522	0.02422	-0.282901947	0.42211	0.561146221	0.11077
PLCB2	-0.981033033	0.02422	-0.940188716	0.03022	-0.334944407	0.42873
SYNPO2	0.522348743	0.02423	-0.082058968	0.72358	-0.154093309	0.50678
ZNF761	-0.307585118	0.02423	-0.124373173	0.35777	-0.131840519	0.33093
MGST1	-0.397118843	0.02425	0.156345149	0.37346	0.131292217	0.45506
TSHZ1	0.426808863	0.02426	-0.149860594	0.43192	0.100459149	0.59845
TTC19	-0.243717449	0.02428	0.100873378	0.34397	0.009284313	0.93099
BEAN1	1.118516833	0.02431	0.120929551	0.81604	0.852991328	0.08992
SLC25A14	-0.40924076	0.02433	0.080224181	0.63976	-0.216158746	0.22207
HDHD1	-0.268057267	0.02436	0.075486763	0.51488	0.02005233	0.86389
OVOL1	-0.41308798	0.02435	-0.671257641	0.00026	-0.159414175	0.38237
EML2	-0.476572887	0.02438	-0.530217198	0.01172	-0.023062707	0.91194
GUCA2A	2.256322494	0.02438	2.261742955	0.02371	-1.307132485	0.26614
ECT2	-0.360199013	0.02439	0.241442685	0.12989	-0.167049927	0.29595
ZNF865	0.575898237	0.02441	-0.217084029	0.41527	0.37952015	0.14145
FILIP1L	0.38502953	0.02442	0.535976658	0.0017	0.219138415	0.20048
PPP4R2	0.237287591	0.02442	0.4705766	7.44E-06	0.287692658	0.00628
NPRL2	-0.47109221	0.02444	-0.280163822	0.17864	-0.299951184	0.15059
HIST1H4A	-0.481272886	0.02445	-0.051963779	0.80777	-0.246813661	0.24819
HOMER3	0.545226015	0.02447	0.081805412	0.7378	-0.102736778	0.67639
TCTEX1D2	-0.584738079	0.02448	-0.128218492	0.61029	-0.854863644	0.00118
ST8SIA6	0.759101863	0.0245	0.803043451	0.01696	0.495883051	0.14316
PAK1IP1	-0.286686308	0.02451	0.2767829	0.0254	-0.192854378	0.12779
DLX1	3.074244046	0.02454	3.060437886	0.02431	1.88722362	0.17069
PANK1	-0.293134681	0.02455	-0.11105413	0.39031	-0.028539005	0.82534
TRIM34	-0.504060694	0.02462	-0.108039399	0.62151	-0.072918869	0.74006
HELB	0.320309394	0.02463	-0.053935877	0.70555	0.019689498	0.89037
UBXN8	-0.421816421	0.02465	-0.301127583	0.10088	-0.216439532	0.24178
CLDN16	1.560008291	0.02467	0.616683251	0.3957	0.783739868	0.26945
DDB1	0.159637842	0.02467	0.124605006	0.07879	0.108443698	0.12679
PAPSS2	0.568894568	0.02474	0.859838762	0.00066	0.141565366	0.57771
RASSF4	0.506125371	0.02474	0.485261685	0.03101	0.078341353	0.73429
P11-137H2	-0.279435453	0.02478	-0.070576391	0.56445	-0.245025758	0.04819
CTSA	0.453767283	0.02484	0.148247573	0.46339	0.160673646	0.42738
C1orf159	0.397792491	0.02485	-0.112984439	0.52958	0.540100585	0.00225
SPEF2	-0.634783214	0.02488	-0.337305029	0.22909	-0.438722897	0.11836
VCP	0.217723829	0.02491	0.26906476	0.00552	0.120719514	0.21359
ITSN2	0.437312877	0.02494	0.204780106	0.29325	0.487699661	0.0122
LNX2	-0.350400687	0.02494	-0.183027023	0.23971	0.106976708	0.4914
SDHAF1	-0.418341647	0.02493	-0.035995494	0.84002	0.043004836	0.81015
SHISA3	1.043443429	0.02494	0.342578314	0.4633	1.307495302	0.00488
NFIA	0.563386944	0.02498	-0.089997628	0.72055	0.140556149	0.57632
PDGFC	0.478754926	0.02498	0.435565937	0.04129	0.099159373	0.64306

CA4	2.115235519	0.02505	0.757217467	0.42541	1.183024207	0.21274
UQCRC1	-0.305169408	0.02507	-0.092151437	0.49789	-0.100729223	0.45883
SOD3	1.04535929	0.02509	0.78591859	0.09245	0.18443698	0.69572
ANKS6	-0.314259417	0.02511	-0.128273732	0.35684	-0.077334475	0.57945
C15orf39	0.383629719	0.02512	-0.388562638	0.02502	0.22589868	0.18856
SIVA1	-0.389608412	0.02512	-0.252980455	0.14471	0.009873592	0.95443
GRB2	0.245302332	0.02522	0.082402645	0.45175	0.259055609	0.018
STAG2	-0.192540262	0.02522	0.151408267	0.07675	-0.017908892	0.83476
HSPA4L	-0.541908859	0.0253	0.361994319	0.13278	-0.339103037	0.16092
ANKEF1	-0.422597387	0.02532	0.157569729	0.39693	0.054256799	0.77146
ERCC6L2	-0.22788408	0.02532	-0.001377001	0.98915	-0.197961459	0.05182
AGO2	0.419763346	0.02534	0.17837877	0.34216	0.178648171	0.34164
DFNA5	-0.591061064	0.02535	-0.126113854	0.62887	-0.489791224	0.06359
LCP1	1.528401973	0.02539	1.461727683	0.03252	0.64554275	0.34664
WRN	-0.324636341	0.02539	0.075404043	0.59876	-0.203073709	0.15957
ZBTB7C	-0.622358451	0.0254	-0.434766279	0.11646	0.194448483	0.48099
COA5	-0.315722034	0.02544	-0.014949229	0.91433	-0.008289796	0.95261
POR	0.339190657	0.02549	-0.096792434	0.52452	0.141669025	0.35165
BAALC	1.388164484	0.02553	1.233271357	0.0475	0.999894304	0.11062
DPYSL4	0.681002916	0.02551	-0.272886973	0.37528	0.033622957	0.91307
GPR110	0.986169724	0.02552	0.398712679	0.36789	0.690125264	0.11907
OLFM2	0.511212032	0.02553	-0.085124227	0.71516	-0.178571949	0.45029
RGN	-0.947221462	0.02555	-0.402215524	0.32488	-0.849082655	0.04528
SBSPON	-0.697059424	0.02554	-0.090415168	0.7687	-0.405619236	0.19158
ZBTB24	-0.308758235	0.02555	0.096004201	0.47824	-0.227794804	0.09768
FGFBP1	1.023036124	0.02556	0.442287116	0.33608	0.942050877	0.03983
INTS10	-0.250606211	0.02557	0.211849353	0.05594	0.073691593	0.50802
SMU1	-0.124494117	0.02561	0.160095293	0.00336	-0.064000316	0.2486
PSMG1	-0.344488563	0.02562	0.330781449	0.02889	-0.048553492	0.75075
LYRM1	0.35678013	0.02565	0.89294562	1.39E-08	0.208304448	0.19339
CBLN3	0.895722941	0.0257	-0.111006702	0.78681	0.481276369	0.23313
TM2D1	-0.239928216	0.02569	-0.082850066	0.43402	-0.130870602	0.21742
FUT6	0.817802966	0.02572	0.352971066	0.3548	-0.246000704	0.54886
GTPBP6	0.324865494	0.02572	0.165273584	0.25611	0.389655021	0.00727
EDNRA	0.581219888	0.02577	0.344307288	0.18657	-0.222516791	0.39423
GUCD1	0.261773537	0.02579	0.069496911	0.5534	0.34889481	0.00295
SASH1	-0.309248414	0.02581	-0.346003631	0.0121	-0.068589139	0.61898
DPP9	0.419860103	0.02583	-0.046766786	0.80473	0.149515405	0.42832
KATNB1	0.455900976	0.0259	0.370652176	0.0694	0.221901985	0.27865
PAAF1	-0.407648537	0.02596	-0.022796008	0.9003	-0.368483791	0.04464
PARP1P1	0.707838423	0.02602	0.996868682	0.00123	0.856564873	0.00633
ACSL5	0.439680078	0.02605	0.595834638	0.00252	0.421487053	0.03286
LRRC20	-0.543353675	0.02606	-0.129047051	0.5898	-0.385541706	0.11287
UGT2B11	-1.083176383	0.02608	-0.301074617	0.52891	-0.84496738	0.08119
EIF3E	-0.355402401	0.02611	0.333692716	0.03656	-0.148442515	0.3526
FILIP1	0.721588521	0.02611	0.80771017	0.01244	0.178362594	0.58764
IRF3	0.271806364	0.02612	0.18131366	0.13821	-0.014477569	0.90603

GTF2H2B	-0.359963075	0.02614	-0.18477904	0.24978	-0.136024805	0.39772
ARHGAP35	0.396522268	0.02615	0.005755244	0.97425	0.27304994	0.1255
CBR4	-0.261753368	0.02617	-0.0676774	0.55958	-0.059534789	0.61038
SPDEF	-1.051341031	0.02624	-0.18759546	0.68979	-0.327165506	0.48692
IKZF2	-0.421753148	0.02626	-0.452442858	0.01693	-0.057466968	0.76128
RPS19BP1	-0.286889662	0.02626	-0.018635839	0.88357	-0.031683064	0.80453
ENPP4	-0.434919586	0.02628	-0.035994135	0.85293	0.00780222	0.96798
TNFAIP8	0.503851682	0.02629	0.67949302	0.00268	0.477234704	0.03532
MAX	0.266784113	0.02641	0.497102658	2.93E-05	0.110589626	0.35842
TTF1	-0.352539081	0.02641	0.088487448	0.56699	-0.095288309	0.54362
ZDHHC3	0.23361713	0.02642	0.061686437	0.55597	0.346642692	0.00093
CXADR	-0.319792598	0.02643	-9.80E-05	0.99946	-0.018529633	0.89746
ATP2C1	-0.219734677	0.02645	0.072154394	0.46251	-0.015146313	0.87781
HIST1H4F	-0.529806989	0.02645	-0.301427829	0.2059	-0.285260424	0.23149
CYP2C8	-1.235048399	0.02647	-0.947037115	0.0856	0.174191155	0.74222
FAM229B	-0.61045746	0.02647	-0.05962274	0.82421	-0.879681457	0.00145
FAM149A	-0.415286839	0.0265	-0.197607709	0.28629	-0.035378856	0.8482
GAP43	2.161846398	0.02653	1.205205715	0.21843	0.651841785	0.50873
POLR2F	-0.265536166	0.02663	0.242432027	0.03618	-0.306849103	0.01045
ZFPL1	0.390856921	0.02662	0.168144855	0.34078	0.37277073	0.0346
AIM1	0.628594435	0.02666	0.57845363	0.04117	1.029459522	0.00027
ATP5F1	-0.210540499	0.02666	0.335033037	0.00039	0.031822317	0.73686
ABCD1	0.473231175	0.02667	-0.459933377	0.03681	-0.14161911	0.51924
NDUFA8	-0.241141904	0.02668	-0.00079368	0.99403	0.066920159	0.52957
PRRG1	0.516995605	0.02669	0.752909506	0.00123	0.354318599	0.12892
CCDC3	0.656013244	0.02671	-0.19821754	0.50491	-0.323623123	0.27749
TTC4	-0.245650853	0.02672	0.236363922	0.027	-0.032025781	0.76915
AKIRIN1	0.255375457	0.02674	0.300999461	0.00883	0.155020712	0.17873
P11-427H3	0.371360679	0.02675	-0.080192974	0.63386	0.003079039	0.98542
TMED2	-0.203989293	0.02677	0.087301031	0.34202	0.107285775	0.2433
OXNAD1	-0.262950711	0.02678	0.132180173	0.24824	-0.049080757	0.67358
G3BP1	-0.207999823	0.02679	0.15822893	0.09135	-0.044080525	0.6385
MMP2	0.398506344	0.02684	-0.02125924	0.90604	-0.167386588	0.35337
RN7SL467F	-0.596719804	0.02684	-0.136266051	0.5951	-0.45495381	0.08619
RORB	1.818576842	0.02684	1.557020847	0.05821	0.628696522	0.45034
STARD9	0.599606707	0.02684	-0.354881619	0.19302	0.129348535	0.63454
ATPIF1	-0.279635205	0.02685	0.04648838	0.71116	0.00284399	0.98196
TMEM254	-0.33553297	0.02687	-0.02488203	0.86727	0.032694318	0.82641
JUNB	0.749512724	0.02689	-0.088069559	0.79516	0.196879889	0.56149
MT-ND5	0.660900194	0.02698	0.937744736	0.0017	1.188755894	6.91E-05
MTX3	-0.329004078	0.02697	-0.094930699	0.51605	-0.38546597	0.00917
SYS1	0.279020771	0.02698	0.191505205	0.12772	0.17092434	0.17582
AJAP1	1.270295321	0.02702	0.353029456	0.55214	1.734231603	0.00204
DNAAF2	-0.316012161	0.02707	0.177641107	0.19683	-0.207599968	0.14248
PIN1	0.259557435	0.02707	0.160476486	0.17348	0.085520684	0.46959
11-574K11	0.761969058	0.02709	-0.968155672	0.00598	0.115556295	0.73767
APOA2	1.917612886	0.0271	2.230480653	0.01011	-1.062932991	0.22496

DPF2	0.263112408	0.0271	0.06594076	0.58109	0.123623124	0.30076
C1orf226	0.603327214	0.02715	0.160068009	0.55875	0.779121332	0.00414
ZBTB14	0.918879598	0.02715	0.12858276	0.76161	0.19478253	0.6483
DHX36	-0.301866043	0.02718	0.023557843	0.86219	-0.119533056	0.38033
ST6GAL2	0.711004316	0.02717	-0.159256083	0.62115	-0.415161243	0.19815
DYX1C1	-0.416793281	0.02721	-0.242267744	0.18252	-0.322883524	0.08192
SERPINE2	0.71769471	0.02721	0.300101291	0.35615	-0.237449504	0.46604
PDE9A	0.347718113	0.02725	0.144444179	0.35823	-0.045837107	0.77219
ERH	-0.264203793	0.02728	0.205851644	0.08374	-0.183425513	0.12508
FSTL1	0.585317092	0.02729	0.559970048	0.03468	0.081359751	0.75903
ANXA3	0.269361117	0.0274	0.540455062	8.73E-06	0.513765374	2.43E-05
PRIM2	-1.089452517	0.02739	0.146464135	0.76165	-0.473923344	0.33021
RPSA	-0.262132483	0.02742	0.177672984	0.13465	-0.042648943	0.71963
KNC01-221	-0.709026209	0.02744	0.235213273	0.42758	-0.848856041	0.0089
DTD1	-0.33589927	0.02748	-0.01795738	0.90428	-0.152937596	0.31073
S1PR1	0.855869135	0.02757	-0.345945468	0.37942	-0.048822533	0.90134
CD44	0.778149116	0.02761	0.740924349	0.03595	0.084269413	0.81164
GMPR2	-0.25227008	0.02762	-0.018577845	0.87001	-0.067335339	0.55526
MDC1	0.479744411	0.02762	-0.295206075	0.17623	0.054464456	0.80322
C7orf73	-0.200177489	0.02765	0.081817934	0.35672	-0.022737946	0.80027
CDK4	-0.283430302	0.02766	-0.018349475	0.88624	-0.27570746	0.03196
STX11	1.856210084	0.02768	1.209179032	0.16432	1.520465433	0.07652
CELF2	0.543285449	0.02769	0.149591049	0.54454	0.064920959	0.79289
TTI1	-0.224773576	0.0277	-0.091310523	0.36349	-0.143807799	0.15621
PSMD8	0.240360057	0.02774	0.555992511	3.23E-07	0.205862511	0.05935
NCAPD3	-0.367776063	0.02784	-0.013325798	0.93596	-0.119945272	0.47109
ABTB2	0.752596737	0.02786	-0.143587196	0.67781	0.660411969	0.05391
EPCAM	-0.434255966	0.02787	0.16910967	0.39145	0.010280473	0.95846
P11-343C2	1.379456017	0.02789	0.869822002	0.16791	0.15233371	0.8132
GALK2	-0.323328922	0.02791	-0.192796709	0.18333	-0.267546198	0.06666
DLG1	0.21307437	0.02793	0.310035606	0.00133	0.171933739	0.07604
PBX2	0.177811553	0.02792	-0.071593799	0.37585	-0.051371682	0.52654
DYNLL1	-0.192795164	0.02797	0.131157361	0.13238	-0.114886334	0.18952
SERPINB6	-0.307832723	0.02801	-0.116668707	0.40374	-0.110811193	0.4281
XPNPEP1	0.236367173	0.02801	0.203050687	0.05829	0.230634274	0.03184
SCAND1	0.706981655	0.02804	0.053467538	0.86879	0.681978791	0.0341
ZC3H7B	0.194791999	0.02805	-0.204490048	0.02153	-0.068954814	0.43837
RHOBTB2	0.385010613	0.02806	-0.127123882	0.47163	-0.203999461	0.24808
NDUFB4	-0.213475149	0.02808	0.224550581	0.01857	-0.212845307	0.02797
TTC30A	-0.260140051	0.02816	0.104389332	0.37001	-0.112365499	0.3397
TBCD	-0.320804994	0.02822	-0.146183466	0.31555	-0.144591009	0.32117
TNS3	0.326626737	0.02822	-0.194170186	0.19261	-0.061453034	0.68015
EXO1	-0.563144647	0.02824	-0.385062075	0.12988	-0.334065461	0.19032
NECAP2	0.24816134	0.02826	0.169978252	0.13191	0.120994872	0.28636
SSBP3	0.358256605	0.02826	-0.173249206	0.29323	0.02476851	0.87995
TBC1D15	-0.308193205	0.02825	0.10803928	0.43483	-0.113340716	0.41891
R3HDM1	0.351935144	0.02829	-0.075943591	0.63539	-0.118216429	0.46296

OCIAD1	-0.20110147	0.02835	0.077907621	0.39159	-0.121271353	0.1842
IFNGR2	0.396390237	0.02837	0.480738527	0.0078	0.426202625	0.01839
MMP12	2.293197362	0.02838	1.674360305	0.11324	4.580503469	7.13E-06
TYMS	-0.381446305	0.02844	0.052649522	0.75973	-0.205930401	0.23449
BIVM	-0.30388731	0.02846	-0.137005322	0.31925	-0.301683607	0.02931
CAMKK2	-0.255378028	0.02848	-0.155377449	0.17724	0.007336956	0.94942
LETM1	0.249847351	0.02848	-0.183758226	0.10726	0.159228873	0.16235
RLIM	-0.164274923	0.02847	0.182009872	0.0144	-0.153572161	0.04029
E2F7	-0.583772499	0.02853	-0.815735607	0.00219	-0.437863085	0.09971
NUCKS1	-0.282237354	0.02858	0.028911634	0.82242	-0.173329886	0.17871
C14orf142	-0.376224522	0.02859	0.022531123	0.88852	-0.162584248	0.32854
GRIPAP1	0.2744577	0.02865	-0.310485614	0.01456	0.255876363	0.04093
E2F4	0.184276012	0.02868	0.056302455	0.50218	0.20753725	0.01352
RASL11A	0.515740107	0.02882	0.157528322	0.50468	0.215048857	0.36272
YY1	-0.200374425	0.02882	0.007573246	0.93395	-0.125765033	0.1695
ZCCHC7	-0.330437216	0.02884	0.12751282	0.39186	-0.366811633	0.01485
PCDHA4	-0.380339581	0.02888	0.028687952	0.86715	-0.082958146	0.63078
MTO1	-0.297749667	0.02889	-0.027755676	0.83772	-0.099087812	0.46642
SUGP1	0.397885294	0.02896	0.528423743	0.00369	0.164500323	0.36807
COL1A1	0.710606799	0.02898	0.030267402	0.9259	-0.093191492	0.7746
MEGF9	-0.212539076	0.02903	0.035257062	0.71172	-0.012358095	0.89776
HCAR2	2.007801788	0.02906	1.487093145	0.10655	1.146877378	0.21475
PARG	-0.417838158	0.02906	-0.306321697	0.10586	-0.437301934	0.02142
RPL29	-0.267442939	0.02909	0.206826591	0.09108	-0.053706748	0.66104
GREM2	0.513030386	0.02912	0.069582183	0.76787	-0.237944613	0.31566
TEAD4	0.4355615	0.02912	0.234834036	0.23995	0.155365385	0.44226
VAV2	-0.353267709	0.02913	-0.162163223	0.31411	-0.301689862	0.06219
SMOX	0.718617472	0.02927	0.175860426	0.59416	-0.253969025	0.44468
X1A-SULT1	0.41139154	0.02932	-0.726374942	0.0002	0.364952355	0.0533
PRLR	-1.524313631	0.02936	-2.500867773	0.00065	-0.38907411	0.56715
TAOK1	0.198698613	0.02938	0.04914072	0.58956	0.202154223	0.02663
TMA16	-0.276182273	0.0294	-0.001945567	0.98745	-0.364415921	0.00377
DPEP1	1.787607877	0.02944	2.239854925	0.00574	0.725626063	0.39402
AB11FIP1F	0.426728842	0.02946	-0.494448765	0.01474	0.203678269	0.30197
KLHL2	-0.404813418	0.02948	-0.099561278	0.58816	0.037177034	0.8402
PSMD10	-0.332382817	0.02948	0.019627787	0.89698	-0.051042188	0.73706
ZHX1	-0.214285394	0.0295	-0.088499921	0.36467	-0.004723309	0.96147
GNB4	0.386864142	0.02952	0.024699145	0.88955	-0.167843981	0.34647
GPS2	0.273103184	0.02951	0.174077711	0.16427	0.224057197	0.07396
ARHGEF2	0.272055754	0.02957	-0.080214463	0.52165	-0.14279312	0.25506
LRRC37A6I	-0.772436844	0.02961	-0.838514578	0.01427	-0.520055669	0.12424
CAPZA2	-0.219514256	0.02964	0.074374186	0.45913	-0.082397706	0.41315
CNOT6L	-0.218164989	0.02965	-0.263849001	0.00809	-0.135268905	0.17597
ING3	-0.322642408	0.02966	-0.092968087	0.52407	-0.051755884	0.72416
EWSR1	0.226162474	0.02969	0.293228144	0.00478	0.196592821	0.05873
FAM126A	0.472749027	0.02981	0.674128452	0.00193	0.466411969	0.03228
SNRPB2	-0.211341704	0.02982	0.333077429	0.00049	-0.147160133	0.12917

UMPS	-0.356875541	0.02984	0.036383485	0.82359	-0.181393684	0.26842
SRPR	0.23757578	0.02985	0.066815568	0.54031	0.288305	0.00827
MMAB	-0.267932092	0.02987	-0.087095168	0.47676	-0.03088283	0.80125
HMGB1	-0.23099579	0.02995	0.237814245	0.02521	-0.170390466	0.1092
RASGRF2	0.987222181	0.02995	1.016873853	0.02539	0.126406819	0.78595
AC112229.4	0.811481919	0.02999	-0.535900589	0.17888	-0.113722314	0.77043
CTCF	-0.159811132	0.02997	0.067033811	0.35623	-0.101899339	0.16484
PPFIA1	0.248295707	0.02999	0.150152644	0.18838	0.301241029	0.00847
RAP2B	-0.228571289	0.02998	-0.192117833	0.06695	-0.059534392	0.5703
ANAPC13	-0.227321769	0.03001	0.282167284	0.00589	-0.136859466	0.18903
CLIC2	-0.681040368	0.03003	0.167836876	0.56678	-0.735073232	0.0158
MSL1	0.18678307	0.03007	-0.264161798	0.00221	0.178029938	0.03829
PIAS1	0.332444102	0.03007	0.265340284	0.083	0.263166441	0.08577
DYNLRB1	0.26516294	0.03008	0.451893683	0.0002	0.245764882	0.04428
PNMT	-1.227739118	0.03013	-1.234408587	0.02348	-0.292090766	0.57717
CEBPZ	-0.909356123	0.03015	0.343356801	0.41058	-0.045499549	0.91332
KIAA1244	-0.40170721	0.03019	-0.296730792	0.10892	0.032553193	0.86031
IFRD2	-0.313067069	0.0302	-0.01182354	0.93418	-0.092962694	0.51786
MEIS2	0.330098588	0.03025	0.018451471	0.90366	0.015938984	0.91679
ANG	-0.444387419	0.03031	-0.137933032	0.49869	0.291328296	0.152
DCHS1	0.583264544	0.03032	-0.476862882	0.07716	-0.240173187	0.37325
MTMR12	0.289481736	0.03035	0.377785751	0.00455	0.327230626	0.01426
NELL1	-1.366419864	0.03035	0.215838868	0.73148	-0.381123407	0.54488
STX2	0.485817672	0.03035	0.671884645	0.00265	-0.075482568	0.73847
STX12	0.244586276	0.03037	0.079024499	0.48256	0.071966702	0.52355
PARM1	0.616092913	0.0304	0.50999553	0.07311	0.169028077	0.55274
PTK2B	0.497718865	0.0304	0.085717412	0.71009	0.402709964	0.08044
TAOK2	0.214428125	0.03039	-0.194492754	0.05089	0.227199694	0.02168
DENND2A	0.59561931	0.03045	-0.062495305	0.82145	0.046988262	0.86525
PEX26	0.369999937	0.03045	0.076844342	0.6541	0.111276693	0.51563
SEC22B	-0.192866313	0.03043	0.02951788	0.73876	-0.177721509	0.04584
FGA	1.490227778	0.03048	0.551945911	0.42357	0.065601177	0.92436
KMO	0.840022275	0.03047	0.321837279	0.41267	0.777300206	0.04855
RAI2	0.89923263	0.03047	-0.469768721	0.29972	0.413055567	0.32821
FLYWCH1	0.532554206	0.03051	-0.412317486	0.09939	0.399058889	0.1075
SERTAD1	0.443617504	0.03052	0.090932427	0.65959	0.298891501	0.14649
TRIM56	0.242519216	0.03053	-0.370913681	0.001	0.15775328	0.15958
PRPSAP2	-0.297162107	0.03056	-0.028745404	0.83183	-0.105965928	0.43751
AGA	-0.437430481	0.03059	-0.056848481	0.77598	-0.140682978	0.48269
KIAA0907	0.310680071	0.03059	0.058499027	0.68409	0.261731958	0.06841
STXBP1	0.885458205	0.03058	0.145440694	0.72511	0.049453496	0.90487
EFCAB13	-0.531553541	0.03061	0.03801235	0.87553	0.302136922	0.21243
NEGR1	0.658863559	0.03068	0.390316519	0.20084	-0.085637776	0.77957
PRR16	1.117580001	0.03066	0.724481661	0.16459	-0.154700632	0.77868
SURF4	0.220813409	0.03065	0.251595818	0.01355	0.060403424	0.5545
TRIO	0.548682421	0.03067	0.06859917	0.78702	0.276648566	0.27593
ZW10	-0.200133124	0.03068	0.200174905	0.02634	-0.157242001	0.08702

CYP2R1	-0.420427344	0.03081	-0.223955903	0.24688	-0.051861499	0.78903
FAM178B	1.407063942	0.0309	-0.064387752	0.92633	1.279389863	0.0503
ATG2A	0.314843955	0.03094	-0.333262053	0.02476	0.205115537	0.16108
SAMD14	0.514636311	0.03094	-0.44312282	0.0701	-0.17969475	0.46241
PTPRG	0.4178707	0.03096	0.169351126	0.38189	-0.209785185	0.28009
SNX27	0.252880442	0.03097	0.00340883	0.97685	0.304707575	0.00932
AK4	-0.37144916	0.03101	-0.045439127	0.79177	0.062267947	0.71746
BTG2	-0.446823522	0.03101	-0.491052448	0.0177	-0.215051531	0.29869
STOX2	-0.596829971	0.03105	-0.925055093	0.00082	0.200766447	0.46622
RNF31	0.381034723	0.03109	-0.007260825	0.96731	0.073745629	0.67737
NSUN7	-0.412448688	0.0311	-0.17365526	0.35803	-0.2368913	0.21086
KDM2A	0.217258618	0.03116	-0.307644237	0.00233	0.015147675	0.88061
MVD	0.469925986	0.03117	0.084377201	0.69914	0.491679899	0.0241
ZNF665	-0.41844538	0.0312	-0.230489258	0.23516	-0.110789305	0.56555
MAPK14	-0.214740093	0.03123	-0.24631997	0.01318	-0.011128648	0.91077
NR3C1	0.441825484	0.03123	0.066440541	0.74614	0.29868331	0.1454
PPM1N	-0.727565503	0.03122	-0.226853795	0.47297	-0.353835833	0.27589
FAAH	-0.309095173	0.03125	-0.525925149	0.00024	-0.062787572	0.6582
DIAPH3	-0.355417246	0.03129	-0.023549827	0.88517	-0.339945195	0.03899
SLC41A2	0.371115732	0.03129	0.663119843	0.00011	0.610457593	0.00038
SPAG1	0.678785668	0.0313	0.713730311	0.02281	0.134461927	0.67073
NRNPA1P2	0.721156715	0.03132	0.033913502	0.92242	-0.362250521	0.32383
TRIB1	0.384321426	0.03132	0.079483167	0.65613	-0.113288415	0.52762
NCKAP1	-0.186195789	0.03137	-0.031675442	0.71366	-0.105206387	0.22344
AFF2	0.995836535	0.03139	1.125807654	0.01496	0.573328144	0.21652
POLR2L	0.303173465	0.03141	0.28071558	0.0456	0.272670623	0.05265
SLC6A19	-1.083725052	0.03148	-1.715822494	0.0008	-0.966557479	0.05373
STXBP5	0.337447106	0.03149	0.141279972	0.36746	0.444185497	0.0046
ATXN7L3B	-0.226643247	0.03154	0.078032968	0.45734	-0.069343224	0.50987
GPBAR1	0.697732023	0.03155	0.255586737	0.43341	-0.040605137	0.90182
HAS2	1.128769799	0.03152	0.716243146	0.17255	0.149182209	0.77669
RGPD5	0.581968349	0.03156	-0.040540632	0.88169	0.020584041	0.93954
SEC31B	0.60718458	0.03155	-0.846244699	0.00324	0.307244774	0.27946
SPON1	0.883743397	0.03154	0.804642648	0.05021	0.215935509	0.59992
UPP1	0.625342916	0.03153	0.339790379	0.24285	0.352894412	0.22663
UQCR10	-0.284774288	0.03152	0.025239078	0.84657	0.04563166	0.7273
SLC25A6	0.266748716	0.03157	0.290476275	0.0191	0.218506008	0.0782
URAD	2.472037673	0.0316	2.41713128	0.03531	0.605516247	0.61282
ZNF571	-0.324011285	0.03162	-0.331187269	0.02725	-0.299995085	0.04799
RANGRF	-0.32119917	0.03166	-0.108848424	0.45439	-0.264113072	0.07585
FAM65A	0.609714381	0.03171	-0.189550598	0.512	0.11468549	0.68983
N4BP2	-0.227433572	0.0317	-0.095306508	0.36655	-0.201470047	0.05691
POLG2	-0.358638416	0.0317	-0.442006296	0.00738	-0.426357517	0.00961
SORCS2	1.127057985	0.03171	0.703420997	0.18094	0.10520286	0.842
ANGPT4	-1.074463499	0.03175	-2.232613074	1.05E-05	-0.253431484	0.61031
PIAS2	0.222701567	0.03175	0.238191051	0.02137	0.175747175	0.08999
PRR15	-0.365479572	0.03176	-0.356070134	0.03576	0.109277485	0.5164

OSBPL9	-0.238261198	0.03179	0.14397869	0.19255	0.065924953	0.55174
CLPP	0.344579673	0.03181	0.45530652	0.00434	0.131448995	0.41449
BACH2	1.302994475	0.03187	0.301164066	0.62048	0.335104209	0.58458
11-1212A2	0.528775289	0.03186	-0.412037603	0.10037	0.331057766	0.18047
RAB33A	0.883042716	0.03191	0.465975098	0.2634	0.301324137	0.47775
LARP4B	0.258891321	0.03197	-0.054326647	0.65219	0.273245958	0.02333
MB21D2	-0.327819798	0.03197	-0.147567372	0.3262	-0.108762501	0.47117
ATP5D	0.77078905	0.03206	0.265840948	0.46361	0.774831455	0.03124
KMT2B	0.44225366	0.03211	-0.153704224	0.4603	0.274138118	0.18448
EPN3	-0.672966311	0.03217	-0.443276005	0.15216	-0.765687706	0.0145
ORMDL3	-0.295739981	0.03221	0.097179888	0.47915	-0.186816607	0.17523
PELI1	0.297410075	0.03221	0.295940148	0.03275	0.511499825	0.00022
RSPH9	0.512641316	0.03224	0.086119891	0.72487	0.623801799	0.00841
ZDHHC11	-0.742174061	0.03228	-0.496058755	0.14789	-0.315130449	0.35618
MT-ND4L	0.658356848	0.03232	0.841444472	0.00619	1.036835602	0.00074
MDH2	0.281562439	0.03233	0.280941097	0.03235	0.356306363	0.00669
EFEMP2	0.401843304	0.03239	0.13955999	0.45799	-0.091048099	0.62904
GPRC5C	0.357639556	0.03241	-0.142726246	0.39433	0.426209451	0.01074
VPS9D1	0.498622477	0.03243	-0.400565173	0.08979	-0.193828974	0.41468
BRMS1	-0.239224713	0.03244	-0.007993435	0.94159	0.085401503	0.43759
2-Mar	-0.477915767	0.03244	-0.440532684	0.04458	-0.33926723	0.12219
KIF18A	-0.288967879	0.03247	0.029525774	0.82466	-0.369897821	0.0062
MTMR9	0.256471796	0.03248	0.110365655	0.35679	0.079656455	0.50795
PCDH18	0.464897778	0.03252	0.381019866	0.07969	-0.076085338	0.72651
VASN	0.638546056	0.03252	-0.208263127	0.49164	-0.422577199	0.16726
ZXDC	0.214998619	0.03253	-0.118033582	0.24429	0.231046882	0.02101
BOK	0.575632554	0.03256	0.113241625	0.67529	-0.084827534	0.75509
ACTR2	0.156851566	0.03259	0.295017062	5.49E-05	0.211129954	0.00396
ELL2	0.420198962	0.03259	0.083679079	0.67016	0.448887193	0.02232
NSFL1C	0.203086876	0.03258	0.357295558	0.00016	0.369695486	9.62E-05
SYNDIG1	1.24284013	0.03259	0.542395037	0.36168	0.575041312	0.34678
GINM1	-0.20455235	0.03261	-0.007355324	0.93748	-0.166648468	0.07972
ACPP	0.518396166	0.03263	0.249428659	0.30394	0.683350218	0.00472
GRB10	0.482366494	0.03266	0.182896943	0.41827	0.163195585	0.47072
GRAMD1C	-0.566072913	0.03269	-0.500659511	0.04857	0.153674835	0.54452
SLC22A18	0.682008162	0.0327	-0.32412451	0.31471	0.426042076	0.18256
PDPK1	0.292743248	0.03272	0.374108399	0.00629	0.459230522	0.00079
DDX47	-0.192048376	0.03276	0.097741116	0.27055	-0.144382424	0.1072
LRRC4B	0.851322057	0.03276	-0.400154979	0.32231	0.212658382	0.59799
TXNL4A	-0.238140129	0.03275	0.163203957	0.13806	-0.064212206	0.5626
SLC25A13	-0.270406846	0.03278	0.034449535	0.78431	-0.023746942	0.85076
FAHD2B	-0.565060924	0.03283	-0.393894494	0.12936	-0.232994138	0.37055
SERPINE1	1.138515117	0.03283	0.952231834	0.07426	-0.04055446	0.93974
TM4SF1	-0.39957549	0.03284	-0.14057488	0.45257	-0.412692847	0.02751
ATG9B	1.049784956	0.03288	1.404791022	0.00443	0.325777711	0.51177
RECQL5	0.298412567	0.03289	-0.370148935	0.00892	0.24031833	0.08442
TNFRSF12A	0.466846876	0.03291	0.261607927	0.23187	-0.026001328	0.90588

ARRDC3	0.672337292	0.03299	0.734631667	0.0198	0.517094674	0.10103
SENP2	-0.334786615	0.03299	0.271523154	0.08251	-0.068998173	0.65991
TCF12	0.296343206	0.03301	0.018964774	0.89139	0.068196855	0.62386
FAM73A	-0.251755999	0.03305	0.070923712	0.54188	-0.074285759	0.52492
STK11IP	0.324785285	0.03305	-0.324959131	0.03528	0.0572171	0.70846
DPM1	-0.182101836	0.03312	0.243880129	0.00318	-0.094933977	0.26249
PCYOX1L	-0.329282517	0.03314	-0.015134777	0.92069	-0.308871765	0.0452
P11-423H2	-0.332321456	0.03313	0.126520194	0.39822	0.152896257	0.31042
TUBA1C	0.331718225	0.03313	0.665157438	1.91E-05	0.32285815	0.0381
CAPRIN1	0.14940367	0.03318	0.293941825	2.66E-05	0.158189775	0.02401
IRF7	0.737200753	0.0332	0.335846771	0.3337	0.581408325	0.09351
NCKIPSD	0.406026829	0.03321	0.06635364	0.7286	0.194084338	0.31
ADAM28	-0.431919966	0.03326	-0.339123573	0.09443	0.254942212	0.20836
KIAA0825	-0.395760132	0.03325	-0.393688072	0.03317	-0.317386444	0.08613
P11-551L14	1.043491794	0.03329	0.829552202	0.09113	-0.117848491	0.81165
T6GALNAC	-0.38656903	0.03329	-0.533965399	0.0032	-0.038402522	0.83194
COBL	-0.488676094	0.03331	-0.426408046	0.06254	-0.267468335	0.24262
CNKSR2	1.436616351	0.03334	0.425001053	0.52979	0.922470388	0.18689
SSXP10	-0.552693535	0.03339	-0.708963311	0.00633	-0.012357934	0.96176
FDFT1	0.313555762	0.03341	0.117856129	0.42397	0.430275788	0.0035
DNAJC25	-0.331549303	0.03343	0.172894766	0.25551	-0.126141365	0.41435
YS1-DBNDI	2.855572198	0.03345	2.97435904	0.02683	2.665433756	0.04886
INPPL1	0.25760045	0.03346	0.023482869	0.84658	0.145518292	0.23036
DNAJC2	-0.241618738	0.03347	0.251376409	0.02477	-0.021585134	0.84838
CREB3	0.217399935	0.03353	0.179116464	0.07808	0.081680373	0.42574
INSL6	0.987939572	0.03355	0.09782019	0.84004	0.728389083	0.1212
LTBP3	0.498719409	0.03354	-0.382714612	0.10514	-0.15658984	0.50715
VRK3	0.256050007	0.03355	-0.126368831	0.29756	0.324557802	0.00691
ZC3H4	0.374035758	0.03354	-0.301805111	0.08728	0.23553715	0.18129
ADRA2C	-0.893056118	0.03364	-1.191981635	0.00485	-0.328462402	0.42774
ZMYND8	0.37023926	0.03365	0.187688842	0.28162	0.356258353	0.04101
ELP3	-0.290072725	0.03366	0.156124656	0.2486	-0.058618107	0.66684
DMWD	0.301931668	0.03368	-0.380402106	0.00878	-0.002942114	0.98366
OST4	0.274338435	0.03371	0.498775091	0.00011	0.276097627	0.03248
MRPS34	-0.326487441	0.03373	0.091483312	0.54489	0.056156132	0.71147
ZNF239	-0.386985114	0.03373	0.098092511	0.57963	-0.18418399	0.30549
RIOK2	-0.240145832	0.03374	0.135109767	0.22273	-0.13772872	0.21989
APOBEC3A	1.565386932	0.03378	1.174598552	0.11416	1.116887176	0.13655
PALM3	-0.691211544	0.03379	-1.038592902	0.00158	-0.015818851	0.95984
SYT11	0.598331734	0.03378	0.016337534	0.95413	-0.28778692	0.31653
CCDC169	-0.567580326	0.03381	-0.592102235	0.02723	-0.486466017	0.06819
FOXG1	2.302096281	0.03388	1.682449656	0.12269	1.738321127	0.11113
PCMTD1	-0.232155381	0.0339	-0.126795626	0.24367	-0.049802488	0.64777
VIL1	-0.535612708	0.03395	-0.432781932	0.08647	0.028927403	0.90876
TCEAL8	-0.178967447	0.03407	0.359723778	1.47E-05	-0.102732861	0.22201
ERCC4	-0.369519562	0.03411	-0.163769531	0.34547	-0.326070799	0.06193
CTSH	-0.423799834	0.03414	0.107223587	0.59089	-0.232145045	0.24527

RBM22P2	0.960321298	0.03418	-0.46949818	0.33301	-0.367934121	0.44922
TAS2R14	-0.682483921	0.03425	-0.529818477	0.09317	-0.419770306	0.18446
HSD11B2	-0.838291707	0.03426	-1.011174032	0.01004	-0.528056698	0.17208
P11-113I24	-0.497177373	0.03429	-0.40824217	0.07497	-0.276125047	0.22852
APRT	-0.344831634	0.03432	-0.10116988	0.53172	0.036482746	0.82151
POLDIP3	0.173741034	0.03433	-0.102275775	0.213	0.108589655	0.18567
CCDC88C	-0.429141826	0.03441	-0.292693301	0.14856	-0.14796784	0.46468
CDIP1	0.502153963	0.03441	0.479030974	0.0427	0.433744021	0.06779
GALNT6	-0.654819384	0.03441	-0.493690301	0.11007	-0.322292423	0.2967
PTMS	0.356903604	0.03439	-0.154420575	0.36136	-0.152551669	0.36761
TMEM9	-0.21322697	0.03447	-0.237493604	0.01789	-0.178897012	0.07564
PDCL	-0.242489697	0.03448	-0.176049391	0.11889	-0.258905456	0.02356
SLC45A3	-0.500391042	0.03454	-0.624641263	0.00826	0.04710796	0.84015
SEC23B	0.27579906	0.03459	0.382016537	0.00331	0.212205595	0.10363
MPRIP	0.281524708	0.03462	-0.079841031	0.54934	0.206500585	0.12145
PRKAB2	-0.357272386	0.03468	-0.252112892	0.13464	-0.080684196	0.63218
MAPK6	0.179245881	0.03469	0.318836363	0.00016	0.11396323	0.17926
GNAS	0.165880727	0.03473	0.040827325	0.60322	0.023220793	0.76762
IL7R	0.64113924	0.03474	0.166279382	0.58395	0.13408455	0.66448
ITGA2	0.592737368	0.03472	0.604576518	0.03121	0.081469965	0.77183
SYTL2	-0.470965432	0.03474	-0.499800316	0.02493	0.03296928	0.88228
TACC2	0.419200909	0.0348	-0.048887597	0.80575	0.172459408	0.38597
KLRF2	-1.274043134	0.03483	-0.503159306	0.38131	-0.28635528	0.61729
KIAA1143	-0.337101072	0.03484	0.067980921	0.66785	-0.104500483	0.512
KDM1A	-0.157019322	0.03486	0.121262412	0.10045	-0.162325101	0.02897
NDE1	0.303063737	0.03495	0.033117451	0.81773	0.016157895	0.91076
NSMAF	-0.243128242	0.03498	0.039969261	0.72497	-0.180683244	0.1152
GUCY1A3	0.751751127	0.03499	0.323003166	0.36522	-0.030432321	0.93216
CHPT1	-0.440791023	0.035	0.125549892	0.54497	-0.646768941	0.00205
ASAH2	0.801589877	0.03503	0.256897625	0.49882	0.250320896	0.50929
PIGR	-1.940608163	0.03511	-0.805241855	0.38165	-1.654027912	0.07249
SCMH1	0.262199151	0.03513	-0.093253081	0.45453	0.192663947	0.12129
SLC8A3	0.720805548	0.03518	0.572395741	0.09437	0.315534877	0.36058
KDM5C	0.17195814	0.03523	-0.280398484	0.00062	0.121221059	0.13808
RAB3C	-1.433580848	0.03522	0.025166001	0.97	-1.437101482	0.03311
RPS3AP44	-0.878834093	0.03521	0.195269537	0.60542	-0.252227965	0.52058
ZNF621	0.246218443	0.03522	0.092387495	0.42833	-0.004612072	0.96864
NISCH	0.293465539	0.03525	-0.318191274	0.02308	-0.163907891	0.24113
KIF13A	-0.264633218	0.03527	-0.346423522	0.00571	-0.064109586	0.60857
PBXIP1	-0.358231394	0.03529	-0.226902612	0.1807	-0.425220124	0.0125
PCP4L1	1.254249431	0.03528	0.837768886	0.16346	0.494942246	0.42123
KMT2A	0.358477582	0.03532	-0.2374152	0.16381	0.260223881	0.12657
C12orf68	0.967935817	0.03535	0.431534451	0.35374	-0.0746909	0.87613
SEL1L3	-0.392337101	0.03537	-0.273084322	0.14246	-0.068151098	0.71433
TMUB1	0.376187757	0.03536	-0.084146229	0.63999	0.409496129	0.02184
ASIC2	-1.187156574	0.03539	-1.16954651	0.03702	-1.024338517	0.06476
SYNE2	-0.404550738	0.03539	-0.358368496	0.06216	-0.25528904	0.1841

IDO2	0.743184169	0.03544	0.03259537	0.9297	0.400575136	0.26807
TYW5	-0.196216376	0.03545	-0.178069709	0.056	-0.115566863	0.21313
CTC1	0.412019819	0.03547	-0.326905584	0.09599	0.293733956	0.1329
EXOSC7	-0.302658051	0.03551	-0.13968393	0.32566	-0.178150315	0.21298
PAICS	-0.290384975	0.03558	0.150298778	0.27585	-0.132477866	0.33732
CDC25B	0.476519917	0.03563	0.144812117	0.52344	0.255540304	0.2607
YPEL5	0.256116058	0.03563	0.268315806	0.02697	0.149702062	0.22039
RILPL2	0.334924216	0.03565	-0.055414242	0.73001	0.095677506	0.55109
CDKN2B	0.733720187	0.03569	0.334524868	0.33836	0.350296793	0.31646
COQ5	-0.205577405	0.03573	-0.110210231	0.24388	-0.080564044	0.40103
TOM1L1	-0.332947768	0.03574	-0.034742407	0.8249	0.057659052	0.71381
RNASE1	-0.703562932	0.0358	0.407893468	0.2223	0.032272155	0.92314
CCDC43	-0.238129908	0.03585	0.171008702	0.12459	-0.171447028	0.12874
SCARF1	0.734730805	0.03594	0.052353898	0.88565	0.684245966	0.05295
ANXA1	0.611817696	0.03598	0.436265846	0.13481	0.274712848	0.34652
ZNF451	-0.243481254	0.036	-0.010659509	0.92663	-0.180397299	0.11989
TSTD1	-0.346195429	0.03601	0.13902563	0.38093	0.225299369	0.15716
NR5A2	0.521170017	0.03608	0.66510817	0.00737	0.841668831	0.0007
DDB2	-0.536056894	0.03609	0.125177107	0.62087	-0.223818099	0.38029
NR4A3	1.768552411	0.03617	1.013766219	0.23458	0.228056694	0.79294
PNRC2	-0.146769246	0.03618	0.193885824	0.00534	0.019525025	0.78002
CNN2P1	0.901058935	0.03621	0.001237702	0.99783	0.044750587	0.92249
UPK1B	-0.530575267	0.03624	-0.758624867	0.00276	-0.119893277	0.63591
BLM	-0.465904302	0.03626	-0.022710246	0.91831	-0.433904845	0.05106
TATDN3	-0.274591652	0.03626	0.084444798	0.49767	-0.123728373	0.33681
BPIFB1	-1.36242821	0.03631	-1.350072351	0.03797	-0.769658302	0.23664
SLC2A3	0.613260816	0.03641	0.510621292	0.08144	-0.462127388	0.11535
LNPEP	-0.178479344	0.03644	0.00053894	0.99494	-0.047631023	0.57544
SEPSECS	-0.364139786	0.03644	-0.267596969	0.11925	-0.201836142	0.24221
METAP1D	-0.548396989	0.03654	-0.139481123	0.58338	-0.112981591	0.65836
CHCHD7	-0.251822532	0.03657	0.067273882	0.57108	0.128528787	0.28105
ABCG2	1.064381895	0.03664	0.510326752	0.31827	0.726022865	0.1528
MTRNR2L1	-0.561685876	0.03665	0.72327325	0.00687	-0.435908788	0.10458
SLC17A7	0.894995684	0.03666	0.127670659	0.77392	0.64778268	0.13442
YBX2	0.797974491	0.03666	0.22157495	0.56908	-0.298047149	0.4516
LYPLA2	0.206035833	0.03682	0.099206541	0.31283	0.082578153	0.40316
PLEC	0.445008881	0.03684	-0.705594184	0.00095	-0.024751357	0.90762
TMEM200C	0.781305676	0.03689	0.441103047	0.23839	-0.254664493	0.50411
PPP1R13B	0.336846215	0.03692	-0.521823508	0.00136	0.045193154	0.7797
PKP3	-0.335018701	0.03698	-0.286003833	0.07481	-0.037967628	0.81258
GTPBP10	-0.283864826	0.03709	0.104449262	0.43561	-0.208890103	0.12185
CD320	-0.446948153	0.03713	0.052489327	0.80448	-0.386014954	0.07097
ZDHHC9	0.247791429	0.03717	0.176997734	0.13637	0.208922766	0.07906
TBL1X	0.257619635	0.03719	-0.093768669	0.44893	0.218374573	0.07687
NUDT8	-0.579918507	0.03724	-0.925639191	0.00101	0.029523464	0.91161
RHOC	0.273371954	0.03723	0.125438654	0.33913	0.232050793	0.07699
HIST1H1E	-0.341994963	0.03725	-0.036133509	0.82556	-0.17169661	0.29533

HDAC8	-0.420545266	0.03726	-0.112383361	0.57252	-0.315329515	0.11667
FOXK1	0.33219026	0.03733	-0.257394925	0.10759	-0.11672666	0.46535
DTX2P1	0.560779024	0.03737	-0.026747983	0.92232	0.629160517	0.01897
LCT	1.811890433	0.0374	2.069098269	0.01687	0.770312268	0.3866
HEPACAM	-0.924226817	0.03741	0.141762175	0.73617	-0.209791004	0.62381
VDAC1	-0.186441082	0.03742	0.213643695	0.01678	0.05781716	0.51805
FUBP1	-0.160420857	0.03749	0.076503883	0.31852	-0.073278271	0.3411
GDI2	-0.158361605	0.03749	0.296045855	9.43E-05	-0.013458262	0.85945
PHLDB1	0.451842214	0.03751	-0.16954553	0.43621	-0.106488723	0.62503
MTND4P23	1.373261372	0.03763	1.68439674	0.00852	2.083443682	0.00097
NAB2	0.444392799	0.03769	0.012848417	0.95217	-0.038656348	0.85703
SEZ6	1.214135458	0.03769	-0.011403862	0.98492	1.036098419	0.07502
ABCB7	-0.345298774	0.0377	0.30364617	0.06279	-0.086174738	0.60104
TMEM200E	0.699308002	0.03777	0.456907906	0.17523	0.132449116	0.69753
RAVER2	-0.227485993	0.03781	0.262492998	0.01505	0.167576618	0.12227
EIF3H	-0.395696161	0.03788	-0.051211984	0.78688	-0.108313899	0.56766
C15orf57	0.199370628	0.03792	0.2832734	0.0029	0.144490089	0.13358
CCDC174	-0.287635006	0.03791	-0.239286345	0.08142	0.167988936	0.22048
MFN1	-0.209070825	0.03792	-0.036974873	0.71138	0.063236098	0.52839
SLC15A2	-0.756284405	0.03791	-0.427831726	0.23646	-0.071686466	0.84102
SNAPC4	0.342992759	0.03796	-0.123932258	0.4595	0.229384916	0.16642
PSMD12	0.227596348	0.03799	0.456174817	2.88E-05	0.116201654	0.28924
NCAPG	-0.386070323	0.038	0.177920832	0.33663	-0.301384875	0.10488
BTF3L4P2	-0.339716613	0.03803	0.124443232	0.43319	-0.128340723	0.42687
POC1A	-0.395369941	0.03803	-0.148673754	0.42558	-0.026024671	0.88925
CDKN1C	-0.392745717	0.03807	-0.714380236	0.00017	-0.833958898	1.25E-05
KCNK5	-0.502927609	0.03806	-0.065376267	0.78652	0.151854907	0.52912
PPA1	-0.228329776	0.03805	0.298478814	0.00635	0.015173472	0.89003
SNRPG	-0.249034747	0.03812	0.376485503	0.00149	-0.017471855	0.88369
VMAC	-0.440837201	0.03811	-0.719794993	0.0008	-0.357946695	0.08847
ANKK1	1.513706537	0.03817	1.068769406	0.14026	1.201433317	0.09533
P11-632K2(	-0.428239664	0.03817	-0.032124991	0.87619	-0.355221951	0.08518
DOCK5	0.465904919	0.03819	0.130157525	0.56273	0.687969218	0.0022
MOB2	1.019672165	0.03819	0.593345981	0.2304	0.866097535	0.07861
HEATR5A	0.355525686	0.03821	0.603638652	0.00041	0.688479281	5.81E-05
KLHL21	-0.384549235	0.03829	-0.592625444	0.00143	-0.205819806	0.26903
AGTR1	-0.686676276	0.0383	-0.308544413	0.35178	-0.351032731	0.28948
CAP2	0.584851729	0.03831	0.940563131	0.00072	-0.330177528	0.26468
ANKRD37	-1.046962644	0.03838	-1.551806333	0.00215	-0.825397395	0.10199
FAM163A	0.980655953	0.03839	0.256752402	0.59801	-0.435052288	0.40148
SLC7A7	0.528117517	0.03838	0.508146401	0.04595	0.217225042	0.39542
APITD1	-0.363104988	0.03841	0.138005365	0.41066	-0.201689943	0.24324
C20orf27	-0.364699713	0.03845	-0.204146137	0.23939	-0.248545116	0.15626
GCSHP5	-0.934503932	0.03852	0.086550879	0.84186	-0.01056231	0.98069
LCORL	-0.294845607	0.03851	-0.138270207	0.32606	-0.125395892	0.37653
TSNARE1	0.433919678	0.03852	-0.275515892	0.19475	0.243066566	0.24024
CPHL1P	0.694806976	0.0386	0.953925623	0.0044	1.146657112	0.00059

LPP	0.619130966	0.03868	0.048019206	0.87305	0.302075047	0.3141
C2CD2L	0.478711262	0.03877	-0.096601304	0.68032	0.268449794	0.24818
CXCL17	-1.297611816	0.03877	-0.610531479	0.32594	0.141071915	0.81965
SUCLG1	-0.278555386	0.03876	0.092695732	0.48776	-0.066666828	0.61916
ZNF205	-0.7188951	0.03876	-0.387812455	0.26145	-0.129066973	0.70771
ATP11A	0.4014912	0.03879	-0.135635188	0.48544	0.336149757	0.0837
RHPN1	-0.551818557	0.0388	-0.751793445	0.00483	-0.001390572	0.99579
NPC1	0.252009442	0.03881	0.094065304	0.44016	0.124404409	0.30817
APOL2	0.567701273	0.03882	0.204929349	0.45689	0.363599918	0.18641
APEX2	0.240976768	0.03886	0.341560381	0.00298	0.207840386	0.07465
MRPS35	-0.274361536	0.03886	0.143754078	0.27548	0.018560795	0.88837
EVI5L	0.44096746	0.03893	0.221649759	0.30312	0.371880003	0.08208
PRKAG1	-0.171791315	0.03897	0.172386025	0.0341	-0.062978664	0.44531
NDUFAF4	-0.278784725	0.039	0.049971808	0.70603	-0.186514944	0.16491
NRARP	0.710515916	0.03899	0.381391243	0.27043	0.955965976	0.00503
ACSF2	-0.25686606	0.03905	-0.589762675	1.89E-06	0.001231443	0.99204
CEP128	-0.45998018	0.03904	-0.30761456	0.16392	-0.554706791	0.01279
TPTE2P6	0.8355159	0.03904	-0.770461852	0.09788	0.365402663	0.38106
CEP192	-0.206165744	0.03907	0.169158562	0.08704	-0.020602622	0.83595
VKORC1L1	-0.262311886	0.03908	0.180194198	0.15415	0.054762222	0.66512
RRS1	-0.424390632	0.03911	-0.01134059	0.95523	-0.195474185	0.33753
KLRK1	0.671845046	0.03912	0.291678412	0.37822	0.666583796	0.04078
MAPT	0.98496158	0.03921	-0.493270138	0.32771	1.003401469	0.03426
SNX15	0.384600831	0.03923	-0.253116062	0.17938	0.007776607	0.96697
KITLG	-0.380045129	0.03927	-0.00724303	0.96857	-0.521966987	0.00465
FANCG	-0.403990098	0.0393	-0.441321803	0.0234	-0.523399006	0.00738
ATP6V0E1P	1.17882517	0.03939	-0.987900814	0.15627	-0.019280548	0.97545
TC-479C5.:	0.566872156	0.03939	-0.045357898	0.87286	0.292201285	0.28872
FUZ	-0.47110288	0.03939	-0.433808384	0.05411	-0.497150974	0.02829
GSTM3	-0.288897165	0.03938	0.470534238	0.00072	-0.135293427	0.33353
RNF166	0.54375438	0.03937	0.332824941	0.2048	0.325137184	0.22162
CRAT	0.340748094	0.03941	-0.190549192	0.25275	0.307924856	0.0635
LGALS3BP	-0.331468295	0.03942	-0.404461309	0.01194	-0.089819051	0.5766
HOXB8	1.127575876	0.03947	0.364282845	0.51171	0.131202919	0.81794
LAMC3	1.176313254	0.03948	-0.479945402	0.40675	-0.193748599	0.73696
CYP11A1	-1.542803279	0.03959	-0.276163406	0.68219	-0.413655429	0.54899
KCTD19	-1.570765556	0.03962	-1.360021486	0.07345	-0.898557778	0.22312
MKKS	-0.279673075	0.03967	0.174935764	0.19289	0.003366912	0.98014
ADAM12	1.422348922	0.03974	1.216077267	0.07864	0.256976929	0.71048
ARHGAP39	0.553872741	0.03973	-0.103601048	0.70497	0.227377917	0.40228
DDX55	-0.262271576	0.03973	-0.155820818	0.21468	-0.294876156	0.02031
TWF1	-0.19583488	0.03972	0.234928196	0.01328	-0.005921354	0.95031
TUBGCP6	0.316754662	0.03976	-0.362398269	0.01964	0.0833893	0.58919
AGTPBP1	-0.301027822	0.03981	0.052901101	0.71556	-0.247784022	0.0894
DPP10	0.499273671	0.0398	0.309942264	0.20169	-0.394635418	0.10639
SGOL2	-0.292500948	0.03981	-0.084986756	0.54527	-0.314049151	0.02684
FAM117A	0.530337586	0.03987	-0.140179644	0.58931	0.564570355	0.02846

CLTA	0.142768765	0.03989	0.35864326	1.85E-07	0.221255048	0.0014
POLM	0.324832479	0.03989	-0.196980768	0.2178	0.157297864	0.3191
SRM	0.337678393	0.0399	0.23780883	0.14687	0.020606233	0.90074
ASB6	0.219300322	0.03992	-0.012733604	0.90536	0.128876523	0.22708
FLOT2	-0.312612826	0.03998	-0.352648973	0.02028	-0.165362552	0.27637
LDHAL6B	0.813030563	0.03999	-0.279595945	0.51429	0.498723663	0.21667
UCK2	-0.299555622	0.04	0.184835986	0.20013	-0.204592972	0.15888
VPS41	-0.230722703	0.04	-0.100243665	0.36903	-0.095917529	0.39197
FANCD2	-0.403595804	0.04004	-0.253985291	0.1949	-0.27209086	0.16579
SKI	0.638717638	0.04006	0.150975499	0.62819	0.283599881	0.36282
DACT1	0.746817336	0.04009	-0.370437024	0.31053	-0.072926404	0.8418
C17orf89	-0.404502843	0.04013	0.230709969	0.22773	-0.151255422	0.43729
PIGL	0.315096173	0.04013	-0.449201921	0.00382	-0.016858205	0.91306
PLXNB1	0.256973224	0.04012	-0.331610507	0.00825	0.055784269	0.65603
CSRP2	0.382196605	0.04019	0.530374612	0.0043	-0.226421985	0.22779
MAN1B1	-0.343803775	0.04019	-0.329182995	0.04883	-0.26949199	0.1072
IRGQ	0.185376839	0.04024	0.021929223	0.80858	0.05750393	0.52607
NSMCE1	-0.283073757	0.04025	-0.090373944	0.49926	-0.048282645	0.72003
PLA2G4B	0.962685111	0.04031	0.415847483	0.3774	0.658856099	0.15758
EVA1A	0.747447253	0.04034	0.578053565	0.11201	-0.073831778	0.84105
PRKCA	0.306360323	0.04038	0.138794037	0.35286	0.303803699	0.04201
P11-61N20	-0.748987216	0.04043	0.507842313	0.13128	-0.185163269	0.59679
CMIP	0.976660255	0.04046	-0.063795443	0.89407	0.620136108	0.1939
EHD2	0.537049837	0.04047	-0.411381375	0.12177	-0.170584639	0.52044
PLEKHF1	0.681200713	0.04052	0.2589953	0.43698	0.293301425	0.38873
METTL18	-0.296124242	0.04054	0.224098138	0.10003	-0.154461548	0.27656
CPOX	-0.309214712	0.04062	-0.096564987	0.51725	-0.11935963	0.42554
PDIA4	-0.254345675	0.04063	-0.083696444	0.50021	-0.05960378	0.63122
MCHR1	1.60937449	0.04065	1.161127423	0.14083	0.959514664	0.22866
TTC7B	0.59702214	0.0407	0.022586215	0.93846	0.209103947	0.47546
LRP5	-0.310449765	0.04076	-0.549905008	0.00029	-0.10659236	0.48163
KIAA1755	1.372071369	0.04085	0.703223246	0.29675	1.243670611	0.06459
MFAP2	0.377351852	0.04085	0.218881253	0.23566	-0.079704286	0.66659
CYP27A1	0.953506167	0.0409	0.998779222	0.03006	0.636991166	0.17661
IRX2	-0.83264813	0.04092	-0.239666686	0.55115	-0.506738232	0.21022
NTRK3	0.917584228	0.04089	0.641072084	0.15195	0.180833221	0.68743
PHYH	-0.323495659	0.04088	0.027043769	0.86066	0.158400154	0.30487
RPS4XP5	-1.222906482	0.04091	-0.104168825	0.84528	-0.66686702	0.23213
CCDC183	-0.483845309	0.04095	-0.913508719	0.00012	-0.122139697	0.60428
SMIM4	-0.544646706	0.04098	-0.699462827	0.00805	-0.219909238	0.39942
ARHGEF10	0.361280471	0.04105	-0.09798037	0.58064	0.379218524	0.03201
FMNL3	0.54527505	0.04107	-0.207912591	0.43778	-0.188613877	0.48212
ACIN1	0.145812165	0.04118	-0.242201848	0.00071	0.03485928	0.62558
AIM1L	0.524128708	0.04122	-0.120666008	0.64101	-0.083059715	0.74756
APOL1	0.810032957	0.04121	-0.270463972	0.49761	0.745747547	0.06017
CDX2	0.77424004	0.04124	0.433891689	0.25283	0.642480449	0.09036
FAM115B	0.554715387	0.0412	-0.077013116	0.77983	-0.265749622	0.34103

MAB21L2	0.511230707	0.04122	0.348018533	0.1646	0.06897805	0.78324
MRPL9	0.255881854	0.04114	0.436057531	0.00043	0.211369229	0.0921
PAN2	-0.372723045	0.04114	-0.529468198	0.0037	-0.054860195	0.76315
RAI1	0.383344215	0.04118	-0.306936217	0.1037	0.17349654	0.35624
RASA4CP	0.655726029	0.04119	-0.438364913	0.18377	0.568661673	0.07745
SEL1L	-0.281278004	0.04118	-0.061538446	0.65479	-0.062043994	0.65236
SNX19P3	-1.084614365	0.04112	-0.519809818	0.29806	-0.336871348	0.49876
SNX25	0.561244053	0.04114	0.234336666	0.39409	0.105988019	0.70029
TIRAP	0.431923069	0.04124	-0.225916701	0.29291	0.389091712	0.06503
ZNF815P	-0.970830719	0.04127	0.011684756	0.97738	-0.19710711	0.65167
TRMT61B	-0.461865465	0.04131	0.224748787	0.309	-0.318071652	0.15505
CYHR1	-0.240266079	0.04145	-0.448395839	0.00016	-0.226975659	0.05361
ZDHHC14	0.390549741	0.04144	0.446752665	0.01767	0.419157489	0.02652
ARHGDIA	0.373806056	0.04146	0.064963613	0.72383	0.201323946	0.27264
MST1R	0.491630371	0.0415	-0.203562864	0.40432	0.836555549	0.00052
GATAD2B	0.400440814	0.04153	-0.060418758	0.75956	0.357675072	0.06866
PABPC5	0.628450916	0.04156	0.25088548	0.41713	-0.254805112	0.41606
TUBGCP3	-0.335607269	0.04164	-0.145980259	0.37086	-0.227739926	0.16437
CDHR5	1.241722792	0.04166	1.135397191	0.06254	0.534601421	0.38091
CTSC	0.329703736	0.0417	0.552426173	0.00064	0.046915143	0.77211
RAB27B	-0.317518822	0.04171	-0.126229906	0.41782	-0.142172025	0.36154
SLC39A4	1.052640147	0.04172	1.303665169	0.01172	1.26106026	0.01473
AGPAT5	-0.303240427	0.04176	0.193341415	0.1915	0.04232932	0.77541
CDNF	-0.981245657	0.04177	-0.35682399	0.40904	-1.193588894	0.00967
GPATCH11	-0.248339364	0.04179	0.137500635	0.24263	-0.015144262	0.89917
ZNF845	-0.375717326	0.04179	0.253985404	0.1641	-0.054103691	0.76804
IVD	-0.389103246	0.04181	-0.307095963	0.1057	-0.368608812	0.05304
MED27	0.221300792	0.04187	0.382680616	0.00031	0.032210256	0.77013
NCOA5	0.289478432	0.04197	0.151380895	0.28706	0.142223747	0.31857
RHOQP1	-0.918578097	0.04202	0.040948675	0.91825	0.803970902	0.03532
CRMP1	0.712941475	0.04203	-0.185700995	0.5983	-0.332816364	0.34645
FCF1P8	1.391504224	0.04211	-0.317413878	0.68086	1.108545973	0.10985
EMILIN2	0.554832497	0.04222	0.447465312	0.10256	0.245429495	0.37375
GRIN2D	1.115194763	0.0422	0.117564223	0.83749	0.023553686	0.96775
POLR2D	-0.251721945	0.04221	0.039015392	0.75067	-0.170048063	0.16896
ADAM17	0.220080978	0.04226	0.264968521	0.01414	0.208895452	0.0537
TMEM132C	-2.031300548	0.04226	-2.179688554	0.02897	-2.508172591	0.01474
EXTL3	0.310285603	0.04229	-0.214955735	0.16021	0.002857493	0.98513
SLC7A6	0.523854076	0.04232	-0.204531984	0.42871	-0.096567035	0.71002
COX14	-0.319603686	0.0424	-0.245211502	0.11177	-0.233230236	0.13448
ID4	0.534897195	0.04244	0.246657696	0.34993	0.408926372	0.12115
ALDH1B1	-0.307116496	0.04247	0.253785243	0.09091	-0.132081385	0.3815
SLC6A6	-1.746975591	0.04248	-1.104524073	0.18842	-1.557918044	0.06756
CERS1	0.740863215	0.04252	0.062979328	0.86497	-0.024466467	0.94804
KCNIP4	-0.493717611	0.04251	-0.154560857	0.50707	-0.236099892	0.32761
SLFN12	0.512037618	0.0425	0.566443057	0.0242	0.00557334	0.98254
ZWILCH	-0.359912867	0.04258	-0.106748197	0.54363	-0.334308743	0.05851

LATS1	0.205055686	0.04262	-0.107976694	0.28598	0.173503492	0.08554
ALKBH6	0.377841334	0.04267	0.036374006	0.84727	0.264545431	0.15613
STRA13	-0.336936555	0.04279	0.145851475	0.36971	0.008631709	0.95802
TMEM41B	-0.19131726	0.0428	-0.190344966	0.04288	-0.046800383	0.61877
COLEC12	0.51748957	0.04286	0.150913883	0.55526	0.067103912	0.79334
MLIP	1.125828276	0.04284	0.101572934	0.86208	1.110953193	0.04671
RBM41	-0.340361183	0.04286	-0.153774602	0.35672	-0.036441183	0.82782
SCAPER	-0.316812299	0.04286	-0.087335699	0.57387	-0.091733342	0.55554
CKMT1B	-0.435942152	0.04289	-0.35277303	0.0991	0.087437333	0.68111
CFL1	0.177621652	0.04292	0.252230495	0.00401	0.221361437	0.01161
CSAD	0.458156107	0.04298	-0.446091182	0.05413	-0.176035395	0.4438
GSG2	-0.425131041	0.04298	-0.155579845	0.45058	-0.201227659	0.33248
MPND	-0.410532449	0.04297	-0.325682734	0.10776	-0.419331355	0.03789
ESCO1	0.193619678	0.04302	-0.088616616	0.35027	0.160048316	0.09308
SHROOM4	0.36978358	0.04303	0.044112091	0.81063	-0.095374359	0.60468
STX5	0.290679384	0.04304	-0.05233466	0.71665	0.003570372	0.98032
ARF6	-0.18767977	0.04312	-0.153670789	0.09665	-0.076358374	0.40949
15-Sep	-0.161128576	0.04315	0.164096618	0.03761	0.011922947	0.88055
P11-159D12	-0.455277245	0.04316	-0.108508498	0.62195	-0.489624635	0.02954
ULBP1	-0.508077066	0.04318	-0.231443007	0.33649	-0.375035121	0.12867
ZNF563	-0.530407088	0.04325	-0.136742481	0.59899	0.162974315	0.5283
ANKRD29	-0.89673124	0.04334	-0.284931774	0.51268	-0.396348822	0.36267
IL1RAPL1	0.598550458	0.04334	0.066891793	0.82494	-0.339224682	0.26898
C1GALT1	-0.312946443	0.04336	0.327662547	0.03378	-0.064182761	0.67819
CSPG4P11	0.578002738	0.04341	-0.507671517	0.08374	-0.280108623	0.33845
ICA1	-0.353171034	0.0434	-0.020418725	0.90627	-0.041255596	0.81244
DYNLL2	-0.223584775	0.04342	-0.276380178	0.01233	-0.184705178	0.09471
TTC33	-0.286961593	0.04353	-0.058606701	0.67694	-0.1677568	0.23605
ZNF564	-0.360369381	0.04353	-0.103314144	0.55246	-0.258005361	0.14462
PRDM10	0.299536807	0.04357	-0.029953394	0.83962	0.190317267	0.19884
STK25	0.16295337	0.04358	-0.054925832	0.49639	-0.018113682	0.82332
TRAPPC10	0.250147923	0.04362	-0.221091689	0.07493	-0.067904225	0.58465
11-495P10	0.813764332	0.04367	0.082540426	0.84362	-0.029204566	0.94547
PROSER2	-0.488007012	0.04372	-0.406966482	0.09073	-0.352028766	0.14484
EBF4	0.420863306	0.04381	-0.180451569	0.39567	0.013085164	0.95031
PON1	-1.234828158	0.0438	-1.234171498	0.04927	-0.216318691	0.71948
TBP	0.458859139	0.04381	0.489178908	0.03075	0.507015034	0.02548
TMEM50B	-0.288192538	0.04378	-0.192535178	0.17439	-0.030115009	0.83194
LTV1	-0.290681082	0.04385	0.323632425	0.02144	-0.107028786	0.45421
LAMB3	0.391282905	0.04392	0.105874392	0.58568	0.343137818	0.07725
SAMD11	1.52068049	0.044	0.612993753	0.41799	0.529549342	0.48498
CCDC124	0.374545539	0.04405	0.43410698	0.01881	0.319710085	0.08581
COL22A1	1.114130392	0.04406	0.644498267	0.24371	0.496046819	0.37795
YIF1B	-0.301270053	0.04406	0.007697336	0.95815	-0.322800275	0.03004
CDHR2	1.617199206	0.04411	1.304523746	0.10441	0.78922584	0.3267
ERLIN2	-0.285759058	0.04417	-0.129219557	0.36143	-0.053894331	0.70367
BNC1	1.216464577	0.04422	0.649285969	0.28364	0.073260756	0.90444

ADD1	0.190774315	0.04426	-0.043329598	0.64767	0.070296294	0.4588
CDS1	-0.300355661	0.04427	-0.118777115	0.42514	0.240532947	0.10588
GHDC	-0.448455758	0.04428	-0.533002783	0.01564	-0.240091414	0.27108
RBMS3	0.664484808	0.04426	0.603700054	0.06768	0.307112435	0.35362
BVES	0.753917597	0.04429	0.654919716	0.08047	0.357257042	0.34374
MST1L	-0.965342036	0.04431	-0.272283912	0.56394	-0.185486422	0.69504
ERO1LB	0.793360844	0.04434	0.095625944	0.80963	0.397803462	0.31399
DDIT4	-0.575218442	0.04437	-0.933763025	0.0011	-0.589218422	0.03943
TFF3	-0.562569996	0.04437	-1.0450435	0.00019	0.258118458	0.35609
GLCE	0.230908083	0.04438	0.524384751	4.27E-06	0.371782404	0.00116
JAGN1	-0.217158132	0.04446	0.166103163	0.11398	-0.059865612	0.57454
POP5	-0.330868857	0.04446	-0.102656925	0.52291	-0.191331234	0.23939
BCAP31	-0.186571942	0.04453	-0.05157449	0.57606	-0.079347134	0.39105
TERT	-1.335514417	0.04456	-1.054871238	0.10535	-0.275378814	0.67088
PRSS22	0.469928671	0.0446	-0.011478853	0.96113	0.470954668	0.04422
IL17B	1.066386173	0.04466	0.900781298	0.08905	0.057826256	0.91796
P11-415I12	2.665697168	0.04466	-0.539510884	0.69931	0.450759335	0.74162
COX7B	-0.207472364	0.04472	0.248562945	0.01537	-0.029750905	0.77286
NFATC2	0.504748526	0.04472	0.300316129	0.23158	0.67877685	0.00673
BMP2K	0.27609137	0.04481	0.140071597	0.30675	0.305309761	0.02645
AFTPH	-0.261874423	0.04486	0.043481956	0.73779	0.108551272	0.40353
NDUFV2	0.239367988	0.04488	0.666146056	2.01E-08	0.525794164	9.98E-06
PPP1R7	-0.209126062	0.04485	0.067746815	0.50813	-0.084246694	0.41578
SHANK2	-0.439494498	0.04487	-0.78534806	0.00034	-0.364326869	0.09572
SLC51B	1.347410618	0.04487	1.686468686	0.01124	0.678976439	0.32008
NR2F1	1.003285965	0.04498	0.267772082	0.59305	0.019652137	0.96878
ATF5	0.403997272	0.04504	-0.232521732	0.25674	0.511147016	0.01109
TMBIM4	-0.220066437	0.04503	0.098337605	0.36517	-0.043662897	0.68922
CCT6P1	-0.388015437	0.04515	-0.460051115	0.01683	-0.249223832	0.1936
SPTSSB	-0.816363834	0.04518	0.506566278	0.20706	0.106877883	0.79082
PPIEL	0.999951833	0.04528	0.133402314	0.80127	0.101039097	0.85137
PTGES3P1	0.870520396	0.04528	1.372207274	0.00121	1.265468369	0.00306
ZNF860	-0.506161681	0.0453	-0.172422422	0.48141	-0.1921617	0.43747
MLH1	-0.296856432	0.04531	0.122671333	0.40478	0.127083924	0.39037
CDC42	0.151392706	0.04536	0.299409586	7.34E-05	0.198873502	0.00853
PCDHB14	0.788150975	0.04536	-0.313304773	0.43391	-0.460195228	0.25611
C8orf48	-0.589819312	0.0454	-0.277524565	0.32971	-0.245177609	0.39228
CLDN1	0.534907134	0.04546	0.089374083	0.73834	0.645420046	0.01577
KLF12	0.464509259	0.04547	0.216422954	0.35093	-0.002765224	0.99051
OTUD7A	1.680708594	0.04549	0.905707614	0.27866	1.310189481	0.12209
NUMBL	0.557620309	0.04551	-0.314228099	0.26601	0.0180187	0.94893
CA14	-0.53939479	0.04552	-0.631854402	0.01808	-0.429384693	0.10773
COPS2	-0.191165741	0.04554	0.268880579	0.00459	-0.129119431	0.17609
TXLNB	-0.656541828	0.04562	-0.429983624	0.18217	-0.035258493	0.91186
CIRBP	-0.250227165	0.04569	-0.196872952	0.11534	-0.241648031	0.0537
HSD17B14	0.983604853	0.0457	1.06470531	0.02995	0.410338627	0.42316
ILDR1	0.62841596	0.04566	0.56730797	0.06875	0.107267376	0.74443

NUP160	-0.235721964	0.0457	-0.18909706	0.10662	-0.145226114	0.21707
RAPGEF1	0.293308054	0.04568	0.023600379	0.87245	0.445031297	0.0024
SLC41A3	-0.402619845	0.0457	-0.387258857	0.05346	-0.403051067	0.04571
USP24	-0.164458558	0.04579	0.015249325	0.85266	-0.013877544	0.86587
ZNF720	-0.297133398	0.04579	-0.178471449	0.2272	-0.284841217	0.0547
DNAJC16	-0.497873333	0.04583	0.04584358	0.85312	-0.084454795	0.73386
CANX	-0.210654402	0.04584	0.224867126	0.03293	-0.058088448	0.58178
AZGP1	-1.684548751	0.04589	0.58680193	0.44804	-0.815314666	0.31567
ETFA	-0.207978243	0.04589	0.086618779	0.40217	0.037092504	0.72074
C20orf112	0.330726251	0.04594	-0.200878599	0.22714	0.330061834	0.04641
GCAT	-0.509607175	0.04604	-0.043546417	0.86192	-0.404570287	0.11104
RBBP4	-0.185574601	0.04606	-0.013950046	0.88036	-0.146926578	0.11384
CDH16	1.684276265	0.04612	1.995161381	0.01789	-0.125322342	0.88881
CWC27	-0.255262299	0.04613	0.192315048	0.127	-0.150108233	0.24036
JAK2	0.451141855	0.04614	0.843067984	0.00019	0.922472687	4.34E-05
NPY2R	1.60631932	0.04618	0.506479055	0.54301	-0.542236347	0.5476
RRP15	-0.316524728	0.04626	0.053777243	0.73299	-0.050957811	0.7473
NXNL2	0.520829906	0.04629	-0.358926098	0.18859	0.289255458	0.27241
INPP5F	-0.259422067	0.04638	-0.010272789	0.93678	-0.275878753	0.03396
RNF165	0.900333896	0.04639	-0.336825875	0.45984	0.075294585	0.86911
TRIM15	1.677705016	0.04641	2.154414866	0.01004	0.610289089	0.47756
KATNA1	0.320159913	0.04647	0.29278841	0.0657	0.161474908	0.31599
STXBP3	-0.280587199	0.04652	-0.141830081	0.31127	-0.015803603	0.91023
BCKDK	0.310894912	0.04655	-0.089583216	0.56677	0.220489024	0.15921
BUB3	-0.197427539	0.04659	0.130273728	0.18551	-0.121296717	0.22049
FAS	-0.53209128	0.04657	0.283234328	0.28487	-0.339702047	0.20275
URGCP	0.277553238	0.04658	0.11785063	0.39787	0.22144932	0.11183
KIAA0754	0.342278525	0.04665	-0.110966232	0.52017	0.0866565	0.61529
AP5Z1	0.368267352	0.04667	-0.339719016	0.07123	0.194610836	0.29525
NPM1P37	-1.022826331	0.04667	-1.12531699	0.02713	-0.546124274	0.25881
GPR146	0.811029723	0.04691	0.491150306	0.23281	0.718443091	0.07782
FAM221A	-0.570418171	0.04692	-0.664245294	0.02084	-0.210160556	0.46341
PRKD2	0.315089822	0.04695	-0.297412363	0.06296	0.117129328	0.46174
MPV17	-0.307021622	0.047	-0.069293738	0.646	-0.050499025	0.73907
GPSM1	0.449213088	0.04704	-0.198721374	0.38347	-0.057406735	0.80155
C3orf58	-0.199779166	0.04706	-0.118677953	0.23593	-0.085859489	0.39193
SREK1	-0.193840809	0.0471	-0.109241625	0.26184	-0.054799278	0.57379
PGF	0.63271092	0.04711	0.580329923	0.06829	0.439730074	0.16954
TMEM165	0.234689296	0.04713	0.271138222	0.02139	0.369598096	0.00174
ABHD15	-0.370170559	0.04715	-0.42426965	0.02224	-0.002317937	0.98994
FAM180B	1.054739928	0.04717	0.627784292	0.24299	0.731466896	0.17447
PACSIN3	-0.368104858	0.04716	-0.466444312	0.01134	-0.313690527	0.08932
ZNF286A	0.240145793	0.04722	0.220735349	0.06696	0.179719877	0.13826
DENND1B	-0.558758028	0.04725	-0.233965472	0.39909	0.044321387	0.87375
ADCY5	0.64320945	0.04733	-0.329530582	0.31567	-0.500370937	0.12855
RPL19	-0.204090814	0.04744	0.291893232	0.00454	-0.07544934	0.4635
GSTCD	-0.274534044	0.04747	-0.042050163	0.7593	0.060036843	0.66304

HNRNPLP2	1.264764893	0.04749	0.479339683	0.46466	0.200944766	0.76497
P11-603K19	1.117182885	0.0475	0.23518985	0.69071	0.72441327	0.2083
SLAIN2	0.353150577	0.04751	0.41640683	0.01928	0.394433386	0.02682
TUBG1	0.245900301	0.0476	0.119603427	0.33424	0.143103646	0.24937
LARP1B	-0.254368261	0.04761	0.005994664	0.96247	-0.009352505	0.9416
IDE	-0.228137049	0.04762	0.226253295	0.04806	0.048710803	0.67151
SMEK1	-0.146224823	0.04764	-0.055590217	0.44802	-0.106391736	0.14837
LAT	0.606888965	0.0477	-0.477315851	0.14091	0.277780686	0.38173
NUP107	-0.227714608	0.04771	0.232916671	0.0409	-0.165162893	0.14957
SERPINA4	-0.315776443	0.04772	-0.26647325	0.09425	0.273996051	0.08462
TAS2R64P	-0.70561004	0.04771	-0.788554106	0.02588	-0.642719151	0.06916
COPS7B	0.295635137	0.04777	0.188761656	0.20767	0.410228612	0.00599
DDX10	-0.365048254	0.04797	0.660887802	0.00031	-0.032386978	0.86065
FGF11	0.498475811	0.04798	0.11546942	0.64712	0.338144372	0.18016
LMOD1	0.636662576	0.04808	0.450267017	0.16209	-0.011135786	0.97267
CSTB	0.447211222	0.04814	0.759600053	0.00078	0.726893953	0.00131
TSKU	0.443035552	0.04814	-0.233376611	0.29955	0.253567789	0.25824
ZNF782	-0.373868541	0.04814	-0.331992975	0.07829	-0.121157501	0.51712
ICOSLG	0.613793019	0.04827	0.439751368	0.15782	0.264839007	0.39436
FFAR4	0.766418547	0.04839	0.476373723	0.22132	0.881150139	0.02259
SUPV3L1	-0.282833361	0.04839	0.381436625	0.00706	0.136864286	0.33874
SLC44A2	-0.281597866	0.04849	-0.541509261	0.00015	0.094554978	0.50619
OMMD3-BM	0.578777328	0.04851	0.417626933	0.15339	0.116400906	0.69378
ZNF681	-0.371376097	0.04854	0.180228553	0.33448	-0.24078831	0.19964
HK1	0.264712514	0.04866	0.177229726	0.18681	0.0759335	0.57215
KCND3	0.722580093	0.04868	0.533622734	0.14543	0.151860789	0.67956
MPC1	-0.228548675	0.04867	0.086571629	0.44031	0.205576613	0.06772
ALDH1L1	0.811396481	0.04871	0.6575835	0.11509	0.674775392	0.10366
SNRPA1	0.236147605	0.04873	0.325136601	0.00608	0.193733332	0.10548
LASP1	0.19990099	0.04881	-0.267095206	0.00857	0.120990862	0.23305
PDE4D	-0.355325031	0.04881	-0.35011185	0.05193	-0.331416981	0.0664
P11-408P14	-0.673787226	0.0488	0.198632058	0.5266	0.178754008	0.57261
SULF2	0.301409775	0.0488	0.126688785	0.40763	-0.243959126	0.11138
VARS	-0.282727745	0.04879	-0.282357652	0.04898	-0.21832583	0.12825
CEACAM19	0.383750015	0.04885	0.007140511	0.97156	0.44694354	0.02071
GLI1	0.733845593	0.04886	-0.068655212	0.85435	0.114811939	0.75883
LPAR5	0.636293459	0.04886	-0.002901017	0.99295	0.803770693	0.01215
PTCH1	0.521552532	0.04885	0.015250969	0.9541	0.146585775	0.58016
AC007256.5	1.224816256	0.04896	0.622664587	0.32687	0.949637552	0.13093
CHRNB2	0.686668089	0.04895	-0.586965487	0.11926	0.035391276	0.92209
MOB3C	0.330778382	0.04895	0.144168495	0.39097	0.248257693	0.13965
BRICD5	0.417140136	0.04903	-0.432016392	0.04757	0.437234708	0.03669
NUBP1	-0.263677817	0.04911	0.000240081	0.99855	0.034376246	0.79594
LRMP	-1.190181174	0.04914	0.438251964	0.43122	-0.496167872	0.37657
HLA-DRB1	1.053857302	0.0492	1.666308532	0.00185	0.395774396	0.46062
CCDC132	-0.242564329	0.04923	0.066231181	0.58578	-0.063588558	0.60349
SERPINA1	-0.628018886	0.04924	0.24251648	0.44754	-0.71571008	0.02502

CCP110	-0.258253411	0.04927	-0.052472624	0.68673	-0.21661678	0.09861
ADAT3	1.068674346	0.04929	-1.021648573	0.10402	0.65137674	0.23839
LGALS4	1.103079056	0.04931	1.516517924	0.00687	0.566505173	0.31279
XRCC6	-0.162509716	0.04931	0.321622388	9.36E-05	-0.004614001	0.95542
SPOCD1	0.729169825	0.04935	0.197645452	0.5966	0.153422109	0.68097
KAT2A	-0.348319725	0.04942	0.022151155	0.90001	-0.217959669	0.21658
KCNH7	-1.025630944	0.04942	-0.482492913	0.34663	-0.686115538	0.18386
THTPA	-0.481124504	0.04955	-0.32954289	0.17286	-0.18640875	0.44294
TAX1BP1	0.193300363	0.04957	0.530221754	6.42E-08	0.268694517	0.0063
GNAI1	-0.264322593	0.04959	-0.017682498	0.89477	-0.304329423	0.02387
PRICKLE3	0.427555524	0.04962	0.026138756	0.9066	0.359147161	0.09928
HNRNPH3	0.322174317	0.04963	0.443947397	0.00672	0.448284192	0.00625
CCDC148	0.574512715	0.04975	0.906071811	0.00137	0.54808173	0.05985
NSUN2	-0.159221945	0.04974	-0.03781202	0.63587	0.012169755	0.87977
PLCZ1	-1.459090181	0.04975	0.854243081	0.19552	-0.526188673	0.44492
ZNF70	0.297338042	0.04975	-0.194692599	0.20345	-0.00333104	0.98261
ARL6IP4	-0.196258802	0.04979	0.054712688	0.58048	-0.127592838	0.20083
ZNF671	-0.415650543	0.04978	-0.39243182	0.06062	-0.186502373	0.37197
MAGEB10	0.793007468	0.04983	-0.22280389	0.60497	-0.763458411	0.0999
SLA2	0.761684443	0.04983	-0.220376855	0.59271	0.372009582	0.3469
C1QTNF5	0.537309754	0.04993	-0.453002197	0.11205	0.260929326	0.3448
ELP2	-0.263715561	0.04995	0.01170105	0.93025	-0.058849382	0.66105
GSKIP	0.340926852	0.04992	0.425666843	0.01405	0.195438013	0.26148
HMHA1	0.399344049	0.04992	0.108198356	0.59711	0.386720689	0.05737
N6AMT1	-0.277208218	0.04994	-0.123174278	0.37549	-0.110481763	0.42966
SAAL1	-0.299542431	0.04994	-0.06492668	0.66552	-0.321305305	0.03458
AC004945.[illegible]	-1.652787798	0.03922	-0.201189517	0.76002	-0.356993424	0.59708
AC005884.[illegible]	-1.753598529	0.03211	0.33164956	0.61112	-0.314125966	0.64743
AC006547.1	3.068367607	0.02935	2.273331593	0.1344	3.230796943	0.02051
AC007229.[illegible]	-3.93597252	0.00878	-1.729044805	0.19196	-0.141269251	0.90543
AC015818.[illegible]	-3.834618201	0.00358	-1.687775913	0.13979	0.129980109	0.89327
AC022210.[illegible]	3.45419932	0.02686	3.203928962	0.04034	1.858290655	0.26221
AC097721.[illegible]	-1.767371337	0.0334	-1.090871887	0.13529	-0.589320111	0.39951
AC104843.[illegible]	-3.813324075	0.01165	1.038903389	0.36793	-3.859946431	0.01067
ACTN2	2.069292557	0.01192	-0.48122343	0.57117	1.139633749	0.17994
AL353997.[illegible]	-2.784372542	0.01275	-2.136928206	0.04628	0.118917812	0.89429
ALOX12B	2.021679203	0.01854	2.169608603	0.01093	1.01574131	0.24661
AOC4P	-2.006255677	0.02143	-2.56452495	0.00619	-0.873200051	0.28063
AP000936.[illegible]	-2.803849859	0.04526	-1.432999341	0.25199	-2.905503106	0.03916
ARHGAP3[illegible]	-3.440833986	0.03095	0.219758977	0.86604	-0.785318623	0.57102
ARPC3P1	-1.889190485	0.00877	-0.860473702	0.15852	-1.99012554	0.00626
ATP4A	2.52279383	0.03244	1.749652947	0.13349	1.710896029	0.15779
BEND3P3	3.667569739	0.04233	2.761929712	0.13152	4.285896664	0.01676
C10orf126	3.472866219	0.01397	0.77825776	0.5975	2.000397301	0.16337
C10orf53	1.979253183	0.01906	1.820779256	0.03084	2.059124125	0.0145
C10orf99	2.770215874	0.04472	3.034665775	0.02701	1.38087785	0.34716
C12orf74	1.301542288	0.04473	-0.058778526	0.93466	1.125985265	0.08594

C19orf38	-2.132653418	0.03504	-1.412122062	0.10164	0.025422408	0.97158
C1orf195	-2.745840178	0.01345	-2.39913419	0.01894	-0.711387348	0.40618
C1QTNF9E	-1.458849488	0.01944	-0.277259225	0.59444	-0.821182603	0.14017
CALHM1	-1.987662464	0.02101	-0.199849672	0.76107	-0.2027414	0.76056
CALML3	4.296346404	0.04134	1.363379415	0.5318	2.847504116	0.18081
CASQ2	3.787022531	0.0087	3.182185089	0.02401	1.035583507	0.49909
CDH20	-1.948134694	0.0459	-0.704817951	0.44824	-2.825699086	0.00929
CITED1	2.017889338	0.0009	1.19737718	0.05173	0.266011806	0.69408
CLCA2	4.406531089	0.0025	3.069903444	0.03521	4.095689561	0.00445
CLCA4	3.12445545	0.00448	3.252513441	0.00297	2.287827898	0.04675
CLEC18C	-3.492689633	0.0197	-0.209601004	0.85481	-0.685472452	0.56561
CNFN	2.037177096	0.02373	1.164202355	0.21354	0.863911282	0.37164
CTA-85E5.6	-3.283422401	0.01908	1.464167126	0.14611	-0.989255741	0.41578
TD-2302A1(	-3.524161684	0.01018	0.629635102	0.52498	-0.650312421	0.55496
CTRB1	3.982999806	0.00901	1.260317246	0.47715	0.006174435	0.99724
CXCR2	2.190979044	0.03197	2.560314914	0.01109	1.164613682	0.27883
CYCSP39	3.466870356	0.01215	2.181442439	0.13182	3.234684188	0.01999
DHDH	1.150047688	0.04743	0.632809394	0.28804	0.881097756	0.13872
DMRT2	2.969267351	0.03476	2.936938994	0.03458	2.079047921	0.17192
DMRT3	4.497685853	0.00327	4.08414775	0.00741	2.822768078	0.07121
DOK7	2.111091375	0.01622	0.492839379	0.60956	0.856149582	0.3607
DSG1	-2.912915742	0.00672	-0.777343923	0.37241	0.174115287	0.83907
DYDC2	-4.537450025	0.01067	-0.380316152	0.79982	0.269707922	0.85623
EGLN1P1	4.73189834	0.03018	2.32462005	0.30671	3.335051859	0.13339
ESRRAP2	-3.423908828	0.01105	-1.009718355	0.34182	-0.355593582	0.73103
FAM196B	1.948344367	0.01099	1.018682183	0.18875	-0.050002349	0.95306
FAM83C	1.173636849	0.04959	0.724440384	0.23401	0.839934325	0.1682
FDCSP	5.261557143	7.28E-05	3.119667265	0.0207	2.69623748	0.04785
FFAR3	3.295697374	0.03431	0.604204139	0.73578	2.303369406	0.15918
FOXI1	-4.355063201	0.01197	-0.721152209	0.624	-0.89246597	0.54854
GABRB1	1.577894841	0.04422	0.253198971	0.75256	-0.914023466	0.31462
GFI1B	1.691069851	0.0108	1.392381406	0.03706	1.254452384	0.06441
GJB5	2.751277961	0.04072	1.634129907	0.23206	1.548325625	0.26031
GOLGA8UI	-2.944080093	0.04	-0.411478372	0.71569	-1.656631574	0.20284
GPR20	30.60901368	1.83E-05	29.74471661	3.14E-05	0.002761126	0.99969
GPR55	5.026614853	0.00036	5.331779507	0.00013	2.690653093	0.08376
GRIA3	3.058603144	0.04052	3.029854698	0.03934	0.511170765	0.75792
GRIK3	4.075688638	0.00696	0.670916123	0.6738	0.228696989	0.88894
GSTM5	2.260310835	0.03194	1.665028088	0.1099	1.005173499	0.33832
GSTT2B	-5.552927234	0.02682	-2.920348249	0.22075	-1.696208706	0.46726
H2AFZP6	3.399959354	0.04085	0.593728562	0.75181	1.906491058	0.28963
HCAR3	2.286181012	0.01206	1.549339843	0.09475	1.608169882	0.08382
HCN2	1.275998625	0.02994	0.086404467	0.89031	0.341673861	0.58185
HEPACAM2	-1.77423063	0.02857	0.385514349	0.54271	0.041475834	0.94909
HES5	3.556180488	0.02912	2.386502512	0.16117	2.703473793	0.10884
HLA-J	1.349744734	0.04875	0.515521861	0.46658	0.211932528	0.77213
HMGN1P1(	2.725532989	0.00964	1.292053711	0.24969	1.874342541	0.08577

NRNPA1P4	-3.920042111	0.00418	-0.161370756	0.87289	-3.245317941	0.01773
NRNPA1P7	1.545202818	0.04018	0.023554201	0.97727	0.630375715	0.42699
HPR	1.373392494	0.01841	1.372409308	0.01688	0.166456228	0.79477
HSD3B2	3.90613102	0.02322	4.734484821	0.00541	0.9981128	0.60219
HSPD1P7	2.753120698	0.04867	1.922106525	0.18471	1.745020213	0.23921
IDO1	2.41374708	0.00853	2.564905723	0.00455	2.848786004	0.00158
IGFL3	-3.587849792	0.02075	-0.130243372	0.91785	-0.401687279	0.75584
IGJ	2.942407499	0.03985	-1.311643645	0.44276	1.299668883	0.39634
IL20	3.465170464	0.03386	-0.127617693	0.94507	0.821749822	0.65732
IL37	2.242163295	0.03464	3.175640988	0.00229	-1.687714097	0.23585
INHBC	2.936489156	0.03722	2.401246138	0.09361	2.619901736	0.06623
INSM2	-3.49319196	0.03757	-0.358807988	0.80289	-2.034204937	0.21726
KCNJ9	2.346568815	0.04878	0.318452677	0.81218	0.417187922	0.75635
KCNV2	1.462547476	0.04921	0.741278921	0.33742	0.41554428	0.60615
KIAA1239	1.637783854	0.03529	0.317407779	0.70404	1.746792965	0.02382
KL	3.407397176	0.00545	2.69246231	0.03246	3.572923521	0.00335
KLRC1	1.959149285	0.03944	1.10825703	0.25919	1.237010846	0.20825
KNG1	2.873457661	0.02152	3.732107731	0.00228	1.43968298	0.2751
KRT18P34	1.620530338	0.02509	0.721124129	0.34174	0.8360528	0.27102
KRT8P48	2.434817535	0.04963	1.413782066	0.27116	-1.917695285	0.21909
LBP	5.101583709	0.00058	3.32978329	0.03059	2.70058651	0.09085
LDHAP5	-1.412018223	0.02287	-0.52154602	0.33952	-0.610367753	0.27404
LDHC	-1.492254879	0.01487	-0.028275221	0.95972	-0.061789294	0.9106
LILRB3	2.961286296	0.03095	2.77863128	0.04571	1.643473401	0.26573
LIPF	-4.385903967	0.0071	0.228980366	0.85949	-3.72001764	0.02027
NC01-131E	3.613731918	0.02586	3.126014168	0.05647	2.684911777	0.11059
MADCAM1	3.081500569	0.00565	-0.662860424	0.64086	1.237292276	0.29396
ARCKSL1F	-1.461622283	0.02545	-2.000211769	0.00446	-0.661144311	0.24645
MORF4L1P	-2.721378137	0.0193	-0.648526898	0.50257	-1.127506459	0.25834
MS4A8	-1.929474215	0.04711	-0.734232291	0.38266	-0.200421931	0.80626
MT1L	3.813658336	0.02108	2.997288805	0.07927	2.025998613	0.2444
MTCYBP3	1.680558305	0.04452	0.030553027	0.97382	0.34264514	0.70784
NAPSB	1.635031376	0.04028	0.926128519	0.25571	0.943724488	0.24884
NETO1	2.289406932	0.02361	2.12476174	0.03135	0.248092219	0.81985
NKAIN2	2.327785834	0.03694	1.335149521	0.2361	1.321393199	0.25148
NT5C3AP1	-2.033140979	0.00608	-0.286477804	0.62879	-3.024715734	0.00071
NT5CP1	3.114640522	0.03412	-0.848964161	0.62375	1.690387633	0.28385
NUTM2F	4.644732756	0.00513	2.464331808	0.16201	4.667154279	0.00487
NXPE1	2.386386585	0.01188	2.602771737	0.00555	1.997129171	0.03312
OR51E1	-2.700777627	0.02755	-2.111565207	0.07346	-0.441599195	0.68652
OR52N3P	3.039260269	0.00635	-0.079751936	0.95314	2.433569712	0.03167
OR52P1P	3.134619575	0.00962	0.003897037	0.99783	2.492358592	0.04258
OR52U1P	2.874839829	0.02961	1.187230635	0.4034	2.071695212	0.12757
OR7E11P	2.72948605	0.00364	2.095626934	0.02769	2.39614781	0.01137
PCOLCE2	1.189394525	0.02553	0.462437654	0.40722	0.113502188	0.84521
PDIA2	1.723340848	0.02909	-0.706815377	0.48342	1.774278131	0.02398
PGLYRP2	5.308302049	6.83E-05	5.175850473	1.00E-04	4.228620556	0.00201

PHBP15	5.490072821	0.0045	-0.127617611	0.9531	4.557181068	0.01925
PIWIL3	-2.11276606	0.01373	-1.136267996	0.11101	-0.45623096	0.49558
POMC	3.240347907	0.03805	2.136160954	0.19058	2.055251587	0.22067
PPP1R14D	2.314776929	0.00606	1.03158442	0.24459	2.04760329	0.01598
PRKRIRP9	2.072885416	0.03589	1.310304006	0.19653	1.138021733	0.2707
PROZ	1.652796984	0.01767	0.571558874	0.44377	0.846156143	0.27065
PRPH	4.447388581	0.01236	0.942742444	0.6243	1.357080857	0.49221
RBMX2P1	-2.956172876	0.03372	0.582869108	0.58051	-0.264197806	0.81813
P1-130L23	1.265737417	0.04059	0.330104021	0.61456	0.83240886	0.18995
P1-168P16	1.31280473	0.04689	-0.972199447	0.22522	-0.227891083	0.75855
P1-278E11	-1.531220763	0.04964	0.027987632	0.96569	-0.879109563	0.21716
P1-315G1.	1.345784637	0.02546	-1.973657203	0.0294	0.884664437	0.15249
P1-39G22.	1.6177624	0.02431	1.075823665	0.14364	0.783154933	0.3038
11-1094M1	2.864669309	0.04988	3.224832931	0.0229	2.407421627	0.10974
P11-10L12	-1.192623512	0.03234	-1.152640125	0.03323	0.139100948	0.77322
P11-113A1	-2.930428258	0.03082	-0.653540484	0.56868	-0.548047656	0.63377
11-1149O2	3.03799772	0.01374	3.358244194	0.00541	1.737847093	0.19729
P11-160A9	-1.790995257	0.04967	-0.742529614	0.33171	-0.519329599	0.49299
P11-166O4	2.784911684	0.03682	1.127687262	0.4509	1.282455317	0.38988
P11-288E1	-2.141838234	0.04294	-0.502867059	0.55825	-2.142443051	0.0414
P11-312J1	-2.677679081	0.04406	0.996521575	0.2902	0.098648181	0.91988
P11-345K2	1.561551105	0.02875	1.426814357	0.0451	1.667136641	0.01845
P11-350G8	3.133749067	0.0176	3.074274351	0.01929	3.304234787	0.01162
P11-359M6	-3.22518783	0.0218	-1.306888459	0.30078	-0.05013001	0.96409
P11-382A2	1.722134016	0.03218	0.088476183	0.92179	2.001079077	0.0115
P11-459D2	-2.540022905	0.00604	-0.645136879	0.40261	-0.654244149	0.39947
P11-466P2	-18.13305527	9.35E-05	2.14677934	0.63585	1.581974228	0.72735
P11-486B1	-2.71189858	0.04735	-0.626170697	0.56525	-1.043194451	0.36919
P11-589M4	1.235045508	0.03019	0.13728559	0.82005	-0.082753611	0.89425
P11-632F7	-4.350596444	0.0182	-1.924095197	0.25181	-0.575519285	0.71883
11-655M14	4.702513518	0.00101	2.263129	0.14988	2.795456571	0.06766
P11-673D1	-1.450737099	0.02378	-0.223017556	0.66828	0.003321476	0.99486
P11-687F6	1.363787864	0.02792	0.616899829	0.3365	1.031112411	0.10184
P11-71H17	-2.565535111	0.03443	-1.734993818	0.0862	-0.644139407	0.46411
P11-730A1	2.739537912	0.03325	2.385985586	0.06647	2.690594621	0.03657
P11-777B9	2.196441524	0.03304	1.774735667	0.08932	2.428499447	0.01693
P11-79D8.	-4.917251506	0.00073	-0.95529837	0.39111	-0.794475682	0.47527
P11-86K22	2.627874792	0.04023	1.191882985	0.38531	2.965238262	0.01906
P11-927P2	2.931828984	0.03963	2.729060291	0.05554	2.041360041	0.16309
P13-672B3	2.033569819	0.04256	0.217446086	0.84754	1.56535786	0.12652
P5-890O15	3.172089957	0.03019	2.583222038	0.08488	2.468198008	0.10575
RPL10AP6	2.102299402	0.02003	1.743366061	0.05497	-1.01796881	0.37106
RPL22P11	-3.863607469	0.02816	-0.074093514	0.9605	0.75639696	0.60761
RPL35P5	-3.593918519	0.01753	1.043187915	0.36913	-0.165833309	0.89244
RPL5P5	3.16941118	0.03609	1.531678842	0.34497	3.017239952	0.04671
RPL7P21	-4.560030684	0.004	-1.218092715	0.3526	0.877365056	0.47872
RPL7P44	-3.518940601	0.0136	-0.490426948	0.66697	-2.12270567	0.12842

RPS2P41	3.114887393	0.02502	0.603317742	0.71417	2.267121295	0.11626
RPS3AP39	-2.995878055	0.04739	-1.135230908	0.37819	0.192121118	0.87218
RPS3AP46	-3.335311786	0.00984	-0.112844954	0.90509	-1.321670387	0.24661
RPS7P14	-2.579281729	0.0031	-0.392996461	0.50007	-1.258040773	0.05818
RTL1	2.20913825	0.01925	1.208235666	0.2154	1.534889089	0.11223
RXRG	3.519473809	0.03238	4.080734378	0.01217	0.006174434	0.99738
SERBP1P6	2.24313982	0.00201	-0.038370013	0.96364	1.121525302	0.14363
SERPINB4	3.957049017	0.00423	4.893279821	0.00027	5.230596193	0.00011
SERPINC1	3.024907684	0.01883	1.551435885	0.24885	2.325313383	0.07689
SETP20	2.755796182	0.02474	2.30811642	0.0628	2.094896785	0.09677
SLC25A41	1.15799763	0.04641	0.204469496	0.74058	0.570821262	0.34465
SLC28A1	4.126689128	0.00446	3.66669313	0.01198	3.637632536	0.01333
SNRPGP12	1.768342066	0.04298	-0.03374173	0.97293	1.425491758	0.10833
SOWAHD	-1.744555268	0.04433	-1.024051071	0.18104	-0.777673019	0.3036
SOX10	2.066631881	0.00776	0.660841317	0.40584	0.685111704	0.40349
SPINK4	-3.827854218	0.0145	-2.106859785	0.12534	-3.450348038	0.02376
SPRR2D	5.06962306	6.86E-05	1.306751695	0.41122	3.005881699	0.02865
SRGN	3.942385187	0.02082	4.540070419	0.00672	3.017393487	0.08389
SSX2	3.724740546	0.00385	3.360845846	0.00932	2.950110124	0.02614
SSXP3	2.494609801	0.02038	1.984023858	0.06894	2.274785543	0.03572
SULT1C3	-1.452246175	0.04484	-1.623273406	0.0248	-1.577855176	0.03036
TBX20	3.513683289	0.00808	1.364108975	0.32639	1.586792035	0.26172
TBX22	-2.587565374	0.04933	-4.412154203	0.0021	-2.238701787	0.0658
TNFRSF18	3.289234977	0.0042	2.614439045	0.02431	2.052952247	0.08565
TNFRSF4	1.855486128	0.02937	1.519459903	0.08163	-0.855273845	0.39734
TTC4P1	2.227753515	0.00384	0.584916416	0.47478	-0.432899779	0.63154
TUBBP10	2.274252012	0.03246	-0.875818425	0.50546	2.506172055	0.01763
UBQLNL	1.765692815	0.00804	0.576377092	0.4195	1.162120981	0.09157
UCN2	1.135928908	0.04525	0.14801738	0.80446	0.029043193	0.96209
WAS	1.786906198	0.0493	0.633859829	0.51161	1.314625115	0.16835
WNT10B	4.898177422	2.06E-05	3.774771365	0.00124	4.489033493	0.00011
XKRX	1.586209275	0.01766	0.873397745	0.20067	0.373244929	0.60578
YBX1P10	3.284804146	0.02214	3.389858402	0.01672	3.269173311	0.02257
YBX1P2	3.464714079	0.0102	2.675371871	0.05588	2.399835247	0.09682
PDCD5	0.157468865	0.16145	0.869508118	4.09E-15	0.087831061	0.43562
PI4KAP2	0.078869234	0.53144	-0.895322787	3.13E-12	0.238050663	0.05795
ETF1	0.138884956	0.08317	0.545501148	6.56E-12	0.177002623	0.02706
IFT140	-0.221606405	0.113	-0.877884233	4.95E-10	-0.221015005	0.1125
RHOA	-0.097041904	0.08554	0.348547166	4.88E-10	0.06919216	0.21885
RHEB	0.109308828	0.16579	0.467481906	1.45E-09	0.085513232	0.27836
BZW1	0.109789623	0.21281	0.530740758	1.62E-09	-0.007831717	0.92919
FER1L4	-0.539345848	0.10016	-1.970387238	3.51E-09	-0.045348175	0.88929
CLSTN1	-0.135672967	0.09187	-0.475103292	4.10E-09	-0.198233931	0.01387
MAMDC4	0.081138156	0.62757	-1.005407989	4.49E-09	0.145194897	0.38438
MAU2	0.076546105	0.49772	-0.657940815	6.62E-09	0.033317534	0.76788
CDK10	-0.103798621	0.37547	-0.679508345	8.63E-09	0.033523782	0.77374
NXT2	0.156824609	0.18233	0.657243822	8.68E-09	0.428747056	0.00022

CERS6	0.033768515	0.74512	0.594129763	9.04E-09	0.213327549	0.03965
CSDE1	-0.018582677	0.77934	0.380142176	9.21E-09	0.065140138	0.32578
C21orf2	-0.023801483	0.8505	-0.740520133	9.96E-09	0.388129278	0.00188
CAPZA1	0.077798108	0.19114	0.336822931	1.23E-08	0.015312332	0.79675
P11-270C12	0.693464709	0.29853	-4.357116916	1.39E-08	1.277057586	0.05505
ACOT11	-0.169106567	0.11956	-0.618235509	1.74E-08	0.02013256	0.85241
DYNLT1	0.075777655	0.4121	0.509507811	2.15E-08	0.061918302	0.50252
POMP	-0.10899134	0.1482	0.409885474	2.45E-08	-0.140479918	0.06217
SPCS2P4	-1.188645319	0.37432	-9.242068832	3.69E-08	0.525281434	0.69391
HNRNPC	-0.032419795	0.73561	0.519846851	5.88E-08	-0.011116925	0.90781
NUMA1	-0.026430332	0.8067	-0.586323745	6.45E-08	-0.077328515	0.47597
STARD3NL	0.026928262	0.73116	0.410523386	6.76E-08	0.017670873	0.82123
HOOK2	-0.107078572	0.4106	-0.701130602	1.04E-07	0.084403156	0.51441
RANBP9	0.164799688	0.06322	0.463579053	1.25E-07	0.066049367	0.45769
TMEM184A	-0.121968601	0.50888	-0.979406977	1.33E-07	0.071416884	0.69728
SUB1	-0.018920624	0.82823	0.45363259	1.71E-07	-0.06542592	0.4529
NBEAL2	-0.084744233	0.59166	-0.82528576	1.96E-07	0.048134476	0.76057
UBA2	0.022733032	0.80924	0.484826168	2.27E-07	0.110708625	0.23897
CAPN8	-0.01482189	0.9273	-0.839111344	2.67E-07	0.235947699	0.14576
ADRA2A	-0.692899247	0.06247	-1.995393346	2.81E-07	0.216464434	0.55288
XRCC5	-0.029722585	0.80983	0.631783422	3.06E-07	0.032268877	0.79385
SLC38A10	-0.159835248	0.11266	-0.512594484	3.77E-07	-0.264918926	0.00842
AACS	-0.217067401	0.06384	-0.594887267	3.91E-07	-0.134312274	0.25038
EML4	-0.054398223	0.45162	0.362333581	4.63E-07	0.027564881	0.70247
PLCXD1	-0.228687789	0.2614	-1.026905575	4.87E-07	-0.063522914	0.75507
LAMTOR3	-0.120890887	0.21861	0.486143857	5.29E-07	0.00191001	0.98443
SRI	0.071488921	0.44176	0.457195858	6.52E-07	0.297852937	0.00127
UXS1	0.179189718	0.06681	0.47891587	6.66E-07	0.0602029	0.53863
RGL2	0.023378623	0.82293	-0.519609353	7.55E-07	-0.102045621	0.32834
GOLGA8A	0.143342755	0.26678	-0.640186753	7.90E-07	-0.026669943	0.83642
TCP1	-0.063383798	0.48782	0.446233899	9.27E-07	0.191833861	0.03534
FAM83E	-0.33961053	0.1002	-1.022041519	1.01E-06	0.212931834	0.29813
RNPEPL1	-0.248372982	0.12586	-0.798717938	1.01E-06	0.019134871	0.9059
POLG	-0.020893022	0.87122	-0.63138309	1.02E-06	0.025864033	0.84116
HBS1L	-0.000236621	0.99789	0.429807145	1.16E-06	0.197060274	0.027
UBLCP1	0.085313806	0.42729	0.508367477	1.24E-06	0.168849251	0.11362
LPGAT1	0.180388899	0.10288	0.531339516	1.42E-06	0.335299372	0.00239
RPL38	0.26035736	0.05405	0.650437893	1.45E-06	0.492365087	0.00027
SBDS	0.021970897	0.82529	0.472392147	1.45E-06	0.175675622	0.07597
TAF8	-0.017948747	0.87526	-0.552558708	1.50E-06	-0.053183939	0.64079
ATP5C1	0.082465392	0.49697	0.581193522	1.56E-06	0.175055903	0.14888
TFF2	-0.181263133	0.61051	-1.709284055	1.57E-06	0.071201745	0.84142
PSMD6	-0.061906733	0.49431	0.429263162	1.63E-06	0.09215739	0.30782
CAPN5	-0.157759484	0.32701	-0.774996155	1.67E-06	0.13379796	0.40411
SKP1	-0.103350291	0.16856	0.355848806	1.80E-06	0.065881574	0.37877
ENGASE	-0.094143311	0.54046	-0.738262228	2.02E-06	0.221923912	0.14598
UBE2N	-0.045566258	0.59863	0.40764529	2.06E-06	0.008529597	0.92135

EME2	0.176240842	0.2008	-0.664048054	2.34E-06	0.201403646	0.14322
IGSF9	-0.432738801	0.08921	-1.212775641	2.38E-06	-0.059530879	0.81425
PTPN23	-0.066967049	0.78735	-1.192505829	2.41E-06	0.066208332	0.7901
TNK2	0.230444055	0.20758	-0.876581423	2.41E-06	0.166113437	0.36402
EPB41L2	0.235605513	0.12482	0.718781592	2.67E-06	0.127734284	0.40592
SOAT1	0.149777809	0.22391	0.574381115	2.74E-06	0.293803534	0.0168
BAHD1	-0.068374796	0.58995	-0.604155844	2.85E-06	-0.051081175	0.687
TRPM4	-0.222072386	0.25184	-0.913854744	2.85E-06	-0.025950708	0.89311
ATMIN	0.175499504	0.05115	0.417180429	3.01E-06	0.119946893	0.18298
RP5-850E9.	0.086375742	0.98612	22.6664332	3.10E-06	22.51257133	3.62E-06
WDR36	-0.050121235	0.71559	0.626466956	3.47E-06	-0.009202906	0.94661
MYRF	0.189511169	0.20655	-0.699012755	3.58E-06	0.235866206	0.11561
FER1L6	0.330989452	0.17703	-1.135456142	3.82E-06	0.498882176	0.04183
2-Sep	0.007001011	0.89798	0.251051932	3.87E-06	0.045581274	0.4035
DIS3	0.072748952	0.44983	0.441475482	3.91E-06	0.105889735	0.27077
AIDA	0.099272985	0.29725	0.43341952	4.13E-06	0.082547672	0.38604
LRIG3	0.217826407	0.05915	0.529396441	4.15E-06	0.442249358	0.00012
GTF2IRD2	-0.281595732	0.19849	-1.030960468	4.32E-06	-0.220164335	0.31208
YWHAB	0.07448237	0.35712	0.368437179	4.70E-06	0.132604596	0.10068
ZNF814	0.108786364	0.35305	-0.541509718	4.91E-06	-0.016103981	0.89085
PSMD7	-0.026741255	0.76006	0.394373934	5.05E-06	0.051155511	0.55774
LPHN1	0.180768037	0.14629	-0.573554259	5.26E-06	0.012247487	0.92183
PDXDC2P	-0.262929822	0.09316	-0.720842934	5.26E-06	-0.183399903	0.2377
SBNO1	0.02460165	0.7323	0.324668898	5.27E-06	0.060610473	0.39843
EIF4A1	0.077722984	0.45569	0.473321752	5.43E-06	0.087750236	0.39965
LAPTM4A	-0.008615932	0.94536	0.569309084	5.44E-06	-0.234814262	0.0621
PIK3C2B	-0.053031969	0.75265	-0.767335079	5.39E-06	0.222771992	0.18508
BBS1	0.064689798	0.71696	-0.813510716	5.46E-06	-0.313649257	0.08224
XRN2	-0.01885664	0.78108	0.304621592	5.72E-06	-0.073989001	0.27557
TMTC3	0.02314221	0.81091	0.435692387	5.76E-06	-0.04571222	0.6362
NRAS	0.052313832	0.45314	0.312697064	6.06E-06	0.137621451	0.04778
DCUN1D1	-0.032861466	0.80063	0.585608584	6.10E-06	-0.096016026	0.45984
NFRKB	0.00366771	0.96979	-0.439888285	6.23E-06	0.050244836	0.60335
AMD1	0.137564233	0.26816	0.558837344	6.37E-06	0.225925079	0.06863
PLEKHA6	-0.026334507	0.87016	-0.728576105	6.41E-06	-0.079359444	0.622
DIP2A	-0.037101164	0.74951	-0.524048333	7.24E-06	0.00708676	0.9513
PAIP1	0.039340873	0.6865	0.432782509	7.57E-06	0.099506511	0.30633
MED12	0.244024979	0.1125	-0.689138012	8.47E-06	-0.173355221	0.2605
SNX3	0.113575491	0.33013	0.517070998	8.48E-06	0.34430429	0.00309
SUMO2	-0.049231622	0.54853	0.363322076	8.74E-06	0.015308167	0.85187
INTS1	0.228236118	0.1748	-0.749560597	9.04E-06	0.070018729	0.67758
MRPL50	-0.129201437	0.23648	0.475733266	9.10E-06	-0.056939042	0.60065
SDCBP	0.244751201	0.21416	0.872471162	9.26E-06	0.197261899	0.31669
PSMA4	-0.123336262	0.15132	0.376473159	9.53E-06	-0.02643818	0.75789
PTOV1	-0.068409968	0.60898	-0.594249977	9.74E-06	-0.16743097	0.21094
GPR56	-0.36018529	0.131	-1.058257977	9.84E-06	-0.488987	0.04029
POTEF	0.028812352	0.8735	-0.804866605	1.11E-05	-0.166963033	0.35733

USMG5	0.050518914	0.6129	0.43453538	1.11E-05	0.124769819	0.21039
KIF2A	0.094045535	0.41549	0.501702076	1.17E-05	-0.022963785	0.84221
MICALL2	-0.275127498	0.23234	-1.024739309	1.17E-05	-0.308691374	0.18092
ZMYM3	0.118957119	0.21497	-0.422695623	1.18E-05	-0.013978573	0.88431
HINT1	-0.048219553	0.56324	0.362020378	1.23E-05	0.130902148	0.1155
SUZ12P	-0.157015357	0.15864	-0.489100882	1.26E-05	-0.252850763	0.02343
SMARCA4	-0.09162232	0.48684	-0.575085309	1.35E-05	-0.469807582	0.00037
PITPNM3	0.03067178	0.90803	-1.181798447	1.57E-05	0.417052715	0.11296
SRP14	-0.236118423	0.12745	0.665804957	1.61E-05	-0.22517578	0.14597
PDE4A	-0.562140212	0.09376	-1.515219025	1.62E-05	-0.701434574	0.03499
IL17RE	-0.418885935	0.08334	-1.042570027	1.80E-05	0.209321756	0.38298
ZNF785	0.055429758	0.63241	-0.50964255	1.80E-05	-0.042593903	0.71452
PNMA1	-0.030567085	0.8323	-0.624403165	1.85E-05	-0.359211717	0.01343
ABCC3	-0.15086288	0.48552	-0.924765077	1.98E-05	0.231597853	0.28351
RPL23	-0.159292586	0.19202	0.520751981	1.97E-05	-0.02627541	0.82957
LY9	0.081841959	0.77929	-1.375864629	2.01E-05	0.001664463	0.99549
MRPL51	-0.096459659	0.34468	0.426456563	2.01E-05	-0.119780633	0.23895
SORD	0.317669357	0.12038	0.870899397	2.01E-05	0.663877035	0.00116
SLC17A9	-0.221663559	0.28038	-0.878825879	2.11E-05	0.219708612	0.28157
TNPO1	0.028913634	0.77514	0.429560268	2.11E-05	-0.16746372	0.09824
QSOX1	-0.202943716	0.23771	-0.731040194	2.12E-05	-0.073429502	0.66918
CCT5	-0.003995424	0.97167	0.47690191	2.13E-05	0.235898652	0.03581
PHC1P1	-0.060589101	0.93432	-3.521934832	2.16E-05	-0.70798679	0.33945
CLTC	0.094080635	0.16567	0.287183917	2.23E-05	0.141156305	0.0374
F2RL3	-0.141280016	0.51128	-0.981173965	2.27E-05	-0.480357523	0.0302
NARS	-0.153087009	0.21355	0.519466758	2.32E-05	0.10291597	0.40239
SMN2	0.50378155	0.05815	1.11632384	2.35E-05	0.463923486	0.08105
GTF2E2	0.105095349	0.42878	0.554523153	2.40E-05	0.286577769	0.03035
EIF2B2	0.19055702	0.07372	0.443884202	2.44E-05	0.110343607	0.30125
ATP6V1B2	0.091625582	0.28502	0.355026176	2.46E-05	0.157047776	0.06582
COL27A1	-0.0542723	0.71134	-0.61898024	2.52E-05	0.029794067	0.83882
EIF5	0.059067152	0.71631	0.684184315	2.52E-05	0.044927229	0.78218
ICT1	-0.121686137	0.39891	0.584895082	2.52E-05	-0.185283796	0.19776
PKP4	0.166201359	0.1323	0.462702907	2.56E-05	0.429980811	9.37E-05
PSMB2	0.116170829	0.23515	0.404641511	2.58E-05	0.07606075	0.43682
UBE2L3	0.081554259	0.38186	0.390101209	2.58E-05	0.117844861	0.20586
SESN2	-0.076746932	0.66064	-0.739783235	2.72E-05	-0.417578193	0.01746
ARGFXP2	-0.646361216	0.13595	-2.386645685	2.92E-05	-0.522732129	0.2181
PRDM16	-0.260800154	0.21247	-0.87815533	2.93E-05	0.289939365	0.16416
UBA3	0.024397774	0.7926	0.381243986	3.07E-05	0.09045225	0.32788
MEF2D	0.316120406	0.07305	-0.754064426	3.09E-05	0.102527108	0.56464
ZNF154	-0.085727023	0.58075	-0.651519935	3.10E-05	-0.080462133	0.60427
MORF4L2	0.141942741	0.07254	0.328538333	3.15E-05	0.143732042	0.06897
WASH3P	0.014217792	0.92588	-0.645696593	3.32E-05	0.000136332	0.99929
TJAP1	0.104859584	0.4782	-0.622730496	3.41E-05	0.087929158	0.55186
CREBBP	0.209185946	0.28817	-0.818430511	3.47E-05	-0.119676409	0.54403
EPS8L1	-0.123612802	0.60043	-0.9864657	3.48E-05	0.015182488	0.94861

HSPG2	-0.054479327	0.85283	-1.21431639	3.58E-05	-0.551210684	0.06056
SMIM5	-0.210362349	0.45232	-1.213446052	3.60E-05	0.160202582	0.56292
ZBTB33	-0.038795637	0.68075	0.382732041	3.60E-05	0.007415111	0.93707
SMG6	0.332571424	0.0649	-0.749748956	3.63E-05	-0.277408801	0.12488
HNRNPH2	-0.048763524	0.53404	0.320195358	3.65E-05	0.13305927	0.08812
ACSL3	0.078507131	0.52835	0.513069341	3.69E-05	0.135156946	0.27738
PRNP	0.052093327	0.67445	0.507162447	3.76E-05	-0.051090396	0.68062
OBSCN	0.041014636	0.807	-0.697050796	3.80E-05	-0.201594125	0.23111
RAF1	-0.045119606	0.49557	-0.271355158	3.80E-05	0.007239713	0.91245
SLC36A4	0.088460986	0.4921	0.522906585	3.81E-05	0.151376783	0.24096
DLG5	0.078712074	0.5008	-0.482884948	3.89E-05	-0.240189972	0.04066
ATP6V1E1	0.159605086	0.10198	0.395728149	4.05E-05	0.053577662	0.58336
TMEM183A	-0.017317186	0.82963	0.325280307	4.06E-05	0.246368016	0.00201
FGFR3	-0.293167565	0.08794	-0.704226773	4.20E-05	-0.126247016	0.46076
CHCHD3	0.007171594	0.9433	0.408713862	4.23E-05	0.15944906	0.11232
PROSC	0.020122473	0.85972	0.459764597	4.34E-05	0.147994743	0.19169
CHST12	-0.009468233	0.95448	-0.689389855	4.36E-05	-0.18324226	0.27104
SCYL2	0.11935455	0.28779	0.457251226	4.40E-05	0.22895805	0.04132
SNRNP70	0.065779871	0.52168	-0.420844203	4.42E-05	0.049505833	0.62968
CNIH4	0.13821574	0.19919	0.436076059	4.51E-05	0.064728145	0.54766
SQLE	0.016641922	0.87897	0.444674315	4.64E-05	0.241096449	0.02725
EIF2S2	-0.030273948	0.75399	0.390473047	4.81E-05	0.068657143	0.47657
LRP1	0.085031525	0.64999	-0.761687232	4.86E-05	0.027304645	0.88416
DIMT1	-0.017953403	0.87868	0.468896686	4.89E-05	-0.020664131	0.85998
CNOT7	0.016825336	0.88806	0.482822024	4.96E-05	0.07349953	0.53827
PER1	-0.194339203	0.53928	-1.294108677	5.03E-05	-0.533057947	0.09391
ZNF335	0.201220535	0.08181	-0.478754922	5.21E-05	0.028838711	0.80406
PBX4	-0.124086163	0.67848	-1.375431629	5.61E-05	-0.164841002	0.58491
RPL26L1	-0.028935844	0.80149	0.443681706	5.83E-05	-0.038291429	0.73834
CSNK1A1	-0.047287081	0.48677	0.271569723	6.01E-05	0.067685957	0.31886
PTPRA	0.249770746	0.10818	0.621791803	6.10E-05	0.247336032	0.11158
SLC22A20	0.141676899	0.47344	-0.844418932	6.10E-05	0.248325234	0.20656
CD99	0.403265594	0.05463	0.837310244	6.42E-05	0.222818847	0.2885
MVB12A	-0.154824518	0.27471	-0.572086353	6.45E-05	-0.163376142	0.24504
ZNF586	-0.198682342	0.07931	-0.450950568	6.47E-05	-0.133148354	0.23351
TRIP12	0.002855239	0.9749	0.361252276	6.62E-05	0.062883084	0.48819
PFKFB3	0.156850687	0.46895	-0.863252004	6.86E-05	-0.188136382	0.38522
NELFE	0.149916616	0.07962	0.335124271	6.99E-05	0.181528003	0.03322
TEP1	-0.139597503	0.42831	-0.700780052	7.20E-05	-0.033141298	0.8507
MORF4L1	0.037900616	0.62268	0.304731299	7.22E-05	-0.061314847	0.42634
NAP1L1	-0.090005427	0.33297	0.367720382	7.40E-05	-0.082619843	0.37403
PFKL	-0.023781778	0.82188	-0.419170855	7.40E-05	0.079118837	0.45325
UPF1	-0.174121423	0.26292	-0.617719155	7.45E-05	-0.202926593	0.19228
CCT3	-0.01113909	0.92006	0.438280116	7.50E-05	0.028532046	0.79702
SUGT1	-0.029839373	0.73507	0.346470207	7.63E-05	0.106917326	0.22383
ATP6V0E1	0.173303788	0.14916	0.472359522	7.72E-05	0.237893276	0.04731
:TB-63M22.	3.863788183	0.43385	-19.92774287	7.71E-05	5.557984321	0.26022

ISCU	-0.01170693	0.90852	0.391814281	7.69E-05	0.018618747	0.85367
ZNF526	-0.036997985	0.78278	-0.539821955	7.67E-05	-0.100089065	0.45621
SPG21	0.160581463	0.09185	0.371632828	7.98E-05	0.115092675	0.22649
EMC4	-0.10550357	0.21943	0.329643721	8.39E-05	0.004220012	0.96039
SLC18B1	0.039310563	0.81238	0.646471338	8.41E-05	0.467610724	0.00455
SYNCRIP	-0.069460268	0.5475	0.452846231	8.40E-05	0.140154454	0.22443
TIMM23B	-0.028145753	0.72827	0.313532661	8.33E-05	-0.029616992	0.71444
VRK2	0.056682321	0.64359	0.470875366	8.41E-05	0.013026579	0.9153
SCNN1A	-0.021132999	0.92661	-0.902652152	8.93E-05	-0.046650705	0.83886
POTEI	0.035712017	0.81771	-0.613420637	9.00E-05	-0.120609433	0.43735
RWDD4P2	1.147409212	0.0719	2.469281433	9.00E-05	0.889118867	0.16422
SLMO2	0.006445388	0.95187	0.414995289	9.04E-05	0.249532195	0.01902
P11-466H18	0.143140491	0.30282	0.542263274	9.13E-05	0.511050558	0.00023
RPN1	-0.123309288	0.17476	0.354570151	9.15E-05	0.046534261	0.60823
YWHAQ	-0.067524545	0.40646	0.316885343	9.17E-05	-0.071292011	0.38058
ZFR	0.130725837	0.31645	0.508896835	9.32E-05	-0.114345458	0.38104
EI24	0.142261517	0.28341	0.515910879	9.46E-05	0.365981612	0.0057
RBM48	-0.22764224	0.15212	-0.624674215	9.55E-05	-0.231204805	0.14627
EIF3J	0.188076203	0.1152	0.461217642	9.79E-05	0.131214694	0.27104
MRPS6	0.11393956	0.21485	0.35209107	9.89E-05	-0.042396314	0.64705
CTDSP1	0.097349173	0.35612	-0.411469262	0.0001	0.068288601	0.51709
EEF1A1P7	-0.207559692	0.33288	0.783455289	0.0001	0.006609335	0.97497
PDPK2	-0.023337266	0.92719	-1.033564801	0.0001	-0.126347238	0.62157
RPS20	-0.167208802	0.09786	0.391262082	0.0001	-0.040457345	0.68863
RAB8A	0.022668799	0.87916	0.576299149	0.00011	0.148872737	0.31762
POLR2B	-0.079906558	0.33608	0.319675515	0.00011	-0.069308921	0.40339
HGSNAT	0.103204255	0.46478	-0.54783676	0.00011	0.033755808	0.81105
MYB	0.266444497	0.48162	1.441213222	0.00011	0.493728702	0.19085
ELOVL4	-0.086824668	0.7388	0.977491153	0.00011	-0.344697728	0.18881
MAPKAP1	-0.004066469	0.96132	0.320286305	0.00011	0.10234061	0.22083
CARNS1	0.193508351	0.64089	-1.644874511	0.00011	0.740018747	0.06914
TRAM1	0.099935024	0.45041	0.510183405	0.00011	0.079254861	0.54936
TXNL1	0.060541102	0.445	0.301294718	0.00012	0.056803385	0.47293
DHX9	0.016665843	0.84787	0.334193746	0.00012	0.097343749	0.2622
NFE2L3	0.437115714	0.07759	0.951250539	0.00012	0.19933993	0.4212
UBA6	0.11611496	0.18912	0.338379412	0.00012	0.225285081	0.01069
RPF2	-0.154013537	0.31675	0.584592815	0.00012	-0.017689624	0.90827
ZNF224	-0.164903954	0.13936	-0.428349569	0.00012	-0.161926635	0.14487
OBSL1	-0.04088617	0.74628	-0.485824196	0.00013	-0.32021706	0.01145
AKAP17A	0.136250185	0.26952	-0.478196107	0.00013	0.051221817	0.67848
CYP2C9	-0.129746379	0.59702	-0.954403355	0.00013	0.316104352	0.19437
FNTA	-0.049892729	0.65142	0.420314692	0.00013	0.063776452	0.56333
AGRN	0.259434644	0.10431	-0.611077208	0.00014	0.039432017	0.80525
TOMM34	0.097885785	0.36444	0.405774051	0.00014	0.049725107	0.645
SMARCA5	-0.171070421	0.07474	0.364475561	0.00014	-0.110945368	0.24751
TEF	-0.064112789	0.80476	-1.000496504	0.00014	-0.298056866	0.25155
SREBF1	0.146093816	0.26071	-0.497994991	0.00014	0.025565468	0.84406

GALT	-0.127725703	0.29471	-0.464330721	0.00014	0.01084332	0.92848
THUMPD3	-0.052427441	0.67924	0.476911768	0.00015	0.085311047	0.50014
SNRPD1	-0.187162337	0.19028	0.538282106	0.00015	-0.063052723	0.65835
RB1CC1	0.064613514	0.54965	0.406624537	0.00015	0.052006419	0.63007
AHSA2	0.047660028	0.66256	-0.415254358	0.00015	0.083335144	0.44404
ARL1	-0.008476272	0.92764	0.350817299	0.00016	0.179446178	0.05351
TCEB1	0.102110319	0.32189	0.38658777	0.00016	0.039235818	0.70318
EIF3M	-0.141322952	0.16032	0.378618786	0.00016	-0.004858432	0.96145
UVSSA	-0.024904817	0.88699	-0.669415375	0.00016	0.023128497	0.8949
ANKRD10	-0.074162589	0.45474	-0.375225717	0.00016	-0.140514375	0.15694
TMEM38B	-0.122965364	0.40924	0.545935141	0.00016	0.115880503	0.42911
RGL3	-0.192217557	0.26144	-0.653713925	0.00017	0.092126699	0.58844
SACS	0.078718154	0.64091	0.6338644	0.00017	-0.224361607	0.18433
TBL1XR1	0.126381761	0.19501	0.366158863	0.00017	0.099680527	0.30631
CDC123	-0.089315627	0.38579	0.38206301	0.00017	-0.029429858	0.7744
NCK1	0.266407698	0.09111	0.586900871	0.00018	0.417608018	0.0081
ZNF274	-0.212404931	0.16549	-0.577430598	0.00018	0.051318324	0.73569
HDAC6	-0.023187459	0.83662	-0.422349215	0.00018	-0.040765234	0.71713
UFD1L	0.151380944	0.12843	0.369950598	0.00018	0.03932919	0.69327
TM2D2	-0.152969145	0.24095	0.480796127	0.00018	-0.082927592	0.52216
PSMA3	0.064694257	0.5298	0.381828991	0.00019	0.102063432	0.32075
PTPN2	0.176014633	0.09821	0.394795705	0.00019	0.135494859	0.20231
CETN2	-0.017248727	0.84286	0.318095432	0.00019	-0.064478825	0.45866
ALCAM	0.298980257	0.11757	0.711939004	0.00019	0.379222201	0.04708
DEPDC7	0.833557827	0.05937	1.622020124	0.00019	0.119642889	0.79095
NUDCD1	-0.105661954	0.3119	0.381503645	0.00019	-0.083076165	0.42468
P11-572P18	-0.06941795	0.6179	0.517943973	0.00019	0.166151213	0.23206
PLGLB1	0.062129609	0.71096	-0.657335433	0.00019	-0.022980907	0.89087
DHRS3	0.361125159	0.17567	-1.111052931	0.00019	0.334981502	0.20901
OLA1	-0.173142425	0.19245	0.491843639	0.0002	0.134217706	0.31154
CBWD2	-0.042137478	0.72328	0.434705775	0.0002	0.111218168	0.34661
DIP2C	-0.133863615	0.42124	-0.62036179	0.0002	-0.273345702	0.10133
AKNA	-0.081846735	0.64857	-0.668318238	0.00021	-0.40584702	0.02394
PSME1	-0.095262739	0.29482	0.331985707	0.00021	0.04414184	0.62545
MRPL3	-0.091633501	0.43251	0.429395554	0.00021	0.094851414	0.41512
LAT2	-0.245814235	0.40131	-1.156053939	0.00022	0.023742613	0.93463
P11-848G14	0.001996973	0.99368	-0.966529215	0.00022	-0.145738534	0.5645
PTPRN2	-0.155614985	0.55628	-0.982227698	0.00022	-0.049925407	0.85017
TTC1	0.026041628	0.83021	0.441906279	0.00023	0.074380429	0.53897
STAT1	0.090000012	0.4471	0.434881373	0.00023	-0.066443742	0.5746
SZT2	0.110794329	0.47044	-0.570762694	0.00023	0.077150089	0.61569
ZC3H15	-0.026453935	0.79269	0.367246341	0.00023	0.108064273	0.28172
AMOT	-0.005319455	0.97691	-0.677142846	0.00023	-0.413448491	0.02471
GCSAM	-0.122818067	0.7028	-1.193386639	0.00024	-0.122543692	0.69446
MIR3916	-0.082234231	0.38824	-0.352330044	0.00024	0.067715999	0.47364
STT3A	0.071679259	0.55878	0.450002495	0.00024	0.183865805	0.13334
PAG1	-0.119725156	0.47437	-0.614558128	0.00024	-0.271182615	0.105

CASR	-0.342184	0.56523	-2.411924114	0.00024	0.527279216	0.36667
PSMB7	-0.023620356	0.78435	0.312437444	0.00024	0.000931305	0.99137
AHCYL1	0.201730589	0.08232	0.42486013	0.00024	0.302065997	0.00921
ACP1	-0.063775559	0.38959	0.268380333	0.00025	0.062089871	0.40011
EIF4E2	-0.081974503	0.41612	0.365872076	0.00025	0.081823689	0.41547
LLGL2	-0.256986876	0.14848	-0.652284829	0.00025	0.092180461	0.60383
AFG3L1P	0.039657468	0.79487	-0.568410421	0.00026	0.036893311	0.8091
CUL2	-0.044901864	0.579	0.289615832	0.00026	0.000239121	0.99764
RAN	-0.044150565	0.6797	0.389462133	0.00026	0.040374746	0.70559
STAM	-0.015843771	0.91542	0.541337699	0.00026	0.096962757	0.51501
MRPL32	-0.169924344	0.16803	0.442057522	0.00027	0.025607687	0.83465
RNF5	0.216118848	0.06908	0.429191317	0.00027	0.250617352	0.03468
SLC39A6	0.300285336	0.09152	0.646273024	0.00027	-0.19527066	0.27354
TOMM6	-0.096790809	0.38935	0.40465309	0.00028	-0.025374795	0.82107
ZNF626	-0.150567945	0.38862	-0.638833076	0.00028	-0.28486933	0.10588
GREB1	-0.110390064	0.63883	-0.86819484	0.00028	-0.411692553	0.08364
ACCS	-0.450882709	0.10578	-1.025854045	0.00029	-0.177594676	0.5174
ZNF74	0.136564237	0.64535	-1.092051506	0.00029	-0.21724115	0.4657
L3MBTL1	-0.070861489	0.72634	-0.746196437	0.0003	-0.071389763	0.72436
YWHAE	0.113501152	0.2342	0.344220126	0.0003	0.093378365	0.32737
ZNF331	-0.130566322	0.35097	-0.507075622	0.0003	-0.290682371	0.03865
APEX1	-0.023514079	0.84046	0.419938429	0.0003	-0.014435846	0.90158
CCDC53	-0.085992562	0.51022	0.456291667	0.00031	0.185204593	0.14894
PIGZ	0.006634596	0.97216	-0.696260481	0.00031	0.289358231	0.12521
WDR90	0.004801734	0.97653	-0.599652525	0.00031	0.233853925	0.15219
C6orf25	-0.157774475	0.66568	-1.542249397	0.00031	0.022604382	0.95014
GHITM	-0.10441829	0.24574	0.322118117	0.00031	0.136542055	0.12771
HP	0.061839819	0.88396	-1.585108141	0.00032	0.220882024	0.6004
RARA	0.126551537	0.42455	-0.576409435	0.00032	-0.224320182	0.1587
ASCC2	0.095809802	0.44212	-0.44751724	0.00033	0.101706514	0.41345
AMDHD1	0.540467677	0.26244	-1.873399145	0.00033	0.361859216	0.45419
ZBTB42	-0.226993102	0.16913	-0.595999148	0.00033	0.048066641	0.76457
KIAA1211	-0.082752582	0.60989	-0.5826445	0.00034	-0.024184397	0.88139
LEPROTL1	0.124554173	0.48098	0.630026163	0.00034	-0.046106431	0.79448
HAUS2	0.082239869	0.53608	0.468971013	0.00035	-0.063537869	0.63195
P11-58H15	0.198754257	0.5734	-1.507838459	0.00035	0.100628425	0.77647
CTSD	-0.019187663	0.89718	-0.53104072	0.00036	-0.156614828	0.29194
DDX3X	0.341456495	0.08675	0.708913198	0.00036	0.652998738	0.00102
LHX4	0.061806504	0.76654	-0.774245733	0.00037	0.114837129	0.57834
TPPP	0.040547902	0.85119	-0.81969002	0.00037	0.175194844	0.41139
NAP1L1P1	-0.311196853	0.06272	0.561647217	0.00038	-0.296303037	0.0751
DNAJC8	0.162344599	0.077	0.32412759	0.00038	0.039070492	0.67061
ARPC5	0.097096925	0.33574	0.35646321	0.00039	0.145117279	0.1499
KIAA1522	0.148610168	0.43327	-0.676511597	0.00039	0.203957087	0.28198
PWP1	-0.178054637	0.06507	0.336247211	0.00038	-0.067026591	0.48607
UBN1	0.103414915	0.44799	-0.486241348	0.00039	0.086452854	0.52587
KLK1	0.405583106	0.45665	1.786351965	0.00039	1.280250365	0.01323

XYLT2	-0.131994017	0.31895	-0.469986179	0.00039	-0.234418526	0.07757
YWHAZ	0.114680942	0.14477	0.278447257	0.00039	0.252042575	0.00134
EDN1	0.282459286	0.30101	0.951739206	0.0004	0.395858923	0.14562
SKA3	0.189698664	0.44839	0.878429878	0.0004	0.443868179	0.07533
SMG9	-0.199008774	0.073	-0.394147744	0.0004	-0.240506782	0.02957
RPL36AL	-0.061108452	0.60616	0.418001743	0.0004	0.142279266	0.22911
DDIT4L	1.023205434	0.07589	2.003081046	0.0004	1.560982927	0.00626
TNFRSF19	0.101090433	0.66255	0.815210058	0.0004	-0.416996696	0.07333
ZNF544	-0.032096991	0.77384	-0.392783119	0.0004	-0.040902311	0.71262
HERC2	-0.126179323	0.46583	-0.612516172	0.0004	0.00348303	0.98394
A4GNT	-0.122958023	0.80242	1.727784495	0.00041	0.850280641	0.08253
SF3B3	0.026854486	0.72938	0.27318078	0.00041	-0.020390513	0.79267
SNX29	0.008326761	0.96871	-0.751464206	0.00042	-0.636467519	0.00287
SRP54	0.049952436	0.58175	0.316890529	0.00041	0.128614537	0.15476
RAB11A	0.070302293	0.4708	0.343055902	0.00042	0.247482913	0.01101
STRN	0.128400041	0.24497	0.386603593	0.00042	0.251525719	0.02223
AC004917.	1.027670456	0.22255	-4.556498549	0.00042	1.034589264	0.21914
MBD6	-0.140874783	0.6905	-1.263536074	0.00042	-0.072387266	0.83744
CCDC47	-0.092123438	0.45603	0.432233991	0.00043	0.24329972	0.04814
SUMO1	0.066901634	0.58113	0.42488435	0.00043	0.119953192	0.32225
CMAS	-0.132907793	0.24412	0.391656279	0.00044	0.166779803	0.13802
KLF13	0.265887809	0.16596	0.67317963	0.00045	0.258350536	0.17798
SETDB1	-0.146038105	0.13796	-0.345183093	0.00045	-0.166852132	0.09009
SLC16A5	0.21454317	0.13412	-0.514923349	0.00045	0.141623374	0.32257
NKX6-2	0.307246311	0.55732	-1.872774001	0.00045	-0.017588158	0.97324
NLRX1	-0.312054182	0.08409	-0.636090759	0.00046	0.082122257	0.64591
ACTR3	0.11212165	0.20149	0.3064582	0.00047	0.031014253	0.72384
ITGB1BP1	-0.014540976	0.90462	0.414356253	0.00047	-0.063756045	0.59811
MED6	0.148553879	0.10238	0.310777314	0.00047	0.110790834	0.22327
MRPS22	-0.166866269	0.07013	0.312463428	0.00047	-0.043805547	0.63172
MYO1F	-0.382261667	0.19126	-1.073130268	0.00047	-0.370118925	0.20229
TMEM79	-0.045108534	0.85186	-0.862804187	0.00049	-0.024018307	0.9205
PHRF1	0.071716984	0.70918	-0.674153415	0.0005	0.077874479	0.68539
RBM18	0.065582384	0.60032	0.428736575	0.0005	-0.0729144	0.56018
RPL3P6	-0.281915545	0.54549	-1.78115368	0.0005	-0.418479195	0.37159
ITFG2	0.028775123	0.86409	-0.58634929	0.0005	-0.066785864	0.69079
ACER2	-0.353665607	0.09798	-0.744575941	0.00051	0.362696677	0.08797
KARS	-0.038757902	0.66091	0.304820886	0.00052	0.05531681	0.53038
HIPK3	0.140072204	0.0872	0.283482479	0.00052	0.159635458	0.05113
HNRNPK	0.098000446	0.15273	0.237491102	0.00052	0.040029697	0.5591
CNOT1	0.269008237	0.0854	0.541998341	0.00052	0.439693917	0.00492
FAHD1	0.083286123	0.41372	0.345708184	0.00052	0.037405639	0.71349
THOC5	0.043396631	0.74402	0.456665105	0.00052	-0.017008013	0.89841
GRM7	-0.684200169	0.17537	-1.766512202	0.00053	-0.307220657	0.53943
RPL26	-0.303783722	0.09627	0.632488788	0.00053	0.009546481	0.95831
CLOCK	0.194328199	0.06262	0.359542822	0.00053	0.429768917	3.61E-05
SLIRP	0.016391044	0.90921	0.492844841	0.00054	0.209151545	0.14406

PSMA2	-0.183673806	0.06733	0.34344132	0.00055	-0.076636951	0.44418
KIFC2	-0.212483685	0.28025	-0.683344788	0.00055	0.158468006	0.41594
PHF6	0.359911934	0.0521	0.63754991	0.00056	0.207581683	0.26291
TMEM178A	0.049831261	0.87836	-1.215179144	0.00056	-0.092679827	0.78393
NIFK	-0.231195574	0.14415	0.541270747	0.00056	-0.116532267	0.46083
MRPL39	-0.17155547	0.20485	0.456405495	0.00057	0.02704772	0.84037
NSA2	-0.21707211	0.18295	0.55788453	0.00057	-0.059590559	0.7143
P11-288K12	-0.30318977	0.32923	-1.122903415	0.00058	-0.240124755	0.4365
DIDO1	0.020505523	0.79082	-0.266833676	0.00059	-0.050233969	0.51626
EPOR	0.071892628	0.67872	-0.618611237	0.00059	0.111858317	0.5171
UCHL3	-0.063194714	0.60259	0.408648364	0.00059	-0.045577375	0.7067
NDUFB1	-0.199856674	0.08366	0.389577531	0.00059	0.0227875	0.84251
NONO	0.288647607	0.10212	0.606143102	0.0006	0.21119912	0.23169
SLC31A1	0.164004869	0.08098	0.320408894	0.00061	0.126138287	0.17917
ATP7B	-0.300915121	0.21988	-0.841385021	0.00061	0.082467341	0.73605
NCLP1	-0.226899625	0.41999	-0.996676014	0.00062	-0.107156802	0.70111
PPIL2	-0.003448787	0.97921	-0.456387862	0.00061	-0.083147089	0.53131
CDC42SE2	0.104696698	0.27392	0.325324324	0.00062	0.293584614	0.00206
FAM103A2P	0.113580494	0.40045	0.449933439	0.00062	0.035508862	0.79303
KRCC1	-0.039117769	0.76514	0.443599863	0.00062	0.229123758	0.0786
STON2	-0.279874568	0.12495	-0.624844683	0.00062	-0.233971589	0.19883
RPH3A	-0.165661212	0.78405	-2.152661993	0.00063	-0.564738092	0.34868
EP400	0.211943052	0.2877	-0.687268495	0.00063	-0.110169284	0.58126
TD-2008A1	0.134176547	0.61877	0.915663774	0.00063	0.848204927	0.00157
P11-22B23	-0.246383562	0.2082	-0.678075245	0.00063	0.041983822	0.82763
SUPT20H	0.194984206	0.06682	0.362030794	0.00063	0.10608833	0.31932
TMEM63A	0.002791514	0.98495	-0.506629121	0.00063	0.149458227	0.3115
EIF2AK2	-0.046521195	0.64479	0.34271257	0.00064	0.135582693	0.17798
BTF3	-0.219143715	0.11928	0.479326483	0.00064	0.001797746	0.9898
MT-ND6	0.595531283	0.08568	1.179919703	0.00065	1.430744918	3.56E-05
SLC10A1	-0.197356308	0.60113	-1.487310757	0.00067	0.159938244	0.66019
TAAR3	0.882488578	0.20217	2.221562601	0.00067	1.878644386	0.00451
MTMR6	0.035180741	0.7304	0.343791611	0.00067	-0.075665006	0.45936
DOC2A	0.056466111	0.88077	-1.365669439	0.00068	-0.391941712	0.3101
DRG1	0.06086674	0.53451	0.326634817	0.00068	0.058422194	0.54854
LIMS1	0.0610774	0.50202	0.306599413	0.0007	-0.055052927	0.54584
SYT8	0.001439771	0.99707	-1.404081161	0.00071	-0.105408821	0.78732
POLR2H	0.011272478	0.92393	0.395933225	0.00072	0.121852617	0.30072
NMRAL1	0.01968736	0.93446	-0.820468571	0.00072	-0.307209698	0.20077
PER2	-0.312826589	0.13399	-0.707303085	0.00073	-0.201632897	0.33362
C12orf29	0.157262215	0.20353	0.410719361	0.00073	0.121046683	0.32516
KLF2P4	0.133309004	0.73458	-1.530603505	0.00075	-0.074340538	0.85163
DNAAF1	-0.088337458	0.82368	-1.375420009	0.00076	-0.115883544	0.76821
DAZAP1	0.210726481	0.07576	0.397626338	0.00076	0.207692657	0.07951
NDUFS5	-0.162130055	0.06439	0.290653281	0.00076	-0.100782624	0.24885
ZNF445	-0.021681088	0.85781	-0.409284404	0.00077	-0.09345251	0.43993
ANXA7	0.078294716	0.48038	0.370000301	0.00077	0.214927599	0.05177

PDE4C	-0.170098598	0.41534	-0.714514191	0.00079	0.453883993	0.02786
IQSEC3	-0.71569336	0.07889	-1.399284326	0.00079	-0.11050295	0.77854
MARCKSL1	-0.011388274	0.88316	-0.260224216	0.00079	-0.193804236	0.01255
SPATA7	0.017878341	0.90458	0.48643918	0.00079	0.187844517	0.20156
PGD	0.044882617	0.60701	0.29143035	0.00079	0.099337191	0.25441
PROM2	-0.531455302	0.1016	-1.089140101	0.0008	-0.438515283	0.17569
MMGT1	0.010700278	0.91709	0.33811226	0.0008	0.076685018	0.45351
CHUK	-0.17024568	0.19055	0.431030338	0.00081	-0.099776031	0.44189
MZF1	0.241456547	0.09086	-0.48917396	0.00083	0.172963978	0.22621
FBXO36	-0.25333213	0.09534	-0.49972032	0.00084	-0.785009642	5.99E-07
IFNAR1	0.119239183	0.1883	0.300784248	0.00084	0.243938906	0.00695
PS10-NUD	0.139744138	0.43274	0.584398595	0.00085	0.04299683	0.80955
C1orf106	0.26546444	0.06481	-0.480342021	0.00086	0.19396396	0.17699
CEACAM22	0.177792004	0.58199	-1.211742218	0.00086	-0.034576901	0.91668
PDE6D	0.168043108	0.20617	0.429782324	0.00086	-0.009877608	0.94135
SRP72	-0.015431715	0.83718	0.247993918	0.00086	-0.036146617	0.62995
ASCC3	-0.038523494	0.69145	0.321746721	0.00089	0.007499258	0.93838
AGGF1	-0.140407905	0.1314	0.305064278	0.00089	-0.08553531	0.35698
CARD10	-0.319259796	0.05876	-0.564559877	0.00089	-0.19491873	0.24865
MRPL46	-0.048885193	0.69555	0.403725662	0.00089	0.207490277	0.0914
BRD1	0.020588842	0.85367	-0.372599473	0.0009	0.07155461	0.52279
HNRNPM	0.165425231	0.24775	0.473950656	0.00091	0.312973317	0.02863
EIF2A	-0.018305375	0.92012	0.603592311	0.00092	0.126556114	0.48787
SLC12A2	0.282900377	0.15286	0.655345221	0.00093	0.553021099	0.00519
PRRG3	0.390580549	0.19113	0.978075087	0.00094	0.048967535	0.87139
RPL5P12	-0.349379274	0.58191	1.844524521	0.00094	-1.810462227	0.01845
COX7A2	-0.114009256	0.17834	0.276582642	0.00094	0.109600647	0.19266
AJC25-GN	0.099153294	0.70232	0.850272023	0.00095	0.473932705	0.0665
HERC2P7	0.345653372	0.73971	-4.713186889	0.00095	-0.929849776	0.38165
KLC4	-0.007425147	0.96439	-0.5510673	0.00095	0.002767537	0.98669
P11-603J24	0.54488976	0.09566	1.074663689	0.00095	0.901184702	0.00568
ITPR3	-0.046101	0.78049	-0.546778506	0.00096	0.062639813	0.70486
P11-305M3	0.251392136	0.22395	0.666818086	0.00096	0.449694649	0.0279
C11orf57	0.044423958	0.75646	0.469442289	0.00097	-0.032551292	0.81994
HEXDC	-0.116289771	0.49504	-0.573852645	0.00097	-0.066061719	0.69656
GNG5	0.095542409	0.34819	0.334117403	0.00098	0.072144372	0.47855
TNRC18	0.047341459	0.75042	-0.492556113	0.00099	0.053332234	0.72027
RAP1GAP	-0.42946636	0.07916	-0.809790451	0.00099	-0.148510664	0.54023
SNX13	0.059065964	0.54978	0.322928096	0.00101	0.199362362	0.04291
ZC3H18	0.317792796	0.05211	-0.538915914	0.00101	-0.163191709	0.32013
TD-2623N2	-0.418257504	0.17164	-1.036387868	0.00101	-0.377233046	0.21519
CCNY	0.206929618	0.11479	0.430003397	0.00102	0.289587754	0.02713
PDZD7	-0.154212007	0.58491	-0.975100818	0.00102	-0.16433093	0.56152
PDCD10	0.010626512	0.9196	0.340231155	0.00102	0.095435244	0.36182
PSMB1	-0.113614264	0.22524	0.304273173	0.00103	0.028802858	0.75761
RABEP2	-0.16093541	0.34029	-0.556371431	0.00105	-0.226679223	0.17949
CPSF7	0.178210636	0.09333	-0.349368848	0.00105	0.13788746	0.19398

ZBTB4	0.113081806	0.36953	-0.415009096	0.00107	0.013075814	0.91742
BBS5	0.053872911	0.76373	-0.596261383	0.00107	-0.047056717	0.79294
MTND4P2(	-0.241023812	0.43762	-1.042127826	0.00108	0.302198978	0.32208
NUTM2G	-0.226808224	0.45136	-1.053689868	0.00107	-0.339001522	0.26265
SLC12A9	-0.087635043	0.57865	-0.519818907	0.00107	-0.181559027	0.24874
XPR1	0.033976022	0.78265	0.400095712	0.00109	0.050535363	0.68141
ZNF789	-0.143165197	0.34119	-0.49941493	0.0011	-0.207342309	0.16746
SLX4	0.172007092	0.22827	-0.474653039	0.0011	-0.114267206	0.42729
SHFM1	-0.076464697	0.50268	0.366341089	0.0011	-0.015290636	0.89269
COG7	-0.10110167	0.37625	-0.371149631	0.00111	-0.071337606	0.52643
HSP90AA1	-0.162178575	0.19484	0.407650945	0.00111	0.318332097	0.0109
PROM1	0.238489058	0.22338	0.637707837	0.00111	0.499992648	0.01062
ONECUT3	-0.096252163	0.73715	-0.941076446	0.00112	0.096653691	0.73562
TST	-0.027775743	0.89428	-0.70411601	0.00112	-0.025283355	0.90324
TOR1AIP1	0.004115243	0.97697	0.462173742	0.00112	0.010406595	0.94178
BCAP29	0.103888716	0.32059	0.334140372	0.00113	-0.062105822	0.55328
SGCE	-0.028129018	0.8564	0.49995859	0.00113	0.105550036	0.49714
ILF2	0.018056871	0.83248	0.27632717	0.00115	0.088705299	0.2983
AL161915.1	1.102629274	0.40736	-5.415304128	0.00115	0.105820199	0.9372
UTP6	-0.075385903	0.48616	0.346981122	0.00116	0.11277066	0.29467
ACAA2	-0.183093549	0.12962	0.388664074	0.00118	-0.021785126	0.85649
BRD7	-0.105327366	0.22808	0.280931475	0.00118	0.114713494	0.18786
NSL1	-0.282877765	0.06802	0.495762958	0.00117	-0.26901797	0.08189
APOBEC3E	0.432769435	0.09016	0.819102824	0.00119	0.357957608	0.16146
SETD3	-0.035403484	0.796	0.440941108	0.00119	0.113178189	0.40736
FLRT3	0.23266961	0.14813	0.520085534	0.0012	0.143712307	0.37179
PGBD2	-0.293656531	0.10502	-0.591230677	0.0012	-0.287374781	0.11183
GRPEL1	0.059603447	0.7401	0.577860415	0.00121	0.247380488	0.16781
LGALS3	0.444511102	0.18035	1.072263482	0.00121	0.254062608	0.44397
C21orf58	0.040438194	0.85703	-0.738494016	0.00122	-0.335108435	0.13681
SEPN1	0.092132794	0.55393	-0.506828373	0.00122	-0.022412412	0.88558
TMEM86B	0.196999913	0.41063	-0.856622102	0.00122	0.102844759	0.67028
C9orf172	-0.009687615	0.96522	-0.741337403	0.00126	0.142811104	0.51615
RBM7	0.153179762	0.22753	0.407083359	0.00126	0.102527645	0.41959
TVP23C	-0.263196167	0.19406	-0.658033833	0.00126	-0.034748211	0.86283
TPP2	-0.292217037	0.08149	0.538746442	0.00126	0.050328201	0.76385
REXO2	0.035031079	0.77777	0.393344701	0.00126	-0.342350811	0.00649
PTGES2	0.321553033	0.05868	0.544903042	0.00127	0.370126682	0.02933
ERV3-1	0.019471317	0.8709	-0.389262368	0.00127	0.185367427	0.11949
LMTK3	-0.158204533	0.48473	-0.75244473	0.00127	0.175351641	0.42808
LPAR2	0.055920386	0.714	-0.497823215	0.00127	-0.062220095	0.68417
NDUFA12	-0.098179185	0.34107	0.325221668	0.00127	0.128906639	0.20611
PRPF40B	-0.209196137	0.2179	-0.5433029	0.00128	-0.102742982	0.54143
NFATC2IP	0.080128683	0.53087	-0.412571165	0.00128	-0.050250288	0.69411
GTF2A2	-0.131142522	0.24553	0.357837497	0.0013	0.064913058	0.5637
NDUFV2P1	0.600000958	0.07361	1.050855296	0.00132	0.83921438	0.0113
RPL24	-0.015268872	0.88721	0.345012614	0.00133	0.053476981	0.61923

SET	-0.177960204	0.05067	0.291569465	0.00134	-0.149792642	0.0999
DYNC1I2	0.135275441	0.08107	0.246892514	0.00135	0.011981779	0.87726
ARSA	-0.143606802	0.4687	-0.649470401	0.00136	0.146116105	0.45542
SRP19	-0.035616444	0.78082	0.404080865	0.00136	-0.081102559	0.52582
TOPORS	0.075144547	0.40755	0.286464689	0.00137	0.007875075	0.93085
STRN3	0.05500448	0.66096	0.3993569	0.00138	-0.056702736	0.65109
SSB	-0.175041346	0.21019	0.444438605	0.0014	-0.004161273	0.9762
COX20	-0.014072435	0.88412	0.30604452	0.0014	0.133711401	0.16453
MAN2C1	0.045854082	0.77545	-0.513988868	0.00142	-0.038831155	0.80898
PLEKHA7	-0.319729711	0.09913	-0.619396673	0.00143	-0.026354674	0.8916
PPP6R1	0.114850419	0.35052	-0.393685868	0.00143	0.074098174	0.54711
RPL27	-0.056684341	0.58946	0.334161828	0.00144	0.130296081	0.21443
CCNH	-0.064203767	0.57634	0.360609633	0.00146	-0.117693861	0.30423
DAD1	-0.156111445	0.0517	0.252251134	0.00146	-0.088108028	0.27055
GSE1	0.234577037	0.13949	-0.50762458	0.00146	-0.084473167	0.59537
HSPH1	-0.229406756	0.32445	0.739292474	0.00146	-0.248090151	0.28641
LGALS2	0.13225514	0.72482	1.172091072	0.00146	1.130505517	0.00219
XKR6	-0.297591388	0.14745	-0.661155341	0.00145	-0.335977397	0.10538
ZFYVE28	-0.377951441	0.17269	-0.898088039	0.00146	-0.181497376	0.51002
EBNA1BP2	-0.108811436	0.44635	0.45079625	0.00147	0.00431697	0.97583
FTH1	0.373868748	0.07922	0.677390091	0.00147	0.255548728	0.23027
PKD1L2	-0.210794006	0.44779	-0.908389583	0.00147	-0.072269559	0.79507
AC016773.1	-0.226291468	0.55785	-1.329629101	0.00148	-0.400676724	0.3037
VTA1	0.088931559	0.38912	0.324708141	0.00148	0.121980425	0.23725
IDUA	-0.06169419	0.78872	-0.7546523	0.00149	-0.048743899	0.83226
RSU1	0.223309008	0.05789	0.371760675	0.0015	0.231567654	0.04891
AP3M1	-0.026841878	0.73168	0.245724272	0.00151	-0.012592494	0.87204
SGTB	0.267958352	0.10149	0.512606737	0.00151	-0.15141756	0.35897
ARHGEF1	0.014526555	0.93724	-0.584615952	0.00151	-0.296969519	0.10755
DDX21	-0.080490586	0.55914	0.435958098	0.00152	-0.020254586	0.88309
ARMCX4	-0.166919957	0.40984	-0.647587146	0.00152	-0.364273628	0.07458
CNDP1	-0.656497145	0.08574	-1.22705898	0.00152	-0.175194241	0.63989
LDHA	0.075353768	0.54829	0.397643238	0.00153	0.156460736	0.21255
TBC1D29	-0.163671088	0.61939	-1.118265639	0.00154	0.029431326	0.92744
GTF3C6	-0.140793354	0.19088	0.334484406	0.00154	-0.156880702	0.1446
SRSF11	0.165420827	0.08376	0.3020831	0.00154	0.036888674	0.69991
AP001007.1	0.056963187	0.79747	-0.743547705	0.00156	0.148385429	0.49937
PNKP	0.17291387	0.24626	-0.477031463	0.00156	-0.062566589	0.67445
NLRP12	-0.127665891	0.67627	-1.025159615	0.00158	-0.496067239	0.11538
SPIRE2	-0.170284591	0.29233	-0.515413425	0.00158	0.183605259	0.25215
BNIP3L	0.250936786	0.0584	0.418216073	0.00159	0.214452913	0.1057
CSNK1G1	0.154329215	0.12261	0.313751503	0.00159	0.134852163	0.17786
MAN2A2	0.267235726	0.1036	-0.519423299	0.00161	0.058960495	0.7202
PTPRS	0.300046561	0.06766	-0.520703529	0.00161	-0.200028514	0.22481
ATP2A3	-0.10292517	0.69554	-0.839574931	0.00162	0.292744545	0.26308
RP11-752P2	0.267921717	0.66932	1.814743134	0.00162	0.447815811	0.46797
ARHGAP1	0.114154361	0.25484	-0.318899601	0.00163	-0.03017524	0.76422

FBXO7	0.106792238	0.12634	0.218107279	0.00164	0.265512861	0.00013
HIC2	-0.110543878	0.40021	-0.414833808	0.00164	-0.12699007	0.33316
P11-453N1[illegible]	-0.082369307	0.72409	-0.771468029	0.00164	-0.226656633	0.33543
D2HGDH	-0.401867972	0.13681	-0.857443721	0.00164	-0.054553456	0.83833
ANLN	0.015061252	0.94122	0.640833105	0.00167	0.074478051	0.71528
SERINC2	0.003723353	0.9783	-0.431633841	0.00167	0.127063011	0.35251
PITRM1	0.159215302	0.09226	0.295540048	0.00167	0.143308625	0.1294
P11-328L1[illegible]	-0.384168261	0.20303	0.94768821	0.00167	-0.260913551	0.38721
EDRF1	-0.038890503	0.77828	-0.431883068	0.00168	-0.078386005	0.57001
RSPRY1	0.113916706	0.30133	0.344107785	0.00168	0.078931879	0.47406
YBX1	-0.028995863	0.80463	0.367773241	0.00169	0.037727531	0.74752
ZNF430	-0.156128633	0.11101	-0.306486103	0.00169	-0.044424909	0.64867
ATF6B	-0.031094376	0.77408	-0.341015016	0.0017	0.041974168	0.69832
FXN	0.029518573	0.86175	0.518832349	0.00169	0.186225772	0.27044
P11-83M16	-0.250086119	0.46885	0.99045221	0.00169	0.091204812	0.78507
TPX2	0.209153731	0.23012	0.546106662	0.0017	-0.027882953	0.87299
CAPN1	0.125134403	0.32994	-0.403329898	0.0017	0.040653113	0.75166
POLR2K	-0.149151182	0.21856	0.373115043	0.00173	-0.03225633	0.78919
PCF11	0.115022631	0.19305	0.275008034	0.00175	0.189712662	0.0315
SLC22A25	-0.585270198	0.12929	-1.267433177	0.00176	-0.486483047	0.20195
TOMM22	0.05677075	0.6048	0.340049238	0.00176	0.267144645	0.01448
SLC9A8	-0.018975195	0.93269	-0.707651327	0.00177	-0.314153179	0.16167
LMX1B	-0.36114659	0.48974	-1.793636821	0.00177	-0.762411632	0.153
PSMB4	0.083821449	0.31548	0.259062842	0.00179	0.039007012	0.64043
RAD9A	0.007642117	0.96685	-0.592108464	0.0018	-0.023847745	0.89712
SELT	0.126165065	0.379	0.445947275	0.00181	0.250994818	0.07956
PSME2	0.004038203	0.9683	0.313336785	0.00182	0.293214843	0.00364
MAP3K7	0.049529305	0.54609	0.253332114	0.00182	-0.149122636	0.06991
ATP5J2	0.00524271	0.9653	0.372856459	0.00183	0.119266197	0.32096
LCMT1	0.086637551	0.61224	0.526704603	0.00184	0.006796606	0.96817
STEAP2	0.120731017	0.45588	0.501690416	0.00185	0.738394682	4.47E-06
AIMP2	0.036606423	0.78072	0.398378316	0.00186	0.15812806	0.2246
MYEOV	0.119826726	0.721	-1.0542511	0.00188	-0.297918065	0.37643
CNN3	0.186668778	0.21804	0.470572035	0.00189	-0.032257833	0.83155
HSP90AB1	-0.071738466	0.49936	0.32980038	0.00189	0.023258955	0.82662
ITGB3BP	0.208957055	0.29884	-0.626824633	0.00189	0.213537976	0.28799
FAR2P1	-0.31020921	0.21101	-0.776166052	0.00189	-0.193198413	0.43578
HDHD3	-0.182442902	0.15971	-0.403913518	0.0019	0.034497985	0.78937
VDAC2	-0.003470214	0.97208	0.306326448	0.0019	0.187559783	0.05785
RAB8B	0.094719708	0.56271	0.506986724	0.00191	0.069711518	0.67027
ERN2	-0.002297767	0.99396	-0.945760493	0.00193	0.00888868	0.97664
AHDC1	0.368875168	0.13997	-0.781329931	0.00196	-0.136155419	0.58684
G3BP2	0.023931935	0.83432	0.353685068	0.00196	-0.074468803	0.51512
ARPP19	-0.050083495	0.63641	0.327166055	0.00198	0.062603149	0.55458
USP28	0.164426411	0.24254	0.432774089	0.00199	0.188323142	0.18002
CASKIN2	0.0194116	0.93509	-0.745056226	0.002	-0.202379736	0.39696
HSPA1B	-0.278907533	0.08088	-0.493910092	0.002	0.205719064	0.19653

SCHIP1	0.578321537	0.12243	1.153621743	0.00201	-0.166538182	0.65859
ARHGAP27	-0.450511704	0.31751	-1.398500883	0.00202	0.468109955	0.29396
CXCR5	0.884102245	0.66446	-7.028182607	0.00202	0.883487636	0.66467
C5orf15	0.155544213	0.21284	0.383817194	0.00202	0.262316326	0.0353
PSMC2	-0.012731097	0.88251	0.263133722	0.00203	-0.086699566	0.31507
SIGLEC15	-0.529449491	0.11764	-1.115545504	0.00203	-0.10294762	0.75383
VPS26A	0.202901916	0.09459	0.372043671	0.00203	0.423138322	0.00047
ADAM9	-0.134492627	0.33767	0.432054706	0.00205	0.155330721	0.26784
ARID1A	0.246561275	0.19614	-0.589706716	0.00205	0.089387506	0.63978
FSD1L	0.096815383	0.56437	0.50672792	0.00205	-0.253683299	0.13599
RPL37	-0.110321046	0.28792	0.319884764	0.00204	-0.034770946	0.73761
RRAS2	-0.115806232	0.32245	0.354774872	0.00204	-0.022150075	0.8493
TMEM201	0.087370046	0.68308	-0.676165813	0.00204	-0.067457998	0.75361
PAIP2	0.062039338	0.57869	0.341104179	0.00205	-0.211265178	0.05956
AHSA1	0.001373288	0.99264	0.456994029	0.00205	0.288710737	0.05198
TRMT6	-0.056692133	0.65485	0.378124278	0.00206	-0.195419626	0.12535
CICP27	0.01598703	0.93515	-0.619374641	0.00207	0.207314565	0.28655
CCS	-0.272012205	0.1124	-0.528448841	0.00208	-0.371886273	0.03062
C8orf82	-0.166466651	0.27309	-0.468920427	0.0021	-0.240054592	0.11276
VPS33A	0.055969971	0.69048	0.429779121	0.0021	0.059768004	0.67111
STX7	0.281638644	0.05407	0.448167197	0.00211	0.464809289	0.00145
LRP4	0.123379048	0.61075	-0.746283298	0.00213	0.46093986	0.05704
RAB32	0.230593014	0.18682	0.525412269	0.00213	0.171751074	0.32578
TLE6	-0.430871781	0.37815	-1.587476763	0.00213	-0.564810995	0.25277
CHERP	0.301468806	0.07904	-0.535291843	0.00216	-0.092215004	0.59409
STRAP	-0.027142282	0.7713	0.284364369	0.00217	0.010930923	0.90668
RRC37A16	0.243097655	0.12291	0.481193092	0.00217	0.170861141	0.27715
AMN	0.160621966	0.49179	-0.726000578	0.00218	0.309810487	0.183
ZNF189	0.054488221	0.65365	0.368284408	0.00218	0.076310313	0.52901
PQBP1	0.188158322	0.08502	0.329745736	0.00219	0.131982931	0.22642
ATP6V1G1	-0.03869751	0.65736	0.26492135	0.00222	-0.032971812	0.70521
TIPRL	-0.151688976	0.06361	0.244885709	0.00223	-0.07689704	0.34418
ARHGEF7	-0.113785428	0.28102	-0.322563101	0.00224	-0.061227765	0.56158
IFT57	0.039229449	0.77325	0.413116194	0.00224	-0.160588716	0.23846
SLC7A2	0.139797649	0.48388	0.60927173	0.00224	0.265598557	0.18323
CLN6	-0.109682356	0.41199	-0.407450937	0.00224	0.042301069	0.74865
B3GALT6	0.057126579	0.62852	0.353028839	0.00225	-0.074709972	0.52876
RNF14	0.120240446	0.20624	0.285343263	0.00227	0.207660571	0.02835
BLZF1	-0.077426431	0.37388	0.258180637	0.00228	0.013150123	0.87887
DCUN1D2	-0.230058427	0.26911	-0.62931622	0.00228	0.027728048	0.89308
EYA1	0.862091417	0.05547	1.342903381	0.00229	0.776265917	0.08107
PTAR1	0.057059767	0.55516	0.293134581	0.00231	0.178509145	0.0645
TLE2	0.05968657	0.71195	-0.495748294	0.00231	-0.102113758	0.52915
KPNA4	0.108098834	0.22112	0.26800373	0.00232	0.10530787	0.2332
CMPK1	0.040575572	0.76388	0.410599391	0.00233	0.468421627	0.00051
CDC27	0.175519392	0.23367	0.447133759	0.00235	0.093241097	0.52686
CD109	0.255479637	0.39708	0.910645233	0.00238	-0.096186083	0.75224

FBXO38	0.141057526	0.3066	0.417646279	0.00237	0.099323476	0.4713
FLT4	0.059109739	0.91444	-1.809333847	0.00238	-0.638379968	0.25029
CORO1C	0.007105749	0.93084	0.247619689	0.00238	-0.18617724	0.02336
PSMD14	-0.118047187	0.29864	0.341917271	0.00239	-0.056507681	0.61811
NCBP1	-0.104329223	0.2685	0.284014229	0.0024	0.068590679	0.46622
CAAP1	-0.343463385	0.08207	0.589811253	0.00241	-0.123616276	0.5286
AZI1	-0.075051853	0.70378	-0.605716141	0.00244	-0.241100346	0.22454
TPCN2	0.214963752	0.22879	-0.552551329	0.00244	0.027766923	0.87651
BNIP3P1	0.944591512	0.29237	2.698206983	0.00245	0.832898817	0.35364
RPL5P29	-0.48115637	0.35191	1.334688871	0.00245	-0.231320586	0.64309
RBFOX3	-0.149894124	0.7182	-1.350162914	0.00246	-0.538852524	0.19912
C1orf216	-0.193575499	0.30816	-0.575445829	0.00246	-0.233939826	0.22228
PKN2	0.028795006	0.80608	0.354164977	0.00247	0.117276924	0.31685
MRPS10	-0.110002555	0.22473	0.267533072	0.00248	0.030560902	0.73357
NOLC1	0.078186258	0.6587	0.534540046	0.00248	0.12838308	0.46801
EIF3I	0.025999529	0.81914	0.342583314	0.00249	0.108818174	0.33793
SGSM3	0.022577196	0.86477	-0.402199261	0.00252	0.152725212	0.24756
RNF213	-0.019355287	0.91001	-0.517536583	0.00253	-0.032788108	0.84811
TRAPPC12	0.018541275	0.91654	-0.538716212	0.00253	-0.15599516	0.37998
PSMB8	0.181555963	0.29826	0.518592568	0.00255	0.370568387	0.03227
NYNRIN	0.130650077	0.49166	-0.574840736	0.00256	-0.031339245	0.86902
PSMB6	-0.110051233	0.2305	0.271980641	0.00257	-0.043791485	0.63189
ALYREF	0.159211991	0.31332	0.473431863	0.00258	0.246890888	0.11753
ANAPC10	-0.165877899	0.21756	0.383416321	0.00258	-0.099580779	0.45382
LRRTM1	-0.030674133	0.95762	-1.852526569	0.00258	-0.146816614	0.79992
AMMECR1	0.184893866	0.13131	0.365698861	0.0026	0.280731352	0.02159
RNF6	0.168592924	0.18557	0.381174675	0.0026	0.309436471	0.01486
CCDC22	-0.204870195	0.29606	-0.595110436	0.00262	-0.202712751	0.29916
TMEM214	0.10590106	0.4319	-0.406671256	0.00262	0.164472679	0.22196
TSPAN10	0.008729284	0.97717	-0.95405146	0.00262	-0.404380676	0.19193
ASH1L	0.083445423	0.37693	-0.284360334	0.00263	0.133574017	0.15692
PDDC1	0.025111988	0.88585	-0.528290533	0.00263	0.153622431	0.37921
NOA1	-0.061472199	0.60808	0.354854854	0.00265	0.084457135	0.47862
ARID1B	0.157422263	0.16903	-0.345249756	0.00267	0.007134017	0.95041
MRPL47	-0.137793979	0.28687	0.38203637	0.00268	-0.061939132	0.63106
IRAK4	0.024100371	0.94408	1.008044576	0.00269	0.496268026	0.14277
MRPL37	-0.062687546	0.68714	0.464252524	0.00269	0.098109847	0.52772
PRPF38A	-0.014836286	0.85138	0.233716911	0.00269	0.054670494	0.48877
TMEM47	0.120749524	0.55314	0.608165396	0.00269	-0.540845504	0.00831
BMS1P5	0.194046889	0.27476	-0.539031835	0.0027	0.065403287	0.71305
CCAR1	0.185059223	0.15157	0.386360178	0.0027	0.149327042	0.24711
\16c-431H6	0.027914833	0.88543	-0.596670162	0.0027	-0.122789726	0.5285
KIF12	-0.486499653	0.09733	-0.879335988	0.00272	-0.412846112	0.15671
SMS	0.086498879	0.43403	0.33016051	0.00272	0.130066721	0.23905
AKAP8L	0.132086485	0.19756	-0.309751796	0.00274	-0.052067018	0.61266
C14orf79	-0.118325172	0.44846	-0.46813858	0.00274	-0.490427422	0.00185
GYG1	-0.050412211	0.67932	0.357737196	0.00275	0.1474721	0.22298

ACBD5	-0.126517787	0.27876	0.346043762	0.00278	0.177176017	0.12691
AP1G2	0.070371269	0.59272	-0.394506794	0.00278	0.348176205	0.00792
EXOC3L4	0.114533309	0.64485	-0.763510209	0.00278	0.468305284	0.05703
PCNP	-0.126190565	0.12866	0.245202735	0.0028	0.078760778	0.34046
SYTL1	-0.424509429	0.09613	-0.765501156	0.00281	-0.028332897	0.91119
P11-419C5	0.04751726	0.79719	-0.561989498	0.00282	0.396314444	0.0295
UNC5B	0.370599912	0.13194	-0.740151304	0.00286	-0.166596732	0.49997
C19orf77	0.290845483	0.59452	1.60457685	0.00287	0.502210323	0.356
MINK1	-0.068384208	0.62339	-0.416902434	0.00287	0.016640486	0.90485
SAMD8	0.153267911	0.17401	0.334189428	0.00287	0.067277188	0.55276
MALSU1	-0.18765406	0.09936	0.325436656	0.0029	-0.11413255	0.31229
CRAMP1L	0.254122547	0.30363	-0.740815009	0.00291	0.151788836	0.53962
SUGP2	0.040085109	0.71851	-0.331218365	0.00293	-0.164402315	0.13976
SARDH	-0.144648457	0.63379	-0.942234742	0.00294	-0.159082315	0.60385
P11-438J1	0.406971745	0.09591	0.713765013	0.00296	0.699779596	0.00376
MTMR4	-0.109402996	0.3131	-0.320824488	0.00297	0.003639506	0.97309
DDX5	0.059315626	0.52701	0.278044295	0.00299	-0.028650435	0.75993
GABRA2	0.675745402	0.06073	1.066905077	0.00299	-0.770814624	0.03513
PHF2	0.023819867	0.84972	-0.374694179	0.003	-0.075099752	0.55065
RAD23B	0.125002612	0.34512	0.392464383	0.003	0.110889843	0.40212
ZNF257	0.484923001	0.48848	-2.098630152	0.003	0.893680983	0.17842
HN1L	0.118697177	0.32325	0.355438966	0.00301	0.190822734	0.11187
KHDRBS1	0.042541646	0.63259	0.262131047	0.00304	0.076638657	0.38838
PSMC5	-0.030450986	0.76371	0.295326804	0.00304	-0.126824754	0.2103
SAP18	-0.008863961	0.91662	0.248440897	0.00304	0.051207295	0.54383
ABCB9	-0.141570239	0.55464	-0.716612624	0.00305	-0.498227617	0.03905
FAM21A	0.020832064	0.85462	0.332112385	0.00307	0.001191042	0.99162
MIOS	-0.056965368	0.6094	0.325299147	0.00306	0.023748851	0.8303
NTAN1P2	-0.10003606	0.79095	-1.230530152	0.00306	-0.527543939	0.17629
H6PD	0.05493393	0.60147	-0.312763538	0.00307	-0.096840099	0.35822
ZNF646	0.078403719	0.64438	-0.505202064	0.00308	0.000916008	0.99571
BTBD1	0.022305363	0.80317	0.263064425	0.00309	0.091400734	0.30648
FICD	-0.262367371	0.21175	-0.623967625	0.00309	-0.330015836	0.11672
TRIM58	0.578361755	0.11207	-1.229629579	0.00316	0.183266143	0.62158
BCLAF1	-0.028692593	0.74077	0.255293279	0.00316	-0.153171424	0.07713
DNM2	0.127128357	0.3746	-0.422993967	0.00316	0.225302576	0.11524
SLC4A7	-0.038096245	0.77428	0.390344786	0.00317	-0.019624149	0.88237
NOL7	-0.111750585	0.254	0.283458771	0.00317	0.036524088	0.70731
KIF13B	-0.103522372	0.42734	-0.385481094	0.00318	0.112101267	0.38948
BTBD10	0.023710383	0.79548	0.266094529	0.00319	-0.035114656	0.70054
SCIMP	0.287828809	0.18086	-0.695351994	0.00319	0.109887936	0.61623
FOXP4	0.225764593	0.23395	-0.563319656	0.0032	0.131478927	0.48867
ANO6	0.127252988	0.25917	0.331262815	0.00322	0.062807058	0.57759
FUK	-0.036160054	0.8123	-0.449802404	0.00323	-0.105983056	0.48447
DNAJB6	0.044928087	0.65772	0.29698003	0.00325	-0.033372519	0.74226
SNRPD3	-0.148175651	0.17915	0.322218776	0.00326	-0.081029394	0.46127
KRR1	-0.167053098	0.23803	0.414377961	0.00327	-0.161313424	0.25421

UBQLN1	0.140984138	0.22898	0.343313468	0.00329	0.208321456	0.07528
CLDND1	0.069809282	0.43516	0.259773939	0.00333	0.00784279	0.93026
FAM206A	-0.060830484	0.65267	0.387975372	0.00333	-0.080682326	0.54799
SLMAP	0.049368578	0.69731	0.371722972	0.00334	0.219666506	0.08326
ST7L	0.25765823	0.12557	0.487972464	0.00334	0.256086204	0.12759
FAM151A	-0.800182389	0.08826	-1.471409221	0.00335	-0.071454484	0.87174
PROX2	-0.099831031	0.7693	-1.118643419	0.00338	0.050379038	0.87971
HIST2H2BD	-0.820811317	0.4523	-3.909122946	0.00339	-0.274187949	0.79919
KIAA0226L	1.687914392	0.07157	2.675707708	0.0034	0.48875977	0.62476
SLC29A2	0.119269755	0.47257	-0.48950274	0.0034	0.231575238	0.16225
CCT7	-0.069473267	0.50318	0.302742803	0.00341	0.06375298	0.53841
YME1L1	0.019105127	0.80722	0.228112058	0.00342	0.031883879	0.68356
PACS2	0.070797187	0.56863	-0.365138023	0.00343	0.073613431	0.55303
MAP3K6	0.017384419	0.92602	-0.555458545	0.00344	-0.270560351	0.15223
P11-767L7	-0.591908471	0.13679	-1.22751162	0.00344	-0.819096079	0.04353
RAB33B	-0.222310436	0.07549	-0.3627531	0.00345	-0.105202223	0.39843
GJB2	0.538468667	0.10088	0.955744955	0.00345	0.707782349	0.03076
SSU72	0.148578226	0.10113	0.263675614	0.00346	0.109600558	0.2267
P11-503N18	0.150562179	0.5834	-0.871437302	0.00349	-0.193796889	0.49135
PCGF5	-0.062623579	0.6756	0.426411583	0.0035	0.095444735	0.51694
SIGLEC10	0.098168328	0.71756	-0.84550157	0.00351	0.007055086	0.97935
EIF3D	0.002275404	0.98197	0.292672269	0.00353	0.099980196	0.32003
EPB41L5	-0.055508513	0.69668	0.413795225	0.00353	0.018326635	0.89761
PTEN	-0.194107782	0.12303	0.3659366	0.00356	-0.085017013	0.49896
TMEM97	0.22306316	0.18432	0.489109367	0.00357	0.315639712	0.06021
DFFA	-0.088901445	0.39519	0.301964898	0.0036	0.121537604	0.24344
INTS8	0.069173482	0.60939	0.39142488	0.00361	0.240927429	0.07424
ENAH	-0.127906893	0.33797	0.387639546	0.00362	-0.174844855	0.19065
PNPT1	-0.238569525	0.1879	0.524154225	0.00362	-0.031757962	0.86065
RCSD1	-0.019913906	0.95931	-1.208354886	0.00362	-0.266886981	0.50236
P11-848G14	-0.756556482	0.26145	-2.014087155	0.00363	-1.762920047	0.01107
BAG5	-0.075248123	0.48001	0.306994641	0.00364	0.013304652	0.90035
TRIM72	-0.043940407	0.81954	-0.569682704	0.00364	-0.07063471	0.71355
CSNK1G3	-0.098694871	0.20085	0.220798176	0.00365	-0.006925481	0.92819
WDR73	-0.095515269	0.48062	-0.393042559	0.00366	-0.150229208	0.26539
TFCP2	0.033878718	0.793	0.369472926	0.00368	0.079751465	0.53542
FAM120AOS	-0.057651257	0.4963	0.24075713	0.0037	-0.063625123	0.45112
TMEM126B	-0.176861456	0.14134	0.339248427	0.00371	-0.200982416	0.0919
CTNNB1	0.128515464	0.17801	0.276643069	0.00372	0.002954921	0.9753
ACAT2	0.101042861	0.56352	0.506126753	0.00374	0.305727162	0.08018
SEC16A	0.189091666	0.25115	-0.479040346	0.00373	-0.034056971	0.83635
SUMO3	-0.036147531	0.70132	0.270235907	0.00374	0.096631076	0.30285
CLVS1	-0.604750357	0.31895	-1.901852156	0.00374	-0.81220612	0.19412
VPS37C	0.014292876	0.90475	-0.350654498	0.00375	0.134251956	0.25914
PSMA5	0.032105754	0.75642	0.297440107	0.00376	0.081467106	0.43
PODNL1	-0.774600339	0.17023	-1.734031319	0.00377	-0.093505083	0.86813
SGSM2	0.069420542	0.58137	-0.366408826	0.00377	0.037574699	0.76503

GUSBP2	-0.393774202	0.12082	-0.741110598	0.00378	-0.294500521	0.24181
ABHD2	-0.202643796	0.31177	-0.580143291	0.00379	0.405345175	0.04286
ERBB2	-0.031223073	0.82164	-0.40107323	0.00378	-0.044462663	0.74801
DMPK	0.174464806	0.36152	-0.565920919	0.0038	-0.004693518	0.98053
GLG1	-0.02838316	0.7258	-0.234212673	0.0038	-0.097206178	0.22971
MAN1A2	0.002119911	0.98446	0.314033512	0.0038	0.149847034	0.16774
MTMR9LP	-0.230371086	0.35625	-0.734810379	0.0038	-0.401205388	0.10909
ASB14	-0.001713036	0.99582	-0.983010986	0.00381	-0.254575114	0.43468
EVPL	0.266413696	0.34112	-0.820000308	0.00383	0.25687316	0.35912
MRPS11	-0.015006075	0.90773	0.370020502	0.00387	0.071252335	0.58151
TRMT10C	-0.162801535	0.24607	0.398177968	0.00387	0.093459006	0.50274
MRAP2	-0.400920223	0.1395	-0.787808755	0.00389	0.642563639	0.01557
UNK	0.056546948	0.64877	-0.360436114	0.00389	-0.092458253	0.45749
NAA10	-0.019598277	0.84745	0.287672688	0.0039	0.009896656	0.92256
SOCS5	0.20313807	0.16829	0.423164478	0.0039	0.082868277	0.57457
GPT2	-0.241430946	0.15808	-0.492369596	0.00395	-0.229621672	0.17858
ETNK1	-0.223088284	0.1356	-0.430355957	0.00397	-0.061674532	0.67963
ZNF710	0.052315932	0.69117	-0.381378394	0.00397	0.116529764	0.37668
LRP10	0.132185405	0.28362	-0.355938449	0.00397	0.071320501	0.56292
UBXN7	0.186149886	0.19341	0.411369024	0.00398	0.03137282	0.8266
NRBF2P5	-0.278050853	0.62349	-1.913743237	0.00399	-0.46693764	0.4149
ARHGEF11	0.193648581	0.13449	-0.374273838	0.004	0.19228711	0.13729
MTRF1L	0.07351795	0.58622	0.384786232	0.00402	0.16816321	0.21218
CCDC9	0.110854345	0.46291	-0.448651493	0.00402	-0.18769949	0.22411
SH3GLB2	-0.044032997	0.72226	-0.357562365	0.00403	0.198942421	0.10666
SLC35B1	-0.090833814	0.31805	0.257926634	0.00403	-0.107504504	0.23661
TRAK1	-0.105383816	0.4669	-0.416305022	0.00406	0.177607354	0.21918
RAB10	-0.11566612	0.36037	0.362331002	0.00407	-0.058619296	0.64278
RNF2	0.028396925	0.79343	0.308050492	0.00408	0.231648045	0.03161
NOTCH3	0.304566797	0.30062	-0.846899423	0.00409	0.028375243	0.92323
DHX15	-0.112399517	0.10779	0.199501701	0.0041	0.020102182	0.77323
RBM11	0.598579004	0.14928	1.155640872	0.0041	0.633010681	0.12396
PHAX	-0.142225565	0.22483	0.333376997	0.00413	-0.010431897	0.92896
CYP4F31P	-0.090360484	0.90726	-2.594908966	0.00414	-1.227549494	0.13169
ZNF518B	0.158462321	0.15753	0.320330407	0.00415	0.270976011	0.01559
SPACA4	0.519054467	0.16516	-1.257832593	0.00416	0.577287656	0.12022
NUP98	0.167463311	0.11929	0.3072815	0.00418	0.197831799	0.06562
GPR35	-0.21345601	0.34944	-0.656523289	0.00418	0.2596677	0.25213
MYH9	0.040681699	0.75731	-0.377033478	0.00419	-0.110993694	0.39921
HELLS	0.09241999	0.68352	0.647511462	0.00423	0.405817552	0.07319
DNAJA2	-0.077943577	0.36541	0.243796078	0.00424	-0.058565373	0.49601
DNM1P47	-0.533901667	0.10756	-0.953443577	0.00424	-0.270513784	0.41323
DIP2B	0.155729014	0.12354	0.287797057	0.00426	0.068819934	0.49657
RP11-3J10.	0.562989803	0.40634	1.896947307	0.00426	-0.785022711	0.26799
AQP7	-0.500979524	0.27597	-1.332445406	0.00437	-1.009143459	0.03092
TOB2	0.086477413	0.38421	-0.284381832	0.00436	0.039696328	0.68955
HNRNPA1	-0.063206106	0.43885	0.232539785	0.00437	0.028195681	0.72978

SFRP5	-0.637254165	0.233	-1.552999141	0.00438	-1.194491314	0.02731
XPOT	-0.00318526	0.97819	0.330318591	0.00439	0.164186751	0.15789
TAF6	0.046580697	0.67396	-0.318102115	0.0044	-0.084443356	0.44884
RPS11	-0.150267078	0.15366	0.299623627	0.00441	0.007979365	0.93959
HDGFRP3	-0.080026894	0.49276	0.330569797	0.00444	-0.332524536	0.00446
BCAS3	-0.165726014	0.40518	-0.568142728	0.00444	-0.065144231	0.74452
ZCCHC9	-0.1226443	0.32413	0.348990303	0.00444	0.012263612	0.92114
BRK1	-0.060553842	0.37397	0.190984044	0.00445	0.039556239	0.55934
CASK	-0.052175303	0.71035	0.398358782	0.00448	0.265192433	0.05886
DLG3	0.105343045	0.43398	0.380840396	0.00448	0.275440162	0.04009
[illegible]OLGA2P[illegible]	0.130160896	0.21093	-0.296450877	0.00449	0.024861583	0.81127
RAB2B	-0.086886043	0.59298	-0.464964629	0.00449	-0.100941991	0.53417
PLEKHA3	0.241307258	0.08482	0.396249848	0.00452	0.099092723	0.4791
ANO8	0.26836262	0.17062	-0.571947072	0.00456	0.174737828	0.37409
REEP1	-0.573145944	0.07091	-0.905594443	0.00457	-0.075210866	0.81037
DTX3L	0.051127214	0.60497	0.278229855	0.00459	0.198451529	0.04395
CTNNA2	-1.113509412	0.08725	-1.956628933	0.00461	-1.208293755	0.06691
[illegible]11-752G1[illegible]	0.013520415	0.98031	-1.826948652	0.00464	-0.41856899	0.45895
EIF5A	0.143480554	0.228	0.336380452	0.00464	0.232405462	0.05072
ATE1	0.138590198	0.33878	0.407771596	0.00466	0.356809842	0.01357
C20orf196	-0.183538619	0.45847	-0.710682157	0.00466	-0.223511115	0.36214
MOCS2	-0.111450215	0.30973	0.30391703	0.00467	0.046980999	0.66649
HIF3A	-0.115119083	0.7183	-0.908690738	0.00468	0.131623832	0.67982
AQR	-0.118415875	0.32926	0.341749011	0.0047	-0.029587203	0.80712
ELOVL6	0.01232527	0.92984	0.394585401	0.00469	0.291766846	0.03676
[illegible]NRNPA1P[illegible]	0.337910871	0.59141	1.675782533	0.00469	-0.632959633	0.34946
PAPOLA	0.065051334	0.42291	0.228560323	0.00472	0.143371397	0.07687
STAG3L5[illegible]	-0.220174832	0.1932	-0.480493031	0.00472	-0.034508827	0.83675
[illegible]DHHC8P[illegible]	0.131380388	0.71451	-1.069318788	0.00471	-0.459185047	0.21507
FOXRED2	-0.010015487	0.96307	-0.616971477	0.00473	-0.167587447	0.44071
NOMO1	0.388293431	0.1717	0.80124991	0.00473	0.373831106	0.1882
SWSAP1	-0.148208919	0.48186	-0.610221093	0.00473	-0.330179625	0.12138
ZNF579	0.026593663	0.90879	-0.676300519	0.00473	-0.083052659	0.72152
EMC7	-0.079169652	0.65899	0.501628548	0.00474	0.236382802	0.18549
SLC27A1	0.106410455	0.46127	-0.41713335	0.00474	-0.403373085	0.00601
PLXNA2	0.340816979	0.08337	-0.557488568	0.00476	0.111423416	0.57164
ZNF467	-0.002266998	0.99447	-0.973122428	0.00477	-0.088531202	0.78881
MBD4	0.05896424	0.63389	0.345031128	0.00479	0.102995955	0.40245
FAM8A1	0.017341107	0.87923	0.319222818	0.00479	0.024318529	0.83108
RRP36	0.013458065	0.88681	0.261789805	0.0048	0.069410627	0.46138
B3GNT3	-0.303235415	0.08931	-0.503158814	0.00483	0.011555986	0.94828
HDAC2	-0.003179388	0.97552	0.290781254	0.00483	0.079588546	0.44188
[illegible]C012358.[illegible]	-0.135450619	0.77063	-1.474257108	0.00485	-0.697104967	0.15133
CLCN3	-0.061992552	0.70358	0.458200055	0.00485	0.029520094	0.85614
FAM161A	-0.369983563	0.0798	-0.591252954	0.00486	-0.643705224	0.00257
MFSD10	0.0268937	0.88184	-0.511640305	0.00485	-0.098432667	0.58666
ATP1B1	0.289163501	0.08759	0.476219192	0.00489	0.089698374	0.59624

T6GALNAC	0.212109673	0.15065	0.412803772	0.0049	0.184884122	0.20958
ISPD	-0.408992047	0.07626	-0.642985547	0.00495	-0.426433218	0.06244
KHNYN	-0.027468941	0.77786	-0.273701298	0.00496	-0.037727706	0.69821
COMMD2	-0.019277081	0.85276	0.287681133	0.00498	0.182282132	0.07772
HSPA9	0.099761972	0.52017	0.435155948	0.00498	0.306308044	0.04822
NNMT	0.939060307	0.10802	1.635613387	0.00501	-0.101374802	0.86317
CBWD5	-0.076708147	0.57836	0.383442176	0.00501	0.059852448	0.66389
UGT1A6	0.241535982	0.72195	1.800955532	0.00506	0.611290162	0.36371
LAYN	0.558501455	0.05279	0.797039637	0.00507	-0.417364272	0.16674
OSBPL7	-0.164243956	0.48026	-0.658609146	0.00507	0.213412404	0.35717
EXOC6	-0.150633533	0.40117	0.49724823	0.00514	0.124477292	0.48518
PARP9	0.15090449	0.23389	0.352601072	0.00514	0.176095655	0.16437
RASSF10	0.221383412	0.39804	0.721267107	0.00513	0.877136075	0.00066
TMC6	-0.090899502	0.58043	-0.463101808	0.00514	0.004584966	0.97773
C12orf5	0.171578764	0.42463	0.59580427	0.00515	-0.026680867	0.90158
RPL12P38	0.030901076	0.8547	0.466447103	0.00517	0.084838995	0.61433
SLCO2A1	0.39606126	0.18544	-0.84016817	0.00517	0.606989179	0.04226
APPL1	0.00633853	0.94128	0.238610943	0.00517	0.25324292	0.0031
RWDD1	-0.132390542	0.20613	0.28840837	0.00518	0.11289692	0.27824
LAMA5	0.094415224	0.62163	-0.536793574	0.00519	-0.04956468	0.79568
WDR52	-0.305684238	0.12196	-0.552029721	0.0052	-0.093834835	0.63412
KPNA1	0.130705384	0.20211	0.285079951	0.00521	0.194480949	0.0574
ZNF395	-0.139259332	0.38548	-0.448430264	0.00521	-0.127425762	0.42707
C1orf172	-0.304013551	0.41584	-1.051481117	0.00523	-0.229323501	0.53882
SEMA4D	-0.090928256	0.5964	-0.481093136	0.00524	0.159398956	0.34856
ICAM4	-0.287767812	0.54467	-1.55519145	0.00526	-0.151463392	0.74504
KAT5	-0.158659285	0.23756	-0.372750889	0.00527	-0.069629395	0.60181
DPP4	0.815568583	0.09386	1.357424305	0.00528	-0.192105224	0.69348
FAM198B	0.001396365	0.99527	0.655838698	0.00529	-0.341169311	0.148
MAGT1	0.008377428	0.98521	-1.348784642	0.00529	-0.418470689	0.36288
SAFB2	-0.047391544	0.62565	-0.272241873	0.00529	-0.00679355	0.9441
ARHGEF16	-0.129913687	0.31796	-0.363284195	0.0053	0.145746015	0.25728
C6orf141	-0.286728542	0.16464	-0.576963164	0.00531	0.299541688	0.14313
HLA-DRB5	1.255831504	0.0711	1.935114901	0.0053	0.526371689	0.45082
MAP3K10	0.243932688	0.2812	-0.656747293	0.00531	-0.038719153	0.86558
TCEAL4	-0.127603021	0.41106	0.429771829	0.00532	-0.235998207	0.12872
CEP85	-0.097933661	0.43482	-0.346413818	0.00532	-0.173399284	0.16528
BRSK2	0.292930566	0.36359	-0.952880541	0.00533	-0.079676473	0.808
SDK2	-0.234876332	0.64466	-1.437970762	0.00533	-0.769025545	0.13301
ASPA	-0.003857945	0.98689	-0.675940981	0.00534	-0.261131593	0.27237
DNAJB11	-0.015353349	0.90082	0.34102364	0.00535	0.002885155	0.9813
CSPG4P12	0.249050596	0.25634	-0.617895184	0.00536	-0.057635368	0.79348
CHCHD2	-0.070131709	0.5363	0.313065398	0.00541	0.137061909	0.2251
P11-644F5.	-0.030276081	0.89943	-0.684364871	0.00544	0.007258616	0.97588
SMIM15	-0.177578697	0.11765	0.312951598	0.00545	0.155840088	0.16784
WDR43	-0.316695231	0.07227	0.48736334	0.00545	-0.229336513	0.19217
ZNF562	0.00622018	0.96949	-0.454658103	0.00546	0.0520254	0.74929

C11orf80	0.313168569	0.12713	0.562856562	0.00549	0.334194086	0.10152
PA2G4	-0.189050707	0.33422	0.542747329	0.00549	-0.066014629	0.73585
ABCA10	0.758395099	0.18631	1.581351496	0.00552	0.439164057	0.44265
SMARCE1	0.040889809	0.69571	0.289027544	0.00554	-0.113087117	0.27966
LRRC19	0.789857875	0.10281	1.340064017	0.00554	0.303790975	0.53123
CDH26	-0.546499071	0.0991	-0.92039212	0.00559	-0.174926712	0.59684
CLEC18A	0.028374974	0.94606	-1.26991706	0.00559	0.009173744	0.98259
FRRS1	-0.516101327	0.06841	-0.783866757	0.00559	-0.081122067	0.77372
KLHL35	-0.325139403	0.48714	-1.348988446	0.00563	-0.587860133	0.20827
PTPLB	0.079183741	0.3795	0.247644293	0.00563	0.248722444	0.00555
EIF2B4	0.415230895	0.06537	0.620424435	0.00567	0.249821949	0.2689
TMEM33	0.095298285	0.36119	0.287846544	0.00566	0.102607476	0.32505
ZNF266	-0.099125059	0.37561	-0.307254578	0.00568	-0.037837003	0.73367
C17orf80	0.064307901	0.55562	0.296809138	0.00569	-0.063535858	0.56066
PI4KA	0.083240827	0.58784	-0.425631264	0.00569	0.094427973	0.53878
DHX34	0.121554429	0.32652	-0.345685114	0.00572	0.006495813	0.9584
ATG10	-0.252134121	0.11035	-0.429751598	0.00575	-0.132707971	0.39778
SYNE3	-0.077782045	0.73906	-0.665921306	0.00577	0.023423637	0.91937
ARL6IP5	-0.054138085	0.61732	0.297481468	0.00578	0.048224431	0.65572
MEGF6	-0.110510501	0.68511	-0.760787077	0.00581	-0.493686785	0.0717
SARM1	0.145314574	0.63962	-0.864844628	0.00583	-0.046644625	0.88105
UTP15	-0.127523633	0.36663	0.382445906	0.00584	-0.125068394	0.37611
ANKS3	-0.108967635	0.55938	-0.519823413	0.00585	-0.051568006	0.78187
B2M	0.244245261	0.07971	0.38355771	0.00591	0.390467078	0.00508
CCZ1	-0.146809082	0.10095	0.242682578	0.00591	-0.100515289	0.26012
UBE2I	0.075389304	0.54542	0.341481696	0.00598	0.033011368	0.7914
ZNF440	-0.02035228	0.87361	-0.35080674	0.00598	-0.074485173	0.55821
LIMA1	0.09697831	0.27951	0.245949625	0.006	0.249874947	0.00528
MTHFD2P1	-0.208582988	0.69912	-1.560433348	0.00602	-0.014237813	0.97878
NRDE2	-0.260990518	0.09538	-0.429369747	0.00602	0.036869672	0.81312
CD58	0.082511604	0.63882	0.473434609	0.00602	0.125880939	0.47308
HSPA8	-0.08492501	0.56331	0.403131815	0.00607	0.112689167	0.44312
RP4-740C4.	-0.416870224	0.16986	-0.867449653	0.00607	0.07057915	0.80412
ATP5J	-0.080674126	0.39319	0.254941071	0.00612	0.135099919	0.14931
C1orf64	0.038970883	0.92066	-1.205788216	0.00612	-0.204333805	0.60776
COPS6	0.104514633	0.27035	0.256188612	0.00611	0.022650544	0.81127
CYP2C19	-0.284640284	0.24015	-0.665790312	0.00612	0.286235624	0.23524
ZNF605	-0.196091914	0.13873	-0.362333278	0.00612	-0.160118779	0.22567
KIAA0319L	-0.153249297	0.18477	-0.316066605	0.00617	0.136215574	0.2368
TMEM143	-0.220128567	0.37196	-0.687991534	0.00618	-0.007883888	0.97424
C5orf28	-0.118376001	0.4626	0.425885726	0.0062	-0.389905897	0.01548
GIGYF1	0.080806831	0.52673	-0.35154821	0.00623	0.067195211	0.59839
UBE2H	0.253376695	0.07258	0.385456689	0.00624	0.21564227	0.12661
AHRR	0.462627594	0.23186	-1.092770899	0.00625	-0.551070335	0.17161
P11-44F14	0.123691027	0.71855	-1.02200224	0.0063	-0.016183712	0.96254
WDR62	0.103671652	0.65241	-0.63386798	0.00629	-0.201372842	0.38263
RIPPLY3	-0.339293264	0.27378	-0.886180587	0.0063	-0.04521684	0.87976

TARS	0.059875237	0.64766	0.356999989	0.00635	0.083829643	0.52214
PTRH2	-0.005541954	0.97196	0.426262676	0.00636	0.077257713	0.62362
C11orf48	-0.067541616	0.54732	0.3034944	0.00636	-0.003246461	0.97685
SFR1	0.116860858	0.47387	0.432004801	0.00637	0.198385501	0.21839
CCDC85C	-0.261413927	0.08786	-0.417805389	0.00638	0.004126284	0.97846
AXDND1	-0.580808933	0.3252	-1.665515013	0.0064	0.68608572	0.22677
COPB2	-0.040816531	0.60429	0.213617831	0.00643	0.051644812	0.51148
EIF2D	-0.152784717	0.1376	0.27629375	0.00643	-0.024262182	0.81273
PTMA	0.077236335	0.46071	0.285197772	0.00643	0.085493417	0.41415
SCO2	0.171120916	0.22241	0.371216807	0.00643	0.273463321	0.0485
POLE	0.129754915	0.38267	-0.405670828	0.00644	-0.108492663	0.4657
DACH2	-1.213125412	0.11442	-2.243976296	0.00646	-0.445667107	0.53856
BCAS2	-0.165416932	0.07611	0.247603721	0.00647	-0.015459071	0.86743
RAP2A	-0.02820837	0.70692	0.201362942	0.00649	0.090160136	0.22669
HECTD4	0.185433425	0.21523	-0.408539582	0.0065	0.03631283	0.8084
KCTD20	0.03174361	0.72901	0.247348677	0.0065	0.080656404	0.37901
SCN9A	-0.317470631	0.14797	-0.596273477	0.00654	0.179210084	0.41199
ATF6	-0.095919914	0.31981	0.260772045	0.00655	0.060202471	0.53221
CLPTM1	0.031513855	0.80827	-0.353845509	0.0066	0.03953183	0.76095
TPM3	0.142916653	0.24742	0.335297559	0.00661	0.223179823	0.0708
CXorf36	-0.565263209	0.133	-1.038136276	0.00663	-0.133401558	0.71476
ZNF334	-0.218056798	0.36886	-0.662162742	0.00663	-0.248886459	0.30367
RGS17P1	-0.414947403	0.25413	-1.052091488	0.00664	0.088579316	0.79606
TRMT1L	0.14414935	0.41963	0.481605154	0.00664	0.290589959	0.1027
LEPROT	-0.426008399	0.18722	-0.877096233	0.00669	-0.242556939	0.45247
MCUR1	-0.005100721	0.97113	0.380579591	0.00673	0.025712703	0.85506
MAP3K14	0.034518884	0.8382	-0.469747391	0.00674	-0.008579742	0.96008
GPR180	0.063486721	0.48662	0.244454021	0.00675	0.098406763	0.27955
RABL2A	-0.055025552	0.77205	-0.521046536	0.00675	-0.016122967	0.93231
SH3BP2	0.157490279	0.35092	-0.458896346	0.00675	-0.104784161	0.5363
P1-179N16	0.160886149	0.75039	-1.752650909	0.00678	0.362821073	0.46329
MAPRE1	0.125164305	0.23545	0.284264433	0.0068	0.010982268	0.91716
MYH8	1.052732377	0.39016	3.199848134	0.0068	2.316716989	0.05223
PSD4	-0.187652506	0.24238	-0.4355213	0.00681	0.049189758	0.75913
P11-234B24	0.413332387	0.12007	0.7113203	0.00682	0.136892233	0.60904
IPO7	-0.15239263	0.20591	0.325418097	0.00684	-0.080282339	0.50499
DCAF6	0.046146509	0.70982	0.333293286	0.00691	0.264941482	0.03213
RSPH1	0.563559188	0.05856	0.794996532	0.00691	0.978588289	0.00081
EDF1	0.08875286	0.31325	0.236531098	0.00692	0.128931355	0.14234
PFDN6	-0.159628815	0.17698	0.308731686	0.00693	0.082530841	0.47795
ARL6IP1	0.152787556	0.17378	0.302623911	0.00695	0.189469287	0.09144
ARAF	-0.023971487	0.84686	-0.337247774	0.00696	0.004883512	0.96866
RSF1	0.107843341	0.29762	0.278591636	0.00697	-0.086908958	0.40118
SH2B1	0.255042036	0.19615	-0.536601226	0.00697	0.266546658	0.17628
VAPB	0.090574107	0.37676	0.274084361	0.00699	0.202094235	0.04773
EPB41L4B	0.037048136	0.88746	0.700143313	0.00701	0.076865549	0.76881
IFT172	-0.188755629	0.23885	-0.430286068	0.00702	-0.145794101	0.36049

CAMSAP3	0.189743248	0.30071	-0.500041827	0.00704	0.173044778	0.34484
DNAJC7	0.033016518	0.74312	0.269182702	0.00705	0.091600229	0.36223
NAA50	0.227587803	0.13225	0.405567728	0.00706	0.418860227	0.00548
FABP3	0.57480959	0.05375	0.798769953	0.00708	0.021395319	0.94328
BOD1	-0.018041736	0.85249	0.257111628	0.00709	0.106903408	0.26728
P11-350D1	-0.232488954	0.30889	-0.624589034	0.00714	-0.28035581	0.21983
EPS8L2	-0.16406724	0.36158	-0.483873616	0.00716	-0.226828912	0.20665
ERAP1	0.134762033	0.40234	0.431762595	0.00721	0.387035409	0.01603
DNAJB13	0.099891916	0.73306	-0.82184377	0.00721	0.202491676	0.48608
STK38	0.139458569	0.0967	0.224124323	0.00724	0.265284342	0.00151
PELP1	0.138635741	0.43475	-0.478152197	0.00727	-0.091509041	0.60682
MTMR3	0.045489166	0.67148	-0.288716576	0.00728	0.082570175	0.44074
GNAI3	0.071324122	0.24591	0.1638209	0.00735	0.026312734	0.66856
HSPA9P1	0.547458164	0.05888	0.755909539	0.00738	0.380343843	0.1936
KCNJ14	-0.038463779	0.88924	-0.792788032	0.00738	0.03173802	0.90745
TULP3	0.231578299	0.09475	0.369785296	0.00736	-0.014013717	0.91964
UQCRFS1	-0.035252295	0.71929	0.260506137	0.00738	0.211259123	0.03032
ZBTB40	0.07825784	0.40148	-0.250517939	0.00736	-0.001460181	0.98749
ZBTB45	0.328899782	0.09138	-0.539467483	0.0074	0.023851977	0.90384
TMEM180	-0.168148926	0.46083	-0.613121216	0.00743	-0.304924802	0.17354
BAG4	-0.052177707	0.56596	0.239384153	0.00744	0.041935787	0.64297
CSTF2	0.022444507	0.87662	0.378452009	0.00744	0.321824674	0.02437
PSMC3	0.052387142	0.61857	0.279965907	0.00746	0.097609546	0.35328
LCA5	0.063656249	0.67718	0.403455292	0.0075	-0.144462259	0.347
P11-484D2	-0.430864109	0.39418	-1.55711482	0.0075	-0.229932967	0.64
DYNC1LI1	0.061970043	0.63935	0.349791976	0.00751	-0.057846152	0.66108
FAM32A	-0.037472006	0.60353	0.189723382	0.00756	-0.068844636	0.33919
ZNF419	-0.129903773	0.4182	-0.430405107	0.00764	-0.441643232	0.00654
C22orf39	0.10199043	0.36451	0.295592568	0.00765	0.271546984	0.01555
MRPS15	-0.12627848	0.33899	0.349169594	0.00769	-0.071348122	0.58825
BCL2L15	0.690138942	0.08541	1.068248727	0.0077	1.157108685	0.00389
TBCB	0.190387701	0.07991	0.288270101	0.0077	0.121211063	0.26784
LRRC8D	-0.0091171	0.94866	0.371650161	0.00771	-0.172717324	0.22202
EDC4	0.154850336	0.24162	-0.35299551	0.00773	-0.13091	0.32381
NELFA	0.037730553	0.81955	-0.443469313	0.00777	-0.248894174	0.13322
VMP1	0.220557553	0.06094	0.313251144	0.00777	0.187966074	0.11043
UQCRC2	-0.111653967	0.25833	0.2614749	0.00779	0.194184257	0.04862
LDLRAD2	-0.990005258	0.05855	-1.373190258	0.0078	-0.683783297	0.17466
PCSK7	0.169381222	0.15234	-0.316123342	0.0078	0.22074565	0.06199
RRN3P1	-0.017065433	0.92184	-0.46791938	0.00781	-0.250726007	0.15343
NOXA1	-0.123919651	0.59105	-0.626323078	0.00784	0.312837921	0.16465
ATAD2B	-0.148659827	0.235	-0.331708007	0.00786	-0.150854748	0.22798
KCNK6	0.156839076	0.40066	-0.498842371	0.00786	0.018381758	0.92144
P4HB	0.071190045	0.66415	0.43573364	0.00785	0.107863374	0.51059
PPP2R1B	-0.068848338	0.5705	0.320628306	0.00785	0.135426321	0.26349
COA3	-0.068777247	0.46012	0.240387163	0.0079	0.049679955	0.58988
ME2	-0.117558315	0.45999	0.420825265	0.0079	0.144645364	0.36251

SNRPC	-0.059687339	0.6007	0.299488123	0.00791	-0.01385318	0.90309
MFSD11	-0.204247275	0.14341	-0.368561085	0.00797	0.01110754	0.93582
KPNA2	0.09572548	0.50356	0.378821929	0.008	0.037829236	0.79152
UBXN1	0.13767988	0.34491	0.384214701	0.008	-0.002208308	0.98792
DBR1	-0.238275589	0.17542	0.459560406	0.00801	0.209790483	0.22902
CLK2	0.159080832	0.09188	-0.251749337	0.00802	0.048770299	0.60599
PCDHA9	-0.086018428	0.69981	-0.601002441	0.00802	-0.196985436	0.37855
DNAJC21	0.01185662	0.88968	0.224293677	0.00803	0.045577397	0.59226
ZNF142	0.264207958	0.19277	-0.539614598	0.00806	-0.209888475	0.30197
KDM2B	0.0463747	0.77156	-0.425565246	0.0081	-0.358572018	0.02575
CHPF2	0.063222989	0.57409	-0.298607867	0.00813	-0.193164542	0.08699
ONECUT1	-0.126237034	0.77079	-1.15777928	0.00813	0.550658774	0.20279
DCTN6	-0.113058674	0.1826	0.218129555	0.00815	-0.100303928	0.23321
PRKRIR	-0.211347141	0.17467	0.410560688	0.00815	-0.264144708	0.08971
RP11-2I17.[illegible]	0.510281781	0.13747	-1.063529251	0.00816	-0.565778747	0.13844
AC024560.[illegible]	0.051681934	0.71039	-0.371303777	0.0082	0.296361071	0.03149
FAM106A	0.232959762	0.52307	-1.080660506	0.00822	-0.044062167	0.90559
RXRA	0.122812526	0.30724	-0.318922295	0.00821	0.090236242	0.45177
WDTC1	0.237909526	0.05858	-0.337301574	0.00822	0.137649139	0.27553
PSMD13	-0.060268703	0.45649	0.210497464	0.0083	0.052205681	0.51627
UTP3	0.03220591	0.81432	0.35672565	0.00833	0.025071345	0.85474
UBE2Q2P[illegible]	-0.217106986	0.06191	0.306238313	0.00834	-0.04290992	0.71196
ATXN10	-0.159701369	0.18862	0.317711257	0.00837	-0.081189477	0.50269
EML3	0.025169441	0.90185	-0.542027204	0.00837	-0.031736216	0.87636
KLHL23	-0.264314673	0.06528	0.375260034	0.00838	-0.012665498	0.9293
DDX51	-0.002593796	0.99012	-0.555276217	0.00842	-0.200348307	0.34033
PWWP2B	-0.155399089	0.49113	-0.600737967	0.00844	0.159274097	0.47499
GKN2	2.580671878	0.05709	-3.842271958	0.00851	0.191772904	0.88805
RPS6KA3	0.005050221	0.95876	0.255918499	0.00853	0.256253341	0.00852
PPP2R4	0.103447301	0.32522	-0.276686638	0.00854	-0.042579726	0.68591
C19orf52	-0.317800475	0.10268	-0.508661828	0.00863	-0.401228517	0.04082
NIM1K	-0.073208297	0.8654	-1.204303623	0.00864	-0.032667536	0.93946
GBF1	0.083257544	0.43322	-0.279514759	0.0087	-0.052973442	0.61842
[illegible]LC25A39P	-0.30678459	0.53164	-1.395815129	0.00871	-0.246797708	0.61224
GMFB	-0.034900965	0.6821	0.221338251	0.00872	0.014758041	0.86217
USP39	0.238991249	0.13377	0.415539404	0.00875	0.123166833	0.43992
SMIM22	-0.240508181	0.19774	-0.491335168	0.00879	0.179947637	0.32926
ATP5B	-0.03728725	0.77241	0.337368432	0.00881	0.130761982	0.31022
PCDHA2	-0.225054339	0.44331	-0.757684473	0.00882	-0.057754357	0.84215
C5orf51	0.056769071	0.48582	0.210622008	0.00884	0.003784955	0.96293
PPP5C	-0.052699648	0.59602	0.257501821	0.00885	-0.077808528	0.43401
CDK8	0.013509376	0.93471	0.428651104	0.00887	0.117315594	0.47595
EEA1	-0.087819396	0.4818	0.325346319	0.00891	0.226911684	0.06876
FRMPD3	-0.115863786	0.79989	-1.280678886	0.00892	-0.626099982	0.1906
SEC14L1	0.30620985	0.06191	0.429711698	0.00892	0.385630656	0.01895
UBE2J1	0.010703274	0.9062	0.235831814	0.00892	0.158011303	0.08082
PUS3	0.031541603	0.8734	0.508186806	0.00893	0.011775645	0.95261

KIAA1430	0.043395567	0.69968	0.291928835	0.00893	-0.079962099	0.47648
LEO1	0.000344283	0.99693	0.229485552	0.00894	-0.014945324	0.86696
HCCS	-0.014221327	0.91332	0.33223221	0.00898	0.14451222	0.26387
BAG6	0.208422583	0.10413	-0.335685371	0.00901	-0.033645496	0.79335
ZNF800	-0.042280204	0.81317	0.464125654	0.00903	0.235562891	0.18618
KCNK1	-0.049355611	0.8136	-0.548151483	0.00904	-0.153750729	0.46228
SLC12A7	0.406573421	0.16286	-0.7693275	0.00904	-0.006872147	0.98128
FBL	-0.099945158	0.45382	0.34404328	0.00908	0.064663549	0.62638
EEF1A1P12	0.023848279	0.8981	0.468153745	0.0091	0.307148179	0.09213
HSD11B1L	-0.215251822	0.34456	-0.602719384	0.00911	0.215523876	0.33264
NPY5R	0.045899644	0.94786	-2.083496471	0.00914	-0.940799611	0.21332
BEX4	-0.048182311	0.68669	0.307456138	0.00916	0.146259047	0.21837
TADA2B	-0.115194952	0.42373	-0.375321432	0.00919	-0.086991526	0.54518
AC093162.5	0.227077404	0.65042	-1.609886214	0.00921	0.718110005	0.13613
CNST	-0.119023587	0.30428	0.296321854	0.0092	-0.058754874	0.61125
TRMT11	-0.18180715	0.18743	0.349736954	0.00923	0.151432805	0.26816
CEP250	-0.180618027	0.24526	-0.405086603	0.00925	-0.276193184	0.07579
ITGA8	-0.191824986	0.3753	-0.562826847	0.00932	-0.426424248	0.04889
NUDT5	-0.268894111	0.05892	0.367157483	0.00932	-0.116545833	0.41237
MTSS1L	-0.158146899	0.40212	-0.491611466	0.00934	-0.200108236	0.29107
SNAP23	0.178255277	0.15543	0.324519788	0.00934	0.222806899	0.07572
MICAL2	0.224368473	0.30484	0.567629307	0.00939	0.331907239	0.129
NPRL3	0.319696176	0.31741	-0.881235754	0.00939	0.338582404	0.2979
COX5B	-0.046335881	0.67838	0.287067443	0.00944	0.150317725	0.17626
FUT11	-0.146645974	0.33041	-0.391127366	0.00946	-0.12476924	0.40732
MFSD9	-0.027238763	0.87419	-0.445127644	0.00945	-0.108656662	0.52629
SH3RF2	-0.37178301	0.0608	-0.514378678	0.00944	0.154287443	0.43497
UTP23	-0.138665644	0.3417	0.372672943	0.00944	0.00810558	0.95555
SDCCAG8	0.230272545	0.50143	0.875178541	0.00947	0.77661763	0.02132
AFAP1	0.181497437	0.22723	-0.390986163	0.00947	-0.168568186	0.26337
TGIF1	0.209769088	0.09295	0.321714424	0.00948	-0.031931883	0.79866
CRKL	-0.122473192	0.15566	-0.223570836	0.00949	-0.169384256	0.04951
TMEM170B	0.002117127	0.98842	0.371674279	0.00952	0.375093603	0.00913
COPS8	0.088201059	0.43039	0.28712414	0.00956	-0.05498291	0.62353
CLK4	-0.102742684	0.55867	-0.454823795	0.00962	-0.163532836	0.35194
NIPA2	-0.068908962	0.4427	0.229714741	0.00962	0.097897627	0.27277
MTHFD2L	-0.175555399	0.29173	0.417640614	0.00964	0.039487034	0.80923
NR4A2	-0.258267575	0.47375	-0.933766432	0.00964	-0.26530506	0.45529
RNF4	0.138934073	0.09448	0.214018202	0.00966	-0.015532332	0.85217
MCOLN1	0.226893671	0.22267	-0.489809989	0.00968	-0.136613815	0.47251
TAS2R12	0.421045515	0.33663	-1.369597538	0.00975	0.229612768	0.60455
TMEM70	-0.062912257	0.64388	0.340912442	0.00975	-0.237874543	0.07854
LYRM4	0.227188703	0.05917	0.308716198	0.00977	-0.050694422	0.67563
SEC63	-0.071852365	0.63191	0.385758412	0.00978	0.298303297	0.04637
CELSR1	-0.161342587	0.44943	-0.551462303	0.0098	-0.108336544	0.61085
HSPD1	-0.252403612	0.09026	0.384488228	0.00981	0.029674129	0.84208
METTL1	0.048524887	0.80461	0.489978153	0.00982	0.042940692	0.82582

NAA38	-0.121371254	0.41111	0.37891864	0.00983	0.050615348	0.73083
PLA2G4F	-0.636715621	0.08911	-0.958910677	0.00982	0.176995239	0.62145
CLGN	0.032954084	0.89117	0.598895917	0.00985	-0.292444853	0.23292
METTL21EI	0.121497764	0.79658	-1.46562336	0.00985	-0.274063857	0.57248
PIK3CA	0.181135988	0.26739	0.419669977	0.00992	0.203918415	0.21173
C21orf33	-0.2044614	0.75023	-1.700105703	0.00993	0.175691622	0.78257
ACER3	0.181083176	0.27289	0.424758042	0.00993	0.182594625	0.26856
MOV10	0.074815144	0.51697	-0.298237377	0.01001	-0.01139214	0.92142
SCAF1	0.334918263	0.12024	-0.560682936	0.01002	-0.099963346	0.64419
CYB5RL	-0.15124832	0.34196	-0.410528655	0.01006	-0.068467065	0.6647
TUBA1B	0.228508243	0.11973	0.377848321	0.01007	0.121149227	0.40944
ZACN	-0.707135447	0.21626	-1.490356202	0.01007	-1.559466834	0.00736
KLHL8	-0.173976162	0.09077	0.257764847	0.01008	0.046819647	0.64454
ATOH8	-0.091843956	0.78535	-0.881874207	0.01011	-0.683610878	0.04805
ZNF550	-0.105620847	0.46496	-0.371790781	0.01015	-0.18772858	0.19513
SGK3	-0.230543673	0.22408	-0.484212418	0.01017	-0.190341073	0.31146
TBX6	-0.336617576	0.21121	-0.706656847	0.01019	-0.028540414	0.91299
EEF1A1P1	0.831615648	0.1428	1.384869882	0.01021	1.654638587	0.00199
GNL2	-0.125420711	0.3767	0.362294608	0.01023	-0.183573481	0.19535
TMEM150A	-0.166520712	0.30919	-0.416835227	0.01023	-0.305263669	0.06151
OLAH	-0.125937771	0.82071	-1.601369913	0.01029	-0.890947499	0.13288
P11-176H8	0.41470558	0.13965	-0.73031935	0.01028	0.110380691	0.69513
SLC22A15	-0.521224366	0.18789	-1.007600824	0.0103	-0.601083079	0.12182
LTK	-0.540080204	0.27573	-1.321930836	0.01032	-0.33218643	0.4967
CYP51A1	0.13198329	0.33349	0.349453035	0.01039	0.357826265	0.00871
DIABLO	-0.029667399	0.72387	0.211848665	0.01039	0.059802864	0.47478
ERP27	0.476515486	0.18687	0.92082001	0.01039	0.135734004	0.70862
TBPL1	0.111725239	0.53285	0.450425704	0.01046	0.116288092	0.51517
ZNF623	-0.291328907	0.08361	-0.43063119	0.01046	-0.152390572	0.36432
MARK3	-0.14017045	0.1329	-0.238026097	0.01052	-0.170978535	0.06657
TRAPPC3	0.119404714	0.22946	0.251072416	0.01053	0.101446383	0.30667
PLAA	0.089454832	0.45899	0.306255139	0.01054	0.016088019	0.894
TUFT1	-0.180332356	0.2402	-0.392770187	0.01056	-0.185173266	0.22685
ANAPC7	0.12452967	0.38132	0.360839911	0.01067	0.122551304	0.38788
C6orf136	-0.319182857	0.11618	-0.532734589	0.0107	-0.291068843	0.15075
EIF1	0.244596705	0.15958	0.443297387	0.01069	0.173900685	0.31739
KPTN	-0.538693997	0.09994	-0.845202061	0.01069	-0.152677907	0.64113
LARP7	-0.153396609	0.37837	0.442281124	0.01067	-0.143778003	0.40834
RORC	-0.402984498	0.46756	-1.573238481	0.0107	-0.820344349	0.15706
DHRS4L2	-0.233207665	0.5302	0.90818751	0.01071	0.017272968	0.96299
CSTF3	-0.068854069	0.36623	0.188751789	0.01073	-0.060334424	0.42752
DBF4B	0.117984789	0.51782	-0.47597638	0.01074	-0.058107348	0.75096
FBXO46	0.192784238	0.30853	-0.487010091	0.01075	0.014201135	0.94044
PQLC2	0.041398039	0.78599	-0.393932796	0.01074	-0.036505918	0.81099
EMC8	0.104894004	0.37711	0.298104623	0.01077	0.158745227	0.17889
AC093724.1	-0.389040288	0.17894	-0.75388109	0.01085	-0.345458371	0.22812
GSDMB	0.199043163	0.33823	-0.533965668	0.01086	0.323517129	0.11928

TMEM50A	-0.013038	0.90729	0.283332137	0.01087	0.006459486	0.95388
MAN2B2	-0.017009597	0.89062	-0.315226383	0.01091	-0.081427356	0.51018
HNRNPH1	0.140914181	0.17527	0.264406947	0.01092	0.060096135	0.56321
ADARB1	0.21647845	0.47084	0.75730749	0.01096	-0.106139019	0.72395
LYPLAL1	-0.299432945	0.05332	0.381767126	0.01098	0.062131511	0.68418
HGAP19-SI	0.248537773	0.88107	-4.439541844	0.01099	0.43454909	0.79383
CHTF18	-0.261788815	0.22135	-0.547566738	0.01102	-0.27768928	0.19403
RPL17	-0.222489324	0.0717	0.313790061	0.01103	-0.028725902	0.81607
ZNF783	-0.150255043	0.42619	-0.483002355	0.01103	-0.162316559	0.39238
TXNRD1	0.148781156	0.30223	0.365571304	0.01107	0.057336205	0.69099
ATF2	0.14706762	0.20999	0.296953845	0.01108	0.202031263	0.08466
DDX1	0.01133763	0.91708	0.273296209	0.01109	0.169790174	0.11652
SF3A3	-0.081709836	0.51849	0.319492746	0.01114	-0.007998581	0.94951
OSER1	0.045804673	0.69684	0.291787744	0.01119	-0.012580671	0.91484
SLC2A4	-0.678355219	0.06303	-0.927592625	0.01123	-0.443690348	0.22136
GK5	-0.274132862	0.07592	-0.391149691	0.01125	-0.393340063	0.01084
TTLL7	-0.192515016	0.52227	-0.763462801	0.01128	-0.240204893	0.42435
MALRD1	0.610683739	0.17435	1.12616492	0.01131	-0.339432915	0.46262
VAMP3	-0.02694767	0.75858	0.220519483	0.01131	-0.000322937	0.99706
TXNDC5	-0.055141418	0.67166	0.328649421	0.01132	0.094322786	0.46798
EPSTI1	0.085414728	0.79218	0.810122875	0.01138	0.183642505	0.57169
OSGIN2	-0.07234455	0.68378	0.444587005	0.01141	0.052488372	0.76713
C1orf63	-0.253701985	0.09546	-0.387014666	0.01145	0.082437031	0.58736
CUL9	-0.077347635	0.63914	-0.419336939	0.01144	0.004744193	0.97704
PIK3R4	0.126584873	0.37869	0.361323427	0.01149	0.193314181	0.17851
MMAA	-0.043213081	0.82403	0.484495325	0.01154	0.098270711	0.6123
KIAA1199	-0.671380272	0.20576	-1.340719943	0.01156	-0.758307825	0.15307
CDC42EP4	0.053727022	0.62383	-0.277373759	0.01157	0.019795101	0.85667
MFF	-0.153219628	0.09657	0.229224323	0.01158	-0.090860436	0.32268
ANO7	0.337485827	0.24197	-0.796683807	0.0116	0.128859637	0.65863
CCDC159	-0.257580997	0.14875	-0.455978672	0.01161	-0.179566562	0.31056
BAGE2	-0.100434082	0.78598	-0.99625111	0.01166	0.133149692	0.71217
CAPN10	0.02808925	0.83304	-0.338267898	0.01165	0.127926915	0.33471
FA2H	0.127259951	0.5913	-0.602559205	0.01163	0.414535936	0.07941
LAMTOR5	-0.0188598	0.85324	0.249672831	0.01165	0.015024759	0.88216
MRPL36	-0.199712238	0.1051	0.298026079	0.01166	-0.087061284	0.4748
SRPK1	-0.059862421	0.68344	0.369507961	0.01164	0.270848925	0.06477
ZNF148	-0.00633403	0.9335	0.190304171	0.01162	0.182109085	0.01599
RALB	0.158878754	0.14578	0.273432548	0.0118	0.117552759	0.28193
TSSC2	-0.08224585	0.73266	-0.61350966	0.01183	0.040695482	0.86534
CDH10	-0.305365081	0.27734	-0.709274005	0.01186	-0.499292647	0.07638
RDH11	0.017155009	0.87531	0.274404303	0.01188	0.222123096	0.04189
RFX1	0.480058844	0.0714	-0.687478427	0.01187	0.247589373	0.35397
LMF1	-0.2764212	0.18303	-0.525974731	0.0119	-0.234863046	0.25608
MPPE1	-0.077289071	0.5534	-0.324994288	0.01193	-0.021464061	0.86693
ECD	0.013775705	0.90034	0.272564401	0.01197	-0.023604406	0.83008
GATC	-0.004085402	0.95972	0.200616153	0.01201	0.095436329	0.23546

NOL9	-0.203399123	0.16819	-0.369491862	0.01202	-0.251190193	0.08891
LYPD2	-0.06752552	0.88361	-1.175508665	0.01206	0.608536844	0.18404
MMP15	0.05729212	0.69069	-0.362184759	0.01207	0.186778378	0.19406
HARS	0.045724008	0.59363	0.213729449	0.01209	-0.06212491	0.47011
HLA-DMB	0.863928675	0.12466	1.394989981	0.01209	0.599230538	0.29059
EEF1A1P1	-0.259925471	0.35275	0.673097965	0.01213	-0.400722204	0.15395
CCR4	0.151585345	0.70134	-1.066663894	0.01216	-0.197254736	0.62376
LIMD1	0.194136921	0.06026	-0.25869126	0.01219	-0.004850677	0.9625
GOLGA7	0.131372254	0.25138	0.285527255	0.01219	0.060270387	0.59864
CREM	0.076306424	0.55731	0.320140538	0.01221	-0.090868125	0.49072
IQGAP1	0.130385839	0.37082	0.364873961	0.01221	0.207298467	0.15465
ACSF3	-0.002060437	0.98824	-0.352989979	0.01224	-0.097926589	0.48278
MAP2K4	-0.010608882	0.92811	0.293369757	0.01231	0.121522414	0.30078
PLEKHH3	0.033023532	0.88669	-0.587142365	0.01236	-0.143079863	0.53816
CCDC129	0.705448749	0.41495	2.106188579	0.01237	0.19187191	0.83581
STIP1	-0.04511231	0.69273	0.284758519	0.01238	-0.094366574	0.40851
RBM34	-0.1961708	0.15621	0.339994338	0.01244	-0.135731141	0.32473
N6AMT2	-0.079935599	0.67479	0.449405003	0.01247	0.033385463	0.85927
ZNF417	0.058108784	0.6765	-0.347186084	0.01247	0.109213136	0.42977
FASTKD5	0.230076694	0.09613	0.342426377	0.01249	0.084017078	0.54473
USP10	0.133180977	0.13904	0.223540796	0.0125	0.073700162	0.41271
APLF	0.075264495	0.62281	0.375193302	0.01253	-0.341025737	0.02828
NUAK1	0.655521186	0.05599	0.852722928	0.01254	-0.006430594	0.98508
PCDH1	0.211208294	0.26912	-0.478554129	0.01254	0.180130246	0.34589
TIMM10B	0.083321823	0.40187	0.245880372	0.01254	0.116034198	0.24187
C2	-0.229918114	0.51479	-0.893080086	0.01256	-0.269705533	0.44524
MLF1	-0.036781374	0.87042	0.55355531	0.01257	-0.10846834	0.63031
CA12	1.0063633	0.08307	1.447794988	0.01258	-0.234523676	0.68742
CCDC36	-0.032565595	0.94538	-1.274862527	0.01262	-1.038272294	0.04125
GTPBP2	0.166351902	0.13461	-0.278579885	0.01267	0.085647618	0.44165
CCR6	0.521532782	0.14835	0.885227586	0.01269	0.849281212	0.0173
ANKAR	-0.371924069	0.19978	-0.721960606	0.01273	0.38140009	0.1826
ANKRD1	0.368152301	0.4517	1.202112555	0.01272	-0.884385104	0.08037
DVL2	-0.097088398	0.54781	-0.402299278	0.01273	-0.11283338	0.48479
EPN1	0.203284709	0.20933	-0.405438631	0.01272	0.147116745	0.36341
TBC1D8	0.017691426	0.87513	-0.281128278	0.01273	0.073276132	0.51266
PRKAG2	0.266200696	0.26202	0.58742396	0.01279	0.643242578	0.00676
ASB16	0.050406297	0.89634	-1.044993139	0.01281	0.058368409	0.87911
FAM122C	0.304534486	0.2684	-0.706297982	0.01284	0.364175772	0.18553
HAPLN4	0.647493204	0.14563	-1.177932071	0.01285	0.036752592	0.93513
ANP32A	-0.033691076	0.75069	0.262705784	0.01286	0.058060089	0.58351
TMEM164	-0.044234167	0.69617	-0.281668533	0.01288	0.098213796	0.3832
LRSAM1	-0.004600716	0.97609	-0.385535388	0.01289	-0.173703954	0.26051
TNKS	-0.079556676	0.53304	-0.317106506	0.01294	-0.037088081	0.77161
FGFRL1	-0.247530499	0.13843	-0.414896279	0.01299	-0.248242228	0.13712
NCDN	0.306534773	0.08295	-0.444368031	0.013	-0.217883543	0.22181
TEN1-CDK	0.036722112	0.88882	-0.67246385	0.013	0.133259999	0.6095

PLCH2	-1.003707806	0.12194	-1.664634652	0.01301	0.128725198	0.84015
P11-252A24	0.275305937	0.24488	-0.603839866	0.01303	0.099900523	0.67343
PLEKHH1	-0.065781422	0.75611	-0.526305715	0.01305	0.324803742	0.12454
FUS	0.330746596	0.1336	0.546112418	0.01306	0.421000236	0.05611
AK2	0.013675254	0.9116	0.30474702	0.01308	0.159716377	0.19419
P11-483P21	-0.625576955	0.16574	-1.153674357	0.01309	-0.477097996	0.28351
DDX41	0.15438673	0.13587	0.255325677	0.01316	0.279531034	0.00699
HSP90AB2P	-0.006646271	0.97808	0.593524664	0.01315	0.091044945	0.70594
BRAT1	-0.05739656	0.7406	-0.431818905	0.01317	0.009947086	0.95422
ANXA13	0.949054901	0.10044	1.429018676	0.01324	-0.396973489	0.49431
CHD1	0.021426949	0.84704	0.274225697	0.01325	-0.045262013	0.68347
CUL4A	0.032621538	0.70769	0.21315173	0.01325	0.322698057	0.0002
EFTUD2	-0.045140493	0.67263	0.263497929	0.01325	0.055700257	0.60159
EIF4H	-0.044524119	0.58194	0.199802914	0.01321	-0.023148111	0.77461
PKDCC	-0.108749433	0.57757	-0.484117784	0.01321	-0.178375636	0.36099
RP11-44D5.	-0.261893895	0.48517	-0.988293524	0.01325	-0.212951457	0.56546
SETD4	-0.186205767	0.222	-0.377958114	0.01326	-0.046953622	0.75667
CD81	-0.107932663	0.44879	-0.352910883	0.0133	-0.208751617	0.14304
YWHAG	0.122731015	0.11373	0.19165903	0.0133	-0.044669051	0.5653
PINX1	-0.072466923	0.66898	0.401650966	0.01332	-0.003952288	0.98122
PYGL	0.202965964	0.18959	0.379008583	0.01333	0.107037238	0.48895
PFDN2	-0.093799698	0.45679	0.301080359	0.01335	0.018631695	0.881
ZCRB1	-0.182870868	0.11029	0.27708005	0.01335	-0.090226269	0.42841
TTC31	0.053484127	0.56641	-0.233565324	0.01338	0.064480652	0.48473
B4GALNT2	-0.270071413	0.15858	-0.473745729	0.01342	-0.078019548	0.6822
CS	0.145216851	0.13474	0.239434773	0.01343	0.343021859	0.0004
MTHFR	0.115547055	0.3668	-0.317999134	0.01343	0.148055549	0.24802
ALKBH3	-0.185208969	0.2654	0.401844578	0.01345	0.032492716	0.84404
P11-286N22	0.32058145	0.17861	0.585524171	0.01347	0.107178173	0.6539
BCR	-0.192271674	0.1992	-0.369890581	0.01353	-0.245353608	0.10153
MRPS30	-0.145262031	0.43414	0.456599415	0.01352	0.069968336	0.70586
SLC35A2	-0.185343391	0.20168	-0.358041964	0.01353	-0.072844169	0.61452
MED21	-0.030508199	0.75619	0.236593065	0.01356	-0.038543489	0.6925
KIAA0195	0.237641362	0.15928	-0.419169089	0.01363	0.373428748	0.02683
CBX8	-0.148149209	0.54155	-0.596154826	0.01365	-0.194387565	0.41761
FBXW4P1	-0.4000429	0.31094	-1.030642128	0.01364	-0.861495583	0.0382
OGFOD1	0.000708545	0.99446	0.248243022	0.01366	-0.228116002	0.0263
NOP16	0.223839888	0.44229	0.704581142	0.0137	0.070669011	0.8084
VGLL3	0.785818012	0.0511	0.990667681	0.01373	0.296544116	0.46188
TGS1	-0.162157136	0.19577	0.304445149	0.01375	-0.05804955	0.64199
TRERF1	0.144860649	0.2608	-0.319523012	0.01378	-0.002916947	0.98196
SNX22	0.244175541	0.13341	-0.412651768	0.01381	0.020795608	0.89929
NAPRT1	-0.048759655	0.77915	-0.429228934	0.01389	0.087561974	0.61187
IQCG	-0.268213381	0.15854	-0.465921541	0.01392	-0.099055943	0.59971
SLC36A2	0.162109305	0.65635	-0.973413384	0.01392	-0.166762879	0.65476
FBF1	-0.146850005	0.66241	-0.834658226	0.01393	-0.213458464	0.52656
DCDC2B	0.341266308	0.24479	-0.793922443	0.01398	0.362307919	0.21486

IQSEC1	-0.52411438	0.1067	-0.800933732	0.01397	0.066797476	0.83609
P11-454F8	0.212100551	0.47864	-0.776106514	0.01398	-0.322557634	0.29543
PSAPL1	-0.26165663	0.42876	-0.814877816	0.01403	-0.357456163	0.27986
PDPR	0.056381362	0.68878	-0.346215819	0.01405	-0.193997281	0.16983
KCNJ13	0.151858015	0.64732	0.801457995	0.01406	0.583254318	0.07677
MGAT4A	0.360741862	0.21171	0.707408551	0.01407	-0.001988456	0.99451
CDK17	0.236352158	0.09223	0.342350881	0.0141	0.238092622	0.08966
C11orf35	0.119983094	0.68547	-0.750910025	0.01417	0.20887099	0.47823
EFCAB4A	-0.277568498	0.2016	-0.534649516	0.01417	0.337173782	0.11223
MYO15B	0.337316833	0.12863	-0.56053409	0.01418	0.513528283	0.01982
ZMPSTE24	-0.077015834	0.48981	0.27106029	0.01418	0.129172836	0.24465
MYL12B	-0.104529556	0.27083	0.232182535	0.01419	0.00925961	0.92223
ZNF20	-0.110832758	0.68295	-0.669332786	0.0142	-0.100914137	0.70531
CATSPERG	0.032601497	0.89204	-0.610001869	0.01422	0.27654388	0.24791
BOLA2B	0.047302531	0.77669	0.402569236	0.01429	0.212444189	0.20024
FKRP	0.001272363	0.99345	-0.383538467	0.01428	-0.070756731	0.65334
LY75-CD302	0.459369399	0.31746	1.118396603	0.01427	0.459250981	0.31728
RPS4X	-0.197988638	0.07516	0.272419311	0.01426	0.044797437	0.68709
HPRT1	-0.381822468	0.08477	0.52414813	0.0143	-0.025262261	0.90721
GP2	-0.225169514	0.68652	-1.368151331	0.01436	-0.400690292	0.47271
VN1R83P	-0.499775855	0.19886	-0.988217021	0.01438	-0.929888444	0.02251
STEAP1	-0.194861317	0.32959	0.48195387	0.01444	0.469294397	0.01747
IF103-CHM	-0.123275635	0.86653	-1.925228664	0.01446	-1.203175382	0.11394
ARL5A	-0.046036275	0.66272	0.256583494	0.01449	0.044785184	0.67078
CCDC82	0.178159253	0.15315	0.301820091	0.01449	0.097718751	0.43447
SH3BGRL	-0.147780672	0.10509	0.221278435	0.01449	-0.099428234	0.2754
ACAP3	0.075419437	0.69446	-0.471904408	0.01456	0.233061569	0.22458
ANKRD61	-0.049115886	0.88225	-0.8751271	0.01457	-0.170730313	0.60946
FEM1B	-0.038155285	0.65253	0.205779749	0.01458	0.139801881	0.09826
GAPDH	-0.064437618	0.63026	0.32663984	0.0146	-0.127828654	0.33964
NKTR	-0.007700447	0.96258	-0.401019936	0.0146	-0.034349063	0.83426
TADA3	0.096503697	0.38175	0.268030314	0.01459	-0.004550703	0.96718
ADCY7	-0.01176829	0.93898	-0.370155643	0.01463	-0.232648167	0.12807
ALG12	-0.216782668	0.16974	-0.386149915	0.01466	-0.125042881	0.4273
FAR2	0.257013912	0.1384	0.418975177	0.01468	0.0816111	0.63796
TAB1	0.112649442	0.43639	-0.354019711	0.01468	0.067583175	0.63789
ADAMDEC1	-1.267288399	0.05073	-1.58796988	0.01474	1.201241722	0.0615
C1orf174	-0.000917352	0.99236	0.228631675	0.01474	0.053390624	0.57458
CD84	0.142934139	0.78212	-1.281038541	0.01479	-0.197826912	0.70156
AQP12B	-0.231787613	0.62552	-1.188797439	0.0148	0.594224077	0.19993
ZBTB22	0.013980077	0.89662	-0.263989233	0.01481	0.048190523	0.6536
PDLIM5	0.263740023	0.06091	0.342273406	0.01485	0.338487303	0.01609
USP32P3	0.266976901	0.62684	-1.479254863	0.01488	-0.495859622	0.38355
RPL7AP10	0.571515284	0.14784	-1.095257331	0.01489	0.305559952	0.44517
FOXO3	0.080234224	0.48595	-0.280504972	0.01491	-0.161843756	0.16052
LCMT2	-0.019254236	0.9144	0.431765611	0.01491	0.014142688	0.93693
TET3	0.02597435	0.86845	-0.382540997	0.01493	0.110519313	0.48051

ZFAT	-0.434470844	0.06816	-0.578556185	0.01493	-0.260210422	0.27364
ZYG11A	-0.56713189	0.25619	-1.268505067	0.01492	-0.395567827	0.42109
NOV	0.193393459	0.71605	1.218369031	0.01497	0.003194805	0.99525
ATP6V1C1	-0.122819434	0.37564	0.335196705	0.01499	0.222570907	0.10718
PPP1R12A	0.125244261	0.4052	0.365604679	0.015	0.123784013	0.41078
C1orf131	-0.222912499	0.21268	0.429525246	0.01501	-0.012297604	0.94485
LIN37	0.284083728	0.33481	-0.727915316	0.01513	-0.356240573	0.23174
LRPPRC	-0.062138252	0.72702	0.431964287	0.01513	0.292454475	0.10019
NXT1	-0.018591246	0.90006	0.350188408	0.01517	0.072772627	0.62045
GGA2	0.13736639	0.21821	0.269733529	0.01525	0.186045605	0.09559
PHF10	0.607093179	0.21768	1.189889064	0.01526	1.552500282	0.00153
ZNF160	0.123616561	0.26856	-0.27129017	0.01526	0.108167853	0.33245
FAM118A	0.155666381	0.23649	-0.320864215	0.01532	-0.021674181	0.86955
KCNA7	0.427310481	0.22844	-0.954465988	0.01531	0.428929485	0.22531
VEZT	0.058278016	0.54369	0.231834374	0.01532	0.050003388	0.60216
PUM1	-0.059893292	0.33169	-0.148987728	0.01535	-0.046325517	0.45229
FGD1	0.180396752	0.30798	-0.433282503	0.01537	-0.212184067	0.23428
MTPN	0.080614601	0.25256	0.169791525	0.01537	-0.001790093	0.97974
SH3GL1P1	-0.0655881	0.85464	-0.916640375	0.01539	-0.167092559	0.64406
RBMXL1	0.114177743	0.28005	0.253578474	0.01541	0.071387596	0.49891
TRAFD1	0.163350442	0.16038	0.278949632	0.01545	0.050637751	0.66302
ERCC2	-0.197748793	0.19069	-0.366456971	0.01546	-0.290075663	0.05559
MAPK1	-0.063033425	0.44709	0.200157076	0.0155	-0.064534452	0.43583
C1orf222	-0.152412221	0.70322	-0.989869247	0.01556	0.08731202	0.8265
RPS16	0.187103375	0.4211	0.562247371	0.01556	0.158956637	0.49428
COL18A1	-0.276692425	0.28134	-0.620987776	0.01562	-0.407055295	0.1134
TNRC6C	0.338688872	0.26611	-0.738068814	0.01562	-0.424458588	0.164
AOAH	1.088995017	0.15132	1.802716963	0.01566	0.017782029	0.98202
ATG3	-0.210497196	0.12056	0.322569909	0.0157	0.053565409	0.6909
MUC5B	-1.035376004	0.07242	-1.392305561	0.01572	-0.313240245	0.5866
RPS3	-0.049276267	0.70581	0.31510726	0.01572	0.144529956	0.26807
MAK	0.307228945	0.23294	-0.671789619	0.01574	-0.337979864	0.2137
IQGAP3	-0.232683837	0.29491	-0.536516874	0.01577	-0.084347589	0.7039
SSR2	-0.070610457	0.46016	0.229889213	0.01581	-0.114567991	0.23076
ERBB2IP	0.074888369	0.55286	0.303901165	0.01587	0.149768699	0.23503
MTERFD3	-0.241635745	0.10878	-0.360168915	0.01593	-0.187518727	0.20815
ITGB4	0.146187615	0.58012	-0.637686214	0.01595	-0.015168836	0.95423
WSB1	0.164754004	0.20981	-0.316901317	0.01596	0.028444078	0.82864
CRK	0.081713202	0.31884	0.196082626	0.01612	0.121558422	0.13736
TSC22D2	0.008453598	0.94709	-0.307155979	0.01615	-0.007527676	0.95296
ZBP1	-0.003374029	0.99555	-1.541394708	0.01616	0.119011537	0.84369
ADAMTSL4	-0.099379699	0.70165	-0.632643985	0.01618	-0.191390404	0.46128
PPP1R35	0.211102066	0.25344	0.438282361	0.01617	0.07480972	0.69079
RAB25	0.218874063	0.18169	0.393378866	0.01617	0.411644391	0.01187
C17orf53	-0.240286415	0.2988	-0.551564854	0.01623	-0.435244599	0.05813
SPSB3	0.030604967	0.87372	-0.468920728	0.01624	-0.093706195	0.6289
CDKN3	-0.380716451	0.07092	0.494492834	0.01626	-0.352821458	0.09332

TLK2	0.16938109	0.09721	0.243825522	0.01629	0.044711231	0.66103
NUP153	-0.013260156	0.90659	0.270237983	0.01632	0.060491348	0.59196
P11-974F1	-0.623469937	0.11301	-0.956709884	0.01636	-0.094695794	0.8021
LRRTM4	-0.099926387	0.75316	-0.763002281	0.01644	-0.185091153	0.56182
HKR1	-0.019869221	0.85992	-0.268415401	0.01649	-0.029801355	0.79136
PSORS1C1	-0.244503102	0.57273	-1.042167759	0.01651	0.198466703	0.64083
CD47	0.062625186	0.70515	0.396159083	0.01657	0.212264351	0.19948
ITGAX	0.356094991	0.37651	-1.082052507	0.01658	0.336536021	0.40312
NAP1L4P1	-0.514572712	0.13623	-0.838695316	0.01659	-0.069865285	0.83333
PUS1	-0.238689288	0.19716	-0.446300733	0.01661	-0.345826155	0.06397
EIF4E	0.042829333	0.73783	0.30558186	0.01664	0.176384083	0.16759
GLE1	-0.07380756	0.50656	0.261712253	0.01664	0.175671948	0.11025
HIAT1	-0.106004954	0.19393	0.193087305	0.01665	-0.134074976	0.10025
ELF3	-0.174415618	0.41175	-0.508322946	0.01675	0.136388114	0.5208
OGT	0.139657587	0.12428	0.21683222	0.01673	0.192173574	0.03409
RABL3	-0.107263915	0.37309	0.284311744	0.01675	0.075929377	0.52634
TSSK4	-0.32723843	0.34851	-0.859477307	0.01686	-0.566080403	0.10821
FUOM	0.463051091	0.09895	0.658420128	0.01691	0.261460172	0.3549
LIPT2	0.298516384	0.18416	0.523540865	0.01692	0.344789198	0.12318
JRKL	-0.13360023	0.25933	0.272688429	0.01696	0.212478898	0.0656
YLPM1	-0.033169684	0.80793	-0.326145405	0.01702	-0.171456895	0.20936
CNPPD1	0.071827354	0.54766	-0.28743757	0.01708	-0.017084429	0.88654
NCK2	0.016609443	0.898	0.30701094	0.01716	0.024177641	0.85208
EPHB4	-0.129174552	0.20162	-0.240879474	0.0172	-0.071297887	0.48033
TMEM8B	0.232545327	0.27861	-0.514540216	0.01725	0.057290377	0.78824
OTUB2	0.156894896	0.43287	0.463679766	0.01728	0.25808394	0.1922
NAT2	0.104833489	0.885	1.624148859	0.0173	-0.136204776	0.85001
RP11-15G8.	-0.306913997	0.47491	-1.086622795	0.0173	-0.587467899	0.18076
DUSP6	0.293242851	0.12479	0.454003021	0.01736	0.165920613	0.3851
PRKD1	0.373140784	0.26692	-0.809024283	0.0174	-0.163157226	0.62746
AKR7L	0.007730694	0.98331	-0.934522032	0.01751	-0.287201396	0.43681
FAM27E4	-0.401413398	0.1424	-0.652270181	0.0175	-0.471076211	0.08549
SPNS1	-0.007637508	0.95	-0.29080636	0.01752	-0.038768768	0.75005
TMEM176B	0.520878056	0.11291	0.779314416	0.01751	0.512946436	0.11846
MYO1G	-0.292906927	0.48945	-1.053275773	0.01754	-0.476998308	0.26616
CHAF1A	-0.23880695	0.19716	-0.438045247	0.01758	-0.071787487	0.69754
SCN1B	-0.010546553	0.98521	-1.420151085	0.01757	0.007216677	0.99013
AKAP13	-0.067486581	0.57055	-0.282671193	0.01761	0.079379709	0.5042
CFTR	0.786500765	0.0594	0.983786405	0.01767	0.377084328	0.36586
PPIB	-0.121530485	0.28185	0.267489075	0.01767	0.123563669	0.2736
PTK7	-0.104861005	0.56569	-0.433296018	0.01765	-0.409906013	0.02488
UBE3B	-0.111363322	0.33732	-0.275828115	0.01768	-0.139991722	0.22785
CNKSR1	0.424454799	0.0801	-0.591897278	0.01772	0.418880155	0.08456
GLRX3	-0.052609889	0.60211	0.237100715	0.01778	-0.019991902	0.84278
PEX3	-0.066717446	0.6689	0.360694074	0.01776	0.0927969	0.54769
ZG16B	0.559626091	0.26553	1.179639834	0.01777	-0.122443671	0.80887
CBLC	-0.220716941	0.23929	-0.444955874	0.01782	-0.10089042	0.58913

GUCY2C	1.265297992	0.1237	1.945140458	0.01783	0.963979734	0.24108
LACTB	0.222159263	0.28648	0.491755971	0.01789	0.217967262	0.295
NPIPP1	0.153557876	0.34043	-0.390295332	0.0179	-0.050998342	0.75363
TSC2	0.290967461	0.08779	-0.405174662	0.0179	0.128565298	0.45138
KAT6B	-0.070861093	0.49214	-0.243614525	0.01794	0.067456742	0.5098
MXD1	0.122104629	0.66276	-0.663850811	0.01797	0.380292663	0.17416
EVL	0.036905442	0.80622	-0.35801557	0.01798	-0.093276335	0.5365
TMEM176A	0.415496131	0.23996	0.835011319	0.018	0.363146987	0.30442
CCNC	-0.117161224	0.25385	0.241221181	0.01801	0.088121424	0.38898
rac-YRM20	-0.202640907	0.38285	-0.552691274	0.01802	-0.000901336	0.99688
APC2	0.104687418	0.73506	-0.764411567	0.01803	0.243737315	0.42589
PALMD	0.63923237	0.13056	0.996795749	0.01804	0.249060871	0.55672
CAMTA1	0.039691221	0.78317	0.33739629	0.01806	0.113342606	0.43231
C9orf64	0.418976792	0.27972	-1.001480681	0.01808	-0.102791561	0.79416
EIF4B	-0.216056028	0.05643	0.2674921	0.01807	0.090794919	0.42251
DOT1L	0.206653721	0.2268	-0.407673157	0.01818	0.050900379	0.76655
LUZP2	0.15155873	0.74866	-1.125425187	0.01826	-0.4459994	0.34478
PRKAR1A	0.106085672	0.39046	0.291351236	0.01827	0.052981879	0.66799
IPO5	-0.172190872	0.09285	0.241191557	0.01828	-0.023910979	0.8153
LPCAT4	0.213613636	0.29131	-0.481760886	0.01833	0.415509352	0.03919
MRFAP1	0.001564757	0.98749	0.234345219	0.01834	-0.022614497	0.82066
TTLL3	0.335503152	0.06397	-0.435684414	0.01833	0.043130101	0.81196
ARL16	0.414840537	0.09912	0.591266516	0.01835	0.269878752	0.2835
EEF1A1P28	0.346493142	0.44752	1.006228357	0.01837	0.69955236	0.11213
PTPN4	-0.105196805	0.41263	0.300751823	0.01839	0.075321468	0.55616
RMND5A	0.160861187	0.18822	0.287322929	0.01842	0.183597428	0.13243
7-Mar	-0.048418233	0.57976	0.204837392	0.01844	-0.004894664	0.95534
C3orf83	-0.023934329	0.93552	-0.720007332	0.01846	0.14398257	0.62247
ST13	0.105612132	0.55388	0.419033037	0.01852	0.550460344	0.00198
SAMHD1	-0.047832814	0.75413	0.350788915	0.01855	0.028652093	0.85007
RABEP1	-0.06558902	0.48867	0.221088857	0.01857	0.016429754	0.86191
B3GALT4	0.002722833	0.99224	-0.67965815	0.01862	0.470476444	0.08472
NBPF10	0.286576994	0.10296	0.411488051	0.01862	-0.210032874	0.23629
MAGED4	0.026108799	0.9139	-0.568035341	0.01866	-0.22577833	0.35097
MRPS7	-0.159318407	0.09201	0.216628554	0.01868	-0.051367161	0.58302
PHLDA3	-0.148585633	0.36607	-0.38734784	0.01868	0.061481062	0.70785
PRODH	-0.271395754	0.53276	-1.028830457	0.01869	-0.372765626	0.39177
RBM8A	0.026940395	0.79142	0.238679387	0.01868	-0.000142354	0.99888
ZNF316	-0.161659115	0.4865	-0.549320653	0.0187	-0.165224581	0.47833
CENPP	-0.325522784	0.25969	-0.678760786	0.01873	-0.562840485	0.05207
GCDH	-0.143542674	0.40655	-0.399525517	0.01883	-0.002762413	0.98706
FAM156B	-0.645931896	0.08866	-0.891860807	0.01886	-0.394371316	0.29624
POF1B	-0.103520425	0.53886	0.394829634	0.01885	0.394903677	0.01887
TFEC	1.427769306	0.10603	2.090887764	0.01888	-0.195871841	0.82795
ABHD11	-0.140550186	0.3302	-0.337484538	0.01899	-0.019397668	0.89208
C19orf33	-0.288377036	0.0795	-0.38496935	0.01901	0.047878063	0.76937
FBXL18	0.290842458	0.10858	-0.440359836	0.01901	-0.13463628	0.46637

STAG3	0.461157697	0.05116	-0.571139628	0.019	0.282566559	0.23266
CYP2S1	-0.24535691	0.28968	-0.544120206	0.01904	0.171622935	0.45782
SDAD1	-0.160144304	0.20928	0.296757602	0.01905	-0.202043177	0.11263
ZNF880	-0.250168763	0.26108	-0.522224784	0.01905	-0.090085568	0.68204
AC027612.(	0.473944974	0.10907	0.687706952	0.01908	-0.582069655	0.05619
CACFD1	-0.123161959	0.48893	-0.419010635	0.01909	0.007749546	0.96487
NUB1	-0.15464082	0.14034	0.24228267	0.0191	-0.009973077	0.92385
P11-705C15	-0.117217803	0.68813	-0.691173572	0.01907	-0.365397384	0.21268
MAST1	-0.212135099	0.58427	-0.936348528	0.01912	-0.233177778	0.54603
MEAF6	-0.07936351	0.48369	0.260364743	0.01915	-0.037037009	0.7431
PNMAL2	0.263992576	0.57438	-1.184233352	0.01914	-0.617752722	0.20755
RAD17P1	-0.472768809	0.12115	-0.716907824	0.01915	-0.19837511	0.50856
P11-330C7	-0.302844882	0.52685	-1.185649796	0.01915	-0.114013539	0.80899
FAM115C	-0.032475287	0.88218	-0.516453383	0.01917	-0.280634704	0.2018
WDR6	0.188120288	0.1858	-0.333450129	0.01916	0.067749513	0.63372
MAP10	-0.155167714	0.40797	0.417150784	0.0193	0.018949886	0.91814
R3HCC1	0.020152741	0.88283	0.315274349	0.01935	0.081552832	0.55037
SULT2A1	0.730955189	0.19558	1.292424837	0.01938	0.605265358	0.28521
FNDC4	0.293052515	0.12117	0.437741616	0.0194	-0.044461711	0.81583
TEX35	-0.071916486	0.84329	-0.889466699	0.01943	-0.4753974	0.19991
TIPARP	-0.297194239	0.17654	-0.513992291	0.01942	-0.351727815	0.10978
CTPS1	-0.057078492	0.74522	0.407500627	0.01947	0.05903259	0.7363
STAT2	0.074628631	0.53055	-0.278282914	0.01949	-0.017751165	0.88145
TFF1	-0.100361178	0.65547	-0.525401059	0.01951	0.035722564	0.87381
C5orf63	-0.504031456	0.21627	-0.961613715	0.01954	0.484373244	0.22723
ELN	0.385265102	0.15485	-0.642175551	0.01959	0.021919352	0.93567
L3MBTL2	-0.080268305	0.58071	-0.33866149	0.01962	-0.16916348	0.24425
GAB2	-0.051674012	0.75462	-0.387182221	0.01969	-0.126521878	0.44393
MRPL49	0.03898793	0.60667	0.173810364	0.01969	0.191633344	0.01054
TCF23	-0.013630363	0.96144	-0.686456336	0.01968	-0.154849341	0.58566
UBE2Q1	0.139923341	0.21974	0.264646236	0.0197	0.006242595	0.95635
ZFP36	0.212392769	0.40837	-0.601500757	0.01969	0.156974872	0.54117
ABCA2	0.131098049	0.63659	-0.648526888	0.01977	-0.231002113	0.40602
CHTF8	0.038915722	0.74292	0.274684168	0.01978	-0.013778357	0.90749
MIR3654	0.022899146	0.92838	-0.600013029	0.01978	-0.137372059	0.5916
C2orf43	-0.177612805	0.25401	0.35932571	0.01982	0.015015265	0.92291
TPD52L2	0.110918711	0.26656	0.231183598	0.01982	0.166623845	0.0943
ANKRD22	0.27333446	0.21212	0.506676494	0.01984	0.305798151	0.1623
PCDH17	0.196202269	0.43638	-0.591339858	0.01991	-0.418030881	0.09962
VPS37A	0.115353017	0.19752	0.2061558	0.01993	0.106980881	0.23087
CSTF1	-0.130182819	0.19792	0.23256785	0.01995	-0.052433622	0.60337
MYRFL	0.968756093	0.12118	1.398233586	0.02	1.010079203	0.10045
NUDT4	-0.149524236	0.19318	0.266407575	0.01999	-0.137363448	0.23176
PAFAH1B3	-0.18371219	0.20296	-0.335335509	0.02	-0.227929787	0.11387
PAQR4	-0.425558175	0.07237	-0.549144241	0.02001	-0.487968169	0.03904
RPTOR	-0.006585286	0.96553	-0.355983444	0.02	-0.248485164	0.10414
ZBTB3	-0.056887119	0.76167	-0.438507098	0.02001	-0.055907628	0.76639

CDC42EP3	0.215374923	0.37219	0.557432948	0.02005	0.045030904	0.85185
MEMO1	0.07742586	0.59621	0.336833592	0.02006	0.320619566	0.02743
FRAS1	-0.156828903	0.52831	-0.578514792	0.02007	-0.422911288	0.08957
CREBL2	-0.063155697	0.54542	0.237285691	0.02013	0.1358453	0.18794
HDAC1P1	0.286933987	0.5482	-1.335917166	0.02012	0.007902786	0.9871
PRKCD	-0.204643959	0.19202	-0.363420508	0.02013	0.161247708	0.29868
RP11-473I1	0.030532235	0.72562	-0.202266233	0.02012	-0.09821405	0.25931
DOLK	0.205907744	0.13759	0.31808149	0.02018	0.167948459	0.22564
SPIN3	-0.175097892	0.40252	-0.494829122	0.02022	0.077418688	0.70614
GCN1L1	0.005331288	0.96074	-0.251272899	0.02034	0.231074309	0.03281
GGT6	-0.153592748	0.65666	-0.834258669	0.02032	-0.27150925	0.43388
INCENP	-0.166063957	0.37316	-0.433295396	0.02034	-0.07022153	0.70672
PTGES3	-0.17678368	0.0739	0.228549641	0.02034	0.040797366	0.67941
RP11-210N1	-0.619764301	0.31053	-1.487592601	0.02036	-1.50851299	0.01924
BANF1	0.181337592	0.1736	0.306784307	0.0204	0.134885412	0.31088
GLUD1P3	0.232517622	0.74314	-1.695586317	0.02048	-0.173834948	0.8067
POU5F1B	-0.182892212	0.68667	-1.110082788	0.0205	-0.081222731	0.85662
FADD	0.017586095	0.86749	0.237790694	0.02051	-0.018747881	0.85872
OXSR1	-0.128332576	0.25458	-0.260431831	0.02053	-0.162146496	0.14972
RBM14	0.090556521	0.49873	-0.310585297	0.02057	0.070204685	0.60005
GABRB3	0.860330787	0.4304	2.433197824	0.0206	1.450974793	0.17366
C10orf118	0.067795359	0.67926	0.378618775	0.02061	-0.025325883	0.87715
PKN1	-0.238048066	0.14283	-0.374968018	0.02063	-0.127819343	0.42966
DOCK7	-0.07614503	0.49141	0.254554404	0.02064	-0.083799577	0.44843
DTX4	-0.078032308	0.62029	-0.36442101	0.0207	0.17587265	0.26332
RP11-390M1	-0.053033603	0.88277	-0.894835022	0.02072	0.307736285	0.37593
MMRN1	0.899165117	0.26832	1.838757928	0.02073	0.363771639	0.65397
RPAP1	0.200975456	0.30011	-0.450781802	0.02075	-0.204410223	0.29287
ANKRD13A	0.043636479	0.67393	0.23776145	0.02079	0.237354459	0.02143
SUGT1P3	-0.629926272	0.19503	-1.166587151	0.02079	-0.201832337	0.66903
MTIF3	-0.136053222	0.23088	0.256658203	0.02085	0.075528912	0.50143
LARGE	0.157237492	0.20465	0.285514834	0.02087	0.065125162	0.59932
ZFYVE20	0.051202184	0.63968	-0.251303638	0.02089	-0.144206159	0.18682
ZNF589	-0.243272399	0.16026	-0.399663416	0.02092	-0.319543641	0.06539
C18orf21	0.210039524	0.15012	0.332185846	0.02094	0.093356409	0.52232
ALDH8A1	0.453805909	0.36751	-1.228628391	0.02097	-0.402782954	0.43255
PRKCSH	-0.083797623	0.45558	-0.258992542	0.02107	-0.208530337	0.06343
PYGB	-0.009877423	0.92594	-0.245306174	0.02108	-0.052177239	0.62345
FECH	0.1011647	0.56256	0.397992855	0.02111	-0.036068754	0.836
MRPL37P1	0.109016023	0.8259	-1.309196565	0.02112	-0.577490834	0.27261
PVRL3	-0.140103083	0.27001	0.288357226	0.02115	0.099053888	0.43136
ARSJ	0.375378884	0.28289	0.803280823	0.02116	0.359421657	0.30403
FAM98A	0.100394614	0.39915	0.272004638	0.02119	0.208245608	0.07927
KCNK10	0.683428752	0.23711	1.30652956	0.02122	1.777070611	0.00193
ZNF414	0.295888173	0.34863	-0.757681528	0.02123	-0.027329454	0.93191
EIF3A	-0.027913033	0.74073	0.193930127	0.02125	0.037929415	0.65277
RPF1	-0.165900039	0.06779	0.204179168	0.02127	-0.142310676	0.11603

TOP3B	0.108296568	0.35923	-0.274396381	0.02127	0.173148265	0.14
F8	-0.677439495	0.12014	-0.999790997	0.02132	0.226683146	0.5974
EPHX3	-0.269750669	0.2935	-0.597812589	0.02132	-0.240015125	0.34714
FLCN	0.021295219	0.88672	-0.345620719	0.02135	-0.094819985	0.52626
NUPL1	0.112784521	0.16534	0.186084125	0.02144	0.152004658	0.0612
EIF3G	0.067323612	0.45019	0.203780368	0.02145	0.126345874	0.15597
C9orf84	-0.122894772	0.7595	-0.980999701	0.02153	-0.525905908	0.1999
AK3	0.009350262	0.94509	0.310453577	0.02158	-0.087085135	0.52109
PTK6	-0.398245196	0.22559	-0.770427665	0.02162	-0.103202469	0.74713
RPL23AP53	0.485858245	0.08816	0.642323034	0.0216	0.310501912	0.27753
RPS6KA4	-0.022914355	0.89362	-0.39460975	0.02161	-0.243552271	0.15608
VWA1	0.012720478	0.93975	-0.386798824	0.02164	0.040221084	0.81097
ZNF667	0.179974769	0.44638	-0.583571246	0.02164	0.055199394	0.81763
AKR1B15	-0.167227091	0.4559	-0.517835447	0.02168	-0.04321435	0.84668
ANKS1A	0.122227415	0.25889	-0.249672698	0.02168	-0.134440755	0.21723
PBX1	0.161165389	0.29065	-0.350538191	0.02168	-0.249675011	0.10251
NAGLU	-0.049370722	0.80369	-0.457517017	0.02171	0.08725258	0.66039
PHF8	-0.097460761	0.60831	-0.437455941	0.02172	0.023161143	0.90312
TRAF5	-0.184584141	0.21045	-0.337720308	0.02173	-0.205275989	0.16323
TTLL12	-0.118507035	0.47233	-0.378277489	0.02173	-0.040814862	0.80427
UBAC1	-0.016754035	0.879	0.245976595	0.02172	0.120518486	0.26752
PTTG1	0.02737811	0.88145	0.418947292	0.02174	-0.095641846	0.60271
ELMOD3	0.0092229	0.95225	-0.354654203	0.02186	-0.108569851	0.47891
P11-15J10	-0.531239729	0.10092	-0.74096388	0.02189	-0.484047599	0.13198
ALG1L2	-0.425524221	0.37778	-1.178851698	0.02193	-0.20170477	0.66876
SPPL2B	0.274216158	0.36696	-0.722438942	0.02193	0.18267028	0.54908
TCF7	0.167051708	0.42607	-0.492714569	0.02198	0.167093671	0.42992
ARHGAP32	0.130948357	0.30574	-0.293271192	0.02203	0.212374457	0.09601
CUL5	-0.093647667	0.4437	0.27802939	0.02203	-0.140569804	0.24995
ENTHD2	0.115753926	0.60202	-0.51543972	0.02203	0.083982233	0.70668
MTCH2	-0.151603853	0.29766	0.331556521	0.02206	0.146502856	0.31279
POU5F2	-0.212049713	0.4386	-0.633814053	0.02206	0.031708792	0.9073
IL17RB	0.04946699	0.78684	0.412884223	0.02215	0.264537143	0.14568
I2-2610K16	-0.835591294	0.14628	-1.355402529	0.02216	-0.227966595	0.67965
KTN1	-0.035510191	0.68191	0.197638377	0.02222	0.154503092	0.07416
PRSS48	-0.610162656	0.26144	-1.335812286	0.02229	-0.354471747	0.48613
11-1023L1	-0.000413001	0.9989	-0.696899328	0.02243	-0.313724555	0.30006
GPATCH8	0.107194757	0.46062	-0.333706761	0.02245	-0.026990566	0.85275
IL15RA	0.092231354	0.79733	-0.829883989	0.02246	0.129835024	0.71712
CELSR3	0.211443698	0.4611	0.658458483	0.0225	0.115888197	0.68637
VAMP8	-0.097826506	0.49033	-0.323571591	0.0225	0.211720153	0.13316
CDC26	-0.148820094	0.2295	0.269333309	0.02254	-0.088242518	0.47166
STK31	-0.298971128	0.37087	-0.758563708	0.02258	-0.246734385	0.45545
LYPD6	0.007690507	0.96841	0.436454422	0.02262	0.478471603	0.01267
DNAJA1	-0.108679839	0.4185	0.305648568	0.02266	-0.044660967	0.73938
MEGF8	-0.249285734	0.31316	-0.564422767	0.02269	-0.517197249	0.03668
USO1	-0.155265313	0.07061	0.194532669	0.0227	-0.07505099	0.3813

WDR4	-0.263744496	0.19434	-0.461245938	0.02275	-0.130619452	0.51835
ZNF546	-0.186834575	0.41832	-0.524964017	0.02278	-0.035798132	0.87715
LYRM7	-0.249617028	0.10939	0.351273509	0.02279	0.039438234	0.79897
HPN	-0.257715209	0.27384	-0.536646557	0.02282	-0.053895102	0.81878
CDH17	0.892749339	0.1093	1.26879047	0.02284	0.581583899	0.29687
ATP6V0D2	-0.172984942	0.68016	-0.979139132	0.02289	0.039916679	0.92421
CIITA	-0.121460174	0.77885	-0.987029791	0.02291	-0.063292043	0.88374
PBOV1	-0.277550348	0.46042	-0.883301674	0.0229	-0.001614627	0.9965
GALR1	-0.453080228	0.11139	-0.647696393	0.02295	-0.147051921	0.59784
ACAD10	-0.092065531	0.6306	-0.436972838	0.02297	-0.090124555	0.63724
RPS2P44	0.057957501	0.90767	-1.3626956	0.02298	0.325788683	0.50082
ZNF692	-0.208441673	0.21164	-0.37929215	0.02298	0.110625225	0.50461
CYC1	-0.013781181	0.91168	0.280460051	0.02299	0.235829592	0.05654
P11-444E17	-0.367154407	0.39489	-1.037368496	0.023	0.071831107	0.86202
USP15	-0.141591354	0.3095	0.315325012	0.02304	0.134925759	0.33206
PPP1R37	0.067722727	0.65005	-0.341058166	0.02311	-0.139310484	0.35237
PRMT7	-0.099681819	0.5364	-0.366223425	0.0231	-0.115677854	0.47283
F10	0.662217416	0.08891	0.876039966	0.02314	-0.104032692	0.79395
RNF123	0.094571867	0.6355	-0.454822036	0.02317	0.121037615	0.54414
DEGS1	0.169336076	0.28637	0.3582131	0.02324	-0.326023848	0.041
PPIC	-0.245507193	0.10267	-0.340766414	0.02322	0.009991112	0.94674
TLR5	0.341749318	0.43263	0.974013317	0.0233	0.581634919	0.17777
DNAAF3	-0.274256616	0.45409	-0.84597412	0.02332	-0.562771355	0.1204
WIPF1	0.294511747	0.09106	-0.399346054	0.02336	0.029958698	0.86508
ARSG	-0.435354243	0.06946	-0.541545963	0.02338	0.048927871	0.83741
P11-568G1	0.142557612	0.72933	-1.070927353	0.02344	-0.888762798	0.05783
NRNPA1P5	-0.034660084	0.87494	-0.502108114	0.02348	-0.086909432	0.69312
C6orf163	-0.333832857	0.22723	-0.634311861	0.02353	-0.114146454	0.67733
POLR2J3	0.138310852	0.46986	-0.435549067	0.02355	0.11901672	0.53436
DNAJB14	-0.040974499	0.64735	0.201247067	0.02358	-0.005244421	0.95326
FAM229A	0.470334337	0.15485	-0.783700497	0.02357	-0.223050389	0.50791
PPIA	0.025916773	0.78247	0.212245516	0.02364	0.080417043	0.39149
AP000347.4	0.250336382	0.41578	-0.727915787	0.02366	0.129310785	0.6723
PDSS1	-0.10416747	0.62397	0.467567483	0.02371	0.147421901	0.48157
TAF9	0.061184206	0.56362	0.23594095	0.02374	0.2141374	0.04168
NUDT1	-0.211825555	0.27604	-0.437050733	0.0238	-0.063456449	0.73982
MAPK8	0.096791189	0.42037	0.269453347	0.02384	0.039605004	0.74145
ZNF45	-0.007843768	0.9626	-0.37754411	0.02386	-0.01724289	0.91818
AP5M1	0.108481069	0.38021	0.277949362	0.02388	0.119986431	0.33084
NEURL1B	0.118636546	0.63696	-0.570718069	0.02398	-0.159616874	0.52643
PLXNA4	-0.075589622	0.8277	-0.792471016	0.02407	-0.092649268	0.79013
SNRPA	0.04995509	0.7408	0.339138075	0.02407	-0.069517246	0.64548
RPL14	0.154056283	0.1933	0.266852867	0.0241	0.24669021	0.03717
TMEM110	-0.255117997	0.26018	-0.510475461	0.02416	-0.078506817	0.72842
C5orf45	0.261863686	0.06199	-0.320053698	0.02418	0.059124537	0.67463
ZC3H14	0.036858677	0.73078	0.240451617	0.02418	0.093944692	0.37975
TP53I11	0.112765266	0.4274	-0.321116021	0.02422	0.396605126	0.00504

ZNF675	-0.009752301	0.93813	-0.282112729	0.02422	0.02050549	0.8699
SLC2A11	-0.103791686	0.67621	-0.567884517	0.02427	-0.102527301	0.68168
AC093838.4	0.055419202	0.72353	-0.357888368	0.02433	-0.025830511	0.86926
COX6A1	0.003237872	0.97238	0.20960596	0.02433	0.216292406	0.02032
METAP2	-0.074156833	0.32884	0.168906684	0.02432	-0.049582918	0.51279
SCRN2	-0.313294288	0.06547	-0.380558333	0.02433	-0.185880767	0.27126
ZNF556	0.235164474	0.36276	-0.601698519	0.02436	-0.207272444	0.43117
MS4A10	-0.725927637	0.05572	-0.848884889	0.02439	-0.201664887	0.57416
RP11-474D14	0.197306745	0.622	-0.991882266	0.02439	0.029478813	0.94181
RPS9	-0.094753735	0.69975	-0.553512199	0.0244	-0.095797685	0.69655
TOP1	0.057412238	0.69075	0.323366977	0.02438	0.14892294	0.30133
FGFR4	-0.199455979	0.322	-0.45302774	0.02442	0.104446889	0.60304
MFSD6	-0.023521475	0.88859	0.375707938	0.02441	0.138959822	0.40633
HS1BP3	-0.297440754	0.10638	-0.410889323	0.02448	-0.219970969	0.22497
LYPLA1	0.109510421	0.61396	0.486010372	0.0245	0.199371107	0.35796
PTK2	0.03624073	0.689	0.202966531	0.02448	0.111028794	0.22002
TRMT1	-0.01940377	0.91753	-0.421202855	0.02451	-0.20292981	0.27913
ABCA7	0.079257087	0.71231	-0.488823153	0.02455	0.199370462	0.35258
CELSR2	-0.039670977	0.88724	-0.634965607	0.02458	-0.380035078	0.17615
PRPF4B	-0.061928131	0.49986	0.205051876	0.02468	0.037847059	0.67948
SLC25A22	0.82713765	0.06157	0.981843693	0.02467	0.305929388	0.49379
TAS2R20	0.207272343	0.56473	-0.87308074	0.02468	0.00946383	0.97924
UFL1	-0.176635313	0.07154	0.217916703	0.02468	-0.01867342	0.8483
WASH6P	0.190839153	0.2545	-0.377919935	0.02466	0.311483653	0.06225
SPHK2	0.185021965	0.26033	-0.373710584	0.02471	-0.033641999	0.83861
EEF1GP1	0.063284375	0.63954	0.298816857	0.02472	0.319082181	0.01691
NAA11	0.020329219	0.96544	-1.161162941	0.02474	0.123602153	0.79043
C8orf44	-0.33078908	0.30011	-0.724329226	0.02484	-0.361286523	0.25599
NDRG3	-0.154667756	0.10645	0.208631665	0.02483	0.055337094	0.55675
PPP2R5C	0.017500949	0.85172	0.208816868	0.02484	-0.048921808	0.60106
ZBTB48	-0.022775711	0.91327	-0.473123635	0.02484	-0.325201218	0.12399
KLF3P1	0.797184838	0.05883	-1.181807824	0.0249	0.038109854	0.93269
RPL5P30	0.75528691	0.19789	1.24838424	0.02493	0.971714813	0.09017
CCDC144A	0.131166791	0.52449	-0.464115961	0.02503	0.118433032	0.56743
PSMD11	-0.00825466	0.92722	0.200985852	0.02509	-0.060405023	0.50392
RP11-820K3	-0.277598315	0.55074	-1.124324163	0.02509	0.111746448	0.80307
SNAP29	0.159380586	0.34125	0.374213228	0.02515	0.161570915	0.33474
NDRG1	-0.384064667	0.1916	-0.658182132	0.02523	0.006388274	0.98267
EIF3L	-0.16924549	0.08368	0.218526761	0.02525	-0.028158603	0.77338
ANXA8	1.30521597	0.11444	1.839590236	0.02526	0.813184488	0.32804
TRMT5	0.056567029	0.76312	0.415940773	0.02528	0.051607433	0.78287
POSTN	0.494558686	0.29656	1.059144088	0.02537	-0.659697007	0.16391
ITGB1BP2	-0.678269998	0.10874	-0.940273812	0.02541	-1.281436662	0.00342
MDM4	-0.061654297	0.74954	-0.430358967	0.0254	-0.015268412	0.93708
TMEM44	-0.074338593	0.64132	-0.3574035	0.02558	0.111673247	0.4774
SPTAN1	-0.119446015	0.20866	-0.211947506	0.02563	0.035682281	0.70704
STAR	0.618330305	0.14079	-1.107926691	0.02567	-0.472363628	0.29014

PPP1R1B	0.975005728	0.10208	1.322356594	0.02576	-0.362500199	0.55777
PSMB5	-0.08298048	0.46545	0.251600316	0.02575	-0.142460423	0.20979
SMURF1	0.071765124	0.48045	-0.227195368	0.02576	0.168541461	0.09765
BCOR	0.247075997	0.14037	-0.37516399	0.02581	-0.0753444	0.6538
ARMC5	0.189636027	0.23145	-0.36018606	0.02587	0.218803414	0.17004
CCDC61	0.047151416	0.86792	-0.653800533	0.02587	0.177684531	0.52748
PIGQ	0.110049586	0.35486	-0.268378953	0.02587	0.044896279	0.70679
DPCR1	1.028219859	0.05437	-1.231983723	0.02599	1.436563135	0.00709
USP36	0.126437197	0.48067	-0.401021173	0.02601	-0.071074242	0.69228
SELPLG	-0.609894255	0.15134	-0.940673162	0.02602	-0.112512531	0.77976
SLC9A3R2	-0.074792454	0.67793	-0.402697331	0.02603	-0.187009479	0.29946
RXRB	0.217136408	0.12817	-0.31906381	0.02623	0.143484468	0.31558
PPP1R21	-0.21165625	0.30469	-0.45683644	0.02625	0.048620839	0.81287
B3GALT2	-0.270477551	0.42539	-0.757300121	0.02626	-0.34350717	0.31153
DRAM2	-0.065470586	0.69914	0.373895511	0.0263	0.18231069	0.28026
PREPL	-0.177208915	0.13215	0.259503408	0.02631	0.032395571	0.78236
DEDD2	-0.098697638	0.5674	-0.384176142	0.02637	-0.01333864	0.93824
PIN4	-0.240442939	0.15386	0.36624978	0.02636	-0.100350293	0.54878
ATG16L2	0.074145533	0.69475	-0.425780814	0.02642	0.612555457	0.00106
AMDHD2	-0.261583302	0.36489	-0.647059251	0.02643	-0.318788621	0.27663
RIMS4	0.670694339	0.39475	1.702938877	0.02646	-0.98433297	0.23533
KDM3B	-0.154974878	0.09192	-0.203607203	0.02652	-0.040806974	0.65669
STAM2	0.105544108	0.25025	0.20190017	0.02658	0.138489873	0.13022
NCEH1	0.427978333	0.05242	0.488193615	0.02659	0.405301011	0.06595
LCN9	-0.276847361	0.34771	-0.655998039	0.02662	-0.059907356	0.83814
CTNND1	0.074570485	0.43329	-0.21096249	0.02664	0.144398194	0.12891
NUFIP2	0.194664599	0.19243	0.330823829	0.02667	-0.00243911	0.98697
RCN1P2	0.275855399	0.06559	0.328684131	0.02673	0.087022016	0.56385
C19orf25	-0.085753342	0.54922	-0.318827611	0.02679	0.022877079	0.87264
AC011290.5	-0.191788883	0.54067	-0.723568863	0.02684	-0.165081057	0.59566
KIF21B	0.328037107	0.10332	-0.45895412	0.02686	0.19538176	0.33788
ZNF514	-0.106467069	0.4061	-0.284534265	0.02686	0.0431362	0.73542
AC110926.4	-0.239148177	0.49014	-0.792077839	0.02689	-0.624202759	0.08017
PLD1	-0.357801	0.17715	-0.586541585	0.02689	0.008673279	0.97389
HNRNPRP1	0.760346605	0.08374	-1.19740399	0.02692	0.15050083	0.74426
NDUFA9P1	-0.031141031	0.93804	-0.96685523	0.02691	0.147218532	0.70767
FAM86EP	-0.404259639	0.15103	-0.624902674	0.02701	-0.51852401	0.06527
LPAR3	0.60080898	0.0987	0.792132846	0.02701	0.75419294	0.03562
RLTPR	0.906656655	0.06406	1.047746736	0.02698	0.270393564	0.58483
WASH2P	0.06685424	0.68062	-0.361632774	0.02698	-0.099791506	0.53958
TOP2B	0.013540205	0.89406	0.224341067	0.02704	-0.138675638	0.17259
ZNF35	0.238399795	0.13481	0.343596857	0.02704	-0.063777384	0.69454
MICA	0.039044089	0.78845	0.318508079	0.02718	-0.11220022	0.44187
B4GALNT3	-0.257484525	0.25877	-0.504014692	0.02719	-0.092550989	0.68379
ZNF611	0.06456511	0.73451	-0.421178488	0.02724	-0.213232137	0.26131
C5AR2	0.090815926	0.69381	-0.523110068	0.02728	-0.137821579	0.55439
ADRB2	0.04611473	0.92788	1.078176361	0.02733	1.146632424	0.01914

COX5A	-0.214954002	0.09768	0.282894529	0.02736	0.108275029	0.40074
NME7	-0.068029499	0.7584	0.486437294	0.02736	0.15154278	0.49287
OSBP2	-0.456671568	0.15374	-0.685915226	0.02734	-0.461344923	0.15094
RP5-886K2.	0.447251936	0.13651	-0.697814118	0.02732	0.025953751	0.93228
HABP2	-0.502399602	0.18148	-0.82944544	0.0274	-0.078644449	0.83426
RNF149	-0.15795703	0.2133	-0.278924041	0.02746	0.033458549	0.79112
EFNA1	-0.564208633	0.09804	-0.751968633	0.02748	-0.49543988	0.1461
MSRB1	-0.293358941	0.67022	-1.537707038	0.0275	-0.713972868	0.30432
AMZ2	-0.137903172	0.43871	0.389712229	0.02758	-0.035980218	0.8396
CRELD2	-0.212245021	0.06173	-0.247914	0.02764	0.022742039	0.83936
DNM1L	-0.062422116	0.40676	-0.164688845	0.02773	0.013039139	0.86202
TD-2666L2:	0.302142182	0.50817	-1.215070444	0.02775	0.477050885	0.28565
LUC7L	0.082411547	0.57667	-0.325262938	0.02778	-0.098903522	0.50342
UAP1	-0.141862905	0.26541	0.277605807	0.02777	0.024529884	0.84667
CNTD1	0.360572109	0.20796	-0.694889423	0.02784	0.288841525	0.31669
ISOC2	-0.157276086	0.23369	-0.289538881	0.02788	0.090633294	0.48676
ZNF259	0.011067394	0.93586	0.300277419	0.02789	0.051133416	0.70967
DCUN1D5	-0.045245663	0.69554	0.247555865	0.02797	-0.190902268	0.10012
ZNF524	-0.10650114	0.63111	-0.488485145	0.02801	0.006937602	0.97467
KCNK3	0.469663635	0.20022	-0.857169119	0.02806	-0.731469384	0.06108
KLHL13	0.556330419	0.18486	0.917956006	0.02807	0.104745157	0.80377
RAB38	0.109560928	0.69818	0.613254906	0.02807	-0.545183151	0.05801
PHYHIPL	0.574548517	0.3329	1.284234562	0.0281	-0.018935235	0.97491
FLJ00104	-0.605046922	0.1498	-0.927898281	0.02816	-0.368863878	0.37441
GPATCH4	-0.034017063	0.80068	0.291632817	0.0282	0.104417974	0.43601
SKA2	-0.216761776	0.14035	0.320647589	0.02822	-0.298103214	0.0426
PLCD3	-0.013259996	0.9537	-0.503442445	0.02826	0.063720623	0.77894
AC241952.:	-0.17718805	0.37708	-0.441090142	0.02832	-0.229167714	0.25334
CREB3L1	0.036274221	0.93818	-1.070550721	0.02838	0.500829902	0.27824
ITGB1	0.063493952	0.60287	0.267347448	0.02839	0.086679738	0.47753
MPEG1	-0.160197793	0.64964	-0.814542181	0.0284	0.115516545	0.73595
BRPF3	0.033874127	0.86309	-0.433697983	0.02845	0.137155136	0.48401
PFKFB2	0.160995511	0.34991	0.375641476	0.02845	0.500982311	0.00353
IL17D	-0.256212272	0.14017	0.357964989	0.02849	-0.23744003	0.16919
AC104600.:	-0.134261515	0.75446	-1.026920094	0.0286	0.246504166	0.54996
ADAM10	0.00459824	0.96103	0.205588527	0.02861	0.070370933	0.45436
ERMAP	-0.14918228	0.26444	-0.291633487	0.02859	-0.07128921	0.59287
PPP2R3A	0.039421102	0.77955	-0.308153589	0.02866	-0.198174242	0.16028
ANGPT2	-0.01233338	0.95253	-0.4557563	0.0287	-0.270275709	0.19571
TMEM27	0.503516232	0.13841	0.73867962	0.02871	0.395087874	0.24544
C9orf156	-0.175709372	0.35059	0.399707488	0.02873	0.08431618	0.65043
ENTPD2	-0.476575208	0.3991	-1.29643486	0.0288	-0.317393467	0.58046
BST1	-0.467981342	0.06093	-0.543890974	0.02886	-0.460920099	0.0644
FOXA3	-0.327004072	0.1418	-0.486364299	0.02885	-0.100625011	0.64975
LACTB2	-0.213430758	0.24048	0.390491804	0.02887	0.112022267	0.53558
LYST	-0.041127543	0.88158	-0.604018602	0.02886	0.633786456	0.02133
14-Sep	0.238342238	0.43242	-0.682199111	0.02887	0.047637968	0.87465

ERVW-1	-0.022453295	0.92791	-0.544533661	0.02906	-0.073982122	0.76507
UBXN4	-0.044650208	0.48807	0.139292808	0.02908	-0.030572591	0.63449
CTC-503J8.	1.658042447	0.05345	-2.117549671	0.02917	0.515271022	0.55404
PHF20L1	0.060379151	0.63136	0.273602712	0.02924	0.202031625	0.10847
MAF1	0.056279123	0.64634	0.266404631	0.02925	0.091253598	0.45744
CHI3L2	0.755184362	0.20367	1.244684236	0.02929	0.474996161	0.42343
LPIN3	-0.065567518	0.75842	-0.466057618	0.02928	0.391245711	0.06542
SNAPC5	-0.236891238	0.10645	0.309861084	0.0293	-0.132544835	0.36262
RER1	0.067332354	0.5522	0.245835521	0.02935	0.036323898	0.74834
FAM227A	0.264778703	0.42711	-0.728943084	0.02938	-0.2006726	0.54891
MAP7D1	0.33477493	0.0681	-0.40218275	0.0294	-0.183816588	0.31944
P11-308D1(	-0.03209919	0.86213	-0.410001656	0.02937	-0.149375996	0.41984
SNCA	0.470904401	0.06709	0.558200297	0.02939	-0.191101172	0.46286
CCDC84	0.152155022	0.44262	-0.442166085	0.02949	-0.09326922	0.64193
TINAGL1	-0.19453676	0.28118	-0.392680841	0.02957	-0.320896421	0.07552
CSK	0.07859134	0.64562	-0.373391593	0.02961	-0.123925456	0.46924
P2RY14	0.092630774	0.81789	0.86157517	0.0296	0.359541331	0.36883
11-793H13	0.234767401	0.52507	-0.858846088	0.02959	-0.583439799	0.13517
CUL1	-0.135316049	0.05887	0.153686503	0.02969	-0.03802704	0.59382
LDHAL6CF	-0.445665222	0.18009	-0.727024328	0.02968	-0.120186323	0.71246
RARRES3	0.553418574	0.20757	0.937333502	0.0297	0.625845957	0.15287
EZR	-0.235790111	0.06391	-0.276545533	0.02971	-0.026822222	0.83298
KIAA0895	0.23453807	0.34871	0.538372053	0.02979	0.01647762	0.94726
NEU1	0.177844375	0.27701	0.354359789	0.02981	0.125238493	0.44385
PCIF1	0.321489902	0.05092	-0.362895725	0.02985	0.157661512	0.33973
ZNF844	0.007415283	0.96875	-0.411624738	0.02986	-0.294780639	0.11723
PSCA	0.933472456	0.26836	-1.836183569	0.02987	0.396869989	0.63801
AP3B1	-0.146194173	0.31479	0.314520782	0.02994	-0.009938548	0.94545
P11-548H1ℇ	0.213227082	0.53636	-0.780313477	0.02993	0.231223594	0.50141
NSUN6	-0.291791736	0.0679	-0.343789715	0.02996	-0.210372909	0.18537
AL139099.1	-0.736812065	0.09773	-0.972454352	0.03006	-0.347078312	0.41266
CHRAC1	0.045847212	0.73239	0.288563712	0.03005	0.068006792	0.6112
KLHL3	-0.330084782	0.11418	-0.44999937	0.03008	0.07645348	0.71174
P11-108K1⁄	-0.171134567	0.51312	-0.573409291	0.03013	-0.402264975	0.12895
ZNF517	0.182630692	0.38448	-0.472208269	0.03012	-0.181378991	0.3978
GAA	0.014792165	0.95731	-0.600093546	0.0302	-0.252554981	0.3617
HERPUD1	-0.381457172	0.18842	-0.628591533	0.0302	-0.522334971	0.07168
VPS8	-0.170762577	0.34091	-0.392076595	0.0302	0.112119075	0.53274
MRPL14	-0.091114131	0.36075	0.211204769	0.03024	0.194500254	0.04741
SPCS2	-0.033817992	0.81384	0.309263956	0.03024	-0.072615584	0.61301
P11-210K2(	0.030062499	0.95636	-1.326056289	0.0303	0.213121488	0.69389
CXorf23	-0.164749059	0.3595	-0.387354396	0.03034	-0.195604464	0.27487
GNB5	-0.182702468	0.22124	-0.319570854	0.03034	-0.225197739	0.12891
NADK	0.007200869	0.94455	-0.223309803	0.03035	0.195539345	0.05739
AOX1	-1.046219525	0.0611	-1.204846542	0.03038	-0.197038462	0.7243
TMEM206	0.354686367	0.19871	0.592389405	0.03039	-0.136779057	0.62341
NR4A1	0.179542017	0.57353	-0.697450188	0.03041	-0.32493592	0.31169

CIAPIN1	0.11785168	0.34082	0.265250411	0.03045	0.208593612	0.09092
SEC61B	-0.103776187	0.3306	0.228152254	0.03047	-0.080761313	0.44813
RAC3	-0.149117646	0.45878	-0.434091754	0.03057	-0.292086279	0.15009
ELOVL7	0.167860966	0.44328	0.470045838	0.03061	-0.023466357	0.91478
FAM102A	0.303751307	0.14197	-0.4501401	0.0307	-0.067583677	0.74421
LARS	-0.157861414	0.11108	0.212862195	0.0307	-0.077509815	0.433
WDR13	0.055511176	0.61953	-0.242605903	0.03068	0.048883656	0.66125
DSCR3	-0.047066427	0.59702	-0.191531235	0.03077	0.074283762	0.40216
SPN	0.267059153	0.33421	-0.611756563	0.03082	0.241255934	0.38349
OTUD4	0.159382412	0.17207	0.25093325	0.03087	0.095149253	0.41466
FOLR1	0.476822475	0.16584	0.734333858	0.03094	-0.254737384	0.47145
SLC25A45	-0.055016928	0.87054	-0.732059045	0.03095	-0.422915758	0.2108
RGS11	-0.583397185	0.23446	-1.099207759	0.03101	-0.300527734	0.54656
ARFIP1	0.216600091	0.15552	0.327390998	0.03104	0.405328326	0.00768
RNPEP	0.004170365	0.97236	-0.25939058	0.03106	0.111533417	0.35176
TNFRSF25	-0.191753408	0.42547	-0.530616976	0.0311	-0.975239999	6.57E-05
SLC30A3	0.533076669	0.24643	-1.082169148	0.03113	0.040056934	0.93228
CKAP2	-0.171325714	0.263	0.328222572	0.03115	0.070650677	0.64402
NNT	-0.191230957	0.15264	0.287105974	0.03117	-0.043314384	0.74545
JD7-PLA2G	0.06248448	0.85309	-0.732467165	0.03133	0.019104933	0.95486
RFX7	0.276980822	0.13539	0.39778262	0.03138	0.464066686	0.01218
TIFA	0.296704637	0.22118	0.518727416	0.03142	0.27496073	0.25668
FYTTD1	-0.03674386	0.7748	0.275421716	0.03144	-0.198701553	0.12198
PPP2R5E	0.144472996	0.21636	0.249568547	0.03148	0.012078465	0.91762
TRA2B	0.072080421	0.49978	0.229355532	0.03148	0.004853658	0.96375
ZNF527	-0.183082146	0.33061	-0.404863982	0.0315	-0.093945025	0.61866
ACTC1	0.532702466	0.59726	2.147406576	0.03156	0.188084772	0.85263
APAF1	-0.041426032	0.78646	0.327690423	0.0316	-0.017122561	0.91076
BRF1	-0.078908596	0.61832	-0.342271229	0.0316	-0.012525394	0.93699
GLUD1	-0.085434447	0.35135	0.196201562	0.03159	0.167415135	0.06705
MCM3AP	-0.046627393	0.72189	-0.281617629	0.0316	-0.153445439	0.24126
TBKBP1	0.442503365	0.15448	-0.674619805	0.0316	-0.339069366	0.27939
EGFR	-0.0482654	0.82827	-0.478166816	0.03169	-0.181061894	0.41595
XPNPEP3	0.024499341	0.85701	0.290974752	0.03168	0.262574496	0.05286
EXOSC3	-0.198455515	0.15646	0.295779154	0.03181	-0.043139207	0.75669
PABPC1L	0.056546179	0.76032	-0.403327869	0.03177	-0.000404989	0.99824
PPP1CB	0.035153729	0.67851	0.181717892	0.03174	0.079917801	0.34566
RNF20	-0.126486419	0.12116	0.173176422	0.03176	-0.020026174	0.80494
P11-192M23	0.013929564	0.97681	-1.158091026	0.03174	-0.053929531	0.91058
P11-229P13	0.228903936	0.52859	-0.848616612	0.03181	-0.005088441	0.98899
ZNF503	0.254003615	0.33475	-0.573039174	0.03172	-0.483866194	0.0701
ERCC6	-0.066808155	0.83744	-0.69707295	0.03183	-0.003132996	0.9923
C1orf115	0.471856548	0.2626	0.90008125	0.03193	0.293738888	0.48601
CTCFL	-0.069159994	0.80237	-0.60996836	0.03194	0.160897719	0.55346
GPX1	0.03279082	0.90591	0.589183466	0.03193	0.05043382	0.85561
ANKRD17	0.045436449	0.63073	0.202215156	0.03202	0.118101844	0.21115
C10orf54	0.091283082	0.81413	-0.841287295	0.03206	0.05303153	0.89149

MAL	0.044502092	0.95779	-2.016894593	0.03212	0.413031345	0.61926
MMP13	0.824414448	0.31445	1.738942256	0.03215	1.605967678	0.04886
RHBDL2	-0.265431706	0.48392	-0.839625632	0.03214	0.681297472	0.05831
NDUFAF2	-0.243247812	0.14712	0.35057045	0.03217	-0.024467104	0.88277
CD151	-0.195836521	0.05118	-0.214380274	0.03219	-0.046567004	0.64185
P11-333E1	0.185585978	0.74662	-1.410517136	0.03225	-0.012432898	0.98291
P11-274B2	0.695610101	0.1564	-1.153982516	0.03228	-0.21103258	0.67868
IVM-ERCC	0.117942068	0.54351	0.412842639	0.03232	0.427319694	0.02695
AC005517.	-0.283318492	0.41178	-0.759466707	0.03234	-0.479248112	0.17073
NUPL2	0.064942693	0.62882	0.282970948	0.03254	0.123399749	0.35573
HAX1	-0.083854047	0.49469	0.258496024	0.03257	0.010175716	0.93352
NMUR1	-0.090676284	0.80925	-0.86551044	0.03257	0.515186387	0.14497
SLC9B1	0.021790862	0.95856	-0.960075898	0.03259	-0.62537442	0.15242
SLC16A3	0.317513412	0.34121	-0.714463088	0.0326	0.598586949	0.07263
PABPC1	0.108316519	0.57797	0.415911691	0.03261	0.311949269	0.10904
ATP5L	-0.017800251	0.85815	0.211382668	0.03263	0.079262391	0.42471
SIRT2	0.108582851	0.37774	-0.263872272	0.03267	0.179655293	0.14188
GPLD1	-0.454622336	0.12421	-0.625716426	0.03278	0.151874436	0.60295
SERINC1	-0.04764563	0.65448	0.226600125	0.03277	-0.11219539	0.29198
ST3GAL5	0.337855176	0.05326	0.369588786	0.0328	0.015679077	0.92912
ZNF133	-0.364369904	0.11232	-0.49007557	0.03281	-0.104645006	0.64746
CBLN2	0.094624773	0.80201	-0.810091965	0.03292	-0.21627411	0.56729
REC8	-0.498895793	0.09196	-0.631476824	0.03293	0.119442683	0.68447
P11-397P1	0.628955116	0.21236	-1.252623365	0.03292	0.304036878	0.5532
CNNM1	-0.077146599	0.7806	-0.594529502	0.03298	0.028991103	0.91645
PLCL2	0.312795395	0.24429	0.567015428	0.03297	0.408284533	0.12689
RPL32	-0.142655325	0.20858	0.241696054	0.033	-0.013994802	0.9018
PLA2G4D	-0.841388328	0.17941	-1.372632123	0.03305	-0.513165027	0.40163
OPN3	-0.176921299	0.22978	0.306367478	0.0331	-0.150488214	0.30763
GPC3	0.468732368	0.18021	0.744671121	0.03313	0.170287482	0.6266
AC079807.	-0.227104255	0.6452	-1.142715195	0.03322	-0.814904445	0.11935
RAB2A	-0.026979399	0.74447	0.174782435	0.03327	0.097625528	0.23646
DCP1B	0.206876503	0.166	0.31412788	0.03337	0.100463416	0.50136
DDX49	0.291852126	0.07629	0.34865021	0.03337	0.331686626	0.04394
EPS15P1	-1.04185384	0.07089	-1.221564754	0.03336	-0.201044497	0.7094
GTF2H1	-0.062321024	0.44272	0.169325445	0.03336	0.078139842	0.33205
MMP11	-0.058854159	0.80703	-0.520453291	0.03338	-0.301072363	0.22866
NME1	-0.205535636	0.18114	0.325422461	0.03335	-0.015891163	0.91749
PLA2G15	0.16420048	0.25942	-0.310585757	0.03338	-0.194208258	0.18556
WTAP	0.101538392	0.26835	0.193832148	0.03336	0.228034364	0.0125
DDX12P	-0.108671412	0.70455	-0.618804007	0.03343	-0.041468037	0.88441
PKM	0.058979312	0.5493	0.209392503	0.03347	0.158387001	0.10778
PGBD4	0.029400473	0.85574	-0.34718384	0.03366	0.096843927	0.54693
AKAP11	0.079377671	0.29213	0.158889254	0.03368	0.221949463	0.00307
QSOX2	-0.099448309	0.69108	-0.536698583	0.0337	-0.162740521	0.51585
APTX	-0.236078888	0.07577	0.276305371	0.03375	-0.102822174	0.43702
PTPN12	0.265712784	0.12048	0.361965505	0.03381	0.193610515	0.25763

P11-290L7	0.62555684	0.34233	-1.630318498	0.03384	-0.362520148	0.60035
ARCKSL1F	-0.160409898	0.74952	-1.181019669	0.03387	-1.373901268	0.01887
C6orf120	-0.160399032	0.05029	0.169814685	0.0339	-0.182912489	0.0254
GALNT1	-0.123847451	0.33418	0.271466926	0.03395	0.004698697	0.97075
ZNF418	-0.235674798	0.331	-0.5183717	0.03401	-0.378283427	0.12614
RTCA	-0.182385866	0.09104	0.225209445	0.03407	-0.071393345	0.50741
CTIF	0.352721064	0.08651	0.436239755	0.03414	-0.091077772	0.66021
INPP4A	0.136833471	0.42416	-0.363620176	0.03414	-0.044286177	0.79608
LGALSL	-0.036785799	0.81524	0.329270551	0.03414	0.133527313	0.39381
SUV420H2	-0.174240697	0.65617	-0.83792413	0.0343	-0.275003701	0.48219
ENPP7P12	0.20295291	0.72113	-1.278620374	0.03439	-0.322325437	0.57738
NRIP1	-0.061413287	0.46047	0.175118399	0.03441	0.061454459	0.45923
TPRG1L	-0.029958077	0.8258	0.283141478	0.0344	0.15185886	0.26034
ZNF236	0.364102669	0.09844	0.464178005	0.03454	-0.105548324	0.63152
FBXL14	-0.176135281	0.44742	-0.492824109	0.03456	-0.369164519	0.11206
ATXN2	0.086530609	0.46563	-0.2506321	0.03479	-0.045489771	0.70187
CPPED1	-0.037961473	0.85537	0.434127998	0.0348	-0.130018147	0.53328
GPR64	0.060286272	0.87781	-0.833139516	0.03478	0.471054919	0.22833
PCNT	-0.119923242	0.42521	-0.31740834	0.03483	-0.055491417	0.71161
RPL18AP3	-0.0750912	0.60069	0.302101048	0.03488	-0.104098545	0.46805
TRANK1	-0.158140623	0.43981	-0.431012378	0.03496	-0.507028775	0.01353
C2orf62	-0.273488112	0.52919	-0.953787872	0.03506	0.065085509	0.88
NIPAL4	0.436353408	0.43911	-1.308108975	0.03509	-0.028303311	0.96101
VSIG2	-0.020204548	0.94757	-0.647611015	0.03519	0.36651442	0.2326
GOLGA3	0.13470435	0.32853	-0.290575788	0.03522	0.114515773	0.40613
HSF4	0.279606345	0.51293	-0.949270341	0.03523	-0.193224905	0.65603
NECAP1	0.104507955	0.56213	0.377774781	0.03525	0.285725154	0.11198
MTMR2	-0.150672592	0.12121	0.202621634	0.03532	0.116107752	0.22971
BET1L	0.249517249	0.05468	-0.275166834	0.03536	0.209303218	0.10741
MTA1	0.215270344	0.08956	-0.26766532	0.03536	0.172116598	0.17513
P11-17M15	0.247555248	0.55616	0.838252492	0.03538	-0.095123391	0.82585
IFIT3	0.530275512	0.06216	0.59424445	0.03539	0.486961417	0.08661
KCNJ16	-0.41359457	0.2027	-0.682776223	0.03543	0.207808578	0.52114
ALMS1	0.53102208	0.1293	0.736591628	0.03545	-0.003891395	0.99115
QKI	0.258066876	0.05351	0.280891272	0.03548	0.059000798	0.65933
CALU	-0.040652476	0.70615	0.22630166	0.03555	-0.169058829	0.11703
PRKD3	0.164132035	0.23769	0.291378384	0.03555	0.225972772	0.10365
RPRM	-0.188877937	0.74946	-1.340011973	0.03553	-0.03100964	0.95771
SLC4A3	0.13990341	0.6385	-0.632972184	0.03553	-0.274279551	0.36323
ZNF208	0.548226599	0.10459	-0.841888989	0.03555	0.648895744	0.05205
SIAE	-0.150401219	0.28677	0.29528581	0.0356	-0.005438982	0.96922
TMEM123	-0.184788212	0.37423	0.436632412	0.03564	0.004369147	0.98323
UPRT	-0.087083011	0.59288	0.334813883	0.03564	-0.114204251	0.48299
ABCA17P	-0.219893206	0.76808	-1.583142545	0.03567	-0.854767694	0.25774
M110-MUS	-0.971703054	0.10283	-1.266362295	0.03571	-0.83475788	0.15741
SYAP1	-0.189516594	0.11051	0.248819884	0.03579	-0.052552363	0.65785
S1-124K5.	-0.368503633	0.17582	-0.578190598	0.036	-0.454639205	0.09861

GALNT14	0.38239185	0.48337	1.087056258	0.03613	0.290745674	0.6003
USF2	-0.050228348	0.6876	-0.262424535	0.03613	-0.060941476	0.62517
NPAS1	-0.325459651	0.47659	-1.037442916	0.03616	-0.354892398	0.45853
TUBB4B	-0.029320373	0.82652	0.279835379	0.03621	0.067223449	0.61516
B4GALT6	0.112352779	0.58236	0.422744331	0.03631	-0.128823764	0.52987
TAS2R3	-0.439189355	0.43456	-1.229400538	0.03632	-0.732368428	0.19973
TRPV4	0.129808364	0.67924	-0.677514976	0.03631	-0.148674612	0.64015
ORAOV1	0.092513445	0.51798	-0.304411594	0.03639	-0.138387959	0.33574
C16orf58	0.057212866	0.61583	-0.238700244	0.03641	-0.016998777	0.88139
CHMP1B	0.08301917	0.42237	0.215275386	0.03641	-0.032443264	0.75418
DRAXIN	-0.059827498	0.83934	-0.633115282	0.03646	-0.166180163	0.57467
INTS9	0.062342721	0.6455	0.278209137	0.03646	0.142793648	0.28907
CC2D2A	0.110801097	0.46648	-0.31666358	0.0365	-0.126001606	0.40772
RREB1	-0.156227447	0.3274	-0.33409581	0.03665	-0.087056641	0.58524
HSPE1	-0.25467604	0.05873	0.280102937	0.03668	0.103492712	0.44102
SCRG1	0.106987933	0.72212	-0.639867402	0.03673	0.050323971	0.86728
SFXN1	-0.088172407	0.43901	0.236745562	0.03674	-0.235498338	0.03876
SMARCA2	-0.366462501	0.05254	-0.394162645	0.03675	-0.056718048	0.76341
CCDC59	-0.208277437	0.12002	0.274385789	0.03678	-0.137427113	0.30226
SLC48A1	-0.069868978	0.72232	-0.413238145	0.03681	0.047404863	0.80788
ZC3H7A	-0.063228564	0.68759	0.326683512	0.03684	0.017987089	0.90883
IL22RA1	0.635136572	0.0622	0.706169249	0.03686	0.548010514	0.10798
NDUFC1	-0.216112942	0.05002	0.221281956	0.0369	-0.183225553	0.09411
IL33	-0.550636349	0.23404	0.948120085	0.03701	-0.531393201	0.24895
NPC1L1	0.178827857	0.43287	-0.477765526	0.03701	0.275126687	0.22703
SEC14L1P1	-0.029539119	0.91916	-0.628499528	0.03697	-0.093012257	0.74959
ZNF320	-0.025402337	0.85074	-0.280891456	0.03702	0.104402038	0.43763
HPS5	-0.125450444	0.40643	-0.312292093	0.03707	-0.185233388	0.21865
LSM12P1	0.05421001	0.87875	0.727898567	0.03711	0.735954433	0.03545
DSTN	-0.160024008	0.09758	0.200552175	0.03717	-0.03437453	0.72155
GRIP1	0.315689743	0.39153	0.766897848	0.03715	0.005088851	0.98908
GYPC	0.325044495	0.07378	0.377376016	0.03716	-0.27980286	0.12842
HLA-B	0.372581227	0.18582	0.586237684	0.03717	-0.165491837	0.55726
PPP2R3C	0.086924194	0.54122	0.292367593	0.03714	0.183507883	0.19618
PPP6C	-0.006990156	0.92766	0.158345747	0.03724	0.026372611	0.73116
FKBP11	-0.237908844	0.24165	0.419255077	0.03727	0.052819954	0.79406
MRPS18C	-0.195985652	0.10603	0.248497812	0.03727	-0.138177665	0.25224
-42389800	0.043507003	0.75864	-0.296322083	0.03734	0.210020434	0.13543
KIAA1456	0.111991703	0.65169	-0.531596861	0.03737	-0.170802624	0.49542
7-Sep	0.026459331	0.82819	0.253349755	0.03738	-0.111956053	0.35863
C006116.2	-0.599417592	0.1863	-0.961983049	0.03741	-0.720165512	0.11323
DTX2	0.232705444	0.19331	-0.376077156	0.03743	0.252200519	0.15918
IP6K2	0.057627032	0.4909	-0.174260736	0.03742	-0.029153095	0.72769
SART3	-0.088294068	0.2329	-0.152161194	0.03744	-0.112976714	0.12591
UBE2A	0.075074972	0.78955	-0.593052115	0.03747	0.231090542	0.41206
C17orf104	0.04028226	0.87029	-0.521129395	0.03753	0.080596332	0.74468
KLF4	-0.182582403	0.54523	-0.62866266	0.03752	0.395940759	0.18865

PFAS	0.039995239	0.79898	-0.326847093	0.03753	-0.058275869	0.71072
POMT2	0.199231743	0.17694	-0.310045741	0.03758	-0.178999885	0.22779
KHDRBS2	-0.229728474	0.59535	-0.936066449	0.03764	-0.315623412	0.46674
P11-214J9	-0.174351839	0.71135	-1.088801853	0.03768	-0.497077751	0.30884
UBFD1	0.134302335	0.17906	0.20693868	0.03773	0.047763629	0.63301
FRMD4B	0.08423486	0.64728	-0.382581932	0.03778	0.124114681	0.49986
HSP90B1	-0.209105365	0.09237	0.257969963	0.0378	-0.090051231	0.46853
NKD2	-0.042525167	0.91569	-0.893742074	0.03779	0.082587528	0.83534
TIMM50	0.134154001	0.18233	0.207187513	0.03778	0.146301101	0.14468
VSIG10L	-0.22771086	0.45533	-0.644744668	0.0378	-0.443443416	0.15085
WTAPP1	0.112522096	0.80762	-0.991896156	0.03779	0.352154398	0.44099
P11-497H1(	-0.316649126	0.56492	-1.160361673	0.03783	-1.170178359	0.03813
DISP2	-0.060764845	0.83921	-0.63330852	0.03788	0.143723596	0.62834
USP3	-0.09743207	0.40834	0.24280321	0.03792	0.065063697	0.57976
HAUS6	-0.220218386	0.0596	0.239450602	0.03794	-0.148893285	0.2014
GPBP1L1	0.129813824	0.23212	0.224841152	0.03796	0.265633725	0.01436
ZRANB1	0.076339818	0.46337	0.21471699	0.03804	0.115793239	0.26515
GTDC1	0.203764969	0.14298	0.28699951	0.03806	0.244949522	0.0776
CCDC83	-0.970915708	0.11719	-1.29668746	0.0381	-0.700701624	0.2425
TRIM45	-0.385163108	0.19102	-0.609617161	0.03809	-0.320379701	0.27647
CCDC57	-0.054995367	0.7343	-0.338450728	0.03813	-0.258589039	0.11155
ATP8B3	0.26063245	0.42105	-0.684399551	0.03818	0.117254234	0.7183
CEP68	-0.118397445	0.42515	-0.307540388	0.03821	-0.153637382	0.30106
ALX4	-0.128463793	0.85175	-1.550937914	0.03827	0.088413387	0.89646
LZTS2	0.163538168	0.22155	-0.277907288	0.03829	0.016846227	0.89991
RP11-1J11.	-0.742925611	0.1374	-1.045335077	0.03828	-0.217208476	0.65286
NOL6	-0.074354663	0.57427	-0.273624311	0.03832	-0.25088077	0.0579
BTNL3	1.058874461	0.15422	1.529518228	0.03841	0.757099118	0.30981
PRSS36	0.339102532	0.55309	1.179379769	0.03843	1.221535293	0.03046
DNTTIP2	-0.164873654	0.21684	0.27489036	0.0385	-0.240231204	0.07174
MYL12A	-0.059719206	0.48252	0.175305117	0.03853	-0.07755501	0.36153
RECQL4	-0.313773286	0.26919	-0.58895314	0.03853	-0.159104106	0.57273
KALRN	-0.235913425	0.08198	-0.280101288	0.03855	-0.023803787	0.8604
ELMO3	-0.183241186	0.17832	-0.278920134	0.03856	0.153843853	0.24482
TMEM182	-0.045123079	0.82151	-0.413945293	0.03861	-0.160723802	0.42174
NAAA	0.598795865	0.09428	0.728236492	0.03864	0.123803422	0.73391
FAM185BF	-0.205414163	0.671	-1.044166844	0.03881	-1.191654165	0.02137
FRZB	-0.161708658	0.48727	-0.481427914	0.03887	-0.081791813	0.72502
CDCA5	-0.390656437	0.05344	-0.414973787	0.03888	-0.385695749	0.05574
SLC6A4	0.471930868	0.29913	0.940827895	0.03898	-0.020668917	0.96469
KIAA1875	0.247816816	0.41041	-0.653455719	0.03902	0.280641828	0.34191
HIATL1	-0.00211183	0.98141	0.185476376	0.03905	0.128865786	0.15325
IKZF3	0.102067426	0.64431	-0.465187155	0.03904	0.180677804	0.41155
TBC1D9B	0.064332228	0.55354	-0.223936184	0.0391	-0.066318181	0.54141
AGAP1	0.063684133	0.68414	-0.322213392	0.03929	-0.123670766	0.42968
FBXO28	0.08349769	0.44119	0.221219364	0.03931	-0.049928201	0.64399
HTRA4	-0.104051526	0.68296	-0.534639446	0.03931	-0.161695425	0.52593

KRT19	-0.133419591	0.30676	-0.269021308	0.0393	-0.065873001	0.61378
S100PBP	-0.005856882	0.97247	-0.3490005	0.03927	-0.277989005	0.1021
MDFIC	0.178711545	0.45302	0.488897158	0.03939	-0.055197481	0.81685
TD-3214H1!	0.297922698	0.32092	0.611149349	0.03941	0.043622543	0.88494
KAZALD1	-0.256688497	0.26098	-0.468811183	0.03949	0.174652634	0.43643
C19orf44	-0.095441104	0.6689	-0.464686443	0.03955	-0.104150994	0.64431
FAM96B	0.128650755	0.40304	0.313829529	0.03954	0.107370595	0.48553
SNX10	-1.132936296	0.11852	-1.517382832	0.03959	-2.425658042	0.0014
CTNNBIP1	-0.180043787	0.16215	-0.263994104	0.03973	-0.049669328	0.69971
DYRK1B	0.048858774	0.788	-0.386571053	0.03976	-0.21147583	0.25644
ACBD3	0.028617381	0.85927	0.331164669	0.03986	0.088841925	0.58174
APOBEC3F	0.080255007	0.74639	0.50561281	0.03986	0.071319537	0.77385
DCDC1	-0.220027978	0.64055	0.945264253	0.03987	0.752135951	0.10287
MROH7	-0.070058087	0.84812	-0.760403925	0.03985	0.101755235	0.77945
MPHOSPH!	-0.088376059	0.51905	0.278951497	0.0399	-0.150609247	0.27253
ZFPM1	-0.171129779	0.55268	-0.595279124	0.03991	0.421095866	0.14346
STAU2	-0.081268778	0.47804	0.232667169	0.03999	0.094799964	0.40567
DSG2	0.089405201	0.47098	0.25439047	0.0401	0.295012359	0.01731
MTDH	0.028262226	0.82959	0.268691626	0.04035	0.101770548	0.4381
TG	1.169877565	0.06694	1.295218583	0.04037	0.810424614	0.20563
RPL23AP82	0.343634006	0.07769	0.396916768	0.04043	0.180464682	0.35455
GPRIN3	-0.443886919	0.15951	-0.646271787	0.04062	-0.643843268	0.04158
RPL23A	-0.223369023	0.10391	0.280984979	0.04065	-0.021637395	0.8748
FKBP1A	0.064340859	0.63895	0.279483266	0.04071	0.126178516	0.35721
AKR1E2	0.017235055	0.96315	-0.81968058	0.04077	0.162446499	0.66112
TIMM8B	-0.169051967	0.12481	0.220343608	0.04078	0.022583242	0.83593
PDCD2	-0.204758058	0.05612	0.216229156	0.04079	-0.0044363	0.96679
RASA4	0.346708979	0.17317	-0.528307329	0.04081	0.234531587	0.35783
IGFN1	1.337529812	0.24635	2.339216158	0.04088	1.510342956	0.18907
NBPF1	-0.125429759	0.41398	-0.313631972	0.04091	0.029626783	0.8464
AC007401.1	-0.007130981	0.9863	-0.904398098	0.04097	0.368020239	0.36248
RAB18	0.00312499	0.97502	0.203291438	0.04096	-0.011288937	0.90993
GLB1L	-0.363219403	0.15997	-0.523459972	0.041	-0.37284351	0.14805
RABL6	0.012956388	0.88729	-0.187071587	0.04101	0.040602805	0.65661
ATG4A	0.171013842	0.35807	0.376566231	0.04104	0.631618562	0.00061
ADRBK1	0.101189842	0.51249	-0.315924139	0.04115	0.055319446	0.72032
MRPS9	-0.095365078	0.44027	0.246946425	0.04129	0.086208413	0.48118
SEC14L5	-0.53055178	0.059	-0.562992777	0.04128	0.102275898	0.6994
BLOC1S3	0.135020533	0.49475	-0.410449834	0.04142	0.168096363	0.39264
CHURC1	-0.263286785	0.05066	0.271476515	0.0414	0.10995798	0.41103
CYP1A2	0.161741859	0.5796	-0.629386623	0.04138	0.115546502	0.69229
FAM83G	-0.086072846	0.61537	-0.349855452	0.0414	-0.117659056	0.49294
FCHO2	-0.001068076	0.99435	0.306025483	0.04138	0.234367064	0.11908
NAA25	-0.016467266	0.89881	0.261975704	0.04138	0.076196988	0.55493
ZNF587B	-0.180626152	0.15831	-0.258734016	0.04156	0.097565268	0.43995
MTMR14	-0.192727719	0.06178	-0.209371155	0.04162	0.004598359	0.96428
NAIP	-0.166881492	0.48859	-0.496941149	0.04163	-0.567349609	0.02352

PGM3	-0.372019961	0.06381	-0.408195831	0.04163	-0.098219753	0.62408
GBA3	1.36260264	0.19361	2.130604806	0.04168	1.172114174	0.26393
UBE2Q2P3	0.070175853	0.83379	-0.710064042	0.04167	-0.163988007	0.6262
P4HA3	0.222141151	0.69139	-1.165280105	0.04173	-0.637111155	0.26345
EIF2S3	-0.090208454	0.43325	0.234144815	0.04178	0.067241648	0.55898
CBLL1	0.303339755	0.05676	0.322438872	0.04183	0.161896052	0.30926
ZNF341	0.126288232	0.49371	-0.390579582	0.04185	0.067493375	0.71519
CCDC12	-0.10501606	0.37787	-0.241988112	0.04191	-0.055122358	0.64198
LHFP	0.759089772	0.07073	0.853279621	0.04195	0.346711828	0.40998
SLC52A3	0.147014822	0.56077	-0.530541885	0.04193	0.120302766	0.64177
TICRR	-0.072314282	0.68157	-0.359062433	0.04196	-0.07964936	0.65093
VSTM4	0.028052116	0.90919	-0.507453531	0.04196	0.015056712	0.95102
BPGM	-0.179801504	0.32067	0.359880964	0.04199	0.066367914	0.71112
HNRNPA0	-0.105250798	0.36537	-0.236208222	0.04211	-0.15980987	0.16933
TBXA2R	-0.801415387	0.07939	-0.922084427	0.04222	-0.485625452	0.27729
ROBO2	0.200684442	0.49648	-0.601424899	0.04237	-0.3223225	0.27627
PCDHB4	0.248288217	0.52299	-0.831286722	0.04242	-0.524586468	0.19487
DHRS1	-0.126995159	0.37774	-0.29332572	0.04243	0.258209988	0.07065
GGCX	-0.157549725	0.14727	-0.219685267	0.04247	-0.192294299	0.07663
NT5E	0.139250982	0.65893	0.638356319	0.04247	0.684804415	0.02958
PRKAA1	0.012272985	0.89493	0.187823704	0.04252	0.069996408	0.4504
TRIM73	0.192263755	0.52306	-0.625409475	0.04258	0.249493882	0.40948
FAM3A	-0.145596411	0.48193	-0.420433215	0.04266	0.264824172	0.19718
PDE8B	0.396840299	0.16005	0.565881212	0.04262	0.360350356	0.20101
SERPINB9	-0.415353408	0.0879	-0.492846533	0.04275	-0.573475298	0.01852
AOC1	1.465973242	0.10031	1.804043984	0.04279	1.604141429	0.07221
ADAM3A	0.183564586	0.71555	-1.134275606	0.04288	-0.981594917	0.08202
CA6	-0.005620609	0.98421	-0.607659155	0.04289	-0.082787119	0.77148
NOMO2	0.982688926	0.05825	1.050277001	0.04288	0.584837069	0.25999
PREP	0.001051683	0.99301	0.241384843	0.04289	0.1203851	0.31481
TBCEL	0.136582857	0.40538	0.328685236	0.04286	-0.301357935	0.06812
YARS2	-0.174454434	0.22064	0.283450984	0.0429	-0.174606569	0.21918
DUSP1	0.314945106	0.39774	-0.759255953	0.04294	-0.397716289	0.28793
SLC25A5	-0.17912184	0.09322	0.215007029	0.04296	0.080569726	0.44901
BBIP1	0.047144308	0.73854	0.285692974	0.04301	-0.081074928	0.5645
MINA	0.079156841	0.6437	0.342979106	0.04303	0.151521352	0.37589
MYBPC2	0.884456052	0.13728	1.176765505	0.04303	0.461375573	0.45763
PCMT1	-0.172052298	0.09406	0.203732264	0.04307	0.011367745	0.91107
C12orf66	-0.012150351	0.95368	0.411520281	0.04314	-0.115150674	0.5813
NT5C3A	-0.206070922	0.14712	0.283488195	0.04316	0.078530197	0.57743
DDX17	0.096878582	0.2365	-0.165446639	0.04326	0.111285022	0.17383
MAP2K3	0.39896209	0.10852	0.492102254	0.0433	0.583890093	0.0166
RP1-283E3.	0.332733283	0.15239	-0.486607569	0.04329	0.249940312	0.28005
CEP104	0.081381499	0.65515	0.367462669	0.04338	0.29702458	0.10279
SLC38A1	-0.086102837	0.49623	0.255061655	0.04344	0.049483314	0.69558
TSEN34	-0.801801216	0.067	-0.880476677	0.04345	-0.88152273	0.04436
LSMEM1	0.320740515	0.45523	-0.922163377	0.04352	-0.233154972	0.59022

EMC3-AS1	0.0461645	0.87271	-0.592778887	0.04359	-0.162624198	0.57461
RAB21	-0.035212952	0.74569	0.218371191	0.04362	-0.025329134	0.81554
TLR2	0.057703951	0.80083	0.450827357	0.04362	0.445926716	0.04707
DGCR14	0.046696261	0.74827	-0.296454611	0.04364	-0.057775619	0.69212
C2orf68	-0.056702096	0.65222	-0.253536801	0.04374	-0.081567149	0.51477
TCTN1	-0.151010733	0.21695	-0.246426961	0.04374	-0.270289428	0.02756
KIF26B	0.494214242	0.1543	-0.704565999	0.04383	-0.656344754	0.06196
SLC25A10	-0.439128896	0.05134	-0.452177575	0.04383	-0.02594223	0.90779
TOP3A	0.046498474	0.72836	-0.270218995	0.04385	-0.252899311	0.06016
EEF1A1P1	-0.198981739	0.29256	0.37422405	0.04388	-0.035845634	0.84874
TTC18	-0.445846135	0.11573	-0.577633139	0.04393	-0.394471801	0.16178
ESRP2	0.097081843	0.44588	-0.257002382	0.04394	0.420420141	0.00091
POC1B	0.028691906	0.86337	0.333928696	0.04396	0.289364922	0.08184
5P-PVRIG2	0.205055559	0.32647	-0.4238276	0.04397	0.271338788	0.19359
CCDC153	-0.216303027	0.54769	-0.763345622	0.044	-0.272835017	0.44843
PGP	-0.241556347	0.08022	-0.27654975	0.04402	-0.053709411	0.69528
TBL2	0.116119901	0.52103	-0.36552219	0.04402	0.141939705	0.43248
DDX20	-0.109229373	0.39624	0.25503859	0.04411	-0.144598371	0.26159
HDAC11	-0.003505747	0.98604	-0.401054937	0.04421	-0.073688568	0.7142
HOGA1	-0.57751175	0.16141	-0.834738874	0.04422	-0.154021564	0.7043
MTRNR2L3	-0.233606128	0.66516	-1.178808114	0.04421	-0.611783278	0.27222
RSAD1	-0.277246341	0.07143	-0.304246345	0.04426	-0.073641027	0.62641
ASNSP1	0.706556205	0.07847	0.795276352	0.0443	0.503745396	0.21417
CHMP2A	-0.145396361	0.07348	0.159345829	0.04439	-0.009237628	0.90851
AC022182.2	-0.389000462	0.38604	-0.935607338	0.04445	-0.372834503	0.40347
ME1	0.281445967	0.17459	0.414856083	0.04457	0.133852145	0.51903
SOX6	0.119154479	0.55051	0.398847243	0.04459	0.315410655	0.1133
ATP6AP1	0.078412022	0.43441	-0.201467795	0.04464	-0.016797247	0.86704
CHKB	0.109891263	0.41585	-0.274370144	0.04464	0.065319763	0.62947
GGPS1	-0.097187177	0.27902	0.176988706	0.04462	-0.117541973	0.1906
PRPF18	-0.272945581	0.05541	0.2762272	0.04468	-0.112711001	0.4221
FAM124A	-0.129013672	0.75466	-0.862129683	0.04477	-0.410592735	0.32708
PRPF4	-0.185031309	0.05636	0.191358499	0.04477	-0.141342939	0.14375
NPAP1	0.134529976	0.64501	-0.604935852	0.04481	-0.320664563	0.28293
PP2D1	0.289557231	0.4911	-0.921160597	0.04482	0.133050785	0.75284
NGRN	-0.00644324	0.94037	0.17116785	0.04484	0.017853339	0.83584
ZNF506	0.028832914	0.81671	-0.251592671	0.04484	0.127924959	0.30475
CCDC30	-0.220941218	0.34318	-0.472468858	0.04488	-0.30342395	0.19546
ZNF536	-0.006828565	0.98953	-1.07432496	0.04488	-0.274310758	0.60252
SHE	0.259289121	0.52382	-0.871721561	0.04492	0.170991085	0.67488
ARL5B	-0.060567157	0.55772	0.206551804	0.04498	-0.10298586	0.31884
FEM1A	-0.139428244	0.17796	-0.206545813	0.04506	-0.167628642	0.10507
LRP2BP	0.376085273	0.06702	-0.419741712	0.04507	0.140682068	0.49631
NFE2L2	0.189223429	0.14376	0.258692459	0.04506	0.200552462	0.1212
ACTN4	0.105296408	0.394	-0.247607298	0.04512	0.035259959	0.77533
OPA1	-0.204665072	0.05047	0.208233188	0.04514	-0.044329042	0.67082
CXCL14	-0.122580624	0.87343	-1.550875441	0.04523	-0.653780471	0.39643

HABP4	-0.026400852	0.84916	0.271310434	0.04523	-0.097280048	0.48404
CCDC66	-0.256966244	0.07117	-0.281580685	0.04524	-0.332473425	0.01962
RPS6KL1	0.284492809	0.38691	-0.671730399	0.04531	-0.043773799	0.89332
MRAS	0.080951628	0.87384	1.009789914	0.04533	0.33900675	0.50179
ARHGAP42	-0.168115148	0.11497	-0.212268473	0.04545	-0.230088751	0.03083
GSPT1	0.003468707	0.97475	0.218792543	0.04544	0.010878674	0.92088
QPCTL	-0.266931441	0.20683	-0.421164102	0.0456	0.002506562	0.99047
KLC2	0.108128226	0.5279	-0.344700337	0.04569	0.063030182	0.71635
CHRNA10	0.201246387	0.63692	-0.900268489	0.04582	-0.256938169	0.56244
UBA52	-0.043296475	0.66991	0.202414711	0.04583	-0.06135938	0.5457
C22orf46	0.22309061	0.28937	-0.423215277	0.04592	0.035602233	0.86565
EGF	1.180176008	0.0617	1.239386142	0.04592	0.168433002	0.79558
GMDS	0.017700815	0.94436	0.505490293	0.04591	0.413264039	0.10283
EAPP	-0.127334977	0.27476	0.228090599	0.04596	-0.152272792	0.19136
P11-680E1	-0.513243131	0.28359	-0.968715378	0.04596	-0.618315696	0.19768
GDF9	1.589609987	0.07951	1.809952929	0.04599	1.828171793	0.04387
P11-512H2	-0.293814378	0.54141	-1.015054627	0.04609	-0.811894234	0.10773
UBE2E1	-0.082909239	0.42451	0.205789819	0.04615	-0.011660698	0.91043
YEATS2	0.062440018	0.52629	-0.196332034	0.04615	-0.064441205	0.51326
KPNB1	-0.035116595	0.65938	0.158287674	0.04638	0.063533381	0.42477
DHX37	-0.06695132	0.60036	-0.25432498	0.04642	-0.206831484	0.1073
CATSPER2	-0.332390596	0.36458	-0.733143246	0.04651	0.27748751	0.44726
ATSPER2P	0.191223679	0.53233	-0.626436821	0.04646	0.159340279	0.60241
CRIPAK	0.213429566	0.41785	-0.543222909	0.04647	-0.004476655	0.98657
PPP4R1	0.154975203	0.1465	0.211752541	0.0465	0.044939021	0.67375
TTYH1	0.68258875	0.23996	1.144424843	0.04649	0.477027131	0.41587
UTS2B	0.036226168	0.90228	-0.598597378	0.04649	0.030796128	0.91566
MYO18A	0.009607365	0.96109	-0.392583425	0.04652	-0.280066655	0.15502
SRPX	0.215695869	0.58237	0.776215073	0.04661	-0.211389617	0.59139
PHF13	0.006492311	0.96597	-0.303878192	0.04667	-0.278257699	0.06957
C20orf144	0.184597677	0.5719	-0.6861358	0.04671	-0.174118142	0.60209
CCDC126	-0.249738727	0.23302	-0.415574121	0.04672	0.20498265	0.32509
SBDSP1	-0.208212595	0.14661	0.274017363	0.04671	-0.104476359	0.46102
RASL10B	0.194161893	0.51532	-0.596768009	0.0468	-0.466157347	0.12515
KCNH2	0.237873777	0.16991	-0.349967273	0.04694	-0.21237403	0.22749
MICAL1	0.029840576	0.90628	-0.507947752	0.047	-0.212794521	0.40385
P11-248J2	-0.645114469	0.23116	-1.101060615	0.047	-0.957100883	0.08245
MCF2L	-0.447659657	0.06081	-0.471215226	0.04709	-0.226047425	0.34224
MISP	-0.061257347	0.75449	-0.389312328	0.04712	0.026800551	0.89106
P11-10O17	0.048111529	0.77438	-0.335424061	0.04716	0.003811944	0.98188
C14orf80	-0.35270071	0.18778	-0.534618726	0.04718	0.335198557	0.20035
ZNF593	-0.039224775	0.84271	0.381160815	0.04726	-0.12212894	0.53756
NDUFA6	0.140498668	0.40653	0.332595919	0.04727	0.08004257	0.6379
PCDP1	0.520316486	0.07041	0.573352413	0.04733	0.399481471	0.16649
P11-454L1	-0.00801489	0.98675	-1.034412686	0.04732	0.09695635	0.83904
NRIP2	0.073422508	0.78281	-0.541298616	0.04738	-0.124208299	0.64361
ADM	-0.454116641	0.20029	-0.703005551	0.04741	-0.431976109	0.22289

RSL24D1	0.079833712	0.54755	0.261653703	0.04762	0.142681624	0.282
PCDHB2	0.115234098	0.44869	-0.30244477	0.04767	-0.346030728	0.02401
RPL17P19	0.314523313	0.61909	-1.426050461	0.04768	0.035161085	0.95625
DIRAS2	0.759541342	0.26234	1.306349259	0.04771	0.284138857	0.68163
RTEL1	-0.137420874	0.49701	-0.4042467	0.04771	-0.227074739	0.26263
HNRNPUL1	0.135400258	0.05199	-0.138011342	0.04775	-0.006150007	0.92979
APCDD1	-0.008619944	0.98826	-1.164553439	0.04785	-1.139157305	0.05323
MSL3P1	-0.00075264	0.99708	0.395188921	0.04783	-0.26890298	0.19614
PANX1	0.107571893	0.35119	0.226163102	0.04778	-0.026541482	0.81862
RBM45	-0.104408737	0.65059	0.449774167	0.04784	0.246133609	0.28055
ANXA5	-0.189220866	0.11847	0.239321336	0.04798	-0.188705138	0.11941
GUSBP5	-0.396966477	0.27485	-0.733076902	0.04796	-0.189139031	0.59418
SNX33	0.105578773	0.46018	-0.284054351	0.04799	0.010828806	0.93972
C20orf24	0.08515338	0.5164	0.257457138	0.04814	-0.061850221	0.63819
C9orf169	0.24370964	0.59732	-1.053375704	0.04822	0.476251113	0.28952
HSPA8P5	0.343966227	0.48423	0.958310381	0.04823	0.086289522	0.86136
IL12RB1	0.305139408	0.40444	-0.764699253	0.04823	0.069116642	0.85146
OMG	-0.165553487	0.65921	-0.746015151	0.04821	-0.034734892	0.92557
PPP1R8	-0.098500674	0.29842	0.184470203	0.04821	0.092351933	0.32586
RPS15	-0.03344337	0.79112	0.249129534	0.04813	0.014350599	0.90948
UTP20	-0.065433471	0.51574	0.196691824	0.04822	-0.010616143	0.91565
KHSRP	-0.133350896	0.41497	-0.322941354	0.0483	-0.074295672	0.64959
EEF2	-0.188802681	0.29161	0.353286788	0.04844	-0.058848069	0.74237
TEPP	-0.147953403	0.78574	-1.118749274	0.04846	-0.316216887	0.55939
ANKRD12	-0.111787279	0.62203	0.447102846	0.04853	0.206193867	0.36297
FASTKD3	-0.043573096	0.82291	0.375814752	0.0486	0.029531639	0.87844
STIM2	0.191835943	0.10925	0.235294753	0.04862	0.092965448	0.43873
DPM3	-0.384931374	0.05732	0.379321123	0.04872	-0.039190851	0.8427
ARAP1	0.161053683	0.27532	-0.291100175	0.04891	0.311711365	0.03448
PPRC1	0.145871704	0.28961	-0.273238681	0.04882	-0.061522453	0.65588
RRAGA	0.103908051	0.30401	0.197019652	0.04887	0.056146972	0.5786
SMPD3	-0.243147002	0.49455	-0.702797309	0.04889	0.317769324	0.36779
SNAP91	1.035891715	0.12333	1.302641184	0.04889	0.5082751	0.46586
SOWAHC	0.127652857	0.31626	0.249250744	0.04886	-0.02007034	0.87509
ZNF233	0.200727869	0.51482	-0.615917988	0.0489	0.037967851	0.90213
G6PC	-0.156203616	0.77096	-1.150270036	0.04897	-0.672426026	0.23085
APOD	1.618384545	0.08529	1.84662201	0.04905	2.456819619	0.00872
GLTPD1	0.130394813	0.4689	-0.359855593	0.04906	-0.059149696	0.74402
ENPP7P4	-0.497996582	0.27485	-0.922965222	0.04912	-0.275655049	0.53463
PLCB4	0.16897149	0.46468	0.45264115	0.04913	0.42933498	0.0625
ARRDC1	0.009695985	0.94849	-0.29846004	0.04919	0.11306368	0.45072
BRD8	-0.00120172	0.99378	-0.302660063	0.04921	-0.02831705	0.85402
CREG2	-0.016409106	0.94965	-0.508778568	0.04919	-0.08291645	0.74575
DBI	-0.2316631	0.05158	0.2331883	0.0492	0.180708607	0.12784
ZNF26	-0.002181384	0.98695	-0.26321511	0.04919	-0.1857934	0.1648
ZNF664	-0.159499867	0.05957	0.16533091	0.04923	0.062500939	0.45851
WWC1	0.35107013	0.05937	0.365754894	0.04935	0.279293862	0.13389

SDHAP3	0.068239394	0.78816	-0.512875167	0.04937	0.204124378	0.41656
RPP21	0.135204744	0.43306	0.333685387	0.04944	0.16930619	0.32407
TRPV3	-0.186538874	0.52269	-0.573157735	0.04943	0.445106496	0.11713
ZNF519	-0.369291373	0.1845	-0.545877432	0.04944	-0.267398369	0.33602
MRPS17P1	-0.277717555	0.47572	-0.781941594	0.04948	0.238380751	0.52812
C10orf11	-0.329600047	0.51022	-0.993586366	0.04954	-0.328186017	0.51351
SLC25A3	-0.122970082	0.27469	0.220655163	0.0496	-0.00784494	0.94442
TGFB2	1.336325584	0.08611	1.528203024	0.0496	0.653537735	0.40194
MUTYH	-0.142851436	0.26505	-0.248901028	0.0497	-0.098365725	0.43869
IGFBP4	0.450502403	0.12348	0.572562049	0.04976	0.092167819	0.75329
ROCK1P1	0.561798236	0.36689	1.211142418	0.04977	0.60516369	0.3298
SETD1A	0.428005221	0.08717	-0.497075232	0.04973	0.189257778	0.45078
FMR1	0.195387344	0.09474	0.227950141	0.04978	-0.103546758	0.37711
LRRC16A	0.108607437	0.43316	0.271187935	0.04983	0.296192953	0.03228
ZNF143	0.112848945	0.39062	0.255328942	0.04997	0.115942973	0.37742
AC005176.2	-0.282212098	0.58173	-1.584442359	0.01089	0.00527449	0.99143
AC006077.4	-1.013596845	0.33586	-3.622387899	0.00743	-1.395169259	0.2013
AC007875.2	-0.257977093	0.70575	-1.730741873	0.02834	-0.127347031	0.85016
AC010468.1	1.070233327	0.32065	2.227938682	0.02961	0.1305768	0.90948
AC018442.1	0.233825612	0.79229	-2.837779227	0.02481	-0.426277786	0.65495
AC018755.1	-1.084441321	0.36231	-2.90154548	0.03599	-1.415401073	0.26004
AC019100.7	-1.33834493	0.06479	-1.796064577	0.01582	-1.199270207	0.09271
AC067940.1	-0.691280124	0.55768	-2.945644172	0.0384	-0.796374439	0.50199
AC068657.2	0.058832945	0.96787	-4.046425211	0.0192	-1.211157872	0.43266
AC069368.3	1.862905242	0.2372	3.566304166	0.01968	2.782932283	0.07212
AC073150.6	-0.656515679	0.41784	-1.99697746	0.02774	-0.390484848	0.62281
AC074338.4	-0.095684376	0.92248	-2.974199242	0.0243	-0.490594157	0.63024
AC090286.2	-0.578805729	0.44939	-2.947084702	0.01122	-0.225283372	0.75712
AC104451.2	-1.644624845	0.0623	-2.122394413	0.02328	-0.010679109	0.9879
AC106827.1	-1.039430333	0.28038	-2.563528037	0.028	0.109913576	0.90022
AC129778.2	0.735844332	0.56681	-3.094449078	0.0487	-2.239310544	0.15374
AC133965.1	-1.255292828	0.39166	-3.977821026	0.0155	-2.395860496	0.13854
AC139426.2	-0.609088564	0.49506	-2.243974329	0.02995	0.325945916	0.70078
ADAD2	0.447949392	0.5749	-2.456760389	0.01769	-0.521339661	0.53314
AKAP4	1.684506626	0.47648	4.354330602	0.04755	0.006174427	0.99793
ATG12P2	0.116795091	0.85774	-1.542301392	0.04538	0.826977459	0.18304
BLACE	-0.208952523	0.83304	-2.772109558	0.03724	-1.298495751	0.23411
BRI3P1	-0.066007777	0.95889	-3.469964326	0.02439	-3.336172179	0.03045
BTF3L4P1	-0.336922539	0.61799	-1.870091693	0.01423	-0.623945172	0.36331
C14orf164	0.062977995	0.93758	-1.963630775	0.03058	0.755042426	0.32749
C18orf25	0.492264257	0.73562	-3.456343069	0.04429	0.307043174	0.83364
C1orf146	1.023097017	0.27026	-2.86397884	0.03139	0.067780398	0.94537
C2orf40	1.728250357	0.18448	2.632703432	0.03191	2.47448137	0.04701
C2orf80	0.14127617	0.82818	-1.607490923	0.04149	0.04784283	0.94166
C4orf26	-0.421245102	0.7162	-2.972567098	0.04373	-0.726645699	0.57108
CACNA1B	1.219395722	0.06412	1.541587833	0.02117	0.411870782	0.54406
CALML6	0.07163158	0.94045	-2.61972896	0.04186	-0.133305867	0.89106

CBFA2T3	1.41199733	0.2048	2.39106138	0.02564	1.373518553	0.21692
CCDC154	-0.937346949	0.35045	-3.046544602	0.01649	-0.875422485	0.37368
CER1	0.744897681	0.46326	2.207073412	0.01685	-1.30077406	0.3229
CFL1P3	-0.036537465	0.96128	-1.881521706	0.04131	-0.665965614	0.40201
CHRNA1	-0.313589135	0.71377	1.591199816	0.02425	0.192684068	0.80642
CLEC2A	-0.587848252	0.27428	-1.622389453	0.00482	-0.903449852	0.09861
COL4A3	0.152352421	0.87251	-2.257154428	0.02558	-0.914111831	0.3567
:TB-175P5.	-0.574658922	0.31682	-1.567974187	0.01378	-1.01846476	0.09013
TC-273B12	0.766894757	0.49689	-4.315968934	0.00376	-0.544550036	0.64617
:TC-398G3.	-0.148447268	0.80581	-1.79639751	0.01101	-0.695729421	0.26904
TD-2017C7	-0.33751345	0.68506	-2.267461863	0.03721	0.000424619	0.99958
TD-2036J7	-1.607597254	0.12353	-2.789651698	0.02572	-2.113804443	0.06188
TD-2561J2	-1.514535816	0.21743	-2.944628854	0.04023	-0.223371076	0.84155
D-2562G1	0.350293644	0.69647	-3.287368107	0.01146	-0.776138681	0.43281
CYCSP34	-0.699353126	0.36492	-2.701575442	0.00964	-1.030914667	0.19691
DNER	1.443540301	0.08011	1.724265919	0.03626	1.879942028	0.02303
DRC1	-0.542244926	0.54851	1.736807642	0.03291	-0.344982242	0.69662
DUSP26	-0.053708152	0.92405	-1.29423476	0.0473	-0.667407918	0.26721
DUTP1	-0.746752087	0.34038	-1.93475903	0.02963	-1.735761714	0.04863
DUTP2	-0.359532821	0.6549	-2.608075503	0.0161	-0.199587728	0.80069
DUXA	-0.510768428	0.49737	-2.233245943	0.02698	0.558914585	0.39943
DYDC1	0.328242595	0.54545	-1.333681091	0.03466	-0.20643314	0.71434
EEF1A1P3	1.615544891	0.0661	2.168332238	0.01038	0.986795292	0.28277
ELAVL3	1.441271622	0.2026	2.76271638	0.01486	1.543198411	0.18104
ENPP7P11	-0.433536595	0.48985	-2.594370966	0.00351	-0.386470928	0.53384
ERICH2	-0.781962892	0.29936	1.253250292	0.03819	0.135713949	0.83834
FCRL6	3.007932754	0.09026	3.678104164	0.03534	3.368415815	0.05171
FLT3	-1.323264234	0.35096	-3.957626742	0.01337	-2.774297838	0.07762
GAPDHP71	1.275022252	0.42903	2.886849799	0.04534	0.728358192	0.66471
GCNT7	0.292733911	0.67413	-1.767504842	0.03057	0.552262106	0.42121
GNLY	-0.675477442	0.31198	-1.678586042	0.02439	0.598903354	0.29922
GRAMD2	1.158396379	0.13493	1.606623148	0.03677	1.058657366	0.1825
GRM8	-1.128249848	0.13657	-1.614153574	0.04536	-0.893370628	0.23251
HIGD1AP1	-2.707515078	0.05052	-2.887929619	0.037	0.549905044	0.59164
HMGB1P2	-0.425375022	0.6817	-3.609911946	0.00658	-1.473200799	0.21717
HMGB3P3	-1.74829642	0.16017	-2.637473801	0.03823	-0.189840728	0.84327
HMGB3P4	0.099804859	0.87524	-1.840073012	0.02195	0.213243675	0.73392
HMGN1P2	-0.311037133	0.63894	-1.63599688	0.03478	-0.024658766	0.96936
NRNPA1P	0.958952424	0.50247	2.570299517	0.04563	-0.71557928	0.65303
HSD3B1	1.761051623	0.07158	2.025052894	0.03512	0.845628902	0.40814
HSPA8P15	0.734817883	0.21695	-2.272918631	0.01082	-1.105352702	0.12965
IL4I1	0.995254063	0.30731	1.848006433	0.04674	1.927162325	0.05003
INGX	-0.051486471	0.9376	-1.847346426	0.01637	-0.224162475	0.73528
JRK	-0.328699472	0.72004	-2.415501678	0.02338	0.69749215	0.42787
KCNC2	0.566483302	0.52209	-2.905370885	0.00412	0.764730245	0.38582
KCP	0.448596472	0.55779	-2.010835596	0.04517	0.604109306	0.42422
KLRG2	-0.761980232	0.60192	-3.357552011	0.0384	0.023427668	0.98654

KRT18P39	0.460360519	0.61321	1.699359883	0.04089	-0.806657427	0.44645
KRTAP5-11	-1.022507864	0.21173	-2.131080969	0.02581	-0.552102634	0.4727
KRTAP9-6	-0.038631237	0.95393	-1.616339483	0.03357	-0.370464522	0.58681
OC1004211	1.475222272	0.40636	3.638269108	0.01859	2.392327567	0.14705
LSMEM2	-0.248799606	0.65794	-1.886843421	0.00559	-0.61066172	0.29241
MAG	-1.526352647	0.20882	-2.856182617	0.03446	0.936081122	0.3728
MARK2P8	0.234609658	0.7307	-1.877568283	0.02323	0.388934516	0.56395
MKRN3	0.446559561	0.51886	-1.569153414	0.03482	-0.022800046	0.97381
MKRN9P	0.159007683	0.75702	-1.503380119	0.01671	-0.174722765	0.74218
MOG	-0.491989651	0.48276	-1.676325423	0.02422	-0.335464064	0.63667
MORF4L1P	0.774142923	0.69125	3.734296243	0.0298	2.720493017	0.12781
MRPS6P2	-0.777718681	0.40499	-3.970957367	0.0015	-0.278173977	0.75019
MTND4P15	-1.144236751	0.06704	-1.703900661	0.00903	-1.365411126	0.03202
MYL1	2.76062446	0.3087	5.454557011	0.04091	2.743648657	0.31101
MYOG	0.52577116	0.76632	3.663670302	0.02473	2.092996167	0.2094
MYOZ2	0.604567993	0.38628	1.390730337	0.03261	1.120882256	0.09294
NFE2L3P1	0.313879527	0.5782	-1.303276626	0.04589	-0.180781943	0.75708
NHLH1	2.009693003	0.17833	2.830066547	0.03854	3.17041601	0.01884
OPCML	2.934405817	0.11017	3.881885908	0.0372	2.093400523	0.26336
OR2D2	-0.477976066	0.44335	-1.397381274	0.04213	-0.533056889	0.39312
OR7A17	-0.876828761	0.14477	-1.438805835	0.02476	-0.253375385	0.64507
PAX4	2.431141703	0.06125	2.829708998	0.02679	1.95948962	0.14115
PAX8	0.897736168	0.26047	2.148859793	0.00383	0.821898374	0.2979
PCDH8	-0.889663275	0.41589	-2.635150148	0.04085	-0.860380417	0.4268
PCDHB8	0.562886146	0.51448	-3.346474035	0.00932	-1.935585261	0.09183
PENK	2.089167952	0.13729	3.246317429	0.01865	0.469699218	0.75032
PPIAP2	-0.960472022	0.30751	-2.76860124	0.02583	-1.355894667	0.17039
PRELID1P3	-0.575645428	0.453	-3.186422837	0.00679	-0.731038321	0.34669
RNF224	-0.699354362	0.13984	-1.475229084	0.00466	-0.568240124	0.21919
RP1-128O3.	-1.216775229	0.28975	-2.692194691	0.04999	-1.146559245	0.31227
P1-131F15	1.268897773	0.13492	1.693707323	0.03849	-0.291531397	0.76999
P1-214M20	0.895014821	0.36336	-2.829070819	0.03811	-0.632735653	0.5688
P1-40G4P.	1.78732263	0.22604	2.999448842	0.02798	-0.715172107	0.6638
RP1-4G17.5	1.030899184	0.23608	-3.986128085	0.00222	1.76210213	0.03725
11-101P17	-2.27804728	0.13668	-3.179808214	0.03776	1.013889662	0.4035
P11-137J7	1.012416175	0.13824	1.888932984	0.00297	0.023394032	0.97542
P11-15E18	0.066337182	0.92938	-3.286721542	0.00605	-1.355486048	0.11556
P11-160A9	-0.921054666	0.45665	-2.897446357	0.04447	0.264596983	0.81287
P11-178C3	0.802587384	0.23238	1.426722647	0.02549	0.863045336	0.19544
P11-178L8	-1.380353352	0.35839	-5.22123614	0.0029	-1.659979638	0.27348
P11-182I10	0.044419007	0.94683	-2.125951867	0.01959	-0.175100052	0.7963
P11-18M17	-1.871991478	0.16256	-3.272706602	0.02407	-1.461913291	0.24773
RP11-1G11.	0.871734725	0.27771	-2.16962143	0.03857	0.273873414	0.73958
P11-214O1	-1.346673441	0.14293	-1.969661246	0.04166	-0.585459001	0.4955
P11-215A2	1.28801817	0.32922	3.52865689	0.00276	3.240128383	0.0065
P11-236F9	-1.324324846	0.09826	-1.726842456	0.03855	-2.24169023	0.01166
P11-23J18	-1.03369034	0.44081	-3.226572855	0.03703	-0.664621746	0.60686

P11-241J12	-0.508663149	0.64118	-2.619890257	0.04986	-0.503258592	0.64259
P11-253M7	0.429017326	0.56231	-2.318138407	0.02964	-0.927179423	0.27592
P11-255L13	-0.964164498	0.4584	-2.952045527	0.04864	-2.105720085	0.15201
P11-274E7	0.642884932	0.67955	3.345506565	0.01288	1.026550557	0.49285
P11-275F13	-0.840019228	0.29756	-2.703724421	0.0107	-0.528705337	0.49621
P11-293A21	0.206824981	0.82906	-3.01971825	0.02246	-0.611312448	0.55329
P11-299L17	1.293283626	0.41615	2.901141953	0.04114	2.0085863	0.17913
P11-315D13	-0.001765525	0.99767	-1.351358964	0.04936	-1.376846966	0.05025
P11-329J18	0.306099493	0.70884	-4.294554453	0.00077	-0.780792802	0.36933
P11-344N10	0.476628216	0.53869	-2.00378741	0.04648	0.481225149	0.53352
P11-351O1	-0.548781362	0.53683	-2.326150848	0.03733	-1.486422674	0.13367
P11-363E7	-2.507575283	0.12312	-3.375123486	0.03797	-1.384123699	0.3646
P11-378J18	0.235127469	0.79209	-3.001005966	0.01876	-1.110283126	0.28841
P11-382N13	-0.251467214	0.818	2.034627331	0.02653	-0.012205011	0.99079
P11-384B12	-0.622433337	0.49332	-2.51358688	0.02086	-0.6133488	0.49807
RP11-3L10.	-1.022253997	0.06508	-1.245713791	0.02514	-1.547769442	0.00782
P11-420K14	-0.476329405	0.315	-1.370784547	0.00966	-0.556092418	0.25346
P11-445J14	-1.185983027	0.19783	-2.27355353	0.02616	-1.674384072	0.08229
P11-445P17	0.804136088	0.6265	3.662868673	0.00795	0.727548707	0.6601
P11-453E17	0.278604206	0.71909	-2.409296767	0.02516	-0.217675775	0.78613
P11-466F5	-0.557861876	0.59703	1.645055613	0.04764	0.379435176	0.68156
P11-476C8	-0.325183271	0.64716	-1.847678338	0.03409	-0.325717378	0.64496
P11-479I16	-0.182118651	0.84897	-2.851352457	0.02648	0.160973715	0.86084
RP11-51L5.	-0.729710642	0.45838	-3.295914735	0.00575	-0.54663305	0.57538
P11-532F6	0.829072524	0.20282	-2.226040112	0.01766	0.388062554	0.56139
P11-535K18	-1.257345873	0.12533	-1.931305902	0.0302	-0.87284937	0.25887
P11-537E18	-0.180304007	0.72802	-1.312527918	0.02742	0.092873497	0.8529
P11-537I16	-0.840432811	0.33763	-2.388861784	0.02857	-0.364419211	0.66032
P11-549L6	-1.590623854	0.06104	-2.122821192	0.01662	-1.836741978	0.0346
P11-556K13	-0.866753262	0.33772	1.558576827	0.03227	0.70083772	0.36003
P11-661D19	0.399536845	0.596	-2.34599909	0.02825	-0.962353303	0.2638
P11-677M14	-1.150706172	0.12814	-1.619241197	0.03961	-0.141188094	0.83559
RP11-6J24.	-0.50722305	0.35131	-1.18026191	0.04197	0.259783444	0.60594
P11-709A23	-0.547986637	0.39751	-1.403372934	0.04647	-0.258793946	0.67967
P11-711M9	-0.888105248	0.24108	-1.669102809	0.04542	0.183546258	0.78392
P11-744O11	-0.431100086	0.39971	-1.944119389	0.00177	-1.285100966	0.02381
P11-889L3	-1.492659308	0.22321	-2.930239745	0.03031	0.000851775	0.99933
P11-894J14	-1.515600627	0.14573	-2.858272774	0.01431	-1.895597274	0.07534
P11-925D8	-0.221639807	0.87153	-3.144020168	0.04656	-1.009812457	0.49208
P11-956A19	-0.690793426	0.36241	-1.857128624	0.03219	-1.936145322	0.03112
RP3-355L5.	-0.094673548	0.89387	-1.924476443	0.03042	0.216415326	0.75333
P3-405J10	0.314278567	0.59779	-1.531057923	0.0285	-0.134728598	0.82579
RP3-522J7.	-0.595476786	0.28389	-1.614802738	0.01079	-0.727106659	0.19581
RP4-633I8.	0.290587439	0.62901	-1.821819072	0.01432	-0.221010632	0.72261
P5-827A12	-1.390938716	0.35898	-3.044132772	0.04892	-1.503288325	0.32347
RPEP4	-0.776617776	0.30601	-2.695107863	0.00861	-0.87437428	0.25219
RPL10AP2	-0.378981388	0.6105	1.256748781	0.04917	0.283118617	0.68124

RPL12P44	0.744779526	0.39019	-3.430112084	0.0082	-0.002574413	0.99773
RPL13AP25	1.0036016	0.53857	3.526105628	0.01719	2.298610097	0.13176
RPL13P6	-0.016712102	0.98892	-3.141214951	0.03106	0.648365429	0.57059
RPL21P23	-0.129987292	0.88701	-3.048899755	0.01665	-0.69557575	0.4748
RPL21P65	-0.179477112	0.79635	-1.923428638	0.02771	-0.919241317	0.22401
RPL23AP50	-0.704984438	0.33425	-1.622526901	0.04808	-0.461848663	0.51166
RPL23AP6	1.843249117	0.14201	2.749736593	0.02089	1.307842275	0.3154
RPL24P7	-0.458623117	0.45669	1.050714951	0.04888	-0.967595286	0.14085
RPL5P9	-1.931406095	0.05918	1.706574768	0.00725	-0.758387565	0.34816
RPL7P48	1.45004086	0.30164	2.64215644	0.04455	1.892070893	0.16516
RPL7P49	0.131545281	0.86439	-1.925736863	0.04973	0.748358043	0.30946
RPL7P50	0.724407963	0.6613	2.904514183	0.03793	2.377537229	0.09922
RPL7P8	0.052796797	0.97516	3.29150258	0.02314	2.446020687	0.11102
RPS15AP12	-0.495893354	0.61081	-3.312798697	0.01141	0.869817001	0.32413
RWDD4P1	0.511628099	0.61779	-2.776312508	0.0454	-0.697493779	0.52472
SETP22	1.509329375	0.40181	3.148101381	0.04978	2.804739582	0.08724
SETP5	-0.756668377	0.3943	-3.082394901	0.01322	-0.170122218	0.83947
SFTPD	-0.530087939	0.62632	-2.86653997	0.03541	-0.560481843	0.59606
SLC10A6	2.402369436	0.16397	3.443430232	0.03616	2.579286406	0.14035
SLC34A3	0.496829384	0.38558	-1.631567495	0.0335	0.464024631	0.41702
SLC35G6	-0.188015421	0.69848	-1.443090018	0.01076	0.317634139	0.48973
SLCO1C1	0.803899759	0.40196	2.157430512	0.01852	2.336197596	0.0117
SLCO6A1	-0.464605853	0.54072	-2.312243641	0.00843	-0.828621454	0.26575
SNTG2	0.448633929	0.74004	2.559695103	0.03758	-1.010728047	0.51116
SNX25P1	-0.227582572	0.76062	-2.003375927	0.02844	-0.020115449	0.97808
SNX6P1	0.292291588	0.59121	-1.421651649	0.0314	-0.11632959	0.83637
SPACA3	-0.371208289	0.47992	-3.07669869	0.00026	-0.804681635	0.14441
SPATA2P1	-0.613731977	0.28428	-1.241725015	0.04275	0.01056679	0.9841
SRMP2	-0.088326468	0.90107	-1.783217181	0.04258	0.242514098	0.72404
ST13P5	1.072262167	0.39561	2.39927803	0.04705	0.438451189	0.73634
SYT3	-0.539276178	0.58666	-4.063995432	0.0015	-0.230042365	0.79216
TBX1	1.972254781	0.06774	2.590114262	0.01901	1.714355131	0.11928
TCEAL5	-0.668548985	0.76432	4.456738854	0.02593	2.345338416	0.2566
TFAP2B	-1.609351003	0.31691	-3.245003358	0.04623	-1.825815732	0.2628
TH	1.270482801	0.18382	-2.94319313	0.02952	-0.269568043	0.79542
TMED10P1	0.990759161	0.61423	-4.660795779	0.03412	-0.021618939	0.99129
TMEM121	-1.221883527	0.25305	-3.266307095	0.01488	-1.302898751	0.21254
TMEM178B	2.130356295	0.07413	3.283701594	0.00589	0.530756127	0.66444
TMEM31	0.632347029	0.38335	1.383956784	0.04268	0.880238185	0.21458
TMEM61	2.071071514	0.08133	2.724596947	0.0183	1.100303612	0.37995
TNNI2	-0.048687669	0.94515	1.37392741	0.02803	0.815388767	0.21068
TSEN15P2	2.040241534	0.06852	2.180032372	0.04844	2.230079202	0.04423
TSPAN32	-0.54553761	0.52334	-1.763166075	0.04555	-0.368929891	0.64463
TSPYL6	-0.850337928	0.2434	-1.729922039	0.02939	-0.803910935	0.26562
UBE2L4	-0.531828841	0.37354	-2.919477538	0.00016	-0.917267	0.13402
UBQLN3	0.128031618	0.91104	-3.686561648	0.01033	-0.774834586	0.5001
WFIKKN1	-0.463300601	0.45772	-1.456182789	0.02763	0.481426691	0.39977

WFIKKN2	1.376294176	0.41933	3.335233389	0.02422	3.110552787	0.03717
WNT7B	-0.71841751	0.35003	-2.20327682	0.01003	-0.606749488	0.43204
XX-CR54.3	-0.123253863	0.90972	-2.821636726	0.04185	-1.361373667	0.27608
SLC11A2	0.335662725	0.07765	0.148693944	0.43419	0.860861974	5.88E-06
DNAH14	-0.202184479	0.06882	-0.026109999	0.81166	-0.499219438	8.35E-06
ARFGEF2	0.122945479	0.13372	0.066151025	0.41848	0.353406341	1.50E-05
CLINT1	0.184036582	0.13002	0.229470077	0.05885	0.508095966	2.87E-05
AC018804.7	-0.438204254	0.19686	-0.151929767	0.64227	-1.564233547	4.33E-05
GALNT4	0.015132007	0.92771	0.285482941	0.08638	0.671346368	5.44E-05
SOX4	0.110926626	0.44736	-0.140619442	0.33556	-0.56226869	0.00012
STS	0.291918738	0.08289	0.091919214	0.58506	0.641026855	0.00013
hsa-mir-672	0.441696075	0.13783	0.450420213	0.128	1.076554096	0.00023
C19orf54	0.537202586	0.09396	0.002285112	0.99442	1.170714641	0.00024
C6orf106	0.137161642	0.15166	0.034781659	0.71518	0.347264125	0.00027
TDRD7	0.188436181	0.22922	0.269665857	0.08332	0.554026124	0.00036
BCL2L2	0.068287979	0.58803	-0.156546126	0.2153	0.44415644	0.00039
DAZAP2	0.184748676	0.12954	0.006887742	0.95487	0.429112549	0.00042
LEPREL4	-0.261226435	0.12782	-0.109057758	0.52296	-0.601510004	0.00047
SNURF	-0.001520092	0.99197	0.062650295	0.67592	-0.535241882	0.00051
TM9SF2	-0.036928906	0.71304	0.179939336	0.07255	0.344212986	0.0006
GCNT4	0.160478066	0.46333	0.280127798	0.19897	0.746263713	0.0006
CP	0.197248342	0.38948	0.38492253	0.09303	0.78261862	0.00064
INHA	-0.31833336	0.2624	-0.522541302	0.0653	-0.975166479	0.00068
TTC28	-0.304303954	0.30025	-0.188320677	0.52639	-1.00115377	0.00069
CYSLTR2	-0.70188399	0.33814	-0.958725713	0.19146	-2.715499028	0.0007
PRRG4	0.182260217	0.22995	0.166489409	0.26925	0.502203627	0.00078
SEC22C	-0.146799929	0.18879	-0.141955519	0.20041	-0.374582992	0.00079
TRIM25	0.017856347	0.89875	-0.226931103	0.10442	0.462481487	0.00093
LAMB1	-0.157691121	0.32554	-0.039888875	0.80353	-0.530936452	0.00094
MFGE8	-0.029710218	0.82046	-0.201453771	0.12281	-0.434049633	0.00097
SPTLC3	-0.35972856	0.17491	-0.391476663	0.1381	-0.87680876	0.00096
TSLP	-0.837191069	0.42211	-1.239396708	0.23377	-3.671184762	0.00097
TMEM53	0.13819882	0.42634	0.085572642	0.62139	0.564605682	0.00102
PKD2	-0.134813299	0.42953	0.058148628	0.73253	-0.562041296	0.00106
BEND5	0.187304654	0.52841	0.198939752	0.50023	-0.986749461	0.00112
GALNT10	0.262759686	0.1595	0.031841504	0.86445	0.605800105	0.00115
PNPLA3	0.26022353	0.21644	-0.061047668	0.77283	0.680959055	0.00117
IDH2	-0.02026307	0.87094	0.048508916	0.69641	-0.406267691	0.00119
MSH5	0.018001926	0.95705	-0.429836642	0.20017	1.086693679	0.0012
CARD6	0.129961696	0.32576	0.009323883	0.94358	0.419897297	0.00131
OXA1L	0.194873439	0.06832	0.003504346	0.97385	0.341755791	0.00134
CYP2E1	0.674524143	0.13037	0.642364728	0.15283	1.415203528	0.00141
KLK6	-0.137454498	0.81454	0.069253753	0.90521	-1.951981102	0.00145
TAF2	-0.198364685	0.05807	0.190374596	0.06635	-0.330242171	0.00157
SLC44A4	0.209987346	0.16385	-0.210362528	0.16327	0.473244695	0.00169
URB1	-0.25550354	0.06457	-0.166185296	0.22788	-0.434159802	0.00169
UBE4A	0.11201489	0.19051	0.066293531	0.43747	0.267553471	0.00173

FAM69B	0.018909494	0.90587	-0.133748901	0.40264	-0.511508043	0.00175
RANBP17	-0.313108655	0.1107	0.115397353	0.55366	-0.616200262	0.00185
CADM4	-0.054928443	0.78379	0.041122661	0.83534	-0.624131279	0.00192
CENPL	-0.317130603	0.07237	-0.138295465	0.42972	-0.549478446	0.00194
ACSS2	0.373820712	0.07288	-0.078357287	0.70708	0.644013046	0.00198
SHC3	-0.406868022	0.2171	-0.383398556	0.24731	-1.036669275	0.00208
WDR35	-0.172116177	0.17994	0.238710505	0.05674	-0.39848481	0.00208
ATRN	-0.030046836	0.73351	-0.029755233	0.73551	0.270239944	0.00212
XXYLT1	-0.105959589	0.42704	0.082736841	0.53024	-0.413548834	0.00212
PPP6R2	0.120552327	0.35317	-0.173342009	0.18145	0.394296137	0.00225
TECRL	0.550437171	0.2634	-0.247173194	0.6229	1.47747818	0.00225
TD-2328D6	0.266750847	0.61107	0.406069052	0.43837	1.593967097	0.0023
GALNT18	-0.419390582	0.05492	-0.204921492	0.33684	-0.675883177	0.0023
TUBB3	0.445108163	0.09651	0.201080811	0.45336	-0.823215754	0.00245
ZNF587	-0.069475011	0.52434	-0.024939086	0.8188	-0.330602241	0.00245
CDK12	0.066887631	0.58227	-0.027716264	0.81971	0.36761918	0.00248
SFRP1	-0.109118212	0.85033	0.209361398	0.71726	-1.750329503	0.00251
VCAN	-0.35289171	0.12548	-0.392088033	0.08867	-0.695905588	0.00252
BMP7	-0.563452767	0.08902	-0.105768481	0.7487	-1.004692536	0.00256
FAM175A	-0.147597253	0.14948	-0.00896703	0.9289	-0.309508839	0.00257
FKBP7	-0.13359046	0.48284	0.061991014	0.74407	-0.577148372	0.00263
DRG2	-0.195520299	0.0948	-0.079584702	0.49431	-0.351678162	0.0027
SLC29A3	0.354945043	0.25611	0.215722898	0.49003	0.925184054	0.00271
BEX1	-0.508223163	0.30717	0.323150089	0.50776	-1.544127455	0.00277
WDR55	0.047929491	0.45532	-0.007734231	0.90319	0.189587484	0.0028
STAT5B	-0.22768219	0.06779	-0.241210966	0.05209	-0.372255573	0.00282
TPD52	-0.232132459	0.11708	-0.150082699	0.30976	0.440896207	0.00283
PITX1	-0.04046454	0.93195	-0.566729209	0.24126	1.36747539	0.00288
ZNF184	-0.191261167	0.09487	-0.041471786	0.71229	-0.342701811	0.00292
YAP1	0.158032066	0.05684	0.110311445	0.18264	-0.24747293	0.00296
CASZ1	0.512429049	0.08764	-0.150726835	0.61912	0.88836711	0.00303
VN1R2	0.556856187	0.14426	-0.107123727	0.78866	1.081996604	0.0031
P11-39K24	1.067190116	0.07276	0.953723066	0.10719	1.685748297	0.0032
TESK2	0.126420715	0.49932	0.009838554	0.95677	0.534448495	0.00323
KBTBD8	-0.242238569	0.41664	0.093895841	0.74488	-0.894804806	0.00324
FREM1	0.422135479	0.20487	0.025305921	0.93944	-0.981371152	0.00328
METTL24	-0.055918248	0.86898	-0.147667912	0.66127	-1.070551448	0.00336
GOSR2	0.009385276	0.92246	0.008768483	0.92681	-0.28355922	0.00348
RAB3IP	-0.035330646	0.82678	0.298020845	0.06335	0.468708088	0.00349
ELMOD2	-0.213506562	0.06778	0.134907418	0.24334	-0.340203809	0.00353
HOXA13	-1.155377272	0.22736	-1.284124895	0.17948	-2.928330901	0.00355
KCTD3	0.099877486	0.44674	0.207450911	0.11288	0.381302422	0.00358
THSD7A	-0.223279358	0.72532	-0.289721365	0.64831	-1.988763078	0.00361
FADS1	0.204897331	0.1607	0.036272993	0.80377	0.423032109	0.00375
ABCA6	0.676589751	0.05773	0.596339941	0.09689	1.022754683	0.0038
GNPTAB	-0.145685326	0.14926	0.000983701	0.99219	-0.291801247	0.00385
TOB1	0.250301554	0.2378	0.286664561	0.17581	0.610029588	0.00393

FKBP10	-0.109333747	0.50994	0.025333876	0.87855	-0.477616412	0.00403
SPAST	-0.220550837	0.05676	-0.116040366	0.31173	-0.333585254	0.00404
INF2	0.281663221	0.06025	-0.240760257	0.11207	0.430305268	0.00414
MBOAT1	-0.04387728	0.78598	-0.02765893	0.86358	0.457789117	0.00425
ARRB1	0.105633134	0.46637	0.083353769	0.56487	0.412680214	0.00431
HAPLN1	0.340854166	0.69587	0.362318008	0.67703	-2.562448001	0.00437
GGA3	0.072257767	0.67258	-0.169412732	0.32637	0.482682948	0.00439
SLC43A3	0.14812971	0.5038	0.242054056	0.27368	-0.639419394	0.0044
AP1M1	0.107596123	0.40544	-0.186571978	0.15108	-0.369624025	0.0045
CALCOCO2	-0.00852118	0.93227	0.040031775	0.68863	0.283363984	0.00452
F11	0.981929041	0.10402	0.286009625	0.64024	1.69722415	0.00459
MCTP2	-0.269832234	0.25269	0.00279984	0.99048	-0.669665739	0.00468
ELF4	0.374512063	0.29165	0.102209304	0.77368	0.99735723	0.00475
SNAP47	-0.067456469	0.77559	-0.013098373	0.95554	-0.685544047	0.00475
MPZL2	0.166204664	0.26316	0.152974337	0.30257	0.418578271	0.00477
ASXL3	0.018175459	0.95017	-0.058803028	0.84041	-0.823967003	0.00486
PRR13	0.251223557	0.07971	0.225838117	0.11477	0.403229225	0.00485
WRAP53	-0.280457025	0.06213	-0.259862217	0.0807	-0.42809543	0.00489
NMB	0.464483623	0.05129	0.461702524	0.05119	0.664173467	0.00495
GID4	0.295007274	0.20866	0.274623809	0.23926	0.656847157	0.00496
NUDT16	0.196271188	0.06679	0.173850389	0.10223	0.299383296	0.00497
GRAMD4	-0.212797657	0.33226	-0.090817446	0.67865	-0.616232499	0.00499
FLNA	0.248006983	0.17268	-0.056567911	0.75581	-0.510098225	0.00505
MGAM	0.40745184	0.41415	0.374594648	0.45243	1.394632462	0.0051
DDX11L2	0.561471214	0.14471	0.407739349	0.28895	1.059103928	0.00515
MYO1A	0.614732569	0.11573	0.211444542	0.58841	1.087472939	0.00531
CEACAM5	0.634028603	0.20344	0.409573506	0.41132	1.386378727	0.00542
PNKD	-0.009002484	0.93226	-0.200861294	0.05894	0.292325845	0.00552
GSTP1	0.030576002	0.75673	0.065736934	0.50489	0.273520437	0.00554
SOCS4	-0.059059429	0.52933	0.092667786	0.31733	-0.259174576	0.00575
TRMT44	0.286360682	0.05177	0.089424916	0.54179	0.401308672	0.00574
C1orf52	-0.157200403	0.24711	-0.114950246	0.39363	-0.376571679	0.00583
GPX7	-0.262469108	0.24047	0.09518945	0.66715	-0.621129155	0.00583
MSX2	-0.15828632	0.60415	-0.464246574	0.12633	-0.886062935	0.0059
SKA1	-0.418906772	0.31896	-0.090132457	0.82939	-1.161427746	0.00602
IGF2R	0.159883406	0.32872	-0.278753609	0.08875	-0.449618141	0.00606
CTSL	-0.068077814	0.72761	0.083386968	0.6681	-0.539707044	0.0061
DNAJB1	0.116588895	0.236	0.105410299	0.28265	0.268543292	0.00621
SERPINH1	-0.014161199	0.91783	-0.184705773	0.17842	-0.375907237	0.00622
HDAC4	0.389581444	0.10296	0.168103323	0.48272	0.653919318	0.00626
IGFBP7	-0.225006771	0.2707	0.290279859	0.15462	-0.558354325	0.00633
ISOC1	0.008452694	0.93052	0.122587839	0.20198	0.262178492	0.00636
MT-ND3	0.378672392	0.08194	0.415976208	0.05594	0.593711483	0.00637
DFNB31	-0.064850359	0.81975	-0.390587488	0.17669	-0.802379609	0.00649
MGST3	-0.143693895	0.17493	0.065199915	0.53548	0.285365584	0.00661
CYB5D1	-0.283450455	0.06821	0.109979468	0.4704	-0.421183876	0.00668
SLC7A8	0.690540869	0.14485	-0.069856974	0.88382	1.280614304	0.00666

ADAMTS6	0.052120501	0.84862	0.403220179	0.13806	-0.75263817	0.00677
DSG3	0.855649411	0.31274	0.516952323	0.54295	2.259667124	0.00679
SHOC2	0.069975408	0.61565	0.144696712	0.2969	0.375365895	0.00692
?11-134E1!	-1.773108245	0.19464	0.980106659	0.46395	-3.983290331	0.00694
ZCCHC2	0.248184804	0.24367	-0.043609179	0.83775	0.573298723	0.00693
RUNX2	0.466909573	0.06867	0.46319893	0.07135	0.690856627	0.00704
TNFRSF11/	0.287437234	0.22077	-0.112885835	0.63074	0.627939934	0.00724
HRASLS5	-0.554224926	0.31931	0.172015436	0.75255	-1.539221232	0.00728
CEP41	-0.140638412	0.44012	-0.064904304	0.72027	-0.490475121	0.00741
KLF3	0.165437271	0.10402	0.060068156	0.55462	0.271405145	0.0076
TPH2	0.065102547	0.77488	0.401061485	0.07526	0.600507526	0.00761
CHST4	0.44118773	0.20578	-0.314587775	0.3804	0.913320907	0.0077
DRD1	-0.601450498	0.19658	0.378950233	0.36796	-1.366579769	0.00773
COX17	0.061295473	0.63237	0.1030563	0.41638	0.336396674	0.00783
MPDH1P1(	1.905760165	0.06021	-1.688987602	0.22272	2.641183781	0.00787
MSANTD4	0.004184948	0.97528	0.215826361	0.10599	-0.359659825	0.00788
TNS1	0.325417567	0.18357	-0.481166562	0.05083	-0.655023363	0.00796
CPNE8	0.020132046	0.90814	0.065486861	0.70617	0.459282424	0.008
LRRC47	-0.193899374	0.19371	-0.190247529	0.20103	-0.395780433	0.00803
NSUN5P1	0.209240663	0.33044	-0.328669921	0.12731	0.566817115	0.00827
OAZ3	-0.666740491	0.11559	-0.324295498	0.45135	-1.168193114	0.00827
MRPS18A	0.109087076	0.35915	0.214929312	0.06884	0.312030289	0.00831
SMPD2	0.109952474	0.484	0.198588267	0.19738	0.40453405	0.00848
BCHE	-0.481506106	0.18376	0.205827357	0.56913	-0.954061912	0.00854
CLMN	0.247520833	0.23291	0.074714599	0.71875	0.544835875	0.00851
KDELC1	-0.193010285	0.30656	0.097286225	0.60268	-0.500037212	0.00855
DYRK1A	0.099771618	0.20569	0.091075688	0.24627	0.206515077	0.00873
IAH1	-0.139475288	0.2701	-0.081262499	0.51753	-0.332788585	0.00872
DENND6A	-0.129920037	0.50219	0.271518299	0.1593	-0.507317698	0.0088
FLVCR1	0.193990322	0.29199	0.345627023	0.05979	0.481588932	0.00879
RNF103	0.193955914	0.23591	0.149024371	0.36211	0.427984631	0.00884
HPS1	0.186367758	0.13899	-0.054342335	0.66617	0.327110298	0.00887
ENSA	0.099962487	0.34873	0.192442138	0.07066	0.278308584	0.00895
SH3GL2	0.118657663	0.82527	-0.06347044	0.90662	-1.557110983	0.00901
NCS1	0.044876599	0.81245	0.00967445	0.95906	-0.498737735	0.00902
C14orf1	0.107006967	0.28688	0.100142724	0.31673	0.260941341	0.00905
PDZRN4	0.072835227	0.86102	-0.162780789	0.69624	-1.119476025	0.00906
E2F6	-0.252384849	0.10349	-0.026185732	0.86383	-0.405456259	0.00919
SDE2	-0.195085684	0.05703	-0.104075883	0.30452	-0.266655078	0.00936
N4BP2L2	-0.136315062	0.13772	-0.102719822	0.26251	-0.238565963	0.00944
PARP4	-0.075696169	0.31609	-0.002492578	0.97356	0.194933429	0.00948
P11-468E2	0.371924706	0.86228	0.331256782	0.87717	-6.138389719	0.00952
KLHL4	-0.703382461	0.1338	-0.015765756	0.97309	-1.220810775	0.00953
CHST3	0.230074114	0.18043	-0.029298826	0.86471	-0.450264966	0.00963
VWA2	-0.116070757	0.79327	0.498206566	0.25305	-1.188408838	0.00968
ATP9A	0.055849896	0.57006	-0.110481935	0.26111	0.253329889	0.00983
S100A11	0.287482156	0.07715	0.283266036	0.08147	0.419793875	0.00983

IKBIP	-0.229271066	0.12378	0.219187685	0.13675	-0.384506618	0.00999
MFSD7	0.125377207	0.57603	-0.421842256	0.06266	0.551428867	0.01008
SLC37A3	0.072443684	0.59028	0.100881714	0.45171	-0.346445243	0.01015
LYL1	-0.172175053	0.72509	-0.787726515	0.11941	-1.474711418	0.01024
MXD4	0.174338662	0.17823	0.08591035	0.50781	0.332291883	0.0102
RAB4B	0.334515057	0.13875	0.124986827	0.58187	0.576427879	0.01021
FP91-CNT	1.375755076	0.05252	0.962960809	0.17625	1.810896943	0.01024
MYO1D	0.253970863	0.06553	0.250066052	0.0693	0.353360306	0.01037
HOXC11	-0.182264707	0.81446	0.167469891	0.82836	-2.117324938	0.01041
MYCBP2	0.125925121	0.38343	0.094375802	0.51315	0.369186127	0.01056
LTBP1	0.018761077	0.9132	-0.040793016	0.81247	-0.440978741	0.0106
SPP2	-0.360889516	0.1744	-0.419352632	0.11293	-0.685537701	0.01068
FRK	0.125972017	0.39416	0.198111256	0.17992	0.376968794	0.01071
ST8SIA3	-0.029410852	0.96984	0.743591001	0.3404	-2.010727865	0.01079
ROR1	-0.165627772	0.54283	-0.294408622	0.2789	-0.69526202	0.01082
GNAQ	0.156972023	0.13712	0.144048729	0.17173	0.268358283	0.0109
IRF6	0.198130276	0.26128	0.112468201	0.523	0.447083475	0.01106
PRSS23	-0.248389956	0.21261	-0.105826258	0.5944	-0.506350842	0.01109
DPH1	-0.180378204	0.26055	-0.14457358	0.3654	-0.407789726	0.01115
PMS2	-0.20301951	0.08065	-0.092561982	0.41363	-0.293759276	0.01118
PRKAB1	0.1443336	0.22194	-0.152426723	0.19784	0.297476343	0.01134
XPC	-0.278079958	0.07467	-0.059412832	0.70197	-0.39454469	0.01136
ARL3	-0.143631691	0.21168	0.18500674	0.09796	-0.292162272	0.01161
SORT1	-0.049881434	0.66819	0.030085641	0.79545	0.292735089	0.01161
C11orf84	0.065088871	0.58071	-0.031286648	0.7902	-0.300080814	0.01169
RAB40A	0.061868231	0.87002	-0.012170396	0.97406	-1.012801049	0.01166
MYOZ3	0.136374243	0.68905	-0.076810891	0.82075	-0.871245496	0.01185
ARHGEF35	-0.071368155	0.55047	-0.023132735	0.84507	0.295594024	0.01196
IL1RAP	0.353294413	0.07879	-0.058373953	0.77156	0.503647674	0.0122
MROH1	0.233964625	0.15327	-0.315720914	0.05698	0.40876286	0.01224
BSN	0.783847657	0.11543	-0.317057957	0.53656	1.258378817	0.01226
USP54	-0.025658629	0.86466	-0.165828235	0.27083	0.375844168	0.01225
CTNNA1	0.131821373	0.0968	0.115905914	0.1439	0.198651021	0.01229
RASGEF1B	0.204179046	0.17478	0.111767921	0.45586	0.372086976	0.01235
TAX1BP3	0.192835716	0.21297	-0.082976798	0.59241	0.385911239	0.01245
PTCHD4	-0.325429286	0.34964	-0.01787631	0.95874	-0.877270804	0.01248
ZMYND10	0.398913291	0.50074	0.780015207	0.18178	1.415972916	0.01248
OSTF1	0.002033936	0.98814	0.008918873	0.94778	0.337772003	0.01273
GLS	-0.095251912	0.53868	0.067091113	0.66408	-0.386157139	0.01275
FMO4	0.530129377	0.18949	0.144371773	0.71986	0.990443457	0.01285
PURA	-0.139140831	0.26291	-0.182511047	0.13916	0.305272828	0.01294
ARFGEF1	0.13123482	0.44277	0.325135302	0.05692	0.424413103	0.01298
PBDC1	-0.012787847	0.92819	-0.039261908	0.7813	0.349349159	0.01299
GNE	0.305935029	0.11626	0.317048124	0.10313	0.48294234	0.013
FAP	0.386740669	0.49952	0.485354821	0.39603	-1.469948946	0.01303
DNMT1	-0.161260076	0.31296	-0.171438631	0.28308	-0.396754633	0.01309
FAM110A	0.340791218	0.11635	-0.309220882	0.16861	0.528505053	0.01322

PARP6	0.176129326	0.20946	0.037267959	0.79088	0.346893261	0.01338
PNMA2	0.219421661	0.43459	0.346906832	0.21641	0.693548396	0.01343
NBAS	-0.123252471	0.27668	0.146992037	0.19258	0.279417103	0.01347
FUT4	0.078442025	0.6564	-0.069000446	0.6954	0.433733294	0.01353
NTNG1	0.671852589	0.22108	0.909200586	0.09672	1.346067466	0.01371
HLX	0.218103019	0.50925	-0.360296051	0.27732	-0.817977141	0.01379
RNGTT	-0.072250578	0.54375	0.192997857	0.09953	-0.293606827	0.01389
PODXL2	-0.190014082	0.47023	-0.223986849	0.3923	-0.657601384	0.01392
USB1	0.567981177	0.08619	0.469674541	0.15723	0.815701872	0.01396
RAB3A	0.648689936	0.09019	0.014088853	0.9715	0.945244244	0.01404
FAM160A1	0.16200986	0.49374	0.238622471	0.31275	0.577430778	0.01418
BRD2	0.236036643	0.05813	0.038763384	0.75576	0.305401615	0.01421
S100A2	0.754925473	0.39865	0.675455419	0.44866	2.166372533	0.01435
HLA-DPA1	-0.785893715	0.0891	-0.424586576	0.34526	-1.19795863	0.01445
LEAP2	0.034071784	0.90726	-0.221556488	0.45168	0.691804236	0.01467
HAGHL	-0.692741199	0.12501	-0.505057638	0.25779	-1.100378255	0.01471
H2AFY2	-0.096432648	0.63946	-0.206131015	0.31628	-0.502676722	0.01506
ETV5	-0.090297764	0.52233	0.203513093	0.14519	-0.344840273	0.0151
TOX3	0.203340496	0.30587	0.015428377	0.93802	0.481522102	0.01515
RAD54B	-0.405410189	0.08231	-0.324379594	0.15892	-0.564324378	0.0152
P11-139H15	-0.377961159	0.06255	-0.168652747	0.39063	-0.495470205	0.01527
RAB27A	0.117125625	0.59329	0.222188197	0.31004	0.529881308	0.01537
TAF5	-0.251246776	0.06523	0.047369305	0.72086	-0.330977137	0.01537
NAP1L3	-0.111503221	0.74679	0.243454368	0.4787	-0.844708826	0.0154
EPHX2	0.243844713	0.2654	0.151280791	0.48584	0.525060934	0.0155
GRHL1	0.079855532	0.64841	0.008840499	0.95952	0.41984076	0.01553
SRC	0.06987849	0.59929	-0.131212591	0.32476	0.321098404	0.01548
MCOLN3	-0.435321635	0.2678	-0.751817177	0.0581	-0.97384466	0.0158
FSCN1	0.271523752	0.34396	-0.18163899	0.52736	-0.696315334	0.01589
KRT5	2.665777293	0.08131	1.191783192	0.4365	3.687064781	0.01588
ZNF93	-0.139381215	0.20735	-0.160441104	0.14245	-0.267421366	0.01596
HMG20B	-0.146717261	0.22528	-0.081245693	0.49996	-0.290853484	0.01617
PPP2R2D	-0.043780409	0.63932	0.033750578	0.71474	-0.226027085	0.01617
PRRC2C	0.275275374	0.08723	0.097149246	0.54612	0.38678543	0.01625
MAGED4B	-0.207148578	0.27128	-0.012208795	0.94817	-0.453093315	0.01632
CHP1	0.021350468	0.88402	-0.149004367	0.30865	0.350692539	0.01649
SLC25A25	0.008873996	0.95773	-0.012357437	0.94087	-0.403343089	0.01646
BCL2L11	0.632092025	0.06793	-0.262557276	0.47671	0.853116397	0.01654
RNF130	-0.151418119	0.1117	0.101163579	0.27975	-0.229163534	0.01655
CD63	0.042771283	0.67732	0.130741884	0.20302	0.246070017	0.01659
SPATA6L	-0.055825646	0.77655	-0.335516201	0.08948	0.465588898	0.01657
RHCG	1.629683316	0.11046	1.300847004	0.20369	2.434633341	0.01674
CORO2A	0.205702165	0.29408	0.210995848	0.28124	0.468010103	0.01677
INRNPA3P	0.467022347	0.23918	0.493122618	0.20583	0.911225974	0.01688
CTSE	-0.00501353	0.98006	-0.163374049	0.41538	0.478422683	0.01706
HSPA13	-0.14421013	0.40583	0.058395458	0.73582	-0.414553878	0.01705
PROCA1	-0.291390551	0.28331	-0.499635905	0.07822	-0.652843303	0.01759

CHMP6	0.118918765	0.47703	-0.148687082	0.37125	0.394411608	0.01763
SFPQ	-0.15346052	0.17848	-0.177721981	0.11909	-0.270438591	0.01778
SS18L2	-0.245821741	0.12078	0.182063826	0.23295	-0.379601666	0.01782
STK17A	-0.110906831	0.39247	0.001873573	0.98838	-0.307865434	0.018
TRIM14	0.188981371	0.33621	0.088585471	0.65028	0.459729518	0.018
ZNF470	0.172390956	0.19653	0.177072294	0.18152	0.315151683	0.01802
ZNF436	-0.297858942	0.08095	-0.299576091	0.0787	-0.403627153	0.01809
GSTA2	-0.853752185	0.10585	0.137211058	0.79262	-1.25248319	0.01831
RBM47	0.297931917	0.07088	0.021943095	0.8941	0.38784086	0.01862
TIMM17B	-0.189901856	0.07229	-0.048553658	0.64061	-0.248672763	0.01873
TMEM17	-0.360831524	0.17024	-0.033908838	0.89315	-0.63132228	0.01873
TOM1L2	0.310105674	0.06615	0.031863035	0.85214	0.398220275	0.01871
INSIG1	0.188550361	0.24688	0.12551202	0.44064	0.382310352	0.01882
COX6B1	0.139508599	0.18112	0.016284243	0.87575	0.244370117	0.0189
HAND2	0.36475135	0.19704	0.057565895	0.83907	-0.674983878	0.01889
CEACAM7	0.712598935	0.19523	-0.307665832	0.57678	1.289233332	0.01902
ZNF766	-0.152251883	0.13366	0.063135932	0.52685	-0.237530625	0.01896
ETFDH	-0.193854416	0.14381	0.100386017	0.44214	0.306595174	0.01905
PRR12	0.694825166	0.06608	-0.218349632	0.56725	0.887530546	0.01919
FKBP14	-0.031409119	0.85588	0.270093866	0.11728	-0.406119402	0.01924
NOS3	-0.146291995	0.55667	-0.319166037	0.20048	-0.587280675	0.01922
NUDT16P1	-0.249360839	0.33555	-0.044245996	0.86045	0.573271062	0.01924
ZNF260	-0.141675743	0.23347	0.113452661	0.33646	-0.279050132	0.01925
IL17REL	0.989798622	0.06123	-0.624404891	0.28489	1.220690467	0.01948
ABHD14A	-0.372518719	0.05627	-0.278706008	0.14704	-0.456714959	0.01958
ASB4	-0.561545567	0.31542	-1.057838598	0.06116	-1.339551869	0.02002
ARRB2	-0.236835394	0.2158	-0.128105319	0.49503	-0.446223387	0.02016
CACNA1A	0.274760075	0.63884	0.127816151	0.82665	1.356902671	0.02035
SOWAHB	-0.099429391	0.57444	-0.091649815	0.60261	0.40470992	0.02036
PYGM	0.480848757	0.20268	0.634990042	0.09204	0.872482959	0.02054
ANXA4	0.431541968	0.09702	0.132642542	0.60999	0.602117819	0.02056
ZNF823	0.152907934	0.35741	0.108311283	0.51068	0.379420048	0.02073
TLR3	-0.224557602	0.46682	0.151886723	0.61884	0.704341282	0.02081
LEPREL1	-0.458690718	0.0935	-0.074197034	0.78563	-0.631833911	0.02083
ARPC1B	0.211142098	0.11871	0.226884975	0.09208	0.312687302	0.02087
CA9	-0.618892824	0.09693	-0.401092739	0.28162	-0.860968603	0.021
RRP1B	-0.164331859	0.12355	-0.011809723	0.91125	-0.246366398	0.02103
KCTD2	0.219057215	0.13285	0.155817956	0.28445	0.335623769	0.02117
ADCY4	0.277627952	0.47193	-0.266997106	0.49665	0.877965175	0.02133
BBS9	-0.21023796	0.3626	-0.106141165	0.64274	-0.533606672	0.02131
PSMB10	0.173946845	0.17209	0.178624007	0.15608	0.29161696	0.02133
CCDC88A	0.083568142	0.61733	0.263716298	0.11454	-0.385857466	0.0214
ZNF283	-0.04568567	0.82538	-0.10194324	0.61597	-0.482202523	0.02147
AHCTF1P1	0.099561732	0.78432	0.199668138	0.57824	0.811766516	0.02153
HADH	-0.477394917	0.12539	-0.430394106	0.16627	-0.71915491	0.02157
C9orf89	0.245284109	0.14958	0.143312104	0.40076	0.388376914	0.0216
ARVCF	-0.006829953	0.97388	-0.380148652	0.07024	-0.4841039	0.02162

FOXK2	0.05620858	0.7225	0.173173971	0.27196	0.361452526	0.02191
GPAM	-0.16240735	0.19963	0.104307389	0.40205	-0.290769638	0.0219
DNAJA3	-0.119661786	0.38109	0.065542893	0.62794	-0.313381618	0.02196
NICN1	0.332237895	0.057	-0.256171606	0.14801	0.397739341	0.02202
PLP2	-0.06445807	0.69841	0.161151251	0.33124	0.379613845	0.02203
ZNF3	-0.010017497	0.9253	-0.030421129	0.77473	-0.245777914	0.02208
YWHAH	-0.053657965	0.53041	0.058864191	0.48891	0.194762365	0.02211
PGM5	-0.09570876	0.79917	-0.47368761	0.20724	-0.865052851	0.02218
PPARGC1E	0.073296865	0.74953	-0.029973291	0.89595	0.521487767	0.02242
GPC1	-0.032926751	0.87559	-0.39218999	0.06263	-0.481198669	0.02258
CCDC80	-0.050113077	0.8541	0.226514457	0.40562	-0.621792291	0.02275
FLYWCH2	-0.140759146	0.47977	-0.046131976	0.8142	-0.460509392	0.02285
BTBD3	0.348070854	0.12472	0.417641054	0.06539	0.514528365	0.02299
GNG11	0.028960828	0.9063	-0.001590434	0.99483	-0.565637301	0.02303
P11-434D12	-0.068133932	0.94945	-0.861784619	0.4262	-2.630266566	0.02319
ZNF441	-0.184694715	0.30076	-0.161728273	0.36018	-0.406842925	0.02344
TTPAL	-0.064611656	0.58567	-0.084329914	0.47489	-0.269512501	0.02355
KRBA1	0.53476702	0.05177	0.185675511	0.50362	0.624499997	0.02361
ENOX2	0.006394831	0.97659	0.124515051	0.56255	0.485216047	0.02374
CDH19	1.394780428	0.05045	0.567973381	0.43378	1.596638082	0.02391
THAP11	-0.254506884	0.10399	-0.231860248	0.13562	-0.354059162	0.02399
TMEM161A	0.261501805	0.14626	-0.130138499	0.47007	0.404405305	0.02397
APOL6	0.198340595	0.22441	-0.013724629	0.93302	0.367161859	0.02424
TMEM63B	0.227218618	0.16817	-0.257692153	0.1185	0.37055798	0.02442
PDE7B	0.912788926	0.17263	0.541810364	0.41869	-1.585217587	0.02446
ST3GAL6	-0.288112553	0.38361	-0.079280228	0.80971	-0.750206188	0.02449
NEK10	-0.296141501	0.4243	-0.247583596	0.50458	-0.858936953	0.02454
POLR3F	-0.108904409	0.49525	0.112674722	0.47026	-0.358653277	0.02456
CD300A	-0.017724561	0.95314	-0.51300462	0.08964	0.675241755	0.0247
NUDT11	0.223360031	0.4108	0.256822612	0.34244	-0.621425988	0.02476
WFDC3	-0.369647342	0.43256	-0.804394646	0.09587	-1.149093603	0.02478
RANBP3L	0.546168783	0.40177	-0.853969542	0.19849	-1.603959877	0.02484
FARS2	-0.264525138	0.0745	-0.077453421	0.59654	-0.33398501	0.02495
SPDYE1	0.251960384	0.45094	-0.197358263	0.55981	0.734296275	0.02501
ANTXR2	-0.028310759	0.84738	0.149879596	0.30702	-0.330079744	0.02518
PLOD1	-0.042703929	0.74915	-0.189061024	0.15688	-0.29918681	0.02518
MVK	0.271277245	0.10091	-0.045089732	0.7854	0.36906778	0.0253
OSBPL2	0.216466171	0.0707	-0.033394498	0.78053	0.267246165	0.02536
GTF2B	-0.196468926	0.05228	0.119745463	0.22554	-0.226682556	0.02542
MRPS31P4	-0.262893098	0.3085	-0.134473063	0.59433	-0.590431501	0.02546
BLNK	-0.071697597	0.76322	0.266973571	0.25941	0.528264728	0.02565
SLC39A9	0.030451246	0.69877	0.139255224	0.07517	0.175066833	0.02564
CPT1B	0.231125034	0.26969	-0.133436594	0.52641	0.465973123	0.02571
RPRD2	0.214940684	0.06579	-0.200667864	0.08712	0.259869111	0.0258
CNTRL	-0.22228931	0.16224	-0.067434035	0.66956	-0.354688581	0.02589
MTND2P28	0.177837704	0.74833	0.84371716	0.12006	1.205170598	0.02582
SMARCB1	-0.039274364	0.67015	-0.11135546	0.22259	-0.207017648	0.02587

KMT2E	0.195313172	0.16339	0.050535312	0.71826	0.311266972	0.02613
ZNF852	0.170759641	0.55927	0.357303745	0.21493	0.633200633	0.02613
GARNL3	-0.151108202	0.46198	-0.193004036	0.34511	-0.454688165	0.02635
KCNS3	0.037657908	0.84107	0.013781108	0.94113	0.411914195	0.02635
MBLAC1	0.691632651	0.13323	-0.532407881	0.28244	1.009670643	0.0264
IL34	1.054651525	0.06215	0.855519462	0.13117	1.27586369	0.02648
AKAP1	-0.074845333	0.72645	-0.051432455	0.80978	0.473200671	0.02667
KIF16B	0.021865425	0.91441	0.267846913	0.18615	0.44852062	0.02681
LRRC48	0.149836339	0.64238	-0.434826945	0.18056	-0.71609471	0.02677
CRB3	0.17242998	0.39389	-0.006953942	0.97263	0.442471471	0.02699
ABHD16A	0.162604066	0.13035	-0.03571673	0.73857	0.23665964	0.02702
KLHL36	0.042503107	0.63564	-0.101477681	0.25473	0.195898538	0.02708
CEP135	0.006352574	0.96258	-0.229580068	0.08945	-0.300433597	0.02733
SLFN11	0.400330539	0.30367	0.682080767	0.07933	0.858349111	0.02727
ZNF827	0.279783337	0.16093	0.072040301	0.71798	0.440120071	0.02736
EEPD1	-0.062146568	0.85205	-0.235185444	0.48131	-0.737211081	0.0274
MGMT	-0.262972743	0.10864	-0.09524511	0.55282	-0.362107503	0.02777
RPSAP14	-0.365586306	0.52005	-0.409032392	0.46776	1.169070381	0.02775
?11-104O1!	0.411601551	0.21139	-0.222746998	0.51963	0.703295147	0.02783
SLC5A9	0.571809524	0.37149	-0.605465001	0.35569	1.402937587	0.02785
HEATR4	0.859407228	0.12588	-0.018162316	0.97477	1.206200955	0.02801
AKAP3	0.392068579	0.40607	0.084451931	0.84994	0.977198394	0.02806
CPSF2	-0.191639479	0.06156	0.123213692	0.22674	-0.224794727	0.02808
ZNF569	-0.422015924	0.07952	0.000210509	0.99929	-0.532027169	0.02864
ASXL2	0.053299244	0.78304	0.130234921	0.50055	0.423001212	0.0287
SFXN5	-0.083242956	0.68302	-0.116048864	0.56756	-0.445270315	0.02871
GDPD2	0.593313973	0.11598	-0.185505956	0.62566	0.823405943	0.02877
COG8	-0.162924374	0.10634	-0.126093598	0.20558	-0.219618528	0.02893
sa-mir-119	0.125138092	0.63709	0.107324641	0.68261	0.566396902	0.02894
ITPRIP	0.026409332	0.88763	-0.067093603	0.71857	-0.413060317	0.02903
KIAA1468	0.086264121	0.50085	-0.096766027	0.44859	0.278628827	0.02897
PKP1	1.684451166	0.17654	0.411078293	0.74949	2.717440036	0.02902
PRDX2P2	0.826928572	0.09141	-0.565115575	0.30409	1.052468995	0.02883
TMEM209	0.011591474	0.91981	0.147032526	0.19556	-0.250626439	0.02918
HTRA1	0.012313146	0.96956	0.399854263	0.20946	-0.716203322	0.02933
PIR	-0.255438882	0.22637	-0.116152807	0.58166	0.457712957	0.0294
BMP3	-0.167914861	0.58359	-0.118269373	0.69925	0.665327648	0.02956
STMN1	-0.200040226	0.30297	0.052144733	0.78813	-0.422486362	0.02967
TBX18	0.787052626	0.15434	0.283167545	0.60862	-1.212967441	0.02992
PACRGL	-0.159544944	0.25746	-0.040944108	0.76764	-0.305931915	0.02997
ZNF43	-0.131441147	0.43162	-0.246783816	0.13749	-0.363297655	0.03002
CORO6	0.051080466	0.92265	0.158216472	0.77381	1.146872556	0.03009
?11-192H2:	0.315391695	0.11544	-0.11789991	0.56165	0.431649147	0.03026
DGKE	-0.239737863	0.23302	-0.16612048	0.40307	-0.432876729	0.03029
MKL2	0.322143188	0.05712	-0.214593894	0.20547	0.366166867	0.03033
TTLL1	-0.283634629	0.15775	-0.277093221	0.16497	-0.441290792	0.03041
RPUSD3	-0.15041302	0.07985	0.027606941	0.7417	-0.185403536	0.0308

CEP120	-0.045960659	0.66385	0.17916684	0.08487	-0.228548075	0.03092
CPA4	-0.065819149	0.82528	0.27022109	0.35896	-0.653613964	0.03091
P11-424C2(	-0.711662331	0.0725	-0.636028562	0.10461	-0.858540597	0.03103
RBM15B	-0.060538177	0.5288	-0.065093888	0.49692	-0.20762614	0.03106
PRELID2	-0.330047177	0.09279	0.224386092	0.25073	0.421007749	0.0311
ZNF177	-0.211896562	0.46954	-0.322943177	0.26217	-0.629386061	0.03116
ADD2	-1.081049181	0.15999	-0.329561203	0.66901	-1.672861493	0.03132
DUSP16	0.310233801	0.11569	0.091660823	0.64181	0.42427108	0.03129
FAM216A	0.096325813	0.49731	0.141105752	0.31228	-0.312998186	0.0313
MSMO1	0.133517141	0.34093	0.036669055	0.7936	0.301788539	0.03128
TM6SF2	0.964615553	0.10843	0.119595206	0.84474	1.288328437	0.03136
FRMD5	-0.204828822	0.42911	-0.056142929	0.82642	-0.564140558	0.0314
DYNC2H1	-0.077687734	0.75078	-0.056375359	0.81738	-0.526797321	0.03155
TMEM35	0.009316448	0.9734	0.244164226	0.37457	-0.619290903	0.03145
WDR5B	-0.107575748	0.43154	-0.220244978	0.10563	-0.297198579	0.03151
D-2583A14	-0.559009387	0.49215	1.05728772	0.16172	1.609046116	0.03171
OSR2	-0.567861294	0.3424	-0.787428054	0.18894	-1.34228275	0.03177
SEMA4F	-0.480454044	0.08477	-0.020262709	0.94062	-0.597352113	0.03175
CKAP2L	-0.335892634	0.10688	0.038483408	0.85255	-0.44722754	0.03194
NKRD20A5	-0.43557412	0.39321	-0.503172695	0.31746	-1.131031442	0.03217
B3GNT1	-0.187087484	0.14503	-0.031290929	0.80414	-0.275542747	0.03224
DIAPH1	0.351617938	0.05685	0.187820594	0.30913	0.395365099	0.03224
IGFBP5	0.140076842	0.43108	-0.1288613	0.4689	-0.380637976	0.03244
C16orf59	-0.377506854	0.16342	-0.270409472	0.31424	-0.578508209	0.03265
SSR1	-0.104123996	0.21159	0.137489186	0.09748	-0.177738496	0.03275
C16orf70	0.060225639	0.58905	-0.13196178	0.23874	-0.239283248	0.03285
CDC25C	-0.306319078	0.16567	0.142855933	0.51106	-0.472180539	0.03284
VPS52	0.072771361	0.60009	-0.038190587	0.7825	0.295165162	0.0328
APOBEC1	0.052809938	0.92086	0.390651406	0.45395	1.096475979	0.033
LRFN4	0.213409718	0.46195	-0.266321231	0.35637	-0.62446425	0.03296
MLTK	-0.042329475	0.65805	0.085308415	0.36823	-0.204191245	0.03301
SOX11	0.195402724	0.38476	-0.121067748	0.59044	-0.481003013	0.03303
DNAJC15	-0.345009001	0.14074	0.172357154	0.45638	-0.498895151	0.03306
ANKRD13E	-0.123999063	0.61661	-0.286703094	0.24823	-0.53465544	0.0331
CCDC51	-0.271439927	0.05274	0.206122267	0.1221	-0.296449666	0.03326
HELQ	-0.158295339	0.46216	-0.015355214	0.94261	-0.458955806	0.03325
KIF5B	-0.012957208	0.8919	0.11970946	0.20865	0.202825431	0.03329
MTRNR2L1	-0.193915534	0.66105	0.325694731	0.44484	0.893321712	0.03324
NVL	-0.079946474	0.53038	0.055504463	0.65991	-0.271194392	0.03328
OSBPL10	-0.209469076	0.23897	-0.296139673	0.09494	-0.379254168	0.03322
RIN2	0.353367396	0.08552	0.214862693	0.29497	0.437284102	0.03323
NCOA3	0.141142022	0.12198	0.012565494	0.89042	0.193662961	0.03359
TFRC	0.224898949	0.2003	0.234642334	0.18132	0.3729117	0.03366
DUSP12	-0.228495113	0.05892	0.027835835	0.81418	-0.25559391	0.03373
KCNMB2	-0.918047121	0.10251	-0.197866932	0.72201	-1.207335508	0.03374
P11-396K3	-0.098126058	0.51062	0.017044441	0.90886	-0.316955441	0.03376
SYNE1	0.213144894	0.17727	-0.100073447	0.52659	-0.336297227	0.03393

B4GALT5	0.149732529	0.12469	0.014287652	0.88344	0.206361124	0.0341
TSPAN1	0.019264238	0.9243	-0.165093875	0.41545	0.429053332	0.03413
MED17	-0.141783228	0.21505	0.187642531	0.0991	-0.242988972	0.03424
ZNF141	-0.186861344	0.06987	-0.187784653	0.06532	-0.217523319	0.03425
IDI1	0.067815081	0.62843	0.212625725	0.12872	0.296202745	0.03437
CLYBL	-0.534106211	0.09266	-0.318867796	0.29284	-0.650750887	0.03475
NTM	-0.097577321	0.82945	-0.162428622	0.71972	-0.981146655	0.03475
PIP5K1B	0.265755329	0.43455	0.490135121	0.14818	0.715561725	0.03476
TECPR1	0.274530208	0.20353	-0.362173212	0.09531	0.456833701	0.03467
ELP5	0.152741002	0.11403	0.161114109	0.09193	0.203271712	0.03486
MAPRE2	-0.063959575	0.7159	0.076138123	0.66391	0.368721536	0.03508
AC125232.1	0.113105988	0.63361	0.062234056	0.79235	0.493895171	0.03541
ALS2	0.080360264	0.40182	0.176755173	0.06413	0.20157616	0.03537
C11orf70	-0.05056196	0.9158	0.516781999	0.27642	-1.081822232	0.03543
DROSHA	0.269681928	0.3188	0.277029324	0.30507	0.568632591	0.03542
PHC3	0.307053788	0.09171	0.152232974	0.40306	0.382545475	0.03554
LUM	0.102387968	0.73014	0.275045592	0.35399	-0.623970526	0.03569
SV2C	-0.436404546	0.38409	0.18479374	0.71382	1.039857778	0.03567
ZBTB26	-0.211445523	0.16431	-0.053983118	0.71679	-0.318959964	0.03595
CCDC110	-0.646895553	0.12625	-0.083670516	0.83265	-0.874127658	0.03615
FABP1	0.387577826	0.48674	0.675128534	0.22567	1.167476745	0.03615
FAM129C	0.095390493	0.57697	-0.247401263	0.15114	0.35319857	0.03612
POC5	-0.187640635	0.24537	-0.033342754	0.83367	-0.340626543	0.03612
ADO	-0.06532327	0.41322	-0.019947614	0.80012	-0.167751623	0.03625
LDLRAP1	0.256059492	0.30429	0.011212894	0.96403	0.519641606	0.03627
TSPYL1	0.122026526	0.14273	0.106778505	0.19849	0.173961992	0.03637
SMURF2P1	0.163627968	0.56645	0.461213636	0.09191	0.573172088	0.03661
KRBOX4	-0.20320181	0.20363	-0.225982036	0.15453	-0.334833476	0.03666
VCPKMT	-0.326856342	0.1308	-0.131374423	0.53568	-0.452601305	0.03673
SECISBP2	-0.123034443	0.19895	-0.004112909	0.96555	-0.20009081	0.0368
DAK	-0.223236362	0.11894	-0.157972388	0.26646	-0.297970367	0.03721
EDN3	-0.108935683	0.77923	0.197053473	0.61161	-0.811011078	0.03731
ORM1	-0.176734658	0.65284	0.227516196	0.56127	0.812662071	0.03767
DUSP3	0.164197587	0.06783	-0.044742011	0.61863	0.186619662	0.03777
SLC25A23	0.152229393	0.15325	-0.178325715	0.0961	0.221137029	0.03789
SNRK	-0.152681403	0.29528	-0.180771026	0.21429	-0.302907261	0.03799
TBCCD1	-0.144592027	0.21151	0.064440438	0.56986	-0.242946186	0.03802
DNAJC12	-0.045235767	0.87241	0.126610472	0.64618	-0.588358581	0.03844
EPHB6	-0.624275464	0.43845	-1.341702491	0.09708	-1.675592332	0.03856
KRT6A	1.783757007	0.17386	1.376020216	0.29457	2.712084994	0.03857
MVP	0.197841487	0.26197	-0.180347948	0.30664	0.364168878	0.0388
ACTR6	-0.231747622	0.09523	0.069756126	0.60466	-0.285567877	0.03899
ENKD1	-0.23338548	0.39913	-0.514441768	0.06259	-0.573076097	0.03901
FGD3	0.084530215	0.90634	-0.545656377	0.4392	-1.53257122	0.03936
GINS4	-0.298429958	0.19988	-0.028903644	0.90072	-0.478421612	0.0394
LMLN	-0.086034587	0.69959	0.034003537	0.87779	0.455628961	0.03948
TDRD12	-0.404057578	0.33523	-0.602162954	0.15247	-0.891924737	0.03956

NR1H3	-0.061328741	0.73262	-0.306170356	0.08833	0.365457361	0.03969
ABI3BP	0.378165937	0.12463	-0.030526482	0.9024	0.506008288	0.03976
MAGEL2	0.596804203	0.05956	-0.629122118	0.05755	-0.688912511	0.0399
C1RL	0.152872042	0.1898	-0.062942034	0.58929	0.239245433	0.04001
KLHDC3	-0.12525394	0.18534	0.011531896	0.90208	-0.194199198	0.04018
IRF5	-0.544176339	0.33413	-1.017428493	0.07039	-1.177185073	0.0402
FBXO43	-0.108358678	0.77865	-0.32231538	0.40369	-0.834371892	0.04063
METTL2A	0.146842591	0.38249	0.290794186	0.08071	0.341371527	0.04068
PDGFD	-0.296957352	0.29602	-0.032139072	0.90971	-0.582687686	0.04068
SNAPC1	-0.07596813	0.65119	0.154027829	0.35269	-0.346994408	0.04068
LONRF3	-0.10863056	0.71032	0.06859594	0.8126	0.579347921	0.04099
CCDC78	0.258111374	0.46009	0.077452178	0.82565	0.709093709	0.04118
GPR75	0.531906527	0.09281	-0.006985231	0.98289	0.639069624	0.04123
PHKG2	-0.074140829	0.54043	-0.107997364	0.37184	0.243902284	0.04141
CTGLF12F	0.443075525	0.13143	0.189999164	0.51875	0.595379531	0.04157
ERMARD	-0.092470677	0.66038	-0.228403964	0.27483	-0.435147316	0.04159
TANGO2	0.222832629	0.09927	-0.013827968	0.91833	0.273443526	0.04157
ZNF765	-0.183501223	0.26994	-0.160807176	0.33192	0.337269096	0.0416
AP001885.1	0.542414726	0.08859	-0.354848118	0.29772	0.641513379	0.04175
IKBKG	0.134856511	0.82042	-0.706862244	0.245	-1.293670466	0.04174
SFTPB	0.22600076	0.55092	-0.684613654	0.07628	0.763407981	0.04185
ACAD9	-0.177517662	0.1129	-0.160753028	0.14696	-0.226669982	0.04208
HSD17B4	0.119731858	0.48449	0.295395045	0.08425	0.347703281	0.04214
SLC20A2	0.141002397	0.212	0.067648096	0.54749	0.229168882	0.04214
ZNF217	0.119283737	0.24893	0.153869686	0.13612	0.210039683	0.04212
RBM20	0.861633832	0.06462	0.441580758	0.34579	0.956840591	0.04249
C1QTNF1	0.302334878	0.34151	-0.155681421	0.62483	-0.664421927	0.0429
ANKRD13C	-0.16262244	0.15969	0.144717106	0.20638	-0.233317541	0.04305
CFP	0.36383634	0.49027	-0.739178475	0.18728	-1.085898572	0.0431
SAV1	0.207066757	0.36114	0.408682923	0.07079	0.457394468	0.0433
TRUB2	-0.216465777	0.09176	-0.106584459	0.40299	-0.25845735	0.04362
UBE2SP2	-0.004166113	0.98527	0.177634773	0.42636	-0.46169526	0.04362
KIAA2018	0.175841175	0.24902	0.070259725	0.64466	0.306916717	0.04379
TCEB3	-0.248218076	0.31291	-0.277092169	0.25898	-0.498584149	0.04378
RNF169	0.102570095	0.13119	0.120795677	0.07251	0.136476711	0.04398
KIAA1467	0.13527908	0.5055	0.045779507	0.82075	0.407526823	0.04409
ANTD3-TME	0.212168198	0.45717	0.367664271	0.19556	-0.582742712	0.04405
POLR2E	0.16402099	0.2659	0.148568849	0.31227	0.295892464	0.04416
MTMR1	-0.249205707	0.08391	0.242808643	0.09056	0.288594472	0.04427
PCSK6	-0.034170715	0.88448	-0.229586674	0.32875	0.472119836	0.04426
ARMC7	0.313956777	0.05977	-0.209900002	0.22355	0.333504719	0.0445
C12orf73	-0.253094836	0.09001	-0.107680486	0.46315	-0.298031112	0.04448
IRGM	0.356911674	0.20902	0.094480458	0.74126	0.564245915	0.04456
P11-400G3	-0.403744962	0.40437	-0.041420537	0.92728	0.852229202	0.04459
ACSL1	-0.344961427	0.3415	0.378319541	0.29595	0.726702509	0.04464
FBXL15	0.047230044	0.84254	-0.006306614	0.97865	0.465072824	0.04475
TRIP13	-0.335693881	0.08437	0.031279627	0.87074	-0.389171009	0.04475

NXPE3	0.334174077	0.05261	0.257657263	0.1357	0.34969418	0.0449
TSPAN15	0.072995083	0.67007	0.091105853	0.59397	0.342370241	0.04489
CAV2	-0.312968128	0.07541	0.11214056	0.52084	-0.352497601	0.04496
DISC1	-0.108498617	0.6781	-0.313616919	0.23136	-0.528832897	0.045
C1orf35	-0.198619432	0.22173	-0.269539889	0.09391	-0.324184029	0.04507
FAM134B	0.066561547	0.83818	0.589740889	0.06784	0.647349298	0.0451
CIRH1A	0.008827672	0.95635	0.134335479	0.40158	-0.323892441	0.04513
P11-1277A3	0.100283992	0.61568	-0.177553743	0.37983	0.391885402	0.04516
C3orf38	-0.122317585	0.16629	0.072572894	0.40246	-0.177209958	0.04522
RMND5B	-0.05972753	0.54288	-0.126212292	0.19651	0.194355502	0.04535
P11-274B2	0.570269758	0.11171	-0.75756141	0.0512	0.711239676	0.04534
UBAC2	0.008740059	0.92771	0.167408983	0.07904	0.191469035	0.04538
SLC25A36	-0.072794573	0.60477	0.12243406	0.38329	0.280869093	0.04545
SYNPO2L	0.22981495	0.51611	-0.658283071	0.08572	-0.773439853	0.04543
MAGED1	-0.09439861	0.42184	-0.026896033	0.81889	-0.235109918	0.0455
TMEM98	-0.190171344	0.24347	-0.22947721	0.15847	-0.326489631	0.04548
ZNRF2	-0.051429025	0.7885	0.237810672	0.21223	0.380411407	0.04559
FBXO4	-0.25933695	0.27373	-0.238328021	0.30612	-0.479145922	0.04573
IMPAD1	0.046702954	0.78251	0.133062182	0.43148	0.337992522	0.04569
USP12	-0.002975632	0.97978	0.193403807	0.09784	0.233681539	0.04574
TRAPPC5	0.294402771	0.12611	0.259796586	0.17741	0.384119474	0.04586
MYL6B	-0.173089135	0.30725	-0.090448205	0.59204	-0.338856132	0.04596
GIF2-C20orf	0.012857001	0.96729	0.248465823	0.42396	0.617792654	0.04595
KRT20	1.092304301	0.1539	1.330891045	0.0822	1.527577808	0.04604
ZNF625	-0.395664196	0.10318	-0.380770981	0.11578	-0.485402206	0.04622
FAM98B	-0.225276901	0.46383	0.264119294	0.3868	0.609430628	0.04632
SEMA3D	-0.173291912	0.38183	0.021592607	0.91288	-0.394944723	0.04651
UFSP2	-0.13451035	0.35526	0.075526856	0.59928	-0.288107167	0.04654
P11-505K9	-0.944049108	0.0718	-0.368362849	0.47243	-1.067361774	0.04661
HGS	0.090350018	0.46109	-0.200817911	0.10272	-0.244584609	0.04666
CBS	0.230566703	0.48587	0.437864989	0.18235	0.652998471	0.04669
GIPR	0.449732309	0.08741	-0.5089345	0.05969	0.521746609	0.04673
SLIT1	-0.613222769	0.23409	-0.879549912	0.08994	-1.034250561	0.04688
HSPD1P6	0.865642764	0.06234	0.832888509	0.06585	0.909024572	0.04708
GPALPP1	-0.170128603	0.09864	0.147386308	0.14598	-0.203970982	0.04738
CARD8	-0.059683269	0.67963	-0.261605475	0.06988	-0.287792974	0.04744
TMEM218	-0.37229279	0.05571	-0.290062342	0.13209	-0.382576176	0.0475
ZSCAN23	-0.075079369	0.73619	-0.280243043	0.21206	-0.444016656	0.0478
SRD5A1	-0.235167392	0.06023	-0.120840338	0.33132	-0.24725685	0.04785
CHIC2	0.193082809	0.24794	0.10982846	0.50891	-0.335175058	0.04793
ZNF226	-0.248502512	0.14325	-0.094865791	0.57525	-0.335312462	0.04802
SCD	0.127391949	0.45381	-0.140718779	0.408	0.33605743	0.04813
TPST1	0.193769515	0.23894	0.127712794	0.43709	-0.327548138	0.04814
DOPEY1	-0.152898464	0.20857	-0.113781096	0.34744	0.239291125	0.04826
GNG2	0.169145696	0.43694	0.092373506	0.67107	-0.431114635	0.04849
NCOR1	0.127754808	0.15649	-0.111704538	0.21485	0.17770199	0.0485
HMGCS1	0.19498833	0.29926	0.038605507	0.83716	0.37038644	0.04861

STAU1	0.058605682	0.58027	0.126530076	0.23115	0.208478855	0.04865
CD68	0.237349108	0.45129	0.347200459	0.27035	0.620172588	0.0489
TRAPPC2	-0.207973803	0.08572	-0.218835858	0.0689	-0.237490709	0.0489
USP43	0.132983163	0.5329	-0.131974855	0.53648	0.416344697	0.04892
JKAMP	-0.038471532	0.7399	0.220616127	0.05307	-0.228395185	0.04901
ADAM19	0.57902833	0.07214	0.288985302	0.36949	-0.63842696	0.04932
SGALNAC	-0.363696174	0.1847	-0.309309219	0.2559	-0.538971588	0.04945
DCAF12	-0.012691673	0.89055	-0.039283788	0.66814	0.179748043	0.04947
ZNF639	-0.022135324	0.91308	-0.004849832	0.98085	-0.39902578	0.04957
ABCC11	-0.075186305	0.92862	0.849298613	0.28239	1.618146252	0.03434
AC005363.!	1.631448204	0.1585	1.825944831	0.11016	2.478817076	0.02813
AC011322.:	0.877060949	0.59867	2.409594451	0.10431	3.201364661	0.02415
AC027612.:	0.873885393	0.32598	-0.110217275	0.9053	-2.708809953	0.03565
AC098824.(	2.090136588	0.20748	-0.127617714	0.94225	3.179735064	0.03916
AC108448.:	-0.208147689	0.86817	-1.132110256	0.40594	-3.0008133	0.04267
AKNAD1	-0.849146538	0.18574	-1.086720301	0.07616	-1.859113717	0.00532
ALG1L9P	-2.166484973	0.22848	-0.733941993	0.65002	-3.675768278	0.04367
ALS2CR11	-0.887776823	0.35753	0.328682732	0.7259	-2.215959892	0.03025
NKRD20A1	1.713849188	0.18926	0.167514874	0.90891	2.776862885	0.02944
AP000349.:	1.609316772	0.26277	2.447512823	0.07911	3.098239074	0.02489
AP000436.4	-1.943218531	0.22309	-1.569771047	0.29923	-3.43330943	0.03388
APOBR	0.848070576	0.21664	0.831354996	0.22085	1.634679423	0.0127
APOL3	1.331048688	0.07561	1.257744056	0.10514	1.95944416	0.01013
C1orf141	0.389664212	0.60858	0.572163034	0.42778	1.462139979	0.03263
8orf44-SGK	-0.88637593	0.69138	-1.029651024	0.64334	-5.775921481	0.01734
CAMKV	-1.415219234	0.15208	-1.088862432	0.23319	-2.750087267	0.02307
CBX3P2	-0.652802628	0.27578	0.21834568	0.69751	-1.348291173	0.0289
CD300LG	0.219912576	0.72914	-0.343977292	0.60797	1.499417177	0.0164
CEND1	1.003769587	0.06046	-0.639754142	0.28825	1.12124658	0.0358
CLEC4A	-0.232901757	0.73926	-0.257465622	0.7064	-1.855149499	0.03418
CPA5	0.99808104	0.57715	2.494840266	0.1191	3.292538389	0.04109
CR2	1.233429614	0.08315	1.013600829	0.14447	1.41338677	0.04068
CTB-20D2.:	-3.161989178	0.05207	-0.974654847	0.50696	-3.208611528	0.0487
D-2104P1	-1.953736616	0.13697	-0.765073775	0.486	-2.817211893	0.03606
CTRB2	-2.009751099	0.09838	-0.851770733	0.41334	-2.939719146	0.0261
ECHDC3	-1.082164083	0.16394	-0.284505351	0.70213	-3.3795866	0.0019
FTH1P20	-0.292446221	0.84767	-0.262611542	0.86186	-3.646594599	0.03742
GAPDHP6	-1.062706934	0.2995	-0.897653679	0.36622	-2.958618608	0.02407
GOLGA8M	0.364933797	0.55296	1.089807008	0.07113	1.338027463	0.02539
GPR61	0.420171584	0.53321	0.135671089	0.84167	1.505361191	0.02127
S1-124K5.:	0.264109224	0.81432	0.236323099	0.83199	-3.393398603	0.01843
GZMM	-0.621243005	0.4742	-1.083438777	0.22381	-2.203168506	0.03585
NRNPA1P	-0.280490375	0.76678	-0.264230142	0.77767	-3.230861484	0.01376
NRNPA1P	-0.550750106	0.62335	-0.385290147	0.72366	-2.975705488	0.03392
HNRNPKP	-0.883339039	0.35245	-0.710777428	0.43193	-2.296301326	0.03793
HSD3BP5	1.800067429	0.062	0.714576146	0.48933	2.061671655	0.02978
HSFY1	2.377795486	0.1447	3.009218702	0.05287	3.375062545	0.02832

HSPE1P27	1.449896419	0.11124	1.349629644	0.13622	1.866441536	0.0352
IL36G	-0.324802732	0.75634	0.286513037	0.77731	2.030542888	0.03208
INSL4	-0.387797923	0.66316	-0.332558815	0.70453	-3.9044271	0.00258
ISM2	-1.093697764	0.27842	-0.497210305	0.61068	-2.336219801	0.04808
\16c-360H6	2.416194081	0.2674	-0.127617578	0.95551	4.775287478	0.02124
LENEP	-0.527642671	0.49876	-1.049338592	0.2011	-1.94502787	0.04499
LINC00116	-0.140278321	0.88234	-0.564677618	0.55675	-3.819239546	0.00441
LIPM	0.639571546	0.24161	0.763134534	0.15168	1.674714411	0.00088
RRC37A13	2.232429054	0.105	-1.60039148	0.33108	2.693177107	0.04648
LTF	-1.22033121	0.3784	-1.636892305	0.19031	-3.638933551	0.0164
LY86	-1.481923005	0.23557	0.143645889	0.89367	-2.996370604	0.03353
NOTUM	-0.020272392	0.97512	-0.493656607	0.45548	-1.546504875	0.03171
PACSIN1	-1.335497025	0.15886	-0.481657359	0.6061	-2.285003553	0.03539
PARP4P1	0.918688944	0.46772	1.496242769	0.21233	2.575964507	0.02531
PLAC1	0.035768212	0.96738	-0.015450182	0.98556	-2.389449893	0.01964
PRB3	-1.665729568	0.13501	-0.959495939	0.34454	-3.548699097	0.00725
QRSL1P1	-0.274451886	0.77161	-1.42945009	0.16164	-2.458977197	0.03893
RD3	0.722785743	0.17682	0.63747055	0.22999	1.082601564	0.03796
P1-290F12	0.706941447	0.49529	-1.40809693	0.26179	-3.330155598	0.01671
P11-1029F8	-1.075751374	0.44175	-1.293951297	0.35725	-3.147317873	0.03811
P11-10K17	-1.217871146	0.34801	-0.993093379	0.42592	-2.964804796	0.0455
P11-129B9	2.335979911	0.06745	2.177669079	0.08793	2.900717232	0.02012
P11-159H3	-0.713160732	0.51184	-0.022416727	0.98208	-2.811995869	0.03664
P11-168J18	2.535904246	0.06922	2.352278963	0.09197	3.090652616	0.02285
P11-256K7	0.77414294	0.68954	1.341801918	0.48533	3.444703623	0.04591
P11-302B13	0.049213558	0.9767	-0.3770923	0.8235	-4.647171152	0.01661
P11-307E17	-1.920727453	0.18674	-1.518871707	0.26358	-3.438063808	0.02062
RP11-30B1.	0.154845553	0.83544	-0.55896896	0.47218	-1.828437399	0.04684
P11-365F18	-2.405531576	0.08662	-0.099475955	0.92871	-3.173500488	0.02379
P11-382D8	-0.355990268	0.71813	-1.022130037	0.32856	-2.896831302	0.02673
P11-390F4	0.204321178	0.63608	-0.492159725	0.28731	1.107735393	0.00505
P11-430L17	0.883910315	0.18812	-0.14302734	0.84618	1.532423253	0.01632
P11-443F16	-1.483008272	0.33512	-2.407124456	0.14468	-3.65369794	0.02782
P11-452K12	-2.26958144	0.11147	-0.712168019	0.56173	-3.03755034	0.03316
P11-475J5	0.410782525	0.66498	0.47235906	0.61106	1.715702445	0.04791
P11-561B11	0.774143323	0.64158	2.634701355	0.07181	3.227524101	0.02345
P11-569G9	1.159628792	0.2026	0.822582384	0.37249	2.21443683	0.00938
P11-641C17	-2.252976951	0.13875	-2.433391465	0.10982	-3.020945745	0.04713
P11-697N18	0.056175376	0.91653	-0.752097572	0.19479	-2.214706902	0.00474
RP11-74D7.	-0.261460351	0.73652	-1.517903761	0.09355	-2.189985153	0.03866
P11-790I12	0.102714887	0.95103	1.074119637	0.47944	2.824356049	0.04583
RP3-477O4.	-0.476153414	0.68686	-0.293201577	0.80654	-3.751681115	0.01158
RPL29P2	-0.534926209	0.53897	-0.645529355	0.45506	-3.495528305	0.00533
RPL31P49	-0.431098436	0.61169	0.001272273	0.99873	-2.550580376	0.0332
RPL37AP8	-0.938238988	0.25159	-1.448260178	0.08826	-2.41181204	0.01637
RPL7P25	0.251572263	0.73532	-0.033962139	0.96394	-2.152486388	0.04169
RPL7P4	-1.190040736	0.05394	0.356800185	0.47351	-1.233201309	0.04575

RPS12P28	-0.518986015	0.70143	0.040196126	0.97543	-3.710999071	0.02017
RPS15AP2	0.524296775	0.62127	0.95248785	0.35889	-3.151285019	0.02687
RPS15P5	-1.852400047	0.14722	0.234205378	0.82035	-3.141397333	0.02459
RXFP1	1.479206642	0.08029	0.420415407	0.6107	1.94573495	0.01665
SMCO2	0.532671149	0.4942	0.629991249	0.40735	1.464246185	0.0423
ST13P20	-0.65614425	0.54217	-0.736184827	0.48884	-3.416797763	0.01024
SYCE2	-1.231069277	0.05973	-0.618538614	0.32475	-1.520708686	0.02129
TMEM233	0.901625154	0.30126	0.25007462	0.78314	1.692908793	0.04113
MPRSS11	-0.068460121	0.95199	0.878537191	0.41483	2.610719184	0.01249
MPRSS11	0.71713786	0.45043	0.622082036	0.51477	2.486666629	0.00978
TNRC18P1	-0.042909472	0.9307	-0.954486791	0.07481	0.907980942	0.04791
TOMM20L	-1.103347086	0.2215	-0.955864867	0.25407	-2.293728232	0.02619
VWF	-1.050695793	0.25315	-0.358865202	0.69334	-1.963945187	0.04129
XCL1	-1.113085397	0.11687	-0.123615776	0.85	-2.507834689	0.00303
ac-B444P2	-0.910763756	0.17823	-0.743668528	0.2537	-1.51652429	0.0375
ZPBP2	-0.564399105	0.43854	-1.142241532	0.1328	-1.81663832	0.03255
