# Supplementary material for: Comparative transcriptional profiling of the early host response to infection by typhoidal and non-typhoidal Salmonella serovars in human intestinal organoids
Source: PLoS Pathog. 2021 Oct 20;17(10):e1009987. doi: 10.1371/journal.ppat.1009987 (PMC8570492; doi:10.1371/journal.ppat.1009987)
Supplement: S3 Table — Significantly upregulated DEGs from STM, SE or ST-infected HIOs at 2.5h were subjected to Reactome pathway analysis. (PDF) [file ppat.1009987.s011.pdf]

Table S3: Reactome Pathways Upregulated 2.5h pi

Description	STM		SE		ST	
	gene ratio	p-value	gene ratio	p-value	gene ratio	p-value
Cytokine Signaling in Immune system	0.2194767	4.65E-28	0.2063953	1.81E-16	0.1802326	1.32E-10
Signaling by Interleukins	0.2359307	7.40E-23	0.2121212	2.44E-12	0.2012987	8.93E-11
Toll Like Receptor 3 (TLR3) Cascade	0.4141414	1.68E-18	0.3030303	4.04E-08	0.2525253	1.58E-05
MyD88-independent TLR4 cascade	0.41	2.62E-18	0.3	5.21E-08	0.26	6.08E-06
TRIF(TICAM1)-mediated TLR4 signaling	0.41	2.62E-18	0.3	5.21E-08	0.26	6.08E-06
Toll Like Receptor 4 (TLR4) Cascade	0.3384615	7.70E-16	0.2615385	2.80E-07	0.2384615	5.73E-06
TRAF6 mediated induction of NFkB and MAP kinases upon TLR7/8 or 9 activation	0.3870968	2.74E-15	0.2795699	1.80E-06	0.2365591	0.00014
Toll Like Receptor 7/8 (TLR7/8) Cascade	0.3829787	4.08E-15	0.2765957	2.24E-06	0.2340426	0.00017
MyD88 dependent cascade initiated on endosome	0.3829787	4.08E-15	0.2765957	2.24E-06	0.2340426	0.00017
Toll Like Receptor 9 (TLR9) Cascade	0.3673469	1.87E-14	0.2755102	1.56E-06	0.2244898	0.00032
MyD88:MAL(TIRAP) cascade initiated on plasma membrane	0.3684211	3.92E-14	0.2631579	9.23E-06	0.2315789	0.0002
Toll Like Receptor TLR6:TLR2 Cascade	0.3684211	3.92E-14	0.2631579	9.23E-06	0.2315789	0.0002
Toll-like Receptor Cascades	0.2967742	4.54E-14	0.2387097	9.99E-07	0.2064516	8.89E-05
Toll Like Receptor TLR1:TLR2 Cascade	0.3571429	1.16E-13	0.255102	1.66E-05	0.2244898	0.00032
Toll Like Receptor 2 (TLR2) Cascade	0.3571429	1.16E-13	0.255102	1.66E-05	0.2244898	0.00032
Toll Like Receptor 10 (TLR10) Cascade	0.3764706	2.44E-13	0.2705882	1.27E-05	0.2352941	0.00031
Toll Like Receptor 5 (TLR5) Cascade	0.3764706	2.44E-13	0.2705882	1.27E-05	0.2352941	0.00031
MyD88 cascade initiated on plasma membrane	0.3764706	2.44E-13	0.2705882	1.27E-05	0.2352941	0.00031
Interleukin-17 signaling	0.3888889	3.04E-12	0.2638889	0.0001	0.2361111	0.00081
MAP kinase activation	0.40625	5.56E-12	0.265625	0.00022	0.234375	0.00175
RAF-independent MAPK1/3 activation	0.6086957	5.61E-10	0.4782609	6.26E-06	0.3478261	0.00146
Interleukin-10 signaling	0.4255319	5.90E-10	0.4042553	7.71E-08	0.3617021	1.96E-06
Death Receptor Signalling	0.2624113	7.12E-10	0.248227	7.31E-07	0.177305	0.00464
TICAM1-dependent activation of IRF3/IRF7	0.8333333	1.45E-09	0.5	0.00067	0.3333333	0.0281
Interleukin-4 and Interleukin-13 signaling	0.287037	1.62E-09	0.287037	9.68E-08	0.2592593	2.86E-06
Interleukin-1 family signaling	0.2446043	2.42E-08	0.1726619	0.00901	0.1942446	0.00084
DDX58/IFIH1-mediated induction of interferon-alpha/beta	0.3076923	2.55E-08	0.2307692	0.00091	0.2307692	0.00077
TAK1 activates NFkB by phosphorylation and activation of IKKs complex	0.4545455	2.88E-08	0.2424242	0.01771	0.2727273	0.00493
Interleukin-1 signaling	0.2718447	3.69E-08	0.1747573	0.0194	0.184466	0.00826
Signaling by the B Cell Receptor (BCR)	0.2589286	6.83E-08	0.1875	0.00547	0.1785714	0.00982

MAPK family signaling cascades	0.1843003	1.01E-07	0.1501706	0.00819	0.1501706	0.00645
Interferon Signaling	0.2081218	1.24E-07	0.2081218	1.09E-05	0.1522843	0.01825
p75NTR signals via NF-kB	0.625	1.27E-07	0.5	8.17E-05	0.375	0.00378
NF-kB is activated and signals survival	0.6923077	1.54E-07	0.5384615	0.00013	0.3846154	0.00726
Ovarian tumor domain proteases	0.3947368	2.82E-07	0.2631579	0.00453	0.2631579	0.00411
ZBP1(DAI) mediated induction of type I IFNs	0.5238095	3.47E-07	0.4285714	0.00013	0.4285714	0.00012
Negative regulators of DDX58/IFIH1 signaling	0.4117647	3.80E-07	0.2941176	0.00185	0.2647059	0.00611
Activation of IRF3/IRF7 mediated by TBK1/IKK epsilon	0.5555556	5.87E-07	0.2777778	0.03303	0.2222222	0.10631
Negative regulation of MAPK pathway	0.375	6.21E-07	0.175	0.11741	0.15	0.22485
Downstream signaling events of B Cell Receptor (BCR)	0.2650602	1.69E-06	0.1686747	0.04726	0.1807229	0.0211
TICAM1, RIP1-mediated IKK complex recruitment	0.5	2.10E-06	0.3	0.01364	0.25	0.04779
Nucleotide-binding domain, leucine rich repeat containing receptor (NLR) signaling pathways	0.3090909	2.59E-06	0.2363636	0.00361	0.2545455	0.00105
Neutrophil degranulation	0.1503132	3.26E-06	0.1085595	0.40075	0.1189979	0.13426
RIP-mediated NFkB activation via ZBP1	0.5294118	3.77E-06	0.4705882	0.00014	0.5294118	1.41E-05
TICAM1,TRAF6-dependent induction of TAK1 complex	0.5294118	3.77E-06	0.1764706	0.25847	0.2352941	0.08965
IRAK2 mediated activation of TAK1 complex upon TLR7/8 or 9 stimulation	0.5294118	3.77E-06	0.1764706	0.25847	0.2352941	0.08965
Adaptive Immune System	0.1349206	4.86E-06	0.1455026	0.00014	0.1269841	0.01573
NOD1/2 Signaling Pathway	0.3611111	5.73E-06	0.2222222	0.02922	0.25	0.00909
Regulation of necroptotic cell death	0.5	6.96E-06	0.2777778	0.03303	0.1666667	0.28082
TRAF6-mediated induction of TAK1 complex within TLR4 complex	0.5	6.96E-06	0.1666667	0.28807	0.2222222	0.10631
p75 NTR receptor-mediated signalling	0.2371134	7.76E-06	0.2371134	0.00012	0.1649485	0.03832
SMAD2/SMAD3:SMAD4 heterotrimer regulates transcription	0.375	8.37E-06	0.15625	0.23709	0.15625	0.22848
Interleukin-6 signaling	0.6363636	9.43E-06	0.5454545	0.00037	0.5454545	0.00034
Regulated proteolysis of p75NTR	0.6363636	9.43E-06	0.3636364	0.02133	0.1818182	0.315
Transcriptional activity of SMAD2/SMAD3:SMAD4 heterotrimer	0.3181818	1.36E-05	0.1818182	0.0822	0.2045455	0.03261
PIP3 activates AKT signaling	0.1692308	1.54E-05	0.1576923	0.00453	0.1807692	8.13E-05
activated TAK1 mediates p38 MAPK activation	0.4166667	1.61E-05	0.1666667	0.23679	0.1666667	0.22925
Diseases of Immune System	0.4166667	1.61E-05	0.375	0.00043	0.375	0.00039
Diseases associated with the TLR signaling cascade	0.4166667	1.61E-05	0.375	0.00043	0.375	0.00039
Interleukin-6 family signaling	0.4166667	1.61E-05	0.375	0.00043	0.375	0.00039

TRAF6 mediated NF-kB activation	0.4166667	1.61E-05	0.3333333	0.00218	0.375	0.00039
Interferon alpha/beta signaling	0.2608696	1.87E-05	0.1884058	0.02461	0.1594203	0.09335
Signaling by TGF-beta family members	0.2254902	1.87E-05	0.1764706	0.01768	0.1568627	0.05676
RIPK1-mediated regulated necrosis	0.45	2.05E-05	0.25	0.05025	0.15	0.33962
Regulated Necrosis	0.45	2.05E-05	0.25	0.05025	0.15	0.33962
NRIF signals cell death from the nucleus	0.5	2.31E-05	0.25	0.07746	0.125	0.5012
MAP3K8 (TPL2)-dependent MAPK1/3 activation	0.5	2.31E-05	0.25	0.07746	0.1875	0.22313
IKK complex recruitment mediated by RIP1	0.4	2.48E-05	0.2	0.11223	0.16	0.25247
Interleukin-12 family signaling	0.2758621	2.54E-05	0.2586207	0.00068	0.1551724	0.13737
TNFR1-induced NFkappaB signaling pathway	0.3666667	2.56E-05	0.2333333	0.03149	0.2666667	0.00911
MAPK targets/ Nuclear events mediated by MAP kinases	0.3548387	3.66E-05	0.3548387	0.00018	0.2580645	0.0112
ERKs are inactivated	0.5384615	4.19E-05	0.2307692	0.14694	0.1538462	0.39285
p75NTR recruits signalling complexes	0.5384615	4.19E-05	0.3846154	0.0077	0.2307692	0.14256
MAPK1/MAPK3 signaling	0.1653543	4.20E-05	0.1377953	0.05225	0.1299213	0.09456
CLEC7A (Dectin-1) signaling	0.2178218	4.98E-05	0.1485149	0.10122	0.1584158	0.05266
TNF signaling	0.3023256	5.08E-05	0.2093023	0.03076	0.1860465	0.06907
IRAK2 mediated activation of TAK1 complex	0.6	6.92E-05	0.1	0.66787	0.2	0.27514
Regulation of TNFR1 signaling	0.3333333	7.10E-05	0.2727273	0.0054	0.2121212	0.04729
Cytosolic sensors of pathogen-associated DNA	0.2539683	7.66E-05	0.2222222	0.00469	0.2222222	0.00414
Membrane binding and targetting of GAG proteins	0.5	7.74E-05	NA	NA	0.0714286	0.78155
Synthesis And Processing Of GAG, GAGPOL Polyproteins	0.5	7.74E-05	NA	NA	0.0714286	0.78155
Downregulation of SMAD2/3:SMAD4 transcriptional activity	0.3913043	7.81E-05	0.2608696	0.02712	0.2608696	0.02553
JNK (c-Jun kinases) phosphorylation and activation mediated by activated human TAK1	0.3913043	7.81E-05	0.0869565	0.70843	0.173913	0.20661
Circadian Clock	0.2428571	8.48E-05	0.2428571	0.00067	0.2857143	1.58E-05
Fc epsilon receptor (FCERI) signaling	0.1940299	8.71E-05	0.1343284	0.15785	0.1641791	0.01813
Signaling by Receptor Tyrosine Kinases	0.1406593	9.20E-05	0.1582418	0.00018	0.1450549	0.00238
Budding and maturation of HIV virion	0.3448276	0.00011	0.1034483	0.59534	0.0689655	0.81482
Downstream signal transduction	0.3448276	0.00011	0.2758621	0.00795	0.3793103	7.82E-05
Interferon gamma signaling	0.2173913	0.00011	0.1956522	0.00618	0.1956522	0.00536
Diseases of signal transduction	0.1455026	0.00012	0.1269841	0.08603	0.1481481	0.00312
IRAK1 recruits IKK complex	0.4666667	0.00013	0.2	0.20085	0.1333333	0.46644
IRAK1 recruits IKK complex upon TLR7/8 or 9 stimulation	0.4666667	0.00013	0.2	0.20085	0.1333333	0.46644
Intracellular signaling by second messengers	0.1535836	0.00014	0.1569966	0.00299	0.1740614	0.00011

Interleukin-27 signaling	0.5454545	0.00014	0.5454545	0.00037	0.2727273	0.09574
Signaling by TGF-beta Receptor Complex	0.2328767	0.00015	0.1780822	0.03736	0.1780822	0.03394
Transcriptional Regulation by TP53	0.1452055	0.00016	0.1616438	0.00039	0.1315068	0.04413
Nuclear Events (kinase and transcription factor activation)	0.36	0.00017	0.32	0.00292	0.24	0.03755
Activation of NF-kappaB in B cells	0.238806	0.00017	0.1492537	0.15627	0.1641791	0.07911
TNFR2 non-canonical NF-kB pathway	0.2058824	0.00017	0.1470588	0.10785	0.1176471	0.35696
Interleukin-3, Interleukin-5 and GM-CSF signaling	0.2708333	0.00018	0.1458333	0.23014	0.2708333	0.00084
Programmed Cell Death	0.1744186	0.0002	0.127907	0.18356	0.1162791	0.31516
MAPK6/MAPK4 signaling	0.2134831	0.00021	0.1123596	0.45212	0.1573034	0.07009
FCERI mediated NF-kB activation	0.2195122	0.00021	0.1097561	0.48877	0.1341463	0.2195
Assembly Of The HIV Virion	0.4375	0.00022	NA	NA	0.0625	0.82425
Regulation of TP53 Expression and Degradation	0.2972973	0.00023	0.2162162	0.03399	0.2162162	0.03164
RAF/MAP kinase cascade	0.1572581	0.00023	0.1330645	0.08498	0.125	0.14643
TP53 Regulates Transcription of Death Receptors and Ligands	0.5	0.00026	0.4166667	0.00518	0.1666667	0.35435
Interleukin-35 Signalling	0.5	0.00026	0.5	0.00067	0.25	0.11828
TGF-beta receptor signaling activates SMADs	0.3125	0.00028	0.21875	0.04342	0.21875	0.04074
TP53 Regulates Transcription of Cell Death Genes	0.2727273	0.00029	0.2272727	0.01341	0.2045455	0.03261
Apoptosis	0.1715976	0.00033	0.1242604	0.22859	0.112426	0.37705
IL-6-type cytokine receptor ligand interactions	0.4117647	0.00035	0.4117647	0.001	0.2941176	0.02467
Signaling by MET	0.2151899	0.00041	0.1392405	0.19753	0.1518987	0.10873
Signaling by Activin	0.4615385	0.00045	0.3076923	0.03906	0.1538462	0.39285
Chemokine receptors bind chemokines	0.25	0.00069	0.2083333	0.02418	0.2291667	0.00826
Interleukin-12 signaling	0.25	0.00069	0.2291667	0.00913	0.125	0.37253
Regulation of IFNG signaling	0.4285714	0.00073	0.4285714	0.00182	0.4285714	0.0017
TRAF3-dependent IRF activation pathway	0.4285714	0.00073	0.3571429	0.01096	0.5	0.00021
ER-Phagosome pathway	0.2048193	0.00074	0.060241	0.94299	0.0843373	0.76367
RHO GTPases activate IQGAPs	0.3	0.00078	0.2666667	0.00988	0.1333333	0.37311
Regulation of TP53 Degradation	0.2777778	0.00079	0.1944444	0.07509	0.1944444	0.07079
Cell junction organization	0.1978022	0.00081	0.1868132	0.01214	0.0989011	0.60135
Transcriptional regulation of white adipocyte differentiation	0.202381	0.00085	0.1904762	0.01227	0.3690476	6.93E-11
Antigen processing-Cross presentation	0.1919192	0.00086	0.0707071	0.90303	0.0808081	0.81202
MAPK3 (ERK1) activation	0.5	0.00089	0.4	0.01477	0.3	0.07518
Interleukin-20 family signaling	0.32	0.00095	0.36	0.00061	0.28	0.01093

Antiviral mechanism by IFN-stimulated genes	0.2051282	0.00103	0.2051282	0.00597	0.1025641	0.559
PTEN Regulation	0.1714286	0.00106	0.1642857	0.01836	0.1785714	0.00421
TRAF6 mediated IRF7 activation in TLR7/8 or 9 signaling	0.4	0.00112	0.0666667	0.80867	NA	NA
Intrinsic Pathway for Apoptosis	0.25	0.00115	0.25	0.00456	0.1818182	0.07717
Downregulation of TGF-beta receptor signaling	0.3076923	0.00127	0.2307692	0.04734	0.1923077	0.12226
Signaling by PDGF	0.2241379	0.00127	0.137931	0.25499	0.2241379	0.00521
C-type lectin receptors (CLRs)	0.1690141	0.0013	0.1267606	0.22399	0.1619718	0.01853
Signaling by ERBB4	0.2444444	0.0014	0.1333333	0.32724	0.1777778	0.08582
Signaling by Leptin	0.4545455	0.00152	0.4545455	0.0033	0.5454545	0.00034
NOTCH4 Activation and Transmission of Signal to the Nucleus	0.4545455	0.00152	0.2727273	0.09889	NA	NA
Spry regulation of FGF signaling	0.375	0.00167	NA	NA	0.3125	0.01899
ERK/MAPK targets	0.3181818	0.00206	0.2727273	0.02193	0.1818182	0.18466
NOTCH2 Activation and Transmission of Signal to the Nucleus	0.3181818	0.00206	0.1818182	0.19112	NA	NA
Regulation of signaling by CBL	0.3181818	0.00206	0.1363636	0.4069	0.2272727	0.06849
Signaling by PTK6	0.2222222	0.00209	0.1666667	0.10485	0.1481481	0.18688
Signaling by Non-Receptor Tyrosine Kinases	0.2222222	0.00209	0.1666667	0.10485	0.1481481	0.18688
Membrane Trafficking	0.1210191	0.00219	0.1066879	0.44111	0.1130573	0.21125
Cell death signalling via NRAGE, NRIF and NADE	0.1973684	0.00219	0.2105263	0.00459	0.1184211	0.38071
Caspase activation via Death Receptors in the presence of ligand	0.3529412	0.00238	0.2352941	0.09328	0.1176471	0.53446
ISG15 antiviral mechanism	0.1971831	0.00306	0.2112676	0.00575	0.0985915	0.60554
TNF receptor superfamily (TNFSF) members mediating non-canonical NF-kB pathway	0.3333333	0.00331	0.3333333	0.00783	0.2777778	0.03134
TCR signaling	0.1680672	0.0034	0.1008403	0.59437	0.1344538	0.1609
Regulation of lipid metabolism by Peroxisome proliferator-activated receptor alpha (PPARalpha)	0.1680672	0.0034	0.1260504	0.257	0.2521008	2.40E-06
Activation of BH3-only proteins	0.2666667	0.00347	0.3	0.00267	0.2	0.08142
Cellular responses to stress	0.1267606	0.00357	0.1361502	0.02	0.0985915	0.64168
Signaling by Erythropoietin	0.2916667	0.00358	0.25	0.03307	0.2916667	0.00862
Growth hormone receptor signaling	0.2916667	0.00358	0.2916667	0.00928	0.25	0.03116
Dissolution of Fibrin Clot	0.3846154	0.00364	0.2307692	0.14694	0.1538462	0.39285
Cell-Cell communication	0.1627907	0.00403	0.1627907	0.02575	0.1162791	0.34824
Activated NOTCH1 Transmits Signal to the Nucleus	0.2580645	0.00433	0.1935484	0.09747	0.0322581	0.96565
Negative regulation of FGFR4 signaling	0.2580645	0.00433	0.0645161	0.84888	0.2580645	0.0112

NOTCH3 Activation and Transmission of Signal to the Nucleus	0.28	0.0046	0.16	0.26052	0.08	0.74422
Antigen Presentation: Folding, assembly and peptide loading of class I MHC	0.28	0.0046	0.12	0.49212	0.12	0.48262
Vesicle-mediated transport	0.1169415	0.00477	0.107946	0.39546	0.1094453	0.30144
Downstream TCR signaling	0.1734694	0.00478	0.0918367	0.70611	0.1326531	0.20523
Endosomal Sorting Complex Required For Transport (ESCRT)	0.25	0.00533	0.03125	0.97072	0.03125	0.9692
Gene and protein expression by JAK-STAT signaling after Interleukin-12 stimulation	0.2307692	0.00565	0.2051282	0.04506	0.0769231	0.78009
PPARA activates gene expression	0.1623932	0.00621	0.1196581	0.33483	0.2393162	1.49E-05
Regulation of TP53 Activity	0.15	0.00642	0.16875	0.00796	0.15625	0.02203
Signaling by NOTCH2	0.2424242	0.00651	0.1818182	0.12319	0.0909091	0.67448
Oncogene Induced Senescence	0.2424242	0.00651	0.1818182	0.12319	0.1515152	0.24833
Negative regulation of FGFR1 signaling	0.2424242	0.00651	0.1212121	0.4563	0.2424242	0.0164
Nuclear signaling by ERBB4	0.2592593	0.00728	0.1481481	0.30913	0.2222222	0.05268
Caspase activation via extrinsic apoptotic signalling pathway	0.2592593	0.00728	0.1481481	0.30913	0.1481481	0.30015
Signaling by NOTCH1 HD Domain Mutants in Cancer	0.3333333	0.00732	0.2666667	0.06311	NA	NA
Constitutive Signaling by NOTCH1 HD Domain Mutants	0.3333333	0.00732	0.2666667	0.06311	NA	NA
Regulation of innate immune responses to cytosolic DNA	0.3333333	0.00732	0.1333333	0.47391	0.1333333	0.46644
Negative regulation of MET activity	0.2857143	0.00772	0.0952381	0.65947	0.1428571	0.36897
Signaling by FGFR4	0.2195122	0.00797	0.097561	0.63195	0.2439024	0.00733
MASTL Facilitates Mitotic Progression	0.4	0.00804	0.1	0.66787	0.1	0.66256
Interleukin-21 signaling	0.4	0.00804	0.3	0.07773	0.3	0.07518
Cellular responses to external stimuli	0.1192843	0.00836	0.1292247	0.03902	0.0874751	0.8949
Rho GTPase cycle	0.1521739	0.00882	0.1956522	0.00092	0.1449275	0.07236
RAB geranylgeranylation	0.1875	0.00891	0.0625	0.9126	0.0625	0.90694
Transcriptional regulation by RUNX3	0.1666667	0.00898	0.1458333	0.1234	0.1979167	0.00376
Pink/Parkin Mediated Mitophagy	0.2727273	0.00984	0.0454545	0.91164	0.0909091	0.6771
TGF-beta receptor signaling in EMT (epithelial to mesenchymal transition)	0.3125	0.00989	0.1875	0.22932	0.125	0.5012
Glycogen storage diseases	0.3125	0.00989	NA	NA	0.1875	0.22313
Signaling by NOTCH1	0.1780822	0.01025	0.2054795	0.00751	0.1506849	0.12617
Signaling by FGFR1	0.2	0.0104	0.14	0.2628	0.2	0.02891
Mitophagy	0.2413793	0.01097	0.0689655	0.82106	0.1034483	0.58559
Negative regulation of FGFR3 signaling	0.2413793	0.01097	0.0344828	0.95922	0.2758621	0.00733

TRAF6 mediated IRF7 activation	0.2413793	0.01097	0.1724138	0.17922	0.2068966	0.07103
Signaling by NOTCH1 PEST Domain Mutants in Cancer	0.1896552	0.01103	0.1724138	0.07543	0.1551724	0.13737
Signaling by NOTCH1 in Cancer	0.1896552	0.01103	0.1724138	0.07543	0.1551724	0.13737
Constitutive Signaling by NOTCH1 PEST Domain Mutants	0.1896552	0.01103	0.1724138	0.07543	0.1551724	0.13737
Signaling by NOTCH1 HD+PEST Domain Mutants in Cancer	0.1896552	0.01103	0.1724138	0.07543	0.1551724	0.13737
Constitutive Signaling by NOTCH1 HD+PEST Domain Mutants	0.1896552	0.01103	0.1724138	0.07543	0.1551724	0.13737
RAB GEFs exchange GTP for GDP on RABs	0.1666667	0.01119	0.1444444	0.14137	0.1222222	0.31977
Rab regulation of trafficking	0.1532258	0.0115	0.1370968	0.14645	0.1290323	0.20369
Endosomal/Vacuolar pathway	0.3636364	0.01176	0.0909091	0.70255	0.0909091	0.69731
Regulation by c-FLIP	0.3636364	0.01176	0.2727273	0.09889	0.0909091	0.69731
Myoclonic epilepsy of Lafora	0.3636364	0.01176	NA	NA	0.1818182	0.315
Type I hemidesmosome assembly	0.3636364	0.01176	0.4545455	0.0033	0.2727273	0.09574
SUMOylation of immune response proteins	0.3636364	0.01176	0.4545455	0.0033	0.3636364	0.02036
CASP8 activity is inhibited	0.3636364	0.01176	0.2727273	0.09889	0.0909091	0.69731
Dimerization of procaspase-8	0.3636364	0.01176	0.2727273	0.09889	0.0909091	0.69731
VEGFA-VEGFR2 Pathway	0.1616162	0.01198	0.1818182	0.0132	0.1212121	0.3188
Cyclin D associated events in G1	0.2045455	0.01274	0.1136364	0.4912	0.0909091	0.67675
G1 Phase	0.2045455	0.01274	0.1136364	0.4912	0.0909091	0.67675
Transport of connexons to the plasma membrane	0.2941176	0.01302	0.4117647	0.001	0.1176471	0.53446
Regulation of MECP2 expression and activity	0.2333333	0.01325	0.2	0.08579	0.3333333	0.00056
Dectin-1 mediated noncanonical NF-kB signaling	0.1833333	0.01415	0.1	0.60624	0.1	0.59234
HSP90 chaperone cycle for steroid hormone receptors (SHR)	0.1886792	0.01556	0.245283	0.00255	0.0566038	0.92069
Regulation of PTEN gene transcription	0.1803279	0.01594	0.2459016	0.00119	0.2131148	0.00813
Josephin domain DUBs	0.3333333	0.01644	0.0833333	0.73361	NA	NA
NF-kB activation through FADD/RIP-1 pathway mediated by caspase-8 and -10	0.3333333	0.01644	0.3333333	0.02941	0.4166667	0.00488
Signaling by NTRK1 (TRKA)	0.1666667	0.01748	0.2435897	0.00032	0.1794872	0.02674
RAF activation	0.24	0.01863	0.04	0.93656	0.16	0.25247
Constitutive Signaling by AKT1 E17K in Cancer	0.24	0.01863	0.24	0.0398	0.16	0.25247
Antigen activates B Cell Receptor (BCR) leading to generation of second messengers	0.21875	0.0188	0.21875	0.04342	0.15625	0.22848
Gap junction trafficking and regulation	0.1914894	0.01935	0.2978723	0.00021	0.106383	0.53888
Regulation of mRNA stability by proteins that bind AU-rich elements	0.1590909	0.02048	0.1022727	0.57691	0.1363636	0.19085

Signaling by FGFR3	0.2	0.02092	0.075	0.80253	0.25	0.00609
Signaling by moderate kinase activity BRAF mutants	0.2	0.02092	0.1	0.61206	0.1	0.60078
Paradoxical activation of RAF signaling by kinase inactive BRAF	0.2	0.02092	0.1	0.61206	0.1	0.60078
Constitutive Signaling by Ligand-Responsive EGFR Cancer Variants	0.2631579	0.02114	0.1052632	0.60413	0.1052632	0.59628
Signaling by EGFR in Cancer	0.2631579	0.02114	0.1052632	0.60413	0.1052632	0.59628
Signaling by Ligand-Responsive EGFR Variants in Cancer	0.2631579	0.02114	0.1052632	0.60413	0.1052632	0.59628
LDL clearance	0.2631579	0.02114	0.2105263	0.129	0.2631579	0.03904
PI3K/AKT Signaling in Cancer	0.1546392	0.02138	0.1752577	0.02209	0.1752577	0.01957
Hemostasis	0.1112903	0.0221	0.1048387	0.50336	0.1064516	0.40247
Initiation of Nuclear Envelope Reformation	0.3076923	0.02213	0.1538462	0.39976	0.1538462	0.39285
Nuclear Envelope Reassembly	0.3076923	0.02213	0.1538462	0.39976	0.1538462	0.39285
Triglyceride biosynthesis	0.3076923	0.02213	0.0769231	0.76143	0.0769231	0.75646
Erythropoietin activates RAS	0.3076923	0.02213	0.2307692	0.14694	0.3076923	0.03737
Cell-cell junction organization	0.171875	0.02233	0.140625	0.21991	0.09375	0.65628
Transcriptional activation of mitochondrial biogenesis	0.1785714	0.02242	0.1607143	0.12446	0.3035714	2.86E-05
Other interleukin signaling	0.2307692	0.02248	0.2692308	0.01467	0.3076923	0.00352
Signaling by VEGF	0.1495327	0.02389	0.1962617	0.00313	0.1121495	0.42185
Degradation of beta-catenin by the destruction complex	0.1585366	0.02563	0.0853659	0.76532	0.097561	0.61732
Signaling by NOTCH4	0.1585366	0.02563	0.1219512	0.35037	0.0853659	0.75251
Negative regulation of FGFR2 signaling	0.2058824	0.02581	0.0882353	0.70359	0.2647059	0.00611
Signaling by Hippo	0.25	0.0262	0.2	0.14869	0.15	0.33962
TP53 Regulates Transcription of Genes Involved in Cytochrome C Release	0.25	0.0262	0.35	0.00301	0.3	0.01279
Listeria monocytogenes entry into host cells	0.25	0.0262	0.05	0.88981	0.15	0.33962
Deactivation of the beta-catenin transactivating complex	0.1904762	0.02751	0.1904762	0.06568	0.1428571	0.2602
Beta-catenin independent WNT signaling	0.137931	0.02812	0.1103448	0.44551	0.1241379	0.23337
Deubiquitination	0.1212121	0.02878	0.1144781	0.30754	0.1414141	0.02031
SEMA3A-Plexin repulsion signaling by inhibiting Integrin adhesion	0.2857143	0.02888	0.2142857	0.1733	0.3571429	0.01035
TP53 regulates transcription of several additional cell death genes whose specific roles in p53-dependent apoptosis remain uncertain	0.2857143	0.02888	0.2142857	0.1733	0.2142857	0.1683

Interleukin-15 signaling	0.2857143	0.02888	0.2857143	0.05029	0.3571429	0.01035
EPH-Ephrin signaling	0.1521739	0.02903	0.2282609	0.00041	0.1086957	0.47733
The role of GTSE1 in G2/M progression after G2 checkpoint	0.16	0.02931	0.0933333	0.67836	0.0666667	0.89673
NIK-->noncanonical NF-kB signaling	0.1694915	0.03123	0.0847458	0.75093	0.0847458	0.73976
Signaling by SCF-KIT	0.1860465	0.0313	0.1627907	0.15573	0.3255814	6.12E-05
Sphingolipid de novo biosynthesis	0.1860465	0.0313	0.1627907	0.15573	0.2093023	0.02844
EPH-ephrin mediated repulsion of cells	0.1764706	0.03161	0.254902	0.00175	0.1372549	0.26799
CD209 (DC-SIGN) signaling	0.2380952	0.03198	0.2380952	0.0605	0.3809524	0.00073
TP53 regulates transcription of additional cell cycle genes whose exact role in the p53 pathway remain uncertain	0.2380952	0.03198	0.2380952	0.0605	0.2857143	0.01638
Interleukin-37 signaling	0.2380952	0.03198	0.2380952	0.0605	0.2857143	0.01638
G2/M Transition	0.128866	0.03277	0.1082474	0.46328	0.0721649	0.9444
RHO GTPases Activate WASPs and WAVEs	0.1944444	0.03441	0.1111111	0.52624	0.0555556	0.89752
Cellular Senescence	0.1282051	0.03458	0.1076923	0.47314	0.1076923	0.44692
Interleukin-2 family signaling	0.1818182	0.03542	0.1590909	0.16964	0.2272727	0.01224
TP53 Regulates Metabolic Genes	0.1511628	0.03627	0.1744186	0.03152	0.0930233	0.6713
Mitotic G2-G2/M phases	0.127551	0.03648	0.1071429	0.48299	0.0714286	0.94934
Activation of BAD and translocation to mitochondria	0.2666667	0.03671	0.2666667	0.06311	0.1333333	0.46644
YAP1- and WWTR1 (TAZ)-stimulated gene expression	0.2666667	0.03671	0.2	0.20085	0.2666667	0.06052
phosphorylation site mutants of CTNNB1 are not targeted to the proteasome by the destruction complex	0.2666667	0.03671	NA	NA	0.0666667	0.80406
Misspliced GSK3beta mutants stabilize beta-catenin	0.2666667	0.03671	NA	NA	0.0666667	0.80406
S33 mutants of beta-catenin aren't phosphorylated	0.2666667	0.03671	NA	NA	0.0666667	0.80406
S37 mutants of beta-catenin aren't phosphorylated	0.2666667	0.03671	NA	NA	0.0666667	0.80406
S45 mutants of beta-catenin aren't phosphorylated	0.2666667	0.03671	NA	NA	0.0666667	0.80406
T41 mutants of beta-catenin aren't phosphorylated	0.2666667	0.03671	NA	NA	0.0666667	0.80406
The NLRP3 inflammasome	0.2666667	0.03671	0.2666667	0.06311	0.3333333	0.01423
InlB-mediated entry of Listeria monocytogenes into host cell	0.2666667	0.03671	0.0666667	0.80867	0.0666667	0.80406
N-Glycan antennae elongation	0.2666667	0.03671	0.2	0.20085	0.2666667	0.06052
Signaling by NOTCH	0.1234043	0.03777	0.1361702	0.0697	0.1191489	0.2315
Signaling by RAS mutants	0.1698113	0.03942	0.0943396	0.66064	0.1320755	0.3015
Gap junction trafficking	0.1777778	0.0399	0.2888889	0.00049	0.0666667	0.85574
Synthesis of active ubiquitin: roles of E1 and E2 enzymes	0.2	0.04304	0.2	0.08579	0.1333333	0.37311
Molecules associated with elastic fibres	0.1842105	0.04472	0.2105263	0.03926	0.1052632	0.559

Microtubule-dependent trafficking of connexons from Golgi to the plasma membrane	0.25	0.04566	0.375	0.00405	0.0625	0.82425
Glycogen synthesis	0.25	0.04566	NA	NA	0.125	0.5012
Rap1 signalling	0.25	0.04566	0.1875	0.22932	0.1875	0.22313
Ub-specific processing proteases	0.1227273	0.04643	0.0954545	0.70055	0.1045455	0.50092
Transcriptional Regulation by MECP2	0.1636364	0.04844	0.1636364	0.11443	0.2181818	0.00891
Signaling by NTRKs	0.1414141	0.04986	0.2222222	0.00045	0.1717172	0.02355
COPI-mediated anterograde transport	0.1414141	0.04986	0.1212121	0.33602	0.0505051	0.97974
PI5P, PP2A and IER3 Regulate PI3K/AKT Signaling	0.1414141	0.04986	0.1313131	0.23003	0.1818182	0.01156
NOTCH1 Intracellular Domain Regulates Transcription	0.1702128	0.04994	0.212766	0.02104	0.212766	0.01928
RUNX1 interacts with co-factors whose precise effect on RUNX1 targets is not known	0.1315789	0.2375	0.3421053	7.21E-05	0.3157895	0.00028
SUMOylation	0.1229947	0.06108	0.1925134	0.0002	0.1604278	0.0091
SUMO E3 ligases SUMOylate target proteins	0.121547	0.07294	0.1933702	0.00022	0.1657459	0.00572
Axon guidance	0.0998185	0.16996	0.1488203	0.00051	0.0889292	0.88266
Extracellular matrix organization	0.1162791	0.05142	0.166113	0.00057	0.1196013	0.19008
Gap junction assembly	0.1764706	0.0726	0.2941176	0.00185	0.0588235	0.87829
p75NTR regulates axonogenesis	0.1	0.60096	0.5	0.00197	0.2	0.27514
L1CAM interactions	0.1025641	0.32913	0.1965812	0.002	0.1111111	0.42862
SUMOylation of RNA binding proteins	0.1304348	0.2129	0.2608696	0.00211	0.0869565	0.71074
NRAGE signals death through JNK	0.1186441	0.25772	0.2372881	0.00247	0.1016949	0.57541
Netrin-1 signaling	0.12	0.27262	0.24	0.00445	0.12	0.41073
Repression of WNT target genes	0.1666667	0.28434	0.4166667	0.00518	0.3333333	0.0281
Semaphorin interactions	0.1076923	0.34358	0.2153846	0.00628	0.1384615	0.22158
EPHB-mediated forward signaling	0.1666667	0.07079	0.2380952	0.00962	0.0952381	0.64007
Laminin interactions	0.1333333	0.26692	0.2666667	0.00988	0.1	0.6092
EPHA-mediated growth cone collapse	0.1	0.49705	0.2666667	0.00988	0.2	0.08142
Ephrin signaling	0.2105263	0.079	0.3157895	0.01045	0.2105263	0.12425
Sema4D in semaphorin signaling	0.16	0.17156	0.28	0.01175	0.2	0.10735
NOTCH4 Intracellular Domain Regulates Transcription	0.05	0.84092	0.3	0.01364	0.25	0.04779
Activation of the AP-1 family of transcription factors	0.2	0.21682	0.4	0.01477	0.2	0.27514
Recycling pathway of L1	0.1111111	0.36038	0.2222222	0.01567	0.1333333	0.31557
Sema4D induced cell migration and growth-cone collapse	0.1428571	0.27853	0.2857143	0.01745	0.1904762	0.16354
Phospholipase C-mediated cascade: FGFR1	0.125	0.41592	0.3125	0.02006	0.1875	0.22313

Formation of annular gap junctions	0.2727273	0.06517	0.3636364	0.02133	0.0909091	0.69731
Erythropoietin activates Phosphoinositide-3-kinase (PI3K)	0.2727273	0.06517	0.3636364	0.02133	0.2727273	0.09574
Regulation of HSF1-mediated heat shock response	0.1029412	0.38784	0.1911765	0.022	0.0735294	0.84224
SUMOylation of DNA damage response and repair proteins	0.0921053	0.50488	0.1842105	0.02417	0.1052632	0.52846
Regulation of RUNX1 Expression and Activity	0.2352941	0.0557	0.2941176	0.02603	0.4117647	0.00092
Formation of tubulin folding intermediates by CCT/TriC	0.1304348	0.32822	0.2608696	0.02712	0.0434783	0.91794
Transcriptional regulation of pluripotent stem cells	0.1111111	0.3898	0.2222222	0.02922	0.2222222	0.02717
Gap junction degradation	0.25	0.08142	0.3333333	0.02941	0.0833333	0.72849
Cooperation of Prefoldin and TriC/CCT in actin and tubulin folding	0.1	0.49705	0.2333333	0.03149	0.0333333	0.9617
G alpha (12/13) signalling events	0.1012658	0.39039	0.1772152	0.03272	0.0632911	0.91988
Pre-NOTCH Processing in Golgi	0.0555556	0.80878	0.2777778	0.03303	0.2222222	0.10631
PI3K Cascade	0.0681818	0.75466	0.2045455	0.03522	0.2045455	0.03261
SUMOylation of DNA replication proteins	0.0681818	0.75466	0.2045455	0.03522	0.0681818	0.84496
Cellular response to heat stress	0.0909091	0.51265	0.1704545	0.0378	0.0909091	0.69648
IRF3-mediated induction of type I IFN	0.1538462	0.318	0.3076923	0.03906	NA	NA
CLEC7A (Dectin-1) induces NFAT activation	0.2307692	0.0992	0.3076923	0.03906	0.2307692	0.14256
Non-integrin membrane-ECM interactions	0.1525424	0.07033	0.1864407	0.03951	0.2033898	0.01553
IRS-related events triggered by IGF1R	0.0961538	0.48479	0.1923077	0.04009	0.1923077	0.03698
Regulation of TP53 Activity through Methylation	0.2105263	0.079	0.2631579	0.0411	0.2105263	0.12425
Export of Viral Ribonucleoproteins from Nucleus	0.125	0.30749	0.21875	0.04342	0.09375	0.65366
Vpr-mediated nuclear import of PICs	0.09375	0.54148	0.21875	0.04342	0.09375	0.65366
RUNX2 regulates bone development	0.09375	0.54148	0.21875	0.04342	0.21875	0.04074
Antigen processing: Ubiquitination & Proteasome degradation	0.0906149	0.45796	0.1359223	0.0438	0.1100324	0.36508
IGF1R signaling cascade	0.0943396	0.50198	0.1886792	0.04498	0.1886792	0.04154
Prefoldin mediated transfer of substrate to CCT/TriC	0.1153846	0.40239	0.2307692	0.04734	0.0384615	0.9408
Class I MHC mediated antigen processing & presentation	0.1105121	0.07213	0.1320755	0.04853	0.1105121	0.33647
RORA activates gene expression	0.1111111	0.47715	0.1111111	0.574	0.4444444	0.00021
Chromatin modifying enzymes	0.0581818	0.97437	0.12	0.21987	0.1709091	0.00032
Chromatin organization	0.0581818	0.97437	0.12	0.21987	0.1709091	0.00032
Transport of inorganic cations/anions and amino acids/oligopeptides	0.1203704	0.15052	0.1111111	0.45431	0.212963	0.00053
Xenobiotics	0.125	0.35309	0.125	0.46433	0.3333333	0.002
Regulation of gene expression by Hypoxia-inducible Factor	0.1818182	0.25052	0.0909091	0.70255	0.4545455	0.00311

Regulation of KIT signaling	0.125	0.41592	0.125	0.50885	0.375	0.00378
BMAL1:CLOCK,NPAS2 activates circadian gene expression	0.1851852	0.0824	0.1481481	0.30913	0.2962963	0.00456
Loss of function of MECP2 in Rett syndrome	0.1538462	0.318	0.0769231	0.76143	0.3846154	0.00726
Pervasive developmental disorders	0.1538462	0.318	0.0769231	0.76143	0.3846154	0.00726
Activation of gene expression by SREBF (SREBP)	0.0952381	0.50957	0.0714286	0.82867	0.2380952	0.00876
Regulation of cholesterol biosynthesis by SREBP (SREBF)	0.1090909	0.35179	0.0909091	0.69291	0.2181818	0.00891
FRS-mediated FGFR2 signaling	0.08	0.65755	0.12	0.49212	0.28	0.01093
FGFR1 mutant receptor activation	0.1612903	0.13067	0.1612903	0.21723	0.2580645	0.0112
Chondroitin sulfate/dermatan sulfate metabolism	0.08	0.65002	0.1	0.60831	0.22	0.01129
Signaling by Insulin receptor	0.0512821	0.9202	0.1282051	0.29375	0.1923077	0.01227
PKMTs methylate histone lysines	0.056338	0.88047	0.084507	0.76402	0.1971831	0.01231
Transcriptional regulation by RUNX1	0.1129707	0.10325	0.1171548	0.28494	0.1506276	0.01231
Defective B4GALT7 causes EDS, progeroid type	0.1	0.5343	0.05	0.88981	0.3	0.01279
Defective B3GAT3 causes JDSSDHD	0.05	0.84092	0.05	0.88981	0.3	0.01279
Defective B3GALT6 causes EDSP2 and SEMDJL1	0.05	0.84092	0.05	0.88981	0.3	0.01279
FRS-mediated FGFR3 signaling	0.1	0.5343	0.05	0.88981	0.3	0.01279
Signaling by FGFR1 in disease	0.1578947	0.11132	0.1578947	0.19974	0.2368421	0.01306
A tetrasaccharide linker sequence is required for GAG synthesis	0.1153846	0.40239	0.0384615	0.94319	0.2692308	0.01366
Bicarbonate transporters	0.1	0.60096	0.1	0.66787	0.4	0.01409
Mitochondrial biogenesis	0.1157895	0.20945	0.1157895	0.40488	0.1789474	0.01613
Insulin receptor signalling cascade	0.0740741	0.70931	0.1666667	0.10485	0.2037037	0.01983
CYP2E1 reactions	NA	NA	0.0909091	0.70255	0.3636364	0.02036
HS-GAG degradation	0.0454545	0.86766	0.0454545	0.91164	0.2727273	0.02061
FRS-mediated FGFR4 signaling	0.1363636	0.30333	0.0909091	0.68473	0.2727273	0.02061
IRS-mediated signalling	0.0833333	0.61752	0.1875	0.05744	0.2083333	0.02218
Negative regulation of the PI3K/AKT network	0.1320755	0.07928	0.1226415	0.31108	0.1698113	0.02243
Phase I - Functionalization of compounds	0.0841121	0.60319	0.0747664	0.88186	0.1682243	0.02447
FRS-mediated FGFR1 signaling	0.1304348	0.32822	0.173913	0.21362	0.2608696	0.02553
Constitutive Signaling by Aberrant PI3K in Cancer	0.1267606	0.16741	0.1549296	0.1171	0.1830986	0.02764
SUMOylation of intracellular receptors	0.1333333	0.26692	0.2	0.08579	0.2333333	0.02948
Downstream signaling of activated FGFR2	0.0666667	0.75326	0.1666667	0.19791	0.2333333	0.02948
Regulation of TP53 Activity through Acetylation	0.1666667	0.11759	0.2	0.08579	0.2333333	0.02948
SLC-mediated transmembrane transport	0.0934959	0.40617	0.0813008	0.90704	0.1422764	0.02956

ABC transporters in lipid homeostasis	0.2222222	0.06682	0.1111111	0.574	0.2777778	0.03134
Signaling by cytosolic FGFR1 fusion mutants	0.1666667	0.20588	0.1666667	0.28807	0.2777778	0.03134
Gastrin-CREB signalling pathway via PKC and MAPK	0.2222222	0.06682	0.1666667	0.28807	0.2777778	0.03134
Signaling by FGFR2	0.1232877	0.18783	0.1232877	0.35053	0.1780822	0.03394
Cytochrome P450 - arranged by substrate type	0.0606061	0.84245	0.1212121	0.38295	0.1818182	0.0352
FGFR2c ligand binding and activation	0.0769231	0.69714	0.1538462	0.39976	0.3076923	0.03737
Notch-HLH transcription pathway	NA	NA	0.2307692	0.14694	0.3076923	0.03737
TNFR1-induced proapoptotic signaling	0.2307692	0.0992	0.2307692	0.14694	0.3076923	0.03737
Downstream signaling of activated FGFR3	0.08	0.65755	0.12	0.49212	0.24	0.03755
NOTCH3 Intracellular Domain Regulates Transcription	0.04	0.89959	0.2	0.11223	0.24	0.03755
Transport of small molecules	0.0871369	0.54436	0.0816044	0.98577	0.1230982	0.03922
Ion channel transport	0.0806452	0.67299	0.0967742	0.66852	0.1451613	0.04135
Signaling by Type 1 Insulin-like Growth Factor 1 Receptor (IGF1R)	0.0925926	0.51895	0.1851852	0.05026	0.1851852	0.04647
RHO GTPases activate CIT	0.1	0.5343	0.2	0.14869	0.25	0.04779
Inflammasomes	0.2	0.09221	0.25	0.05025	0.25	0.04779
Defective EXT2 causes exostoses 2	0.0714286	0.72375	0.0714286	0.78635	0.2857143	0.04818
Defective EXT1 causes exostoses 1, TRPS2 and CHDS	0.0714286	0.72375	0.0714286	0.78635	0.2857143	0.04818
Activation of RAC1	0.2142857	0.11838	0.2857143	0.05029	0.2857143	0.04818
Regulation of TP53 Activity through Association with Co-factors	0.1428571	0.35127	0.2142857	0.1733	0.2857143	0.04818
Tryptophan catabolism	0.0714286	0.72375	0.2142857	0.1733	0.2857143	0.04818
RUNX3 regulates NOTCH signaling	0.0714286	0.72375	0.2142857	0.1733	0.2857143	0.04818
