# Supplementary material for: Comparative transcriptional profiling of the early host response to infection by typhoidal and non-typhoidal Salmonella serovars in human intestinal organoids
Source: PLoS Pathog. 2021 Oct 20;17(10):e1009987. doi: 10.1371/journal.ppat.1009987 (PMC8570492; doi:10.1371/journal.ppat.1009987)
Supplement: S4 Table — Significantly downregulated DEGs from STM, SE or ST-infected HIOs at 2.5h were subjected to Reactome pathway analysis. (PDF) [file ppat.1009987.s012.pdf]

Table S4: Reactome Pathways Downregulated 2.5h pi

Description	STM		SE		ST	
	gene ratio	p-value	gene ratio	p-value	gene ratio	p-value
Mitochondrial translation initiation	0.2643678	2.40E-08	0.3908046	3.95E-13	0.5632184	1.04E-23
Mitochondrial translation	0.2473118	9.26E-08	0.3978495	1.84E-14	0.5483871	6.72E-24
Metabolism of RNA	0.1261128	1.04E-07	0.1824926	4.67E-12	0.2893175	5.87E-36
Mitochondrial translation elongation	0.2528736	1.15E-07	0.4137931	9.66E-15	0.5747126	1.01E-24
Mitochondrial translation termination	0.2528736	1.15E-07	0.4022989	6.32E-14	0.5517241	1.01E-22
Mitotic Prometaphase	0.1818182	1.45E-07	0.0757576	0.89276	0.1818182	0.0052
Cell Cycle	0.1232449	7.87E-07	0.0826833	0.93457	0.1809672	9.74E-07
Cell Cycle, Mitotic	0.125	3.52E-06	0.0876866	0.83338	0.1921642	2.23E-07
Cell Cycle Checkpoints	0.1433447	9.73E-06	0.0750853	0.93585	0.2286689	4.82E-08
AURKA Activation by TPX2	0.2328767	1.05E-05	0.0821918	0.74138	0.2328767	0.00419
Cilium Assembly	0.160804	1.09E-05	0.080402	0.84251	0.1859296	0.00317
mRNA Splicing	0.1623037	1.23E-05	0.1256545	0.13124	0.4188482	1.89E-26
Processing of Capped Intronless Pre-mRNA	0.3571429	1.28E-05	0.2142857	0.0524	0.5	7.59E-07
tRNA processing	0.1981132	1.50E-05	0.0943396	0.61124	0.1509434	0.18018
Processing of Capped Intron-Containing Pre-mRNA	0.1481481	2.03E-05	0.1193416	0.16535	0.3703704	5.60E-25
Regulation of PLK1 Activity at G2/M Transition	0.2045455	3.86E-05	0.1022727	0.51023	0.2386364	0.00112
Resolution of Sister Chromatid Cohesion	0.1774194	5.67E-05	0.0403226	0.99555	0.1935484	0.00954
M Phase	0.1246819	7.94E-05	0.0763359	0.95094	0.1933842	7.12E-06
Translation	0.1340206	9.21E-05	0.3814433	3.04E-39	0.4261168	1.29E-41
mRNA Splicing - Major Pathway	0.1530055	9.41E-05	0.1256831	0.13697	0.420765	1.19E-25
G2/M Transition	0.1494845	0.00011	0.1134021	0.28032	0.2525773	1.31E-07
Mitotic Spindle Checkpoint	0.1785714	0.00011	0.0535714	0.97118	0.1875	0.02058
Organelle biogenesis and maintenance	0.1326531	0.00012	0.0884354	0.75717	0.1666667	0.00748
Amplification of signal from the kinetochores	0.1875	0.00013	0.0416667	0.98866	0.1666667	0.09615
Amplification of signal from unattached kinetochores via a MAD2 inhibitory signal	0.1875	0.00013	0.0416667	0.98866	0.1666667	0.09615
Mitotic G2-G2/M phases	0.1479592	0.00013	0.1122449	0.29708	0.25	1.84E-07
Recruitment of mitotic centrosome proteins and complexes	0.195122	0.00018	0.1097561	0.42372	0.1829268	0.05477
Centrosome maturation	0.195122	0.00018	0.1097561	0.42372	0.1829268	0.05477
Processing of Intronless Pre-mRNAs	0.3684211	0.00021	0.2105263	0.11139	0.4210526	0.00084
SUMOylation	0.144385	0.00033	0.0802139	0.83859	0.171123	0.01902
Loss of Nlp from mitotic centrosomes	0.2	0.00035	0.0714286	0.83504	0.2	0.03214

Loss of proteins required for interphase microtubule organization from the centrosome	0.2	0.00035	0.0714286	0.83504	0.2	0.03214
Anchoring of the basal body to the plasma membrane	0.1734694	0.00051	0.0816327	0.76597	0.1836735	0.03661
tRNA modification in the nucleus and cytosol	0.2325581	0.0007	0.0930233	0.62684	0.1860465	0.12727
Recruitment of NuMA to mitotic centrosomes	0.173913	0.00071	0.0978261	0.56566	0.1847826	0.03937
RHO GTPases Activate Formins	0.1521739	0.00073	0.0362319	0.99848	0.1594203	0.08658
Separation of Sister Chromatids	0.1382979	0.00082	0.0797872	0.84424	0.2340426	5.13E-06
Intraflagellar transport	0.2115385	0.00091	0.0576923	0.89976	0.1923077	0.07972
Mitotic Metaphase and Anaphase	0.135	0.00096	0.08	0.84791	0.225	1.20E-05
Mitotic Anaphase	0.1306533	0.0019	0.080402	0.84251	0.2211055	2.37E-05
G0 and Early G1	0.2592593	0.00228	0.1481481	0.27527	0.3333333	0.00278
SUMO E3 ligases SUMOylate target proteins	0.1325967	0.00229	0.0773481	0.86779	0.160221	0.0527
Cleavage of Growing Transcript in the Termination Region	0.1791045	0.00248	0.1791045	0.02998	0.3731343	5.96E-08
RNA Polymerase II Transcription Termination	0.1791045	0.00248	0.1791045	0.02998	0.3731343	5.96E-08
Activation of E2F1 target genes at G1/S	0.25	0.00286	0.1071429	0.53319	0.25	0.03994
G1/S-Specific Transcription	0.25	0.00286	0.1071429	0.53319	0.25	0.03994
APC/C-mediated degradation of cell cycle proteins	0.1627907	0.00286	0.1395349	0.13975	0.3837209	1.96E-10
Regulation of mitotic cell cycle	0.1627907	0.00286	0.1395349	0.13975	0.3837209	1.96E-10
Peroxisomal lipid metabolism	0.2413793	0.00354	0.2758621	0.00578	0.137931	0.452
Transcription of E2F targets under negative control by p107 (RBL1) and p130 (RBL2) in complex with HDAC1	0.3125	0.00408	0.125	0.47945	0.25	0.1106
Peroxisomal protein import	0.1746032	0.00453	0.2698413	9.07E-05	0.1269841	0.46942
Glyoxylate metabolism and glycine degradation	0.2258065	0.00527	0.1290323	0.36825	0.1612903	0.29979
Beta-catenin phosphorylation cascade	0.2941176	0.00545	0.2352941	0.07989	0.1176471	0.61269
Condensation of Prometaphase Chromosomes	0.3636364	0.00563	0.0909091	0.6822	0.2727273	0.13137
SLBP Dependent Processing of Replication-Dependent Histone Pre-mRNAs	0.3636364	0.00563	0.1818182	0.29804	0.6363636	6.71E-05
tRNA processing in the nucleus	0.1785714	0.00565	0.1071429	0.48283	0.1428571	0.33863
TP53 Regulates Transcription of Genes Involved in G2 Cell Cycle Arrest	0.2777778	0.00711	0.2222222	0.09502	0.2222222	0.15452
Activation of APC/C and APC/C:Cdc20 mediated degradation of mitotic proteins	0.1578947	0.00713	0.1447368	0.12717	0.3684211	1.34E-08
Activation of the TFAP2 (AP-2) family of transcription factors	0.3333333	0.00797	NA	NA	0.0833333	0.77845
G2/M Checkpoints	0.125	0.00821	0.0952381	0.6025	0.2440476	3.53E-06

Transcription of E2F targets under negative control by DREAM complex	0.2631579	0.00909	0.2105263	0.11139	0.2631579	0.06435
Phosphorylation of the APC/C	0.2631579	0.00909	0.1052632	0.57376	0.3684211	0.00436
DNA Repair	0.1075949	0.01022	0.1012658	0.47201	0.1740506	0.00184
Regulation of APC/C activators between G1/S and early anaphase	0.15	0.01068	0.1375	0.16401	0.375	2.49E-09
Chk1/Chk2(Cds1) mediated inactivation of Cyclin B:Cdk1 complex	0.3076923	0.01087	0.0769231	0.74203	0.3076923	0.0576
SUMOylation of transcription cofactors	0.1777778	0.01309	0.1111111	0.46324	0.2444444	0.01348
Mitochondrial tRNA aminoacylation	0.2380952	0.01414	0.3333333	0.00304	0.1904762	0.2299
Mitochondrial Fatty Acid Beta-Oxidation	0.1891892	0.01423	0.2702703	0.00249	0.2432432	0.02508
APC/C:Cdc20 mediated degradation of mitotic proteins	0.1466667	0.01662	0.1466667	0.11874	0.3733333	9.51E-09
G2/M DNA damage checkpoint	0.1368421	0.01686	0.0526316	0.96476	0.2	0.01409
RHO GTPase Effectors	0.1044304	0.01699	0.0379747	0.99999	0.1708861	0.00303
Protein localization	0.1259843	0.01817	0.2362205	4.48E-06	0.1811024	0.02335
mRNA decay by 5' to 3' exoribonuclease	0.2666667	0.01853	0.1333333	0.44528	0.4	0.00518
mRNA 3'-end processing	0.1551724	0.02061	0.1724138	0.05647	0.3275862	2.06E-05
TP53 Regulates Transcription of Cell Cycle Genes	0.1632653	0.02135	0.1020408	0.54061	0.2653061	0.00358
Diseases associated with N-glycosylation of proteins	0.2352941	0.02885	0.3529412	0.00438	0.2941176	0.04169
SUMOylation of DNA methylation proteins	0.2352941	0.02885	0.1176471	0.5123	0.1764706	0.3245
Cyclin A/B1/B2 associated events during G2/M transition	0.2	0.02919	0.08	0.72335	0.2	0.16459
mRNA Splicing - Minor Pathway	0.1538462	0.02958	0.0961538	0.59503	0.4423077	4.35E-09
SLBP independent Processing of Histone Pre-mRNAs	0.3	0.02962	0.2	0.2597	0.6	0.00037
Processing and activation of SUMO	0.3	0.02962	0.1	0.64728	0.5	0.00343
APC:Cdc20 mediated degradation of cell cycle proteins prior to satisfation of the cell cycle checkpoint	0.1369863	0.03343	0.1506849	0.10287	0.369863	2.20E-08
SUMOylation of DNA replication proteins	0.1590909	0.0345	0.0454545	0.94076	0.1590909	0.25771
Regulation of TP53 Activity	0.1125	0.03574	0.1	0.52095	0.13125	0.33474
DNA Replication	0.1181102	0.03623	0.1181102	0.27255	0.2677165	2.69E-06
Beta-oxidation of very long chain fatty acids	0.2727273	0.03862	0.3636364	0.0178	0.1818182	0.37882
CDC6 association with the ORC:origin complex	0.2727273	0.03862	0.0909091	0.6822	0.1818182	0.37882
Regulation of TP53 Degradation	0.1666667	0.03989	0.0833333	0.70539	0.1388889	0.42215
Synthesis of DNA	0.1176471	0.04325	0.1176471	0.2869	0.2689076	4.75E-06
Dual incision in TC-NER	0.1363636	0.04342	0.1515152	0.11309	0.2575758	0.00132

Transcriptional Regulation by TP53	0.0958904	0.04366	0.1342466	0.01628	0.1808219	0.00023
Activation of ATR in response to replication stress	0.1621622	0.04486	0.1351351	0.30123	0.1621622	0.26676
Regulation of TP53 Expression and Degradation	0.1621622	0.04486	0.0810811	0.72308	0.1351351	0.44643
RNA Polymerase III Transcription Initiation From Type 3 Promoter	0.1785714	0.04534	0.0714286	0.77991	0.2142857	0.10403
G1/S Transition	0.1145038	0.04572	0.129771	0.14868	0.2900763	7.35E-08
Fatty acid metabolism	0.1073446	0.04747	0.1581921	0.0083	0.1299435	0.34263
Kinesins	0.1403509	0.04771	0.0701754	0.82864	0.1578947	0.22478
HDR through Homologous Recombination (HRR) or Single Strand Annealing (SSA)	0.1136364	0.04835	0.0833333	0.76833	0.1742424	0.035
Post-chaperonin tubulin folding pathway	0.2	0.04961	0.1	0.6023	0.35	0.00603
Metabolism of cofactors	0.2	0.04961	0.2	0.12891	0.2	0.20375
Eukaryotic Translation Elongation	0.0430108	0.90596	0.6021505	4.56E-33	0.4408602	4.37E-15
Peptide chain elongation	0.0449438	0.88686	0.6067416	3.64E-32	0.4269663	1.55E-13
Viral mRNA Translation	0.0449438	0.88686	0.6067416	3.64E-32	0.4157303	8.75E-13
Selenocysteine synthesis	0.0537634	0.80161	0.5913978	6.46E-32	0.4193548	1.50E-13
Eukaryotic Translation Termination	0.0537634	0.80161	0.5806452	8.74E-31	0.4408602	4.37E-15
Nonsense Mediated Decay (NMD) independent of the Exon Junction Complex (EJC)	0.0526316	0.81631	0.5684211	3.92E-30	0.4105263	3.43E-13
L13a-mediated translational silencing of Ceruloplasmin expression	0.036036	0.96093	0.5225225	1.27E-29	0.3963964	4.69E-14
Formation of a pool of free 40S subunits	0.039604	0.9358	0.5445545	2.30E-29	0.4059406	1.34E-13
GTP hydrolysis and joining of the 60S ribosomal subunit	0.0357143	0.96287	0.5178571	2.39E-29	0.3928571	6.87E-14
Selenoamino acid metabolism	0.0508475	0.85452	0.4915254	8.78E-28	0.3898305	2.59E-14
Nonsense-Mediated Decay (NMD)	0.0782609	0.43557	0.4956522	1.45E-27	0.3826087	2.08E-13
Nonsense Mediated Decay (NMD) enhanced by the Exon Junction Complex (EJC)	0.0782609	0.43557	0.4956522	1.45E-27	0.3826087	2.08E-13
Eukaryotic Translation Initiation	0.0336134	0.97411	0.487395	1.55E-27	0.3865546	3.76E-14
Cap-dependent Translation Initiation	0.0336134	0.97411	0.487395	1.55E-27	0.3865546	3.76E-14
SRP-dependent cotranslational protein targeting to membrane	0.0803571	0.40446	0.5	2.37E-27	0.4107143	2.51E-15
Influenza Viral RNA Transcription and Replication	0.0526316	0.84473	0.4285714	1.59E-23	0.3609023	2.22E-13
Influenza Life Cycle	0.0559441	0.80813	0.3986014	1.21E-21	0.3566434	6.75E-14
Regulation of expression of SLITs and ROBOs	0.0701754	0.56532	0.3625731	6.73E-21	0.3508772	9.65E-16

Major pathway of rRNA processing in the nucleolus and cytosol	0.0756757	0.44774	0.3459459	2.73E-20	0.2864865	3.29E-10
Influenza Infection	0.0519481	0.86636	0.3701299	8.38E-20	0.3311688	1.84E-12
rRNA processing in the nucleus and cytosol	0.0769231	0.4177	0.3333333	1.30E-19	0.2871795	9.34E-11
rRNA processing	0.0780488	0.38966	0.3170732	2.47E-18	0.2731707	7.55E-10
Metabolism of amino acids and derivatives	0.0648649	0.71597	0.2459459	5.38E-17	0.2324324	2.40E-10
Signaling by ROBO receptors	0.0642202	0.6965	0.2889908	1.31E-15	0.3027523	1.26E-13
Translation initiation complex formation	0.0172414	0.98642	0.4482759	5.53E-12	0.3448276	5.18E-06
Ribosomal scanning and start codon recognition	0.0172414	0.98642	0.4482759	5.53E-12	0.3448276	5.18E-06
Activation of the mRNA upon binding of the cap-binding complex and eIFs, and subsequent binding to 43S	0.0169492	0.9874	0.440678	8.97E-12	0.3559322	1.68E-06
Formation of the ternary complex, and subsequently, the 43S complex	0.0196078	0.97716	0.4705882	1.00E-11	0.3529412	1.05E-05
Infectious disease	0.0497382	0.96675	0.2068063	1.00E-10	0.2643979	9.33E-16
Asparagine N-linked glycosylation	0.0728477	0.48938	0.1953642	2.08E-07	0.1754967	0.00184
Axon guidance	0.0471869	0.99303	0.1506352	5.31E-05	0.1887477	4.81E-07
Respiratory electron transport	0.08	0.42062	0.23	8.86E-05	0.36	2.44E-10
Neddylation	0.0982906	0.07191	0.1794872	9.39E-05	0.2051282	7.84E-05
Transport to the Golgi and subsequent modification	0.0710383	0.5464	0.1912568	9.70E-05	0.1584699	0.05928
ER to Golgi Anterograde Transport	0.0657895	0.64989	0.1973684	0.00017	0.1447368	0.18123
COPII-mediated vesicle transport	0.0294118	0.95965	0.2352941	0.00078	0.1470588	0.27755
TP53 Regulates Metabolic Genes	0.1162791	0.08436	0.2093023	0.00162	0.244186	0.00081
Respiratory electron transport, ATP synthesis by chemiosmotic coupling, and heat production by uncoupling proteins.	0.0650407	0.65709	0.1869919	0.00198	0.300813	3.82E-08
mitochondrial fatty acid beta-oxidation of saturated fatty acids	0.0909091	0.5567	0.4545455	0.0026	0.0909091	0.74879
The citric acid (TCA) cycle and respiratory electron transport	0.0689655	0.59006	0.1666667	0.00345	0.2701149	2.58E-08
Cargo concentration in the ER	0.0909091	0.42102	0.2727273	0.00378	0.1818182	0.18699
N-glycan trimming in the ER and Calnexin/Calreticulin cycle	0.0857143	0.45946	0.2571429	0.00579	0.2571429	0.01763
COPI-mediated anterograde transport	0.0808081	0.40955	0.1818182	0.00783	0.1515152	0.1859
Antigen processing: Ubiquitination & Proteasome degradation	0.0970874	0.0512	0.1423948	0.00832	0.1618123	0.01209
Degradation of cysteine and homocysteine	NA	NA	0.3571429	0.00877	NA	NA
Mitochondrial protein import	0.078125	0.48346	0.203125	0.00889	0.234375	0.0065
Class I MHC mediated antigen processing & presentation	0.0862534	0.14922	0.1374663	0.00938	0.1617251	0.00649
Iron uptake and transport	NA	NA	0.2068966	0.01012	0.2413793	0.0064

Pentose phosphate pathway	NA	NA	0.3333333	0.0121	0.1333333	0.54275
Gamma carboxylation, hypusine formation and arylsulfatase activation	0.0512821	0.77709	0.2307692	0.01214	0.2051282	0.08152
Gamma-carboxylation of protein precursors	NA	NA	0.4	0.01228	0.5	0.00343
Regulation of FZD by ubiquitination	0.1428571	0.18518	0.2857143	0.01364	0.0952381	0.72742
TGF-beta receptor signaling in EMT (epithelial to mesenchymal transition)	0.125	0.3174	0.3125	0.0162	0.125	0.57879
Glycogen synthesis	0.0625	0.69382	0.3125	0.0162	0.1875	0.29049
Complex I biogenesis	0.0545455	0.76128	0.2	0.01728	0.3636364	2.01E-06
Gamma-carboxylation, transport, and amino-terminal cleavage of proteins	NA	NA	0.3636364	0.0178	0.4545455	0.00567
tRNA Aminoacylation	0.1428571	0.07528	0.2142857	0.01956	0.2380952	0.02162
Golgi Associated Vesicle Biogenesis	0.0357143	0.91618	0.1964286	0.01965	0.125	0.49624
Biosynthesis of the N-glycan precursor (dolichol lipid-linked oligosaccharide, LLO) and transfer to a nascent protein	0.0897436	0.32003	0.1794872	0.01972	0.2051282	0.01837
Membrane Trafficking	0.0557325	0.95332	0.1226115	0.02624	0.1401274	0.04585
Hh mutants abrogate ligand secretion	0.0847458	0.41241	0.1864407	0.02817	0.3389831	6.98E-06
Downstream TCR signaling	0.0612245	0.70768	0.1632653	0.03044	0.3163265	1.37E-07
Antigen Presentation: Folding, assembly and peptide loading of class I MHC	NA	NA	0.24	0.0317	0.24	0.066
Mitochondrial iron-sulfur cluster biogenesis	0.0769231	0.61768	0.3076923	0.03288	0.2307692	0.19143
Purine salvage	0.0769231	0.61768	0.3076923	0.03288	0.0769231	0.80462
Ubiquitin Mediated Degradation of Phosphorylated Cdc25A	0.1132075	0.17368	0.1886792	0.03289	0.3396226	1.94E-05
p53-Independent DNA Damage Response	0.1132075	0.17368	0.1886792	0.03289	0.3396226	1.94E-05
p53-Independent G1/S DNA damage checkpoint	0.1132075	0.17368	0.1886792	0.03289	0.3396226	1.94E-05
Translesion Synthesis by POLH	0.1578947	0.14939	0.2631579	0.03364	0.2105263	0.17856
Gene and protein expression by JAK-STAT signaling after Interleukin-12 stimulation	0.025641	0.94432	0.2051282	0.03435	0.2051282	0.08152
Defective CFTR causes cystic fibrosis	0.0819672	0.44111	0.1803279	0.03516	0.3442623	3.12E-06
Calnexin/calreticulin cycle	0.0384615	0.85401	0.2307692	0.03788	0.3076923	0.00813
rRNA modification in the nucleus and cytosol	0.0967742	0.27894	0.1774194	0.03908	0.2096774	0.02677
Synthesis of PA	0.075	0.55016	0.2	0.03936	0.1	0.71059
Interleukin-12 signaling	0.0208333	0.97146	0.1875	0.04329	0.1875	0.10585
Diseases of carbohydrate metabolism	0.0294118	0.91932	0.2058824	0.04554	0.1764706	0.20603

Gluconeogenesis	NA	NA	0.2058824	0.04554	0.2058824	0.09812
Amino acid synthesis and interconversion (transamination)	0.0294118	0.91932	0.2058824	0.04554	0.1764706	0.20603
Hh mutants that don't undergo autocatalytic processing are degraded by ERAD	0.0892857	0.36903	0.1785714	0.046	0.3392857	1.16E-05
trans-Golgi Network Vesicle Budding	0.0277778	0.96854	0.1666667	0.04889	0.1388889	0.34144
Clathrin derived vesicle budding	0.0277778	0.96854	0.1666667	0.04889	0.1388889	0.34144
Interleukin-37 signaling	NA	NA	0.2380952	0.04996	0.047619	0.92854
ER Quality Control Compartment (ERQC)	0.047619	0.78856	0.2380952	0.04996	0.2380952	0.09264
SCF(Skp2)-mediated degradation of p27/p21	0.0833333	0.42679	0.15	0.13444	0.4166667	4.21E-09
Cyclin E associated events during G1/S transition	0.0843373	0.37918	0.1445783	0.1154	0.3493976	2.95E-08
Cyclin A:Cdk2-associated events at S phase entry	0.0823529	0.40303	0.1411765	0.13134	0.3411765	5.40E-08
Switching of origins to a post-replicative state	0.1111111	0.10634	0.1222222	0.27494	0.3333333	5.81E-08
Cdc20:Phospho-APC/C mediated degradation of Cyclin A	0.125	0.0688	0.1527778	0.09542	0.3611111	7.08E-08
CDK-mediated phosphorylation and removal of Cdc6	0.1111111	0.13888	0.1388889	0.17086	0.3611111	7.08E-08
HIV Infection	0.0560345	0.85207	0.0948276	0.61651	0.2327586	5.36E-07
The role of GTSE1 in G2/M progression after G2 checkpoint	0.1066667	0.16357	0.12	0.32222	0.3333333	7.33E-07
Mitotic G1-G1/S phases	0.1073826	0.06481	0.1275168	0.14947	0.261745	9.46E-07
S Phase	0.1055901	0.06619	0.1242236	0.16969	0.2546584	1.09E-06
APC/C:Cdh1 mediated degradation of Cdc20 and other APC/C:Cdh1 targeted proteins in late mitosis/early G1	0.1111111	0.13888	0.1527778	0.09542	0.3333333	1.22E-06
Autodegradation of Cdh1 by Cdh1:APC/C	0.0952381	0.2915	0.1587302	0.08905	0.3492063	1.38E-06
FBXL7 down-regulates AURKA during mitotic entry and in early mitosis	0.0925926	0.3401	0.1666667	0.08122	0.3703704	1.44E-06
G1/S DNA Damage Checkpoints	0.1029412	0.20877	0.1470588	0.13096	0.3382353	1.52E-06
SCF-beta-TrCP mediated degradation of Emi1	0.0909091	0.35455	0.1454545	0.17254	0.3636364	2.01E-06
ESR-mediated signaling	0.0705128	0.55966	0.0705128	0.91416	0.25	3.24E-06
Vpu mediated degradation of CD4	0.0769231	0.5127	0.1538462	0.13737	0.3653846	3.32E-06
p53-Dependent G1 DNA Damage Response	0.1060606	0.18846	0.1363636	0.20177	0.3333333	3.37E-06
p53-Dependent G1/S DNA damage checkpoint	0.1060606	0.18846	0.1363636	0.20177	0.3333333	3.37E-06
Orc1 removal from chromatin	0.1126761	0.13109	0.1267606	0.26651	0.3239437	3.53E-06
APC/C:Cdc20 mediated degradation of Securin	0.0895522	0.3427	0.1492537	0.12184	0.3283582	4.46E-06
TCR signaling	0.0672269	0.6199	0.1344538	0.12665	0.2689076	4.75E-06
Negative regulation of NOTCH4 signaling	0.0740741	0.54299	0.1481481	0.1604	0.3518519	6.34E-06
NIK-->noncanonical NF-kB signaling	0.0677966	0.61418	0.1355932	0.2249	0.3389831	6.98E-06

Estrogen-dependent gene expression	0.0733333	0.50604	0.0733333	0.8878	0.2466667	8.03E-06
Cellular responses to external stimuli	0.0675944	0.65407	0.0735586	0.98194	0.1829026	8.74E-06
Dectin-1 mediated noncanonical NF-kB signaling	0.0666667	0.62757	0.1333333	0.23881	0.3333333	9.32E-06
DNA Damage/Telomere Stress Induced Senescence	0.1	0.20879	0.05	0.96286	0.3	9.75E-06
Signaling by Nuclear Receptors	0.0653266	0.66871	0.0653266	0.96391	0.2261307	1.05E-05
Metabolism of polyamines	0.0697674	0.58189	0.1395349	0.13975	0.2906977	1.18E-05
FGFR2 alternative splicing	0.1538462	0.10982	0.1538462	0.25257	0.4615385	1.36E-05
Signaling by NOTCH4	0.0731707	0.53439	0.1463415	0.10786	0.2926829	1.55E-05
Cellular responses to stress	0.0704225	0.55539	0.0704225	0.98519	0.185446	2.30E-05
CDT1 association with the CDC6:ORC:origin complex	0.1186441	0.12466	0.1525424	0.12453	0.3220339	2.70E-05
Regulation of activated PAK-2p34 by proteasome mediated degradation	0.08	0.48151	0.16	0.11615	0.34	3.23E-05
RUNX1 regulates transcription of genes involved in differentiation of HSCs	0.0538462	0.82774	0.0692308	0.90689	0.2461538	3.37E-05
Degradation of GLI1 by the proteasome	0.0666667	0.62757	0.1333333	0.23881	0.3166667	3.52E-05
Degradation of GLI2 by the proteasome	0.0666667	0.62757	0.1333333	0.23881	0.3166667	3.52E-05
GLI3 is processed to GLI3R by the proteasome	0.0666667	0.62757	0.1333333	0.23881	0.3166667	3.52E-05
Hedgehog ligand biogenesis	0.0769231	0.49734	0.1692308	0.05262	0.3076923	3.53E-05
ABC transporter disorders	0.0657895	0.63828	0.1447368	0.12717	0.2894737	4.19E-05
Regulation of ornithine decarboxylase (ODC)	0.0784314	0.49721	0.1372549	0.23636	0.3333333	4.32E-05
AUF1 (hnRNP D0) binds and destabilizes mRNA	0.0714286	0.57229	0.1428571	0.18509	0.3214286	4.50E-05
Transcriptional regulation by RUNX2	0.0661157	0.63876	0.1157025	0.30861	0.2479339	5.09E-05
Autodegradation of the E3 ubiquitin ligase COP1	0.0961538	0.31135	0.1538462	0.13737	0.3269231	5.73E-05
Ubiquitin-dependent degradation of Cyclin D1	0.0769231	0.5127	0.1730769	0.06689	0.3269231	5.73E-05
Ubiquitin-dependent degradation of Cyclin D	0.0769231	0.5127	0.1730769	0.06689	0.3269231	5.73E-05
Activation of NF-kappaB in B cells	0.0597015	0.71269	0.119403	0.34268	0.2985075	5.73E-05
Degradation of DVL	0.0701754	0.58654	0.1754386	0.05106	0.3157895	5.87E-05
Regulation of RAS by GAPs	0.0882353	0.35566	0.1470588	0.13096	0.2941176	7.23E-05
Regulation of Apoptosis	0.0754717	0.52796	0.1509434	0.14867	0.3207547	7.51E-05
Vif-mediated degradation of APOBEC3G	0.0740741	0.54299	0.1481481	0.1604	0.3148148	9.78E-05
Formation of TC-NER Pre-Incision Complex	0.1111111	0.18449	0.1481481	0.1604	0.3148148	9.78E-05
Cross-presentation of soluble exogenous antigens (endosomes)	0.08	0.48151	0.12	0.37383	0.32	0.00013
Degradation of AXIN	0.0727273	0.55777	0.1454545	0.17254	0.3090909	0.00013
Regulation of RUNX3 expression and activity	0.0727273	0.55777	0.1454545	0.17254	0.3090909	0.00013

Signaling by FGFR2 IIIa TM	0.1052632	0.39673	0.1052632	0.57376	0.4736842	0.00013
Abortive elongation of HIV-1 transcript in the absence of Tat	0.0869565	0.49523	0.1304348	0.40111	0.4347826	0.00014
Degradation of beta-catenin by the destruction complex	0.1219512	0.0654	0.1585366	0.05843	0.2682927	0.00015
FCERI mediated NF-kB activation	0.0487805	0.84525	0.1219512	0.2907	0.2682927	0.00015
Oxygen-dependent proline hydroxylation of Hypoxia-inducible Factor Alpha	0.0606061	0.70146	0.1212121	0.32734	0.2878788	0.00015
Nucleotide Excision Repair	0.1081081	0.09634	0.1171171	0.3024	0.2432432	0.00017
Downstream signaling events of B Cell Receptor (BCR)	0.0722892	0.54649	0.0963855	0.58524	0.2650602	0.00018
ER-Phagosome pathway	0.060241	0.71404	0.1325301	0.19473	0.2650602	0.00018
Stabilization of p53	0.1052632	0.21842	0.1578947	0.10594	0.2982456	0.00021
HIV Transcription Initiation	0.0425532	0.85788	0.106383	0.50251	0.3191489	0.00021
RNA Polymerase II HIV Promoter Escape	0.0425532	0.85788	0.106383	0.50251	0.3191489	0.00021
RNA Polymerase II Promoter Escape	0.0425532	0.85788	0.106383	0.50251	0.3191489	0.00021
RNA Polymerase II Transcription Pre-Initiation And Promoter Opening	0.0425532	0.85788	0.106383	0.50251	0.3191489	0.00021
RNA Polymerase II Transcription Initiation	0.0425532	0.85788	0.106383	0.50251	0.3191489	0.00021
RNA Polymerase II Transcription Initiation And Promoter Clearance	0.0425532	0.85788	0.106383	0.50251	0.3191489	0.00021
Transcription of the HIV genome	0.0547945	0.77355	0.109589	0.43589	0.2739726	0.00021
Regulation of RUNX2 expression and activity	0.0547945	0.77355	0.1369863	0.18166	0.2739726	0.00021
CLEC7A (Dectin-1) signaling	0.0594059	0.73473	0.1089109	0.41558	0.2475248	0.00022
Assembly of the pre-replicative complex	0.1029412	0.20877	0.1323529	0.22687	0.2794118	0.00023
mRNA Capping	0.0689655	0.62233	0.0689655	0.79638	0.3793103	0.00027
Host Interactions of HIV factors	0.0625	0.70043	0.1015625	0.50355	0.2265625	0.00036
Protein folding	0.0707071	0.5648	0.1212121	0.27197	0.2424242	0.0004
Regulation of mRNA stability by proteins that bind AU-rich elements	0.0909091	0.28949	0.1136364	0.37136	0.25	0.00043
Apoptosis	0.0650888	0.66674	0.1005917	0.50902	0.2071006	0.00058
RNA Polymerase II Pre-transcription Events	0.0595238	0.72388	0.1190476	0.31714	0.25	0.00058
Late Phase of HIV Life Cycle	0.057971	0.77598	0.0942029	0.61642	0.2173913	0.00061
RNA Pol II CTD phosphorylation and interaction with CE during HIV infection	0.037037	0.86444	0.0740741	0.76229	0.3703704	0.00064
RNA Pol II CTD phosphorylation and interaction with CE	0.037037	0.86444	0.0740741	0.76229	0.3703704	0.00064
Signaling by NOTCH	0.0553191	0.86385	0.0851064	0.79364	0.1914894	0.00064

HIV Life Cycle	0.0596026	0.75745	0.0860927	0.74179	0.2119205	0.00065
Transcription-Coupled Nucleotide Excision Repair (TC-NER)	0.1265823	0.05314	0.1392405	0.15434	0.2531646	0.00066
DNA Replication Pre-Initiation	0.0941176	0.25824	0.1411765	0.13134	0.2470588	0.00069
snRNP Assembly	0.1346154	0.07424	0.1346154	0.25175	0.2884615	0.00071
Metabolism of non-coding RNA	0.1346154	0.07424	0.1346154	0.25175	0.2884615	0.00071
RNA polymerase II transcribes snRNA genes	0.1081081	0.15512	0.1081081	0.45139	0.2567568	0.00074
ABC-family proteins mediated transport	0.0582524	0.75175	0.1456311	0.08155	0.2330097	0.00074
Programmed Cell Death	0.0639535	0.68922	0.0988372	0.53952	0.2034884	0.00081
Regulation of PTEN stability and activity	0.0724638	0.5513	0.1449275	0.14042	0.2608696	0.00082
Regulation of Hypoxia-inducible Factor (HIF) by oxygen	0.0666667	0.62651	0.12	0.32222	0.2533333	0.00088
Cellular response to hypoxia	0.0666667	0.62651	0.12	0.32222	0.2533333	0.00088
Asymmetric localization of PCP proteins	0.0625	0.67807	0.125	0.29705	0.265625	0.00091
Formation of the Early Elongation Complex	0.0606061	0.69242	0.0909091	0.64718	0.3333333	0.00097
Formation of the HIV-1 Early Elongation Complex	0.0606061	0.69242	0.0909091	0.64718	0.3333333	0.00097
Hedgehog 'off' state	0.0630631	0.68548	0.0900901	0.66978	0.2252252	0.00098
Antigen processing-Cross presentation	0.0606061	0.7169	0.1212121	0.27197	0.2323232	0.00098
Pausing and recovery of Tat-mediated HIV elongation	0.0588235	0.70817	0.1470588	0.24232	0.3235294	0.00128
Tat-mediated HIV elongation arrest and recovery	0.0588235	0.70817	0.1470588	0.24232	0.3235294	0.00128
APC-Cdc20 mediated degradation of Nek2A	0.16	0.09817	0.2	0.09429	0.36	0.00151
UCH proteinases	0.0686275	0.59696	0.1078431	0.42868	0.2254902	0.00151
MHC class II antigen presentation	0.0826446	0.36059	0.0991736	0.54132	0.214876	0.00163
HIV Transcription Elongation	0.0652174	0.64619	0.1086957	0.483	0.2826087	0.00194
Formation of HIV-1 elongation complex containing HIV-1 Tat	0.0652174	0.64619	0.1086957	0.483	0.2826087	0.00194
Tat-mediated elongation of the HIV-1 transcript	0.0652174	0.64619	0.1086957	0.483	0.2826087	0.00194
PCP/CE pathway	0.0434783	0.90148	0.0978261	0.56566	0.2282609	0.00203
HIV elongation arrest and recovery	0.0555556	0.73766	0.1388889	0.2813	0.3055556	0.00215
Pausing and recovery of HIV elongation	0.0555556	0.73766	0.1388889	0.2813	0.3055556	0.00215
Apoptosis induced DNA fragmentation	0.0769231	0.61768	0.0769231	0.74203	0.4615385	0.00219
Activation of DNA fragmentation factor	0.0769231	0.61768	0.0769231	0.74203	0.4615385	0.00219
Packaging Of Telomere Ends	0.0769231	0.5127	0.0384615	0.97047	0.2692308	0.00219
Cellular Senescence	0.0769231	0.4177	0.0666667	0.95635	0.1897436	0.00221
Transcriptional regulation by small RNAs	0.0666667	0.62787	0.0380952	0.99437	0.2190476	0.00226
Translocation of SLC2A4 (GLUT4) to the plasma membrane	0.1142857	0.12354	0.1	0.54669	0.2428571	0.00262
Diseases of signal transduction	0.0687831	0.60518	0.1137566	0.18381	0.1666667	0.00266

Formation of HIV elongation complex in the absence of HIV Tat	0.0625	0.67477	0.1041667	0.52172	0.2708333	0.00294
Gap-filling DNA repair synthesis and ligation in TC-NER	0.1230769	0.08938	0.1538462	0.10471	0.2461538	0.00299
MAPK6/MAPK4 signaling	0.0674157	0.61584	0.1235955	0.26287	0.2247191	0.00311
PTEN Regulation	0.0785714	0.41362	0.1071429	0.4124	0.2	0.00335
TNFR2 non-canonical NF-kB pathway	0.0490196	0.86098	0.1176471	0.30721	0.2156863	0.00341
Methylation	0.2142857	0.07272	0.1428571	0.40988	0.4285714	0.00345
APC/C:Cdc20 mediated degradation of Cyclin B	0.173913	0.07674	0.173913	0.1874	0.3478261	0.00352
Fc epsilon receptor (FCERI) signaling	0.0522388	0.85008	0.1044776	0.45651	0.2014925	0.00353
Signaling by Hedgehog	0.0612245	0.72903	0.0884354	0.7063	0.1972789	0.00353
FGFR2 mutant receptor activation	0.0606061	0.69242	0.0606061	0.8518	0.3030303	0.00362
Interleukin-1 signaling	0.0485437	0.86655	0.0873786	0.70159	0.2135922	0.00387
Formation of RNA Pol II elongation complex	0.0655738	0.64065	0.1147541	0.39929	0.2459016	0.00403
RNA Polymerase II Transcription Elongation	0.0655738	0.64065	0.1147541	0.39929	0.2459016	0.00403
Hedgehog 'on' state	0.0705882	0.57025	0.0941176	0.61288	0.2235294	0.00415
Signaling by FGFR2	0.0684932	0.60229	0.109589	0.43589	0.2328767	0.00419
Senescence-Associated Secretory Phenotype (SASP)	0.0545455	0.80501	0.0545455	0.96726	0.2090909	0.00421
Translocation of ZAP-70 to Immunological synapse	NA	NA	0.1578947	0.28869	0.3684211	0.00436
MicroRNA (miRNA) biogenesis	0.0833333	0.51823	0.125	0.42862	0.3333333	0.00474
Condensation of Prophase Chromosomes	0.1216216	0.07896	0.0135135	0.99956	0.2297297	0.00485
Chaperonin-mediated protein folding	0.0430108	0.90596	0.1075269	0.44021	0.2150538	0.00528
Signaling by the B Cell Receptor (BCR)	0.0625	0.69451	0.1071429	0.43055	0.2053571	0.00531
Signaling by FGFR	0.0804598	0.42684	0.137931	0.14844	0.2183908	0.00542
Inhibition of the proteolytic activity of APC/C required for the onset of anaphase by mitotic spindle checkpoint components	0.15	0.167	0.15	0.31688	0.35	0.00603
Inactivation of APC/C via direct inhibition of the APC/C complex	0.15	0.167	0.15	0.31688	0.35	0.00603
Cooperation of Prefoldin and TriC/CCT in actin and tubulin folding	0.0666667	0.64094	0.1	0.58125	0.3	0.00612
RHO GTPases activate PKNs	0.0526316	0.81631	0.0210526	0.99945	0.2105263	0.00676
Transport of Mature Transcript to Cytoplasm	0.0963855	0.23802	0.0843373	0.72598	0.2168675	0.00721
Transcriptional regulation by RUNX3	0.0625	0.68864	0.125	0.23817	0.2083333	0.00762
Viral Messenger RNA Synthesis	0.0714286	0.58384	0.0714286	0.79927	0.2619048	0.0079
Prefoldin mediated transfer of substrate to CCT/TriC	0.0769231	0.56207	0.1153846	0.48216	0.3076923	0.00813
Transcriptional regulation by RUNX1	0.0753138	0.43882	0.0920502	0.6726	0.1715481	0.00844
Ionotropic activity of kainate receptors	0.0833333	0.58832	NA	NA	0.4166667	0.00879

Activation of Ca-permeable Kainate Receptor	0.0833333	0.58832	NA	NA	0.4166667	0.00879
B-WICH complex positively regulates rRNA expression	0.0769231	0.47405	0.0549451	0.95408	0.2087912	0.00894
Signaling by FGFR2 in disease	0.0697674	0.60006	0.0697674	0.8122	0.255814	0.00951
Processing of DNA double-strand break ends	0.1020408	0.15912	0.0612245	0.93165	0.2040816	0.0096
Meiotic synapsis	0.0759494	0.49734	0.0379747	0.98774	0.2151899	0.0096
Phosphorylation of CD3 and TCR zeta chains	0.0454545	0.80366	0.1363636	0.37324	0.3181818	0.01075
Citric acid cycle (TCA cycle)	NA	NA	0.0909091	0.65498	0.3181818	0.01075
NoRC negatively regulates rRNA expression	0.0943396	0.22242	0.0471698	0.98346	0.1981132	0.01134
Positive epigenetic regulation of rRNA expression	0.0849057	0.34253	0.0566038	0.95791	0.1981132	0.01134
HDACs deacetylate histones	0.0957447	0.22554	0.0425532	0.98679	0.2021277	0.01262
Nonhomologous End-Joining (NHEJ)	0.115942	0.11622	0.057971	0.92039	0.2173913	0.01319
PD-1 signaling	NA	NA	0.173913	0.1874	0.3043478	0.01392
Gene Silencing by RNA	0.0814815	0.36721	0.0444444	0.99391	0.1851852	0.01431
Recruitment and ATM-mediated phosphorylation of repair and signaling proteins at DNA double strand breaks	0.1052632	0.17223	0.0657895	0.88305	0.2105263	0.01447
C-type lectin receptors (CLRs)	0.0422535	0.94492	0.0774648	0.84278	0.1830986	0.01459
TCF dependent signaling in response to WNT	0.0901288	0.15734	0.111588	0.28643	0.167382	0.01479
Negative epigenetic regulation of rRNA expression	0.0917431	0.24841	0.0458716	0.98663	0.1926606	0.01542
Ub-specific processing proteases	0.0772727	0.4002	0.1	0.51216	0.1681818	0.01607
DNA Double Strand Break Response	0.1038961	0.18109	0.0649351	0.88976	0.2077922	0.01633
RNA Polymerase I Chain Elongation	0.0777778	0.46232	0.0444444	0.98214	0.2	0.01658
Disorders of transmembrane transporters	0.0416667	0.94948	0.1041667	0.45685	0.1805556	0.01729
TP53 Regulates Transcription of Genes Involved in G1 Cell Cycle Arrest	0.0714286	0.64496	0.0714286	0.76758	0.3571429	0.01818
Amyloid fiber formation	0.0377358	0.94979	0.0849057	0.73353	0.1886792	0.02201
Removal of aminoterminal propeptides from gamma-carboxylated proteins	NA	NA	0.3	0.06825	0.4	0.02253
Activated PKN1 stimulates transcription of AR (androgen receptor) regulated genes KLK2 and KLK3	0.0597015	0.71269	0.0298507	0.99235	0.2089552	0.02268
Neutrophil degranulation	0.0354906	0.99977	0.091858	0.72441	0.1482255	0.02372
RHO GTPases Activate ROCKs	0.1	0.42226	0.05	0.87572	0.3	0.02398
Transport of Mature mRNA derived from an Intron-Containing Transcript	0.0810811	0.43361	0.0945946	0.60725	0.2027027	0.0243

Deposition of new CENPA-containing nucleosomes at the centromere	0.0810811	0.43361	0.027027	0.99599	0.2027027	0.0243
Nucleosome assembly	0.0810811	0.43361	0.027027	0.99599	0.2027027	0.0243
Pyruvate metabolism and Citric Acid (TCA) cycle	0.0727273	0.55777	0.1090909	0.46494	0.2181818	0.02443
Formation of Incision Complex in GG-NER	0.1162791	0.1891	0.0930233	0.62684	0.2325581	0.02529
Macroautophagy	0.0735294	0.53806	0.1029412	0.51498	0.2058824	0.02556
DARPP-32 events	0.0384615	0.85401	0.0384615	0.93357	0.2692308	0.0273
Telomere Maintenance	0.0853659	0.36728	0.0609756	0.91857	0.195122	0.02851
Phase II - Conjugation of compounds	0.1009174	0.1527	0.1376147	0.11787	0.1834862	0.02898
Signaling by FGFR in disease	0.0634921	0.66591	0.0793651	0.75992	0.2063492	0.03022
Synthesis of substrates in N-glycan biosythesis	0.047619	0.83627	0.1428571	0.16649	0.2063492	0.03022
RNA Polymerase I Promoter Opening	0.0634921	0.66591	0.015873	0.99862	0.2063492	0.03022
ERCC6 (CSB) and EHMT2 (G9a) positively regulate rRNA expression	0.0657895	0.63828	0.0263158	0.99667	0.1973684	0.03031
Chromosome Maintenance	0.0909091	0.2573	0.0545455	0.96726	0.1818182	0.03164
SeMet incorporation into proteins	0.0909091	0.5567	0.1818182	0.29804	0.3636364	0.03217
Beta-catenin independent WNT signaling	0.0413793	0.95163	0.0689655	0.9186	0.1724138	0.03238
Homology Directed Repair	0.1086957	0.06633	0.0797101	0.81554	0.173913	0.03242
Cargo trafficking to the periciliary membrane	0.1372549	0.06823	0.0980392	0.57729	0.2156863	0.03292
Generation of second messenger molecules	0.030303	0.91311	0.0909091	0.64718	0.2424242	0.03441
Association of TriC/CCT with target proteins during biosynthesis	0.0512821	0.77709	0.1538462	0.18342	0.2307692	0.03451
Meiosis	0.0762712	0.46652	0.0338983	0.99802	0.1779661	0.03477
Intracellular signaling by second messengers	0.0716724	0.5222	0.0989761	0.52827	0.1535836	0.0375
DNA methylation	0.0615385	0.68992	0.0153846	0.99888	0.2	0.03806
TP53 Regulates Transcription of DNA Repair Genes	0.0461538	0.85151	0.0923077	0.63238	0.2	0.03806
Epigenetic regulation of gene expression	0.0945946	0.16918	0.0472973	0.99313	0.1689189	0.04025
E3 ubiquitin ligases ubiquitinate target proteins	0.0677966	0.61418	0.0847458	0.70666	0.2033898	0.04027
HSP90 chaperone cycle for steroid hormone receptors (SHR)	0.0566038	0.73867	0.0188679	0.99605	0.2075472	0.04243
HSF1 activation	NA	NA	0.1666667	0.33604	0.3333333	0.04385
PRC2 methylates histones and DNA	0.0684932	0.60229	0.0273973	0.9956	0.1917808	0.04418
Vesicle-mediated transport	0.0524738	0.98195	0.1154423	0.0814	0.1394303	0.04558
mTORC1-mediated signalling	0.0434783	0.81768	0.173913	0.1874	0.2608696	0.04603
BBSome-mediated cargo-targeting to cilium	0.173913	0.07674	0.0434783	0.90913	0.2608696	0.04603

Mitochondrial calcium ion transport	0.1304348	0.2229	0.1304348	0.40111	0.2608696	0.04603
PIP3 activates AKT signaling	0.0769231	0.39395	0.1076923	0.34554	0.1538462	0.04657
Energy dependent regulation of mTOR by LKB1-AMPK	0.0689655	0.62233	0.1724138	0.15309	0.2413793	0.04749
Activation of anterior HOX genes in hindbrain development during early embryogenesis	0.0491803	0.87512	0.0491803	0.98506	0.1721311	0.04763
Activation of HOX genes during differentiation	0.0491803	0.87512	0.0491803	0.98506	0.1721311	0.04763
