# Supplementary material for: Comparative transcriptional profiling of the early host response to infection by typhoidal and non-typhoidal Salmonella serovars in human intestinal organoids
Source: PLoS Pathog. 2021 Oct 20;17(10):e1009987. doi: 10.1371/journal.ppat.1009987 (PMC8570492; doi:10.1371/journal.ppat.1009987)
Supplement: S5 Table — Significantly upregulated DEGs from STM, SE or ST-infected HIOs at 8hpi were subjected to Reactome pathway analysis. (PDF) [file ppat.1009987.s013.pdf]

## Table S5: Reactome Pathways Upregulated 8h pi

| | STM | | SE | | ST | |
|---|---|---|---|---|---|---|
| **Description** | **gene ratio** | **p-value** | **gene ratio** | **p-value** | **gene ratio** | **p-value** |
| Cytokine Signaling in Immune system | 0.26889535 | 2.58E-28 | 0.255814 | 2.36E-23 | 0.1627907 | 3.57E-17 |
| Signaling by Interleukins | 0.28354978 | 2.67E-22 | 0.2662338 | 6.48E-18 | 0.1818182 | 7.52E-16 |
| Interleukin-4 and Interleukin-13 signaling | 0.38888889 | 7.97E-13 | 0.2222222 | 0.00249 | 0.1759259 | 0.00023 |
| Extracellular matrix organization | 0.23920266 | 4.99E-09 | 0.0996678 | 0.90882 | 0.0498339 | 0.95334 |
| Toll Like Receptor 3 (TLR3) Cascade | 0.33333333 | 2.17E-08 | 0.2828283 | 1.33E-05 | 0.2525253 | 1.96E-08 |
| MyD88-independent TLR4 cascade | 0.33 | 2.87E-08 | 0.28 | 1.64E-05 | 0.25 | 2.44E-08 |
| TRIF(TICAM1)-mediated TLR4 signaling | 0.33 | 2.87E-08 | 0.28 | 1.64E-05 | 0.25 | 2.44E-08 |
| Toll Like Receptor 4 (TLR4) Cascade | 0.3 | 3.24E-08 | 0.2769231 | 1.42E-06 | 0.2230769 | 2.99E-08 |
| Rab regulation of trafficking | 0.2983871 | 8.49E-08 | 0.1774194 | 0.04625 | 0.2258065 | 3.91E-08 |
| Interleukin-10 signaling | 0.42553191 | 1.41E-07 | 0.2978723 | 0.00109 | 0.2978723 | 3.21E-06 |
| Vesicle-mediated transport | 0.1874063 | 1.62E-07 | 0.1589205 | 0.00244 | 0.131934 | 1.10E-08 |
| Interferon gamma signaling | 0.32608696 | 1.64E-07 | 0.25 | 0.00056 | 0.2065217 | 2.33E-05 |
| Membrane Trafficking | 0.18949045 | 1.78E-07 | 0.1624204 | 0.00144 | 0.138535 | 1.22E-09 |
| Signaling by Receptor Tyrosine Kinases | 0.2021978 | 2.52E-07 | 0.1692308 | 0.00177 | 0.1208791 | 8.83E-05 |
| Toll Like Receptor 7/8 (TLR7/8) Cascade | 0.31914894 | 2.80E-07 | 0.287234 | 1.39E-05 | 0.2446809 | 1.35E-07 |
| MyD88 dependent cascade initiated on endosome | 0.31914894 | 2.80E-07 | 0.287234 | 1.39E-05 | 0.2446809 | 1.35E-07 |
| Toll Like Receptor 10 (TLR10) Cascade | 0.32941176 | 3.33E-07 | 0.3294118 | 4.68E-07 | 0.2588235 | 8.64E-08 |
| Toll Like Receptor 5 (TLR5) Cascade | 0.32941176 | 3.33E-07 | 0.3294118 | 4.68E-07 | 0.2588235 | 8.64E-08 |
| MyD88 cascade initiated on plasma membrane | 0.32941176 | 3.33E-07 | 0.3294118 | 4.68E-07 | 0.2588235 | 8.64E-08 |
| MyD88:MAL(TIRAP) cascade initiated on plasma membrane | 0.31578947 | 3.62E-07 | 0.3052632 | 1.74E-06 | 0.2526316 | 3.73E-08 |
| Toll Like Receptor TLR6:TLR2 Cascade | 0.31578947 | 3.62E-07 | 0.3052632 | 1.74E-06 | 0.2526316 | 3.73E-08 |
| MAPK family signaling cascades | 0.221843 | 5.50E-07 | 0.21843 | 2.10E-06 | 0.105802 | 0.01928 |
| Toll-like Receptor Cascades | 0.26451613 | 6.55E-07 | 0.2645161 | 1.01E-06 | 0.1935484 | 4.85E-07 |
| Interleukin-1 family signaling | 0.27338129 | 6.86E-07 | 0.3884892 | 8.89E-16 | 0.2014388 | 4.98E-07 |
| TRAF6 mediated induction of NFkB and MAP kinases upon TLR7/8 or 9 activation | 0.31182796 | 7.61E-07 | 0.2903226 | 1.12E-05 | 0.2473118 | 1.10E-07 |
| Toll Like Receptor 9 (TLR9) Cascade | 0.30612245 | 7.66E-07 | 0.2959184 | 3.51E-06 | 0.2346939 | 3.06E-07 |
| Toll Like Receptor TLR1:TLR2 Cascade | 0.30612245 | 7.66E-07 | 0.2959184 | 3.51E-06 | 0.244898 | 7.11E-08 |
| Toll Like Receptor 2 (TLR2) Cascade | 0.30612245 | 7.66E-07 | 0.2959184 | 3.51E-06 | 0.244898 | 7.11E-08 |
| Clathrin-mediated endocytosis | 0.26428571 | 2.33E-06 | 0.1857143 | 0.01916 | 0.1571429 | 0.0004 |
| Interleukin-1 signaling | 0.29126214 | 2.44E-06 | 0.4466019 | 2.17E-16 | 0.2135922 | 3.01E-06 |
| DDX58/IFIH1-mediated induction of interferon-alpha/beta | 0.32051282 | 2.49E-06 | 0.2179487 | 0.01212 | 0.1666667 | 0.0035 |

| | | | | | | |
|---|---|---|---|---|---|---|
| Death Receptor Signalling | 0.26241135 | 2.80E-06 | 0.1702128 | 0.05841 | 0.1631206 | 0.00017 |
| Interleukin-6 signaling | 0.72727273 | 5.14E-06 | 0.5454545 | 0.00089 | 0.3636364 | 0.00582 |
| MAPK1/MAPK3 signaling | 0.21653543 | 8.75E-06 | 0.2322835 | 6.33E-07 | 0.0944882 | 0.10222 |
| TBC/RABGAPs | 0.36956522 | 1.22E-05 | 0.1956522 | 0.10289 | 0.2391304 | 0.00032 |
| RAB GEFs exchange GTP for GDP on RABs | 0.28888889 | 1.33E-05 | 0.1888889 | 0.04451 | 0.2333333 | 1.10E-06 |
| TNFR2 non-canonical NF-kB pathway | 0.2745098 | 1.80E-05 | 0.4313725 | 4.69E-15 | 0.1666667 | 0.0009 |
| Syndecan interactions | 0.44444444 | 2.73E-05 | 0.2592593 | 0.0398 | 0.1111111 | 0.30678 |
| Interleukin-12 family signaling | 0.32758621 | 2.79E-05 | 0.4310345 | 3.96E-09 | 0.2413793 | 4.54E-05 |
| MAP kinase activation | 0.3125 | 3.75E-05 | 0.3125 | 4.76E-05 | 0.25 | 8.11E-06 |
| RAF/MAP kinase cascade | 0.20967742 | 3.86E-05 | 0.2298387 | 1.41E-06 | 0.0887097 | 0.17921 |
| Adaptive Immune System | 0.16798942 | 4.20E-05 | 0.1957672 | 1.48E-09 | 0.1177249 | 1.66E-06 |
| Glycosaminoglycan metabolism | 0.25 | 4.89E-05 | 0.1209677 | 0.56248 | 0.0645161 | 0.67627 |
| RIP-mediated NFkB activation via ZBP1 | 0.52941176 | 5.13E-05 | 0.3529412 | 0.01249 | 0.3529412 | 0.00084 |
| TAK1 activates NFkB by phosphorylation and activation of IKKs complex | 0.39393939 | 5.94E-05 | 0.3030303 | 0.00477 | 0.2727273 | 0.00039 |
| ZBP1(DAI) mediated induction of type I IFNs | 0.47619048 | 6.27E-05 | 0.2857143 | 0.03576 | 0.2857143 | 0.00289 |
| Interleukin-17 signaling | 0.29166667 | 7.55E-05 | 0.3055556 | 2.99E-05 | 0.2361111 | 9.75E-06 |
| Diseases of signal transduction | 0.18783069 | 7.76E-05 | 0.2195767 | 4.99E-08 | 0.0925926 | 0.07273 |
| Transcriptional regulation of white adipocyte differentiation | 0.27380952 | 0.0001 | 0.1547619 | 0.22443 | 0.1547619 | 0.00668 |
| Signaling by TGF-beta family members | 0.25490196 | 0.00014 | 0.1862745 | 0.03983 | 0.0980392 | 0.19712 |
| Regulation of lipid metabolism by Peroxisome proliferator-activated receptor alpha (PPARalpha) | 0.24369748 | 0.00014 | 0.1428571 | 0.28622 | 0.1932773 | 1.06E-05 |
| Interferon Signaling | 0.21319797 | 0.00014 | 0.2538071 | 2.72E-07 | 0.1319797 | 0.00187 |
| Circadian Clock | 0.28571429 | 0.00015 | 0.2285714 | 0.0093 | 0.1714286 | 0.00389 |
| Elastic fibre formation | 0.33333333 | 0.00015 | 0.1333333 | 0.47917 | 0.0222222 | 0.96546 |
| SUMOylation of transcription cofactors | 0.33333333 | 0.00015 | 0.2666667 | 0.00654 | 0.1333333 | 0.10137 |
| Integrin alphaIIb beta3 signaling | 0.40740741 | 0.00015 | 0.2222222 | 0.10407 | 0.1851852 | 0.04084 |
| Integrin signaling | 0.40740741 | 0.00015 | 0.2222222 | 0.10407 | 0.1851852 | 0.04084 |
| RAF-independent MAPK1/3 activation | 0.43478261 | 0.00016 | 0.3043478 | 0.01694 | 0.2608696 | 0.00474 |
| Nucleotide-binding domain, leucine rich repeat containing receptor (NLR) signaling pathways | 0.30909091 | 0.00016 | 0.2909091 | 0.00065 | 0.1636364 | 0.01572 |
| NOD1/2 Signaling Pathway | 0.36111111 | 0.00017 | 0.25 | 0.02639 | 0.2222222 | 0.00343 |
| p75NTR signals via NF-kB | 0.5 | 0.00023 | 0.25 | 0.12297 | 0.1875 | 0.10296 |
| L1CAM interactions | 0.23931624 | 0.00025 | 0.2051282 | 0.00727 | 0.1196581 | 0.04088 |

| | | | | | | |
|---|---|---|---|---|---|---|
| Interleukin-6 family signaling | 0.41666667 | 0.00025 | 0.2916667 | 0.02146 | 0.25 | 0.00595 |
| TRAF6 mediated NF-kB activation | 0.41666667 | 0.00025 | 0.25 | 0.06467 | 0.25 | 0.00595 |
| Cargo recognition for clathrin-mediated endocytosis | 0.25 | 0.00026 | 0.2 | 0.01762 | 0.16 | 0.00196 |
| Signaling by NOTCH1 | 0.2739726 | 0.00028 | 0.1917808 | 0.05761 | 0.1369863 | 0.0353 |
| Ovarian tumor domain proteases | 0.34210526 | 0.00031 | 0.2368421 | 0.03661 | 0.1842105 | 0.01719 |
| Platelet activation, signaling and aggregation | 0.19465649 | 0.00032 | 0.1564885 | 0.05797 | 0.0839695 | 0.25368 |
| TNF signaling | 0.3255814 | 0.00034 | 0.255814 | 0.0125 | 0.2325581 | 0.00076 |
| Interleukin-12 signaling | 0.3125 | 0.00034 | 0.3958333 | 1.43E-06 | 0.25 | 0.00011 |
| Hemostasis | 0.16612903 | 0.00035 | 0.1306452 | 0.28001 | 0.0758065 | 0.37275 |
| Interferon alpha/beta signaling | 0.27536232 | 0.00037 | 0.2173913 | 0.01819 | 0.1884058 | 0.00111 |
| Negative regulators of DDX58/IFIH1 signaling | 0.35294118 | 0.00038 | 0.2352941 | 0.04908 | 0.1764706 | 0.0323 |
| Axon guidance | 0.16878403 | 0.00039 | 0.2232305 | 7.49E-12 | 0.0889292 | 0.06954 |
| Signal attenuation | 0.6 | 0.00041 | 0.2 | 0.35149 | 0.2 | 0.15846 |
| Non-integrin membrane-ECM interactions | 0.28813559 | 0.00042 | 0.1355932 | 0.43738 | 0.0847458 | 0.42033 |
| PIP3 activates AKT signaling | 0.19230769 | 0.00049 | 0.2307692 | 6.45E-07 | 0.1230769 | 0.00192 |
| Signaling by NTRKs | 0.24242424 | 0.00055 | 0.1818182 | 0.05463 | 0.1515152 | 0.00457 |
| Regulation of TP53 Activity through Association with Co-factors | 0.5 | 0.00058 | 0.0714286 | 0.83973 | 0.2142857 | 0.07436 |
| Regulation of IFNG signaling | 0.5 | 0.00058 | 0.5 | 0.00064 | 0.2857143 | 0.01484 |
| PPARA activates gene expression | 0.23076923 | 0.00059 | 0.1452991 | 0.26245 | 0.1880342 | 2.58E-05 |
| RORA activates gene expression | 0.44444444 | 0.00062 | 0.1666667 | 0.38207 | 0.2222222 | 0.03611 |
| Cell-extracellular matrix interactions | 0.44444444 | 0.00062 | 0.2777778 | 0.06 | 0.0555556 | 0.73933 |
| TNF receptor superfamily (TNFSF) members mediating non-canonical NF-kB pathway | 0.44444444 | 0.00062 | 0.2222222 | 0.17062 | 0.2222222 | 0.03611 |
| Assembly of collagen fibrils and other multimeric structures | 0.27868852 | 0.00064 | 0.1147541 | 0.63211 | 0.0655738 | 0.64803 |
| Signaling by NTRK1 (TRKA) | 0.25641026 | 0.00072 | 0.2051282 | 0.02536 | 0.1666667 | 0.0035 |
| Interleukin-27 signaling | 0.54545455 | 0.00082 | 0.6363636 | 8.59E-05 | 0.2727273 | 0.03954 |
| Integrin cell surface interactions | 0.24705882 | 0.00091 | 0.1411765 | 0.34592 | 0.0470588 | 0.86925 |
| Smooth Muscle Contraction | 0.32432432 | 0.00092 | 0.2162162 | 0.07537 | 0.027027 | 0.93711 |
| NOTCH1 Intracellular Domain Regulates Transcription | 0.29787234 | 0.00092 | 0.1914894 | 0.11419 | 0.1276596 | 0.11878 |
| Cytosolic sensors of pathogen-associated DNA | 0.26984127 | 0.00096 | 0.2539683 | 0.00313 | 0.1587302 | 0.01377 |
| YAP1- and WWTR1 (TAZ)-stimulated gene expression | 0.46666667 | 0.00097 | 0.0666667 | 0.85939 | 0.2 | 0.08817 |
| IGF1R signaling cascade | 0.28301887 | 0.0011 | 0.1509434 | 0.32077 | 0.1320755 | 0.08371 |
| Regulation of TNFR1 signaling | 0.33333333 | 0.00116 | 0.2121212 | 0.10095 | 0.2121212 | 0.00794 |
| Chemokine receptors bind chemokines | 0.29166667 | 0.00116 | 0.2916667 | 0.00136 | 0.2916667 | 4.24E-06 |

| | | | | | | |
|---|---|---|---|---|---|---|
| Molecules associated with elastic fibres | 0.31578947 | 0.0012 | 0.1052632 | 0.70103 | 0.0263158 | 0.94165 |
| Diseases of Immune System | 0.375 | 0.00126 | 0.3333333 | 0.00599 | 0.2083333 | 0.02566 |
| Diseases associated with the TLR signaling cascade | 0.375 | 0.00126 | 0.3333333 | 0.00599 | 0.2083333 | 0.02566 |
| Growth hormone receptor signaling | 0.375 | 0.00126 | 0.2916667 | 0.02146 | 0.0833333 | 0.52335 |
| Signaling by Type 1 Insulin-like Growth Factor 1 Receptor (IGF1R) | 0.27777778 | 0.00136 | 0.1481481 | 0.33998 | 0.1296296 | 0.09054 |
| Semaphorin interactions | 0.26153846 | 0.0014 | 0.1538462 | 0.26956 | 0.0615385 | 0.69703 |
| TNFs bind their physiological receptors | 0.34482759 | 0.00144 | 0.2413793 | 0.05644 | 0.1724138 | 0.05344 |
| COPI-independent Golgi-to-ER retrograde traffic | 0.28571429 | 0.00145 | 0.3877551 | 2.07E-06 | 0.1428571 | 0.05948 |
| NOTCH2 intracellular domain regulates transcription | 0.5 | 0.00147 | 0.0833333 | 0.79179 | 0.0833333 | 0.59183 |
| Interleukin-35 Signalling | 0.5 | 0.00147 | 0.5833333 | 0.00018 | 0.25 | 0.04998 |
| TICAM1-dependent activation of IRF3/IRF7 | 0.5 | 0.00147 | 0.25 | 0.17425 | 0.25 | 0.04998 |
| Intracellular signaling by second messengers | 0.18088737 | 0.00148 | 0.2116041 | 8.89E-06 | 0.1228669 | 0.00107 |
| Transcriptional activity of SMAD2/SMAD3:SMAD4 heterotrimer | 0.29545455 | 0.00151 | 0.1590909 | 0.28998 | 0.1363636 | 0.0932 |
| Negative regulation of the PI3K/AKT network | 0.22641509 | 0.00153 | 0.1698113 | 0.09333 | 0.1320755 | 0.01932 |
| Platelet Aggregation (Plug Formation) | 0.30769231 | 0.00154 | 0.1538462 | 0.34215 | 0.1282051 | 0.14555 |
| Gene and protein expression by JAK-STAT signaling after Interleukin-12 stimulation | 0.30769231 | 0.00154 | 0.4102564 | 5.60E-06 | 0.2564103 | 0.00033 |
| Regulation of cholesterol biosynthesis by SREBP (SREBF) | 0.27272727 | 0.00167 | 0.2 | 0.06713 | 0.3454545 | 3.62E-09 |
| Transcriptional Regulation by MECP2 | 0.27272727 | 0.00167 | 0.1272727 | 0.51832 | 0.1090909 | 0.20127 |
| CLEC7A (Dectin-1) signaling | 0.22772277 | 0.00175 | 0.3960396 | 2.38E-12 | 0.1782178 | 0.00028 |
| Signaling by VEGF | 0.22429907 | 0.00175 | 0.1682243 | 0.10001 | 0.1495327 | 0.00395 |
| Nuclear Events (kinase and transcription factor activation) | 0.36 | 0.00176 | 0.24 | 0.07665 | 0.28 | 0.00148 |
| Recycling pathway of L1 | 0.28888889 | 0.0019 | 0.2444444 | 0.01757 | 0.2 | 0.00415 |
| RHO GTPases activate IQGAPs | 0.33333333 | 0.00193 | 0.3333333 | 0.00218 | 0.2333333 | 0.00457 |
| Regulation of MECP2 expression and activity | 0.33333333 | 0.00193 | 0.1 | 0.72942 | 0.1666667 | 0.06049 |
| Collagen formation | 0.23333333 | 0.00198 | 0.1 | 0.78883 | 0.0444444 | 0.8961 |
| p75 NTR receptor-mediated signalling | 0.22680412 | 0.0023 | 0.1340206 | 0.40954 | 0.1237113 | 0.04491 |
| NF-kB is activated and signals survival | 0.46153846 | 0.00245 | 0.2307692 | 0.2071 | 0.1538462 | 0.23919 |
| Dissolution of Fibrin Clot | 0.46153846 | 0.00245 | 0.1538462 | 0.48533 | 0.1538462 | 0.23919 |
| Erythropoietin activates RAS | 0.46153846 | 0.00245 | 0.3076923 | 0.06474 | 0.3846154 | 0.0015 |
| Metabolism of lipids | 0.15468114 | 0.00247 | 0.1289009 | 0.30864 | 0.1126187 | 2.10E-05 |
| HS-GAG biosynthesis | 0.32258065 | 0.00254 | 0.1290323 | 0.53702 | 0.0645161 | 0.66394 |
| RHO GTPases Activate WASPs and WAVEs | 0.30555556 | 0.00256 | 0.3055556 | 0.00291 | 0.2222222 | 0.00343 |

| | | | | | | |
|---|---|---|---|---|---|---|
| IRS-related events triggered by IGF1R | 0.26923077 | 0.00268 | 0.1346154 | 0.45688 | 0.1346154 | 0.07719 |
| Signaling by NOTCH1 PEST Domain Mutants in Cancer | 0.25862069 | 0.00294 | 0.2068966 | 0.04593 | 0.0862069 | 0.40579 |
| Signaling by NOTCH1 in Cancer | 0.25862069 | 0.00294 | 0.2068966 | 0.04593 | 0.0862069 | 0.40579 |
| Constitutive Signaling by NOTCH1 PEST Domain Mutants | 0.25862069 | 0.00294 | 0.2068966 | 0.04593 | 0.0862069 | 0.40579 |
| Signaling by NOTCH1 HD+PEST Domain Mutants in Cancer | 0.25862069 | 0.00294 | 0.2068966 | 0.04593 | 0.0862069 | 0.40579 |
| Constitutive Signaling by NOTCH1 HD+PEST Domain Mutants | 0.25862069 | 0.00294 | 0.2068966 | 0.04593 | 0.0862069 | 0.40579 |
| VEGFA-VEGFR2 Pathway | 0.22222222 | 0.00302 | 0.1818182 | 0.05463 | 0.1616162 | 0.00176 |
| PI5P, PP2A and IER3 Regulate PI3K/AKT Signaling | 0.22222222 | 0.00302 | 0.1616162 | 0.14969 | 0.1313131 | 0.02467 |
| Activation of gene expression by SREBF (SREBP) | 0.28571429 | 0.0031 | 0.1904762 | 0.1348 | 0.4047619 | 1.42E-09 |
| BMAL1:CLOCK,NPAS2 activates circadian gene expression | 0.33333333 | 0.00322 | 0.2222222 | 0.10407 | 0.2592593 | 0.00241 |
| Interleukin receptor SHC signaling | 0.33333333 | 0.00322 | 0.2592593 | 0.0398 | 0.1481481 | 0.12515 |
| Signaling by RAS mutants | 0.26415094 | 0.00325 | 0.1132075 | 0.64482 | 0.1132075 | 0.17892 |
| SMAD2/SMAD3:SMAD4 heterotrimer regulates transcription | 0.3125 | 0.0033 | 0.1875 | 0.19091 | 0.125 | 0.19516 |
| FCERI mediated NF-kB activation | 0.23170732 | 0.00345 | 0.4756098 | 3.13E-15 | 0.1707317 | 0.00197 |
| Interleukin-3, Interleukin-5 and GM-CSF signaling | 0.27083333 | 0.00355 | 0.2291667 | 0.02784 | 0.1875 | 0.00648 |
| Rho GTPase cycle | 0.20289855 | 0.00368 | 0.0797101 | 0.95972 | 0.1014493 | 0.12039 |
| MAPK3 (ERK1) activation | 0.5 | 0.00377 | 0.4 | 0.02561 | 0.4 | 0.00392 |
| SEMA3A-Plexin repulsion signaling by inhibiting Integrin adhesion | 0.42857143 | 0.00385 | 0.1428571 | 0.52602 | 0.1428571 | 0.26666 |
| RUNX3 regulates NOTCH signaling | 0.42857143 | 0.00385 | 0.0714286 | 0.83973 | NA | NA |
| Interleukin-15 signaling | 0.42857143 | 0.00385 | 0.2857143 | 0.08216 | 0.2142857 | 0.07436 |
| TRAF3-dependent IRF activation pathway | 0.42857143 | 0.00385 | 0.0714286 | 0.83973 | 0.1428571 | 0.26666 |
| Insulin receptor signalling cascade | 0.25925926 | 0.00391 | 0.1481481 | 0.33998 | 0.1296296 | 0.09054 |
| Signaling by PTK6 | 0.25925926 | 0.00391 | 0.1851852 | 0.11797 | 0.1296296 | 0.09054 |
| Signaling by Non-Receptor Tyrosine Kinases | 0.25925926 | 0.00391 | 0.1851852 | 0.11797 | 0.1296296 | 0.09054 |
| Signaling by BMP | 0.32142857 | 0.00425 | 0.1785714 | 0.25381 | 0.1428571 | 0.13819 |
| G-protein beta:gamma signalling | 0.32142857 | 0.00425 | 0.1785714 | 0.25381 | 0.0357143 | 0.87662 |
| Transcriptional regulation by RUNX3 | 0.21875 | 0.00451 | 0.3229167 | 1.91E-07 | 0.1354167 | 0.01959 |
| Degradation of the extracellular matrix | 0.2 | 0.00454 | 0.0857143 | 0.93476 | 0.05 | 0.88487 |
| Neutrophil degranulation | 0.16075157 | 0.00458 | 0.2296451 | 1.66E-11 | 0.1169102 | 0.00019 |
| MHC class II antigen presentation | 0.20661157 | 0.00458 | 0.214876 | 0.00278 | 0.107438 | 0.09431 |
| Heparan sulfate/heparin (HS-GAG) metabolism | 0.25454545 | 0.00468 | 0.0909091 | 0.82042 | 0.0363636 | 0.91377 |
| Activation of NMDA receptors and postsynaptic events | 0.27272727 | 0.00471 | 0.1818182 | 0.16372 | 0.1363636 | 0.0932 |
| Interleukin-2 family signaling | 0.27272727 | 0.00471 | 0.2045455 | 0.08232 | 0.1136364 | 0.20702 |
| Fc epsilon receptor (FCERI) signaling | 0.20149254 | 0.00474 | 0.358209 | 1.27E-12 | 0.1567164 | 0.00056 |

| | | | | | | |
|---|---|---|---|---|---|---|
| Signaling by TGF-beta Receptor Complex | 0.23287671 | 0.00522 | 0.1917808 | 0.05761 | 0.0958904 | 0.27031 |
| Signaling by MET | 0.2278481 | 0.00526 | 0.2405063 | 0.00265 | 0.1392405 | 0.02522 |
| activated TAK1 mediates p38 MAPK activation | 0.33333333 | 0.00541 | 0.2916667 | 0.02146 | 0.2916667 | 0.00113 |
| Signaling by Erythropoietin | 0.33333333 | 0.00541 | 0.25 | 0.06467 | 0.2083333 | 0.02566 |
| Transcriptional activation of mitochondrial biogenesis | 0.25 | 0.00556 | 0.1964286 | 0.07473 | 0.1964286 | 0.00186 |
| C-type lectin receptors (CLRs) | 0.1971831 | 0.00557 | 0.3028169 | 7.72E-09 | 0.1549296 | 0.00049 |
| Regulated proteolysis of p75NTR | 0.45454545 | 0.00623 | 0.2727273 | 0.14305 | 0.1818182 | 0.18489 |
| Dermatan sulfate biosynthesis | 0.45454545 | 0.00623 | 0.1818182 | 0.39781 | 0.0909091 | 0.56017 |
| Signaling by Leptin | 0.45454545 | 0.00623 | 0.1818182 | 0.39781 | 0.0909091 | 0.56017 |
| SUMOylation of immune response proteins | 0.45454545 | 0.00623 | 0.6363636 | 8.59E-05 | 0.2727273 | 0.03954 |
| RHO GTPases activate KTN1 | 0.45454545 | 0.00623 | 0.4545455 | 0.00669 | 0.4545455 | 0.00061 |
| Erythropoietin activates Phosphoinositide-3-kinase (PI3K) | 0.45454545 | 0.00623 | 0.3636364 | 0.03642 | 0.1818182 | 0.18489 |
| Signaling by moderate kinase activity BRAF mutants | 0.275 | 0.00626 | 0.15 | 0.36508 | 0.15 | 0.06428 |
| Paradoxical activation of RAF signaling by kinase inactive BRAF | 0.275 | 0.00626 | 0.15 | 0.36508 | 0.15 | 0.06428 |
| GPVI-mediated activation cascade | 0.28571429 | 0.00672 | 0.2285714 | 0.05706 | 0.1428571 | 0.10316 |
| NOTCH4 Intracellular Domain Regulates Transcription | 0.35 | 0.0068 | NA | NA | 0.05 | 0.77554 |
| Signaling by the B Cell Receptor (BCR) | 0.20535714 | 0.00688 | 0.4017857 | 5.40E-14 | 0.1785714 | 0.00013 |
| Laminin interactions | 0.3 | 0.00705 | 0.1666667 | 0.30338 | 0.0666667 | 0.6461 |
| TNFR1-induced NFkappaB signaling pathway | 0.3 | 0.00705 | 0.2 | 0.15333 | 0.2 | 0.01817 |
| G beta:gamma signalling through PI3Kgamma | 0.32 | 0.00714 | 0.2 | 0.18408 | 0.04 | 0.84557 |
| TCR signaling | 0.20168067 | 0.00738 | 0.3781513 | 7.07E-13 | 0.1176471 | 0.04614 |
| Diseases associated with glycosaminoglycan metabolism | 0.26829268 | 0.00764 | 0.1707317 | 0.23096 | 0.0487805 | 0.80471 |
| MET promotes cell motility | 0.26829268 | 0.00764 | 0.195122 | 0.1214 | 0.1463415 | 0.07094 |
| Collagen degradation | 0.234375 | 0.00791 | 0.09375 | 0.8124 | 0.0625 | 0.68525 |
| Oncogenic MAPK signaling | 0.22368421 | 0.00797 | 0.1184211 | 0.59611 | 0.1052632 | 0.17841 |
| MAPK targets/ Nuclear events mediated by MAP kinases | 0.29032258 | 0.0089 | 0.2580645 | 0.0296 | 0.2258065 | 0.00554 |
| Arachidonic acid metabolism | 0.23728814 | 0.00904 | 0.1525424 | 0.29357 | 0.1355932 | 0.05918 |
| Synthesis of Leukotrienes (LT) and Eoxins (EX) | 0.33333333 | 0.00915 | 0.0952381 | 0.74756 | 0.1428571 | 0.18874 |
| Signal transduction by L1 | 0.33333333 | 0.00915 | 0.2857143 | 0.03576 | 0.1428571 | 0.18874 |
| SHC-mediated cascade:FGFR1 | 0.33333333 | 0.00915 | 0.1428571 | 0.48426 | 0.2380952 | 0.01468 |
| TP53 Regulates Transcription of Death Receptors and Ligands | 0.41666667 | 0.00963 | 0.1666667 | 0.44253 | 0.0833333 | 0.59183 |
| Interleukin-2 signaling | 0.41666667 | 0.00963 | 0.25 | 0.17425 | 0.1666667 | 0.21187 |
| Signaling by Insulin receptor | 0.21794872 | 0.01038 | 0.1794872 | 0.09036 | 0.1282051 | 0.05204 |
| PI3K/AKT Signaling in Cancer | 0.20618557 | 0.01076 | 0.1443299 | 0.2976 | 0.1237113 | 0.04491 |

| | | | | | | |
|---|---|---|---|---|---|---|
| CREB phosphorylation through the activation of Ras | 0.28125 | 0.01108 | 0.21875 | 0.08842 | 0.15625 | 0.0761 |
| Neurotransmitter receptors and postsynaptic signal transmission | 0.18589744 | 0.01113 | 0.1153846 | 0.64453 | 0.0641026 | 0.69314 |
| IL-6-type cytokine receptor ligand interactions | 0.35294118 | 0.01155 | 0.2352941 | 0.14604 | 0.2941176 | 0.00567 |
| Metabolism of steroids | 0.18666667 | 0.0118 | 0.16 | 0.10226 | 0.1666667 | 6.24E-05 |
| Downstream TCR signaling | 0.20408163 | 0.01203 | 0.4285714 | 2.60E-14 | 0.122449 | 0.04799 |
| ERK/MAPK targets | 0.31818182 | 0.01205 | 0.1818182 | 0.27988 | 0.2272727 | 0.0179 |
| Regulation of actin dynamics for phagocytic cup formation | 0.2295082 | 0.01217 | 0.2459016 | 0.00576 | 0.2131148 | 0.00032 |
| EPH-Ephrin signaling | 0.20652174 | 0.0125 | 0.2065217 | 0.01472 | 0.1521739 | 0.0058 |
| PI3K Cascade | 0.25 | 0.01325 | 0.1136364 | 0.6403 | 0.1136364 | 0.20702 |
| Signaling by NOTCH2 | 0.27272727 | 0.01365 | 0.1515152 | 0.37981 | 0.0606061 | 0.69744 |
| Chondroitin sulfate/dermatan sulfate metabolism | 0.24 | 0.01376 | 0.14 | 0.41501 | 0.04 | 0.88383 |
| p75NTR recruits signalling complexes | 0.38461538 | 0.01412 | 0.1538462 | 0.48533 | 0.1538462 | 0.23919 |
| Loss of function of MECP2 in Rett syndrome | 0.38461538 | 0.01412 | 0.0769231 | 0.81732 | 0.2307692 | 0.0616 |
| Pervasive developmental disorders | 0.38461538 | 0.01412 | 0.0769231 | 0.81732 | 0.2307692 | 0.0616 |
| JNK (c-Jun kinases) phosphorylation and activation mediated by activated human TAK1 | 0.30434783 | 0.01557 | 0.3043478 | 0.01694 | 0.2608696 | 0.00474 |
| FRS-mediated FGFR1 signaling | 0.30434783 | 0.01557 | 0.0869565 | 0.7919 | 0.2173913 | 0.02155 |
| Gastrin-CREB signalling pathway via PKC and MAPK | 0.33333333 | 0.01559 | 0.2222222 | 0.17062 | 0.3888889 | 0.00015 |
| Activation of IRF3/IRF7 mediated by TBK1/IKK epsilon | 0.33333333 | 0.01559 | 0.1666667 | 0.38207 | 0.1111111 | 0.37513 |
| EPH-ephrin mediated repulsion of cells | 0.23529412 | 0.01608 | 0.1372549 | 0.436 | 0.1372549 | 0.07098 |
| Signaling by NOTCH4 | 0.20731707 | 0.01691 | 0.4146341 | 2.34E-11 | 0.1097561 | 0.13355 |
| Post NMDA receptor activation events | 0.25 | 0.01779 | 0.175 | 0.21226 | 0.15 | 0.06428 |
| Costimulation by the CD28 family | 0.21428571 | 0.018 | 0.2 | 0.04243 | 0.1285714 | 0.06237 |
| Transport to the Golgi and subsequent modification | 0.17486339 | 0.01885 | 0.1693989 | 0.03733 | 0.0819672 | 0.33716 |
| Downstream signaling events of B Cell Receptor (BCR) | 0.20481928 | 0.01895 | 0.4578313 | 3.35E-14 | 0.1566265 | 0.00603 |
| Tryptophan catabolism | 0.35714286 | 0.01983 | 0.2142857 | 0.24118 | 0.2142857 | 0.07436 |
| PTK6 Regulates RHO GTPases, RAS GTPase and MAP kinases | 0.35714286 | 0.01983 | 0.2857143 | 0.08216 | 0.2142857 | 0.07436 |
| RET signaling | 0.24390244 | 0.02107 | 0.097561 | 0.75701 | 0.1219512 | 0.16913 |
| PI Metabolism | 0.20238095 | 0.02118 | 0.1190476 | 0.58885 | 0.0833333 | 0.40043 |
| HSP90 chaperone cycle for steroid hormone receptors (SHR) | 0.22641509 | 0.02158 | 0.3962264 | 3.94E-07 | 0.1320755 | 0.08371 |
| Activation of BH3-only proteins | 0.26666667 | 0.02241 | 0.3 | 0.00785 | 0.2 | 0.01817 |
| Regulation of TP53 Activity through Acetylation | 0.26666667 | 0.02241 | 0.1 | 0.72942 | 0.0333333 | 0.89377 |
| Hedgehog 'off' state | 0.18918919 | 0.02325 | 0.3603604 | 7.73E-11 | 0.0990991 | 0.17372 |

| | | | | | | |
|---|---|---|---|---|---|---|
| Diseases of glycosylation | 0.18115942 | 0.0236 | 0.115942 | 0.63277 | 0.0434783 | 0.93819 |
| Signaling by high-kinase activity BRAF mutants | 0.25 | 0.02398 | 0.1388889 | 0.45597 | 0.1111111 | 0.25791 |
| Interleukin-21 signaling | 0.4 | 0.02423 | 0.4 | 0.02561 | 0.2 | 0.15846 |
| IRAK2 mediated activation of TAK1 complex | 0.4 | 0.02423 | 0.3 | 0.11397 | 0.4 | 0.00392 |
| ER to Golgi Anterograde Transport | 0.17763158 | 0.02431 | 0.1710526 | 0.04796 | 0.0723684 | 0.53685 |
| Signaling by BRAF and RAF fusions | 0.21666667 | 0.02436 | 0.15 | 0.31118 | 0.1166667 | 0.13772 |
| Sema4D in semaphorin signaling | 0.28 | 0.02465 | 0.2 | 0.18408 | 0.08 | 0.54568 |
| Interleukin-20 family signaling | 0.28 | 0.02465 | 0.24 | 0.07665 | 0.12 | 0.26653 |
| NOTCH3 Intracellular Domain Regulates Transcription | 0.28 | 0.02465 | 0.08 | 0.82921 | NA | NA |
| Antigen Presentation: Folding, assembly and peptide loading of class I MHC | 0.28 | 0.02465 | 0.28 | 0.02674 | 0.24 | 0.00736 |
| G alpha (12/13) signalling events | 0.20253165 | 0.02471 | 0.0759494 | 0.93332 | 0.0506329 | 0.82936 |
| IRS-mediated signalling | 0.22916667 | 0.02498 | 0.125 | 0.54474 | 0.125 | 0.128 |
| Signalling to RAS | 0.3 | 0.0264 | 0.2 | 0.22349 | 0.2 | 0.05105 |
| TICAM1, RIP1-mediated IKK complex recruitment | 0.3 | 0.0264 | 0.3 | 0.0284 | 0.25 | 0.01187 |
| Signaling by Hippo | 0.3 | 0.0264 | 0.15 | 0.45099 | 0.1 | 0.427 |
| Receptor-type tyrosine-protein phosphatases | 0.3 | 0.0264 | NA | NA | 0.15 | 0.17029 |
| Unblocking of NMDA receptors, glutamate binding and activation | 0.3 | 0.0264 | 0.15 | 0.45099 | 0.05 | 0.77554 |
| TP53 Regulates Transcription of Genes Involved in Cytochrome C Release | 0.3 | 0.0264 | 0.25 | 0.08858 | 0.15 | 0.17029 |
| Activation of NF-kappaB in B cells | 0.20895522 | 0.02677 | 0.5074627 | 1.64E-14 | 0.1343284 | 0.04948 |
| Synthesis of bile acids and bile salts via 27-hydroxycholesterol | 0.33333333 | 0.02686 | 0.2 | 0.27607 | 0.1333333 | 0.29412 |
| GRB2:SOS provides linkage to MAPK signaling for Integrins | 0.33333333 | 0.02686 | 0.1333333 | 0.56448 | 0.2 | 0.08817 |
| p130Cas linkage to MAPK signaling for integrins | 0.33333333 | 0.02686 | 0.2 | 0.27607 | 0.1333333 | 0.29412 |
| MECP2 regulates neuronal receptors and channels | 0.33333333 | 0.02686 | 0.2 | 0.27607 | 0.1333333 | 0.29412 |
| Trafficking of AMPA receptors | 0.25806452 | 0.02711 | 0.1290323 | 0.53702 | 0.0645161 | 0.66394 |
| Glutamate binding, activation of AMPA receptors and synaptic plasticity | 0.25806452 | 0.02711 | 0.1290323 | 0.53702 | 0.0645161 | 0.66394 |
| Uptake and actions of bacterial toxins | 0.25806452 | 0.02711 | 0.2903226 | 0.00988 | 0.1935484 | 0.0212 |
| Downstream signaling of activated FGFR1 | 0.25806452 | 0.02711 | 0.1612903 | 0.3287 | 0.1612903 | 0.06804 |
| Defective B3GALTL causes Peters-plus syndrome (PpS) | 0.24324324 | 0.02841 | 0.0810811 | 0.8484 | 0.027027 | 0.93711 |
| TP53 Regulates Transcription of Cell Cycle Genes | 0.2244898 | 0.02882 | 0.1020408 | 0.73347 | 0.122449 | 0.13757 |
| Signaling by SCF-KIT | 0.23255814 | 0.02889 | 0.1627907 | 0.26989 | 0.1627907 | 0.03222 |
| Signaling by EGFR | 0.23255814 | 0.02889 | 0.3023256 | 0.00139 | 0.1162791 | 0.19409 |

| | | | | | | |
|---|---|---|---|---|---|---|
| Other interleukin signaling | 0.26923077 | 0.03033 | 0.1538462 | 0.39672 | 0.1923077 | 0.03529 |
| Post-translational protein phosphorylation | 0.18518519 | 0.03239 | 0.1759259 | 0.06517 | 0.0648148 | 0.66705 |
| Thrombin signalling through proteinase activated receptors (PARs) | 0.25 | 0.03246 | 0.15625 | 0.35422 | 0.15625 | 0.0761 |
| RUNX2 regulates bone development | 0.25 | 0.03246 | 0.0625 | 0.91685 | 0.15625 | 0.0761 |
| Nuclear Receptor transcription pathway | 0.22 | 0.03307 | 0.04 | 0.98854 | 0.08 | 0.48889 |
| Presynaptic function of Kainate receptors | 0.28571429 | 0.0333 | 0.1428571 | 0.48426 | NA | NA |
| CD209 (DC-SIGN) signaling | 0.28571429 | 0.0333 | 0.1428571 | 0.48426 | 0.1428571 | 0.18874 |
| PI-3K cascade:FGFR1 | 0.28571429 | 0.0333 | 0.0952381 | 0.74756 | 0.1904762 | 0.05961 |
| TP53 regulates transcription of additional cell cycle genes whose exact role in the p53 pathway remain uncertain | 0.28571429 | 0.0333 | 0.1904762 | 0.25135 | 0.1904762 | 0.05961 |
| Negative regulation of MET activity | 0.28571429 | 0.0333 | 0.2857143 | 0.03576 | 0.1428571 | 0.18874 |
| Interleukin-37 signaling | 0.28571429 | 0.0333 | 0.2857143 | 0.03576 | 0.1904762 | 0.05961 |
| O-glycosylation of TSR domain-containing proteins | 0.23684211 | 0.03338 | 0.0789474 | 0.86103 | 0.0263158 | 0.94165 |
| Intrinsic Pathway for Apoptosis | 0.22727273 | 0.0335 | 0.3181818 | 0.00052 | 0.1363636 | 0.0932 |
| Plasma lipoprotein assembly, remodeling, and clearance | 0.20289855 | 0.03373 | 0.115942 | 0.62147 | 0.1884058 | 0.00111 |
| Regulation of gene expression by Hypoxia-inducible Factor | 0.36363636 | 0.03452 | 0.0909091 | 0.76268 | 0.1818182 | 0.18489 |
| Signaling by activated point mutants of FGFR1 | 0.36363636 | 0.03452 | NA | NA | 0.1818182 | 0.18489 |
| Type I hemidesmosome assembly | 0.36363636 | 0.03452 | 0.1818182 | 0.39781 | 0.1818182 | 0.18489 |
| MET activates RAP1 and RAC1 | 0.36363636 | 0.03452 | 0.3636364 | 0.03642 | 0.3636364 | 0.00582 |
| Activated NTRK2 signals through FRS2 and FRS3 | 0.36363636 | 0.03452 | 0.1818182 | 0.39781 | 0.1818182 | 0.18489 |
| Regulation of KIT signaling | 0.3125 | 0.03529 | 0.0625 | 0.87664 | 0.25 | 0.02407 |
| FGFR1 ligand binding and activation | 0.3125 | 0.03529 | 0.0625 | 0.87664 | 0.1875 | 0.10296 |
| Microtubule-dependent trafficking of connexons from Golgi to the plasma membrane | 0.3125 | 0.03529 | 0.375 | 0.009 | 0.125 | 0.32143 |
| Regulation of gene expression in late stage (branching morphogenesis) pancreatic bud precursor cells | 0.3125 | 0.03529 | NA | NA | NA | NA |
| Phospholipase C-mediated cascade: FGFR1 | 0.3125 | 0.03529 | 0.0625 | 0.87664 | 0.1875 | 0.10296 |
| MAPK6/MAPK4 signaling | 0.19101124 | 0.03544 | 0.3707865 | 1.51E-09 | 0.1460674 | 0.0108 |
| XBP1(S) activates chaperone genes | 0.21052632 | 0.03658 | 0.122807 | 0.55783 | 0.1052632 | 0.22457 |
| Endogenous sterols | 0.25925926 | 0.03682 | 0.1481481 | 0.42575 | 0.1111111 | 0.30678 |
| Intra-Golgi and retrograde Golgi-to-ER traffic | 0.165 | 0.03696 | 0.16 | 0.06759 | 0.1 | 0.08325 |
| Translocation of SLC2A4 (GLUT4) to the plasma membrane | 0.2 | 0.03767 | 0.3 | 6.09E-05 | 0.1571429 | 0.01081 |
| CD28 co-stimulation | 0.24242424 | 0.03847 | 0.2121212 | 0.10095 | 0.2121212 | 0.00794 |

| | | | | | | |
|---|---|---|---|---|---|---|
| Negative regulation of FGFR1 signaling | 0.24242424 | 0.03847 | 0.0909091 | 0.78746 | 0.1515152 | 0.08464 |
| Formation of Fibrin Clot (Clotting Cascade) | 0.23076923 | 0.03892 | 0.1282051 | 0.52929 | 0.0512821 | 0.7816 |
| RAB geranylgeranylation | 0.203125 | 0.03951 | 0.234375 | 0.00916 | 0.203125 | 0.00053 |
| Golgi-to-ER retrograde transport | 0.17557252 | 0.04003 | 0.1908397 | 0.01549 | 0.1145038 | 0.04888 |
| Beta-catenin independent WNT signaling | 0.17241379 | 0.04045 | 0.262069 | 3.18E-06 | 0.0896552 | 0.24329 |
| Signaling by PDGF | 0.20689655 | 0.04126 | 0.1896552 | 0.09148 | 0.1206897 | 0.12084 |
| Constitutive Signaling by Aberrant PI3K in Cancer | 0.1971831 | 0.04192 | 0.1126761 | 0.6543 | 0.1126761 | 0.13614 |
| Cell junction organization | 0.18681319 | 0.04276 | 0.1098901 | 0.69132 | 0.0879121 | 0.33078 |
| Regulation of Insulin-like Growth Factor (IGF) transport and uptake by Insulin-like Growth Factor Binding Proteins (IGFBPs) | 0.176 | 0.04287 | 0.16 | 0.12695 | 0.064 | 0.685 |
| G alpha (q) signalling events | 0.16190476 | 0.04385 | 0.0857143 | 0.9651 | 0.0904762 | 0.17775 |
| MAP2K and MAPK activation | 0.225 | 0.04505 | 0.15 | 0.36508 | 0.1 | 0.32391 |
| Transport of connexons to the plasma membrane | 0.29411765 | 0.04518 | 0.4117647 | 0.0026 | 0.1764706 | 0.11866 |
| Regulation of RUNX1 Expression and Activity | 0.29411765 | 0.04518 | 0.0588235 | 0.89178 | 0.0588235 | 0.7191 |
| TICAM1,TRAF6-dependent induction of TAK1 complex | 0.29411765 | 0.04518 | 0.1764706 | 0.34683 | 0.2941176 | 0.00567 |
| IRAK2 mediated activation of TAK1 complex upon TLR7/8 or 9 stimulation | 0.29411765 | 0.04518 | 0.1764706 | 0.34683 | 0.2941176 | 0.00567 |
| IRE1alpha activates chaperones | 0.20338983 | 0.04634 | 0.1186441 | 0.59584 | 0.1016949 | 0.2487 |
| PTEN Regulation | 0.17142857 | 0.04659 | 0.2785714 | 4.42E-07 | 0.1 | 0.13086 |
| FGFR1c ligand binding and activation | 0.33333333 | 0.04696 | NA | NA | 0.1666667 | 0.21187 |
| Caspase-mediated cleavage of cytoskeletal proteins | 0.33333333 | 0.04696 | NA | NA | 0.0833333 | 0.59183 |
| COPI-mediated anterograde transport | 0.18181818 | 0.04782 | 0.2121212 | 0.00786 | 0.0808081 | 0.41975 |
| Fcgamma receptor (FCGR) dependent phagocytosis | 0.18604651 | 0.04992 | 0.2093023 | 0.01514 | 0.1976744 | 0.00011 |
| Response to elevated platelet cytosolic Ca2+ | 0.17164179 | 0.04998 | 0.1567164 | 0.14022 | 0.0597015 | 0.75677 |
| Metabolism of RNA | 0.07418398 | 0.99998 | 0.2759644 | 4.35E-29 | 0.0667656 | 0.72569 |
| Translation | 0.02405498 | 1 | 0.3642612 | 3.58E-27 | 0.0412371 | 0.98979 |
| Infectious disease | 0.10209424 | 0.88817 | 0.3115183 | 1.70E-23 | 0.091623 | 0.08154 |
| Signaling by ROBO receptors | 0.09633028 | 0.89035 | 0.3348624 | 1.11E-16 | 0.087156 | 0.22241 |
| Regulation of expression of SLITs and ROBOs | 0.05847953 | 0.99793 | 0.3625731 | 3.23E-16 | 0.0643275 | 0.69361 |
| NIK-->noncanonical NF-kB signaling | 0.16949153 | 0.16723 | 0.559322 | 9.03E-16 | 0.1355932 | 0.05918 |
| Dectin-1 mediated noncanonical NF-kB signaling | 0.18333333 | 0.10112 | 0.55 | 1.77E-15 | 0.1333333 | 0.0642 |
| Negative regulation of NOTCH4 signaling | 0.14814815 | 0.32322 | 0.5740741 | 2.50E-15 | 0.1296296 | 0.09054 |
| The role of GTSE1 in G2/M progression after G2 checkpoint | 0.17333333 | 0.11212 | 0.4933333 | 3.65E-15 | 0.1333333 | 0.04147 |
| ER-Phagosome pathway | 0.18072289 | 0.0696 | 0.4698795 | 5.22E-15 | 0.1566265 | 0.00603 |

| | | | | | | |
|---|---|---|---|---|---|---|
| Autodegradation of Cdh1 by Cdh1:APC/C | 0.11111111 | 0.6489 | 0.5238095 | 1.20E-14 | 0.0952381 | 0.29889 |
| HIV Infection | 0.10775862 | 0.75593 | 0.3103448 | 1.63E-14 | 0.0905172 | 0.16269 |
| APC/C:Cdc20 mediated degradation of Securin | 0.10447761 | 0.71252 | 0.5074627 | 1.64E-14 | 0.0895522 | 0.35076 |
| FBXL7 down-regulates AURKA during mitotic entry and in early mitosis | 0.14814815 | 0.32322 | 0.5555556 | 2.38E-14 | 0.1111111 | 0.18997 |
| Eukaryotic Translation Initiation | 0.00840336 | 1 | 0.394958 | 3.20E-14 | 0.0252101 | 0.99295 |
| Cap-dependent Translation Initiation | 0.00840336 | 1 | 0.394958 | 3.20E-14 | 0.0252101 | 0.99295 |
| SCF-beta-TrCP mediated degradation of Emi1 | 0.12727273 | 0.50015 | 0.5454545 | 4.63E-14 | 0.1090909 | 0.20127 |
| Vpu mediated degradation of CD4 | 0.15384615 | 0.28608 | 0.5576923 | 5.66E-14 | 0.1153846 | 0.16814 |
| AUF1 (hnRNP D0) binds and destabilizes mRNA | 0.14285714 | 0.36115 | 0.5357143 | 8.82E-14 | 0.125 | 0.1051 |
| Regulation of Apoptosis | 0.13207547 | 0.45971 | 0.5471698 | 1.10E-13 | 0.1132075 | 0.17892 |
| Degradation of GLI2 by the proteasome | 0.15 | 0.29414 | 0.5166667 | 1.26E-13 | 0.1166667 | 0.13772 |
| GLI3 is processed to GLI3R by the proteasome | 0.15 | 0.29414 | 0.5166667 | 1.26E-13 | 0.1166667 | 0.13772 |
| Regulation of activated PAK-2p34 by proteasome mediated degradation | 0.14 | 0.39792 | 0.56 | 1.34E-13 | 0.12 | 0.14745 |
| L13a-mediated translational silencing of Ceruloplasmin expression | 0.00900901 | 1 | 0.3963964 | 1.82E-13 | 0.027027 | 0.98862 |
| Vif-mediated degradation of APOBEC3G | 0.12962963 | 0.48004 | 0.537037 | 2.11E-13 | 0.1111111 | 0.18997 |
| Antigen processing-Cross presentation | 0.17171717 | 0.08284 | 0.4141414 | 2.15E-13 | 0.1515152 | 0.00457 |
| Defective CFTR causes cystic fibrosis | 0.13114754 | 0.45693 | 0.5081967 | 2.26E-13 | 0.1147541 | 0.14657 |
| APC/C:Cdh1 mediated degradation of Cdc20 and other APC/C:Cdh1 targeted proteins in late mitosis/early G1 | 0.09722222 | 0.78047 | 0.4722222 | 2.46E-13 | 0.0833333 | 0.4164 |
| GTP hydrolysis and joining of the 60S ribosomal subunit | 0.00892857 | 1 | 0.3928571 | 2.65E-13 | 0.0267857 | 0.98928 |
| Regulation of mRNA stability by proteins that bind AU-rich elements | 0.15909091 | 0.16919 | 0.4318182 | 3.34E-13 | 0.1363636 | 0.02316 |
| Autodegradation of the E3 ubiquitin ligase COP1 | 0.15384615 | 0.28608 | 0.5384615 | 5.04E-13 | 0.1346154 | 0.07719 |
| Ubiquitin-dependent degradation of Cyclin D1 | 0.13461538 | 0.43921 | 0.5384615 | 5.04E-13 | 0.1153846 | 0.16814 |
| Ubiquitin-dependent degradation of Cyclin D | 0.13461538 | 0.43921 | 0.5384615 | 5.04E-13 | 0.1153846 | 0.16814 |
| Hh mutants that don't undergo autocatalytic processing are degraded by ERAD | 0.16071429 | 0.22786 | 0.5178571 | 7.23E-13 | 0.1071429 | 0.21281 |
| Host Interactions of HIV factors | 0.125 | 0.47851 | 0.3671875 | 7.67E-13 | 0.078125 | 0.44069 |
| Ubiquitin Mediated Degradation of Phosphorylated Cdc25A | 0.13207547 | 0.45971 | 0.5283019 | 9.44E-13 | 0.1132075 | 0.17892 |
| p53-Independent DNA Damage Response | 0.13207547 | 0.45971 | 0.5283019 | 9.44E-13 | 0.1132075 | 0.17892 |
| p53-Independent G1/S DNA damage checkpoint | 0.13207547 | 0.45971 | 0.5283019 | 9.44E-13 | 0.1132075 | 0.17892 |

| | | | | | | |
|---|---|---|---|---|---|---|
| SCF(Skp2)-mediated degradation of p27/p21 | 0.11666667 | 0.59612 | 0.5 | 9.59E-13 | 0.1 | 0.26103 |
| Degradation of GLI1 by the proteasome | 0.16666667 | 0.18045 | 0.5 | 9.59E-13 | 0.1333333 | 0.0642 |
| APC/C:Cdc20 mediated degradation of mitotic proteins | 0.09333333 | 0.81509 | 0.4533333 | 1.08E-12 | 0.08 | 0.45549 |
| Cross-presentation of soluble exogenous antigens (endosomes) | 0.14 | 0.39792 | 0.54 | 1.20E-12 | 0.12 | 0.14745 |
| Cdc20:Phospho-APC/C mediated degradation of Cyclin A | 0.09722222 | 0.78047 | 0.4583333 | 1.59E-12 | 0.0833333 | 0.4164 |
| CDK-mediated phosphorylation and removal of Cdc6 | 0.09722222 | 0.78047 | 0.4583333 | 1.59E-12 | 0.0833333 | 0.4164 |
| Programmed Cell Death | 0.15697674 | 0.08981 | 0.3255814 | 1.62E-12 | 0.1162791 | 0.02226 |
| Activation of APC/C and APC/C:Cdc20 mediated degradation of mitotic proteins | 0.09210526 | 0.82564 | 0.4473684 | 1.72E-12 | 0.0789474 | 0.4684 |
| Regulation of APC/C activators between G1/S and early anaphase | 0.0875 | 0.86317 | 0.4375 | 1.81E-12 | 0.075 | 0.51906 |
| Hedgehog ligand biogenesis | 0.15384615 | 0.25297 | 0.4769231 | 2.02E-12 | 0.0923077 | 0.32468 |
| Regulation of ornithine decarboxylase (ODC) | 0.1372549 | 0.4186 | 0.5294118 | 2.26E-12 | 0.1176471 | 0.15765 |
| APC:Cdc20 mediated degradation of cell cycle proteins prior to satisfation of the cell cycle checkpoint | 0.09589041 | 0.79252 | 0.4520548 | 2.56E-12 | 0.0821918 | 0.42948 |
| Apoptosis | 0.15384615 | 0.113 | 0.3254438 | 2.61E-12 | 0.112426 | 0.03447 |
| Degradation of AXIN | 0.12727273 | 0.50015 | 0.5090909 | 3.12E-12 | 0.1090909 | 0.20127 |
| Regulation of RUNX3 expression and activity | 0.14545455 | 0.34211 | 0.5090909 | 3.12E-12 | 0.1272727 | 0.09767 |
| Cellular responses to external stimuli | 0.13717694 | 0.13513 | 0.2306163 | 3.32E-12 | 0.0854871 | 0.13257 |
| Oxygen-dependent proline hydroxylation of Hypoxia-inducible Factor Alpha | 0.18181818 | 0.09403 | 0.469697 | 3.36E-12 | 0.1363636 | 0.04561 |
| Hh mutants abrogate ligand secretion | 0.15254237 | 0.27709 | 0.4915254 | 4.00E-12 | 0.1016949 | 0.2487 |
| APC/C-mediated degradation of cell cycle proteins | 0.08139535 | 0.90683 | 0.4186047 | 4.31E-12 | 0.0697674 | 0.59116 |
| Regulation of mitotic cell cycle | 0.08139535 | 0.90683 | 0.4186047 | 4.31E-12 | 0.0697674 | 0.59116 |
| Degradation of beta-catenin by the destruction complex | 0.13414634 | 0.40104 | 0.4268293 | 4.34E-12 | 0.097561 | 0.23562 |
| Influenza Infection | 0.03896104 | 0.9999 | 0.3311688 | 7.85E-12 | 0.0519481 | 0.87284 |
| Degradation of DVL | 0.15789474 | 0.24391 | 0.4912281 | 9.60E-12 | 0.1052632 | 0.22457 |
| Stabilization of p53 | 0.14035088 | 0.38029 | 0.4912281 | 9.60E-12 | 0.122807 | 0.11283 |
| G2/M Transition | 0.15979381 | 0.0606 | 0.3041237 | 1.03E-11 | 0.0824742 | 0.32083 |
| Mitotic G2-G2/M phases | 0.15816327 | 0.06773 | 0.3010204 | 1.65E-11 | 0.0816327 | 0.3357 |
| Processing of Capped Intron-Containing Pre-mRNA | 0.09465021 | 0.91568 | 0.2798354 | 1.91E-11 | 0.0781893 | 0.38622 |
| CDT1 association with the CDC6:ORC:origin complex | 0.11864407 | 0.57765 | 0.4745763 | 2.76E-11 | 0.1016949 | 0.2487 |
| ABC transporter disorders | 0.14473684 | 0.30579 | 0.4210526 | 5.58E-11 | 0.1052632 | 0.17841 |
| Influenza Life Cycle | 0.02797203 | 0.99999 | 0.3286713 | 7.02E-11 | 0.048951 | 0.89716 |

| | | | | | | |
|---|---|---|---|---|---|---|
| Regulation of PTEN stability and activity | 0.14492754 | 0.31693 | 0.4347826 | 8.47E-11 | 0.115942 | 0.12082 |
| Regulation of RUNX2 expression and activity | 0.12328767 | 0.52474 | 0.4246575 | 8.57E-11 | 0.1232877 | 0.07721 |
| Mitotic Anaphase | 0.09547739 | 0.88883 | 0.2914573 | 1.03E-10 | 0.0603015 | 0.77753 |
| Switching of origins to a post-replicative state | 0.07777778 | 0.9288 | 0.3888889 | 1.03E-10 | 0.0666667 | 0.63592 |
| Mitotic Metaphase and Anaphase | 0.095 | 0.89353 | 0.29 | 1.28E-10 | 0.06 | 0.78321 |
| Regulation of Hypoxia-inducible Factor (HIF) by oxygen | 0.18666667 | 0.06231 | 0.4133333 | 1.96E-10 | 0.12 | 0.0882 |
| Cellular response to hypoxia | 0.18666667 | 0.06231 | 0.4133333 | 1.96E-10 | 0.12 | 0.0882 |
| Orc1 removal from chromatin | 0.09859155 | 0.76792 | 0.4225352 | 2.01E-10 | 0.084507 | 0.40328 |
| Separation of Sister Chromatids | 0.09042553 | 0.92267 | 0.2925532 | 2.69E-10 | 0.0585106 | 0.80257 |
| mRNA Splicing - Major Pathway | 0.09836066 | 0.85255 | 0.295082 | 2.75E-10 | 0.0819672 | 0.33716 |
| Asymmetric localization of PCP proteins | 0.109375 | 0.66555 | 0.4375 | 3.02E-10 | 0.09375 | 0.31174 |
| Regulation of RAS by GAPs | 0.14705882 | 0.30057 | 0.4264706 | 3.07E-10 | 0.1029412 | 0.21531 |
| SRP-dependent cotranslational protein targeting to membrane | 0.01785714 | 0.99999 | 0.3482143 | 4.32E-10 | 0.0357143 | 0.96483 |
| ABC-family proteins mediated transport | 0.11650485 | 0.59567 | 0.3592233 | 4.40E-10 | 0.0970874 | 0.20533 |
| Cellular responses to stress | 0.13615023 | 0.17415 | 0.2276995 | 4.61E-10 | 0.084507 | 0.17485 |
| mRNA Splicing | 0.09424084 | 0.89541 | 0.2879581 | 5.18E-10 | 0.078534 | 0.40015 |
| p53-Dependent G1 DNA Damage Response | 0.12121212 | 0.54974 | 0.4242424 | 7.21E-10 | 0.1060606 | 0.19456 |
| p53-Dependent G1/S DNA damage checkpoint | 0.12121212 | 0.54974 | 0.4242424 | 7.21E-10 | 0.1060606 | 0.19456 |
| Cyclin E associated events during G1/S transition | 0.13253012 | 0.41718 | 0.3855422 | 8.45E-10 | 0.0843373 | 0.38839 |
| Formation of a pool of free 40S subunits | 0.00990099 | 1 | 0.3564356 | 9.71E-10 | 0.029703 | 0.97958 |
| Assembly of the pre-replicative complex | 0.10294118 | 0.72715 | 0.4117647 | 1.65E-09 | 0.0882353 | 0.36387 |
| G1/S DNA Damage Checkpoints | 0.11764706 | 0.58503 | 0.4117647 | 1.65E-09 | 0.1029412 | 0.21531 |
| Cyclin A:Cdk2-associated events at S phase entry | 0.14117647 | 0.32492 | 0.3764706 | 1.71E-09 | 0.0823529 | 0.41246 |
| Metabolism of polyamines | 0.13953488 | 0.33991 | 0.372093 | 2.41E-09 | 0.1046512 | 0.16401 |
| PCP/CE pathway | 0.16304348 | 0.13728 | 0.3586957 | 4.02E-09 | 0.1195652 | 0.06523 |
| Class I MHC mediated antigen processing & presentation | 0.14285714 | 0.10491 | 0.2264151 | 9.39E-09 | 0.1105121 | 0.00376 |
| Influenza Viral RNA Transcription and Replication | 0.02255639 | 0.99999 | 0.3082707 | 9.68E-09 | 0.037594 | 0.96699 |
| Translation initiation complex formation | NA | NA | 0.4137931 | 2.15E-08 | NA | NA |
| Ribosomal scanning and start codon recognition | NA | NA | 0.4137931 | 2.15E-08 | NA | NA |
| Signaling by Hedgehog | 0.16326531 | 0.07424 | 0.292517 | 2.42E-08 | 0.0816327 | 0.36732 |
| Selenoamino acid metabolism | 0.02542373 | 0.99996 | 0.3135593 | 3.08E-08 | 0.0338983 | 0.97427 |
| Activation of the mRNA upon binding of the cap-binding complex and eIFs, and subsequent binding to 43S | NA | NA | 0.4067797 | 3.20E-08 | NA | NA |

| | | | | | | |
|---|---|---|---|---|---|---|
| Nonsense Mediated Decay (NMD) independent of the Exon Junction Complex (EJC) | 0.01052632 | 1 | 0.3368421 | 3.95E-08 | 0.0421053 | 0.91798 |
| Mitochondrial translation initiation | 0.02298851 | 0.99983 | 0.3448276 | 5.74E-08 | 0.0574713 | 0.75925 |
| rRNA processing | 0.0195122 | 1 | 0.2585366 | 6.21E-08 | 0.0243902 | 0.9993 |
| rRNA processing in the nucleus and cytosol | 0.01538462 | 1 | 0.2615385 | 7.38E-08 | 0.025641 | 0.99876 |
| Mitochondrial translation | 0.02150538 | 0.99991 | 0.3333333 | 8.45E-08 | 0.0537634 | 0.80817 |
| Eukaryotic Translation Termination | 0.01075269 | 0.99999 | 0.3333333 | 8.45E-08 | 0.0430108 | 0.90977 |
| Major pathway of rRNA processing in the nucleolus and cytosol | 0.01621622 | 1 | 0.2648649 | 8.68E-08 | 0.027027 | 0.99782 |
| Hedgehog 'on' state | 0.15294118 | 0.21977 | 0.3411765 | 1.24E-07 | 0.0941176 | 0.26634 |
| Metabolism of amino acids and derivatives | 0.07297297 | 0.99911 | 0.2162162 | 1.75E-07 | 0.0567568 | 0.89796 |
| Transcriptional regulation by RUNX2 | 0.16528926 | 0.08763 | 0.2975207 | 2.08E-07 | 0.1157025 | 0.05186 |
| Mitochondrial translation termination | 0.02298851 | 0.99983 | 0.3333333 | 2.20E-07 | 0.0574713 | 0.75925 |
| UCH proteinases | 0.11764706 | 0.58154 | 0.3137255 | 2.57E-07 | 0.0980392 | 0.19712 |
| Peptide chain elongation | 0.01123596 | 0.99999 | 0.3258427 | 3.81E-07 | 0.0337079 | 0.95963 |
| Synthesis of DNA | 0.05882353 | 0.99209 | 0.2941176 | 4.15E-07 | 0.0504202 | 0.86577 |
| DNA Replication Pre-Initiation | 0.08235294 | 0.90051 | 0.3294118 | 4.68E-07 | 0.0705882 | 0.57953 |
| Nonsense-Mediated Decay (NMD) | 0.03478261 | 0.99972 | 0.2956522 | 5.30E-07 | 0.0434783 | 0.92368 |
| Nonsense Mediated Decay (NMD) enhanced by the Exon Junction Complex (EJC) | 0.03478261 | 0.99972 | 0.2956522 | 5.30E-07 | 0.0434783 | 0.92368 |
| Antigen processing: Ubiquitination & Proteasome degradation | 0.12944984 | 0.33793 | 0.2200647 | 7.58E-07 | 0.0970874 | 0.05648 |
| Mitochondrial translation elongation | 0.02298851 | 0.99983 | 0.3218391 | 8.01E-07 | 0.0574713 | 0.75925 |
| Eukaryotic Translation Elongation | 0.01075269 | 0.99999 | 0.311828 | 1.07E-06 | 0.0322581 | 0.96774 |
| Selenocysteine synthesis | 0.01075269 | 0.99999 | 0.311828 | 1.07E-06 | 0.0322581 | 0.96774 |
| Viral mRNA Translation | 0.01123596 | 0.99999 | 0.3146067 | 1.34E-06 | 0.0337079 | 0.95963 |
| DNA Replication | 0.05511811 | 0.99594 | 0.2755906 | 2.24E-06 | 0.0472441 | 0.90218 |
| Neddylation | 0.11538462 | 0.62691 | 0.2307692 | 2.33E-06 | 0.0854701 | 0.24121 |
| Prefoldin mediated transfer of substrate to CCT/TriC | 0.23076923 | 0.08433 | 0.5 | 2.93E-06 | 0.1538462 | 0.11266 |
| Cooperation of Prefoldin and TriC/CCT in actin and tubulin folding | 0.2 | 0.14494 | 0.4666667 | 3.46E-06 | 0.1333333 | 0.1658 |
| Ub-specific processing proteases | 0.09090909 | 0.93357 | 0.2318182 | 3.85E-06 | 0.0772727 | 0.41534 |
| Formation of tubulin folding intermediates by CCT/TriC | 0.2173913 | 0.1351 | 0.5217391 | 4.01E-06 | 0.173913 | 0.07884 |
| Formation of the ternary complex, and subsequently, the 43S complex | NA | NA | 0.372549 | 4.19E-06 | NA | NA |
| M Phase | 0.08396947 | 0.99278 | 0.2010178 | 4.33E-06 | 0.0559796 | 0.91483 |

| | | | | | | |
|---|---|---|---|---|---|---|
| Disorders of transmembrane transporters | 0.13888889 | 0.28278 | 0.2569444 | 7.01E-06 | 0.1111111 | 0.05396 |
| Deubiquitination | 0.11447811 | 0.65424 | 0.2087542 | 1.39E-05 | 0.0841751 | 0.23246 |
| HIV Life Cycle | 0.08609272 | 0.92994 | 0.2450331 | 2.22E-05 | 0.0794702 | 0.40305 |
| G1/S Transition | 0.09160305 | 0.8796 | 0.2519084 | 3.35E-05 | 0.0534351 | 0.84052 |
| Mitotic G1-G1/S phases | 0.11409396 | 0.63378 | 0.2416107 | 3.94E-05 | 0.0738255 | 0.50958 |
| RNA polymerase II transcribes snRNA genes | 0.14864865 | 0.27532 | 0.2972973 | 4.78E-05 | 0.1351351 | 0.0383 |
| Cytosolic tRNA aminoacylation | 0.125 | 0.56587 | 0.4583333 | 4.87E-05 | 0.0833333 | 0.52335 |
| TP53 Regulates Metabolic Genes | 0.08139535 | 0.90683 | 0.2790698 | 6.85E-05 | 0.1162791 | 0.08845 |
| S Phase | 0.08074534 | 0.96016 | 0.2298137 | 9.68E-05 | 0.0434783 | 0.94978 |
| Late Phase of HIV Life Cycle | 0.08695652 | 0.91685 | 0.2391304 | 0.0001 | 0.0869565 | 0.28911 |
| Cellular response to heat stress | 0.10227273 | 0.74871 | 0.2727273 | 0.0001 | 0.0909091 | 0.29815 |
| Signaling by NOTCH | 0.15744681 | 0.05259 | 0.2085106 | 0.00011 | 0.093617 | 0.12207 |
| Antiviral mechanism by IFN-stimulated genes | 0.11538462 | 0.60787 | 0.2820513 | 0.00011 | 0.0641026 | 0.66927 |
| Cell Cycle, Mitotic | 0.09701493 | 0.96576 | 0.1753731 | 0.00016 | 0.0559701 | 0.9439 |
| RUNX1 regulates transcription of genes involved in differentiation of HSCs | 0.08461538 | 0.92526 | 0.2384615 | 0.00017 | 0.0461538 | 0.91347 |
| ISG15 antiviral mechanism | 0.09859155 | 0.76792 | 0.2816901 | 0.00024 | 0.0422535 | 0.89375 |
| Processing of Capped Intronless Pre-mRNA | 0.10714286 | 0.6729 | 0.3928571 | 0.00026 | 0.1428571 | 0.13819 |
| Cell Cycle Checkpoints | 0.04778157 | 1 | 0.1911263 | 0.00041 | 0.0409556 | 0.99058 |
| Folding of actin by CCT/TriC | 0.1 | 0.72331 | 0.6 | 0.00045 | 0.2 | 0.15846 |
| Mitochondrial protein import | 0.03125 | 0.99742 | 0.28125 | 0.00049 | 0.078125 | 0.49178 |
| G2/M Checkpoints | 0.05357143 | 0.999 | 0.2142857 | 0.00051 | 0.0535714 | 0.8619 |
| FGFR2 alternative splicing | 0.07692308 | 0.8385 | 0.3846154 | 0.00061 | 0.1153846 | 0.2866 |
| Chaperonin-mediated protein folding | 0.15053763 | 0.22608 | 0.2473118 | 0.00066 | 0.0752688 | 0.50729 |
| Protein folding | 0.14141414 | 0.30309 | 0.2424242 | 0.00069 | 0.0707071 | 0.57486 |
| Cleavage of Growing Transcript in the Termination Region | 0.10447761 | 0.71252 | 0.2686567 | 0.00088 | 0.1044776 | 0.20483 |
| RNA Polymerase II Transcription Termination | 0.10447761 | 0.71252 | 0.2686567 | 0.00088 | 0.1044776 | 0.20483 |
| RNA Polymerase II Pre-transcription Events | 0.11904762 | 0.56701 | 0.25 | 0.00096 | 0.0952381 | 0.25597 |
| The citric acid (TCA) cycle and respiratory electron transport | 0.05172414 | 0.9994 | 0.2068966 | 0.001 | 0.0804598 | 0.37102 |
| snRNP Assembly | 0.01923077 | 0.99876 | 0.2884615 | 0.00105 | NA | NA |
| Metabolism of non-coding RNA | 0.01923077 | 0.99876 | 0.2884615 | 0.00105 | NA | NA |
| mRNA Splicing - Minor Pathway | 0.05769231 | 0.9588 | 0.2884615 | 0.00105 | 0.0576923 | 0.73266 |
| Macroautophagy | 0.14705882 | 0.30057 | 0.2647059 | 0.00106 | 0.1029412 | 0.21531 |
| Regulation of HSF1-mediated heat shock response | 0.07352941 | 0.92502 | 0.2647059 | 0.00106 | 0.0882353 | 0.36387 |

| | | | | | | |
|---|---|---|---|---|---|---|
| Processing of Intronless Pre-mRNAs | 0.15789474 | 0.40611 | 0.4210526 | 0.00108 | 0.2105263 | 0.04321 |
| Transcriptional Regulation by TP53 | 0.1260274 | 0.39553 | 0.1780822 | 0.00108 | 0.0849315 | 0.1885 |
| HIV Transcription Initiation | 0.06382979 | 0.93409 | 0.2978723 | 0.00109 | 0.0851064 | 0.4405 |
| RNA Polymerase II HIV Promoter Escape | 0.06382979 | 0.93409 | 0.2978723 | 0.00109 | 0.0851064 | 0.4405 |
| RNA Polymerase II Promoter Escape | 0.06382979 | 0.93409 | 0.2978723 | 0.00109 | 0.0851064 | 0.4405 |
| RNA Polymerase II Transcription Pre-Initiation And Promoter Opening | 0.06382979 | 0.93409 | 0.2978723 | 0.00109 | 0.0851064 | 0.4405 |
| RNA Polymerase II Transcription Initiation | 0.06382979 | 0.93409 | 0.2978723 | 0.00109 | 0.0851064 | 0.4405 |
| RNA Polymerase II Transcription Initiation And Promoter Clearance | 0.06382979 | 0.93409 | 0.2978723 | 0.00109 | 0.0851064 | 0.4405 |
| tRNA Aminoacylation | 0.07142857 | 0.8963 | 0.3095238 | 0.00109 | 0.047619 | 0.81543 |
| Cell Cycle | 0.08424337 | 0.99904 | 0.1622465 | 0.00135 | 0.0514821 | 0.98709 |
| RHO GTPase Effectors | 0.12974684 | 0.32975 | 0.1803797 | 0.00158 | 0.0981013 | 0.04746 |
| HSF1 activation | 0.16666667 | 0.43379 | 0.5 | 0.0016 | 0.1666667 | 0.21187 |
| MAP3K8 (TPL2)-dependent MAPK1/3 activation | 0.25 | 0.11748 | 0.4375 | 0.00171 | 0.125 | 0.32143 |
| Regulation of PLK1 Activity at G2/M Transition | 0.14772727 | 0.25857 | 0.2386364 | 0.0018 | 0.0681818 | 0.6139 |
| Respiratory electron transport | 0.03 | 0.99973 | 0.23 | 0.00189 | 0.09 | 0.29146 |
| Transcription of the HIV genome | 0.08219178 | 0.88803 | 0.2465753 | 0.00254 | 0.0821918 | 0.42948 |
| Citric acid cycle (TCA cycle) | 0.13636364 | 0.50495 | 0.3636364 | 0.00325 | 0.1818182 | 0.06888 |
| mRNA 3'-end processing | 0.12068966 | 0.55879 | 0.2586207 | 0.00345 | 0.1206897 | 0.12084 |
| Processing and activation of SUMO | NA | NA | 0.5 | 0.00405 | NA | NA |
| Formation of Incision Complex in GG-NER | 0.06976744 | 0.90514 | 0.2790698 | 0.0044 | 0.0232558 | 0.95988 |
| mTORC1-mediated signalling | 0.04347826 | 0.94803 | 0.3478261 | 0.00446 | 0.0434783 | 0.82065 |
| Abortive elongation of HIV-1 transcript in the absence of Tat | 0.04347826 | 0.94803 | 0.3478261 | 0.00446 | 0.0869565 | 0.50029 |
| RHO GTPases Activate Formins | 0.14492754 | 0.22094 | 0.2028986 | 0.00463 | 0.0869565 | 0.28911 |
| Formation of the Early Elongation Complex | 0.03030303 | 0.98566 | 0.3030303 | 0.00477 | 0.0606061 | 0.69744 |
| Formation of the HIV-1 Early Elongation Complex | 0.03030303 | 0.98566 | 0.3030303 | 0.00477 | 0.0606061 | 0.69744 |
| Constitutive Signaling by Ligand-Responsive EGFR Cancer Variants | 0.21052632 | 0.18858 | 0.3684211 | 0.0054 | 0.2105263 | 0.04321 |
| Signaling by EGFR in Cancer | 0.21052632 | 0.18858 | 0.3684211 | 0.0054 | 0.2105263 | 0.04321 |
| Signaling by Ligand-Responsive EGFR Variants in Cancer | 0.21052632 | 0.18858 | 0.3684211 | 0.0054 | 0.2105263 | 0.04321 |
| Signaling by FGFR2 IIIa TM | 0.10526316 | 0.68619 | 0.3684211 | 0.0054 | 0.0526316 | 0.75811 |
| Formation of RNA Pol II elongation complex | 0.13114754 | 0.45693 | 0.2459016 | 0.00576 | 0.0819672 | 0.44922 |
| RNA Polymerase II Transcription Elongation | 0.13114754 | 0.45693 | 0.2459016 | 0.00576 | 0.0819672 | 0.44922 |

| | | | | | | |
|---|---|---|---|---|---|---|
| Synthesis of very long-chain fatty acyl-CoAs | 0.16666667 | 0.32674 | 0.3333333 | 0.00599 | 0.2083333 | 0.02566 |
| mRNA Capping | 0.03448276 | 0.976 | 0.3103448 | 0.00615 | 0.0344828 | 0.88552 |
| Activation of BAD and translocation to mitochondria | 0.2 | 0.26759 | 0.4 | 0.00626 | 0.2 | 0.08817 |
| Constitutive Signaling by EGFRvIII | 0.26666667 | 0.09693 | 0.4 | 0.00626 | 0.2666667 | 0.01911 |
| Signaling by EGFRvIII in Cancer | 0.26666667 | 0.09693 | 0.4 | 0.00626 | 0.2666667 | 0.01911 |
| The NLRP3 inflammasome | 0.2 | 0.26759 | 0.4 | 0.00626 | 0.0666667 | 0.67381 |
| SeMet incorporation into proteins | 0.09090909 | 0.75669 | 0.4545455 | 0.00669 | NA | NA |
| rRNA modification in the nucleus and cytosol | NA | NA | 0.2419355 | 0.00675 | 0.016129 | 0.99035 |
| mTOR signalling | 0.125 | 0.53741 | 0.275 | 0.00708 | 0.125 | 0.15716 |
| Post-chaperonin tubulin folding pathway | 0.25 | 0.08377 | 0.35 | 0.00744 | 0.1 | 0.427 |
| Inflammasomes | 0.2 | 0.21478 | 0.35 | 0.00744 | 0.05 | 0.77554 |
| EGFR downregulation | 0.24 | 0.07187 | 0.32 | 0.00788 | 0.12 | 0.26653 |
| HIV Transcription Elongation | 0.08695652 | 0.8217 | 0.2608696 | 0.00789 | 0.0652174 | 0.65254 |
| Formation of HIV-1 elongation complex containing HIV-1 Tat | 0.08695652 | 0.8217 | 0.2608696 | 0.00789 | 0.0652174 | 0.65254 |
| Tat-mediated elongation of the HIV-1 transcript | 0.08695652 | 0.8217 | 0.2608696 | 0.00789 | 0.0652174 | 0.65254 |
| Host Interactions with Influenza Factors | 0.07317073 | 0.88671 | 0.2682927 | 0.00863 | 0.0243902 | 0.95339 |
| Transcriptional regulation by RUNX1 | 0.15481172 | 0.06459 | 0.1757322 | 0.00966 | 0.0794979 | 0.35837 |
| Asparagine N-linked glycosylation | 0.14900662 | 0.07605 | 0.1688742 | 0.01022 | 0.0662252 | 0.68371 |
| Viral Messenger RNA Synthesis | 0.04761905 | 0.96953 | 0.2619048 | 0.01043 | 0.0238095 | 0.95676 |
| EPHB-mediated forward signaling | 0.21428571 | 0.05917 | 0.2619048 | 0.01043 | 0.2142857 | 0.00254 |
| Cooperation of PDCL (PhLP1) and TRiC/CCT in G-protein beta folding | 0.11904762 | 0.58256 | 0.2619048 | 0.01043 | 0.0714286 | 0.59031 |
| Formation of HIV elongation complex in the absence of HIV Tat | 0.08333333 | 0.84653 | 0.25 | 0.01122 | 0.0625 | 0.68101 |
| Formation of TC-NER Pre-Incision Complex | 0.07407407 | 0.90414 | 0.2407407 | 0.01175 | 0.0555556 | 0.75591 |
| RNA Pol II CTD phosphorylation and interaction with CE during HIV infection | 0.03703704 | 0.96895 | 0.2962963 | 0.01295 | 0.037037 | 0.86704 |
| RNA Pol II CTD phosphorylation and interaction with CE | 0.03703704 | 0.96895 | 0.2962963 | 0.01295 | 0.037037 | 0.86704 |
| Pyruvate metabolism and Citric Acid (TCA) cycle | 0.10909091 | 0.66483 | 0.2363636 | 0.01371 | 0.0909091 | 0.36196 |
| Signaling by FGFR2 | 0.16438356 | 0.16366 | 0.2191781 | 0.01392 | 0.1232877 | 0.07721 |
| Recruitment of NuMA to mitotic centrosomes | 0.16304348 | 0.13728 | 0.2065217 | 0.01472 | 0.076087 | 0.49567 |
| SUMOylation of DNA replication proteins | 0.04545455 | 0.9755 | 0.25 | 0.01488 | NA | NA |
| SUMOylation of SUMOylation proteins | 0.03030303 | 0.98566 | 0.2727273 | 0.01511 | NA | NA |
| Chk1/Chk2(Cds1) mediated inactivation of Cyclin B:Cdk1 complex | NA | NA | 0.3846154 | 0.01511 | 0.1538462 | 0.23919 |

| | | | | | | |
|---|---|---|---|---|---|---|
| Signaling by WNT | 0.12424242 | 0.44236 | 0.1636364 | 0.01536 | 0.0727273 | 0.50845 |
| RNA Polymerase III Transcription Initiation From Type 3 Promoter | 0.14285714 | 0.44134 | 0.2857143 | 0.01623 | 0.1071429 | 0.32701 |
| NS1 Mediated Effects on Host Pathways | 0.05128205 | 0.95788 | 0.2564103 | 0.01661 | 0.025641 | 0.94586 |
| Association of TriC/CCT with target proteins during biosynthesis | 0.07692308 | 0.86515 | 0.2564103 | 0.01661 | 0.1025641 | 0.30723 |
| tRNA processing | 0.03773585 | 0.99928 | 0.1981132 | 0.01691 | 0.0377358 | 0.95225 |
| Signaling by FGFR | 0.16091954 | 0.15878 | 0.2068966 | 0.01698 | 0.1149425 | 0.09386 |
| Pausing and recovery of Tat-mediated HIV elongation | 0.11764706 | 0.59922 | 0.2647059 | 0.01838 | 0.0882353 | 0.44648 |
| Tat-mediated HIV elongation arrest and recovery | 0.11764706 | 0.59922 | 0.2647059 | 0.01838 | 0.0882353 | 0.44648 |
| Budding and maturation of HIV virion | 0.17241379 | 0.26686 | 0.2758621 | 0.02007 | 0.2068966 | 0.01545 |
| Mitophagy | 0.13793103 | 0.46925 | 0.2758621 | 0.02007 | 0.1034483 | 0.34723 |
| SUMOylation of RNA binding proteins | 0.10869565 | 0.66515 | 0.2391304 | 0.02062 | 0.0434783 | 0.85322 |
| Early Phase of HIV Life Cycle | 0.07142857 | 0.83456 | 0.3571429 | 0.02118 | NA | NA |
| Golgi Cisternae Pericentriolar Stack Reorganization | 0.07142857 | 0.83456 | 0.3571429 | 0.02118 | 0.0714286 | 0.64849 |
| Attenuation phase | 0.21428571 | 0.23346 | 0.3571429 | 0.02118 | 0.2857143 | 0.01484 |
| MicroRNA (miRNA) biogenesis | 0.16666667 | 0.32674 | 0.2916667 | 0.02146 | 0.0833333 | 0.52335 |
| Transport of Mature Transcript to Cytoplasm | 0.09638554 | 0.79839 | 0.2048193 | 0.02193 | 0.060241 | 0.72175 |
| Interactions of Vpr with host cellular proteins | 0.05714286 | 0.93559 | 0.2571429 | 0.02213 | NA | NA |
| Interactions of Rev with host cellular proteins | 0.02857143 | 0.98892 | 0.2571429 | 0.02213 | NA | NA |
| Organelle biogenesis and maintenance | 0.13265306 | 0.28368 | 0.1632653 | 0.02219 | 0.0952381 | 0.07685 |
| RNA Polymerase III Transcription | 0.14634146 | 0.37278 | 0.2439024 | 0.02338 | 0.097561 | 0.34064 |
| RNA Polymerase III Abortive And Retractive Initiation | 0.14634146 | 0.37278 | 0.2439024 | 0.02338 | 0.097561 | 0.34064 |
| RNA Polymerase I Promoter Escape | 0.1 | 0.71859 | 0.2666667 | 0.02451 | 0.0666667 | 0.6461 |
| Respiratory electron transport, ATP synthesis by chemiosmotic coupling, and heat production by uncoupling proteins. | 0.02439024 | 0.99998 | 0.1869919 | 0.02456 | 0.0731707 | 0.52871 |
| Transport of small molecules | 0.12171508 | 0.47778 | 0.1466113 | 0.02532 | 0.0885201 | 0.04608 |
| SLBP independent Processing of Histone Pre-mRNAs | NA | NA | 0.4 | 0.02561 | NA | NA |
| TCF dependent signaling in response to WNT | 0.10300429 | 0.82373 | 0.167382 | 0.02568 | 0.0643777 | 0.71046 |
| HIV elongation arrest and recovery | 0.11111111 | 0.64552 | 0.25 | 0.02639 | 0.0833333 | 0.48455 |
| Pausing and recovery of HIV elongation | 0.11111111 | 0.64552 | 0.25 | 0.02639 | 0.0833333 | 0.48455 |
| Striated Muscle Contraction | 0.13888889 | 0.44121 | 0.25 | 0.02639 | NA | NA |
| RNA Polymerase III Transcription Initiation | 0.11111111 | 0.64552 | 0.25 | 0.02639 | 0.0833333 | 0.48455 |
| Deadenylation of mRNA | 0.08 | 0.82185 | 0.28 | 0.02674 | 0.08 | 0.54568 |
| Nucleotide Excision Repair | 0.07207207 | 0.96538 | 0.1891892 | 0.02732 | 0.027027 | 0.98862 |

| | | | | | | |
|---|---|---|---|---|---|---|
| Protein ubiquitination | 0.11392405 | 0.62361 | 0.2025316 | 0.02831 | 0.0506329 | 0.82936 |
| RHO GTPases activate CIT | 0.2 | 0.21478 | 0.3 | 0.0284 | 0.05 | 0.77554 |
| RHO GTPases Activate ROCKs | 0.2 | 0.21478 | 0.3 | 0.0284 | 0.05 | 0.77554 |
| Listeria monocytogenes entry into host cells | 0.2 | 0.21478 | 0.3 | 0.0284 | 0.15 | 0.17029 |
| phosphorylation site mutants of CTNNB1 are not targeted to the proteasome by the destruction complex | 0.06666667 | 0.85453 | 0.3333333 | 0.02864 | NA | NA |
| Misspliced GSK3beta mutants stabilize beta-catenin | 0.06666667 | 0.85453 | 0.3333333 | 0.02864 | NA | NA |
| S33 mutants of beta-catenin aren't phosphorylated | 0.06666667 | 0.85453 | 0.3333333 | 0.02864 | NA | NA |
| S37 mutants of beta-catenin aren't phosphorylated | 0.06666667 | 0.85453 | 0.3333333 | 0.02864 | NA | NA |
| S45 mutants of beta-catenin aren't phosphorylated | 0.06666667 | 0.85453 | 0.3333333 | 0.02864 | NA | NA |
| T41 mutants of beta-catenin aren't phosphorylated | 0.06666667 | 0.85453 | 0.3333333 | 0.02864 | NA | NA |
| Diseases associated with glycosylation precursor biosynthesis | 0.13333333 | 0.55505 | 0.3333333 | 0.02864 | 0.1333333 | 0.29412 |
| IRAK1 recruits IKK complex | 0.26666667 | 0.09693 | 0.3333333 | 0.02864 | 0.2 | 0.08817 |
| TRAF6 mediated IRF7 activation in TLR7/8 or 9 signaling | 0.26666667 | 0.09693 | 0.3333333 | 0.02864 | 0.2 | 0.08817 |
| IRAK1 recruits IKK complex upon TLR7/8 or 9 stimulation | 0.26666667 | 0.09693 | 0.3333333 | 0.02864 | 0.2 | 0.08817 |
| AURKA Activation by TPX2 | 0.1369863 | 0.38392 | 0.2054795 | 0.02936 | 0.0684932 | 0.61069 |
| Fatty acyl-CoA biosynthesis | 0.10810811 | 0.6673 | 0.2432432 | 0.03121 | 0.1621622 | 0.04659 |
| Signaling by FGFR2 in disease | 0.13953488 | 0.41793 | 0.2325581 | 0.03195 | 0.1162791 | 0.19409 |
| Transport of Mature mRNA derived from an Intron-Containing Transcript | 0.08108108 | 0.89544 | 0.2027027 | 0.03281 | 0.0540541 | 0.78882 |
| Nuclear import of Rev protein | 0.03125 | 0.98369 | 0.25 | 0.03537 | NA | NA |
| FCERI mediated MAPK activation | 0.21875 | 0.0825 | 0.25 | 0.03537 | 0.15625 | 0.0761 |
| RNA Polymerase I Transcription Termination | 0.09375 | 0.75916 | 0.25 | 0.03537 | 0.0625 | 0.68105 |
| Endosomal Sorting Complex Required For Transport (ESCRT) | 0.125 | 0.54946 | 0.25 | 0.03537 | 0.15625 | 0.0761 |
| Deadenylation-dependent mRNA decay | 0.07142857 | 0.91857 | 0.2142857 | 0.03607 | 0.0892857 | 0.37658 |
| Endosomal/Vacuolar pathway | 0.27272727 | 0.13789 | 0.3636364 | 0.03642 | 0.2727273 | 0.03954 |
| SLBP Dependent Processing of Replication-Dependent Histone Pre-mRNAs | NA | NA | 0.3636364 | 0.03642 | NA | NA |
| NOTCH4 Activation and Transmission of Signal to the Nucleus | 0.18181818 | 0.38952 | 0.3636364 | 0.03642 | 0.1818182 | 0.18489 |
| Signaling by FGFR in disease | 0.12698413 | 0.49468 | 0.2063492 | 0.03951 | 0.0793651 | 0.47772 |
| Mitotic Prometaphase | 0.0959596 | 0.88397 | 0.1666667 | 0.0398 | 0.0555556 | 0.85177 |
| Transport of the SLBP independent Mature mRNA | 0.06060606 | 0.92061 | 0.2424242 | 0.04185 | 0.030303 | 0.91514 |
| FGFR2 mutant receptor activation | 0.15151515 | 0.36611 | 0.2424242 | 0.04185 | 0.0909091 | 0.42702 |
| Loss of Nlp from mitotic centrosomes | 0.14285714 | 0.33348 | 0.2 | 0.04243 | 0.0714286 | 0.5728 |

| | | | | | | |
|---|---|---|---|---|---|---|
| Loss of proteins required for interphase microtubule organization from the centrosome | 0.14285714 | 0.33348 | 0.2 | 0.04243 | 0.0714286 | 0.5728 |
| Pink/Parkin Mediated Mitophagy | 0.09090909 | 0.76229 | 0.2727273 | 0.04423 | 0.0454545 | 0.80672 |
| Iron uptake and transport | 0.06896552 | 0.93102 | 0.2068966 | 0.04593 | 0.137931 | 0.05442 |
| Beta-catenin phosphorylation cascade | 0.05882353 | 0.88753 | 0.2941176 | 0.04801 | NA | NA |
| Tristetraprolin (TTP, ZFP36) binds and destabilizes mRNA | 0.11764706 | 0.62505 | 0.2941176 | 0.04801 | 0.1176471 | 0.34847 |
| Transport of the SLBP Dependant Mature mRNA | 0.05882353 | 0.92847 | 0.2352941 | 0.04908 | 0.0294118 | 0.92126 |
| Transport of Mature mRNA Derived from an Intronless Transcript | 0.1 | 0.727 | 0.225 | 0.04923 | 0.05 | 0.79344 |
| Negative regulation of MAPK pathway | 0.15 | 0.35022 | 0.225 | 0.04923 | 0.05 | 0.79344 |
| Cholesterol biosynthesis | 0.24 | 0.07187 | 0.24 | 0.07665 | 0.48 | 3.82E-08 |
| Plasma lipoprotein clearance | 0.15151515 | 0.36611 | 0.1212121 | 0.58841 | 0.2424242 | 0.0019 |
| Formation of the cornified envelope | 0.09302326 | 0.86678 | 0.0775194 | 0.9628 | 0.1472868 | 0.00212 |
| Mitochondrial biogenesis | 0.16842105 | 0.10333 | 0.1684211 | 0.11504 | 0.1578947 | 0.00306 |
| Nicotinate metabolism | 0.19354839 | 0.16256 | 0.1935484 | 0.17169 | 0.2258065 | 0.00554 |
| Apoptotic cleavage of cell adhesion proteins | 0.09090909 | 0.75669 | 0.1818182 | 0.39781 | 0.3636364 | 0.00582 |
| Golgi Associated Vesicle Biogenesis | 0.14285714 | 0.36115 | 0.1785714 | 0.14112 | 0.1785714 | 0.00603 |
| Antigen activates B Cell Receptor (BCR) leading to generation of second messengers | 0.1875 | 0.18103 | 0.21875 | 0.08842 | 0.21875 | 0.00666 |
| TRAF6-mediated induction of TAK1 complex within TLR4 complex | 0.27777778 | 0.05656 | 0.1666667 | 0.38207 | 0.2777778 | 0.00739 |
| VLDLR internalisation and degradation | 0.25 | 0.1682 | 0.1666667 | 0.44253 | 0.3333333 | 0.00824 |
| Nicotinamide salvaging | 0.26315789 | 0.06943 | 0.2105263 | 0.19651 | 0.2631579 | 0.00945 |
| LDL clearance | 0.21052632 | 0.18858 | 0.2105263 | 0.19651 | 0.2631579 | 0.00945 |
| trans-Golgi Network Vesicle Budding | 0.11111111 | 0.65135 | 0.1666667 | 0.16559 | 0.1527778 | 0.01326 |
| Clathrin derived vesicle budding | 0.11111111 | 0.65135 | 0.1666667 | 0.16559 | 0.1527778 | 0.01326 |
| VEGFR2 mediated cell proliferation | 0.0952381 | 0.7389 | 0.1428571 | 0.48426 | 0.2380952 | 0.01468 |
| DCC mediated attractive signaling | 0.28571429 | 0.07825 | 0.2857143 | 0.08216 | 0.2857143 | 0.01484 |
| Downstream signal transduction | 0.17241379 | 0.26686 | 0.2413793 | 0.05644 | 0.2068966 | 0.01545 |
| DAP12 signaling | 0.2 | 0.14494 | 0.2 | 0.15333 | 0.2 | 0.01817 |
| Rap1 signalling | 0.25 | 0.11748 | 0.25 | 0.12297 | 0.25 | 0.02407 |
| HSF1-dependent transactivation | 0.20833333 | 0.15472 | 0.25 | 0.06467 | 0.2083333 | 0.02566 |
| Acetylcholine regulates insulin secretion | 0.3 | 0.1097 | 0.1 | 0.72951 | 0.3 | 0.03035 |
| MET receptor recycling | 0.1 | 0.72331 | 0.1 | 0.72951 | 0.3 | 0.03035 |
| Metal ion SLC transporters | 0.15384615 | 0.38436 | 0.1923077 | 0.20653 | 0.1923077 | 0.03529 |

| | | | | | | |
|---|---|---|---|---|---|---|
| SHC-mediated cascade:FGFR3 | 0.27777778 | 0.05656 | 0.1111111 | 0.66602 | 0.2222222 | 0.03611 |
| Lysosome Vesicle Biogenesis | 0.11428571 | 0.62282 | 0.1428571 | 0.43079 | 0.1714286 | 0.03669 |
| Signaling by Rho GTPases | 0.14189189 | 0.09235 | 0.1418919 | 0.11662 | 0.0945946 | 0.04015 |
| Gap junction trafficking and regulation | 0.21276596 | 0.05031 | 0.1914894 | 0.11419 | 0.1489362 | 0.0492 |
| FCGR activation | 0.08333333 | 0.78605 | 0.0833333 | 0.79179 | 0.25 | 0.04998 |
| PECAM1 interactions | 0.25 | 0.1682 | 0.1666667 | 0.44253 | 0.25 | 0.04998 |
| CD28 dependent Vav1 pathway | 0.25 | 0.1682 | 0.1666667 | 0.44253 | 0.25 | 0.04998 |
| Transport of organic anions | 0.16666667 | 0.43379 | 0.1666667 | 0.44253 | 0.25 | 0.04998 |
