# Supplementary material for: Comparative transcriptional profiling of the early host response to infection by typhoidal and non-typhoidal Salmonella serovars in human intestinal organoids
Source: PLoS Pathog. 2021 Oct 20;17(10):e1009987. doi: 10.1371/journal.ppat.1009987 (PMC8570492; doi:10.1371/journal.ppat.1009987)
Supplement: S6 Table — Significantly downregulated DEGs from STM, SE or ST-infected HIOs at 8hpi were subjected to Reactome pathway analysis. (PDF) [file ppat.1009987.s014.pdf]

Table S6: Reactome Pathways Downregulated 8h pi

Description	STM gene ratio	STM p-value	SE gene ratio	SE p-value	ST gene ratio	ST p-value
Translation	0.4261168	2.59E-39	0.0137457	1	0.089347	0.00439
Metabolism of RNA	0.268546	4.24E-26	0.05192878	0.99764	0.086053	7.03E-05
Influenza Viral RNA Transcription and Replication	0.4962406	5.92E-26	0.01503759	0.99977	0.142857	4.89E-05
Eukaryotic Translation Termination	0.5806452	7.68E-26	0.01075269	0.9995	0.129032	0.00285
Influenza Life Cycle	0.4755245	2.58E-25	0.02097902	0.99927	0.13986	4.25E-05
Selenoamino acid metabolism	0.5169492	2.61E-25	0.00847458	0.99994	0.118644	0.00294
Eukaryotic Translation Elongation	0.5698925	7.64E-25	0.01075269	0.9995	0.139785	0.00092
rRNA processing in the nucleus and cytosol	0.4102564	2.72E-24	0.03076923	0.99835	0.092308	0.01187
SRP-dependent cotranslational protein targeting to membrane	0.5178571	3.57E-24	0.00892857	0.99989	0.133929	0.00061
Peptide chain elongation	0.5730337	4.19E-24	0.01123596	0.9993	0.146067	0.0006
Viral mRNA Translation	0.5730337	4.19E-24	0.01123596	0.9993	0.134831	0.00196
Selenocysteine synthesis	0.5591398	7.28E-24	0.01075269	0.9995	0.139785	0.00092
Major pathway of rRNA processing in the nucleolus and cytosol	0.4108108	3.57E-23	0.03243243	0.99705	0.091892	0.01483
Influenza Infection	0.4415584	5.39E-23	0.01948052	0.99966	0.12987	0.00012
rRNA processing	0.3902439	1.28E-22	0.02926829	0.99908	0.087805	0.01898
Cell Cycle	0.2605304	1.62E-22	0.07332293	0.70025	0.134165	6.29E-17
Nonsense Mediated Decay (NMD) independent of the Exon Junction Complex (EJC)	0.5368421	2.44E-22	0.02105263	0.9961	0.126316	0.00341
Formation of a pool of free 40S subunits	0.5148515	1.20E-21	0.00990099	0.99974	0.118812	0.00563
L13a-mediated translational silencing of Ceruloplasmin expression	0.4864865	6.21E-21	0.00900901	0.99989	0.117117	0.00457
GTP hydrolysis and joining of the 60S ribosomal subunit	0.4821429	1.06E-20	0.00892857	0.99989	0.116071	0.00494
Nonsense-Mediated Decay (NMD)	0.4608696	3.20E-19	0.03478261	0.98235	0.121739	0.00231
Nonsense Mediated Decay (NMD) enhanced by the Exon Junction Complex (EJC)	0.4608696	3.20E-19	0.03478261	0.98235	0.121739	0.00231
Eukaryotic Translation Initiation	0.4537815	3.55E-19	0.00840336	0.99994	0.109244	0.00819
Cap-dependent Translation Initiation	0.4537815	3.55E-19	0.00840336	0.99994	0.109244	0.00819
Cell Cycle, Mitotic	0.255597	9.29E-18	0.06343284	0.91941	0.13806	2.69E-15
Cell Cycle Checkpoints	0.3105802	9.72E-18	0.07167235	0.6925	0.156997	8.51E-12
Metabolism of amino acids and derivatives	0.2864865	1.22E-17	0.06216216	0.89941	0.089189	0.00146
S Phase	0.3664596	1.66E-15	0.08074534	0.49199	0.217391	2.05E-13
Activation of the pre-replicative complex	0.7272727	1.80E-15	0.12121212	0.25476	0.424242	2.62E-10
Activation of ATR in response to replication stress	0.6756757	7.25E-15	0.10810811	0.32663	0.486486	3.55E-14

DNA Repair	0.2816456	1.73E-14	0.09177215	0.20472	0.129747	3.87E-08
G1/S Transition	0.3816794	3.80E-14	0.07633588	0.57727	0.221374	1.54E-11
DNA strand elongation	0.6875	1.79E-13	0.125	0.2373	0.5	5.58E-13
DNA Replication	0.3779528	1.91E-13	0.07086614	0.66787	0.23622	1.14E-12
Regulation of expression of SLITs and ROBOs	0.3333333	5.45E-13	0.02339181	0.99947	0.099415	0.00706
Mitotic G1-G1/S phases	0.3489933	7.54E-13	0.08053691	0.49937	0.208054	1.69E-11
Synthesis of DNA	0.3697479	4.69E-12	0.05882353	0.83088	0.226891	4.29E-11
G2/M Checkpoints	0.3214286	1.13E-11	0.06547619	0.77085	0.172619	7.63E-09
Chromosome Maintenance	0.3636364	8.01E-11	0.06363636	0.7654	0.172727	2.97E-06
Homology Directed Repair	0.3333333	9.67E-11	0.10144928	0.18871	0.115942	0.00194
HDR through Homologous Recombination (HRR) or Single Strand Annealing (SSA)	0.3333333	2.47E-10	0.10606061	0.14864	0.121212	0.00122
HDR through Homologous Recombination (HRR)	0.4328358	2.64E-10	0.17910448	0.00521	0.208955	6.16E-06
Mitochondrial translation	0.3763441	4.11E-10	NA	NA	0.064516	0.34667
Infectious disease	0.2356021	5.79E-10	0.02879581	0.99999	0.089005	0.00129
Extension of Telomeres	0.6	8.48E-10	0.13333333	0.20332	0.366667	1.38E-07
Signaling by ROBO receptors	0.2752294	9.67E-10	0.02752294	0.99958	0.082569	0.03266
The citric acid (TCA) cycle and respiratory electron transport	0.2931034	1.68E-09	0.02873563	0.99828	0.051724	0.54466
DNA Double-Strand Break Repair	0.2951807	2.71E-09	0.08433735	0.42225	0.10241	0.00526
Homologous DNA Pairing and Strand Exchange	0.5	3.15E-09	0.19047619	0.01458	0.261905	6.13E-06
Presynaptic phase of homologous DNA pairing and strand exchange	0.5128205	4.24E-09	0.20512821	0.00934	0.25641	2.00E-05
Mitochondrial translation elongation	0.3678161	4.49E-09	NA	NA	0.068966	0.29159
Mitochondrial translation termination	0.3678161	4.49E-09	NA	NA	0.068966	0.29159
DNA Replication Pre-Initiation	0.3647059	9.90E-09	0.07058824	0.66078	0.211765	2.38E-07
HDR through Single Strand Annealing (SSA)	0.5135135	1.01E-08	0.21621622	0.00673	0.216216	0.00048
Telomere C-strand (Lagging Strand) Synthesis	0.625	1.02E-08	0.16666667	0.11269	0.375	1.57E-06
Processing of Capped Intron-Containing Pre-mRNA	0.255144	1.27E-08	0.03703704	0.99726	0.106996	0.00032
Respiratory electron transport	0.34	1.53E-08	NA	NA	0.07	0.25663
Mitochondrial translation initiation	0.3563218	1.88E-08	NA	NA	0.068966	0.29159
Activation of E2F1 target genes at G1/S	0.5714286	2.00E-08	0.14285714	0.17096	0.357143	6.89E-07
G1/S-Specific Transcription	0.5714286	2.00E-08	0.14285714	0.17096	0.357143	6.89E-07
tRNA Aminoacylation	0.4761905	2.18E-08	0.07142857	0.64741	0.071429	0.36904
Processing of DNA double-strand break ends	0.3367347	3.26E-08	0.09183673	0.35654	0.112245	0.01188

Formation of the ternary complex, and subsequently, the 43S complex	0.4313725	4.13E-08	0.01960784	0.98433	0.137255	0.01501
Unwinding of DNA	0.8333333	4.32E-08	0.08333333	0.62319	0.666667	1.95E-08
Telomere Maintenance	0.3536585	6.50E-08	0.04878049	0.89197	0.170732	6.78E-05
Mitotic Spindle Checkpoint	0.3125	1.06E-07	0.08035714	0.51482	0.151786	5.57E-05
Translation initiation complex formation	0.3965517	1.35E-07	0.01724138	0.99116	0.12069	0.0287
Ribosomal scanning and start codon recognition	0.3965517	1.35E-07	0.01724138	0.99116	0.12069	0.0287
G2/M DNA damage checkpoint	0.3263158	1.90E-07	0.09473684	0.32288	0.157895	9.55E-05
Activation of the mRNA upon binding of the cap-binding complex and eIFs, and subsequent binding to 43S	0.3898305	1.95E-07	0.01694915	0.99185	0.118644	0.03117
CDC6 association with the ORC:origin complex	0.8181818	2.93E-07	0.27272727	0.04873	0.636364	2.56E-07
Respiratory electron transport, ATP synthesis by chemiosmotic coupling, and heat production by uncoupling proteins.	0.2926829	4.33E-07	0.00813008	0.99996	0.065041	0.3008
Dual Incision in GG-NER	0.4390244	5.14E-07	0.07317073	0.63099	0.195122	0.001
Resolution of Sister Chromatid Cohesion	0.2903226	5.38E-07	0.07258065	0.63957	0.145161	6.21E-05
Lagging Strand Synthesis	0.6	6.05E-07	0.15	0.20178	0.4	3.44E-06
Transcriptional Regulation by TP53	0.2136986	6.58E-07	0.08767123	0.26996	0.084932	0.00423
Amplification of signal from the kinetochores	0.3125	8.53E-07	0.08333333	0.47788	0.15625	0.00011
Amplification of signal from unattached kinetochores via a MAD2 inhibitory signal	0.3125	8.53E-07	0.08333333	0.47788	0.15625	0.00011
M Phase	0.2086514	9.54E-07	0.05089059	0.98787	0.096692	0.00013
Mitotic Prometaphase	0.2474747	1.13E-06	0.07575758	0.58778	0.121212	8.13E-05
Transcription-Coupled Nucleotide Excision Repair (TC-NER)	0.3291139	1.52E-06	0.07594937	0.589	0.164557	0.00018
Mitotic Metaphase and Anaphase	0.245	1.55E-06	0.05	0.95573	0.12	9.54E-05
Dual incision in TC-NER	0.3484848	1.99E-06	0.07575758	0.59451	0.166667	0.0005
mRNA Splicing	0.2460733	2.21E-06	0.04188482	0.98501	0.104712	0.00199
Resolution of D-loop Structures through Holliday Junction Intermediates	0.4545455	2.70E-06	0.24242424	0.00319	0.212121	0.00123
tRNA processing	0.2924528	2.75E-06	0.08490566	0.44757	0.103774	0.02043
Mitochondrial Fatty Acid Beta-Oxidation	0.4324324	2.83E-06	0.16216216	0.06475	0.108108	0.12111
Mitotic Anaphase	0.241206	3.16E-06	0.05025126	0.9539	0.115578	0.00023
Resolution of D-loop Structures through Synthesis-Dependent Strand Annealing (SDSA)	0.5	3.35E-06	0.26923077	0.00304	0.230769	0.00173
Regulation of TP53 Activity through Phosphorylation	0.3043478	3.52E-06	0.11956522	0.10224	0.130435	0.0026

mRNA Splicing - Major Pathway	0.2459016	3.69E-06	0.03825137	0.99082	0.103825	0.00281
Resolution of D-Loop Structures	0.4411765	4.29E-06	0.26470588	0.00091	0.205882	0.00148
Gap-filling DNA repair synthesis and ligation in TC-NER	0.3384615	5.73E-06	0.09230769	0.39762	0.184615	0.0001
Recognition of DNA damage by PCNA-containing replication complex	0.4516129	6.42E-06	0.06451613	0.70876	0.290323	1.75E-05
Nucleotide Excision Repair	0.2792793	7.89E-06	0.06306306	0.77349	0.144144	0.00017
Separation of Sister Chromatids	0.2393617	7.90E-06	0.05319149	0.92904	0.111702	0.00068
Fanconi Anemia Pathway	0.4	9.61E-06	0.15	0.08801	0.2	0.00084
Mitochondrial tRNA aminoacylation	0.5238095	1.09E-05	0.0952381	0.49621	0.142857	0.09095
PCNA-Dependent Long Patch Base Excision Repair	0.5238095	1.09E-05	0.14285714	0.22272	0.333333	5.74E-05
Mismatch Repair	0.6	1.67E-05	0.06666667	0.70483	0.4	6.12E-05
Processive synthesis on the lagging strand	0.6	1.67E-05	0.13333333	0.32934	0.333333	0.0007
Global Genome Nucleotide Excision Repair (GG-NER)	0.297619	1.78E-05	0.05952381	0.79517	0.154762	0.00034
E2F mediated regulation of DNA replication	0.5	1.94E-05	0.13636364	0.24407	0.318182	8.05E-05
Switching of origins to a post-replicative state	0.2888889	2.19E-05	0.03333333	0.9756	0.166667	5.03E-05
Resolution of AP sites via the multiple-nucleotide patch replacement pathway	0.4615385	2.27E-05	0.11538462	0.3316	0.307692	3.24E-05
Metabolism of nucleotides	0.2772277	2.48E-05	0.07920792	0.53664	0.079208	0.14933
snRNP Assembly	0.3461538	2.84E-05	0.01923077	0.98556	0.192308	0.00027
Metabolism of non-coding RNA	0.3461538	2.84E-05	0.01923077	0.98556	0.192308	0.00027
Branched-chain amino acid catabolism	0.4782609	3.31E-05	0.08695652	0.54578	0.173913	0.02832
Interactions of Rev with host cellular proteins	0.4	3.47E-05	0.02857143	0.94215	0.171429	0.00825
Protein localization	0.2519685	5.47E-05	0.03937008	0.97427	0.03937	0.79049
Complex I biogenesis	0.3272727	6.65E-05	NA	NA	0.090909	0.15234
SUMOylation	0.2245989	7.74E-05	0.0855615	0.3888	0.085561	0.03204
Rev-mediated nuclear export of HIV RNA	0.3939394	7.97E-05	0.03030303	0.9319	0.181818	0.00614
Methylation	0.5714286	8.20E-05	0.28571429	0.0196	0.142857	0.16054
Mismatch repair (MMR) directed by MSH2:MSH6 (MutSalpha)	0.5714286	8.20E-05	0.07142857	0.6798	0.428571	3.84E-05
Mismatch repair (MMR) directed by MSH2:MSH3 (MutSbeta)	0.5714286	8.20E-05	0.07142857	0.6798	0.357143	0.00048
Removal of the Flap Intermediate	0.5714286	8.20E-05	0.07142857	0.6798	0.285714	0.00462
Gap-filling DNA repair synthesis and ligation in GG-NER	0.44	8.61E-05	0.12	0.30956	0.28	0.0002
Viral Messenger RNA Synthesis	0.3571429	8.67E-05	0.02380952	0.96733	0.166667	0.0052
SUMOylation of DNA damage response and repair proteins	0.2894737	8.89E-05	0.05263158	0.85456	0.105263	0.04126
rRNA modification in the nucleus and cytosol	0.3064516	0.00011	0.0483871	0.87249	0.080645	0.21429

NEP/NS2 Interacts with the Cellular Export Machinery	0.4	0.00013	0.03333333	0.91303	0.2	0.00375
Base Excision Repair	0.3589744	0.00014	0.17948718	0.02939	0.230769	0.00013
Resolution of Abasic Sites (AP sites)	0.3589744	0.00014	0.17948718	0.02939	0.230769	0.00013
Regulation of HSF1-mediated heat shock response	0.2941176	0.00014	0.05882353	0.78812	0.132353	0.00784
Assembly of the pre-replicative complex	0.2941176	0.00014	0.05882353	0.78812	0.205882	7.40E-06
Peroxisomal protein import	0.3015873	0.00015	0.07936508	0.55228	0.047619	0.6374
Nucleobase biosynthesis	0.5333333	0.00016	0.13333333	0.32934	0.133333	0.17924
SUMO E3 ligases SUMOylate target proteins	0.2209945	0.00016	0.08839779	0.3392	0.082873	0.04695
DNA Damage Bypass	0.3265306	0.00017	0.06122449	0.74767	0.183673	0.00078
Deposition of new CENPA-containing nucleosomes at the centromere	0.2837838	0.00017	0.04054054	0.93564	0.108108	0.03602
Nucleosome assembly	0.2837838	0.00017	0.04054054	0.93564	0.108108	0.03602
Meiosis	0.2457627	0.00019	0.05084746	0.90711	0.101695	0.01838
G0 and Early G1	0.4074074	0.0002	0.07407407	0.63431	0.222222	0.00213
DNA Damage/Telomere Stress Induced Senescence	0.275	0.0002	0.0625	0.75875	0.1	0.05322
TP53 Regulates Transcription of DNA Repair Genes	0.2923077	0.00023	0.07692308	0.58068	0.153846	0.00168
Pyruvate metabolism and Citric Acid (TCA) cycle	0.3090909	0.00023	0.07272727	0.63188	0.018182	0.94597
Transport of Mature mRNAs Derived from Intronless Transcripts	0.3414634	0.00026	0.02439024	0.96455	0.195122	0.001
Export of Viral Ribonucleoproteins from Nucleus	0.375	0.00026	0.0625	0.72526	0.1875	0.00525
Nuclear import of Rev protein	0.375	0.00026	0.03125	0.92612	0.15625	0.02269
Termination of translesion DNA synthesis	0.375	0.00026	0.09375	0.46112	0.1875	0.00525
Orc1 removal from chromatin	0.2816901	0.00027	0.02816901	0.97846	0.183099	5.80E-05
RHO GTPases Activate Formins	0.2318841	0.00029	0.07246377	0.644	0.123188	0.00072
tRNA processing in the nucleus	0.3035714	0.00029	0.05357143	0.82376	0.142857	0.00756
Cellular response to heat stress	0.2613636	0.00033	0.07954545	0.53674	0.113636	0.01481
mRNA Splicing - Minor Pathway	0.3076923	0.00037	0.03846154	0.92164	0.173077	0.00121
Translesion synthesis by POLI	0.4705882	0.00047	0.11764706	0.3875	0.294118	0.00132
Transport of the SLBP Dependant Mature mRNA	0.3529412	0.0005	0.02941176	0.93724	0.176471	0.00714
Nuclear Pore Complex (NPC) Disassembly	0.3529412	0.0005	0.02941176	0.93724	0.176471	0.00714
Transport of Ribonucleoproteins into the Host Nucleus	0.3666667	0.00059	0.03333333	0.91303	0.166667	0.01746
Regulation of Glucokinase by Glucokinase Regulatory Protein	0.3666667	0.00059	0.03333333	0.91303	0.166667	0.01746
Calnexin/calreticulin cycle	0.3846154	0.00067	0.07692308	0.61352	0.076923	0.3906
N-glycan trimming in the ER and Calnexin/Calreticulin cycle	0.3428571	0.00067	0.08571429	0.52185	0.085714	0.26887
Polymerase switching on the C-strand of the telomere	0.5	0.00069	0.14285714	0.29966	0.357143	0.00048

Polymerase switching	0.5	0.00069	0.14285714	0.29966	0.357143	0.00048
Leading Strand Synthesis	0.5	0.00069	0.14285714	0.29966	0.357143	0.00048
Transport of Mature mRNA Derived from an Intronless Transcript	0.325	0.00073	0.025	0.96153	0.175	0.00393
Defects in vitamin and cofactor metabolism	0.4090909	0.00074	0.13636364	0.24407	NA	NA
Pyruvate metabolism	0.3548387	0.00081	0.09677419	0.44014	NA	NA
Diseases of metabolism	0.2380952	0.00085	0.1047619	0.19544	NA	NA
Glutathione conjugation	0.3333333	0.0009	0.02777778	0.94669	0.111111	0.11233
Mitotic Prophase	0.2198582	0.00094	0.02836879	0.99636	0.099291	0.01389
Processive synthesis on the C-strand of the telomere	0.5454545	0.00095	0.09090909	0.59125	0.363636	0.00172
SeMet incorporation into proteins	0.5454545	0.00095	NA	NA	NA	NA
mitochondrial fatty acid beta-oxidation of saturated fatty acids	0.5454545	0.00095	0.09090909	0.59125	0.181818	0.10721
Nuclear Envelope Breakdown	0.2941176	0.00095	0.03921569	0.91631	0.156863	0.00424
Regulation of TP53 Activity	0.2125	0.00103	0.09375	0.26685	0.08125	0.06956
Vpr-mediated nuclear import of PICs	0.34375	0.00109	0.03125	0.92612	0.15625	0.02269
Translesion Synthesis by POLH	0.4210526	0.00116	0.05263158	0.78688	0.263158	0.00227
Transcription of E2F targets under negative control by DREAM complex	0.4210526	0.00116	0.10526316	0.44333	0.263158	0.00227
Transport of the SLBP independent Mature mRNA	0.3333333	0.00146	0.03030303	0.9319	0.151515	0.02565
SUMOylation of SUMOylation proteins	0.3333333	0.00146	0.03030303	0.9319	0.151515	0.02565
Meiotic recombination	0.244186	0.00153	0.03488372	0.96874	0.081395	0.15434
Cytosolic tRNA aminoacylation	0.375	0.00154	0.04166667	0.85818	NA	NA
Formation of Incision Complex in GG-NER	0.3023256	0.00155	0.02325581	0.96989	0.116279	0.06868
Purine ribonucleoside monophosphate biosynthesis	0.5	0.0017	0.16666667	0.23983	0.083333	0.47027
Metabolism of cofactors	0.4	0.00173	NA	NA	0.1	0.27582
SUMOylation of chromatin organization proteins	0.2571429	0.00174	0.04285714	0.91878	0.085714	0.15116
Formation of TC-NER Pre-Incision Complex	0.2777778	0.00181	0.05555556	0.8043	0.111111	0.05815
Translesion synthesis by REV1	0.4375	0.00183	0.0625	0.72791	0.3125	0.00097
Transcription of E2F targets under negative control by p107 (RBL1) and p130 (RBL2) in complex with HDAC1	0.4375	0.00183	0.125	0.35866	0.1875	0.0462
Transcriptional regulation by small RNAs	0.2285714	0.00195	0.00952381	0.99981	0.095238	0.04391
Translesion synthesis by Y family DNA polymerases bypasses lesions on DNA template	0.3076923	0.00197	0.07692308	0.5966	0.153846	0.01395
Transport of Mature Transcript to Cytoplasm	0.2409639	0.00233	0.01204819	0.99886	0.108434	0.02661
Interactions of Vpr with host cellular proteins	0.3142857	0.00249	0.02857143	0.94215	0.142857	0.03227

ER Quality Control Compartment (ERQC)	0.3809524	0.0025	0.04761905	0.81892	0.095238	0.29528
Deadenylation-dependent mRNA decay	0.2678571	0.00267	0.05357143	0.82376	0.017857	0.94877
Reproduction	0.2083333	0.00272	0.05555556	0.88347	0.083333	0.06811
Translesion synthesis by POLK	0.4117647	0.00278	0.05882353	0.74919	0.294118	0.00132
HIV Life Cycle	0.205298	0.00296	0.05298013	0.9115	0.099338	0.01107
Phase II - Conjugation of compounds	0.2201835	0.0033	0.05504587	0.86303	0.091743	0.05424
Cellular responses to stress	0.1690141	0.00341	0.05868545	0.95153	0.075117	0.02048
RNA Polymerase I Transcription Initiation	0.2765957	0.00372	0.06382979	0.72161	0.06383	0.4393
SUMOylation of ubiquitinylation proteins	0.2972973	0.00404	0.02702703	0.95086	0.135135	0.03986
Removal of the Flap Intermediate from the C-strand	0.5	0.00427	NA	NA	0.3	0.01245
RNA Polymerase I Transcription	0.2142857	0.00475	0.02678571	0.99406	0.0625	0.35603
tRNA modification in the nucleus and cytosol	0.2790698	0.00483	0.13953488	0.11525	0.069767	0.38327
Cellular Senescence	0.1897436	0.00509	0.07179487	0.66828	0.076923	0.07869
Processing of Capped Intronless Pre-mRNA	0.3214286	0.00513	0.03571429	0.89763	0.285714	5.88E-05
Fatty acid metabolism	0.1920904	0.0058	0.10734463	0.09682	0.033898	0.89972
SUMOylation of DNA replication proteins	0.2727273	0.00591	0.04545455	0.86838	0.136364	0.02434
NS1 Mediated Effects on Host Pathways	0.2820513	0.00628	0.02564103	0.95826	0.128205	0.04846
mRNA decay by 5' to 3' exoribonuclease	0.4	0.00663	0.06666667	0.70483	0.066667	0.54812
RNA Polymerase I Promoter Clearance	0.2110092	0.00685	0.02752294	0.99277	0.055046	0.49453
Meiotic synapsis	0.2278481	0.00703	0.03797468	0.95217	0.088608	0.11196
Establishment of Sister Chromatid Cohesion	0.4545455	0.00703	0.09090909	0.59125	0.090909	0.44145
Condensation of Prometaphase Chromosomes	0.4545455	0.00703	0.09090909	0.59125	0.181818	0.10721
SLBP Dependent Processing of Replication-Dependent Histone Pre-mRNAs	0.4545455	0.00703	NA	NA	0.363636	0.00172
Gene Silencing by RNA	0.2	0.00762	0.01481481	0.9998	0.081481	0.08863
Epigenetic regulation of gene expression	0.1959459	0.00781	0.02702703	0.99766	0.081081	0.08001
Inhibition of the proteolytic activity of APC/C required for the onset of anaphase by mitotic spindle checkpoint components	0.35	0.00792	0.1	0.47017	0.15	0.08092
Inactivation of APC/C via direct inhibition of the APC/C complex	0.35	0.00792	0.1	0.47017	0.15	0.08092
Transport of Mature mRNA derived from an Intron-Containing Transcript	0.2297297	0.00795	0.01351351	0.99761	0.081081	0.18116
APC-Cdc20 mediated degradation of Nek2A	0.32	0.00844	0.08	0.59184	0.12	0.13563
Interconversion of nucleotide di- and triphosphates	0.3	0.00846	0.03333333	0.91303	0.166667	0.01746
SUMOylation of RNA binding proteins	0.2608696	0.00863	0.04347826	0.88418	0.130435	0.02965

Nonhomologous End-Joining (NHEJ)	0.2318841	0.00899	0.05797101	0.79758	0.057971	0.47903
Metabolism of vitamins and cofactors	0.1851852	0.00925	0.07936508	0.51304	0.042328	0.76506
Apoptotic execution phase	0.25	0.00933	0.11538462	0.21762	0.038462	0.75662
Host Interactions with Influenza Factors	0.2682927	0.00939	0.02439024	0.96455	0.121951	0.05806
Formation of Senescence-Associated Heterochromatin Foci (SAHF)	0.375	0.00952	0.1875	0.12408	0.1875	0.0462
Positive epigenetic regulation of rRNA expression	0.2075472	0.00981	0.01886792	0.99826	0.075472	0.18002
Late Phase of HIV Life Cycle	0.1956522	0.01022	0.05072464	0.92156	0.101449	0.01165
Glyoxylate metabolism and glycine degradation	0.2903226	0.01064	0.06451613	0.70876	0.064516	0.47976
Tetrahydrobiopterin (BH4) synthesis, recycling, salvage and regulation	0.4166667	0.01084	NA	NA	0.083333	0.47027
Ethanol oxidation	0.4166667	0.01084	0.16666667	0.23983	0.083333	0.47027
Cyclin E associated events during G1/S transition	0.2168675	0.01183	0.06024096	0.78649	0.120482	0.01002
RNA Polymerase I Chain Elongation	0.2111111	0.01314	0.01111111	0.99936	0.077778	0.18144
Metabolism of nitric oxide	0.3529412	0.01319	0.05882353	0.74919	0.058824	0.59357
eNOS activation and regulation	0.3529412	0.01319	0.05882353	0.74919	0.058824	0.59357
RNA Polymerase I Transcription Termination	0.28125	0.01321	0.03125	0.92612	0.09375	0.22679
RNA Pol II CTD phosphorylation and interaction with CE during HIV infection	0.2962963	0.01384	0.03703704	0.88894	0.148148	0.04779
RNA Pol II CTD phosphorylation and interaction with CE	0.2962963	0.01384	0.03703704	0.88894	0.148148	0.04779
Citric acid cycle (TCA cycle)	0.3181818	0.01397	NA	NA	0.045455	0.68821
Cyclin A:Cdk2-associated events at S phase entry	0.2117647	0.01505	0.05882353	0.80357	0.117647	0.01177
Metabolism of water-soluble vitamins and cofactors	0.195122	0.01534	0.08130081	0.49513	0.02439	0.9564
Cleavage of Growing Transcript in the Termination Region	0.2238806	0.01546	0.01492537	0.99576	0.134328	0.00712
RNA Polymerase II Transcription Termination	0.2238806	0.01546	0.01492537	0.99576	0.134328	0.00712
Biological oxidations	0.1748879	0.01582	0.07623318	0.57807	0.058296	0.36416
Apoptosis induced DNA fragmentation	0.3846154	0.01584	NA	NA	NA	NA
The activation of arylsulfatases	0.3846154	0.01584	0.30769231	0.01491	0.230769	0.02645
eNOS activation	0.3846154	0.01584	NA	NA	0.076923	0.4976
Activation of DNA fragmentation factor	0.3846154	0.01584	NA	NA	NA	NA
Synthesis of PC	0.2857143	0.01732	0.21428571	0.01895	0.071429	0.42717
Synthesis of IP3 and IP4 in the cytosol	0.2857143	0.01732	0.21428571	0.01895	NA	NA
Condensation of Prophase Chromosomes	0.2162162	0.01749	0.01351351	0.99761	0.067568	0.33375
RNA Polymerase III Chain Elongation	0.3333333	0.01776	0.05555556	0.7688	0.166667	0.06243

Protein folding	0.2020202	0.01781	0.06060606	0.79514	0.131313	0.00165
Gamma carboxylation, hypusine formation and arylsulfatase activation	0.2564103	0.0179	0.15384615	0.07981	0.153846	0.01395
Association of TriC/CCT with target proteins during biosynthesis	0.2564103	0.0179	0.07692308	0.5966	0.102564	0.13952
APC/C:Cdc20 mediated degradation of Cyclin B	0.3043478	0.01798	0.04347826	0.84614	0.130435	0.11243
BBSome-mediated cargo-targeting to cilium	0.3043478	0.01798	0.13043478	0.26571	0.086957	0.33392
Mitochondrial calcium ion transport	0.3043478	0.01798	0.04347826	0.84614	NA	NA
RHO GTPase Effectors	0.164557	0.01898	0.06329114	0.86626	0.072785	0.05963
NoRC negatively regulates rRNA expression	0.1981132	0.01904	0.00943396	0.99983	0.075472	0.18002
Metabolism of carbohydrates	0.1672727	0.02011	0.10545455	0.05925	0.069091	0.11842
Peroxisomal lipid metabolism	0.2758621	0.02139	0.17241379	0.07123	0.068966	0.44502
Mitophagy	0.2758621	0.02139	0.03448276	0.90565	0.034483	0.78493
mRNA Capping	0.2758621	0.02139	0.03448276	0.90565	0.137931	0.05972
Glycogen metabolism	0.2758621	0.02139	0.17241379	0.07123	NA	NA
Defects in cobalamin (B12) metabolism	0.3571429	0.02218	0.14285714	0.29966	NA	NA
TP53 Regulates Transcription of Genes Involved in G1 Cell Cycle Arrest	0.3571429	0.02218	0.14285714	0.29966	0.071429	0.52353
Recruitment and ATM-mediated phosphorylation of repair and signaling proteins at DNA double strand breaks	0.2105263	0.02225	0.05263158	0.85456	0.065789	0.35445
Glycolysis	0.2142857	0.02267	0.07142857	0.64719	0.114286	0.02692
Phosphorylation of the APC/C	0.3157895	0.02329	0.05263158	0.78688	0.157895	0.07141
Protein methylation	0.3157895	0.02329	NA	NA	0.105263	0.25633
Transcriptional Regulation by E2F6	0.2571429	0.02369	0.08571429	0.52185	0.142857	0.03227
DNA Double Strand Break Response	0.2077922	0.02496	0.05194805	0.86147	0.064935	0.36482
Negative epigenetic regulation of rRNA expression	0.1926606	0.02544	0.00917431	0.99986	0.073394	0.19966
RNA Polymerase I Promoter Escape	0.2666667	0.0261	0.03333333	0.91303	0.1	0.19951
SLBP independent Processing of Histone Pre-mRNAs	0.4	0.02662	NA	NA	0.3	0.01245
Cohesin Loading onto Chromatin	0.4	0.02662	0.1	0.55659	0.1	0.41106
Folding of actin by CCT/TriC	0.4	0.02662	NA	NA	0.1	0.41106
HDR through MMEJ (alt-NHEJ)	0.4	0.02662	NA	NA	0.1	0.41106
Ketone body metabolism	0.4	0.02662	0.2	0.18074	0.3	0.01245
Pentose phosphate pathway	0.3333333	0.02995	NA	NA	0.066667	0.54812
Neutrophil degranulation	0.1524008	0.03375	0.07098121	0.74771	0.052192	0.50449
TP53 Regulates Transcription of Cell Cycle Genes	0.2244898	0.03454	0.08163265	0.53897	0.081633	0.24487

Cellular responses to external stimuli	0.1510934	0.03657	0.05964215	0.95623	0.063618	0.12619
Biotin transport and metabolism	0.3636364	0.03782	0.09090909	0.59125	NA	NA
Apoptotic cleavage of cell adhesion proteins	0.3636364	0.03782	0.09090909	0.59125	0.090909	0.44145
Organelle biogenesis and maintenance	0.1598639	0.03881	0.0952381	0.15783	0.07483	0.0505
Apoptotic cleavage of cellular proteins	0.2368421	0.03904	0.13157895	0.17165	0.052632	0.59017
mRNA decay by 3' to 5' exoribonuclease	0.3125	0.03925	NA	NA	NA	NA
Processing of SMDT1	0.3125	0.03925	0.0625	0.72791	NA	NA
ERCC6 (CSB) and EHMT2 (G9a) positively regulate rRNA expression	0.1973684	0.04425	0.02631579	0.98482	0.052632	0.55571
Formation of the Early Elongation Complex	0.2424242	0.04439	0.06060606	0.74097	0.090909	0.24069
Formation of the HIV-1 Early Elongation Complex	0.2424242	0.04439	0.06060606	0.74097	0.090909	0.24069
Pink/Parkin Mediated Mitophagy	0.2727273	0.04643	NA	NA	0.045455	0.68821
Mitochondrial protein import	0.203125	0.04786	NA	NA	0.03125	0.84941
Signaling by Nuclear Receptors	0.1658291	0.04846	0.08040201	0.4894	0.045226	0.70407
Glucose metabolism	0.1888889	0.0488	0.07777778	0.56112	0.088889	0.09201
Nuclear Receptor transcription pathway	0.12	0.59818	0.24	0.00036	0.06	0.48001
G alpha (12/13) signalling events	0.0759494	0.93786	0.17721519	0.00294	0.063291	0.38558
Sema4D induced cell migration and growth-cone collapse	0.0952381	0.75361	0.28571429	0.00436	NA	NA
Rho GTPase cycle	0.1014493	0.82484	0.14492754	0.00511	0.028986	0.93042
Hydrolysis of LPC	0.1	0.73386	0.4	0.00529	NA	NA
NRAGE signals death through JNK	0.0677966	0.94527	0.18644068	0.00537	0.050847	0.59268
HS-GAG degradation	0.0909091	0.77649	0.27272727	0.0056	0.090909	0.31466
Metabolism of lipids	0.1316147	0.27221	0.10312076	0.00669	0.040706	0.93264
Sema4D in semaphorin signaling	0.08	0.83431	0.24	0.01087	NA	NA
Phospholipid metabolism	0.1367925	0.31267	0.12264151	0.01417	0.042453	0.77119
Inositol phosphate metabolism	0.2	0.08355	0.18	0.01417	0.02	0.92951
Cell death signalling via NRAGE, NRIF and NADE	0.0789474	0.92224	0.15789474	0.01418	0.039474	0.7583
Diseases of carbohydrate metabolism	0.1764706	0.23979	0.20588235	0.0144	NA	NA
p75 NTR receptor-mediated signalling	0.0824742	0.92611	0.1443299	0.01801	0.030928	0.88306
Acyl chain remodelling of PC	0.1071429	0.69215	0.21428571	0.01895	NA	NA
Stimuli-sensing channels	0.0917431	0.8828	0.13761468	0.02192	0.045872	0.66834
Glycerophospholipid biosynthesis	0.1705426	0.07355	0.13178295	0.02269	0.054264	0.50009
Chromatin modifying enzymes	0.1418182	0.20418	0.11272727	0.02412	0.069091	0.11842
Chromatin organization	0.1418182	0.20418	0.11272727	0.02412	0.069091	0.11842

Laminin interactions	0.0666667	0.9013	0.2	0.02616	0.1	0.19951
Glycogen storage diseases	0.1875	0.31796	0.25	0.03148	NA	NA
Ion channel transport	0.0913978	0.93436	0.11827957	0.03267	0.032258	0.92293
Base-Excision Repair, AP Site Formation	0.2	0.357	0.3	0.03757	0.1	0.41106
Sphingolipid metabolism	0.1797753	0.07925	0.13483146	0.04329	0.078652	0.17448
Circadian Clock	0.0571429	0.97995	0.14285714	0.04427	0.028571	0.88257
Synthesis of glycosylphosphatidylinositol (GPI)	0.2777778	0.06254	0.22222222	0.04677	0.055556	0.61455
Bile acid and bile salt metabolism	0.1395349	0.44534	0.1627907	0.04711	0.046512	0.65777
Death Receptor Signalling	0.070922	0.98553	0.12056738	0.04776	0.028369	0.93732
GAB1 signalosome	NA	NA	0.27272727	0.04873	NA	NA
CYP2E1 reactions	0.0909091	0.76688	0.27272727	0.04873	NA	NA
Mucopolysaccharidoses	0.1818182	0.40371	0.27272727	0.04873	NA	NA
Type I hemidesmosome assembly	NA	NA	0.27272727	0.04873	NA	NA
RHO GTPases activate KTN1	NA	NA	0.27272727	0.04873	NA	NA
Chk1/Chk2(Cds1) mediated inactivation of Cyclin B:Cdk1 complex	0.3076923	0.06706	NA	NA	0.384615	0.00033
Chaperonin-mediated protein folding	0.1827957	0.0634	0.06451613	0.74339	0.129032	0.00285
Intraflagellar transport	0.2115385	0.05088	0.09615385	0.38323	0.153846	0.00479
Polo-like kinase mediated events	0.25	0.12697	0.0625	0.72791	0.25	0.00774
Cyclin A/B1/B2 associated events during G2/M transition	0.24	0.08017	NA	NA	0.2	0.00804
MASTL Facilitates Mitotic Progression	0.2	0.357	NA	NA	0.3	0.01245
Cilium Assembly	0.1507538	0.14706	0.09045226	0.29125	0.090452	0.01441
Processing of Intronless Pre-mRNAs	0.2105263	0.20225	0.05263158	0.78688	0.210526	0.01458
Cooperation of PDCL (PhLP1) and TRiC/CCT in G-protein beta folding	0.2142857	0.06839	0.07142857	0.64741	0.142857	0.01971
Thrombin signalling through proteinase activated receptors (PARs)	0.03125	0.9856	0.0625	0.72526	0.15625	0.02269
Cyclin D associated events in G1	0.2045455	0.08714	0.11363636	0.25764	0.136364	0.02434
G1 Phase	0.2045455	0.08714	0.11363636	0.25764	0.136364	0.02434
ADP signalling through P2Y purinoceptor 12	0.0909091	0.77649	0.04545455	0.83308	0.181818	0.02436
CDT1 association with the CDC6:ORC:origin complex	0.2033898	0.05541	0.01694915	0.99185	0.118644	0.03117
HIV Transcription Initiation	0.212766	0.05893	0.04255319	0.8914	0.12766	0.03256
RNA Polymerase II HIV Promoter Escape	0.212766	0.05893	0.04255319	0.8914	0.12766	0.03256
RNA Polymerase II Promoter Escape	0.212766	0.05893	0.04255319	0.8914	0.12766	0.03256
RNA Polymerase II Transcription Pre-Initiation And Promoter Opening	0.212766	0.05893	0.04255319	0.8914	0.12766	0.03256

RNA Polymerase II Transcription Initiation	0.212766	0.05893	0.04255319	0.8914	0.12766	0.03256
RNA Polymerase II Transcription Initiation And Promoter Clearance	0.212766	0.05893	0.04255319	0.8914	0.12766	0.03256
Cytosolic sulfonation of small molecules	0.0833333	0.81673	0.04166667	0.85818	0.166667	0.03264
Transcription of the HIV genome	0.1643836	0.18777	0.04109589	0.93175	0.109589	0.03358
Prefoldin mediated transfer of substrate to CCT/TriC	0.2307692	0.09381	NA	NA	0.153846	0.04238
