# Supplementary material for: Comparative transcriptional profiling of the early host response to infection by typhoidal and non-typhoidal Salmonella serovars in human intestinal organoids
Source: PLoS Pathog. 2021 Oct 20;17(10):e1009987. doi: 10.1371/journal.ppat.1009987 (PMC8570492; doi:10.1371/journal.ppat.1009987)
Supplement: Attachment — Submitted filename: Response to Reviewers.docx [file ppat.1009987.s016.docx]

We appreciate the interest of the reviewers in the findings of our study, as well as suggestions to improve the manuscript, especially regarding additional characterization of the organoid infection model as well as comparisons to previous *Salmonella* transcriptomics studies.

**Major issues**

*Reviewer #1: • The intestinal organoid – Salmonella infection model is relatively superficially characterized here. (1) Although the authors do not observe any major epithelial tissue damage, did they quantitatively measure cell death to detect more subtle differences between the infections with the different serovars and/or over time?*

We have added additional characterization of the infection model by providing TUNEL analysis in Figure 5E-F to further investigate the cell death phenotype. While we found that the HIOs largely remained intact, there were some regions where we observed an increase in TUNEL-positive cells in *S*. Typhimurium-infected HIOs, but not during infection with the other serovars. These findings support our H&E analysis in Figure 1 where we observed some damaged regions in the HIOs during *S*. Typhimurium infection that were defined by extruding cells from the epithelial monolayer. We have clarified this point in the main text as well.

*(2) Did they perform assays (e.g. imaging-based) to distinguish between infection rate and intracellular replication? Note that determination of the intracellular load (Fig. 1B) cannot discriminate between these two parameters.*

We appreciate this comment; while we cannot definitively separate infection rate and intracellular replication in fixed tissue, we did our best to address this question by providing image quantifications of intracellular bacterial load and percentage of infected cells in Figure 1D-F. We found that with all three serovars there were a number of infected host cells that had greater than one bacterium, but that there were also many singly-infected cells. These findings suggest that there is a combination of continued invasion and intracellular replication that leads to an increase in intracellular burden at the later time point.

*(3) To what extent does the infection cycle of the different serovars depend on the major pathogenicity islands (SPI-1, SPI-2)? Could the authors combine established reporters strains with infection time course experiments to address this? All this information would be important to better interpret the transcriptomics data. For example, it is currently hard to disentangle to what extent the serovar-specific host responses are due to dose effects (e.g. different percentages of invaded host cells, altered intracellular replication kinetics), different bacterial virulence induction kinetics, different localization, etc.*

These three serovars may indeed have similar or different requirements for SPI-1 and SPI-2 to colonize and invade the HIOs, which could lead to differential host responses. However, this is still an indication of serovar-specific pathogenesis that drives differential host responses. The primary aim of this study was to characterize the host response to wild-type bacteria and identifying the contribution of SPI-1 and SPI-2 to these outcomes is an additional question that would require rigorous investigation. We recently characterized *S.* Typhimurium SPI-1 and SPI-2-dependent host responses in the HIO model (*Lawrence, Abuaita, et al, mBio 2021* [*https://doi.org/10.1128/mBio.00399-21*](https://doi.org/10.1128/mBio.00399-21)), but further characterization with the other serovars will be addressed in future studies. However, we have provided expression data of SPI-1 and SPI-2 effectors (Supplementary Figure 1) showing variations in expression of several effector genes that may explain some differences in the infection cycle between serovars.

*• Validation and functional assessment of serovar-specific host responses: This is an interesting part of the study. However, not all serovar-specific host responses prominently discussed in the text were confirmed via independent methods. For example, the inhibition of cell cycle progression could be supported by qRT-PCR-based validation of cell cycle-associated mRNAs and/or via cell cycle analysis of DNA-stained cells by flow cytometry. Besides, following the authors’ hypothesis that the SPI-2 effectors SseF/G might contribute to this host effect, expression of these effectors may be measured and compared in the different serovars during infection of the organoid model.*

We have provided validation of the cell cycle phenotype by monitoring cellular proliferation by performing EdU staining and quantitation in Figure 5C-D. These results are largely consistent with our transcriptomics analysis and show that STM infection leads to a significant reduction in the number of EdU-positive cells, suggesting that there are fewer proliferating cells in these HIOs. We did not measure a significant change in the number of EdU cells in SE or ST-infected HIOs correlating with a reduced enrichment of cell cycle pathways in these conditions.

We also proposed in the discussion that SPI-2 effectors may contribute to changes in cell cycle regulation and to further address this hypothesis, we analyzed expression of several SPI-2 effectors across the three serovars during infection including SseF/G which are now presented in Figure S1. We found that SseF/G expression increased at 8h during ST and SE infection and concluded that there may be alternative mechanisms leading to cell cycle suppression in the HIOs (lines 511-514).

*• A comprehensive body of literature exists on measuring host responses to Salmonella Typhimurium infection via transcriptomics approaches, but few papers are cited in this context. E.g. the important, early studies from the Galan lab, who measured the epithelial host response to Salmonella infection via microarray (PMID: 19662166; 24098123), should be mentioned in the introduction and/or discussion section. Likewise, given the rationale of the current study to use more complex models for Salmonella infection studies, papers that combined RNA-seq with tissue engineering and Salmonella infection should be discussed (e.g. PMID: 32071273). In particular, to demarcate the here identified host responses to S. Typhimurium from those previous models that were based on immortalized cells, the authors should extend their study and cross-compare the global host response profile between the present and those previous transcriptomics dataset (also including the data from refs. [7], [8]and [22]). Which branches of the host response to S. Typhimurium are shared among the different infection models and which ones are specific to the organoid model? This cross-comparison would be important to support the authors’ claim that simple cell cultures models fail to recapitulate important aspects of the epithelial host response to infection (see e.g. p. 13, second paragraph).*

We have provided a more detailed bioinformatics comparison between our RNA-seq results with previously published transcriptomics studies now presented in supplementary Figure 4 and described in the results section (Lines 195-203). A more comprehensive discussion has been included and the above-mentioned studies are now referenced in the discussion as well (Lines 462-479). To reduce variability, we restricted our analysis to those performed using RNA-seq instead of microarray. We compared the top 30-50 significant genes from each study that were published to our significant gene sets.

*Reviewer #2: - The authors analyzed merely one strain per serovar. Due to substantial strain to strain differences at the level of gene content and virulence (e.g., see doi:10.1128/IAI.74.1.632–644.2006), it remains unclear, if the observed virulence phenotypes will indeed be representative for the entire serovar.*

Although defining host specific responses to different strains within a specific serovar is important, that is beyond the scope of this study. To compare the infectivity and host responses to several strains of each serovar would require generation of many additional HIOs, work that might take at least 6 additional months if not more. It would also not be feasible to perform all these experiments in an individual day to directly compare and contrast infections of all three serovars with multiple strains due to the time-consuming procedure of microinjecting bacteria into the organoid lumen. We have chosen to study well characterized strains of these three serovars to establish baseline differences between them, and we agree that future studies should be performed to answer this question.

*- S. Typhi strains tend to lose their capacity of casule gene expression during in vitro passage. As capsules massively affect virulence (e.g., see doi: 10.1128/mBio.00579-12 and doi: 10.1128/IAI.00172-09.) the authors should explain in the main text, if the tested strain expresses a capsule, or not.*

We tested for presence of capsule after overnight growth to determine whether the bacteria that were microinjected into the HIOs were expressing capsule under our culture conditions, and also stained histology sections of infected HIOs to determine if capsule is expressed during infection. These results are shown in Supplementary Figure 7 and described in the Results section (Line 276-278).

*- Fig 1A+B show differences in bacterial loads for the different serovars. Do these differences influence triggered responses? Can the inoculum be tweaked in a way to end up with similar numbers of bacteria per condition to ensure that it is not bacterial loads alone which result in the differences in responses analyzed in this manuscript? Do the bacterial colonies within IECs look similar? Is there a formation of microcolonies indicating intracellular proliferation? Is the localization of the different serovars within the IECs the same or different? Are specific IEC subsets targeted by the different serovars?*

*- Please include bacterial loads for 8 hpi time points, as this time point is analyzed throughout the manuscript*

We microinjected the same CFU per serovar into the lumen of the HIOs in a given experiment; we interpret the differences in invasion between these three serovars as representing the biological variability of infection. Changing the relative inoculum between serovars would presumably lead to higher PAMP exposure in any effort to normalize intracellular bacteria. Additionally, due to luminal replication of the bacteria, even altering the initial inoculum might not lead to equivalent intracellular CFU at later time points. We have previously characterized the HIO response to WT *Salmonella* Typhimurium and the SPI-1 mutant that has a severe invasion defect (*Lawrence, Abuaita, et al, mBio 2021* [*https://doi.org/10.1128/mBio.00399-21*](https://doi.org/10.1128/mBio.00399-21)). Much of the gene expression program revealed by RNAseq occurred in both WT and SPI-1 infections suggesting that a dominant component of the HIO response is driven by luminal bacteria. In this study, we also found that some responses, including cytokine production, did not correlate with intracellular bacterial burden (Figure 4D).

We have now provided image quantification to determine percentage of infected cells, as well as intracellular bacterial load and localization of all 3 serovars at 8hpi (Figure 1D-F). These results are consistent with CFU data at 24h as STM infected more cells and had higher intracellular bacterial load per cell, compared to SE and ST. The percentage of infected cells in SE- and ST-injected HIOs as well as their intracellular load were comparable.

The bacterial burden for the 8h time point is now presented in Figure S3.

Reviewer #3:

Major comments

*- Intestinal model (Figure 1): the intestinal organoids would deserve a better characterization to demonstrate that the major cellular types expected in the intestine are recapitulated.*

This has previously been characterized by our collaborator, Dr. Jason Spence (please see reference [3])

*- Infection assay: it is not clear what is the localization of the bacteria. Is the majority of the bacteria extracellular or intracellular? What is the percentage of infected cells? Panel A and B have a different scale and they should be combined in one plot to show the relative proportion of the extracellular/intracellular bacteria. This said, it is not clear how it is possible that the bacteria are still extracellular as a gentamicin treatment is applied*?

We have provided a model in Figure 1A to more clearly explain the experimental set-up of the HIO model. The HIO epithelial cells self-organize into 3-dimensional structures forming a lumen where the bacteria are microinjected. Due to the cell-impermeable nature of gentamicin, the bacteria present within the lumen of the HIOs as well as those that have invaded the epithelium would be gentamicin-protected. The majority of the bacteria appear to be present within the lumen of the HIO instead of residing within epithelial cells. We have added additional data to Figure 1, which now includes the percentage of infected cells as quantified by immunofluorescence microscopy in Figure 1E. Total and intracellular bacterial loads originally presented in panel A and B are now in panels B and C respectively and the scales have been adjusted to be consistent between the two assays.

*- Dose of infection: it is not clear why two doses of infection have been used 10^5 CFU/HIO and 10^3 CFU/HIO as input for 2.5 and 24 hrs. The experimental design should be clarified in the text. Instead of CFU/HIO, the authors should use the MOI to quantify the infection.*

The two doses of infection were based on the anticipated endpoint of the experiment. If the experiment was intended to reach an 8h timepoint, we microinjected 10^5 bacteria so that more epithelial cells were likely to become infected early, a suitable protocol for population RNA-seq analysis at 2.5h and 8h. We have emphasized this rationale more in the revised text (Lines 173-175). However, this larger inoculum can result in more damage to the infected HIOs at 24h and limits overall bacterial replication since nutrients within the HIO are quickly consumed. If the experiment was intended to mimic a more natural infection, we microinjected 10^3 bacteria which allowed for a less efficient initial infection and more replication over time, with the infected HIOs largely remaining intact over 24h.

*- Most probably, many extracellular bacteria are dead. Live/dead should be quantified. It would be also very interesting to show a complete microscopic picture of the organoids before and after infection with a bacteria staining (for example with a fluorescently tagged Salmonella).*

The extracellular luminal bacteria are protected from gentamicin by the epithelial barrier. Live/dead staining only works for live staining and it is difficult to image the entire HIO at the resolution required to see individual bacteria by live microscopy. Unfortunately, fluorescently-tagged strains of *Salmonella* lose their fluorescence during fixation and histological processing. However, we have provided a movie for the reviewers showing that *Salmonella* is motile within the HIO lumen suggesting that many bacteria are still viable (*Reviewer material: Movie R1*).

*- Figure 1A and 1B: if so few bacteria are present intracellularly (10 times less compared to the extracellular ones), it is not clear if the replication observed between 2.5 and 8 hpi is coming from the intracellular milieu or just extracellularly. This point should be clarified experimentally.*

Our data suggest that bacterial replication occurs both in the lumen and within epithelial cells. Beyond observing an increase in both the total and intracellular CFU at 24h pi compared to the initial timepoint in Figures 1B-C, we now provide quantitation of immunofluorescence images in Fig. 1D to show that many cells contain multiple bacteria, consistent with intracellular replication.

*- Figure 1C and D: What is the information exactly convey on these figures? In the text (pages 6 and 7), the authors claim that the figure demonstrates that mucus production is increased but only a single field of view is provided and no quantification allows to measure the extend of the mucus production.*

We now provide quantitation of the Alcian Blue/PAS staining in Supplemental Figure S2.

*- Figure 2 nicely displays the transcriptomics data using a principal component analysis and demonstrates that the host response of the mucosal layer is different when exposed to the different serovar. Figure 2A could be completed with PCA specific for each time point.*

We now provide these PCA plots in Figure 2B-C.

*- Figure 3: it is not exactly clear what input has been used to generate the different panels. Figure 3C needs major improvement: what do these volcano plots demonstrate exactly?*

We have removed the volcano plots showing how cell cycle gene expression changes during infection; a more detailed characterization of cell cycle gene expression changes during infection is now highlighted in Fig. 5.

*- Overall Figure 2A, Figure 3B, and Figure 4A-C show that the three serovars elicit a pretty similar host response and also major differences (Fig 2B). While the authors show a venn diagram in figure 2B, it would be also interesting also to display a heatmap that captures the common response and differences between the conditions (x axis the conditions and y axis the genes). For clarity, the authors can display the top 50/100 genes for example between the different conditions.*

The top similar and different responses between the serovars are now included as heatmaps in Figure 2 F-G.

--------------------

Part III – Minor Issues: Editorial and Data Presentation Modifications

*Reviewer #1: • The authors switch back and forth between “post infection” (e.g. p. 6) and “post injection” (e.g. legend to Fig. 2). In order to avoid confusion, they should settle on one and use this expression consistently throughout the manuscript*.

We have changed the text to use “post infection” throughout the manuscript.

*• Page 6, last line: It is mentioned that alcian blue staining was performed to detect the mucus layer. However, neither is this staining procedure described in the methods section, nor are any data shown from alcian blue staining.*

We added more detail to H&E and Alcian Blue/PAS staining detail to the Methods. We changed the label of Figure 1G and also modified the text to state Alcian Blue/PAS.

*• ELISA assays: The authors measured cytokine/chemokine concentrations in the supernatant. This analysis revealed divergent, serovar-specific secreted protein levels despite similar transcript levels. As an explanation, the authors propose post-transcriptional control of cytokine/chemokine mRNAs to be responsible for the different secretion levels into the supernatant. However, did they also determine cytokine/chemokine release into the luminal compartment? Beyond mere reference to previous studies, a bit more experimental support for the proposed post-transcriptional control mechanism in the present context would be appreciated*.

What cytokines are released into the luminal compartment is indeed an interesting question, although technically challenging to answer. The goal of the study was to compare global gene expression patterns between serovars to uncover key differences for future studies. We proposed post-transcriptional control as a possible explanation for some of our observations, but a rigorous investigation into this specific question will benefit from its own separate study.

*• Page 14, second paragraph: “showed [d]ifferential”*…

Corrected.

*• Page 16, second line: “Salmonella” should be italicized.*

Corrected.

*• Page 18: Please better explain when the two CFU values (10^5 and 10^3) per HIO were used. I assume the low dose for the 24 h time point and the high dose for early time points, but this is not directly clear from this sentence.*

We have clarified this in the text (Line 173-175, 588-590).

*• Fig. 1B: To selectively determine the intracellular bacterial load, the HIOs were cut open and treated with gentamicin for 10 min. However, it remains unclear if this treatment was sufficient to effectively kill all remaining luminal bacteria (particularly, as it is unclear if the host mucus would provide a barrier against the antibiotic). An important control to include here, would be a SPI-1-deficient Salmonella mutant injected into the HIO lumen. If their gentamicin protection assay selectively recovers intracellular bacteria, no CFUs would be expected for the SPI-1 mutant.*

We have established this protocol using WT vs SPI-1 mutant STM in *Lawrence, Abuaita, et al, mBio 2021* [*https://doi.org/10.1128/mBio.00399-21*](https://doi.org/10.1128/mBio.00399-21). and have clarified this point in the main text (line 127-129).

*• Page 19: The methods section is relatively vague when it comes to the RNA-seq experiments. Can the authors please add more detail to these sections? It would e.g. be appreciated if the kits used for cDNA library preparation were indicated, if the authors described if/how the RNA samples were fragmented, how deep the resulting cDNA libraries were sequenced, etc. Moreover, it is common practice (and a prerequisite for publication in many journals) that all raw sequencing data referred to in a given study are uploaded on a publically accessible repository (e.g. the GEO database). In the present case, the authors simply state that these data are available upon request (p. 20).*

We modified the RNAseq method section to include these suggestions (lines 649-657). All sequences are now deposited in the EMBL-EBI Arrayexpress database (E-MTAB-10451).

*• Figures: It is generally preferable to plot individual data points rather than (or in addition to) bar charts of the average/mean.*

This has been corrected.

*• Fig. 4: It would be appreciated if the cytokines/chemokines measured in the ELISA experiments were highlighted in the RNA-seq heatmaps (panels A-C), e.g. in bold font. Additionally, I couldn’t find the mRNA expression data for BD-2.*

We have clarified this by better labelling of the graph titles especially for BD-2 to indicate the gene name DEFB4.

*• Legend to Fig. 5D: Please define “ROS MFI Ratio”.*

This has been defined in the figure legend (now Fig. 6D; lines 429-432).

*Reviewer #2: - Please indicate the stated “damaged regions of the HIO epithelial lining” referred to at the bottom of page 6 (Fig 1C)*

We have clarified this in the text (lines 157-159).

*- Please provide a quantitative analysis for Fig 1C+D*

We have provided quantitation of the Alcian Blue/PAS staining in supplemental Figure S2.

*- Please provide information about the “core transcriptional responses” and “serovar-specific responses” stated in the end of the paragraph for Fig 2 (pg 8)*

We have added this analysis to show the top 25 similar and different responses to the serovars in Figure 2F-G. Description of these results are stated in lines 203-210.

*- Please state the total number of bacteria for each experiment injected per organoid and the approximate number of IECs/organoid*

The number of bacteria injected per HIO is described in the Methods section. HIOs were microinjected with 10^5^ CFU to maximize invasion at 2.5h and 8h time points whereas HIOs were injected with 10^3^ CFU for 24h to allow for replication within the HIO during infection. The HIOs vary in size, however we can roughly estimate that per 5 μm section there are about 1000 cells with about 100 sections per HIO leading to approximately 10^5^ IECs per HIO.

*- On page 10, the authors refer to Fig 3C-E, however D-E do not seem to exist*

Corrected.

*- Fig 5 C: the ROS staining is difficult to tell apart from autofluorescent material accumulating in the organoid lumen, especially as the organoid shown in the SE image seems to be specifically big. Please provide images with better resolution and in combination with an epithelial marke*r.

This experiment was performed using live microscopy showing whole organoids and cannot be combined with epithelial cell markers that must be visualized with antibody staining. There is no autofluorescence which is indicated by the low fluorescence signal in PBS, ST or STM injected organoids.

*- The authors might want to discuss their findings in the light of Forbester et al., 2015 Infection and Immunity 10.1128/IAI.00161-15*

We have added new data to compare to other studies in supplementary Figure S3.

*- “Many previous studies of ST infection employed different cell lines which may overlook particular aspects of epithelial function” (pg 13) -> this, in comparison to organoids, has been previously addressed, e.g. in* [*https://doi.org/10.1111/cmi.13191*](https://doi.org/10.1111/cmi.13191)

This study used mouse organoids and did not use an infection model but indeed did reinforce that primary epithelial cells are different from immortalized cells. We added this reference to the Discussion section (line 458-462).

*- Why would ST induce less cell proliferation compared to the NTS serovars? Please discuss*

We did not conclude that ST induces less cell proliferation. We now add new data (Figure 5D) to clarify this point, which emphasizes that only STM infection reduces cell proliferation.

- *The inflammasome-activated cytokine IL18 is mainly regulated on a protein level by cleavage of pro-IL18 to IL18. As inflammasomes and subsequent activation of IL18 have been shown to be of importance of early responses to intracellular infection of IECs with STM (in mice), it might be relevant to add this cytokine to Fig 4D (see Sellin et al., Cell Host & Microbe 2014 10.1016/j.chom.2014.07.001; Rauch et al., Immunity 2017* [*https://doi.org/10.1016/j.immuni.2017.03.016*](https://doi.org/10.1016/j.immuni.2017.03.016)*). Could induction of inflammasome-mediated cell expulsion explain differences in the induction of cell cycle associated genes?*

Defining whether the inflammasome mediates these responses is definitely a question of interest to us. We did measure production of both IL1b and IL18 and did not detect either in culture supernatant of the infected HIOs, although we cannot rule out that these cytokines were preferentially secreted on the luminal side. We feel that more investigation is needed to specifically address the role of the inflammasome in HIO epithelium, including effects on cell cycle and cell death.

*- The paragraphs on Fig 4 A-C in the results versus discussion section interpret the data in opposite ways – once the authors claim to see a difference, once not. In any case, plotting the data in a different way and performing a statistical test on the differences should provide clarity here*

In the Results section we were referring to a general theme of transcriptional profiles of immune mediators during STM, SE or ST infection. We stated that the majority of these mediators followed the specific trend of being highly induced during STM infection, moderately induced during SE infection and only weakly induced during ST infection. In the Discussion, we referred to specific mediators including IL-6, IL-8, BD-2 and ELAFIN. We have now modified the Discussion to state that ST infection produced less IL-6, IL-8, BD-2 and ELAFIN when compared to STM and SE infection despite induction at the transcriptional level (line 486-488).

*- At the end of the paragraph describing Fig 4 (pg 11), the authors say that “Our data reflects previous reports that ST…”. Please cite those previous reports*

This sentence has been deleted.

*- In Fig 5, the authors observe that SE induces more pronounced ROS formation. Please discuss how this ROS formation would be specifically triggered by SE. As SE and ST show similar intracellular loads in Fig 1, it would be interesting to see how ST-infected organoids behave with regard to ROS production. Intuitively, would it not be expected that increased inflammatory responses correlate with increased ROS formation? Please discuss why the opposite seems to be the case.*

We speculate that genes unique to SE cause the accumulation of ROS in the HIOs. Additional future experiments would be required to test this hypothesis. However, we have included data (now in Figure 6E) showing ROS data in ST-infected HIOs.

*- Salmonella is equipped with a wide range of virulence factors which can directly or indirectly interact with and modify host responses. Could the expression of specific virulence factors by specific serovars lead to the difference in responses? This could be tested with the help of reporter strains.*

We have provided data showing that differences in SPI-1 and SPI-2 effector expression across the three serovars in supplementary Figure 1. We now discuss the possibility that these differences may account for differences in invasion and other aspects of pathogenesis across the three serovars.

Reviewer #3: Minor comments:

*- Figure 1C-D: scale should be added*

We have added a scale bar to the original Figure 1C-D (now Figure 1G-H).

*- Method RNA-seq: more detail should be provided on the method used to generate the libraries.*

Details of the RNAseq method have been added to include the kit used to generate the libraries and the sequencing depths.

*- Method RNA-seq: the depth of sequencing should be provided.*

Details of the RNAseq method have been added to include the sequencing depths.

*- Method RNA-seq data analysis: the authors are not mentioning if how gene expression is quantified and how normalization has been done between the libraries.*

Normalized gene counts were determined using the tximport package (doi: [10.12688/f1000research.7563.2](https://dx.doi.org/10.12688%2Ff1000research.7563.2)). Gene expression during infection is quantified relative to PBS samples using the *DESeq2* package (doi: [10.1186/s13059-014-0550-8](https://doi.org/10.1186/s13059-014-0550-8)). We now have included the information in the Method section (line).

*- Page 14: typo “ifferential”*

Corrected.
