# Supplementary material for: Comparative transcriptional profiling of the early host response to infection by typhoidal and non-typhoidal Salmonella serovars in human intestinal organoids
Source: PLoS Pathog. 2021 Oct 20;17(10):e1009987. doi: 10.1371/journal.ppat.1009987 (PMC8570492; doi:10.1371/journal.ppat.1009987)
Supplement: Attachment — Submitted filename: Reviewer response R2.docx [file ppat.1009987.s017.docx]

**Reviewer Response**

We appreciate the positive feedback from the reviewers and have addressed the additional specific points as noted below.

**Reviewer #1**

*The authors have added some clarification regarding the inoculum sizes used, but it remains unclear which inoculum size was used for the 2.5h time point. It seems that in Fig. 1, 10^3 CFU were injected (according to l. 124-125), while for the transcriptome data set it sounds like the inoculum size for the 2.5h time point was 10^5 CFU (l. 173-175). Unfortunately, nothing is stated in the methods section regarding this time point. This point needs to be clarified, especially as the low inoculum seems to result in differences in Salmonella loads for the different strains tested, while there is no evident difference with the higher inoculum at 8 hpi. If the 2.5h infection for the transcriptome data set was indeed performed with a different inoculum size than the data presented in Fig. 1B, it would be highly important for the interpretability of the data set to add the CFU data also for this time point and inoculum size. Stating the respective inoculum sizes for the different experiments in the figure legends (Fig. 1 and 2) would make it much easier for the reader to understand the experimental setup.*

We have added additional clarification and rationale for the specific inoculum used in Figs. 1 and 2 in the figure legends and methods section.

*While it is evident that the authors cannot test numerous strains per serovar and it is reasonable to choose the best-characterized ones for the experimental setup used here, the substantial differences between strains of one serovar should anyhow be discussed to make clear to the reader that host responses likely vary substantially even between strains of one serovar. It should be pointed out that the response of one strain cannot be generalized to the entire serovar.*

This is now noted in the Discussion.

*- Please plot individual data points instead/in addition for Fig 5D, S3*

*- L. 103: "…to compare the transcriptomes of intestinal…"*

*- L. 174: please clarify what you mean with "similar" (similar to which condition?)*

*- L. 213: add "at 8 hpi" to clarify*

*- L. 319: "..during these infections were not sufficient…"*

*- L. 456-457 "…other serovars exhibit different infection patterns in different organisms": please provide references - added*

*- L. 487: "…despite inducing their…"*

Revised as suggested.

**Reviewer #2**

*still have a strong concern on the massive experimental variability between the three serovars that the authors outline in Figure 1 (Figure 1B and 1C). This variability is clearly bounded to the experimental set-up and I believe that further studies will refine this problem. Furthermore, in the current setting, the authors cannot discriminate the host response induced solely by the extracellular versus the intracellular bacteria. The discussion should clearly outline that the heterogeneity in the host response can be due to these confounding factors.*

We appreciate the reviewer’s perspective and have addressed both points in the Discussion. It is likely that the lower inoculum used in Fig. 1 better mimics physiological infection, and the variability that we observe there may indicate meaningful differences in invasion or intracellular replication. We used the higher inoculum for the transcriptomic analyses to lessen the influence of having differential intracellular bacterial burden. Rather than consider these confounding factors, we view the HIOs as a holistic model that can allow for integrating input of luminal and intracellular bacteria, as well as ongoing bacterial invasion, which may better reflect the natural course of infection.
